# Intrathecal AAV9/*AP4M1* gene therapy for hereditary spastic paraplegia 50 shows safety and efficacy in preclinical studies

**DOI:** 10.1172/JCI164575

**Published:** 2023-05-15

**Authors:** Xin Chen, Thomas Dong, Yuhui Hu, Raffaella De Pace, Rafael Mattera, Kathrin Eberhardt, Marvin Ziegler, Terry Pirovolakis, Mustafa Sahin, Juan S. Bonifacino, Darius Ebrahimi-Fakhari, Steven J. Gray

**Affiliations:** 1 Department of Pediatrics, UT Southwestern Medical Center, Dallas, Texas, USA.; 2 Neurosciences and Cellular and Structural Biology Division, Eunice Kennedy Shriver National Institute of Child Health and Human Development, NIH, Bethesda, Maryland, USA.; 3 Department of Neurology and F.M. Kirby Neurobiology Center, Boston Children’s Hospital, Harvard Medical School, Boston, Massachusetts, USA.; 4 CureSPG50 Foundation, Toronto, Ontario, Canada.

**Keywords:** Neuroscience, Neurological disorders

## Abstract

Spastic paraplegia 50 (SPG50) is an ultrarare childhood-onset neurological disorder caused by biallelic loss-of-function variants in the *AP4M1* gene. SPG50 is characterized by progressive spastic paraplegia, global developmental delay, and subsequent intellectual disability, secondary microcephaly, and epilepsy. We preformed preclinical studies evaluating an adeno-associated virus (AAV)/*AP4M1* gene therapy for SPG50 and describe in vitro studies that demonstrate transduction of patient-derived fibroblasts with AAV2/*AP4M1*, resulting in phenotypic rescue. To evaluate efficacy in vivo, *Ap4m1*-KO mice were intrathecally (i.t.) injected with 5 × 10^11^, 2.5 × 10^11^, or 1.25 × 10^11^ vector genome (vg) doses of AAV9/*AP4M1* at P7–P10 or P90. Age- and dose-dependent effects were observed, with early intervention and higher doses achieving the best therapeutic benefits. In parallel, three toxicology studies in WT mice, rats, and nonhuman primates (NHPs) demonstrated that AAV9/*AP4M1* had an acceptable safety profile up to a target human dose of 1 × 10^15^ vg. Of note, similar degrees of minimal-to-mild dorsal root ganglia (DRG) toxicity were observed in both rats and NHPs, supporting the use of rats to monitor DRG toxicity in future i.t. AAV studies. These preclinical results identify an acceptably safe and efficacious dose of i.t.-administered AAV9/*AP4M1,* supporting an investigational gene transfer clinical trial to treat SPG50.

## Graphical abstract

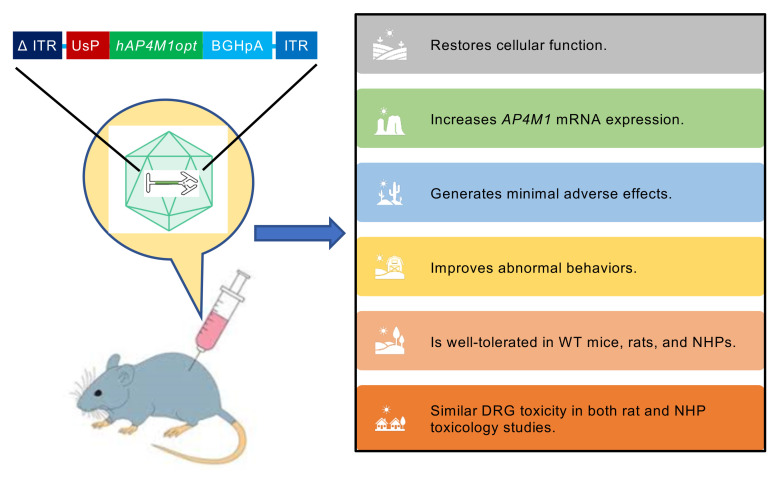

## Introduction

Hereditary spastic paraplegia 50 (SPG50; OMIM: 612936) is an ultrarare childhood-onset neurological disorder characterized by progressive spastic paraplegia, global developmental delay, and subsequent intellectual disability, secondary microcephaly, early-onset epilepsy, and other symptoms (1, 2). SPG50 is an autosomal recessive disorder caused by biallelic loss-of-function mutations in the *AP4M1* gene (3). Since the initial identification of these mutations in the *AP4M1* gene in 2009 (3), the spectrum of disease-associated variants has been found to mainly consist of nonsense or frameshift mutations, with only a subset of patients carrying missense variants or splice-site variants (1). Biallelic loss-of-function mutations in the genes encoding the 3 other subunits of the adaptor protein complex 4 (AP-4), namely *AP4B1*, *AP4E1*, and *AP4S1*, also lead to childhood-onset hereditary spastic paraplegia (SPG47, SPG51, and SPG52), establishing a common phenotype, an “AP-4 deficiency syndrome” (1, 2, 4).

The *AP4M1* gene encodes a 453–amino acid protein, AP4M1, which is part of the AP-4, an evolutionarily conserved and obligate heterotetrametric complex (5–9). The AP-4 complex localizes to the *trans*-Golgi network (TGN), where it plays an important role in vesicle-mediated protein transport by selecting/packaging cargo proteins for export to endosomes, lysosomes, or autophagosomes (9–14). Recent in vitro and in vivo studies have demonstrated that the autophagy-related protein 9A (ATG9A) is sorted and transported by the AP-4 complex and that loss of AP-4 leads to ATG9A retention in the TGN, potentially affecting the transport and function of ATG9A in axons (12–15). More recently, diacylglycerol lipase-β (DAGLB) was also identified as AP-4 vesicle cargo and was found to be involved in driving axonal growth (16).

The incidence and prevalence of SPG50 disease are unknown. The first cross-sectional analysis, the Registry and Natural History Study for Early Onset Hereditary Spastic Paraplegia (NCT04712812), was conducted in 2019 and included 59 patients from around the world (1). To date, there is no approved treatment for patients with SPG50. Management is limited to physical therapy, occupational therapy, speech and language therapy, and symptomatic interventions with antispasticity agents or antiseizure medications (2). Because all aspects of SPG50 disease stem from the loss of AP4M1 gene function, gene replacement therapy presents a rational therapeutic avenue.

Over the last two decades, there have been numerous viral vector–based gene therapy approaches tested for other disorders. Collective evidence has demonstrated that gene therapy can be clinically effective and well tolerated, resolving in some cases the majority of symptoms associated with rare inherited diseases (17). Recombinant adeno-associated viral vector type 9 (AAV9), in particular, has been shown to represent a safe and efficacious neurotropic vector to deliver transgenes to the CNS (18). These recombinant AAV vectors are relatively nonpathogenic and nonreplicating, and they transduce both dividing and nondividing cells. Importantly, they are incapable of coding viral proteins and are primarily nonintegrating (19). AAV9 mediates broad gene transfer across the entire CNS in a way that translates from mice to larger animal models (18–28). Furthermore, AAV9 can be purified in large quantities and high concentrations to deliver a functional copy of a gene to the CNS using intrathecal (i.t.) administration (18–28). i.t. administration of an AAV9 vector is being used in ongoing gene therapy clinical trials for giant axonal neuropathy (20) and CLN7 Batten disease (29), among others.

In this study, utilizing an approach similar to that used in our prior work on giant axonal neuropathy and CLN7 gene therapies, we evaluated the efficacy and safety of *AP4M1* gene transfer in vitro in fibroblasts from patients with SPG50 and in vivo in *Ap4m1*-KO mice. To study the relative risks associated with AAV9/*AP4M1*, we also conducted a dose-ranging, 1-year non–good laboratory practice (non-GLP) toxicology study in WT C57BL/6J mice treated by i.t. administration, including a comprehensive histopathological assessment at several time points. Furthermore, we conducted dose-ranging, 3-month GLP toxicology studies in both WT Sprague-Dawley (SD) rats and nonhuman primates (NHPs), again using i.t. administration. Here, we present these preclinical data in rodents and NHPs to support the clinical development of i.t.-administered AAV9/*AP4M1* as a potential therapy for patients with SPG50. An investigational phase I i.t. gene transfer trial using 1 × 10^15^ vector genome (vg) per patient of AAV9/*AP4M1* to treat SPG50 was approved by Health Canada on December 30, 2021, and by the FDA on August 11, 2022.

## Results

### AAV2/AP4M1 vector restored ATG9A trafficking and AP4E1 levels in primary fibroblasts from patients with SPG50.

**Figure 1:**
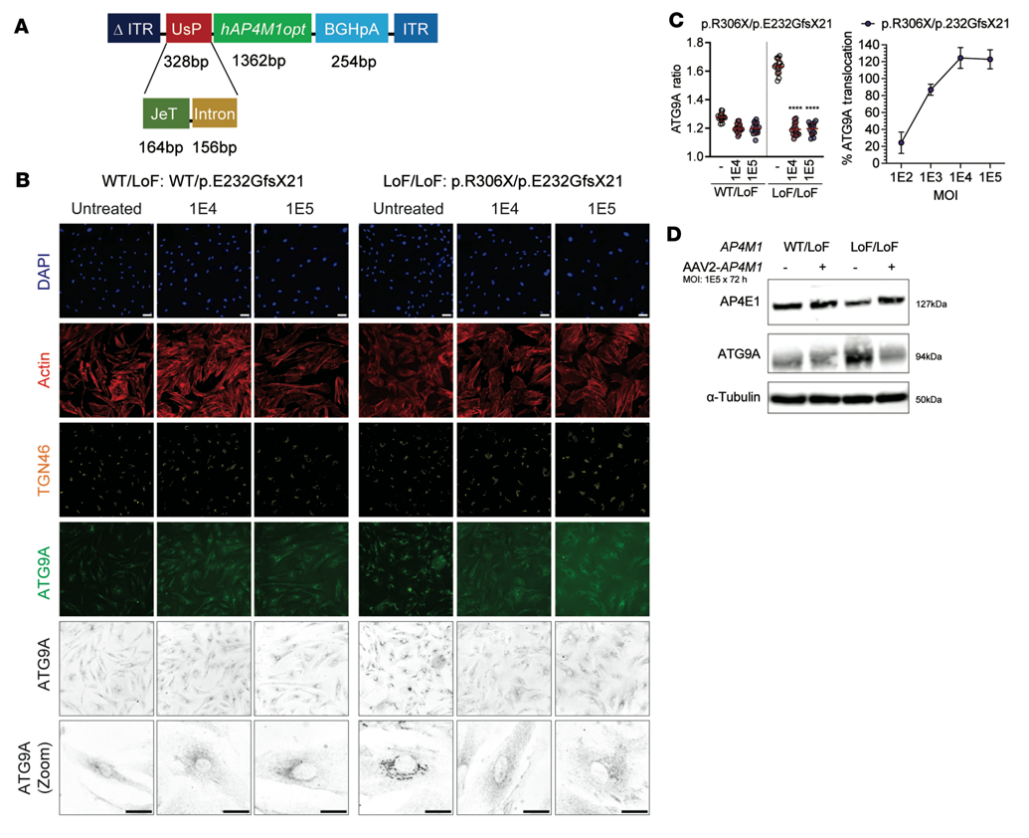
AAV2/*AP4M1* vector construct expressing human *AP4M1* and its restoration of ATG9A trafficking and AP4E1 level in primary fibroblasts from a patient with SPG50. (**A**) Schematic of AAV2/*AP4M1* construct comprising a mutant AAV2 ITR with the D element deleted (Δ ITR), UsP promoter (JeT + Intron), *hAP4M1opt*, synthetic BGHpA signal, and WT AAV2 ITR. (**B–D**) Fibroblasts from a clinically unaffected heterozygous carrier (same-sex parent, WT/pE232GfsX21) or patient with biallelic LoF variants in AP4M1 (p.R306X/pE232GfsX21) were treated with AAV2/*AP4M1* vector at an MOI of 1 × 10^4^ or 1 × 10^5^ for 72 hours. The fibroblasts were then fixed for (**B** and **C**) immunocytochemistry and automated image analysis or harvested for (**D**) Western blotting. Scale bar: 20 μm. (**C**) Left: The mean ATG9A ratio for all conditions. Scatter plots show the mean ± SD for each well (*n* = 16 wells/group). Data sets were compared using 1-way ANOVA, with α set at 0.05, and Dunnett’s correction. Right: The percentage translocation of ATG9A. Translocation refers to the change (in %) relative to the difference between the positive (ATG9A ratio of fibroblasts with heterozygous *AP4M1* variants) and negative controls (ATG9A ratio of fibroblasts with homozygous LoF variants in *AP4M1*) of the same assay plate. A dose-dependent effect becomes evident. ****P < 0.0001 compared with untreated fibroblasts. (**D**) Western blot of whole-cell lysates of fibroblasts from a clinically unaffected heterozygous carrier (parent) and patient with SPG50 treated with AAV2/*AP4M1* at an MOI of 1 × 10^5^ for 72 hours. AAV2, adeno-associated virus 2; AP4E1, adaptor protein complex, subunit ɛ; AP4M1, adaptor protein complex, subunit μ4; ATG9A, autophagy-related protein 9A; BGHpA, bovine growth hormone polyadenylation; *hAP4M1opt*, human *AP4M1* codon-optimized coding sequence; ITR, inverted terminal repeat; LoF, loss of function; TGN, *trans*-Golgi network.

To determine whether AAV-mediated expression of WT *AP4M1* restores ATG9A trafficking and the level of AP4E1 (as a surrogate for AP-4 complex formation) in primary fibroblasts from patients with SPG50, we created a self-complementary (sc)AAV2/*AP4M1* vector, which was packaged with an expression cassette comprising a mutant AAV2 inverted terminal repeat (ITR) with the D element deleted (Δ ITR), the expressing UsP promoter, the human *AP4M1* codon-optimized coding sequence (h*AP4M1opt*), the bovine growth factor polyadenylation signal (BGHpA), and WT AAV2 ITR (Figure 1A). The UsP promoter is a combination of the JeT promoter (20, 29, 30) and an added intron sequence (31).

As initial proof-of-concept studies for human *AP4M1* gene therapy, AAV2/*AP4M1* efficacy at improving cellular function in cultured fibroblasts from patients with SPG50 was tested independently by the laboratories of DEF and JSB. In both laboratories, multiple patient fibroblast lines were transduced for 72 hours using an AAV2/*AP4M1* vector at MOIs of 1 × 10^2^, 1 × 10^3^, 1 × 10^4^, and 1 × 10^5^ vg per cell. These assays used an AAV2-based vector to deliver the *AP4M1* expression cassette and assess the effect of *AP4M1* transgene expression because fibroblasts are not readily transduced by the AAV9 vector.

In AP-4–deficient cells, including fibroblasts from patients with SPG50, the AP-4 cargo protein, ATG9A, robustly accumulates in the TGN (12, 14, 15, 32). Using a high-throughput phenotypic assay (32), DEF’s group screened fibroblasts derived from a patient with well-characterized SPG50 (Supplemental Table 1; supplemental material available online with this article; https://doi.org/10.1172/JCI164575DS1), carrying 2 truncating variants (p.R306X/p.E232GfsX21) treated with AAV2/*AP4M1* vector at an MOI of 1 × 10^4^ or 1 × 10^5^, respectively. Expression of *AP4M1* for 72 hours reduced the amount of TGN-localized ATG9A close to that of controls, indicating redistribution of ATG9A and phenotypic rescue in the vast majority of cells (Figure 1, B and C). Western blotting from whole-cell lysates confirmed an increase in AP4E1s level following treatment with AAV2/*AP4M1*, indicative of stable formation of AP-4, and a reduction in ATG9A to levels similar to controls (Figure 1D). Reduced cell numbers were observed with the MOI of 1 × 10^5^ in both patient and control fibroblasts, suggesting potential cell toxicity (Supplemental Figure 1A). Reduction of the MOI to 1 × 10^2^ and 1 × 10^3^ led to dose-dependent rescue of ATG9A trafficking and minimal cell toxicity in 3 fibroblast lines from well-characterized patients with SPG50 (p.R306X/p.E232GfsX21; p.N73KfsX43/p.Y284S; and IVS13+2dupT/p.T69P; Supplemental Figure 1, B–F, and Supplemental Table 1).

**Figure 2:**
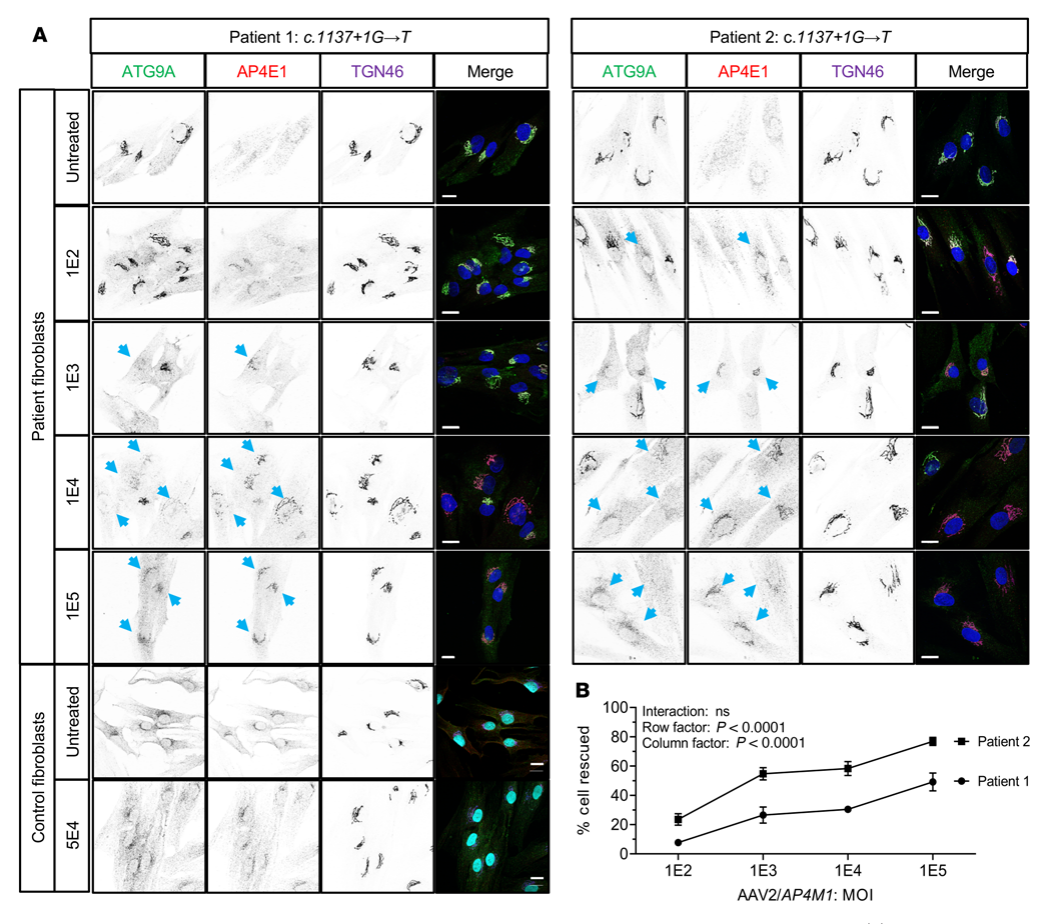
AAV2/*AP4M1* vector restored ATG9A trafficking and AP4E1 levels in primary fibroblasts from patients with SPG50. (**A**) Fibroblasts from 2 sibling patients with a donor splice site pathogenic mutation in intron 14 of the *AP4M1* gene (*c.1137+1G*→*T*) or normal control fibroblasts were treated without or with AAV2/*AP4M1* vector at the indicated MOI for 72 hours. Fibroblasts were then fixed for immunofluorescence analysis of ATG9A, AP4E1, and TGN46, as described in Methods. Nuclei were stained with DAPI (in blue, merge). Single channels are shown in inverted grayscale. Note that expression of AP4M1 by the viral vector caused dispersal of the ATG9A signal and increased AP4E1 staining at the TGN (i.e., phenotypic rescue; indicated by arrows). Scale bar: 20 μm. (**B**) The percentage of rescued cells was counted and is represented as the mean ± SEM from 2 independent experiments (see Supplemental Table 2 for details). Statistical analysis was done using 2-way ANOVA with repeated measures. AAV2, adeno-associated virus 2; AP4E1, adaptor protein complex, subunit ɛ; AP4M1, adaptor protein complex, subunit μ4; ATG9A, autophagy-related protein 9A; SPG50, spastic paraplegia 50; TGN, *trans*-Golgi network.

Comparable dose-dependent rescue results were obtained by JSB’s laboratory using 2 additional fibroblasts from siblings with SPG50 caused by a homozygous splice site pathogenic mutation in intron 14 of the *AP4M1* gene (c.1137+1G→T) (3, 33). At the MOI of 1 × 10^5^, up to 49% and 77% of the fibroblasts from patients 1 and 2, respectively, exhibited rescue of both ATG9A localization and AP4E1 staining (Figure 2 and Supplemental Table 2). In these experiments, there was no sign of cell toxicity, with nuclei, cell size, and shape looking normal, even at the high MOI of 1 × 10^5^. Taken together, collected data (Figures 1 and 2, Supplemental Figure 1, and Supplemental Tables 1 and 2) demonstrated that UsP-driven *AP4M1* expression restored ATG9A trafficking and AP4E1 levels in fibroblasts from 5 patients with SPG50 with 4 different biallelic mutations in a dose-dependent manner, with potential cell toxicity at the high MOI of 1 × 10^5^ AAV2/*AP4M1* vector.

### AAV9/AP4M1 gene therapy increased AP4M1 mRNA expression in the CNS of Ap4m1-KO mice.

**Figure 3:**
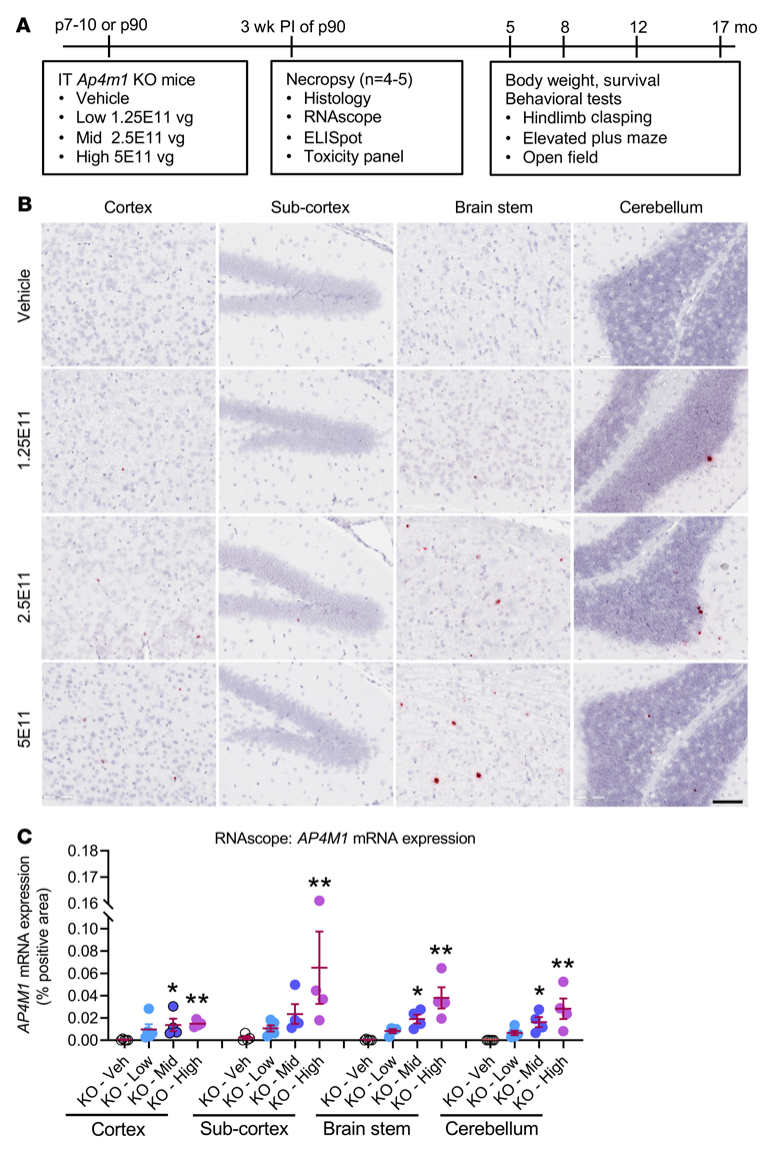
The experimental design for in vivo efficacy study and increased *AP4M1* mRNA expression in the CNS of *Ap4m1*-KO mice following i.t. treatment with AAV9/*AP4M1*. (**A**) Vehicle or low (1.25 × 10^11^ vg/mouse), mid (2.5 × 10^11^ vg/mouse), or high (5 × 10^11^ vg/mouse) doses of AAV9/*AP4M1* vector were administered intrathecally to balanced numbers of male and female *Ap4m1*-KO mice at P7–P10 (before manifesting) or P90 (early manifestation). Study readouts at each time point at specified ages are listed from left to right. (**B**) Brains from mice treated at P90 for 3 weeks were analyzed by RNAscope staining to detect *hAP4M1opt* mRNA. Histology images with 1 section per animal were digitized with a ScanScope slide scanner and analyzed using custom analysis settings in HALO Image Analysis Platform. Scale bars: 100 μm. (**C**) Results are presented as percentage area positively stained for *hAP4M1opt* mRNA in the indicated brain regions. Each data point represents measurement from an individual animal (*n* = 4–5), with lines representing the mean ± SEM. Data sets that passed tests for normality or homogeneity of variance were analyzed using 1-way ANOVA, with α set at 0.05, and Dunnett’s correction for relevant pairwise comparisons. Data sets that did not pass tests for normality or homogeneity of variance were analyzed using Kruskal-Wallis test, with α set at 0.05, and Dunn’s correction for relevant pairwise comparisons. *P < 0.05, **P < 0.01 compared with KO mice treated with vehicle (Veh). AAV9, adeno-associated virus 9; AP4M1, adaptor protein complex, subunit μ4; *hAP4M1opt*, human *AP4M1* codon-optimized coding sequence; i.t., intrathecal; PI, postinjection; vg, vector genome.

To test whether the AAV9/*AP4M1* vector rescues the phenotypes in *Ap4m1^–/–^* (*Ap4m1-*KO) mice, balanced groups of male and female KO mice were injected i.t. at P7–P10 (cohort before disease manifestation) or P90 (cohort with early disease manifestation) with vehicle or low (1.25 × 10^11^ vg/mouse), mid (2.5 × 10^11^ vg/mouse), or high (5 × 10^11^ vg/mouse) dose of AAV9/*AP4M1* vector (Figure 3A). At 3 weeks after injection, 4–5 mice from each group treated at P90 were used to evaluate *AP4M1* mRNA expression by RNAscope (Figure 3, B and C). The KO mice receiving mid and high doses of AAV9/*AP4M1* vector exhibited dose-dependent increased levels of *AP4M1* mRNA in all brain regions assessed compared with vehicle-treated animals (Figure 3C).

### AAV9/AP4M1 gene therapy generated minimal adverse effects in Ap4m1-KO mice.

**Figure 4:**
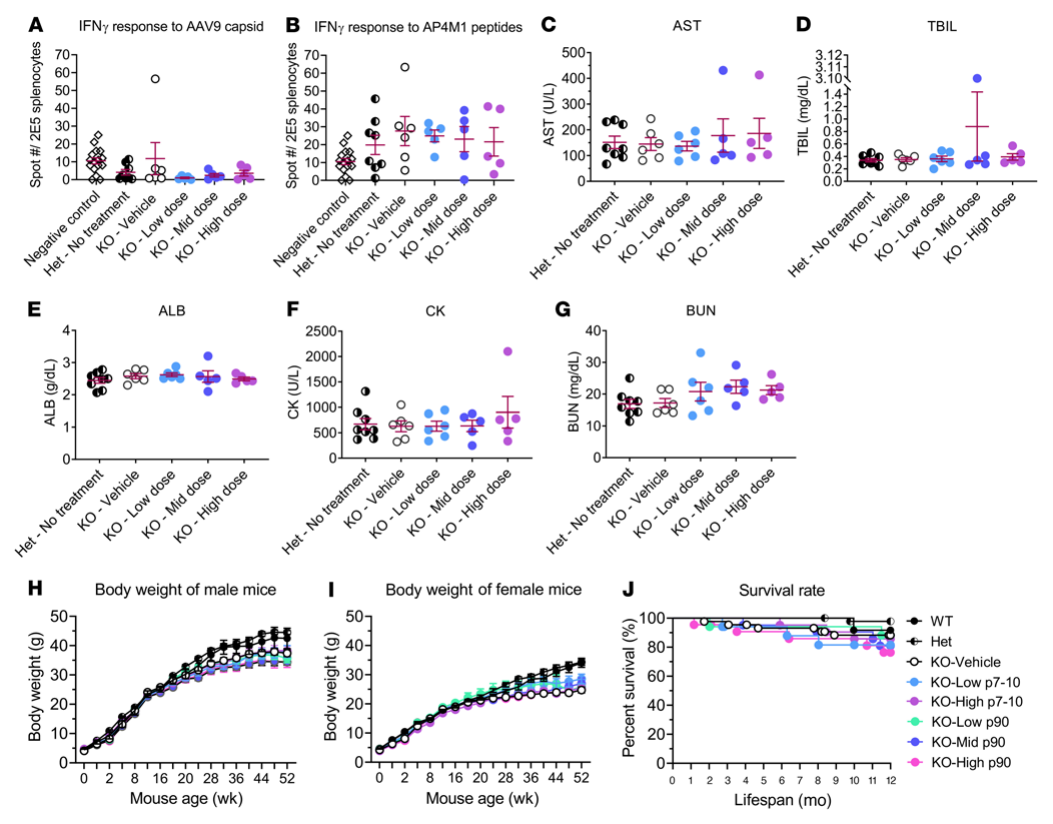
i.t. AAV9/*AP4M1* treatment generated minimal IFN-γ responses to AAV9 capsid or AP4M1 peptides, minimal effects on serum toxicity panels, male or female body weight, or survival rates. (**A–G**) Vehicle or low (1.25 × 10^11^ vg/mouse), mid (2.5 × 10^11^ vg/mouse), or high (5 × 10^11^ vg/mouse) doses of AAV9/*AP4M1* vector were administered intrathecally to balanced numbers of male and female KO mice at P90, with WT and Het mice as normal controls. At 3 weeks after injection, (**A** and **B**) mouse splenocytes and serum were collected for ELISpot and (**C–G**) toxicity panel analyses. Each data point represents a measurement from an individual animal (*n* = 5–8), with lines representing the mean ± SEM. Data sets that passed tests for normality or homogeneity of variance were analyzed using 1-way ANOVA, with α set at 0.05, and Dunnett’s correction for relevant pairwise comparisons. Data sets that did not pass tests for normality or homogeneity of variance were analyzed using Kruskal-Wallis test, with α set at 0.05, and Dunn’s correction for relevant pairwise comparisons. No significant differences were observed. (**H** and **I**) Male (*n* = 7–26) and female (*n* = 5–24) mouse body weights were monitored up to 52 weeks of age. Two-way ANOVA with repeated measures was used for statistical analysis. (**J**) Mouse survival shown with Kaplan-Meier survival curves compared with log-rank (Mantel-Cox) test. No significant differences were observed. AAV9, adeno-associated virus 9; ALB, albumin; AP4M1, adaptor protein complex, subunit μ4; AST, aspartate transaminase; BUN, blood urea nitrogen; CK, creatine kinase; Het, heterozygotes; i.t., intrathecal; TBIL, total bilirubin; vg, vector genome.

Immune responses to the AAV capsid and/or transgene product remain a major potential challenge for translating experimental drugs to clinical approval (34, 35). While natural AAV infection can prevent patients from receiving AAV gene therapies, immune responses to these therapies can result in loss of transgene expression and even tissue damage. Such immune responses could also confound the results of preclinical experiments. To evaluate any IFN-γ immune response to the AAV9/*AP4M1* vector in the *Ap4m1*-KO mice, splenocytes from mice treated with AAV9/*AP4M1* for 3 weeks were plated and treated in vitro with either AAV9 capsid or AP4M1 peptide pools for 2 days along with both negative (no peptide) and positive (PMA + ionomycin) controls. While the negative control had a mean of 10 spots (Figure 4, A and B), the positive control had too many spots to count. Figure 4A shows the IFN-γ response to the AAV9 capsid peptide pool. Treating the *Ap4m1*-KO mice with vehicle or low (1.25 × 10^11^ vg/mouse), mid (2.5 × 10^11^ vg/mouse), or high (5 × 10^11^ vg/mouse) doses of AAV9/*AP4M1* did not significantly increase the numbers of spots. The same pattern was seen in the IFN-γ response to an AP4M1 peptide pool (Figure 4B). Taken together, these results demonstrated that the AAV9/*AP4M1* gene therapy generated minimal IFN-γ immune response to either AAV9 capsid or AP4M1 protein in the *Ap4m1*-KO mice.

To test whether treatment with AAV9/*AP4M1* leads to organ toxicity in *Ap4m1-*KO mice, mouse sera were analyzed using a toxicology panel, including aspartate transaminase (AST), total bilirubin (TBIL), albumin, creatine kinase (CK), and blood urea nitrogen (BUN). Animals receiving AAV9/*AP4M1* had overall normal levels of these serum markers at 3 weeks after injection in all but 1 mouse in both the mid-dose and high-dose groups (Figure 4, C–G). The animal in the mid-dose group (2.5 × 10^11^ vg/mouse) had serum AST and TBIL levels of 431 U/L and 3.1 mg/dL, respectively. The animal in the high-dose (5 × 10^11^ vg/mouse) group had serum AST and CK levels of 413 U/L and 2102 U/L, respectively. Overall, these results demonstrate that the *Ap4m1-*KO mice tolerated the AAV9/*AP4M1* vector well, with an infrequent occurrence of toxicity observed in liver and muscles.

To further test whether the AAV9/*AP4M1* vector generates adverse effects in the *Ap4m1-*KO mice, body weight throughout life and animal survival were monitored regularly. Both male and female *Ap4m1-*KO mice had significantly reduced body weight compared with WT and heterozygous (Het) littermates, which was not significantly affected by i.t. administration of any dose of the AAV9/*AP4M1* vector at P7–P10 or P90 (Figure 4, H and I). Similarly, no significant differences in survival rates among groups were observed (Figure 4J). The long-term efficacy study lasted for 20.5 months after treatment in SPG50 mice. All livers were harvested during necropsy and any macroscopical abnormalities in liver were further checked microscopically for pathological diagnosis by a certified veterinary pathologist. As summarized in Supplemental Table 3, we did not notice any increase in the incidence of liver tumors in the KO vehicle group compared with WT control group, and AAV9/*AP4M1* treatment did not affect the incidence of any liver abnormalities, including liver tumors. Taken together, AAV9/*AP4M1* gene therapy generated minimal adverse effects in *Ap4m1*-KO mice.

### AAV9/AP4M1 gene therapy partially restored abnormal behavioral phenotypes in Ap4m1-KO mice.

**Figure 5:**
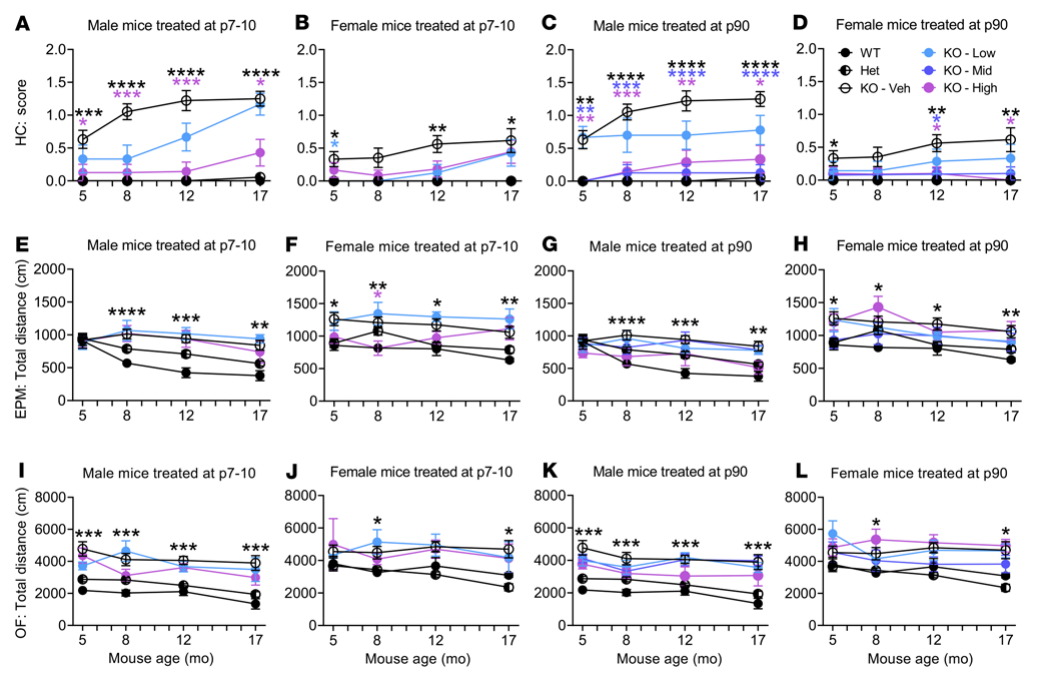
i.t. AAV9/*AP4M1* treatment partially improved abnormal behavioral phenotypes in *Ap4m1*-KO mice. Vehicle or low (1.25 × 10^11^ vg/mouse), mid (2.5 × 10^11^ vg/mouse), or high (5 × 10^11^ vg/mouse) doses of AAV9/*AP4M1* vector were administered intrathecally to balanced numbers of male and female mice at P7–P10 or P90, with WT and Het mice as normal controls. The mice were subjected to (**A–D**) hind limb clasping, (**E–H**) elevated plus maze, and (**I–L**) open-field tests at 5, 8, 12, and 17 months of age. All data are presented as mean ± SEM (male, *n* = 7–26, and female, *n* = 5–24). Two-way ANOVA with repeated measures was used for statistical analysis. *P < 0.05, **P < 0.01, ***P < 0.001, ****P < 0.0001 compared with KO mice treated with vehicle (Veh). AAV9, adeno-associated virus 9; AP4M1, adaptor protein complex, subunit μ4; EPM, elevated plus maze test; HC, hind limb clasping test; Het, Heterozygotes; i.t., intrathecal; OF, open-field test; vg, vector genome.

*Ap4m1*-KO mice were not phenotypically characterized prior to this study. To test if the KO mice displayed abnormal behavioral phenotypes and AAV9/*AP4M1* gene therapy improves these alterations, mouse cohorts underwent a battery of behavioral tests, including hind limb clasping, elevated plus maze, open-field, rotarod, grip strength, and wire hang tests. These tests were originally selected based on the reported neurological phenotypes in *Ap4e1*-KO mice (33) as well as for their ability to be repeated longitudinally. While *Ap4m1*-KO mice did not show alterations in the rotarod, grip strength, and wire hang tests (Supplemental Figure 2), they did display behavioral alterations from the hind limb clasping, elevated plus maze, and open-field tests, which are presented in Figure 5. Hind limb clasping has been shown to occur in various neurological disease mouse models (33, 36). Both elevated plus maze and open-field test in mice are tools to assess novel environment exploration, anxiety-related behavior, as well as general locomotor activity (37).

Minimal behavioral deficits were observed when comparing WT or Het control animals and KO vehicle- or KO AAV9/*AP4M1*-dosed animals at 3 months of age (data not shown). However, at 5 months of age, significantly higher scores in the hind limb clasping test were observed in the male KO vehicle-treated group compared with male WT or Het controls, with a deficit rescue observable in the P7–P10 high-dose group (Figure 5A) and P90 mid- and high-dose groups (Figure 5C). The hind limb clasping assessment provided the clearest phenotype across both sexes of all the behavioral tests conducted. More significant results were obtained at 8, 12, and 17 months of age in male mice. Similar trends were also seen in female mice, with the exception that hind limb clasping scores were lower in the KO vehicle-treated female group compared with those in the KO vehicle-treated male group (Figure 5, A–D). As assessed by hind limb clasping, significant and dose-responsive rescues were seen in both sexes and across the length of the study in response to treatment (Figure 5, B and D).

In the elevated plus maze test, female KO vehicle-treated animals at 5, 8, 12, and 17 months of age were hyperactive, traveling longer total distances during the testing period compared with female WT controls (Figure 5F). This phenotype was improved at 8 months of age in the P7–P10 high-dose group, which performed significantly better than the KO vehicle group and was not significantly different from the WT or Het control group (Figure 5F). Treating the female mice at P90 did not generate any discernible benefit by this test (Figure 5H). In male mice, however, the hyperactive phenotype (traveling a longer total distance during the test period) was observed at 8, 12, and 17 months of age in KO vehicle-treated animals when compared with WT controls and was not clearly altered in response to treatment at P7–P10 or P90 (Figure 5, E and G).

In the open-field test, both male and female KO vehicle-treated animals were hyperactive and traveled a longer total distance during the test period compared with WT animals (Figure 5, I–L). While this phenotype was consistent with the findings in elevated plus maze test described above, it was not significantly altered in response to the treatment at P7–P10 or P90 in male or female mice (Figure 5, I–L).

Taken together, the data point to significant behavioral deficits in *Ap4m1*-KO mice starting at 5 months of age. The alterations seen in hind limb clasping and elevated plus maze tests were partially restored in the P7–P10 high-dose as well as P90 mid- and high-dose groups, and a trend toward improvement in other treatment groups was also observed. Overall, these results indicate a positive trajectory following treatment with AAV9/*AP4M1* in *Ap4m1*-KO mice.

### AAV9/AP4M1 gene therapy was safe and well tolerated in WT mice in a non-GLP study.

**Figure 6:**
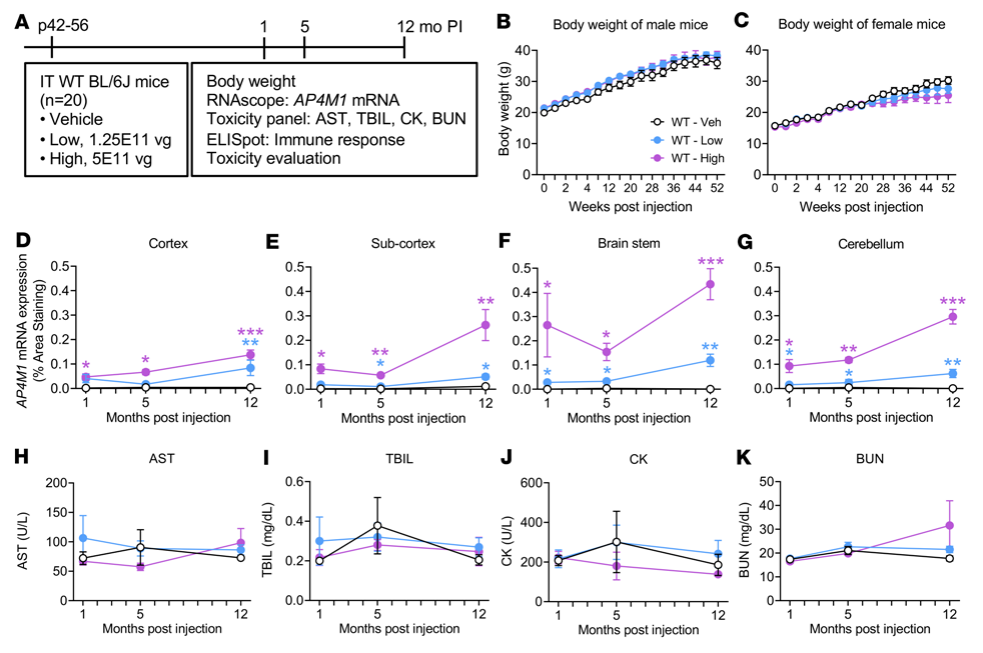
Experimental design for in vivo safety study in WT mice and changes in body weight, *hAP4M1opt* mRNA expression in the CNS, and serum toxicity panels in i.t. AAV9/*AP4M1*-treated mice. (**A**) Vehicle or low (1.25 × 10^11^ vg/mouse), or high (5 × 10^11^ vg/mouse) doses of AAV9/*AP4M1* vector were administered intrathecally to male and female mice at P42–P56 (*n* = 10/group/sex). Study readouts at each time point and specified ages are listed from left to right. (**B** and **C**) Body weights were monitored regularly up to 52 weeks of age. At 1, 5, and 12 months after injection, mouse brain and serum were harvested for RNAscope staining (**D–G**) to detect *hAP4M1opt* mRNA expression and (**H–K**) for assessment of serum toxicity, respectively. Histology images (1 section per animal) were digitized with a ScanScope slide scanner and analyzed using custom analysis settings in HALO Image Analysis Platform. (**D–G**) Results are presented as percentage area staining positive for *hAP4M1opt* mRNA by tissue region. All data are presented as the mean ± SEM. Two-way ANOVA with repeated measures was used for statistical analysis. *P < 0.05, **P < 0.01, ***P < 0.001 compared with WT mice treated with vehicle. AAV9, adeno-associated virus 9; AP4M1, adaptor protein complex, subunit μ4; AST, aspartate transaminase; BUN, blood urea nitrogen; CK, creatine kinase; *hAP4M1opt*, human *AP4M1* codon-optimized coding sequence; i.t., intrathecal; PI, postinjection; TBIL, total bilirubin; vg, vector genome.

To analyze the long-term safety of the AAV9/*AP4M1* therapy, WT C57BL/6J mice were injected i.t. with the AAV9/*AP4M1* vector in a non-GLP toxicology study (Figure 6A). The mice were randomized to different groups, injected i.t. with 5 μL vehicle or different doses of AAV9/*AP4M1* vectors from University of North Carolina Vector Core (UNC-VC) and monitored up to 1 year following injection for changes in body weight and survival rates, presence of adverse events, and histopathology evaluation. In this long-term study, there were no significant differences in body weight between groups (compared within male or female subgroups) at all time points and doses tested (Figure 6, B and C). Similarly, there were no obvious signs of morbidity or any mortality in the adult male and female WT mice treated with up to 5 × 10^11^ vg AAV9/*AP4M1* during the entire study.

At 1, 5, and 12 months after the injection, mouse brain and serum were harvested for RNAscope detection of *AP4M1* mRNA expression and for a serum toxicology panel. Animals receiving AAV9/*AP4M1* had detectable levels of *AP4M1* mRNA in all brain regions assessed, with a dose-dependent increase of mRNA levels compared with that in control animals (Figure 6, D–G). Moreover, *AP4M1* mRNA expression was sustained for up to 12 months after injection. Animals receiving AAV9/*AP4M1* did not display serum toxicity, as indicated by levels of AST, TBIL, CK, and BUN at 1 month after injection, with the exception of 1 male mouse (Figure 6, H–K). In this male mouse, which received a low dose of 1.25 × 10^11^ vg/mouse, AST and TBIL reached 293 U/L and 0.9 mg/dL, respectively. At 5 months after injection, serum toxicity panels were normal, with the exception of 1 vehicle-treated female mouse, which exhibited 180 U/L and 0.76 mg/dL AST and TBIL, respectively. At 12 months after injection, serum toxicology markers were also normal in all but 2 male mice, which received the 5 × 10^11^ vg high dose. In one of these mice, AST, TBIL, and BUN reached 187 U/L, 0.72 mg/dL, and 124 mg/dL, respectively; in the other, AST reached 283 U/L. In conclusion, our results suggest that most of the WT mice tolerated AAV9/*AP4M1* well, with infrequent signs of liver and kidney toxicity across groups.

No other obvious macroscopic abnormalities were observed during necropsy except for some granular appearance in the livers of 2 male mice and 1 female mouse, which were treated with the high dose (5 × 10^11^ vg/mouse) of AAV9/*AP4M1* for 12 months. Microscopically, the 2 male mice (20% of the group) and 1 female mouse (10% of the group) were diagnosed with hepatocellular adenomas (Supplemental Figure 3). If a vector integration event led to clonal expansion, we would expect high amounts of transgene-positive cells within the adenomas, but RNAscope staining was within the levels of all other mice at that dose (Supplemental Figure 3). It is important to mention that hepatocellular adenomas are expected in mice as they age. The National Toxicology Program Historical Controls_B6C3F1/N strain reported that the incidence rate of hepatocellular adenomas reaches as high as 50% in male and 18% in female mice (38). The conclusions of the histological evaluation were that (a) all microscopic changes present in the mice harvested at 1 month and 5 months after injection were considered variations on normal microanatomy for mice of this age, and (b) the tumors and increased number of inflammatory cell infiltrates and degenerative lesions seen in the mice harvested at 12 months after injection were expected in mice as they age (Supplemental Data Files 1–3). Taken together, i.t.-administered AAV9/*AP4M1* doses up to 5 × 10^11^ vg/mouse were overall well tolerated in WT mice, with infrequent occurrence of hepatocellular adenomas.

### AAV9/AP4M1 gene therapy was safe and well tolerated in WT rats in a GLP study.

**Figure 7:**
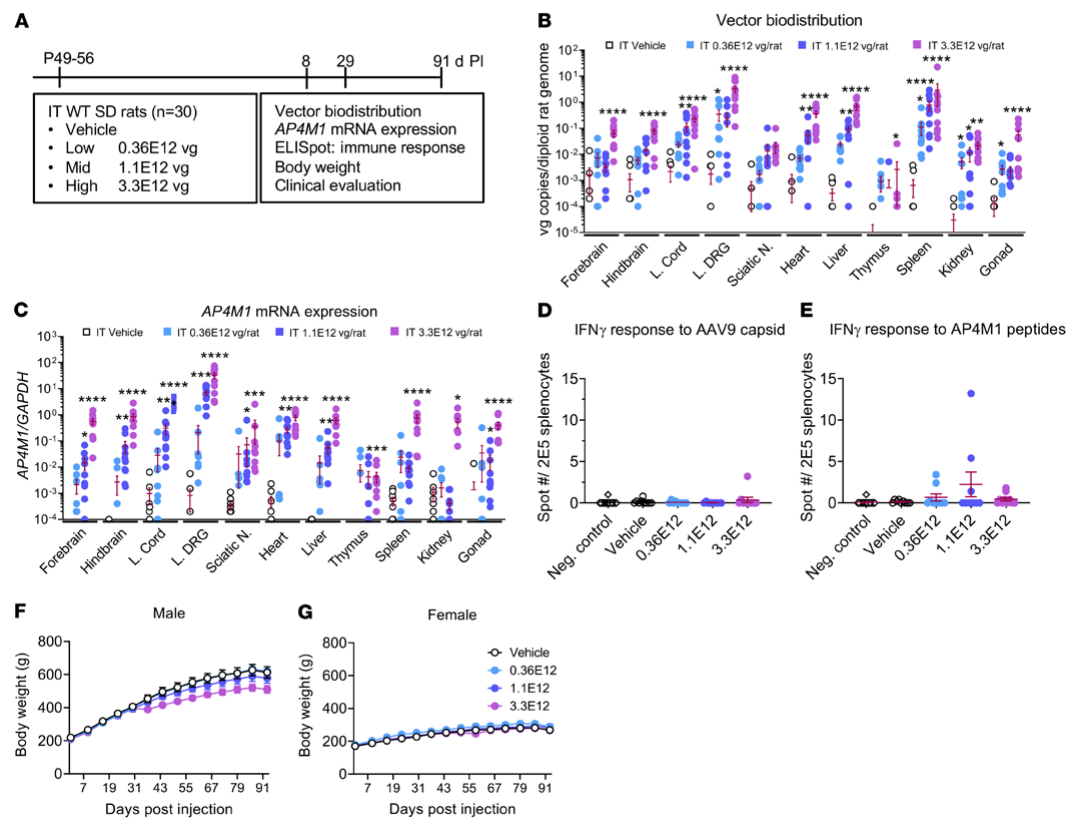
Experimental design for in vivo safety study in WT rats and vector biodistribution, *AP4M1* mRNA expression, effects on IFN-γ responses to AAV9 capsid or AP4M1 peptides, and body weight in i.t. AAV9/*AP4M1*-treated rats. (**A**) Vehicle or low (0.36 × 10^12^ vg/rat), mid (1.1 × 10^12^ × 10^12^ vg/rat), or high (3.3 × 10^12^ vg/rat) doses of AAV9/*AP4M1* vector were administered intrathecally to male and female rats at P49–P56 (*n* = 5/group/sex). Study readouts at each time point and specified ages are listed from left to right. At 29 days after injection, rat organs were harvested for (**B**) measurement of vector biodistribution and (**C**) *AP4M1* mRNA expression by qPCR. Rat splenocytes were prepared for IFN-γ responses to (**D**) AAV9 capsid or (**E**) AP4M1 peptides by ELISpot. (**F** and **G**) Rat body weights were monitored regularly up to 91 days after injection. All data in **B–G** are presented as the mean ± SEM. (**B–E**) Data sets that passed tests for normality or homogeneity of variance were analyzed using 1-way ANOVA, with α set at 0.05, and Dunnett’s correction for relevant pairwise comparisons. Data sets that did not pass tests for normality or homogeneity of variance were analyzed using Kruskal-Wallis test, with α set at 0.05, and Dunn’s correction for relevant pairwise comparisons. *P < 0.05, **P < 0.01, ***P < 0.001, ****P < 0.0001 compared with i.t. vehicle. (**F** and **G**) Two-way ANOVA with repeated measures was used for statistical analysis of male and female rat body weight. AAV9, adeno-associated virus 9; AP4M1, adaptor protein complex, subunit μ4; i.t., intrathecal; L. Cord, lumbar spinal cord; L. DRG, lumbar dorsal root ganglion; Sciatic N., sciatic nerve; Neg, negative; PI, postinjection; SD, Sprague-Dawley; vg, vector genome.

To demonstrate the safety and biodistribution pattern of the AAV9/*AP4M1* treatment, a GLP study was conducted in WT SD rats (Figure 7A). Male and female rats were randomized into cohorts, with 5 male and 5 female rats per cohort, and dosed by a qualified laboratory technician. At the initiation of dosing, the animals assigned to the study were 49–56 days old and were injected i.t. with a single dose of 0.36 × 10^12^, 1.1 × 10^12^, or 3.3 × 10^12^ vg/rat (Figure 7A). All animals were monitored for up to 91 days following the injection for mortality, body weight, food consumption, clinical/behavioral changes, clinical pathology parameters (hematology, coagulation, clinical chemistry, and urinalysis), splenocyte analysis (ELISpot), tissue gene expression, organ weights, and macroscopic/microscopic examinations. Rats were sacrificed on days 8, 29, or 91 after injection. i.t. delivery of AAV9/*AP4M1* vector resulted in dose-dependent increases of *AP4M1* vector DNA across the CNS (brain and spinal cord) and peripheral organs (heart, lung, liver, kidney, ovary, and testes) (Figure 7B). Similarly, the *AP4M1* vector DNA was widely detected at high levels in multiple brain regions. In the peripheral organs, similar high amounts of *AP4M1* DNA persisted in the heart, liver, and spleen and, to a lower extent, in other organs tested. Consistent with this *AP4M1* DNA biodistribution data, *AP4M1* transgene expression was also widely and readily detected in multiple CNS and peripheral tissues (Figure 7C). Collectively, i.t. delivery of AAV9/*AP4M1* resulted in broad *AP4M1* biodistribution and expression across the rat body.

To evaluate T cell IFN-γ immune responses to the AAV9/*AP4M1* vector, splenocytes from WT rats i.t. injected with AAV9/*AP4M1* vectors 29 days before were plated and treated in vitro with either AAV9 capsid or AP4M1 protein peptide pools for 2 days, along with both negative (no peptide) or positive (PMA + ionomycin) controls. While the negative controls had 0 spots, none of the splenocytes from rats injected with vehicle or low (0.36 × 10^12^ vg), mid (1.1 × 10^12^ vg), or high (3.3 × 10^12^ vg) doses of AAV9/*AP4M1* vector displayed a significant increase in spots compared with the negative controls. This indicates that the AAV9/*AP4M1* vector generated minimal T cell immune response to either AAV9 or the human AP4M1 protein in WT rats (Figure 7, D and E).

There was no AAV9/*AP4M1*-related mortality or effect on food consumption, clinical changes, pathological urinalysis parameters, macroscopic findings, or organ weights over the duration of the study. AAV9/*AP4M1*-related significant (*P* < 0.05) decreases in group mean body weight (17% decrease compared with controls) were noted in male rats at 3.3 × 10^12^ vg starting from day 35 and persisting until the end of the study (Figure 7F). This was also reflected in a decreased overall mean body weight gain from day 28 to 84 (24% lower than controls). The changes in male rats were related to slower weight gain after day 28 compared with controls, rather than weight loss. There were no AAV9/*AP4M1*-related decreases in the group mean body weight in female animals (Figure 7G).

Other adverse findings included neuronal degeneration noted microscopically in the lumbar dorsal root ganglion (DRG) at equal to or more than 1.1 × 10^12^ vg (Supplemental Figure 4), increased excitability and/or activity at 3.3 × 10^12^ vg, and changes in clinical pathology parameters at equal to or more than 0.36 × 10^12^ vg. Due to the neuronal degeneration, the no-observed-adverse-effect level (NOAEL) was considered as 0.36 × 10^12^ vg (Supplemental Data File 3, report from Charles River Laboratories [CRL], 5550008). Taken together, i.t. AAV9/*AP4M1* doses up to 3.3 × 10^12^ vg/rat were deemed to have an acceptable tolerability, especially in the context of a disorder such as SPG50 with no alternative disease-modifying treatments available.

### AAV9/AP4M1 gene therapy was safe and well tolerated in WT NHPs in a GLP study.

**Figure 8:**
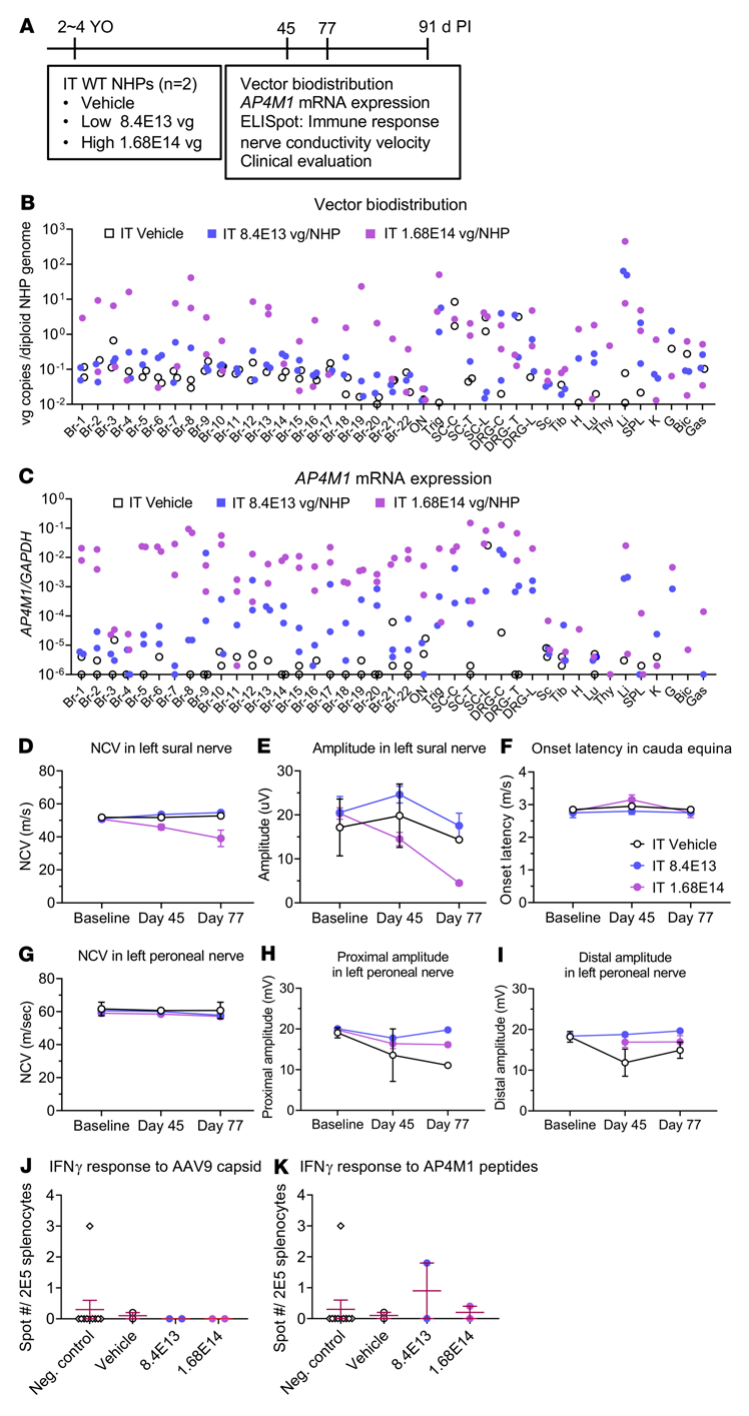
Experimental design for in vivo safety study in WT NHPs and vector biodistribution, *AP4M1* mRNA expression, effects on NCV or amplitude in peripheral nerves, and IFN-γ responses to AAV9 capsid or AP4M1 peptides in i.t. AAV9/*AP4M1*-treated NHPs. (**A**) Vehicle or low (8.4 × 10^13^ vg/NHP), or high (1.68 × 10^14^ vg/NHP) doses of AAV9/*AP4M1* vector were administered intrathecally to NHPs at 2–4 years of age (*n* = 2/group). Study readouts at each time point and specified ages are listed from left to right. At 91 days after injection, monkey organs were harvested for (**B**) measurement of vector biodistribution and (**C**) *AP4M1* mRNA expression by qPCR. (**D–I**) NCV tests were performed at baseline and day 45 and day 77 after injection. Monkey splenocytes were prepared for IFN-γ responses to (**J**) AAV9 capsid or (**K**) AP4M1 peptides by ELISpot. Each dot in **B** and **C** represents an individual monkey. All data in **D–K** are presented as the mean measurement ± SEM. AAV9, adeno-associated virus 9; AP4M1, adaptor protein complex, subunit μ4; i.t., intrathecal; YO, years old; PI, postinjection; NHPs, nonhuman primates; vg, vector genome; Br, brain; ON, optic nerve; Trig, trigeminal ganglion; SC-C, cervical spinal cord; SC-T, thoracic spinal cord; SC-L, lumbar spinal cord; DRG-C, cervical dorsal root ganglion; DRG-T, thoracic dorsal root ganglion; DRG-L, lumbar dorsal root ganglion; Sc, sciatic nerve; Tib, tibia nerve; H, heart; Lu, lung; Thy, Thymus; Li, liver; SPL, spleen; K, kidney; G, gonad; Bic, biceps femoris; Gas, gastrocnemius; NCV, nerve conduction velocity; Neg, Negative.

To further investigate the safety and biodistribution pattern of the AAV9/*AP4M1* vector in a larger animal model, a GLP study was conducted in WT cynomolgus monkeys (Figure 8A). Male and female NHPs were randomized into cohorts, with 2 NHPs per cohort, and dosed by a qualified laboratory technician. At the initiation of treatment, the animals assigned to the study were 2–4 years old and were administered a single i.t. injection at doses of 8.4 × 10^13^ or 1.64 × 10^14^ vg/NHP (Figure 8A). A 1 mL sample of cerebrospinal fluid (CSF) was withdrawn from each animal immediately prior to vector injection to verify needle placement and avoid elevating intracranial pressure. Furthermore, all animals received i.v. methylprednisolone starting on day 1 until the study termination (10 mg/kg/d on day 1 and 1 mg/kg/d thereafter) and rapamycin starting 12 days prior to injection until the termination of the study (0.01 mg/kg, twice daily). All animals were monitored up to 91 days following the injection for mortality, body weight, food consumption, clinical/behavioral changes, neurological examinations, nerve conduction velocity (NCV) evaluation, clinical pathology parameters (hematology, coagulation, clinical chemistry, and urinalysis), cytokines analysis, vector DNA biodistribution, transgene expression, splenocyte analysis (ELISpot), organ weights, and macroscopic/microscopic examinations.

There was no mortality over the duration of the study. There were also no AAV9/*AP4M1*-related adverse clinical observations or effects on body weight, appetence, hematology, clinical chemistry, or urinalysis parameters. Furthermore, there were no changes in blood IL-6, IL-10, TNF-α, MCP-1, and IP-10 levels; no macroscopic findings; and no abnormal organ weights. However, we observed AAV9/*AP4M1*-related increases in blood IL-8 in female animals treated with ≥ 8.4 × 10^13^ vg. This change was considered not adverse due to its low magnitude.

i.t. delivery of AAV9/*AP4M1* vector in NHPs resulted in distribution of *AP4M1* vector DNA across the CNS and peripheral organs (Figure 8B and Supplemental Figure 5). The *AP4M1* vector DNA was widely and readily detected in multiple brain regions. In the peripheral organs, higher levels of *AP4M1* DNA persisted in liver and, to a lesser extent, in other organs. Consistent with this *AP4M1* DNA biodistribution data, *AP4M1* transgene expression was also widely and readily detected in multiple CNS and peripheral tissues (Figure 8C).

In the peripheral nerves (sciatic, sural, and tibial) and DRG (Supplemental Figure 6), we observed an increase in the severity of axonal degeneration at 1.68 × 10^14^ vg in both sexes. The degeneration in the nerves correlated with a decrease in NCV and response amplitude of the sural nerve noted on days 45 and 77 (Figure 8, D and E), but there were no changes in onset latency in the cauda equina (Figure 8F) or with NCV and amplitude in the peroneal nerve (Figure 8, G–I).

To evaluate any T cell IFN-γ immune response to the AAV9/*AP4M1* vector, splenocytes from all NHPs were plated and treated in vitro with either AAV9 capsid or AP4M1 protein peptide pools for 2 days, along with both negative (no peptide) and positive (PMA + ionomycin) controls. While the negative controls had less than 3 spots, none of the splenocytes from NHPs injected with vehicle or low (8.4 × 10^13^ vg) or high (1.68 × 10^14^ vg) doses of AAV9/*AP4M1* vector generated any increase in spots compared with the negative controls. This indicates that the AAV9/*AP4M1* vector did not generate a detectable T cell immune response to either AAV9 or the human AP4M1 protein in WT NHPs under the immunosuppressant protocol (Figure 8, J and K).

In conclusion, i.t. administration of AAV9/*AP4M1* by a single i.t. injection in cynomolgus monkeys was well tolerated at 8.4 × 10^13^ vg. Adverse findings at 1.68 × 10^14^ vg included axonal or neuronal degeneration observed microscopically in the spinal cord (including the injection site), lumbar DRG, dorsal nerve roots, brain, trigeminal ganglion, and peripheral nerves (sciatic, sural, and tibial), with associated decreases in NCV and neurological effects. Based on these results, the NOAEL was considered as 8.4 × 10^13^ vg (Supplemental Data File 2, report from CRL, 5550014).

### Similar degrees of minimal-to-mild DRG toxicity were observed in both rat and NHP toxicology studies.

In the rat toxicology study, one of the main AAV9/*AP4M1*-related findings on day 8 was in the lumbar DRG, where minimal mononuclear cell infiltration was noticed in male and female animals at equal to or more than 1.1 × 10^12^ vg/rat (Table 1 and Supplemental Figure 4). These findings were, in general, more severe on day 29 and day 91 compared with day 8, coupled with increased frequency and/or severity of minimal-to-mild axonal and neuronal degeneration. Most microscopic findings, however, showed decreased abundance and/or severity at day 8 compared with day 29, with a trending pattern toward resolution of the microscopic findings by day 91. In the NHP toxicology study, there were minimal mononuclear cell infiltrates in lumbar DRG in all AAV9/*AP4M1*-dosed animals at equal to or more than 8.4 × 10^13^ vg (Table 1 and Supplemental Figure 6). Minimal neuronal degeneration was also noticed in both male and female animals at 1.64 × 10^14^ vg. The latter change was characterized by the effacement/loss of rare neuronal cell bodies with presence of glial and/or mononuclear cells. Taken together, similar degrees of minimal-to-mild toxicity in lumbar DRG were observed in both rat and NHP toxicity studies.

## Discussion

Recombinant AAV9-mediated gene therapy has been extensively used in preclinical and clinical studies for the treatment of CNS disorders. Efficacy with AAV9 has been demonstrated in numerous preclinical models of CNS disorders and in an increasing number of clinical studies using an i.t. administration route (20, 29, 39, 40). Here, we tested the feasibility and efficacy of an AAV-based strategy to deliver the codon-optimized human *AP4M1* gene in fibroblasts from patients with SPG50 in vitro, as well as in vivo in *Ap4m1-*KO mice, to investigate whether this would predict a benefit to pediatric patients with SPG50 disease.

Proof-of-concept in vitro studies by DEF’s group demonstrated that transduction of patient-derived fibroblasts with AAV2/*AP4M1* resulted in a phenotypic rescue, including restoration of ATG9A trafficking and increased AP4E1 levels. While cells treated at an MOI of 1 × 10^4^ showed no toxicity, treatment at an MOI of 1 × 10^5^ led to a reduction in cell survival, suggesting that high amounts of cellular AP4M1 expression are capable of driving toxicity. Similar efficacy results were obtained independently in studies by JSB’s group using 2 additional fibroblast cell lines from 2 sibling patients with SPG50. However, these fibroblasts did not show any sign of toxicity following infection with AAV2/*AP4M1*, even at the MOI of 1 × 10^5^. Although there was a discrepancy regarding the cell toxicity at high dose of AAV2/*AP4M1*, both groups generated supportive proof-of-concept efficacy data, indicating that this vector design is capable of rescuing cellular disease phenotypes in cultured patient fibroblasts.

In an in vivo efficacy study, *Ap4m1*-KO mice were treated i.t. with low (1.25 × 10^11^ vg), mid (2.5 × 10^11^ vg), or high (5 × 10^11^ vg) doses of AAV9/*AP4M1* at P7–P10 (cohort before disease manifestation) or P90 (cohort with early disease manifestation). We view treatment at P7–P10 to model the best-case scenario of earliest possible intervention in humans, whereas treatment at P90 addresses whether our treatment can halt and/or reverse disease progression. Data collected from the efficacy study demonstrated clear age- and dose-dependent effects with early intervention and high dose achieving the best therapeutic benefits. Considering that (a) there were no signs of morbidity and no differences in weight or survival seen in the treated groups compared with the untreated controls and (b) treated mice at both premanifesting and early manifesting stages displayed partially improved phenotypic deficits, these data from *Ap4m1*-KO mice suggest a favorable long-term efficacy and safety profile of AAV9/*AP4M1* out to over 1-year after injection. Taken together, the efficacy study demonstrated dose-dependent expression of *AP4M1* mRNA across both sexes, with a dose of 5 × 10^11^ vg providing the best, albeit partial, normalization of behavioral phenotypes and a lower dose of 2.5 × 10^11^ providing significant, albeit lower, benefit to the mice. Of note, the lowest dose of 1.25 × 10^11^ vg in mice did not provide a clear behavioral benefit, indicating that 2.5 × 10^11^ vg is the minimally effective AAV9/*AP4M1* dose in mice. It should be noted that even though clear benefits to treatment were observed, even the highest dose did not completely normalize all behavioral phenotypes of the mice.

Studies in mice and NHPs have demonstrated a widespread distribution of the transgene across the spinal cord, DRG, and brain after a single i.t. injection of the AAV9 vector carrying the reporter gene GFP (24, 41). Compared with an i.v. route of administration, the i.t. route directs a higher percentage of the vector to the CNS relative to that of peripheral organs (24–26, 41–43). Moreover, the i.t. approach is scalable to humans and reduces the risk of low-to-moderate levels of systemic anti-AAV9 neutralizing antibodies as well as transgene overexpression in peripheral organs. Qualitative mRNA expression analysis (RNAscope) in both *Ap4m1*-KO mouse models and WT mice, as well as quantitative biodistribution and expression of AAV9/*AP4M1* in WT rats and NHPs following i.t. AAV9/*AP4M1* administration, demonstrated that the distribution and expression of transgene is comparable to what has been observed in other i.t. studies (20, 29, 39, 40). As we did not directly compare the i.t. route to an i.v. administration in this study, we cannot speculate whether additional biodistribution to peripheral organs could have led to additional treatment benefits. Combining the i.t. dose with i.v. administration, optimizing vector administration approaches, and/or an improved AAV capsid represent possible avenues to increase treatment efficacy. Conversely, we must consider that the lack of complete rescue of the mouse model at the high dose and early intervention observed in this study may indicate (a) the ceiling of AAV9’s ability to fully rescue a disorder when delivering a gene that acts in a cell-autonomous manner, unless further steps are taken to improve the gene transfer technology, and/or (b) any potential developmental defects that cannot be corrected postnatally at P7–P10.

In parallel to the in vivo efficacy study, three toxicology studies, all in WT animals, were conducted to evaluate the safety of AAV9/*AP4M1* administration. These included a non-GLP 1-year study in C57BL/6J mice, a GLP 3-month study in SD rats, and a GLP 3-month study in NHPs. All three studies demonstrated an acceptable safety profile for AAV9/*AP4M1* up to a target “human equivalent” dose (HED) of 1 × 10^15^ vg based on CSF volume for each species (see below). Some adverse effects were noted at the highest dose (HED > 1 × 10^15^ vg), such as (a) hepatocellular adenomas in the mouse study at 12 months after administration; (b) increased excitability, increased activity, and decreased body weight in the rat study at 12 weeks after administration at the highest dose and neuronal degeneration in the lumbar DRG at higher doses with no recovery; and (c) decreased NCV that occurred in the sural nerve at the 1.68 × 10^14^ vg dose in the NHP study, consistent with dose-dependent damage to sensory but not motor neurons. It is worth noting that additional safety data were collected from our efficacy study in *AP4M1-*KO mice up to 20.5 months after injection, and this study did not show any increased incidence of hepatoxicity (including liver tumors) for any treatment group (Supplemental Table 3). While some adverse findings were noted across the safety studies, those occurring at a HED of equal to or less than 1 × 10^15^ total vg were deemed acceptable in the context of SPG50, given the severity of the disease and profound unmet patient need. Taken together, these preclinical results identify an acceptably safe and efficacious i.t.-delivered HED of 1 × 10^15^ total vg AAV9/*AP4M1*, supporting AAV9/*AP4M1* gene replacement therapy as a viable treatment strategy for the treatment of SPG50.

There is still some debate regarding the evidence of increased cancer risk associated with AAV vectors (44–47). One large-scale study in mice found no evidence for tumorigenesis following AAV administration (48), despite other studies having found evidence for limited AAV integration (49, 50). While clonal integration of WT AAV2 has been detected in patient hepatocellular carcinomas (51), integration and increased cancer risk from AAV has not been identified in a clinical setting, and it is considered a very low risk for the proposed clinical trial (52). Moreover, our vector utilized a relatively weaker promoter, UsP, which drives approximately 15% of the transgene activity compared with a CMV promoter (31) and is thus expected to lessen the risk of transactivation/overexpression of tumor-promoting factors if the vg integrates nearby (50). Although several WT mice treated with the high dose for 12 months had liver tumors in the non-GLP mouse safety study, none of the *Ap4m1*-KO mouse treatment groups had an increased incidence of liver abnormalities (including tumors) at 20.5 months after injection. Taking the studies in WT mice and *Ap4m1*-KO mice together, along with the absence of any tumors in the shorter-term studies in rats and NHPs, our data do not indicate any clear oncogenesis risk of the AAV9/*AP4M1* treatment.

An identified risk of inflammatory damage to DRG has been reported from recent nonclinical studies in NHPs as well as piglets (53–55) and is an emerging safety concern for CNS-directed AAV gene therapy. In a study assessing i.t. administration of AAV across over 200 NHPs in one center, minimal-to-mild DRG histopathology was found consistently across most animals, independent of the transgene (54). However, the same study noted minimal correlations to any adverse behavioral symptoms or physiological biomarkers such as altered nerve conduction. In a follow-up report, incorporation of a miRNA binding site specific to DRG resolved this histopathology, suggesting that high transgene expression was driving the toxicity in DRG (55). Aside from NHPs and the single study in piglets, it has been unclear if other animals could appropriately model this drug class effect to the DRG. Without a better understanding of this specific type of toxicity and how it could be modeled, one mitigation strategy would be to conduct an NHP toxicology study for every i.t. AAV product before consideration for human use. Interestingly, we noticed a similar occurrence and severity of DRG toxicity in both rat and NHP toxicity studies, which advances the knowledge in this field and supports the possibility of using the rat model to monitor DRG toxicity in future studies. We view this as an important finding, as it suggests that NHP studies may not be a requisite to model the possibility of this specific toxicity from AAV.

All our efficacy and safety data were gathered in rodents and NHPs. To calculate the HED, we extrapolated the doses by CSF volume assuming the following normative volumes for each species: 0.035 mL for mouse, 0.25 mL for rat, 12 mL for NHP, and 140 mL for human. The efficacy studies in mice predicted a benefit to mice at 2.5 × 10^11^ vg, equivalent to the target human dose of 1 × 10^15^ vg. In terms of safety, studies in mice up to 5 × 10^11^ vg (HED of 2 × 10^15^ vg) were tolerated well up to 1-year after injection with no significant drug-related effects. Studies in rats found increased activity, reduced weight, and histopathological findings at 3.3 × 10^12^ vg (HED of 1.8 × 10^15^ vg), whereas lower doses were better tolerated. Studies in NHPs found reduced sensory conduction at 1.68 × 10^14^ vg (HED of 2.2 × 10^15^ vg) with associated microscopic changes but otherwise showed minimal in-life adverse effects, and the lower dose of 8.4 × 10^13^ (HED of 1.1 × 10^15^ vg) was well tolerated. Overall, the toxicology studies across three species provide safety data up to an approximately 2-fold overdose in the HED. While the preclinical toxicology studies predicted the possibility of some DRG-specific pathology at the human 1 × 10^15^ vg dose, this HED was not associated with adverse clinical findings in the animals. Considering AAV9/*AP4M1* as a one-time treatment for this severe neurological condition without an option to redose, a proposed dose of 1 × 10^15^ vg for patients at or older than 3 years of age should maximize benefit with acceptable risks.

In conclusion, the results obtained in this study demonstrate that AAV9/*AP4M1* is both effective and acceptably tolerated in preclinical models, providing proof-of-concept evidence that AAV9/*AP4M1* gene therapy should be considered for human translation. Because the CSF volume of humans is relatively static after 3 years of age (56), we suggest a final target dose of 1 × 10^15^ vg total vg in 10 mL in patients with SPG50 at or older than 3 years of age. If patients younger than 3 years require treatment, it might be appropriate to scale down the dose to account for reduced brain and CSF volume. It is worth noting that AP4M1 is not a secreted protein and that SPG50 disease is not thought to benefit from cross-correction. Thus, the conclusions from this study may translate to other AAV9-based gene replacement strategies for related neurological disorders involving genes with cell-autonomous effects. An investigational phase I i.t. gene transfer trial using 1 × 10^15^ vg per patient of AAV9/*AP4M1* to treat SPG50 was approved by Health Canada on December 30, 2021, and by the FDA on August 11, 2022 (NCT05518188). All of the preclinical efficacy and safety data (including GLP toxicology study reports) that supported the approved Canadian clinical trial agreement and FDA investigational new drug application are provided within this paper and its supplemental materials. Our hope is that this information could help provide a road map for other similar CNS-directed gene therapy approaches to translate into clinical trials.

## Methods

### Plasmid design and development.

We designed and developed the UsP-*hAP4M1opt*-BGHpA plasmid (Figure 1A) containing the transgene of a human *AP4M1* codon-optimized construct (*hAP4M1opt*). The transgene consists of a human *AP4M1* codon-optimized DNA-coding sequence of 1,362 bp (ATUM) between a 328 bp UsP promoter and a 254 bp BGHpA polyadenylation signal. The UsP promoter is a combination of the minimal synthetic JeT promoter (20, 29, 30) and a synthetic intron sequence (31), which is utilized in this study for its small size, which allows for packaging into an scAAV vector as well as to mediate a moderate level of ubiquitous transgene expression (31).

### scAAV2/AP4M1 and scAAV9/AP4M1 vector preparation.

The UsP-*hAP4M1opt*-BGHpA plasmid was packaged into scAAV2 and scAAV9 vectors (57), which are 10–100 times more efficient at transduction compared with traditional single-stranded AAV vectors (58, 59). The scAAV2/*AP4M1* vector (lot 8,829; 2.81 × 10^12^ vg/mL) was produced at the UNC-VC for all in vitro cell culture studies. The scAAV9/*AP4M1* vector (lot LAV-112-conc; 1.03 × 10^14^ vg/mL with 88% full capsid) was produced at UNC-VC for all in vivo toxicity and efficacy studies in mice. The AAV2 and the AAV9 vectors made at UNC-VC were titered by qPCR and confirmed by silver staining compared with an internal reference standard at UNC-VC (60). Another scAAV9/*AP4M1* vector (lot T-GEMINIS-033; 5.43 × 10^13^ vg/mL, 84% full capsids) was produced at Viralgen for all in vivo toxicity studies in WT rats and monkeys. The T-GEMINIS-033 lot was titered by Viralgen’s ITR-based ddPCR titering assay; using this ddPCR method, the LAV-112-conc lot showed a titer of 9.9 × 10^13^ vg/mL. The vectors produced at Viralgen underwent additional quality control release testing. The quality control summaries of the scAAV9/*AP4M1* vectors are included in the Supplemental Methods.

### In vitro SPG50 patient fibroblast culture and treatment.

DEF’s group tested the AAV2/*AP4M1* vector in fibroblast lines from 3 different patients with SPG50 and Het controls (same sex parent) (Supplemental Table 1). For high-content imaging and automated image analysis, high-throughput confocal imaging was performed using the ImageXpress Micro Confocal Screening System (Molecular Devices) using an experimental pipeline described in Behne et al. (61) and Ebrahimi-Fakhari et al. (32). Fibroblasts were treated in 96-well microplates, subjected to confocal microscopy using a high-content imager, and analyzed using an automated image analysis pipeline. The analysis pipeline identified cells based on presence of a DAPI-positive nucleus inside a phalloidin-positive (actin) area. Different masks were generated: an actin mask to outline the cell, a TGN46 mask to outline the area of the trans-Golgi network, and an ATG9A mask based on intracellular ATG9A fluorescence. ATG9A fluorescence intensity was measured within the TGN mask as well as the actin-positive cytoplasm outside the trans-Golgi network (a mask generated by subtracting the TGN46 mask from the actin mask). The ATG9A ratio was calculated for each cell by dividing the ATG9A fluorescence inside and outside of TGN. JSB’s group tested the vector in fibroblast lines from 2 sibling patients with a donor splice site pathogenic mutation in intron 14 of the *AP4M1* gene (c.1137+1G→T) (patient 1, 87RD38, and patient 2, 87RD39) (3, 33) comparing them with skin fibroblasts from an individual who served as a control (85E0344) (see Supplemental Methods).

### Ap4m1-KO mice.

The *Ap4m1*-KO mouse model was generated through targeted mutation 1b by Wellcome Trust Sanger Institute (http://www.informatics.jax.org/allele/MGI:5636962) and recovered by The Jackson Laboratory. Briefly, the critical exons 5–12 were flanked by loxP sites, and subsequent cre expression excised this critical sequence, resulting in an *Ap4m1*-KO mouse. Genotyping details are provided in Supplemental Methods.

### Efficacy study plan in Ap4m1-KO mice.

The experimental design for the in vivo efficacy study is summarized in Figure 3A. In brief, both male and female *Ap4m1*-KO mice were randomized into treatment cohorts and injected i.t. at P7–P10 (cohort before disease manifestation) or P90 (cohort with early disease manifestation). For i.t., 5 μL vehicle or 1.25 × 10^11^, 2.5 × 10^11^, or 5 × 10^11^ vg/mouse of AAV9/*AP4M1* vector was administrated via lumber i.t. injection. WT and Het mice without treatment were used as normal controls. All mice were weighed regularly and observed for overt signs of adverse effects at the times of weighing. The survival rate was calculated, and all unexpected deaths were investigated by the UT Southwestern Medical Center veterinary staff. Starting at 5 months of age, behavioral testing was carried out on all study cohorts and then repeated at 8, 12, and 17 months after dosing. A battery of behaviors, focusing mainly on motor function and including hind limb clasping, elevated plus maze, and open-field tests, was assessed blindly through the mouse behavior phenotyping core facility. At 3 weeks after injection, subsets of mice treated at P90 from each group were sacrificed to evaluate human *AP4M1* mRNA expression by RNAscope. Splenocytes and sera were used for ELISpot assays and the measurement of toxicology markers, respectively. All remaining mice were maintained to evaluate long-term survival and safety until 20.5 months of age, when all major organs were harvested for archive or further analyses.

### Tissue preparation for RNAscope staining and image analysis, ELISpot analysis, and behavioral tests.

See Supplemental Methods for detailed information.

### Non-GLP safety study in WT BL/6J mice.

The non-GLP study presented in Figure 6A was designed to identify any long-term safety issues associated with the experimental therapy. The mice were randomized to different groups (*n* = 10/group/sex) and injected i.t. with 5 μL vehicle or different doses (1.25 × 10^11^, or 5 × 10^11^ vg/mouse) of AAV9/*AP4M1* vector. Mice were monitored following the treatment, and appropriate supportive or therapeutic interventions were offered. A detailed necropsy was performed to investigate the reason for the ailment. Mouse sera were collected for a serum toxicity panel. Tissue samples including brain, heart, liver, lung, gonad, spleen, kidney, eyeball, sciatic nerve, cervical spinal cord, and lumbar spinal cord at 12 months following the treatment were collected for RNAscope staining and histopathological assessment (Supplemental Methods).

### GLP safety study in WT SD rats.

This animal study was performed at CRL Inc. Male and female SD rats were randomized into cohorts (*n* = 5/group/sex) and dosed as shown in Figure 7A. At the initiation of dosing, the animals assigned to the study were 49–56 days old. The AAV9/*AP4M1* vector was injected i.t. once in each animal by a qualified laboratory technician, in a volume of 20 or 60 μL and a final dose of 0.36 × 10^12^, 1.1 × 10^12^, or 3.6 × 10^12^ vg/rat. All animals were monitored up to 91 days following the injection. Rats were sacrificed on day 8, 29, or 91 after injection; splenocytes were prepared for ELISpot analysis; and tissues were collected for vector biodistribution, *AP4M1* expression, and toxicity evaluation (Supplemental Methods).

### GLP safety study in WT NHPs.

This animal study was also performed at CRL Inc. Cynomolgus monkeys were randomized into cohorts (*n* = 2/group) and dosed as shown in Figure 8A. At the initiation of dosing, the animals assigned to the study were 2–4 years old. The AAV9/*AP4M1* vector was injected i.t. once in each animal by a qualified laboratory technician in a final dose of 8.4 × 10^13^ or 1.68 × 10^14^ vg/monkey. One mL of CSF was withdrawn from each animal immediately prior to vector injection, which verified needle placement. All animals were monitored up to 91 days following the injection. NCV testing was performed at baseline, day 45, and day 77 after injection. Monkeys were sacrificed on day 91 after injection, splenocytes were prepared for ELISpot analysis, and tissues were collected for vector biodistribution, *AP4M1* expression, and toxicity evaluation (Supplemental Methods).

### Statistics.

All quantitative data in this paper are presented as mean ± SEM and were analyzed and graphed using GraphPad Prism Software (v. 9.2.0). Data were tested for normal distribution (Shapiro-Wilk normality test) and homogeneity of variance (Brown-Forsythe test). Data sets that passed these two tests were analyzed using the Student’s 2-tailed unpaired *t* test for two-group comparisons or 1-way ANOVA for comparisons of three or more groups, with Dunnett’s correction for relevant pairwise comparisons. Data sets that did not pass tests for normality or homogeneity of variance were analyzed using the Mann-Whitney test for two-group comparisons or Kruskal-Wallis test with Dunn’s correction for relevant pairwise comparisons. Two-way ANOVA with repeated measures was used for the analyses of time course data, including body weight, behavior results, mRNA expression, and serum toxicity panel. For survival analysis, data shown in the Kaplan-Meier survival curves were compared with the log-rank (Mantel-Cox) test. A *P* value of less than 0.05 was considered as significant for all statistical analyses.

### Study approval.

All research working with mice was approved by the IACUC of the UT Southwestern Medical Center. The GLP toxicology studies performed in live animals at CRL were approved by their IACUC. Fibroblasts from patients (patient 1, 87RD38, and patient 2, 87RD39) and an individual who served as a control (85E0344) used in JSB’s lab were a gift from Grazia Mancini (Erasmus Medical Center, Rotterdam, The Netherlands) and were obtained according to Erasmus Medical Center Institutional Review Board requirements (METC-2012387).

## Author contributions

XC and SJG designed the experiments, coordinated studies with collaborators and core facilities, and wrote the manuscript. XC, TD, YH, RDP, RM, KE, MZ, MS, JSB, and DEF performed the experiments. XC, TD, YH, RDP, RM, KE, MZ, and MS analyzed all data and prepared all figures for the manuscript. YH, RDP, RM, KE, MZ, MS, JSB, TP, and DEF helped prepare and review the manuscript. TP was instrumental in coordinating key aspects of the project, including AAV manufacture and GLP toxicology studies. SJG oversaw all activities related to the project and acquired most of the funding for the work.

## Supplementary Material

Supplemental data

Supplemental data set 1

Supplemental data set 2

Supplemental data set 3

## Figures and Tables

**Table 1:**
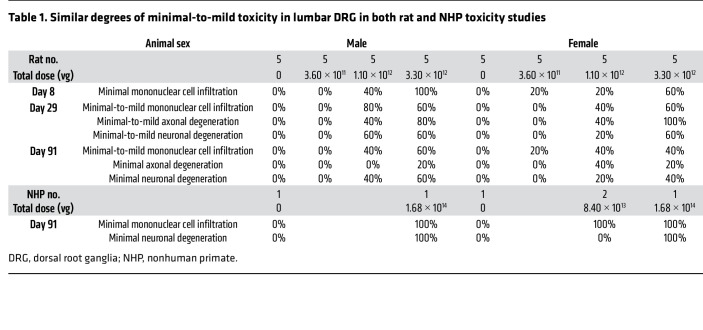
Similar degrees of minimal-to-mild toxicity in lumbar DRG in both rat and NHP toxicity studies

## Version 1 03/23/2023

In-Press Preview

## Version 2 05/15/2023

Electronic publication
