# Supplementary material for: Intrathecal AAV9/AP4M1 gene therapy for hereditary spastic paraplegia 50 shows safety and efficacy in preclinical studies
Source: J Clin Invest. 2023 May 15;133(10):e164575. doi: 10.1172/JCI164575 (PMC10178841; doi:10.1172/JCI164575)
Supplement: Supplemental data set 3 [file jci-133-164575-s060.pdf]

FINAL REPORT

Test Facility Study No. 5550008

Sponsor Reference No. UTSW.Gray-003

A Single-Dose Toxicity Study of AAV9/AP4M1 by Intrathecal Injection in Rats

GLP

SPONSOR:
University of Texas Southwestern Medical Center
5323 Harry Hines Blvd
Dallas, TX 75390
USA

TEST FACILITY:
Charles River Laboratories Montreal ULC
Senneville Site (CR-SEN)
22022 Transcanadienne
Senneville, QC H9X 3R3
Canada

TABLE OF CONTENTS

LIST OF FIGURES 3
LIST OF TABLES 4
LIST OF APPENDICES 5
QUALITY ASSURANCE STATEMENT 6
COMPLIANCE STATEMENT AND REPORT APPROVAL 8
1. RESPONSIBLE PERSONNEL 9
2. SUMMARY 10
3. INTRODUCTION 13
4. MATERIALS AND METHODS 13
4.1. Test Material Identification 13
4.2. Study Design 14
4.3. Deviations 15
5. COMPUTERIZED SYSTEMS 18
6. RETENTION OF RECORDS, SAMPLES, AND SPECIMENS 19
7. RESULTS 20
7.1. Mortality 20
7.2. Clinical Observations 20
7.3. Body Weights and Body Weight Gains 20
7.4. Food Consumption 21
7.5. Neurobehavioral Evaluation 21
7.6. Hematology 21
7.7. Coagulation 21
7.8. Clinical Chemistry 21
7.9. Urinalysis 22
7.10. Biodistribution, Gene Expression, and Splenocyte Analysis 22
7.11. Macroscopic Pathology 24
7.12. Organ Weights 24
7.13. Microscopic Evaluations 25
7.13.1. Terminal Euthanasia Animals (Day 8) 25
7.13.2. Recovery Euthanasia Animals (Day 29) 26
7.13.3. Recovery Euthanasia Animals (Day 91) 28
8. CONCLUSION 31
9. REFERENCES 32

LIST OF FIGURES

Figures 1-2 Summary of Body Weights 33

LIST OF TABLES

Table 1 Summary of Clinical Observations 35
Table 2 Summary of Body Weights 54
Table 3 Summary of Body Weight Gains 60
Table 4 Summary of Food Consumption 66
Table 5 Summary of Neurobehavorial Evaluation: Activity 71
Table 6 Summary of Neurobehavioral Evaluation: Autonomic 79
Table 7 Summary of Neurobehavioral Evaluation: Excitability 87
Table 8 Summary of Neurobehavioral Evaluation: Neuromuscular 95
Table 9 Summary of Neurobehavioral Evaluation: Physiological 107
Table 10 Summary of Neurobehavioral Evaluation: Sensorimotor 111

LIST OF APPENDICES

Appendix 1 Amended Study Plan and Study Plan....115
Appendix 2 Test and Reference Item Characterization 180
Appendix 3 Individual Mortality 196
Appendix 4 Individual Clinical Observations....204
Appendix 5 Individual Palpable Masses....230
Appendix 6 Individual Body Weights 231
Appendix 7 Individual Body Weight Gains....255
Appendix 8 Individual Food Consumption Values....279
Appendix 9 Individual Neurobehavioral Evaluation: Activity 296
Appendix 10 Individual Neurobehavioral Evaluation: Autonomic 304
Appendix 11 Individual Neurobehavioral Evaluation: Excitability 320
Appendix 12 Individual Neurobehavioral Evaluation: Neuromuscular 328
Appendix 13 Individual Neurobehavioral Evaluation: Physiological 352
Appendix 14 Individual Neurobehavioral Evaluation: Sensorimotor....360
Appendix 15 SOP-FOB 368
Appendix 16 Clinical Pathology Report 379
Appendix 17 Tissue Gene Expression Report 530
Appendix 18 Pathology Report....556
Appendix 19 Peer Review Certificate....948

QUALITY ASSURANCE STATEMENT

Study Number: 5550008

This Study has been audited by Quality Assurance in accordance with the applicable Good Laboratory Practice regulations. Reports were submitted in accordance with SOPs as follows:

		Dates Findings Submitted to:	
**Date(s) of Audit**	**Phase(s) Audited**	**Study Director**	**Test Facility Management**
11Feb2021	Final Study Plan	16Feb2021	16Feb2021
16Feb2021	Study Plan Amendment 01	23Feb2021	23Feb2021
16Feb2021	Addition of Study Plan to Provantis	17Feb2021	17Feb2021
22Feb2021	Dose Preparation	23Feb2021	23Feb2021
22Feb2021	Dose Administration	23Feb2021	23Feb2021
23Feb2021	Study Plan Amendment 02	24Feb2021	24Feb2021
26Feb2021	Study Plan Amendment 03	26Feb2021	26Feb2021
26Feb2021	Necropsy	26Feb2021	26Feb2021
26Feb2021	Cerebrospinal Fluid Collection	26Feb2021	26Feb2021
26Feb2021	Sample Collection	26Feb2021	26Feb2021
08Mar2021	Study Plan Amendment 04	09Mar2021	09Mar2021
14Jun2021 - 15Jun2021	Data Review - Bioanalysis & Immunology	15Jun2021	15Jun2021
21Jun2021	Study Plan Amendment 05	02Jul2021	02Jul2021
21Jun2021	Data Review - Sample Management	06Jul2021	06Jul2021
22Jun2021	Data Review - Clinical Pathology	06Jul2021	06Jul2021
22Jun2021	Data Review - Formulations	06Jul2021	06Jul2021
25Jun2021	Data Review - Animal Care	06Jul2021	06Jul2021
28Jun2021	Data Review - Veterinary Services	06Jul2021	06Jul2021
28Jun2021	Data Review - Shipping/Receiving	06Jul2021	06Jul2021
29Jun2021 - 30Jun2021	Data Review - Technical Operations	06Jul2021	06Jul2021
30Jun2021	Report Preparation	06Jul2021	06Jul2021
02Jul2021	Data Review - Necropsy	06Jul2021	06Jul2021
02Jul2021	Data Review - Histology	06Jul2021	06Jul2021
05Jul2021	Report Preparation	06Jul2021	06Jul2021

QUALITY ASSURANCE STATEMENT

Study Number: 5550008

This Study has been audited by Quality Assurance in accordance with the applicable Good Laboratory Practice regulations. Reports were submitted in accordance with SOPs as follows:

		Dates Findings Submitted to:	
**Date(s) of Audit**	**Phase(s) Audited**	**Study Director**	**Test Facility Management**
16Jul2021	Phase Report - Clinical Pathology	19Jul2021	19Jul2021
19Jul2021	Study Plan Amendment 06	26Jul2021	26Jul2021
23Jul2021	Report - Materials and Methods	26Jul2021	26Jul2021
03Aug2021	Report - Results	03Aug2021	03Aug2021
03Aug2021	Phase Report - Deviation Log	03Aug2021	03Aug2021
15Sep2021	Study Plan Amendment 07	17Sep2021	17Sep2021
28Feb2022	Final Report	28Feb2022	28Feb2022
28Feb2022	Data Review - Shipping/Receiving	28Feb2022	28Feb2022
01Mar2022	Final Phase Report - Pathology Peer Review	02Mar2022	02Mar2022
04Mar2022	Study Plan Amendment 08	07Mar2022	07Mar2022

In addition to the above-mentioned audits, process-based and/or routine facility inspections were also conducted during the course of this study. Inspection findings, if any, specific to this study were reported by Quality Assurance to the Study Director and Test Facility Management and listed as a Phase Audit on this Quality Assurance Statement.

The Quality Assurance Statements for any work conducted at Test Sites were reviewed and included in the appropriate section of this report, as applicable.

The Final Report has been reviewed to assure that it accurately describes the materials and methods, and that the reported results accurately reflect the raw data.

All electronic signatures appear at the end of the document upon finalization.

COMPLIANCE STATEMENT AND REPORT APPROVAL

The study was performed in accordance with the OECD Principles of Good Laboratory Practice and as accepted by Regulatory Authorities throughout the European Union, United States of America (FDA), Japan (MHLW), and other countries that are signatories to the OECD Mutual Acceptance of Data Agreement.

Any portion of this study conducted in the USA was performed in accordance with the US Department of Health and Human Services, Food and Drug Administration, United States Code of Federal Regulations, Title 21, Part 58: Good Laboratory Practice for Nonclinical Laboratory Studies and as accepted by Regulatory Authorities throughout the European Union (OECD Principles of Good Laboratory Practice), Japan (MHLW), and other countries that are signatories to the OECD Mutual Acceptance of Data Agreement.

SEND datasets were not subject to QA audit nor used as the basis for the Study Director interpretation of the final study results.

Exceptions from the above regulations are listed below.

- Characterization of the test and reference items was performed by the Sponsor subcontractor according to established SOPs, controls, and approved test methodologies to ensure integrity and validity of the results generated; these analyses were not conducted in compliance with the GLP or GMP regulations.
- Stability testing of the supplied test and reference items was not performed in this study although required by the Study Plan. This had no significant impact due to the known high degree of stability of AAV9 vector under the conditions of this study[1,2].
- Concentration, stability, and homogeneity of the test and reference item formulations were not determined in this study.
- Tissue bioanalysis, gene expression, and splenocyte analysis was performed by the Sponsor according to established SOPs, controls, and approved test methodologies to ensure integrity and validity of the results generated; these analyses were not conducted in compliance with the GLP regulations.
- A Principal Investigator for splenocyte analysis was not identified in the Study Plan, although analysis had already been performed. This deviation did not affect the overall integrity of the study as samples were analyzed as per Study Plan requirements.

This study was conducted in accordance with the procedures described herein. All deviations authorized/acknowledged by the Study Director are documented in the study records. The report represents an accurate and complete record of the results obtained.

There were no deviations from the above regulations that affected the overall integrity of the study or the interpretation of the study results and conclusions.

All electronic signatures appear at the end of the document upon finalization.

1. RESPONSIBLE PERSONNEL

Role/Phase	Quality Assurance Program (QAP)	Name	Contact Information
Study Director	Charles River	Stefania Cinquino, BSc	Address as cited for Test Facility
Test Facility Management	Charles River	Julie Douville, PhD	Address as cited for Test Facility
Test Facility QAP	Charles River	Nooshin Davani, BSc, RQAP-GLP	Address as cited for Test Facility
**Individual Scientist (IS)**			
Clinical Pathology	Charles River	Virginie Allegret, DVM, IPSAV, DES, DACVP	Address as cited for Test Facility
Immuno-toxicology Splenocyte Preparation [a]	Charles River	Philippe Rousseau, PhD	Address as cited for Test Facility
**Principal Investigator (PI)**			
Tissue Biodistribution Analysis (Bioanalysis)[b]	N/A	Steven Gray, PhD	Address as cited for Sponsor
Tissue Gene expression Analysis[b]	N/A	Steven Gray, PhD	Address as cited for Sponsor
Splenocyte Analysis[b]	N/A	Steven Gray, PhD	Address as cited for Sponsor
Pathology[c]	Charles River Frederick, MD	Maureen T. O'Brien, DVM, MS, DACVP	Charles River Laboratories, Inc. Frederick, MD, USA

[a] No formal report for this phase.
[b] Sponsor.
[c] Test Facility-designated Test Site.

2. SUMMARY

The objective of this study was to characterize the toxicity, biodistribution, and gene expression of AAV9/AP4M1, for the treatment of Spastic Paraplegia Type 50 (SPG50) caused by the AP4M1 gene mutation, when given by a single intrathecal injection to rats and to evaluate the potential reversibility and/or progression of any findings.

The study design was as follows:

Text Table 1
Experimental Design

Group No.	Test Material	Dose Level (vg)	Dose Volume (µL)	Dose Concentration (vg/µL)	No. of Animals					
					Main Study		Recovery Study			
					Necropsy Day 8		Necropsy Day 29		Necropsy Day 91	
					M	F	M	F	M	F
1	Reference Item[a]	0	60	0	5	5	5	5	5	5
2	AAV9/AP4M1	0.36×10^{12}	20	0.18×10^{11}	5	5	5	5	5	5
3	AAV9/AP4M1	1.1×10^{12}	20	0.55×10^{11}	5	5	5	5	5	5
4	AAV9/AP4M1	3.3×10^{12}	60	0.55×10^{11}	5	5	5	5	5	5

M = Males; F = Females.
[a] PBS containing 5% D-sorbitol and 0.001% Pluronic F-68.

The following parameters and endpoints were evaluated in this study: mortality, clinical observations, body weights, food consumption, neurobehavioral evaluations, clinical pathology parameters (hematology, coagulation, clinical chemistry, and urinalysis), bioanalysis (serum and tissue [biodistribution]), splenocyte analysis, gene expression analyses in tissues, organ weights, and macroscopic and microscopic examinations.

There was no AAV9/AP4M1-related mortality during the course of the study.

There were no AAV9/AP4M1-related clinical observations, or effects on food consumption, urinalysis parameters, macroscopic findings, or organ weights.

AAV9/AP4M1-related decreases in group mean body weight (as low as 17% decrease compared with controls) were noted in males at 3.3×10^{12} vg starting from Day 35, and persisting until the end of the study. This was also was reflected in the decreased overall mean body weight gain from Days 28 to 84 (24% lower than controls). Due to the magnitude of the decrease, this was considered adverse.

During Weeks 4 and/or 13, there were AAV9/AP4M1-related increases in excitability and/or activity in males and females at 3.3×10^{12} vg noted during the neurobehavioral assessment. Due to the lack of any correlating findings, these changes were considered nonadverse.

On Day 8, clinical pathology results revealed increases in alanine aminotransferase activity in females at $\geq 0.36 \times 10^{12}$ vg and males at $\geq 1.1 \times 10^{12}$ vg, and increases in fibrinogen in 2/5 males at 3.3×10^{12} vg. During the recovery period, there were increases in neutrophils and total white blood cells in both sexes at 3.3×10^{12} vg (Day 91) and increases in glucose only in males at 3.3×10^{12} vg (Days 29 and 91). The aforementioned increases lacked any microscopic correlates, and therefore, were considered nonadverse.

Nonadverse AAV9/AP4M1-related microscopic findings at Day 8 included minimal mononuclear cell infiltration in the lumbar dorsal root ganglia in males at ≥ 1.1 x 10^{12} vg/dose and females at ≥ 0.36 x 10^{12} vg/dose. Minimal axonal degeneration was noted in the dorsal lumbar nerve root in males at ≥ 1.1x10^{12} vg/dose, in the sciatic nerve in males at ≥ 1.1 x 10^{12} vg/dose and females at 3.3 x 10^{12} vg/dose, and in the tibial nerve in males at ≥ 0.36 x 10^{12} vg/dose and females at 3.3 x 10^{12} vg/dose.

At Day 29, AAV9/AP4M1-related findings were noted at the injection site and included minimal to mild leptomeningeal mononuclear cell infiltration in animals at ≥ 0.36 x 10^{12} vg/dose and minimal to moderate cauda equina axonal degeneration in males at ≥ 0.36 x 10^{12} vg/dose and females at ≥ 1.1 x 10^{12} vg/dose. In the lumbar dorsal root ganglion, findings included minimal to mild axonal degeneration, neuronal degeneration, and mononuclear cell infiltration at ≥ 1.1 x 10^{12} vg/dose; neuronal degeneration persisted through Day 91 and due to the nature and the absence of recovery of this finding, it was considered adverse. In the lumbar dorsal nerve roots and peripheral nerves (sciatic and tibial), findings included axonal degeneration in animals at ≥ 1.1 x 10^{12} vg/dose and in females at 0.36 x 10^{12} vg/dose (dorsal nerve root only). One Group 4 male was also noted with mononuclear cell infiltration in the lumbar dorsal nerve roots on Day 29. There was a low incidence of axonal degeneration and/or mononuclear cell infiltration in the cervical and thoracic dorsal root ganglia on Day 29 with complete recovery by Day 91. There was also minimal to mild mononuclear cell infiltration within the adipose tissue surrounding the vertebral body that had partial recovery by Day 91.

Microscopic findings on Day 91 occurred at the injection site and in the lumbar dorsal root ganglia, lumbar dorsal nerve roots, sciatic and tibial nerves, and adipose tissue adjacent to the thoracic spinal column. Generally, there was partial or complete recovery of most, but not all, microscopic findings on Day 91 compared to Day 29. Findings at the injection site included minimal leptomeningeal mononuclear cell infiltration in animals at ≥ 0.36 x10^{12} vg/dose with a partial recovery in males compared to Day 29. There was also minimal axonal degeneration in the cauda equina of males and females, with partial recovery based on a decreased severity. In the lumbar dorsal root ganglion, findings included mononuclear cell infiltration in males ≥ 1.1 x 10^{12} vg/dose and females at ≥ 0.36 x 10^{12} vg/dose, with partial recovery; minimal axonal degeneration in males ≥ 1.1 x 10^{12} vg/dose and females at ≥ 3.3 x 10^{12} vg/dose, with partial recovery; and minimal neuronal degeneration at ≥ 1.1 x 10^{12} vg/dose, with no recovery. In the lumbar dorsal nerve roots, there was minimal to mild axonal degeneration at ≥ 0.36 x 10^{12} vg/dose with equivocal recovery (severity was decreased but incidence was higher). There was no mononuclear cell infiltration in lumbar dorsal nerve roots, indicating complete recovery of this finding. There was a dose-dependent increased incidence of minimal peripheral nerve degeneration (sciatic and tibial nerves) that occurred at ≥ 0.36 x 10^{12} vg/dose with equivocal recovery (severity was decreased but incidence was higher). There were no AAV9/AP4M1-related findings in the thoracic or cervical dorsal root ganglia, indicating complete recovery of the axonal degeneration and mononuclear cell infiltration observed on Day 29.

AAV9/AP4M1 resulted in a dose dependent increase of AP4M1 vector DNA across the central nervous system and peripheral organs; the pattern of AP4M1 biodistribution in this study was consistent with that expected from AAV9. AP4M1 transgene expression was also widely detected at high levels in multiple CNS and peripheral tissues. AAV9/AP4M1 generated minimal T-cell immune response to either AAV9 or the human AP4M1 protein.

In conclusion, administration of AAV9/AP4M1 by a single intrathecal injection in rats at 0.36×10^{12}, 1.1×10^{12}, and 3.3×10^{12} vg was well tolerated. Adverse findings included neuronal degeneration noted microscopically in the lumbar dorsal root ganglion at $\geq 1.1 \times 10^{12}$ vg and decreases in body weight at 3.3×10^{12} vg. Other AAV9/AP4M1-related, nonadverse, findings included neurobehavioral effects of increased excitability and/or activity at 3.3×10^{12} vg, changes in clinical pathology parameters at $\geq 0.36 \times 10^{12}$ vg, and microscopic findings in the lumbar dorsal nerve roots, dorsal root ganglia, cauda equina and leptomeninges in the injection site, spinal cord, and peripheral nerves (sciatic/tibial nerves) of mononuclear cell infiltration and axonal degeneration. Due to the adversity of the neuronal degeneration, the no-observed-adverse-effect level (NOAEL) was considered to be 0.36×10^{12} vg.

Furthermore, it was concluded that AAV9 delivered intrathecally can achieve broad distribution across the nervous system and peripheral organs without generating a significant immune response, although the level of gene transfer in the brain is sub-saturating with a minority of cells receiving the transgene.

3. INTRODUCTION

The objective of this study was to characterize the toxicity, biodistribution, and gene expression of AAV9/AP4M1, for the treatment of Spastic Paraplegia Type 50 (SPG50) caused by the AP4M1 gene mutation, when given by a single intrathecal injection to rats and to evaluate the potential reversibility and/or progression of any findings.

Study Initiation Date:	09 Feb 2021
Initiation of Dosing:	16 Feb 2021
Completion of In-life:	23 May 2021
Experimental Starting Date:	09 Feb 2021
Experimental Completion Date:	Signature date of Pathology Report

4. MATERIALS AND METHODS

The study was conducted in accordance with the Study Plan and last Amended Study Plan presented in Appendix 1 with the following requirements and exceptions provided in the sections below.

4.1. Test Material Identification

Text Table 2
Test Material Identification

	Test Item
**Identification:**	AAV9/AP4M1
**Alternate Identification:**	rAAV9.AP4M1
**Batch No.:**	T-GEMINIS-033
**Expiration/Retest Date:**	Not available
**Physical Description:**	Colorless, clear to slightly opalescent, free of visible particles
**Concentration: (Based on ddPCR Results)**	5.43E13 vg/mL
**Storage Conditions (Temperature Set to Maintain):**	-80ºC (upon thawing of a vial, it may be stored at 4ºC and used on a subsequent dosing day)
**Provided by:**	Sponsor

Text Table 3
Reference Item Identification

	Reference Item
**Identification**	1X dPBS containing 5% D-sorbitol and 0.001% Pluronic F-68
**Batch No.:**	2021-02-09/01
**Expiration/Retest Date:**	Not available
**Storage Conditions (Temperature Set to Maintain):**	-80°C
**Provided by:**	Sponsor

4.2. Study Design

Text Table 4
Experimental Design

Group No.	Test Material	Dose Level (vg)	Dose Volume (µL)	Dose Concentration (vg/µL)	Animal Numbers					
					Main Study		Recovery Study			
					Necropsy Day 8		Necropsy Day 29		Necropsy Day 91	
					M	F	M	F	M	F
1	Reference Item[a]	0	60	0	1001, 1002, 1013, 1014, 1015	1501, 1502, 1505, 1506, 1507	1003, 1004, 1008, 1009, 1010	1508, 1509, 1513, 1514, 1515	1005, 1006, 1007, 1011, 1012	1503, 1504, 1510, 1511, 1512
2	AAV9/AP4M1	0.36×10^{12}	20	0.18×10^{11}	2004, 2009, 2010, 2014, 2015	2505, 2506, 2510, 2511, 2512	2001, 2002, 2011, 2012, 2013	2501, 2502, 2507, 2508, 2509	2103, 2005, 2006, 2007, 2008	2503, 2504, 2613, 2614, 2615
3	AAV9/AP4M1	1.1×10^{12}	20	0.55×10^{11}	3001, 3002, 3003, 3012, 3013	3507, 3508, 3511, 3512, 3513	3004, 3005, 3009, 3010, 3011	3504, 3505, 3506, 3514, 3615	3006, 3007, 3008, 3014, 3015	3501, 3502, 3503, 3509, 3510
4	AAV9/AP4M1	3.3×10^{12}	60	0.55×10^{11}	4001, 4002, 4006, 4007, 4008	4601, 4502, 4508, 4509, 4510	4003, 4004, 4005, 4012, 4013	4505, 4506, 4507, 4511, 4512	4009, 4010, 4011, 4014, 4015	4503, 4504, 4513, 4514, 4515

M = Males; F = Females.
[a] PBS containing 5% D-sorbitol and 0.001% Pluronic F-68.

On Day 1, Female No. 4501 stopped breathing following dosing and though reanimation was attempted, the animal died and was subsequently replaced with a spare animal which became Female No. 4601.

On Day 1, a slight reflux was noted for Male No. 2003 when removing the dosing needle. In addition, due to a resistance noted during dosing, Female No. 3515 only received half of the intended dose volume. As a result of the incomplete doses, both animals were replaced by spare animals which became Male No. 2103 and Female No. 3615, respectively.

On Day 1, Female Nos. 2513, 2514, and 2515 were anesthetized for dose administration however, due to insufficient volume of test item formulation, the animals were could not be dosed at that time. As a second anesthesia was not recommended, the animals were replaced by spare animals which became Female Nos. 2613, 2614, and 2615, respectively, and were dosed along with the next cohort.

Text Table 5
Additional Study Plan Deliverables

**Age at Initiation of Dosing**	7 weeks old
**Body Weight Range at Initiation of Dosing**	168 – 285 g males<br>143 – 206 g females
**Number of Acclimation days**	7, 8, 10, or 13 days

None of the following Study Events had an adverse impact on the study or the interpretation of the data unless otherwise indicated.

Text Table 6
Study Events

**Event**
• Starting during Week-1, all animals were provided additional enrichment in the form of wood block, diamond twist, and/or bio hut, which were rotated weekly during bin changes. However, on occasion there was no data to support that the enrichment was provided.<br>• The right sciatic nerve of Female No. 1504 (euthanized on Day 91) for bioanalysis was dropped on the floor during removal however nonetheless submitted for analysis. This event had no impact as the tissues for Day 91 were not analyzed.<br>• The left iliac lymph node from Female No. 1508 was lost and therefore could not be submitted for histopathological evaluation. Tissues were collected from other animals to allow for microscopic evaluation.

4.3. Deviations

All deviations that occurred during the study have been authorized/acknowledged by the Study Director, assessed for impact, and documented in the study records. All Study Plan deviations and those SOP deviations that could have impacted the quality or integrity of the study are listed below. Minor SOP deviations that did not impact the quality or integrity of the study have been included at the discretion of the Study Director.

None of the deviations were considered to have impacted the overall integrity of the study or the interpretation of the study results and conclusions.

**SOP**	
**Deviation**	**Impact**
At termination on Day 29, the clinical chemistry sample tube for Male No. 2012 was received with an EDTA tube cap. As a result, the calcium parameter could not be determined. Additionally, the clinical chemistry tube for this animal was left at room temperature for more than 5 hours prior to centrifugation, therefore, all clinical chemistry parameters were out of established stability.	The data for this animal was excluded from group mean calculations. Sufficient data was collected from other animals within the same group and sex to allow for interpretation of the data.
The blood smears for Male Nos. 1003 and 1004 were prepared after more than 4 hours following collection.	The evaluation of the blood smears from both animals were not required.

Analytical Procedure		
Deviation		Impact
On Day 91, the splenocytes from Female Nos. 1503 and 1504 were incubated in the ammonium chloride solution for 11 minutes instead of the required 10 minutes. Additionally, following incubation, these splenocytes were inadvertently centrifuged at a temperature set to maintain 21°C.		Although the wrong incubation time and centrifugation temperature were used, the cell viability on Day 91 for Female Nos.1503 and 1504 was 95.7% and 92.6%, respectively, which was considered to be appropriate and within the range of viabilities observed for other samples in this study that were not affected by this deviation. Moreover, the amount of cells recovered was as expected. Cell viability for Female Nos. 1503 and 1504 was considered appropriate and within the range of viabilities observed for other samples in this study.
**Study Plan**		
**Husbandry**		
**Study Plan Section No.**	**Deviation**	**Impact**
8.2	Once during the prestudy period, Male Nos. 4009, 4012, 4014, and male spares 61, 63, and 65, were not provided a hiding device.	There was no impact on the welfare of the animals as animals were provided with other forms of enrichment such as group-housing.
8.4	On Day 70, the food consumption for Male Nos. 1005 to 1007, 2005 to 2007, and 3006 to 3008 was performed using the wrong type of food.	This had no impact on the overall evaluation of the food consumption data.
8.5	Once during the prestudy period, Male Nos. 4006, 4007, and 4008 were found with no access to water.	This lack of water did not result in any ill effects on the animals.
**In-Life Procedures, Observations, and Measurements**		
**Study Plan Section No.**	**Deviation**	**Impact**
10	The food in the cage of Male Nos. 1008, 1009, and 1010 was dropped prior to weighing on Day 7, therefore the total food consumption for the interval of Day -1 to 7 could not be calculated.	Sufficient data was collected from other animals to allow for interpretation of the data.
**Laboratory Evaluations**		
**Study Plan Section No.**	**Deviation**	**Impact**
12.2	Serum bioanalysis was not performed in this study although required by the Study Plan.	This had no impact on the objectives of the study as biodistribution of the test item was determined by analyzing tissue samples. The analysis of the serum samples is considered to not have added any additional information on biodistribution; this was inadvertently included in the study plan as a required assessment. Furthermore, serum anti-AAV9 neutralizing antibodies are unequivocally expected to increase in response to AAV9/AP4M1 injection as an effect of this class of drug, however, does not pose a safety risk.

13.8 and 13.10	Tissue biodistribution, gene expression, and splenocyte analysis was only performed on samples collected on Day 29, instead of all occasions, as required by the Study Plan.	This had no impact on the objectives of the study as the biodistribution values from Day 29 matched the expected pattern for AAV9 by this route of administration (consistent with literature on AAV9). It is expected that the vector genome copies from AAV9/AP4M1 should remain constant across all time points. Therefore, it was deemed not necessary to analyze samples from other time points.
**Postmortem and Pathology**		
**Study Plan Section No.**	**Deviation**	**Impact**
13.6	The brain weight for Female No. 2613 was inadvertently not recorded.	Sufficient data was collected from other animals within the same group and sex to allow for appropriate evaluation.
13.7	The vagina of Female No. 1504 was lost during necropsy, prior to examination.	Sufficient tissues were collected from other animals to allow for evaluation.
13.9	The samples collected for biodistribution and gene expression of all required tissues collected from Animal Nos. 1001, 1002, 1501, 1502, 1505, 1506, 1507, 2004, 2505, 2506, 3001, 3002, 3003, 4001, 4002, 4006, 4007, 4008, and 4502 weighed between 0.0040 to 0.0050 g instead of the required 0.0400 to 0.0500 g.	No impact as the amount of sample provided still allowed DNA and RNA extraction and sufficient nucleic acid was present in most samples to accurately quantify the AP4M1 sequence.
**Other**		
**Study Plan Section No.**	**Deviation**	**Impact**
13.8	For several animals across all occasions, less than 2 x 10^7 live splenocytes were obtained, therefore, 900 µL of cell suspension could not be frozen as required the by Study Plan. Moreover, for some animals, a second or third aliquot was frozen with less than 2 x 10^7 live splenocytes.	For Male Nos. 4008 and 2010, only the 2nd and 3rd aliquots were impacted. For the other animals, due to the limited number of live cells isolated following spleen processing, a minimum of 2 x 10^7 cells in 900µL of RPMI - 10% DMSO could not be frozen. The volumes of RPMI + 10% DMSO was therefore reduced in order to keep the same cell concentration. A minimum of 500 µL of media was used when the number of live cells was below 1 x 10^7.<br>For Female No. 1508 (control group), no live cells could be counted, which was confirmed by a trypan blue staining, followed by a count using a microscope, thus, no vial was frozen for this sample. No impact on the overall interpretation of these results as samples behaved appropriately to control conditions in downstream assays conducted by the Sponsor.
**Planned Deviations**		
13.9	Prior to the first necropsy on Day 8, a planned deviation was issued to provide instructions on the storage of remaining tissues following collection of all tissues designated for biodistribution analysis. The Study Plan was later amended to reflect this change for subsequent cohorts and necropsy occasions.	

5. COMPUTERIZED SYSTEMS

Critical computerized systems used in the study are listed below or presented in the appropriate Phase Report. All computerized systems used in the conduct of this study have been validated; when a particular system has not satisfied all requirements, appropriate administrative and procedural controls were implemented to assure the quality and integrity of data.

Text Table 7
Critical Computerized Systems

System Name	Version No.	Description of Data Collected and/or Analyzed
Provantis®	10	In-life: clinical pathology; postmortem; statistical analysis; Test Material receipt, accountability and/or formulation activities. Statistical Analysis of in-life (body weights, food consumption, neurobehavioral evaluation); clinical pathology (clinical chemistry, coagulation, hematology, urinalysis); and postmortem (organ weights).
Deviation Information Library	2.1	Deviations
Share Document Management System	1.0	Reporting
M-Files®	21.6	Reporting and collection of 21 CFR Part 11 compliant signature
DocuSign™	19	Collection of 21 CFR Part 11 compliant signature
eInfotree	7.6.0	Excel Module for collection of 21 CFR Part 11 compliance requirements, security, audit trail and electronic signatures
Mesa Laboratories AmegaView CMS	v3.0 Build 1209.08	Continuous Monitoring System. Monitoring of standalone fridges, freezers, incubators, and selected laboratories to measure temperature, relative humidity, and CO_2, as appropriate
Johnson Controls Metasys	MVE 7.0	Building Automation System. Control of HVAC and other building systems, as well as temperature/humidity control and trending in selected laboratories and animal rooms

6. RETENTION OF RECORDS, SAMPLES, AND SPECIMENS

All study-specific raw data, documentation, Study Plan, samples, specimens, and final reports from this study were archived at the Test Facility by no later than the date of Final Report issue. At least 1 year after issue of the Draft Report, the Sponsor will be contacted.

Electronic data generated by the Test Facility were archived as noted above, except that the data collected using Provantis®, reporting files stored on SDMS, M-Files®, and deviations were archived at the Charles River Laboratories facility location in Wilmington, MA.

All records, retained samples and specimens, and reports generated from phases or segments performed by Test Facility-designated subcontractors were returned to the Test Facility for archiving.

All records, retained samples and specimens, and reports generated from phases or segments performed by the Sponsor were archived by the Sponsor. Archival location is detailed in the applicable PI reports.

Text Table 8
Disposition of Residual/Retained Samples

Sample Type	Schedule
Bioanalytical<br>(CSF, serum, splenocytes, and tissues [biodistribution/gene expression and frozen])	Samples archived by the Sponsor.

7. RESULTS

7.1. Mortality

(Appendix 3)

There was 1 unscheduled death throughout the course of the study. Control Animal No. 1013, administered the reference item, was euthanized early on Day 7. Clinical signs prior to its euthanasia included decreased activity, labored breathing, limited usage of the hindlimbs, low carriage, lying on its side, prostration, weakness, thinness, and prominent backbone. Clinical pathology alterations were non-conclusive and included mild to moderate increases in albumin, sodium, chloride, potassium and fibrinogen. These changes were nonspecific and may have been secondary to hemoconcentration, real or secondary to sample evaporation (increases in albumin, sodium, chloride, potassium and fibrinogen) or inflammation (increase in fibrinogen). Gross findings at necropsy included abnormal dark and/or firm content in the jejunum, colon, and cecum, a small spleen, and small dark foci on the glandular stomach mucosa. There were no microscopic correlates for the gross observations; microscopic findings were nonspecific, and the cause of clinical moribundity was undetermined.

7.2. Clinical Observations

(Table 1, Appendix 4, and Appendix 5)

There were no AAV9/AP4M1-related clinical signs noted throughout the study.

Clinical observations including, but not limited to, skin lesion/redness/dryness/scab, thin cover, or staining of the fur were attributed to normal variations in this species and were unrelated to AAV9/AP4M1 due to their low incidence, lack of correlating findings, or similar incidence in the control group.

7.3. Body Weights and Body Weight Gains

(Figure 1, Figure 2, Table 2, Table 3, Appendix 6, and Appendix 7)

AAV9/AP4M1-related changes in group mean body weights and body weight gains were noted in males at 3.3 x 10^{12} vg. Decreases (down to 17% decreases from control mean) in group mean body weight were observed starting from Day 35, persisted until the end of the study, and was reflected in the decreased overall mean body weight gain from Days 28 to 84 (24% lower than controls).

Any other variations noted, including those that reached statistical significance, were within the range of the concurrent control group data, lacked a dose relationship, and/or were due to biological variation.

7.4. Food Consumption

(Table 4 and Appendix 8)

There were no AAV9/AP4M1-related changes in food consumption noted throughout the study.

Any variations noted were within the range of the concurrent control group data, lacked a dose relationship, and/or were considered to be due to biological variation.

7.5. Neurobehavioral Evaluation

(Table 5 to Table 10, Appendix 9 to Appendix 14, and Appendix 15)

During Weeks 4 and 13, there were AAV9/AP4M1-related effects on neurobehavioral parameters, specifically excitability and/or activity in males and females at 3.3 x 10^{12} vg.

During Weeks 4 and 13, males and females displayed increased arousal/alertness and rearing, within the open field box. In addition, males exhibited an exaggerated startle response during the Week 4 assessment.

7.6. Hematology

(Appendix 16)

Administration of AAV9/AP4M1 was associated with minimal to mild increases in neutrophil counts (1.45X to 1.87X control group mean) and total white blood cell counts (1.29X to 1.31X) in both sexes at 3.3 x 10^{12} vg at the end of the recovery on Day 91.

All differences in hematology parameters, regardless of statistical significance, were not considered AAV9/AP4M1-related based on their small magnitude, inconsistent direction, absence of a dose response, general overlap of individual values with the range of control and baseline values, and/or were of a magnitude of change commonly observed in rats under similar study conditions.

7.7. Coagulation

(Appendix 16)

Administration of AAV9/AP4M1 was associated with minimal changes in coagulation parameters limited to increases in fibrinogen (1.18x control group mean) in 2/5 males (Nos. 4006 and 4007) at 3.3 x 10^{12} vg at the end of the main study on Day 8.

Remaining differences in coagulation parameters were not considered AAV9/AP4M1-related based on their small magnitude, inconsistent direction, absence of a dose response, general overlap of individual values with the range of control and baseline values, and/or were of a magnitude of change commonly observed in rats under similar study conditions.

7.8. Clinical Chemistry

(Appendix 16)

Administration of AAV9/AP4M1 was associated with transient minimal and non-dose-related increases in alanine aminotransferase activity in females at ≥ 0.36 x 10^{12} vg and males at ≥ 1.1 x 10^{12} vg (1.23X to 1.56X control group mean) at the end of the main study on Day 8.

There were minimal increases (1.22X to 1.43X control group mean) in glucose in males at 3.3×10^{12} vg on Days 29 and 91.

Remaining differences in clinical chemistry parameters, regardless of statistical significance, were not considered AAV9/AP4M1-related based on their small magnitude, inconsistent direction, absence of a dose response, general overlap of individual values with the range of control and baseline values, and/or were of a magnitude of change commonly observed in rats under similar study conditions.

7.9. Urinalysis

(Appendix 16)

No AAV9/AP4M1-related urinalysis changes were noted at any dose level.

All differences in urinalysis parameters, regardless of statistical significance, were not considered AAV9/AP4M1-related based on their small magnitude, inconsistent direction, absence of a dose response, general overlap of individual values with the range of control and baseline values, and/or were of a magnitude of change commonly observed in rats under similar study conditions.

7.10. Biodistribution, Gene Expression, and Splenocyte Analysis

(Appendix 17)

AAV9/AP4M1 resulted in a dose dependent increase of AP4M1 vector DNA across the central nervous system and peripheral organs. The AP4M1 vector DNA was widely detected at high levels in multiple brain regions. In the peripheral organs, similar high amounts of AP4M1 DNA persisted in the heart, liver, and spleen and to a lesser extent in other organs. The pattern of AP4M1 biodistribution in this study was consistent with that expected from AAV9. AP4M1 vector biodistribution results are shown in Text Figure 1.

Consistent with the AP4M1 DNA biodistribution data, AP4M1 transgene expression was also widely detected at high levels in multiple central nervous system (CNS) and peripheral tissues (Text Figure 2). Collectively, AAV9/AP4M1 resulted in broad AP4M1 biodistribution and expression in rats.

Text Figure 1
AP4M1 Tissue Biodistribution on Day 29

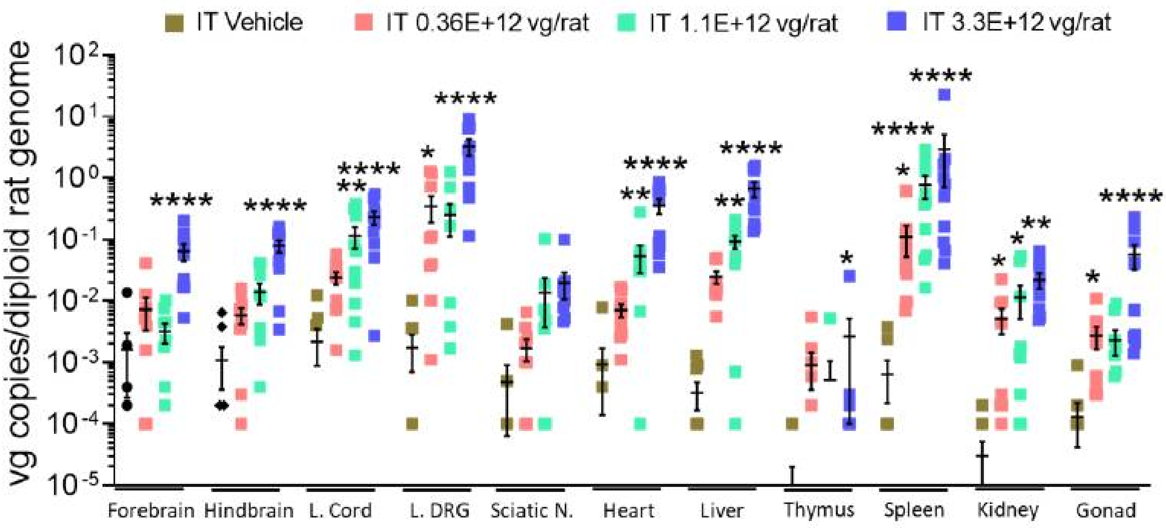

Text Figure 2
AP4M1 Tissue Expression on Day 29

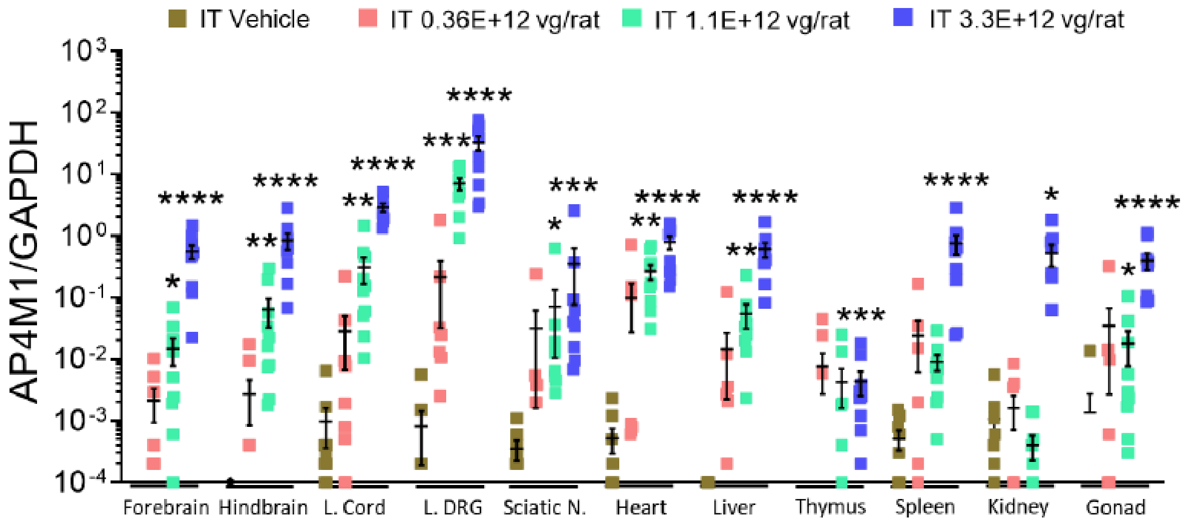

The AAV9/AP4M1 vector generated minimal T-cell immune response to either AAV9 or the human AP4M1 protein, as shown in Text Figure 3.

Text Figure 3
Immune Response of Splenocytes on Day 29

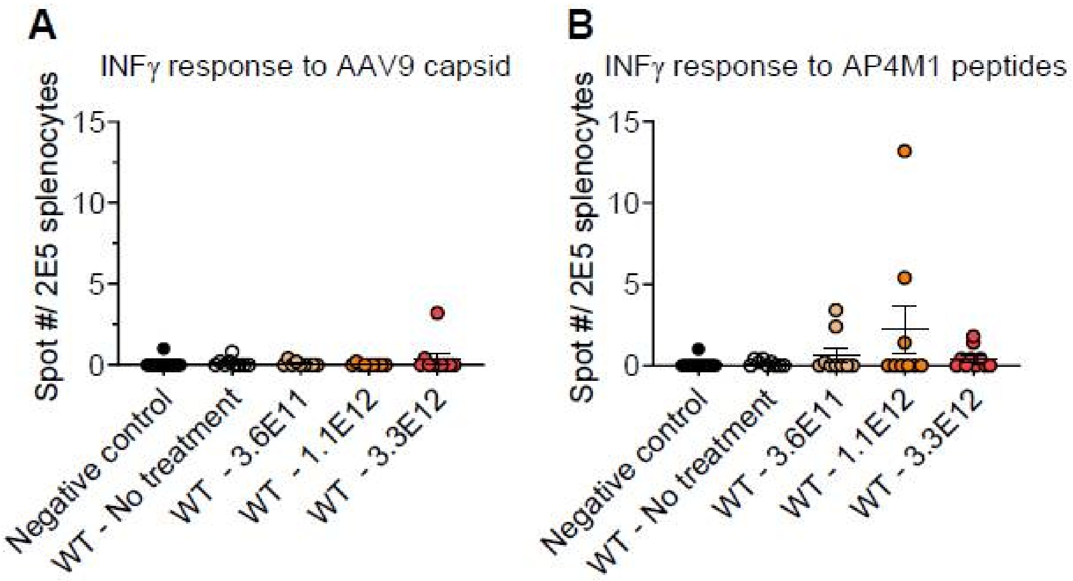

7.11. Macroscopic Pathology

(Appendix 18)

No AAV9/AP4M1-related gross findings were noted. The gross findings observed were considered incidental, of the nature commonly observed in this strain and age of rats, and/or were of similar incidence in control and AAV9/AP4M1-treated animals and, therefore, were considered unrelated to administration of AAV9/AP4M1.

7.12. Organ Weights

(Appendix 18)

No AAV9/AP4M1-related organ weight changes were noted.

A significant decrease in absolute and relative uterine weights was observed on Day 29 in females receiving $\geq 0.36 \times 10^{12}$ vg but was considered unrelated to the administration of AAV9/AP4M1 due to the absence of microscopic correlates and the lack of corresponding weight changes on Day 8 and Day 91. There were other isolated organ weight values that were statistically different from their respective controls. There were, however, no patterns, trends, or correlating data to suggest these values were toxicologically relevant. Thus, the organ weight differences observed were considered incidental and/or related to difference of sexual maturity and unrelated to administration of AAV9/AP4M1.

7.13. Microscopic Evaluations

(Appendix 18)

7.13.1. Terminal Euthanasia Animals (Day 8)

AAV9/AP4M1-related microscopic findings are summarized in the following table.

Text Table 9
Summary of Microscopic Findings – Scheduled Euthanasia (Day 8)

	Males				Females			
**Group**	**1**	**2**	**3**	**4**	**1**	**2**	**3**	**4**
**Dose Level (vg)**	**0**	**0.36 x 10^{12}**	**1.1 x 10^{12}**	**3.3 x 10^{12}**	**0**	**0.36 x 10^{12}**	**1.1 x 10^{12}**	**3.3 x 10^{12}**
**No. Animals per Group**	**4**	**5**	**5**	**5**	**5**	**5**	**5**	**5**
Lumbar Region:								
**Ganglion, Dorsal Root, Lumbar (No. Examined)**	4	5	5	5	5	5	5	5
Infiltration, mononuclear cell	(0)	(0)	(2)	(5)	(0)	(1)	(1)	(3)
Minimal	0	0	2	5	0	1	1	3
**Nerve Root, Dorsal, Lumbar (No. Examined)**	4	5	5	5	5	5	5	5
Degeneration, axonal	(1)	(0)	(2)	(1)	(0)	(0)	(0)	(0)
Minimal	1	0	2	1	0	0	0	0
**Nerve, Sciatic (No. Examined)** [a]	4	5	5	5	5	5	5	5
Degeneration, axonal	(0)	(0)	(1)	(1)	(0)	(0)	(0)	(1)
Minimal	0	0	1	1	0	0	0	1
**Nerve, Tibia (No. Examined)**	4	5	5	5	5	5	5	5
Degeneration, axonal	(0)	(1)	(2)	(2)	(0)	(0)	(0)	(1)
Minimal	0	1	2	2	0	0	0	1

[a] Numbers in parentheses represent the number of animals with the finding.

AAV9/AP4M1-related findings on Day 8 occurred in the lumbar dorsal root ganglia, lumbar dorsal nerve roots, and sciatic and tibial nerves. Findings in the lumbar dorsal root ganglion included minimal mononuclear cell infiltration in males and females at ≥ 1.1/0.36 x10^{12} vg. In the dorsal lumbar nerve root, minimal axonal degeneration was considered AAV9/AP4M1-related in males at ≥ 1.1x10^{12} vg due to the increased magnitude/severity of the finding at subsequent timepoints, although this finding was also observed in one control (Group 1) male on Day 8. Somewhat dose-dependent AAV9/AP4M1-related increased incidence of minimal peripheral nerve degeneration occurred in males and females ≥ 1.1/3.3x10^{12} vg/dose in the sciatic nerve and ≥ 0.36/3.3x10^{12} vg/dose in the tibial nerve.

Generally, microscopic findings at the injection site on Day 8 in all groups included minimal axonal degeneration and/or minimal mononuclear cell infiltration within the leptomeninges. As these findings occurred with similar incidence and/or severity in both control and AAV9/AP4M1-treated animals on Day 8, they were considered unrelated to administration of AAV9/AP4M1. There were no AAV9/AP4M1-related findings on Day 8 at the injection site, in the cervical and thoracic nerve roots/dorsal root ganglia, or in the ventral nerve roots at any dose level (cervical, thoracic, lumbar).

Other microscopic findings observed were considered incidental, of the nature commonly observed in this strain and age of rats, and/or were of similar incidence and severity in control and treated animals and, therefore, were considered unrelated to administration of AAV9/AP4M1.

7.13.2. Recovery Euthanasia Animals (Day 29)

Microscopic findings noted at the terminal euthanasia were observed at the end of the recovery period (Day 29) and are summarized in the following table.

Text Table 10
Summary of Microscopic Findings – Scheduled Euthanasia (Day 29)

	Males				Females			
**Group**	**1**	**2**	**3**	**4**	**1**	**2**	**3**	**4**
**Dose Level (vg)**	**0**	**0.36 x 10^{12}**	**1.1 x 10^{12}**	**3.3 x 10^{12}**	**0**	**0.36 x 10^{12}**	**1.1 x 10^{12}**	**3.3 x 10^{12}**
**No. Animals per Group**	**5**	**5**	**5**	**5**	**5**	**5**	**5**	**5**
**Injection Site (No. Examined)[a]**	5	5	5	5	5	5	5	5
Degeneration, axonal, cauda equina	(0)	(2)	(3)	(4)	(0)	(0)	(3)	(5)
Minimal	0	2	3	2	0	0	2	0
Mild	0	0	0	2	0	0	1	3
Moderate	0	0	0	0	0	0	0	2
Infiltration, mononuclear cell, leptomeninges	(0)	(2)	(1)	(4)	(1)	(4)	(2)	(4)
Minimal	0	2	1	3	1	4	2	4
Mild	0	0	0	1	0	0	0	0
Lumbar region:								
**Ganglion, Dorsal Root, Lumbar (No. Examined)**	5	5	5	5	5	5	5	5
Degeneration, axonal	(0)	(0)	(2)	(4)	(0)	(0)	(2)	(5)
Minimal	0	0	2	1	0	0	2	4
Mild	0	0	0	3	0	0	0	1
Degeneration, neuronal	(0)	(0)	(3)	(3)	(0)	(0)	(1)	(3)
Minimal	0	0	3	2	0	0	1	3
Mild	0	0	0	1	0	0	0	0
Infiltration, mononuclear cell	(0)	(0)	(4)	(4)	(0)	(0)	(3)	(5)
Minimal	0	0	4	3	0	0	2	3
Mild	0	0	0	1	0	0	1	2
**Nerve Root, Dorsal, Lumbar (No. Examined)**	5	5	5	5	5	5	5	5
Degeneration, axonal	(0)	(1)	(4)	(4)	(0)	(0)	(4)	(5)
Minimal	0	1	3	1	0	0	3	1
Mild	0	0	1	3	0	0	1	3
Moderate	0	0	0	0	0	0	0	1

	Males				Females			
**Group**	**1**	**2**	**3**	**4**	**1**	**2**	**3**	**4**
**Dose Level (vg)**	**0**	**0.36 x 10^{12}**	**1.1 x 10^{12}**	**3.3 x 10^{12}**	**0**	**0.36 x 10^{12}**	**1.1 x 10^{12}**	**3.3 x 10^{12}**
**No. Animals per Group**	**5**	**5**	**5**	**5**	**5**	**5**	**5**	**5**
Thoracic region:								
**Ganglion, Dorsal Root, Thoracic (No. Examined)**	5	5	5	5	5	5	5	5
Degeneration, axonal	(0)	(0)	(0)	(0)	(0)	(0)	(0)	(1)
Minimal	0	0	0	0	0	0	0	1
Infiltration, mononuclear cell	(0)	(0)	(0)	(0)	(0)	(0)	(0)	(1)
Minimal	0	0	0	0	0	0	0	1
**Spinal Cord, Thoracic (No. Examined)**	5	5	5	5	5	5	5	5
Infiltration, mononuclear cell, adipose tissue	(0)	(0)	(0)	(1)	(0)	(1)	(0)	(2)
Minimal	0	0	0	0	0	1	0	2
Mild	0	0	0	1	0	0	0	0
Cervical region:								
**Ganglion, Dorsal Root, Cervical (No. Examined)**	5	5	5	5	5	5	5	5
Degeneration, axonal	(0)	(0)	(0)	(0)	(0)	(0)	(0)	(1)
Minimal	0	0	0	0	0	0	0	1
Infiltration, mononuclear cell	(0)	(0)	(0)	(0)	(0)	(0)	(0)	(1)
Minimal	0	0	0	0	0	0	0	1
**Nerve, Sciatic (No. Examined)**	5	5	5	5	5	5	5	5
Degeneration, axonal	(0)	(0)	(5)	(5)	(0)	(0)	(5)	(5)
Minimal	0	0	4	4	0	0	5	2
Mild	0	0	1	1	0	0	0	3
**Nerve, Tibia (No. Examined)**	5	5	5	5	5	5	5	5
Degeneration, axonal	(0)	(0)	(4)	(4)	(0)	(0)	(4)	(5)
Minimal	0	0	3	3	0	0	4	3
Mild	0	0	1	1	0	0	0	2

[a] Numbers in parentheses represent the number of animals with the finding.

AAV9/AP4M1-related findings on Day 29 occurred in the injection site, lumbar dorsal root ganglia, lumbar dorsal nerve roots, thoracic dorsal root ganglion, cervical dorsal root ganglia, sciatic and tibial nerves, and adipose tissue adjacent to the thoracic spinal column. Findings were generally most severe (highest magnitude/severity grades) on Day 29 compared to Day 8 and Day 91. AAV9/AP4M1-related findings at the injection site included AAV9/AP4M1-related (i.e. changes related to treatment without dose-dependency) increased incidence and/or severity of minimal to mild leptomeningeal mononuclear cell infiltration in males and females at $\geq$ 0.36 x10^{12} vg and dose-dependent increased incidence and/or severity of minimal to moderate axonal degeneration in the cauda equina of males and females at $\geq$ 0.36/1.1 x10^{12} vg.

AAV9/AP4M1-related findings in the lumbar dorsal root ganglia and lumbar dorsal nerve roots included generally dose-dependent increased incidence and/or severity of minimal-to-mild (ganglion) or minimal-to-moderate (nerve roots) axonal degeneration in males and females at $\geq$ 0.36/1.1x10^{12} vg/dose. There was minimal-to-mild lumbar dorsal root ganglion neuronal degeneration in males and females at $\geq$ 1.1 x10^{12} vg/dose. Additionally, there was

AAV9/AP4M1-related, treatment-dependent minimal-to-mild mononuclear cell infiltration in lumbar dorsal root ganglia in males and females at ≥ 1.1 x10^{12} vg/dose and minimal mononuclear cell infiltration in the lumbar dorsal nerve root of one male administered 3.3 x 10^{12} vg/dose.

AAV9/AP4M1-related findings in the cervical and thoracic dorsal root ganglion included minimal axonal degeneration and/or mononuclear cell infiltration in one female administered 3.3x10^{12} vg (No. 4511), as well as mononuclear cell infiltration in the thoracic dorsal root ganglion of another female administered 3.3x10^{12} vg (No. 4507).

Generally dose-dependent AAV9/AP4M1-related increased incidence and/or severity of minimal to mild peripheral nerve degeneration (sciatic and tibial nerves) occurred in males and females at ≥ 1.1x10^{12} vg. Findings were increased in incidence and magnitude (severity) compared to Day 8.

There was also minimal to mild mononuclear cell infiltration within the adipose tissue surrounding the vertebral body that was considered AAV9/AP4M1-related due to low incidence in males and females receiving 3.3x10^{12} vg only.

There were no AAV9/AP4M1-related findings on Day 29 in the cervical or thoracic dorsal nerve roots, or in the cervical spinal cord or ventral nerve roots at any level (cervical, thoracic, lumbar).

Other microscopic findings observed were considered incidental, of the nature commonly observed in this strain and age of rats, and/or were of similar incidence and severity in control and treated animals and, therefore, were considered unrelated to administration of AAV9/AP4M1.

7.13.3. Recovery Euthanasia Animals (Day 91)

Microscopic findings noted at the terminal euthanasia were observed at the end of the recovery period (Day 91) and are summarized in the following table.

Text Table 11
Summary of Microscopic Findings – Scheduled Euthanasia (Day 91)

	Males				Females			
**Group**	**1**	**2**	**3**	**4**	**1**	**2**	**3**	**4**
**Dose Level (vg)**	**0**	**0.36 x 10^{12}**	**1.1 x 10^{12}**	**3.3 x 10^{12}**	**0**	**0.36 x 10^{12}**	**1.1 x 10^{12}**	**3.3 x 10^{12}**
**No. Animals per Group**	**5**	**5**	**5**	**5**	**5**	**5**	**5**	**5**
**Injection Site (No. Examined)[a]**	5	5	5	5	5	5	5	5
Degeneration, axonal, cauda equina	(0)	(0)	(0)	(4)	(0)	(0)	(3)	(4)
Minimal	0	0	0	4	0	0	3	4
Infiltration, mononuclear cell, leptomeninges	(1)	(1)	(2)	(1)	(1)	(4)	(4)	(4)
Minimal	1	1	2	1	1	4	4	4

	Males				Females			
**Group**	**1**	**2**	**3**	**4**	**1**	**2**	**3**	**4**
**Dose Level (vg)**	**0**	**0.36 x 10^{12}**	**1.1 x 10^{12}**	**3.3 x 10^{12}**	**0**	**0.36 x 10^{12}**	**1.1 x 10^{12}**	**3.3 x 10^{12}**
**No. Animals per Group**	**5**	**5**	**5**	**5**	**5**	**5**	**5**	**5**
Lumbar region:								
**Ganglion, Dorsal Root, Lumbar (No. Examined)**	5	5	5	5	5	5	5	5
Degeneration, axonal	(0)	(0)	(0)	(1)	(0)	(0)	(2)	(1)
Minimal	0	0	0	1	0	0	2	1
Degeneration, neuronal	(0)	(0)	(2)	(3)	(0)	(0)	(1)	(2)
Minimal	0	0	2	3	0	0	1	2
Infiltration, mononuclear cell	(0)	(0)	(2)	(3)	(0)	(1)	(2)	(2)
Minimal	0	0	2	2	0	1	2	2
Mild	0	0	0	1	0	0	0	0
**Nerve Root, Dorsal, Lumbar (No. Examined)**	5	5	5	5	5	5	5	5
Degeneration, axonal	(0)	(1)	(4)	(4)	(0)	(2)	(5)	(5)
Minimal	0	1	4	2	0	2	5	1
Mild	0	0	0	2	0	0	0	4
**Spinal Cord, Lumbar (No. Examined)**	5	5	5	5	5	5	5	5
Infiltration, mononuclear cell, leptomeninges	(0)	(0)	(0)	(0)	(0)	(0)	(2)	(0)
Minimal	0	0	0	0	0	0	2	0
Thoracic region:								
**Spinal Cord, Thoracic (No. Examined)**	5	5	5	5	5	5	5	5
Infiltration, mononuclear cell, adipose tissue	(0)	(0)	(0)	(0)	(0)	(0)	(1)	(2)
Minimal	0	0	0	0	0	0	1	2
**Nerve, Sciatic (No. Examined)**	5	4	5	5	5	5	5	5
Degeneration, axonal	(0)	(2)	(3)	(4)	(0)	(1)	(5)	(5)
Minimal	0	2	3	4	0	1	5	5
**Nerve, Tibia (No. Examined)**	5	5	5	5	5	5	5	5
Degeneration, axonal	(0)	(2)	(2)	(4)	(0)	(2)	(4)	(5)
Minimal	0	2	2	4	0	2	4	5

[a] Numbers in parentheses represent the number of animals with the finding.

AAV9/AP4M1-related findings on Day 91 occurred in the injection site, lumbar dorsal root ganglia, lumbar dorsal nerve roots, sciatic and tibial nerves, and adipose tissue adjacent to the thoracic spinal column. Most, but not all, microscopic findings generally had decreased incidence and/or severity compared to Day 29. Treatment-related (i.e. changes related to treatment without dose-dependency) findings at the injection site included treatment-related increased incidence of minimal leptomeningeal mononuclear cell infiltration in males and females at ≥ 0.36 x10^{12} vg that had higher incidence in females versus males. There was also generally dose-dependent increased incidence of minimal axonal degeneration in the cauda equina of males and females at ≥ 3.3/1.1 x10^{12} vg. Although incidence of leptomeningeal mononuclear cell infiltration and axonal degeneration in the cauda equina is similar to Day 29, the magnitude (severity) of the finding has decreased, indicating partial recovery.

AAV9/AP4M1-related findings in the lumbar dorsal root ganglion on Day 91 included generally dose-dependent increased incidence and/or severity of minimal-to-mild mononuclear cell infiltration in males and females at $\geq 1.1/0.36 \times 10^{12}$ vg/dose, with slightly decreased incidence and severity compared to Day 29 indicating partial recovery of this finding. A generally dose-dependent incidence of minimal axonal degeneration was noted in males and females at $3.3/\geq 1.1 \times 10^{12}$ vg/dose, with slightly decreased incidence and severity compared to Day 29 indicating partial recovery of this finding. There was also minimal lumbar dorsal root ganglion neuronal degeneration in males and females at $\geq 1.1 \times 10^{12}$ vg/dose, with generally similar incidence to Day 29 indicating no recovery of this finding. Due to the nature of this finding, the neuronal degeneration was considered adverse.

In the lumbar dorsal nerve roots, there was minimal-to-mild axonal degeneration in males and females at $\geq 0.36 \times 10^{12}$ vg; while severity has decreased compared to Day 29, incidence has increased to include the lowest dose group (Group 2, where findings were limited to Group 3 and Group 4 on Day 29) indicating equivocal recovery. There was no mononuclear cell infiltration in lumbar dorsal nerve roots on Day 91, indicating complete recovery of this finding.

On Day 91, there were no AAV9/AP4M1-related findings in the thoracic or cervical dorsal root ganglia, indicating complete recovery of the axonal degeneration and mononuclear cell infiltration observed in a single Group 4 male on Day 29.

There was generally dose-dependent AAV9/AP4M1-related increased incidence of minimal peripheral nerve degeneration (sciatic and tibial nerves) that occurred in males and females at $\geq 0.36 \times 10^{12}$ vg. Although magnitude (severity) has decreased from minimal to mild compared to Day 29, incidence has increased to include the lowest dose group (Group 2) indicating equivocal recovery.

There was also minimal mononuclear cell infiltration within the adipose tissue surrounding the vertebral body that was considered AAV9/AP4M1-related that occurred on Day 91 with low incidence in females only administered $\geq 1.1 \times 10^{12}$ vg; the slightly lower incidence and/or magnitude (severity) indicates partial recovery of this finding.

There were no AAV9/AP4M1-related findings on Day 91 in the cervical or thoracic dorsal nerve roots, or in the cervical spinal cord or ventral nerve roots at any level (cervical, thoracic, lumbar). Other microscopic findings observed were considered incidental, of the nature commonly observed in this strain and age of rats, and/or were of similar incidence and severity in control and treated animals and, therefore, were considered unrelated to administration of AAV9/AP4M1.

8. CONCLUSION

In conclusion, administration of AAV9/AP4M1 by a single intrathecal injection in rats at 0.36×10^{12}, 1.1×10^{12}, and 3.3×10^{12} vg was well tolerated. Adverse findings included neuronal degeneration noted microscopically in the lumbar dorsal root ganglion at $\geq 1.1 \times 10^{12}$ vg and decreases in body weight at 3.3×10^{12} vg. Other AAV9/AP4M1-related, nonadverse, findings included neurobehavioral effects of increased excitability and/or activity at 3.3×10^{12} vg, changes in clinical pathology parameters at $\geq 0.36 \times 10^{12}$ vg, and microscopic findings in the lumbar dorsal nerve roots, dorsal root ganglia, cauda equina and leptomeninges in the injection site, spinal cord, and peripheral nerves (sciatic/tibial nerves) of mononuclear cell infiltration and axonal degeneration. Due to adversity of the neuronal degeneration, the no-observed-adverse-effect level (NOAEL) was considered to be 0.36×10^{12} vg.

Furthermore, it was concluded that AAV9 delivered intrathecally can achieve broad distribution across the nervous system and peripheral organs without generating a significant immune response, although the level of gene transfer in the brain is sub-saturating with a minority of cells receiving the transgene.

9. REFERENCES

Bee JS, Zhang Y, Phillippi MK, et al. Impact of Time Out of Intended Storage and Freeze-thaw Rates on the Stability of Adeno-associated Virus 8 and 9, Journal of Pharmaceutical Sciences, 111(2) (2022).

Gruntman AM, Su L, Su Q, et al. Stability and Compatibility of Recombinant Adeno-Associated Virus Under Conditions Commonly Encountered in Human Gene Therapy Trials, Human Gene Therapy Methods, 26(2): 71–76 (2015).

Figure 1

Summary of Body Weights

5550008

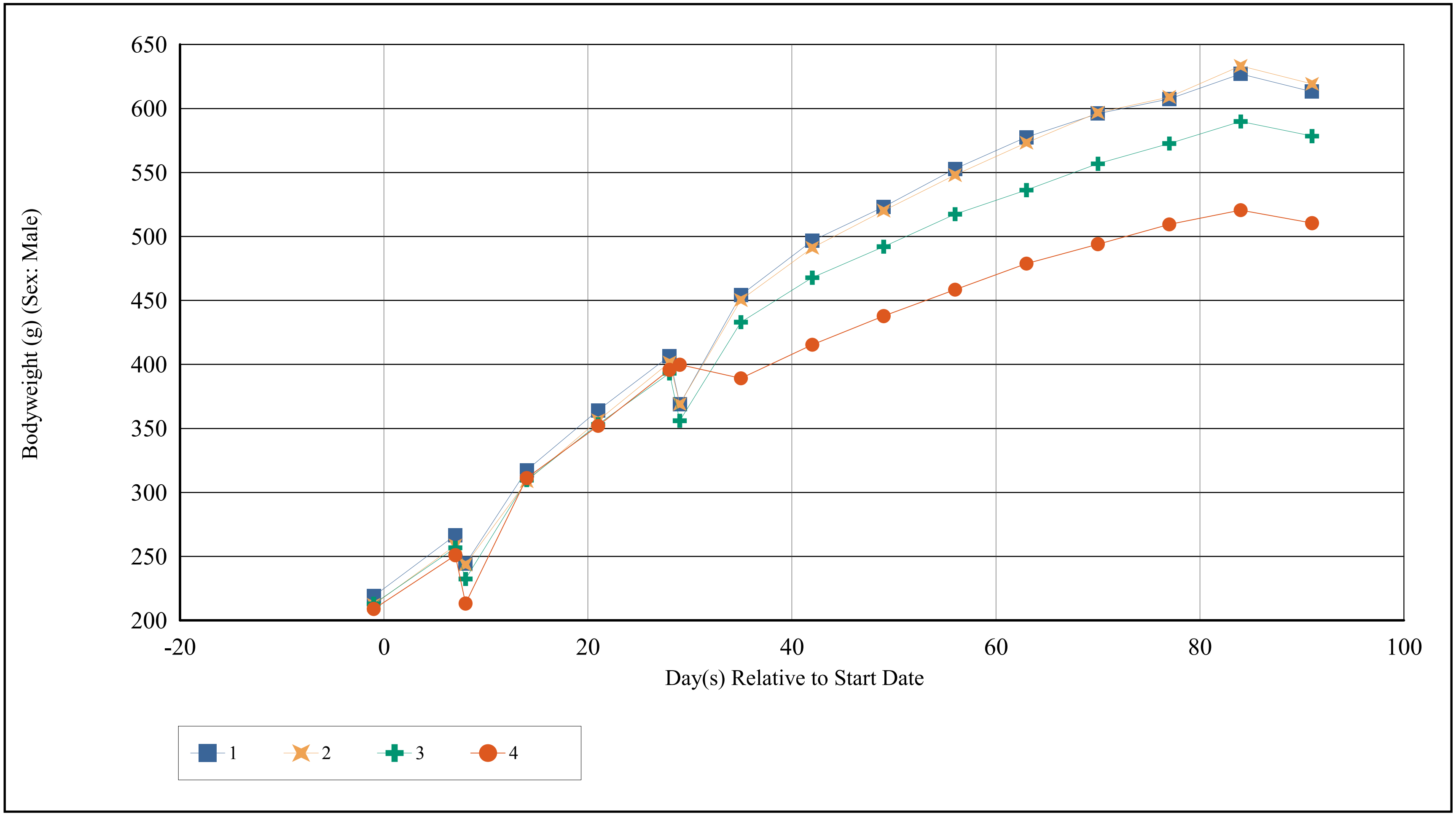

Figure 2

Summary of Body Weights

5550008

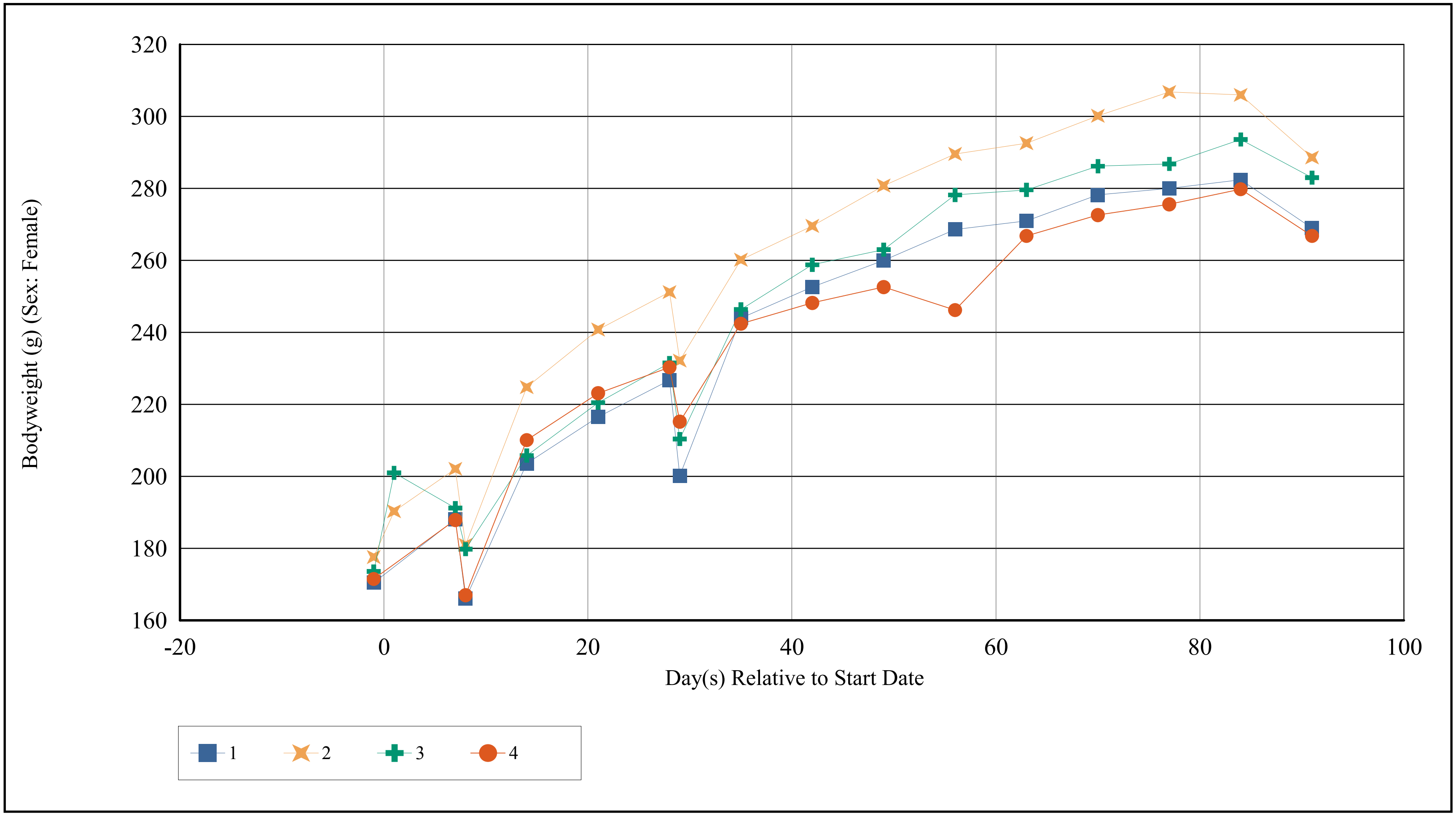

Table 1

Summary of Clinical Observations

5550008

Observation Type: All Types	Male				Female			
From Day -13 (Start Date) to -1 (Start Date)	0 vg Group 1	0.36x 10E12 vg Group 2	1.1x 10E12 vg Group 3	3.3x 10E12 vg Group 4	0 vg Group 1	0.36x 10E12 vg Group 2	1.1x 10E12 vg Group 3	3.3x 10E12 vg Group 4
**Tail, Bent (PT)**								
Number of Animals Affected	0	0	1	0	0	0	0	0
Number of Times Recorded	0	0	1	0	0	0	0	0
% of Affected Animals	0	0	7	0	0	0	0	0
First to Last seen	-	-	-1 - -1	-	-	-	-	-
**Fur, Staining, Muzzle**								
Number of Animals Affected	0	1	0	0	0	0	0	0
Number of Times Recorded	0	1	0	0	0	0	0	0
% of Affected Animals	0	6	0	0	0	0	0	0
First to Last seen	-	-1 - -1	-	-	-	-	-	-
**Fur, Thin Cover, Dorsal Cervical**								
Number of Animals Affected	1	0	0	2	0	0	0	0
Number of Times Recorded	1	0	0	2	0	0	0	0
% of Affected Animals	7	0	0	13	0	0	0	0
First to Last seen	-12 - -12	-	-	-9 - -1	-	-	-	-
**Fur, Thin Cover, Dorsal Thoracic**								
Number of Animals Affected	0	0	0	2	0	0	0	0
Number of Times Recorded	0	0	0	2	0	0	0	0
% of Affected Animals	0	0	0	13	0	0	0	0
First to Last seen	-	-	-	-9 - -1	-	-	-	-
**Fur, Thin Cover, Interscapular**								
Number of Animals Affected	1	0	0	1	0	0	0	0
Number of Times Recorded	1	0	0	1	0	0	0	0
% of Affected Animals	7	0	0	7	0	0	0	0
First to Last seen	-12 - -12	-	-	-1 - -1	-	-	-	-

Table 1

Summary of Clinical Observations

5550008

Observation Type: All Types	Male				Female			
From Day -13 (Start Date) to -1 (Start Date)	0 vg Group 1	0.36x 10E12 vg Group 2	1.1x 10E12 vg Group 3	3.3x 10E12 vg Group 4	0 vg Group 1	0.36x 10E12 vg Group 2	1.1x 10E12 vg Group 3	3.3x 10E12 vg Group 4
**Fur, Thin Cover, Lumbar**								
Number of Animals Affected	0	0	0	1	0	0	0	0
Number of Times Recorded	0	0	0	1	0	0	0	0
% of Affected Animals	0	0	0	7	0	0	0	0
First to Last seen	-	-	-	-9 - -9	-	-	-	-
**Fur, Thin Cover, Scapular, Right**								
Number of Animals Affected	0	1	0	0	0	0	0	0
Number of Times Recorded	0	1	0	0	0	0	0	0
% of Affected Animals	0	6	0	0	0	0	0	0
First to Last seen	-	-7 - -7	-	-	-	-	-	-

Table 1

Summary of Clinical Observations

5550008

Observation Type: All Types	Male				Female			
From Day 1 (Start Date) to 8 (Start Date)	0 vg Group 1	0.36x 10E12 vg Group 2	1.1x 10E12 vg Group 3	3.3x 10E12 vg Group 4	0 vg Group 1	0.36x 10E12 vg Group 2	1.1x 10E12 vg Group 3	3.3x 10E12 vg Group 4
**Limited Usage, Hindlimb, Left**								
Number of Animals Affected	1	0	0	0	0	0	0	0
Number of Times Recorded	1	0	0	0	0	0	0	0
% of Affected Animals	7	0	0	0	0	0	0	0
First to Last seen	7 - 7	-	-	-	-	-	-	-
**Limited Usage, Hindlimb, Right**								
Number of Animals Affected	1	0	0	0	0	0	0	0
Number of Times Recorded	1	0	0	0	0	0	0	0
% of Affected Animals	7	0	0	0	0	0	0	0
First to Last seen	7 - 7	-	-	-	-	-	-	-
**Limited Usage, Hindpaw, Left**								
Number of Animals Affected	1	0	0	0	0	0	0	0
Number of Times Recorded	1	0	0	0	0	0	0	0
% of Affected Animals	7	0	0	0	0	0	0	0
First to Last seen	7 - 7	-	-	-	-	-	-	-
**Limited Usage, Hindpaw, Right**								
Number of Animals Affected	1	0	0	0	0	0	0	0
Number of Times Recorded	1	0	0	0	0	0	0	0
% of Affected Animals	7	0	0	0	0	0	0	0
First to Last seen	7 - 7	-	-	-	-	-	-	-
**Breathing, Labored**								
Number of Animals Affected	1	0	0	0	0	0	0	0
Number of Times Recorded	1	0	0	0	0	0	0	0
% of Affected Animals	7	0	0	0	0	0	0	0
First to Last seen	7 - 7	-	-	-	-	-	-	-

Table 1

Summary of Clinical Observations

5550008

Observation Type: All Types	Male				Female			
From Day 1 (Start Date) to 8 (Start Date)	0 vg Group 1	0.36x 10E12 vg Group 2	1.1x 10E12 vg Group 3	3.3x 10E12 vg Group 4	0 vg Group 1	0.36x 10E12 vg Group 2	1.1x 10E12 vg Group 3	3.3x 10E12 vg Group 4
**Tail, Bent (PT)**								
Number of Animals Affected	0	0	1	0	0	0	0	0
Number of Times Recorded	0	0	1	0	0	0	0	0
% of Affected Animals	0	0	7	0	0	0	0	0
First to Last seen	-	-	7 - 7	-	-	-	-	-
**Prostrate**								
Number of Animals Affected	1	0	0	0	0	0	0	0
Number of Times Recorded	1	0	0	0	0	0	0	0
% of Affected Animals	7	0	0	0	0	0	0	0
First to Last seen	7 - 7	-	-	-	-	-	-	-
**Fur, Staining, Muzzle**								
Number of Animals Affected	0	1	0	0	0	0	0	0
Number of Times Recorded	0	2	0	0	0	0	0	0
% of Affected Animals	0	6	0	0	0	0	0	0
First to Last seen	-	7 - 8	-	-	-	-	-	-
**Fur, Thin Cover, Cranium**								
Number of Animals Affected	0	0	1	0	0	0	0	0
Number of Times Recorded	0	0	1	0	0	0	0	0
% of Affected Animals	0	0	7	0	0	0	0	0
First to Last seen	-	-	7 - 7	-	-	-	-	-
**Fur, Thin Cover, Dorsal Cervical**								
Number of Animals Affected	0	0	1	0	0	0	0	0
Number of Times Recorded	0	0	1	0	0	0	0	0
% of Affected Animals	0	0	7	0	0	0	0	0
First to Last seen	-	-	7 - 7	-	-	-	-	-

Table 1

Summary of Clinical Observations

5550008

Observation Type: All Types	Male				Female			
From Day 1 (Start Date) to 8 (Start Date)	0 vg Group 1	0.36x 10E12 vg Group 2	1.1x 10E12 vg Group 3	3.3x 10E12 vg Group 4	0 vg Group 1	0.36x 10E12 vg Group 2	1.1x 10E12 vg Group 3	3.3x 10E12 vg Group 4
**Fur, Thin Cover, Lumbar**								
Number of Animals Affected	0	0	0	1	0	0	0	0
Number of Times Recorded	0	0	0	2	0	0	0	0
% of Affected Animals	0	0	0	7	0	0	0	0
First to Last seen	-	-	-	7 - 8	-	-	-	-
**Fur, Thin Cover, Scapular, Right**								
Number of Animals Affected	0	0	1	0	0	0	0	0
Number of Times Recorded	0	0	2	0	0	0	0	0
% of Affected Animals	0	0	7	0	0	0	0	0
First to Last seen	-	-	7 - 8	-	-	-	-	-
**Skin, Discolored, Lumbar**								
Number of Animals Affected	0	0	0	0	1	0	0	0
Number of Times Recorded	0	0	0	0	1	0	0	0
% of Affected Animals	0	0	0	0	7	0	0	0
First to Last seen	-	-	-	-	8 - 8	-	-	-
**Skin, Discolored, Tail**								
Number of Animals Affected	0	1	0	0	0	0	0	0
Number of Times Recorded	0	1	0	0	0	0	0	0
% of Affected Animals	0	6	0	0	0	0	0	0
First to Last seen	-	7 - 7	-	-	-	-	-	-
**Skin, Scab, Dorsal Cervical**								
Number of Animals Affected	0	0	0	1	1	1	1	1
Number of Times Recorded	0	0	0	1	1	1	1	1
% of Affected Animals	0	0	0	7	7	7	7	7
First to Last seen	-	-	-	7 - 7	7 - 7	7 - 7	7 - 7	7 - 7

Table 1

Summary of Clinical Observations

5550008

Observation Type: All Types	Male				Female			
From Day 1 (Start Date) to 8 (Start Date)	0 vg Group 1	0.36x 10E12 vg Group 2	1.1x 10E12 vg Group 3	3.3x 10E12 vg Group 4	0 vg Group 1	0.36x 10E12 vg Group 2	1.1x 10E12 vg Group 3	3.3x 10E12 vg Group 4
**Skin, Scab, Dorsal Thoracic**								
Number of Animals Affected	0	0	0	3	1	1	0	0
Number of Times Recorded	0	0	0	3	1	1	0	0
% of Affected Animals	0	0	0	20	7	7	0	0
First to Last seen	-	-	-	8 - 8	8 - 8	8 - 8	-	-
**Skin, Scab, Lumbar**								
Number of Animals Affected	0	0	0	1	0	2	3	1
Number of Times Recorded	0	0	0	2	0	2	6	1
% of Affected Animals	0	0	0	7	0	13	20	7
First to Last seen	-	-	-	7 - 8	-	7 - 7	7 - 8	7 - 7
**Skin, Scab, Periorbital, Right**								
Number of Animals Affected	0	0	0	0	0	0	1	0
Number of Times Recorded	0	0	0	0	0	0	1	0
% of Affected Animals	0	0	0	0	0	0	7	0
First to Last seen	-	-	-	-	-	-	8 - 8	-
**Skin, Scab, Tail**								
Number of Animals Affected	0	0	0	0	0	1	0	0
Number of Times Recorded	0	0	0	0	0	1	0	0
% of Affected Animals	0	0	0	0	0	7	0	0
First to Last seen	-	-	-	-	-	7 - 7	-	-
**Thin**								
Number of Animals Affected	1	0	0	0	0	0	0	0
Number of Times Recorded	1	0	0	0	0	0	0	0
% of Affected Animals	7	0	0	0	0	0	0	0
First to Last seen	7 - 7	-	-	-	-	-	-	-

Table 1

Summary of Clinical Observations

5550008

Observation Type: All Types	Male				Female			
From Day 1 (Start Date) to 8 (Start Date)	0 vg Group 1	0.36x 10E12 vg Group 2	1.1x 10E12 vg Group 3	3.3x 10E12 vg Group 4	0 vg Group 1	0.36x 10E12 vg Group 2	1.1x 10E12 vg Group 3	3.3x 10E12 vg Group 4
**Hypersensitive**								
Number of Animals Affected	0	0	0	0	0	0	0	1
Number of Times Recorded	0	0	0	0	0	0	0	1
% of Affected Animals	0	0	0	0	0	0	0	7
First to Last seen	-	-	-	-	-	-	-	7 - 7
**Swollen (Cageside), Lumbar**								
Number of Animals Affected	0	0	0	0	1	0	0	0
Number of Times Recorded	0	0	0	0	1	0	0	0
% of Affected Animals	0	0	0	0	7	0	0	0
First to Last seen	-	-	-	-	8 - 8	-	-	-
**Swollen, Lumbar**								
Number of Animals Affected	0	0	1	0	0	0	0	0
Number of Times Recorded	0	0	1	0	0	0	0	0
% of Affected Animals	0	0	7	0	0	0	0	0
First to Last seen	-	-	7 - 7	-	-	-	-	-
**Weak**								
Number of Animals Affected	1	0	0	0	0	0	0	0
Number of Times Recorded	1	0	0	0	0	0	0	0
% of Affected Animals	7	0	0	0	0	0	0	0
First to Last seen	7 - 7	-	-	-	-	-	-	-
**Backbone Prominent**								
Number of Animals Affected	1	0	0	0	0	0	0	0
Number of Times Recorded	1	0	0	0	0	0	0	0
% of Affected Animals	7	0	0	0	0	0	0	0
First to Last seen	7 - 7	-	-	-	-	-	-	-

Table 1

Summary of Clinical Observations

5550008

Observation Type: All Types	Male				Female			
From Day 1 (Start Date) to 8 (Start Date)	0 vg Group 1	0.36x 10E12 vg Group 2	1.1x 10E12 vg Group 3	3.3x 10E12 vg Group 4	0 vg Group 1	0.36x 10E12 vg Group 2	1.1x 10E12 vg Group 3	3.3x 10E12 vg Group 4
**Teeth, Discolored**								
Number of Animals Affected	0	0	0	0	0	0	0	1
Number of Times Recorded	0	0	0	0	0	0	0	1
% of Affected Animals	0	0	0	0	0	0	0	7
First to Last seen	-	-	-	-	-	-	-	7 - 7
**Low Carriage**								
Number of Animals Affected	1	0	0	0	0	0	0	0
Number of Times Recorded	1	0	0	0	0	0	0	0
% of Affected Animals	7	0	0	0	0	0	0	0
First to Last seen	7 - 7	-	-	-	-	-	-	-
**Lying On Side**								
Number of Animals Affected	1	0	0	0	0	0	0	0
Number of Times Recorded	1	0	0	0	0	0	0	0
% of Affected Animals	7	0	0	0	0	0	0	0
First to Last seen	7 - 7	-	-	-	-	-	-	-
**Activity Decreased**								
Number of Animals Affected	1	0	0	0	0	0	0	0
Number of Times Recorded	1	0	0	0	0	0	0	0
% of Affected Animals	7	0	0	0	0	0	0	0
First to Last seen	7 - 7	-	-	-	-	-	-	-
**Pinna, Missing (PT), Left**								
Number of Animals Affected	0	0	0	0	0	0	0	1
Number of Times Recorded	0	0	0	0	0	0	0	1
% of Affected Animals	0	0	0	0	0	0	0	7
First to Last seen	-	-	-	-	-	-	-	7 - 7

Table 1

Summary of Clinical Observations

5550008

Observation Type: All Types	Male				Female			
From Day 9 (Start Date) to 29 (Start Date)	0 vg Group 1	0.36x 10E12 vg Group 2	1.1x 10E12 vg Group 3	3.3x 10E12 vg Group 4	0 vg Group 1	0.36x 10E12 vg Group 2	1.1x 10E12 vg Group 3	3.3x 10E12 vg Group 4
**Tail, Bent (PT)**								
Number of Animals Affected	0	0	1	0	0	0	0	0
Number of Times Recorded	0	0	3	0	0	0	0	0
% of Affected Animals	0	0	10	0	0	0	0	0
First to Last seen	-	-	14 - 28	-	-	-	-	-
**Fur, Staining, Abdominal**								
Number of Animals Affected	0	0	0	0	0	0	1	0
Number of Times Recorded	0	0	0	0	0	0	1	0
% of Affected Animals	0	0	0	0	0	0	10	0
First to Last seen	-	-	-	-	-	-	28 - 28	-
**Fur, Staining, Dorsal Cervical**								
Number of Animals Affected	0	0	0	0	0	1	0	0
Number of Times Recorded	0	0	0	0	0	2	0	0
% of Affected Animals	0	0	0	0	0	10	0	0
First to Last seen	-	-	-	-	-	28 - 29	-	-
**Fur, Thin Cover, Cranium**								
Number of Animals Affected	0	0	0	1	0	0	0	1
Number of Times Recorded	0	0	0	1	0	0	0	3
% of Affected Animals	0	0	0	10	0	0	0	10
First to Last seen	-	-	-	28 - 28	-	-	-	21 - 29
**Fur, Thin Cover, Dorsal Cervical**								
Number of Animals Affected	0	0	0	0	1	0	0	1
Number of Times Recorded	0	0	0	0	2	0	0	3
% of Affected Animals	0	0	0	0	10	0	0	10
First to Last seen	-	-	-	-	28 - 29	-	-	21 - 29

Table 1

Summary of Clinical Observations

5550008

Observation Type: All Types	Male				Female			
From Day 9 (Start Date) to 29 (Start Date)	0 vg Group 1	0.36x 10E12 vg Group 2	1.1x 10E12 vg Group 3	3.3x 10E12 vg Group 4	0 vg Group 1	0.36x 10E12 vg Group 2	1.1x 10E12 vg Group 3	3.3x 10E12 vg Group 4
**Fur, Thin Cover, Dorsal Thoracic**								
Number of Animals Affected	0	0	0	0	1	0	0	0
Number of Times Recorded	0	0	0	0	2	0	0	0
% of Affected Animals	0	0	0	0	10	0	0	0
First to Last seen	-	-	-	-	28 - 29	-	-	-
**Fur, Thin Cover, Forelimb, Left**								
Number of Animals Affected	0	0	0	0	0	1	0	1
Number of Times Recorded	0	0	0	0	0	4	0	3
% of Affected Animals	0	0	0	0	0	10	0	10
First to Last seen	-	-	-	-	-	14 - 29	-	21 - 29
**Fur, Thin Cover, Forelimb, Right**								
Number of Animals Affected	0	0	0	0	0	0	0	1
Number of Times Recorded	0	0	0	0	0	0	0	3
% of Affected Animals	0	0	0	0	0	0	0	10
First to Last seen	-	-	-	-	-	-	-	21 - 29
**Fur, Thin Cover, Hindlimb, Left**								
Number of Animals Affected	0	0	0	0	0	0	0	1
Number of Times Recorded	0	0	0	0	0	0	0	3
% of Affected Animals	0	0	0	0	0	0	0	10
First to Last seen	-	-	-	-	-	-	-	21 - 29
**Fur, Thin Cover, Hindlimb, Right**								
Number of Animals Affected	0	0	0	0	0	0	0	1
Number of Times Recorded	0	0	0	0	0	0	0	3
% of Affected Animals	0	0	0	0	0	0	0	10
First to Last seen	-	-	-	-	-	-	-	21 - 29

Table 1

Summary of Clinical Observations

5550008

Observation Type: All Types	Male				Female			
From Day 9 (Start Date) to 29 (Start Date)	0 vg Group 1	0.36x 10E12 vg Group 2	1.1x 10E12 vg Group 3	3.3x 10E12 vg Group 4	0 vg Group 1	0.36x 10E12 vg Group 2	1.1x 10E12 vg Group 3	3.3x 10E12 vg Group 4
**Fur, Thin Cover, Interscapular**								
Number of Animals Affected	0	0	0	0	2	0	1	0
Number of Times Recorded	0	0	0	0	2	0	1	0
% of Affected Animals	0	0	0	0	20	0	10	0
First to Last seen	-	-	-	-	28 - 28	-	28 - 28	-
**Fur, Thin Cover, Lumbar**								
Number of Animals Affected	5	3	4	0	0	1	0	0
Number of Times Recorded	8	6	6	0	0	2	0	0
% of Affected Animals	50	30	40	0	0	10	0	0
First to Last seen	21 - 29	21 - 29	28 - 29	-	-	28 - 29	-	-
**Fur, Thin Cover, Sacral**								
Number of Animals Affected	0	0	0	0	2	0	1	0
Number of Times Recorded	0	0	0	0	3	0	1	0
% of Affected Animals	0	0	0	0	20	0	10	0
First to Last seen	-	-	-	-	28 - 29	-	28 - 28	-
**Fur, Thin Cover, Ventral Thoracic**								
Number of Animals Affected	0	0	0	0	0	0	0	1
Number of Times Recorded	0	0	0	0	0	0	0	3
% of Affected Animals	0	0	0	0	0	0	0	10
First to Last seen	-	-	-	-	-	-	-	21 - 29
**Skin, Discolored, Tail**								
Number of Animals Affected	0	1	0	0	0	1	0	0
Number of Times Recorded	0	4	0	0	0	2	0	0
% of Affected Animals	0	10	0	0	0	10	0	0
First to Last seen	-	14 - 29	-	-	-	21 - 28	-	-

Table 1

Summary of Clinical Observations

5550008

Observation Type: All Types	Male				Female			
From Day 9 (Start Date) to 29 (Start Date)	0 vg Group 1	0.36x 10E12 vg Group 2	1.1x 10E12 vg Group 3	3.3x 10E12 vg Group 4	0 vg Group 1	0.36x 10E12 vg Group 2	1.1x 10E12 vg Group 3	3.3x 10E12 vg Group 4
**Skin, Scab, Cranium**								
Number of Animals Affected	0	0	0	1	0	0	0	0
Number of Times Recorded	0	0	0	1	0	0	0	0
% of Affected Animals	0	0	0	10	0	0	0	0
First to Last seen	-	-	-	28 - 28	-	-	-	-
**Skin, Scab, Dorsal Cervical**								
Number of Animals Affected	0	0	0	0	0	1	0	0
Number of Times Recorded	0	0	0	0	0	2	0	0
% of Affected Animals	0	0	0	0	0	10	0	0
First to Last seen	-	-	-	-	-	14 - 21	-	-
**Skin, Scab, Lumbar**								
Number of Animals Affected	0	3	3	3	0	1	2	2
Number of Times Recorded	0	3	6	11	0	4	3	6
% of Affected Animals	0	30	30	30	0	10	20	20
First to Last seen	-	28 - 28	14 - 29	14 - 29	-	14 - 29	28 - 29	14 - 28
**Skin, Scab, Tail**								
Number of Animals Affected	0	0	1	0	0	1	0	0
Number of Times Recorded	0	0	2	0	0	1	0	0
% of Affected Animals	0	0	10	0	0	10	0	0
First to Last seen	-	-	21 - 28	-	-	14 - 14	-	-
**Skin, Staining, Tail**								
Number of Animals Affected	0	0	1	0	0	0	0	0
Number of Times Recorded	0	0	2	0	0	0	0	0
% of Affected Animals	0	0	10	0	0	0	0	0
First to Last seen	-	-	28 - 29	-	-	-	-	-

Table 1

Summary of Clinical Observations

5550008

Observation Type: All Types	Male				Female			
From Day 9 (Start Date) to 29 (Start Date)	0 vg Group 1	0.36x 10E12 vg Group 2	1.1x 10E12 vg Group 3	3.3x 10E12 vg Group 4	0 vg Group 1	0.36x 10E12 vg Group 2	1.1x 10E12 vg Group 3	3.3x 10E12 vg Group 4
**Hypersensitive**								
Number of Animals Affected	0	0	0	0	1	0	0	1
Number of Times Recorded	0	0	0	0	1	0	0	2
% of Affected Animals	0	0	0	0	10	0	0	10
First to Last seen	-	-	-	-	28 - 28	-	-	14 - 21
**Swollen, Lumbar**								
Number of Animals Affected	0	0	1	0	0	0	0	0
Number of Times Recorded	0	0	2	0	0	0	0	0
% of Affected Animals	0	0	10	0	0	0	0	0
First to Last seen	-	-	14 - 21	-	-	-	-	-
**Teeth, Discolored**								
Number of Animals Affected	0	0	0	0	1	0	0	1
Number of Times Recorded	0	0	0	0	2	0	0	3
% of Affected Animals	0	0	0	0	10	0	0	10
First to Last seen	-	-	-	-	21 - 28	-	-	14 - 28
**Pinna, Missing (PT), Left**								
Number of Animals Affected	0	0	0	0	0	0	0	1
Number of Times Recorded	0	0	0	0	0	0	0	4
% of Affected Animals	0	0	0	0	0	0	0	10
First to Last seen	-	-	-	-	-	-	-	14 - 29
**Pinna, Missing (PT), Right**								
Number of Animals Affected	0	0	0	0	1	0	0	0
Number of Times Recorded	0	0	0	0	2	0	0	0
% of Affected Animals	0	0	0	0	10	0	0	0
First to Last seen	-	-	-	-	21 - 28	-	-	-

Table 1

Summary of Clinical Observations

5550008

Observation Type: All Types	Male				Female			
From Day 30 (Start Date) to 91 (Start Date)	0 vg Group 1	0.36x 10E12 vg Group 2	1.1x 10E12 vg Group 3	3.3x 10E12 vg Group 4	0 vg Group 1	0.36x 10E12 vg Group 2	1.1x 10E12 vg Group 3	3.3x 10E12 vg Group 4
**Vocalization**								
Number of Animals Affected	0	0	0	0	1	0	0	0
Number of Times Recorded	0	0	0	0	1	0	0	0
% of Affected Animals	0	0	0	0	20	0	0	0
First to Last seen	-	-	-	-	35 - 35	-	-	-
**Tail, Bent (PT)**								
Number of Animals Affected	0	0	1	0	0	0	0	0
Number of Times Recorded	0	0	9	0	0	0	0	0
% of Affected Animals	0	0	20	0	0	0	0	0
First to Last seen	-	-	35 - 91	-	-	-	-	-
**Fur, Staining, Cranium**								
Number of Animals Affected	0	0	0	0	0	0	2	2
Number of Times Recorded	0	0	0	0	0	0	4	8
% of Affected Animals	0	0	0	0	0	0	40	40
First to Last seen	-	-	-	-	-	-	35 - 56	35 - 91
**Fur, Staining, Dorsal Cervical**								
Number of Animals Affected	0	0	0	0	0	1	1	3
Number of Times Recorded	0	0	0	0	0	2	7	15
% of Affected Animals	0	0	0	0	0	20	20	60
First to Last seen	-	-	-	-	-	42 - 49	35 - 91	42 - 91
**Fur, Staining, Ventral Cervical**								
Number of Animals Affected	0	0	0	0	0	0	1	1
Number of Times Recorded	0	0	0	0	0	0	3	8
% of Affected Animals	0	0	0	0	0	0	20	20
First to Last seen	-	-	-	-	-	-	77 - 91	42 - 91

Table 1

Summary of Clinical Observations

5550008

Observation Type: All Types	Male				Female			
From Day 30 (Start Date) to 91 (Start Date)	0 vg Group 1	0.36x 10E12 vg Group 2	1.1x 10E12 vg Group 3	3.3x 10E12 vg Group 4	0 vg Group 1	0.36x 10E12 vg Group 2	1.1x 10E12 vg Group 3	3.3x 10E12 vg Group 4
**Fur, Thin Cover, Cranium**								
Number of Animals Affected	0	0	0	1	2	1	0	1
Number of Times Recorded	0	0	0	9	6	8	0	1
% of Affected Animals	0	0	0	20	40	20	0	20
First to Last seen	-	-	-	35 - 91	77 - 91	42 - 91	-	91 - 91
**Fur, Thin Cover, Dorsal Cervical**								
Number of Animals Affected	0	0	0	1	0	1	0	1
Number of Times Recorded	0	0	0	4	0	4	0	2
% of Affected Animals	0	0	0	20	0	20	0	20
First to Last seen	-	-	-	70 - 91	-	42 - 63	-	56 - 63
**Fur, Thin Cover, Dorsal Thoracic**								
Number of Animals Affected	2	0	1	2	0	1	0	0
Number of Times Recorded	6	0	4	2	0	2	0	0
% of Affected Animals	40	0	20	40	0	20	0	0
First to Last seen	42 - 77	-	70 - 91	63 - 63	-	56 - 63	-	-
**Fur, Thin Cover, Forepaw, Left**								
Number of Animals Affected	1	0	0	0	1	0	0	0
Number of Times Recorded	2	0	0	0	5	0	0	0
% of Affected Animals	20	0	0	0	20	0	0	0
First to Last seen	56 - 63	-	-	-	35 - 63	-	-	-
**Fur, Thin Cover, Forepaw, Right**								
Number of Animals Affected	1	0	0	0	1	0	0	0
Number of Times Recorded	2	0	0	0	5	0	0	0
% of Affected Animals	20	0	0	0	20	0	0	0
First to Last seen	56 - 63	-	-	-	35 - 63	-	-	-

Table 1

Summary of Clinical Observations

5550008

Observation Type: All Types	Male				Female			
From Day 30 (Start Date) to 91 (Start Date)	0 vg Group 1	0.36x 10E12 vg Group 2	1.1x 10E12 vg Group 3	3.3x 10E12 vg Group 4	0 vg Group 1	0.36x 10E12 vg Group 2	1.1x 10E12 vg Group 3	3.3x 10E12 vg Group 4
**Fur, Thin Cover, Hindlimb, Left**								
Number of Animals Affected	0	0	0	0	0	0	0	1
Number of Times Recorded	0	0	0	0	0	0	0	6
% of Affected Animals	0	0	0	0	0	0	0	20
First to Last seen	-	-	-	-	-	-	-	56 - 91
**Fur, Thin Cover, Hindlimb, Right**								
Number of Animals Affected	0	0	0	0	0	0	0	1
Number of Times Recorded	0	0	0	0	0	0	0	6
% of Affected Animals	0	0	0	0	0	0	0	20
First to Last seen	-	-	-	-	-	-	-	56 - 91
**Fur, Thin Cover, Interscapular**								
Number of Animals Affected	0	0	0	0	2	0	1	0
Number of Times Recorded	0	0	0	0	6	0	5	0
% of Affected Animals	0	0	0	0	40	0	20	0
First to Last seen	-	-	-	-	35 - 63	-	35 - 63	-
**Fur, Thin Cover, Lumbar**								
Number of Animals Affected	4	2	3	4	0	1	0	5
Number of Times Recorded	13	10	7	10	0	4	0	29
% of Affected Animals	80	40	60	80	0	20	0	100
First to Last seen	35 - 91	35 - 91	35 - 77	49 - 91	-	56 - 77	-	35 - 91
**Fur, Thin Cover, Sacral**								
Number of Animals Affected	0	0	0	0	1	0	1	1
Number of Times Recorded	0	0	0	0	5	0	8	8
% of Affected Animals	0	0	0	0	20	0	20	20
First to Last seen	-	-	-	-	35 - 63	-	35 - 91	42 - 91

Table 1

Summary of Clinical Observations

5550008

Observation Type: All Types	Male				Female			
From Day 30 (Start Date) to 91 (Start Date)	0 vg Group 1	0.36x 10E12 vg Group 2	1.1x 10E12 vg Group 3	3.3x 10E12 vg Group 4	0 vg Group 1	0.36x 10E12 vg Group 2	1.1x 10E12 vg Group 3	3.3x 10E12 vg Group 4
**Fur, Thin Cover, Scapular, Left**								
Number of Animals Affected	0	0	0	0	0	0	0	1
Number of Times Recorded	0	0	0	0	0	0	0	1
% of Affected Animals	0	0	0	0	0	0	0	20
First to Last seen	-	-	-	-	-	-	-	35 - 35
**Fur, Thin Cover, Scapular, Right**								
Number of Animals Affected	0	0	0	0	0	0	0	1
Number of Times Recorded	0	0	0	0	0	0	0	7
% of Affected Animals	0	0	0	0	0	0	0	20
First to Last seen	-	-	-	-	-	-	-	35 - 77
**Skin, Discolored, Tail**								
Number of Animals Affected	0	0	0	0	0	1	0	0
Number of Times Recorded	0	0	0	0	0	1	0	0
% of Affected Animals	0	0	0	0	0	20	0	0
First to Last seen	-	-	-	-	-	35 - 35	-	-
**Skin, Scab, Cranium**								
Number of Animals Affected	0	0	0	1	2	1	0	0
Number of Times Recorded	0	0	0	4	6	7	0	0
% of Affected Animals	0	0	0	20	40	20	0	0
First to Last seen	-	-	-	70 - 91	77 - 91	42 - 91	-	-
**Skin, Scab, Dorsal Thoracic**								
Number of Animals Affected	1	0	0	0	1	0	0	0
Number of Times Recorded	1	0	0	0	1	0	0	0
% of Affected Animals	20	0	0	0	20	0	0	0
First to Last seen	91 - 91	-	-	-	70 - 70	-	-	-

Table 1

Summary of Clinical Observations

5550008

Observation Type: All Types	Male				Female			
From Day 30 (Start Date) to 91 (Start Date)	0 vg Group 1	0.36x 10E12 vg Group 2	1.1x 10E12 vg Group 3	3.3x 10E12 vg Group 4	0 vg Group 1	0.36x 10E12 vg Group 2	1.1x 10E12 vg Group 3	3.3x 10E12 vg Group 4
**Skin, Scab, Lumbar**								
Number of Animals Affected	0	2	3	1	2	1	0	2
Number of Times Recorded	0	17	13	1	9	3	0	7
% of Affected Animals	0	40	60	20	40	20	0	40
First to Last seen	-	35 - 91	35 - 91	77 - 77	42 - 91	56 - 70	-	35 - 56
**Skin, Scab, Tail**								
Number of Animals Affected	0	1	0	0	0	2	2	0
Number of Times Recorded	0	3	0	0	0	4	4	0
% of Affected Animals	0	20	0	0	0	40	40	0
First to Last seen	-	35 - 49	-	-	-	42 - 91	42 - 49	-
**Skin, Staining, Mouth**								
Number of Animals Affected	0	0	0	0	1	0	0	0
Number of Times Recorded	0	0	0	0	1	0	0	0
% of Affected Animals	0	0	0	0	20	0	0	0
First to Last seen	-	-	-	-	35 - 35	-	-	-
**Mass Present**								
Number of Animals Affected	0	0	0	1	0	0	0	0
Number of Times Recorded	0	0	0	8	0	0	0	0
% of Affected Animals	0	0	0	20	0	0	0	0
First to Last seen	-	-	-	42 - 91	-	-	-	-
**Hypersensitive**								
Number of Animals Affected	0	1	0	0	1	0	2	0
Number of Times Recorded	0	5	0	0	3	0	4	0
% of Affected Animals	0	20	0	0	20	0	40	0
First to Last seen	-	35 - 63	-	-	35 - 63	-	42 - 56	-

Table 1

Summary of Clinical Observations

5550008

Observation Type: All Types	Male				Female			
From Day 30 (Start Date) to 91 (Start Date)	0 vg Group 1	0.36x 10E12 vg Group 2	1.1x 10E12 vg Group 3	3.3x 10E12 vg Group 4	0 vg Group 1	0.36x 10E12 vg Group 2	1.1x 10E12 vg Group 3	3.3x 10E12 vg Group 4
**Teeth, Discolored**								
Number of Animals Affected	0	0	0	0	1	0	0	1
Number of Times Recorded	0	0	0	0	1	0	0	4
% of Affected Animals	0	0	0	0	20	0	0	20
First to Last seen	-	-	-	-	35 - 35	-	-	35 - 70
**Pinna, Missing (PT), Right**								
Number of Animals Affected	0	0	0	0	1	0	0	0
Number of Times Recorded	0	0	0	0	9	0	0	0
% of Affected Animals	0	0	0	0	20	0	0	0
First to Last seen	-	-	-	-	35 - 91	-	-	-

Table 2

Summary of Body Weights

5550008

Bodyweight (g)

Sex: Male		Day(s) Relative to Start Date						
		-1 [G]	7 [G]	8 [G]	14 [G]	21 [G1]	28 [G1]	29 [G1]
0	Mean	218.9	266.6	244.3	317.4	364.1	406.5	368.8
vg	SD	27.8	25.8	16.5	27.8	33.2	36.4	23.7
	N	15	14	4	10	10	10	5
Group 1		-	-	-	-	-	-	-
0.36x	Mean	212.8	258.5	243.6	308.8	356.3	401.5	369.0
10E12	SD	26.1	25.9	38.6	11.8	15.4	17.8	15.7
vg	N	15	15	5	10	10	10	5
Group 2	%Diff	-2.8	-3.1	-0.3	-2.7	-2.1	-1.2	0.1
1.1x	Mean	212.9	256.7	232.4	309.6	353.2	393.1	356.0
10E12	SD	23.0	22.6	23.5	24.5	25.1	26.7	25.4
vg	N	15	15	5	10	10	10	5
Group 3	%Diff	-2.7	-3.7	-4.9	-2.5	-3.0	-3.3	-3.5
3.3x	Mean	208.9	250.9	213.2	311.2	352.0	395.8	399.8
10E12	SD	30.1	33.0	28.4	41.9	52.8	66.7	71.9
vg	N	15	15	5	10	10	10	5
Group 4	%Diff	-4.5	-5.9	-12.7	-2.0	-3.3	-2.6	8.4

[G] - Anova & Dunnett

[G1] - Kruskal-Wallis & Dunn

Table 2

Summary of Body Weights

5550008
Bodyweight (g)

Sex: Male		Day(s) Relative to Start Date						
		35	42	49	56	63	70	77
0	Mean	454.2	496.6	523.0	552.8	577.6	596.0	607.4
vg	SD	50.0	56.7	59.5	63.2	64.1	70.7	74.6
	N	5	5	5	5	5	5	5
Group 1		-	-	-	-	-	-	-
0.36x	Mean	450.4	491.2	520.2	548.0	573.2	596.8	609.0
10E12	SD	21.2	24.8	19.9	23.3	21.4	25.8	30.0
vg	N	5	5	5	5	5	5	5
Group 2	%Diff	-0.8	-1.1	-0.5	-0.9	-0.8	0.1	0.3
1.1x	Mean	433.0	467.8	492.0	517.4	536.2	556.8	572.6
10E12	SD	34.6	39.4	45.5	52.0	59.2	65.2	64.4
vg	N	5	5	5	5	5	5	5
Group 3	%Diff	-4.7	-5.8	-5.9	-6.4	-7.2	-6.6	-5.7
3.3x	Mean	389.2*	415.4*	437.8*	458.4*	478.8*	494.0*	509.4*
10E12	SD	21.9	27.4	23.6	32.2	37.5	41.3	47.5
vg	N	5	5	5	5	5	5	5
Group 4	%Diff	-14.3	-16.4	-16.3	-17.1	-17.1	-17.1	-16.1

Anova & Dunnett: * = p ≤ 0.05

Table 2

Summary of Body Weights

5550008

Bodyweight (g)

Sex: Male		Day(s) Relative to Start Date	
		84	91
0 vg Group 1	Mean	627.0	613.2
	SD	75.4	75.8
	N	5	5
		-	-
0.36x 10E12 vg Group 2	Mean	633.2	619.2
	SD	31.5	33.7
	N	5	5
	%Diff	1.0	1.0
1.1x 10E12 vg Group 3	Mean	589.8	578.4
	SD	70.4	67.2
	N	5	5
	%Diff	-5.9	-5.7
3.3x 10E12 vg Group 4	Mean	520.6*	510.4*
	SD	46.9	51.5
	N	5	5
	%Diff	-17.0	-16.8

Anova & Dunnett: * = $p \leq 0.05$

Table 2

Summary of Body Weights

5550008

Bodyweight (g)

Sex: Female		Day(s) Relative to Start Date						
		-7 [I]	-6 [I]	-4 [I]	-1 [G]	1 [I]	7 [G]	8 [G1]
0	Mean	-	-	-	170.5	-	188.1	166.0
vg	SD	-	-	-	13.9	-	14.4	6.4
	N	-	-	-	15	-	15	5
Group 1		-	-	-	-	-	-	-
0.36x	Mean	-	165.5n	184.0n	177.6	190.3n	202.1	181.0
10E12	SD	-	21.9	-	20.2	15.2	18.1	19.9
vg	N	-	2	1	12	3	15	5
Group 2	%Diff	-	-	-	4.1	-	7.5	9.0
1.1x	Mean	175.0n	-	-	173.6	201.0n	191.2	179.8
10E12	SD	-	-	-	18.2	-	14.4	6.6
vg	N	1	-	-	14	1	15	5
Group 3	%Diff	-	-	-	1.8	-	1.7	8.3
3.3x	Mean	-	-	-	171.5	-	187.9	167.0
10E12	SD	-	-	-	15.3	-	15.7	11.0
vg	N	-	-	-	15	-	15	5
Group 4	%Diff	-	-	-	0.6	-	-0.1	0.6

[G] - Anova & Dunnett
[G1] - Kruskal-Wallis & Dunn
[I] - n - Inappropriate for statistics

Table 2

Summary of Body Weights

5550008

Bodyweight (g)

Sex: Female		Day(s) Relative to Start Date						
		14	21	28	29	35	42	49
0	Mean	203.6	216.5	226.7	200.2	244.0	252.6	260.0
vg	SD	19.2	20.3	20.6	18.6	21.9	20.4	22.0
	N	10	10	10	5	5	5	5
Group 1		-	-	-	-	-	-	-
0.36x	Mean	224.8	240.8*	251.2*	232.2	260.2	269.6	280.8
10E12	SD	22.1	21.6	22.2	18.1	30.8	27.6	35.3
vg	N	10	10	10	5	5	5	5
Group 2	%Diff	10.4	11.2	10.8	16.0	6.6	6.7	8.0
1.1x	Mean	205.8	220.5	231.5	210.4	246.4	258.8	263.0
10E12	SD	18.8	19.7	18.9	18.1	27.2	24.2	28.2
vg	N	10	10	10	5	5	5	5
Group 3	%Diff	1.1	1.8	2.1	5.1	1.0	2.5	1.2
3.3x	Mean	210.1	223.1	230.3	215.2	242.4	248.2	252.6
10E12	SD	18.4	18.3	17.3	25.2	16.6	13.0	16.7
vg	N	10	10	10	5	5	5	5
Group 4	%Diff	3.2	3.0	1.6	7.5	-0.7	-1.7	-2.8

Anova & Dunnett: * = $p \leq 0.05$

Table 2

Summary of Body Weights

5550008

Bodyweight (g)

Sex: Female		Day(s) Relative to Start Date					
		56	63	70	77	84	91
0	Mean	268.6	271.0	278.2	280.0	282.4	269.0
vg	SD	20.1	24.3	29.4	28.0	27.8	23.9
	N	5	5	5	5	5	5
Group 1		-	-	-	-	-	-
0.36x	Mean	289.6	292.6	300.2	306.8	306.0	288.6
10E12	SD	36.3	34.5	33.6	32.8	35.0	32.3
vg	N	5	5	5	5	5	5
Group 2	%Diff	7.8	8.0	7.9	9.6	8.4	7.3
1.1x	Mean	278.2	279.6	286.2	286.8	293.6	283.0
10E12	SD	40.5	31.1	37.0	34.0	43.9	49.1
vg	N	5	5	5	5	5	5
Group 3	%Diff	3.6	3.2	2.9	2.4	4.0	5.2
3.3x	Mean	246.2	266.8	272.6	275.6	279.8	266.8
10E12	SD	17.0	23.5	24.3	24.0	26.8	27.1
vg	N	5	5	5	5	5	5
Group 4	%Diff	-8.3	-1.5	-2.0	-1.6	-0.9	-0.8

Anova & Dunnett

Table 3

Summary of Body Weight Gains (g)

5550008

Bodyweight Gain (Interval)

Sex: Male		Day(s) Relative to Start Date						
		-1 → 7 [G]	7 → 8 [G]	7 → 14 [G]	14 → 21 [G]	21 → 28 [G]	7 → 28 [G1]	28 → 29 [G1]
0	Mean	47.8	-32.0	54.6	46.7	42.4	143.7	-27.6
vg	SD	10.8	5.2	10.5	6.6	7.1	15.6	4.3
	N	14	4	10	10	10	10	5
Group 1		-	-	-	-	-	-	-
0.36x	Mean	45.7	-27.2	56.5	47.5	45.2	149.2	-26.2
10E12	SD	7.2	4.9	9.6	5.4	6.3	16.6	4.1
vg	N	15	5	10	10	10	10	5
Group 2		-	-	-	-	-	-	-
1.1x	Mean	43.8	-26.0	53.8	43.6	39.9	137.3	-32.2
10E12	SD	7.5	3.4	8.2	7.9	7.5	18.1	4.3
vg	N	15	5	10	10	10	10	5
Group 3		-	-	-	-	-	-	-
3.3x	Mean	42.0	-24.8	53.8	40.8	43.8	138.4	-31.0
10E12	SD	10.0	4.1	14.3	13.6	15.4	41.8	9.7
vg	N	15	5	10	10	10	10	5
Group 4		-	-	-	-	-	-	-

[G] - Anova & Dunnett
[G1] - Kruskal-Wallis & Dunn

Table 3

Summary of Body Weight Gains (g)

5550008

Bodyweight Gain (Interval)

Sex: Male		Day(s) Relative to Start Date						
		28 → 35	35 → 42	42 → 49	49 → 56	56 → 63	63 → 70	70 → 77
0	Mean	37.6	42.4	26.4	29.8	24.8	18.4	11.4
vg	SD	12.4	7.2	6.6	4.8	2.2	8.7	4.8
	N	5	5	5	5	5	5	5
Group 1		-	-	-	-	-	-	-
0.36x	Mean	42.6	40.8	29.0	27.8	25.2	23.6	12.2
10E12	SD	5.1	4.5	7.0	3.7	8.7	6.4	5.8
vg	N	5	5	5	5	5	5	5
Group 2		-	-	-	-	-	-	-
1.1x	Mean	35.0	34.8	24.2	25.4	18.8	20.6	15.8
10E12	SD	11.2	10.1	7.2	8.3	10.8	6.1	7.0
vg	N	5	5	5	5	5	5	5
Group 3		-	-	-	-	-	-	-
3.3x	Mean	28.4	26.2**	22.4	20.6	20.4	15.2	15.4
10E12	SD	7.8	6.9	7.7	8.9	7.1	7.3	8.2
vg	N	5	5	5	5	5	5	5
Group 4		-	-	-	-	-	-	-

Anova & Dunnett: ** = $p \leq 0.01$

Table 3

Summary of Body Weight Gains (g)

5550008

Bodyweight Gain (Interval)

Sex: Male		Day(s) Relative to Start Date		
		77 → 84	28 → 84	84 → 91
0	Mean	19.6	210.4	-13.8
vg	SD	3.7	35.5	5.2
	N	5	5	5
Group 1		-	-	-
0.36x	Mean	24.2	225.4	-14.0
10E12	SD	4.1	19.2	7.1
vg	N	5	5	5
Group 2		-	-	-
1.1x	Mean	17.2	191.8	-11.4
10E12	SD	8.8	48.2	10.3
vg	N	5	5	5
Group 3		-	-	-
3.3x	Mean	11.2	159.8	-10.2
10E12	SD	4.4	37.4	7.7
vg	N	5	5	5
Group 4		-	-	-

Anova & Dunnett

Table 3

Summary of Body Weight Gains (g)

5550008

Bodyweight Gain (Interval)

Sex: Female		Day(s) Relative to Start Date						
		-4 → 1 [I]	-6 → 1 [I]	-7 → 1 [I]	1 → 7 [I]	-1 → 7 [G]	7 → 8 [G]	7 → 14 [G]
0	Mean	-	-	-	-	17.5	-19.8	14.4
vg	SD	-	-	-	-	6.3	2.4	4.7
	N	-	-	-	-	15	5	10
Group 1		-	-	-	-	-	-	-
0.36x	Mean	9.0n	23.5n	-	20.0n	22.5	-22.2	23.2*
10E12	SD	-	0.7	-	1.7	4.5	4.8	9.6
vg	N	1	2	-	3	12	5	10
Group 2		-	-	-	-	-	-	-
1.1x	Mean	-	-	26.0n	9.0n	16.3	-23.0	20.4
10E12	SD	-	-	-	-	7.2	2.5	8.6
vg	N	-	-	1	1	14	5	10
Group 3		-	-	-	-	-	-	-
3.3x	Mean	-	-	-	-	16.3	-21.6	22.6*
10E12	SD	-	-	-	-	5.4	5.1	4.7
vg	N	-	-	-	-	15	5	10
Group 4		-	-	-	-	-	-	-

[G] - Anova & Dunnett: * = $p \leq 0.05$
[I] - n - Inappropriate for statistics

Table 3

Summary of Body Weight Gains (g)

5550008

Bodyweight Gain (Interval)

Sex: Female		Day(s) Relative to Start Date						
		14 → 21	21 → 28	7 → 28	28 → 29	28 → 35	35 → 42	42 → 49
0	Mean	12.9	10.2	37.5	-17.8	8.6	8.6	7.4
vg	SD	7.1	6.0	9.4	5.4	8.0	6.9	7.0
	N	10	10	10	5	5	5	5
Group 1		-	-	-	-	-	-	-
0.36x	Mean	16.0	10.4	49.6	-18.0	8.0	9.4	11.2
10E12	SD	13.2	8.6	13.4	4.5	6.2	6.1	8.4
vg	N	10	10	10	5	5	5	5
Group 2		-	-	-	-	-	-	-
1.1x	Mean	14.7	11.0	46.1	-19.0	12.8	12.4	4.2
10E12	SD	4.3	6.0	9.1	4.4	12.3	6.7	4.9
vg	N	10	10	10	5	5	5	5
Group 3		-	-	-	-	-	-	-
3.3x	Mean	13.0	7.2	42.8	-16.2	13.2	5.8	4.4
10E12	SD	5.6	5.1	8.7	2.9	12.4	7.9	8.2
vg	N	10	10	10	5	5	5	5
Group 4		-	-	-	-	-	-	-

Anova & Dunnett

Table 3

Summary of Body Weight Gains (g)

5550008

Bodyweight Gain (Interval)

Sex: Female		Day(s) Relative to Start Date						
		49 → 56	56 → 63	63 → 70	70 → 77	77 → 84	28 → 84	84 → 91
0	Mean	8.6	2.4	7.2	1.8	2.4	47.0	-13.4
vg	SD	3.1	9.8	8.7	6.0	5.7	11.0	5.9
	N	5	5	5	5	5	5	5
Group 1		-	-	-	-	-	-	-
0.36x	Mean	8.8	3.0	7.6	6.6	-0.8	53.8	-17.4
10E12	SD	5.0	7.8	6.6	6.8	7.2	9.6	4.0
vg	N	5	5	5	5	5	5	5
Group 2		-	-	-	-	-	-	-
1.1x	Mean	15.2	1.4	6.6	0.6	6.8	60.0	-10.6
10E12	SD	15.3	12.3	8.0	6.0	13.2	31.2	6.7
vg	N	5	5	5	5	5	5	5
Group 3		-	-	-	-	-	-	-
3.3x	Mean	-6.4	20.6	5.8	3.0	4.2	50.6	-13.0
10E12	SD	16.2	17.1	3.6	6.6	7.8	21.1	5.2
vg	N	5	5	5	5	5	5	5
Group 4		-	-	-	-	-	-	-

Anova & Dunnett

Table 4

Summary of Food Consumption

5550008

Food Mean Daily Consumption (g/animal/day)

Sex: Male		Day(s) Relative to Start Date						
		-1 → 7	7 → 14	14 → 21	21 → 28	28 → 35	35 → 42	42 → 49
0	Mean	25.55	29.81	30.66	26.10	34.77	33.63	28.69
vg	SD	4.06	1.90	1.27	13.35	0.77	1.16	0.23
	N	12	10	10	10	5	5	5
Group 1		.	.	.	.	.	.	.
0.36x	Mean	27.04	31.23	28.97	33.18	35.03	33.71	28.29
10E12	SD	1.72	1.47	4.42	2.12	0.50	0.07	0.26
vg	N	15	10	10	8	5	5	5
Group 2	%Diff	5.85	4.74	-5.50	27.12	0.74	0.25	-1.39
1.1x	Mean	26.69	29.60	29.87	32.49	33.71	30.86	30.23
10E12	SD	1.92	2.45	1.17	1.48	0.33	1.17	2.40
vg	N	15	10	10	10	5	5	5
Group 3	%Diff	4.45	-0.72	-2.56	24.47	-3.04	-8.24	5.38
3.3x	Mean	25.34	34.67	31.90	31.10	30.14	27.57	25.26
10E12	SD	1.46	8.56	3.71	4.37	0.46	0.98	1.59
vg	N	15	10	10	7	5	5	5
Group 4	%Diff	-0.80	16.29	4.05	19.16	-13.31	-18.01	-11.95

Table 4

Summary of Food Consumption

5550008

Food Mean Daily Consumption (g/animal/day)

Sex: Male		Day(s) Relative to Start Date					
		49 → 56	56 → 63	63 → 70	70 → 77	77 → 84	84 → 90
0	Mean	31.46	33.86	32.14	31.46	32.31	31.53
vg	SD	0.42	0.98	0.39	1.85	0.03	1.57
	N	5	5	5	5	5	5
Group 1		.	.	.	.	.	.
0.36x	Mean	32.46	34.63	33.69	31.71	33.23	33.63
10E12	SD	0.37	0.73	0.29	1.76	0.40	0.41
vg	N	5	5	5	5	5	5
Group 2	%Diff	3.18	2.28	4.80	0.82	2.83	6.66
1.1x	Mean	30.29	30.29	32.26	32.51	31.51	32.37
10E12	SD	0.13	4.56	0.61	0.83	0.12	0.05
vg	N	5	5	5	5	5	5
Group 3	%Diff	-3.72	-10.55	0.36	3.36	-2.48	2.64
3.3x	Mean	26.37	28.97	28.66	27.31	28.23	28.53
10E12	SD	0.21	0.63	0.73	0.23	0.47	0.49
vg	N	5	5	5	5	5	5
Group 4	%Diff	-16.17	-14.43	-10.84	-13.17	-12.64	-9.51

Table 4

Summary of Food Consumption

5550008

Food Mean Daily Consumption (g/animal/day)

Sex: Female		Day(s) Relative to Start Date						
		-1 → 1	1 → 7	-1 → 7	7 → 14	14 → 21	21 → 28	28 → 35
0	Mean	.	.	18.36	19.19	19.69	18.96	23.60
vg	SD	.	.	0.90	1.39	1.71	1.01	3.30
	N	.	.	15	10	10	10	5
Group 1		.	.	.	.	.	.	.
0.36x	Mean	.	20.44	19.42	21.67	21.76	18.59	20.06
10E12	SD	.	0.00	1.14	1.03	1.28	4.91	0.53
vg	N	.	3	12	10	10	10	5
Group 2	%Diff	.	.	5.74	12.96	10.52	-1.96	-15.01
1.1x	Mean	26.50	20.42	18.25	20.96	20.47	20.51	20.60
10E12	SD	.	0.00	0.66	1.45	1.32	0.63	1.46
vg	N	1	2	13	10	10	7	5
Group 3	%Diff	.	.	-0.59	9.23	3.99	8.19	-12.71
3.3x	Mean	.	.	19.17	20.50	20.64	20.40	20.74
10E12	SD	.	.	2.56	1.37	1.16	0.74	0.81
vg	N	.	.	15	10	10	10	5
Group 4	%Diff	.	.	4.40	6.85	4.86	7.61	-12.11

Table 4

Summary of Food Consumption

5550008

Food Mean Daily Consumption (g/animal/day)

Sex: Female		Day(s) Relative to Start Date						
		35 → 42	42 → 49	49 → 56	56 → 63	63 → 70	70 → 77	77 → 84
0	Mean	20.00	17.23	18.46	18.71	19.29	17.54	19.17
vg	SD	1.30	1.49	0.37	1.83	0.59	0.63	1.08
	N	5	5	5	5	5	5	5
Group 1		.	.	.	.	.	.	.
0.36x	Mean	19.94	20.11	19.06	17.91	18.69	17.69	18.14
10E12	SD	1.75	0.68	0.73	2.27	2.37	2.45	0.13
vg	N	5	5	5	5	5	5	5
Group 2	%Diff	-0.29	16.75	3.25	-4.27	-3.11	0.81	-5.37
1.1x	Mean	20.86	17.00	19.54	17.17	19.11	15.89	20.11
10E12	SD	2.09	1.24	1.67	0.95	1.60	0.63	3.36
vg	N	5	5	5	5	5	5	5
Group 3	%Diff	4.29	-1.33	5.88	-8.24	-0.89	-9.45	4.92
3.3x	Mean	17.97	19.03	14.83	17.77	18.49	18.31	19.69
10E12	SD	1.72	1.13	2.63	2.01	0.08	3.22	1.28
vg	N	5	5	5	5	5	5	5
Group 4	%Diff	-10.14	10.45	-19.66	-5.04	-4.15	4.40	2.68

Table 4

Summary of Food Consumption

5550008

Food Mean Daily Consumption (g/animal/day)

Sex: Female		Day(s) Relative to Start Date 84 → 90
0 vg Group 1	Mean	18.03
	SD	0.27
	N	5
		.
0.36x 10E12 vg Group 2	Mean	18.20
	SD	0.81
	N	5
	%Diff	0.92
1.1x 10E12 vg Group 3	Mean	19.87
	SD	2.01
	N	5
	%Diff	10.17
3.3x 10E12 vg Group 4	Mean	20.27
	SD	0.17
	N	5
	%Diff	12.38

Table 5

Summary of Neurobehavorial Evaluation: Activity

5550008
Week: 4 Relative to Start Date (P1)

Sex: Male		0 vg Group 1	0.36x 10E12 vg Group 2	1.1x 10E12 vg Group 3	3.3x 10E12 vg Group 4
Arousal/Alertness	N	5	5	5	5
-2 Very low	N+ve	0	0	0	0
-1 Low	N+ve	2	0	0	0
0 Normal	N+ve	3	4	5	2
1 High	N+ve	0	1	0	3
2 Very High	N+ve	0	0	0	0
Posture/Body Carriage	N	5	5	5	5
-2 Prostrate or flattened	N+ve	0	0	0	0
-1 Hunched or lying low	N+ve	0	0	0	0
0 Normal (asleep/sit/stand)	N+ve	5	5	5	5
Stereotypy (Home Cage)	N	5	5	5	5
0 Not present; Normal	N+ve	5	5	5	5
1 Licking, biting, grooming	N+ve	0	0	0	0
2 Mouth movements/weaving	N+ve	0	0	0	0
3 Circling, retropulsion	N+ve	0	0	0	0
4 Self-mutilation	N+ve	0	0	0	0

Table 5

Summary of Neurobehavorial Evaluation: Activity

5550008

Week: 4 Relative to Start Date (P1)

Sex: Male		0 vg Group 1	0.36x 10E12 vg Group 2	1.1x 10E12 vg Group 3	3.3x 10E12 vg Group 4
Stereotypy (Open Field)	N	5	5	5	5
0 Not present; Normal	N+ve	5	5	5	5
1 Licking, biting, grooming	N+ve	0	0	0	0
2 Mouth movements/weaving	N+ve	0	0	0	0
3 Circling, retropulsion	N+ve	0	0	0	0
4 Self-mutilation	N+ve	0	0	0	0
Rearing [G]	Mean	8.0	14.8	13.6	19.8**
	SD	6.6	3.6	6.6	4.5
	N	5	5	5	5
	%Diff	-	85.0	70.0	147.5
Appearance	N	5	5	5	5
0 Clean and groomed	N+ve	5	5	5	5
1 Slightly unkempt	N+ve	0	0	0	0
2 Extremely unkempt	N+ve	0	0	0	0

[G] - Anova & Dunnett: ** = $p \leq 0.01$

Table 5

Summary of Neurobehavorial Evaluation: Activity

5550008

Week: 4 Relative to Start Date (P1)

Sex: Female		0 vg Group 1	0.36x 10E12 vg Group 2	1.1x 10E12 vg Group 3	3.3x 10E12 vg Group 4
Arousal/Alertness	N	5	5	5	5
-2 Very low	N+ve	0	0	0	0
-1 Low	N+ve	0	0	0	0
0 Normal	N+ve	5	5	5	2
1 High	N+ve	0	0	0	3
2 Very High	N+ve	0	0	0	0
Posture/Body Carriage	N	5	5	5	5
-2 Prostrate or flattened	N+ve	0	0	0	0
-1 Hunched or lying low	N+ve	0	0	0	0
0 Normal (asleep/sit/stand)	N+ve	5	5	5	5
Stereotypy (Home Cage)	N	5	5	5	5
0 Not present; Normal	N+ve	5	5	4	5
1 Licking, biting, grooming	N+ve	0	0	1	0
2 Mouth movements/weaving	N+ve	0	0	0	0
3 Circling, retropulsion	N+ve	0	0	0	0
4 Self-mutilation	N+ve	0	0	0	0

Table 5

Summary of Neurobehavorial Evaluation: Activity

5550008
Week: 4 Relative to Start Date (P1)

Sex: Female		0<br>vg<br><br>Group 1	0.36x<br>10E12<br>vg<br>Group 2	1.1x<br>10E12<br>vg<br>Group 3	3.3x<br>10E12<br>vg<br>Group 4
Stereotypy (Open Field)	N	5	5	5	5
0 Not present; Normal	N+ve	5	5	5	5
1 Licking, biting, grooming	N+ve	0	0	0	0
2 Mouth movements/weaving	N+ve	0	0	0	0
3 Circling, retropulsion	N+ve	0	0	0	0
4 Self-mutilation	N+ve	0	0	0	0
Rearing [G]	Mean<br>SD<br>N<br>%Diff	10.0<br>3.4<br>5<br>-	14.2<br>5.6<br>5<br>42.0	14.2<br>0.8<br>5<br>42.0	22.0**<br>6.7<br>5<br>120.0
Appearance	N	5	5	5	5
0 Clean and groomed	N+ve	5	5	5	5
1 Slightly unkempt	N+ve	0	0	0	0
2 Extremely unkempt	N+ve	0	0	0	0

[G] - Kruskal-Wallis & Dunn: ** = $p \leq 0.01$

Table 5

Summary of Neurobehavorial Evaluation: Activity

5550008
Week: 13 Relative to Start Date (P2)

Sex: Male		0 vg Group 1	0.36x 10E12 vg Group 2	1.1x 10E12 vg Group 3	3.3x 10E12 vg Group 4
Arousal/Alertness	N	5	5	5	5
-2 Very low	N+ve	0	0	0	0
-1 Low	N+ve	1	0	0	0
0 Normal	N+ve	4	5	4	2
1 High	N+ve	0	0	1	3
2 Very High	N+ve	0	0	0	0
Posture/Body Carriage	N	5	5	5	5
-2 Prostrate or flattened	N+ve	0	0	0	0
-1 Hunched or lying low	N+ve	0	0	0	0
0 Normal (asleep/sit/stand)	N+ve	5	5	5	5
Stereotypy (Home Cage)	N	5	5	5	5
0 Not present; Normal	N+ve	5	5	5	5
1 Licking, biting, grooming	N+ve	0	0	0	0
2 Mouth movements/weaving	N+ve	0	0	0	0
3 Circling, retropulsion	N+ve	0	0	0	0
4 Self-mutilation	N+ve	0	0	0	0

Table 5

Summary of Neurobehavorial Evaluation: Activity

5550008

Week: 13 Relative to Start Date (P2)

Sex: Male		0<br>vg<br><br>Group 1	0.36x<br>10E12<br>vg<br>Group 2	1.1x<br>10E12<br>vg<br>Group 3	3.3x<br>10E12<br>vg<br>Group 4
Stereotypy (Open Field)	N	5	5	5	5
0 Not present; Normal	N+ve	5	5	5	5
1 Licking, biting, grooming	N+ve	0	0	0	0
2 Mouth movements/weaving	N+ve	0	0	0	0
3 Circling, retropulsion	N+ve	0	0	0	0
4 Self-mutilation	N+ve	0	0	0	0
Rearing [G]	Mean<br>SD<br>N<br>%Diff	5.2<br>6.2<br>5<br>-	11.8<br>6.3<br>5<br>126.9	12.6<br>5.3<br>5<br>142.3	13.2<br>6.6<br>5<br>153.8
Appearance	N	5	5	5	5
0 Clean and groomed	N+ve	5	5	5	5
1 Slightly unkempt	N+ve	0	0	0	0
2 Extremely unkempt	N+ve	0	0	0	0

[G] - Anova & Dunnett

Table 5

Summary of Neurobehavorial Evaluation: Activity

5550008
Week: 13 Relative to Start Date (P2)

Sex: Female		0 vg Group 1	0.36x 10E12 vg Group 2	1.1x 10E12 vg Group 3	3.3x 10E12 vg Group 4
Arousal/Alertness	N	5	5	5	5
-2 Very low	N+ve	0	0	0	0
-1 Low	N+ve	0	0	0	0
0 Normal	N+ve	4	3	3	1
1 High	N+ve	1	1	2	4
2 Very High	N+ve	0	1	0	0
Posture/Body Carriage	N	5	5	5	5
-2 Prostrate or flattened	N+ve	0	0	0	0
-1 Hunched or lying low	N+ve	0	0	0	0
0 Normal (asleep/sit/stand)	N+ve	5	5	5	5
Stereotypy (Home Cage)	N	5	5	5	5
0 Not present; Normal	N+ve	5	5	5	5
1 Licking, biting, grooming	N+ve	0	0	0	0
2 Mouth movements/weaving	N+ve	0	0	0	0
3 Circling, retropulsion	N+ve	0	0	0	0
4 Self-mutilation	N+ve	0	0	0	0

Table 5

Summary of Neurobehavorial Evaluation: Activity

5550008

Week: 13 Relative to Start Date (P2)

Sex: Female		0 vg Group 1	0.36x 10E12 vg Group 2	1.1x 10E12 vg Group 3	3.3x 10E12 vg Group 4
Stereotypy (Open Field)	N	5	5	5	5
0 Not present; Normal	N+ve	5	5	5	5
1 Licking, biting, grooming	N+ve	0	0	0	0
2 Mouth movements/weaving	N+ve	0	0	0	0
3 Circling, retropulsion	N+ve	0	0	0	0
4 Self-mutilation	N+ve	0	0	0	0
Rearing [G]	Mean	11.6	16.0	12.8	19.4
	SD	4.7	11.9	6.3	7.7
	N	5	5	5	5
	%Diff	-	37.9	10.3	67.2
Appearance	N	5	5	5	5
0 Clean and groomed	N+ve	5	5	5	5
1 Slightly unkempt	N+ve	0	0	0	0
2 Extremely unkempt	N+ve	0	0	0	0

[G] - Anova & Dunnett

Table 6

Summary of Neurobehavioral Evaluation: Autonomic

5550008

Week: 4 Relative to Start Date (P1)

Sex: Male		0 vg Group 1	0.36x 10E12 vg Group 2	1.1x 10E12 vg Group 3	3.3x 10E12 vg Group 4
Exophthalmus	N	5	5	5	5
0 Not present	N+ve	5	5	5	5
1 Present	N+ve	0	0	0	0
Lacrimation	N	5	5	5	5
0 None	N+ve	5	5	5	5
1 Slight	N+ve	0	0	0	0
2 Severe	N+ve	0	0	0	0
Erected Fur	N	5	5	5	5
0 Not present	N+ve	5	5	5	5
1 Present	N+ve	0	0	0	0
Pupil Response	N	5	5	5	5
0 Normal constriction	N+ve	5	5	5	5
1 Partial/no constriction	N+ve	0	0	0	0
2 Pre-existing constriction	N+ve	0	0	0	0
Salivation	N	5	5	5	5
0 None	N+ve	5	5	5	5
1 Slight	N+ve	0	0	0	0

Table 6

Summary of Neurobehavioral Evaluation: Autonomic

5550008

Week: 4 Relative to Start Date (P1)

Sex: Male		0<br>vg<br><br>Group 1	0.36x<br>10E12<br>vg<br>Group 2	1.1x<br>10E12<br>vg<br>Group 3	3.3x<br>10E12<br>vg<br>Group 4
2 Severe	N+ve	0	0	0	0
Defecation	N	5	5	5	5
-1 Absent	N+ve	3	2	4	3
0 Normal	N+ve	2	3	1	2
1 Abnormal	N+ve	0	0	0	0
Palpebral Closure/Ptosis (HC)	N	5	5	5	5
0 Eyelids wide open	N+ve	5	5	5	5
1 Eyelids droop halfway	N+ve	0	0	0	0
2 Eyelids completely shut	N+ve	0	0	0	0
Palpebral Closure/Ptosis (OF)	N	5	5	5	5
0 Eyelids wide open	N+ve	5	5	5	5
1 Eyelids droop halfway	N+ve	0	0	0	0
2 Eyelids completely shut	N+ve	0	0	0	0

Table 6

Summary of Neurobehavioral Evaluation: Autonomic

5550008

Week: 4 Relative to Start Date (P1)

Sex: Female		0 vg Group 1	0.36x 10E12 vg Group 2	1.1x 10E12 vg Group 3	3.3x 10E12 vg Group 4
Exophthalmus	N	5	5	5	5
0 Not present	N+ve	5	5	5	5
1 Present	N+ve	0	0	0	0
Lacrimation	N	5	5	5	5
0 None	N+ve	5	5	5	5
1 Slight	N+ve	0	0	0	0
2 Severe	N+ve	0	0	0	0
Erected Fur	N	5	5	5	5
0 Not present	N+ve	5	5	5	5
1 Present	N+ve	0	0	0	0
Pupil Response	N	5	5	5	5
0 Normal constriction	N+ve	5	5	5	5
1 Partial/no constriction	N+ve	0	0	0	0
2 Pre-existing constriction	N+ve	0	0	0	0
Salivation	N	5	5	5	5
0 None	N+ve	5	5	5	5
1 Slight	N+ve	0	0	0	0

Table 6

Summary of Neurobehavioral Evaluation: Autonomic

5550008
Week: 4 Relative to Start Date (P1)

Sex: Female		0 vg Group 1	0.36x 10E12 vg Group 2	1.1x 10E12 vg Group 3	3.3x 10E12 vg Group 4
2 Severe	N+ve	0	0	0	0
Defecation	N	5	5	5	5
-1 Absent	N+ve	5	5	5	5
0 Normal	N+ve	0	0	0	0
1 Abnormal	N+ve	0	0	0	0
Palpebral Closure/Ptosis (HC)	N	5	5	5	5
0 Eyelids wide open	N+ve	5	5	5	5
1 Eyelids droop halfway	N+ve	0	0	0	0
2 Eyelids completely shut	N+ve	0	0	0	0
Palpebral Closure/Ptosis (OF)	N	5	5	5	5
0 Eyelids wide open	N+ve	5	5	5	5
1 Eyelids droop halfway	N+ve	0	0	0	0
2 Eyelids completely shut	N+ve	0	0	0	0

Table 6

Summary of Neurobehavioral Evaluation: Autonomic

5550008
Week: 13 Relative to Start Date (P2)

Sex: Male		0 vg Group 1	0.36x 10E12 vg Group 2	1.1x 10E12 vg Group 3	3.3x 10E12 vg Group 4
Exophthalmus	N	5	5	5	5
0 Not present	N+ve	5	5	5	5
1 Present	N+ve	0	0	0	0
Lacrimation	N	5	5	5	5
0 None	N+ve	5	5	5	5
1 Slight	N+ve	0	0	0	0
2 Severe	N+ve	0	0	0	0
Erected Fur	N	5	5	5	5
0 Not present	N+ve	5	5	5	5
1 Present	N+ve	0	0	0	0
Pupil Response	N	5	5	5	5
0 Normal constriction	N+ve	5	5	5	5
1 Partial/no constriction	N+ve	0	0	0	0
2 Pre-existing constriction	N+ve	0	0	0	0
Salivation	N	5	5	5	5
0 None	N+ve	5	5	5	5
1 Slight	N+ve	0	0	0	0

Table 6

Summary of Neurobehavioral Evaluation: Autonomic

5550008

Week: 13 Relative to Start Date (P2)

Sex: Male		0 vg Group 1	0.36x 10E12 vg Group 2	1.1x 10E12 vg Group 3	3.3x 10E12 vg Group 4
2 Severe	N+ve	0	0	0	0
Defecation	N	5	5	5	5
-1 Absent	N+ve	2	2	5	2
0 Normal	N+ve	3	3	0	3
1 Abnormal	N+ve	0	0	0	0
Palpebral Closure/Ptosis (HC)	N	5	5	5	5
0 Eyelids wide open	N+ve	5	5	5	5
1 Eyelids droop halfway	N+ve	0	0	0	0
2 Eyelids completely shut	N+ve	0	0	0	0
Palpebral Closure/Ptosis (OF)	N	5	5	5	5
0 Eyelids wide open	N+ve	5	5	5	5
1 Eyelids droop halfway	N+ve	0	0	0	0
2 Eyelids completely shut	N+ve	0	0	0	0

Table 6

Summary of Neurobehavioral Evaluation: Autonomic

5550008
Week: 13 Relative to Start Date (P2)

Sex: Female		0 vg Group 1	0.36x 10E12 vg Group 2	1.1x 10E12 vg Group 3	3.3x 10E12 vg Group 4
Exophthalmus	N	5	5	5	5
0 Not present	N+ve	5	5	5	5
1 Present	N+ve	0	0	0	0
Lacrimation	N	5	5	5	5
0 None	N+ve	5	5	5	5
1 Slight	N+ve	0	0	0	0
2 Severe	N+ve	0	0	0	0
Erected Fur	N	5	5	5	5
0 Not present	N+ve	5	5	5	5
1 Present	N+ve	0	0	0	0
Pupil Response	N	5	5	5	5
0 Normal constriction	N+ve	5	5	5	5
1 Partial/no constriction	N+ve	0	0	0	0
2 Pre-existing constriction	N+ve	0	0	0	0
Salivation	N	5	5	5	5
0 None	N+ve	5	5	5	5
1 Slight	N+ve	0	0	0	0

Table 6

Summary of Neurobehavioral Evaluation: Autonomic

5550008
Week: 13 Relative to Start Date (P2)

Sex: Female		0 vg Group 1	0.36x 10E12 vg Group 2	1.1x 10E12 vg Group 3	3.3x 10E12 vg Group 4
2 Severe	N+ve	0	0	0	0
Defecation	N	5	5	5	5
-1 Absent	N+ve	5	5	5	4
0 Normal	N+ve	0	0	0	1
1 Abnormal	N+ve	0	0	0	0
Palpebral Closure/Ptosis (HC)	N	5	5	5	5
0 Eyelids wide open	N+ve	5	5	5	5
1 Eyelids droop halfway	N+ve	0	0	0	0
2 Eyelids completely shut	N+ve	0	0	0	0
Palpebral Closure/Ptosis (OF)	N	5	5	5	5
0 Eyelids wide open	N+ve	5	5	5	5
1 Eyelids droop halfway	N+ve	0	0	0	0
2 Eyelids completely shut	N+ve	0	0	0	0

Table 7

Summary of Neurobehavioral Evaluation: Excitability

5550008
Week: 4 Relative to Start Date (P1)

Sex: Male		0 vg Group 1	0.36x 10E12 vg Group 2	1.1x 10E12 vg Group 3	3.3x 10E12 vg Group 4
Vocalizations	N	5	5	5	5
0 Once/not at all	N+ve	5	5	5	5
1 More than once	N+ve	0	0	0	0
Startle Response	N	5	5	5	5
-1 No reaction	N+ve	0	0	0	0
0 Normal reaction	N+ve	5	5	5	3
1 Exaggerated Reaction	N+ve	0	0	0	2
Ease of Removal	N	5	5	5	5
-1 Very easy	N+ve	0	0	0	0
0 Easy	N+ve	4	5	5	4
1 Moderate	N+ve	1	0	0	1
2 Difficult	N+ve	0	0	0	0
Handling Reactivity	N	5	5	5	5
-1 Very low	N+ve	0	0	0	0
0 Low	N+ve	4	5	5	4
1 Moderate	N+ve	1	0	0	1
2 High	N+ve	0	0	0	0

Table 7

Summary of Neurobehavioral Evaluation: Excitability

5550008
Week: 4 Relative to Start Date (P1)

Sex: Male		0 vg Group 1	0.36x 10E12 vg Group 2	1.1x 10E12 vg Group 3	3.3x 10E12 vg Group 4
Convulsions (Home Cage)	N	5	5	5	5
0 None	N+ve	5	5	5	5
1 Clonic movement	N+ve	0	0	0	0
2 Clonic tremors	N+ve	0	0	0	0
3 Severe clonus	N+ve	0	0	0	0
4 Whole body convulsions	N+ve	0	0	0	0
Convulsions (Open Field)	N	5	5	5	5
0 None	N+ve	5	5	5	5
1 Clonic movement	N+ve	0	0	0	0
2 Clonic tremors	N+ve	0	0	0	0
3 Severe clonus	N+ve	0	0	0	0
4 Whole body convulsions	N+ve	0	0	0	0

Table 7

Summary of Neurobehavioral Evaluation: Excitability

5550008
Week: 4 Relative to Start Date (P1)

Sex: Female		0 vg Group 1	0.36x 10E12 vg Group 2	1.1x 10E12 vg Group 3	3.3x 10E12 vg Group 4
Vocalizations	N	5	5	5	5
0 Once/not at all	N+ve	5	5	5	5
1 More than once	N+ve	0	0	0	0
Startle Response	N	5	5	5	5
-1 No reaction	N+ve	0	0	0	0
0 Normal reaction	N+ve	5	5	4	5
1 Exaggerated Reaction	N+ve	0	0	1	0
Ease of Removal	N	5	5	5	5
-1 Very easy	N+ve	0	0	0	0
0 Easy	N+ve	5	5	3	5
1 Moderate	N+ve	0	0	2	0
2 Difficult	N+ve	0	0	0	0
Handling Reactivity	N	5	5	5	5
-1 Very low	N+ve	0	0	0	0
0 Low	N+ve	5	5	5	5
1 Moderate	N+ve	0	0	0	0
2 High	N+ve	0	0	0	0

Table 7

Summary of Neurobehavioral Evaluation: Excitability

5550008
Week: 4 Relative to Start Date (P1)

Sex: Female		0 vg Group 1	0.36x 10E12 vg Group 2	1.1x 10E12 vg Group 3	3.3x 10E12 vg Group 4
Convulsions (Home Cage)	N	5	5	5	5
0 None	N+ve	5	5	5	5
1 Clonic movement	N+ve	0	0	0	0
2 Clonic tremors	N+ve	0	0	0	0
3 Severe clonus	N+ve	0	0	0	0
4 Whole body convulsions	N+ve	0	0	0	0
Convulsions (Open Field)	N	5	5	5	5
0 None	N+ve	5	5	5	5
1 Clonic movement	N+ve	0	0	0	0
2 Clonic tremors	N+ve	0	0	0	0
3 Severe clonus	N+ve	0	0	0	0
4 Whole body convulsions	N+ve	0	0	0	0

Table 7

Summary of Neurobehavioral Evaluation: Excitability

5550008
Week: 13 Relative to Start Date (P2)

Sex: Male		0 vg Group 1	0.36x 10E12 vg Group 2	1.1x 10E12 vg Group 3	3.3x 10E12 vg Group 4
Vocalizations	N	5	5	5	5
0 Once/not at all	N+ve	4	5	5	5
1 More than once	N+ve	1	0	0	0
Startle Response	N	5	5	5	5
-1 No reaction	N+ve	0	0	0	0
0 Normal reaction	N+ve	5	5	5	5
1 Exaggerated Reaction	N+ve	0	0	0	0
Ease of Removal	N	5	5	5	5
-1 Very easy	N+ve	0	0	0	0
0 Easy	N+ve	5	5	5	5
1 Moderate	N+ve	0	0	0	0
2 Difficult	N+ve	0	0	0	0
Handling Reactivity	N	5	5	5	5
-1 Very low	N+ve	0	0	0	0
0 Low	N+ve	5	5	5	5
1 Moderate	N+ve	0	0	0	0
2 High	N+ve	0	0	0	0

Table 7

Summary of Neurobehavioral Evaluation: Excitability

5550008
Week: 13 Relative to Start Date (P2)

Sex: Male		0 vg Group 1	0.36x 10E12 vg Group 2	1.1x 10E12 vg Group 3	3.3x 10E12 vg Group 4
Convulsions (Home Cage)	N	5	5	5	5
0 None	N+ve	5	5	5	5
1 Clonic movement	N+ve	0	0	0	0
2 Clonic tremors	N+ve	0	0	0	0
3 Severe clonus	N+ve	0	0	0	0
4 Whole body convulsions	N+ve	0	0	0	0
Convulsions (Open Field)	N	5	5	5	5
0 None	N+ve	5	5	5	5
1 Clonic movement	N+ve	0	0	0	0
2 Clonic tremors	N+ve	0	0	0	0
3 Severe clonus	N+ve	0	0	0	0
4 Whole body convulsions	N+ve	0	0	0	0

Table 7

Summary of Neurobehavioral Evaluation: Excitability

5550008

Week: 13 Relative to Start Date (P2)

Sex: Female		0 vg Group 1	0.36x 10E12 vg Group 2	1.1x 10E12 vg Group 3	3.3x 10E12 vg Group 4
Vocalizations	N	5	5	5	5
0 Once/not at all	N+ve	4	5	5	5
1 More than once	N+ve	1	0	0	0
Startle Response	N	5	5	5	5
-1 No reaction	N+ve	0	0	0	0
0 Normal reaction	N+ve	5	5	5	5
1 Exaggerated Reaction	N+ve	0	0	0	0
Ease of Removal	N	5	5	5	5
-1 Very easy	N+ve	0	0	0	0
0 Easy	N+ve	5	5	5	5
1 Moderate	N+ve	0	0	0	0
2 Difficult	N+ve	0	0	0	0
Handling Reactivity	N	5	5	5	5
-1 Very low	N+ve	0	0	0	0
0 Low	N+ve	5	5	5	5
1 Moderate	N+ve	0	0	0	0
2 High	N+ve	0	0	0	0

Table 7

Summary of Neurobehavioral Evaluation: Excitability

5550008
Week: 13 Relative to Start Date (P2)

Sex: Female		0 vg Group 1	0.36x 10E12 vg Group 2	1.1x 10E12 vg Group 3	3.3x 10E12 vg Group 4
Convulsions (Home Cage)	N	5	5	5	5
0 None	N+ve	5	5	5	5
1 Clonic movement	N+ve	0	0	0	0
2 Clonic tremors	N+ve	0	0	0	0
3 Severe clonus	N+ve	0	0	0	0
4 Whole body convulsions	N+ve	0	0	0	0
Convulsions (Open Field)	N	5	5	5	5
0 None	N+ve	5	5	5	5
1 Clonic movement	N+ve	0	0	0	0
2 Clonic tremors	N+ve	0	0	0	0
3 Severe clonus	N+ve	0	0	0	0
4 Whole body convulsions	N+ve	0	0	0	0

Table 8

Summary of Neurobehavioral Evaluation: Neuromuscular

5550008

Week: 4 Relative to Start Date (P1)

Sex: Male		0 vg Group 1	0.36x 10E12 vg Group 2	1.1x 10E12 vg Group 3	3.3x 10E12 vg Group 4
Gait/Mobility	N	5	5	5	5
0 Normal	N+ve	5	5	5	5
1 Slightly impaired	N+ve	0	0	0	0
2 Moderately impaired	N+ve	0	0	0	0
3 Severely impaired	N+ve	0	0	0	0
Forelimb Grip Strength Mean [G]	Mean	1005.53	922.13	853.80*	993.13
	SD	105.69	100.42	61.60	50.52
	N	5	5	5	5
	%Diff	-	-8.29	-15.09	-1.23
Hindlimb Grip Strength Mean [G1]	Mean	450.40	470.80	434.27	427.00
	SD	32.31	92.78	32.96	56.23
	N	5	5	5	5
	%Diff	-	4.53	-3.58	-5.20
Air Righting Reflex	N	5	5	5	5

[G] - Anova & Dunnett: * = $p \leq 0.05$
[G1] - Kruskal-Wallis & Dunn

Table 8

Summary of Neurobehavioral Evaluation: Neuromuscular

5550008
Week: 4 Relative to Start Date (P1)

Sex: Male		0 vg Group 1	0.36x 10E12 vg Group 2	1.1x 10E12 vg Group 3	3.3x 10E12 vg Group 4
0 Normal	N+ve	5	5	5	5
1 Abnormal	N+ve	0	0	0	0
2 Severely impaired	N+ve	0	0	0	0
Tremor (Home Cage)	N	5	5	5	5
0 None	N+ve	5	5	5	5
1 Slight	N+ve	0	0	0	0
2 Moderate	N+ve	0	0	0	0
3 Severe	N+ve	0	0	0	0
Tremor (Open Field)	N	5	5	5	5
0 None	N+ve	5	5	5	5
1 Slight	N+ve	0	0	0	0
2 Moderate	N+ve	0	0	0	0
3 Severe	N+ve	0	0	0	0
Body Tone	N	5	5	5	5

Table 8

Summary of Neurobehavioral Evaluation: Neuromuscular

5550008

Week: 4 Relative to Start Date (P1)

Sex: Male		0 vg Group 1	0.36x 10E12 vg Group 2	1.1x 10E12 vg Group 3	3.3x 10E12 vg Group 4
-1 Decreased	N+ve	0	0	0	0
0 Normal	N+ve	5	5	5	5
1 Increased	N+ve	0	0	0	0

Table 8

Summary of Neurobehavioral Evaluation: Neuromuscular

5550008

Week: 4 Relative to Start Date (P1)

Sex: Female		0 vg Group 1	0.36x 10E12 vg Group 2	1.1x 10E12 vg Group 3	3.3x 10E12 vg Group 4
Gait/Mobility	N	5	5	5	5
0 Normal	N+ve	5	5	5	5
1 Slightly impaired	N+ve	0	0	0	0
2 Moderately impaired	N+ve	0	0	0	0
3 Severely impaired	N+ve	0	0	0	0
Forelimb Grip Strength Mean [G]	Mean	903.80	907.07	865.27	879.93
	SD	63.81	33.18	75.61	39.58
	N	5	5	5	5
	%Diff	-	0.36	-4.26	-2.64
Hindlimb Grip Strength Mean [G]	Mean	397.40	399.53	399.27	385.33
	SD	4.67	36.83	46.30	37.81
	N	5	5	5	5
	%Diff	-	0.54	0.47	-3.04
Air Righting Reflex	N	5	5	5	5

[G] - Anova & Dunnett

Table 8

Summary of Neurobehavioral Evaluation: Neuromuscular

5550008
Week: 4 Relative to Start Date (P1)

Sex: Female		0 vg Group 1	0.36x 10E12 vg Group 2	1.1x 10E12 vg Group 3	3.3x 10E12 vg Group 4
0 Normal	N+ve	5	5	5	5
1 Abnormal	N+ve	0	0	0	0
2 Severely impaired	N+ve	0	0	0	0
Tremor (Home Cage)	N	5	5	5	5
0 None	N+ve	5	5	5	5
1 Slight	N+ve	0	0	0	0
2 Moderate	N+ve	0	0	0	0
3 Severe	N+ve	0	0	0	0
Tremor (Open Field)	N	5	5	5	5
0 None	N+ve	5	5	5	5
1 Slight	N+ve	0	0	0	0
2 Moderate	N+ve	0	0	0	0
3 Severe	N+ve	0	0	0	0
Body Tone	N	5	5	5	5

Table 8

Summary of Neurobehavioral Evaluation: Neuromuscular

5550008

Week: 4 Relative to Start Date (P1)

Sex: Female		0 vg Group 1	0.36x 10E12 vg Group 2	1.1x 10E12 vg Group 3	3.3x 10E12 vg Group 4
-1 Decreased	N+ve	0	0	0	0
0 Normal	N+ve	5	5	5	5
1 Increased	N+ve	0	0	0	0

Table 8

Summary of Neurobehavioral Evaluation: Neuromuscular

5550008
Week: 13 Relative to Start Date (P2)

Sex: Male		0 vg Group 1	0.36x 10E12 vg Group 2	1.1x 10E12 vg Group 3	3.3x 10E12 vg Group 4
Gait/Mobility	N	5	5	5	5
0 Normal	N+ve	5	5	5	5
1 Slightly impaired	N+ve	0	0	0	0
2 Moderately impaired	N+ve	0	0	0	0
3 Severely impaired	N+ve	0	0	0	0
Forelimb Grip Strength Mean [G]	Mean	1249.60	1191.47	1164.33	1264.80
	SD	124.65	70.92	51.15	108.89
	N	5	5	5	5
	%Diff	-	-4.65	-6.82	1.22
Hindlimb Grip Strength Mean [G]	Mean	605.13	559.47	581.00	599.47
	SD	55.77	47.69	59.53	29.94
	N	5	5	5	5
	%Diff	-	-7.55	-3.99	-0.94
Air Righting Reflex	N	5	5	5	5

[G] - Anova & Dunnett

Table 8

Summary of Neurobehavioral Evaluation: Neuromuscular

5550008
Week: 13 Relative to Start Date (P2)

Sex: Male		0 vg Group 1	0.36x 10E12 vg Group 2	1.1x 10E12 vg Group 3	3.3x 10E12 vg Group 4
0 Normal	N+ve	5	5	5	5
1 Abnormal	N+ve	0	0	0	0
2 Severely impaired	N+ve	0	0	0	0
Tremor (Home Cage)	N	5	5	5	5
0 None	N+ve	5	5	5	5
1 Slight	N+ve	0	0	0	0
2 Moderate	N+ve	0	0	0	0
3 Severe	N+ve	0	0	0	0
Tremor (Open Field)	N	5	5	5	5
0 None	N+ve	5	5	5	5
1 Slight	N+ve	0	0	0	0
2 Moderate	N+ve	0	0	0	0
3 Severe	N+ve	0	0	0	0
Body Tone	N	5	5	5	5

Table 8

Summary of Neurobehavioral Evaluation: Neuromuscular

5550008
Week: 13 Relative to Start Date (P2)

Sex: Male		0 vg Group 1	0.36x 10E12 vg Group 2	1.1x 10E12 vg Group 3	3.3x 10E12 vg Group 4
-1 Decreased	N+ve	0	0	0	0
0 Normal	N+ve	5	5	5	5
1 Increased	N+ve	0	0	0	0

Table 8

Summary of Neurobehavioral Evaluation: Neuromuscular

5550008

Week: 13 Relative to Start Date (P2)

Sex: Female		0 vg Group 1	0.36x 10E12 vg Group 2	1.1x 10E12 vg Group 3	3.3x 10E12 vg Group 4
Gait/Mobility	N	5	5	5	5
0 Normal	N+ve	5	5	5	5
1 Slightly impaired	N+ve	0	0	0	0
2 Moderately impaired	N+ve	0	0	0	0
3 Severely impaired	N+ve	0	0	0	0
Forelimb Grip Strength Mean [G]	Mean	1011.07	932.67	996.33	984.13
	SD	71.55	51.23	112.29	92.94
	N	5	5	5	5
	%Diff	-	-7.75	-1.46	-2.66
Hindlimb Grip Strength Mean [G]	Mean	480.00	489.07	457.53	494.67
	SD	55.95	48.18	47.31	41.67
	N	5	5	5	5
	%Diff	-	1.89	-4.68	3.06
Air Righting Reflex	N	5	5	5	5

[G] - Anova & Dunnett

Table 8

Summary of Neurobehavioral Evaluation: Neuromuscular

5550008

Week: 13 Relative to Start Date (P2)

Sex: Female		0 vg Group 1	0.36x 10E12 vg Group 2	1.1x 10E12 vg Group 3	3.3x 10E12 vg Group 4
0 Normal	N+ve	5	5	5	5
1 Abnormal	N+ve	0	0	0	0
2 Severely impaired	N+ve	0	0	0	0
Tremor (Home Cage)	N	5	5	5	5
0 None	N+ve	5	5	5	5
1 Slight	N+ve	0	0	0	0
2 Moderate	N+ve	0	0	0	0
3 Severe	N+ve	0	0	0	0
Tremor (Open Field)	N	5	5	5	5
0 None	N+ve	5	5	5	5
1 Slight	N+ve	0	0	0	0
2 Moderate	N+ve	0	0	0	0
3 Severe	N+ve	0	0	0	0
Body Tone	N	5	5	5	5

Table 8

Summary of Neurobehavioral Evaluation: Neuromuscular

5550008

Week: 13 Relative to Start Date (P2)

Sex: Female		0 vg Group 1	0.36x 10E12 vg Group 2	1.1x 10E12 vg Group 3	3.3x 10E12 vg Group 4
-1 Decreased	N+ve	0	0	0	0
0 Normal	N+ve	5	5	5	5
1 Increased	N+ve	0	0	0	0

Table 9

Summary of Neurobehavioral Evaluation: Physiological

5550008
Week: 4 Relative to Start Date (P1)

Sex: Male		0 vg Group 1	0.36x 10E12 vg Group 2	1.1x 10E12 vg Group 3	3.3x 10E12 vg Group 4
Respiration	N	5	5	5	5
-1 Slowed	N+ve	0	0	0	0
0 Normal	N+ve	5	5	5	5
1 Rapid	N+ve	0	0	0	0
2 Abnormal	N+ve	0	0	0	0
Body Temperature (oC) [G]	Mean	37.82	37.74	38.38	38.68**
	SD	0.33	0.40	0.43	0.22
	N	5	5	5	5
	%Diff	-	-0.21	1.48	2.27

[G] - Anova & Dunnett: ** = p ≤ 0.01

Table 9

Summary of Neurobehavioral Evaluation: Physiological

5550008

Week: 4 Relative to Start Date (P1)

Sex: Female		0 vg Group 1	0.36x 10E12 vg Group 2	1.1x 10E12 vg Group 3	3.3x 10E12 vg Group 4
Respiration	N	5	5	5	5
-1 Slowed	N+ve	0	0	0	0
0 Normal	N+ve	5	5	5	5
1 Rapid	N+ve	0	0	0	0
2 Abnormal	N+ve	0	0	0	0
Body Temperature (oC) [G]	Mean	38.46	38.46	38.80	38.84
	SD	0.50	0.53	0.40	0.30
	N	5	5	5	5
	%Diff	-	0.00	0.88	0.99

[G] - Anova & Dunnett

Table 9

Summary of Neurobehavioral Evaluation: Physiological

5550008
Week: 13 Relative to Start Date (P2)

Sex: Male		0<br>vg<br><br>Group 1	0.36x<br>10E12<br>vg<br>Group 2	1.1x<br>10E12<br>vg<br>Group 3	3.3x<br>10E12<br>vg<br>Group 4
Respiration	N	5	5	5	5
-1 Slowed	N+ve	0	0	0	0
0 Normal	N+ve	5	5	5	5
1 Rapid	N+ve	0	0	0	0
2 Abnormal	N+ve	0	0	0	0
Body Temperature (oC) [G]	Mean	37.94	37.94	38.60	38.72
	SD	0.62	0.34	0.75	0.28
	N	5	5	5	5
	%Diff	-	0.00	1.74	2.06

[G] - Anova & Dunnett

Table 9

Summary of Neurobehavioral Evaluation: Physiological

5550008
Week: 13 Relative to Start Date (P2)

Sex: Female		0 vg Group 1	0.36x 10E12 vg Group 2	1.1x 10E12 vg Group 3	3.3x 10E12 vg Group 4
Respiration	N	5	5	5	5
-1 Slowed	N+ve	0	0	0	0
0 Normal	N+ve	5	5	5	5
1 Rapid	N+ve	0	0	0	0
2 Abnormal	N+ve	0	0	0	0
Body Temperature (oC) [G]	Mean	39.00	39.10	39.08	39.04
	SD	0.16	0.60	0.51	0.40
	N	5	5	5	5
	%Diff	-	0.26	0.21	0.10

[G] - Anova & Dunnett

Table 10

Summary of Neurobehavioral Evaluation: Sensorimotor

5550008
Week: 4 Relative to Start Date (P1)

Sex: Male		0 vg Group 1	0.36x 10E12 vg Group 2	1.1x 10E12 vg Group 3	3.3x 10E12 vg Group 4
Touch Resp/Tactile Reflex	N	5	5	5	5
-1 No reaction	N+ve	0	0	0	0
0 Normal reaction	N+ve	5	5	5	4
1 Exaggerated reaction	N+ve	0	0	0	1
Tail Pinch Response	N	5	5	5	5
-1 No reaction	N+ve	0	0	0	0
0 Normal Reaction	N+ve	5	5	5	5
1 Exaggerated reaction	N+ve	0	0	0	0

Table 10

Summary of Neurobehavioral Evaluation: Sensorimotor

5550008
Week: 4 Relative to Start Date (P1)

Sex: Female		0 vg Group 1	0.36x 10E12 vg Group 2	1.1x 10E12 vg Group 3	3.3x 10E12 vg Group 4
Touch Resp/Tactile Reflex	N	5	5	5	5
-1 No reaction	N+ve	0	0	0	0
0 Normal reaction	N+ve	5	5	5	5
1 Exaggerated reaction	N+ve	0	0	0	0
Tail Pinch Response	N	5	5	5	5
-1 No reaction	N+ve	0	0	0	0
0 Normal Reaction	N+ve	5	5	5	5
1 Exaggerated reaction	N+ve	0	0	0	0

Table 10

Summary of Neurobehavioral Evaluation: Sensorimotor

5550008
Week: 13 Relative to Start Date (P2)

Sex: Male		0 vg Group 1	0.36x 10E12 vg Group 2	1.1x 10E12 vg Group 3	3.3x 10E12 vg Group 4
Touch Resp/Tactile Reflex	N	5	5	5	5
-1 No reaction	N+ve	0	0	0	0
0 Normal reaction	N+ve	5	5	5	5
1 Exaggerated reaction	N+ve	0	0	0	0
Tail Pinch Response	N	5	5	5	5
-1 No reaction	N+ve	0	0	0	0
0 Normal Reaction	N+ve	5	5	5	5
1 Exaggerated reaction	N+ve	0	0	0	0

Table 10

Summary of Neurobehavioral Evaluation: Sensorimotor

5550008
Week: 13 Relative to Start Date (P2)

Sex: Female		0 vg Group 1	0.36x 10E12 vg Group 2	1.1x 10E12 vg Group 3	3.3x 10E12 vg Group 4
Touch Resp/Tactile Reflex	N	5	5	5	5
-1 No reaction	N+ve	0	0	0	0
0 Normal reaction	N+ve	5	5	5	5
1 Exaggerated reaction	N+ve	0	0	0	0
Tail Pinch Response	N	5	5	5	5
-1 No reaction	N+ve	0	0	0	0
0 Normal Reaction	N+ve	5	5	5	5
1 Exaggerated reaction	N+ve	0	0	0	0

STUDY PLAN AMENDMENT 8

Test Facility Study No. 5550008

Sponsor Reference No. UTSW.Gray-003

A Single-Dose Toxicity Study of AAV9/AP4M1 by Intrathecal Injection in Rats

GLP

SPONSOR:
University of Texas Southwestern Medical Center
5323 Harry Hines Blvd
Dallas, TX 75390
USA

TEST FACILITY:
Charles River Laboratories Montreal ULC
Senneville Site (CR-SEN)
22022 Transcanadienne
Senneville, QC H9X 3R3
Canada

Appendix 1

SUMMARY OF CHANGES AND JUSTIFICATIONS

Study Plan effective date: 09 Feb 2021

Note: When applicable, additions are indicated in bold underlined text and deletions are indicated in bold strikethrough text in the affected sections of the document.

Item or Section(s)	Justification
**Amendment 1**	**Date: 12 Feb 2021**
2. PROPOSED STUDY SCHEDULE	To include the initiation of dosing for replicate 2 and to correct the replicate numbers.
5.2. Test Item Identification	To include missing test item information, based on documentation provided by the Sponsor.<br>As no official documentation is available for freeze-thaw stability, opened vials will be stored at 4ºC, to be used on a subsequent dosing day.
9. EXPERIMENTAL DESIGN	Based on the actual concentration of the test item provided, the dose levels and concentrations were updated and/or clarified for more accuracy.
13. TERMINAL PROCEDURES	The terminal procedures tables were updated based on the tissue list in Attachment A; only a select tissue list is to be processed histologically (those to be examined microscopically and processing and evaluation of gross lesions were added.
13.8. Splenocyte Sample Collection Analysis	The spleen will be collected using clean technique; this detail was added.
13.9. Tissue Collection for Biodistribution and Gene Expression Determination	In order to avoid tissue cross-contamination, the order of tissue collection was revised.
ATTACHMENT A	Tissue list updated to clarify the sections for certain tissues or removed the collection for remaining frozen based on the small tissue size.
**Amendment 2**	**Date: 22 Feb 2021**
4. RESPONSIBLE PERSONNEL	To assign the IS for the spleenocyte preparation; the IS will not provide a formal report.
13.8. Splenocyte Sample Collection Analysis	To clarify and to be aligned with CR-SEN standard processes.
**Amendment 3**	**Date: 25 Feb 2021**
6.1. Preparation of Formulations, ATTACHMENT B	Any remaining formulation will be returned to the sponsor.
13.9. Tissue Collection for Biodistribution and Gene Expression Determination	To include the storage of remaining tissue samples.
18.2. Test Site(s)/Subcontractor(s) - *SECTION REMOVED*	Section was removed as the phases being performed by Test Site(s)/Subcontractor will not be done as per GLPs.
ATTACHMENT A	To remove a redundant footnote for the DRG.<br>The side of tissue collection was added for clarification for the liver and testis.<br>To remove the cervical and lumbar regions of the spinal cord for archival; due to the bioanalytical, gene expression and histopathology sections, and due to the small size of the tissues, there will not be remaining for archive.<br>Due to the small size of the tissues and to ensure there is sufficient tissues for microscopical evaluation, the T4 segment of the thoracic spinal cord

Appendix 1

Item or Section(s)	Justification
	and DRG will not be archived and will be submitted for histopathological evaluation instead.<br>Similarly, due to the size of the spinal cord and DRG, transverse and oblique sections will be collected if there is sufficient tissue; transverse section will be prioritized. Footnote "e" was updated accordingly.
Attachment B	To include the shipment of the remaining dose formulation samples.
**Amendment 4**	**Date: 08 Mar 2021**
Summary of changes and Justifications	To include the change for Attachment B inadvertently omitted in Amendment 3.
10.1. Neurobehavioral Evaluation – *NEW SECTION*	In order to captured any potential neurological effects, neurobehavioral evaluation will be added.
12.3. Bioanalytical Sample Analysis	To include the bioanalytical details for the serum samples.
13.5. Bioanalytical Cerebrospinal Fluid (CSF) Collection	CSF samples will be kept for potential future analysis; details added.
15.3. Inferential Statistical Methods	To include the statistical analysis of the neurobehavioral evaluation data.
16. COMPUTERIZED SYSTEMS	To include the computerized system for the neurobehavioral evaluation data.
ATTACHMENT A	To include the microscopic evaluation of additional tissues where tissue weights are being collected; in order to allow for correlation of potential microscopic findings if any organ effects are noted.
**Amendment 5**	**Date: 31 Mar 2021**
2. PROPOSED STUDY SCHEDULE	The Draft Report will be fully audited; section updated accordingly.<br>To include a Final Report and experimental completion date.
4. RESPONSIBLE PERSONNEL	To assign the pathologist and specify the site for QA responsibilities of this phase.<br>To clarify the reporting requirements for the IS for the immunotoxicology splenocyte preparation phase of the study.
6.1. Preparation of Formulations	To include the disposition of the remaining dose formulations.
10.1. Neurobehavioral Evaluation	As the neurobehavioral evaluation was included on the study after the animals arrived and due to **limitations** ~~imitations~~ of Provantis, the technicians performing the evaluation will not be blinded to the treatment group.
13.10. Biodistribution/Gene Expression Analysis	To include that tissues selected for analysis will be added by amendment.
**Amendment 6**	**Date: 07 Jul 2021**
Summary of changes and Justifications	To correct a typographical error in the Summary of changes and Justifications for the change made in Section 10.1 in Amendment 5.<br>In addition, there was no change to Attachment A in Amendment 5; this justification as remove from the Summary of changes and Justifications.
3. SPONSOR	To include the sponsor study monitor.
4. RESPONSIBLE PERSONNEL	The parameters to be analyzed for bioanalysis was updated as CSF will be kept for possible future analysis.
5.2. Test Item Identification	To include the retest date of the test item.
13.10. Biodistribution/Gene Expression Analysis	To include the selected tissues for biodistribution/gene expression analysis.
16. COMPUTERIZED SYSTEMS	To include a computerized system now being used for signatures of study plan, amendments, and reports.
17. REGULATORY COMPLIANCE	As the pathology phase of the study is being performed in the US, the regulations to be followed was added.

Appendix 1

Item or Section(s)	Justification
**Amendment 7**	**Date: 10 Sep 2021**
Summary of changes and Justifications	To include the justification for the change made in Section 5.2 in Amendment 6.
2. PROPOSED STUDY SCHEDULE	Based on type of study, SEND data was not deemed required; any reference to SEND removed.
14.3. Pathology Peer Review – *NEW SECTION*	At the request of the Sponsor in consideration of the microscopic findings noted (specifically in the DRG), a pathology peer review was added.
22.1. SEND Datasets – *SECTION REMOVED*	Based on type of study, SEND data was not deemed required; any reference to SEND removed.
ATTACHMENT B	To include the shipping details for the slides for pathology peer review.
**Amendment 8**	**Date: 04 Mar 2022**
4. RESPONSIBLE PERSONNEL	Phases performed by the Sponsor were not conducted according to GLP regulations; any reference to QA for these phases was removed.

TABLE OF CONTENTS

SUMMARY OF CHANGES AND JUSTIFICATIONS 2
1. OBJECTIVE 7
2. PROPOSED STUDY SCHEDULE 7
3. SPONSOR 7
4. RESPONSIBLE PERSONNEL 8
5. TEST MATERIALS 10
6. DOSE FORMULATION AND ANALYSIS 11
7. TEST SYSTEM 11
8. HUSBANDRY 12
9. EXPERIMENTAL DESIGN 14
10. IN-LIFE PROCEDURES, OBSERVATIONS, AND MEASUREMENTS 15
11. CLINICAL PATHOLOGY 16
12. BIOANALYSIS 18
13. TERMINAL PROCEDURES 18
14. HISTOLOGY AND MICROSCOPIC EVALUATION 22
15. STATISTICAL ANALYSIS 23
16. COMPUTERIZED SYSTEMS 25
17. REGULATORY COMPLIANCE 26
18. QUALITY ASSURANCE 26
19. AMENDMENTS AND DEVIATIONS 26
20. RETENTION AND DISPOSITION OF RECORDS, SAMPLES, AND SPECIMENS 27
21. STUDY CLASSIFICATION 27
22. REPORTING 28
23. JUSTIFICATIONS AND GUIDELINES 28
24. ANIMAL WELFARE 29
25. REFERENCES 29
AMENDMENT APPROVAL 30
SPONSOR APPROVAL 31
ATTACHMENT A 32

ATTACHMENT B 35

Appendix 1

1. OBJECTIVE

The objective of this study is to characterize the toxicity, biodistribution, and gene expression of AAV9/AP4M1, for the treatment of Spastic Paraplegia Type 50 (SPG50) caused by the AP4M1 gene mutation, when given by a single intrathecal injection to rats and to evaluate the potential reversibility and/or progression of any findings.

2. PROPOSED STUDY SCHEDULE

Proposed study dates are listed below. Actual dates will be included in the Final Report.

Experimental Starting Date:	09 Feb 2021<br>(First date of study-specific data collection)
Experimental Completion Date:	28 Jan 2022<br>(Last date on which data are collected)
Animal Arrival:	09 Feb 2021
Initiation of Dosing:	16 Feb 2021 (Replicate 1)<br>17 Feb 2021 (Replicate 2)<br>19 Feb 2021 (Replicate 3)<br>22 Feb 2021 (Replicate 4)
Completion of In-life:	01 Mar 2021 (Day 8)<br>22 Mar 2021 (Day 29)<br>23 May 2021 (Day 91)<br>(Last date of necropsy)
Audited Draft Report:	30 Jul 2021<br>(9 weeks following completion of in-life)
Final Report:	28 Jan 2022<br>(Expected date of Study Director signature of report. Target to be within 6 months of issue of Draft Report)

3. SPONSOR

Role/Phase	Name	Contact Information
Sponsor Representative	Steven Gray, PhD	University of Texas Southwestern Medical Center<br>5323 Harry Hines Blvd<br>University of Texas Southwestern Medical Center<br>Dallas, TX 75390<br>Tel: 214.648.0670<br>E-mail:<br>steven.gray@UTsouthwestern.edu

Role/Phase	Name	Contact Information
Sponsor Study Monitor	Roxana Ploski	University of Texas Southwestern Medical Center<br>5323 Harry Hines Blvd<br>University of Texas Southwestern Medical Center<br>Dallas, TX 75390<br>Tel: 214.648.9828<br>E-mail: roxana.ploski@UTsouthwestern.edu
Alternate Study Contact	Terry Pirovol	CureSPG50<br>6 Topham Road<br>Toronto, ON M4B 3K2<br>Canada<br>Tel:<br>E-mail: tpirovol@hotmail.com

4. RESPONSIBLE PERSONNEL

Role/Phase	Quality Assurance Program (QAP)	Name	Contact Information
Study Director	Charles River	Stefania Cinquino, BSc	Address as cited for Test Facility<br>Tel: 514.630.8200, ext 2151<br>E-mail: stefania.cinquino@crl.com
Test Facility Management	Charles River	Julie Douville, PhD	Address as cited for Test Facility<br>Tel: 514.630.8200, ext 8309<br>E-mail: julie.douville@crl.com
Test Facility QAP	Charles River	Nooshin Davani, BSc, RQAP-GLP	Address as cited for Test Facility<br>Tel: 514.630.8200, ext 2605<br>E-mail: nooshin.davani@crl.com
**Individual Scientist (IS)**			
Clinical Pathology	Charles River	Virginie Allegret, DVM, IPSAV, DES, DACVP	Address as cited for Test Facility<br>Tel: (514) 630-8200, ext 2028<br>E-mail: virginie.allegret@crl.com
Immuno-toxicology Splenocyte Preparation	Charles River	Philippe Rousseau, PhD	Address as cited for Test Facility<br>Tel: (514) 630-8200, ext 2154<br>E-mail: Philippe.Rousseau@crl.com
**Principal Investigator (PI)**			
Serum, and Tissue Biodistribution Analysis (Bioanalysis) [a]	**Not applicable**<br>~~**Test Site**~~	Steven Gray, PhD	University of Texas Southwestern Medical Center<br>5323 Harry Hines Blvd<br>University of Texas Southwestern Medical Center<br>Dallas, TX 75390<br>Tel: 214-648-0670<br>E-mail: steven.gray@UTsouthwestern.edu

Role/Phase	Quality Assurance Program (QAP)	Name	Contact Information
Tissue Gene expression Analysis [a]	**Not applicable** ~~Test Site~~	Steven Gray, PhD	University of Texas Southwestern Medical Center<br>5323 Harry Hines Blvd<br>University of Texas Southwestern Medical Center<br>Dallas, TX 75390<br>Tel: 214-648-0670<br>E-mail:<br>steven.gray@UTsouthwestern.edu
Pathology [b]	Charles River Frederick, MD	Maureen T. O'Brien, DVM, MS, DACVP	Charles River Laboratories, Inc.<br>15 Worman's Mill Ct., Suite I<br>Frederick, MD 21701<br>Tel: 301.360.1710<br>Email: Maureen.O'Brien@crl.com

[a] Sponsor.
[b] Test Facility-designated Test Site.

Each IS and PI is required to report all deviations or other circumstances that could affect the quality or integrity of the study to the Study Director in a timely manner for authorization/acknowledgement. Each IS and PI, with the exception of those listed below, will provide a report addressing their assigned phase of the study, which will be included as an appendix to the Final Report.

The IS phase report will include the following:

- A listing of critical computerized systems used in the conduct and/or interpretation of the assigned study phase

The IS for the Immunotoxicology Splenocyte Preparation phase will not provide a formal report.

The PI phase report will include the following:

- A Statement of Compliance (if the applicable phase is GLP)
- A QA Statement (for Sponsor-designated PI or for Test Facility-designated PI if audited by a QAP other than that of the Test Facility and if the applicable phase is GLP)
- The archive site for all records, samples, specimens and reports generated from the phase or segment (alternatively, details regarding the retention of the materials may be provided to the Study Director for inclusion in the Final Report)
- A listing of critical computerized systems used in the conduct and/or interpretation of the assigned study phase

5. TEST MATERIALS

5.1. Test and Reference Item Characterization

The Sponsor will provide to the Test Facility documentation of the identity, strength, purity, composition, and stability for the test and reference items. A Certificate of Analysis or equivalent documentation will be provided for inclusion in the Final Report.

The Sponsor has appropriate documentation on file concerning the method of synthesis, fabrication or derivation of the test and reference items, and this information is available to the appropriate regulatory agencies should it be requested.

5.2. Test Item Identification

Test Item Identification

	**Test Item**
**Identification:**	AAV9/AP4M1
**Alternate Identification:**	rAAV9.AP4M1
**Batch/Lot No.:**	T-GEMINIS-033
**Expiration/Retest Date:**	29 Apr 2021
**Physical Description:**	Colorless, clear to slightly opalescent, free of visible particles
**Concentration:** **(based on ddPCR results)**	5.43E13 vg/mL
**Storage Conditions** **(temperature set to maintain):**	-80ºC (upon thawing of a vial, it may be stored at 4ºC and used on a subsequent dosing day)
**Provided by:**	Sponsor

5.3. Reference Item/Vehicle Identification

Reference Item/Vehicle Identification

	**Reference Item**
**Identification:**	PBS containing 5% D-sorbitol and 0.001% pluronic F-68
**Storage Conditions** **(temperature set to maintain):**	-80°C
**Provided by:**	Sponsor

5.4. Reserve Samples

Due to the study duration, a reserve sample will not be collected.

5.5. Test and Reference Item Inventory and Disposition

Records of the receipt, distribution, storage, and disposition of test materials will be maintained. All unused Sponsor-supplied bulk test materials will be returned to the Sponsor following issuance of the Draft Report unless otherwise requested (documentation will be retained in the study record). An earlier shipment of these materials may also be requested and authorized by

the Study Director and Sponsor. See Shipment of Samples and Study Records (Attachment B) for shipping details.

5.6. Safety

The safety precautions for the test item and dose formulations will be documented in a Test Material Safety Data Sheet (TMSDS) based on the SDS or similar document.

6. DOSE FORMULATION AND ANALYSIS

6.1. Preparation of Formulations

Dose formulations will be divided into aliquots, where required, and dispensed on each dosing occasion.

Preparation Details

Dose Formulation	Frequency of Preparation	Storage Conditions (temperature set to maintain)
Reference Item	Used as received	4°C
Test Item	At least once	4°C

Any residual volumes from each dosing occasion will be shipped back to the Sponsor; see Attachment B. Residual volumes will be archived by the Sponsor and will be kept for potential future analysis. Analysis, if conducted, will be added by amendment.

6.2. Preparation Details

Dosing formulations will be prepared under a laminar flow hood using clean procedures.

Dosing formulations will be prepared based on Sponsor's instructions at appropriate concentrations to meet dose level requirements.

6.3. Sample Collection and Analysis

Samples for dose formulation analysis will not be collected by the Test Facility.

7. TEST SYSTEM

Species:	Rat
Strain:	Crl:CD(SD) Sprague Dawley rat
Condition:	Purpose-bred, naive
Source:	Charles River Labs International Inc., Raleigh, NC, USA
Number of Males to be Assigned:	60 (plus 6 alternates)

Number of Females to be Assigned: 60 (plus 6 alternates)

Target Age at the Initiation of Dosing: 7 to 8 weeks

Target Weight at the Initiation of Dosing: 170 to 225 g (males)/140 to 200 g (females)

The actual age and weight of the animals at the initiation of dosing will be listed in the Final Report.

7.1. Animal Identification

Method: Subcutaneously implanted electronic identification chip

7.2. Environmental Acclimation

Method: At least 5 days will be allowed between animal receipt and the start of dosing in order to accustom the animals to the laboratory environment.

7.3. Selection, Assignment, Replacement, and Disposition of Animals

Selection: Animals will be randomly assigned to groups. Males and females will be randomized separately. Animals in poor health will not be assigned to groups. Body weights will be monitored and any replacements will be undertaken prior to initiation of dosing to allow for comparable group means between groups/sex.

Replacement: Before the initiation of dosing, any assigned animals considered unsuitable for use in the study will be replaced by alternate animals obtained from the same shipment and maintained under the same environmental conditions. After initiation of dosing, study animals may be replaced during the replacement period with alternate animals in the event of accidental injury, non-test item-related health issues, or similar circumstances. The alternate animals may be used as replacements on the study within 2 days. General in-life assessments will include alternate animals until released from study.

Disposition: The disposition of all animals will be documented in the study records.

8. HUSBANDRY

8.1. Housing

Housing: Group-housed (up to 3 animals of the same sex and same dosing group together).

Caging:	Polycarbonate cages containing appropriate bedding. Where possible, control group animals will be housed on a separate rack from the test item-treated animals.
Cage Identification:	Color-coded cage card indicating study, group, animal number(s), and sex.

Housing set-up is as described in the *Guide for the Care and Use of Laboratory Animals* (National Research Council, 2011) Animals will be separated during designated procedures/activities or will be separated as required for monitoring and/or health purposes, as deemed appropriate by Study Director and/or Clinical Veterinarian. Cages will be arranged on the racks in group order.

8.2. Animal Enrichment

Psychological/ Environmental Enrichment:	Animals will be socially housed and will be provided with items such as a hiding device, and a chewing object, except during study procedures/activities.

8.3. Environmental Conditions

The targeted conditions for animal room environment will be as follows:

Temperature:	22 ±3°C
Humidity:	30% to 70%
Light Cycle:	12 hours light and 12 hours dark (except during designated procedures)

8.4. Food

Diet:	Lab Diet Certified CR Rodent Diet 5CR4
Type:	Pellets (same diet in meal form may be provided to individual animals as warranted by clinical signs, e.g., broken/damaged incisors or other health changes)
Frequency:	Ad libitum, except during designated procedures
Analysis:	Results of analysis for nutritional components and environmental contaminants are provided by the supplier and are kept on file at the Test Facility. It is considered that there are no known contaminants in the feed that would interfere with the objectives of the study.

8.5. Water

Type:	Municipal tap water, treated by reverse osmosis and ultraviolet irradiation.

Frequency/Ration: Freely available to each animal via an automatic watering system (except during designated procedures). Water bottles can be provided, if required.

Analysis: Periodic analysis of the water is performed, and results of these analyses are kept on file at the Test Facility. It is considered that there are no known contaminants in the water that would interfere with the outcome of the study.

8.6. Veterinary Care

Veterinary care will be available throughout the course of the study and animals will be examined by the veterinary staff as warranted by clinical signs or other changes. In the event that animals show signs of illness or distress, the responsible veterinarian may make initial recommendations about treatment of the animal(s) and/or alteration of study procedures, which must be approved by the Study Director or Scientific designate. Treatment of the animal(s) for minor injuries or ailments may be approved without prior consultation with the Sponsor representative when such treatment does not impact fulfillment of the study objectives. If the condition of the animal(s) warrants significant therapeutic intervention or alterations in study procedures, the Sponsor representative will be contacted, when possible, to discuss appropriate action. If the condition of the animal(s) is such that emergency measures must be taken, the Study Director (or Scientific designate) and/or veterinarian will attempt to consult with the Sponsor representative prior to responding to the medical crisis, but the Study Director (or Scientific designate) and/or veterinarian has authority to act immediately at his/her discretion to alleviate suffering. The Sponsor representative will be fully informed of any such events.

9. EXPERIMENTAL DESIGN

Experimental Design

Group No.	Test Material	Dose Level (vg)	Dose Volume (µL)	Dose Concentration (vg/µL)	No. of Animals					
					Main Study		Recovery Study			
					Day 8 Necropsy[a]		Day 29 Necropsy[b]		Day 91 Necropsy[c]	
					M	F	M	F	M	F
1	Reference Item	0	60	0	5	5	5	5	5	5
2	AAV9/AP4M1	0.36×10^{12}	20	0.18×10^{11}	5	5	5	5	5	5
3	AAV9/AP4M1	1.1×10^{12}	20	0.55×10^{11}	5	5	5	5	5	5
4	AAV9/AP4M1	3.3×10^{12}	60	0.55×10^{11}	5	5	5	5	5	5

M = Males; F = Females
[a] Animals scheduled for Necropsy on Day 8.
[b] Animals scheduled for Necropsy on Day 29.
[c] Animals scheduled for Necropsy on Day 91.

9.1. Administration of Test and Reference Items

Dose Route: Intrathecal bolus injection at the lumbar level via direct puncture

Frequency: Once

Duration: Single Dose

Method: The day of dosing will be designated as Day 1. The animals will be anesthetized with isoflurane, administered eye lubricant, shaved and prepared for surgery according to the appropriate SOP. The test and reference items will be administered by intrathecal bolus injection through direct puncture as per SOP.

10. IN-LIFE PROCEDURES, OBSERVATIONS, AND MEASUREMENTS

General In-life Assessments - Main and Recovery Study Animals

Parameter	Frequency	Comments
**Mortality/ Moribundity Checks**[a]	Twice daily (morning and afternoon) starting upon arrival through termination.	Animals will be observed within their cage unless removal is necessary for identification or confirmation of possible findings.
**Postdose Observations**[a]	2 hours (± 30 minutes) postdose after the end of each group for each sex	Cage side observations unless removal is necessary for identification or confirmation of possible findings.
**Detailed Clinical Observations**[a]	Weekly starting Day -1, and at least every 2 weeks during the prestudy period.	Animals are removed from the cage.
**Individual Body Weights**[a]	Weekly starting Day -1, and at least every 2 weeks during the prestudy period.	Fasted weight on the day of necropsy. No terminal body weights collected from animals found dead or preterminally euthanized.
**Food Consumption**[a]	Weekly starting Day -1 and throughout the study	Quantitatively measured

[a] Minimum required frequency for this parameter indicated

10.1. Neurobehavioral Evaluation

Frequency: During Weeks 4 and 13 from animals designated for necropsy on Day 91 (5/sex/group). Animals will be observed in their home cage except for parameters requiring removal for assessment.

Procedure: The technicians performing the evaluation will not be blinded to the treatment group.

The following assessments will be performed according to SOP HRM-096:

Activity:<br>- Arousal/Alertness<br>- Rearing<br>- Stereotypy Posture/Body Carriage<br>- Appearance	Excitability:<br>- Vocalizations<br>- Handling Reactivity<br>- Ease of Removal<br>- Startle Response<br>- Convulsions	Autonomic:<br>- Exophthalmus<br>- Palpebral Closure/Ptosis<br>- Pupil Response Lacrimation<br>- Defecation<br>- Erected Fur<br>- Salivation
Neuromuscular:<br>- Gait/Mobility	Sensorimotor:<br>- Pain Response (tail pinch)	Physiological:<br>- Respiration

- Body Tone<br>- Air Righting Reflex<br>- Grip Strength<br>- Tremor	- Touch Response/Tactile Reflex	- Body Temperature

11. CLINICAL PATHOLOGY

11.1. Sample Collection

Clinical Pathology Sample Collection – Main and Recovery Study Animals

Group Nos.	Occasion/ Time Point	Hematology	Coagulation	Clinical Chemistry	Urinalysis
1 to 4	Day 8	X	X	X	X
1 to 4	Day 29	X	X	X	X
1 to 4	Day 91	X	X	X	X
Unscheduled euthanasia (when possible)		X	X	X	-
**Overnight Fasting:**		-	-	Yes	Yes
**Method/Comments:**		Abdominal aorta at termination[a]			Individual housed in metabolism cages; overnight collection
**Volume (mL):**		Up to 1 mL	1.2	0.7	As available
**Anticoagulant:**		EDTA	Sodium citrate	None, in SST	-
**Special Requirements:**		-	-	-	-
**Processing:**		None	Plasma	Serum	-

X = Sample to be collected; - = Not applicable; SST = serum separator tube.
[a] Immediately prior to necropsy while the animals are under isoflurane anesthesia.

11.2. Hematology

Hematology Parameters

Red blood cell count<br>Hemoglobin concentration<br>Hematocrit<br>Mean corpuscular volume<br>Red blood cell distribution width<br>Mean corpuscular hemoglobin concentration<br>Mean corpuscular hemoglobin<br>Reticulocyte count (absolute)	Platelet count<br>White blood cell count<br>Neutrophil count (absolute)<br>Lymphocyte count (absolute)<br>Monocyte count (absolute)<br>Eosinophil count (absolute)<br>Basophil count (absolute)<br>Large unstained cells (absolute)

A blood smear will be prepared from each hematology sample and may be examined for confirmation of hematology results.

11.3. Coagulation

Coagulation Parameters

Activated partial thromboplastin time<br>Fibrinogen	Prothrombin time<br>Sample quality

11.4. Clinical Chemistry

Clinical Chemistry Parameters

Alanine aminotransferase	Total protein
Aspartate aminotransferase	Albumin
Alkaline phosphatase	Globulin
Gamma-glutamyltransferase	Albumin/globulin ratio
Creatine kinase	Glucose
Total bilirubin[a]	Cholesterol
Urea nitrogen	Triglycerides
Creatinine	Sodium
Calcium	Potassium
Phosphorus	Chloride
	Sample quality

[a] When total bilirubin is > 0.5 mg/dL, direct bilirubin will also be measured and indirect bilirubin will be calculated.

11.5. Urinalysis

Urinalysis Parameters

Color	Protein[a]
Appearance/Clarity	Glucose[a]
Specific gravity	Bilirubin
Volume	Ketones
pH	Blood

[a] Semi-quantitative measurement

11.6. Bone Marrow Smear Evaluation

Bone marrow smears will be collected and prepared as described in Attachment A and may be examined for confirmation of results.

12. BIOANALYSIS

12.1. Bioanalytical Sample Collection

In addition to those listed below, a sample will be collected from animals pre-terminally euthanized, when possible.

Bioanalytical Sample Collection

Group Nos.	Time Points
	At termination
1 to 4	X
**Method/Comments:**	Abdominal aorta at termination[a]
**Target Volume[a] (mL):**	1
**Anticoagulant:**	None, in SST
**Special Requirements:**	None
**Processing:**	Serum

X = Sample to be collected; - = Not applicable; SST = serum separator tube
[a] Immediately prior to necropsy while the animals are under isoflurane anesthesia.

12.2. Bioanalytical Sample Processing

The samples will be centrifuged and the resultant serum will be separated, transferred to 2 uniquely labeled polypropylene tubes of approximately equal volume, frozen immediately over dry ice and transferred in a freezer set to maintain -80°C.

The samples will be shipped to the University of Texas Southwestern Medical Center, see Attachment B. The bioanalytical laboratory will be notified before shipment of the samples. Samples will be stored at the bioanalytical laboratory in a freezer set to maintain -60ºC or below until analysis.

12.3. Bioanalytical Sample Analysis

Serum samples will be analyzed for anti-AAV9 antibodies using a cell-based neutralizing antibody assay.

13. TERMINAL PROCEDURES

Terminal procedures are summarized in the following tables:

Replaced, Found Dead, and Unscheduled Euthanasia Animals

Animals	Necropsy Procedures				Histology Processing	Microscopic Evaluation
	CSF Collection[d]	Necropsy	Tissue Collection	Organ Weights		
Animals replaced prestudy	-	X	Standard Diagnostic List	-	-	-
Animals replaced after dosing start	-	X	Full List[a]	-	-	-

Appendix 1

Animals	Necropsy Procedures				Histology Processing	Microscopic Evaluation
	CSF Collection[d]	Necropsy	Tissue Collection	Organ Weights		
Unscheduled deaths after dosing start	X (unscheduled euthanasia only)	X	Full List[a]	-	Select Tissues[a] Gross Lesions	Select Tissues[a] Gross Lesions

Main Study Animals

Group No.	Scheduled Euthanasia Day	Necropsy Procedures				Histology Processing	Microscopic Evaluation
		CSF Collection[b]	Necropsy	Tissue Collection	Organ Weights		
1	8	X	X	Full List[a]	Full List[a]	Select Tissues[a] Gross Lesions	Select Tissues[a] Gross Lesions
2						Select Tissues[a] Gross Lesions	Select Tissues[a] Gross Lesions
3						Select Tissues[a] Gross Lesions	Select Tissues[a] Gross Lesions
4						Select Tissues[a] Gross Lesions	Select Tissues[a] Gross Lesions

Recovery Study Animals

Group No.	Scheduled Euthanasia Day	Necropsy Procedures				Histology Processing	Microscopic Evaluation
		CSF Collection[b]	Necropsy	Tissue Collection	Organ Weights		
1	29	X	X	Full List[a]	Full List[a]	Select Tissues[a] Gross Lesions	Select Tissues[a] Gross Lesions
2						Select Tissues[a] Gross Lesions	Select Tissues[a] Gross Lesions
3						Select Tissues[a] Gross Lesions	Select Tissues[a] Gross Lesions
4						Select Tissues[a] Gross Lesions	Select Tissues[a] Gross Lesions

Recovery Study Animals

Group No.	Scheduled Euthanasia Day	Necropsy Procedures				Histology Processing	Microscopic Evaluation
		CSF Collection[b]	Necropsy	Tissue Collection	Organ Weights		
1	91	X	X	Full List[a]	Full List[a]	Select Tissues[a]	Select Tissues[a]
2						Select Tissues[a]	Select Tissues[a]
3						Select Tissues[a]	Select Tissues[a]
4						Select Tissues[a]	Select Tissues[a]

X = Procedure to be conducted; - = Not applicable.

[a] See Tissue Weighing, Collection, Processing and Evaluation table in Attachment A for list of tissues applicable to each procedure.

[b] As much CSF as possible will be collected from the cisterna magna at necropsy.

13.1. Method of Euthanasia

The animals will undergo exsanguination by incision from the abdominal aorta following isoflurane anesthesia, unless deemed inappropriate by the Study Director and/or the clinical veterinarian.

13.2. Unscheduled Euthanasia

Main and Recovery Study animals to be euthanized for humane reasons before the scheduled time will undergo sample collection for evaluation of clinical pathology parameters and bioanalysis, if possible as specified in Section 11.1 and Section 12.1.

Tissues from animal replaced after the start of dosing will be retained (as per Tissue Collection and Preservation section) and any data generated will not be included in the report unless deemed appropriate by the Study Director.

13.3. Scheduled Euthanasia

Main and Recovery Study animals surviving until scheduled euthanasia will be food deprived overnight. Animal will undergo blood sample collection for evaluation of clinical pathology parameters and bioanalysis. When possible, the animals will be euthanized rotating across dose groups such that similar numbers of animals from each group, including controls, will be necropsied throughout the day.

13.4. Necropsy

Animals as detailed in the Terminal Procedures section will be subjected to a complete necropsy examination, which will include evaluation of the carcass and musculoskeletal system; all external surfaces and orifices; cranial cavity and external surfaces of the brain; and thoracic, abdominal, and pelvic cavities with their associated organs and tissues. Necropsy examinations will be performed by qualified personnel with appropriate training and experience in animal anatomy and gross pathology. A veterinary pathologist, or other suitably qualified person, will be available for consultation. Images may be generated for illustration of or consultation on gross observations. These images will not be used for data generation or interpretation, and will not be archived or included in the final report.

13.5. Bioanalytical Cerebrospinal Fluid (CSF) Collection

At scheduled termination and for preterminally euthanized animals, samples of CSF will be collected from all animals while under isoflurane anesthesia. CSF samples (as much as possible) will be collected under via the cisterna magna, and transferred into uniquely labeled clear polypropylene tubes. The CSF samples will be placed on dry ice and then transferred in a freezer set to maintain -80°C. CSF samples will then be shipped to the University of Texas Southwestern Medical Center, see Attachment B.

CSF samples will be kept for possible future analysis. Analysis, if performed, will be added by amendment.

13.6. Organ Weights

The organs detailed in the Terminal Procedures section and Tissue Weighing, Collection, Processing and Evaluation (Attachment A) table will be weighed at necropsy. Paired organs will be weighed together. In the event of gross abnormalities, in addition to the combined weight, the weight of each organ of a pair may be taken and entered as a tissue comment. Organ weight as a percent of body weight (using the terminal body weight) and organ weight as a percent of brain weight will be calculated.

13.7. Tissue Collection and Preservation

Representative samples of tissues will be collected and preserved in 10% neutral buffered formalin, except for tissues requiring alternate fixatives as defined by standard operating procedures, as detailed in the Terminal Procedures section and Tissue Weighing, Collection, Processing and Evaluation (Attachment A) table. Additional tissue samples may be collected to elucidate abnormal findings.

13.8. Splenocyte Sample Collection Analysis

The spleen will be collected using cell culture clean procedures from all animals at scheduled necropsy. Dissected spleens, approximately half, will be placed into prechilled tubes containing RPMI media and stored at 2°C to 8°C or on wet ice before processing to splenocytes. Splenocytes will then be cryopreserved/frozen until analysis.

Samples will be processed at ambient temperature according to standard CR-SEN SOP and analytical procedure AP.5550008.SPL.xx (where xx denote the version of the procedure), except that all washes will be performed with RPMI-1640 Media and samples will be frozen as follows:

Prepare a sufficient number of 2 mL cryovials to hold the cells at 2e7 cells/mL. Open the vials and add 100 µL of Hybridoma-Grade DMSO (Freezing media is 90% heat-inactivated FBS and 10 % DMSO). Add sufficient heat-inactivated FBS to the cell pellet to put the cells at 2e7/mL when added to the DMSO. Gently resuspend the pellet via pipetting and then add to the tubes containing DMSO. The act of addition and higher density of FBS will mix the FBS and DMSO yielding an evenly distributed freezing media. 1-2 gentle inversions can be used to assure even mixing. If adequate splenocytes are available, 3 aliquots of cells at 2e7/mL will be prepared for each animal. Splenocytes will be stored in the vapor phase of liquid nitrogen until shipped to the Sponsor on dry ice. See Attachment B for shipping details.

The splenocytes will be analyzed for T-cell responses against AAV9 and AP4M1. All analytical work will be conducted by the Sponsor, using an analytical method developed and qualified by that laboratory.

13.9. Tissue Collection for Biodistribution and Gene Expression Determination

At scheduled termination and for preterminally euthanized animals, tissues in the Tissue Weighing, Collection, Processing and Evaluation (Attachment A) table from animals identified in the Terminal Procedures section will be collected for biodistribution and gene expression.

Samples (between 40 and 50 mg, when possible) will be collected in duplicate, snap-frozen in liquid nitrogen and stored on dry ice until transferred to a freezer set to maintain -80°C until shipped to the University of Texas Southwestern Medical Center for analysis, see Attachment B.

Samples will collected using clean removal techniques.

When possible, the order of tissue collection will be as follows: spleen, liver, kidney, brain then remaining tissues. The brain will be hemisected at necropsy, and samples for biodistribution and gene expression determination will be collected from the right hemisphere, while the left hemisphere will be fixed in formalin for histopathological evaluation. For animals found dead, the whole brain will be fixed in formalin.

Tissues for histopathology will be collected prior to sample collection for any other parameters.

Following sample collection of all tissues designated for biodistribution analysis, remaining tissues will be snap-frozen in liquid nitrogen, placed on dry ice until being stored in a freezer set to maintain ≤ -60ºC) for possible future analysis.

13.10. Biodistribution/Gene Expression Analysis

Sponsor-selected tissue samples (forebrain, hindbrain, lumbar spinal cord, lumbar DRG, sciatic nerve, heart, liver, thymus, spleen, kidney, and testes/ovaries) will be analyzed for concentration of vector DNA (as per Section 12.3.) and gene expression by qPCR or ddPCR.

All analytical work will be conducted by the Sponsor, using an analytical method developed and qualified by that laboratory that conforms to FDA guidelines but not in adherence with GLP. The work performed in conjunction with this study will not be conducted in compliance with GLPs and will not subject to review by the Quality Assurance Unit (QAU) of that laboratory. A Final Report will be prepared and submitted to Testing Facility for inclusion as an appendix in the main study Final Report.

14. HISTOLOGY AND MICROSCOPIC EVALUATION

14.1. Histology

Tissues in Terminal Procedures section and Tissue Weighing, Collection, Processing and Evaluation (Attachment A) table from animals identified in the Terminal Procedures section will be embedded in paraffin, sectioned, mounted on glass slides, and stained with hematoxylin and eosin.

14.2. Microscopic Evaluation

Tissues as detailed in the Terminal Procedures section and Tissue Weighing, Collection, Processing and Evaluation (Attachment A) table will be evaluated histopathologically by a veterinary pathologist with training and experience in laboratory animal pathology.

Special stains may be used at the discretion of the pathologist to further characterize lesions and changes identified during routine evaluation of individual animals. Any special stains will be

documented in the individual animal data. Any additional stains or evaluations, if deemed necessary by the pathologist, may be added by study plan amendment following discussion with the Study Director and in consultation the Sponsor. Efforts will be made to evaluate all study plan-required tissues microscopically; however, it is not always feasible for every study plan-required tissue to be present on every slide. Study plan-required tissues that are not examined will be documented in the histopathology data and the impact of these missing tissues on the study will be documented in the pathology report.

Images may be generated for illustration of or consultation on histological observations. These images will not be used for data generation or interpretation, and will not be archived or included in the Final Report.

14.3. Pathology Peer Review

A pathology peer review will be conducted histopathologically by light microscopy on the following tissues:

Tissues:	DRG (cervical, lumbar and thoracic, with dorsal and ventral nerve roots),<br>Nerves (sciatic and tibial), and<br>Spinal cord (cervical, lumbar and thoracic, injection site)
Peer Review Pathologist:	Melissa M. Schutten, DVM, PhD, DACVP<br>Ultragenyx Pharmaceutical Inc.<br>32 Leveroni Court<br>Novato, CA 94949<br>USA<br>Tel: 415.475.6527<br>E-mail: mschutten@ultragenyx.com

Histopathology slides will be shipped to the pathologist, see Attachment B for shipping details. The peer review statement or equivalent documentation will be included as an appendix to the Final Report.

15. STATISTICAL ANALYSIS

Any data collected during the prestudy period will be tabulated, summarized or statistically analyzed. All statistical analyses will be performed within the respective study phase, unless otherwise noted. Numerical data collected on scheduled occasions will be summarized and statistically analyzed as indicated below according to sex and occasion.

15.1. Constructed Variables

Body Weight Changes:	Calculated between each scheduled interval as well as between the following intervals: from beginning to end of each phase

Organ Weight Relative to Body Weight:	Calculated against the terminal body weight for scheduled intervals
Organ Weight Relative to Brain Weight:	Calculated against the brain weight for scheduled intervals

Additional or alternative body weight or food consumption intervals may be evaluated to elucidate study results at the discretion of the Study Director.

15.2. Descriptive Statistical Analyses

Means, standard deviations (or % coefficient of variation or standard error, when deemed appropriate), ratios, percentages, numbers, and/or incidences will be reported as appropriate by dataset.

15.3. Inferential Statistical Methods

All statistical tests will be conducted at the 5% significance level. All pairwise comparisons will be conducted using two sided tests and will be reported at the 1% and 5% levels, unless otherwise noted.

The pairwise comparisons of interest are listed below:

Group 2 vs. Group 1

Group 3 vs. Group 1

Group 4 vs. Group 1

Analyses will be performed according to the matrix below when possible, but will exclude any group with less than 3 observations.

Statistical Matrix

Variables for Inferential Analysis	Statistical Method<br>Parametric/<br>Non-parametric
Body Weight	X
Body Weight Changes	X
Hematology Variables	X
Coagulation Variables	X
Clinical Chemistry Variables	X
Urinalysis Variables	X
Neurobehavioral Evaluation	X
Organ Weights	X
Organ Weight relative to Body Weight	X
Organ Weight relative to Brain Weight	X

15.4. Parametric/Non-parametric

Levene's test will be used to assess the homogeneity of group variances.

The groups will be compared using an overall one-way ANOVA F-test if Levene's test is not significant or the Kruskal-Wallis test if it is significant. If the overall F-test or Kruskal-Wallis test is found to be significant, then pairwise comparisons will be conducted using Dunnett's or Dunn's test, respectively. Datasets with two groups will be compared using a Dunnett's test (equivalent to t-test in Nevis 2012 tables) or Dunn's test (equivalent to Wilcoxon Rank-Sum test in Nevis 2012 tables).

16. COMPUTERIZED SYSTEMS

The following critical computerized systems may be used in the study. The actual computerized systems will be documented in the study data.

Critical Computerized Systems

System Name	Description of Data Collected and/or Analyzed
Provantis®	Test material receipt, accountability, formulation activities, in-life (e.g., clinical observations, body weights, food consumption, neurobehavioral evaluation), clinical pathology (clinical chemistry, coagulation, hematology, urinalysis), and/or postmortem (e.g., pathology)
Deviation Information Library	Deviations
Share Document Management System	Reporting
DocuSign™	Collection of 21 CFR Part 11 compliant signature
M-Files®	Reporting and collection of 21 CFR Part 11 compliant signature
Provantis® and/or SRS (CR-SEN in-house application built with SAS) and/or in-house reporting software Nevis 2012 (using SAS)	Statistical Analysis of in-life (body weights, food consumption, neurobehavioral evaluation); clinical pathology (clinical chemistry, coagulation, hematology, urinalysis); and postmortem (organ weights)
Mesa Laboratories AmegaView CMS	Continuous Monitoring System. Monitoring of standalone fridges, freezers, incubators, and selected laboratories to measure temperature, relative humidity, and CO_2, as appropriate
Johnson Controls Metasys	Building Automation System. Control of HVAC and other building systems, as well as temperature/humidity control and trending in selected laboratories and animal rooms
Phoenix	Computation of non-compartmental analysis, descriptive statistics and ratios, as well as graphical and tabular output

Data for parameters not required by the Study Plan, which are automatically generated by analytical devices used will be retained on file but not reported. Statistical analysis results that are generated by the program but are not required by the Study Plan and/or are not scientifically relevant will be retained on file but will not be included in the tabulations.

17. REGULATORY COMPLIANCE

The study will be performed in accordance with the OECD Principles of Good Laboratory Practice and as accepted by Regulatory Authorities throughout the European Union, United States of America (FDA), Japan (MHLW), and other countries that are signatories to the OECD Mutual Acceptance of Data Agreement.

Any portion of this study conducted in the USA will be performed in accordance with the US Department of Health and Human Services, Food and Drug Administration, United States Code of Federal Regulations, Title 21, Part 58: Good Laboratory Practice for Nonclinical Laboratory Studies and as accepted by Regulatory Authorities throughout the European Union (OECD Principles of Good Laboratory Practice), Japan (MHLW), and other countries that are signatories to the OECD Mutual Acceptance of Data Agreement.

Exceptions to GLPs include the following study elements:

- Characterization of the test and reference items will be/were performed by the Sponsor or Sponsor subcontractor according to established SOPs, controls, and approved test methodologies to ensure integrity and validity of the results generated; these analyses will not be/were not conducted in compliance with the GLP or GMP regulations.
- Stability testing of the supplied test and reference items will be/was performed by the Sponsor or Sponsor subcontractor according to established SOPs, controls, and approved test methodologies to ensure integrity and validity of the results generated; these analyses will not be/were not conducted in compliance with the GLP or GMP regulations.
- Concentration, stability, and homogeneity of the test and reference item formulations will not be determined in this study.
- Bioanalysis, gene expression, and splenocyte analysis will not be conducted in compliance with GLP regulations.

18. QUALITY ASSURANCE

18.1. Test Facility

The Test Facility Quality Assurance Program (QAP) will monitor the study to assure the facilities, equipment, personnel, methods, practices, records, and controls are in conformance with Good Laboratory Practice regulations. The QAP will review the Study Plan, conduct inspections at intervals adequate to assure the integrity of the study, and audit the Final Report to assure that it accurately describes the methods and standard operating procedures and that the reported results accurately reflect the raw data of the study.

19. AMENDMENTS AND DEVIATIONS

Changes to the approved Study Plan shall be made in the form of an amendment, which will be signed and dated by the Study Director. Every reasonable effort will be made to discuss any

necessary study plan changes in advance with the Sponsor. The Study Director will notify the Sponsor of deviations that may result in a significant impact on the study as soon as possible.

20. RETENTION AND DISPOSITION OF RECORDS, SAMPLES, AND SPECIMENS

All study-specific raw data, electronic data, documentation, Study Plan (and amendments, if any), retained samples and specimens, and interim (if applicable) and final reports will be archived by no later than the date of Final Report issue. All materials generated by Charles River from this study will be transferred to a Charles River archive. At least 1 year after issue of the Draft Report, the Sponsor will be contacted.

Disposition of residual/retained analytical samples will be as described in the table below.

Disposition of Residual/Retained Samples

Sample Type	Disposition	Schedule
Bioanalytical	Archived by the Sponsor	Samples will be archived by the Sponsor.

Records to be maintained will include, but will not be limited to, documentation and data for the following:

- Deviations, study plan, and study plan amendments
- Study schedule
- Study-related correspondence
- Test system receipt, health, and husbandry
- Test and reference item receipt, identification, preparation, and analysis
- In-life measurements and observations
- Clinical pathology sample collection and evaluation
- Bioanalytical sample collection and evaluation
- Gross and microscopic observations and related data
- Organ weight measurements
- Statistical analysis results

21. STUDY CLASSIFICATION

Study Category:	Toxicology
Study Type:	Single Dose Toxicity
Study Design:	Parallel
Primary Treatment CAS Registry Number:	Not Available
Primary Treatment Unique Ingredient ID:	Not Available

Class of Compound:	AAV9 Vector
Administration Dose Form:	Solution

22. REPORTING

A comprehensive Draft Report will be prepared following completion of the study and will be finalized following consultation with the Sponsor. The report will include all information necessary to provide a complete and accurate description of the experimental methods and results and any circumstances that may have affected the quality or integrity of the study.

The Sponsor will receive an electronic version of the Draft and Final Report provided in Adobe Acrobat PDF format (hyperlinked and searchable) along with a Microsoft Word version of the text. The PDF document will be created from native electronic files to the extent possible, including text and tables generated by the Test Facility. Report components not available in native electronic files and/or original signature pages will be scanned and converted to PDF image files for incorporation.

Reports should be finalized within 6 months of issue of the Draft Report. If the Sponsor has not provided comments to the report within 6 months of draft issue, the report will be finalized by the Test Facility unless other arrangements are made by the Sponsor.

23. JUSTIFICATIONS AND GUIDELINES

23.1. Justification of Test System and Number of Animals

At this time, studies in laboratory animals provide the best available basis for extrapolation to humans and are required to support regulatory submissions. Acceptable models that do not use live animals currently do not exist.

The Sprague Dawley rat was chosen as the animal model for this study as it is an accepted rodent species for nonclinical toxicity testing by regulatory agencies.

The total number of animals to be used in this study is considered to be the minimum required to properly characterize the effects of the test item. This study has been designed such that it does not require an unnecessary number of animals to accomplish its objectives.

23.2. Justification of Route and Dose Levels

The intrathecal injection route of exposure was selected because this is the intended route of human exposure.

The dose levels were selected based on information provided by the Sponsors' prior studies with AAV9/AP4M1 in mice, as well as prior pharmacology for other similar AAV9 vectors in mice, rats, pigs, and nonhuman primates. Based on those prior pharmacology studies, the chosen middle dose is anticipated to provide efficacy in ongoing mouse studies which should translate to humans, and the low dose may provide some minimal benefit. No clear dose-limiting toxicities were observed at doses equivalent to the middle dose in mice (2x below the middle dose and 2x

above the middle dose). At one year postdose, 3 out of 10 mice receiving the high dose had hepatocellular adenoma, which could have been caused by the test item. The middle dose in this rat study corresponds to a nearly maximum feasible dose (MFD) due to limitations on injection volume and test item concentration, and this is the target dose for human translation. This is bracketed by a high dose using a higher injection volume, and a 3-fold lower dose. None of the doses are expected to generate more than a minimal to moderate toxic effect. If any adverse effects are observed, the range of doses in the study design is an attempt to produce a graded response.

23.3. Guidelines for Study

The design of this study was based on the study objective(s), the overall product development strategy for the test item, and the following study design guidelines:

- ICH Harmonised Tripartite Guideline M3 (R2). *Nonclinical Safety Studies for the Conduct of Human Clinical Trials and Marketing Authorization for Pharmaceuticals.*
- ICH Harmonised Tripartite Guideline S6 (R1). *Preclinical Safety Evaluation of Biotechnology-Derived Pharmaceuticals*.
- Japanese Guidelines for Nonclinical Studies of Drugs Manual (1995). *Guidelines for Toxicity Studies of Drugs (Chapter 2, Single Dose Toxicity Study).*

24. ANIMAL WELFARE

The Study Plan and any amendment(s) or procedures involving the care and use of animals in this study will be reviewed and approved by CR-SEN Institutional Animal Care and Use Committee (IACUC). During the study, the care and use of animals will be conducted with guidance from the USA National Research Council and the Canadian Council on Animal Care (CCAC).

25. REFERENCES

Office of Laboratory Animal Welfare. *Public Health Services Policy on Humane Care and Use of Laboratory Animals*. Bethesda, MD: National Institutes of Health. Current edition.

AMENDMENT APPROVAL

All electronic signatures appear at the end of the document upon finalization.

Appendix 1

SPONSOR APPROVAL

The Study Plan Amendment was approved by the Sponsor by e-mail on the date designated below. The correspondence giving approval will be archived, as appropriate with other Sponsor communications.

04 Mar 2022
Date of Sponsor Approval

ATTACHMENT A

Tissue Weighing, Collection, Processing, and Evaluation Table

Organ	Weigh	Macroscopic Evaluation and Collection	Histology Processing	Microscopic Evaluation	Bioanalysis and Gene Expression	Remaining frozen tissue to archive
Animal ID	-	X	-	-	-	-
Artery, aorta	-	X	-	-	-	-
Body cavity, nasal	-	X	-	-	-	-
Bone marrow, sternum	-	X	-	-	-	-
Bone marrow smear	-	X[a]	-	-	-	-
Bone, femur, right	-	X	-	-	-	-
Bone, sternum	-	X	-	-	-	-
Brain	X	X	X	X (left)	X (right) (forebrain, midbrain, and hindbrain)	X (right) (forebrain, midbrain, and hindbrain)
Epididymis	X (2)	X (2)	X	X	-	-
Esophagus	-	X	-	-	-	-
Eye	-	X (2)	X (1) (left)	X (1) (left)	-	X (1) (right)
Ganglion, dorsal root, cervical, lumbar, and thoracic, with dorsal and ventral nerve roots[d,f]	-	X (2)	X (2)	X (2)	X (C6-C7, T1-T2, L6)	X (T3, T9-T12)
Gland, adrenal	X (2)	X (2)	X (2)	X (2)	-	-
Gland, clitoral	-	X (2)	-	-	-	-
Gland, Harderian	-	X (2)	-	-	-	-
Gland, lacrimal	-	X (2) (extra-orbital)	-	-	-	-
Gland, mammary	-	X	-	-	-	-
Gland, parathyroid	-[b]	X (2)	X (2)	X (2)	-	-
Gland, pituitary	X	X	X	X	-	-
Gland, preputial	-	X (2)	-	-	-	-
Gland, prostate	X	X	X	X	-	-
Gland, salivary, submandibular	-	X (2)	-	-	-	-
Gland, salivary, sublingual	-	X (2)	-	-	-	-
Gland salivary, parotid	-	X (2)	-	-	-	-
Gland, seminal vesicle	-	X (2)	-	-	-	-
Gland, thyroid	X (2)	X (2)	X (2)	X (2)	-	-
Gland, Zymbal's	-	X (2)	-	-	-	-
Gut-associated lymphoid tissue[c]	-	X	-	-	-	-
Heart	X	X	X	X	X	-
Joint, femorotibial, right	-	X	-	-	-	-
Kidney	X (2)	X (2)	X (left)	X (left)	X (right)	X (right)

Appendix 1

Organ	Weigh	Macroscopic Evaluation and Collection	Histology Processing	Microscopic Evaluation	Bioanalysis and Gene Expression	Remaining frozen tissue to archive
Large intestine, cecum	-	X	-	-	-	-
Large intestine, colon	-	X	-	-	-	X
Large intestine, rectum	-	X	-	-	-	-
Larynx	-	X	-	-	-	-
Liver	X	X	X	X	X (left lateral lobe)	X (left lateral lobe)
Lung	-	X	X	X	-	X (right caudal)
Lymph node(s) draining administration site(s): deep cervical and iliac	-	X (2)	X (1, left)	X (1, left)	-	X (1, right)
Lymph node, mandibular	-	X (2)	X (1, left)	X (1, left)	-	X (1, right)
Lymph node, mesenteric	-	X	X	X	-	X (1 lymph node)
Muscle, skeletal, gastrocnemius	-	X (2)	X (1, left)	X (1, left)	X (1, right)	X (1, right)
Muscle, skeletal, Biceps Femoris	-	X (2)	X (1, left)	X (1, left)	X (1, right)	X (right)
Nerve, optic	-	X (2)	X (left)	X (left)	-	X (right)
Nerve, sciatic	-	X (2)	X (1, left)	X (1, left)	X (1, right)	-
Nerve, tibial	-	X (2)	X (1, left)	X (1, left)	-	X (right)
Ovary	X (2)	X (2)	X (1, left)	X (1, left)	X (1, right)	-
Oviduct	-	X (2, left in NBF)	-	-	-	X (1, right)
Pancreas	-	X	-	-	-	X
Skin	-	X	-	-	-	X (left inguinal)
Small intestine, duodenum	-	X	-	-	-	X
Small intestine, ileum	-	X	-	-	-	X
Small intestine, jejunum	-	X	-	-	-	X
Spinal cord; cervical, thoracic, and lumbar and injection site [d,e]	-	X	X	X	X (C6-C7, T9-T12, L6)	X (T1-T3)
Spleen	X	X	X	X	X	-
Stomach	-	X	-	-	-	X (1 cm from both glandular and non-glandular portion)
Testis	X (2)	X (2)	X (Left)	X (Left)	X (Right)	X (Right)
Thymus	X	X	X	X	X	X
Tongue	-	X	-	-	-	-
Trachea	-	X	-	-	-	-

Appendix 1

Organ	Weigh	Macroscopic Evaluation and Collection	Histology Processing	Microscopic Evaluation	Bioanalysis and Gene Expression	Remaining frozen tissue to archive
Ureter	-	X (2, left in NBF)	-	-	-	X (1, right)
Urinary bladder	-	X	-	-	-	X (half)
Uterus/Cervix	X	X	X	X	-	-
Vagina	-	X	-	-	-	-

X = Procedure to be conducted. - = Not applicable. (1) = one side. (2) = both sides.
Macroscopic abnormalities in the organs listed and in other organs will be sampled at necropsy, processed for histology and examined microscopically.

[a] Two bone marrow smears will be collected from the femur at scheduled and unscheduled necropsies (for possible examination). Smears will not be collected from animals that are found dead or from animals that were euthanized moribund and then stored in the refrigerator prior to necropsy. Bone marrow smears are allowed to air dry and are not fixed in formalin.
[b] Weigh with gland, thyroid.
[c] From small intestine: Peyer's patch or solitary lymphoid follicle.
[d] In situ, decalcified before sectioning
[e] Transverse and/or oblique sections
[f] Nerve roots will be examined if present in section of dorsal root ganglia.

Appendix 1

ATTACHMENT B

Shipment of Samples and Study Records

Matrix	Purpose	Day/ Week/ Aliquot	Proposed Shipment Date	Conditions for Shipment	Recipient/Address
Unused Sponsor-supplied bulk test materials/ remaining dose formulations	Return to sponsor	Not Applicable	Following the end of dosing	Dry ice	Attention to:<br>Steven Gray, PhD<br>University of Texas Southwestern Medical Center<br>NA2.508<br>5323 Harry Hines Blvd<br>University of Texas Southwestern Medical Center<br>Dallas, TX 75390<br>Tel: 214-648-0670<br>E-mail:<br>steven.gray@UTSouthwestern.edu
Serum, CSF, splenocytes, and frozen tissue	Bioanalysis	X	Within 2 weeks from collection	Dry ice	Steven Gray, PhD<br>University of Texas Southwestern Medical Center<br>NA02.508<br>5323 Harry Hines Blvd<br>University of Texas Southwestern Medical Center<br>Dallas, TX 75390<br>Tel: 214-648-0670<br>E-mail:<br>steven.gray@UTsouthwestern.edu
Tissue slides	Pathology peer review	All slides	Following completion of the microscopic evaluation	Ambient conditions	Attn: Taira Saracco<br>Ultragenyx Pharmaceutical, Inc.<br>32 Leveroni Court<br>Novato, CA<br>USA<br>Tel: 415.475.6527<br>E-mail: tvidler@ultragenyx.com,<br>jorogers@ultragenyx.com,<br>kpoon@ultragenyx.com

Alternate shipping details may be provided thereafter and will be documented in study correspondence.

Appendix 1

SIGNATURE(S) FOR DOCUMENT: 5550008 - Study Plan Amendment 8

**Study Director Approval:**	I approve this document.
Name:	**Cinquino, Stefania**
Cinquino, Stefania	04-Mar-2022 19:29:46 (UTC+00:00)
Electronically Signed in M-Files	Timestamp

FINAL STUDY PLAN

Test Facility Study No. 5550008

Sponsor Reference No. UTSW.Gray-003

A Single-Dose Toxicity Study of AAV9/AP4M1 by Intrathecal Injection in Rats

GLP

SPONSOR:
University of Texas Southwestern Medical Center
5323 Harry Hines Blvd
Dallas, TX 75390
USA

TEST FACILITY:
Charles River Laboratories Montreal ULC
Senneville Site (CR-SEN)
22022 Transcanadienne
Senneville, QC H9X 3R3
Canada

TABLE OF CONTENTS

1. OBJECTIVE....................3
2. PROPOSED STUDY SCHEDULE....................3
3. SPONSOR....................3
4. RESPONSIBLE PERSONNEL....................4
5. TEST MATERIALS....................5
6. DOSE FORMULATION AND ANALYSIS....................7
7. TEST SYSTEM....................7
8. HUSBANDRY....................8
9. EXPERIMENTAL DESIGN....................10
10. IN-LIFE PROCEDURES, OBSERVATIONS, AND MEASUREMENTS....................10
11. CLINICAL PATHOLOGY....................11
12. BIOANALYSIS....................13
13. TERMINAL PROCEDURES....................13
14. HISTOLOGY AND MICROSCOPIC EVALUATION....................17
15. STATISTICAL ANALYSIS....................17
16. COMPUTERIZED SYSTEMS....................19
17. REGULATORY COMPLIANCE....................20
18. QUALITY ASSURANCE....................20
19. AMENDMENTS AND DEVIATIONS....................20
20. RETENTION AND DISPOSITION OF RECORDS, SAMPLES, AND SPECIMENS......21
21. STUDY CLASSIFICATION....................21
22. REPORTING....................22
23. JUSTIFICATIONS AND GUIDELINES....................22
24. ANIMAL WELFARE....................23
25. REFERENCES....................23
TEST FACILITY APPROVAL....................24
SPONSOR APPROVAL....................25
ATTACHMENT A....................26
ATTACHMENT B....................29

1. OBJECTIVE

The objective of this study is to characterize the toxicity, biodistribution, and gene expression of AAV9/AP4M1, for the treatment of Spastic Paraplegia Type 50 (SPG50) caused by the AP4M1 gene mutation, when given by a single intrathecal injection to rats and to evaluate the potential reversibility and/or progression of any findings.

2. PROPOSED STUDY SCHEDULE

Proposed study dates are listed below. Actual dates will be included in the Final Report.

Experimental Starting Date:	09 Feb 2021<br>(First date of study-specific data collection)
Experimental Completion Date:	dd MMM yyyy<br>(Last date on which data are collected)
Animal Arrival:	09 Feb 2021
Initiation of Dosing:	16 Feb 2021 (Replicate 1)<br>19 Feb 2021 (Replicate 1)<br>22 Feb 2021 (Replicate 1)
Completion of In-life:	01 Mar 2021 (Day 8)<br>22 Mar 2021 (Day 29)<br>23 May 2021 (Day 91)<br>(Last date of necropsy)
Draft Report:	30 Jul 2021<br>(9 weeks following completion of in-life)
Final Report:	dd MMM yyyy<br>(Expected date of Study Director signature of report)
SEND Dataset Package Delivery	Based on regulatory submission date

3. SPONSOR

Role/Phase	Name	Contact Information
Sponsor Representative	Steven Gray, PhD	University of Texas Southwestern Medical Center<br>5323 Harry Hines Blvd<br>University of Texas Southwestern Medical Center<br>Dallas, TX 75390<br>Tel: 214.648.0670<br>E-mail:<br>steven.gray@UTsouthwestern.edu
Sponsor Study Monitor	To be added by amendment	Address as cited for the Sponsor<br>To be added by amendment

Role/Phase	Name	Contact Information
Alternate Study Contact	Terry Pirovol	CureSPG50<br>6 Topham Road<br>Toronto, ON M4B 3K2<br>Canada<br>Tel:<br>E-mail: tpirovol@hotmail.com

4. RESPONSIBLE PERSONNEL

Role/Phase	Quality Assurance Program (QAP)	Name	Contact Information
Study Director	Charles River	Stefania Cinquino, BSc	Address as cited for Test Facility<br>Tel: 514.630.8200, ext 2151<br>E-mail: stefania.cinquino@crl.com
Test Facility Management	Charles River	Julie Douville, PhD	Address as cited for Test Facility<br>Tel: 514.630.8200, ext 8309<br>E-mail: julie.douville@crl.com
Test Facility QAP	Charles River	Nooshin Davani, BSc, RQAP-GLP	Address as cited for Test Facility<br>Tel: 514.630.8200, ext 2605<br>E-mail: nooshin.davani@crl.com
**Individual Scientist (IS)**			
Clinical Pathology	Charles River	Virginie Allegret, DVM, IPSAV, DES, DACVP	Address as cited for Test Facility<br>Tel: (514) 630-8200, ext 2028<br>E-mail: virginie.allegret@crl.com
Pathology	Charles River	To be added by amendment	Address as cited for Test Facility<br>To be added by amendment
**Principal Investigator (PI)**			
Serum, CSF, and Tissue BiodistributionAnalysis (Bioanalysis) [a]	Test Site	Steven Gray, PhD	University of Texas Southwestern Medical Center<br>5323 Harry Hines Blvd<br>University of Texas Southwestern Medical Center<br>Dallas, TX 75390<br>Tel: 214-648-0670<br>E-mail: steven.gray@UTsouthwestern.edu
Tissue Gene expression Analysis [a]	Test Site	Steven Gray, PhD	University of Texas Southwestern Medical Center<br>5323 Harry Hines Blvd<br>University of Texas Southwestern Medical Center<br>Dallas, TX 75390<br>Tel: 214-648-0670<br>E-mail: steven.gray@UTsouthwestern.edu

[a] Sponsor.

Each IS and PI is required to report all deviations or other circumstances that could affect the quality or integrity of the study to the Study Director in a timely manner for

authorization/acknowledgement. Each IS and PI will provide a report addressing their assigned phase of the study, which will be included as an appendix to the Final Report.

The IS phase report will include the following:

- A listing of critical computerized systems used in the conduct and/or interpretation of the assigned study phase

The PI phase report will include the following:

- A Statement of Compliance (if the applicable phase is GLP)
- A QA Statement (for Sponsor-designated PI or for Test Facility-designated PI if audited by a QAP other than that of the Test Facility and if the applicable phase is GLP)
- The archive site for all records, samples, specimens and reports generated from the phase or segment (alternatively, details regarding the retention of the materials may be provided to the Study Director for inclusion in the Final Report)
- A listing of critical computerized systems used in the conduct and/or interpretation of the assigned study phase

5. TEST MATERIALS

5.1. Test and Reference Item Characterization

The Sponsor will provide to the Test Facility documentation of the identity, strength, purity, composition, and stability for the test and reference items. A Certificate of Analysis or equivalent documentation will be provided for inclusion in the Final Report.

The Sponsor has appropriate documentation on file concerning the method of synthesis, fabrication or derivation of the test and reference items, and this information is available to the appropriate regulatory agencies should it be requested.

5.2. Test Item Identification

Test Item Identification

	Test Item
**Identification:**	AAV9/AP4M1
**Alternate Identification:**	To be added by amendment
**Batch/Lot No.:**	To be added by amendment
**Expiration/Retest Date:**	To be added by amendment
**Physical Description:**	To be added by amendment
**Purity:**	To be added by amendment<br>(not used for calculation purposes)
**Concentration:**	XX vg/mL; to be added by amendment
**Storage Conditions (temperature set to maintain):**	-80°C
**Provided by:**	Sponsor

5.3. Reference Item/Vehicle Identification

Reference Item/Vehicle Identification

	Reference Item
**Identification:**	PBS containing 5% D-sorbitol and 0.001% pluronic F-68
**Storage Conditions (temperature set to maintain):**	-80°C
**Provided by:**	Sponsor

5.4. Reserve Samples

Due to the study duration, a reserve sample will not be collected.

5.5. Test and Reference Item Inventory and Disposition

Records of the receipt, distribution, storage, and disposition of test materials will be maintained. All unused Sponsor-supplied bulk test materials will be returned to the Sponsor following issuance of the Draft Report unless otherwise requested (documentation will be retained in the study record). An earlier shipment of these materials may also be requested and authorized by the Study Director and Sponsor. See Shipment of Samples and Study Records (Attachment B) for shipping details.

5.6. Safety

The safety precautions for the test item and dose formulations will be documented in a Test Material Safety Data Sheet (TMSDS) based on the SDS or similar document.

6. DOSE FORMULATION AND ANALYSIS

6.1. Preparation of Formulations

Dose formulations will be divided into aliquots, where required, and dispensed on each dosing occasion.

Preparation Details

Dose Formulation	Frequency of Preparation	Storage Conditions (temperature set to maintain)
Reference Item	Used as received	4°C
Test Item	At least once	4°C

Any residual volumes from each dosing occasion will be discarded unless otherwise requested by the Study Director.

6.2. Preparation Details

Dosing formulations will be prepared under a laminar flow hood using clean procedures.

Dosing formulations will be prepared based on Sponsor's instructions at appropriate concentrations to meet dose level requirements.

6.3. Sample Collection and Analysis

Samples for dose formulation analysis will not be collected by the Test Facility.

7. TEST SYSTEM

Species: Rat

Strain: Crl:CD(SD) Sprague Dawley rat

Condition: Purpose-bred, naive

Source: Charles River Labs International Inc., Raleigh, NC, USA

Number of Males to be Assigned: 60 (plus 6 alternates)

Number of Females to be Assigned: 60 (plus 6 alternates)

Target Age at the Initiation of Dosing: 7 to 8 weeks

Target Weight at the Initiation of Dosing: 170 to 225 g (males)/140 to 200 g (females)

The actual age and weight of the animals at the initiation of dosing will be listed in the Final Report.

7.1. Animal Identification

Method:	Subcutaneously implanted electronic identification chip

7.2. Environmental Acclimation

Method:	At least 5 days will be allowed between animal receipt and the start of dosing in order to accustom the animals to the laboratory environment.

7.3. Selection, Assignment, Replacement, and Disposition of Animals

Selection:	Animals will be randomly assigned to groups. Males and females will be randomized separately. Animals in poor health will not be assigned to groups. Body weights will be monitored and any replacements will be undertaken prior to initiation of dosing to allow for comparable group means between groups/sex.
Replacement:	Before the initiation of dosing, any assigned animals considered unsuitable for use in the study will be replaced by alternate animals obtained from the same shipment and maintained under the same environmental conditions. After initiation of dosing, study animals may be replaced during the replacement period with alternate animals in the event of accidental injury, non-test item-related health issues, or similar circumstances. The alternate animals may be used as replacements on the study within 2 days. General in-life assessments will include alternate animals until released from study.
Disposition:	The disposition of all animals will be documented in the study records.

8. HUSBANDRY

8.1. Housing

Housing:	Group-housed (up to 3 animals of the same sex and same dosing group together).
Caging:	Polycarbonate cages containing appropriate bedding. Where possible, control group animals will be housed on a separate rack from the test item-treated animals.
Cage Identification:	Color-coded cage card indicating study, group, animal number(s), and sex.

Housing set-up is as described in the *Guide for the Care and Use of Laboratory Animals* (National Research Council, 2011) Animals will be separated during designated procedures/activities or will be separated as required for monitoring and/or health purposes, as deemed appropriate by Study Director and/or Clinical Veterinarian. Cages will be arranged on the racks in group order.

8.2. Animal Enrichment

Psychological/ Environmental Enrichment:	Animals will be socially housed and will be provided with items such as a hiding device, and a chewing object, except during study procedures/activities.

8.3. Environmental Conditions

The targeted conditions for animal room environment will be as follows:

Temperature:	22 ±3°C
Humidity:	30% to 70%
Light Cycle:	12 hours light and 12 hours dark (except during designated procedures)

8.4. Food

Diet:	Lab Diet Certified CR Rodent Diet 5CR4
Type:	Pellets (same diet in meal form may be provided to individual animals as warranted by clinical signs, e.g., broken/damaged incisors or other health changes)
Frequency:	Ad libitum, except during designated procedures
Analysis:	Results of analysis for nutritional components and environmental contaminants are provided by the supplier and are kept on file at the Test Facility. It is considered that there are no known contaminants in the feed that would interfere with the objectives of the study.

8.5. Water

Type:	Municipal tap water, treated by reverse osmosis and ultraviolet irradiation.
Frequency/Ration:	Freely available to each animal via an automatic watering system (except during designated procedures). Water bottles can be provided, if required.
Analysis:	Periodic analysis of the water is performed, and results of these analyses are kept on file at the Test Facility. It is considered that there are no known contaminants in the water that would interfere with the outcome of the study.

8.6. Veterinary Care

Veterinary care will be available throughout the course of the study and animals will be examined by the veterinary staff as warranted by clinical signs or other changes. In the event that animals show signs of illness or distress, the responsible veterinarian may make initial recommendations about treatment of the animal(s) and/or alteration of study procedures, which

must be approved by the Study Director or Scientific designate. Treatment of the animal(s) for minor injuries or ailments may be approved without prior consultation with the Sponsor representative when such treatment does not impact fulfillment of the study objectives. If the condition of the animal(s) warrants significant therapeutic intervention or alterations in study procedures, the Sponsor representative will be contacted, when possible, to discuss appropriate action. If the condition of the animal(s) is such that emergency measures must be taken, the Study Director (or Scientific designate) and/or veterinarian will attempt to consult with the Sponsor representative prior to responding to the medical crisis, but the Study Director (or Scientific designate) and/or veterinarian has authority to act immediately at his/her discretion to alleviate suffering. The Sponsor representative will be fully informed of any such events.

9. EXPERIMENTAL DESIGN

Experimental Design

Group No.	Test Material	Dose Level (vg)	Dose Volume (µL)	Dose Concentration (vg/µL)	No. of Animals					
					Main Study		Recovery Study			
					Day 8 Necropsy[a]		Day 29 Necropsy[b]		Day 91 Necropsy[c]	
					M	F	M	F	M	F
1	Reference Item	0	60	0	5	5	5	5	5	5
2	AAV9/AP4M1	0.33×10^{12}	20	0.17×10^{11}	5	5	5	5	5	5
3	AAV9/AP4M1	1×10^{12}	20	0.5×10^{11}	5	5	5	5	5	5
4	AAV9/AP4M1	3×10^{12}	60	0.5×10^{11}	5	5	5	5	5	5

M = Males; F = Females
[a] Animals scheduled for Necropsy on Day 8.
[b] Animals scheduled for Necropsy on Day 29.
[c] Animals scheduled for Necropsy on Day 91.

9.1. Administration of Test and Reference Items

Dose Route: Intrathecal bolus injection at the lumbar level via direct puncture

Frequency: Once

Duration: Single Dose

Method: The day of dosing will be designated as Day 1. The animals will be anesthetized with isoflurane, administered eye lubricant, shaved and prepared for surgery according to the appropriate SOP. The test and reference items will be administered by intrathecal bolus injection through direct puncture as per SOP.

10. IN-LIFE PROCEDURES, OBSERVATIONS, AND MEASUREMENTS

General In-life Assessments - Main and Recovery Study Animals

Parameter	Frequency	Comments

Parameter	Frequency	Comments
**Mortality/ Moribundity Checks**[a]	Twice daily (morning and afternoon) starting upon arrival through termination.	Animals will be observed within their cage unless removal is necessary for identification or confirmation of possible findings.
**Postdose Observations**[a]	2 hours (± 30 minutes) postdose after the end of each group for each sex	Cage side observations unless removal is necessary for identification or confirmation of possible findings.
**Detailed Clinical Observations**[a]	Weekly starting Day -1, and at least every 2 weeks during the prestudy period.	Animals are removed from the cage.
**Individual Body Weights**[a]	Weekly starting Day -1, and at least every 2 weeks during the prestudy period.	Fasted weight on the day of necropsy. No terminal body weights collected from animals found dead or preterminally euthanized.
**Food Consumption**[a]	Weekly starting Day -1 and throughout the study	Quantitatively measured

[a] Minimum required frequency for this parameter indicated

11. CLINICAL PATHOLOGY

11.1. Sample Collection

Clinical Pathology Sample Collection – Main and Recovery Study Animals

Group Nos.	Occasion/ Time Point	Hematology	Coagulation	Clinical Chemistry	Urinalysis
1 to 4	Day 8	X	X	X	X
1 to 4	Day 29	X	X	X	X
1 to 4	Day 91	X	X	X	X
Unscheduled euthanasia (when possible)		X	X	X	-
**Overnight Fasting:**		-	-	Yes	Yes
**Method/Comments:**		Abdominal aorta at termination[a]			Individual housed in metabolism cages; overnight collection
**Volume (mL):**		Up to 1 mL	1.2	0.7	As available
**Anticoagulant:**		EDTA	Sodium citrate	None, in SST	-
**Special Requirements:**		-	-	-	-
**Processing:**		None	Plasma	Serum	-

X = Sample to be collected; - = Not applicable; SST = serum separator tube.
[a] Immediately prior to necropsy while the animals are under isoflurane anesthesia.

11.2. Hematology

Hematology Parameters

Red blood cell count	Platelet count
Hemoglobin concentration	White blood cell count
Hematocrit	Neutrophil count (absolute)
Mean corpuscular volume	Lymphocyte count (absolute)
Red blood cell distribution width	Monocyte count (absolute)
Mean corpuscular hemoglobin concentration	Eosinophil count (absolute)
Mean corpuscular hemoglobin	Basophil count (absolute)
Reticulocyte count (absolute)	Large unstained cells (absolute)

A blood smear will be prepared from each hematology sample and may be examined for confirmation of hematology results.

11.3. Coagulation

Coagulation Parameters

Activated partial thromboplastin time<br>Fibrinogen	Prothrombin time<br>Sample quality

11.4. Clinical Chemistry

Clinical Chemistry Parameters

Alanine aminotransferase<br>Aspartate aminotransferase<br>Alkaline phosphatase<br>Gamma-glutamyltransferase<br>Creatine kinase<br>Total bilirubin[a]<br>Urea nitrogen<br>Creatinine<br>Calcium<br>Phosphorus	Total protein<br>Albumin<br>Globulin<br>Albumin/globulin ratio<br>Glucose<br>Cholesterol<br>Triglycerides<br>Sodium<br>Potassium<br>Chloride<br>Sample quality

[a] When total bilirubin is > 0.5 mg/dL, direct bilirubin will also be measured and indirect bilirubin will be calculated.

11.5. Urinalysis

Urinalysis Parameters

Color<br>Appearance/Clarity<br>Specific gravity<br>Volume<br>pH	Protein[a]<br>Glucose[a]<br>Bilirubin<br>Ketones<br>Blood

[a] Semi-quantitative measurement

11.6. Bone Marrow Smear Evaluation

Bone marrow smears will be collected and prepared as described in Attachment A and may be examined for confirmation of results.

12. BIOANALYSIS

12.1. Bioanalytical Sample Collection

In addition to those listed below, a sample will be collected from animals pre-terminally euthanized, when possible.

Bioanalytical Sample Collection

Group Nos.	Time Points
	At termination
1 to 4	X
**Method/Comments:**	Abdominal aorta at termination[a]
**Target Volume[a] (mL):**	1
**Anticoagulant:**	None, in SST
**Special Requirements:**	None
**Processing:**	Serum

X = Sample to be collected; - = Not applicable; SST = serum separator tube
[a] Immediately prior to necropsy while the animals are under isoflurane anesthesia.

12.2. Bioanalytical Sample Processing

The samples will be centrifuged and the resultant serum will be separated, transferred to 2 uniquely labeled polypropylene tubes of approximately equal volume, frozen immediately over dry ice and transferred in a freezer set to maintain -80°C.

The samples will be shipped to the University of Texas Southwestern Medical Center, see Attachment B. The bioanalytical laboratory will be notified before shipment of the samples. Samples will be stored at the bioanalytical laboratory in a freezer set to maintain -60ºC or below until analysis.

12.3. Bioanalytical Sample Analysis

Analysis will be added by amendment.

13. TERMINAL PROCEDURES

Terminal procedures are summarized in the following tables:

Replaced, Found Dead, and Unscheduled Euthanasia Animals

Animals	Necropsy Procedures				Histology Processing	Microscopic Evaluation
	CSF Collection[d]	Necropsy	Tissue Collection	Organ Weights		
Animals replaced prestudy	-	X	Standard Diagnostic List	-	-	-
Animals replaced after dosing start	-	X	Full List[a]	-	-	-

Animals	Necropsy Procedures				Histology Processing	Microscopic Evaluation
	CSF Collection[d]	Necropsy	Tissue Collection	Organ Weights		
Unscheduled deaths after dosing start	X (unscheduled euthanasia only)	X	Full List[a]	-	Full List[a]	Select Tissues[a]

Main Study Animals

Group No.	Scheduled Euthanasia Day	Necropsy Procedures				Histology Processing	Microscopic Evaluation
		CSF Collection[b]	Necropsy	Tissue Collection	Organ Weights		
1	8	X	X	Full List[a]	Full List[a]	Full List[a]	Select Tissues[a]
2						Full List[a]	Select Tissues[a]
3						Full List[a]	Select Tissues[a]
4						Full List[a]	Select Tissues[a]

Recovery Study Animals

Group No.	Scheduled Euthanasia Day	Necropsy Procedures				Histology Processing	Microscopic Evaluation
		CSF Collection[b]	Necropsy	Tissue Collection	Organ Weights		
1	29	X	X	Full List[a]	Full List[a]	Full List[a]	Select Tissues[a]
2						Full List[a]	Select Tissues[a]
3						Full List[a]	Select Tissues[a]
4						Full List[a]	Select Tissues[a]

Recovery Study Animals

Group No.	Scheduled Euthanasia Day	Necropsy Procedures				Histology Processing	Microscopic Evaluation
		CSF Collection[b]	Necropsy	Tissue Collection	Organ Weights		
1	91	X	X	Full List[a]	Full List[a]	Full List[a]	Select Tissues[a]
2						Full List[a]	Select Tissues[a]
3						Full List[a]	Select Tissues[a]
4						Full List[a]	Select Tissues[a]

X = Procedure to be conducted; - = Not applicable.
[a] See Tissue Weighing, Collection, Processing and Evaluation table in Attachment A for list of tissues applicable to each procedure.
[b] As much CSF as possible will be collected from the cisterna magna at necropsy.

13.1. Method of Euthanasia

The animals will undergo exsanguination by incision from the abdominal aorta following isoflurane anesthesia, unless deemed inappropriate by the Study Director and/or the clinical veterinarian.

13.2. Unscheduled Euthanasia

Main and Recovery Study animals to be euthanized for humane reasons before the scheduled time will undergo sample collection for evaluation of clinical pathology parameters and bioanalysis, if possible as specified in Section 11.1 and Section 12.1.

Tissues from animal replaced after the start of dosing will be retained (as per Tissue Collection and Preservation section) and any data generated will not be included in the report unless deemed appropriate by the Study Director.

13.3. Scheduled Euthanasia

Main and Recovery Study animals surviving until scheduled euthanasia will be food deprived overnight. Animal will undergo blood sample collection for evaluation of clinical pathology parameters and bioanalysis. When possible, the animals will be euthanized rotating across dose groups such that similar numbers of animals from each group, including controls, will be necropsied throughout the day.

13.4. Necropsy

Animals as detailed in the Terminal Procedures section will be subjected to a complete necropsy examination, which will include evaluation of the carcass and musculoskeletal system; all external surfaces and orifices; cranial cavity and external surfaces of the brain; and thoracic, abdominal, and pelvic cavities with their associated organs and tissues. Necropsy examinations will be performed by qualified personnel with appropriate training and experience in animal anatomy and gross pathology. A veterinary pathologist, or other suitably qualified person, will be available for consultation. Images may be generated for illustration of or consultation on gross observations. These images will not be used for data generation or interpretation, and will not be archived or included in the final report.

13.5. Bioanalytical Cerebrospinal Fluid (CSF) Collection

At scheduled termination and for preterminally euthanized animals, samples of CSF will be collected from all animals while under isoflurane anesthesia. CSF samples (as much as possible) will be collected under via the cisterna magna, and transferred into uniquely labeled clear polypropylene tubes. The CSF samples will be placed on dry ice and then transferred in a freezer set to maintain -80°C. CSF samples will then be shipped to the University of Texas Southwestern Medical Center, see Attachment B.

13.6. Organ Weights

The organs detailed in the Terminal Procedures section and Tissue Weighing, Collection, Processing and Evaluation (Attachment A) table will be weighed at necropsy. Paired organs will be weighed together. In the event of gross abnormalities, in addition to the combined weight, the weight of each organ of a pair may be taken and entered as a tissue comment. Organ weight as a percent of body weight (using the terminal body weight) and organ weight as a percent of brain weight will be calculated.

13.7. Tissue Collection and Preservation

Representative samples of tissues will be collected and preserved in 10% neutral buffered formalin, except for tissues requiring alternate fixatives as defined by standard operating

procedures, as detailed in the Terminal Procedures section and Tissue Weighing, Collection, Processing and Evaluation (Attachment A) table. Additional tissue samples may be collected to elucidate abnormal findings.

13.8. Splenocyte Sample Collection Analysis

The spleen will be collected from all animals at scheduled necropsy. Dissected spleens, half, will be placed into prechilled tubes containing RPMI media and stored at 2°C to 8°C or on wet ice before processing to splenocytes. Splenocytes will then be cryopreserved/frozen until analysis.

Samples will be processed according to standard CR-SEN SOP, except that all washes will be performed with RPMI Media and samples will be frozen as follows:

Prepare a sufficient number of 2 mL cryovials to hold the cells at 2e7 cells/mL. Open the vials and add 100 mcL of Hybridoma-Grade DMSO (Freezing media is 90% heat-inactivated FBS and 10 % DMSO). Add sufficient heat-inactivated FBS to the cell pellet to put the cells at 2e7/mL when added to the DMSO. Gently resuspend the pellet via pipetting and then add to the tubes containing DMSO. The act of addition and higher density of FBS will mix the FBS and DMSO yielding an evenly distributed freezing media. 1-2 gentle inversions can be used to assure even mixing. If adequate splenocytes are available, 3 aliquots of cells at 2e7/mL will be prepared for each animal. Splenocytes will be stored in frozen liquid nitrogen until shipped to the Sponsor on dry ice. See Attachment B for shipping details.

The splenocytes will be analyzed for T-cell responses against AAV9 and AP4M1. All analytical work will be conducted by the Sponsor, using an analytical method developed and qualified by that laboratory.

13.9. Tissue Collection for Biodistribution and Gene Expression Determination

At scheduled termination and for preterminally euthanized animals, tissues in the Tissue Weighing, Collection, Processing and Evaluation (Attachment A) table from animals identified in the Terminal Procedures section will be collected for biodistribution and gene expression. Samples (between 40 and 50 mg, when possible) will be collected in duplicate, snap-frozen in liquid nitrogen and stored on dry ice until transferred to a freezer set to maintain -80°C until shipped to the University of Texas Southwestern Medical Center for analysis, see Attachment B.

Samples will collected using clean removal techniques.

When possible, the brain will be collected first, then the liver followed by major organs (kidney, lung, heart, spleen), then remaining tissues. The brain will be hemisected at necropsy, and samples for biodistribution and gene expression determination will be collected from the right hemisphere, while the left hemisphere will be fixed in formalin for histopathological evaluation. For animals found dead, the whole brain will be fixed in formalin.

Tissues for histopathology will be collected prior to sample collection for any other parameters.

Following sample collection of all tissues designated for biodistribution analysis, remaining tissues will be stored in a freezer set to maintain ≤ -60ºC) for possible future analysis.

13.10. Biodistribution/Gene Expression Analysis

Sponsor-selected tissue samples will be analyzed for concentration of vector DNA (as per Section 12.3.) and gene expression by qPCR or ddPCR.

All analytical work will be conducted by the Sponsor, using an analytical method developed and qualified by that laboratory that conforms to FDA guidelines but not in adherence with GLP. The work performed in conjunction with this study will not be conducted in compliance with GLPs and will not subject to review by the Quality Assurance Unit (QAU) of that laboratory. A Final Report will be prepared and submitted to Testing Facility for inclusion as an appendix in the main study Final Report.

14. HISTOLOGY AND MICROSCOPIC EVALUATION

14.1. Histology

Tissues in Terminal Procedures section and Tissue Weighing, Collection, Processing and Evaluation (Attachment A) table from animals identified in the Terminal Procedures section will be embedded in paraffin, sectioned, mounted on glass slides, and stained with hematoxylin and eosin.

14.2. Microscopic Evaluation

Tissues as detailed in the Terminal Procedures section and Tissue Weighing, Collection, Processing and Evaluation (Attachment A) table will be evaluated histopathologically by a veterinary pathologist with training and experience in laboratory animal pathology.

Special stains may be used at the discretion of the pathologist to further characterize lesions and changes identified during routine evaluation of individual animals. Any special stains will be documented in the individual animal data. Any additional stains or evaluations, if deemed necessary by the pathologist, may be added by study plan amendment following discussion with the Study Director and in consultation the Sponsor. Efforts will be made to evaluate all study plan-required tissues microscopically; however, it is not always feasible for every study plan-required tissue to be present on every slide. Study plan-required tissues that are not examined will be documented in the histopathology data and the impact of these missing tissues on the study will be documented in the pathology report.

Images may be generated for illustration of or consultation on histological observations. These images will not be used for data generation or interpretation, and will not be archived or included in the Final Report.

15. STATISTICAL ANALYSIS

Any data collected during the prestudy period will be tabulated, summarized or statistically analyzed. All statistical analyses will be performed within the respective study phase, unless otherwise noted. Numerical data collected on scheduled occasions will be summarized and statistically analyzed as indicated below according to sex and occasion.

15.1. Constructed Variables

Body Weight Changes:	Calculated between each scheduled interval as well as between the following intervals: from beginning to end of each phase
Organ Weight Relative to Body Weight:	Calculated against the terminal body weight for scheduled intervals
Organ Weight Relative to Brain Weight:	Calculated against the brain weight for scheduled intervals

Additional or alternative body weight or food consumption intervals may be evaluated to elucidate study results at the discretion of the Study Director.

15.2. Descriptive Statistical Analyses

Means, standard deviations (or % coefficient of variation or standard error, when deemed appropriate), ratios, percentages, numbers, and/or incidences will be reported as appropriate by dataset.

15.3. Inferential Statistical Methods

All statistical tests will be conducted at the 5% significance level. All pairwise comparisons will be conducted using two sided tests and will be reported at the 1% and 5% levels, unless otherwise noted.

The pairwise comparisons of interest are listed below:

Group 2 vs. Group 1

Group 3 vs. Group 1

Group 4 vs. Group 1

Analyses will be performed according to the matrix below when possible, but will exclude any group with less than 3 observations.

Statistical Matrix

Variables for Inferential Analysis	Statistical Method
	Parametric/ Non-parametric
Body Weight	X
Body Weight Changes	X
Hematology Variables	X
Coagulation Variables	X
Clinical Chemistry Variables	X
Urinalysis Variables	X
Organ Weights	X
Organ Weight relative to Body Weight	X
Organ Weight relative to Brain Weight	X

15.4. Parametric/Non-parametric

Levene's test will be used to assess the homogeneity of group variances.

The groups will be compared using an overall one-way ANOVA F-test if Levene's test is not significant or the Kruskal-Wallis test if it is significant. If the overall F-test or Kruskal-Wallis test is found to be significant, then pairwise comparisons will be conducted using Dunnett's or Dunn's test, respectively. Datasets with two groups will be compared using a Dunnett's test (equivalent to t-test in Nevis 2012 tables) or Dunn's test (equivalent to Wilcoxon Rank-Sum test in Nevis 2012 tables).

16. COMPUTERIZED SYSTEMS

The following critical computerized systems may be used in the study. The actual computerized systems will be documented in the study data.

Critical Computerized Systems

System Name	Description of Data Collected and/or Analyzed
Provantis®	Test material receipt, accountability, formulation activities, in-life (e.g., clinical observations, body weights, food consumption), clinical pathology (clinical chemistry, coagulation, hematology, urinalysis), and/or postmortem (e.g., pathology)
Deviation Information Library	Deviations
Share Document Management System	Reporting
DocuSign™	Collection of 21 CFR Part 11 compliant signature
Provantis® and/or SRS (CR-SEN in-house application built with SAS) and/or in-house reporting software Nevis 2012 (using SAS)	Statistical Analysis of in-life (body weights, food consumption); clinical pathology (clinical chemistry, coagulation, hematology, urinalysis); and postmortem (organ weights)
Mesa Laboratories AmegaView CMS	Continuous Monitoring System. Monitoring of standalone fridges, freezers, incubators, and selected laboratories to measure temperature, relative humidity, and CO_2, as appropriate
Johnson Controls Metasys	Building Automation System. Control of HVAC and other building systems, as well as temperature/humidity control and trending in selected laboratories and animal rooms
Phoenix	Computation of non-compartmental analysis, descriptive statistics and ratios, as well as graphical and tabular output

Data for parameters not required by the Study Plan, which are automatically generated by analytical devices used will be retained on file but not reported. Statistical analysis results that are generated by the program but are not required by the Study Plan and/or are not scientifically relevant will be retained on file but will not be included in the tabulations.

17. REGULATORY COMPLIANCE

The study will be performed in accordance with the OECD Principles of Good Laboratory Practice and as accepted by Regulatory Authorities throughout the European Union, United States of America (FDA), Japan (MHLW), and other countries that are signatories to the OECD Mutual Acceptance of Data Agreement.

Exceptions to GLPs include the following study elements:

- Characterization of the test and reference items will be/were performed by the Sponsor or Sponsor subcontractor according to established SOPs, controls, and approved test methodologies to ensure integrity and validity of the results generated; these analyses will not be/were not conducted in compliance with the GLP or GMP regulations.
- Stability testing of the supplied test and reference items will be/was performed by the Sponsor or Sponsor subcontractor according to established SOPs, controls, and approved test methodologies to ensure integrity and validity of the results generated; these analyses will not be/were not conducted in compliance with the GLP or GMP regulations.
- Concentration, stability, and homogeneity of the test and reference item formulations will not be determined in this study.
- Bioanalysis, gene expression, and splenocyte analysis will not be conducted in compliance with GLP regulations.

18. QUALITY ASSURANCE

18.1. Test Facility

The Test Facility Quality Assurance Program (QAP) will monitor the study to assure the facilities, equipment, personnel, methods, practices, records, and controls are in conformance with Good Laboratory Practice regulations. The QAP will review the Study Plan, conduct inspections at intervals adequate to assure the integrity of the study, and audit the Final Report to assure that it accurately describes the methods and standard operating procedures and that the reported results accurately reflect the raw data of the study.

18.2. Test Site(s)/Subcontractor(s)

For all study phase(s) inspected by Test Site/subcontractor QAP(s), copies of each periodic inspection report will be made available to the Study Director, Test Facility Management, and the Test Facility QAP.

19. AMENDMENTS AND DEVIATIONS

Changes to the approved Study Plan shall be made in the form of an amendment, which will be signed and dated by the Study Director. Every reasonable effort will be made to discuss any necessary study plan changes in advance with the Sponsor. The Study Director will notify the Sponsor of deviations that may result in a significant impact on the study as soon as possible.

20. RETENTION AND DISPOSITION OF RECORDS, SAMPLES, AND SPECIMENS

All study-specific raw data, electronic data, documentation, Study Plan (and amendments, if any), retained samples and specimens, and interim (if applicable) and final reports will be archived by no later than the date of Final Report issue. All materials generated by Charles River from this study will be transferred to a Charles River archive. At least 1 year after issue of the Draft Report, the Sponsor will be contacted.

Disposition of residual/retained analytical samples will be as described in the table below.

Disposition of Residual/Retained Samples

Sample Type	Disposition	Schedule
Bioanalytical	Archived by the Sponsor	Samples will be archived by the Sponsor.

Records to be maintained will include, but will not be limited to, documentation and data for the following:

- Deviations, study plan, and study plan amendments
- Study schedule
- Study-related correspondence
- Test system receipt, health, and husbandry
- Test and reference item receipt, identification, preparation, and analysis
- In-life measurements and observations
- Clinical pathology sample collection and evaluation
- Bioanalytical sample collection and evaluation
- Gross and microscopic observations and related data
- Organ weight measurements
- Statistical analysis results

21. STUDY CLASSIFICATION

Study Category:	Toxicology
Study Type:	Single Dose Toxicity
Study Design:	Parallel
Primary Treatment CAS Registry Number:	Not Available
Primary Treatment Unique Ingredient ID:	Not Available
Class of Compound:	AAV9 Vector
Administration Dose Form:	Solution

22. REPORTING

A comprehensive Draft Report will be prepared following completion of the study and will be finalized following consultation with the Sponsor. The report will include all information necessary to provide a complete and accurate description of the experimental methods and results and any circumstances that may have affected the quality or integrity of the study.

The Sponsor will receive an electronic version of the Draft and Final Report provided in Adobe Acrobat PDF format (hyperlinked and searchable) along with a Microsoft Word version of the text. The PDF document will be created from native electronic files to the extent possible, including text and tables generated by the Test Facility. Report components not available in native electronic files and/or original signature pages will be scanned and converted to PDF image files for incorporation.

Reports should be finalized within 6 months of issue of the Draft Report. If the Sponsor has not provided comments to the report within 6 months of draft issue, the report will be finalized by the Test Facility unless other arrangements are made by the Sponsor.

22.1. SEND Datasets

At the request of the Sponsor, SEND datasets will be generated and provided outside the context of the Final GLP Report. These datasets will not be subject to QA Audit nor will they be used as the basis for the Study Director interpretation of the study results. SEND datasets will be provided for the Final Report based on regulatory submission date. The Sponsor is expected to provide a submission date.

23. JUSTIFICATIONS AND GUIDELINES

23.1. Justification of Test System and Number of Animals

At this time, studies in laboratory animals provide the best available basis for extrapolation to humans and are required to support regulatory submissions. Acceptable models that do not use live animals currently do not exist.

The Sprague Dawley rat was chosen as the animal model for this study as it is an accepted rodent species for nonclinical toxicity testing by regulatory agencies.

The total number of animals to be used in this study is considered to be the minimum required to properly characterize the effects of the test item. This study has been designed such that it does not require an unnecessary number of animals to accomplish its objectives.

23.2. Justification of Route and Dose Levels

The intrathecal injection route of exposure was selected because this is the intended route of human exposure.

The dose levels were selected based on information provided by the Sponsors' prior studies with AAV9/AP4M1 in mice, as well as prior pharmacology for other similar AAV9 vectors in mice,

rats, pigs, and nonhuman primates. Based on those prior pharmacology studies, the chosen middle dose is anticipated to provide efficacy in ongoing mouse studies which should translate to humans, and the low dose may provide some minimal benefit. No clear dose-limiting toxicities were observed at doses equivalent to the middle dose in mice (2x below the middle dose and 2x above the middle dose). At one year postdose, 3 out of 10 mice receiving the high dose had hepatocellular adenoma, which could have been caused by the test item. The middle dose in this rat study corresponds to a nearly maximum feasible dose (MFD) due to limitations on injection volume and test item concentration, and this is the target dose for human translation. This is bracketed by a high dose using a higher injection volume, and a 3-fold lower dose. None of the doses are expected to generate more than a minimal to moderate toxic effect. If any adverse effects are observed, the range of doses in the study design is an attempt to produce a graded response.

23.3. Guidelines for Study

The design of this study was based on the study objective(s), the overall product development strategy for the test item, and the following study design guidelines:

- ICH Harmonised Tripartite Guideline M3 (R2). *Nonclinical Safety Studies for the Conduct of Human Clinical Trials and Marketing Authorization for Pharmaceuticals.*
- ICH Harmonised Tripartite Guideline S6 (R1). *Preclinical Safety Evaluation of Biotechnology-Derived Pharmaceuticals*.
- Japanese Guidelines for Nonclinical Studies of Drugs Manual (1995). *Guidelines for Toxicity Studies of Drugs (Chapter 2, Single Dose Toxicity Study).*

24. ANIMAL WELFARE

The Study Plan and any amendment(s) or procedures involving the care and use of animals in this study will be reviewed and approved by CR-SEN Institutional Animal Care and Use Committee (IACUC). During the study, the care and use of animals will be conducted with guidance from the USA National Research Council and the Canadian Council on Animal Care (CCAC).

25. REFERENCES

Office of Laboratory Animal Welfare. *Public Health Services Policy on Humane Care and Use of Laboratory Animals*. Bethesda, MD: National Institutes of Health. Current edition.

Appendix 1

TEST FACILITY APPROVAL

The signature below indicates that Test Facility Management approves the Study Director identified in this study plan and management's responsibility to the study as defined by the relevant GLP regulations.

DocuSigned by:

Signer Name: Julie Douville
Signing Reason: I approve this document
Signing Time: 09-Feb-2021 | 09:53:53 EST
B2DF78DE59474E8493719FA388399F90

Julie Douville, PhD
Test Facility Management

The signature below indicates that the Study Director approves the Study Plan.

DocuSigned by:

Stefania Cinquino

Signer Name: Stefania Cinquino
Signing Reason: I approve this document
Signing Time: 09-Feb-2021 | 11:56:40 EST
BA8C7FBD394B436288599953720340CE

Stefania Cinquino, BSc
Study Director

Appendix 1

SPONSOR APPROVAL

The Study Plan was approved by the Sponsor by e-mail on the date designated below. The correspondence giving approval will be archived, as appropriate with other Sponsor communications.

09 Feb 2021
Date of Sponsor Approval

Appendix 1

ATTACHMENT A

Tissue Weighing, Collection, Processing, and Evaluation Table

Organ	Weigh	Macroscopic Evaluation and Collection	Histology Processing	Microscopic Evaluation	Bioanalysis and Gene Expression	Remaining frozen tissue to archive
Animal ID	-	X	-	-	-	-
Artery, aorta	-	X	-	-	-	-
Body cavity, nasal	-	X	-	-	-	-
Bone marrow, sternum	-	X	-	-	-	-
Bone marrow smear	-	X[a]	-	-	-	-
Bone, femur, right	-	X	-	-	-	-
Bone, sternum	-	X	-	-	-	-
Brain	X	X	X	X (left)	X (right) (forebrain, midbrain, and hindbrain)	X (right) (forebrain, midbrain, and hindbrain)
Epididymis	X (2)	X (2)	X	X	-	-
Esophagus	-	X	-	-	-	-
Eye	-	X (2)	X (1) (left)	X (1) (left)	-	X (1) (right)
Ganglion, dorsal root, cervical, lumbar, and thoracic, with dorsal and ventral nerve roots [d,f]	-	X (2)	X (2)	X (2)	X (2)	X
Gland, adrenal	X (2)	X (2)	-	-	-	-
Gland, clitoral	-	X (2)	-	-	-	-
Gland, Harderian	-	X (2)	-	-	-	-
Gland, lacrimal	-	X (2) (extra-orbital)	-	-	-	-
Gland, mammary	-	X	-	-	-	-
Gland, parathyroid	-[b]	X (2)	-	-	-	-
Gland, pituitary	X	X	-	-	-	-
Gland, preputial	-	X (2)	-	-	-	-
Gland, prostate	X	X	-	-	-	-
Gland, salivary, submandibular	-	X (2)	-	-	-	-
Gland, salivary, sublingual	-	X (2)	-	-	-	-
Gland salivary, parotid	-	X (2)	-	-	-	-
Gland, seminal vesicle	-	X (2)	-	-	-	-
Gland, thyroid	X (2)	X (2)	-	-	-	-
Gland, Zymbal's	-	X (2)	-	-	-	-
Gut-associated lymphoid tissue[c]	-	X	-	-	-	-
Heart	X	X	X	X	X	X
Joint, femorotibial, right	-	X	-	-	-	-

Appendix 1

Organ	Weigh	Macroscopic Evaluation and Collection	Histology Processing	Microscopic Evaluation	Bioanalysis and Gene Expression	Remaining frozen tissue to archive
Kidney	X (2)	X (2)	X (left)	X (left)	X (right)	X (right)
Large intestine, cecum	-	X	-	-	-	-
Large intestine, colon	-	X	-	-	-	X
Large intestine, rectum	-	X	-	-	-	-
Larynx	-	X	-	-	-	-
Liver	X	X	X	X	X	X
Lung	-	X	X	X	-	X
Lymph node(s) draining administration site(s): deep cervical and iliac	-	X (2)	X (1)	X (1)	-	X (1)
Lymph node, mandibular	-	X (2)	X (1)	X (1)	-	X (1)
Lymph node, mesenteric	-	X	X	X	-	X (1)
Muscle, skeletal, gastrocnemius	-	X (2)	X (1)	X (1)	X (1)	X (1)
Muscle, skeletal, Biceps Femoris	-	X (2)	X (1)	X (1)	X (1)	X (right)
Nerve, optic	-	X (2)	X (left)	X (left)	-	X (right)
Nerve, sciatic	-	X (2)	X	X	X (1)	X (1)
Nerve, tibial	-	X (2)	X	X	-	X (right)
Ovary	X (2)	X (2)	X (1)	X (1)	X (1)	X (2)
Oviduct	-	X (2)	-	-	-	X
Pancreas	-	X	-	-	-	X
Skin	-	X	-	-	-	X
Small intestine, duodenum	-	X	-	-	-	X
Small intestine, ileum	-	X	-	-	-	X
Small intestine, jejunum	-	X	-	-	-	X
Spinal cord; cervical, thoracic, and lumbar and injection site [d,e]	-	X	X	X	X	X
Spleen	X	X	X	X	X	X
Stomach	-	X	-	-		X (Right)
Testis	X (2)	X (2)	X (Left)	X (Left)	X (Right)	X
Thymus	X	X	X	X	X	X
Tongue	-	X	-	-	-	-
Trachea	-	X	-	-	-	-
Ureter	-	X (2)	-	-	-	X
Urinary bladder	-	X	-	-	-	X
Uterus/Cervix	X	X	-	-	-	-
Vagina	-	X	-	-	-	-

Appendix 1

Organ	Weigh	Macroscopic Evaluation and Collection	Histology Processing	Microscopic Evaluation	Bioanalysis and Gene Expression	Remaining frozen tissue to archive

X = Procedure to be conducted. - = Not applicable. (1) = one side. (2) = both sides.
Macroscopic abnormalities in the organs listed and in other organs will be sampled at necropsy, processed for histology and examined microscopically.

[a] Two bone marrow smears will be collected from the femur at scheduled and unscheduled necropsies (for possible examination). Smears will not be collected from animals that are found dead or from animals that were euthanized moribund and then stored in the refrigerator prior to necropsy. Bone marrow smears are allowed to air dry and are not fixed in formalin.
[b] Weigh with gland, thyroid.
[c] From small intestine: Peyer's patch or solitary lymphoid follicle.
[d] In situ, decalcified before sectioning
[e] Transverse and oblique sections
[f] Nerve roots will be examined if present in section of dorsal root ganglia.

Appendix 1

ATTACHMENT B

Shipment of Samples and Study Records

Matrix	Purpose	Day/ Week/ Aliquot	Proposed Shipment Date	Conditions for Shipment	Recipient/Address
Unused Sponsor-suppl ied bulk test materials	Return to sponsor	Not Applicable	Following the end of dosing	Dry ice	Attention to:<br>Steven Gray, PhD<br>University of Texas Southwestern Medical Center<br>NA2.508<br>5323 Harry Hines Blvd<br>University of Texas Southwestern Medical Center<br>Dallas, TX 75390<br>Tel: 214-648-0670<br>E-mail:<br>steven.gray@UTSouthwestern.edu
Serum, CSF, splenocytes, and frozen tissue	Bioanalysis	X	Within 2 weeks from collection	Dry ice	Steven Gray, PhD<br>University of Texas Southwestern Medical Center<br>NA02.508<br>5323 Harry Hines Blvd<br>University of Texas Southwestern Medical Center<br>Dallas, TX 75390<br>Tel: 214-648-0670<br>E-mail:<br>steven.gray@UTsouthwestern.edu

Alternate shipping details may be provided thereafter and will be documented in study correspondence.

Appendix 2

VIRALGEN

Certificate Of Analysis

Research grade - Not for human use.

Client identification: CURE SPG50

Product Name and type of product: rAAV9, sterile suspension of rAAV9 vector carrying the AP4M1 therapeutic gene.

Batch Number: T-GEMINIS-033

Quality grade: Research grade

Production process: 50 Liters batch

Manufacturing date: 22 December 2020

Formulation buffer (FMR-T-0043): MilliQ Water, 1X dPBS, 5% D-Sorbitol, 0.001% Pluronic

Vials Shipped: 34 x 0.5 ml vialed in polypropylene Cryotubes

Shipment Date: 19 January 2021

Transport conditions: Dry ice

Storage conditions: ≤-60ºC

For Research Use Only

Performed by:	Paz López (QC Coordinator)	Date: 29- APRIL - 2021
Reviewed by	Sandy Douthe (QC Manager)	Date: 29 APRIL 2021
Reviewed by	Begoña Ortega (QA Technician)	Date: 29 - APRIL 2021

Parque Tecnológico de Guipuzcoa - Paseo Mikeletegui, 83, 2ª Planta
20009 San Sebastián, Spain - Tel: +34 943 477 733 - viralgenvc.com

Appendix 2

rAAV Purified Bulk / Bulk Drug Substance assays:

ASSAY	METHOD	TEST SITE	SPECIFICATION	RESULT
**SAFETY ASSAYS**				
Replication competent AAV	PT/133 & PT/025 Infection of permissive cell line/Rep2 qPCR (based on ITRqPCR titration)	Genosafe	Report result	Not Detected <10 rcAAV in 1x10^{11} vg
**STRENGHT ASSAYS**				
Vector genome titer (vg/mL)	PNT-CC-005 ITR qPCR	Viralgen	report results	1.71x10^{14}
Vector genome titer (vg/ml)	PNT-CC-049 ITRddPCR	Viralgen	report results	5.17x10^{13}
**PURITY ASSAYS**				
General Purity	PNT-CC-012 SDS-Page/ silver stain	Viralgen	Report result	Detection VP1, VP2 and VP3. Presence of extra bands between 150-250 KDa
Residual Host Cell Protein (ng/mL)	PNT-CC-023 HEK293 ELISA Assay	Viralgen	Report result	<100
Residual Host Cell DNA (pg/mL with and without DNase)	PNT-CC-033 18S qPCR (123 and 254 bp amplicons)	Viralgen	Report result	123 bp: 6.86x10^{6} (+DNase I) 7.38x10^{6} (-DNase I) 254 bp: 5.02x10^{6} (+DNAseI) 5.21x10^{6} (-DNase I)
Residual Host Cell DNA (pg/mL, without DNase)	SP-M.8303 E1A qPCR	SGS-Vitrology	Report result	2.6x10^{6}
Residual Plasmid DNA (copies/mL with and without DNase)	PNT-CC-014 KanaR qPCR	Viralgen	Report result	9.61x10^{11} (+Dnase I) 1.26x10^{12} (-Dnase I)
Full/Empty particles ratio	V6725 CryoTEM	Vironova	>50% full	Filled: 84% Empty: 9% Uncertain: 7%

For Research Use Only

Parque Tecnológico de Guipuzcoa - Paseo Mikeletegui, 83, 2ª Planta
20009 San Sebastián, Spain - Tel: +34 943 477 733 - viralgenvc.com

Appendix 2

VIRALGEN

Product: rAAV9-AP4M1
Batch Number: T-GEMINIS-033

ASSAY	METHOD	TEST SITE	SPECIFICATION	RESULT
**PURITY ASSAYS**				
Aggregation	V0149 & V0692 nsTEM	Vironova	Report result	92% of the individual particles at <40nm
Residual Chemical (Transfection reagent)	Refer to DMF	Docuchem	Report result	<25.13 ppm >12.57 ppm
Residual Chemical (Lysis reagent)	Refer to DMF		Report result	<LOD (1 ppm)
Residual Chemical (Clarifying reagent)	Refer to DMF		Report result	<1.03 ppm >0.21 ppm
Residual Chemical (Iodixanol)	HPLC		Report result	<3.12 ppm >1.04 ppm
Residual Chemical (Antifoam)	Refer to DMF		Report result	<LOD (5 ppm)
Residual Affinity Ligand (ng/mL)	PNT-CC-037 ELISA	Viralgen	Report result	511.3
**IDENTITY ASSAYS**				
Protein Identity	PNT-CC-003 SDS-PAGE/ Western Blot	Viralgen	Detection of VP1, VP2 & VP3	Detection of VP1, VP2 and VP3
Genome identity	PNT-143 Sequencing (Sanger)	Secugen	100% conform to sequence of reference	Conform

Parque Tecnológico de Guipuzcoa - Paseo Mikeletegui, 83, 2ª Planta
20009 San Sebastián, Spain - Tel: +34 943 477 733 - viralgenvc.com

For Research Use Only

Appendix 2

VIRALGEN

Product: rAAV9-AP4M1
Batch Number: T-GEMINIS-033

rAAV Final Product / Drug Product assays:

ASSAY	METHOD	TEST SITE	SPECIFICATION	RESULT
**SAFETY ASSAYS**				
Sterility	LTMI-009	Biolab S.L	No growth	No growth
Endotoxin (EU/ml)	PNT-CC-015 Kinetic chromogenic	Viralgen	<0.2	<0.051
[1]Mycoplasma	PNT-CC-001 PCR end point	Viralgen	Negative	Negative
**STRENGHT ASSAYS**				
Vector genome titer (vg/mL)	PNT-CC-005 ITR qPCR	Viralgen	$0.75x10^{14}$-$4x10^{14}$ Target $1.70x10^{14}$	$2.17x10^{14}$
Vector genome titer (vg/ml)	PNT-CC-049 ITRddPCR	Viralgen	Report Result	$5.43x10^{13}$
Infectious titer (TCID50/mL)	PNT-CC-004 TCID50/ITRqPCR	Viralgen	Report result	$1.02x10^{10}$
vg/TCID50 (ITRqPCR) ratio	N/A	Viralgen	Report result	21237.16
**QUALITY ASSAYS**				
Osmolality (mOsm/Kg)	PNT-CC-016 Freezing point	Viralgen	587 +/-50	570
pH	PNT-CC-044 Potentiometry	Viralgen	7.4±0.4	7.21
Appearence	PNT-CC-017 Visual inspection	Viralgen	Colorless, clear to slightly opalescent, free of visible particles	Not done because vialed in polypropylene cryovials
Particles size distribution	PNT-CC-053 DLS	Viralgen	Report Results	99-99.5% of particulate volume between 23.98-25.25 nm in mean diameter

[1] Mycoplasma assay performed in the transfection pool.

For Research Use

Parque Tecnológico de Guipuzcoa - Paseo Mikeletegui, 83, 2ª Planta
20009 San Sebastián, Spain - Tel: +34 943 477 733 - viralgenvc.com

Appendix 2

VIRALGEN

Product: rAAV9-AP4M1
Batch Number: T-GEMINIS-033

1. <u>Test Information</u>: **General purity and protein identity by SDS-PAGE/Silver staining and Western Blot analysis**

According to the standard operating procedure PNT-CC-012_Pureza del vector por SDS_PAGE_TINCION DE PLATA, and the PNT-CC-003_Identificación de proteínas por Western Blot.

1.1 Gel Properties: 10% Acrylamide gel

1.2. SDS-PAGE Results:

SDS-PAGE/Silver staining
$5x10^9$ vg/well (based on ITRqPCR titer result)

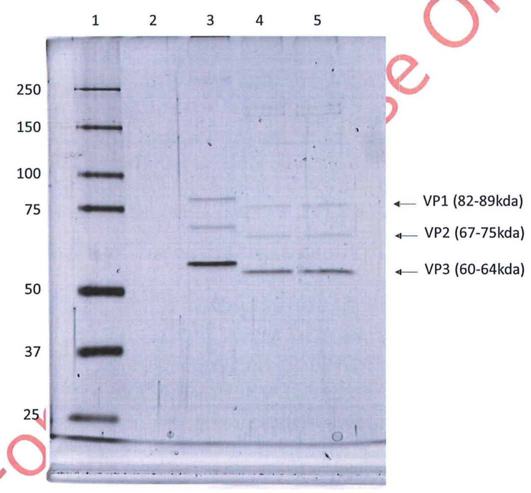

Lane 1: Ladder
Lane 2: Negative Control. Formulation Buffer FMR-T-0043
Lane 3: rAAV8 Standard vector ATCC VR-1816
Lane 4: T-GEMINIS-033 BDS
Lane 5: T-GEMINIS-033 BDS

Presence of extra-bands between 150-250 KDa. An investigational analysis was done and the extra bands were identified as encapsidated (or not accessible to DNase) DNA.

Parque Tecnológico de Guipuzcoa - Paseo Mikeletegui, 83, 2ª Planta
20009 San Sebastián, Spain - Tel: +34 943 477 733 - viralgenvc.com

Appendix 2

1.3. SDS-PAGE/Western blot Results:

SDS-Page/Western Blot Results
5x10^9 vg/well (based on ITRqPCR titer result)

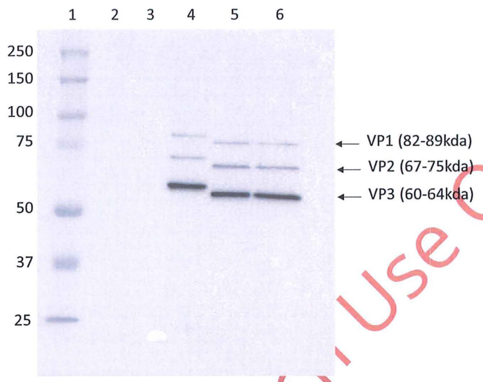

Lane 1: Ladder.
Lane 2: Empty
Lane 3: Negative Control. Formulation buffer FMT-T-0043
Lane 4: rAAV8 Standard vector ATCC VR-1816
Lane 5: T-GEMINIS-033 BDS
Lane 6: T-GEMINIS-033 BDS

For Research Use Only

Parque Tecnológico de Guipuzcoa - Paseo Mikeletegui, 83, 2ª Planta
20009 San Sebastián, Spain - Tel: +34 943 477 733 - viralgenvc.com

Appendix 2

Certificate Of Analysis

Research grade - Not for human use

FOR RESEARCH USE ONLY

Name and type of product: FMR-T-0043/ Formulation buffer

Batch Number:2021-02-09/01

Date of manufacture: 9Th February 2021 (vialing date).

Composition:1xdPBS,5% sorbitol and 0.001% pluronic

Quality grade: Research grade

Primary container: Polypropilene Cryotubes 1,2 ml

Batch size: 30 vials containing 0,5 ml of buffer

Name and address of the manufacturer:

VIRALGEN - Parque Tecnológico de Gipuzkoa - Paseo Mikeletegi 83, 2ª Planta.

CP20009 San Sebastián, SPAIN. Tel: +34 943 477 733.

Appendix 2

Product Name: FMR-T-0043

Batch Number:2021-02-09/01

VIRALGEN

TEST	METHOD	TEST SITE	RESULT
(1)Bioburden	PNT-CC-026 Filtration	Viralgen	<1CFU/10 ml
(1)Endotoxins (EU/mL)	PNT-CC-015 Kinetic chromogenic	Viralgen	<0.050
(2)pH	Potenciometry	Viralgen	7.35
(2)Conductivity (mS/cm)	Conductimetry	Viralgen	14.08

(1) Assays done at Viralgen Vector Core Quality Control laboratories

(2) Assays done at the time of buffer preparation by the downstream Process team following the formulation record procedure described on FMR-T-0043

Performed by: Paz López (QC Deputy Director) **Date:** 05th January 2022

PLM

FOR RESEARCH USE ONLY

Parque Tecnológico de Gipuzkoa. Paseo Mikeletegi 83, 2ª Planta. CP20009 San Sebastián, SPAIN. Tel: +34 943 477 733
viralgenvc.com

Individual Mortality Explanation Page

Abbreviation	Description	Abbreviation	Description
-	Not scheduled to be performed / Not seen	Path	Pathology
AM SIR	Signs of ill health or reaction to treatment check in the morning	PM SIR	Signs of ill health or reaction to treatment check in the afternoon
NR	Not recorded		

Note: This is a comprehensive list of abbreviations. All of the abbreviations listed may not be applicable to this report.

Dosing Information

Dosing information is abbreviated on various data outputs; the following represents the dosing information for this study.

Group No.	Test Material	Dose Level (vg)
1	Reference Item	0
2	AAV9/AP4M1	0.36×10^{12}
3	AAV9/AP4M1	1.1×10^{12}
4	AAV9/AP4M1	3.3×10^{12}

Individual Clinical Observations Explanation Page

Abbreviation	Description	Abbreviation	Description
./-	Not scheduled to be performed / Not seen / Dead	PAM	Detailed examination in the afternoon
AM_S	Signs of ill health or reaction to treatment check in the morning	Part	Particles
CAM	Cage side observation in the morning	PM_S	Signs of ill health or reaction to treatment check in the afternoon
Cp #	Cage side observation post dose	pr #/PR #/Pre	Observation predose
CPM	Cage side observation in the afternoon	(PT)	Permanent
Cpr	Cage side observation predose	Sev Not App	Severity not applicable
CSO	Cage side observation	Sev Not Rec	Severity not recorded
DAM	Detailed examination in the morning	SIRT	Signs of ill health or reaction to treatment
DE/D	Detailed examination	U #/Up #	Unscheduled observation post dose
DuRx	Observation during dosing	UDu #	Unscheduled observation during dosing
DW	Detailed examination weekly	Un #/Unsc #	Unscheduled observation
Fev	Food evaluation	Upr #	Unscheduled observation predose
Inj	Injection	Vet	Anything observed by veterinary staff
Inter	Internal	(VET)	Symptom recorded by veterinary staff
OTHR	Other	w/	with
p #/ P #	Observation post dose	#	Number to avoid using the same timeslot/animal/day

Note: This is a comprehensive list of abbreviations. All of the abbreviations listed may not be applicable to this report.

Note: Only animals with findings are presented in this appendix.

Dosing Information

Dosing information is abbreviated on various data outputs; the following represents the dosing information for this study.

Group No.	Test Material	Dose Level (vg)
1	Reference Item	0
2	AAV9/AP4M1	0.36×10^{12}
3	AAV9/AP4M1	1.1×10^{12}
4	AAV9/AP4M1	3.3×10^{12}

Individual Palpable Masses Explanation Page

Abbreviation	Description
NR	Not recorded

Note: This is a comprehensive list of abbreviations. All of the abbreviations listed may not be applicable to this report.

Note: Only animals with findings are presented in this appendix.

Dosing Information

Dosing information is abbreviated on various data outputs; the following represents the dosing information for this study.

Group No.	Test Material	Dose Level (vg)
1	Reference Item	0
2	AAV9/AP4M1	0.36×10^{12}
3	AAV9/AP4M1	1.1×10^{12}
4	AAV9/AP4M1	3.3×10^{12}

Individual Body Weights Explanation Page

Abbreviation	Description	Abbreviation	Description
./-	Not scheduled to be performed/dead	OA	Omitted activity
< or >	Out of range	RC	Result comment
FC	Flag comment	TERR	Technical error
NT	Not taken	UPTD	Unable to perform due to technical difficulty

Note: This is a comprehensive list of abbreviations. All of the abbreviations listed may not be applicable to this report.

Dosing Information

Dosing information is abbreviated on various data outputs; the following represents the dosing information for this study.

Group No.	Test Material	Dose Level (vg)
1	Reference Item	0
2	AAV9/AP4M1	0.36×10^{12}
3	AAV9/AP4M1	1.1×10^{12}
4	AAV9/AP4M1	3.3×10^{12}

Individual Body Weight Gains Explanation Page

Abbreviation	Description	Abbreviation	Description
./-	Not scheduled to be performed/dead	RC	Result comment
< or >	Out of range	TERR	Technical error
NC	Not calculable	UPTD	Unable to perform due to technical difficulty

Note: This is a comprehensive list of abbreviations. All of the abbreviations listed may not be applicable to this report.

Dosing Information

Dosing information is abbreviated on various data outputs; the following represents the dosing information for this study.

Group No.	Test Material	Dose Level (vg)
1	Reference Item	0
2	AAV9/AP4M1	0.36×10^{12}
3	AAV9/AP4M1	1.1×10^{12}
4	AAV9/AP4M1	3.3×10^{12}

Individual Food Consumption Explanation Page

Abbreviation	Description	Abbreviation	Description
-	Dead / Not scheduled to be performed / See Marker Information	OA	Omitted activity
ANH	Animal found with no hopper during measurement interval	POWF	Powdered food
ANIC	Animal not in cage or in incorrect cage during measurement	RC	Result comment
ANW	Animal found with no water access during measurement intervals	REHO	Animal rehoused during measurement interval
AV	Aberrant value	REPL	Animal replaced during measurement interval
Cons	Consumption	SPIL	Spilled food (by animal)
FC	Flag comment	TERR	Technical error
FSNC	Food supplementation given during interval, value not calculable	UPTD	Unable to perform due to technical difficulty
NC	Not calculable	WETF	Wet or contaminated food (in container)

Note: This is a comprehensive list of abbreviations. All of the abbreviations listed may not be applicable to this report.

Cage Assignment

Cage Number	Animal Numbers	Cage Number	Animal Numbers
1001	1001, 1002	1003	1003, 1004
1005	1005, 1006, 1007	1008	1008, 1009, 1010
1011	1011, 1012	1013	[1013], 1014, 1015
1501	1501, 1502	1503	1503, 1504
1505	1505, 1506, 1507	1508	1508, 1509
1510	1510, 1511, 1512	1513	1513, 1514, 1515
2001	2001, 2002	2003	[2003], {2004}, [2004]
2005	2005, 2006, 2007	2009	2009, 2010
2011	2011, 2012, 2013	2014	2014, 2015
2103	2103, 2008	2501	2501, 2502
2503	2503, 2504	2505	2505, 2506
2507	2507, 2508, 2509	2510	2510, 2511, 2512
2613	2613, 2614, 2615	3001	3001, 3002, 3003
3004	3004, 3005	3006	3006, 3007, 3008
3009	3009, 3010, 3011	3012	3012, 3013
3014	3014, 3015	3501	3501, 3502, 3503
3504	3504, 3505, 3506	3507	3507, 3508
3509	3509, 3510	3511	3511, 3512, 3513
3514	3514, {3615}, [3515]	4001	4001, 4002
4003	4003, 4004, 4005	4006	4006, 4007, 4008
4009	4009, 4010, 4011	4012	4012, 4013
4014	4014, 4015	4501	{4502}, [4502], [4501]
4503	4503, 4504	4505	4505, 4506, 4507
4509	4509, 4510	4511	4511, 4512
4513	4513, 4514, 4515	4601	4601, 4508

[]=Animal exited cage during results period
{}=Animal entered cage during results period

Dosing Information

Dosing information is abbreviated on various data outputs; the following represents the dosing information for this study.

Group No.	Test Material	Dose Level (vg)
1	Reference Item	0
2	AAV9/AP4M1	0.36×10^{12}
3	AAV9/AP4M1	1.1×10^{12}
4	AAV9/AP4M1	3.3×10^{12}

Individual Neurobehavorial Evaluation Explanation Page

Abbreviation	Description	Abbreviation	Description
. / NSCH	Not scheduled to be performed	OF	Open field
AV/ AVS	Suspected aberrant value	P#	Postdose
E/ Exc	Excluded from mean	Pre	Predose
FC	Flag Comment	RC	Result comment
Grp	Grip	Refl	Reflex
HC	Home cage	Resp	Response
I/Incl	Include	RSV	Refer to source data
N+ve	Number positive	T#	Trial
NR	Not recorded	TERR	Technical error
OA	Omitted activity	TNR	Test not reported

Note: This is a comprehensive list of abbreviations. All of the abbreviations listed may not be applicable to this report.

Dosing Information

Dosing information is abbreviated on various data outputs; the following represents the dosing information for this study.

Group No.	Test Material	Dose Level (vg)
1	Reference Item	0
2	AAV9/AP4M1	0.36×10^{12}
3	AAV9/AP4M1	1.1×10^{12}
4	AAV9/AP4M1	3.3×10^{12}

Appendix 3

Individual Mortality

5550008

Sex: Male Day(s): - Relative to Start Date

0<br>vg<br>Group 1	Day of Death	Removal Date	Path Removal Reason
1001	8	23-Feb-2021	TERM
1002	8	23-Feb-2021	TERM
1003	29	16-Mar-2021	REC
1004	29	16-Mar-2021	REC
1005	91	18-May-2021	REC
1006	91	18-May-2021	REC
1007	91	18-May-2021	REC
1008	29	19-Mar-2021	REC
1009	29	19-Mar-2021	REC
1010	29	19-Mar-2021	REC
1011	91	20-May-2021	REC
1012	91	20-May-2021	REC
1013	7	28-Feb-2021	UNSC
1014	8	01-Mar-2021	TERM
1015	8	01-Mar-2021	TERM

TE/TERM = Terminal Euthanasia FD = Found Dead UE/UNSC = Unscheduled Euthanasia
REL = Released
AD/ACCD = Accidental death
IE/INTM = Interim Euthanasia REC = Recovery Euthanasia

Appendix 3

Individual Mortality

5550008

Sex: Male Day(s): - Relative to Start Date

0.36x 10E12 vg Group 2	Day of Death	Removal Date	Path Removal Reason
2001	29	16-Mar-2021	REC
2002	29	16-Mar-2021	REC
2103	91	20-May-2021	REC
2004	8	23-Feb-2021	TERM
2005	91	18-May-2021	REC
2006	91	18-May-2021	REC
2007	91	18-May-2021	REC
2008	91	20-May-2021	REC
2009	8	26-Feb-2021	TERM
2010	8	26-Feb-2021	TERM
2011	29	19-Mar-2021	REC
2012	29	19-Mar-2021	REC
2013	29	19-Mar-2021	REC
2014	8	01-Mar-2021	TERM
2015	8	01-Mar-2021	TERM

TE/TERM = Terminal Euthanasia FD = Found Dead UE/UNSC = Unscheduled Euthanasia
REL = Released
AD/ACCD = Accidental death
IE/INTM = Interim Euthanasia REC = Recovery Euthanasia

Appendix 3

Individual Mortality

5550008

Sex: Male Day(s): - Relative to Start Date

1.1x 10E12 vg Group 3	Day of Death	Removal Date	Path Removal Reason
3001	8	23-Feb-2021	TERM
3002	8	23-Feb-2021	TERM
3003	8	23-Feb-2021	TERM
3004	29	17-Mar-2021	REC
3005	29	17-Mar-2021	REC
3006	91	18-May-2021	REC
3007	91	18-May-2021	REC
3008	91	18-May-2021	REC
3009	29	19-Mar-2021	REC
3010	29	19-Mar-2021	REC
3011	29	19-Mar-2021	REC
3012	8	01-Mar-2021	TERM
3013	8	01-Mar-2021	TERM
3014	91	23-May-2021	REC
3015	91	23-May-2021	REC

TE/TERM = Terminal Euthanasia FD = Found Dead UE/UNSC = Unscheduled Euthanasia
REL = Released
AD/ACCD = Accidental death
IE/INTM = Interim Euthanasia REC = Recovery Euthanasia

Appendix 3

Individual Mortality

5550008

Sex: Male Day(s): - Relative to Start Date

3.3x 10E12 vg Group 4	Day of Death	Removal Date	Path Removal Reason
4001	8	23-Feb-2021	TERM
4002	8	23-Feb-2021	TERM
4003	29	16-Mar-2021	REC
4004	29	16-Mar-2021	REC
4005	29	16-Mar-2021	REC
4006	8	24-Feb-2021	TERM
4007	8	24-Feb-2021	TERM
4008	8	24-Feb-2021	TERM
4009	91	20-May-2021	REC
4010	91	20-May-2021	REC
4011	91	20-May-2021	REC
4012	29	22-Mar-2021	REC
4013	29	22-Mar-2021	REC
4014	91	23-May-2021	REC
4015	91	23-May-2021	REC

TE/TERM = Terminal Euthanasia FD = Found Dead UE/UNSC = Unscheduled Euthanasia
REL = Released
AD/ACCD = Accidental death
IE/INTM = Interim Euthanasia REC = Recovery Euthanasia

Appendix 3

Individual Mortality

5550008

Sex: Female Day(s): - Relative to Start Date

0 vg Group 1	Day of Death	Removal Date	Path Removal Reason
1501	8	23-Feb-2021	TERM
1502	8	23-Feb-2021	TERM
1503	91	17-May-2021	REC
1504	91	17-May-2021	REC
1505	8	24-Feb-2021	TERM
1506	8	24-Feb-2021	TERM
1507	8	24-Feb-2021	TERM
1508	29	19-Mar-2021	REC
1509	29	19-Mar-2021	REC
1510	91	20-May-2021	REC
1511	91	20-May-2021	REC
1512	91	20-May-2021	REC
1513	29	22-Mar-2021	REC
1514	29	22-Mar-2021	REC
1515	29	22-Mar-2021	REC

TE/TERM = Terminal Euthanasia FD = Found Dead UE/UNSC = Unscheduled Euthanasia
REL = Released
AD/ACCD = Accidental death
IE/INTM = Interim Euthanasia REC = Recovery Euthanasia

Appendix 3

Individual Mortality

5550008

Sex: Female Day(s): - Relative to Start Date

0.36x 10E12 vg Group 2	Day of Death	Removal Date	Path Removal Reason
2501	29	16-Mar-2021	REC
2502	29	16-Mar-2021	REC
2503	91	17-May-2021	REC
2504	91	17-May-2021	REC
2505	8	24-Feb-2021	TERM
2506	8	24-Feb-2021	TERM
2507	29	17-Mar-2021	REC
2508	29	17-Mar-2021	REC
2509	29	17-Mar-2021	REC
2510	8	26-Feb-2021	TERM
2511	8	26-Feb-2021	TERM
2512	8	26-Feb-2021	TERM
2613	91	23-May-2021	REC
2614	91	23-May-2021	REC
2615	91	23-May-2021	REC

TE/TERM = Terminal Euthanasia FD = Found Dead UE/UNSC = Unscheduled Euthanasia
REL = Released
AD/ACCD = Accidental death
IE/INTM = Interim Euthanasia REC = Recovery Euthanasia

Appendix 3

Individual Mortality

5550008

Sex: Female Day(s): - Relative to Start Date

1.1x 10E12 vg Group 3	Day of Death	Removal Date	Path Removal Reason
3501	91	17-May-2021	REC
3502	91	17-May-2021	REC
3503	91	17-May-2021	REC
3504	29	17-Mar-2021	REC
3505	29	17-Mar-2021	REC
3506	29	17-Mar-2021	REC
3507	8	26-Feb-2021	TERM
3508	8	26-Feb-2021	TERM
3509	91	20-May-2021	REC
3510	91	20-May-2021	REC
3511	8	01-Mar-2021	TERM
3512	8	01-Mar-2021	TERM
3513	8	01-Mar-2021	TERM
3514	29	22-Mar-2021	REC
3615	29	22-Mar-2021	REC

TE/TERM = Terminal Euthanasia FD = Found Dead UE/UNSC = Unscheduled Euthanasia
REL = Released
AD/ACCD = Accidental death
IE/INTM = Interim Euthanasia REC = Recovery Euthanasia

Appendix 3

Individual Mortality

5550008

Sex: Female Day(s): - Relative to Start Date

3.3x 10E12 vg Group 4	Day of Death	Removal Date	Path Removal Reason
4601	8	26-Feb-2021	TERM
4502	8	23-Feb-2021	TERM
4503	91	17-May-2021	REC
4504	91	17-May-2021	REC
4505	29	17-Mar-2021	REC
4506	29	17-Mar-2021	REC
4507	29	17-Mar-2021	REC
4508	8	26-Feb-2021	TERM
4509	8	26-Feb-2021	TERM
4510	8	26-Feb-2021	TERM
4511	29	22-Mar-2021	REC
4512	29	22-Mar-2021	REC
4513	91	23-May-2021	REC
4514	91	23-May-2021	REC
4515	91	23-May-2021	REC

TE/TERM = Terminal Euthanasia FD = Found Dead UE/UNSC = Unscheduled Euthanasia
REL = Released
AD/ACCD = Accidental death
IE/INTM = Interim Euthanasia REC = Recovery Euthanasia

Appendix 4

Individual Clinical Observations

5550008

0<br>vg<br>Group 1<br>Sex: Male	Observation Type: All Types	Day(s) Relative to Start Date<br>-12<br>Vet	 7<br>AM_S	 21<br>DE	 28<br>DE	 29<br>DE	 35<br>DE	 42<br>DE
1004	Fur, Thin Cover, Lumbar	.	.	X	X	X	.	.
1005	Fur, Thin Cover, Dorsal Thoracic	.	.	.	.	.	.	.
	Fur, Thin Cover, Lumbar	.	.	.	X	.	X	X
	Skin, Scab, Dorsal Thoracic	.	.	.	.	.	.	.
1006	Fur, Thin Cover, Lumbar	.	.	X	X	.	X	X
1007	Fur, Thin Cover, Forepaw, Left	.	.	.	.	.	.	.
	Fur, Thin Cover, Forepaw, Right	.	.	.	.	.	.	.
	Fur, Thin Cover, Lumbar	.	.	.	X	.	X	X
1008	Fur, Thin Cover, Lumbar	.	.	.	.	X	.	.
1011	Fur, Thin Cover, Dorsal Thoracic	.	.	.	.	.	.	X
	Fur, Thin Cover, Lumbar	.	.	.	.	.	.	.
1013	Limited Usage, Hindlimb, Left, Severe	.	X	.	.	.	.	.
	Limited Usage, Hindlimb, Right, Severe	.	X	.	.	.	.	.
	Limited Usage, Hindpaw, Left, Severe	.	X	.	.	.	.	.
	Limited Usage, Hindpaw, Right, Severe	.	X	.	.	.	.	.
	Breathing, Labored	.	X	.	.	.	.	.
	Prostrate	.	X	.	.	.	.	.
	Fur, Thin Cover, Dorsal Cervical	X	.	.	.	.	.	.
	Fur, Thin Cover, Interscapular	X	.	.	.	.	.	.
	Thin	.	X	.	.	.	.	.
	Weak	.	X	.	.	.	.	.
	Backbone Prominent	.	X	.	.	.	.	.
	Low Carriage	.	X	.	.	.	.	.
	Lying On Side	.	X	.	.	.	.	.
	Activity Decreased	.	X	.	.	.	.	.

X=Present

Appendix 4

Individual Clinical Observations

5550008

0<br>vg<br>Group 1<br>Sex: Male	Observation Type: All Types	Day(s) Relative to Start Date<br>49<br>DE	56<br>DE	63<br>DE	70<br>DE	77<br>DE	84<br>DE	91<br>DE
1004	Fur, Thin Cover, Lumbar	.	.	.	.	.	.	.
1005	Fur, Thin Cover, Dorsal Thoracic	.	.	.	X	X	.	.
	Fur, Thin Cover, Lumbar	.	.	.	.	.	.	.
	Skin, Scab, Dorsal Thoracic	.	.	.	.	.	.	X
1006	Fur, Thin Cover, Lumbar	.	.	.	.	.	.	.
1007	Fur, Thin Cover, Forepaw, Left	.	X	X	.	.	.	.
	Fur, Thin Cover, Forepaw, Right	.	X	X	.	.	.	.
	Fur, Thin Cover, Lumbar	X	X	X	.	.	.	.
1008	Fur, Thin Cover, Lumbar	.	.	.	.	.	.	.
1011	Fur, Thin Cover, Dorsal Thoracic	X	X	X	.	.	.	.
	Fur, Thin Cover, Lumbar	.	.	.	X	X	X	X
1013	Limited Usage, Hindlimb, Left, Severe	.	.	.	.	.	.	.
	Limited Usage, Hindlimb, Right, Severe	.	.	.	.	.	.	.
	Limited Usage, Hindpaw, Left, Severe	.	.	.	.	.	.	.
	Limited Usage, Hindpaw, Right, Severe	.	.	.	.	.	.	.
	Breathing, Labored	.	.	.	.	.	.	.
	Prostrate	.	.	.	.	.	.	.
	Fur, Thin Cover, Dorsal Cervical	.	.	.	.	.	.	.
	Fur, Thin Cover, Interscapular	.	.	.	.	.	.	.
	Thin	.	.	.	.	.	.	.
	Weak	.	.	.	.	.	.	.
	Backbone Prominent	.	.	.	.	.	.	.
	Low Carriage	.	.	.	.	.	.	.
	Lying On Side	.	.	.	.	.	.	.
	Activity Decreased	.	.	.	.	.	.	.

X=Present

Appendix 4

Individual Clinical Observations

5550008

0.36x 10E12 vg Group 2 Sex: Male	Observation Type: All Types	Day(s) Relative to Start Date						
		-7 Vet	-1 DE	7 DE	8 DE	14 DE	21 DE	28 DE
2001	Skin, Discolored, Tail, Red	.	.	X	.	X	X	X
2002	Fur, Thin Cover, Lumbar	.	.	.	.	.	X	X
2103	Fur, Thin Cover, Lumbar	.	.	.	.	.	.	.
	Skin, Scab, Lumbar	.	.	.	.	.	.	X
2004	Fur, Staining, Muzzle, Black	.	X	X	X	.	.	.
2006	Fur, Thin Cover, Lumbar	.	.	.	.	.	.	X
	Skin, Scab, Lumbar	.	.	.	.	.	.	X
	Skin, Scab, Tail	.	.	.	.	.	.	.
2007	Fur, Thin Cover, Scapular, Right	X	.	.	.	.	.	.
2008	Hypersensitive	.	.	.	.	.	.	.
2011	Fur, Thin Cover, Lumbar	.	.	.	.	.	.	X
2012	Skin, Scab, Lumbar	.	.	.	.	.	.	X

X=Present

Appendix 4

Individual Clinical Observations

5550008

0.36x 10E12 vg Group 2 Sex: Male	Observation Type: All Types	Day(s) Relative to Start Date						
		29 DE	35 DE	42 DE	49 DE	56 DE	63 DE	70 DE
2001	Skin, Discolored, Tail, Red	X	.	.	.	.	.	.
2002	Fur, Thin Cover, Lumbar	X	.	.	.	.	.	.
2103	Fur, Thin Cover, Lumbar	.	.	X	X	X	X	X
	Skin, Scab, Lumbar	.	.	X	X	X	X	X
2004	Fur, Staining, Muzzle, Black	.	.	.	.	.	.	.
2006	Fur, Thin Cover, Lumbar	.	X	X	.	.	.	.
	Skin, Scab, Lumbar	.	X	X	X	X	X	X
	Skin, Scab, Tail	.	X	X	X	.	.	.
2007	Fur, Thin Cover, Scapular, Right	.	.	.	.	.	.	.
2008	Hypersensitive	.	X	X	X	X	X	.
2011	Fur, Thin Cover, Lumbar	X	.	.	.	.	.	.
2012	Skin, Scab, Lumbar	.	.	.	.	.	.	.

X=Present

Appendix 4

Individual Clinical Observations

5550008

0.36x 10E12 vg Group 2 Sex: Male	Observation Type: All Types	Day(s) Relative to Start Date						
		77 DE	84 DE	91 DE				
2001	Skin, Discolored, Tail, Red	.	.	.				
2002	Fur, Thin Cover, Lumbar	.	.	.				
2103	Fur, Thin Cover, Lumbar	X	X	X				
	Skin, Scab, Lumbar	X	X	X				
2004	Fur, Staining, Muzzle, Black	.	.	.				
2006	Fur, Thin Cover, Lumbar	.	.	.				
	Skin, Scab, Lumbar	X	X	X				
	Skin, Scab, Tail	.	.	.				
2007	Fur, Thin Cover, Scapular, Right	.	.	.				
2008	Hypersensitive	.	.	.				
2011	Fur, Thin Cover, Lumbar	.	.	.				
2012	Skin, Scab, Lumbar	.	.	.				

X=Present

Appendix 4

Individual Clinical Observations

5550008

1.1x 10E12 vg Group 3 Sex: Male	Observation Type: All Types	Day(s) Relative to Start Date						
		-1 DE	7 DE	8 DE	14 DE	21 DE	28 DE	29 DE
3003	Fur, Thin Cover, Cranium	.	X	.	.	.	.	.
	Fur, Thin Cover, Dorsal Cervical	.	X	.	.	.	.	.
	Fur, Thin Cover, Scapular, Right	.	X	X	.	.	.	.
3005	Fur, Thin Cover, Lumbar	.	.	.	.	.	X	X
	Skin, Staining, Tail, Red	.	.	.	.	.	X	X
3006	Tail, Bent (PT)	X	X	.	X	X	X	.
	Fur, Thin Cover, Dorsal Thoracic	.	.	.	.	.	.	.
	Fur, Thin Cover, Lumbar	.	.	.	.	.	.	.
3007	Fur, Thin Cover, Lumbar	.	.	.	.	.	X	.
	Skin, Scab, Lumbar	.	.	.	.	.	.	.
3008	Fur, Thin Cover, Lumbar	.	.	.	.	.	X	.
	Skin, Scab, Lumbar	.	.	.	.	.	X	.
3011	Fur, Thin Cover, Lumbar	.	.	.	.	.	X	X
	Skin, Scab, Lumbar	.	.	.	.	.	X	X
3014	Skin, Scab, Lumbar	.	.	.	X	X	X	.
	Swollen, Lumbar, Slight, Soft	.	X	.	X	X	.	.
3015	Skin, Scab, Tail	.	.	.	.	X	X	.

X=Present

Appendix 4

Individual Clinical Observations

5550008

1.1x 10E12 vg Group 3 Sex: Male	Observation Type: All Types	Day(s) Relative to Start Date						
		35 DE	42 DE	49 DE	56 DE	63 DE	70 DE	77 DE
3003	Fur, Thin Cover, Cranium	.	.	.	.	.	.	.
	Fur, Thin Cover, Dorsal Cervical	.	.	.	.	.	.	.
	Fur, Thin Cover, Scapular, Right	.	.	.	.	.	.	.
3005	Fur, Thin Cover, Lumbar	.	.	.	.	.	.	.
	Skin, Staining, Tail, Red	.	.	.	.	.	.	.
3006	Tail, Bent (PT)	X	X	X	X	X	X	X
	Fur, Thin Cover, Dorsal Thoracic	.	.	.	.	.	X	X
	Fur, Thin Cover, Lumbar	.	X	.	.	.	.	.
3007	Fur, Thin Cover, Lumbar	X	X	.	.	.	.	.
	Skin, Scab, Lumbar	.	.	.	X	X	X	X
3008	Fur, Thin Cover, Lumbar	X	X	.	.	.	X	X
	Skin, Scab, Lumbar	X	X	.	.	.	.	.
3011	Fur, Thin Cover, Lumbar	.	.	.	.	.	.	.
	Skin, Scab, Lumbar	.	.	.	.	.	.	.
3014	Skin, Scab, Lumbar	X	X	X	X	X	.	.
	Swollen, Lumbar, Slight, Soft	.	.	.	.	.	.	.
3015	Skin, Scab, Tail	.	.	.	.	.	.	.

X=Present

Appendix 4

Individual Clinical Observations

5550008

1.1x 10E12 vg Group 3 Sex: Male	Observation Type: All Types	Day(s) Relative to Start Date						
		84 DE	91 DE					
3003	Fur, Thin Cover, Cranium	.	.					
	Fur, Thin Cover, Dorsal Cervical	.	.					
	Fur, Thin Cover, Scapular, Right	.	.					
3005	Fur, Thin Cover, Lumbar	.	.					
	Skin, Staining, Tail, Red	.	.					
3006	Tail, Bent (PT)	X	X					
	Fur, Thin Cover, Dorsal Thoracic	X	X					
	Fur, Thin Cover, Lumbar	.	.					
3007	Fur, Thin Cover, Lumbar	.	.					
	Skin, Scab, Lumbar	X	X					
3008	Fur, Thin Cover, Lumbar	.	.					
	Skin, Scab, Lumbar	.	.					
3011	Fur, Thin Cover, Lumbar	.	.					
	Skin, Scab, Lumbar	.	.					
3014	Skin, Scab, Lumbar	.	.					
	Swollen, Lumbar, Slight, Soft	.	.					
3015	Skin, Scab, Tail	.	.					

X=Present

Appendix 4

Individual Clinical Observations

5550008

3.3x 10E12 vg Group 4 Sex: Male	Observation Type: All Types	Day(s) Relative to Start Date						
		-9 Vet	-1 DE	7 DE	8 DE	14 DE	21 DE	28 DE
4002	Fur, Thin Cover, Lumbar	.	.	X	X	.	.	.
	Skin, Scab, Lumbar	.	.	X	X	.	.	.
4003	Skin, Scab, Lumbar	.	.	.	.	.	X	X
4006	Skin, Scab, Dorsal Thoracic	.	.	.	X	.	.	.
4007	Fur, Thin Cover, Dorsal Cervical	.	X	.	.	.	.	.
	Fur, Thin Cover, Dorsal Thoracic	.	X	.	.	.	.	.
	Fur, Thin Cover, Interscapular	.	X	.	.	.	.	.
	Skin, Scab, Dorsal Thoracic	.	.	.	X	.	.	.
4008	Skin, Scab, Dorsal Thoracic	.	.	.	X	.	.	.
4009	Fur, Thin Cover, Dorsal Cervical	X	.	.	.	.	.	.
	Fur, Thin Cover, Dorsal Thoracic	X	.	.	.	.	.	.
	Fur, Thin Cover, Lumbar	X	.	.	.	.	.	.
4010	Fur, Thin Cover, Dorsal Thoracic	.	.	.	.	.	.	.
	Fur, Thin Cover, Lumbar	.	.	.	.	.	.	.
4011	Fur, Thin Cover, Dorsal Thoracic	.	.	.	.	.	.	.
	Fur, Thin Cover, Lumbar	.	.	.	.	.	.	.
	Skin, Scab, Lumbar	.	.	.	.	.	.	.
4012	Skin, Scab, Dorsal Cervical	.	.	X	.	.	.	.
	Skin, Scab, Lumbar	.	.	.	.	X	X	X
4013	Skin, Scab, Lumbar	.	.	.	.	X	X	X
4014	Fur, Thin Cover, Lumbar	.	.	.	.	.	.	.
4015	Fur, Thin Cover, Cranium	.	.	.	.	.	.	X
	Fur, Thin Cover, Dorsal Cervical	.	.	.	.	.	.	.
	Skin, Scab, Cranium	.	.	.	.	.	.	X

X=Present

Appendix 4

Individual Clinical Observations

5550008

3.3x 10E12 vg Group 4 Sex: Male	Observation Type: All Types	Day(s) Relative to Start Date						
		-9 Vet	-1 DE	7 DE	8 DE	14 DE	21 DE	28 DE
4015	Mass Present	.	.	.	.	.	.	.

Appendix 4

Individual Clinical Observations

5550008

3.3x 10E12 vg Group 4 Sex: Male	Observation Type: All Types	Day(s) Relative to Start Date 29 DE	35 DE	42 DE	49 DE	56 DE	63 DE	70 DE
4002	Fur, Thin Cover, Lumbar	.	.	.	.	.	.	.
	Skin, Scab, Lumbar	.	.	.	.	.	.	.
4003	Skin, Scab, Lumbar	X	.	.	.	.	.	.
4006	Skin, Scab, Dorsal Thoracic	.	.	.	.	.	.	.
4007	Fur, Thin Cover, Dorsal Cervical	.	.	.	.	.	.	.
	Fur, Thin Cover, Dorsal Thoracic	.	.	.	.	.	.	.
	Fur, Thin Cover, Interscapular	.	.	.	.	.	.	.
	Skin, Scab, Dorsal Thoracic	.	.	.	.	.	.	.
4008	Skin, Scab, Dorsal Thoracic	.	.	.	.	.	.	.
4009	Fur, Thin Cover, Dorsal Cervical	.	.	.	.	.	.	.
	Fur, Thin Cover, Dorsal Thoracic	.	.	.	.	.	.	.
	Fur, Thin Cover, Lumbar	.	.	.	.	.	.	.
4010	Fur, Thin Cover, Dorsal Thoracic	.	.	.	.	.	X	.
	Fur, Thin Cover, Lumbar	.	.	.	.	.	.	.
4011	Fur, Thin Cover, Dorsal Thoracic	.	.	.	.	.	X	.
	Fur, Thin Cover, Lumbar	.	.	.	.	.	.	X
	Skin, Scab, Lumbar	.	.	.	.	.	.	.
4012	Skin, Scab, Dorsal Cervical	.	.	.	.	.	.	.
	Skin, Scab, Lumbar	X	.	.	.	.	.	.
4013	Skin, Scab, Lumbar	X	.	.	.	.	.	.
4014	Fur, Thin Cover, Lumbar	.	.	.	X	X	.	.
4015	Fur, Thin Cover, Cranium	.	X	X	X	X	X	X
	Fur, Thin Cover, Dorsal Cervical	.	.	.	.	.	.	X
	Skin, Scab, Cranium	.	.	.	.	.	.	X

X=Present

Appendix 4

Individual Clinical Observations

5550008

3.3x 10E12 vg Group 4 Sex: Male	Observation Type: All Types	Day(s) Relative to Start Date						
		29 DE	35 DE	42 DE	49 DE	56 DE	63 DE	70 DE
4015	Mass Present	.	.	X	X	X	X	X

X=Present

Appendix 4

Individual Clinical Observations

5550008

3.3x 10E12 vg Group 4 Sex: Male	Observation Type: All Types	Day(s) Relative to Start Date						
		77 DE	84 DE	91 DE				
4002	Fur, Thin Cover, Lumbar	.	.	.				
	Skin, Scab, Lumbar	.	.	.				
4003	Skin, Scab, Lumbar	.	.	.				
4006	Skin, Scab, Dorsal Thoracic	.	.	.				
4007	Fur, Thin Cover, Dorsal Cervical	.	.	.				
	Fur, Thin Cover, Dorsal Thoracic	.	.	.				
	Fur, Thin Cover, Interscapular	.	.	.				
	Skin, Scab, Dorsal Thoracic	.	.	.				
4008	Skin, Scab, Dorsal Thoracic	.	.	.				
4009	Fur, Thin Cover, Dorsal Cervical	.	.	.				
	Fur, Thin Cover, Dorsal Thoracic	.	.	.				
	Fur, Thin Cover, Lumbar	X	.	.				
4010	Fur, Thin Cover, Dorsal Thoracic	.	.	.				
	Fur, Thin Cover, Lumbar	X	X	X				
4011	Fur, Thin Cover, Dorsal Thoracic	.	.	.				
	Fur, Thin Cover, Lumbar	X	X	X				
	Skin, Scab, Lumbar	X	.	.				
4012	Skin, Scab, Dorsal Cervical	.	.	.				
	Skin, Scab, Lumbar	.	.	.				
4013	Skin, Scab, Lumbar	.	.	.				
4014	Fur, Thin Cover, Lumbar	.	.	.				
4015	Fur, Thin Cover, Cranium	X	X	X				
	Fur, Thin Cover, Dorsal Cervical	X	X	X				
	Skin, Scab, Cranium	X	X	X				

X=Present

Appendix 4

Individual Clinical Observations

5550008

3.3x 10E12 vg Group 4 Sex: Male	Observation Type: All Types	Day(s) Relative to Start Date						
		77 DE	84 DE	91 DE				
4015	Mass Present	X	X	X				

X=Present

Appendix 4

Individual Clinical Observations

5550008

0<br>vg<br>Group 1<br>Sex: Female	Observation Type: All Types	Day(s) Relative to Start Date						
		7<br>DE	8<br>DE	8<br>PM_S	21<br>DE	28<br>DE	29<br>DE	35<br>DE
1503	Fur, Thin Cover, Forepaw, Left	.	.	.	.	.	.	X
	Fur, Thin Cover, Forepaw, Right	.	.	.	.	.	.	X
1504	Skin, Scab, Dorsal Thoracic	.	.	.	.	.	.	.
	Skin, Scab, Lumbar	.	.	.	.	.	.	.
1506	Skin, Scab, Dorsal Thoracic	.	X	.	.	.	.	.
1508	Fur, Thin Cover, Dorsal Cervical	.	.	.	.	X	X	.
1509	Fur, Thin Cover, Dorsal Thoracic	.	.	.	.	X	X	.
1510	Fur, Thin Cover, Cranium	.	.	.	.	.	.	.
	Fur, Thin Cover, Interscapular	.	.	.	.	X	.	X
	Fur, Thin Cover, Sacral	.	.	.	.	X	.	X
	Skin, Scab, Cranium	.	.	.	.	.	.	.
	Skin, Scab, Lumbar	.	.	.	.	.	.	.
	Teeth, Discolored, White	.	.	.	X	X	.	X
	Pinna, Missing (PT), Right, Partly	.	.	.	X	X	.	X
1511	Vocalization, Increased	.	.	.	.	.	.	X
	Fur, Thin Cover, Cranium	.	.	.	.	.	.	.
	Skin, Scab, Cranium	.	.	.	.	.	.	.
	Hypersensitive	.	.	.	.	X	.	X
1512	Fur, Thin Cover, Interscapular	.	.	.	.	X	.	X
	Skin, Staining, Mouth, Red	.	.	.	.	.	.	X
1513	Skin, Discolored, Lumbar, Blue	.	.	X	.	.	.	.
	Swollen (Cageside), Lumbar, Slight	.	.	X	.	.	.	.
1514	Fur, Thin Cover, Sacral	.	.	.	.	X	X	.
1515	Skin, Scab, Dorsal Cervical	X	.	.	.	.	.	.

X=Present

Individual Clinical Observations

5550008

0<br>vg<br>Group 1<br>Sex: Female	Observation Type: All Types	Day(s) Relative to Start Date						
		42<br>DE	49<br>DE	56<br>DE	63<br>DE	70<br>DE	77<br>DE	84<br>DE
1503	Fur, Thin Cover, Forepaw, Left	X	X	X	X	.	.	.
	Fur, Thin Cover, Forepaw, Right	X	X	X	X	.	.	.
1504	Skin, Scab, Dorsal Thoracic	.	.	.	.	X	.	.
	Skin, Scab, Lumbar	.	.	.	.	.	.	.
1506	Skin, Scab, Dorsal Thoracic	.	.	.	.	.	.	.
1508	Fur, Thin Cover, Dorsal Cervical	.	.	.	.	.	.	.
1509	Fur, Thin Cover, Dorsal Thoracic	.	.	.	.	.	.	.
1510	Fur, Thin Cover, Cranium	.	.	.	.	.	X	X
	Fur, Thin Cover, Interscapular	X	X	X	X	.	.	.
	Fur, Thin Cover, Sacral	X	X	X	X	.	.	.
	Skin, Scab, Cranium	.	.	.	.	.	X	X
	Skin, Scab, Lumbar	X	X	X	X	X	X	X
	Teeth, Discolored, White	.	.	.	.	.	.	.
	Pinna, Missing (PT), Right, Partly	X	X	X	X	X	X	X
1511	Vocalization, Increased	.	.	.	.	.	.	.
	Fur, Thin Cover, Cranium	.	.	.	.	.	X	X
	Skin, Scab, Cranium	.	.	.	.	.	X	X
	Hypersensitive	.	.	X	X	.	.	.
1512	Fur, Thin Cover, Interscapular	.	.	.	.	.	.	.
	Skin, Staining, Mouth, Red	.	.	.	.	.	.	.
1513	Skin, Discolored, Lumbar, Blue	.	.	.	.	.	.	.
	Swollen (Cageside), Lumbar, Slight	.	.	.	.	.	.	.
1514	Fur, Thin Cover, Sacral	.	.	.	.	.	.	.
1515	Skin, Scab, Dorsal Cervical	.	.	.	.	.	.	.

X=Present

Appendix 4

Individual Clinical Observations

5550008

0<br>vg<br>Group 1<br>Sex: Female	Observation Type: All Types	Day(s) Relative to Start Date<br>91<br>DE						
1503	Fur, Thin Cover, Forepaw, Left	.						
	Fur, Thin Cover, Forepaw, Right	.						
1504	Skin, Scab, Dorsal Thoracic	.						
	Skin, Scab, Lumbar	X						
1506	Skin, Scab, Dorsal Thoracic	.						
1508	Fur, Thin Cover, Dorsal Cervical	.						
1509	Fur, Thin Cover, Dorsal Thoracic	.						
1510	Fur, Thin Cover, Cranium	X						
	Fur, Thin Cover, Interscapular	.						
	Fur, Thin Cover, Sacral	.						
	Skin, Scab, Cranium	X						
	Skin, Scab, Lumbar	X						
	Teeth, Discolored, White	.						
	Pinna, Missing (PT), Right, Partly	X						
1511	Vocalization, Increased	.						
	Fur, Thin Cover, Cranium	X						
	Skin, Scab, Cranium	X						
	Hypersensitive	.						
1512	Fur, Thin Cover, Interscapular	.						
	Skin, Staining, Mouth, Red	.						
1513	Skin, Discolored, Lumbar, Blue	.						
	Swollen (Cageside), Lumbar, Slight	.						
1514	Fur, Thin Cover, Sacral	.						
1515	Skin, Scab, Dorsal Cervical	.						

X=Present

Appendix 4

Individual Clinical Observations

5550008

0.36x 10E12 vg Group 2 Sex: Female	Observation Type: All Types	Day(s) Relative to Start Date						
		7 DE	8 DE	14 DE	21 DE	28 DE	29 DE	35 DE
2501	Fur, Thin Cover, Forelimb, Left	.	.	X	X	X	X	.
2503	Fur, Thin Cover, Cranium	.	.	.	.	.	.	.
	Skin, Discolored, Tail, Red	.	.	.	X	X	.	X
	Skin, Scab, Cranium	.	.	.	.	.	.	.
2504	Fur, Thin Cover, Dorsal Thoracic	.	.	.	.	.	.	.
	Fur, Thin Cover, Lumbar	.	.	.	.	.	.	.
	Skin, Scab, Lumbar	.	.	.	.	.	.	.
	Skin, Scab, Tail	.	.	.	.	.	.	.
2506	Skin, Scab, Dorsal Thoracic	.	X	.	.	.	.	.
2508	Skin, Scab, Tail	X	.	X	.	.	.	.
2509	Fur, Staining, Dorsal Cervical, Red	.	.	.	.	X	X	.
	Fur, Thin Cover, Lumbar	.	.	.	.	X	X	.
	Skin, Scab, Lumbar	.	.	X	X	X	X	.
2613	Fur, Thin Cover, Dorsal Cervical	.	.	.	.	.	.	.
	Skin, Scab, Dorsal Cervical	X	.	X	X	.	.	.
	Skin, Scab, Lumbar	X	.	.	.	.	.	.
2614	Fur, Staining, Dorsal Cervical, Red	.	.	.	.	.	.	.
	Skin, Scab, Lumbar	X	.	.	.	.	.	.
	Skin, Scab, Tail	.	.	.	.	.	.	.

X=Present

Appendix 4

Individual Clinical Observations

5550008

0.36x 10E12 vg Group 2 Sex: Female	Observation Type: All Types	Day(s) Relative to Start Date						
		42 DE	49 DE	56 DE	63 DE	70 DE	77 DE	84 DE
2501	Fur, Thin Cover, Forelimb, Left	.	.	.	.	.	.	.
2503	Fur, Thin Cover, Cranium	X	X	X	X	X	X	X
	Skin, Discolored, Tail, Red	.	.	.	.	.	.	.
	Skin, Scab, Cranium	X	X	X	X	X	.	X
2504	Fur, Thin Cover, Dorsal Thoracic	.	.	X	X	.	.	.
	Fur, Thin Cover, Lumbar	.	.	X	X	X	X	.
	Skin, Scab, Lumbar	.	.	X	X	X	.	.
	Skin, Scab, Tail	X	X	X	.	.	.	.
2506	Skin, Scab, Dorsal Thoracic	.	.	.	.	.	.	.
2508	Skin, Scab, Tail	.	.	.	.	.	.	.
2509	Fur, Staining, Dorsal Cervical, Red	.	.	.	.	.	.	.
	Fur, Thin Cover, Lumbar	.	.	.	.	.	.	.
	Skin, Scab, Lumbar	.	.	.	.	.	.	.
2613	Fur, Thin Cover, Dorsal Cervical	X	X	X	X	.	.	.
	Skin, Scab, Dorsal Cervical	.	.	.	.	.	.	.
	Skin, Scab, Lumbar	.	.	.	.	.	.	.
2614	Fur, Staining, Dorsal Cervical, Red	X	X	.	.	.	.	.
	Skin, Scab, Lumbar	.	.	.	.	.	.	.
	Skin, Scab, Tail	.	.	.	.	.	.	.

X=Present

Appendix 4

Individual Clinical Observations

5550008

0.36x 10E12 vg Group 2 Sex: Female	Observation Type: All Types	Day(s) Relative to Start Date						
		91 DE						
2501	Fur, Thin Cover, Forelimb, Left	.						
2503	Fur, Thin Cover, Cranium	X						
	Skin, Discolored, Tail, Red	.						
	Skin, Scab, Cranium	X						
2504	Fur, Thin Cover, Dorsal Thoracic	.						
	Fur, Thin Cover, Lumbar	.						
	Skin, Scab, Lumbar	.						
	Skin, Scab, Tail	.						
2506	Skin, Scab, Dorsal Thoracic	.						
2508	Skin, Scab, Tail	.						
2509	Fur, Staining, Dorsal Cervical, Red	.						
	Fur, Thin Cover, Lumbar	.						
	Skin, Scab, Lumbar	.						
2613	Fur, Thin Cover, Dorsal Cervical	.						
	Skin, Scab, Dorsal Cervical	.						
	Skin, Scab, Lumbar	.						
2614	Fur, Staining, Dorsal Cervical, Red	.						
	Skin, Scab, Lumbar	.						
	Skin, Scab, Tail	X						

X=Present

Appendix 4

Individual Clinical Observations

5550008

1.1x 10E12 vg Group 3 Sex: Female	Observation Type: All Types	Day(s) Relative to Start Date						
		7 DE	8 DE	28 DE	29 DE	35 DE	42 DE	49 DE
3501	Fur, Staining, Cranium, Red	.	.	.	.	.	.	.
	Fur, Staining, Ventral Cervical, Red	.	.	.	.	.	.	.
3503	Fur, Staining, Cranium, Red	.	.	.	.	X	X	X
	Fur, Staining, Dorsal Cervical, Red	.	.	.	.	X	X	X
	Skin, Scab, Tail	.	.	.	.	.	X	X
3504	Skin, Scab, Lumbar	.	.	X	X	.	.	.
3505	Fur, Staining, Abdominal, Red	.	.	X	.	.	.	.
	Skin, Scab, Lumbar	.	.	X	.	.	.	.
3509	Fur, Thin Cover, Interscapular	.	.	X	.	X	X	X
	Fur, Thin Cover, Sacral	.	.	X	.	X	X	X
	Hypersensitive	.	.	.	.	.	X	X
3510	Skin, Scab, Tail	.	.	.	.	.	X	X
	Hypersensitive	.	.	.	.	.	.	.
3511	Skin, Scab, Dorsal Cervical	X	.	.	.	.	.	.
	Skin, Scab, Lumbar	X	X	.	.	.	.	.
3512	Skin, Scab, Lumbar	X	X	.	.	.	.	.
	Skin, Scab, Periorbital, Right	.	X	.	.	.	.	.
3513	Skin, Scab, Lumbar	X	X	.	.	.	.	.

X=Present

Appendix 4

Individual Clinical Observations

5550008

1.1x 10E12 vg Group 3 Sex: Female	Observation Type: All Types	Day(s) Relative to Start Date 56 DE	63 DE	77 DE	84 DE	91 DE		
3501	Fur, Staining, Cranium, Red	X	.	.	.	.		
	Fur, Staining, Ventral Cervical, Red	.	.	X	X	X		
3503	Fur, Staining, Cranium, Red	.	.	.	.	.		
	Fur, Staining, Dorsal Cervical, Red	X	.	X	X	X		
	Skin, Scab, Tail	.	.	.	.	.		
3504	Skin, Scab, Lumbar	.	.	.	.	.		
3505	Fur, Staining, Abdominal, Red	.	.	.	.	.		
	Skin, Scab, Lumbar	.	.	.	.	.		
3509	Fur, Thin Cover, Interscapular	X	X	.	.	.		
	Fur, Thin Cover, Sacral	X	X	X	X	X		
	Hypersensitive	X	.	.	.	.		
3510	Skin, Scab, Tail	.	.	.	.	.		
	Hypersensitive	X	.	.	.	.		
3511	Skin, Scab, Dorsal Cervical	.	.	.	.	.		
	Skin, Scab, Lumbar	.	.	.	.	.		
3512	Skin, Scab, Lumbar	.	.	.	.	.		
	Skin, Scab, Periorbital, Right	.	.	.	.	.		
3513	Skin, Scab, Lumbar	.	.	.	.	.		

X=Present

Appendix 4

Individual Clinical Observations

5550008

3.3x 10E12 vg Group 4 Sex: Female	Observation Type: All Types	Day(s) Relative to Start Date						
		7 DE	14 DE	21 DE	28 DE	29 DE	35 DE	42 DE
4503	Fur, Thin Cover, Lumbar	.	.	.	.	.	.	.
	Teeth, Discolored, White	X	X	X	X	.	X	X
4504	Fur, Staining, Cranium, Red	.	.	.	.	.	X	X
	Fur, Staining, Dorsal Cervical, Red	.	.	.	.	.	.	.
	Fur, Thin Cover, Dorsal Cervical	.	.	.	.	.	.	.
	Fur, Thin Cover, Hindlimb, Left	.	.	.	.	.	.	.
	Fur, Thin Cover, Hindlimb, Right	.	.	.	.	.	.	.
	Fur, Thin Cover, Lumbar	.	.	.	.	.	.	X
	Fur, Thin Cover, Sacral	.	.	.	.	.	.	X
	Fur, Thin Cover, Scapular, Left	.	.	.	.	.	X	.
	Fur, Thin Cover, Scapular, Right	.	.	.	.	.	X	X
4511	Fur, Thin Cover, Cranium	.	.	X	X	X	.	.
	Fur, Thin Cover, Dorsal Cervical	.	.	X	X	X	.	.
	Fur, Thin Cover, Forelimb, Left	.	.	X	X	X	.	.
	Fur, Thin Cover, Forelimb, Right	.	.	X	X	X	.	.
	Fur, Thin Cover, Hindlimb, Left	.	.	X	X	X	.	.
	Fur, Thin Cover, Hindlimb, Right	.	.	X	X	X	.	.
	Fur, Thin Cover, Ventral Thoracic	.	.	X	X	X	.	.
4512	Skin, Scab, Dorsal Cervical	X	.	.	.	.	.	.
	Pinna, Missing (PT), Left, Partly	X	X	X	X	X	.	.
4513	Fur, Thin Cover, Lumbar	.	.	.	.	.	.	X
	Skin, Scab, Lumbar	X	X	X	X	.	X	X
4514	Fur, Staining, Dorsal Cervical, Red	.	.	.	.	.	.	X
	Fur, Thin Cover, Lumbar	.	.	.	.	.	.	X

X=Present

Individual Clinical Observations

5550008

3.3x 10E12 vg Group 4 Sex: Female	Observation Type: All Types	Day(s) Relative to Start Date						
		7 DE	14 DE	21 DE	28 DE	29 DE	35 DE	42 DE
4514	Skin, Scab, Lumbar	.	X	X	X	.	X	X
	Hypersensitive	X	X	X	.	.	.	.
4515	Fur, Staining, Cranium, Red	.	.	.	.	.	.	.
	Fur, Staining, Dorsal Cervical, Red	.	.	.	.	.	.	.
	Fur, Staining, Ventral Cervical, Red	.	.	.	.	.	.	X
	Fur, Thin Cover, Cranium	.	.	.	.	.	.	.
	Fur, Thin Cover, Lumbar	.	.	.	.	.	X	X

X=Present

Appendix 4

Individual Clinical Observations

5550008

3.3x 10E12 vg Group 4 Sex: Female	Observation Type: All Types	Day(s) Relative to Start Date						
		49 DE	56 DE	63 DE	70 DE	77 DE	84 DE	91 DE
4503	Fur, Thin Cover, Lumbar	.	.	.	.	X	.	.
	Teeth, Discolored, White	X	.	.	X	.	.	.
4504	Fur, Staining, Cranium, Red	X	X	X	X	X	.	.
	Fur, Staining, Dorsal Cervical, Red	.	.	.	X	X	X	X
	Fur, Thin Cover, Dorsal Cervical	.	X	X	.	.	.	.
	Fur, Thin Cover, Hindlimb, Left	.	X	X	X	X	X	X
	Fur, Thin Cover, Hindlimb, Right	.	X	X	X	X	X	X
	Fur, Thin Cover, Lumbar	X	X	X	X	X	X	X
	Fur, Thin Cover, Sacral	X	X	X	X	X	X	X
	Fur, Thin Cover, Scapular, Left	.	.	.	.	.	.	.
	Fur, Thin Cover, Scapular, Right	X	X	X	X	X	.	.
4511	Fur, Thin Cover, Cranium	.	.	.	.	.	.	.
	Fur, Thin Cover, Dorsal Cervical	.	.	.	.	.	.	.
	Fur, Thin Cover, Forelimb, Left	.	.	.	.	.	.	.
	Fur, Thin Cover, Forelimb, Right	.	.	.	.	.	.	.
	Fur, Thin Cover, Hindlimb, Left	.	.	.	.	.	.	.
	Fur, Thin Cover, Hindlimb, Right	.	.	.	.	.	.	.
	Fur, Thin Cover, Ventral Thoracic	.	.	.	.	.	.	.
4512	Skin, Scab, Dorsal Cervical	.	.	.	.	.	.	.
	Pinna, Missing (PT), Left, Partly	.	.	.	.	.	.	.
4513	Fur, Thin Cover, Lumbar	X	X	X	X	X	X	X
	Skin, Scab, Lumbar	X	.	.	.	.	.	.
4514	Fur, Staining, Dorsal Cervical, Red	X	X	X	X	X	X	.
	Fur, Thin Cover, Lumbar	X	X	.	.	.	.	.

X=Present

Appendix 4

Individual Clinical Observations

5550008

3.3x 10E12 vg Group 4 Sex: Female	Observation Type: All Types	Day(s) Relative to Start Date						
		49 DE	56 DE	63 DE	70 DE	77 DE	84 DE	91 DE
4514	Skin, Scab, Lumbar	X	X	.	.	.	.	.
	Hypersensitive	.	.	.	.	.	.	.
4515	Fur, Staining, Cranium, Red	.	.	.	.	.	.	X
	Fur, Staining, Dorsal Cervical, Red	.	.	.	X	X	X	X
	Fur, Staining, Ventral Cervical, Red	X	X	X	X	X	X	X
	Fur, Thin Cover, Cranium	.	.	.	.	.	.	X
	Fur, Thin Cover, Lumbar	X	X	X	X	X	X	X

X=Present

Appendix 5

Individual Palpable Masses

5550008

Group: 4 Animal: 4015 Sex: Male

Mass	Day (Week)	Time	Location	Size	Description
1	42(6)	13:19	Inguinal, Left	15-19 mm	Soft, Subcutaneous, Movable, Granular, Normal surface
1	56(8)	11:01	Inguinal, Left	15-19 mm	Soft, Subcutaneous, Movable, Granular, Normal surface
1	63(9)	9:11	Inguinal, Left	15-19 mm	Soft, Subcutaneous, Movable, Granular, Normal surface
1	70(10)	10:52	Inguinal, Left	15-19 mm	Soft, Subcutaneous, Movable, Normal surface
1	77(11)	11:23	Inguinal, Left	15-19 mm by 1 mm	Soft, Subcutaneous, Movable, Normal surface
1	77(11)	13:54	Inguinal, Left	45-49 mm	Soft, Subcutaneous, Movable, Normal surface
1	84(12)	11:43	Inguinal, Left	45-49 mm	Soft, Subcutaneous, Movable, Normal surface
1	91(13)	7:28	Inguinal, Left	45-49 mm	Soft, Subcutaneous, Movable, Normal surface

Appendix 6

Individual Body Weights

5550008
Sex: Male Bodyweight (g)

0 vg Group 1	Day(s) Relative to Start Date -1	7	8	14	21	28	29
1001	216	268 [a]	242 [a]	-	-	-	-
1002	201	254 [a]	224 [a]	-	-	-	-
1003	190	248 [a]	-	300 [a]	339	381	349 [a]
1004	182	237 [a]	-	292 [a]	327	368	345 [a]
1005	179	224 [a]	-	285 [a]	334 [a]	371 [a]	-
1006	177	232 [a]	-	298 [a]	341 [a]	373 [a]	-
1007	241	298 [a]	-	374 [a]	429 [a]	478 [a]	-
1008	228	262	-	305	349	392 [a]	365
1009	241	275	-	323	370	408 [a]	384
1010	245	295	-	337	389	433 [a]	401
1011	243	296	-	346	401	441	-
1012	212	261	-	314 [a]	362	420	-
1013	219	-	-	-	-	-	-
1014	249	302 [a]	264 [a]	-	-	-	-
1015	260	281 [a]	247	-	-	-	-
Mean	218.9	266.6	244.3	317.4	364.1	406.5	368.8
SD	27.8	25.8	16.5	27.8	33.2	36.4	23.7
N	15	14	4	10	10	10	5

[a] [RC:Value Confirmed]

Appendix 6

Individual Body Weights

5550008
Sex: Male Bodyweight (g)

0<br>vg<br>Group 1	Day(s) Relative to Start Date						
	35	42	49	56	63	70	77
1001	-	-	-	-	-	-	-
1002	-	-	-	-	-	-	-
1003	-	-	-	-	-	-	-
1004	-	-	-	-	-	-	-
1005	413 [a]	453 [a]	477	507	533	553	564
1006	409 [a]	446 [a]	465	492	515	529	535
1007	533 [a]	588 [a]	615	653	679	712	730
1008	-	-	-	-	-	-	-
1009	-	-	-	-	-	-	-
1010	-	-	-	-	-	-	-
1011	462	501 [a]	538	564	591	603	611
1012	454	495 [a]	520	548	570	583	597
1013	-	-	-	-	-	-	-
1014	-	-	-	-	-	-	-
1015	-	-	-	-	-	-	-
Mean	454.2	496.6	523.0	552.8	577.6	596.0	607.4
SD	50.0	56.7	59.5	63.2	64.1	70.7	74.6
N	5	5	5	5	5	5	5

[a] [RC:Value Confirmed]

Appendix 6

Individual Body Weights

5550008
Sex: Male Bodyweight (g)

0<br>vg<br>Group 1	Day(s) Relative to Start Date<br>84	 91
1001	-	-
1002	-	-
1003	-	-
1004	-	-
1005	579 [a]	568
1006	553 [a]	543
1007	749 [a]	739
1008	-	-
1009	-	-
1010	-	-
1011	632	616 [a]
1012	622	600
1013	-	-
1014	-	-
1015	-	-
Mean	627.0	613.2
SD	75.4	75.8
N	5	5

[a] [RC:Value Confirmed]

Appendix 6

Individual Body Weights

5550008
Sex: Male Bodyweight (g)

0.36x 10E12 vg Group 2	Day(s) Relative to Start Date						
	-1	7	8	14	21	28	29
2001	183	238 [a]	-	303 [a]	351	403	380 [a]
2002	203	256 [a]	-	313 [a]	357	392	368 [a]
2103	215	269	-	318	363	406	-
2004	170	208 [a]	188	-	-	-	-
2005	189	241	-	311 [a]	364 [a]	409 [a]	-
2006	207	255	-	318 [a]	369 [a]	412 [a]	-
2007	206	259	-	328 [a]	378	430 [a]	-
2008	202	247	-	294	331	382	-
2009	272	314	281 [a]	-	-	-	-
2010	251	302	272 [a]	-	-	-	-
2011	209	252	-	297	341	380	356
2012	225	261	-	314 [a]	370	422	389
2013	204	245	-	292	339	379	352
2014	240	282 [a]	256	-	-	-	-
2015	216	248 [a]	221	-	-	-	-
Mean	212.8	258.5	243.6	308.8	356.3	401.5	369.0
SD	26.1	25.9	38.6	11.8	15.4	17.8	15.7
N	15	15	5	10	10	10	5
%Diff	-2.8	-3.1	-0.3	-2.7	-2.1	-1.2	0.1

[a] [RC:Value Confirmed]

Appendix 6

Individual Body Weights

5550008
Sex: Male Bodyweight (g)

0.36x 10E12 vg Group 2	Day(s) Relative to Start Date						
	35	42	49	56	63	70	77
2001	-	-	-	-	-	-	-
2002	-	-	-	-	-	-	-
2103	442	482	507	532	570	590	604
2004	-	-	-	-	-	-	-
2005	453	498 [a]	530	557	583	615	634
2006	457	494 [a]	519	547	561	587	600
2007	479 [a]	525 [a]	548	582	604	629	641
2008	421	457	497	522	548	563	566
2009	-	-	-	-	-	-	-
2010	-	-	-	-	-	-	-
2011	-	-	-	-	-	-	-
2012	-	-	-	-	-	-	-
2013	-	-	-	-	-	-	-
2014	-	-	-	-	-	-	-
2015	-	-	-	-	-	-	-
Mean	450.4	491.2	520.2	548.0	573.2	596.8	609.0
SD	21.2	24.8	19.9	23.3	21.4	25.8	30.0
N	5	5	5	5	5	5	5
%Diff	-0.8	-1.1	-0.5	-0.9	-0.8	0.1	0.3

[a] [RC:Value Confirmed]

Appendix 6

Individual Body Weights

5550008
Sex: Male Bodyweight (g)

0.36x 10E12 vg Group 2	Day(s) Relative to Start Date	
	84	91
2001	-	-
2002	-	-
2103	634	610
2004	-	-
2005	660 [a]	656
2006	619 [a]	605
2007	665 [a]	650
2008	588	575
2009	-	-
2010	-	-
2011	-	-
2012	-	-
2013	-	-
2014	-	-
2015	-	-
Mean	633.2	619.2
SD	31.5	33.7
N	5	5
%Diff	1.0	1.0

[a] [RC:Value Confirmed]

Appendix 6

Individual Body Weights

5550008
Sex: Male Bodyweight (g)

1.1x 10E12 vg Group 3	Day(s) Relative to Start Date						
	-1	7	8	14	21	28	29
3001	188	233 [a]	211	-	-	-	-
3002	215	269 [a]	244	-	-	-	-
3003	180	228 [a]	204	-	-	-	-
3004	196	240	-	291 [a]	329	372	337 [a]
3005	212	259	-	319 [a]	366	409	377 [a]
3006	204	260	-	320 [a]	355	383	-
3007	205	255	-	324 [a]	377	419	-
3008	184	225	-	282 [a]	336	385	-
3009	218	264	-	311	363	408	372
3010	221	262	-	313	358	406	373
3011	203	230	-	269	310	346	321
3012	229	274	245	-	-	-	-
3013	250	288	258	-	-	-	-
3014	226	259	-	310 [a]	341	369 [a]	-
3015	262	304	-	357 [a]	397	434 [a]	-
Mean	212.9	256.7	232.4	309.6	353.2	393.1	356.0
SD	23.0	22.6	23.5	24.5	25.1	26.7	25.4
N	15	15	5	10	10	10	5
%Diff	-2.7	-3.7	-4.9	-2.5	-3.0	-3.3	-3.5

[a] [RC:Value Confirmed]

Appendix 6

Individual Body Weights

5550008
Sex: Male Bodyweight (g)

1.1x 10E12 vg Group 3	Day(s) Relative to Start Date						
	35	42	49	56	63	70	77
3001	-	-	-	-	-	-	-
3002	-	-	-	-	-	-	-
3003	-	-	-	-	-	-	-
3004	-	-	-	-	-	-	-
3005	-	-	-	-	-	-	-
3006	420	451 [a]	467	486	491	507	528
3007	472	519 [a]	554	594	616	645	666
3008	415	459 [a]	481	504	528	546	563
3009	-	-	-	-	-	-	-
3010	-	-	-	-	-	-	-
3011	-	-	-	-	-	-	-
3012	-	-	-	-	-	-	-
3013	-	-	-	-	-	-	-
3014	392	417	438	461	472	487	503
3015	466 [a]	493	520	542	574	599	603
Mean	433.0	467.8	492.0	517.4	536.2	556.8	572.6
SD	34.6	39.4	45.5	52.0	59.2	65.2	64.4
N	5	5	5	5	5	5	5
%Diff	-4.7	-5.8	-5.9	-6.4	-7.2	-6.6	-5.7

[a] [RC:Value Confirmed]

Appendix 6

Individual Body Weights

5550008
Sex: Male Bodyweight (g)

1.1x 10E12 vg Group 3	Day(s) Relative to Start Date	
	84	91
3001	-	-
3002	-	-
3003	-	-
3004	-	-
3005	-	-
3006	546 [a]	527
3007	686 [a]	672
3008	577 [a]	580
3009	-	-
3010	-	-
3011	-	-
3012	-	-
3013	-	-
3014	508	503
3015	632	610
Mean	589.8	578.4
SD	70.4	67.2
N	5	5
%Diff	-5.9	-5.7

[a] [RC:Value Confirmed]

Appendix 6

Individual Body Weights

5550008
Sex: Male Bodyweight (g)

3.3x 10E12 vg Group 4	Day(s) Relative to Start Date						
	-1	7	8	14	21	28	29
4001	197	237 [a]	213	-	-	-	-
4002	179	228 [a]	206	-	-	-	-
4003	189	239 [a]	-	300	340	381	357 [a]
4004	235	301 [a]	-	380	453	530	487 [a]
4005	170	209 [a]	-	270	316	357	332 [a]
4006	199	230	207	-	-	-	-
4007	168	204	181	-	-	-	-
4008	237	291	259	-	-	-	-
4009	199	233	-	282 [a]	315	361	-
4010	223	258	-	291	315	345	-
4011	202	244	-	289	322	365	-
4012	225	256	-	304	343	378 [a]	355
4013	285	321	-	394 [a]	445 [a]	508 [a]	468
4014	206	242	-	287	319	345	-
4015	220	271 [a]	-	315	352	388	-
Mean	208.9	250.9	213.2	311.2	352.0	395.8	399.8
SD	30.1	33.0	28.4	41.9	52.8	66.7	71.9
N	15	15	5	10	10	10	5
%Diff	-4.5	-5.9	-12.7	-2.0	-3.3	-2.6	8.4

[a] [RC:Value Confirmed]

Appendix 6

Individual Body Weights

5550008
Sex: Male Bodyweight (g)

3.3x 10E12 vg Group 4	Day(s) Relative to Start Date						
	35	42	49	56	63	70	77
4001	-	-	-	-	-	-	-
4002	-	-	-	-	-	-	-
4003	-	-	-	-	-	-	-
4004	-	-	-	-	-	-	-
4005	-	-	-	-	-	-	-
4006	-	-	-	-	-	-	-
4007	-	-	-	-	-	-	-
4008	-	-	-	-	-	-	-
4009	399	424	451	480	508	534	558
4010	363	381	404	411	420	430	434
4011	399	423	450	473	494	501	523
4012	-	-	-	-	-	-	-
4013	-	-	-	-	-	-	-
4014	370	397	423	440	464	481	497
4015	415	452	461	488	508	524	535
Mean	389.2	415.4	437.8	458.4	478.8	494.0	509.4
SD	21.9	27.4	23.6	32.2	37.5	41.3	47.5
N	5	5	5	5	5	5	5
%Diff	-14.3	-16.4	-16.3	-17.1	-17.1	-17.1	-16.1

Appendix 6

Individual Body Weights

5550008

Sex: Male Bodyweight (g)

3.3x 10E12 vg Group 4	Day(s) Relative to Start Date	
	84	91
4001	-	-
4002	-	-
4003	-	-
4004	-	-
4005	-	-
4006	-	-
4007	-	-
4008	-	-
4009	562	558
4010	444	430
4011	538	521
4012	-	-
4013	-	-
4014	510	494
4015	549	549
Mean	520.6	510.4
SD	46.9	51.5
N	5	5
%Diff	-17.0	-16.8

Appendix 6

Individual Body Weights

5550008
Sex: Female Bodyweight (g)

0<br>vg<br><br>Group 1	Day(s) Relative to Start Date<br>-1	7	8	14	21	28	29
1501	169	190	171 [a]	-	-	-	-
1502	165	188	166 [a]	-	-	-	-
1503	182	203	-	221	235	232	-
1504	177	195	-	211	238	249	-
1505	162	190	169	-	-	-	-
1506	154	171	155	-	-	-	-
1507	161	190	169	-	-	-	-
1508	154	169	-	181	185	195	182
1509	169	182	-	197	208	223	204
1510	163	176	-	194	213	225	-
1511	193	213	-	234	243	257	-
1512	169	185	-	193	201	214	-
1513	203	216 [a]	-	227	232	249 [a]	224 [a]
1514	177	183	-	201	219	222	210
1515	160	170 [a]	-	177	191	201	181
Mean	170.5	188.1	166.0	203.6	216.5	226.7	200.2
SD	13.9	14.4	6.4	19.2	20.3	20.6	18.6
N	15	15	5	10	10	10	5

[a] [RC:Value Confirmed]

Appendix 6

Individual Body Weights

5550008
Sex: Female Bodyweight (g)

0 vg Group 1	Day(s) Relative to Start Date 35	42	49	56	63	70	77
1501	-	-	-	-	-	-	-
1502	-	-	-	-	-	-	-
1503	249	249	259	269	266	278	284
1504	252	266	281	285	284	297	299
1505	-	-	-	-	-	-	-
1506	-	-	-	-	-	-	-
1507	-	-	-	-	-	-	-
1508	-	-	-	-	-	-	-
1509	-	-	-	-	-	-	-
1510	225	242 [a]	254	261	253 [a]	264 [a]	273
1511	274	279	279	289	306	314	308 [a]
1512	220	227	227	239	246	238 [a]	236
1513	-	-	-	-	-	-	-
1514	-	-	-	-	-	-	-
1515	-	-	-	-	-	-	-
Mean	244.0	252.6	260.0	268.6	271.0	278.2	280.0
SD	21.9	20.4	22.0	20.1	24.3	29.4	28.0
N	5	5	5	5	5	5	5

[a] [RC:Value Confirmed]

Appendix 6

Individual Body Weights

5550008
Sex: Female Bodyweight (g)

0<br>vg<br>Group 1	Day(s) Relative to Start Date	
	84	91
1501	-	-
1502	-	-
1503	284	275
1504	306	283 [a]
1505	-	-
1506	-	-
1507	-	-
1508	-	-
1509	-	-
1510	267	256 [a]
1511	311	296 [a]
1512	244	235
1513	-	-
1514	-	-
1515	-	-
Mean	282.4	269.0
SD	27.8	23.9
N	5	5

[a] [RC:Value Confirmed]

Appendix 6

Individual Body Weights

5550008
Sex: Female Bodyweight (g)

0.36x 10E12 vg Group 2	Day(s) Relative to Start Date						
	-6	-4	-1	1	7	8	14
2501	-	-	184	-	202	-	227
2502	-	-	170	-	195	-	228
2503	-	-	150	-	177	-	189
2504	-	-	196	-	224	-	249
2505	-	-	152	-	179	155	-
2506	-	-	165	-	182	167	-
2507	-	-	165	-	193	-	222 [a]
2508	-	-	198	-	214	-	237 [a]
2509	-	-	156	-	180	-	192
2510	-	-	200	-	219	191 [a]	-
2511	-	-	206	-	228	205 [a]	-
2512	-	-	189	-	208	187 [a]	-
2613	150	-	-	174	192	-	211
2614	181	-	-	204	225	-	238
2615	-	184	-	193	214	-	255 [a]
Mean	165.5	184.0	177.6	190.3	202.1	181.0	224.8
SD	21.9	-	20.2	15.2	18.1	19.9	22.1
N	2	1	12	3	15	5	10
%Diff	-	-	4.1	-	7.5	9.0	10.4

[a] [RC:Value Confirmed]

Appendix 6

Individual Body Weights

5550008
Sex: Female Bodyweight (g)

0.36x 10E12 vg Group 2	Day(s) Relative to Start Date						
	21	28	29	35	42	49	56
2501	262	259	248 [a]	-	-	-	-
2502	248	269	249 [a]	-	-	-	-
2503	205	219	-	218	236 [a]	236	250
2504	275	288	-	299	308	328	342
2505	-	-	-	-	-	-	-
2506	-	-	-	-	-	-	-
2507	236	238	221 [a]	-	-	-	-
2508	246	259	236 [a]	-	-	-	-
2509	216	226	207 [a]	-	-	-	-
2510	-	-	-	-	-	-	-
2511	-	-	-	-	-	-	-
2512	-	-	-	-	-	-	-
2613	223	242 [a]	-	257	259	273	279
2614	257	274 [a]	-	279	285	302	309
2615	240 [a]	238	-	248	260	265	268
Mean	240.8	251.2	232.2	260.2	269.6	280.8	289.6
SD	21.6	22.2	18.1	30.8	27.6	35.3	36.3
N	10	10	5	5	5	5	5
%Diff	11.2	10.8	16.0	6.6	6.7	8.0	7.8

[a] [RC:Value Confirmed]

Appendix 6

Individual Body Weights

5550008

Sex: Female Bodyweight (g)

0.36x 10E12 vg Group 2	Day(s) Relative to Start Date				
	63	70	77	84	91
2501	-	-	-	-	-
2502	-	-	-	-	-
2503	249	262	268	265	249 [a]
2504	336	349	347	351	331 [a]
2505	-	-	-	-	-
2506	-	-	-	-	-
2507	-	-	-	-	-
2508	-	-	-	-	-
2509	-	-	-	-	-
2510	-	-	-	-	-
2511	-	-	-	-	-
2512	-	-	-	-	-
2613	279	290	296	305	285 [a]
2614	318	317	334	329	309
2615	281	283	289	280	269
Mean	292.6	300.2	306.8	306.0	288.6
SD	34.5	33.6	32.8	35.0	32.3
N	5	5	5	5	5
%Diff	8.0	7.9	9.6	8.4	7.3

[a] [RC:Value Confirmed]

Appendix 6

Individual Body Weights

5550008
Sex: Female Bodyweight (g)

1.1x 10E12 vg Group 3	Day(s) Relative to Start Date						
	-7	-1	1	7	8	14	21
3501	-	167	-	177	-	199	213
3502	-	171	-	191	-	231 [a]	252
3503	-	153	-	171	-	187	196
3504	-	157	-	180	-	194	207
3505	-	149	-	173	-	190	204
3506	-	146	-	174	-	186	209
3507	-	201	-	212	187 [a]	-	-
3508	-	181	-	196	175 [a]	-	-
3509	-	167	-	192	-	213	227
3510	-	196	-	202	-	217	233
3511	-	197	-	213	187	-	-
3512	-	182	-	199	176	-	-
3513	-	189	-	194	174	-	-
3514	-	174	-	184	-	201	212
3615	175	-	201	210	-	240 [a]	252
Mean	175.0	173.6	201.0	191.2	179.8	205.8	220.5
SD	-	18.2	-	14.4	6.6	18.8	19.7
N	1	14	1	15	5	10	10
%Diff	-	1.8	-	1.7	8.3	1.1	1.8

[a] [RC:Value Confirmed]

Appendix 6

Individual Body Weights

5550008
Sex: Female Bodyweight (g)

1.1x 10E12 vg Group 3	Day(s) Relative to Start Date						
	28	29	35	42	49	56	63
3501	224	-	236	246	251	261	262
3502	254	-	288	297	305	346	328 [a]
3503	209	-	216	232 [a]	228	241	245
3504	220	206 [a]	-	-	-	-	-
3505	216	193 [a]	-	-	-	-	-
3506	226	208 [a]	-	-	-	-	-
3507	-	-	-	-	-	-	-
3508	-	-	-	-	-	-	-
3509	228 [a]	-	236	258	262	262	278
3510	253	-	256	261	269	281	285
3511	-	-	-	-	-	-	-
3512	-	-	-	-	-	-	-
3513	-	-	-	-	-	-	-
3514	220	204	-	-	-	-	-
3615	265	241	-	-	-	-	-
Mean	231.5	210.4	246.4	258.8	263.0	278.2	279.6
SD	18.9	18.1	27.2	24.2	28.2	40.5	31.1
N	10	5	5	5	5	5	5
%Diff	2.1	5.1	1.0	2.5	1.2	3.6	3.2

[a] [RC:Value Confirmed]

Appendix 6

Individual Body Weights

5550008
Sex: Female Bodyweight (g)

1.1x 10E12 vg Group 3	Day(s) Relative to Start Date			
	70	77	84	91
3501	264	268	268	262
3502	348	340	369 [a]	367
3503	253	250	259	241
3504	-	-	-	-
3505	-	-	-	-
3506	-	-	-	-
3507	-	-	-	-
3508	-	-	-	-
3509	279	282	280	264
3510	287	294	292	281
3511	-	-	-	-
3512	-	-	-	-
3513	-	-	-	-
3514	-	-	-	-
3615	-	-	-	-
Mean	286.2	286.8	293.6	283.0
SD	37.0	34.0	43.9	49.1
N	5	5	5	5
%Diff	2.9	2.4	4.0	5.2

[a] [RC:Value Confirmed]

Appendix 6

Individual Body Weights

5550008
Sex: Female Bodyweight (g)

3.3x 10E12 vg Group 4	Day(s) Relative to Start Date						
	-1	7	8	14	21	28	29
4601	190	207	179 [a]	-	-	-	-
4502	157	171	157	-	-	-	-
4503	166	175	-	202	216	232	-
4504	167	192	-	207	219	222	-
4505	143	159	-	180	203	207	194 [a]
4506	180	197	-	227	247	252	239 [a]
4507	151	164	-	184	197	205	187 [a]
4508	166	183	160 [a]	-	-	-	-
4509	179	202	179 [a]	-	-	-	-
4510	154	180	160 [a]	-	-	-	-
4511	180	196	-	217	223	233	214
4512	196	213	-	238	254	260	242
4513	180	191	-	214	222	227	-
4514	182	199	-	226	238	238	-
4515	182	189	-	206	212	227	-
Mean	171.5	187.9	167.0	210.1	223.1	230.3	215.2
SD	15.3	15.7	11.0	18.4	18.3	17.3	25.2
N	15	15	5	10	10	10	5
%Diff	0.6	-0.1	0.6	3.2	3.0	1.6	7.5

[a] [RC:Value Confirmed]

Appendix 6

Individual Body Weights

5550008
Sex: Female Bodyweight (g)

3.3x 10E12 vg Group 4	Day(s) Relative to Start Date						
	35	42	49	56	63	70	77
4601	-	-	-	-	-	-	-
4502	-	-	-	-	-	-	-
4503	243	259 [a]	258	274	283	293	285
4504	231	238	235	236	243	252	254
4505	-	-	-	-	-	-	-
4506	-	-	-	-	-	-	-
4507	-	-	-	-	-	-	-
4508	-	-	-	-	-	-	-
4509	-	-	-	-	-	-	-
4510	-	-	-	-	-	-	-
4511	-	-	-	-	-	-	-
4512	-	-	-	-	-	-	-
4513	249	253	254	237 [a]	272	275	283
4514	266	260	277	251 [a]	294	299	307
4515	223	231	239	233 [a]	242	244	249
Mean	242.4	248.2	252.6	246.2	266.8	272.6	275.6
SD	16.6	13.0	16.7	17.0	23.5	24.3	24.0
N	5	5	5	5	5	5	5
%Diff	-0.7	-1.7	-2.8	-8.3	-1.5	-2.0	-1.6

[a] [RC:Value Confirmed]

Appendix 6

Individual Body Weights

5550008
Sex: Female Bodyweight (g)

3.3x 10E12 vg Group 4	Day(s) Relative to Start Date	
	84	91
4601	-	-
4502	-	-
4503	298	279
4504	250	237
4505	-	-
4506	-	-
4507	-	-
4508	-	-
4509	-	-
4510	-	-
4511	-	-
4512	-	-
4513	279	274
4514	314	302
4515	258	242
Mean	279.8	266.8
SD	26.8	27.1
N	5	5
%Diff	-0.9	-0.8

Appendix 7

Individual Body Weight Gains (g)

5550008
Sex: Male Bodyweight Gain (Interval)

0<br>vg<br>Group 1	Day(s) Relative to Start Date -1 → 7	7 → 8	7 → 14	14 → 21	21 → 28	28 → 29	28 → 35
1001	52	-26	-	-	-	-	-
1002	53	-30	-	-	-	-	-
1003	58	-	52	39	42	-32	-
1004	55	-	55	35	41	-23	-
1005	45	-	61	49	37	-	42
1006	55	-	66	43	32	-	36
1007	57	-	76	55	49	-	55
1008	34	-	43	44	43	-27	-
1009	34	-	48	47	38	-24	-
1010	50	-	42	52	44	-32	-
1011	53	-	50	55	40	-	21
1012	49	-	53	48	58	-	34
1014	53	-38	-	-	-	-	-
1015	21	-34	-	-	-	-	-
Mean	47.8	-32.0	54.6	46.7	42.4	-27.6	37.6
SD	10.8	5.2	10.5	6.6	7.1	4.3	12.4
N	14	4	10	10	10	5	5

Appendix 7

Individual Body Weight Gains (g)

5550008
Sex: Male Bodyweight Gain (Interval)

0<br>vg<br>Group 1	Day(s) Relative to Start Date						
	35 → 42	42 → 49	49 → 56	56 → 63	63 → 70	70 → 77	77 → 84
1001	-	-	-	-	-	-	-
1002	-	-	-	-	-	-	-
1003	-	-	-	-	-	-	-
1004	-	-	-	-	-	-	-
1005	40	24	30	26	20	11	15
1006	37	19	27	23	14	6	18
1007	55	27	38	26	33	18	19
1008	-	-	-	-	-	-	-
1009	-	-	-	-	-	-	-
1010	-	-	-	-	-	-	-
1011	39	37	26	27	12	8	21
1012	41	25	28	22	13	14	25
1014	-	-	-	-	-	-	-
1015	-	-	-	-	-	-	-
Mean	42.4	26.4	29.8	24.8	18.4	11.4	19.6
SD	7.2	6.6	4.8	2.2	8.7	4.8	3.7
N	5	5	5	5	5	5	5

Appendix 7

Individual Body Weight Gains (g)

5550008
Sex: Male Bodyweight Gain (Interval)

0<br>vg<br>Group 1	Day(s) Relative to Start Date<br>84 → 91
1001	-
1002	-
1003	-
1004	-
1005	-11
1006	-10
1007	-10
1008	-
1009	-
1010	-
1011	-16
1012	-22
1014	-
1015	-
Mean	-13.8
SD	5.2
N	5

Appendix 7

Individual Body Weight Gains (g)

5550008
Sex: Male Bodyweight Gain (Interval)

0.36x 10E12 vg Group 2	Day(s) Relative to Start Date						
	-1 → 7	7 → 8	7 → 14	14 → 21	21 → 28	28 → 29	28 → 35
2001	55	-	65	48	52	-23	-
2002	53	-	57	44	35	-24	-
2103	54	-	49	45	43	-	36
2004	38	-20	-	-	-	-	-
2005	52	-	70	53	45	-	44
2006	48	-	63	51	43	-	45
2007	53	-	69	50	52	-	49
2008	45	-	47	37	51	-	39
2009	42	-33	-	-	-	-	-
2010	51	-30	-	-	-	-	-
2011	43	-	45	44	39	-24	-
2012	36	-	53	56	52	-33	-
2013	41	-	47	47	40	-27	-
2014	42	-26	-	-	-	-	-
2015	32	-27	-	-	-	-	-
Mean	45.7	-27.2	56.5	47.5	45.2	-26.2	42.6
SD	7.2	4.9	9.6	5.4	6.3	4.1	5.1
N	15	5	10	10	10	5	5

Appendix 7

Individual Body Weight Gains (g)

5550008
Sex: Male Bodyweight Gain (Interval)

0.36x 10E12 vg Group 2	Day(s) Relative to Start Date						
	35 → 42	42 → 49	49 → 56	56 → 63	63 → 70	70 → 77	77 → 84
2001	-	-	-	-	-	-	-
2002	-	-	-	-	-	-	-
2103	40	25	25	38	20	14	30
2004	-	-	-	-	-	-	-
2005	45	32	27	26	32	19	26
2006	37	25	28	14	26	13	19
2007	46	23	34	22	25	12	24
2008	36	40	25	26	15	3	22
2009	-	-	-	-	-	-	-
2010	-	-	-	-	-	-	-
2011	-	-	-	-	-	-	-
2012	-	-	-	-	-	-	-
2013	-	-	-	-	-	-	-
2014	-	-	-	-	-	-	-
2015	-	-	-	-	-	-	-
Mean	40.8	29.0	27.8	25.2	23.6	12.2	24.2
SD	4.5	7.0	3.7	8.7	6.4	5.8	4.1
N	5	5	5	5	5	5	5

Appendix 7

Individual Body Weight Gains (g)

5550008
Sex: Male Bodyweight Gain (Interval)

0.36x 10E12 vg Group 2	Day(s) Relative to Start Date
	84 → 91
2001	-
2002	-
2103	-24
2004	-
2005	-4
2006	-14
2007	-15
2008	-13
2009	-
2010	-
2011	-
2012	-
2013	-
2014	-
2015	-
Mean	-14.0
SD	7.1
N	5

Appendix 7

Individual Body Weight Gains (g)

5550008
Sex: Male Bodyweight Gain (Interval)

1.1x 10E12 vg Group 3	Day(s) Relative to Start Date						
	-1 → 7	7 → 8	7 → 14	14 → 21	21 → 28	28 → 29	28 → 35
3001	45	-22	-	-	-	-	-
3002	54	-25	-	-	-	-	-
3003	48	-24	-	-	-	-	-
3004	44	-	51	38	43	-35	-
3005	47	-	60	47	43	-32	-
3006	56	-	60	35	28	-	37
3007	50	-	69	53	42	-	53
3008	41	-	57	54	49	-	30
3009	46	-	47	52	45	-36	-
3010	41	-	51	45	48	-33	-
3011	27	-	39	41	36	-25	-
3012	45	-29	-	-	-	-	-
3013	38	-30	-	-	-	-	-
3014	33	-	51	31	28	-	23
3015	42	-	53	40	37	-	32
Mean	43.8	-26.0	53.8	43.6	39.9	-32.2	35.0
SD	7.5	3.4	8.2	7.9	7.5	4.3	11.2
N	15	5	10	10	10	5	5

Appendix 7

Individual Body Weight Gains (g)

5550008
Sex: Male Bodyweight Gain (Interval)

1.1x 10E12 vg Group 3	Day(s) Relative to Start Date						
	35 → 42	42 → 49	49 → 56	56 → 63	63 → 70	70 → 77	77 → 84
3001	-	-	-	-	-	-	-
3002	-	-	-	-	-	-	-
3003	-	-	-	-	-	-	-
3004	-	-	-	-	-	-	-
3005	-	-	-	-	-	-	-
3006	31	16	19	5	16	21	18
3007	47	35	40	22	29	21	20
3008	44	22	23	24	18	17	14
3009	-	-	-	-	-	-	-
3010	-	-	-	-	-	-	-
3011	-	-	-	-	-	-	-
3012	-	-	-	-	-	-	-
3013	-	-	-	-	-	-	-
3014	25	21	23	11	15	16	5
3015	27	27	22	32	25	4	29
Mean	34.8	24.2	25.4	18.8	20.6	15.8	17.2
SD	10.1	7.2	8.3	10.8	6.1	7.0	8.8
N	5	5	5	5	5	5	5

Appendix 7

Individual Body Weight Gains (g)

5550008
Sex: Male Bodyweight Gain (Interval)

1.1x 10E12 vg Group 3	Day(s) Relative to Start Date 84 → 91
3001	-
3002	-
3003	-
3004	-
3005	-
3006	-19
3007	-14
3008	3
3009	-
3010	-
3011	-
3012	-
3013	-
3014	-5
3015	-22
Mean	-11.4
SD	10.3
N	5

Appendix 7

Individual Body Weight Gains (g)

5550008
Sex: Male Bodyweight Gain (Interval)

3.3x 10E12 vg Group 4	Day(s) Relative to Start Date						
	-1 → 7	7 → 8	7 → 14	14 → 21	21 → 28	28 → 29	28 → 35
4001	40	-24	-	-	-	-	-
4002	49	-22	-	-	-	-	-
4003	50	-	61	40	41	-24	-
4004	66	-	79	73	77	-43	-
4005	39	-	61	46	41	-25	-
4006	31	-23	-	-	-	-	-
4007	36	-23	-	-	-	-	-
4008	54	-32	-	-	-	-	-
4009	34	-	49	33	46	-	38
4010	35	-	33	24	30	-	18
4011	42	-	45	33	43	-	34
4012	31	-	48	39	35	-23	-
4013	36	-	73	51	63	-40	-
4014	36	-	45	32	26	-	25
4015	51	-	44	37	36	-	27
Mean	42.0	-24.8	53.8	40.8	43.8	-31.0	28.4
SD	10.0	4.1	14.3	13.6	15.4	9.7	7.8
N	15	5	10	10	10	5	5

Appendix 7

Individual Body Weight Gains (g)

5550008
Sex: Male Bodyweight Gain (Interval)

3.3x 10E12 vg Group 4	Day(s) Relative to Start Date						
	35 → 42	42 → 49	49 → 56	56 → 63	63 → 70	70 → 77	77 → 84
4001	-	-	-	-	-	-	-
4002	-	-	-	-	-	-	-
4003	-	-	-	-	-	-	-
4004	-	-	-	-	-	-	-
4005	-	-	-	-	-	-	-
4006	-	-	-	-	-	-	-
4007	-	-	-	-	-	-	-
4008	-	-	-	-	-	-	-
4009	25	27	29	28	26	24	4
4010	18	23	7	9	10	4	10
4011	24	27	23	21	7	22	15
4012	-	-	-	-	-	-	-
4013	-	-	-	-	-	-	-
4014	27	26	17	24	17	16	13
4015	37	9	27	20	16	11	14
Mean	26.2	22.4	20.6	20.4	15.2	15.4	11.2
SD	6.9	7.7	8.9	7.1	7.3	8.2	4.4
N	5	5	5	5	5	5	5

Appendix 7

Individual Body Weight Gains (g)

5550008
Sex: Male Bodyweight Gain (Interval)

3.3x 10E12 vg Group 4	Day(s) Relative to Start Date 84 → 91
4001	-
4002	-
4003	-
4004	-
4005	-
4006	-
4007	-
4008	-
4009	-4
4010	-14
4011	-17
4012	-
4013	-
4014	-16
4015	0
Mean	-10.2
SD	7.7
N	5

Appendix 7

Individual Body Weight Gains (g)

5550008
Sex: Female Bodyweight Gain (Interval)

0<br>vg<br>Group 1	Day(s) Relative to Start Date						
	-1 → 7	7 → 8	7 → 14	14 → 21	21 → 28	28 → 29	28 → 35
1501	21	-19	-	-	-	-	-
1502	23	-22	-	-	-	-	-
1503	21	-	18	14	-3	-	17
1504	18	-	16	27	11	-	3
1505	28	-21	-	-	-	-	-
1506	17	-16	-	-	-	-	-
1507	29	-21	-	-	-	-	-
1508	15	-	12	4	10	-13	-
1509	13	-	15	11	15	-19	-
1510	13	-	18	19	12	-	0
1511	20	-	21	9	14	-	17
1512	16	-	8	8	13	-	6
1513	13	-	11	5	17	-25	-
1514	6	-	18	18	3	-12	-
1515	10	-	7	14	10	-20	-
Mean	17.5	-19.8	14.4	12.9	10.2	-17.8	8.6
SD	6.3	2.4	4.7	7.1	6.0	5.4	8.0
N	15	5	10	10	10	5	5

Appendix 7

Individual Body Weight Gains (g)

5550008
Sex: Female Bodyweight Gain (Interval)

0<br>vg<br>Group 1	Day(s) Relative to Start Date<br>35 → 42	42 → 49	49 → 56	56 → 63	63 → 70	70 → 77	77 → 84
1501	-	-	-	-	-	-	-
1502	-	-	-	-	-	-	-
1503	0	10	10	-3	12	6	0
1504	14	15	4	-1	13	2	7
1505	-	-	-	-	-	-	-
1506	-	-	-	-	-	-	-
1507	-	-	-	-	-	-	-
1508	-	-	-	-	-	-	-
1509	-	-	-	-	-	-	-
1510	17	12	7	-8	11	9	-6
1511	5	0	10	17	8	-6	3
1512	7	0	12	7	-8	-2	8
1513	-	-	-	-	-	-	-
1514	-	-	-	-	-	-	-
1515	-	-	-	-	-	-	-
Mean	8.6	7.4	8.6	2.4	7.2	1.8	2.4
SD	6.9	7.0	3.1	9.8	8.7	6.0	5.7
N	5	5	5	5	5	5	5

Appendix 7

Individual Body Weight Gains (g)

5550008
Sex: Female Bodyweight Gain (Interval)

0<br>vg<br>Group 1	Day(s) Relative to Start Date<br>84 → 91
1501	-
1502	-
1503	-9
1504	-23
1505	-
1506	-
1507	-
1508	-
1509	-
1510	-11
1511	-15
1512	-9
1513	-
1514	-
1515	-
Mean	-13.4
SD	5.9
N	5

Appendix 7

Individual Body Weight Gains (g)

5550008
Sex: Female Bodyweight Gain (Interval)

0.36x 10E12 vg Group 2	Day(s) Relative to Start Date						
	-4 → 1	-6 → 1	1 → 7	-1 → 7	7 → 8	7 → 14	14 → 21
2501	-	-	-	18	-	25	35
2502	-	-	-	25	-	33	20
2503	-	-	-	27	-	12	16
2504	-	-	-	28	-	25	26
2505	-	-	-	27	-24	-	-
2506	-	-	-	17	-15	-	-
2507	-	-	-	28	-	29	14
2508	-	-	-	16	-	23	9
2509	-	-	-	24	-	12	24
2510	-	-	-	19	-28	-	-
2511	-	-	-	22	-23	-	-
2512	-	-	-	19	-21	-	-
2613	-	24	18	-	-	19	12
2614	-	23	21	-	-	13	19
2615	9	-	21	-	-	41	-15
Mean	9.0	23.5	20.0	22.5	-22.2	23.2	16.0
SD	-	0.7	1.7	4.5	4.8	9.6	13.2
N	1	2	3	12	5	10	10

Appendix 7

Individual Body Weight Gains (g)

5550008
Sex: Female Bodyweight Gain (Interval)

0.36x 10E12 vg Group 2	Day(s) Relative to Start Date						
	21 → 28	28 → 29	28 → 35	35 → 42	42 → 49	49 → 56	56 → 63
2501	-3	-11	-	-	-	-	-
2502	21	-20	-	-	-	-	-
2503	14	-	-1	18	0	14	-1
2504	13	-	11	9	20	14	-6
2505	-	-	-	-	-	-	-
2506	-	-	-	-	-	-	-
2507	2	-17	-	-	-	-	-
2508	13	-23	-	-	-	-	-
2509	10	-19	-	-	-	-	-
2510	-	-	-	-	-	-	-
2511	-	-	-	-	-	-	-
2512	-	-	-	-	-	-	-
2613	19	-	15	2	14	6	0
2614	17	-	5	6	17	7	9
2615	-2	-	10	12	5	3	13
Mean	10.4	-18.0	8.0	9.4	11.2	8.8	3.0
SD	8.6	4.5	6.2	6.1	8.4	5.0	7.8
N	10	5	5	5	5	5	5

Appendix 7

Individual Body Weight Gains (g)

5550008
Sex: Female Bodyweight Gain (Interval)

0.36x 10E12 vg Group 2	Day(s) Relative to Start Date			
	63 → 70	70 → 77	77 → 84	84 → 91
2501	-	-	-	-
2502	-	-	-	-
2503	13	6	-3	-16
2504	13	-2	4	-20
2505	-	-	-	-
2506	-	-	-	-
2507	-	-	-	-
2508	-	-	-	-
2509	-	-	-	-
2510	-	-	-	-
2511	-	-	-	-
2512	-	-	-	-
2613	11	6	9	-20
2614	-1	17	-5	-20
2615	2	6	-9	-11
Mean	7.6	6.6	-0.8	-17.4
SD	6.6	6.8	7.2	4.0
N	5	5	5	5

Appendix 7

Individual Body Weight Gains (g)

5550008

Sex: Female Bodyweight Gain (Interval)

1.1x 10E12 vg Group 3	Day(s) Relative to Start Date						
	-7 → 1	1 → 7	-1 → 7	7 → 8	7 → 14	14 → 21	21 → 28
3501	-	-	10	-	22	14	11
3502	-	-	20	-	40	21	2
3503	-	-	18	-	16	9	13
3504	-	-	23	-	14	13	13
3505	-	-	24	-	17	14	12
3506	-	-	28	-	12	23	17
3507	-	-	11	-25	-	-	-
3508	-	-	15	-21	-	-	-
3509	-	-	25	-	21	14	1
3510	-	-	6	-	15	16	20
3511	-	-	16	-26	-	-	-
3512	-	-	17	-23	-	-	-
3513	-	-	5	-20	-	-	-
3514	-	-	10	-	17	11	8
3615	26	9	-	-	30	12	13
Mean	26.0	9.0	16.3	-23.0	20.4	14.7	11.0
SD	-	-	7.2	2.5	8.6	4.3	6.0
N	1	1	14	5	10	10	10

Appendix 7

Individual Body Weight Gains (g)

5550008
Sex: Female Bodyweight Gain (Interval)

1.1x 10E12 vg Group 3	Day(s) Relative to Start Date						
	28 → 29	28 → 35	35 → 42	42 → 49	49 → 56	56 → 63	63 → 70
3501	-	12	10	5	10	1	2
3502	-	34	9	8	41	-18	20
3503	-	7	16	-4	13	4	8
3504	-14	-	-	-	-	-	-
3505	-23	-	-	-	-	-	-
3506	-18	-	-	-	-	-	-
3507	-	-	-	-	-	-	-
3508	-	-	-	-	-	-	-
3509	-	8	22	4	0	16	1
3510	-	3	5	8	12	4	2
3511	-	-	-	-	-	-	-
3512	-	-	-	-	-	-	-
3513	-	-	-	-	-	-	-
3514	-16	-	-	-	-	-	-
3615	-24	-	-	-	-	-	-
Mean	-19.0	12.8	12.4	4.2	15.2	1.4	6.6
SD	4.4	12.3	6.7	4.9	15.3	12.3	8.0
N	5	5	5	5	5	5	5

Appendix 7

Individual Body Weight Gains (g)

5550008
Sex: Female Bodyweight Gain (Interval)

1.1x 10E12 vg Group 3	Day(s) Relative to Start Date		
	70 → 77	77 → 84	84 → 91
3501	4	0	-6
3502	-8	29	-2
3503	-3	9	-18
3504	-	-	-
3505	-	-	-
3506	-	-	-
3507	-	-	-
3508	-	-	-
3509	3	-2	-16
3510	7	-2	-11
3511	-	-	-
3512	-	-	-
3513	-	-	-
3514	-	-	-
3615	-	-	-
Mean	0.6	6.8	-10.6
SD	6.0	13.2	6.7
N	5	5	5

Appendix 7

Individual Body Weight Gains (g)

5550008
Sex: Female Bodyweight Gain (Interval)

3.3x 10E12 vg Group 4	Day(s) Relative to Start Date						
	-1 → 7	7 → 8	7 → 14	14 → 21	21 → 28	28 → 29	28 → 35
4601	17	-28	-	-	-	-	-
4502	14	-14	-	-	-	-	-
4503	9	-	27	14	16	-	11
4504	25	-	15	12	3	-	9
4505	16	-	21	23	4	-13	-
4506	17	-	30	20	5	-13	-
4507	13	-	20	13	8	-18	-
4508	17	-23	-	-	-	-	-
4509	23	-23	-	-	-	-	-
4510	26	-20	-	-	-	-	-
4511	16	-	21	6	10	-19	-
4512	17	-	25	16	6	-18	-
4513	11	-	23	8	5	-	22
4514	17	-	27	12	0	-	28
4515	7	-	17	6	15	-	-4
Mean	16.3	-21.6	22.6	13.0	7.2	-16.2	13.2
SD	5.4	5.1	4.7	5.6	5.1	2.9	12.4
N	15	5	10	10	10	5	5

Appendix 7

Individual Body Weight Gains (g)

5550008
Sex: Female Bodyweight Gain (Interval)

3.3x 10E12 vg Group 4	Day(s) Relative to Start Date						
	35 → 42	42 → 49	49 → 56	56 → 63	63 → 70	70 → 77	77 → 84
4601	-	-	-	-	-	-	-
4502	-	-	-	-	-	-	-
4503	16	-1	16	9	10	-8	13
4504	7	-3	1	7	9	2	-4
4505	-	-	-	-	-	-	-
4506	-	-	-	-	-	-	-
4507	-	-	-	-	-	-	-
4508	-	-	-	-	-	-	-
4509	-	-	-	-	-	-	-
4510	-	-	-	-	-	-	-
4511	-	-	-	-	-	-	-
4512	-	-	-	-	-	-	-
4513	4	1	-17	35	3	8	-4
4514	-6	17	-26	43	5	8	7
4515	8	8	-6	9	2	5	9
Mean	5.8	4.4	-6.4	20.6	5.8	3.0	4.2
SD	7.9	8.2	16.2	17.1	3.6	6.6	7.8
N	5	5	5	5	5	5	5

Appendix 7

Individual Body Weight Gains (g)

5550008
Sex: Female Bodyweight Gain (Interval)

3.3x 10E12 vg Group 4	Day(s) Relative to Start Date 84 → 91
4601	-
4502	-
4503	-19
4504	-13
4505	-
4506	-
4507	-
4508	-
4509	-
4510	-
4511	-
4512	-
4513	-5
4514	-12
4515	-16
Mean	-13.0
SD	5.2
N	5

Appendix 8

Individual Food Consumption

5550008

Sex: Male Daily Food Cons Per Animal (g)

0<br>vg<br>Group 1	No. in Cage	Day(s) Relative to Animal Start Date<br>-1 → 7	7 → 14	14 → 21	21 → 28	28 → 35	35 → 42	42 → 49
1001	2	27	-	-	-	-	-	-
1003	2	28	30	30	1	-	-	-
1005	3	28	28	31	33	35	34	29
1008	3	20 E a	30	30	32	-	-	-
1011	2	28	33	33	33	34	32	28
1013	3	19	-	-	-	-	-	-
	Mean	25.9	30.1	30.8	24.6	34.6	33.4	28.6
	SD	4.0	2.1	1.5	15.9	1.0	1.5	0.3
	N	5	4	4	4	2	2	2

E = Exclude

[a] [RC:WRONG HOPPER WEIGHTED; Food Drop on floor, FC:wrong activity]

Appendix 8

Individual Food Consumption

5550008
Sex: Male Daily Food Cons Per Animal (g)

0<br>vg<br>Group 1	No. in Cage	Day(s) Relative to Animal Start Date					
		49 → 56	56 → 63	63 → 70	70 → 77	77 → 84	84 → 90
1001	2	-	-	-	-	-	-
1003	2	-	-	-	-	-	-
1005	3	32	35	32	33	32	30
1008	3	-	-	-	-	-	-
1011	2	31	33	33	29	32	33
1013	3	-	-	-	-	-	-
	Mean	31.4	33.7	32.2	31.1	32.3	31.8
	SD	0.5	1.3	0.5	2.4	0.0	2.0
	N	2	2	2	2	2	2

Appendix 8

Individual Food Consumption

5550008
Sex: Male Daily Food Cons Per Animal (g)

0.36x 10E12 vg Group 2	No. in Cage	Day(s) Relative to Animal Start Date						
		-1 → 7	7 → 14	14 → 21	21 → 28	28 → 35	35 → 42	42 → 49
2001	2	28	30	33	-1 [E a]	-	-	-
2003	1	23	-	-	-	-	-	-
2005	3	27	30	23	31	35	34	28
2009	2	31	-	-	-	-	-	-
2011	3	27	33	32	36	-	-	-
2014	2	26	-	-	-	-	-	-
2103	2	27	32	30	32	36	34	28
	Mean	26.8	31.2	29.3	33.1	35.1	33.7	28.2
	SD	2.1	1.6	4.6	2.3	0.6	0.1	0.3
	N	7	4	4	3	2	2	2
	%Diff	3.4	3.7	-4.7	34.7	1.4	0.9	-1.4

E = Exclude
[a] [FC:negative value]

Appendix 8

Individual Food Consumption

5550008
Sex: Male Daily Food Cons Per Animal (g)

0.36x 10E12 vg Group 2	No. in Cage	Day(s) Relative to Animal Start Date					
		49 → 56	56 → 63	63 → 70	70 → 77	77 → 84	84 → 90
2001	2	-	-	-	-	-	-
2003	1	-	-	-	-	-	-
2005	3	32	34	33	33	34	33
2009	2	-	-	-	-	-	-
2011	3	-	-	-	-	-	-
2014	2	-	-	-	-	-	-
2103	2	33	35	34	30	33	34
	Mean	32.5	34.8	33.7	31.4	33.2	33.7
	SD	0.5	0.9	0.4	2.3	0.5	0.5
	N	2	2	2	2	2	2
	%Diff	3.6	3.2	4.7	0.9	2.6	5.9

Appendix 8

Individual Food Consumption

5550008

Sex: Male Daily Food Cons Per Animal (g)

1.1x 10E12 vg Group 3	No. in Cage	Day(s) Relative to Animal Start Date						
		-1 → 7	7 → 14	14 → 21	21 → 28	28 → 35	35 → 42	42 → 49
3001	3	25	-	-	-	-	-	-
3004	2	25	28	29	31	-	-	-
3006	3	26	28	31	33	34	32	28
3009	3	26	29	31	31	-	-	-
3012	2	30	-	-	-	-	-	-
3014	2	29	34	28	34	33	30	33
Mean		27.0	29.9	29.6	32.5	33.7	30.6	30.7
SD		2.2	2.9	1.3	1.7	0.4	1.5	3.1
N		6	4	4	4	2	2	2
%Diff		3.9	-0.6	-3.7	32.2	-2.8	-8.3	7.1

Appendix 8

Individual Food Consumption

5550008
Sex: Male Daily Food Cons Per Animal (g)

1.1x 10E12 vg Group 3	No. in Cage	Day(s) Relative to Animal Start Date					
		49 → 56	56 → 63	63 → 70	70 → 77	77 → 84	84 → 90
3001	3	-	-	-	-	-	-
3004	2	-	-	-	-	-	-
3006	3	30	34	32	32	31	32
3009	3	-	-	-	-	-	-
3012	2	-	-	-	-	-	-
3014	2	30	25	33	33	32	32
Mean		30.3	29.5	32.4	32.7	31.5	32.4
SD		0.2	5.9	0.8	1.1	0.2	0.1
N		2	2	2	2	2	2
%Diff		-3.6	-12.5	0.5	5.0	-2.4	1.7

Appendix 8

Individual Food Consumption

5550008
Sex: Male Daily Food Cons Per Animal (g)

3.3x 10E12 vg Group 4	No. in Cage	Day(s) Relative to Animal Start Date						
		-1 → 7	7 → 14	14 → 21	21 → 28	28 → 35	35 → 42	42 → 49
4001	2	23	-	-	-	-	-	-
4003	3	26	46	34	-4 [E a]	-	-	-
4006	3	25	-	-	-	-	-	-
4009	3	24	27	27	28	30	28	24
4012	2	26	35	37	38	-	-	-
4014	2	28	29	31	29	30	27	27
Mean		25.4	34.2	32.2	31.5	30.1	27.4	25.5
SD		1.7	8.8	4.0	5.2	0.6	1.3	2.1
N		6	4	4	3	2	2	2
%Diff		-2.1	13.7	4.6	28.4	-13.2	-18.0	-10.8

E = Exclude
[a] [FC:negative value]

Appendix 8

Individual Food Consumption

5550008

Sex: Male Daily Food Cons Per Animal (g)

3.3x 10E12 vg Group 4	No. in Cage	Day(s) Relative to Animal Start Date					
		49 → 56	56 → 63	63 → 70	70 → 77	77 → 84	84 → 90
4001	2	-	-	-	-	-	-
4003	3	-	-	-	-	-	-
4006	3	-	-	-	-	-	-
4009	3	27	29	29	27	29	29
4012	2	-	-	-	-	-	-
4014	2	26	28	28	28	28	28
	Mean	26.3	28.9	28.5	27.4	28.1	28.4
	SD	0.3	0.8	0.9	0.3	0.6	0.6
	N	2	2	2	2	2	2
	%Diff	-16.1	-14.3	-11.5	-12.1	-12.9	-10.6

Appendix 8

Individual Food Consumption

5550008

Sex: Female Daily Food Cons Per Animal (g)

0<br>vg<br>Group 1	No. in Cage	Day(s) Relative to Animal Start Date<br>-1 → 7	7 → 14	14 → 21	21 → 28	28 → 35	35 → 42	42 → 49
1501	2	18	-	-	-	-	-	-
1503	2	20	21	22	20	27	21	19
1505	3	19	-	-	-	-	-	-
1508	2	18	18	17	17	-	-	-
1510	3	18	20	19	20	21	19	16
1513	3	18	18	21	19	-	-	-
	Mean	18.4	19.2	19.7	18.9	24.2	20.2	17.5
	SD	1.0	1.7	2.0	1.2	4.3	1.7	1.9
	N	6	4	4	4	2	2	2

Appendix 8

Individual Food Consumption

5550008
Sex: Female Daily Food Cons Per Animal (g)

0<br>vg<br><br>Group 1	No. in Cage	Day(s) Relative to Animal Start Date<br>49 → 56	56 → 63	63 → 70	70 → 77	77 → 84	84 → 90
1501	2	-	-	-	-	-	-
1503	2	19	17	20	17	20	18
1505	3	-	-	-	-	-	-
1508	2	-	-	-	-	-	-
1510	3	18	20	19	18	18	18
1513	3	-	-	-	-	-	-
	Mean	18.5	18.4	19.4	17.4	19.4	18.1
	SD	0.5	2.4	0.8	0.8	1.4	0.4
	N	2	2	2	2	2	2

Appendix 8

Individual Food Consumption

5550008

Sex: Female Daily Food Cons Per Animal (g)

0.36x 10E12 vg Group 2	No. in Cage	Day(s) Relative to Animal Start Date						
		1 → 7	-1 → 7	7 → 14	14 → 21	21 → 28	28 → 35	35 → 42
2501	2	-	20	23	24	9	-	-
2503	2	-	18	20	22	21	21	22
2505	2	-	18	-	-	-	-	-
2507	3	-	19	21	20	21	-	-
2510	3	-	21	-	-	-	-	-
2613	3	20	-	22	22	21	20	19
	Mean	20.4	19.3	21.7	22.0	18.0	20.2	20.3
	SD	-	1.2	1.2	1.4	5.8	0.7	2.3
	N	1	5	4	4	4	2	2
	%Diff	-	4.9	12.6	11.5	-4.8	-16.7	0.1

Appendix 8

Individual Food Consumption

5550008
Sex: Female Daily Food Cons Per Animal (g)

0.36x 10E12 vg Group 2	No. in Cage	Day(s) Relative to Animal Start Date						
		42 → 49	49 → 56	56 → 63	63 → 70	70 → 77	77 → 84	84 → 90
2501	2	-	-	-	-	-	-	-
2503	2	21	20	15	21	15	18	19
2505	2	-	-	-	-	-	-	-
2507	3	-	-	-	-	-	-	-
2510	3	-	-	-	-	-	-	-
2613	3	20	19	20	17	19	18	18
Mean		20.2	19.2	17.5	19.1	17.2	18.1	18.3
SD		0.9	0.9	2.9	3.1	3.2	0.2	1.0
N		2	2	2	2	2	2	2
%Diff		15.6	3.6	-4.8	-1.4	-1.1	-6.5	1.5

Appendix 8

Individual Food Consumption

5550008

Sex: Female Daily Food Cons Per Animal (g)

1.1x 10E12 vg Group 3	No. in Cage	Day(s) Relative to Animal Start Date -1 → 1	1 → 7	-1 → 7	7 → 14	14 → 21	21 → 28	28 → 35
3501	3	-	-	17	21	21	20	22
3504	3	-	-	18	20	19	-5 E a	-
3507	2	-	-	19	-	-	-	-
3509	2	-	-	18	20	20	20	19
3511	3	-	-	19	-	-	-	-
3514	1	27	20 n=2	-	24 n=2	22 n=2	21 n=2	-
Mean		26.5	20.4	18.3	21.1	20.6	20.6	20.3
SD		-	-	0.7	1.7	1.5	0.8	1.9
N		1	1	5	4	4	3	2
%Diff		-	-	-0.3	9.8	4.5	8.9	-16.0

n = Number of Animals in Cage; E = Exclude

[a] [RC:Value Confirmed NO DATA FLAGS RECORDED AND NO PELLETS LEFT IN BIN, FC:negative value]

Appendix 8

Individual Food Consumption

5550008

Sex: Female Daily Food Cons Per Animal (g)

1.1x 10E12 vg Group 3	No. in Cage	Day(s) Relative to Animal Start Date						
		35 → 42	42 → 49	49 → 56	56 → 63	63 → 70	70 → 77	77 → 84
3501	3	22	18	21	16	20	15	23
3504	3	-	-	-	-	-	-	-
3507	2	-	-	-	-	-	-	-
3509	2	19	16	18	18	17	17	16
3511	3	-	-	-	-	-	-	-
3514	1	-	-	-	-	-	-	-
Mean		20.5	16.8	19.2	17.3	18.8	16.0	19.5
SD		2.7	1.6	2.2	1.2	2.1	0.8	4.3
N		2	2	2	2	2	2	2
%Diff		1.2	-4.1	3.9	-5.6	-2.9	-8.2	0.7

Appendix 8

Individual Food Consumption

5550008
Sex: Female Daily Food Cons Per Animal (g)

1.1x 10E12 vg Group 3	No. in Cage	Day(s) Relative to Animal Start Date 84 → 90
3501	3	21
3504	3	-
3507	2	-
3509	2	18
3511	3	-
3514	1	-
	Mean	19.5
	SD	2.6
	N	2
	%Diff	7.8

Appendix 8

Individual Food Consumption

5550008

Sex: Female Daily Food Cons Per Animal (g)

3.3x 10E12 vg Group 4	No. in Cage	Day(s) Relative to Animal Start Date						
		-1 → 7	7 → 14	14 → 21	21 → 28	28 → 35	35 → 42	42 → 49
4501	1	17	-	-	-	-	-	-
4503	2	17	19	20	19	20	20	18
4505	3	17	20	19	20	-	-	-
4509	2	21	-	-	-	-	-	-
4511	2	24	23	23	21	-	-	-
4513	3	19	20	21	21	21	17	20
4601	2	20	-	-	-	-	-	-
	Mean	19.2	20.6	20.8	20.3	20.6	18.3	18.8
	SD	2.7	1.7	1.3	0.8	1.0	2.2	1.5
	N	7	4	4	4	2	2	2
	%Diff	4.4	7.1	5.4	7.7	-14.9	-9.6	7.6

Appendix 8

Individual Food Consumption

5550008
Sex: Female Daily Food Cons Per Animal (g)

3.3x 10E12 vg Group 4	No. in Cage	Day(s) Relative to Animal Start Date					
		49 → 56	56 → 63	63 → 70	70 → 77	77 → 84	84 → 90
4501	1	-	-	-	-	-	-
4503	2	18	16	19	15	18	20
4505	3	-	-	-	-	-	-
4509	2	-	-	-	-	-	-
4511	2	-	-	-	-	-	-
4513	3	13	19	18	21	21	20
4601	2	-	-	-	-	-	-
	Mean	15.3	17.4	18.5	17.7	19.5	20.2
	SD	3.4	2.6	0.1	4.2	1.6	0.2
	N	2	2	2	2	2	2
	%Diff	-17.4	-5.3	-4.6	1.7	0.4	11.9

Appendix 9

Individual Neurobehavioral Evaluation: Activity

5550008
Sex: Male

0<br>vg<br>Group 1	Week(s) Relative to Start Date	Activity<br>Arousal/ Alertness	Posture/ BodyCarriage	Stereotypy (HC)	Stereotypy (OF)	Rearing	Appearance
1005	4 (P1)	-1	0	0	0	0	0
	13 (P2)	0	0	0	0	0	0
1006	4 (P1)	0	0	0	0	11	0
	13 (P2)	0	0	0	0	3	0
1007	4 (P1)	-1	0	0	0	2	0
	13 (P2)	-1	0	0	0	3	0
1011	4 (P1)	0	0	0	0	12	0
	13 (P2)	0	0	0	0	16	0
1012	4 (P1)	0	0	0	0	15	0
	13 (P2)	0	0	0	0	4	0

Appendix 9

Individual Neurobehavioral Evaluation: Activity

5550008
Sex: Male

0.36x 10E12 vg Group 2	Week(s) Relative to Start Date	Activity					
		Arousal/ Alertness	Posture/ BodyCarriage	Stereotypy (HC)	Stereotypy (OF)	Rearing	Appearance
2103	4 (P1)	0	0	0	0	19	0
	13 (P2)	0	0	0	0	12	0
2005	4 (P1)	0	0	0	0	14	0
	13 (P2)	0	0	0	0	5	0
2006	4 (P1)	0	0	0	0	12	0
	13 (P2)	0	0	0	0	18	0
2007	4 (P1)	1	0	0	0	18	0
	13 (P2)	0	0	0	0	6	0
2008	4 (P1)	0	0	0	0	11	0
	13 (P2)	0	0	0	0	18	0

Appendix 9

Individual Neurobehavioral Evaluation: Activity

5550008

Sex: Male

1.1x 10E12 vg Group 3	Week(s) Relative to Start Date	Activity					
		Arousal/ Alertness	Posture/ BodyCarriage	Stereotypy (HC)	Stereotypy (OF)	Rearing	Appearance
3006	4 (P1)	0	0	0	0	2	0
	13 (P2)	0	0	0	0	16	0
3007	4 (P1)	0	0	0	0	18	0
	13 (P2)	0	0	0	0	16	0
3008	4 (P1)	0	0	0	0	15	0
	13 (P2)	0	0	0	0	9	0
3014	4 (P1)	0	0	0	0	17	0
	13 (P2)	1	0	0	0	17	0
3015	4 (P1)	0	0	0	0	16	0
	13 (P2)	0	0	0	0	5	0

Appendix 9

Individual Neurobehavioral Evaluation: Activity

5550008
Sex: Male

3.3x 10E12 vg Group 4	Week(s) Relative to Start Date	Activity					
		Arousal/ Alertness	Posture/ BodyCarriage	Stereotypy (HC)	Stereotypy (OF)	Rearing	Appearance
4009	4 (P1)	1	0	0	0	23	0
	13 (P2)	0	0	0	0	12	0
4010	4 (P1)	1	0	0	0	22	0
	13 (P2)	1	0	0	0	22	0
4011	4 (P1)	0	0	0	0	16	0
	13 (P2)	0	0	0	0	4	0
4014	4 (P1)	1	0	0	0	24	0
	13 (P2)	1	0	0	0	12	0
4015	4 (P1)	0	0	0	0	14	0
	13 (P2)	1	0	0	0	16	0

Appendix 9

Individual Neurobehavioral Evaluation: Activity

5550008
Sex: Female

0 vg Group 1	Week(s) Relative to Start Date	Activity					
		Arousal/ Alertness	Posture/ BodyCarriage	Stereotypy (HC)	Stereotypy (OF)	Rearing	Appearance
1503	4 (P1)	0	0	0	0	16	0
	13 (P2)	0	0	0	0	12	0
1504	4 (P1)	0	0	0	0	9	0
	13 (P2)	0	0	0	0	12	0
1510	4 (P1)	0	0	0	0	8	0
	13 (P2)	0	0	0	0	7	0
1511	4 (P1)	0	0	0	0	9	0
	13 (P2)	0	0	0	0	8	0
1512	4 (P1)	0	0	0	0	8	0
	13 (P2)	1	0	0	0	19	0

Appendix 9

Individual Neurobehavioral Evaluation: Activity

5550008

Sex: Female

0.36x 10E12 vg Group 2	Week(s) Relative to Start Date	Activity					
		Arousal/ Alertness	Posture/ BodyCarriage	Stereotypy (HC)	Stereotypy (OF)	Rearing	Appearance
2503	4 (P1)	0	0	0	0	15	0
	13 (P2)	0	0	0	0	6	0
2504	4 (P1)	0	0	0	0	7	0
	13 (P2)	0	0	0	0	10	0
2613	4 (P1)	0	0	0	0	19	0
	13 (P2)	2	0	0	0	35	0
2614	4 (P1)	0	0	0	0	10	0
	13 (P2)	0	0	0	0	9	0
2615	4 (P1)	0	0	0	0	20	0
	13 (P2)	1	0	0	0	20	0

Appendix 9

Individual Neurobehavioral Evaluation: Activity

5550008

Sex: Female

1.1x 10E12 vg Group 3	Week(s) Relative to Start Date	Activity					
		Arousal/ Alertness	Posture/ BodyCarriage	Stereotypy (HC)	Stereotypy (OF)	Rearing	Appearance
3501	4 (P1)	0	0	1 [a]	0	15	0
	13 (P2)	0	0	0	0	5	0
3502	4 (P1)	0	0	0	0	14	0
	13 (P2)	0	0	0	0	15	0
3503	4 (P1)	0	0	0	0	13	0
	13 (P2)	1	0	0	0	12	0
3509	4 (P1)	0	0	0	0	14	0
	13 (P2)	1	0	0	0	22	0
3510	4 (P1)	0	0	0	0	15	0
	13 (P2)	0	0	0	0	10	0

[a] [RC:Animal move his head and neck to left and Right]

Appendix 9

Individual Neurobehavioral Evaluation: Activity

5550008

Sex: Female

3.3x 10E12 vg Group 4	Week(s) Relative to Start Date	Activity					
		Arousal/ Alertness	Posture/ BodyCarriage	Stereotypy (HC)	Stereotypy (OF)	Rearing	Appearance
4503	4 (P1)	0	0	0	0	18	0
	13 (P2)	1	0	0	0	17	0
4504	4 (P1)	1	0	0	0	25	0
	13 (P2)	1	0	0	0	17	0
4513	4 (P1)	1	0	0	0	32	0
	13 (P2)	1	0	0	0	26	0
4514	4 (P1)	0	0	0	0	15	0
	13 (P2)	0	0	0	0	9	0
4515	4 (P1)	1	0	0	0	20	0
	13 (P2)	1	0	0	0	28	0

Appendix 10

Individual Neurobehavioral Evaluation: Autonomic

5550008

Sex: Male

0<br>vg<br>Group 1	Week(s) Relative to Start Date	Autonomic<br>Exophthalmus	Lacrimation	Palpebral Closure (HC)	Palpebral Closure (OF)	Erected Fur	Pupil Response
1005	4 (P1)	0	0	0	0	0	0
	13 (P2)	0	0	0	0	0	0
1006	4 (P1)	0	0	0	0	0	0
	13 (P2)	0	0	0	0	0	0
1007	4 (P1)	0	0	0	0	0	0
	13 (P2)	0	0	0	0	0	0
1011	4 (P1)	0	0	0	0	0	0
	13 (P2)	0	0	0	0	0	0
1012	4 (P1)	0	0	0	0	0	0
	13 (P2)	0	0	0	0	0	0

Appendix 10

Individual Neurobehavioral Evaluation: Autonomic

5550008

Sex: Male

0<br>vg<br><br>Group 1	Week(s) Relative to Start Date	Autonomic	
		Salivation	Defecation
1005	4 (P1)	0	0
	13 (P2)	0	0
1006	4 (P1)	0	-1
	13 (P2)	0	0
1007	4 (P1)	0	-1
	13 (P2)	0	0
1011	4 (P1)	0	-1
	13 (P2)	0	-1
1012	4 (P1)	0	0
	13 (P2)	0	-1

Appendix 10

Individual Neurobehavioral Evaluation: Autonomic

5550008
Sex: Male

0.36x 10E12 vg Group 2	Week(s) Relative to Start Date	Autonomic					
		Exophthalmus	Lacrimation	Palpebral Closure (HC)	Palpebral Closure (OF)	Erected Fur	Pupil Response
2103	4 (P1)	0	0	0	0	0	0
	13 (P2)	0	0	0	0	0	0
2005	4 (P1)	0	0	0	0	0	0
	13 (P2)	0	0	0	0	0	0
2006	4 (P1)	0	0	0	0	0	0
	13 (P2)	0	0	0	0	0	0
2007	4 (P1)	0	0	0	0	0	0
	13 (P2)	0	0	0	0	0	0
2008	4 (P1)	0	0	0	0	0	0
	13 (P2)	0	0	0	0	0	0

Appendix 10

Individual Neurobehavioral Evaluation: Autonomic

5550008

Sex: Male

0.36x 10E12 vg Group 2	Week(s) Relative to Start Date	Autonomic	
		Salivation	Defecation
2103	4 (P1)	0	0
	13 (P2)	0	0
2005	4 (P1)	0	-1
	13 (P2)	0	0
2006	4 (P1)	0	0
	13 (P2)	0	-1
2007	4 (P1)	0	-1
	13 (P2)	0	-1
2008	4 (P1)	0	0
	13 (P2)	0	0

Appendix 10

Individual Neurobehavioral Evaluation: Autonomic

5550008

Sex: Male

1.1x 10E12 vg Group 3	Week(s) Relative to Start Date	Autonomic					
		Exophthalmus	Lacrimation	Palpebral Closure (HC)	Palpebral Closure (OF)	Erected Fur	Pupil Response
3006	4 (P1)	0	0	0	0	0	0
	13 (P2)	0	0	0	0	0	0
3007	4 (P1)	0	0	0	0	0	0
	13 (P2)	0	0	0	0	0	0
3008	4 (P1)	0	0	0	0	0	0
	13 (P2)	0	0	0	0	0	0
3014	4 (P1)	0	0	0	0	0	0
	13 (P2)	0	0	0	0	0	0
3015	4 (P1)	0	0	0	0	0	0
	13 (P2)	0	0	0	0	0	0

Appendix 10

Individual Neurobehavioral Evaluation: Autonomic

5550008

Sex: Male

1.1x 10E12 vg Group 3	Week(s) Relative to Start Date	Autonomic	
		Salivation	Defecation
3006	4 (P1)	0	-1
	13 (P2)	0	-1
3007	4 (P1)	0	-1
	13 (P2)	0	-1
3008	4 (P1)	0	0
	13 (P2)	0	-1
3014	4 (P1)	0	-1
	13 (P2)	0	-1
3015	4 (P1)	0	-1
	13 (P2)	0	-1

Appendix 10

Individual Neurobehavioral Evaluation: Autonomic

5550008
Sex: Male

3.3x 10E12 vg Group 4	Week(s) Relative to Start Date	Autonomic					
		Exophthalmus	Lacrimation	Palpebral Closure (HC)	Palpebral Closure (OF)	Erected Fur	Pupil Response
4009	4 (P1)	0	0	0	0	0	0
	13 (P2)	0	0	0	0	0	0
4010	4 (P1)	0	0	0	0	0	0
	13 (P2)	0	0	0	0	0	0
4011	4 (P1)	0	0	0	0	0	0
	13 (P2)	0	0	0	0	0	0
4014	4 (P1)	0	0	0	0	0	0
	13 (P2)	0	0	0	0	0	0
4015	4 (P1)	0	0	0	0	0	0
	13 (P2)	0	0	0	0	0	0

Appendix 10

Individual Neurobehavioral Evaluation: Autonomic

5550008

Sex: Male

3.3x 10E12 vg Group 4	Week(s) Relative to Start Date	Autonomic	
		Salivation	Defecation
4009	4 (P1)	0	-1
	13 (P2)	0	-1
4010	4 (P1)	0	0
	13 (P2)	0	0
4011	4 (P1)	0	0
	13 (P2)	0	0
4014	4 (P1)	0	-1
	13 (P2)	0	0
4015	4 (P1)	0	-1
	13 (P2)	0	-1

Appendix 10

Individual Neurobehavioral Evaluation: Autonomic

5550008

Sex: Female

0 vg Group 1	Week(s) Relative to Start Date	Autonomic					
		Exophthalmus	Lacrimation	Palpebral Closure (HC)	Palpebral Closure (OF)	Erected Fur	Pupil Response
1503	4 (P1)	0	0	0	0	0	0
	13 (P2)	0	0	0	0	0	0
1504	4 (P1)	0	0	0	0	0	0
	13 (P2)	0	0	0	0	0	0
1510	4 (P1)	0	0	0	0	0	0
	13 (P2)	0	0	0	0	0	0
1511	4 (P1)	0	0	0	0	0	0
	13 (P2)	0	0	0	0	0	0
1512	4 (P1)	0	0	0	0	0	0
	13 (P2)	0	0	0	0	0	0

Appendix 10

Individual Neurobehavioral Evaluation: Autonomic

5550008

Sex: Female

0<br>vg<br><br>Group 1	Week(s) Relative to Start Date	Autonomic	
		Salivation	Defecation
1503	4 (P1)	0	-1
	13 (P2)	0	-1
1504	4 (P1)	0	-1
	13 (P2)	0	-1
1510	4 (P1)	0	-1
	13 (P2)	0	-1
1511	4 (P1)	0	-1
	13 (P2)	0	-1
1512	4 (P1)	0	-1
	13 (P2)	0	-1

Appendix 10

Individual Neurobehavioral Evaluation: Autonomic

5550008
Sex: Female

0.36x 10E12 vg Group 2	Week(s) Relative to Start Date	Autonomic					
		Exophthalmus	Lacrimation	Palpebral Closure (HC)	Palpebral Closure (OF)	Erected Fur	Pupil Response
2503	4 (P1)	0	0	0	0	0	0
	13 (P2)	0	0	0	0	0	0
2504	4 (P1)	0	0	0	0	0	0
	13 (P2)	0	0	0	0	0	0
2613	4 (P1)	0	0	0	0	0	0
	13 (P2)	0	0	0	0	0	0
2614	4 (P1)	0	0	0	0	0	0
	13 (P2)	0	0	0	0	0	0
2615	4 (P1)	0	0	0	0	0	0
	13 (P2)	0	0	0	0	0	0

Appendix 10

Individual Neurobehavioral Evaluation: Autonomic

5550008

Sex: Female

0.36x 10E12 vg Group 2	Week(s) Relative to Start Date	Autonomic	
		Salivation	Defecation
2503	4 (P1)	0	-1
	13 (P2)	0	-1
2504	4 (P1)	0	-1
	13 (P2)	0	-1
2613	4 (P1)	0	-1
	13 (P2)	0	-1
2614	4 (P1)	0	-1
	13 (P2)	0	-1
2615	4 (P1)	0	-1
	13 (P2)	0	-1

Appendix 10

Individual Neurobehavioral Evaluation: Autonomic

5550008
Sex: Female

1.1x 10E12 vg Group 3	Week(s) Relative to Start Date	Autonomic					
		Exophthalmus	Lacrimation	Palpebral Closure (HC)	Palpebral Closure (OF)	Erected Fur	Pupil Response
3501	4 (P1)	0	0	0	0	0	0
	13 (P2)	0	0	0	0	0	0
3502	4 (P1)	0	0	0	0	0	0
	13 (P2)	0	0	0	0	0	0
3503	4 (P1)	0	0	0	0	0	0
	13 (P2)	0	0	0	0	0	0
3509	4 (P1)	0	0	0	0	0	0
	13 (P2)	0	0	0	0	0	0
3510	4 (P1)	0	0	0	0	0	0
	13 (P2)	0	0	0	0	0	0

Appendix 10

Individual Neurobehavioral Evaluation: Autonomic

5550008
Sex: Female

1.1x 10E12 vg Group 3	Week(s) Relative to Start Date	Autonomic	
		Salivation	Defecation
3501	4 (P1)	0	-1
	13 (P2)	0	-1
3502	4 (P1)	0	-1
	13 (P2)	0	-1
3503	4 (P1)	0	-1
	13 (P2)	0	-1
3509	4 (P1)	0	-1
	13 (P2)	0	-1
3510	4 (P1)	0	-1
	13 (P2)	0	-1

Appendix 10

Individual Neurobehavioral Evaluation: Autonomic

5550008

Sex: Female

3.3x 10E12 vg Group 4	Week(s) Relative to Start Date	Autonomic					
		Exophthalmus	Lacrimation	Palpebral Closure (HC)	Palpebral Closure (OF)	Erected Fur	Pupil Response
4503	4 (P1)	0	0	0	0	0	0
	13 (P2)	0	0	0	0	0	0
4504	4 (P1)	0	0	0	0	0	0
	13 (P2)	0	0	0	0	0	0
4513	4 (P1)	0	0	0	0	0	0
	13 (P2)	0	0	0	0	0	0
4514	4 (P1)	0	0	0	0	0	0
	13 (P2)	0	0	0	0	0	0
4515	4 (P1)	0	0	0	0	0	0
	13 (P2)	0	0	0	0	0	0

Appendix 10

Individual Neurobehavioral Evaluation: Autonomic

5550008
Sex: Female

3.3x 10E12 vg Group 4	Week(s) Relative to Start Date	Autonomic	
		Salivation	Defecation
4503	4 (P1)	0	-1
	13 (P2)	0	-1
4504	4 (P1)	0	-1
	13 (P2)	0	-1
4513	4 (P1)	0	-1
	13 (P2)	0	-1
4514	4 (P1)	0	-1
	13 (P2)	0	0
4515	4 (P1)	0	-1
	13 (P2)	0	-1

Appendix 11

Individual Neurobehavioral Evaluation: Excitability

5550008
Sex: Male

0 vg Group 1	Week(s) Relative to Start Date	Excitability Vocal-izations	Startle Response	Ease of Removal	Handling Reactivity	Convulsions (HC)	Convulsions (OF)
1005	4 (P1)	0	0	0	0	0	0
	13 (P2)	0	0	0	0	0	0
1006	4 (P1)	0	0	0	0	0	0
	13 (P2)	1	0	0	0	0	0
1007	4 (P1)	0	0	1	1	0	0
	13 (P2)	0	0	0	0	0	0
1011	4 (P1)	0	0	0	0	0	0
	13 (P2)	0	0	0	0	0	0
1012	4 (P1)	0	0	0	0	0	0
	13 (P2)	0	0	0	0	0	0

Appendix 11

Individual Neurobehavioral Evaluation: Excitability

5550008
Sex: Male

0.36x 10E12 vg Group 2	Week(s) Relative to Start Date	Excitability: Vocal-izations	Excitability: Startle Response	Excitability: Ease of Removal	Excitability: Handling Reactivity	Excitability: Convulsions (HC)	Excitability: Convulsions (OF)
2103	4 (P1)	0	0	0	0	0	0
	13 (P2)	0	0	0	0	0	0
2005	4 (P1)	0	0	0	0	0	0
	13 (P2)	0	0	0	0	0	0
2006	4 (P1)	0	0	0	0	0	0
	13 (P2)	0	0	0	0	0	0
2007	4 (P1)	0	0	0	0	0	0
	13 (P2)	0	0	0	0	0	0
2008	4 (P1)	0	0	0	0	0	0
	13 (P2)	0	0	0	0	0	0

Appendix 11

Individual Neurobehavioral Evaluation: Excitability

5550008
Sex: Male

1.1x 10E12 vg Group 3	Week(s) Relative to Start Date	Excitability					
		Vocal-izations	Startle Response	Ease of Removal	Handling Reactivity	Convulsions (HC)	Convulsions (OF)
3006	4 (P1)	0	0	0	0	0	0
	13 (P2)	0	0	0	0	0	0
3007	4 (P1)	0	0	0	0	0	0
	13 (P2)	0	0	0	0	0	0
3008	4 (P1)	0	0	0	0	0	0
	13 (P2)	0	0	0	0	0	0
3014	4 (P1)	0	0	0	0	0	0
	13 (P2)	0	0	0	0	0	0
3015	4 (P1)	0	0	0	0	0	0
	13 (P2)	0	0	0	0	0	0

Appendix 11

Individual Neurobehavioral Evaluation: Excitability

5550008

Sex: Male

3.3x 10E12 vg Group 4	Week(s) Relative to Start Date	Excitability					
		Vocal-izations	Startle Response	Ease of Removal	Handling Reactivity	Convulsions (HC)	Convulsions (OF)
4009	4 (P1)	0	0	0	0	0	0
	13 (P2)	0	0	0	0	0	0
4010	4 (P1)	0	1	1	1	0	0
	13 (P2)	0	0	0	0	0	0
4011	4 (P1)	0	1	0	0	0	0
	13 (P2)	0	0	0	0	0	0
4014	4 (P1)	0	0	0	0	0	0
	13 (P2)	0	0	0	0	0	0
4015	4 (P1)	0	0	0	0	0	0
	13 (P2)	0	0	0	0	0	0

Appendix 11

Individual Neurobehavioral Evaluation: Excitability

5550008

Sex: Female

0<br>vg<br>Group 1	Week(s) Relative to Start Date	Excitability					
		Vocal-izations	Startle Response	Ease of Removal	Handling Reactivity	Convulsions (HC)	Convulsions (OF)
1503	4 (P1)	0	0	0	0	0	0
	13 (P2)	0	0	0	0	0	0
1504	4 (P1)	0	0	0	0	0	0
	13 (P2)	1	0	0	0	0	0
1510	4 (P1)	0	0	0	0	0	0
	13 (P2)	0	0	0	0	0	0
1511	4 (P1)	0	0	0	0	0	0
	13 (P2)	0	0	0	0	0	0
1512	4 (P1)	0	0	0	0	0	0
	13 (P2)	0	0	0	0	0	0

Appendix 11

Individual Neurobehavioral Evaluation: Excitability

5550008
Sex: Female

0.36x 10E12 vg Group 2	Week(s) Relative to Start Date	Excitability					
		Vocal-izations	Startle Response	Ease of Removal	Handling Reactivity	Convulsions (HC)	Convulsions (OF)
2503	4 (P1)	0	0	0	0	0	0
	13 (P2)	0	0	0	0	0	0
2504	4 (P1)	0	0	0	0	0	0
	13 (P2)	0	0	0	0	0	0
2613	4 (P1)	0	0	0	0	0	0
	13 (P2)	0	0	0	0	0	0
2614	4 (P1)	0	0	0	0	0	0
	13 (P2)	0	0	0	0	0	0
2615	4 (P1)	0	0	0	0	0	0
	13 (P2)	0	0	0	0	0	0

Appendix 11

Individual Neurobehavioral Evaluation: Excitability

5550008

Sex: Female

1.1x 10E12 vg Group 3	Week(s) Relative to Start Date	Excitability					
		Vocal-izations	Startle Response	Ease of Removal	Handling Reactivity	Convulsions (HC)	Convulsions (OF)
3501	4 (P1)	0	1	1	0	0	0
	13 (P2)	0	0	0	0	0	0
3502	4 (P1)	0	0	0	0	0	0
	13 (P2)	0	0	0	0	0	0
3503	4 (P1)	0	0	0	0	0	0
	13 (P2)	0	0	0	0	0	0
3509	4 (P1)	0	0	1	0	0	0
	13 (P2)	0	0	0	0	0	0
3510	4 (P1)	0	0	0	0	0	0
	13 (P2)	0	0	0	0	0	0

Appendix 11

Individual Neurobehavioral Evaluation: Excitability

5550008
Sex: Female

3.3x 10E12 vg Group 4	Week(s) Relative to Start Date	Excitability					
		Vocal-izations	Startle Response	Ease of Removal	Handling Reactivity	Convulsions (HC)	Convulsions (OF)
4503	4 (P1)	0	0	0	0	0	0
	13 (P2)	0	0	0	0	0	0
4504	4 (P1)	0	0	0	0	0	0
	13 (P2)	0	0	0	0	0	0
4513	4 (P1)	0	0	0	0	0	0
	13 (P2)	0	0	0	0	0	0
4514	4 (P1)	0	0	0	0	0	0
	13 (P2)	0	0	0	0	0	0
4515	4 (P1)	0	0	0	0	0	0
	13 (P2)	0	0	0	0	0	0

Appendix 12

Individual Neurobehavioral Evaluation: Neuromuscular

5550008

Sex: Male

0<br>vg<br>Group 1	Week(s) Relative to Start Date	Neuromuscular<br>Gait/ Mobility	Forelimb Grp Strength T1 (g)	Forelimb Grp Strength T2 (g)	Forelimb Grp Strength T3 (g)	Forelimb Grip Mean	Hindlimb Grp Strength T1 (g)
1005	4 (P1)	0	864	869	829	854.0	431
	13 (P2)	0	1067	1114	971	1050.7	476
1006	4 (P1)	0	1036	970	996	1000.7	370
	13 (P2)	0	1268	1273	1125	1222.0	659
1007	4 (P1)	0	1117	1169	1141	1142.3	559
	13 (P2)	0	1502	1172	1135	1269.7	713
1011	4 (P1)	0	1057	1048	1053	1052.7	442
	13 (P2)	0	1518	1397	1123	1346.0	610
1012	4 (P1)	0	952	960	1022	978.0	423
	13 (P2)	0	1492	1262	1325	1359.7	704

Appendix 12

Individual Neurobehavioral Evaluation: Neuromuscular

5550008
Sex: Male

0 vg Group 1	Week(s) Relative to Start Date	Neuromuscular Hindlimb Grp Strength T2 (g)	Hindlimb Grp Strength T3 (g)	Hindlimb Grip Mean	Air Righting Reflex	Tremor (HC)	Tremor (OF)
1005	4 (P1)	419	397	415.7	0	0	0
	13 (P2)	506	555	512.3	0	0	0
1006	4 (P1)	470	462	434.0	0	0	0
	13 (P2)	480	687	608.7	0	0	0
1007	4 (P1)	432	516	502.3	0	0	0
	13 (P2)	579	583	625.0	0	0	0
1011	4 (P1)	465	447	451.3	0	0	0
	13 (P2)	644	598	617.3	0	0	0
1012	4 (P1)	443	480	448.7	0	0	0
	13 (P2)	659	624	662.3	0	0	0

Appendix 12

Individual Neurobehavioral Evaluation: Neuromuscular

5550008
Sex: Male

0<br>vg<br>Group 1	Week(s) Relative to Start Date	Neuromuscular<br>Body Tone
1005	4 (P1)	0
	13 (P2)	0
1006	4 (P1)	0
	13 (P2)	0
1007	4 (P1)	0
	13 (P2)	0
1011	4 (P1)	0
	13 (P2)	0
1012	4 (P1)	0
	13 (P2)	0

Appendix 12

Individual Neurobehavioral Evaluation: Neuromuscular

5550008
Sex: Male

0.36x 10E12 vg Group 2	Week(s) Relative to Start Date	Neuromuscular					
		Gait/ Mobility	Forelimb Grp Strength T1 (g)	Forelimb Grp Strength T2 (g)	Forelimb Grp Strength T3 (g)	Forelimb Grip Mean	Hindlimb Grp Strength T1 (g)
2103	4 (P1)	0	916	914	967	932.3	552
	13 (P2)	0	1307	954	1311	1190.7	508
2005	4 (P1)	0	742	777	804	774.3	457
	13 (P2)	0	1165	1193	1072	1143.3	568
2006	4 (P1)	0	1007	1032	1084	1041.0	584
	13 (P2)	0	1032	1124	1220	1125.3	557
2007	4 (P1)	0	824	934	901	886.3	326
	13 (P2)	0	1289	1193	1090	1190.7	489
2008	4 (P1)	0	923	976	1031	976.7	527
	13 (P2)	0	1356	1313	1253	1307.3	600

Appendix 12

Individual Neurobehavioral Evaluation: Neuromuscular

5550008

Sex: Male

0.36x 10E12 vg Group 2	Week(s) Relative to Start Date	Neuromuscular					
		Hindlimb Grp Strength T2 (g)	Hindlimb Grp Strength T3 (g)	Hindlimb Grip Mean	Air Right ing Reflex	Tremor (HC)	Tremor (OF)
2103	4 (P1)	587	543	560.7	0	0	0
	13 (P2)	505	520	511.0	0	0	0
2005	4 (P1)	452	356	421.7	0	0	0
	13 (P2)	512	460	513.3	0	0	0
2006	4 (P1)	447	578	536.3	0	0	0
	13 (P2)	538	600	565.0	0	0	0
2007	4 (P1)	320	356	334.0	0	0	0
	13 (P2)	589	681	586.3	0	0	0
2008	4 (P1)	415	562	501.3	0	0	0
	13 (P2)	644	621	621.7	0	0	0

Appendix 12

Individual Neurobehavioral Evaluation: Neuromuscular

5550008
Sex: Male

0.36x 10E12 vg Group 2	Week(s) Relative to Start Date	Neuromuscular Body Tone
2103	4 (P1)	0
	13 (P2)	0
2005	4 (P1)	0
	13 (P2)	0
2006	4 (P1)	0
	13 (P2)	0
2007	4 (P1)	0
	13 (P2)	0
2008	4 (P1)	0
	13 (P2)	0

Appendix 12

Individual Neurobehavioral Evaluation: Neuromuscular

5550008
Sex: Male

1.1x 10E12 vg Group 3	Week(s) Relative to Start Date	Neuromuscular					
		Gait/ Mobility	Forelimb Grp Strength T1 (g)	Forelimb Grp Strength T2 (g)	Forelimb Grp Strength T3 (g)	Forelimb Grip Mean	Hindlimb Grp Strength T1 (g)
3006	4 (P1)	0	846	784	791	807.0	416
	13 (P2)	0	1073	1110	1098	1093.7	652
3007	4 (P1)	0	782	967	876	875.0	381
	13 (P2)	0	1121	1234	1353	1236.0	692
3008	4 (P1)	0	883	948	975	935.3	386
	13 (P2)	0	1175	1165	1196	1178.7	535
3014	4 (P1)	0	873	862	882	872.3	437
	13 (P2)	0	1193	1229	1056	1159.3	447
3015	4 (P1)	0	803	801	734	779.3	444
	13 (P2)	0	1180	1191	1091	1154.0	591

Appendix 12

Individual Neurobehavioral Evaluation: Neuromuscular

5550008
Sex: Male

1.1x 10E12 vg Group 3	Week(s) Relative to Start Date	Neuromuscular					
		Hindlimb Grp Strength T2 (g)	Hindlimb Grp Strength T3 (g)	Hindlimb Grip Mean	Air Right ing Reflex	Tremor (HC)	Tremor (OF)
3006	4 (P1)	471	404	430.3	0	0	0
	13 (P2)	580	507	579.7	0	0	0
3007	4 (P1)	419	463	421.0	0	0	0
	13 (P2)	624	677	664.3	0	0	0
3008	4 (P1)	407	371	388.0	0	0	0
	13 (P2)	545	644	574.7	0	0	0
3014	4 (P1)	506	454	465.7	0	0	0
	13 (P2)	513	530	496.7	0	0	0
3015	4 (P1)	449	506	466.3	0	0	0
	13 (P2)	637	541	589.7	0	0	0

Appendix 12

Individual Neurobehavioral Evaluation: Neuromuscular

5550008

Sex: Male

1.1x 10E12 vg Group 3	Week(s) Relative to Start Date	Neuromuscular Body Tone
3006	4 (P1)	0
	13 (P2)	0
3007	4 (P1)	0
	13 (P2)	0
3008	4 (P1)	0
	13 (P2)	0
3014	4 (P1)	0
	13 (P2)	0
3015	4 (P1)	0
	13 (P2)	0

Appendix 12

Individual Neurobehavioral Evaluation: Neuromuscular

5550008
Sex: Male

3.3x 10E12 vg Group 4	Week(s) Relative to Start Date	Neuromuscular					
		Gait/ Mobility	Forelimb Grp Strength T1 (g)	Forelimb Grp Strength T2 (g)	Forelimb Grp Strength T3 (g)	Forelimb Grip Mean	Hindlimb Grp Strength T1 (g)
4009	4 (P1)	0	963	1077	1057	1032.3	339
	13 (P2)	0	1298	1409	1380	1362.3	517
4010	4 (P1)	0	973	1050	1045	1022.7	468
	13 (P2)	0	1386	1222	1304	1304.0	623
4011	4 (P1)	0	966	920	968	951.3	383
	13 (P2)	0	1166	1017	1057	1080.0	576
4014	4 (P1)	0	954	858	967	926.3	389
	13 (P2)	0	1265	1539	1132	1312.0	617
4015	4 (P1)	0	1034	1016	1049	1033.0	475
	13 (P2)	0	1229	1245	1323	1265.7	604

Appendix 12

Individual Neurobehavioral Evaluation: Neuromuscular

5550008

Sex: Male

3.3x 10E12 vg Group 4	Week(s) Relative to Start Date	Neuromuscular					
		Hindlimb Grp Strength T2 (g)	Hindlimb Grp Strength T3 (g)	Hindlimb Grip Mean	Air Right ing Reflex	Tremor (HC)	Tremor (OF)
4009	4 (P1)	386	354	359.7	0	0	0
	13 (P2)	644	622	594.3	0	0	0
4010	4 (P1)	455	500	474.3	0	0	0
	13 (P2)	627	570	606.7	0	0	0
4011	4 (P1)	381	361	375.0	0	0	0
	13 (P2)	621	477	558.0	0	0	0
4014	4 (P1)	454	495	446.0	0	0	0
	13 (P2)	668	640	641.7	0	0	0
4015	4 (P1)	495	470	480.0	0	0	0
	13 (P2)	514	672	596.7	0	0	0

Appendix 12

Individual Neurobehavioral Evaluation: Neuromuscular

5550008

Sex: Male

3.3x 10E12 vg Group 4	Week(s) Relative to Start Date	Neuromuscular Body Tone
4009	4 (P1)	0
	13 (P2)	0
4010	4 (P1)	0
	13 (P2)	0
4011	4 (P1)	0
	13 (P2)	0
4014	4 (P1)	0
	13 (P2)	0
4015	4 (P1)	0
	13 (P2)	0

Appendix 12

Individual Neurobehavioral Evaluation: Neuromuscular

5550008
Sex: Female

0<br>vg<br>Group 1	Week(s) Relative to Start Date	Neuromuscular					
		Gait/ Mobility	Forelimb Grp Strength T1 (g)	Forelimb Grp Strength T2 (g)	Forelimb Grp Strength T3 (g)	Forelimb Grip Mean	Hindlimb Grp Strength T1 (g)
1503	4 (P1)	0	777	831	813	807.0	417
	13 (P2)	0	929	870	935	911.3	474
1504	4 (P1)	0	933	941	959	944.3	404
	13 (P2)	0	935	1038	1089	1020.7	504
1510	4 (P1)	0	866	826	933	875.0	409
	13 (P2)	0	992	1042	942	992.0	539
1511	4 (P1)	0	934	1029	937	966.7	445
	13 (P2)	0	1079	1136	1118	1111.0	519
1512	4 (P1)	0	930	897	951	926.0	371
	13 (P2)	0	957	1107	997	1020.3	605

Appendix 12

Individual Neurobehavioral Evaluation: Neuromuscular

5550008

Sex: Female

0 vg Group 1	Week(s) Relative to Start Date	Neuromuscular Hindlimb Grp Strength T2 (g)	Hindlimb Grp Strength T3 (g)	Hindlimb Grip Mean	Air Right ing Reflex	Tremor (HC)	Tremor (OF)
1503	4 (P1)	411	361	396.3	0	0	0
	13 (P2)	333	401	402.7	0	0	0
1504	4 (P1)	393	391	396.0	0	0	0
	13 (P2)	415	463	460.7	0	0	0
1510	4 (P1)	419	384	404.0	0	0	0
	13 (P2)	519	445	501.0	0	0	0
1511	4 (P1)	344	385	391.3	0	0	0
	13 (P2)	461	460	480.0	0	0	0
1512	4 (P1)	395	432	399.3	0	0	0
	13 (P2)	527	535	555.7	0	0	0

Appendix 12

Individual Neurobehavioral Evaluation: Neuromuscular

5550008

Sex: Female

0<br>vg<br><br>Group 1	Week(s) Relative to Start Date	Neuromuscular<br>Body Tone
1503	4 (P1)	0
	13 (P2)	0
1504	4 (P1)	0
	13 (P2)	0
1510	4 (P1)	0
	13 (P2)	0
1511	4 (P1)	0
	13 (P2)	0
1512	4 (P1)	0
	13 (P2)	0

Appendix 12

Individual Neurobehavioral Evaluation: Neuromuscular

5550008
Sex: Female

0.36x 10E12 vg Group 2	Week(s) Relative to Start Date	Neuromuscular					
		Gait/ Mobility	Forelimb Grp Strength T1 (g)	Forelimb Grp Strength T2 (g)	Forelimb Grp Strength T3 (g)	Forelimb Grip Mean	Hindlimb Grp Strength T1 (g)
2503	4 (P1)	0	860	810	927	865.7	394
	13 (P2)	0	1006	896	964	955.3	479
2504	4 (P1)	0	851	874	938	887.7	382
	13 (P2)	0	786	931	973	896.7	477
2613	4 (P1)	0	902	859	948	903.0	398
	13 (P2)	0	969	1061	989	1006.3	452
2614	4 (P1)	0	921	940	928	929.7	336
	13 (P2)	0	997	778	852	875.7	518
2615	4 (P1)	0	926	939	983	949.3	370
	13 (P2)	0	931	968	889	929.3	521

Appendix 12

Individual Neurobehavioral Evaluation: Neuromuscular

5550008

Sex: Female

0.36x 10E12 vg Group 2	Week(s) Relative to Start Date	Neuromuscular					
		Hindlimb Grp Strength T2 (g)	Hindlimb Grp Strength T3 (g)	Hindlimb Grip Mean	Air Right ing Reflex	Tremor (HC)	Tremor (OF)
2503	4 (P1)	403	385	394.0	0	0	0
	13 (P2)	418	430	442.3	0	0	0
2504	4 (P1)	553	420	451.7	0	0	0
	13 (P2)	587	565	543.0	0	0	0
2613	4 (P1)	351	335	361.3	0	0	0
	13 (P2)	400	482	444.7	0	0	0
2614	4 (P1)	360	417	371.0	0	0	0
	13 (P2)	424	498	480.0	0	0	0
2615	4 (P1)	470	419	419.7	0	0	0
	13 (P2)	566	519	535.3	0	0	0

Appendix 12

Individual Neurobehavioral Evaluation: Neuromuscular

5550008

Sex: Female

0.36x 10E12 vg Group 2	Week(s) Relative to Start Date	Neuromuscular Body Tone
2503	4 (P1)	0
	13 (P2)	0
2504	4 (P1)	0
	13 (P2)	0
2613	4 (P1)	0
	13 (P2)	0
2614	4 (P1)	0
	13 (P2)	0
2615	4 (P1)	0
	13 (P2)	0

Appendix 12

Individual Neurobehavioral Evaluation: Neuromuscular

5550008

Sex: Female

1.1x 10E12 vg Group 3	Week(s) Relative to Start Date	Neuromuscular					
		Gait/ Mobility	Forelimb Grp Strength T1 (g)	Forelimb Grp Strength T2 (g)	Forelimb Grp Strength T3 (g)	Forelimb Grip Mean	Hindlimb Grp Strength T1 (g)
3501	4 (P1)	0	898	816	900	871.3	486
	13 (P2)	0	1006	932	938	958.7	544
3502	4 (P1)	0	778	867	864	836.3	450
	13 (P2)	0	986	1271	1125	1127.3	470
3503	4 (P1)	0	974	964	936	958.0	463
	13 (P2)	0	898	1052	908	952.7	327
3509	4 (P1)	0	715	817	737	756.3	272
	13 (P2)	0	789	934	832	851.7	459
3510	4 (P1)	0	921	827	965	904.3	352
	13 (P2)	0	1150	1017	1107	1091.3	457

Appendix 12

Individual Neurobehavioral Evaluation: Neuromuscular

5550008

Sex: Female

1.1x 10E12 vg Group 3	Week(s) Relative to Start Date	Neuromuscular					
		Hindlimb Grp Strength T2 (g)	Hindlimb Grp Strength T3 (g)	Hindlimb Grip Mean	Air Righting Reflex	Tremor (HC)	Tremor (OF)
3501	4 (P1)	463	385	444.7	0	0	0
	13 (P2)	488	410	480.7	0	0	0
3502	4 (P1)	395	430	425.0	0	0	0
	13 (P2)	487	429	462.0	0	0	0
3503	4 (P1)	360	373	398.7	0	0	0
	13 (P2)	411	390	376.0	0	0	0
3509	4 (P1)	345	352	323.0	0	0	0
	13 (P2)	536	495	496.7	0	0	0
3510	4 (P1)	442	421	405.0	0	0	0
	13 (P2)	472	488	472.3	0	0	0

Appendix 12

Individual Neurobehavioral Evaluation: Neuromuscular

5550008

Sex: Female

1.1x 10E12 vg Group 3	Week(s) Relative to Start Date	Neuromuscular Body Tone
3501	4 (P1)	0
	13 (P2)	0
3502	4 (P1)	0
	13 (P2)	0
3503	4 (P1)	0
	13 (P2)	0
3509	4 (P1)	0
	13 (P2)	0
3510	4 (P1)	0
	13 (P2)	0

Appendix 12

Individual Neurobehavioral Evaluation: Neuromuscular

5550008

Sex: Female

3.3x 10E12 vg Group 4	Week(s) Relative to Start Date	Neuromuscular					
		Gait/ Mobility	Forelimb Grp Strength T1 (g)	Forelimb Grp Strength T2 (g)	Forelimb Grp Strength T3 (g)	Forelimb Grip Mean	Hindlimb Grp Strength T1 (g)
4503	4 (P1)	0	883	886	921	896.7	420
	13 (P2)	0	1024	1045	1004	1024.3	406
4504	4 (P1)	0	883	939	981	934.3	372
	13 (P2)	0	964	1086	1036	1028.7	461
4513	4 (P1)	0	752	830	929	837.0	397
	13 (P2)	0	1086	1018	1062	1055.3	486
4514	4 (P1)	0	889	886	882	885.7	419
	13 (P2)	0	809	851	810	823.3	576
4515	4 (P1)	0	788	832	918	846.0	338
	13 (P2)	0	1128	1019	820	989.0	505

Appendix 12

Individual Neurobehavioral Evaluation: Neuromuscular

5550008

Sex: Female

3.3x 10E12 vg Group 4	Week(s) Relative to Start Date	Neuromuscular					
		Hindlimb Grp Strength T2 (g)	Hindlimb Grp Strength T3 (g)	Hindlimb Grip Mean	Air Right ing Reflex	Tremor (HC)	Tremor (OF)
4503	4 (P1)	364	341	375.0	0	0	0
	13 (P2)	438	547	463.7	0	0	0
4504	4 (P1)	464	393	409.7	0	0	0
	13 (P2)	470	474	468.3	0	0	0
4513	4 (P1)	402	419	406.0	0	0	0
	13 (P2)	460	577	507.7	0	0	0
4514	4 (P1)	392	427	412.7	0	0	0
	13 (P2)	608	503	562.3	0	0	0
4515	4 (P1)	295	337	323.3	0	0	0
	13 (P2)	431	478	471.3	0	0	0

Appendix 12

Individual Neurobehavioral Evaluation: Neuromuscular

5550008

Sex: Female

3.3x 10E12 vg Group 4	Week(s) Relative to Start Date	Neuromuscular Body Tone
4503	4 (P1)	0
	13 (P2)	0
4504	4 (P1)	0
	13 (P2)	0
4513	4 (P1)	0
	13 (P2)	0
4514	4 (P1)	0
	13 (P2)	0
4515	4 (P1)	0
	13 (P2)	0

Appendix 13

Individual Neurobehavioral Evaluation: Physiological

5550008
Sex: Male

0<br>vg<br>Group 1	Week(s) Relative to Start Date	Physiological<br>Respiration	Physiological<br>Body Temperature (oC)
1005	4 (P1)	0	37.6
	13 (P2)	0	37.5
1006	4 (P1)	0	38.3
	13 (P2)	0	38.0
1007	4 (P1)	0	37.5
	13 (P2)	0	37.3
1011	4 (P1)	0	38.0
	13 (P2)	0	38.9
1012	4 (P1)	0	37.7
	13 (P2)	0	38.0

Appendix 13

Individual Neurobehavioral Evaluation: Physiological

5550008

Sex: Male

0.36x 10E12 vg Group 2	Week(s) Relative to Start Date	Physiological	
		Respiration	Body Temperature (oC)
2103	4 (P1)	0	38.3
	13 (P2)	0	38.0
2005	4 (P1)	0	37.5
	13 (P2)	0	37.8
2006	4 (P1)	0	37.3
	13 (P2)	0	37.6
2007	4 (P1)	0	37.6
	13 (P2)	0	37.8
2008	4 (P1)	0	38.0
	13 (P2)	0	38.5

Appendix 13

Individual Neurobehavioral Evaluation: Physiological

5550008
Sex: Male

1.1x 10E12 vg Group 3	Week(s) Relative to Start Date	Physiological	
		Respiration	Body Temperature (oC)
3006	4 (P1)	0	38.1
	13 (P2)	0	38.8
3007	4 (P1)	0	38.1
	13 (P2)	0	38.7
3008	4 (P1)	0	38.0
	13 (P2)	0	37.3
3014	4 (P1)	0	38.9
	13 (P2)	0	39.0
3015	4 (P1)	0	38.8
	13 (P2)	0	39.2

Appendix 13

Individual Neurobehavioral Evaluation: Physiological

5550008
Sex: Male

3.3x 10E12 vg Group 4	Week(s) Relative to Start Date	Physiological	
		Respiration	Body Temperature (oC)
4009	4 (P1)	0	38.8
	13 (P2)	0	38.5
4010	4 (P1)	0	38.6
	13 (P2)	0	38.8
4011	4 (P1)	0	38.5
	13 (P2)	0	38.8
4014	4 (P1)	0	38.5
	13 (P2)	0	38.4
4015	4 (P1)	0	39.0
	13 (P2)	0	39.1

Appendix 13

Individual Neurobehavioral Evaluation: Physiological

5550008

Sex: Female

0<br>vg<br><br>Group 1	Week(s) Relative to Start Date	Physiological<br>Respiration	Physiological<br>Body Temperature (oC)
1503	4 (P1)	0	38.9
	13 (P2)	0	39.0
1504	4 (P1)	0	37.8
	13 (P2)	0	39.1
1510	4 (P1)	0	38.4
	13 (P2)	0	38.8
1511	4 (P1)	0	39.0
	13 (P2)	0	39.2
1512	4 (P1)	0	38.2
	13 (P2)	0	38.9

Appendix 13

Individual Neurobehavioral Evaluation: Physiological

5550008

Sex: Female

0.36x 10E12 vg Group 2	Week(s) Relative to Start Date	Physiological	
		Respiration	Body Temperature (oC)
2503	4 (P1)	0	38.3
	13 (P2)	0	38.2
2504	4 (P1)	0	37.6
	13 (P2)	0	39.0
2613	4 (P1)	0	38.7
	13 (P2)	0	39.6
2614	4 (P1)	0	38.8
	13 (P2)	0	39.7
2615	4 (P1)	0	38.9
	13 (P2)	0	39.0

Appendix 13

Individual Neurobehavioral Evaluation: Physiological

5550008

Sex: Female

1.1x 10E12 vg Group 3	Week(s) Relative to Start Date	Physiological	
		Respiration	Body Temperature (oC)
3501	4 (P1)	0	39.3
	13 (P2)	0	39.2
3502	4 (P1)	0	38.6
	13 (P2)	0	38.3
3503	4 (P1)	0	38.3
	13 (P2)	0	39.7
3509	4 (P1)	0	38.7
	13 (P2)	0	39.0
3510	4 (P1)	0	39.1
	13 (P2)	0	39.2

Appendix 13

Individual Neurobehavioral Evaluation: Physiological

5550008

Sex: Female

3.3x 10E12 vg Group 4	Week(s) Relative to Start Date	Physiological	
		Respiration	Body Temperature (oC)
4503	4 (P1)	0	38.4
	13 (P2)	0	38.6
4504	4 (P1)	0	38.8
	13 (P2)	0	38.9
4513	4 (P1)	0	39.2
	13 (P2)	0	39.3
4514	4 (P1)	0	38.8
	13 (P2)	0	38.8
4515	4 (P1)	0	39.0
	13 (P2)	0	39.6

Appendix 14

Individual Neurobehavioral Evaluation: Sensorimotor

5550008

Sex: Male

0<br>vg<br><br>Group 1	Week(s) Relative to Start Date	Sensorimotor<br>Touch Resp/ Tactile Refl	Sensorimotor<br>Tail Pinch Response
1005	4 (P1)	0	0
	13 (P2)	0	0
1006	4 (P1)	0	0
	13 (P2)	0	0
1007	4 (P1)	0	0
	13 (P2)	0	0
1011	4 (P1)	0	0
	13 (P2)	0	0
1012	4 (P1)	0	0
	13 (P2)	0	0

Appendix 14

Individual Neurobehavioral Evaluation: Sensorimotor

5550008
Sex: Male

0.36x 10E12 vg Group 2	Week(s) Relative to Start Date	Sensorimotor	
		Touch Resp/ Tactile Refl	Tail Pinch Response
2103	4 (P1)	0	0
	13 (P2)	0	0
2005	4 (P1)	0	0
	13 (P2)	0	0
2006	4 (P1)	0	0
	13 (P2)	0	0
2007	4 (P1)	0	0
	13 (P2)	0	0
2008	4 (P1)	0	0
	13 (P2)	0	0

Appendix 14

Individual Neurobehavioral Evaluation: Sensorimotor

5550008

Sex: Male

1.1x 10E12 vg Group 3	Week(s) Relative to Start Date	Sensorimotor	
		Touch Resp/ Tactile Refl	Tail Pinch Response
3006	4 (P1)	0	0
	13 (P2)	0	0
3007	4 (P1)	0	0
	13 (P2)	0	0
3008	4 (P1)	0	0
	13 (P2)	0	0
3014	4 (P1)	0	0
	13 (P2)	0	0
3015	4 (P1)	0	0
	13 (P2)	0	0

Appendix 14

Individual Neurobehavioral Evaluation: Sensorimotor

5550008

Sex: Male

3.3x 10E12 vg Group 4	Week(s) Relative to Start Date	Sensorimotor	
		Touch Resp/ Tactile Refl	Tail Pinch Response
4009	4 (P1)	0	0
	13 (P2)	0	0
4010	4 (P1)	1	0
	13 (P2)	0	0
4011	4 (P1)	0	0
	13 (P2)	0	0
4014	4 (P1)	0	0
	13 (P2)	0	0
4015	4 (P1)	0	0
	13 (P2)	0	0

Appendix 14

Individual Neurobehavioral Evaluation: Sensorimotor

5550008
Sex: Female

0<br>vg<br>Group 1	Week(s) Relative to Start Date	Sensorimotor<br>Touch Resp/ Tactile Refl	Sensorimotor<br>Tail Pinch Response
1503	4 (P1)	0	0
	13 (P2)	0	0
1504	4 (P1)	0	0
	13 (P2)	0	0
1510	4 (P1)	0	0
	13 (P2)	0	0
1511	4 (P1)	0	0
	13 (P2)	0	0
1512	4 (P1)	0	0
	13 (P2)	0	0

Appendix 14

Individual Neurobehavioral Evaluation: Sensorimotor

5550008

Sex: Female

0.36x 10E12 vg Group 2	Week(s) Relative to Start Date	Sensorimotor	
		Touch Resp/ Tactile Refl	Tail Pinch Response
2503	4 (P1)	0	0
	13 (P2)	0	0
2504	4 (P1)	0	0
	13 (P2)	0	0
2613	4 (P1)	0	0
	13 (P2)	0	0
2614	4 (P1)	0	0
	13 (P2)	0	0
2615	4 (P1)	0	0
	13 (P2)	0	0

Appendix 14

Individual Neurobehavioral Evaluation: Sensorimotor

5550008

Sex: Female

1.1x 10E12 vg Group 3	Week(s) Relative to Start Date	Sensorimotor	
		Touch Resp/ Tactile Refl	Tail Pinch Response
3501	4 (P1)	0	0
	13 (P2)	0	0
3502	4 (P1)	0	0
	13 (P2)	0	0
3503	4 (P1)	0	0
	13 (P2)	0	0
3509	4 (P1)	0	0
	13 (P2)	0	0
3510	4 (P1)	0	0
	13 (P2)	0	0

Appendix 14

Individual Neurobehavioral Evaluation: Sensorimotor

5550008

Sex: Female

3.3x 10E12 vg Group 4	Week(s) Relative to Start Date	Sensorimotor	
		Touch Resp/ Tactile Refl	Tail Pinch Response
4503	4 (P1)	0	0
	13 (P2)	0	0
4504	4 (P1)	0	0
	13 (P2)	0	0
4513	4 (P1)	0	0
	13 (P2)	0	0
4514	4 (P1)	0	0
	13 (P2)	0	0
4515	4 (P1)	0	0
	13 (P2)	0	0

Title:<br><br>**RODENT NEUROBEHAVIORAL EVALUATION**	SOP Number:<br>HRM-096	Effective Date:<br>07Oct2020
	Version Number:<br>1.0	Site:<br>Harmonized

Management Approval

DocuSigned by:
A. Vick
Signer Name: Andy Vick
Signing Reason: I approve this document
Signing Time: 11-Sep-2020 | 12:58:40 EDT
E0B893B4C327427DA03877A22C8524C1

Andrew Vick, PhD
Regional General Manager &
Corporate Vice President

DocuSigned by:
Michel Provence
Signer Name: Michel Provence
Signing Reason: I approve this document
Signing Time: 11-Sep-2020 | 13:06:53 EDT
7B40A05D53394EF6BD6C64CBA9B45FBB

Michel Provence, PhD
Regional General Manager &
Corporate Vice President

DocuSigned by:
Rebecca Walker Comba
Signer Name: Rebecca Walker Comba
Signing Reason: I approve this document
Signing Time: 11-Sep-2020 | 16:09:22 EDT
5E55538F0A984B2AAB8B196B974C8578

Rebecca Walker-Comba, PhD
Regional General Manager &
Corporate Vice President

DocuSigned by:
Brian Bathgate
Signer Name: Brian Bathgate
Signing Reason: I approve this document
Signing Time: 14-Sep-2020 | 03:56:56 EDT
6858627B7B6641688CAD5A23900C7395

Brian Bathgate BSc, PhD, DSc, FRSB
Corporate Senior Vice President

Additional Management Approval (if required)

Title: RODENT NEUROBEHAVIORAL EVALUATION	SOP Number: HRM-096	Effective Date: 07Oct2020
	Version Number: 1.0	Site: Harmonized

1.0 Purpose

For use with Provantis 10.

The purpose of this standard operating procedure is to detail the procedures and scoring to be used in evaluation of neurobehavioral function in rodents. The overall evaluation consists of six functional domains used to identify neurobehavioral effects as follows.

Activity

Arousal/Alertness	Rearing	Stereotypy
Posture/Body Carriage	Appearance	

Excitability

Vocalizations	Handling Reactivity	Ease of Removal
Startle Response	Convulsions	

Autonomic

Exophthalmus	Palpebral Closure/Ptosis	Pupil Response
Lacrimation	Defecation	Urination (optional)
Erected Fur	Salivation	

Neuromuscular

Gait/Mobility	Body Tone	Air Righting Reflex
Grip Strength	Tremor	

Sensorimotor

Pain Response	Touch Response/Tactile Reflex

Physiological

Respiration	Body Temperature

2.0 Scope

This SOP applies to all trained personnel performing the activities listed in this document.

3.0 Responsibilities

Role	Responsibilities
Study Director	Ensures appropriate endpoints are specified in the study protocol
Observer	Technical staff performing the evaluations; can also enter data into Provantis
Recorder	Technical staff entering data into Provantis

4.0 Definitions/Abbreviations

SOP- Standard Operating Procedure

Title: RODENT NEUROBEHAVIORAL EVALUATION	SOP Number: HRM-096	Effective Date: 07Oct2020
	Version Number: 1.0	Site: Harmonized

5.0 Materials

Stopwatch/timer

Counter

Mechanical clicker

Penlight

Forceps (if applicable)

Grip strength strain meter or inverted screen (if applicable)

Open field arena

Rectal thermometer and lubricating jelly (if applicable)

6.0 Procedures

6.1 Study Preparation

6.1.1 Prior to the neurobehavioral evaluations appropriate personnel will obtain equipment used for the neurobehavioral evaluations. Inspect all equipment prior to use. Document the equipment used in conducting the neurobehavioral evaluations.

6.1.2 Calibrate the grip-strength meters, as needed, as described in a separate SOP.

6.2 General Study Conduct

6.2.1 If the study is performed under blinded conditions, personal protective equipment need not be changed between dosing or handling of animals. Note that when blinding is required, it need not apply to objective measures (e.g., body temperature, grip strength).

6.2.2 The neurobehavioral evaluations will be performed in the order indicated below unless indicated otherwise

6.2.3 The details of body temperature and grip strength measurements are described in separate SOPs.

6.2.4 Enter all data in Provantis™ at the time of measurement. If Provantis™ is unavailable, collect all data on an approved form.

Comments will be added, as needed, to clarify the observations documented in Provantis. In the event that an observation occurs outside of the specified section below (e.g., Tremors observed during the Handling Observations) a comment can be added at that time to describe this observation.

6.2.5 Animals showing abnormalities, pain, distress or discomfort, which are considered not transient in nature or are likely to become more severe, during and after the observations, must be reported to the study director and veterinary staff.

Title:	SOP Number:	Effective Date:
**RODENT NEUROBEHAVIORAL EVALUATION**	HRM-096	07Oct2020
	Version Number: 1.0	Site: Harmonized

6.3 Home Cage Observations:

Perform the assessments of Posture/Body Carriage, Convulsions, Stereotypy, Tremor, and Palpebral Closure/Ptosis prior to removing the animal from the home cage. If necessary, personnel can gently pull the cage from the bank or remove the cage cover in order to see the animals' eyes. Ratings under these sections are as follows:

6.3.1 Posture/Body Carriage:

-2	Prostrate (e.g., limbs in the air) or flattened to cage floor (e.g., limbs spread out)
-1	Hunched or low carriage
0	Normal; Asleep/lying down, sitting or standing normally

6.3.2 Convulsions: Clonic Movements are characterized by alternating contraction and relaxation of musculature. Tonic Movements are characterized by continuous muscular contractions. For scores other than 0, the specific behavior observed will be recorded as a comment.

0	None
1	Clonic movement of limbs, ears, head, or skin, repetitive movements of mouth and jaw; Contraction of extensors such that limbs are rigid, extended
2	Clonic tremors or clonus of the limbs or whole body; Opisthotonus (head and body rigidly arched backwards) or emprosthotonus (head and body rigidly extended forward)
3	Severe clonus of the limbs or whole body; Myoclonic jerks; Explosive jumps into the air with all feet leaving surface
4	Clonic convulsions affecting the whole body; Clonic-tonic convulsions, possibly resulting in dyspnea, or death

6.3.3 Stereotypy: Any behavior that is excessive or repetitive or exceeds that observed in normal animals. For scores other than 0, the specific behavior observed will be recorded as a comment.

0	Not present; Normal
1	Continuous licking, biting/gnawing of the wall or floor, continuous or excessive grooming
2	Repetitive mouth movements, and/or head weaving/bobbing
3	Circling, retropulsion, forepaw treading
4	Self-Injurious behavior; Licking, biting, or gnawing of the body

6.3.4 Tremor: Uncontrollable shaking of all or part of the body. For scores other than 0, the specific body location affected will be recorded as a comment.

0	None
1	Slight (barely perceptible)
2	Moderate (apparent, but still able to perform other activities)
3	Severe (impairs general activity)

6.3.5 Palpebral Closure/Ptosis:

0	Eyelids wide open
1	Eyelids drooping approximately half-way
2	Eyelids completely shut

6.4 Handling Observations:

Perform the assessments of Ease of Removal and Handling Reactivity upon removal of the animal from the home cage. Ratings under these sections are as follows:

6.4.1 Ease of Removal:

-1	Very easy (animal is sluggish or limp)
0	Easy (sits quietly, allows observer to pick it up with minimal resistance)
1	Moderate (e.g., rearing, following observer's hand, vocalization)
2	Difficult (e.g., running around cage, is hard to grab, vocalization, tail and throat rattles, attacking observer's hand)

6.4.2 Handling Reactivity:

-1	Very low (animal is totally limp or otherwise unresponsive)
0	Low (minimal resistance, is easy to handle)
1	Moderate (slight resistance)
2	High (e.g., freezing, tense or rigid in hand, squirming, twisting, or attempting to bite)

6.5 Open Field Observations:

Place the animal in the center of a standard open-field testing box made of opaque material (e.g., black plexiglass). Using a stopwatch or timer, time the animal for 2 minutes. The number of rearing counts will be recorded after the completion of the 2-minute observation period. The assessments of Arousal/Alertness, Gait/Mobility, Vocalization, Tremor, Respiration, Defecation, Urination (optional), Stereotypy, Convulsions, Appearance, Lacrimation, Salivation, Exophthalmus, Palpebral Closure/Ptosis and Erected Fur will be performed after the 2 minutes have ended.

If an observation defined in another section of this SOP (e.g., palpebral closure) is not present under those conditions (e.g., Home Cage or Handling Observations), but is present during the Open Field Observations, this can be noted as an unscheduled clinical observation.

Clean the open field-testing box as necessary after each animal is evaluated ensuring that all feces are removed, and the surface is wiped dry.

Counts and ratings under this section are as follows:

6.5.1 Rearing: The number of times an animal rears onto its hindlimbs with both forelimbs raised off the surface of the open field box is recorded over the 2-minute observation period. A rear can be brief or low raising of both forelimbs. A rear in which the animal supports itself on the side of the open field box with one or both forelimbs is also counted.

Title: RODENT NEUROBEHAVIORAL EVALUATION	SOP Number: HRM-096	Effective Date: 07Oct2020
	Version Number: 1.0	Site: Harmonized

6.5.2 Arousal/Alertness: The level of unprovoked activity is assessed

-2	Very low (stuporous, comatose, little to no movement)
-1	Low (sluggish, some exploratory movement)
0	Normal (alert with exploratory movement)
1	High (tense, some bouts of startle, darting, or freezing without stimuli)
2	Very High (excited, multiple bouts of startle, running, darting, freezing, or body movements without stimuli)

6.5.3 Gait/Mobility: If the animal does not move during the 2-minute observation period, it can be gently prodded in order to observe the gait of the animal.

0	Normal gait/mobility
1	Slightly impaired (any or all of the following can be evident): mild ataxia, rocks or lurches during ambulation; hunched or crouched body position; walks on tiptoe
2	Moderately impaired (any or all of the following can be evident): marked ataxia; feet point outwards from the body; hind limbs show exaggerated or overcompensated movements, drag, or are splayed
3	Severely impaired (any or all of the following can be evident): Forelimbs drag or are unable to support weight; body drags or is flattened against surface

6.5.4 Vocalizations:

0	Spontaneous vocalizations occur once or not at all
1	Spontaneous vocalizations occur more than once

6.5.5 Tremor: Uncontrollable shaking of all or part of the body. For scores other than 0, the specific body location affected will be recorded as a comment.

0	None
1	Slight (barely perceptible)
2	Moderate (apparent, but still able to perform other activities)
3	Severe (impairs general activity)

6.5.6 Respiration: For scores of 2, the specific respiratory character (e.g., labored or shallow breathing, rales, gasping) will be recorded as a comment.

-1	Slowed
0	Normal
1	Rapid
2	Abnormal

6.5.7 Defecation: The appearance of the feces is noted. For scores of 1, the specific appearance will be documented as a comment.

-1	Absent
0	Normal
1	Abnormal

Title: RODENT NEUROBEHAVIORAL EVALUATION	SOP Number: HRM-096	Effective Date: 07Oct2020
	Version Number: 1.0	Site: Harmonized

6.5.8 Urination (optional, as directed by protocol): The appearance of the urine is noted. For scores of 1, the specific appearance will be documented as a comment.

-1	Absent
0	Normal
1	Abnormal

6.5.9 Stereotypy: Any behavior that is excessive or repetitive or exceeds that observed in normal animals. For scores other than 0, the specific behavior observed will be recorded as a comment.

0	Not present; Normal
1	Continuous licking, biting/gnawing of the wall or floor, continuous or excessive grooming
2	Repetitive mouth movements, and/or head weaving/bobbing
3	Circling, retropulsion, forepaw treading
4	Self-Injurious behavior; Licking, biting, or gnawing of the body

6.5.10 Convulsions: Clonic Movements are characterized by alternating contraction and relaxation of musculature. Tonic Movements are characterized by continuous muscular contractions. For scores other than 0, the specific behavior observed will be recorded as a comment.

0	None
1	Clonic movement of limbs, ears, head, or skin, repetitive movements of mouth and jaw; Contraction of extensors such that limbs are rigid, extended
2	Clonic tremors or clonus of the limbs or whole body; Opisthotonus (head and body rigidly arched backwards) or emprosthotonus (head and body rigidly extended forward)
3	Severe clonus of the limbs or whole body; Myoclonic jerks; Explosive jumps into the air with all feet leaving surface
4	Clonic convulsions affecting the whole body; Clonic-tonic convulsions, possibly resulting in dyspnea, or death

6.5.11 Appearance:

0	Clean and groomed
1	Slightly unkempt (slightly discolored / crusty fur)
2	Extremely unkempt (markedly discolored, matted, and/or crusty fur)

6.5.12 Lacrimation:

0	None (no excess lacrimation)
1	Slight (excess moisture at the margin of the eyelid)
2	Severe (dampness extends beyond the margin of the eyelid)

6.5.13 Salivation:

0	None (no excess salivation)
1	Slight (margin of mouth wet)
2	Severe (wet zone extends beyond the margin of the mouth)

Title: RODENT NEUROBEHAVIORAL EVALUATION	SOP Number: HRM-096	Effective Date: 07Oct2020
	Version Number: 1.0	Site: Harmonized

6.5.14 Exophthalmus:

0	Not present
1	Present

6.5.15 Palpebral Closure/Ptosis:

0	Eyelids wide open
1	Eyelids drooping approximately half-way
2	Eyelids completely shut

6.5.16 Erected Fur:

0	Not present
1	Present (coat does not lie down after stroking)

6.6 Sensorimotor Observations: Perform the Touch Response/Tactile Reflex, Startle Response, Pain Response and Pupil Response assessments after the 2-minute time period is over and the previous measurements have been recorded. Remove the animal from the open field apparatus for the Pain Response (if necessary) and Pupil Response assessments. Measurements and rating under this section are as follows:

6.6.1 Touch Response/Tactile Reflex: The rump of the animal is gently touched with a blunt object, such as a pen or a soft-end plastic rod or equivalent.

-1	No reaction
0	Normal reaction (e.g., turns towards or away from stimulus)
1	Exaggerated reaction (freezes or flinches, jumps, bites, or attacks)

6.6.2 Startle Response: A mechanical clicker is placed approximately 5 cm above the back of the animal and a click is sounded suddenly.

-1	No reaction
0	Normal reaction (some evidence that the noise was heard; e.g., normal freezing, flinching, or jumping behaviors)
1	Exaggerated reaction (e.g., excessive jumping, attacking, or vocalization)

6.6.3 Pain Response: One or more of the following (Tail Pinch Response, Thermal Response, and/or Tail Flick Assessment) will be performed to assess the response to pain, as directed by protocol:

Tail Pinch Response: Gently squeeze the tail with forceps approximately 2-3 cm from the tip.

-1	No reaction
0	Normal reaction (some evidence that the pinch was noted e.g., normal flinching, jumping behaviors or vocalization)
1	Exaggerated reaction (e.g., excessive jumping, attacking, or vocalization)

Title:<br><br>**RODENT NEUROBEHAVIORAL EVALUATION**	SOP Number:<br>HRM-096	Effective Date:<br>07Oct2020
	Version Number:<br>1.0	Site:<br>Harmonized

Thermal Response: Place the animal on a hot plate with the temperature set to 52°C (±1.0 degrees or other temperature as required by protocol) and close the top of the plastic enclosure. Start the timer when the feet of the rodent touch the hot plate. Once a response is emitted or the maximum allowable duration has been reached, stop the timer and record the time in seconds to two decimal places in Provantis™ or on an approved form. An animal can exhibit one or more of the following behaviors or escape responses: licking of the fore or hind paws, an increase in ambulatory activity, rapidly repeated elevations of either hind paw, and/or jumping. If an animal exhibits an unusual behavior or escape response, such as clinging to the top of the enclosure, this too can be considered a response and the timer will be stopped. A comment can be recorded to explain the behavior.

The maximum allowable durations on the hotplate per species are as follows (unless otherwise specified by the protocol):

Rats: 60.00 seconds

Mice: 30.00 seconds

Tail Flick Assessment: Tail flick will be performed using the Ugo Basile Tail Flick apparatus (or similar).

Calibration of the tail flick apparatus must be completed using a Heat-Flux I.R. radiometer before each testing session. To calibrate, fit the tail flick adapter onto the I.R. window on the tail flick unit. Switch on the Heat-Flux Meter. Switch on the Tail Flick unit and set the desired I.R. intensity value. The meter will indicate the “stand-by” power level ca 0 to 50 mW/cm2. Press the START key. The reading will settle in a few seconds. Record the power flux reading. An acceptable reading is 260 mW/cm2 ± 10 mW/cm2. If the reading is outside the acceptable limits adjust the I.R. intensity on the tail flick unit until an acceptable reading is reached. Refer to instruction manual for method of adjustment. Record the results in the relevant space in the study log.

For measurement of pain perception, hold the animal securely on the platform of the testing device with its tail free. Position the tail over the infra-red-light source approximately 2 cm from the base of the tail. It is important that the animal does not struggle during the trial or false readings will be obtained. When the animal is settled, trigger the start of the trial using the footswitch. Record the reaction time displayed on the tail flick unit. Important: If the animal has not responded within 10 sec (according to the display on the testing platform), remove it from the light source and do not retest at the same site. This result is recorded as 10. If a false reading has been obtained because of accidental movement or struggling of the animal, repeat the trial ca 1 cm away from the preceding site. If the animal repeatedly struggles, the tail must be monitored carefully and if damage becomes evident the test must be abandoned and recorded as such.

6.6.4 Pupil Response: The animal's eyes are covered by placing a hand over the head or the animal is placed in a dark location for a minimum of 5 seconds. The beam from a pen light is brought in from the side of the head to near the surface of the eye and the effect on pupil size is noted. This procedure is repeated for each eye. If a response is noted in only one eye, this will be documented as a comment.

0	Normal constriction of pupils
1	Partial, slow, or lack of constriction of pupils
2	Pupils are fully constricted prior to performing the test (i.e., "pinpoint pupils")

6.7 Body Temperature: Measure/record the body temperature of the animal using either an implanted microchip or rectal thermometer.

6.8 Neuromuscular Observations: Perform Body Tone, Grip Strength/Response, and Air Righting Reflex. Measurements and rating under this section are as follows:

Body Tone: The animal is grasped with one hand while the other hand gently palpates the lower thorax and pelvic area to evaluate body tone. Body tone can also be evaluated by raising the animal off the surface in a vertical position such that the observer can evaluate the presence/strength of the extensor thrust reflex via digital palpation by gently pressing into the middle of the plantar surface (i.e. footpads) of each hind limb. Either method can be used.

-1	Decreased (flaccidity)
0	Normal
1	Increased (resistance to palpation; rigidity)

6.8.1 Grip Strength/Response Measurement: One or more of the following will be performed to assess the grip strength of the animal, as directed by protocol.

For Rats and/or Mice: Test animals using of a digital strain gauge with metal grasping bars/grid. Forelimb grip strength (and hindlimb, if directed by protocol) is measured. Obtain and record three readings for the forelimbs and three readings for the hindlimbs (if performed).

For Mice (Optional): Test animals by the use of an inverted screen. Place the mouse on the top of a screen that is then inverted. Record the total time, to a maximum of 20 seconds that the animal remains (hanging) on the screen. If the mouse climbs to the top of the screen, terminate the trial, and assign that animal a score of 20 seconds.

For Rats and Mice: If the animal is paralyzed or severely affected, this test will not be conducted, and a comment will be recorded to explain the reason for the lack of testing.

6.8.2 Air Righting Reflex: Hold the animal supine, and then drop from a height of approximately 30 cm. If the animal is paralyzed or severely impaired, this test will not be conducted, and the animal will be given a score of "2".

0	Normal (animal lands on four feet)
1	Abnormal
2	Animal severely impaired (not tested)

Title: **RODENT NEUROBEHAVIORAL EVALUATION**	SOP Number: HRM-096	Effective Date: 07Oct2020
	Version Number: 1.0	Site: Harmonized

7.0 Reference

Alshahrani, S., Ferndandez-Conti, F., Agaujo, A., DiFulvio, M. Rapid determination of the thermal nociceptive threshold in diabetic rats. Journal of Visual Experiments, 17, e3785, 2012.

Ankier, S. New hot plate tests to quantify anti-nociceptive and narcotic antagonist activities. European Journal of Pharmacology, 27, 1-4, 1974.

Meyer, O., Tilson, H., Byrd, W. and Riley, M. A method for the routine assessment of fore- and hind limb grip strength of rats and mice. Neurobehavioral Toxicology, 1, 233-236, 1979.

Moser V., Ross J. US EPA/AIHC training video and reference manual for a functional observational battery. Washington, D.C.: US Environmental Protection Agency, 1996.

Redfern W., Dymond A., Strang I., Storey S., Grant C., Marks L. Barnard C, Heys C., Moyser K., Greenwood K., Cobey D., Moore N., Karp N., Prior H. The functional observational battery and modified Irwin test as global neurobehavioral assessments in the rat: Pharmacological validation data and a comparison of methods. Journal of Pharmacological and Toxicological Methods, 98, 2019.

Tegeris, J. and Balster, R. A Comparison of the acute behavioral effects of alkylbenzene using a functional observational battery in mice. Fundamental and Applied Toxicology, 22, 240-250, 1994.

8.0 Revision History

Version	Effective Date	Reason For Revision
1.0	07Oct2020	SOP moved to new Harmonized template<br>Added optional urination evaluation.

FINAL REPORT

Study Phase: Clinical Pathology

Test Facility Study No. 5550008

Sponsor Reference No. UTSW.Gray-003

GLP

TEST FACILITY:
Charles River Laboratories Montreal ULC
Senneville Site (CR-SEN)

TABLE OF CONTENTS

LIST OF TABLES ... 3
LIST OF APPENDICES ... 4
1. SUMMARY ... 5
2. INTRODUCTION ... 5
3. MATERIALS AND METHODS ... 5
3.1. Experimental Design ... 5
3.2. Computerized Systems ... 6
4. RESULTS AND DISCUSSIONS ... 6
4.1. Mortality ... 6
4.2. Hematology ... 7
4.3. Coagulation ... 7
4.4. Clinical Chemistry ... 8
4.5. Urinalysis ... 8
5. CONCLUSIONS ... 10
6. REPORT APPROVAL ... 11

LIST OF TABLES

Table 1 Summary of Hematology Values 12
Table 2 Summary of Coagulation Values 30
Table 3 Summary of Clinical Chemistry Values..36
Table 4 Summary of Urinalysis Values 54

LIST OF APPENDICES

Appendix 1 Individual Hematology Values 71
Appendix 2 Individual Coagulation Values 95
Appendix 3 Individual Clinical Chemistry Values 103
Appendix 4 Individual Urinalysis Values 135

1. SUMMARY

Four groups of rat were administered AAV9/AP4M1 via single intrathecal injection at doses of 0 (Reference Item), 0.36 x 10^{12}, 1.1 x 10^{12}, or 3.3 x 10^{12} vg. Blood and urine samples were collected for the evaluation of clinical pathology parameters (hematology, coagulation, clinical chemistry and urinalysis). Animals designated for the terminal necropsy (5/sex/group) were euthanized on Day 8, and animals designated for the recovery necropsy (5/sex/group) were euthanized on Day 29 and Day 91.

Administration of AAV9/AP4M1 to rats when given by single intrathecal injection at doses of 0.36 x 10^{12}, 1.1 x 10^{12} or 3.3 x 10^{12} vg elicited minimal to mild clinical pathology changes limited to increases in alanine aminotransferase activity in females at ≥ 0.36 x 10^{12} vg and males at ≥ 1.1 x 10^{12} vg, and increases in fibrinogen in 2/5 males at 3.3 x 10^{12} vg at the end of the main study. During the recovery period, increases in neutrophils and total white blood cells were seen in both sexes at 3.3 x 10^{12} vg and increases in glucose were noted in males only at 3.3 x 10^{12} vg.

2. INTRODUCTION

This report presents the pathology findings in rats assigned to Study No. 5550008. The objective of this study was to characterize the toxicity, biodistribution, and gene expression of AAV9/AP4M1, for the treatment of Spastic Paraplegia Type 50 (SPG50) caused by the AP4M1 gene mutation, when given by a single intrathecal injection to rats and evaluated the potential reversibility and/or progression of any findings.

This study phase was started on 17 Feb 2021 and completed on 23 May 2021.

3. MATERIALS AND METHODS

3.1. Experimental Design

Experimental procedures applicable to clinical pathology are summarized in Text Table 1.

Text Table 1
Experimental Design

Group No.	Test Material	Dose Level (vg)	Dose Volume (µL)	Dose Concentration (vg/µL)	No. of Animals					
					Main Study		Recovery Study			
					Day 8 Necropsy[a]		Day 29 Necropsy[b]		Day 91 Necropsy[c]	
					M	F	M	F	M	F
1	Reference Item	0	60	0	5	5	5	5	5	5
2	AAV9/AP4M1	0.36 x 10^{12}	20	0.18 x 10^{11}	5	5	5	5	5	5
3	AAV9/AP4M1	1.1 x 10^{12}	20	0.55 x 10^{11}	5	5	5	5	5	5
4	AAV9/AP4M1	3.3 x 10^{12}	60	0.55 x 10^{11}	5	5	5	5	5	5

M = Males; F = Females.
[a] Animals scheduled for Necropsy on Day 8.
[b] Animals scheduled for Necropsy on Day 29.
[c] Animals scheduled for Necropsy on Day 91.

Text Table 2
Clinical Pathology Evaluation

Group Nos.	Occasion/ Time Point	Hematology	Coagulation	Clinical Chemistry	Urinalysis
1 to 4	Day 8	X	X	X	X
1 to 4	Day 29	X	X	X	X
1 to 4	Day 91	X	X	X	X
Unscheduled euthanasia (when possible)		X	X	X	-

X = Sample collected.

3.2. Computerized Systems

Critical computerized systems used in this study phase are listed in Text Table 3.

Text Table 3
Computerized Systems

System Name	Version No.	Description of Data Collected and/or Analyzed
Provantis®	10	Hematology, coagulation, clinical chemistry and urinalysis<br>Statistical analyses of numerical in-life and terminal data.
ADVIA 120 Analyzer	6.9.0 MS	Hematology parameters
Cobas 6000 c501 Analyzer	06-01	Clinical biochemistry parameters (serum)
STA Compact Stago Analyzer	108.06	Coagulation parameters
Clinitek NOVUS	1.3.3	Urinalysis parameters
Deviation Information Library	2.1	Deviations
Share Document Management System	1.0	Reporting
M-Files®	21.1	Reporting and collection of 21 CFR Part 11 compliant signature
Docusign™	19	Collection of 21 CFR Part 11 compliant signature

4. RESULTS AND DISCUSSIONS

For the purpose of this report, treated animals' values were compared to control (or reference item) animals' values. Fold change (x) in clinical pathology parameters (except urine specific gravity) was determined by comparing the AAV9/AP4M1 group mean to the respective control group mean unless otherwise noted.

4.1. Mortality

One control male (Animal No. 1013) underwent an unscheduled euthanasia on Day 7. Laboratory investigations (hematology, coagulation, and clinical chemistry) were performed prior to its unscheduled death. Only the complete coagulation panel and a few clinical chemistry parameters were generated. Clinical pathology alterations were non-conclusive and included mild to moderate increases in albumin, sodium, chloride, potassium, and fibrinogen. These changes were non-specific and may have been secondary to hemoconcentration, real or secondary to sample evaporation (increases in albumin, sodium, chloride, potassium, and fibrinogen) or inflammation (increase in fibrinogen).

4.2. Hematology

(Table 1 and Appendix 1)

Administration of AAV9/AP4M1 to rats was associated with changes in hematology parameters at 3.3 x 10^{12} vg. These changes, presented in the following Text Table 4, occurred neutrophil counts (NEUT) and total white blood cell counts (WBC).

Text Table 4
AAV9/AP4M1-Related Hematology Changes

Group	2		3		4	
Dose (vg)	0.36 x 10^{12}	0.36 x 10^{12}	1.1 x 10^{12}	1.1 x 10^{12}	3.3 x 10^{12}	3.3 x 10^{12}
Sex	M	F	M	F	M	F
**NEUT**						
Day 8	–	–	–	–	–	–
Day 29	–	–	–	–	–	–
Day 91	–	–	–	–	**1.87x**	1.45x
**WBC**						
Day 8	–	–	–	–	–	–
Day 29	–	–	–	–	–	–
Day 91	–	–	–	–	1.31x	1.29x

M = Males F = Females.
A dash (—) indicates absence of change. Numerical values indicate fold changes of treated group value relative to control group mean value. Bolded values indicate mean values were statistically different from controls at $P \leq 0.05$.

There were minimal to mild increases in NEUT and WBC in both sexes at 3.3 x 10^{12} vg at the end of the recovery on Day 91.

All differences in hematology parameters, regardless of statistical significance, were not considered AAV9/AP4M1-related based on their small magnitude, inconsistent direction, absence of a dose response, general overlap of individual values with the range of control and baseline values, and/or were of a magnitude of change commonly observed in rats under similar study conditions.

4.3. Coagulation

(Table 2 and Appendix 2)

Administration of AAV9/AP4M1 to rats was associated with minimal changes in coagulation parameters limited to increases in fibrinogen (1.18x control group mean) in 2/5 males at 3.3 x 10^{12} vg (Animal Nos. 4006 and 4007) at the end of the main study on Day 8.

Remaining differences in coagulation parameters were not considered AAV9/AP4M1-related based on their small magnitude, inconsistent direction, absence of a dose response, general overlap of individual values with the range of control and baseline values, and/or were of a magnitude of change commonly observed in rats under similar study conditions.

4.4. Clinical Chemistry

(Table 3 and Appendix 3)

Administration of AAV9/AP4M1 to rats was associated with changes in clinical chemistry parameters at ≥ 0.36 x 10^{12} vg. These changes, presented in the following Text Table 5, occurred in alanine aminotransferase activity (ALT) and glucose (GLUC) concentrations.

Text Table 5
AAV9/AP4M1-Related Clinical Chemistry Changes

**Group**	**2**		**3**		**4**	
**Dose (vg)**	**0.36 x 10^{12}**	**0.36 x 10^{12}**	**1.1 x 10^{12}**	**1.1 x 10^{12}**	**3.3 x 10^{12}**	**3.3 x 10^{12}**
**Sex**	**M**	**F**	**M**	**F**	**M**	**F**
**ALT**						
Day 8	–	**1.41x**	**1.23x**	**1.38x**	**1.25x**	**1.56x**
Day 29	–	–	–	–	–	–
Day 91	–	–	–	–	–	–
**GLUC**						
Day 8	–	–	–	–	–	–
Day 29	–	–	–	–	1.43x	–
Day 91	–	–	–	–	1.22x	–

M = Males F = Females.
A dash (—) indicates absence of change. Numerical values indicate fold changes of treated group value relative to control group mean value. Bolded values indicate mean values were statistically different from controls at P ≤ 0.05; P <=0.01

There were transient minimal and non-dose-related increases in ALT in females at ≥ 0.36 x 10^{12} vg and males at ≥ 1.1 x 10^{12} vg at the end of the main study on Day 8.

There were minimal increases in GLUC in males at 3.3 x 10^{12} vg during and at the end of the recovery period on Days 29 and 91, respectively.

Remaining differences in clinical chemistry parameters, regardless of statistical significance, were not considered AAV9/AP4M1-related based on their small magnitude, inconsistent direction, absence of a dose response, general overlap of individual values with the range of control and baseline values, and/or were of a magnitude of change commonly observed in rats under similar study conditions.

4.5. Urinalysis

(Table 4 and Appendix 4)

No AAV9/AP4M1-related urinalysis changes were noted at any dose levels.

All differences in urinalysis parameters, regardless of statistical significance, were not considered AAV9/AP4M1-related based on their small magnitude, inconsistent direction, absence of a dose response, general overlap of individual values with the range of control and

baseline values, and/or were of a magnitude of change commonly observed in rats under similar study conditions.

5. CONCLUSIONS

Administration of AAV9/AP4M1 to rats when given by single intrathecal injection at doses of 0.36×10^{12}, 1.1×10^{12} or 3.3×10^{12} vg elicited minimal to mild clinical pathology changes starting at 0.36×10^{12} vg.

At the end of the main study, there were increases in alanine aminotransferase activity in females at $\geq 0.36 \times 10^{12}$ vg and males at $\geq 1.1 \times 10^{12}$ vg, and increases in fibrinogen in 2/5 males at 3.3×10^{12} vg.

During or at the end of the recovery period, there were increases in neutrophils and total white blood cells in both sexes at 3.3×10^{12} vg and increases in glucose only in males at 3.3×10^{12} vg.

There were no changes in urinalysis parameters at any dose level.

6. REPORT APPROVAL

All electronic signatures appear at the end of the document upon finalization.

Appendix 16

Table 1

Summary of Hematology Values

5550008

Day: 8 Relative to Start Date

Sex: Male		Reporting Hematology WBC (10^3/uL) [G]	NEUT (10^3/uL) [G]	LYMPH (10^3/uL) [G]	MONO (10^3/uL) [G]	EOS (10^3/uL) [G]	BASO (10^3/uL) [G]	LUC (10^3/uL) [G]
0	Mean	6.848	1.023	5.508	0.195	0.048	0.008	0.060
vg	SD	1.760	0.430	1.708	0.081	0.028	0.005	0.026
	N	4	4	4	4	4	4	4
Group 1		-	-	-	-	-	-	-
0.36x	Mean	6.568	1.254	5.026	0.190	0.044	0.006	0.050
10E12	SD	2.370	0.573	1.748	0.084	0.024	0.005	0.020
vg	N	5	5	5	5	5	5	5
Group 2	tCtrl	0.96	1.23	0.91	0.97	0.93	0.80	0.83
1.1x	Mean	7.006	1.062	5.634	0.216	0.038	0.006	0.046
10E12	SD	2.476	0.530	1.987	0.090	0.008	0.005	0.036
vg	N	5	5	5	5	5	5	5
Group 3	tCtrl	1.02	1.04	1.02	1.11	0.80	0.80	0.77
3.3x	Mean	7.708	1.096	6.272	0.242	0.044	0.010	0.046
10E12	SD	1.627	0.354	1.407	0.116	0.017	0.007	0.021
vg	N	5	5	5	5	5	5	5
Group 4	tCtrl	1.13	1.07	1.14	1.24	0.93	1.33	0.77

[G] - Anova & Dunnett

Appendix 16

Table 1

Summary of Hematology Values

5550008

Day: 8 Relative to Start Date

Sex: Male		RBC (10^6/uL) [G]	HGB (g/dL) [G]	HCT (%) [G1]	MCV (fL) [G1]	MCH (pg) [G1]	MCHC (g/dL) [G1]	RDW (%) [G1]
0	Mean	6.130	12.28	38.23	62.28	20.05	32.15	13.60
vg	SD	0.590	1.31	4.32	1.58	0.39	0.25	0.68
	N	4	4	4	4	4	4	4
Group 1		-	-	-	-	-	-	-
0.36x	Mean	6.416	12.54	39.80	62.10	19.56	31.52	13.24
10E12	SD	0.359	0.64	2.62	3.19	0.44	1.12	0.42
vg	N	5	5	5	5	5	5	5
Group 2	tCtrl	1.05	1.02	1.04	1.00	0.98	0.98	0.97
1.1x	Mean	6.372	12.66	39.86	62.56	19.84	31.70	13.06
10E12	SD	0.286	0.47	1.67	2.24	0.89	0.41	0.65
vg	N	5	5	5	5	5	5	5
Group 3	tCtrl	1.04	1.03	1.04	1.00	0.99	0.99	0.96
3.3x	Mean	6.626	13.36	42.26	63.82	20.14	31.60	13.34
10E12	SD	0.353	0.87	2.73	2.38	0.68	0.42	0.51
vg	N	5	5	5	5	5	5	5
Group 4	tCtrl	1.08	1.09	1.11	1.02	1.00	0.98	0.98

Reporting Hematology

[G] - Kruskal-Wallis & Dunn
[G1] - Anova & Dunnett

Appendix 16

Table 1

Summary of Hematology Values

5550008

Day: 8 Relative to Start Date

Sex: Male		Reporting Hematology PLT (10^3/uL) [G]	Reporting Hematology RETIC (10^9/L) [G1]
0	Mean	1256.3	389.53
vg	SD	106.6	147.92
	N	4	4
Group 1		-	-
0.36x	Mean	1185.6	437.10
10E12	SD	251.9	39.32
vg	N	5	5
Group 2	tCtrl	0.94	1.12
1.1x	Mean	1315.8	428.40
10E12	SD	97.2	56.95
vg	N	5	5
Group 3	tCtrl	1.05	1.10
3.3x	Mean	1242.6	454.88
10E12	SD	94.1	26.25
vg	N	5	5
Group 4	tCtrl	0.99	1.17

[G] - Anova & Dunnett
[G1] - Kruskal-Wallis & Dunn

Appendix 16

Table 1

Summary of Hematology Values

5550008

Day: 8 Relative to Start Date

Sex: Female		Reporting Hematology						
		WBC (10^3/uL) [G]	NEUT (10^3/uL) [G]	LYMPH (10^3/uL) [G]	MONO (10^3/uL) [G]	EOS (10^3/uL) [G]	BASO (10^3/uL) [G]	LUC (10^3/uL) [G1]
0	Mean	5.245	1.050	3.990	0.120	0.045	0.003	0.030
vg	SD	0.776	0.388	0.770	0.026	0.017	0.005	0.012
	N	4	4	4	4	4	4	4
Group 1		-	-	-	-	-	-	-
0.36x	Mean	7.312	0.764	6.228 *	0.182	0.064	0.008	0.066 *
10E12	SD	2.017	0.387	1.672	0.064	0.026	0.004	0.023
vg	N	5	5	5	5	5	5	5
Group 2	tCtrl	1.39	0.73	1.56	1.52	1.42	3.20	2.20
1.1x	Mean	5.896	0.924	4.722	0.156	0.050	0.004	0.034
10E12	SD	1.575	0.310	1.302	0.055	0.016	0.005	0.009
vg	N	5	5	5	5	5	5	5
Group 3	tCtrl	1.12	0.88	1.18	1.30	1.11	1.60	1.13
3.3x	Mean	5.060	0.718	4.108	0.138	0.060	0.006	0.030
10E12	SD	1.001	0.243	0.805	0.044	0.027	0.005	0.014
vg	N	5	5	5	5	5	5	5
Group 4	tCtrl	0.96	0.68	1.03	1.15	1.33	2.40	1.00

[G] - Anova & Dunnett: * = p ≤ 0.05
[G1] - Kruskal-Wallis & Dunn: * = p ≤ 0.05

Appendix 16

Table 1

Summary of Hematology Values

5550008

Day: 8 Relative to Start Date

Sex: Female		RBC (10^6/uL) [G]	HGB (g/dL) [G]	HCT (%) [G1]	MCV (fL) [G]	MCH (pg) [G]	MCHC (g/dL) [G]	RDW (%) [G]
0	Mean	6.463	12.85	38.70	59.95	19.90	33.18	11.48
vg	SD	0.139	0.25	0.66	2.23	0.73	0.62	0.50
	N	4	4	4	4	4	4	4
Group 1		-	-	-	-	-	-	-
0.36x	Mean	6.548	13.16	40.20	61.40	20.14	32.80	11.04
10E12	SD	0.106	0.50	2.09	3.32	0.80	0.50	0.62
vg	N	5	5	5	5	5	5	5
Group 2	tCtrl	1.01	1.02	1.04	1.02	1.01	0.99	0.96
1.1x	Mean	6.806	13.36	40.24	59.14	19.62	33.16	11.32
10E12	SD	0.304	0.45	1.09	2.29	0.58	0.44	0.72
vg	N	5	5	5	5	5	5	5
Group 3	tCtrl	1.05	1.04	1.04	0.99	0.99	1.00	0.99
3.3x	Mean	6.764	13.10	40.60	60.06	19.42	32.28 *	11.12
10E12	SD	0.341	0.46	1.40	1.49	0.59	0.24	0.58
vg	N	5	5	5	5	5	5	5
Group 4	tCtrl	1.05	1.02	1.05	1.00	0.98	0.97	0.97

Reporting Hematology

[G] - Anova & Dunnett: * = p ≤ 0.05
[G1] - Kruskal-Wallis & Dunn

Appendix 16

Table 1

Summary of Hematology Values

5550008

Day: 8 Relative to Start Date

Sex: Female		Reporting Hematology PLT (10^3/uL) [G]	Reporting Hematology RETIC (10^9/L) [G1]
0	Mean	1252.3	315.68
vg	SD	246.2	54.21
	N	4	4
Group 1		-	-
0.36x	Mean	1266.2	299.60
10E12	SD	87.2	66.70
vg	N	5	5
Group 2	tCtrl	1.01	0.95
1.1x	Mean	1104.4	249.10
10E12	SD	60.7	45.46
vg	N	5	5
Group 3	tCtrl	0.88	0.79
3.3x	Mean	1264.6	295.42
10E12	SD	200.3	37.28
vg	N	5	5
Group 4	tCtrl	1.01	0.94

[G] - Kruskal-Wallis & Dunn
[G1] - Anova & Dunnett

Appendix 16

Table 1

Summary of Hematology Values

5550008

Day: 29 Relative to Start Date

Sex: Male		WBC (10^3/uL) [G]	NEUT (10^3/uL) [G]	LYMPH (10^3/uL) [G]	MONO (10^3/uL) [G1]	EOS (10^3/uL) [G]	BASO (10^3/uL) [G]	LUC (10^3/uL) [G1]
0	Mean	9.624	1.748	7.520	0.216	0.080	0.012	0.046
vg	SD	3.108	0.297	2.731	0.100	0.010	0.008	0.015
	N	5	5	5	5	5	5	5
Group 1		-	-	-	-	-	-	-
0.36x	Mean	6.858	0.956 *	5.658	0.134	0.054	0.008	0.048
10E12	SD	1.974	0.298	1.824	0.048	0.024	0.008	0.026
vg	N	5	5	5	5	5	5	5
Group 2	tCtrl	0.71	0.55	0.75	0.62	0.68	0.67	1.04
1.1x	Mean	7.748	1.340	6.136	0.170	0.056	0.006	0.040
10E12	SD	2.153	0.335	2.052	0.086	0.030	0.005	0.019
vg	N	5	5	5	5	5	5	5
Group 3	tCtrl	0.81	0.77	0.82	0.79	0.70	0.50	0.87
3.3x	Mean	9.065	1.663	7.013	0.253	0.063	0.020	0.058
10E12	SD	4.703	0.742	3.832	0.142	0.036	0.020	0.038
vg	N	4	4	4	4	4	4	4
Group 4	tCtrl	0.94	0.95	0.93	1.17	0.78	1.67	1.25

Reporting Hematology

[G] - Anova & Dunnett: * = $p \leq 0.05$
[G1] - Kruskal-Wallis & Dunn

Appendix 16

Table 1

Summary of Hematology Values

5550008

Day: 29 Relative to Start Date

Sex: Male		Reporting Hematology RBC (10^6/uL) [G]	HGB (g/dL) [G]	HCT (%) [G]	MCV (fL) [G1]	MCH (pg) [G1]	MCHC (g/dL) [G]	RDW (%) [G]
0	Mean	6.972	13.34	40.38	57.94	19.14	33.06	11.98
vg	SD	0.262	0.54	1.32	1.23	0.24	0.75	1.06
	N	5	5	5	5	5	5	5
Group 1		-	-	-	-	-	-	-
0.36x	Mean	6.894	12.84	39.66	57.56	18.62	32.36	12.10
10E12	SD	0.427	0.69	1.85	1.57	0.50	0.30	0.51
vg	N	5	5	5	5	5	5	5
Group 2	tCtrl	0.99	0.96	0.98	0.99	0.97	0.98	1.01
1.1x	Mean	7.130	13.32	40.44	56.76	18.70	32.98	11.60
10E12	SD	0.319	0.54	1.72	0.80	0.19	0.22	0.35
vg	N	5	5	5	5	5	5	5
Group 3	tCtrl	1.02	1.00	1.00	0.98	0.98	1.00	0.97
3.3x	Mean	6.898	13.38	40.93	59.50	19.48	32.75	12.28
10E12	SD	0.441	0.36	1.02	4.14	1.26	0.24	0.50
vg	N	4	4	4	4	4	4	4
Group 4	tCtrl	0.99	1.00	1.01	1.03	1.02	0.99	1.02

[G] - Anova & Dunnett
[G1] - Kruskal-Wallis & Dunn

Appendix 16

Table 1

Summary of Hematology Values

5550008

Day: 29 Relative to Start Date

Sex: Male		Reporting Hematology PLT (10^3/uL) [G]	Reporting Hematology RETIC (10^9/L) [G1]
0 vg Group 1	Mean	1094.2	242.26
	SD	152.6	15.54
	N	5	5
		-	-
0.36x 10E12 vg Group 2	Mean	1110.2	239.68
	SD	78.5	28.77
	N	5	5
	tCtrl	1.01	0.99
1.1x 10E12 vg Group 3	Mean	1017.8	242.82
	SD	81.2	21.94
	N	5	5
	tCtrl	0.93	1.00
3.3x 10E12 vg Group 4	Mean	1119.8	246.38
	SD	170.9	11.29
	N	4	4
	tCtrl	1.02	1.02

[G] - Kruskal-Wallis & Dunn
[G1] - Anova & Dunnett

Appendix 16

Table 1

Summary of Hematology Values

5550008

Day: 29 Relative to Start Date

Sex: Female		Reporting Hematology						
		WBC (10^3/uL) [G]	NEUT (10^3/uL) [G]	LYMPH (10^3/uL) [G]	MONO (10^3/uL) [G]	EOS (10^3/uL) [G1]	BASO (10^3/uL) [G1]	LUC (10^3/uL) [G]
0	Mean	4.825	0.638	3.968	0.123	0.063	0.000	0.028
vg	SD	2.220	0.326	1.814	0.060	0.033	0.000	0.015
	N	4	4	4	4	4	4	4
Group 1		-	-	-	-	-	-	-
0.36x	Mean	5.908	1.176	4.488	0.136	0.074	0.004	0.032
10E12	SD	1.554	1.008	0.622	0.046	0.023	0.005	0.008
vg	N	5	5	5	5	5	5	5
Group 2	tCtrl	1.22	1.84	1.13	1.11	1.18	-	1.16
1.1x	Mean	6.792	1.068	5.470	0.162	0.048	0.006	0.042
10E12	SD	2.024	0.475	1.619	0.035	0.024	0.005	0.013
vg	N	5	5	5	5	5	5	5
Group 3	tCtrl	1.41	1.68	1.38	1.32	0.77	-	1.53
3.3x	Mean	6.724	0.572	5.894	0.148	0.056	0.004	0.052 *
10E12	SD	0.557	0.075	0.488	0.122	0.005	0.005	0.011
vg	N	5	5	5	5	5	5	5
Group 4	tCtrl	1.39	0.90	1.49	1.21	0.90	-	1.89

[G] - Anova & Dunnett: * = p ≤ 0.05
[G1] - Kruskal-Wallis & Dunn

Appendix 16

Table 1

Summary of Hematology Values

5550008

Day: 29 Relative to Start Date

Sex: Female		Reporting Hematology RBC (10^6/uL) [G]	HGB (g/dL) [G]	HCT (%) [G]	MCV (fL) [G]	MCH (pg) [G]	MCHC (g/dL) [G]	RDW (%) [G]
0	Mean	7.450	14.05	40.90	54.93	18.85	34.33	10.30
vg	SD	0.279	0.69	1.30	0.56	0.26	0.66	0.29
	N	4	4	4	4	4	4	4
Group 1		-	-	-	-	-	-	-
0.36x	Mean	7.198	13.62	40.12	55.74	18.90	33.94	10.68
10E12	SD	0.161	0.65	1.76	1.36	0.46	0.35	0.52
vg	N	5	5	5	5	5	5	5
Group 2	tCtrl	0.97	0.97	0.98	1.01	1.00	0.99	1.04
1.1x	Mean	7.532	14.02	41.60	55.30	18.66	33.70	10.60
10E12	SD	0.422	0.76	1.80	1.60	0.72	0.60	0.60
vg	N	5	5	5	5	5	5	5
Group 3	tCtrl	1.01	1.00	1.02	1.01	0.99	0.98	1.03
3.3x	Mean	7.130	13.60	39.98	56.08	19.10	34.04	10.58
10E12	SD	0.368	0.80	2.13	1.03	0.49	0.66	0.52
vg	N	5	5	5	5	5	5	5
Group 4	tCtrl	0.96	0.97	0.98	1.02	1.01	0.99	1.03

[G] - Anova & Dunnett

Appendix 16

Table 1

Summary of Hematology Values

5550008

Day: 29 Relative to Start Date

Sex: Female		Reporting Hematology PLT (10^3/uL) [G]	Reporting Hematology RETIC (10^9/L) [G1]
0 vg Group 1	Mean	891.8	169.70
	SD	109.2	25.94
	N	4	4
		-	-
0.36x 10E12 vg Group 2	Mean	1075.2	172.04
	SD	141.9	11.04
	N	5	5
	tCtrl	1.21	1.01
1.1x 10E12 vg Group 3	Mean	1011.2	196.96
	SD	100.4	16.92
	N	5	5
	tCtrl	1.13	1.16
3.3x 10E12 vg Group 4	Mean	1066.6	186.82
	SD	61.6	36.77
	N	5	5
	tCtrl	1.20	1.10

[G] - Anova & Dunnett
[G1] - Kruskal-Wallis & Dunn

Appendix 16

Table 1

Summary of Hematology Values

5550008

Day: 91 Relative to Start Date

Sex: Male		Reporting Hematology						
		WBC (10^3/uL) [G]	NEUT (10^3/uL) [G1]	LYMPH (10^3/uL) [G]	MONO (10^3/uL) [G]	EOS (10^3/uL) [G]	BASO (10^3/uL) [G]	LUC (10^3/uL) [G]
0	Mean	7.264	1.004	5.936	0.154	0.102	0.014	0.050
vg	SD	2.675	0.197	2.417	0.059	0.033	0.009	0.024
	N	5	5	5	5	5	5	5
Group 1		-	-	-	-	-	-	-
0.36x	Mean	7.864	1.436	6.056	0.204	0.108	0.018	0.048
10E12	SD	2.385	0.317	2.243	0.090	0.041	0.008	0.020
vg	N	5	5	5	5	5	5	5
Group 2	tCtrl	1.08	1.43	1.02	1.32	1.06	1.29	0.96
1.1x	Mean	6.566	1.048	5.180	0.182	0.094	0.012	0.050
10E12	SD	1.947	0.279	1.829	0.096	0.031	0.004	0.024
vg	N	5	5	5	5	5	5	5
Group 3	tCtrl	0.90	1.04	0.87	1.18	0.92	0.86	1.00
3.3x	Mean	9.536	1.874 *	7.260	0.244	0.086	0.020	0.050
10E12	SD	2.646	0.656	2.222	0.067	0.030	0.007	0.017
vg	N	5	5	5	5	5	5	5
Group 4	tCtrl	1.31	1.87	1.22	1.58	0.84	1.43	1.00

[G] - Anova & Dunnett

[G1] - Kruskal-Wallis & Dunn: * = p ≤ 0.05

Appendix 16

Table 1

Summary of Hematology Values

5550008

Day: 91 Relative to Start Date

Sex: Male		RBC (10^6/uL) [G]	HGB (g/dL) [G]	HCT (%) [G1]	MCV (fL) [G]	MCH (pg) [G]	MCHC (g/dL) [G]	RDW (%) [G]
0 vg Group 1	Mean	7.894	14.10	41.86	53.08	17.86	33.64	13.46
	SD	0.274	0.52	1.40	1.46	0.73	0.74	1.10
	N	5	5	5	5	5	5	5
		-	-	-	-	-	-	-
0.36x 10E12 vg Group 2	Mean	7.730	14.38	41.74	54.06	18.60	34.42	13.22
	SD	0.233	0.39	0.54	1.09	0.58	0.81	0.59
	N	5	5	5	5	5	5	5
	tCtrl	0.98	1.02	1.00	1.02	1.04	1.02	0.98
1.1x 10E12 vg Group 3	Mean	8.128	14.44	42.80	52.66	17.78	33.80	13.18
	SD	0.472	0.80	2.47	0.57	0.69	1.09	1.08
	N	5	5	5	5	5	5	5
	tCtrl	1.03	1.02	1.02	0.99	1.00	1.00	0.98
3.3x 10E12 vg Group 4	Mean	8.114	14.24	43.02	53.02	17.56	33.12	13.12
	SD	0.318	0.70	2.22	1.12	0.40	0.33	1.29
	N	5	5	5	5	5	5	5
	tCtrl	1.03	1.01	1.03	1.00	0.98	0.98	0.97

Reporting Hematology

[G] - Anova & Dunnett
[G1] - Kruskal-Wallis & Dunn

Appendix 16

Table 1

Summary of Hematology Values

5550008

Day: 91 Relative to Start Date

Sex: Male		Reporting Hematology PLT (10^3/uL) [G]	Reporting Hematology RETIC (10^9/L) [G]
0	Mean	978.8	226.94
vg	SD	97.2	28.52
	N	5	5
Group 1		-	-
0.36x	Mean	927.2	189.98
10E12	SD	102.7	29.61
vg	N	5	5
Group 2	tCtrl	0.95	0.84
1.1x	Mean	1076.6	209.84
10E12	SD	79.9	46.96
vg	N	5	5
Group 3	tCtrl	1.10	0.92
3.3x	Mean	1014.4	229.22
10E12	SD	129.3	25.36
vg	N	5	5
Group 4	tCtrl	1.04	1.01

[G] - Anova & Dunnett

Appendix 16

Table 1

Summary of Hematology Values

5550008

Day: 91 Relative to Start Date

Sex: Female		Reporting Hematology						
		WBC (10^3/uL) [G]	NEUT (10^3/uL) [G]	LYMPH (10^3/uL) [G]	MONO (10^3/uL) [G]	EOS (10^3/uL) [G]	BASO (10^3/uL) [G]	LUC (10^3/uL) [G1]
0	Mean	4.625	0.675	3.723	0.138	0.063	0.008	0.020
vg	SD	1.179	0.168	1.227	0.029	0.013	0.005	0.008
	N	4	4	4	4	4	4	4
Group 1		-	-	-	-	-	-	-
0.36x	Mean	3.234	0.432	2.658	0.082	0.046	0.006	0.016
10E12	SD	1.346	0.166	1.163	0.040	0.015	0.005	0.017
vg	N	5	5	5	5	5	5	5
Group 2	tCtrl	0.70	0.64	0.71	0.60	0.74	0.80	0.80
1.1x	Mean	4.472	0.772	3.432	0.172	0.062	0.004	0.034
10E12	SD	1.350	0.422	1.330	0.072	0.024	0.005	0.017
vg	N	5	5	5	5	5	5	5
Group 3	tCtrl	0.97	1.14	0.92	1.25	0.99	0.53	1.70
3.3x	Mean	5.974	0.978	4.726	0.148	0.070	0.008	0.040
10E12	SD	1.880	0.382	1.508	0.048	0.022	0.008	0.034
vg	N	5	5	5	5	5	5	5
Group 4	tCtrl	1.29	1.45	1.27	1.08	1.12	1.07	2.00

[G] - Anova & Dunnett
[G1] - Kruskal-Wallis & Dunn

Appendix 16

Table 1

Summary of Hematology Values

5550008

Day: 91 Relative to Start Date

Sex: Female		RBC (10^6/uL) [G]	HGB (g/dL) [G]	HCT (%) [G]	MCV (fL) [G]	MCH (pg) [G1]	MCHC (g/dL) [G]	RDW (%) [G]
0	Mean	7.513	14.08	40.43	53.90	18.78	34.78	11.28
vg	SD	0.255	0.76	1.73	3.70	1.32	0.76	0.24
	N	4	4	4	4	4	4	4
Group 1		-	-	-	-	-	-	-
0.36x	Mean	7.434	13.94	41.52	55.86	18.76	33.60	11.72
10E12	SD	0.182	0.59	1.74	1.96	0.70	0.50	0.48
vg	N	5	5	5	5	5	5	5
Group 2	tCtrl	0.99	0.99	1.03	1.04	1.00	0.97	1.04
1.1x	Mean	7.672	13.82	40.56	52.92	18.02	34.08	11.62
10E12	SD	0.201	0.24	1.61	2.75	0.47	0.93	0.49
vg	N	5	5	5	5	5	5	5
Group 3	tCtrl	1.02	0.98	1.00	0.98	0.96	0.98	1.03
3.3x	Mean	7.622	13.78	40.82	53.58	18.12	33.78	12.16 *
10E12	SD	0.422	0.72	2.04	1.40	0.54	0.40	0.36
vg	N	5	5	5	5	5	5	5
Group 4	tCtrl	1.01	0.98	1.01	0.99	0.97	0.97	1.08

Reporting Hematology

[G] - Anova & Dunnett: * = p ≤ 0.05
[G1] - Kruskal-Wallis & Dunn

Appendix 16

Table 1

Summary of Hematology Values

5550008

Day: 91 Relative to Start Date

Sex: Female		Reporting Hematology PLT (10^3/uL) [G]	Reporting Hematology RETIC (10^9/L) [G]
0	Mean	960.3	158.45
vg	SD	63.9	38.28
	N	4	4
Group 1		-	-
0.36x	Mean	946.4	175.92
10E12	SD	95.4	23.25
vg	N	5	5
Group 2	tCtrl	0.99	1.11
1.1x	Mean	968.6	155.84
10E12	SD	49.0	39.72
vg	N	5	5
Group 3	tCtrl	1.01	0.98
3.3x	Mean	1023.0	176.90
10E12	SD	37.8	59.39
vg	N	5	5
Group 4	tCtrl	1.07	1.12

[G] - Anova & Dunnett

Appendix 16

Table 2

Summary of Coagulation Values

5550008

Day: 8 Relative to Start Date

Sex: Male		Reporting Coagulation PT (sec) [G]	APTT (sec) [G]	FIB (mg/dL) [G1]
0	Mean	15.40	17.88	269.8
vg	SD	0.59	0.92	20.1
	N	4	4	4
Group 1		-	-	-
0.36x	Mean	14.68	19.14	279.2
10E12	SD	0.76	2.01	11.1
vg	N	5	5	5
Group 2	tCtrl	0.95	1.07	1.04
1.1x	Mean	15.26	17.52	269.0
10E12	SD	0.34	1.47	16.2
vg	N	5	5	5
Group 3	tCtrl	0.99	0.98	1.00
3.3x	Mean	15.32	17.08	319.2
10E12	SD	0.33	3.00	47.6
vg	N	5	5	5
Group 4	tCtrl	0.99	0.96	1.18

[G] - Anova & Dunnett
[G1] - Kruskal-Wallis & Dunn

Appendix 16

Table 2

Summary of Coagulation Values

5550008

Day: 8 Relative to Start Date

Sex: Female		PT (sec) [G]	APTT (sec) [G]	FIB (mg/dL) [G1]
0	Mean	15.03	16.97	236.3
vg	SD	0.74	2.14	8.4
	N	3	3	3
Group 1		-	-	-
0.36x	Mean	14.66	18.82	215.8
10E12	SD	0.74	0.98	19.0
vg	N	5	5	5
Group 2	tCtrl	0.98	1.11	0.91
1.1x	Mean	14.48	16.82	235.0
10E12	SD	0.28	1.36	8.4
vg	N	5	5	5
Group 3	tCtrl	0.96	0.99	0.99
3.3x	Mean	14.42	17.82	225.8
10E12	SD	0.48	1.59	20.6
vg	N	5	5	5
Group 4	tCtrl	0.96	1.05	0.96

Reporting Coagulation

[G] - Anova & Dunnett
[G1] - Kruskal-Wallis & Dunn

Appendix 16

Table 2

Summary of Coagulation Values

5550008

Day: 29 Relative to Start Date

Sex: Male		Reporting Coagulation PT (sec) [G]	Reporting Coagulation APTT (sec) [G]	Reporting Coagulation FIB (mg/dL) [G]
0	Mean	15.18	18.64	285.2
vg	SD	0.61	1.06	11.1
	N	5	5	5
Group 1		-	-	-
0.36x	Mean	15.00	19.34	289.6
10E12	SD	0.68	1.40	16.9
vg	N	5	5	5
Group 2	tCtrl	0.99	1.04	1.02
1.1x	Mean	15.12	19.02	290.4
10E12	SD	0.36	1.99	48.6
vg	N	5	5	5
Group 3	tCtrl	1.00	1.02	1.02
3.3x	Mean	15.00	20.25	272.8
10E12	SD	0.23	0.58	12.2
vg	N	4	4	4
Group 4	tCtrl	0.99	1.09	0.96

[G] - Anova & Dunnett

Appendix 16

Table 2

Summary of Coagulation Values

5550008

Day: 29 Relative to Start Date

Sex: Female		Reporting Coagulation PT (sec) [G]	APTT (sec) [G]	FIB (mg/dL) [G]
0 vg Group 1	Mean	14.58	17.60	215.8
	SD	0.56	1.32	20.0
	N	4	4	4
		-	-	-
0.36x 10E12 vg Group 2	Mean	15.00	19.92	216.4
	SD	0.44	1.37	13.3
	N	5	5	5
	tCtrl	1.03	1.13	1.00
1.1x 10E12 vg Group 3	Mean	14.54	19.28	214.2
	SD	0.52	1.13	10.7
	N	5	5	5
	tCtrl	1.00	1.10	0.99
3.3x 10E12 vg Group 4	Mean	14.68	18.32	226.8
	SD	0.70	1.40	8.2
	N	5	5	5
	tCtrl	1.01	1.04	1.05

[G] - Anova & Dunnett

Appendix 16

Table 2

Summary of Coagulation Values

5550008

Day: 91 Relative to Start Date

Sex: Male		Reporting Coagulation PT (sec) [G]	APTT (sec) [G]	FIB (mg/dL) [G]
0	Mean	16.18	20.44	256.4
vg	SD	0.42	2.44	84.7
	N	5	5	5
Group 1		-	-	-
0.36x	Mean	15.68	20.20	303.8
10E12	SD	0.71	2.68	18.8
vg	N	4	4	4
Group 2	tCtrl	0.97	0.99	1.18
1.1x	Mean	15.50	20.84	317.6
10E12	SD	0.52	1.79	13.4
vg	N	5	5	5
Group 3	tCtrl	0.96	1.02	1.24
3.3x	Mean	15.24	17.96	309.0
10E12	SD	0.56	1.21	29.4
vg	N	5	5	5
Group 4	tCtrl	0.94	0.88	1.21

[G] - Anova & Dunnett

Appendix 16

Table 2

Summary of Coagulation Values

5550008

Day: 91 Relative to Start Date

Sex: Female		Reporting Coagulation		
		PT (sec) [G]	APTT (sec) [G]	FIB (mg/dL) [G]
0 vg Group 1	Mean	15.03	19.53	203.0
	SD	0.24	0.79	15.5
	N	4	4	4
		-	-	-
0.36x 10E12 vg Group 2	Mean	14.80	19.34	209.4
	SD	0.39	1.16	13.9
	N	5	5	5
	tCtrl	0.99	0.99	1.03
1.1x 10E12 vg Group 3	Mean	14.62	19.72	195.2
	SD	0.31	0.55	13.6
	N	5	5	5
	tCtrl	0.97	1.01	0.96
3.3x 10E12 vg Group 4	Mean	14.56	16.98	192.4
	SD	0.50	4.23	29.8
	N	5	5	5
	tCtrl	0.97	0.87	0.95

[G] - Anova & Dunnett

Appendix 16

Table 3

Summary of Clinical Chemistry Values

5550008

Day: 8 Relative to Start Date

Sex: Male		AST (U/L) [G]	ALT (U/L) [G1]	ALP (U/L) [G]	GGT (U/L) [G]	CK (U/L) [G]	TBIL (mg/dL) [G]	UREAN (mg/dL) [G1]
0	Mean	91.5	29.3	269.5	1.5	454.0	0.033	11.5
vg	SD	8.9	3.0	19.6	0.0	175.6	0.039	2.1
	N	4	4	4	4	4	4	4
Group 1		-	-	-	-	-	-	-
0.36x	Mean	94.0	29.8	240.4	1.5	452.2	0.046	14.0
10E12	SD	13.5	1.6	46.1	0.0	203.0	0.034	6.2
vg	N	5	5	5	5	5	5	5
Group 2	tCtrl	1.03	1.02	0.89	1.00	1.00	1.42	1.22
1.1x	Mean	94.4	36.0 *	282.8	1.5	390.4	0.036	15.8
10E12	SD	5.1	3.4	50.8	0.0	70.0	0.021	4.0
vg	N	5	5	5	5	5	5	5
Group 3	tCtrl	1.03	1.23	1.05	1.00	0.86	1.11	1.37
3.3x	Mean	102.4	36.6 *	323.6	1.5	483.0	0.046	12.0
10E12	SD	2.9	5.9	85.2	0.0	53.2	0.005	3.3
vg	N	5	5	5	5	5	5	5
Group 4	tCtrl	1.12	1.25	1.20	1.00	1.06	1.42	1.04

Reporting Biochemistry

[G] - Kruskal-Wallis & Dunn

[G1] - Anova & Dunnett: * = $p \leq 0.05$

Appendix 16

Table 3

Summary of Clinical Chemistry Values

5550008

Day: 8 Relative to Start Date

Sex: Male		Reporting Biochemistry CREAT (mg/dL) [G]	GLUC (mg/dL) [G]	CHOL (mg/dL) [G1]	TRIG (mg/dL) [G]	TPROT (g/dL) [G]	ALB (g/dL) [G1]	GLOB (g/dL) [G]
0	Mean	0.28	198.8	72.3	40.5	4.85	3.65	1.20
vg	SD	0.05	19.3	8.6	12.4	0.13	0.06	0.14
	N	4	4	4	4	4	4	4
Group 1		-	-	-	-	-	-	-
0.36x	Mean	0.30	225.8	75.8	24.0	5.14	3.76	1.38
10E12	SD	0.07	18.0	4.5	12.2	0.21	0.11	0.11
vg	N	5	5	5	5	5	5	5
Group 2	tCtrl	1.09	1.14	1.05	0.59	1.06	1.03	1.15
1.1x	Mean	0.26	239.6	71.6	34.2	4.88	3.66	1.22
10E12	SD	0.05	31.1	15.4	9.1	0.38	0.29	0.11
vg	N	5	5	5	5	5	5	5
Group 3	tCtrl	0.95	1.21	0.99	0.84	1.01	1.00	1.02
3.3x	Mean	0.26	186.0	77.0	27.4	4.98	3.74	1.24
10E12	SD	0.05	45.8	17.5	4.9	0.15	0.17	0.17
vg	N	5	5	5	5	5	5	5
Group 4	tCtrl	0.95	0.94	1.07	0.68	1.03	1.02	1.03

[G] - Anova & Dunnett
[G1] - Kruskal-Wallis & Dunn

Appendix 16

Table 3

Summary of Clinical Chemistry Values

5550008

Day: 8 Relative to Start Date

Sex: Male		A/G (ratio) [G]	CA (mg/dL) [G]	PHOS (mg/dL) [G1]	NA (mmol/L) [G1]	K (mmol/L) [G]	CL (mmol/L) [G]
0	Mean	3.10	9.75	11.08	142.0	4.35	100.8
vg	SD	0.36	0.44	0.50	3.2	0.44	2.5
	N	4	4	4	4	4	4
Group 1		-	-	-	-	-	-
0.36x	Mean	2.72	9.56	10.56	141.8	4.70	100.0
10E12	SD	0.16	0.50	1.99	2.2	0.66	2.2
vg	N	5	5	5	5	5	5
Group 2	tCtrl	0.88	0.98	0.95	1.00	1.08	0.99
1.1x	Mean	3.00	9.82	9.86	139.6	5.12	99.6
10E12	SD	0.19	0.54	1.28	3.2	0.50	2.3
vg	N	5	5	5	5	5	5
Group 3	tCtrl	0.97	1.01	0.89	0.98	1.18	0.99
3.3x	Mean	3.06	10.00	11.18	139.4	4.76	100.0
10E12	SD	0.47	0.29	1.44	1.1	0.56	1.2
vg	N	5	5	5	5	5	5
Group 4	tCtrl	0.99	1.03	1.01	0.98	1.09	0.99

Reporting Biochemistry

[G] - Anova & Dunnett
[G1] - Kruskal-Wallis & Dunn

Appendix 16

Table 3

Summary of Clinical Chemistry Values

5550008

Day: 8 Relative to Start Date

Sex: Female		AST (U/L) [G]	ALT (U/L) [G]	ALP (U/L) [G]	GGT (U/L) [G1]	CK (U/L) [G]	TBIL (mg/dL) [G]	UREAN (mg/dL) [G]
0	Mean	89.6	23.6	154.2	1.5	408.2	0.028	17.0
vg	SD	23.0	3.9	24.2	0.0	305.3	0.016	4.6
	N	5	5	5	5	5	5	5
Group 1		-	-	-	-	-	-	-
0.36x	Mean	92.6	33.2 *	142.8	1.5	448.2	0.022	19.2
10E12	SD	27.2	4.8	20.9	0.0	238.7	0.020	4.0
vg	N	5	5	5	5	5	5	5
Group 2	tCtrl	1.03	1.41	0.93	1.00	1.10	0.79	1.13
1.1x	Mean	86.6	32.6 *	118.4	1.5	265.4	0.042	21.4
10E12	SD	18.8	6.4	19.7	0.0	93.0	0.027	1.5
vg	N	5	5	5	5	5	5	5
Group 3	tCtrl	0.97	1.38	0.77	1.00	0.65	1.50	1.26
3.3x	Mean	87.6	36.8 **	144.0	1.5	293.0	0.024	21.0
10E12	SD	11.3	6.0	32.7	0.0	90.1	0.023	2.8
vg	N	5	5	5	5	5	5	5
Group 4	tCtrl	0.98	1.56	0.93	1.00	0.72	0.86	1.24

Reporting Biochemistry

[G] - Anova & Dunnett: * = p ≤ 0.05; ** = p ≤ 0.01
[G1] - Kruskal-Wallis & Dunn

Appendix 16

Table 3

Summary of Clinical Chemistry Values

5550008

Day: 8 Relative to Start Date

Sex: Female		CREAT (mg/dL) [G]	GLUC (mg/dL) [G1]	CHOL (mg/dL) [G1]	TRIG (mg/dL) [G1]	TPROT (g/dL) [G]	ALB (g/dL) [G]	GLOB (g/dL) [G1]
0	Mean	0.28	160.8	56.0	19.6	5.46	4.06	1.40
vg	SD	0.04	27.1	17.6	3.9	0.17	0.15	0.22
	N	5	5	5	5	5	5	5
Group 1		-	-	-	-	-	-	-
0.36x	Mean	0.34	200.6	56.6	17.4	5.38	4.00	1.38
10E12	SD	0.05	48.8	6.0	5.2	0.08	0.14	0.08
vg	N	5	5	5	5	5	5	5
Group 2	tCtrl	1.21	1.25	1.01	0.89	0.99	0.99	0.99
1.1x	Mean	0.26	169.8	52.0	18.8	5.64	4.24	1.40
10E12	SD	0.05	9.0	14.1	3.9	0.40	0.29	0.21
vg	N	5	5	5	5	5	5	5
Group 3	tCtrl	0.93	1.06	0.93	0.96	1.03	1.04	1.00
3.3x	Mean	0.30	200.6	50.2	16.6	5.36	4.14	1.22
10E12	SD	0.00	13.3	8.2	6.2	0.32	0.30	0.11
vg	N	5	5	5	5	5	5	5
Group 4	tCtrl	1.07	1.25	0.90	0.85	0.98	1.02	0.87

Reporting Biochemistry

[G] - Kruskal-Wallis & Dunn
[G1] - Anova & Dunnett

Appendix 16

Table 3

Summary of Clinical Chemistry Values

5550008

Day: 8 Relative to Start Date

Sex: Female		Reporting Biochemistry					
		A/G (ratio) [G]	CA (mg/dL) [G]	PHOS (mg/dL) [G]	NA (mmol/L) [G]	K (mmol/L) [G]	CL (mmol/L) [G]
0	Mean	2.98	9.78	7.54	138.8	4.08	100.8
vg	SD	0.54	0.49	1.03	0.8	0.40	2.6
	N	5	5	5	5	5	5
Group 1		-	-	-	-	-	-
0.36x	Mean	2.92	9.86	8.34	139.4	4.46	99.8
10E12	SD	0.27	0.44	1.15	2.6	0.25	1.6
vg	N	5	5	5	5	5	5
Group 2	tCtrl	0.98	1.01	1.11	1.00	1.09	0.99
1.1x	Mean	3.08	9.90	8.14	142.4 *	4.02	103.8
10E12	SD	0.46	0.31	1.02	2.5	0.25	1.6
vg	N	5	5	5	5	5	5
Group 3	tCtrl	1.03	1.01	1.08	1.03	0.99	1.03
3.3x	Mean	3.44	10.06	8.60	141.2	4.18	101.4
10E12	SD	0.42	0.42	1.56	1.1	0.40	1.5
vg	N	5	5	5	5	5	5
Group 4	tCtrl	1.15	1.03	1.14	1.02	1.02	1.01

[G] - Anova & Dunnett: * = p ≤ 0.05

Appendix 16

Table 3

Summary of Clinical Chemistry Values

5550008

Day: 29 Relative to Start Date

Sex: Male		AST (U/L) [G]	ALT (U/L) [G]	ALP (U/L) [G]	GGT (U/L) [G1]	CK (U/L) [G]	TBIL (mg/dL) [G]	UREAN (mg/dL) [G1]
0	Mean	96.8	32.8	160.2	1.5	453.2	0.052	14.8
vg	SD	30.3	3.8	39.4	0.0	331.4	0.034	1.9
	N	5	5	5	5	5	5	5
Group 1		-	-	-	-	-	-	-
0.36x	Mean	89.3	28.3	210.0	1.5	484.3	0.023	14.8
10E12	SD	16.6	2.5	49.7	0.0	284.1	0.026	5.6
vg	N	4	4	4	4	4	4	4
Group 2	tCtrl	0.92	0.86	1.31	1.00	1.07	0.43	1.00
1.1x	Mean	99.2	35.4	161.6	1.5	523.6	0.054	14.6
10E12	SD	32.3	5.2	37.3	0.0	449.6	0.018	0.9
vg	N	5	5	5	5	5	5	5
Group 3	tCtrl	1.02	1.08	1.01	1.00	1.16	1.04	0.99
3.3x	Mean	71.6	30.0	155.0	1.5	277.8	0.042	14.4
10E12	SD	5.5	6.9	20.9	0.0	111.6	0.008	1.7
vg	N	5	5	5	5	5	5	5
Group 4	tCtrl	0.74	0.91	0.97	1.00	0.61	0.81	0.97

Reporting Biochemistry

[G] - Anova & Dunnett
[G1] - Kruskal-Wallis & Dunn

Appendix 16

Table 3

Summary of Clinical Chemistry Values

5550008

Day: 29 Relative to Start Date

Sex: Male		CREAT (mg/dL) [G]	GLUC (mg/dL) [G]	CHOL (mg/dL) [G1]	TRIG (mg/dL) [G1]	TPROT (g/dL) [G]	ALB (g/dL) [G]	GLOB (g/dL) [G]
		Reporting Biochemistry						
0	Mean	0.34	180.8	59.8	29.6	5.72	4.02	1.70
vg	SD	0.05	54.2	3.5	2.3	0.11	0.11	0.16
	N	5	5	5	5	5	5	5
Group 1		-	-	-	-	-	-	-
0.36x	Mean	0.33	232.8	63.8	32.8	5.60	3.88	1.73
10E12	SD	0.05	23.0	11.4	6.2	0.34	0.17	0.22
vg	N	4	4	4	4	4	4	4
Group 2	tCtrl	0.96	1.29	1.07	1.11	0.98	0.96	1.01
1.1x	Mean	0.30	200.2	57.4	36.2	5.56	3.88	1.68
10E12	SD	0.07	52.9	3.4	16.4	0.32	0.29	0.11
vg	N	5	5	5	5	5	5	5
Group 3	tCtrl	0.88	1.11	0.96	1.22	0.97	0.97	0.99
3.3x	Mean	0.32	259.0	70.6	47.4	5.78	3.96	1.82
10E12	SD	0.04	44.1	12.2	12.5	0.29	0.11	0.23
vg	N	5	5	5	5	5	5	5
Group 4	tCtrl	0.94	1.43	1.18	1.60	1.01	0.99	1.07

[G] - Anova & Dunnett
[G1] - Kruskal-Wallis & Dunn

Appendix 16

Table 3

Summary of Clinical Chemistry Values

5550008

Day: 29 Relative to Start Date

Sex: Male		Reporting Biochemistry A/G (ratio) [G]	CA (mg/dL) [G]	PHOS (mg/dL) [G]	NA (mmol/L) [G]	K (mmol/L) [G]	CL (mmol/L) [G]
0	Mean	2.40	9.84	8.74	142.0	4.88	101.6
vg	SD	0.27	0.19	0.79	2.1	0.37	2.9
	N	5	5	5	5	5	5
Group 1		-	-	-	-	-	-
0.36x	Mean	2.28	9.73	9.05	141.8	5.23	101.8
10E12	SD	0.28	0.17	0.79	1.7	0.28	1.7
vg	N	4	4	4	4	4	4
Group 2	tCtrl	0.95	0.99	1.04	1.00	1.07	1.00
1.1x	Mean	2.32	9.70	8.42	143.0	4.64	101.6
10E12	SD	0.24	0.31	1.21	2.9	0.17	1.3
vg	N	5	5	5	5	5	5
Group 3	tCtrl	0.97	0.99	0.96	1.01	0.95	1.00
3.3x	Mean	2.22	9.90	8.12	142.8	5.08	101.8
10E12	SD	0.27	0.29	1.13	3.0	0.40	1.9
vg	N	5	5	5	5	5	5
Group 4	tCtrl	0.93	1.01	0.93	1.01	1.04	1.00

[G] - Anova & Dunnett

Appendix 16

Table 3

Summary of Clinical Chemistry Values

5550008

Day: 29 Relative to Start Date

Sex: Female		AST (U/L) [G]	ALT (U/L) [G]	ALP (U/L) [G]	GGT (U/L) [G1]	CK (U/L) [G1]	TBIL (mg/dL) [G]	UREAN (mg/dL) [G]
0	Mean	71.6	22.8	90.0	1.5	203.4	0.078	15.2
vg	SD	6.2	3.3	11.0	0.0	67.6	0.024	1.6
	N	5	5	5	5	5	5	5
Group 1		-	-	-	-	-	-	-
0.36x	Mean	79.2	25.8	85.6	1.5	323.8	0.068	15.6
10E12	SD	13.3	7.6	20.1	0.0	234.3	0.019	2.1
vg	N	5	5	5	5	5	5	5
Group 2	tCtrl	1.11	1.13	0.95	1.00	1.59	0.87	1.03
1.1x	Mean	79.8	23.8	103.0	1.5	188.6	0.056	17.0
10E12	SD	16.2	3.7	13.5	0.0	49.7	0.011	2.0
vg	N	5	5	5	5	5	5	5
Group 3	tCtrl	1.11	1.04	1.14	1.00	0.93	0.72	1.12
3.3x	Mean	70.0	26.2	83.6	1.5	178.6	0.068	21.6 **
10E12	SD	5.7	2.8	27.2	0.0	31.1	0.029	3.2
vg	N	5	5	5	5	5	5	5
Group 4	tCtrl	0.98	1.15	0.93	1.00	0.88	0.87	1.42

Reporting Biochemistry

[G] - Anova & Dunnett: ** = p ≤ 0.01
[G1] - Kruskal-Wallis & Dunn

Appendix 16

Table 3

Summary of Clinical Chemistry Values

5550008

Day: 29 Relative to Start Date

Sex: Female		CREAT (mg/dL) [G]	GLUC (mg/dL) [G]	CHOL (mg/dL) [G1]	TRIG (mg/dL) [G]	TPROT (g/dL) [G]	ALB (g/dL) [G]	GLOB (g/dL) [G]
0	Mean	0.36	174.2	71.4	27.2	6.08	4.56	1.52
vg	SD	0.05	31.1	15.7	4.0	0.43	0.33	0.19
	N	5	5	5	5	5	5	5
Group 1		-	-	-	-	-	-	-
0.36x	Mean	0.42	157.2	59.4	24.6	6.30	4.58	1.72
10E12	SD	0.11	10.2	16.2	8.3	0.25	0.19	0.11
vg	N	5	5	5	5	5	5	5
Group 2	tCtrl	1.17	0.90	0.83	0.90	1.04	1.00	1.13
1.1x	Mean	0.38	185.4	65.8	26.0	6.14	4.48	1.66
10E12	SD	0.04	33.6	6.0	6.1	0.31	0.27	0.05
vg	N	5	5	5	5	5	5	5
Group 3	tCtrl	1.06	1.06	0.92	0.96	1.01	0.98	1.09
3.3x	Mean	0.36	193.8	67.8	24.0	6.22	4.44	1.78 *
10E12	SD	0.05	41.8	5.4	4.6	0.50	0.38	0.13
vg	N	5	5	5	5	5	5	5
Group 4	tCtrl	1.00	1.11	0.95	0.88	1.02	0.97	1.17

Reporting Biochemistry

[G] - Anova & Dunnett: * = p ≤ 0.05
[G1] - Kruskal-Wallis & Dunn

Appendix 16

Table 3

Summary of Clinical Chemistry Values

5550008

Day: 29 Relative to Start Date

Sex: Female		Reporting Biochemistry					
		A/G (ratio) [G]	CA (mg/dL) [G1]	PHOS (mg/dL) [G1]	NA (mmol/L) [G1]	K (mmol/L) [G1]	CL (mmol/L) [G]
0	Mean	3.04	10.28	7.84	145.0	4.06	105.0
vg	SD	0.34	0.34	1.23	4.7	0.40	3.2
	N	5	5	5	5	5	5
Group 1		-	-	-	-	-	-
0.36x	Mean	2.66	10.28	7.56	142.6	4.04	102.8
10E12	SD	0.15	0.23	0.67	3.2	0.50	0.8
vg	N	5	5	5	5	5	5
Group 2	tCtrl	0.88	1.00	0.96	0.98	1.00	0.98
1.1x	Mean	2.68	10.06	8.10	144.4	3.88	104.0
10E12	SD	0.13	0.27	1.57	3.0	0.25	1.9
vg	N	5	5	5	5	5	5
Group 3	tCtrl	0.88	0.98	1.03	1.00	0.96	0.99
3.3x	Mean	2.50 **	10.18	8.44	143.6	4.30	103.0
10E12	SD	0.07	0.57	0.80	3.4	0.61	3.0
vg	N	5	5	5	5	5	5
Group 4	tCtrl	0.82	0.99	1.08	0.99	1.06	0.98

[G] - Kruskal-Wallis & Dunn: ** = p ≤ 0.01
[G1] - Anova & Dunnett

Appendix 16

Table 3

Summary of Clinical Chemistry Values

5550008

Day: 91 Relative to Start Date

Sex: Male		Reporting Biochemistry						
		AST (U/L) [G]	ALT (U/L) [G]	ALP (U/L) [G]	GGT (U/L) [G1]	CK (U/L) [G]	TBIL (mg/dL) [G]	UREAN (mg/dL) [G]
0	Mean	73.2	28.2	69.6	1.5	320.4	0.050	13.2
vg	SD	7.3	4.1	15.7	0.0	140.2	0.021	2.2
	N	5	5	5	5	5	5	5
Group 1		-	-	-	-	-	-	-
0.36x	Mean	87.4	33.4	82.8	1.5	375.2	0.062	13.2
10E12	SD	14.4	5.9	19.0	0.0	234.0	0.013	1.5
vg	N	5	5	5	5	5	5	5
Group 2	tCtrl	1.19	1.18	1.19	1.00	1.17	1.24	1.00
1.1x	Mean	86.2	39.6	72.8	1.5	268.6	0.052	12.2
10E12	SD	25.2	11.7	15.1	0.0	148.2	0.015	1.3
vg	N	5	5	5	5	5	5	5
Group 3	tCtrl	1.18	1.40	1.05	1.00	0.84	1.04	0.92
3.3x	Mean	103.2	42.0	78.0	1.5	401.8	0.072	13.2
10E12	SD	66.8	23.8	22.4	0.0	372.4	0.029	1.9
vg	N	5	5	5	5	5	5	5
Group 4	tCtrl	1.41	1.49	1.12	1.00	1.25	1.44	1.00

[G] - Anova & Dunnett
[G1] - Kruskal-Wallis & Dunn

Appendix 16

Table 3

Summary of Clinical Chemistry Values

5550008

Day: 91 Relative to Start Date

Sex: Male		CREAT (mg/dL) [G]	GLUC (mg/dL) [G1]	CHOL (mg/dL) [G1]	TRIG (mg/dL) [G1]	TPROT (g/dL) [G1]	ALB (g/dL) [G1]	GLOB (g/dL) [G1]
0	Mean	0.36	203.8	74.6	101.0	6.08	3.80	2.28
vg	SD	0.05	20.2	8.0	91.1	0.43	0.19	0.43
	N	5	5	5	5	5	5	5
Group 1		-	-	-	-	-	-	-
0.36x	Mean	0.30	215.2	86.4	96.2	6.18	3.90	2.28
10E12	SD	0.00	43.4	24.3	10.5	0.23	0.16	0.13
vg	N	5	5	5	5	5	5	5
Group 2	tCtrl	0.83	1.06	1.16	0.95	1.02	1.03	1.00
1.1x	Mean	0.34	225.8	83.6	70.0	6.26	3.86	2.40
10E12	SD	0.05	29.8	14.3	34.9	0.11	0.05	0.10
vg	N	5	5	5	5	5	5	5
Group 3	tCtrl	0.94	1.11	1.12	0.69	1.03	1.02	1.05
3.3x	Mean	0.30	248.6	70.8	53.2	5.98	3.74	2.24
10E12	SD	0.00	27.0	25.9	37.3	0.28	0.15	0.27
vg	N	5	5	5	5	5	5	5
Group 4	tCtrl	0.83	1.22	0.95	0.53	0.98	0.98	0.98

Reporting Biochemistry

[G] - Kruskal-Wallis & Dunn
[G1] - Anova & Dunnett

Appendix 16

Table 3

Summary of Clinical Chemistry Values

5550008

Day: 91 Relative to Start Date

Sex: Male		Reporting Biochemistry					
		A/G (ratio) [G]	CA (mg/dL) [G]	PHOS (mg/dL) [G]	NA (mmol/L) [G]	K (mmol/L) [G]	CL (mmol/L) [G]
0	Mean	1.70	9.64	7.04	141.0	5.00	102.2
vg	SD	0.35	0.38	1.17	1.6	0.25	2.3
	N	5	5	5	5	5	5
Group 1		-	-	-	-	-	-
0.36x	Mean	1.72	9.96	7.32	140.8	4.66	103.4
10E12	SD	0.08	0.21	0.57	2.2	0.19	2.3
vg	N	5	5	5	5	5	5
Group 2	tCtrl	1.01	1.03	1.04	1.00	0.93	1.01
1.1x	Mean	1.62	9.74	7.40	139.6	4.60	102.2
10E12	SD	0.08	0.34	0.89	1.5	0.34	1.5
vg	N	5	5	5	5	5	5
Group 3	tCtrl	0.95	1.01	1.05	0.99	0.92	1.00
3.3x	Mean	1.68	9.56	7.10	140.6	4.70	102.2
10E12	SD	0.28	0.22	0.98	1.5	0.35	1.3
vg	N	5	5	5	5	5	5
Group 4	tCtrl	0.99	0.99	1.01	1.00	0.94	1.00

[G] - Anova & Dunnett

Appendix 16

Table 3

Summary of Clinical Chemistry Values

5550008

Day: 91 Relative to Start Date

Sex: Female		AST (U/L) [G]	ALT (U/L) [G]	ALP (U/L) [G]	GGT (U/L) [G1]	CK (U/L) [G]	TBIL (mg/dL) [G]	UREAN (mg/dL) [G]
0	Mean	118.6	42.4	38.6	1.5	281.4	0.074	19.0
vg	SD	35.7	20.2	11.5	0.0	216.6	0.019	2.9
	N	5	5	5	5	5	5	5
Group 1		-	-	-	-	-	-	-
0.36x	Mean	98.6	31.8	34.2	1.5	283.0	0.088	15.6
10E12	SD	19.2	5.2	8.7	0.0	96.5	0.032	1.3
vg	N	5	5	5	5	5	5	5
Group 2	tCtrl	0.83	0.75	0.89	1.00	1.01	1.19	0.82
1.1x	Mean	98.4	38.4	33.6	1.5	444.8	0.090	14.6
10E12	SD	27.3	13.1	6.5	0.0	236.1	0.019	3.2
vg	N	5	5	5	5	5	5	5
Group 3	tCtrl	0.83	0.91	0.87	1.00	1.58	1.22	0.77
3.3x	Mean	90.0	29.8	34.2	1.5	219.8	0.078	14.8
10E12	SD	23.0	11.9	6.2	0.0	60.9	0.013	4.3
vg	N	5	5	5	5	5	5	5
Group 4	tCtrl	0.76	0.70	0.89	1.00	0.78	1.05	0.78

Reporting Biochemistry

[G] - Anova & Dunnett
[G1] - Kruskal-Wallis & Dunn

Appendix 16

Table 3

Summary of Clinical Chemistry Values

5550008

Day: 91 Relative to Start Date

Sex: Female		CREAT (mg/dL) [G]	GLUC (mg/dL) [G]	CHOL (mg/dL) [G]	TRIG (mg/dL) [G]	TPROT (g/dL) [G]	ALB (g/dL) [G]	GLOB (g/dL) [G]
0	Mean	0.46	189.4	70.0	34.4	6.80	4.88	1.92
vg	SD	0.05	52.8	7.7	13.2	0.51	0.51	0.13
	N	5	5	5	5	5	5	5
Group 1		-	-	-	-	-	-	-
0.36x	Mean	0.44	171.6	87.0	39.2	7.32	5.28	2.04
10E12	SD	0.11	35.8	22.3	14.6	0.36	0.31	0.18
vg	N	5	5	5	5	5	5	5
Group 2	tCtrl	0.96	0.91	1.24	1.14	1.08	1.08	1.06
1.1x	Mean	0.44	172.8	81.4	37.8	7.08	5.18	1.90
10E12	SD	0.09	49.3	21.8	13.0	0.65	0.47	0.20
vg	N	5	5	5	5	5	5	5
Group 3	tCtrl	0.96	0.91	1.16	1.10	1.04	1.06	0.99
3.3x	Mean	0.42	196.6	72.4	38.6	7.06	5.12	1.94
10E12	SD	0.04	26.4	14.7	22.1	0.65	0.63	0.09
vg	N	5	5	5	5	5	5	5
Group 4	tCtrl	0.91	1.04	1.03	1.12	1.04	1.05	1.01

Reporting Biochemistry

[G] - Anova & Dunnett

Appendix 16

Table 3

Summary of Clinical Chemistry Values

5550008

Day: 91 Relative to Start Date

Sex: Female		A/G (ratio) [G]	CA (mg/dL) [G]	PHOS (mg/dL) [G]	NA (mmol/L) [G]	K (mmol/L) [G]	CL (mmol/L) [G]
0	Mean	2.54	10.00	6.74	139.0	4.02	103.2
vg	SD	0.36	0.42	0.56	2.7	0.51	2.2
	N	5	5	5	5	5	5
Group 1		-	-	-	-	-	-
0.36x	Mean	2.62	10.28	7.12	140.0	4.12	101.8
10E12	SD	0.31	0.52	1.00	2.0	0.86	0.8
vg	N	5	5	5	5	5	5
Group 2	tCtrl	1.03	1.03	1.06	1.01	1.02	0.99
1.1x	Mean	2.74	10.34	6.28	139.2	4.08	102.6
10E12	SD	0.18	0.31	1.13	2.4	0.24	1.5
vg	N	5	5	5	5	5	5
Group 3	tCtrl	1.08	1.03	0.93	1.00	1.01	0.99
3.3x	Mean	2.64	10.06	6.44	139.2	3.92	100.8
10E12	SD	0.31	0.19	0.73	1.3	0.31	1.3
vg	N	5	5	5	5	5	5
Group 4	tCtrl	1.04	1.01	0.96	1.00	0.98	0.98

Reporting Biochemistry

[G] - Anova & Dunnett

Appendix 16

Table 4

Summary of Urinalysis Values

5550008

Day: 8 Relative to Start Date

Sex: Male		Reporting Urinalysis		
		VOLUME (mL) [G]	SPECIFIC GRAVITY [G1]	URINE pH [G]
0	Mean	18.98	1.0178	6.75
vg	SD	14.63	0.0144	0.96
	N	4	4	4
Group 1		-	-	-
0.36x	Mean	8.44	1.0198	6.70
10E12	SD	2.84	0.0073	0.27
vg	N	5	5	5
Group 2	tCtrl	0.44	-	0.99
1.1x	Mean	23.38	1.0132	6.70
10E12	SD	21.46	0.0079	0.27
vg	N	5	5	5
Group 3	tCtrl	1.23	-	0.99
3.3x	Mean	7.84	1.0244	7.20
10E12	SD	8.23	0.0106	0.27
vg	N	5	5	5
Group 4	tCtrl	0.41	-	1.07

[G] - Kruskal-Wallis & Dunn
[G1] - Anova & Dunnett

Appendix 16

Table 4

Summary of Urinalysis Values

5550008

Day: 8 Relative to Start Date

Sex: Female		Reporting Urinalysis VOLUME (mL) [G]	SPECIFIC GRAVITY [G]	URINE pH [G]
0	Mean	6.22	1.0280	6.80
vg	SD	2.16	0.0132	0.45
	N	5	5	5
Group 1		-	-	-
0.36x	Mean	11.88	1.0168	6.60
10E12	SD	6.28	0.0057	0.42
vg	N	5	5	5
Group 2	tCtrl	1.91	-	0.97
1.1x	Mean	7.14	1.0250	6.80
10E12	SD	2.97	0.0116	0.45
vg	N	5	5	5
Group 3	tCtrl	1.15	-	1.00
3.3x	Mean	12.08	1.0210	6.70
10E12	SD	7.86	0.0165	0.57
vg	N	5	5	5
Group 4	tCtrl	1.94	-	0.99

[G] - Anova & Dunnett

Appendix 16

Table 4

Summary of Urinalysis Values

5550008

Day: 29 Relative to Start Date

Sex: Male		Reporting Urinalysis		
		VOLUME (mL) [G]	SPECIFIC GRAVITY [G1]	URINE pH [G]
0 vg Group 1	Mean	29.64	1.0090	7.50
	SD	11.08	0.0028	0.00
	N	5	5	5
		-	-	-
0.36x 10E12 vg Group 2	Mean	8.78 **	1.0328 **	6.80 **
	SD	6.35	0.0150	0.27
	N	5	5	5
	tCtrl	0.30	-	0.91
1.1x 10E12 vg Group 3	Mean	9.26 **	1.0272 *	7.00 *
	SD	6.17	0.0157	0.35
	N	5	5	5
	tCtrl	0.31	-	0.93
3.3x 10E12 vg Group 4	Mean	13.46 *	1.0270	6.90 **
	SD	8.14	0.0129	0.22
	N	5	5	5
	tCtrl	0.45	-	0.92

[G] - Anova & Dunnett: * = p ≤ 0.05; ** = p ≤ 0.01
[G1] - Kruskal-Wallis & Dunn: * = p ≤ 0.05; ** = p ≤ 0.01

Appendix 16

Table 4

Summary of Urinalysis Values

5550008

Day: 29 Relative to Start Date

Sex: Female		Reporting Urinalysis		
		VOLUME (mL) [G]	SPECIFIC GRAVITY [G1]	URINE pH [G1]
0 vg Group 1	Mean	6.56	1.0240	6.80
	SD	4.51	0.0108	0.27
	N	5	5	5
		-	-	-
0.36x 10E12 vg Group 2	Mean	9.02	1.0248	6.60
	SD	6.34	0.0160	0.42
	N	5	5	5
	tCtrl	1.38	-	0.97
1.1x 10E12 vg Group 3	Mean	9.20	1.0230	6.70
	SD	8.01	0.0122	0.27
	N	5	5	5
	tCtrl	1.40	-	0.99
3.3x 10E12 vg Group 4	Mean	11.10	1.0372	6.70
	SD	12.79	0.0375	0.45
	N	5	5	5
	tCtrl	1.69	-	0.99

[G] - Kruskal-Wallis & Dunn
[G1] - Anova & Dunnett

Appendix 16

Table 4

Summary of Urinalysis Values

5550008

Day: 91 Relative to Start Date

Sex: Male		Reporting Urinalysis VOLUME (mL) [G]	Reporting Urinalysis SPECIFIC GRAVITY [G]	Reporting Urinalysis URINE pH [G]
0	Mean	6.14	1.0464	7.10
vg	SD	5.32	0.0243	0.96
	N	5	5	5
Group 1		-	-	-
0.36x	Mean	10.22	1.0262	7.60
10E12	SD	2.80	0.0082	0.22
vg	N	5	5	5
Group 2	tCtrl	1.66	-	1.07
1.1x	Mean	11.62	1.0336	7.40
10E12	SD	10.53	0.0246	0.42
vg	N	5	5	5
Group 3	tCtrl	1.89	-	1.04
3.3x	Mean	8.98	1.0408	7.20
10E12	SD	7.74	0.0200	0.57
vg	N	5	5	5
Group 4	tCtrl	1.46	-	1.01

[G] - Anova & Dunnett

Appendix 16

Table 4

Summary of Urinalysis Values

5550008

Day: 91 Relative to Start Date

Sex: Female		Reporting Urinalysis VOLUME (mL) [G]	Reporting Urinalysis SPECIFIC GRAVITY [G]	Reporting Urinalysis URINE pH [G]
0	Mean	4.06	1.0448	6.50
vg	SD	6.32	0.0236	0.41
	N	5	4	4
Group 1		-	-	-
0.36x	Mean	10.64	1.0236	6.50
10E12	SD	13.38	0.0132	0.35
vg	N	5	5	5
Group 2	tCtrl	2.62	-	1.00
1.1x	Mean	8.86	1.0216	6.40
10E12	SD	6.77	0.0126	0.42
vg	N	5	5	5
Group 3	tCtrl	2.18	-	0.98
3.3x	Mean	7.64	1.0226	6.60
10E12	SD	2.13	0.0083	0.22
vg	N	5	5	5
Group 4	tCtrl	1.88	-	1.02

[G] - Anova & Dunnett

Individual Hematology Values Explanation Page

ADVIA 120/2120i Analyzer
Analyzed Parameter Descriptions

Parameter	Abbreviation	Units	Methodology
Hematocrit	HCT	%	Calculated
Hemoglobin	HGB	g/dL	Colorimetric
Mean Corpuscular Hemoglobin	MCH	pg	Calculated
Mean Corpuscular Hemoglobin Concentration	MCHC	g/dL	Calculated
Mean Corpuscular Volume	MCV	fL	Calculated
Mean Platelet Volume	MPV	fL	Calculated
Platelet Distribution Width	PDW	%	Calculated
Platelet Count	PLT	$x10^3/\mu L$	Light scatter
Red Blood Cell Count	RBC	$x10^6/\mu L$	Light scatter
Red Blood Cell Distribution Width	RDW	%	Calculated
Reticulocytes	RETIC	$x10^9/L$	Calculated
Reticulocytes Percent	RETIC	%	Light scatter
White Blood Cell Count	WBC	$x10^3/\mu L$	Light scatter
White Blood Cell Differential Count			
Neutrophils Percent	NEUT	%	Light scatter
Lymphocytes Percent	LYMPH	%	Light scatter
Monocytes Percent	MONO	%	Light scatter
Eosinophils Percent	EOS	%	Light scatter
Basophils Percent	BASO	%	Light scatter
Large Unstained Cells Percent	LUC	%	Light scatter
Neutrophils	NEUT	$x10^3/\mu L$	Calculated
Lymphocytes	LYMPH	$x10^3/\mu L$	Calculated
Monocytes	MONO	$x10^3/\mu L$	Calculated
Eosinophils	EOS	$x10^3/\mu L$	Calculated
Basophils	BASO	$x10^3/\mu L$	Calculated
Large Unstained Cells	LUC	$x10^3/\mu L$	Calculated

Manual and Visual
Analyzed Parameter Descriptions

Parameter	Abbreviation	Units	Methodology
White Blood Cell Differential Count		% and/or $x10^3/\mu L$	Microscopic enumeration (100 white cells)
- Immature Neutrophils Count	IMM NEUT		
- Immature Neutrophils Percent	IMM NEUT		
- Immature Cells Percent	IMM CELL		
- Immature Cells Count	IMM CELL		
- Large Platelets	LPLT		
- Neutrophils Band Form	NEUT BAND		
- Neutrophils Band Form Percent	NEUT BAND		
- Packed Cell Volume	PCV		
- Neutrophils	NEUT		

Appendix 16

- Lymphocytes	LYMPH		
- Monocytes	MONO		
- Eosinophils	EOS		
- Basophils	BASO		
Others			
Bone Marrow Stain		None	Manual, Wright-Giemsa stain
Bone Marrow Slide Fixation		None	Manual, Fixative

Aerospray Automated Slide Stainer

Analyzed Parameter Descriptions

Parameter	Abbreviation	Units	Methodology
White Blood Cell Differential Stain		None	2 parts aqueous stain (Eosin-Thiazin)

Midas III Slide Stainer

Analyzed Parameter Descriptions

Parameter	Abbreviation	Units	Methodology
White Blood Cell Differential Stain		None	Wright-Giemsa stain
Bone Marrow Stain		None	Wright-Giemsa stain
Bone Marrow Slide Fixation		None	Fixative

Other Abbreviations

Abbreviation	Description	Abbreviation	Description
./-	Not required for veterinary monitoring / Not scheduled to be performed / No findings / Not evaluated / Dead	OA	Omitted activity
ADQ	Adequate	QNS	Quantity not sufficient
CLOT	Sample clotted	RC	Result comment
COMM	Comment added	SC	Sample comment
DEC	Decreased	SNR	Sample not received
FC	Flag comment	Unsc	Unscheduled
INC	Increased	UPTD	Unable to perform due to technical difficulty
LIF	Laboratory Investigation Form	UTD	Unable to determine
MDIFF	Manual differential	UTDM	Unable to determine, not confirmed by microscopy
NAF	No abnormal findings	UTDR	Unable to determine, results not reproducible
NSCH	Not scheduled to be performed	Vet	Collection for veterinary monitoring
NT	Not taken		

Note: This is a comprehensive list of systems, parameters and/or abbreviations. Everything listed above may not be applicable to this report.

Note: Additional morphology for flagged samples may be reported if applicable.

Appendix 16

Dosing Information

Dosing information is abbreviated on various data outputs; the following represents the dosing information for this study.

Group No.	Test Material	Dose Level (vg)
1	Reference Item	0
2	AAV9/AP4M1	0.36×10^{12}
3	AAV9/AP4M1	1.1×10^{12}
4	AAV9/AP4M1	3.3×10^{12}

Individual Coagulation Values Explanation Page

START 4 Compact Stago Analyzer

Analyzed Parameter Descriptions

Parameter	Abbreviation	Units	Methodology
Activated Partial Thromboplastin Time	APTT	sec	Viscosity
Fibrinogen	FIB	mg/dL	Viscosity
Prothrombin Time	PT	sec	Viscosity

STA Compact Stago Analyzer

Analyzed Parameter Descriptions

Parameter	Abbreviation	Units	Methodology
Activated Partial Thromboplastin Time	APTT	sec	Viscosity
Fibrinogen	FIB	mg/dL	Viscosity
Prothrombin Time	PT	sec	Viscosity

Plasma Appearance
(Reported as SAMQ Coagulation)

Analyzed Parameter Descriptions

Parameter	Abbreviation	Degree is graded as	Methodology
Normal sample	N	Normal	Manual and visual
Hemolyzed sample	H	+ = slight (pale/light red)<br>++ = moderate (red)<br>+++ = severe (dark red)	Manual and visual
Lipemic sample	L	+ = slight (cloudy)<br>++ = moderate (turbid)<br>+++ = severe (lactescent)	Manual and visual
Icterus sample	I	+ = slight (dark yellow)<br>++ = moderate (very dark yellow)<br>+++ = severe (dark yellow-green)	Manual and visual

Other Abbreviations

Abbreviation	Description	Abbreviation	Description
./-	Not required for veterinary monitoring / Not scheduled to be performed / No findings / Not evaluated / Dead	QNS	Quantity not sufficient
CLOT	Sample clotted	RC	Result comment
COMM	Comment added	SC	Sample comment
FC	Flag comment	SNR	Sample not received
LIF	Laboratory Investigation Form	Unsc	Unscheduled
NCD	No clot detected	UPTD	Unable to perform due to technical difficulty
NSCH	Not scheduled to be performed	UTD	Unable to determine
NT	Not taken	UTDR	Unable to determine, results not reproducible
OA	Omitted activity	Vet	Collection for veterinary monitoring

Note: This is a comprehensive list of systems, parameters and/or abbreviations. Everything listed above may not be applicable to this report.

Dosing Information

Dosing information is abbreviated on various data outputs; the following represents the dosing information for this study.

Group No.	Test Material	Dose Level (vg)
1	Reference Item	0
2	AAV9/AP4M1	0.36×10^{12}
3	AAV9/AP4M1	1.1×10^{12}
4	AAV9/AP4M1	3.3×10^{12}

Individual Clinical Chemistry Values Explanation Page

Modular Analytics / Cobas 6000 Analyzer

Analyzed Parameter Descriptions

Parameter	Abbreviation	Units	Methodology
Alanine Aminotransferase	ALT	U/L	ALT IFCC UV
Albumin	ALB	g/dL	Bromcresol green colorimetric
Alkaline Phosphatase	ALP	U/L	ALP IFCC liquid colorimetric
Amylase	AMYL	U/L	Enzymatic colorimetric
Aspartate Aminotransferase	AST	U/L	AST IFCC UV
Calcium (Alternate)	CA	mg/dL	O-cresolphthalein complexone colorimetric
Cholesterol	CHOL	mg/dL	CHOD-PAP enzymatic colorimetric
Creatinine	CREAT	mg/dL	Jaffe kinetic colorimetric. Rate-blanked and compensated
Creatine Kinase	CK	U/L	NAC activated UV
Direct Bilirubin	DBIL	mg/dL	Jendrassik colorimetric
GAMMA-Glutamyl Transferase	GGT	U/L	Nitro-Anilide, Glycylglycine; enzymatic colorimetric
Glutamate Dehydrogenase	GLDH	U/L	Kinetic UV
Glucose	GLUC	mg/dL	Hexokinase UV
Iron	FE	µg/dL	Colorimetric
Lactate	LACT	mg/dL	Enzymatic colorimetric
Magnesium	MG	mg/dL	Colorimetric
Phosphorus	PHOS	mg/dL	Molybdate UV
Sodium, Potassium, Chloride (SI)	NA,K,CL	mmol/L	Indirect measurement (Ion selective electrode)
Total Bilirubin	TBIL	mg/dL	DPD colorimetric
Total Protein	TPROT	g/dL	Biuret colorimetric
Triglycerides	TRIG	mg/dL	GPO-PAP enzymatic colorimetric
Urea Nitrogen	UREAN	mg/dL	Urease kinetic UV

Calculations

Analyzed Parameter Descriptions

Parameter	Abbreviation	Units	Calculation
Albumin/Globulin ratio	A/G	ratio	Albumin / Globulin
Globulin	GLOB	g/dL	Total Protein - Albumin
Indirect Bilirubin	IBIL	mg/dL	Total Bilirubin - Direct Bilirubin
Urea Nitrogen / Creatinine ratio	UREAN/CREAT	None	Urea Nitrogen / Creatinine

Serum Appearance (Reported as SAMQ)

Analyzed Parameter Descriptions

Parameter	Abbreviation	Key to Results (Code)	Methodology
Normal sample	N	Normal	Manual and visual
Hemolyzed sample	H	+ = slight (pale/light red)<br>++ = moderate (red)<br>+++ = severe (dark red)	Manual and visual
Lipemic sample	L	+ = slight (cloudy)<br>++ = moderate (turbid)<br>+++ = severe (lactescent)	Manual and visual
Icterus sample	I	+ = slight (dark yellow)<br>++ = moderate (very dark yellow)<br>+++ = severe (dark yellow-green)	Manual and visual

Other Abbreviations

Abbreviation	Description	Abbreviation	Description
./-	Not required for veterinary monitoring / Not scheduled to be performed / No findings / Not evaluated / Dead	SC	Sample comment
CLOT	Sample clotted	SNR	Sample not received
COMM	Comment added	TNR	Test not reported
FC	Flag comment	TTSM	Sample was analyzed 3 times (original, 1st and 2nd repeats), values not comparable (not reported)
LIF	Laboratory Investigation Form	Unsc	Unscheduled
LLOQ	Less than lower limit of quantitation	UPTD	Unable to perform due to technical difficulty
NC	Not calculable	UTD	Unable to determine
NSCH	Not scheduled to be performed	UTDH	Unable to determine due to marked hemolysis
NT	Not taken	UTDL	Unable to determine due to marked lipemia
OA	Omitted activity	UTDR	Unable to determine, results not reproducible
QNS	Quantity not sufficient	Vet	Collection for veterinary monitoring
RC	Result comment	EDTA	Ethylenediaminetetraacetic acid
SST	Serum separator tube		

Note: This is a comprehensive list of systems, parameters and/or abbreviations. Everything listed above may not be applicable to this report.

Appendix 16

Dosing Information

Dosing information is abbreviated on various data outputs; the following represents the dosing information for this study.

Group No.	Test Material	Dose Level (vg)
1	Reference Item	0
2	AAV9/AP4M1	0.36×10^{12}
3	AAV9/AP4M1	1.1×10^{12}
4	AAV9/AP4M1	3.3×10^{12}

Appendix 16

Individual Urinalysis Values Explanation Page

Clinitek ATLAS/ Clinitek NOVUS

Analyzed Parameter Descriptions

Parameter	Abbreviation	Key to Results (Code)<br>Macroscopic Urinalysis	Methodology
Urine Bilirubin	BIL	Negative (Neg), 1+ (small), 2+ (Moderate), 3+ (Large)	Automated reflectance spectrophotometer
Urine Blood	BLD	Negative (Neg), 1+ (Trace), 2+ (Small), 3+ (Moderate), 4+ (Large)	Automated reflectance spectrophotometer
Urine Color	COLOR	Co = Colorless, LY = Light Yellow, DY = Dark Yellow, Or = Orange, Re= Red, Br = Brown, Gr = Green, Ot = Other (color is identified)	Automated reflectance spectrophotometer
Urine Clarity	CLARITY	Clr = Clear, Cld = Cloudy, Tur= Turbid	Automated reflectance spectrophotometer
Urine Glucose Alpha	GLUC	Negative (Neg), 1+ (5.5 mmol/L), 2+ (14 mmol/L) ,3+ (28 mmol/L), 4+ (≥55 mmol/L)	Automated reflectance spectrophotometer
Urine Ketones	KET	Negative (Neg), 1+ (Trace), 2+ (1.5 mmol/L), 3+ (3.9 mmol /L), 4+ (≥ 7.8 mmol /L)	Automated reflectance spectrophotometer
Urine Nitrite	NIT	Negative (Neg) (-), Positive (Pos) (+)	Automated reflectance spectrophotometer
Urine pH	URINE pH	4.5, 5.0, 5.5, 6.0, 6.5, 7.0, 7.5, 8.0, 8.5, 9.0, ≥ 9.0	Automated reflectance spectrophotometer
Urine Protein Alpha	PROT	Negative (Neg), 1+ (Trace), 2+ (0.3g/L), 3+ (1.0 g/L), 4+ (≥3.0 g/L)	Automated reflectance spectrophotometer
Urine Specific Gravity	SPECIFIC GRAVITY	1.000 to 1.099 (>=1.099)	Automated reflectance spectrophotometer
Urine Urobilinogen	UROBIL	Negative (Neg), 1+ (16µmol/L), 2+ (33 µmol/L), 3+ (66 µmol/L), 4+ (≥ 131 µmol/L)	Automated reflectance spectrophotometer

Manual and Visual

Analyzed Parameter Descriptions

Parameter	Abbreviation	Key to Results (Code)/Unit	Methodology
Urine Bilirubin	BIL	Negative (Neg), 1+ (small), 2+ (Moderate), 3+ (Large)	Manual Results – Multistix strip
Urine Blood	BLD	Negative (Neg), 1+ (Trace), 2+ (Small), 3+ (Moderate), 4+ (Large)	Manual Results – Multistix strip
Urine Color	COLOR	Co = Colorless, LY = Light Yellow, DY = Dark Yellow, Or = Orange, Re= Red, Br = Brown, Gr = Green, Ot = Other (color is identified)	Manual and visual
Urine Clarity	CLARITY	Clr = Clear, Cld = Cloudy, Tur= Turbid	Manual and visual

Urine Glucose Alpha	GLUC	Negative (Neg), 1+ (5.5 mmol/L), 2+ (14 mmol/L) 3+ (28 mmol/L), 4+ (55 mmol/L or ≥ 111 mmol/L)	Manual Results – Multistix strip
Urine Ketones	KET	Negative (Neg), 1+ (0.5 mmol/L), 2+ (1.5 mmol/L), 3+ (4.0 mmol /L), 4+ (8 mmol /L or 16 mmol/L)	Manual Results – Multistix strip
Urine Nitrite	NIT	Negative (Neg) (-), Positive (Pos) (+)	Manual Results – Multistix strip
Urine pH	URINE pH	4.5, 5.0, 5.5, 6.0, 6.5, 7.0, 7.5, 8.0, 8.5	Manual Results – Multistix strip
Urine Protein Alpha	PROT	Negative (Neg), 1+ (Trace), 2+ (0.3g/L), 3+ (1.0 g/L), 4+ (≥3.0 g/L or ≥ 20.0 g/L)	Manual Results – Multistix strip
Urine Specific Gravity	SPECIFIC GRAVITY	None	Manual Results – Refractive index using the Refractometer
Urine Urobilinogen	UROBIL	Neg (3.2 µmol/L), 1+ (16 µmol/L), 2+ (33 µmol/L), 3+ (66 µmol/L), 4+ (131 µmol/L)	Manual Results – Multistix strip
Urine Volume	VOLUME	mL	Manual and visual

Other Abbreviations

Abbreviation	Description	Abbreviation	Description
./-	Not required for veterinary monitoring / No findings / Not evaluated / Dead	QNS	Quantity not sufficient
COM	Contaminated with organic material	RC	Result comment
COMM	Comment added	SC	Sample comment
FC	Flag comment	SNR	Sample not received
GFC	Gross fecal contamination present	Unsc	Unscheduled collection
LIF	Laboratory Investigation Form	UPTD	Unable to perform due to technical difficulty
NAF	No abnormal findings	UTD	Unable to determine
NSCH	Not scheduled to be performed	UTDR	Unable to determine, results not reproducible
NT	Not taken	Vet	Collection for veterinary monitoring
OA	Omitted activity		

Note: This is a comprehensive list of systems, parameters and/or abbreviations. Everything listed above may not be applicable to this report.

Appendix 16

Dosing Information

Dosing information is abbreviated on various data outputs; the following represents the dosing information for this study.

Group No.	Test Material	Dose Level (vg)
1	Reference Item	0
2	AAV9/AP4M1	0.36×10^{12}
3	AAV9/AP4M1	1.1×10^{12}
4	AAV9/AP4M1	3.3×10^{12}

Appendix 16

Appendix 1

Individual Hematology Values

5550008

Sex: Male

0 vg Group 1	Day(s) Relative to Start Date	WBC (10^3/uL)	NEUT (10^3/uL)	LYMPH (10^3/uL)	MONO (10^3/uL)	EOS (10^3/uL)	BASO (10^3/uL)
		Reporting Hematology					
1001	8	5.95	0.98	4.68	0.21	0.02	0.01
1002	8	9.47	0.95	8.05	0.30	0.06	0.01
1003	29	7.59	1.80	5.49	0.17	0.09	0.01
1004	29	10.67	1.74	8.50	0.28	0.07	0.02
1005	91	6.32	0.94	5.05	0.17	0.10	0.02
1006	91	11.01	1.19	9.48	0.16	0.08	0.02
1007	91	8.87	1.19	7.17	0.24	0.16	0.02
1008	29	8.81	1.67	6.88	0.13	0.08	0.01
1009	29	6.58	1.35	4.98	0.14	0.07	0.00
1010	29	14.47	2.18	11.75	0.36	0.09	0.02
1011	91	4.22	0.72	3.31	0.09	0.08	0.01
1012	91	5.90	0.98	4.67	0.11	0.09	0.00
1013	7 (Unsc)	QNS	QNS	QNS	QNS	QNS	QNS
1014	8	6.23	1.60	4.39	0.11	0.08	0.01
1015	8	5.74	0.56	4.91	0.16	0.03	0.00

Appendix 16

Appendix 1

Individual Hematology Values

5550008

Sex: Male

0 vg Group 1	Day(s) Relative to Start Date	Reporting Hematology LUC (10^3/uL)	RBC (10^6/uL)	HGB (g/dL)	HCT (%)	MCV (fL)	MCH (pg)
1001	8	0.05	6.62	13.2	40.9	61.7	19.9
1002	8	0.09	6.66	13.6	42.9	64.5	20.5
1003	29	0.03	6.87	12.9	39.6	57.6	18.8
1004	29	0.06	7.00	13.4	41.8	59.8	19.2
1005	91	0.04	7.57	14.2	40.8	53.9	18.7
1006	91	0.07	8.17	14.9	44.3	54.3	18.2
1007	91	0.08	8.07	14.1	41.7	51.7	17.5
1008	29	0.05	7.19	14.0	40.9	56.9	19.4
1009	29	0.03	6.58	12.7	38.5	58.5	19.3
1010	29	0.06	7.22	13.7	41.1	56.9	19.0
1011	91	0.02	7.63	13.8	41.3	54.2	18.1
1012	91	0.04	8.03	13.5	41.2	51.3	16.8
1013	7 (Unsc)	QNS	QNS	QNS	QNS	QNS	QNS
1014	8	0.03	5.66	11.1	34.4	60.8	19.6
1015	8	0.07	5.58	11.2	34.7	62.1	20.2

Appendix 16

Appendix 1

Individual Hematology Values

5550008

Sex: Male

0 vg Group 1	Day(s) Relative to Start Date	Reporting Hematology MCHC (g/dL)	RDW (%)	PLT (10^3/uL)	RETIC (10^9/L)
1001	8	32.2	12.9	1377	506.8
1002	8	31.8	13.2	1284	458.1
1003	29	32.7	12.4	1249	251.1
1004	29	32.1	13.5	988	260.7
1005	91	34.8	14.1	1050	215.4
1006	91	33.5	12.4	894	235.0
1007	91	33.8	13.3	1077	263.0
1008	29	34.1	11.4	1263	225.9
1009	29	33.0	11.9	932	226.3
1010	29	33.4	10.7	1039	247.3
1011	91	33.3	12.5	858	185.9
1012	91	32.8	15.0	1015	235.4
1013	7 (Unsc)	QNS	QNS	QNS	QNS
1014	8	32.2	14.4	1120	418.9
1015	8	32.4	13.9	1244	174.3

Appendix 16

Appendix 1

Individual Hematology Values

5550008

Sex: Male

0.36x 10E12 vg Group 2	Day(s) Relative to Start Date	Reporting Hematology WBC (10^3/uL)	NEUT (10^3/uL)	LYMPH (10^3/uL)	MONO (10^3/uL)	EOS (10^3/uL)	BASO (10^3/uL)
2001	29	3.96	0.62	3.21	0.08	0.02	0.00
2002	29	7.24	0.74	6.29	0.09	0.07	0.01
2103	91	6.11	1.30	4.56	0.14	0.07	0.01
2004	8	4.35	0.65	3.46	0.17	0.03	0.01
2005	91	5.66	1.79	3.53	0.20	0.09	0.01
2006	91	7.80	0.99	6.51	0.15	0.08	0.02
2007	91	11.71	1.68	9.41	0.36	0.17	0.03
2008	91	8.04	1.42	6.27	0.17	0.13	0.02
2009	8	6.33	1.11	4.88	0.22	0.06	0.00
2010	8	8.35	1.83	6.06	0.32	0.07	0.01
2011	29	7.71	0.99	6.41	0.14	0.08	0.01
2012	29	6.12	1.39	4.49	0.18	0.04	0.00
2013	29	9.26	1.04	7.89	0.18	0.06	0.02
2014	8	9.56	1.88	7.42	0.13	0.05	0.01
2015	8	4.25	0.80	3.31	0.11	0.01	0.00

Appendix 16

Appendix 1

Individual Hematology Values

5550008

Sex: Male

0.36x 10E12 vg Group 2	Day(s) Relative to Start Date	LUC (10^3/uL)	RBC (10^6/uL)	HGB (g/dL)	HCT (%)	MCV (fL)	MCH (pg)
		Reporting Hematology					
2001	29	0.02	6.37	12.1	37.8	59.3	19.0
2002	29	0.04	7.52	13.8	42.5	56.5	18.3
2103	91	0.03	8.01	14.1	42.1	52.6	17.6
2004	8	0.04	6.65	12.9	40.9	61.6	19.3
2005	91	0.04	7.41	14.1	40.8	55.1	19.0
2006	91	0.07	7.81	14.7	41.8	53.6	18.8
2007	91	0.07	7.83	14.9	42.1	53.8	19.0
2008	91	0.03	7.59	14.1	41.9	55.2	18.6
2009	8	0.06	5.91	11.9	37.9	64.2	20.2
2010	8	0.06	6.52	12.9	43.3	66.4	19.8
2011	29	0.08	7.05	12.8	39.3	55.8	18.2
2012	29	0.03	6.72	12.3	38.4	57.1	18.3
2013	29	0.07	6.81	13.2	40.3	59.1	19.3
2014	8	0.07	6.20	11.8	36.6	59.0	19.1
2015	8	0.02	6.80	13.2	40.3	59.3	19.4

Appendix 16

Appendix 1

Individual Hematology Values

5550008

Sex: Male

0.36x 10E12 vg Group 2	Day(s) Relative to Start Date	Reporting Hematology MCHC (g/dL)	RDW (%)	PLT (10^3/uL)	RETIC (10^9/L)
2001	29	32.0	12.4	1058	248.1
2002	29	32.4	12.2	1141	249.4
2103	91	33.5	13.1	981	201.2
2004	8	31.4	13.7	1179	472.9
2005	91	34.5	13.9	906	232.2
2006	91	35.1	12.3	871	152.4
2007	91	35.3	13.5	806	176.5
2008	91	33.7	13.3	1072	187.6
2009	8	31.4	13.5	1491	423.0
2010	8	29.8	12.9	791	484.0
2011	29	32.6	11.4	1114	188.9
2012	29	32.1	12.7	1221	251.8
2013	29	32.7	11.8	1017	260.2
2014	8	32.3	13.4	1225	411.5
2015	8	32.7	12.7	1242	394.1

Appendix 16

Appendix 1

Individual Hematology Values

5550008

Sex: Male

1.1x 10E12 vg Group 3	Day(s) Relative to Start Date	Reporting Hematology WBC (10^3/uL)	NEUT (10^3/uL)	LYMPH (10^3/uL)	MONO (10^3/uL)	EOS (10^3/uL)	BASO (10^3/uL)
3001	8	8.90	1.75	6.76	0.30	0.03	0.01
3002	8	7.18	0.56	6.36	0.15	0.04	0.00
3003	8	9.42	1.38	7.57	0.32	0.04	0.01
3004	29	6.64	1.64	4.85	0.10	0.02	0.01
3005	29	5.11	1.16	3.82	0.07	0.03	0.00
3006	91	7.38	0.90	6.09	0.21	0.10	0.01
3007	91	8.97	1.24	7.33	0.23	0.10	0.02
3008	91	6.60	1.35	4.76	0.30	0.11	0.01
3009	29	8.98	0.84	7.82	0.17	0.08	0.01
3010	29	10.69	1.57	8.70	0.27	0.09	0.01
3011	29	7.32	1.49	5.49	0.24	0.06	0.00
3012	8	3.18	0.52	2.50	0.12	0.03	0.00
3013	8	6.35	1.10	4.98	0.19	0.05	0.01
3014	91	6.25	0.65	5.32	0.11	0.12	0.01
3015	91	3.63	1.10	2.40	0.06	0.04	0.01

Appendix 16

Appendix 1

Individual Hematology Values

5550008

Sex: Male

1.1x 10E12 vg Group 3	Day(s) Relative to Start Date	Reporting Hematology LUC (10^3/uL)	RBC (10^6/uL)	HGB (g/dL)	HCT (%)	MCV (fL)	MCH (pg)
3001	8	0.04	6.88	13.0	41.7	60.5	18.8
3002	8	0.06	6.23	12.9	40.0	64.2	20.7
3003	8	0.10	6.22	12.5	39.2	63.0	20.1
3004	29	0.03	7.39	13.9	42.6	57.7	18.9
3005	29	0.02	7.27	13.6	41.0	56.4	18.7
3006	91	0.08	8.58	15.5	45.7	53.3	18.1
3007	91	0.06	7.54	13.5	40.0	53.1	17.9
3008	91	0.06	7.84	14.7	41.4	52.7	18.7
3009	29	0.06	6.86	12.9	39.0	56.9	18.8
3010	29	0.06	6.72	12.6	38.4	57.2	18.7
3011	29	0.03	7.41	13.6	41.2	55.6	18.4
3012	8	0.01	6.23	11.9	37.4	60.0	19.0
3013	8	0.02	6.30	13.0	41.0	65.1	20.6
3014	91	0.03	8.63	14.7	45.1	52.3	17.1
3015	91	0.02	8.05	13.8	41.8	51.9	17.1

Appendix 16

Appendix 1

Individual Hematology Values

5550008

Sex: Male

1.1x 10E12 vg Group 3	Day(s) Relative to Start Date	Reporting Hematology MCHC (g/dL)	RDW (%)	PLT (10^3/uL)	RETIC (10^9/L)
3001	8	31.1	13.7	1402	521.2
3002	8	32.2	12.6	1210	421.7
3003	8	31.9	12.8	1242	428.3
3004	29	32.8	11.1	1131	273.5
3005	29	33.2	12.0	951	238.5
3006	91	34.0	12.5	1051	188.2
3007	91	33.8	14.7	1057	273.6
3008	91	35.5	13.1	1128	216.4
3009	29	33.1	11.8	933	224.3
3010	29	32.7	11.7	1013	256.1
3011	29	33.1	11.4	1061	221.7
3012	8	31.7	13.8	1294	403.0
3013	8	31.6	12.4	1431	367.8
3014	91	32.7	11.9	969	146.4
3015	91	33.0	13.7	1178	224.6

Appendix 16

Appendix 1

Individual Hematology Values

5550008

Sex: Male

3.3x 10E12 vg Group 4	Day(s) Relative to Start Date	Reporting Hematology WBC (10^3/uL)	NEUT (10^3/uL)	LYMPH (10^3/uL)	MONO (10^3/uL)	EOS (10^3/uL)	BASO (10^3/uL)
4001	8	7.53	1.63	5.49	0.31	0.04	0.01
4002	8	10.15	1.11	8.46	0.41	0.07	0.02
4003	29	5.23	0.99	4.08	0.12	0.01	0.01
4004	29	15.90	2.70	12.62	0.36	0.08	0.05
4005	29	CLOT	CLOT	CLOT	CLOT	CLOT	CLOT
4006	8	7.57	1.00	6.37	0.14	0.03	0.01
4007	8	5.57	0.64	4.69	0.15	0.05	0.01
4008	8	7.72	1.10	6.35	0.20	0.03	0.00
4009	91	9.80	1.91	7.40	0.33	0.10	0.02
4010	91	10.52	1.39	8.73	0.24	0.10	0.02
4011	91	5.55	1.05	4.25	0.15	0.05	0.01
4012	29	7.10	1.65	5.21	0.14	0.07	0.01
4013	29	8.03	1.31	6.14	0.39	0.09	0.01
4014	91	8.99	2.52	6.02	0.22	0.12	0.02
4015	91	12.82	2.50	9.90	0.28	0.06	0.03

Appendix 16

Appendix 1

Individual Hematology Values

5550008

Sex: Male

3.3x 10E12 vg Group 4	Day(s) Relative to Start Date	Reporting Hematology LUC (10^3/uL)	RBC (10^6/uL)	HGB (g/dL)	HCT (%)	MCV (fL)	MCH (pg)
4001	8	0.05	6.32	12.9	40.8	64.5	20.4
4002	8	0.08	6.40	12.8	41.6	65.1	20.1
4003	29	0.02	6.89	12.9	39.7	57.6	18.8
4004	29	0.09	6.53	13.7	42.1	64.4	20.9
4005	29	CLOT	CLOT	CLOT	CLOT	CLOT	CLOT
4006	8	0.03	7.16	14.9	47.1	65.9	20.8
4007	8	0.03	6.81	13.0	40.7	59.8	19.0
4008	8	0.04	6.44	13.2	41.1	63.8	20.4
4009	91	0.05	7.84	13.7	41.3	52.7	17.4
4010	91	0.04	8.60	15.0	45.8	53.3	17.5
4011	91	0.04	7.96	13.6	40.8	51.3	17.1
4012	29	0.03	7.52	13.6	41.3	54.9	18.1
4013	29	0.09	6.65	13.3	40.6	61.1	20.1
4014	91	0.08	8.27	15.0	44.9	54.3	18.2
4015	91	0.04	7.90	13.9	42.3	53.5	17.6

Appendix 16

Appendix 1

Individual Hematology Values

5550008

Sex: Male

3.3x 10E12 vg Group 4	Day(s) Relative to Start Date	Reporting Hematology MCHC (g/dL)	RDW (%)	PLT (10^3/uL)	RETIC (10^9/L)
4001	8	31.7	13.1	1129	420.6
4002	8	30.9	13.7	1207	490.6
4003	29	32.6	12.4	948	241.8
4004	29	32.5	12.8	1230	260.8
4005	29	CLOT	CLOT	CLOT	CLOT
4006	8	31.6	13.9	1207	468.2
4007	8	31.8	12.6	1373	450.5
4008	8	32.0	13.4	1297	444.5
4009	91	33.1	15.0	1107	234.7
4010	91	32.8	11.8	1083	227.1
4011	91	33.4	13.5	1131	260.0
4012	29	33.0	11.6	1299	234.2
4013	29	32.9	12.3	1002	248.7
4014	91	33.5	12.0	902	189.7
4015	91	32.8	13.3	849	234.6

Appendix 16

Appendix 1

Individual Hematology Values

5550008

Sex: Female

0<br>vg<br>Group 1	Day(s) Relative to Start Date	Reporting Hematology<br>WBC (10^3/uL)	NEUT (10^3/uL)	LYMPH (10^3/uL)	MONO (10^3/uL)	EOS (10^3/uL)	BASO (10^3/uL)
1501	8	4.14	0.95	3.05	0.09	0.03	0.00
1502	8	CLOT	CLOT	CLOT	CLOT	CLOT	CLOT
1503	91	4.67	0.77	3.68	0.12	0.06	0.01
1504	91	3.58	0.84	2.48	0.18	0.05	0.00
1505	8	5.55	1.62	3.73	0.11	0.04	0.01
1506	8	5.35	0.76	4.36	0.13	0.07	0.00
1507	8	5.94	0.87	4.82	0.15	0.04	0.00
1508	29	7.81	1.12	6.32	0.21	0.10	0.00
1509	29	4.05	0.46	3.41	0.11	0.03	0.00
1510	91	CLOT	CLOT	CLOT	CLOT	CLOT	CLOT
1511	91	6.26	0.63	5.40	0.13	0.08	0.01
1512	91	3.99	0.46	3.33	0.12	0.06	0.01
1513	29	CLOT	CLOT	CLOT	CLOT	CLOT	CLOT
1514	29	4.91	0.55	4.17	0.08	0.08	0.00
1515	29	2.53	0.42	1.97	0.09	0.04	0.00

Appendix 16

Appendix 1

Individual Hematology Values

5550008

Sex: Female

0 vg Group 1	Day(s) Relative to Start Date	Reporting Hematology LUC (10^3/uL)	RBC (10^6/uL)	HGB (g/dL)	HCT (%)	MCV (fL)	MCH (pg)
1501	8	0.02	6.31	13.2	39.1	62.0	20.9
1502	8	CLOT	CLOT	CLOT	CLOT	CLOT	CLOT
1503	91	0.03	7.62	13.5	38.6	50.7	17.7
1504	91	0.02	7.65	13.4	39.3	51.4	17.6
1505	8	0.04	6.49	12.6	38.3	59.1	19.4
1506	8	0.02	6.64	12.8	38.0	57.2	19.3
1507	8	0.04	6.41	12.8	39.4	61.5	20.0
1508	29	0.04	7.81	15.0	42.7	54.7	19.2
1509	29	0.04	7.21	13.6	39.7	55.1	18.9
1510	91	CLOT	CLOT	CLOT	CLOT	CLOT	CLOT
1511	91	0.01	7.65	15.0	41.9	54.7	19.6
1512	91	0.02	7.13	14.4	41.9	58.8	20.2
1513	29	CLOT	CLOT	CLOT	CLOT	CLOT	CLOT
1514	29	0.02	7.25	13.5	40.3	55.6	18.6
1515	29	0.01	7.53	14.1	40.9	54.3	18.7

Appendix 16

Appendix 1

Individual Hematology Values

5550008

Sex: Female

0 vg Group 1	Day(s) Relative to Start Date	Reporting Hematology MCHC (g/dL)	RDW (%)	PLT (10^3/uL)	RETIC (10^9/L)
1501	8	33.7	10.8	1181	237.6
1502	8	CLOT	CLOT	CLOT	CLOT
1503	91	34.9	11.1	942	125.7
1504	91	34.1	11.6	1050	160.6
1505	8	32.8	11.4	1536	341.0
1506	8	33.7	11.9	954	323.5
1507	8	32.5	11.8	1338	360.6
1508	29	35.1	10.1	750	168.5
1509	29	34.3	10.0	885	149.9
1510	91	CLOT	CLOT	CLOT	CLOT
1511	91	35.8	11.1	950	136.0
1512	91	34.3	11.3	899	211.5
1513	29	CLOT	CLOT	CLOT	CLOT
1514	29	33.5	10.5	1014	153.7
1515	29	34.4	10.6	918	206.7

Appendix 16

Appendix 1

Individual Hematology Values

5550008

Sex: Female

0.36x 10E12 vg Group 2	Day(s) Relative to Start Date	Reporting Hematology WBC (10^3/uL)	NEUT (10^3/uL)	LYMPH (10^3/uL)	MONO (10^3/uL)	EOS (10^3/uL)	BASO (10^3/uL)
2501	29	5.03	0.66	4.13	0.13	0.08	0.00
2502	29	8.47	2.94	5.20	0.21	0.09	0.01
2503	91	4.33	0.62	3.50	0.13	0.05	0.01
2504	91	2.31	0.28	1.84	0.11	0.07	0.00
2505	8	7.77	0.74	6.74	0.13	0.05	0.01
2506	8	3.84	0.38	3.27	0.11	0.05	0.00
2507	29	4.43	0.44	3.71	0.14	0.10	0.00
2508	29	6.05	0.84	5.03	0.09	0.05	0.01
2509	29	5.56	1.00	4.37	0.11	0.05	0.00
2510	8	7.72	0.69	6.70	0.20	0.06	0.01
2511	8	9.09	1.41	7.24	0.27	0.11	0.01
2512	8	8.14	0.60	7.19	0.20	0.05	0.01
2613	91	1.64	0.27	1.30	0.03	0.03	0.00
2614	91	3.04	0.40	2.52	0.06	0.04	0.01
2615	91	4.85	0.59	4.13	0.08	0.04	0.01

Appendix 16

Appendix 1

Individual Hematology Values

5550008

Sex: Female

0.36x 10E12 vg Group 2	Day(s) Relative to Start Date	LUC (10^3/uL)	RBC (10^6/uL)	HGB (g/dL)	HCT (%)	MCV (fL)	MCH (pg)
		Reporting Hematology					
2501	29	0.02	7.45	14.6	42.4	57.0	19.5
2502	29	0.03	7.06	12.9	38.0	53.9	18.3
2503	91	0.04	7.27	13.5	39.4	54.2	18.6
2504	91	0.02	7.61	14.7	43.5	57.1	19.3
2505	8	0.09	6.41	12.8	38.6	60.2	20.0
2506	8	0.04	6.55	12.7	38.5	58.7	19.4
2507	29	0.04	7.07	13.4	39.4	55.7	19.0
2508	29	0.04	7.25	13.9	41.4	57.1	19.1
2509	29	0.03	7.16	13.3	39.4	55.0	18.6
2510	8	0.06	6.48	13.7	42.9	66.2	21.2
2511	8	0.05	6.67	12.9	39.0	58.5	19.4
2512	8	0.09	6.63	13.7	42.0	63.4	20.7
2613	91	0.00	7.57	13.3	40.3	53.3	17.6
2614	91	0.00	7.21	13.8	41.4	57.5	19.2
2615	91	0.02	7.51	14.4	43.0	57.2	19.1

Appendix 16

Appendix 1

Individual Hematology Values

5550008

Sex: Female

0.36x 10E12 vg Group 2	Day(s) Relative to Start Date	Reporting Hematology MCHC (g/dL)	RDW (%)	PLT (10^3/uL)	RETIC (10^9/L)
2501	29	34.3	11.0	831	176.8
2502	29	33.9	11.0	1081	177.7
2503	91	34.3	11.7	914	198.8
2504	91	33.9	11.7	813	193.9
2505	8	33.2	11.1	1230	341.9
2506	8	33.1	10.6	1277	203.8
2507	29	34.2	10.6	1165	153.0
2508	29	33.4	11.0	1179	180.4
2509	29	33.9	9.8	1120	172.3
2510	8	32.0	12.1	1412	379.7
2511	8	33.1	10.7	1221	281.0
2512	8	32.6	10.7	1191	291.6
2613	91	33.0	12.5	952	151.4
2614	91	33.4	11.2	1075	184.8
2615	91	33.4	11.5	978	150.7

Appendix 16

Appendix 1

Individual Hematology Values

5550008

Sex: Female

1.1x 10E12 vg Group 3	Day(s) Relative to Start Date	Reporting Hematology WBC (10^3/uL)	NEUT (10^3/uL)	LYMPH (10^3/uL)	MONO (10^3/uL)	EOS (10^3/uL)	BASO (10^3/uL)
3501	91	5.75	0.75	4.68	0.19	0.09	0.01
3502	91	4.61	1.26	3.11	0.17	0.05	0.00
3503	91	3.79	1.12	2.27	0.28	0.06	0.00
3504	29	7.52	0.93	6.39	0.13	0.02	0.00
3505	29	8.16	1.48	6.42	0.16	0.05	0.01
3506	29	8.66	1.65	6.76	0.16	0.03	0.01
3507	8	3.15	0.53	2.48	0.07	0.03	0.00
3508	8	6.41	1.28	4.84	0.19	0.05	0.00
3509	91	2.54	0.26	2.13	0.09	0.03	0.00
3510	91	5.67	0.47	4.97	0.13	0.08	0.01
3511	8	6.12	0.71	5.18	0.13	0.04	0.00
3512	8	7.06	0.94	5.82	0.20	0.07	0.01
3513	8	6.74	1.16	5.29	0.19	0.06	0.01
3514	29	5.96	0.71	4.91	0.22	0.08	0.01
3615	29	3.66	0.57	2.87	0.14	0.06	0.00

Appendix 16

Appendix 1

Individual Hematology Values

5550008

Sex: Female

1.1x 10E12 vg Group 3	Day(s) Relative to Start Date	Reporting Hematology LUC (10^3/uL)	RBC (10^6/uL)	HGB (g/dL)	HCT (%)	MCV (fL)	MCH (pg)
3501	91	0.04	7.70	13.5	38.5	50.0	17.5
3502	91	0.02	7.80	14.1	40.9	52.5	18.1
3503	91	0.06	7.87	13.8	39.8	50.6	17.6
3504	29	0.05	7.25	14.3	41.7	57.6	19.8
3505	29	0.05	7.81	14.5	42.3	54.1	18.6
3506	29	0.05	7.03	12.7	38.6	54.9	18.1
3507	8	0.03	6.67	12.8	38.8	58.2	19.2
3508	8	0.04	6.46	13.1	40.1	62.0	20.2
3509	91	0.02	7.35	13.7	40.7	55.4	18.6
3510	91	0.03	7.64	14.0	42.9	56.1	18.3
3511	8	0.04	6.67	13.5	40.8	61.1	20.3
3512	8	0.02	7.22	14.0	41.7	57.7	19.3
3513	8	0.04	7.01	13.4	39.8	56.7	19.1
3514	29	0.04	8.08	14.5	43.4	53.7	18.0
3615	29	0.02	7.49	14.1	42.0	56.2	18.8

Appendix 16

Appendix 1

Individual Hematology Values

5550008

Sex: Female

1.1x 10E12 vg Group 3	Day(s) Relative to Start Date	Reporting Hematology MCHC (g/dL)	RDW (%)	PLT (10^3/uL)	RETIC (10^9/L)
3501	91	34.9	11.2	942	137.2
3502	91	34.4	11.3	989	149.6
3503	91	34.8	11.8	1028	103.0
3504	29	34.3	10.8	964	194.7
3505	29	34.3	10.0	891	213.1
3506	29	32.9	10.1	982	193.9
3507	8	32.9	10.2	1095	275.4
3508	8	32.6	11.5	1193	290.6
3509	91	33.6	11.4	984	187.2
3510	91	32.7	12.4	900	202.2
3511	8	33.1	11.1	1024	217.0
3512	8	33.5	11.7	1092	277.0
3513	8	33.7	12.1	1118	185.5
3514	29	33.5	10.6	1151	171.4
3615	29	33.5	11.5	1068	211.7

Appendix 16

Appendix 1

Individual Hematology Values

5550008

Sex: Female

3.3x 10E12 vg Group 4	Day(s) Relative to Start Date	Reporting Hematology WBC (10^3/uL)	NEUT (10^3/uL)	LYMPH (10^3/uL)	MONO (10^3/uL)	EOS (10^3/uL)	BASO (10^3/uL)
4601	8	4.86	0.54	4.13	0.12	0.03	0.00
4502	8	3.44	0.45	2.76	0.14	0.04	0.00
4503	91	7.76	0.97	6.43	0.19	0.07	0.01
4504	91	3.60	0.55	2.82	0.16	0.04	0.01
4505	29	7.40	0.62	6.58	0.09	0.06	0.00
4506	29	6.01	0.44	5.34	0.13	0.06	0.00
4507	29	6.31	0.61	5.55	0.05	0.05	0.01
4508	8	5.88	0.72	4.83	0.20	0.10	0.01
4509	8	5.27	0.81	4.22	0.15	0.06	0.01
4510	8	5.85	1.07	4.60	0.08	0.07	0.01
4511	29	6.93	0.59	5.87	0.36	0.06	0.00
4512	29	6.97	0.60	6.13	0.11	0.05	0.01
4513	91	7.82	1.54	5.97	0.19	0.08	0.02
4514	91	6.09	1.11	4.69	0.12	0.10	0.00
4515	91	4.60	0.72	3.72	0.08	0.06	0.00

Appendix 16

Appendix 1

Individual Hematology Values

5550008

Sex: Female

3.3x 10E12 vg Group 4	Day(s) Relative to Start Date	Reporting Hematology LUC (10^3/uL)	RBC (10^6/uL)	HGB (g/dL)	HCT (%)	MCV (fL)	MCH (pg)
4601	8	0.04	6.93	13.3	41.1	59.4	19.2
4502	8	0.05	7.06	13.3	41.6	58.9	18.9
4503	91	0.09	8.04	14.2	41.6	51.8	17.7
4504	91	0.02	7.55	13.6	40.2	53.2	18.0
4505	29	0.05	6.86	12.6	38.0	55.4	18.4
4506	29	0.04	7.18	13.9	41.5	57.8	19.4
4507	29	0.05	6.91	13.1	38.3	55.5	19.0
4508	8	0.02	6.79	13.6	41.7	61.3	20.0
4509	8	0.02	6.18	12.4	38.3	62.0	20.1
4510	8	0.02	6.86	12.9	40.3	58.7	18.9
4511	29	0.05	7.75	14.7	42.9	55.4	19.0
4512	29	0.07	6.95	13.7	39.2	56.3	19.7
4513	91	0.02	7.72	14.6	42.9	55.6	18.9
4514	91	0.06	7.86	13.8	41.8	53.2	17.6
4515	91	0.01	6.94	12.7	37.6	54.1	18.4

Appendix 16

Appendix 1

Individual Hematology Values

5550008

Sex: Female

3.3x 10E12 vg Group 4	Day(s) Relative to Start Date	Reporting Hematology MCHC (g/dL)	RDW (%)	PLT (10^3/uL)	RETIC (10^9/L)
4601	8	32.3	11.2	1457	293.5
4502	8	32.0	10.2	1034	279.7
4503	91	34.1	11.9	1080	108.4
4504	91	33.8	11.7	1040	130.0
4505	29	33.2	10.5	1045	205.6
4506	29	33.6	11.5	974	206.2
4507	29	34.2	10.3	1086	223.9
4508	8	32.6	11.1	1489	242.4
4509	8	32.4	11.8	1135	331.6
4510	8	32.1	11.3	1208	329.9
4511	29	34.3	10.3	1089	164.6
4512	29	34.9	10.3	1139	133.8
4513	91	34.0	12.5	1011	229.0
4514	91	33.1	12.5	999	243.9
4515	91	33.9	12.2	985	173.2

Appendix 16

Appendix 2

Individual Coagulation Values

5550008

Sex: Male

0<br>vg<br><br>Group 1	Day(s) Relative to Start Date	Reporting Coagulation<br>PT (sec)	APTT (sec)	FIB (mg/dL)	SAMQ Coagulation
1001	8	14.8	18.9	299	N
1002	8	16.2	18.4	256	N
1003	29	15.1	19.3	271	N
1004	29	15.2	18.3	282	N
1005	91	16.4	21.5	284	N
1006	91	16.7	20.1	115	N
1007	91	16.3	23.9	338	N
1008	29	14.3	18.4	301	N
1009	29	15.3	17.2	290	N
1010	29	16.0	20.0	282	N
1011	91	15.7	17.4	253	N
1012	91	15.8	19.3	292	N
1013	7 (Unsc)	16.2	19.5	462	N
1014	8	15.4	17.1	257	N
1015	8	15.2	17.1	267	N

Appendix 16

Appendix 2

Individual Coagulation Values

5550008

Sex: Male

0.36x 10E12 vg Group 2	Day(s) Relative to Start Date	Reporting Coagulation			
		PT (sec)	APTT (sec)	FIB (mg/dL)	SAMQ Coagulation
2001	29	15.6	18.7	260	N
2002	29	14.9	20.6	297	N
2103	91	15.5	21.5	305	N
2004	8	15.4	19.2	267	N
2005	91	14.8	16.2	277	N
2006	91	16.5	21.9	319	N
2007	91	CLOT	CLOT	CLOT	CLOT
2008	91	15.9	21.2	314	N
2009	8	14.9	20.2	290	N
2010	8	15.2	20.8	268	N
2011	29	15.5	20.3	294	N
2012	29	15.1	19.9	303	N
2013	29	13.9	17.2	294	N
2014	8	14.4	19.8	282	N
2015	8	13.5	15.7	289	N

Appendix 16

Appendix 2

Individual Coagulation Values

5550008

Sex: Male

1.1x 10E12 vg Group 3	Day(s) Relative to Start Date	Reporting Coagulation PT (sec)	APTT (sec)	FIB (mg/dL)	SAMQ Coagulation
3001	8	15.3	19.0	261	N
3002	8	15.7	18.2	281	N
3003	8	15.4	17.8	245	N
3004	29	14.8	20.4	252	N
3005	29	15.6	20.2	289	N
3006	91	16.1	21.3	312	N
3007	91	15.8	23.3	331	N
3008	91	14.9	19.7	323	N
3009	29	14.9	19.0	264	N
3010	29	15.4	15.6	374	N
3011	29	14.9	19.9	273	N
3012	8	15.1	17.5	273	N
3013	8	14.8	15.1	285	N
3014	91	15.7	21.3	297	N
3015	91	15.0	18.6	325	N

Appendix 16

Appendix 2

Individual Coagulation Values

5550008

Sex: Male

3.3x 10E12 vg Group 4	Day(s) Relative to Start Date	Reporting Coagulation PT (sec)	APTT (sec)	FIB (mg/dL)	SAMQ Coagulation
4001	8	15.8	19.1	279	N
4002	8	15.4	17.4	292	N
4003	29	15.2	19.8	266	N
4004	29	14.8	20.8	284	N
4005	29	CLOT	CLOT	CLOT	CLOT
4006	8	14.9	17.8	343	N
4007	8	15.2	19.2	392	N
4008	8	15.3	11.9	290	N
4009	91	14.7	18.2	358	N
4010	91	14.9	16.0	296	N
4011	91	15.0	17.8	312	N
4012	29	14.8	19.7	282	N
4013	29	15.2	20.7	259	N
4014	91	15.5	19.2	282	N
4015	91	16.1	18.6	297	N

Appendix 16

Appendix 2

Individual Coagulation Values

5550008

Sex: Female

0 vg Group 1	Day(s) Relative to Start Date	Reporting Coagulation PT (sec)	APTT (sec)	FIB (mg/dL)	SAMQ Coagulation
1501	8	15.6	18.3	232	N
1502	8	CLOT	CLOT	CLOT	CLOT
1503	91	15.0	19.0	187	N
1504	91	14.7	19.1	203	N
1505	8	15.3	18.1	246	N
1506	8	CLOT	CLOT	CLOT	CLOT
1507	8	14.2	14.5	231	N
1508	29	15.1	18.2	236	N
1509	29	14.8	17.8	228	N
1510	91	CLOT	CLOT	CLOT	CLOT
1511	91	15.2	19.3	198	N
1512	91	15.2	20.7	224	N
1513	29	CLOT	CLOT	CLOT	CLOT
1514	29	14.6	18.7	192	N
1515	29	13.8	15.7	207	N

Appendix 16

Appendix 2

Individual Coagulation Values

5550008

Sex: Female

0.36x 10E12 vg Group 2	Day(s) Relative to Start Date	Reporting Coagulation			
		PT (sec)	APTT (sec)	FIB (mg/dL)	SAMQ Coagulation
2501	29	15.2	19.3	201	N
2502	29	15.2	18.8	232	N
2503	91	14.7	20.3	216	N
2504	91	14.3	17.9	222	N
2505	8	15.1	18.0	232	N
2506	8	15.7	17.6	205	N
2507	29	14.4	21.5	205	N
2508	29	14.7	21.3	218	N
2509	29	15.5	18.7	226	N
2510	8	14.4	19.1	237	N
2511	8	14.3	19.8	191	N
2512	8	13.8	19.6	214	N
2613	91	15.2	19.8	188	N
2614	91	15.2	18.3	203	N
2615	91	14.6	20.4	218	N

Appendix 16

Appendix 2

Individual Coagulation Values

5550008

Sex: Female

1.1x 10E12 vg Group 3	Day(s) Relative to Start Date	Reporting Coagulation PT (sec)	APTT (sec)	FIB (mg/dL)	SAMQ Coagulation
3501	91	14.3	19.1	188	N
3502	91	15.0	20.3	203	N
3503	91	14.4	20.3	181	N
3504	29	14.5	19.8	210	N
3505	29	13.7	20.7	228	N
3506	29	14.7	17.6	222	N
3507	8	14.9	16.4	228	N
3508	8	14.4	19.2	228	N
3509	91	14.5	19.4	215	N
3510	91	14.9	19.5	189	N
3511	8	14.3	16.4	246	N
3512	8	14.6	15.7	231	N
3513	8	14.2	16.4	242	N
3514	29	14.7	19.2	210	N
3615	29	15.1	19.1	201	N

Appendix 16

Appendix 2

Individual Coagulation Values

5550008

Sex: Female

3.3x 10E12 vg Group 4	Day(s) Relative to Start Date	Reporting Coagulation			
		PT (sec)	APTT (sec)	FIB (mg/dL)	SAMQ Coagulation
4601	8	14.2	19.7	239	N
4502	8	15.1	15.3	202	N
4503	91	14.4	19.0	149	N
4504	91	15.0	9.7	197	N
4505	29	14.9	19.3	227	N
4506	29	15.5	19.3	227	N
4507	29	14.9	18.5	240	N
4508	8	13.8	17.9	234	N
4509	8	14.6	17.9	206	N
4510	8	14.4	18.3	248	N
4511	29	14.5	18.6	221	N
4512	29	13.6	15.9	219	N
4513	91	15.0	17.3	216	N
4514	91	14.6	20.5	222	N
4515	91	13.8	18.4	178	N

Appendix 16

Appendix 3

Individual Clinical Chemistry Values

5550008

Sex: Male

0 vg Group 1	Day(s) Relative to Start Date	Reporting Biochemistry AST (U/L)	ALT (U/L)	ALP (U/L)	GGT (U/L)	CK (U/L)	TBIL (mg/dL)
1001	8	103	26	279	2 [a]	654	0.00 [a]
1002	8	94	28	285	2 [a]	512	0.08
1003	29	92	31	152	2 [a]	466	0.04
1004	29	79	32	146	2 [a]	241	0.00 [a]
1005	91	78	30	80	2 [a]	529	0.08
1006	91	67	32	62	2 [a]	228	0.06
1007	91	76	22	58	2 [a]	224	0.03
1008	29	150	33	187	2 [a]	1022	0.06
1009	29	77	29	107	2 [a]	235	0.07
1010	29	86	39	209	2 [a]	302	0.09
1011	91	64	26	92	2 [a]	218	0.03
1012	91	81	31	56	2 [a]	403	0.05
1013	7 (Unsc)	QNS	11	178	QNS	QNS	QNS
1014	8	84	30	241	2 [a]	414	0.05
1015	8	85	33	273	2 [a]	236	0.00 [a]

[a] [RC:Assigned value below the reportable range]

Appendix 16

Appendix 3

Individual Clinical Chemistry Values

5550008

Sex: Male

0 vg Group 1	Day(s) Relative to Start Date	Reporting Biochemistry UREAN (mg/dL)	CREAT (mg/dL)	GLUC (mg/dL)	CHOL (mg/dL)	TRIG (mg/dL)	TPROT (g/dL)
1001	8	12	0.2	197	60	56	5.0
1002	8	9	0.3	222	74	29	4.8
1003	29	14	0.3	206	60	31	5.6
1004	29	16	0.4	239	64	30	5.9
1005	91	14	0.4	209	80	98	6.5
1006	91	16	0.4	222	66	26	6.0
1007	91	11	0.3	189	66	256	6.5
1008	29	12	0.3	126	55	29	5.7
1009	29	15	0.3	120	62	32	5.7
1010	29	17	0.4	213	58	26	5.7
1011	91	11	0.3	222	78	46	5.5
1012	91	14	0.4	177	83	79	5.9
1013	7 (Unsc)	QNS	QNS	QNS	QNS	QNS	QNS
1014	8	11	0.3	201	75	32	4.7
1015	8	14	0.3	175	80	45	4.9

Appendix 16

Appendix 3

Individual Clinical Chemistry Values

5550008

Sex: Male

0<br>vg<br>Group 1	Day(s) Relative to Start Date	Reporting Biochemistry<br>ALB (g/dL)	GLOB (g/dL)	A/G (ratio)	CA (mg/dL)	PHOS (mg/dL)	NA (mmol/L)
1001	8	3.6	1.4	2.6	9.6	11.0	138
1002	8	3.7	1.1	3.4	10.0	11.5	141
1003	29	4.0	1.6	2.5	9.6	8.5	140
1004	29	4.0	1.9	2.1	9.7	7.5	142
1005	91	4.1	2.4	1.7	9.8	6.7	140
1006	91	3.8	2.2	1.7	9.6	9.1	139
1007	91	3.6	2.9	1.2	10.2	6.7	141
1008	29	4.0	1.7	2.4	9.9	9.3	140
1009	29	3.9	1.8	2.2	9.9	9.5	145
1010	29	4.2	1.5	2.8	10.1	8.9	143
1011	91	3.8	1.7	2.2	9.3	6.4	142
1012	91	3.7	2.2	1.7	9.3	6.3	143
1013	7 (Unsc)	5.1	QNS	QNS	QNS	QNS	159
1014	8	3.6	1.1	3.3	9.2	10.4	145
1015	8	3.7	1.2	3.1	10.2	11.4	144

Appendix 16

Appendix 3

Individual Clinical Chemistry Values

5550008

Sex: Male

0<br>vg<br>Group 1	Day(s) Relative to Start Date	Reporting Biochemistry<br>K (mmol/L)	CL (mmol/L)	SAMQ
1001	8	4.8	98	N
1002	8	4.6	101	N
1003	29	4.6	101	N
1004	29	4.5	102	N
1005	91	4.9	100	N
1006	91	5.4	100	N
1007	91	5.0	102	N
1008	29	4.8	98	N
1009	29	5.1	106	N
1010	29	5.4	101	N
1011	91	5.0	105	N
1012	91	4.7	104	N
1013	7 (Unsc)	10.1 [a]	118	N
1014	8	4.2	104	N
1015	8	3.8	100	N

[a] [RC:Assigned value above the reportable range]

Appendix 16

Appendix 3

Individual Clinical Chemistry Values

5550008

Sex: Male

0.36x 10E12 vg Group 2	Day(s) Relative to Start Date	Reporting Biochemistry AST (U/L)	ALT (U/L)	ALP (U/L)	GGT (U/L)	CK (U/L)	TBIL (mg/dL)
2001	29	72	29	263	2 [b]	215	0.00 [b]
2002	29	98	31	219	2 [b]	743	0.00 [b]
2103	91	98	38	95	2 [b]	351	0.06
2004	8	105	28	304	2 [b]	481	0.09
2005	91	66	40	65	2 [b]	164	0.05
2006	91	96	32	110	2 [b]	673	0.05
2007	91	79	32	71	2 [b]	140	0.07
2008	91	98	25	73	2 [b]	548	0.08
2009	8	104	29	238	2 [b]	583	0.00 [b]
2010	8	83	31	220	2 [b]	277	0.03
2011	29	108	28	215	2 [b]	716	0.05
2012	29	101 E[a]	33 E[a]	164 E[a]	2 E[c]	451 E[a]	0.03 E[a]
2013	29	79	25	143	2 [b]	263	0.04
2014	8	102	29	180	2 [b]	701	0.05
2015	8	76	32	260	2 [b]	219	0.06

E = Exclude

[a] [FC:Sample analysed outside of established stability, results for information only]

[b] [RC:Assigned value below the reportable range]

[c] [RC:Assigned value below the reportable range, FC:Sample analysed outside of established stability, results for information only]

Appendix 16

Appendix 3

Individual Clinical Chemistry Values

5550008

Sex: Male

0.36x 10E12 vg Group 2	Day(s) Relative to Start Date	Reporting Biochemistry					
		UREAN (mg/dL)	CREAT (mg/dL)	GLUC (mg/dL)	CHOL (mg/dL)	TRIG (mg/dL)	TPROT (g/dL)
2001	29	13	0.3	263	59	31	5.5
2002	29	11	0.3	231	75	41	6.1
2103	91	13	0.3	167	118	108	6.4
2004	8	12	0.2	224	74	23	4.9
2005	91	11	0.3	217	85	88	6.0
2006	91	13	0.3	209	54	104	6.4
2007	91	14	0.3	285	75	83	5.9
2008	91	15	0.3	198	100	98	6.2
2009	8	11	0.3	246	71	21	5.4
2010	8	10	0.4	242	82	24	5.1
2011	29	23	0.4	230	50	33	5.4
2012	29	19 E[a]	0.4 E[a]	269 E[a]	58 E[a]	43 E[a]	5.4 E[a]
2013	29	12	0.3	207	71	26	5.4
2014	8	12	0.3	212	79	43	5.0
2015	8	25	0.3	205	73	9	5.3

E = Exclude

[a] [FC:Sample analysed outside of established stability, results for information only]

Appendix 16

Appendix 3

Individual Clinical Chemistry Values

5550008

Sex: Male

0.36x 10E12 vg Group 2	Day(s) Relative to Start Date	Reporting Biochemistry ALB (g/dL)	GLOB (g/dL)	A/G (ratio)	CA (mg/dL)	PHOS (mg/dL)	NA (mmol/L)
2001	29	3.7	1.8	2.1	9.7	9.6	140
2002	29	4.1	2.0	2.0	9.9	9.7	141
2103	91	4.1	2.3	1.8	10.0	8.1	141
2004	8	3.6	1.3	2.8	10.1	12.1	141
2005	91	3.8	2.2	1.7	10.2	7.2	140
2006	91	3.9	2.5	1.6	9.8	6.5	141
2007	91	3.7	2.2	1.7	10.1	7.4	138
2008	91	4.0	2.2	1.8	9.7	7.4	144
2009	8	3.9	1.5	2.6	9.6	8.4	139
2010	8	3.8	1.3	2.9	10.0	12.6	144
2011	29	3.9	1.5	2.6	9.5	8.0	142
2012	29	3.8 E[a]	1.6 E[a]	2.4 E[a]	UTD [b]	8.1 E[a]	140 E[a]
2013	29	3.8	1.6	2.4	9.8	8.9	144
2014	8	3.7	1.3	2.8	9.0	11.2	141
2015	8	3.8	1.5	2.5	9.1	8.5	144

E = Exclude

[a] [FC:Sample analysed outside of established stability, results for information only]

[b] [RC:EDTA cap was put by inadvertly on SST tube]

Appendix 16

Appendix 3

Individual Clinical Chemistry Values

5550008

Sex: Male

0.36x 10E12 vg Group 2	Day(s) Relative to Start Date	Reporting Biochemistry K (mmol/L)	CL (mmol/L)	SAMQ
2001	29	5.4	101	N
2002	29	5.1	100	N
2103	91	4.9	102	N
2004	8	3.8	97	N
2005	91	4.4	103	N
2006	91	4.8	104	N
2007	91	4.6	101	N
2008	91	4.6	107	N
2009	8	5.0	99	N
2010	8	5.5	101	N
2011	29	5.5	102	N
2012	29	5.3 E[a]	101 E[a]	N
2013	29	4.9	104	N
2014	8	4.9	100	N
2015	8	4.3	103	N

E = Exclude

[a] [FC:Sample analysed outside of established stability, results for information only]

Appendix 16

Appendix 3

Individual Clinical Chemistry Values

5550008

Sex: Male

1.1x 10E12 vg Group 3	Day(s) Relative to Start Date	Reporting Biochemistry AST (U/L)	ALT (U/L)	ALP (U/L)	GGT (U/L)	CK (U/L)	TBIL (mg/dL)
3001	8	89	40	212	2 [a]	376	0.00 [a]
3002	8	89	36	311	2 [a]	455	0.04
3003	8	99	31	273	2 [a]	341	0.04
3004	29	152	39	138	2 [a]	1296	0.08
3005	29	103	41	161	2 [a]	541	0.06
3006	91	77	37	82	2 [a]	188	0.03
3007	91	72	32	67	2 [a]	294	0.05
3008	91	131	60	65	2 [a]	515	0.07
3009	29	79	31	169	2 [a]	245	0.03
3010	29	93	37	219	2 [a]	228	0.05
3011	29	69	29	121	2 [a]	308	0.05
3012	8	99	38	348	2 [a]	470	0.05
3013	8	96	35	270	2 [a]	310	0.05
3014	91	73	32	94	2 [a]	202	0.06
3015	91	78	37	56	2 [a]	144	0.05

[a] [RC:Assigned value below the reportable range]

Appendix 16

Appendix 3

Individual Clinical Chemistry Values

5550008

Sex: Male

1.1x 10E12 vg Group 3	Day(s) Relative to Start Date	Reporting Biochemistry UREAN (mg/dL)	CREAT (mg/dL)	GLUC (mg/dL)	CHOL (mg/dL)	TRIG (mg/dL)	TPROT (g/dL)
3001	8	16	0.2	241	81	30	4.7
3002	8	9	0.3	285	61	25	4.6
3003	8	18	0.2	198	89	49	4.6
3004	29	15	0.4	159	53	26	5.8
3005	29	14	0.3	133	56	23	5.9
3006	91	14	0.4	183	91	64	6.4
3007	91	12	0.3	244	103	131	6.3
3008	91	11	0.4	207	65	52	6.2
3009	29	14	0.3	224	59	43	5.4
3010	29	16	0.3	222	62	27	5.1
3011	29	14	0.2	263	57	62	5.6
3012	8	19	0.3	243	76	36	5.0
3013	8	17	0.3	231	51	31	5.5
3014	91	13	0.3	254	77	59	6.3
3015	91	11	0.3	241	82	44	6.1

Appendix 16

Appendix 3

Individual Clinical Chemistry Values

5550008

Sex: Male

1.1x 10E12 vg Group 3	Day(s) Relative to Start Date	ALB (g/dL)	GLOB (g/dL)	A/G (ratio)	CA (mg/dL)	PHOS (mg/dL)	NA (mmol/L)
		Reporting Biochemistry					
3001	8	3.5	1.2	2.9	9.7	11.0	138
3002	8	3.4	1.2	2.8	9.7	10.3	137
3003	8	3.5	1.1	3.2	10.7	10.8	137
3004	29	4.0	1.8	2.2	9.5	9.4	147
3005	29	4.2	1.7	2.5	9.8	9.7	145
3006	91	3.9	2.5	1.6	10.0	7.8	139
3007	91	3.9	2.4	1.6	10.1	6.7	142
3008	91	3.9	2.3	1.7	9.8	6.5	139
3009	29	3.9	1.5	2.6	9.8	6.7	141
3010	29	3.4	1.7	2.0	9.3	8.4	140
3011	29	3.9	1.7	2.3	10.1	7.9	142
3012	8	3.8	1.2	3.2	9.2	9.3	142
3013	8	4.1	1.4	2.9	9.8	7.9	144
3014	91	3.8	2.5	1.5	9.3	8.7	138
3015	91	3.8	2.3	1.7	9.5	7.3	140

Appendix 16

Appendix 3

Individual Clinical Chemistry Values

5550008

Sex: Male

1.1x 10E12 vg Group 3	Day(s) Relative to Start Date	Reporting Biochemistry K (mmol/L)	Reporting Biochemistry CL (mmol/L)	Reporting Biochemistry SAMQ
3001	8	5.4	100	N
3002	8	5.2	98	N
3003	8	5.7	97	N
3004	29	4.4	103	N
3005	29	4.6	103	N
3006	91	4.3	104	N
3007	91	4.4	102	N
3008	91	5.1	103	N
3009	29	4.8	101	N
3010	29	4.6	101	N
3011	29	4.8	100	N
3012	8	4.4	100	N
3013	8	4.9	103	N
3014	91	4.8	100	N
3015	91	4.4	102	N

Appendix 16

Appendix 3

Individual Clinical Chemistry Values

5550008

Sex: Male

3.3x 10E12 vg Group 4	Day(s) Relative to Start Date	Reporting Biochemistry AST (U/L)	ALT (U/L)	ALP (U/L)	GGT (U/L)	CK (U/L)	TBIL (mg/dL)
4001	8	103	33	432	2 [a]	475	0.04
4002	8	106	35	391	2 [a]	441	0.04
4003	29	75	28	127	2 [a]	323	0.04
4004	29	71	40	176	2 [a]	245	0.04
4005	29	79	32	167	2 [a]	441	0.03
4006	8	100	34	303	2 [a]	446	0.05
4007	8	99	34	234	2 [a]	480	0.05
4008	8	104	47	258	2 [a]	573	0.05
4009	91	217	82	112	2 [a]	1048	0.12
4010	91	82	39	87	2 [a]	221	0.04
4011	91	58	25	73	2 [a]	171	0.07
4012	29	67	29	139	2 [a]	238	0.05
4013	29	66	21	166	2 [a]	142	0.05
4014	91	54	23	56	2 [a]	176	0.06
4015	91	105	41	62	2 [a]	393	0.07

[a] [RC:Assigned value below the reportable range]

Appendix 16

Appendix 3

Individual Clinical Chemistry Values

5550008

Sex: Male

3.3x 10E12 vg Group 4	Day(s) Relative to Start Date	UREAN (mg/dL)	CREAT (mg/dL)	GLUC (mg/dL)	CHOL (mg/dL)	TRIG (mg/dL)	TPROT (g/dL)
		Reporting Biochemistry					
4001	8	17	0.2	196	60	20	4.8
4002	8	13	0.3	255	59	30	4.9
4003	29	16	0.3	223	60	50	5.7
4004	29	14	0.3	290	79	53	6.2
4005	29	12	0.3	291	59	33	5.4
4006	8	12	0.3	187	85	33	5.2
4007	8	9	0.3	159	100	28	5.0
4008	8	9	0.2	133	81	26	5.0
4009	91	10	0.3	235	116	119	6.3
4010	91	14	0.3	220	55	33	5.8
4011	91	15	0.3	278	61	29	5.6
4012	29	16	0.3	200	68	64	5.7
4013	29	14	0.4	291	87	37	5.9
4014	91	13	0.3	277	68	39	6.1
4015	91	14	0.3	233	54	46	6.1

Appendix 16

Appendix 3

Individual Clinical Chemistry Values

5550008

Sex: Male

3.3x 10E12 vg Group 4	Day(s) Relative to Start Date	Reporting Biochemistry					
		ALB (g/dL)	GLOB (g/dL)	A/G (ratio)	CA (mg/dL)	PHOS (mg/dL)	NA (mmol/L)
4001	8	3.7	1.1	3.4	9.8	12.2	141
4002	8	3.7	1.2	3.1	10.4	13.1	139
4003	29	4.0	1.7	2.4	10.1	9.6	141
4004	29	4.1	2.1	2.0	10.2	8.3	140
4005	29	3.9	1.5	2.6	10.0	8.6	141
4006	8	3.9	1.3	3.0	9.9	9.5	139
4007	8	3.5	1.5	2.3	9.7	10.6	138
4008	8	3.9	1.1	3.5	10.2	10.5	140
4009	91	3.9	2.4	1.6	9.9	6.7	141
4010	91	3.9	1.9	2.1	9.5	6.9	141
4011	91	3.6	2.0	1.8	9.3	6.3	142
4012	29	3.8	1.9	2.0	9.5	6.6	147
4013	29	4.0	1.9	2.1	9.7	7.5	145
4014	91	3.6	2.5	1.4	9.6	6.8	138
4015	91	3.7	2.4	1.5	9.5	8.8	141

Appendix 16

Appendix 3

Individual Clinical Chemistry Values

5550008

Sex: Male

3.3x 10E12 vg Group 4	Day(s) Relative to Start Date	Reporting Biochemistry K (mmol/L)	CL (mmol/L)	SAMQ
4001	8	4.5	102	N
4002	8	5.5	99	N
4003	29	5.4	101	N
4004	29	5.1	99	N
4005	29	5.2	102	N
4006	8	4.0	99	N
4007	8	4.8	100	N
4008	8	5.0	100	N
4009	91	5.2	104	N
4010	91	4.9	103	N
4011	91	4.6	102	N
4012	29	4.4	103	N
4013	29	5.3	104	N
4014	91	4.5	101	N
4015	91	4.3	101	N

Appendix 16

Appendix 3

Individual Clinical Chemistry Values

5550008

Sex: Female

0 vg Group 1	Day(s) Relative to Start Date	AST (U/L)	ALT (U/L)	ALP (U/L)	GGT (U/L)	CK (U/L)	TBIL (mg/dL)
1501	8	73	22	164	2 [a]	202	0.03
1502	8	81	29	122	2 [a]	303	0.04
1503	91	121	29	51	2 [a]	659	0.05
1504	91	137	43	28	2 [a]	216	0.10
1505	8	123	22	180	2 [a]	949	0.04
1506	8	68	19	169	2 [a]	278	0.03
1507	8	103	26	136	2 [a]	309	0.00 [a]
1508	29	75	27	79	2 [a]	153	0.04
1509	29	67	23	99	2 [a]	224	0.10
1510	91	160	77	50	2 [a]	252	0.08
1511	91	111	35	37	2 [a]	136	0.08
1512	91	64	28	27	2 [a]	144	0.06
1513	29	68	22	87	2 [a]	172	0.09
1514	29	81	24	104	2 [a]	313	0.09
1515	29	67	18	81	2 [a]	155	0.07

[a] [RC:Assigned value below the reportable range]

Appendix 16

Appendix 3

Individual Clinical Chemistry Values

5550008

Sex: Female

0 vg Group 1	Day(s) Relative to Start Date	UREAN (mg/dL)	CREAT (mg/dL)	GLUC (mg/dL)	CHOL (mg/dL)	TRIG (mg/dL)	TPROT (g/dL)
		Reporting Biochemistry					
1501	8	15	0.3	171	52	16	5.4
1502	8	13	0.2	163	80	26	5.5
1503	91	18	0.5	124	81	26	7.0
1504	91	17	0.5	205	74	57	7.1
1505	8	16	0.3	162	61	20	5.6
1506	8	16	0.3	117	31	17	5.2
1507	8	25	0.3	191	56	19	5.6
1508	29	13	0.3	175	56	21	5.6
1509	29	16	0.3	225	72	31	6.0
1510	91	19	0.4	172	66	32	6.0
1511	91	17	0.4	268	68	33	6.6
1512	91	24	0.5	178	61	24	7.3
1513	29	16	0.4	143	56	30	5.8
1514	29	14	0.4	157	81	28	6.3
1515	29	17	0.4	171	92	26	6.7

Appendix 16

Appendix 3

Individual Clinical Chemistry Values

5550008

Sex: Female

0 vg Group 1	Day(s) Relative to Start Date	Reporting Biochemistry ALB (g/dL)	GLOB (g/dL)	A/G (ratio)	CA (mg/dL)	PHOS (mg/dL)	NA (mmol/L)
1501	8	4.0	1.4	2.9	9.4	6.0	139
1502	8	4.0	1.5	2.7	10.6	8.8	140
1503	91	4.9	2.1	2.3	9.5	7.3	135
1504	91	5.2	1.9	2.7	9.9	6.1	138
1505	8	3.9	1.7	2.3	9.7	7.8	138
1506	8	4.1	1.1	3.7	9.4	7.9	139
1507	8	4.3	1.3	3.3	9.8	7.2	138
1508	29	4.3	1.3	3.3	10.4	8.0	140
1509	29	4.6	1.4	3.3	10.8	8.3	140
1510	91	4.2	1.8	2.3	9.8	6.9	139
1511	91	4.6	2.0	2.3	10.2	6.2	141
1512	91	5.5	1.8	3.1	10.6	7.2	142
1513	29	4.3	1.5	2.9	9.9	6.2	148
1514	29	4.5	1.8	2.5	10.2	7.2	147
1515	29	5.1	1.6	3.2	10.1	9.5	150

Appendix 16

Appendix 3

Individual Clinical Chemistry Values

5550008

Sex: Female

0 vg Group 1	Day(s) Relative to Start Date	Reporting Biochemistry K (mmol/L)	CL (mmol/L)	SAMQ
1501	8	4.0	104	N
1502	8	4.1	100	N
1503	91	3.5	100	N
1504	91	3.7	103	N
1505	8	4.0	101	N
1506	8	3.6	102	N
1507	8	4.7	97	N
1508	29	4.2	102	N
1509	29	4.0	101	N
1510	91	4.8	106	H+
1511	91	3.9	103	N
1512	91	4.2	104	N
1513	29	4.6	108	N
1514	29	4.0	107	N
1515	29	3.5	107	N

Appendix 16

Appendix 3

Individual Clinical Chemistry Values

5550008

Sex: Female

0.36x 10E12 vg Group 2	Day(s) Relative to Start Date	AST (U/L)	ALT (U/L)	ALP (U/L)	GGT (U/L)	CK (U/L)	TBIL (mg/dL)
2501	29	85	38	78	2 [a]	256	0.08
2502	29	67	22	79	2 [a]	159	0.04
2503	91	130	40	49	2 [a]	306	0.08
2504	91	100	28	29	2 [a]	175	0.07
2505	8	112	35	115	2 [a]	593	0.00 [a]
2506	8	70	26	152	2 [a]	243	0.00 [a]
2507	29	98	28	79	2 [a]	712	0.07
2508	29	80	19	71	2 [a]	358	0.09
2509	29	66	22	121	2 [a]	134	0.06
2510	8	129	39	146	2 [a]	783	0.04
2511	8	86	32	131	2 [a]	398	0.04
2512	8	66	34	170	2 [a]	224	0.03
2613	91	96	33	34	2 [a]	342	0.05
2614	91	80	31	32	2 [a]	193	0.11
2615	91	87	27	27	2 [a]	399	0.13

[a] [RC:Assigned value below the reportable range]

Appendix 16

Appendix 3

Individual Clinical Chemistry Values

5550008

Sex: Female

0.36x 10E12 vg Group 2	Day(s) Relative to Start Date	UREAN (mg/dL)	CREAT (mg/dL)	GLUC (mg/dL)	CHOL (mg/dL)	TRIG (mg/dL)	TPROT (g/dL)
		Reporting Biochemistry					
2501	29	14	0.3	168	52	21	6.0
2502	29	13	0.4	167	41	17	6.1
2503	91	15	0.3	187	105	35	7.3
2504	91	15	0.4	209	115	65	7.7
2505	8	24	0.4	156	50	10	5.3
2506	8	23	0.4	176	57	17	5.3
2507	29	17	0.6	145	77	38	6.6
2508	29	16	0.4	150	76	27	6.5
2509	29	18	0.4	156	51	20	6.3
2510	8	16	0.3	184	63	16	5.4
2511	8	17	0.3	205	51	20	5.5
2512	8	16	0.3	282	62	24	5.4
2613	91	17	0.6	125	82	35	7.2
2614	91	17	0.5	143	70	31	6.8
2615	91	14	0.4	194	63	30	7.6

Appendix 16

Appendix 3

Individual Clinical Chemistry Values

5550008

Sex: Female

0.36x 10E12 vg Group 2	Day(s) Relative to Start Date	Reporting Biochemistry ALB (g/dL)	GLOB (g/dL)	A/G (ratio)	CA (mg/dL)	PHOS (mg/dL)	NA (mmol/L)
2501	29	4.3	1.7	2.5	10.1	7.5	137
2502	29	4.5	1.6	2.8	10.5	7.6	143
2503	91	5.5	1.8	3.1	10.3	7.3	138
2504	91	5.6	2.1	2.7	11.1	8.8	139
2505	8	4.0	1.3	3.1	9.3	6.7	136
2506	8	3.8	1.5	2.5	9.5	9.5	138
2507	29	4.7	1.9	2.5	10.5	7.9	144
2508	29	4.8	1.7	2.8	10.3	8.3	145
2509	29	4.6	1.7	2.7	10.0	6.5	144
2510	8	4.0	1.4	2.9	10.0	9.3	140
2511	8	4.2	1.3	3.2	10.3	8.4	143
2512	8	4.0	1.4	2.9	10.2	7.8	140
2613	91	5.2	2.0	2.6	9.7	6.5	143
2614	91	4.8	2.0	2.4	10.0	6.6	141
2615	91	5.3	2.3	2.3	10.3	6.4	139

Appendix 16

Appendix 3

Individual Clinical Chemistry Values

5550008

Sex: Female

0.36x 10E12 vg Group 2	Day(s) Relative to Start Date	Reporting Biochemistry K (mmol/L)	CL (mmol/L)	SAMQ
2501	29	4.9	102	N
2502	29	3.7	104	N
2503	91	4.2	102	N
2504	91	5.5	101	N
2505	8	4.6	99	N
2506	8	4.6	102	N
2507	29	3.9	102	N
2508	29	3.7	103	N
2509	29	4.0	103	N
2510	8	4.7	99	N
2511	8	4.1	101	N
2512	8	4.3	98	N
2613	91	3.5	102	N
2614	91	3.3	103	N
2615	91	4.1	101	N

Appendix 16

Appendix 3

Individual Clinical Chemistry Values

5550008

Sex: Female

1.1x 10E12 vg Group 3	Day(s) Relative to Start Date	AST (U/L)	ALT (U/L)	ALP (U/L)	GGT (U/L)	CK (U/L)	TBIL (mg/dL)
		Reporting Biochemistry					
3501	91	95	36	39	2 [a]	449	0.08
3502	91	62	22	25	2 [a]	268	0.09
3503	91	97	31	41	2 [a]	403	0.09
3504	29	71	23	111	2 [a]	139	0.06
3505	29	71	21	114	2 [a]	204	0.06
3506	29	79	26	112	2 [a]	252	0.05
3507	8	94	34	112	2 [a]	416	0.04
3508	8	66	24	131	2 [a]	177	0.00 [a]
3509	91	139	49	33	2 [a]	841	0.12
3510	91	99	54	30	2 [a]	263	0.07
3511	8	81	29	98	2 [a]	243	0.06
3512	8	115	35	146	2 [a]	283	0.04
3513	8	77	41	105	2 [a]	208	0.07
3514	29	108	29	83	2 [a]	211	0.07
3615	29	70	20	95	2 [a]	137	0.04

[a] [RC:Assigned value below the reportable range]

Appendix 16

Appendix 3

Individual Clinical Chemistry Values

5550008

Sex: Female

1.1x 10E12 vg Group 3	Day(s) Relative to Start Date	Reporting Biochemistry					
		UREAN (mg/dL)	CREAT (mg/dL)	GLUC (mg/dL)	CHOL (mg/dL)	TRIG (mg/dL)	TPROT (g/dL)
3501	91	15	0.4	132	79	19	7.2
3502	91	12	0.5	185	53	50	7.1
3503	91	13	0.5	127	108	37	7.4
3504	29	16	0.3	203	76	33	6.2
3505	29	18	0.4	175	64	19	6.2
3506	29	20	0.4	226	60	31	5.8
3507	8	20	0.2	179	34	15	5.3
3508	8	21	0.3	178	59	17	5.2
3509	91	20	0.5	171	97	50	7.7
3510	91	13	0.3	249	70	33	6.0
3511	8	21	0.3	161	44	17	6.1
3512	8	24	0.2	160	52	20	5.6
3513	8	21	0.3	171	71	25	6.0
3514	29	16	0.4	136	65	21	6.6
3615	29	15	0.4	187	64	26	5.9

Appendix 16

Appendix 3

Individual Clinical Chemistry Values

5550008

Sex: Female

1.1x 10E12 vg Group 3	Day(s) Relative to Start Date	Reporting Biochemistry ALB (g/dL)	GLOB (g/dL)	A/G (ratio)	CA (mg/dL)	PHOS (mg/dL)	NA (mmol/L)
3501	91	5.4	1.8	3.0	10.1	6.5	139
3502	91	5.1	2.0	2.5	10.4	5.1	136
3503	91	5.4	2.0	2.7	10.4	5.3	138
3504	29	4.5	1.7	2.6	9.9	6.0	142
3505	29	4.5	1.7	2.6	10.2	7.9	142
3506	29	4.2	1.6	2.6	9.7	7.3	143
3507	8	4.0	1.3	3.1	9.7	8.4	140
3508	8	4.1	1.1	3.7	9.5	6.5	141
3509	91	5.6	2.1	2.7	10.8	6.6	142
3510	91	4.4	1.6	2.8	10.0	7.9	141
3511	8	4.5	1.6	2.8	10.0	8.9	144
3512	8	4.0	1.6	2.5	10.0	9.0	146
3513	8	4.6	1.4	3.3	10.3	7.9	141
3514	29	4.9	1.7	2.9	10.1	9.7	146
3615	29	4.3	1.6	2.7	10.4	9.6	149

Appendix 16

Appendix 3

Individual Clinical Chemistry Values

5550008

Sex: Female

1.1x 10E12 vg Group 3	Day(s) Relative to Start Date	Reporting Biochemistry		
		K (mmol/L)	CL (mmol/L)	SAMQ
3501	91	3.7	103	N
3502	91	4.2	101	N
3503	91	4.0	101	N
3504	29	3.9	102	N
3505	29	4.1	104	N
3506	29	3.8	103	N
3507	8	4.4	103	N
3508	8	3.8	103	N
3509	91	4.3	104	N
3510	91	4.2	104	N
3511	8	4.1	105	N
3512	8	3.8	106	N
3513	8	4.0	102	N
3514	29	4.1	104	N
3615	29	3.5	107	N

Appendix 16

Appendix 3

Individual Clinical Chemistry Values

5550008

Sex: Female

3.3x 10E12 vg Group 4	Day(s) Relative to Start Date	Reporting Biochemistry AST (U/L)	ALT (U/L)	ALP (U/L)	GGT (U/L)	CK (U/L)	TBIL (mg/dL)
4601	8	105	35	166	2 [a]	370	0.00 [a]
4502	8	75	32	190	2 [a]	381	0.03
4503	91	72	19	43	2 [a]	298	0.09
4504	91	78	21	37	2 [a]	211	0.06
4505	29	62	27	81	2 [a]	146	0.03
4506	29	73	22	102	2 [a]	185	0.07
4507	29	74	25	68	2 [a]	227	0.05
4508	8	87	41	112	2 [a]	190	0.00 [a]
4509	8	81	31	126	2 [a]	206	0.05
4510	8	90	45	126	2 [a]	318	0.04
4511	29	75	29	118	2 [a]	158	0.09
4512	29	66	28	49	2 [a]	177	0.10
4513	91	85	26	34	2 [a]	182	0.07
4514	91	85	35	27	2 [a]	262	0.08
4515	91	130	48	30	2 [a]	146	0.09

[a] [RC:Assigned value below the reportable range]

Appendix 16

Appendix 3

Individual Clinical Chemistry Values

5550008

Sex: Female

3.3x 10E12 vg Group 4	Day(s) Relative to Start Date	UREAN (mg/dL)	CREAT (mg/dL)	GLUC (mg/dL)	CHOL (mg/dL)	TRIG (mg/dL)	TPROT (g/dL)
		Reporting Biochemistry					
4601	8	25	0.3	212	53	27	5.7
4502	8	17	0.3	217	61	11	4.9
4503	91	11	0.5	162	66	45	6.9
4504	91	11	0.4	184	71	24	6.6
4505	29	27	0.3	221	67	20	5.9
4506	29	20	0.4	242	61	28	5.9
4507	29	22	0.3	205	65	18	6.1
4508	8	21	0.3	186	46	17	5.6
4509	8	21	0.3	196	52	14	5.2
4510	8	21	0.3	192	39	14	5.4
4511	29	19	0.4	156	75	27	6.1
4512	29	20	0.4	145	71	27	7.1
4513	91	14	0.4	194	58	19	6.7
4514	91	17	0.4	212	70	74	6.9
4515	91	21	0.4	231	97	31	8.2

Appendix 16

Appendix 3

Individual Clinical Chemistry Values

5550008

Sex: Female

3.3x 10E12 vg Group 4	Day(s) Relative to Start Date	ALB (g/dL)	GLOB (g/dL)	A/G (ratio)	CA (mg/dL)	PHOS (mg/dL)	NA (mmol/L)
		Reporting Biochemistry					
4601	8	4.4	1.3	3.4	9.7	7.0	140
4502	8	3.8	1.1	3.5	10.6	10.4	141
4503	91	5.1	1.8	2.8	9.9	5.8	139
4504	91	4.7	1.9	2.5	10.0	6.1	138
4505	29	4.2	1.7	2.5	10.0	8.6	140
4506	29	4.2	1.7	2.5	9.6	7.5	143
4507	29	4.4	1.7	2.6	9.9	7.8	141
4508	8	4.5	1.1	4.1	10.1	8.1	141
4509	8	3.9	1.3	3.0	10.3	10.1	141
4510	8	4.1	1.3	3.2	9.6	7.4	143
4511	29	4.3	1.8	2.4	10.3	8.8	148
4512	29	5.1	2.0	2.5	11.1	9.5	146
4513	91	4.7	2.0	2.3	10.0	5.9	140
4514	91	4.9	2.0	2.5	10.0	7.5	138
4515	91	6.2	2.0	3.1	10.4	6.9	141

Appendix 16

Appendix 3

Individual Clinical Chemistry Values

5550008

Sex: Female

3.3x 10E12 vg Group 4	Day(s) Relative to Start Date	Reporting Biochemistry K (mmol/L)	CL (mmol/L)	SAMQ
4601	8	4.2	101	N
4502	8	4.8	101	N
4503	91	3.4	100	N
4504	91	4.0	102	N
4505	29	5.0	100	N
4506	29	4.8	104	N
4507	29	4.0	100	N
4508	8	4.0	100	N
4509	8	4.2	101	N
4510	8	3.7	104	N
4511	29	3.5	107	N
4512	29	4.2	104	N
4513	91	4.2	102	N
4514	91	4.1	101	N
4515	91	3.9	99	N

Appendix 16

Appendix 4

Individual Urinalysis Values

5550008

Sex: Male

0 vg Group 1	Day(s) Relative to Start Date	VOLUME (mL)	COLOR	CLARITY	GLUC	BIL	KET
		Reporting Urinalysis					
1001	8	2.5	DY	Cld	Neg	Neg	2+
1002	8	13.5	LY	Cld	Neg	Neg	1+
1003	29	48.5	LY	Clr	Neg	Neg	Neg
1004	29	21.8	LY	Cld	Neg	Neg	1+
1005	91	15.0	LY	Clr	Neg	Neg	2+
1006	91	7.0	LY	Clr	Neg	Neg	2+
1007	91	4.2	LY	Cld	Neg	Neg	2+
1008	29	21.8	LY	Clr	Neg	Neg	1+
1009	29	26.1	LY	Clr	Neg	Neg	1+
1010	29	30.0	LY	Clr	Neg	Neg	1+
1011	91	2.2	DY	Tur	1+	1+	2+
1012	91	2.3	DY	Cld	Neg	1+	2+
1014	8	22.9	LY	Clr	Neg	Neg	Neg
1015	8	37.0	LY	Clr	Neg	Neg	Neg

Appendix 16

Appendix 4

Individual Urinalysis Values

5550008

Sex: Male

0<br>vg<br>Group 1	Day(s) Relative to Start Date	Reporting Urinalysis<br>SPECIFIC GRAVITY	BLD	URINE pH	PROT
1001	8	1.039	Neg	7.0	2+
1002	8	1.012	Neg	8.0	1+
1003	29	1.006	Neg	7.5	Neg
1004	29	1.012	Neg	7.5	1+
1005	91	1.016	Neg	7.5	1+
1006	91	1.033	Neg	7.0	2+
1007	91	1.041	Neg	8.5	3+
1008	29	1.011	Neg	7.5	1+
1009	29	1.010	Neg	7.5	Neg
1010	29	1.006	Neg	7.5	Neg
1011	91	1.074	Neg	6.0	3+
1012	91	1.068	Neg	6.5	3+
1014	8	1.013	Neg	6.0	Neg
1015	8	1.007	Neg	6.0	Neg

Appendix 16

Appendix 4

Individual Urinalysis Values

5550008

Sex: Male

0.36x 10E12 vg Group 2	Day(s) Relative to Start Date	Reporting Urinalysis VOLUME (mL)	COLOR	CLARITY	GLUC	BIL	KET
2001	29	15.4	LY	Cld	Neg	Neg	1+
2002	29	5.2	DY	Cld	Neg	Neg	2+
2103	91	8.9	LY	Cld	Neg	Neg	1+
2004	8	7.5	LY	Cld	Neg	Neg	Neg
2005	91	6.0	LY	Cld	Neg	Neg	2+
2006	91	12.0	LY	Cld	Neg	Neg	2+
2007	91	13.0	LY	Clr	Neg	Neg	2+
2008	91	11.2	LY	Cld	Neg	Neg	1+
2009	8	9.3	LY	Clr	Neg	Neg	1+
2010	8	12.5	LY	Clr	Neg	Neg	2+
2011	29	3.7	LY	Cld	Neg	Neg	2+
2012	29	3.6	DY	Clr	Neg	Neg	2+
2013	29	16.0	LY	Cld	Neg	Neg	1+
2014	8	4.7	LY	Cld	Neg	Neg	2+
2015	8	8.2	LY	Cld	Neg	Neg	1+

Appendix 16

Appendix 4

Individual Urinalysis Values

5550008

Sex: Male

0.36x 10E12 vg Group 2	Day(s) Relative to Start Date	Reporting Urinalysis SPECIFIC GRAVITY	BLD	URINE pH	PROT
2001	29	1.019	Neg	7.0	2+
2002	29	1.045	Neg	6.5	2+
2103	91	1.027	Neg	7.5	2+
2004	8	1.015	Neg	6.5	Neg
2005	91	1.040	1+	7.5	2+
2006	91	1.022	Neg	7.5	2+
2007	91	1.023	Neg	7.5	1+
2008	91	1.019	Neg	8.0	2+
2009	8	1.021	Neg	6.5	2+
2010	8	1.016	Neg	6.5	1+
2011	29	1.037	Neg	7.0	2+
2012	29	1.048	Neg	7.0	3+
2013	29	1.015	4+	6.5	2+
2014	8	1.032	Neg	7.0	2+
2015	8	1.015	Neg	7.0	Neg

Appendix 16
Appendix 4

Individual Urinalysis Values

5550008
Sex: Male

1.1x 10E12 vg Group 3	Day(s) Relative to Start Date	Reporting Urinalysis					
		VOLUME (mL)	COLOR	CLARITY	GLUC	BIL	KET
3001	8	13.3	LY	Clr	Neg	Neg	Neg
3002	8	33.6	LY	Cld	Neg	Neg	Neg
3003	8	8.5	LY	Clr	Neg	Neg	2+
3004	29	16.0	LY	Clr	Neg	Neg	1+
3005	29	14.0	LY	Clr	Neg	Neg	2+
3006	91	3.0	DY	Tur	Neg	1+	2+
3007	91	13.0	LY	Clr	Neg	Neg	2+
3008	91	3.0	DY	Cld	Neg	Neg	2+
3009	29	3.4	DY	Cld	Neg	Neg	2+
3010	29	2.3	DY	Cld	Neg	Neg	2+
3011	29	10.6	LY	Clr	Neg	Neg	2+
3012	8	5.2	LY	Tur	Neg	Neg	2+
3013	8	56.3	LY	Clr	Neg	Neg	Neg
3014	91	10.4	LY	Cld	Neg	Neg	1+
3015	91	28.7	LY	Cld	Neg	Neg	1+

Appendix 16

Appendix 4

Individual Urinalysis Values

5550008

Sex: Male

1.1x 10E12 vg Group 3	Day(s) Relative to Start Date	Reporting Urinalysis			
		SPECIFIC GRAVITY	BLD	URINE pH	PROT
3001	8	1.011	Neg	7.0	Neg
3002	8	1.007	Neg	6.5	Neg
3003	8	1.017	Neg	6.5	1+
3004	29	1.016	Neg	7.5	1+
3005	29	1.016	Neg	7.0	2+
3006	91	1.071	Neg	7.0	3+
3007	91	1.021	Neg	7.5	2+
3008	91	1.046	Neg	7.0	2+
3009	29	1.039	Neg	7.0	2+
3010	29	1.049	Neg	6.5	2+
3011	29	1.016	Neg	7.0	1+
3012	8	1.025	Neg	6.5	2+
3013	8	1.006	Neg	7.0	Neg
3014	91	1.017	Neg	7.5	1+
3015	91	1.013	Neg	8.0	1+

Appendix 16

Appendix 4

Individual Urinalysis Values

5550008

Sex: Male

3.3x 10E12 vg Group 4	Day(s) Relative to Start Date	Reporting Urinalysis VOLUME (mL)	COLOR	CLARITY	GLUC	BIL	KET
4001	8	3.1	DY	Cld	Neg	Neg	2+
4002	8	2.1	DY	Clr	Neg	Neg	2+
4003	29	5.3	DY	Cld	Neg	Neg	2+
4004	29	12.1	LY	Cld	Neg	Neg	2+
4005	29	6.9	LY	Cld	Neg	Neg	1+
4006	8	8.0	LY	Clr	Neg	Neg	1+
4007	8	4.0	LY	Cld	Neg	Neg	1+
4008	8	22.0	LY	Clr	Neg	Neg	Neg
4009	91	9.6	LY	Clr	Neg	Neg	2+
4010	91	22.1	LY	Cld	Neg	Neg	1+
4011	91	4.8	DY	Cld	Neg	Neg	1+
4012	29	18.0	LY	Clr	Neg	Neg	1+
4013	29	25.0	LY	Clr	Neg	Neg	1+
4014	91	2.8	DY	Cld	Neg	Neg	2+
4015	91	5.6	DY	Cld	Neg	Neg	3+

Appendix 16

Appendix 4

Individual Urinalysis Values

5550008

Sex: Male

3.3x 10E12 vg Group 4	Day(s) Relative to Start Date	Reporting Urinalysis SPECIFIC GRAVITY	BLD	URINE pH	PROT
4001	8	1.036	Neg	7.5	2+
4002	8	1.034	Neg	7.0	2+
4003	29	1.043	Neg	7.0	2+
4004	29	1.029	Neg	7.0	2+
4005	29	1.035	Neg	7.0	2+
4006	8	1.018	Neg	7.0	1+
4007	8	1.023	Neg	7.0	1+
4008	8	1.011	Neg	7.5	Neg
4009	91	1.029	Neg	7.5	2+
4010	91	1.012	Neg	8.0	1+
4011	91	1.054	3+	6.5	3+
4012	29	1.015	1+	7.0	1+
4013	29	1.013	4+	6.5	2+
4014	91	1.061	Neg	7.0	3+
4015	91	1.048	Neg	7.0	2+

Appendix 16

Appendix 4

Individual Urinalysis Values

5550008

Sex: Female

0 vg Group 1	Day(s) Relative to Start Date	Reporting Urinalysis VOLUME (mL)	COLOR	CLARITY	GLUC	BIL	KET
1501	8	9.7	LY	Clr	Neg	Neg	Neg
1502	8	6.4	LY	Clr	Neg	Neg	Neg
1503	91	0.2	QNS	QNS	QNS	QNS	QNS
1504	91	1.4	DY	Cld	Neg	Neg	Neg
1505	8	6.0	LY	Clr	Neg	Neg	Neg
1506	8	4.0	DY	Cld	Neg	Neg	Neg
1507	8	5.0	DY	Clr	Neg	Neg	Neg
1508	29	2.5	LY	Clr	Neg	Neg	1+
1509	29	2.1	LY	Clr	Neg	Neg	Neg
1510	91	1.4	DY	Clr	Neg	1+	1+
1511	91	15.3	LY	Clr	Neg	Neg	Neg
1512	91	2.0	DY	Clr	1+	1+	1+
1513	29	8.4	LY	Clr	Neg	Neg	Neg
1514	29	6.8	LY	Clr	Neg	Neg	Neg
1515	29	13.0	LY	Clr	Neg	Neg	Neg

Appendix 16

Appendix 4

Individual Urinalysis Values

5550008

Sex: Female

0<br>vg<br>Group 1	Day(s) Relative to Start Date	Reporting Urinalysis<br>SPECIFIC GRAVITY	BLD	URINE pH	PROT
1501	8	1.013	Neg	7.0	Neg
1502	8	1.019	Neg	7.5	1+
1503	91	QNS	QNS	QNS	QNS
1504	91	1.046	Neg	6.0	2+
1505	8	1.025	Neg	6.5	Neg
1506	8	1.044	Neg	6.5	2+
1507	8	1.039	Neg	6.5	1+
1508	29	1.036	Neg	6.5	1+
1509	29	1.035	Neg	7.0	2+
1510	91	1.059	Neg	6.5	2+
1511	91	1.011	Neg	7.0	Neg
1512	91	1.063	Neg	6.5	2+
1513	29	1.018	Neg	7.0	Neg
1514	29	1.019	Neg	6.5	Neg
1515	29	1.012	Neg	7.0	Neg

Appendix 16

Appendix 4

Individual Urinalysis Values

5550008

Sex: Female

0.36x 10E12 vg Group 2	Day(s) Relative to Start Date	Reporting Urinalysis VOLUME (mL)	COLOR	CLARITY	GLUC	BIL	KET
2501	29	9.8	LY	Cld	Neg	Neg	Neg
2502	29	11.3	LY	Clr	Neg	Neg	Neg
2503	91	5.0	LY	Clr	Neg	Neg	Neg
2504	91	3.0	DY	Clr	Neg	Neg	Neg
2505	8	5.0	LY	Clr	Neg	Neg	Neg
2506	8	12.0	LY	Clr	Neg	Neg	Neg
2507	29	18.0	LY	Clr	Neg	Neg	Neg
2508	29	2.0	LY	Clr	Neg	Neg	1+
2509	29	4.0	DY	Cld	Neg	Neg	1+
2510	8	22.1	LY	Clr	Neg	Neg	Neg
2511	8	10.0	LY	Clr	Neg	Neg	Neg
2512	8	10.3	LY	Clr	Neg	Neg	Neg
2613	91	5.8	LY	Clr	Neg	Neg	Neg
2614	91	4.9	LY	Clr	Neg	Neg	Neg
2615	91	34.5	LY	Clr	Neg	Neg	Neg

Appendix 16

Appendix 4

Individual Urinalysis Values

5550008

Sex: Female

0.36x 10E12 vg Group 2	Day(s) Relative to Start Date	Reporting Urinalysis SPECIFIC GRAVITY	BLD	URINE pH	PROT
2501	29	1.014	Neg	6.0	Neg
2502	29	1.017	Neg	7.0	Neg
2503	91	1.028	Neg	6.5	1+
2504	91	1.043	Neg	6.0	2+
2505	8	1.026	Neg	6.5	Neg
2506	8	1.012	Neg	6.5	Neg
2507	29	1.009	Neg	7.0	Neg
2508	29	1.040	Neg	6.5	2+
2509	29	1.044	Neg	6.5	2+
2510	8	1.012	Neg	7.0	Neg
2511	8	1.017	Neg	7.0	Neg
2512	8	1.017	Neg	6.0	Neg
2613	91	1.019	Neg	6.5	Neg
2614	91	1.021	Neg	6.5	Neg
2615	91	1.007	Neg	7.0	Neg

Appendix 16

Appendix 4

Individual Urinalysis Values

5550008

Sex: Female

1.1x 10E12 vg Group 3	Day(s) Relative to Start Date	Reporting Urinalysis					
		VOLUME (mL)	COLOR	CLARITY	GLUC	BIL	KET
3501	91	7.0	LY	Clr	Neg	Neg	Neg
3502	91	10.0	LY	Clr	Neg	Neg	Neg
3503	91	20.0	LY	Clr	Neg	Neg	Neg
3504	29	5.0	DY	Cld	Neg	Neg	1+
3505	29	3.0	DY	Cld	Neg	Neg	1+
3506	29	3.0	LY	Cld	Neg	Neg	1+
3507	8	7.4	LY	Clr	Neg	Neg	Neg
3508	8	5.2	LY	Cld	Neg	Neg	Neg
3509	91	3.8	LY	Clr	Neg	Neg	1+
3510	91	3.5	LY	Clr	Neg	Neg	Neg
3511	8	7.5	LY	Clr	Neg	Neg	Neg
3512	8	3.9	LY	Cld	Neg	Neg	Neg
3513	8	11.7	LY	Clr	Neg	Neg	Neg
3514	29	14.0	LY	Clr	Neg	Neg	Neg
3615	29	21.0	LY	Clr	Neg	Neg	Neg

Appendix 16

Appendix 4

Individual Urinalysis Values

5550008

Sex: Female

1.1x 10E12 vg Group 3	Day(s) Relative to Start Date	Reporting Urinalysis SPECIFIC GRAVITY	BLD	URINE pH	PROT
3501	91	1.017	Neg	6.5	Neg
3502	91	1.018	Neg	6.0	Neg
3503	91	1.007	Neg	7.0	Neg
3504	29	1.032	Neg	6.5	1+
3505	29	1.038	2+	7.0	2+
3506	29	1.023	Neg	6.5	1+
3507	8	1.019	Neg	6.5	Neg
3508	8	1.030	Neg	7.0	2+
3509	91	1.041	Neg	6.5	1+
3510	91	1.025	Neg	6.0	1+
3511	8	1.019	Neg	7.5	Neg
3512	8	1.043	Neg	6.5	2+
3513	8	1.014	Neg	6.5	Neg
3514	29	1.011	Neg	6.5	Neg
3615	29	1.011	Neg	7.0	Neg

Appendix 16

Appendix 4

Individual Urinalysis Values

5550008

Sex: Female

3.3x 10E12 vg Group 4	Day(s) Relative to Start Date	Reporting Urinalysis VOLUME (mL)	COLOR	CLARITY	GLUC	BIL	KET
4601	8	24.0	LY	Clr	Neg	Neg	Neg
4502	8	8.3	LY	Clr	Neg	Neg	Neg
4503	91	9.0	LY	Clr	Neg	Neg	Neg
4504	91	6.0	LY	Clr	Neg	Neg	Neg
4505	29	0.7	LY	Clr	Neg	1+	Neg
4506	29	23.0	LY	Clr	Neg	Neg	Neg
4507	29	2.8	LY	Clr	Neg	Neg	1+
4508	8	15.2	LY	Clr	Neg	Neg	Neg
4509	8	3.5	DY	Clr	Neg	Neg	Neg
4510	8	9.4	LY	Clr	Neg	Neg	Neg
4511	29	2.0	LY	Clr	Neg	Neg	1+
4512	29	27.0	LY	Clr	Neg	Neg	Neg
4513	91	5.1	LY	Cld	Neg	Neg	Neg
4514	91	7.8	LY	Clr	Neg	Neg	Neg
4515	91	10.3	LY	Clr	Neg	Neg	Neg

Appendix 16

Appendix 4

Individual Urinalysis Values

5550008

Sex: Female

3.3x 10E12 vg Group 4	Day(s) Relative to Start Date	Reporting Urinalysis SPECIFIC GRAVITY	BLD	URINE pH	PROT
4601	8	1.008	Neg	7.5	Neg
4502	8	1.021	Neg	6.5	Neg
4503	91	1.017	Neg	6.5	Neg
4504	91	1.021	Neg	6.5	Neg
4505	29	1.100 [a]	Neg	6.0	2+
4506	29	1.007	Neg	7.0	Neg
4507	29	1.033	Neg	7.0	2+
4508	8	1.010	Neg	7.0	Neg
4509	8	1.049	Neg	6.0	2+
4510	8	1.017	Neg	6.5	Neg
4511	29	1.036	Neg	6.5	2+
4512	29	1.010	1+	7.0	Neg
4513	91	1.036	Neg	6.5	1+
4514	91	1.024	Neg	7.0	Neg
4515	91	1.015	Neg	6.5	Neg

[a] [RC:Assigned value above the reportable range]

SIGNATURE(S) FOR DOCUMENT: 5550008 - 5550008 Clinical Pathology Final Report

**Principal Investigator:**	I approve this document.
Name:	**Allegret, Virginie**

Allegret, Virginie

24-Feb-2022 20:18:02 (UTC+00:00)

Electronically Signed in M-Files

Timestamp

Appendix 17

Tissue Biodistribution, Gene Expression, and Splenocyte Analysis Report for Charles River Laboratories Montreal ULC Senneville Site (CR-SEN)

Corrections to the Tissue Biodistribution, Gene Expression, and Splenocyte Analysis report from the Sponsor issued on 19 January 2022. Additions are indicated in bold underlined text and deletions are indicated in bold strikethrough text according to the sections indicated below.

Section, Page Number: 3.2 Animal Studies, page 5

Error/Original wording:

Table 1. Experimental Design

Group No.	Test Material	Dose Level (vg/animal)	Dose Concentration (vg/ **µL**~~**mL**~~)	Dose Volume (µL)	Animal Numbers	
					Necropsy at day 29	
					Male	Female
1	Vehicle	0	0	60	5	5
2	AAV9/*AP4M1*	0.36 x 10^{12}	**0.18 x 10^{11}** ~~**5.43×10^{13}**~~	20	5	5
3	AAV9/*AP4M1*	1.1 x 10^{12}	**0.55 x 10^{11}** ~~**5.43×10^{13}**~~	20	5	5
4	AAV9/*AP4M1*	3.3 x 10^{12}	**0.55 x 10^{11}** ~~**5.43×10^{13}**~~	60	5	5

REPORT

AAV9/*AP4M1* study in rats: Biodistribution, Expression, and Immune Response

Work conducted at University of Texas Southwestern Medical Center in the laboratory of Dr. Steven Gray

Personnel and Role

- Xin Chen: Genomic DNA preparation, data compilation, and report preparation.
- Kathryn McMillan: ELISpot assay and report preparation.
- Sandra Unorji and Melissa Hyatt: qPCR assay
- Yang Yu: qPCR analysis and report preparation.
- Steven Gray: Supervisor and report approval.

I certify that, to the best of my knowledge, the information in this report is correct and a true representation of the work carried out.

Xin Chen Digitally signed by Xin Chen DN: cn=Xin Chen, c=US, o=UTSW Medical Center, email=Xin.Chen@UTSouthwestern.edu Reason: I am approving this document Date: 2022.01.18 17:08:20 -06'00'

Xin Chen Date

Kathryn McMillan Digitally signed by Kathryn McMillan DN: cn=Kathryn McMillan, c=US, o=UT Southwestern, email=kathryn.mcmillan@utsouthwestern.edu Date: 2022.01.18 16:44:24 -06'00' 01/18/2022

Kathryn McMillan Date

Sandra Unorji Digitally signed by Sandra Unorji DN: cn=Sandra Unorji, c=US, o=UT Southwestern Medical Center, ou=AAV Vector Core, email=sandra.unorji@utsouthwestern.edu Date: 2022.01.18 15:28:21 -06'00'

Sandra Unorji Date

Melissa Hyatt Digitally signed by Melissa Hyatt DN: cn=Melissa Hyatt, c=US, o=UT Southwestern Medical Center, ou=AAV Vector Core, email=melissa.hyatt@utsouthwestern.edu Date: 2022.01.18 15:21:42 -06'00'

Melissa Hyatt Date

Yang Yu Digitally signed by Yang Yu DN: cn=Yang Yu, c=US, o=UT Southwestern Medical Center, email=yang.yu@utsouthwestern.edu Date: 2022.01.18 15:38:12 -06'00'

Yang Yu Date

Steven James Gray Digitally signed by Steven James Gray DN: cn=Steven James Gray, c=US, o=University of Texas Southwestern Medical Center, ou=Department of Pediatrics, email=steven.gray@utsouthwestern.edu Date: 2022.01.18 16:56:02 -06'00'

Steven Gray Date

Table of Contents

1. OBJECTIVE 4
2. ABBREVIATIONS 4
3. MATERIALS AND METHODS 4
4. RESULTS AND DISCUSSION 5
5. CONCLUSIONS 7
6. REFERENCES 8
7. APPENDICES 17

List of Tables and Figures

Table 1. Experimental Design 5
Table 2. Raw data of biodistribution: copies of AP4M1 per rat diploid genome 17
Table 3. Raw data of expression: copies of AP4M1 per copy of GAPDH 19
Table 4. Raw data of immune response: Spot# in ELISpot plate 21
Figure 1. *AP4M1* biodistribution in WT rats. 6
Figure 2. *AP4M1* expression in WT rats. 6
Figure 3. Immune response by ELISpot assay 7

1. OBJECTIVE

The objective for this study was to characterize the biodistribution, expression, and immune response of self-complementary Adeno-Associated Virus serotype 9 (scAAV9)/UsP-*hAP4M1opt*-BGHpA (AAV9/*AP4M1*), following a single lumbar intrathecal (IT) injection in wild-type (WT) rats.

2. ABBREVIATIONS

AAV	Adeno-Associated Virus
AP4M1	Adaptor-related protein complex AP-4, μ4
BGH	Bovine growth hormone
DNA	Deoxyribonucleic acid
ELISpot	Enzyme-linked immunospot
INFγ	Interferon γ
IT	Lumbar intrathecal
PBS	Phosphate-buffered saline
PMA	Phorbol 12-myristate 13-acetate
qPCR	Ribonucleic acid
RNA	Quantitative PCR
Sc	Self-complementary
UsP	Promoter
vg	Vector genomes
WT	Wild type

3. MATERIALS AND METHODS

3.1 AAV9/*AP4M1* Production

AAV9/*AP4M1*in Phosphate-buffered saline (PBS), 5% Sorbitol, pH 7.4 containing 0.001% F-68 was produced by Viralgen in Spain. The final vector product (lot # T-GEMINIS-033) was prepared with a titer of 5.43×10^{13}vector genomes (vg) /mL (COA in section 7. APPENDICES).

3.2 Animal Studies

Animal study was performed by Charles River Laboratories, Inc. Quebec, Canada. Male and female rats were randomized into cohorts and dosed as shown in Table 1. At the initiation of dosing, the animals assigned to study were 7 weeks of age and weighed between 168-285g for males and 143-206g for females. AAV9/*AP4M1* vector was injected IT once on day 1 in each animal, in a volume of 20 or 60uL and a final dose of 0, 0.36×10^{12} 1.1×10^{12}, or 3.3×10^{12} vg/rat. Rats were sacrificed on day 8, 29, or91. Tissues were collected frozen, and lymphocytes were prepared, and then sent to Dr. Gray's laboratory on dry ice. Samples collected at day 29 were analyzed in this study.

Table 1. Experimental Design

Group No.	Test Material	Dose Level (vg/animal)	Dose Concentration (vg/mL)	Dose Volume (uL)	Animal Numbers	
					Necropsy at day 29	
					Male	Female
1	Vehicle	0	0	60	5	5
2	AAV9/*AP4M1*	0.36×10^{12}	5.43×10^{13}	20	5	5
3	AAV9/*AP4M1*	1.1×10^{12}	5.43×10^{13}	20	5	5
4	AAV9/*AP4M1*	3.3×10^{12}	5.43×10^{13}	60	5	5

3.3 Biodistribution and Expression Analysis by qPCR

Total genomic DNA was purified from tissue samples collected at necropsy day 29, using a Qiagen Qiacube HT kits. cDNA was synthesized from purified RNA and qPCR was used to determine the quantity of AAV9/*AP4M1* vector DNA biodistribution and *AP4M1* RNA transgene expression. Qualification of the qPCR assay is attached in section 6. REFERENCES.

3.4 Immune response by ELISpot

Both peptide library pools of AAV9 capsid and AP4M1 protein were purchased from Mimotopes, Victoria, Australia. The peptides libraries were comprised of 10-mers with a 5 amino acid offset. The AAV9 capsid library pool contained 147 peptides and he AP4M1 protein library pool contained 89 peptides. Both pools were stored at -80°C before use. ELISpot assays were performed using Rat IFN-γ Single-Color ELISPOT (ImmunoSpot, 96-well White). Briefly, splenocytes were thawed, washed, and resuspended in cRMPI-1640 medium for counting. 2×10^5 splenocytes in 100uL of cRPMI-1640 medium were plated into each well of an ELISpot plate. 100uL of cRPMI-1640 medium containing AAV9 capsid pool or AP4M1 protein pool at 1ug/well was then added to the wells. The controls included cells with no peptide, cells stimulated with a mixture of Phorbol 12-myristate 13-acetate (PMA) and Ionomycin (Invitrogen, 00-4970-93), medium with Recombinant Murine IFN-γ (PeproTech, 315-05), or medium only. The splenocytes were incubated for 48 hours in a humidified 37°C CO_2 incubator. The detailed steps of the ELISpot assay are attached in the references.

4. RESULTS AND DISCUSSION

An IT study was conducted in WT rats with each animal receiving a single injection of AAV9/*AP4M1* vector at a dose of 0, 0.36×10^{12}, 1.1×10^{12}, or 3.3×10^{12} vg. Genomic DNA was purified from the samples collected at necropsy day 29. *AP4M1* vector biodistribution was quantified by qPCR and provided in Figure 1. IT delivery of AAV9/*AP4M1* vector results in dose dependent increase of *AP4M1* vector DNA across the central nervous system and peripheral organs. The *AP4M1* vector DNA is widely detected at high level in multiple brain regions. In the peripheral organs, similar high amounts of *AP4M1* DNA persist in heart, liver, and spleen and to the less extent in other organs tested. The pattern of AP4M1 biodistribution in this study is consistent with that expected from AAV9. Consistent with this *AP4M1* DNA biodistribution data, *AP4M1* transgene expression is also widely detected at high level in multiple CNS and peripheral tissues (Figure 2). Collectively, IT delivery of AAV9/AP4M1 results in broad AP4M1 biodistribution and expression across the body of rats.

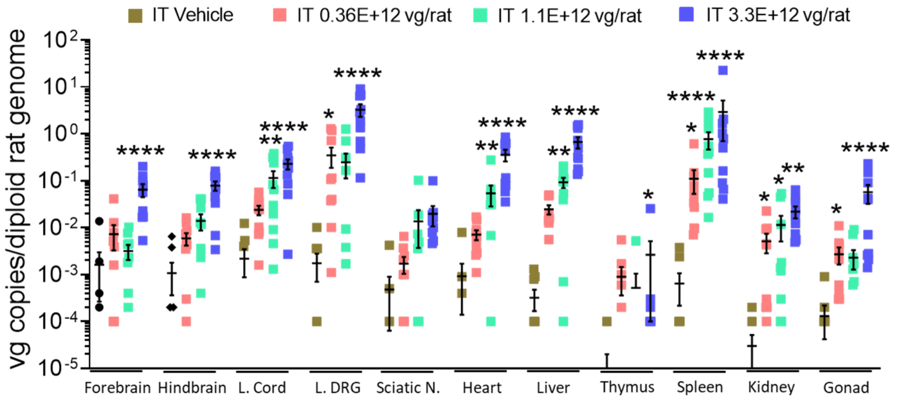

Figure 1. ***AP4M1* biodistribution in WT rats.**
WT Rats received a single IT injection of AAV9/*AP4M1* at a dose of 0, 0.36×10^{12}, 1.1×10^{12}, or 3.3×10^{12} vg. Genomic DNA was purified from the samples collected at necropsy day 29, and *AP4M1* vector biodistribution across the central nervous and peripheral organs was quantified by qPCR. Results (n=10) were presented as Mean±SEM. Data sets that passed tests for normality or homogeneity of variance were analyzed using the one-way ANOVA with Dunnett's correction for relevant pairwise comparisons. Data sets that did not pass tests for normality or homogeneity of variance were analyzed using Kruskal-Wallis test with Dunn's correction for relevant pairwise comparisons. *p<0.05, **p<0.01, ***p<0.001, and ****p<0.0001 compared to vehicle-treated group.

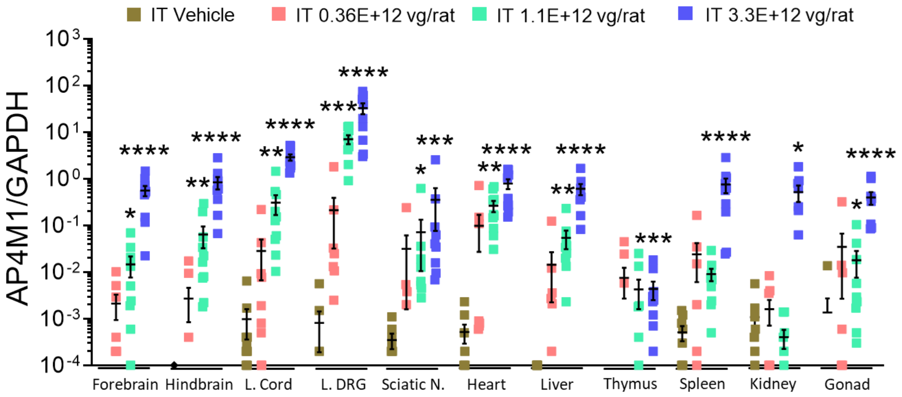

Figure 2. ***AP4M1* expression in WT rats.**
WT Rats received a single IT injection of AAV9/*AP4M1* at a dose of 0, 0.36×10^{12}, 1.1×10^{12}, or 3.3×10^{12} vg. Total RNA was purified from the samples collected at necropsy day 29. cDNA was synthesized from purified RNA and *AP4M1* expression across the central nervous and peripheral organs was quantified by qPCR. Results (n=10) were presented as Mean±SEM. Data sets that passed tests for normality or homogeneity of variance were analyzed using the one-way ANOVA with Dunnett's correction for relevant pairwise comparisons. Data sets that did not pass tests for normality or homogeneity of variance were analyzed using Kruskal-Wallis test with Dunn's correction for relevant pairwise comparisons. *p<0.05, **p<0.01, ***p<0.001, and ****p<0.0001 compared to vehicle-treated group.

Immune responses to AAV and/or transgene remain a major challenge that can confound the safety and efficacy of AAV-mediated gene transfer This study evaluate the T-cell INFγ immune response to AAV9/*AP4M1* vector in WT rats. Specifically, splenocytes from WT rats IT injected with AAV9/*AP4M1* vectors 29 days prior were plated and treated *in vitro* with either AAV9 capsid or AP4M1 protein peptide pools for 2 days along with both negative (no peptide) and positive (PMA + Ionomycin) controls.

While the negative control has 0 spots (Figure 3), the positive control has many spots (Table 4). Panel A and B in Figure 3 show the INFγ response to the AAV9 capsid pool and AP4M1 protein pool, respectively. None of the vehicle, low (0.36×10^{12} vg), mid (1.1×10^{12} vg), or high (3.3×10^{12} vg) dose of AAV9/*AP4M1* vector generated significant increase of the spot numbers compared to negative control, indicating that the AAV9/*AP4M1* vector generated minimal T-cell immune response in WT rats, to either AAV9 or the human AP4M1 protein.

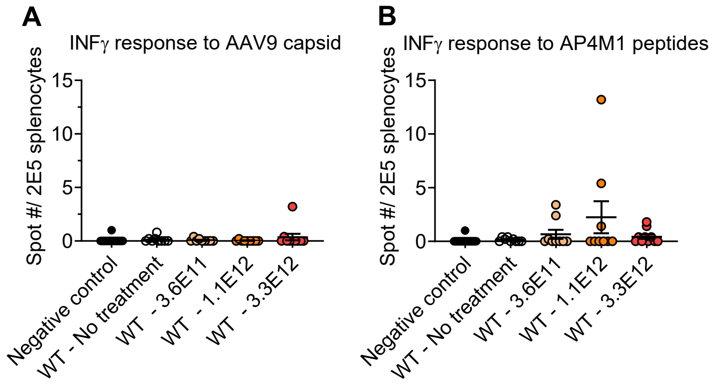

Figure 3. Immune response by ELISpot assay

Spot numbers in each well were blindly counted with a specialized automated ELISpot reader. Each data point represents the mean value of duplicate assay for negative control or the mean value of quadruplicate assay from an individual animal, with lines representing the mean measurement ± SEM. Data sets in Figure 3 did not pass tests for normality or homogeneity of variance and therefore were analyzed using Kruskal-Wallis test with Dunn's correction for relevant pairwise comparisons. No significance between any groups were observed.

5. CONCLUSIONS

The AAV9 vector genome DNA biodistribution pattern should be dependent on the capsid, regardless of the DNA cargo that it carries, which is further supported by the similar pattern of DNA biodistribution from this study using AAV9/*AP4M1* as that expected for AAV9. The exceptions to this would be if an immune response led to viral clearance, or if there was toxicity against the expressed transgene that led to cell death and loss of viral genomes. In this study, it was concluded that AAV9 delivered IT can achieve broad distribution across the nervous system and peripheral organs without generating significant immune response, although the level of gene transfer in the brain is sub-saturating with a minority of cells receiving the transgene. Thus, this study is considered to portray the normal biodistribution and expression pattern expected for an AAV9 vector in Rats. These results do not suggest any loss of vector due to cellular toxicity.

6. REFERENCES

6.1 Standard Operating Procedure

6.1.1 DNA Extraction from Animal Tissues using QIAamp 96 DNA QIAcube HT Kit (5) (Qiagen, 51331).

DNA extraction was performed per manufacture recommendation except that the buffer used was not RTL buffer but ATL buffer. Prepared ATL + Proteinase K at (33mL ATL + 3 mL ProK). Used 400uL for non-CNS tissues and 300uL for CNS tissues. After 2 rounds of Tissuelyser II, incubated overnight and transferred 200uL to fresh S-block for extraction. Considered safe outside of BSL-2 post incubation.

6.1.2 Total RNA Extraction from Animal Tissues using RNeasy 96 QIAcube HT Kit (5) (Qiagen, 74171).

RNA extraction was performed per manufacture recommendation except that buffer was Qiazol reagent, 750mL. Homogenized twice, incubated at room temperature for 5 min, added 150uL chloroform, shaken vigorously. Spun at top speed for 1min, then pulled top layer (300uL) into new S-block for extraction. Considered safe outside of BSL-2 after chloroform step.

6.1.3 cDNA Synthesis for qPCR with RT2 HT First Strand Kit (Qiagen, 330411)

cDNA synthesis was performed per manufacture recommendation.

6.1.4 Quantification of AP4M1 inrat gDNA samples.

Prepared by Yang Yu, UTSW Medical Center
Version: **09-06-2021**

A. Overview
This protocol is designed to use quantitative PCR (qPCR) to determine the double-stranded copies of the AP4M1 DNA present in a purified genomic DNA sample. This SOP has been optimized and validated for use with rat genomic DNA. The total amount of sample DNA (host genomes) is determined by SYBR green qPCR analysis with primers specific to rat GADPH, and the copies of hAP4M1opt DNA within each sample is determined by SYBR green qPCR analysis with primers specific for hAP4M1opt.

B. Quantification of hAP4M1 DNA in gDNA sample

1. *Make plasmid DNA standards*

Dilute linearized plasmid DNA (pTRS-UsP-*hAP4M1opt*-BGHpA) to 1.59×10^9 double-stranded copies/ µL stock in 10 mM Tris-EDTA buffer pH 8 (Invitrogen Cat# 9858) in 1.5 mL siliconized tubes (Fisher Cat# 02681331). Make a first dilution 1:200 to 7.95×10^6 copies/µL following by serial 1:10 dilutions to 7.95copies/µL. 2 µL of each dilution are loaded to each reaction. All dilutions are prepared with 10 mM Tris prepared from 1M Tris pH 8 (Invitrogen Cat# AM9855G) with UltraPure DNase/RNase-free distilled water (Invitrogen Cat# 10977-015). Vortex and spin briefly in every step.

1.59×10^7 copies/reaction — 2 µL of 1.59×10^8 copies/µL stock + 398 µL of 10 mM Tris
10 µL ↓
1.59×10^6 copies/reaction — + 90 µL of 10 mM Tris
10 µL ↓

1.59×10^5 copies/reaction + 90 µL of 10 mM Tris
10 µL
1.59×10^4 copies/reaction + 90 µL of 10 mM Tris
10 µL
1.59×10^3 copies/reaction + 90 µL of 10 mM Tris
10 µL
1.59×10^2 copies/reaction + 90 µL of 10 mM Tris
10 µL
15.9copies/reaction

2. *Prepare the qPCR SYBR master reactions*
   1) Set up gDNA samples as follows.
      2× SYBR master mix 10 µL (Roche Cat# 04887352001)
      Forward Primer (20 µM) 0.5 µL (CCCTGGGCGAAGGAACTATC, IDT, Inc)
      Reverse Primer (20 µM) 0.5 µL (CACAGCCTCGGTCTGAATGA, IDT, Inc)
      H_2O 7 µL (Teknova Cat# W3440)
      a. Pipet 18 µL of master mix into each well intended for gDNA samples.
      b. Add 2 µL of sample gDNA to the well.

   2) Set up standard plasmid DNA as follows.
      2× SYBR mater mix 10 µL (Roche Cat# 04887352001)
      Forward Primer (20 µM) 0.5 µL (CCCTGGGCGAAGGAACTATC, IDT, Inc)
      Reverse Primer (20 µM) 0.5 µL (CACAGCCTCGGTCTGAATGA, IDT, Inc)
      H_2O 7 µL (Teknova Cat# W3440)
      a. Pipet 18 µL of master mix into each well intended for standard curve samples.
      b. Add 2 µL of plasmid DNA standard to the appropriate well.
      c. Add H_2O as no template control.
      d. Seal the plate with the seal for qPCR usage. (Roche Cat# 04-729-749-001)
      e. Spin down the plate @ 1800 rpm for 10 sec.
      f. Cycle in the Roche LightCycler480.

C. Quantification of Rat GAPDH

1. *Make genomic DNA standards*
   1) Measure the rat liver gDNA concentration using the LVis plate with the CLARIOstar plate reader (BMG LABTECH). Use the concentration of 45.35 ng/µL as the highest dilution stock.
   2) Make 6 serial 1:4 dilutions with 10 mM Tris from 45.35 ng/µL to 0.0111ng/µL.

2. *Preparation of gDNA samples for qPCR run*
   1) Mix samples by pipetting up and down, then spin at 1800 rpm for 10 sec.

3. *Prepare the qPCR SYBR master reactions*
   1) Set up standard gDNA and gDNA samples as follows.
      2× SYBR mater mix 10 µL (Roche Cat# 04887352001)
      Forward Primer (20 µM) 0.25 µL (ACTCTACCCACGGCAAGTTC, Sigma)
      Reverse Primer (20 µM) 0.25 µL (TGGGTTTCCCGTTGATGACC, Sigma)
      H_2O 7.5 µL (PCR water Teknova Cat# W3440)

a. Pipet 18 µL of master mix into each well intended for gDNA samples.
b. Add 2 µL of gDNA standard or gDNA samples
c. Add H_2O as no template control.
d. Seal the plate with the seal for qPCR usage. (Roche Cat# 04-729-749-001)
e. Spin down the plate @ 1800 rpm for 10 sec.
f. Cycle in the Roche LightCycler480.

D. Running Cycles

hAP4M1opt

	**Target (^{0}C)**	**Time (hh:mm:ss)**	**Cycle**	**Acquisition Mode**	**Ramp Rate**	**Acquisitions**	**Detect Mode**
Denature	95	0:10:00	1	none	4.8		SyBr green I/HRM Dye
Amplification	95	0:00:10	55	none	4.8		
	60	0:00:10		none	2.5		
	72	0:00:10		single	4.8		
Melt	95	0:00:05	1	none	4.8		
	65	0:01:00		none	2.5		
	95			continuous	0.11	5/^{0}C	
Cool	40	0:00:10	1	none	2.5		

Rat GAPDH

	**Target (^{0}C)**	**Time (hh:mm:ss)**	**Cycle**	**Acquisition Mode**	**Ramp Rate**	**Acquisitions**	**Detect Mode**
Denature	95	0:10:00	1	none	4.8		SyBr green I/HRM Dye
Amplification	95	0:00:10	45	none	4.8		
	60	0:00:10		none	2.5		
	72	0:00:10		single	4.8		
Melt	95	0:00:05	1	none	4.8		
	65	0:01:00		none	2.5		
	95			continuous	0.11	5/^{0}C	
Cool	40	0:00:10	1	none	2.5		

E. Analysis

Use Ab Quant/ 2nd derivative max in LightCycler 480 v1.5 software to calculate the number of genomes per sample relative to the plasmid DNA standard curve or the amount of host DNA in ng relative to the rat gDNA standard. Use Tm calling as a quality control to check whether the specific product was amplified.

Calculation of number of copies of viral genome/µL

$$= \frac{\mathit{Copy\ of\ virus\ genome\ as\ double\ strand\ DNA\ relative\ to\ the\ copy\ number\ of\ plasmid\ DNA\ standard}}{2}$$

- *Calculation of genome copies and amount (µg) of host DNA relative to the ng amount of host DNA standard*

This calculation assumes that the average weight of a bp of a double strand DNA is 620 g/mol, thus the molecular weight of DNA of 3 billion bps in a haploid cell is 1.85×10^{12} g/mol. The quantity of DNA in each cell contains (1.85×10^{12} g/mol)×(1 mole/6.022×10^{23} molecules)×2=6 pg of diploid DNA, so 1 pg of DNA contains 0.167 double strand copies=0.334 single-stranded copies of DNA, and 1 ng=334 single-stranded copies.

Number of rat GAPDH genome copies = $\frac{ng*334\ (single\ stranded\ copies=genome)}{dilution\ \ factor*2}$

Conversion from ng/µL to µg/µL amount of host DNA= ng/µL of host DNA ×1000

- *Calculation of number of copies of Viral genomes normalized to number of copies of genome host*

$=\frac{Copies\ of\ viral\ genome/\mu L}{Copies\ of\ genome\ DNA\ in\ host/\mu L}$

- Calculation of number of copies of Viral genomes normalized to amount of host DNA host (µg)

$=\frac{Copies\ of\ viral\ genome/\mu L}{amount\ of\ DNA\ in\ host\ (\mu g)/\mu L}$

6.1.5 Quantification of AP4M1 in monkey cDNA samples.

Prepared by Yang Yu, UTSW Medical Center
Version: **09-06-2021**

A. Overview

This protocol is designed to use quantitative PCR (qPCR) to determine the single-stranded copies of the *AP4M1* cDNA present in a synthesized cDNA sample. This SOP has been optimized and validated for use with monkey cDNA. The total amount of sample cDNA (host genomes) is determined by SYBR green qPCR analysis with primers specific to Monkey GADPH, and the copies of hAP4M1opt cDNA within each sample is determined by SYBR green qPCR analysis with primers specific for hAP4M1opt.

B. Quantification of hAP4M1 DNA in cDNA sample

1. *Make plasmid DNA standards*

Dilute linearized plasmid DNA (pTRS-UsP-*hAP4M1opt*-BGHpA) to 1.59×10^9 double-stranded copies/ µL stock in 10 mM Tris-EDTA buffer pH 8 (Invitrogen Cat# 9858) in 1.5 mL siliconized tubes (Fisher Cat# 02681331). Make a first dilution 1:200 to 7.95×10^6 copies/µL following by serial 1:10 dilutions to 7.95copies/µL. 2 µL of each dilution are loaded to each reaction. All dilutions are prepared with 10 mM Tris prepared from 1M Tris pH 8 (Invitrogen Cat# AM9855G) with UltraPure DNase/RNase-free distilled water (Invitrogen Cat# 10977-015). Vortex and spin briefly in every step.

1.59×10^7 copies/reaction — 10 µL of 1.59×10^9 copies/µL stock + 90 µL of 10 mM Tris
10 µL ↓
1.59×10^6 copies/reaction — + 90 µL of 10 mM Tris
10 µL ↓
1.59×10^5 copies/reaction — + 90 µL of 10 mM Tris
10 µL ↓
1.59×10^4 copies/reaction — + 90 µL of 10 mM Tris
10 µL ↓
1.59×10^3 copies/reaction — + 90 µL of 10 mM Tris
10 µL ↓

1.59×10^2 copies/reaction + 90 µL of 10 mM Tris
10 µL
15.9copies/reaction

2. *Prepare the qPCR SYBR master reactions*
   1) Set up cDNA samples as follows.

2× SYBR master mix	10 µL (Roche Cat# 04887352001)
Forward Primer (20 µM)	0.5 µL (CCCTGGGCGAAGGAACTATC, IDT, Inc)
Reverse Primer (20 µM)	0.5 µL (CACAGCCTCGGTCTGAATGA, IDT, Inc)
H_2O	7 µL (Teknova Cat# W3440)

   a. Pipet 18 µL of master mix into each well intended for cDNA samples.
   b. Add 2 µL of sample cDNA to the well.

   2) Set up standard plasmid DNA as follows.

2× SYBR mater mix	10 µL (Roche Cat# 04887352001)
Forward Primer (20 µM)	0.5 µL (CCCTGGGCGAAGGAACTATC, IDT, Inc)
Reverse Primer (20 µM)	0.5 µL (CACAGCCTCGGTCTGAATGA, IDT, Inc)
H_2O	7 µL (Teknova Cat# W3440)

   g. Pipet 18 µL of master mix into each well intended for standard curve samples.
   h. Add 2 µL of plasmid DNA standard to the appropriate well.
   i. Add H_2O as no template control.
   j. Seal the plate with the seal for qPCR usage. (Roche Cat# 04-729-749-001)
   k. Spin down the plate @ 1800 rpm for 10 sec.
   l. Cycle in the Roche LightCycler480.

C. Quantification of Monkey GAPDH

1. *Make genomic DNA standards*
   1) Measure the monkey liver gDNA concentration using the LVis plate with the CLARIOstar plate reader (BMG LABTECH). Use the concentration of 58.075ng/µL as the highest dilution stock.
   2) Make 6 serial 1:4 dilutions with 10 mM Tris from 58.075ng /µL to 0.0142ng/µL.

2. *Preparation of cDNA samples for qPCR run*
   1) Mix samples by pipetting up and down, then spin at 1800 rpm for 10 sec.

3. *Prepare the qPCR SYBR master reactions*
   1) Set up standard gDNA and cDNA samples as follows.

2× SYBR mater mix	10 µL (Roche Cat# 04887352001)
Forward Primer (20 µM)	0.25 µL (GGCCTCCAAGGAGTAAGACC, Sigma)
Reverse Primer (20 µM)	0.25 µL (TCTCTTCCTCTTGTGCTCTCG, Sigma)
H_2O	7.5 µL (PCR water Teknova Cat# W3440)

      a. Pipet 18 µL of master mix into each well intended for cDNA samples.
      b. Add 2 µL of gDNA standard or cDNA samples
      c. Add H_2O as no template control.
      d. Seal the plate with the seal for qPCR usage. (Roche Cat#04-729-692-001)
      e. Spin down the plate @ 1800 rpm for 10 sec.
      f. Cycle in the Roche LightCycler480.

D. Running Cycles

hAP4M1opt

	**Target (^{0}C)**	**Time (hh:mm:ss)**	**Cycle**	**Acquisition Mode**	**Ramp Rate**	**Acquisitions**	**Detect Mode**
Denature	95	0:10:00	1	none	4.8		SyBr green I/HRM Dye
Amplification	95	0:00:10	55	none	4.8		
	60	0:00:10		none	2.5		
	72	0:00:10		single	4.8		
Melt	95	0:00:05	1	none	4.8		
	65	0:01:00		none	2.5		
	95			continuous	0.11	5/^{0}C	
Cool	40	0:00:10	1	none	2.5		

Monkey GAPDH

	**Target (^{0}C)**	**Time (hh:mm:ss)**	**Cycle**	**Acquisition Mode**	**Ramp Rate**	**Acquisitions**	**Detect Mode**
Denature	95	0:10:00	1	none	4.8		SyBr green I/HRM Dye
Amplification	95	0:00:10	45	none	4.8		
	60	0:00:10		none	2.5		
	72	0:00:10		single	4.8		
Melt	95	0:00:05	1	none	4.8		
	65	0:01:00		none	2.5		
	95			continuous	0.11	5/^{0}C	
Cool	40	0:00:10	1	none	2.5		

E. Analysis

Use Ab Quant/ 2nd derivative max in LightCycler 480 v 1.5 software to calculate the number of genomes per sample relative to the plasmid DNA standard curve or the amount of host cDNA in ng relative to the monkey gDNA standard. Use Tm calling as a quality control to check whether the specific product was amplified.

Calculation of number of copies of viral genome/µL

= Copy of virus genome as single strand cDNA relative to the copy number of plasmid DNA standard

- *Calculation of genome copies and amount (µg) of host cDNA relative to the ng amount of host DNA standard*

This calculation assumes that the average weight of a bp of a double strand DNA is 620 g/mol, thus the molecular weight of DNA of 3 billion bps in a haploid cell is 1.85×10^{12} g/mol. The quantity of DNA in each cell contains (1.85×10^{12} g/mol)×(1 mole/6.022×10^{23} molecules)×2=6 pg of diploid DNA, so 1 pg of DNA

contains 0.167 double strand copies=0.334 single-stranded copies of DNA, and 1 ng=334 single-stranded copies of DNA.

$$\text{Number of monkey GAPDH genome copies} = \frac{ng*334\ (single\ stranded\ copies=genome)}{dilution\ \ factor*2}$$

Conversion from ng/μL to μg/μL amount of host DNA= ng/μL of host DNA ×1000

- *Calculation of number of copies of Viral genomes normalized to number of copies of genome host*

$$= \frac{Copies\ of\ viral\ genome/\mu L}{Copies\ of\ genome\ DNA\ in\ host/\mu L}$$

- Calculation of number of copies of Viral genomes normalized to amount of host DNA host (μg)

$$= \frac{Copies\ of\ viral\ genome/\mu L}{amount\ of\ DNA\ in\ host\ (\mu g)/\mu L}$$

6.1.6 Biodistribution qPCR Validation Studies.

Prepared by Yang Yu, UTSW Medical Center

Version: **01-13-2022**

A. Validation of pTRS-UsP-hAP4M1opt-BGHpA detection in no matrix.

Overview

The plasmid, pTRS-UsP-*hAP4M1opt*-BGHpA, was used as a template in 2 SYBR reactions using the hAP4M1opt primer set 2 to validate PCR efficiency and lower detection threshold.

Plasmid dilutions in no matrix

The plasmid was diluted from 1.59×10^7 copies to 1 copy, and 2 replicates of serial dilution were used in 2 independent runs (4 replicates total). The 2nd derivative max and fit points algorithm were applied to the analysis. T_m analysis was used to determine PCR product purity, which is also confirmed by using agarose gel electrophoresis. Among all samples, 100% of replicates at or above 1 copy per reaction were successfully detected. Conservatively, we set the lower limit of detection (LLOD) at 4 copies of the plasmid per reaction. One out of 4 replicates amplified in the negative control, and it was detected as 1.36 copies, which is below the LLOD (4 copies) that was used for data analysis. The PCR amplification efficiency was between 1.916 and 1.935 using the 2nd derivative max algorithm.

The variability (range) of the lower limit of quantitation (LLOQ) for detection of the AP4M1opt plasmid sequence was 11.1 to 20.8 copies detected with an input of 15.9 copies,3.69 to 8.75 copies detected at 8 copies level, 2.2 to 8.47 copies at 4 copies,1.06 to 4.78 at 2 copies, and 0.984 to 2.22 copies at 1 copy level in SYBR reaction with AP4M1opt primer set 2.

Results

Our qPCR validation studies detected the plasmid pTRS-UsP-hAP4M1opt-BGHpA, LLOD, and LLOQ when plasmid DNA alone was measured. Overall, our LLOD was 100% success in detection of 4 copies of AP4M1opt per reaction.

B. Validation ofpTRS-UsP-hAP4M1opt-BGHpAdetection in a rat genomic DNA matrix.

Overview

The plasmid, pTRS-UsP-*hAP4M1opt*-BGHpA, together with a matrix of rat genomic DNA, were used as a template in 2 SYBR reactions using the hAP4M1opt primer set 2 to validate PCR efficiency and lower detection threshold.

Plasmid dilutions in gDNA matrix

The plasmid was diluted from 1.59×10^7 copies to 1 copy, and 2 replicates of serial dilution were used in 2 independent runs (4 replicates total). All the dilutions were carried out in a matrix of 100 ng rat genomic DNA. The 2nd derivative max and fit points algorithm were applied to the analysis. T_m analysis was used to determine PCR product purity, which is also confirmed by using agarose gel electrophoresis. For these results, minor background amplification was detected above a background signal. Among all reactions, 100% of replicates at or above 1 copy per reaction were successfully detected. Conservatively, the LLOD was set at 4 copies of plasmid per reaction. Two out of 4 replicates amplified in the negative control reactions, and they were detected as 0.134 and 1.39 copies, which is below the LLOD (4 copies) that was used for data analysis. The efficiency was between 1.938 and 1.946 using the 2nd derivative max algorithm.

The variability (range) of the LLOQ for detection of the AP4M1opt plasmid sequence in the matrix of 100 ng rat genomic DNA was 7.89 to 16.2 copies detected with an input of 15.9 copies, 4.21 to 12.9 copies detected at 8 copies level, 0.816 to 5.31copies at 4 copies, 0.777 to 2.79 at 2 copies, and 1.15 to 6.58 copies at 1 copy level in SYBR reaction with AP4M1opt primer set 2.

Results

Our qPCR validation studies detected the plasmid pTRS-UsP-hAP4M1opt-BGHpAat comparable efficiencies, LLOD, and LLOQ regardless of whether plasmid DNA alone was measured, or the plasmid DNA was detected in a matrix of rat genomic DNA. In the data analysis of the actual samples that were run, any values below the threshold were considered as too low to call and thus excluded. Overall, our LLOD was 100% successful in the detection of 4 copies of AP4M1opt sequence in 100 ng of rat genomic DNA (40 copies/ug), which conforms to the Food and Drug Administration (FDA) guidelines on conducting vector genome biodistribution studies. This plasmid is our production plasmid to make the proposed clinical vector scAAV9/AP4M1opt. We conclude that these PCR conditions can be used to detect the copies ofAP4M1opt viral genome in rat genomic DNA samples.

6.1.7 ELISpot Using CTL Test Kits.

Prepared by Kathryn McMillan, UTSW Medical Center
Version:**1.2**

Under sterile conditions, prepped Capture solution according to manufacturer's specifications. 40uL anti-INFγ capture solution was added to 10 mL of Diluent A, and 80uL of solution was added to each well. Plate was incubated overnight at 4°C in a fridge for 16 hours.

After 16-hour incubation period, the sample prep was begun on in a designated BSL-2 hood under sterile conditions. 15 mL conical tubes were prepped with 9mL of warmed media to do a 1:10 dilution with frozen cells. Frozen splenocytes and lymphocytes were pulled out of liquid nitrogen storage onto dry ice for transport, then thawed in a 37°C water bath. Each cell sample mix was pipetted gently twice, then placed into the designated conical with warmed media. Samples were spun at 330xg for 10 minutes to pellet live cells, media was aspirated, and another 1mL of media was added. Cells were resuspended for counting on a

hemocytometer. Cells were counted and diluted to a final concentration of 2.0e6 cells/mL. Cell suspensions were then placed on wet ice while controls were prepared to avoid cell death. AAV9 peptide pools were prepared by adding 156.9uL of prepared AAV9 peptide pool stock to 10mL media. AP4M1 peptide pools were prepared by adding 100.6uL of prepared AP4M1 peptide pool stock to 10mL media. DMSO concentrations were matched by adding 12.6uL DMSO. Positive and negative controls were prepped according to current SOP. Cells+ No Peptide was prepped by adding 6.24uL DMSO to 400uL media in a 1.5mL centrifuge tube. Cells+ Stim was prepped by adding 6.24uL DMSO and 0.8uL P+I Stimulation Cocktail to 400uL media in a 1.5mL centrifuge tube. No Cells+ INFγ control was prepped by adding 10uL IFNγ aliquot prepped ahead of time to 400uL media in a 1.5mL centrifuge tube. No Cells control was prepped by placing 400uL media in a 1.5mL centrifuge tube.

Under sterile conditions, ELISpot plate was aspirated, and each well was washed in 150uL 1× D-PBS then aspirated again. 100uL of each cell suspension was loaded into the corresponding wells designated on the plate layout. Remaining cell suspensions were pooled together. To Cells-No Peptide control wells, 100uL of prepared control was added to the well after 100uL of cell pool was loaded in. To Cells- Stim control wells, 100uL of prepared control was added to the well after 100uL of cell pool was loaded in. To No Cells + IFNγ control wells, 100uL of peptide mix was added to the well after 100uL of prepared control was loaded in. To No Cells control wells, 100uL of peptide mix was added to the well after 100uL of media was loaded in. With a multichannel pipette, 100uL of peptide mix was added to all remaining experimental wells. Plate was wrapped in foil and incubated 24 hours at 37°C with 5-7% CO_2.

30 minutes before incubation is complete, 1 liter of 1x PBS, distilled water and 1X Tween-PBS 0.05% wash solutions for developing the plate were prepared according to manufacturer's directions. Just before incubation was complete, Detection solution was prepared by diluting 40 ul of anti-INFγ Biotinylated Antibody in 10mL Diluent B. Plate washed twice in 200uL of PBS, and twice in 200uL of PBS-T. Plate was loaded with 80uL Detection Solution in each well, then incubated at room temperature for 2 hours. Just before incubation was complete, Tertiary Solution was prepared by diluting 10uL streptavidin supplied by the manufacturer into 10mL of diluent C. Plate was washed 3 times in 200uL of PBS-T and once in 200uL of PBS, then 80uL of Tertiary Solution was added into each well and incubated at room temperature for 30 minutes. Just before incubation was complete, Developer Solution was prepared according to the manufacturer. To 10mL of Diluent Blue, 160uL of S1 was added and mixed by inversion four times. 160uL S2 was then added and mixed by inversion four times. 93uL S3 was then added and mixed by inversion four times. The conical tube was wrapped in foil to protect from light. Plate was washed twice in 200uL of PBS-T and twice in 200uL of distilled water, then 80uL of Developer Solution was added into each well and incubated at room temperature for 15 minutes. Reaction was stopped by gently rinsing plate membrane under running tap water and decanting several times according to manufacturer's instructions. Plate was left on paper towels to dry overnight before scanning and counting spots.

7. APPENDICES

7.1 Raw Data

Table 2. Raw data of biodistribution: copies of AP4M1 per rat diploid genome

ID	Forebrain	Hindbrain	L. Cord	L. DRG	Sciatic	Heart	Liver	Thymus	Spleen	Kidney	Gonad
1003	0.0000	0.0000	0.0000	0.0000	0.0000	0.0000	0.0009	0.0000	0.0000	0.0000	0.0000
1004	0.0000	0.0000	0.0000	0.0000	0.0000	0.0000	0.0001	0.0000	0.0001	0.0000	0.0000
1008	0.0000	0.0002	0.0000	0.0001	0.0000	0.0004	0.0000	0.0000	0.0000	0.0000	0.0001
1009	0.0002	0.0000	0.0000	0.0000	0.0001	0.0000	0.0001	0.0000	0.0000	0.0000	0.0001
1010	0.0000	0.0002	0.0000	0.0000	0.0000	0.0009	0.0000	0.0000	0.0000	0.0002	0.0000
1508	0.0000	0.0000	0.0000	0.0000	0.0005	0.0000	0.0000	0.0000	0.0000	0.0000	0.0000
1509	0.0000	0.0000	0.0000	0.0000	0.0000	0.0000	0.0000	0.0000	0.0000	0.0000	0.0000
1513	0.0004	0.0000	0.0053	0.0036	0.0000	0.0000	0.0000	0.0000	0.0001	0.0000	0.0000
1514	0.0019	0.0038	0.0040	0.0036	0.0000	0.0000	0.0008	0.0001	0.0038	0.0001	0.0002
1515	0.0137	0.0065	0.0124	0.0101	0.0042	0.0079	0.0013	0.0000	0.0024	0.0000	0.0009
2001	0.0051	0.0130	0.0296	0.0375	0.0021	0.0078	0.0056	0.0017	0.0502	0.0002	0.0006
2002	0.0412	0.0161	0.0255	0.0101	0.0065	0.0169	0.0205	0.0055	0.0098	0.0043	0.0021
2011	0.0128	0.0087	0.0103	0.1145	0.0010	0.0092	0.0496	0.0006	0.0096	0.0226	0.0034
2012	0.0016	0.0053	0.0081	1.1743	0.0037	0.0052	0.0157	0.0002	0.1260	0.0093	0.0044
2013	0.0079	0.0035	0.0016	0.7169	0.0010	0.0026	0.0485	0.0000	0.0271	0.0045	0.0047
2501	0.0039	0.0074	0.0072	0.0399	0.0025	0.0035	0.0162	0.0010	0.6136	0.0097	0.0109
2502	0.0001	0.0043	0.0359	0.0011	0.0000	0.0066	0.0126	0.0000	0.0788	0.0000	0.0000
2507	0.0000	0.0001	0.0395	0.1067	0.0000	0.0011	0.0139	0.0000	0.1481	0.0002	0.0004
2508	0.0000	0.0000	0.0569	0.0000	0.0001	0.0029	0.0148	0.0000	0.0070	0.0001	0.0003
2509	0.0001	0.0003	0.0242	1.2817	0.0001	0.0149	0.0475	0.0000	0.0337	0.0003	0.0003
3004	0.0000	0.0004	0.0091	0.0038	0.0000	0.0661	0.0391	0.0000	0.3697	0.0117	0.0017
3005	0.0002	0.0036	0.0046	0.1662	0.0036	0.0308	0.0992	0.0000	1.8860	0.0448	0.0091
3009	0.0004	0.0117	0.2389	0.0000	0.0049	0.0068	0.1246	0.0000	2.9036	0.0001	0.0000
3010	0.0018	0.0263	0.3798	0.7030	0.0073	0.0511	0.0531	0.0000	0.4082	0.0012	0.0072
3011	0.0023	0.0128	0.3160	0.3129	0.0001	0.0001	0.1151	0.0000	0.0469	0.0003	0.0007
3504	0.0044	0.0410	0.0299	0.0000	0.0000	0.0432	0.0007	0.0052	0.5560	0.0014	0.0000
3505	0.0103	0.0412	0.0652	1.2609	0.1014	0.2751	0.0933	0.0000	0.0547	0.0531	0.0013
3506	0.0000	0.0000	0.0013	0.0000	0.0000	0.0000	0.0001	0.0000	0.0165	0.0000	0.0000
3514	0.0046	0.0023	0.0825	0.0017	0.0187	0.0311	0.1924	0.0000	1.2855	0.0018	0.0006
3515	0.0077	0.0000	0.0196	0.0093	0.0001	0.0348	0.2064	0.0000	0.1475	0.0001	0.0023
4003	0.0184	0.0455	0.4740	6.7598	0.0189	0.6674	1.3190	0.0000	0.0654	0.0053	0.0026
4004	0.0565	0.0335	0.1347	0.1151	0.0140	0.0782	0.2980	0.0001	0.0903	0.0056	0.0909
4005	0.0225	0.1612	0.1850	0.4821	0.0079	0.8545	0.1567	0.0000	0.1631	0.0122	0.1485
4012	0.2009	0.1176	0.2693	3.2566	0.0100	0.5967	1.5607	0.0002	1.6513	0.0639	0.0423
4013	0.1262	0.1452	0.2278	8.9895	0.0000	0.4276	0.1791	0.0001	0.7792	0.0074	0.0028
4505	0.0167	0.1247	0.3150	0.6948	0.0162	0.0886	0.4999	0.0003	2.0549	0.0417	0.0021
4506	0.0053	0.0068	0.0853	6.3848	0.0046	0.1149	0.1372	0.0001	0.0406	0.0049	0.0071
4507	0.0168	0.0034	0.0513	2.8277	0.0052	0.0352	0.7226	0.0001	22.6697	0.0186	0.2291

4511	0.1004	0.0649	0.5397	1.3556	0.0991	0.6347	1.4692	0.0000	1.0303	0.0235	0.0396
4512	0.0801	0.0863	0.0027	1.8793	0.0210	0.0581	0.3194	0.0254	0.5016	0.0365	0.0014

Table 3. Raw data of expression: copies of AP4M1 per copy of GAPDH

	Forebrain	Hindbrain	L. Cord	L. DRG	Sciatic	Heart	Liver	Thymus	Spleen	Kidney	Gonad
1003	0.0000	0.0000	0.0017	0.0000	0.0011	0.0001	0.0001	0.0000	0.0006		0.0000
1004	0.0000	0.0000	0.0004	0.0002	0.0003	0.0023	0.0001	0.0000	0.0001	0.0017	0.0000
1008	0.0000	0.0000	0.0004	0.0000	0.0006	0.0012	0.0001	0.0000	0.0003	0.0000	0.0000
1009	0.0000	0.0000	0.0001	0.0000	0.0002	0.0005	0.0000	0.0000	0.0008	0.0004	0.0000
1010	0.0000	0.0000	0.0000	0.0000	0.0004	0.0001	0.0001	0.0000	0.0000	0.0056	0.0000
1508	0.0000	0.0000	0.0002			0.0002	0.0000	0.0000		0.0000	0.0000
1509	0.0000	0.0000	0.0001	0.0056		0.0005	0.0000	0.0000	0.0015	0.0011	0.0000
1513	0.0000	0.0001	0.0003	0.0000	0.0002	0.0002	0.0000	0.0000	0.0012	0.0006	0.0000
1514	0.0000	0.0000	0.0064	0.0000	0.0000	0.0000	0.0000	0.0000	0.0001	0.0002	0.0000
1515	0.0000	0.0000	0.0002	0.0015	0.0000	0.0001	0.0000	0.0000	0.0000	0.0001	0.0136
2001	0.0002	0.0000	0.0001	0.0106	0.0000	0.0009	0.0121	0.0000	0.0002	0.0000	0.0001
2002	0.0000	0.0000	0.0008	0.0000		0.0000	0.0000	0.0000	0.0000	0.0000	0.0000
2011		0.0004	0.0019	0.0025	0.0000	0.0008	0.0021	0.0000		0.0000	0.0000
2012	0.0002	0.0000	0.0005	0.0131	0.0000	0.0006	0.0000	0.0000	0.0000	0.0000	0.0001
2013	0.0004	0.0000	0.0092	0.0327	0.0000	0.0007	0.0002	0.0243	0.0347	0.0000	0.0006
2501	0.0051	0.0000	0.0077	0.0247	0.2399	0.7157	0.1230	0.0058	0.1636	0.0084	0.3218
2502	0.0000	0.0000	0.0001	0.0000	0.0020	0.0000	0.0000	0.0000	0.0001	0.0035	0.0000
2507	0.0000	0.0000	0.0001	0.0000		0.0000	0.0000	0.0000	0.0000	0.0040	0.0000
2508	0.0029	0.0173	0.2196	1.8093	0.0054	0.1501	0.0027	0.0000	0.0150	0.0001	0.0140
2509	0.0102	0.0094	0.0430	0.2153	0.0038	0.1089	0.0036	0.0449	0.0020	0.0001	0.0096
3004	0.0006	0.0163	0.0501	0.9061	0.0127	0.6067	0.0023	0.0019	0.0140	0.0014	0.0057
3005	0.0000	0.0223	0.0230	13.0086	0.0053	0.0600	0.2303	0.0000	0.0049	0.0014	0.0023
3009	0.0050	0.0337	0.5279	10.7340	0.0028	0.3604	0.0337	0.0000	0.0074	0.0005	0.0185
3010	0.0019	0.0074	0.0103	2.0037	0.0045	0.0305	0.0755	0.0133	0.0291	0.0004	0.0003
3011	0.0024	0.0022	0.1533	4.1827	0.0191	0.1711	0.0134	0.0000	0.0024	0.0001	0.0029
3504	0.0206	0.0079	0.0601	7.9482	0.0052	0.1600	0.0487	0.0000	0.0005	0.0000	0.0005
3505	0.0001	0.0018	0.4549	4.8610	0.6230	0.3513	0.0253	0.0250	0.0089	0.0002	0.0017
3506	0.0687	0.0565	1.4540	6.1582	0.0063	0.0859		0.0018	0.0020	0.0000	0.0024
3514	0.0133	0.1959	0.1265		0.0358	0.6710	0.0370	0.0004	0.0068	0.0000	0.1033
3515	0.0337	0.2926	0.1995	13.4590	0.0027	0.1462	0.0199	0.0001	0.0143	0.0000	0.0412
4003	0.1168	1.3073	1.9045	6.7040	0.0158	0.2615	0.1614	0.0000	0.0245	0.0000	0.0891
4004	0.0223	0.0669	2.2856	38.2787	0.0068	1.0419	0.0821	0.0002	0.0265	0.0000	0.1066
4005	0.1505	0.1640	1.3239	3.3574	2.5451	0.2730	1.6705	0.0182	0.1943		1.0095

4012	0.4539	0.6038	5.0538	60.9372	0.0926	1.5530	0.4036	0.0023	1.1368	0.7944	1.1294
4013	0.4964	0.3606	1.9695	2.9830	0.0401	0.1941	0.8756	0.0007	0.7876	1.8052	0.3259
4505	0.6379	0.9857	2.8639	74.2034		0.1513	0.6294	0.0118	0.2873	0.9046	0.3752
4506	0.5355	0.8989	2.7503	19.5864	0.3681	1.2033	0.8870	0.0012	2.8247	0.6143	0.3177
4507	0.7221	2.8023	1.6930	56.2453	0.0343	0.3786	0.4110	0.0038	1.0469	0.2080	0.0844
4511	1.0126	0.6018	3.7987	50.4517	0.0095	1.2170	0.3558	0.0030	0.6286	0.2499	0.4212
4512	1.4574	0.5641	5.1745	13.0433	0.0626	1.6012		0.0025	0.5592	0.0627	0.0887

Appendix 17

Table 4. Raw data of immune response: Spot# in ELISpot plate

ID	AAV9					AP4M1					Negative control	Positive control
1003	0	0	0	0	0	0	0	0	0	0	1	381
1004	0	0	0	0	0	0	0	0	2	0	0	491
1008	0	0	0	0	0	0	0	0	1	0	0	459
1009	2	1	0	0	1	0	0	0	0	0	0	526
1010	0	0	0	0	0	0	0	0	0	0	0	96
1508											0	56
1509	0	0	0	1	0	0	0	1	0	1	0	258
1513	0	1	0	0	0	0	0	0	0	0	0	175
1514	0	0	0	0	0	0	0	1	0	0	0	156
1515	0	0	0	0	0	0	0	0	0	0	0	117
2001	0	0	0	0	0	2	10	2	3	0	0	267
2002	0	0	0	0	0	0	0	0	0	0	0	184
2011	0	1	0	1	0	1	1	5	3	2	0	58
2012	0	0	0	0	0	0	1	0	0	0	0	56
2013											0	94
2501	0	0	0	0	0	0	0	0	0	0	0	148
2502	0	0	0	0	0	0	0	0	0	0	0	603
2507	0	0	0	0	0	0	0	0	0	0	0	609
2508	0	0	0	0	0	0	0	0	0	0	0	564
2509	0	1	0	0	0	0	0	0	0	0	0	694
3004	0	0	0	0	0	23	16	4	11	12		
3005	0	0	0	0	0	0	0	0	0	0		
3009	0	0	0	0	0	3	0	3	0	1		
3010	0	0	0	0	0	14	0	2	7	4		
3011	0	0	0	0	0	0	0	0	0	0		
3504	0	0	0	0	0	0	0	0	0	0		
3505	0	0	0	0	0	0	0	0	0	0		
3506	0	0	1	0	0	0	0	0	0	0		
3514	0	0	0	0	0	0	0	0	0	0		
3615												
4003	0	0	2	0	0	0	1	1	0	0		
4004	0	0	0	0	0	0	0	0	0	0		
4005	0	0	0	0	0	0	0	0	0	0		
4012	0	0	0	0	0	0	0	0	0	0		
4013	0	0	0	0	0	2	2	1	1	1		
4505	0	0	0	0	0	0	1	1	0	0		
4506	0	0	0	0	0	0	0	0	0	0		
4507	0	0	0	0	0	1	0	1	0	0		
4511	6	3	6	1	0	2	1	0	4	2		
4512	0	0	0	0	0	0	0	0	0	0		

7.2 Test Article Certificate of Analysis

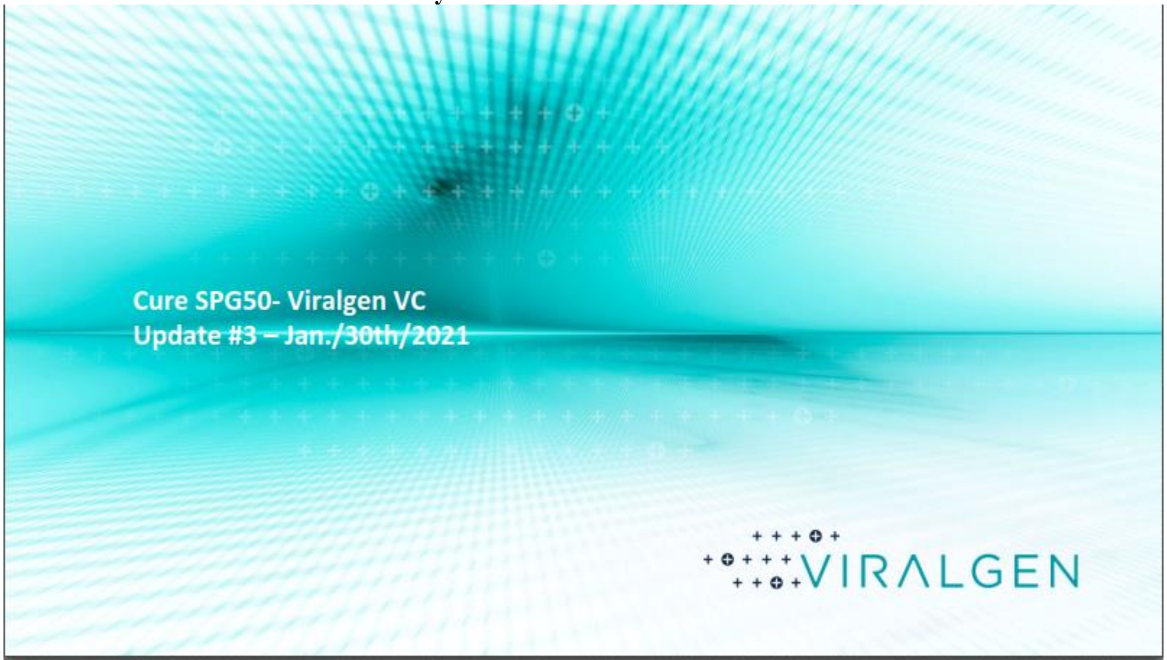

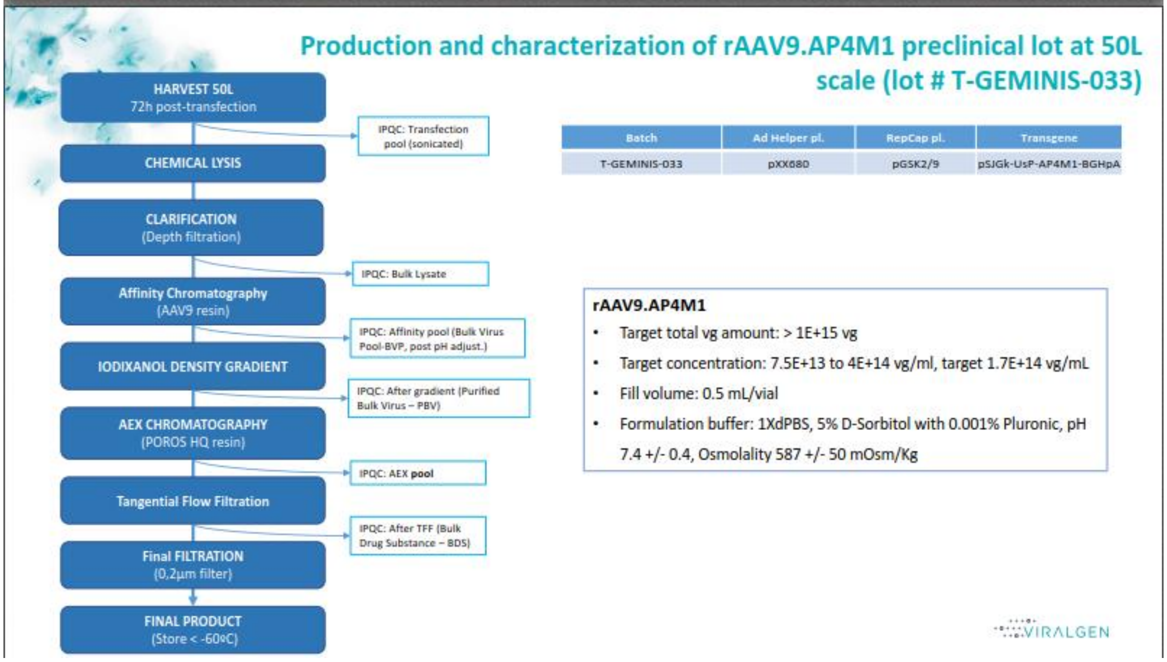

Production and characterization of rAAV9.AP4M1 preclinical lot at 50L scale (lot # T-GEMINIS-033)

	T-GEMINIS-033		
	vg/mL	Volume (mL)	Total vg
Transfection Pool (TP)	9,46E+11	50000	4,73E+16
Bulk lysate (BL)	2,51E+11	57789	1,45E+16
**Clarification yield from TP**		**31%**	
Affinity load ratio (vg/mL resin)		2,90E+13	
NRF looses		<LOQ	
Bulk Virus Pool (BVP)	2,67E+13	468,6	1,25E+16
**Affinity Chromatography yield**		**86%**	
Final Purified Bulk Virus (PBV)	4,97E+13	333	1,66E+16
**Ultracentrifugation yield (final PBV)**		**132%**	
AEX load ratio (vg/mL resin)		1,08E+14	
NRF looses		5%	
AEX pool	2,21E+14	59,8	1,32E+16
**AEX Chromatography yield**		**80%**	
Post-TFF	1,71E+14	42,9	7,34E+15
**TFF yield**		**56%**	
BDS (after sampling)	1,71E+14	35	5,99E+15
Final Product	2,17E+14	34,5	7,49E+15

VIRALGEN

Production and characterization of rAAV9.AP4M1 preclinical lot at 50L scale (lot # T-GEMINIS-033)

SDS-PAGE Silver Stain (ITR qPCR titer)

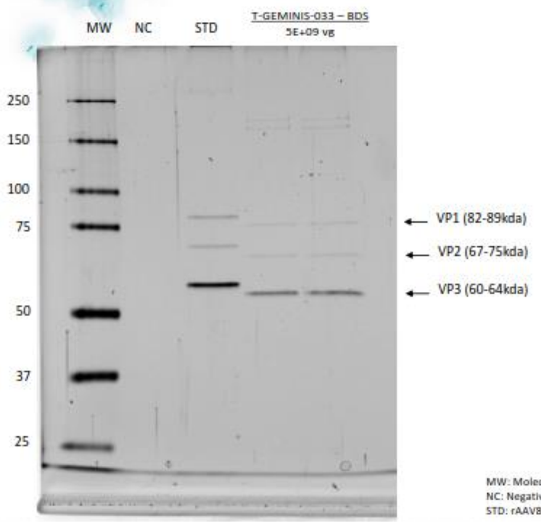

SDS-PAGE Western blot (ITR qPCR titer)

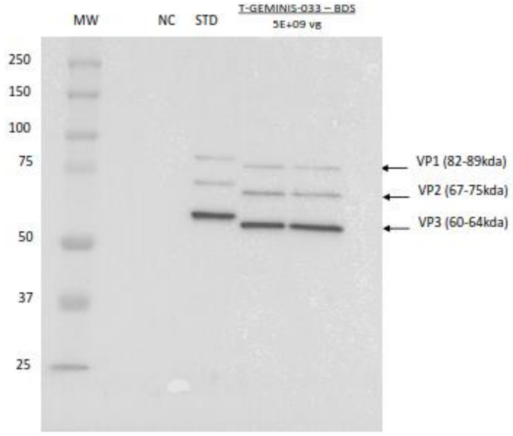

MW: Molecular weight marker
NC: Negative control
STD: rAAV8 (VR-1816) 5E+9vg

VIRALGEN

Appendix 17

Production and characterization of rAAV9.AP4M1 preclinical lot at 50L scale (lot # T-GEMINIS-033) – Drug substance

Test	Method	Specification	Result
**STRENGTH ASSAYS**			
Vector genome titer (vg/mL)	EP 2.6.21; USP<1127> ITR2 qPCR	> TBD vg/mL	1,71E+14
Vector genome titer (vg/mL)	EP 2.6.21; USP<1127> ITR2 ddPCR	> TBD vg/mL	5,17E+13
**PURITY ASSAYS**			
General purity	EP2.2.31; USP<1056><br>SDS-PAGE / Silver staining	Report result	Detection of VP1,VP2 and VP3. Additional extra bands at 150-200 Kda
Residual HCP	ELISA HEK293	Report result	< 100 ng/mL
Residual Host Cell DNA (pg / mL)	EP 2.6.21; USP<1127><br>qPCR 18S (2 amplicon sizes)	Report result	123bp<br>Dnase + :6,86E+06<br>Dnase - : 7,38E+06<br>254bp<br>Dnase +: 5,02E+06<br>Dnase - : 5,21E+06
Residual Host Cell DNA (pg/mL)	EP 2.6.21; USP<1127><br>qPCR E1A	Report result	On-going
Residual Plasmid DNA (copies/mL)	EP 2.6.21; USP<1127><br>qPCR Antibiotic-R	Report result	Dnase + : 9,61E+11<br>Dnase - : 1,26E+12
Full/Empty particles ratio	NS/Cryo-TEM	>50% full	On-going

VIRALGEN

Production and characterization of rAAV9.AP4M1 preclinical lot at 50L scale (lot # T-GEMINIS-033) – Drug substance

Test	Method	Specification	Result
**PURITY ASSAYS**			
Aggregation	nsTEM	Report result	On-going
Residual cells lysis reagent	HPLC	Report result	<LOD (1 ppm)
Residual clarifying reagent	HPLC	Report result	>0.21 ppm and <1.03ppm
Residual transfection reagent	HPLC	Report result	>12.57 ppm and <25.13 ppm
Residual Iodixanol	HPLC	Report result	>1.04 ppm and <3.12 ppm
Residual antifoam	ICP-OES	Report result	<LOD (5 ppm)
Residual Immunoaffinity ligand	ELISA	Report result	497.58 ng/mL
rcAAV	Infection of permissive cell line/qPCR	Report result	On-going
**IDENTITY ASSAYS**			
Protein Identity	SDS-PAGE/Western Blot	Detection of VP1, VP2 and VP3	Detection of VP1,VP2 and VP3
Genome identity	Sequencing	100% conform to sequence of reference	On-going

VIRALGEN

Production and characterization of rAAV9.AP4M1 preclinical lot at 50L scale (lot # T-GEMINIS-033) – Drug product

Test	Method	Specification	Result
**SAFETY ASSAYS**			
Sterility	EP 2.6.1; USP<71>	No growth	No growth
Endotoxin (LAL kinetic chromogenic assay)	EP 2.6.14; USP<85>	< 0.2 EU / mL	0,123 EU/mL
**STRENGTH ASSAYS**			
Vector genome titer (vg/mL)	EP 2.6.21; USP<1127> ITR2 qPCR	7,5E13 to 4E14 vg/mL, targeting 1.7E14 vg/mL	2,17E+14
Vector genome titer (vg/mL)	EP 2.6.21; USP<1127> ITR2 ddPCR	.Report result	5,43E+13
Infectious particles titer (TCID50/mL)	TCID50	Report result	1,02E+10
VG/IP ratio	-	-	2,1E+04
TEA/BAA	CURE SPG50	To be reported by CURE SPG50	TBD
**QUALITY ASSAYS**			
Osmolality (mOsm/Kg)	EP 2.2.35; USP<785>	587 +/-50 mOsm/Kg	570,33
pH	EP 2.2.3; USP<791>	7.4±0.4	7,21
Appearance	Visual inspection	Colorless, clear to slightly opalescent, free of visible particles	Not done – DP vialled in opaque PP vials
Particle size distribution	DLS	Report result	On-going

VIRALGEN

Production and characterization of rAAV9.AP4M1 preclinical lot at 50L scale (lot # T-GEMINIS-033)

Final filling T-GEMINIS-033 -> December 22nd, 2020

- **Fill volume:** **nominal volume 0.5 mL/vial -> actual 0.65 mL/vial**
- **Filled vials:**
  - **69 x 0.5 mL**
  - **1 x 0.04 mL**
- **Shipment to UTSW on January 19th, 2021**

FINAL PRODUCT - Sampling plan	Actual vials
DP characterization	2 x 0.5 mL
Sterility	2 x 0.5 mL
Retention	2 x 0.5 mL
Reference for GMP lot	2x 0.5 mL
Stability study	27 x 0.5 mL
**TOTAL QC**	**35 x 0.5 mL**
**Final amount of vials available for CURE SPG50**	**34 x 0.5 mL**

VIRALGEN

FINAL REPORT

Study Phase: Pathology

Test Facility Study No. 5550008

Sponsor Reference No. UTSW.Gray-003

TEST FACILITY:
Charles River Laboratories Montreal ULC
Senneville Site (CR-SEN)
22022 Transcanadienne
Senneville, QC
Canada

TEST SITE:
Charles River Laboratories, Inc.
15 Worman's Mill Court, Suite I
Frederick, MD 21701
USA

TABLE OF CONTENTS

LIST OF TABLES 3
LIST OF APPENDICES 4
QUALITY ASSURANCE STATEMENT 5
1. SUMMARY 7
2. RESPONSIBLE PERSONNEL 9
3. INTRODUCTION 9
4. MATERIALS AND METHODS 9
4.1. Computerized Systems 10
4.2. Disposition of Study Materials 10
5. RESULTS AND DISCUSSIONS 11
5.1. Mortality 11
5.2. Gross Pathology 11
5.2.1. Terminal Euthanasia Animals (Day 8) 11
5.2.2. Recovery Euthanasia Animals (Day 29) 11
5.2.3. Recovery Euthanasia Animals (Day 91) 11
5.3. Organ Weights 12
5.3.1. Terminal Euthanasia Animals (Day 8) 12
5.3.2. Recovery Euthanasia Animals (Day 29) 12
5.3.3. Recovery Euthanasia Animals (Day 91) 12
5.4. Histopathology 12
5.4.1. Terminal Euthanasia Animals (Day 8) 12
5.4.2. Recovery Euthanasia Animals (Day 29) 14
5.4.3. Recovery Euthanasia Animals (Day 91) 16
6. CONCLUSIONS 23

LIST OF TABLES

Table 1 Summary of Macroscopic Pathology: Terminal Euthanasia....................................25
Table 2 Summary of Organ Weights: Terminal Euthanasia - Absolute...............................52
Table 3 Summary of Organ Weights: Terminal Euthanasia - Relative to BW 65
Table 4 Summary of Organ Weights: Terminal Euthanasia - Relative to Brain Wt...........78
Table 5 Summary of Microscopic Pathology: Terminal Euthanasia 91

LIST OF APPENDICES

Appendix 1 Individual Organ Weights: Terminal Euthanasia - Absolute 117
Appendix 2 Individual Organ Weights: Terminal Euthanasia - Relative to BW.....................166
Appendix 3 Individual Organ Weights: Terminal Euthanasia - Relative to Brain Wt. 215
Appendix 4 Individual Macroscopic and Microscopic Pathology..264

Appendix 18

QUALITY ASSURANCE STATEMENT

Study Number: 5550008

This phase has been audited by Quality Assurance in accordance with the applicable Good Laboratory Practice regulations. Reports were submitted in accordance with standard operating procedures as follows:

QA INSPECTION DATES

		Dates Findings Submitted to:			
**Date(s) of Audit**	**Phase(s) Audited**	**Principal Investigator**	**Test Site Management**	**Study Director**	**Testing Facility Management**
12-Jul-2021 - 13-Jul-2021 15-Jul-2021	Phase Report - Pathology	15-Jul-2021	15-Jul-2021	15-Jul-2021	15-Jul-2021
28-Jul-2021	Phase Report - Pathology	28-Jul-2021	28-Jul-2021	28-Jul-2021	28-Jul-2021
12-Jan-2022	Final Phase Report - Pathology	12-Jan-2022	12-Jan-2022	12-Jan-2022	12-Jan-2022

In addition to the above-mentioned audits, process-based and/or routine facility inspections were also conducting during the course of this phase. Inspection findings, if any specific to this phase were reported by Quality Assurance to the Principal Investigator, Test Site Management, Study Director and Testing Facility Management and listed as a Phase audit on this Quality Assurance statement.

The Final Phase Report has been reviewed to assure that it accurately describes the materials and methods, and that the reported results accurately reflect the raw data.

All electronic signatures appear at the end of the document upon finalization.

Joshua Mendez Santos
Quality Assurance Auditor

Appendix 18

COMPLIANCE STATEMENT AND REPORT APPROVAL

The pathology phase of this study conducted in the USA was performed in accordance with the U.S. Department of Health and Human Services, Food and Drug Administration, United States Code of Federal Regulations, Title 21, Part 58: Good Laboratory Practice for Nonclinical Laboratory Studies and as accepted by Regulatory Authorities throughout the European Union (OECD Principles of Good Laboratory Practice), Japan (MHLW), and other countries that are signatories to the OECD Mutual Acceptance of Data Agreement.

This phase of the study was conducted in accordance with the procedures described herein. All deviations (if any) authorized/acknowledged by the Study Director are documented in the Study Records. The report represents an accurate and complete record of the results obtained for this study phase. Although photomicrographs are included as part of this report, the histopathologic findings contained herein were obtained using the original histologic preparations, not the photos.

There were no deviations from the above regulations that affected the overall integrity of this study phase or the interpretation of the phase results and conclusions.

All electronic signatures appear at the end of the document upon finalization.

Maureen T. O'Brien, DVM, MS, DACVP
Study Pathologist

1. SUMMARY

The objective of this study was to characterize the toxicity, biodistribution, and gene expression of AAV9/AP4M1, for the treatment of Spastic Paraplegia Type 50 (SPG50) caused by the AP4M1 gene mutation, when given at 0, 3.6E11, 1.1E12, or 3.3E12 vg/dose by a single intrathecal injection to male and female rats and to evaluate the potential reversibility and/or progression of any findings.

There were no definitive AAV9/AP4M1-related organ weight changes at any time point (Day 8, Day 29, Day 91). A significant decrease in absolute and relative uterine weights was observed on Day 29 in females receiving ≥ 3.6E11 vg/dose but was considered unrelated to administration of AAV9/AP4M1 due to the absence of microscopic correlate(s) and the lack of corresponding weight changes on Day 8 and Day 91. There were no AAV9/AP4M1-related gross findings at necropsy at any time point (Day 8, Day 29, Day 91), but AAV9/AP4M1-related microscopic findings occurred at all time points. Microscopic findings across all time points are also summarized in Text Table 6 within the narrative. For all time points, the most affected/consistently affected anatomic structures/regions included the lumbar dorsal nerve roots, lumbar dorsal root ganglion, cauda equina in the injection site region, and peripheral nerves (sciatic/tibial nerves). Incidence/severity of most microscopic findings was generally lowest on Day 8, and incidence/severity of most microscopic findings increased from Day 8 to Day 29, followed by a decrease in incidence/severity for most, but not all findings, from Day 29 to Day 91.

AAV9/AP4M1-related findings on Day 8 generally had the lowest incidence and/or magnitude of all time points and occurred in the lumbar dorsal root ganglia, lumbar dorsal nerve roots, and sciatic and tibial nerves. AAV9/AP4M1-related findings in the dorsal root ganglion comprised minimal mononuclear cell infiltration in males and females. In the dorsal lumbar nerve root, minimal axonal degeneration was considered treatment-related in males due to the increased magnitude/severity of the finding at subsequent timepoints, although this finding was also observed in one (1) Control Group (Group 1) male on Day 8. Somewhat dose-dependent AAV9/AP4M1-related increased incidence of minimal peripheral nerve degeneration occurred in males in the tibial and sciatic nerves. There were no AAV9/AP4M1-related findings on Day 8 at the injection site, in the cervical and thoracic nerve roots/dorsal root ganglia, cervical/thoracic spinal cord, or in the ventral nerve roots at any level (cervical, thoracic, lumbar).

AAV9/AP4M1-related findings on Day 29 occurred in the injection site, lumbar dorsal root ganglia, lumbar dorsal nerve roots, thoracic dorsal root ganglion, cervical dorsal root ganglia, sciatic and tibial nerves, and adipose tissue adjacent to the thoracic spinal column. Findings on Day 29 generally had higher incidence and/or severity across all timepoints (exceptions to this observation are listed for Day 91 when present). AAV9/AP4M1-related findings at the injection site on Day 29 included treatment-related increased incidence and/or severity of minimal-to mild leptomeningeal mononuclear cell infiltration in males and females and dose-dependent increased incidence and/or severity of minimal-to moderate cauda equina axonal degeneration in males and females. In the lumbar dorsal root ganglion and lumbar dorsal nerve roots there was generally dose-dependent increased incidence and/or severity of minimal-to-mild (ganglion) or minimal-to-moderate (nerve roots) axonal degeneration in males and females. There was minimal-to-mild

lumbar dorsal root ganglion neuronal degeneration in males and females on Day 29. Additionally, one Group 4 male had AAV9/AP4M1-related minimal mononuclear cell infiltration in lumbar dorsal nerve roots on Day 29. One Group 4 female (4511) had minimal axonal degeneration and/or mononuclear cell infiltration in the cervical and thoracic dorsal root ganglia and one Group 4 female (4507) had mononuclear cell infiltration in the thoracic dorsal root ganglia on Day 29. Generally dose-dependent AAV9/AP4M1-related increased incidence and/or severity of minimal-to-mild peripheral nerve degeneration (sciatic and tibial nerves) occurred in males and females. There was also minimal-to-mild mononuclear cell infiltration within the adipose tissue surrounding the vertebral body that was considered AAV9/AP4M1-related due to incidence in Group 4 males and females only. There were no AAV9/AP4M1-related findings on Day 29 in the cervical/thoracic dorsal nerve roots, or in the cervical spinal cord or ventral nerve roots at any level (cervical, thoracic, lumbar).

AAV9/AP4M1-related findings on Day 91 occurred in the injection site, lumbar dorsal root ganglia, lumbar dorsal nerve roots, sciatic and tibial nerves, and adipose tissue adjacent to the thoracic spinal column. Generally, there was partial or complete recovery of most, but not all, microscopic findings on Day 91 compared to Day 29. AAV9/AP4M1-related findings at the injection site included treatment-related increased incidence of minimal leptomeningeal mononuclear cell infiltration in males and females that had higher incidence in females vs. males. There was also generally dose-dependent increased incidence of minimal axonal degeneration in the cauda equina of males and females. Although incidence of leptomeningeal mononuclear cell infiltration and axonal degeneration in the cauda equina is similar to Day 29, the magnitude (severity) of the finding has decreased, indicating partial recovery of this finding. AAV9/AP4M1-related findings in the lumbar dorsal root ganglion on Day 91 included generally dose-dependent increased incidence and/or severity of minimal-to-mild mononuclear cell infiltration in males and females, with slightly decreased incidence and severity compared to Day 29 indicating partial recovery. There was minimal lumbar dorsal root ganglion neuronal degeneration in males and females, with generally similar incidence to Day 29 indicating no recovery of this finding. Other findings in the lumbar dorsal root ganglion on Day 91 included generally dose-dependent incidence of minimal axonal degeneration in males and females, with slightly decreased incidence and/or severity compared to Day 29 indicating partial recovery of this finding. In the lumbar dorsal nerve roots on Day 91, there was minimal-to-mild axonal degeneration in males and females; while severity has decreased compared to Day 29, incidence has increased to include the lowest dose group (Group 2, where findings were limited to Group 3 and Group 4 on Day 29) indicating equivocal recovery. There was no mononuclear cell infiltration in lumbar dorsal nerve roots on Day 91, indicating complete recovery of this finding. There was dose-dependent AAV9/AP4M1-related increased incidence of minimal peripheral nerve degeneration (sciatic and tibial nerves) that occurred in males and females at ≥ 3.6E11 vg/dose. Although magnitude (severity) has decreased to only minimal, from minimal-to-mild compared to Day 29, incidence has increased to include the lowest dose group (Group 2) indicating equivocal recovery. AAV9/AP4M1-related minimal mononuclear cell infiltration within the adipose tissue surrounding the vertebral body remained with a low incidence in females only on Day 91; the slightly lower incidence and/or magnitude (severity) indicates partial recovery of this finding. There were no AAV9/AP4M1-related findings in the thoracic or cervical dorsal root ganglia, indicating complete recovery of the axonal degeneration and

mononuclear cell infiltration observed on Day 29. There were no AAV9/AP4M1-related findings on Day 91 in the cervical or thoracic dorsal nerve roots, or in the cervical spinal cord or ventral nerve roots at any level (cervical, thoracic, lumbar).

2. RESPONSIBLE PERSONNEL

Study Pathologist, Histopathology	Maureen T. O'Brien, DVM, MS, DACVP<br>Charles River Laboratories, Inc.<br>Frederick, Maryland
Test Site Management	James T. Raymond, DVM, MS, DACVP, DABT<br>Charles River Laboratories, Inc.<br>Frederick, Maryland

3. INTRODUCTION

This report presents the pathology findings in rats assigned to Study No. 5550008. The objective of this study was to characterize the toxicity, biodistribution, and gene expression of AAV9/AP4M1, for the treatment of Spastic Paraplegia Type 50 (SPG50) caused by the AP4M1 gene mutation, when given by a single intrathecal injection to rats and evaluated the potential reversibility and/or progression of any findings.

The study was sponsored by University of Texas Southwestern Medical Center, Dallas, TX. Stefania Cinquino, BSc, served as the Study Director.

4. MATERIALS AND METHODS

Experimental procedures applicable to pathology investigations are summarized in Text Table 1.

Text Table 1
Experimental Design

Group No.	Test Material	Dose Level (vg)	Dose Volume (µL)	Dose Concentration (vg/µL)	No. of Animals					
					Main Study		Recovery Study			
					Day 8 Necropsy[a]		Day 29 Necropsy[b]		Day 91 Necropsy[c]	
					M	F	M	F	M	F
1	Reference Item	0	60	0	5	5	5	5	5	5
2	AAV9/AP4M1	3.6E11	20	1.8E10	5	5	5	5	5	5
3	AAV9/AP4M1	1.1E12	20	5.5E10	5	5	5	5	5	5
4	AAV9/AP4M1	3.3E12	60	5.5E10	5	5	5	5	5	5

M = Males; F = Females
[a] Animals scheduled for Necropsy on Day 8.
[b] Animals scheduled for Necropsy on Day 29.
[c] Animals scheduled for Necropsy on Day 91.

Animals were submitted for necropsy on Day 8 (main study animals) or Day 29 or 91 (recovery animals). Necropsies were performed and organs were collected by Testing Facility personnel. Tissues required for microscopic evaluation were trimmed, processed routinely, embedded in paraffin, and stained with hematoxylin and eosin. Microscopic evaluation was conducted by the Principal Investigator, a board-certified veterinary pathologist on all protocol-specified tissues from all animals

Tissues that were supposed to be microscopically evaluated per study plan but were not available on the slide (and therefore not evaluated) are listed in the Individual Animal Data of the pathology report as not present. These missing tissues did/not affect the outcome or interpretation of the pathology portion of the study because changes were not present in tissues evaluated in other animals and sufficient tissues were available for evaluation.

4.1. Computerized Systems

Critical computerized systems used in this study phase are listed in Text Table 2.

Text Table 2
Computerized Systems

System Name	Version No.	Description of Data Collected and/or Analyzed
Provantis®	10	Histopathology
M-Files®	21	Reporting and collection of 21 CFR Part 11 compliant signature
Share Document Management System	1	Reporting

4.2. Disposition of Study Materials

All study specific raw data, pathology materials, documentation and Final Report generated from this study phase are to be sent to the Testing Facility for archiving. Study materials will be

retained for a period of 1 year following issue of the audited Draft Report. Electronic Provantis data generated by the Test Site will be archived, and the software and hardware required to produce it in a readable form will be maintained and available. The report files stored on the SHARE Document Management System (SDMS) will be archived in Charles River Laboratories, Inc., Wilmington, MA.

5. RESULTS AND DISCUSSIONS

5.1. Mortality

There was one unscheduled euthanasia on Day 7: a Group 1 male (No. 1013). Gross findings at necropsy included abnormal dark and/or firm content in the jejunum, colon, and cecum, a small spleen, and small dark foci on the glandular stomach mucosa. There were no microscopic correlates for gross observations, microscopic findings were nonspecific, and the cause of clinical moribundity was undetermined but is unrelated to administration of AAV9/AP4M1 as this is a Control Group (Group 1) animal.

5.2. Gross Pathology

5.2.1. Terminal Euthanasia Animals (Day 8)

(Table 1 and Appendix 1)

No AAV9/AP4M1-related gross findings were noted. The gross findings observed were considered incidental, of the nature commonly observed in this strain and age of rats, and/or were of similar incidence in control and treated animals and, therefore, were considered unrelated to administration of AAV9/AP4M1.

5.2.2. Recovery Euthanasia Animals (Day 29)

(Table 1 and Appendix 1)

No AAV9/AP4M1-related gross findings were noted. The gross findings observed were considered incidental, of the nature commonly observed in this strain and age of rats and/or were of similar incidence in control and treated animals and, therefore, were considered unrelated to administration of AAV9/AP4M1.

5.2.3. Recovery Euthanasia Animals (Day 91)

(Table 1 and Appendix 1)

No AAV9/AP4M1-related gross findings were noted. The gross findings observed were considered incidental, of the nature commonly observed in this strain and age of rats and/or were of similar incidence in control and treated animals and, therefore, were considered unrelated to administration of AAV9/AP4M1.

5.3. Organ Weights

5.3.1. Terminal Euthanasia Animals (Day 8)

(Table 2, Table 3, Table 4, Appendix 1, Appendix 2, Appendix 3)

No AAV9/AP4M1-related organ weight changes were noted. There were isolated organ weight values that were statistically different from their respective controls. There were, however, no patterns, trends, or correlating data to suggest these values were toxicologically relevant. Thus, the organ weight differences observed were considered incidental and/or related to difference of sexual maturity and unrelated to administration of AAV9/AP4M1.

5.3.2. Recovery Euthanasia Animals (Day 29)

(Table 2, Table 3, Table 4, Appendix 1, Appendix 2, Appendix 3)

No AAV9/AP4M1-related organ weight changes were noted. A significant decrease in absolute and relative uterine weights was observed on Day 29 in females receiving ≥ 3.6E11 vg/dose but was considered unrelated to administration of AAV9/AP4M1 due to the absence of microscopic correlate(s) and the lack of corresponding weight changes on Day 8 and Day 91. There were other isolated organ weight values that were statistically different from their respective controls. There were, however, no patterns, trends, or correlating data to suggest these values were toxicologically relevant. Thus, the organ weight differences observed were considered incidental and/or related to difference of sexual maturity and unrelated to administration of AAV9/AP4M1.

5.3.3. Recovery Euthanasia Animals (Day 91)

(Table 2, Table 3, Table 4, Appendix 1, Appendix 2, Appendix 3)

No AAV9/AP4M1-related organ weight changes were noted. There were isolated organ weight values that were statistically different from their respective controls. There were, however, no patterns, trends, or correlating data to suggest these values were toxicologically relevant. Thus, the organ weight differences observed were considered incidental and/or related to difference of sexual maturity and unrelated to administration of AAV9/AP4M1.

5.4. Histopathology

5.4.1. Terminal Euthanasia Animals (Day 8)

(Table 5 and Appendix 4)

AAV9/AP4M1-related microscopic findings are summarized in Text Table 3. AAV9/AP4M1-related findings across all time points is provided in Text Table 6 in section 5.4.3. Diagnostic criteria used for AAV9/AP4M1-related microscopic findings for all time points are summarized in Text Table 7.

Text Table 3
Summary of Microscopic Findings – Scheduled Euthanasia (Day 8)

	Males				Females			
**Group**	**1**	**2**	**3**	**4**	**1**	**2**	**3**	**4**
**Dose Level (vg)**	**0**	**3.6E11**	**1.1E12**	**3.3E12**	**0**	**3.6E11**	**1.1E12**	**3.3E12**
**No. Animals per Group**	**4**	**5**	**5**	**5**	**5**	**5**	**5**	**5**
Lumbar Region:								
**Ganglion, Dorsal Root, Lumbar (No. Examined)**	4	5	5	5	5	5	5	5
Infiltration, mononuclear cell	(0)	(0)	(2)	(5)	(0)	(1)	(1)	(3)
Minimal	0	0	2	5	0	1	1	3
**Nerve Root, Dorsal, Lumbar (No. Examined)**	4	5	5	5	5	5	5	5
Degeneration, axonal	(1)	(0)	(2)	(1)	(0)	(0)	(0)	(0)
Minimal	1	0	2	1	0	0	0	0
**Nerve, Sciatic (No. Examined)** [a]	4	5	5	5	5	5	5	5
Degeneration, axonal	(0)	(0)	(1)	(1)	(0)	(0)	(0)	(1)
Minimal	0	0	1	1	0	0	0	1
**Nerve, Tibia (No. Examined)**	4	5	5	5	5	5	5	5
Degeneration, axonal	(0)	(1)	(2)	(2)	(0)	(0)	(0)	(1)
Minimal	0	1	2	2	0	0	0	1

[a] Numbers in parentheses represent the number of animals with the finding.

AAV9/AP4M1-related findings on Day 8 occurred in the lumbar dorsal root ganglia, lumbar dorsal nerve roots, and sciatic and tibial nerves. Treatment-related findings in the lumbar dorsal root ganglion included minimal mononuclear cell infiltration in males and females at ≥ 1.1E12/3.6E11 vg/dose. In the dorsal lumbar nerve root, minimal axonal degeneration was considered treatment-related in males at ≥ 1.1E12 vg/dose due to the increased magnitude/severity of the finding at subsequent timepoints, although this finding was also observed in one (1) Control Group (Group 1) male on Day 8. Somewhat dose-dependent AAV9/AP4M1-related increased incidence of minimal peripheral nerve degeneration occurred in males and females at ≥ 1.1/3.3E12 vg/dose in the sciatic nerve and ≥ 3.6E11/3.3E12 vg/dose in the tibial nerve.

Generally, microscopic findings at the injection site on Day 8 in all Groups included minimal axonal degeneration and/or minimal mononuclear cell infiltration within the leptomeninges. As these findings occurred with similar incidence and/or severity in both control and treated animals on Day 8 they were considered unrelated to administration of AAV9/AP4M1. There were no AAV9/AP4M1-related findings on Day 8 at the injection site, in the cervical and thoracic nerve roots/dorsal root ganglia, or in the ventral nerve roots at any level (cervical, thoracic, lumbar).

Other microscopic findings observed were considered incidental, of the nature commonly observed in this strain and age of rats, and/or were of similar incidence and severity in control

and treated animals and, therefore, were considered unrelated to administration of AAV9/AP4M1.

5.4.2. Recovery Euthanasia Animals (Day 29)

(Table 5 and Appendix 4)

Microscopic findings noted at the terminal euthanasia were observed at the end of the recovery period (Day 29) and are summarized in Text Table 4.

Text Table 4
Summary of Microscopic Findings – Scheduled Euthanasia (Day 29)

	**Males**				**Females**			
**Group**	**1**	**2**	**3**	**4**	**1**	**2**	**3**	**4**
**Dose Level (vg)**	**0**	**3.6E11**	**1.1E12**	**3.3E12**	**0**	**3.6E11**	**1.1E12**	**3.3E12**
**No. Animals per Group**	**5**	**5**	**5**	**5**	**5**	**5**	**5**	**5**
**Injection Site (No. Examined)**[a]	5	5	5	5	5	5	5	5
Degeneration, axonal, cauda equina	(0)	(2)	(3)	(4)	(0)	(0)	(3)	(5)
Minimal	0	2	3	2	0	0	2	0
Mild	0	0	0	2	0	0	1	3
Moderate	0	0	0	0	0	0	0	2
Infiltration, mononuclear cell, leptomeninges	(0)	(2)	(1)	(4)	(1)	(4)	(2)	(4)
Minimal	0	2	1	3	1	4	2	4
Mild	0	0	0	1	0	0	0	0
Lumbar region:								
**Ganglion, Dorsal Root, Lumbar (No. Examined)**	5	5	5	5	5	5	5	5
Degeneration, axonal	(0)	(0)	(2)	(4)	(0)	(0)	(2)	(5)
Minimal	0	0	2	1	0	0	2	4
Mild	0	0	0	3	0	0	0	1
Degeneration, neuronal	(0)	(0)	(3)	(3)	(0)	(0)	(1)	(3)
Minimal	0	0	3	2	0	0	1	3
Mild	0	0	0	1	0	0	0	0
Infiltration, mononuclear cell	(0)	(0)	(4)	(4)	(0)	(0)	(3)	(5)
Minimal	0	0	4	3	0	0	2	3
Mild	0	0	0	1	0	0	1	2
**Nerve Root, Dorsal, Lumbar (No. Examined)**	5	5	5	5	5	5	5	5
Degeneration, axonal	(0)	(1)	(4)	(4)	(0)	(0)	(4)	(5)
Minimal	0	1	3	1	0	0	3	1
Mild	0	0	1	3	0	0	1	3
Moderate	0	0	0	0	0	0	0	1
Thoracic region:								
**Ganglion, Dorsal Root, Thoracic (No. Examined)**	5	5	5	5	5	5	5	5
Degeneration, axonal	(0)	(0)	(0)	(0)	(0)	(0)	(0)	(1)
Minimal	0	0	0	0	0	0	0	1

	Males				Females			
**Group**	**1**	**2**	**3**	**4**	**1**	**2**	**3**	**4**
**Dose Level (vg)**	**0**	**3.6E11**	**1.1E12**	**3.3E12**	**0**	**3.6E11**	**1.1E12**	**3.3E12**
**No. Animals per Group**	**5**	**5**	**5**	**5**	**5**	**5**	**5**	**5**
Infiltration, mononuclear cell	(0)	(0)	(0)	(0)	(0)	(0)	(0)	(1)
Minimal	0	0	0	0	0	0	0	1
**Spinal Cord, Thoracic (No. Examined)**	5	5	5	5	5	5	5	5
Infiltration, mononuclear cell, adipose tissue	(0)	(0)	(0)	(1)	(0)	(1)	(0)	(2)
Minimal	0	0	0	0	0	1	0	2
Mild	0	0	0	1	0	0	0	0
Cervical region:								
**Ganglion, Dorsal Root, Cervical (No. Examined)**	5	5	5	5	5	5	5	5
Degeneration, axonal	(0)	(0)	(0)	(0)	(0)	(0)	(0)	(1)
Minimal	0	0	0	0	0	0	0	1
Infiltration, mononuclear cell	(0)	(0)	(0)	(0)	(0)	(0)	(0)	(1)
Minimal	0	0	0	0	0	0	0	1
**Nerve, Sciatic (No. Examined)**	5	5	5	5	5	5	5	5
Degeneration, axonal	(0)	(0)	(5)	(5)	(0)	(0)	(5)	(5)
Minimal	0	0	4	4	0	0	5	2
Mild	0	0	1	1	0	0	0	3
**Nerve, Tibia (No. Examined)**	5	5	5	5	5	5	5	5
Degeneration, axonal	(0)	(0)	(4)	(4)	(0)	(0)	(4)	(5)
Minimal	0	0	3	3	0	0	4	3
Mild	0	0	1	1	0	0	0	2

[a] Numbers in parentheses represent the number of animals with the finding.

AAV9/AP4M1-related findings on Day 29 occurred in the injection site, lumbar dorsal root ganglia, lumbar dorsal nerve roots, thoracic dorsal root ganglion, cervical dorsal root ganglia, sciatic and tibial nerves, and adipose tissue adjacent to the thoracic spinal column. Findings are generally most severe (highest magnitude/severity grades) on Day 29 compared to Day 8 and Day 91. AAV9/AP4M1-related findings at the injection site included treatment-related (i.e. changes related to treatment without dose-dependency) increased incidence and/or severity of minimal-to mild leptomeningeal mononuclear cell infiltration in males and females at ≥ 3.6E11 vg/dose and dose-dependent increased incidence and/or severity of minimal-to moderate axonal degeneration in the cauda equina of males and females at ≥ 3.6E11/1.1E12 vg/dose. AAV9/AP4M1-related findings in the lumbar dorsal root ganglia and lumbar dorsal nerve roots included generally dose-dependent increased incidence and/or severity of minimal-to-mild (ganglion) or minimal-to-moderate (nerve roots) axonal degeneration in males and females at ≥ 3.6E11/1.1E12 vg/dose. There was minimal-to-mild lumbar dorsal root ganglion neuronal degeneration in males and females at ≥ 1.1E12 vg/dose. Additionally, there was AAV9/AP4M1-related, treatment-dependent minimal-to-mild mononuclear cell infiltration in lumbar dorsal root

ganglia in males and females at ≥ 1.1E12 vg/dose and minimal mononuclear cell infiltration in the lumbar dorsal nerve root of one male administered 3.3E12 vg/dose.

AAV9/AP4M1 treatment-related findings in the cervical and thoracic dorsal root ganglion included minimal axonal degeneration and/or mononuclear cell infiltration in one (1) female administered 3.3E12 vg/dose (4511), as well as mononuclear cell infiltration in the thoracic dorsal root ganglion of another female administered 3.3E12 vg/dose (4507).

Generally dose-dependent AAV9/AP4M1-related increased incidence and/or severity of minimal-to-mild peripheral nerve degeneration (sciatic and tibial nerves) occurred in males and females at ≥ 1.1E12 vg/dose. Findings are increased in incidence and magnitude (severity) compared to Day 8.

There was also minimal-to-mild mononuclear cell infiltration within the adipose tissue surrounding the vertebral body that was considered AAV9/AP4M1-related due to low incidence in males and females receiving 3.3E12 vg/dose only.

There were no AAV9/AP4M1-related findings on Day 29 in the cervical or thoracic dorsal nerve roots, or in the cervical spinal cord or ventral nerve roots at any level (cervical, thoracic, lumbar).

Other microscopic findings observed were considered incidental, of the nature commonly observed in this strain and age of rats, and/or were of similar incidence and severity in control and treated animals and, therefore, were considered unrelated to administration of AAV9/AP4M1.

5.4.3. Recovery Euthanasia Animals (Day 91)

(Table 5 and Appendix 4)

Microscopic findings noted at the terminal euthanasia were observed at the end of the recovery period (Day 91) and are summarized in Text Table 5.

Text Table 5
Summary of Microscopic Findings – Scheduled Euthanasia (Day 91)

	**Males**				**Females**			
**Group**	**1**	**2**	**3**	**4**	**1**	**2**	**3**	**4**
**Dose Level (vg)**	**0**	**3.6E11**	**1.1E12**	**3.3E12**	**0**	**3.6E11**	**1.1E12**	**3.3E12**
**No. Animals per Group**	**5**	**5**	**5**	**5**	**5**	**5**	**5**	**5**
**Injection Site (No. Examined)**[a]	5	5	5	5	5	5	5	5
Degeneration, axonal, cauda equina	(0)	(0)	(0)	(4)	(0)	(0)	(3)	(4)
Minimal	0	0	0	4	0	0	3	4
Infiltration, mononuclear cell, leptomeninges	(1)	(1)	(2)	(1)	(1)	(4)	(4)	(4)
Minimal	1	1	2	1	1	4	4	4
Lumbar region:								

	Males				Females			
**Group**	**1**	**2**	**3**	**4**	**1**	**2**	**3**	**4**
**Dose Level (vg)**	**0**	**3.6E11**	**1.1E12**	**3.3E12**	**0**	**3.6E11**	**1.1E12**	**3.3E12**
**No. Animals per Group**	**5**	**5**	**5**	**5**	**5**	**5**	**5**	**5**
**Ganglion, Dorsal Root, Lumbar (No. Examined)**	5	5	5	5	5	5	5	5
Degeneration, axonal	(0)	(0)	(0)	(1)	(0)	(0)	(2)	(1)
Minimal	0	0	0	1	0	0	2	1
Degeneration, neuronal	(0)	(0)	(2)	(3)	(0)	(0)	(1)	(2)
Minimal	0	0	2	3	0	0	1	2
Infiltration, mononuclear cell	(0)	(0)	(2)	(3)	(0)	(1)	(2)	(2)
Minimal	0	0	2	2	0	1	2	2
Mild	0	0	0	1	0	0	0	0
**Nerve Root, Dorsal, Lumbar (No. Examined)**	5	5	5	5	5	5	5	5
Degeneration, axonal	(0)	(1)	(4)	(4)	(0)	(2)	(5)	(5)
Minimal	0	1	4	2	0	2	5	1
Mild	0	0	0	2	0	0	0	4
**Spinal Cord, Lumbar (No. Examined)**	5	5	5	5	5	5	5	5
Infiltration, mononuclear cell, leptomeninges	(0)	(0)	(0)	(0)	(0)	(0)	(2)	(0)
Minimal	0	0	0	0	0	0	2	0
Thoracic region:								
**Spinal Cord, Thoracic (No. Examined)**	5	5	5	5	5	5	5	5
Infiltration, mononuclear cell, adipose tissue	(0)	(0)	(0)	(0)	(0)	(0)	(1)	(2)
Minimal	0	0	0	0	0	0	1	2
**Nerve, Sciatic (No. Examined)**	5	4	5	5	5	5	5	5
Degeneration, axonal	(0)	(2)	(3)	(4)	(0)	(1)	(5)	(5)
Minimal	0	2	3	4	0	1	5	5
**Nerve, Tibia (No. Examined)**	5	5	5	5	5	5	5	5
Degeneration, axonal	(0)	(2)	(2)	(4)	(0)	(2)	(4)	(5)
Minimal	0	2	2	4	0	2	4	5

[a] Numbers in parentheses represent the number of animals with the finding.

AAV9/AP4M1-related findings on Day 91 occurred in the injection site, lumbar dorsal root ganglia, lumbar dorsal nerve roots, sciatic and tibial nerves, and adipose tissue adjacent to the thoracic spinal column. Most, but not all, microscopic findings generally had decreased incidence and/or severity compared to Day 29. Treatment-related (i.e. changes related to treatment without dose-dependency) findings at the injection site included treatment-related increased incidence of minimal leptomeningeal mononuclear cell infiltration in males and females at ≥ 3.6E11 vg/dose that had higher incidence in females vs. males. There was also generally dose-dependent increased incidence of minimal axonal degeneration in the cauda equina of males and females at ≥ 3.3E12/1.1E12 vg/dose. Although incidence of leptomeningeal

mononuclear cell infiltration and axonal degeneration in the cauda equina is similar to Day 29, the magnitude (severity) of the finding has decreased, indicating partial recovery.

AAV9/AP4M1-related findings in the lumbar dorsal root ganglion on Day 91 included generally dose-dependent increased incidence and/or severity of minimal-to-mild mononuclear cell infiltration in males and females at ≥ 1.1E12/3.6E11 vg/dose, with slightly decreased incidence and severity compared to Day 29 indicating partial recovery of this finding. There was minimal lumbar dorsal root ganglion neuronal degeneration in males and females at ≥ 1.1E12 vg/dose, with generally similar incidence to Day 29 indicating no recovery of this finding. AAV9/AP4M1-related findings in the lumbar dorsal root ganglion on Day 91 also included generally dose-dependent incidence of minimal axonal degeneration in males and females at 3.3E12/≥1.1E12 vg/dose, with slightly decreased incidence and severity compared to Day 29 indicating partial recovery of this finding.

In the lumbar dorsal nerve roots, there was minimal-to-mild axonal degeneration in males and females at ≥ 3.6E11 vg/dose; while severity has decreased compared to Day 29, incidence has increased to include the lowest dose group (Group 2, where findings were limited to Group 3 and Group 4 on Day 29) indicating equivocal recovery. There was no mononuclear cell infiltration in lumbar dorsal nerve roots on Day 91, indicating complete recovery of this finding.

On Day 91, there were no AAV9/AP4M1-related findings in the thoracic or cervical dorsal root ganglia, indicating complete recovery of the axonal degeneration and mononuclear cell infiltration observed in a single Group 4 male on Day 29.

There was generally dose-dependent AAV9/AP4M1-related increased incidence of minimal peripheral nerve degeneration (sciatic and tibial nerves) that occurred in males and females at ≥ 3.6E11 vg/dose. Although magnitude (severity) has decreased from minimal-to-mild compared to Day 29, incidence has increased to include the lowest dose group (Group 2) indicating equivocal recovery.

There was also minimal mononuclear cell infiltration within the adipose tissue surrounding the vertebral body that was considered AAV9/AP4M1-related that occurred on Day 91 with low incidence in females only administered ≥ 1.1E12 vg/dose; the slightly lower incidence and/or magnitude (severity) indicates partial recovery of this finding.

There were no AAV9/AP4M1-related findings on Day 91 in the cervical or thoracic dorsal nerve roots, or in the cervical spinal cord or ventral nerve roots at any level (cervical, thoracic, lumbar). Other microscopic findings observed were considered incidental, of the nature commonly observed in this strain and age of rats, and/or were of similar incidence and severity in control and treated animals and, therefore, were considered unrelated to administration of AAV9/AP4M1.

Text Table 6
Summary of AAV9/AP4M1-Related Microscopic Findings Across All Timepoints

Site/*relevant finding*	Day 8	Day 29	Day 91
**Injection site**	No TA-related findings.	TA-related findings present.	TA-related findings present with partial recovery.
Degeneration, axonal, cauda equina	No TA-related findings.	Generally dose-dependent increased incidence and/or severity of minimal-to moderate axonal degeneration in the cauda equina of males and females at ≥ 1.1E12/3.6E11 vg/dose	Partial recovery by Day 91. Somewhat dose-dependent increased incidence of minimal axonal degeneration in the cauda equina of males and females at ≥ 1.1E12/3.3E12 vg/dose. Incidence generally similar to Day 29 but magnitude has decreased from Day 29 (partial recovery).
Infiltration, mononuclear cell, leptomeninges	No TA-related findings.	TA-related treatment related increased incidence and severity in males and females at ≥ 3.6E11 vg/dose.	Partial recovery by Day 91. Treatment-related increased incidence of minimal leptomeningeal mononuclear cell infiltration in males and females at ≥ 3.6E11 vg/dose. Incidence generally similar to Day 29 but magnitude has decreased from Day 29 (partial recovery). Higher incidence in females vs. males.
**Nerve Root, Dorsal, Lumbar**	TA-related findings present.	TA-related findings present; increased incidence/magnitude vs. Day 8.	TA-related findings present.
Degeneration, axonal	Minimal axonal degeneration considered treatment-related in males at ≥ 1.1E12 vg/dose due to increased magnitude/severity of the finding at subsequent timepoints, although this finding also observed in one (1) Control Group (Group 1) male on Day 8.	Increased incidence/magnitude vs. Day 8. Generally dose dependent minimal-to-moderate axonal degeneration in males/females at ≥ 1.1E12/3.6E11 vg/dose	Equivocal recovery of finding. Generally dose dependent minimal-to-mild axonal degeneration in males/females at ≥ 3.6E11 vg/dose. Magnitude/severity has decreased (limited to minimal) but there is higher incidence that includes the lowest dose group (Group 2).
Infiltration, mononuclear cell	No TA-related findings.	Minimal mononuclear cell infiltration in one (1) female at 3.3E12 vg/dose.	Complete recovery of mononuclear cell infiltration on Day 91 in dorsal lumbar nerve root.
**Ganglion, Dorsal Root, Lumbar (No. Examined)**	TA-related findings present.	TA-related findings present; increased incidence/magnitude vs. Day 8.	TA-related findings present with variable recovery.
Infiltration, mononuclear cell	Minimal mononuclear cell infiltration in males and females at ≥ 1.1E12/3.6E11 vg/dose.	Increased incidence/magnitude vs. Day 8. Treatment-related increased incidence and/or severity of minimal-to-mild mononuclear cell infiltration	Partial recovery by Day 91 based on decreased incidence/severity compared to Day 29; however, generally dose-dependent increased incidence and/or

Site/*relevant finding*	Day 8	Day 29	Day 91
		in males/females at ≥ 1.1E12 vg/dose.	magnitude of minimal-to-mild mononuclear cell infiltration in males/females remains at ≥ 1.1E12/≥3.3E12 vg/dose.
Degeneration, axonal	No TA-related findings.	Generally dose dependent minimal-to-moderate axonal degeneration in males/females at ≥ 1.1E12 vg/dose.	Partial recovery by Day 91 based on decreased incidence/severity compared to Day 29; however, treatment-related incidence of minimal axonal degeneration males/females remains at ≥ 3.6E11/≥3.3E12 vg/dose.
Degeneration, neuronal	No TA-related findings.	Minimal-to-mild lumbar dorsal root ganglion neuronal degeneration in males and females at ≥ 1.1E12 vg/dose.	No recovery of this finding by Day 91; incidence/magnitude are generally similar to Day 29 and minimal lumbar dorsal root ganglion neuronal degeneration persisted in males and females at ≥ 1.1E12 vg/dose.
**Ganglion, Dorsal Root, Thoracic (No. Examined)**	No TA-related findings.	TA-related findings present.	Complete recovery of TA-related findings observed on Day 29.
Degeneration, axonal	No TA-related findings.	One Group 4 (4511) female had minimal axonal degeneration on Day 29.	Complete recovery of this finding by Day 91. No TA-related findings on Day 91.
Infiltration, mononuclear cell	No TA-related findings.	One Group 4 (4507) female had minimal mononuclear cell infiltration on Day 29.	Complete recovery of this finding by Day 91. No TA-related findings on Day 91.
**Ganglion, Dorsal Root, Cervical (No. Examined)**	No TA-related findings.	TA-related findings present.	Complete recovery of TA-related findings observed on Day 29.
Degeneration, axonal	No TA-related findings.	One Group 4 female (4511) had minimal axonal degeneration on Day 29.	Complete recovery of this finding by Day 91. No TA-related findings on Day 91.
Infiltration, mononuclear cell	No TA-related findings.	One Group 4 female (4511) had minimal mononuclear cell infiltration on Day 29.	Complete recovery of this finding by Day 91. No TA-related findings on Day 91.
**Spinal Cord, Thoracic (No. Examined)**	No TA-related findings.	TA-related findings present.	TA-related findings present with partial recovery.
Infiltration, mononuclear cell, adipose	No TA-related findings.	Minimal-to-mild mononuclear cell infiltration within the adipose tissue surrounding the vertebral body that was considered AAV9/AP4M1-related due to low incidence in males and females receiving 3.6E11/≥3.3E12 vg/dose	Minimal mononuclear cell infiltration within the adipose tissue surrounding the vertebral body in females only at ≥1.1E12 vg/dose. Slightly decreased incidence and decreased magnitude (severity) indicates partial recovery by Day 91.

Appendix 18

**Site**/*relevant finding*	**Day 8**	**Day 29**	**Day 91**
**Nerve, Sciatic (No. Examined)**	TA-related trends present on Day 8.	TA-related trends present; increased incidence/magnitude vs. Day 8.	TA-related trends present, equivocal recovery vs. Day 29
Degeneration, axonal	Minimal axonal degeneration in one (1) Group 4 male. No findings in females on Day 8.	Generally dose-dependent increased incidence/severity of minimal-to-mild axonal degeneration in males/females at ≥ 1.1E12 vg/dose.	Generally dose-dependent increased incidence/severity of minimal-to-mild axonal degeneration in males/females at ≥ 3.6E11 vg/dose. Magnitude/severity has decreased (limited to minimal) but there is higher incidence that includes the lowest dose group (Group 2).
**Nerve, Tibia (No. Examined)**	TA-related trends present on Day 8	TA-related trends present; increased incidence/magnitude vs. Day 8.	TA-related trends present, equivocal recovery vs. Day 29
Degeneration, axonal	Minimal axonal degeneration in one (1) Group 3 male and one (1) Group 4 male. No findings in females on Day 8.	Generally dose-dependent increased incidence/severity of minimal-to-mild axonal degeneration in males/females at ≥ 1.1E12 vg/dose.	Generally dose-dependent increased incidence/severity of minimal-to-mild axonal degeneration in males/females at ≥ 3.6E11 vg/dose. Magnitude/severity has decreased (limited to minimal) but there is higher incidence that includes the lowest dose group (Group 2).

Text Table 7
Criteria for Test Article-Related Microscopic Diagnoses

Diagnostic/Descriptive Terminology	Criteria for Diagnosis
**Degeneration, axonal (cauda equina, nerve roots, peripheral nerves [tibial/sciatic])**	May include any or all of the following changes: vacuolation of the axonal sheath, swelling of the axon (characterized by an eosinophilic spheroid evident on cross section and/or tangential/longitudinal section), and/or eosinophilic globular to amorphous debris within the axonal sheath (with or without macrophages evident).
**Degeneration, neuronal**	Characterized by degeneration and/or loss (necrosis) of individual neurons within the dorsal root ganglia. Changes included any/all of the following: loss of cellular detail/loss of differential staining, fading/loss of nissl substance, and satellitosis and/or effacement of the neuronal cell body by glial and/or mononuclear inflammatory cells.
**Infiltration, mononuclear cell**	Infiltration of small lymphocytes +/- rare macrophages and/or plasma cells. When relevant, locations of leptomeninges (pia/arachnoid layers) and/or adipose tissue are specified.

6. CONCLUSIONS

Intrathecal administration of 0, 3.6E11, 1.1E12, or 3.3E12 vg/dose AAV9/AP4M1 in male and female rats at doses resulted in no AAV9/AP4M1-related organ weight changes or gross findings at necropsy. AAV9/AP4M1-related microscopic findings occurred at all time points; incidence/severity of most microscopic findings is generally lowest on Day 8, and incidence/severity of most microscopic findings increased from Day 8 to Day 29, followed by a decrease in incidence/severity for most, but not all findings, from Day 29 to Day 91.

AAV9/AP4M1-related findings on Day 8 occurred in the lumbar dorsal root ganglia, lumbar dorsal nerve roots, and sciatic and tibial nerves. AAV9/AP4M1-related findings in the dorsal root ganglion were limited to mononuclear cell infiltration on Day 8. In the dorsal lumbar nerve root, there was minimal axonal degeneration that was considered treatment-related in males. AAV9/AP4M1-related increased incidence of minimal peripheral nerve degeneration occurred in the tibial and sciatic nerves of males only.

AAV9/AP4M1-related findings on Day 29 occurred in the injection site, lumbar dorsal root ganglia, lumbar dorsal nerve roots, thoracic dorsal root ganglion, cervical dorsal root ganglia, sciatic and tibial nerves, and adipose tissue adjacent to the thoracic spinal column. Findings on Day 29 generally had higher incidence and/or severity across all timepoints (exceptions to this observation are listed for Day 91 when present). AAV9/AP4M1-related findings at the injection site on Day 29 included treatment-related leptomeningeal mononuclear cell infiltration and cauda equina axonal degeneration in males and females. In the lumbar dorsal root ganglion and lumbar dorsal nerve roots there was generally dose-dependent increased incidence and/or severity of axonal degeneration in males and females and one Group 4 male had AAV9/AP4M1-related mononuclear cell infiltration in the lumbar dorsal nerve roots on Day 29. There was minimal-to-mild lumbar dorsal root ganglion neuronal degeneration in males and females on Day 29 that persisted in males and females through Day 91. There was a low incidence of axonal degeneration and/or mononuclear cell infiltration in the cervical and thoracic dorsal root ganglia on Day 29 with complete recovery by Day 91. There was dose-dependent AAV9/AP4M1-related peripheral nerve degeneration in sciatic and tibial nerves of males and females. There was also AAV9/AP4M1-related minimal-to-mild mononuclear cell infiltration within the adipose tissue surrounding the vertebral body that had partial recovery by Day 91.

AAV9/AP4M1-related findings on Day 91 occurred in the injection site, lumbar dorsal root ganglia, lumbar dorsal nerve roots, sciatic and tibial nerves, and adipose tissue adjacent to the thoracic spinal column. Generally, there was partial or complete recovery of most, but not all, microscopic findings on Day 91 compared to Day 29. AAV9/AP4M1-related findings at the injection site included minimal leptomeningeal mononuclear cell infiltration in males with partial recovery by Day 91, although females had a higher incidence in females vs. males. There was also minimal axonal degeneration in the cauda equina of males and females, with partial recovery by Day 91. AAV9/AP4M1-related findings in the lumbar dorsal root ganglion on Day 91 included mononuclear cell infiltration in males and females, with partial recovery, and neuronal degeneration with no recovery. Other findings in the lumbar dorsal root ganglion on Day 91 included minimal axonal degeneration in males and females with partial recovery. In the lumbar dorsal nerve roots on Day 91, there was minimal-to-mild axonal degeneration in males

and females with equivocal recovery (severity is decreased but incidence is higher). There was no mononuclear cell infiltration in lumbar dorsal nerve roots on Day 91, indicating complete recovery of this finding. There was dose-dependent AAV9/AP4M1-related increased incidence of minimal peripheral nerve degeneration (sciatic and tibial nerves) that occurred in males and females with equivocal recovery (severity is decreased but incidence is higher). There were no AAV9/AP4M1-related findings in the thoracic or cervical dorsal root ganglia, indicating complete recovery of the axonal degeneration and mononuclear cell infiltration observed on Day 29.

Appendix 18

Table 1

Summary of Macroscopic Pathology Explanation Page

**Abbreviation**	**Description**
GALT	Gut Associated Lymphoid Tissue

Note: This is a comprehensive list of abbreviations. All of the abbreviations listed may not be applicable to this report.

Dosing Information

Dosing information is abbreviated on various data outputs; the following represents the dosing information for this study:

Group No.	Test Material	Dose Level (vg)
1	Reference Item	0
2	AAV9/AP4M1	3.6E11
3	AAV9/AP4M1	1.1E12
4	AAV9/AP4M1	3.3E12

Appendix 18
Table 1

Summary of Macroscopic Pathology: Day 8

5550008

Removal Reason(s): TERMINAL EUTHANASIA	Male				Female			
Summary: Incidence	0 vg Group 1	0.36x 10E12 vg Group 2	1.1x 10E12 vg Group 3	3.3x 10E12 vg Group 4	0 vg Group 1	0.36x 10E12 vg Group 2	1.1x 10E12 vg Group 3	3.3x 10E12 vg Group 4
Number of Animals:	4	5	5	5	5	5	5	5
**ARTERY, AORTA**								
Submitted	4	5	5	5	5	5	5	5
No Visible Lesions	4	5	5	5	5	5	5	5
**BODY CAVITY, NASAL**								
Submitted	4	5	5	5	5	5	5	5
No Visible Lesions	4	5	5	5	5	5	5	5
**BONE MARROW, STERNUM**								
Submitted	4	5	5	5	5	5	5	5
No Visible Lesions	4	5	5	5	5	5	5	5
**BONE, FEMUR**								
Submitted	4	5	5	5	5	5	5	5
No Visible Lesions	4	5	5	5	5	5	5	5
**BONE, STERNUM**								
Submitted	4	5	5	5	5	5	5	5
No Visible Lesions	4	5	5	5	5	5	5	5
**BRAIN**								
Submitted	4	5	5	5	5	5	5	5
No Visible Lesions	4	5	5	5	5	5	5	5
**CERVIX**								
Submitted	.	.	.	.	5	5	5	5
No Visible Lesions	.	.	.	.	5	5	5	5
**EPIDIDYMIS**								
Submitted	4	5	5	5	.	.	.	.
No Visible Lesions	4	5	5	5	.	.	.	.
**ESOPHAGUS**								
Submitted	4	5	5	5	5	5	5	5
No Visible Lesions	4	5	5	5	5	5	5	5
**EYE**								
Submitted	4	5	5	5	5	5	5	5
No Visible Lesions	4	5	5	5	5	5	5	5
**GALT**								
Submitted	4	5	5	5	5	5	5	5
No Visible Lesions	4	5	5	5	5	5	5	5

Appendix 18

Table 1

Summary of Macroscopic Pathology: Day 8

5550008

Removal Reason(s): TERMINAL EUTHANASIA	Male				Female			
Summary: Incidence	0 vg Group 1	0.36x 10E12 vg Group 2	1.1x 10E12 vg Group 3	3.3x 10E12 vg Group 4	0 vg Group 1	0.36x 10E12 vg Group 2	1.1x 10E12 vg Group 3	3.3x 10E12 vg Group 4
Number of Animals:	4	5	5	5	5	5	5	5
**GANGLION, DORSAL ROOT, CERVICAL**								
Submitted	4	5	5	5	5	5	5	5
No Visible Lesions	4	5	5	5	5	5	5	5
**GANGLION, DORSAL ROOT, LUMBAR**								
Submitted	4	5	5	5	5	5	5	5
No Visible Lesions	4	5	5	5	5	5	5	5
**GANGLION, DORSAL ROOT, THORACIC**								
Submitted	4	5	5	5	5	5	5	5
No Visible Lesions	4	5	5	5	5	5	5	5
**GLAND, ADRENAL**								
Submitted	4	5	5	5	5	5	5	5
No Visible Lesions	4	5	5	5	5	5	5	5
**GLAND, CLITORAL**								
Submitted	.	.	.	.	5	5	5	5
No Visible Lesions	.	.	.	.	5	5	5	5
**GLAND, HARDERIAN**								
Submitted	4	5	5	5	5	5	5	5
No Visible Lesions	4	5	5	5	5	5	5	5
**GLAND, LACRIMAL**								
Submitted	4	5	5	5	5	5	5	5
No Visible Lesions	4	5	5	5	5	5	5	5
**GLAND, MAMMARY**								
Submitted	4	5	5	5	5	5	5	5
No Visible Lesions	4	5	5	5	5	5	5	5
**GLAND, PARATHYROID**								
Submitted	4	5	5	5	5	5	5	5
No Visible Lesions	4	5	5	5	5	5	5	5
**GLAND, PITUITARY**								
Submitted	4	5	5	5	5	5	5	5
No Visible Lesions	4	5	5	3	5	5	5	5
Discoloration, dark	0	0	0	2	0	0	0	0

Appendix 18

Table 1

Summary of Macroscopic Pathology: Day 8

5550008

Removal Reason(s): TERMINAL EUTHANASIA	Male				Female			
Summary: Incidence	0 vg Group 1	0.36x 10E12 vg Group 2	1.1x 10E12 vg Group 3	3.3x 10E12 vg Group 4	0 vg Group 1	0.36x 10E12 vg Group 2	1.1x 10E12 vg Group 3	3.3x 10E12 vg Group 4
Number of Animals:	4	5	5	5	5	5	5	5
**GLAND, PREPUTIAL**								
Submitted	4	5	5	5	.	.	.	.
No Visible Lesions	4	5	5	5	.	.	.	.
**GLAND, PROSTATE**								
Submitted	4	5	5	5	.	.	.	.
No Visible Lesions	4	5	5	5	.	.	.	.
**GLAND, SALIVARY, MANDIBULAR**								
Submitted	4	5	5	5	5	5	5	5
No Visible Lesions	4	5	5	5	5	5	5	5
**GLAND, SALIVARY, PAROTID**								
Submitted	4	5	5	5	5	5	5	5
No Visible Lesions	4	5	5	5	5	5	5	5
**GLAND, SALIVARY, SUBLINGUAL**								
Submitted	4	5	5	5	5	5	5	5
No Visible Lesions	4	5	5	5	5	5	5	5
**GLAND, SEMINAL VESICLE**								
Submitted	4	5	5	5	.	.	.	.
No Visible Lesions	4	5	5	5	.	.	.	.
**GLAND, THYROID**								
Submitted	4	5	5	5	5	5	5	5
No Visible Lesions	4	5	5	5	5	5	5	4
Small	0	0	0	0	0	0	0	1
**GLAND, ZYMBALS**								
Submitted	4	5	5	5	5	5	5	5
No Visible Lesions	4	5	5	5	5	5	5	5
**HEART**								
Submitted	4	5	5	5	5	5	5	5
No Visible Lesions	4	5	5	4	5	5	5	5
Focus, depressed	0	0	0	1	0	0	0	0

Appendix 18

Table 1

Summary of Macroscopic Pathology: Day 8

5550008

Removal Reason(s): TERMINAL EUTHANASIA	Male				Female			
Summary: Incidence	0 vg Group 1	0.36x 10E12 vg Group 2	1.1x 10E12 vg Group 3	3.3x 10E12 vg Group 4	0 vg Group 1	0.36x 10E12 vg Group 2	1.1x 10E12 vg Group 3	3.3x 10E12 vg Group 4
Number of Animals:	4	5	5	5	5	5	5	5
**JOINT, FEMOROTIBIAL**								
Submitted	4	5	5	5	5	5	5	5
No Visible Lesions	4	5	5	5	5	5	5	5
**KIDNEY**								
Submitted	4	5	5	5	5	5	5	5
No Visible Lesions	4	5	5	5	5	5	5	5
**LARGE INTESTINE, CECUM**								
Submitted	4	5	5	5	5	5	5	5
No Visible Lesions	4	5	5	5	5	5	5	5
**LARGE INTESTINE, COLON**								
Submitted	4	5	5	5	5	5	5	5
No Visible Lesions	4	5	5	5	5	5	5	5
**LARGE INTESTINE, RECTUM**								
Submitted	4	5	5	5	5	5	5	5
No Visible Lesions	4	5	5	5	5	5	5	5
**LARYNX**								
Submitted	4	5	5	5	5	5	5	5
No Visible Lesions	4	5	5	5	5	5	5	5
**LIVER**								
Submitted	4	5	5	5	5	5	5	5
No Visible Lesions	3	4	3	5	3	5	4	5
Focus, pale	1	1	2	0	2	0	1	0
**LUNG**								
Submitted	4	5	5	5	5	5	5	5
No Visible Lesions	4	5	5	4	5	5	5	5
Discoloration, dark	0	0	0	1	0	0	0	0
**LYMPH NODE**								
Submitted	.	.	.	.	0	1	0	1
Discoloration, mottled	.	.	.	.	.	1	.	1
**LYMPH NODE, CERVICAL**								
Submitted	4	5	5	5	5	5	5	5
No Visible Lesions	4	4	3	4	5	4	5	5
Discoloration, dark	0	0	0	1	0	0	0	0

Appendix 18

Table 1

Summary of Macroscopic Pathology: Day 8

5550008

Removal Reason(s): TERMINAL EUTHANASIA	Male				Female			
Summary: Incidence	0 vg Group 1	0.36x 10E12 vg Group 2	1.1x 10E12 vg Group 3	3.3x 10E12 vg Group 4	0 vg Group 1	0.36x 10E12 vg Group 2	1.1x 10E12 vg Group 3	3.3x 10E12 vg Group 4
Number of Animals:	4	5	5	5	5	5	5	5
**LYMPH NODE, CERVICAL (Continued...)**								
Discoloration, mottled	0	0	0	0	0	1	0	0
Focus, dark	0	1	2	0	0	0	0	0
**LYMPH NODE, ILIAC**								
Submitted	4	5	5	5	5	5	5	5
No Visible Lesions	4	4	5	5	5	5	5	5
Enlargement	0	1	0	0	0	0	0	0
**LYMPH NODE, MANDIBULAR**								
Submitted	4	5	5	5	5	5	5	5
No Visible Lesions	4	5	3	4	5	5	5	4
Focus, dark	0	0	1	0	0	0	0	1
Discoloration, mottled	0	0	1	1	0	0	0	0
**LYMPH NODE, MESENTERIC**								
Submitted	4	5	5	5	5	5	5	5
No Visible Lesions	4	5	5	5	5	5	5	5
**MUSCLE, BICEPS FEMORIS**								
Submitted	4	5	5	5	5	5	5	5
No Visible Lesions	4	5	5	5	5	5	5	5
**MUSCLE, GASTROCNEMIUS**								
Submitted	4	5	5	5	5	5	5	5
No Visible Lesions	4	5	5	5	5	5	5	5
**NERVE, OPTIC**								
Submitted	4	5	5	5	5	5	5	5
No Visible Lesions	4	5	5	5	5	5	5	5
**NERVE, SCIATIC**								
Submitted	4	5	5	5	5	5	5	5
No Visible Lesions	4	5	5	5	5	5	5	5
**NERVE, TIBIAL**								
Submitted	4	5	5	5	5	5	5	5
No Visible Lesions	4	5	5	5	5	5	5	5

Appendix 18

Table 1

Summary of Macroscopic Pathology: Day 8

5550008

Removal Reason(s): TERMINAL EUTHANASIA	Male				Female			
Summary: Incidence	0 vg Group 1	0.36x 10E12 vg Group 2	1.1x 10E12 vg Group 3	3.3x 10E12 vg Group 4	0 vg Group 1	0.36x 10E12 vg Group 2	1.1x 10E12 vg Group 3	3.3x 10E12 vg Group 4
Number of Animals:	4	5	5	5	5	5	5	5
**NERVE ROOT, DORSAL, CERVICAL**								
Submitted	4	5	5	5	5	5	5	5
No Visible Lesions	4	5	5	5	5	5	5	5
**NERVE ROOT, DORSAL, LUMBAR**								
Submitted	4	5	5	5	5	5	5	5
No Visible Lesions	4	5	5	5	5	5	5	5
**NERVE ROOT, DORSAL, THORACIC**								
Submitted	4	5	5	5	5	5	5	5
No Visible Lesions	4	5	5	5	5	5	5	5
**NERVE ROOT, VENTRAL, CERVICAL**								
Submitted	4	5	5	5	5	5	5	5
No Visible Lesions	4	5	5	5	5	5	5	5
**NERVE ROOT, VENTRAL, LUMBAR**								
Submitted	4	5	5	5	5	5	5	5
No Visible Lesions	4	5	5	5	5	5	5	5
**NERVE ROOT, VENTRAL, THORACIC**								
Submitted	4	5	5	5	5	5	5	5
No Visible Lesions	4	5	5	5	5	5	5	5
**OVARY**								
Submitted	.	.	.	.	5	5	5	5
No Visible Lesions	.	.	.	.	5	5	5	5
**OVIDUCT**								
Submitted	.	.	.	.	5	5	5	5
No Visible Lesions	.	.	.	.	5	5	5	5
**PANCREAS**								
Submitted	4	5	5	5	5	5	5	5
No Visible Lesions	4	5	5	5	5	5	5	5

Appendix 18
Table 1

Summary of Macroscopic Pathology: Day 8

5550008

Removal Reason(s): TERMINAL EUTHANASIA	Male				Female			
Summary: Incidence	0 vg Group 1	0.36x 10E12 vg Group 2	1.1x 10E12 vg Group 3	3.3x 10E12 vg Group 4	0 vg Group 1	0.36x 10E12 vg Group 2	1.1x 10E12 vg Group 3	3.3x 10E12 vg Group 4
Number of Animals:	4	5	5	5	5	5	5	5
**SITE, INJECTION**								
Submitted	4	5	5	5	5	5	5	5
No Visible Lesions	4	5	5	5	5	5	5	5
**SITE, SURGICAL**								
Submitted	.	.	.	.	0	1	0	1
Focus, dark	.	.	.	.	.	1	.	1
**SKIN**								
Submitted	4	5	5	5	5	5	5	5
No Visible Lesions	4	5	5	5	5	5	5	5
**SMALL INTESTINE, DUODENUM**								
Submitted	4	5	5	5	5	5	5	5
No Visible Lesions	4	5	5	5	5	5	5	5
**SMALL INTESTINE, ILEUM**								
Submitted	4	5	5	5	5	5	5	5
No Visible Lesions	4	5	5	5	5	5	5	5
**SMALL INTESTINE, JEJUNUM**								
Submitted	4	5	5	5	5	5	5	5
No Visible Lesions	4	5	5	5	5	5	5	5
**SPINAL CORD, CERVICAL**								
Submitted	4	5	5	5	5	5	5	5
No Visible Lesions	4	5	5	5	5	5	5	5
**SPINAL CORD, LUMBAR**								
Submitted	4	5	5	5	5	5	5	5
No Visible Lesions	4	5	5	5	5	5	5	5
**SPINAL CORD, THORACIC**								
Submitted	4	5	5	5	5	5	5	5
No Visible Lesions	4	5	5	5	5	5	5	5
**SPLEEN**								
Submitted	4	5	5	5	5	5	5	5
No Visible Lesions	4	5	5	5	5	5	5	5

Appendix 18

Table 1

Summary of Macroscopic Pathology: Day 8

5550008

Removal Reason(s): TERMINAL EUTHANASIA	Male				Female			
Summary: Incidence	0 vg Group 1	0.36x 10E12 vg Group 2	1.1x 10E12 vg Group 3	3.3x 10E12 vg Group 4	0 vg Group 1	0.36x 10E12 vg Group 2	1.1x 10E12 vg Group 3	3.3x 10E12 vg Group 4
Number of Animals:	4	5	5	5	5	5	5	5
**STOMACH**								
Submitted	4	5	5	5	5	5	5	5
No Visible Lesions	4	5	5	5	5	5	5	5
**SUBCUTIS**								
Submitted	0	0	1	0	.	.	.	.
Focus, dark	.	.	1	.	.	.	.	.
**TESTIS**								
Submitted	4	5	5	5	.	.	.	.
No Visible Lesions	4	5	5	5	.	.	.	.
**THYMUS**								
Submitted	4	5	5	5	5	5	5	5
No Visible Lesions	3	3	5	2	4	4	4	4
Focus, dark	1	2	0	3	1	1	1	0
Discoloration, mottled	0	0	0	0	0	0	0	1
**TONGUE**								
Submitted	4	5	5	5	5	5	5	5
No Visible Lesions	4	5	5	5	5	5	5	5
**TRACHEA**								
Submitted	4	5	5	5	5	5	5	5
No Visible Lesions	4	5	5	5	5	5	5	5
**URETER**								
Submitted	4	5	5	5	5	5	5	5
No Visible Lesions	4	5	5	5	5	5	5	5
**URINARY BLADDER**								
Submitted	4	5	5	5	5	5	5	5
No Visible Lesions	4	5	5	5	5	5	5	5
**UTERUS**								
Submitted	.	.	.	.	5	5	5	5
No Visible Lesions	.	.	.	.	5	5	5	4
Cyst, pale	.	.	.	.	0	0	0	1
**VAGINA**								
Submitted	.	.	.	.	5	5	5	5
No Visible Lesions	.	.	.	.	5	5	5	5

Appendix 18
Table 1

Summary of Macroscopic Pathology: Day 29

5550008

Removal Reason(s): RECOVERY EUTHANASIA	Male				Female			
Summary: Incidence	0 vg Group 1	0.36x 10E12 vg Group 2	1.1x 10E12 vg Group 3	3.3x 10E12 vg Group 4	0 vg Group 1	0.36x 10E12 vg Group 2	1.1x 10E12 vg Group 3	3.3x 10E12 vg Group 4
Number of Animals:	5	5	5	5	5	5	5	5
**ARTERY, AORTA**								
Submitted	5	5	5	5	5	5	5	5
No Visible Lesions	5	5	5	5	5	5	5	5
**BODY CAVITY, NASAL**								
Submitted	5	5	5	5	5	5	5	5
No Visible Lesions	5	5	5	5	5	5	5	5
**BONE MARROW, STERNUM**								
Submitted	5	5	5	5	5	5	5	5
No Visible Lesions	5	5	5	5	5	5	5	5
**BONE, FEMUR**								
Submitted	5	5	5	5	5	5	5	5
No Visible Lesions	5	5	5	5	5	5	5	5
**BONE, STERNUM**								
Submitted	5	5	5	5	5	5	5	5
No Visible Lesions	5	5	5	5	5	5	5	5
**BRAIN**								
Submitted	5	5	5	5	5	5	5	5
No Visible Lesions	5	5	5	5	5	5	5	5
**CERVIX**								
Submitted	.	.	.	.	5	5	5	5
No Visible Lesions	.	.	.	.	5	5	5	5
**EPIDIDYMIS**								
Submitted	5	5	5	5	.	.	.	.
No Visible Lesions	5	5	4	5	.	.	.	.
Small	0	0	1	0	.	.	.	.
**ESOPHAGUS**								
Submitted	5	5	5	5	5	5	5	5
No Visible Lesions	5	5	5	5	5	5	5	5
**EYE**								
Submitted	5	5	5	5	5	5	5	5
No Visible Lesions	5	5	5	5	5	5	5	5
**GALT**								
Submitted	5	5	5	5	5	5	5	5

Summary of Macroscopic Pathology: Day 29

5550008

Removal Reason(s): RECOVERY EUTHANASIA	Male				Female			
Summary: Incidence	0 vg Group 1	0.36x 10E12 vg Group 2	1.1x 10E12 vg Group 3	3.3x 10E12 vg Group 4	0 vg Group 1	0.36x 10E12 vg Group 2	1.1x 10E12 vg Group 3	3.3x 10E12 vg Group 4
Number of Animals:	5	5	5	5	5	5	5	5
**GALT (Continued...)**								
No Visible Lesions	5	5	5	5	5	5	5	5
**GANGLION, DORSAL ROOT, CERVICAL**								
Submitted	5	5	5	5	5	5	5	5
No Visible Lesions	5	5	5	5	5	5	5	5
**GANGLION, DORSAL ROOT, LUMBAR**								
Submitted	5	5	5	5	5	5	5	5
No Visible Lesions	5	5	5	5	5	5	5	5
**GANGLION, DORSAL ROOT, THORACIC**								
Submitted	5	5	5	5	5	5	5	5
No Visible Lesions	5	5	5	5	5	5	5	5
**GLAND, ADRENAL**								
Submitted	5	5	5	5	5	5	5	5
No Visible Lesions	5	5	5	5	5	5	5	5
**GLAND, CLITORAL**								
Submitted	.	.	.	.	5	5	5	5
No Visible Lesions	.	.	.	.	5	5	5	5
**GLAND, HARDERIAN**								
Submitted	5	5	5	5	5	5	5	5
No Visible Lesions	5	5	5	5	5	5	5	5
**GLAND, LACRIMAL**								
Submitted	5	5	5	5	5	5	5	5
No Visible Lesions	5	5	5	5	5	5	5	5
**GLAND, MAMMARY**								
Submitted	5	5	5	5	5	5	5	5
No Visible Lesions	5	5	5	5	5	5	5	5
**GLAND, PARATHYROID**								
Submitted	5	5	5	5	5	5	5	5
No Visible Lesions	5	5	5	5	5	5	5	5
**GLAND, PITUITARY**								
Submitted	5	5	5	5	5	5	5	5

Appendix 18

Table 1

Summary of Macroscopic Pathology: Day 29

5550008

Removal Reason(s): RECOVERY EUTHANASIA<br>Summary: Incidence	Male				Female			
	0 vg Group 1	0.36x 10E12 vg Group 2	1.1x 10E12 vg Group 3	3.3x 10E12 vg Group 4	0 vg Group 1	0.36x 10E12 vg Group 2	1.1x 10E12 vg Group 3	3.3x 10E12 vg Group 4
Number of Animals:	5	5	5	5	5	5	5	5
**GLAND, PITUITARY (Continued...)**								
No Visible Lesions	5	4	5	5	5	5	5	5
Discoloration, dark	0	1	0	0	0	0	0	0
**GLAND, PREPUTIAL**								
Submitted	5	5	5	5	.	.	.	.
No Visible Lesions	5	5	5	5	.	.	.	.
**GLAND, PROSTATE**								
Submitted	5	5	5	5	.	.	.	.
No Visible Lesions	5	5	5	5	.	.	.	.
**GLAND, SALIVARY, MANDIBULAR**								
Submitted	5	5	5	5	5	5	5	5
No Visible Lesions	5	5	5	5	5	5	5	5
**GLAND, SALIVARY, PAROTID**								
Submitted	5	5	5	5	5	5	5	5
No Visible Lesions	5	5	5	5	5	5	5	5
**GLAND, SALIVARY, SUBLINGUAL**								
Submitted	5	5	5	5	5	5	5	5
No Visible Lesions	5	5	5	5	5	5	5	5
**GLAND, SEMINAL VESICLE**								
Submitted	5	5	5	5	.	.	.	.
No Visible Lesions	5	5	5	5	.	.	.	.
**GLAND, THYROID**								
Submitted	5	5	5	5	5	5	5	5
No Visible Lesions	5	5	5	4	4	5	5	5
Enlargement	0	0	0	1	0	0	0	0
Focus, dark	0	0	0	0	1	0	0	0
**GLAND, ZYMBALS**								
Submitted	5	5	5	5	5	5	5	5
No Visible Lesions	5	5	5	5	5	5	5	5

Appendix 18

Table 1

Summary of Macroscopic Pathology: Day 29

5550008

Removal Reason(s): RECOVERY EUTHANASIA	Male				Female			
Summary: Incidence	0 vg Group 1	0.36x 10E12 vg Group 2	1.1x 10E12 vg Group 3	3.3x 10E12 vg Group 4	0 vg Group 1	0.36x 10E12 vg Group 2	1.1x 10E12 vg Group 3	3.3x 10E12 vg Group 4
Number of Animals:	5	5	5	5	5	5	5	5
**HEART**								
Submitted	5	5	5	5	5	5	5	5
No Visible Lesions	5	5	5	5	5	5	5	5
**JOINT, FEMOROTIBIAL**								
Submitted	5	5	5	5	5	5	5	5
No Visible Lesions	5	5	5	5	5	5	5	5
**KIDNEY**								
Submitted	5	5	5	5	5	5	5	5
No Visible Lesions	5	5	5	5	5	5	5	5
**LARGE INTESTINE, CECUM**								
Submitted	5	5	5	5	5	5	5	5
No Visible Lesions	5	5	5	5	5	5	5	5
**LARGE INTESTINE, COLON**								
Submitted	5	5	5	5	5	5	5	5
No Visible Lesions	5	5	5	5	5	5	5	5
**LARGE INTESTINE, RECTUM**								
Submitted	5	5	5	5	5	5	5	5
No Visible Lesions	5	5	5	5	5	5	5	5
**LARYNX**								
Submitted	5	5	5	5	5	5	5	5
No Visible Lesions	5	5	5	5	5	5	5	5
**LIVER**								
Submitted	5	5	5	5	5	5	5	5
No Visible Lesions	4	5	3	4	5	4	4	4
Focus, pale	1	0	2	1	0	1	1	1
**LUNG**								
Submitted	5	5	5	5	5	5	5	5
No Visible Lesions	5	5	5	5	5	5	5	5
**LYMPH NODE**								
Submitted	0	1	0	1	1	0	2	1
Discoloration, mottled	.	1	.	0	0	.	1	1
Discoloration, dark	.	0	.	1	0	.	0	0
Focus, dark	.	0	.	0	1	.	1	0

Appendix 18

Table 1

Summary of Macroscopic Pathology: Day 29

5550008

Removal Reason(s): RECOVERY EUTHANASIA	Male				Female			
Summary: Incidence	0 vg Group 1	0.36x 10E12 vg Group 2	1.1x 10E12 vg Group 3	3.3x 10E12 vg Group 4	0 vg Group 1	0.36x 10E12 vg Group 2	1.1x 10E12 vg Group 3	3.3x 10E12 vg Group 4
Number of Animals:	5	5	5	5	5	5	5	5
**LYMPH NODE (Continued...)**								
Enlargement	.	0	.	0	0	.	2	0
**LYMPH NODE, CERVICAL**								
Submitted	5	5	5	5	5	5	5	5
No Visible Lesions	5	5	5	5	5	5	4	5
Focus, dark	0	0	0	0	0	0	1	0
**LYMPH NODE, ILIAC**								
Submitted	5	5	5	5	4	5	5	5
No Visible Lesions	5	5	5	5	5	4	5	5
Discoloration, mottled	0	0	0	0	0	1	0	0
**LYMPH NODE, MANDIBULAR**								
Submitted	5	5	5	5	5	5	5	5
No Visible Lesions	5	4	4	5	5	4	4	5
Focus, dark	0	1	1	0	0	0	1	0
Enlargement	0	0	1	0	0	1	0	0
**LYMPH NODE, MESENTERIC**								
Submitted	5	5	5	5	5	5	5	5
No Visible Lesions	5	5	5	5	5	5	5	5
**MUSCLE, BICEPS FEMORIS**								
Submitted	5	5	5	5	5	5	5	5
No Visible Lesions	5	5	5	5	5	5	5	5
**MUSCLE, GASTROCNEMIUS**								
Submitted	5	5	5	5	5	5	5	5
No Visible Lesions	5	5	5	5	5	5	5	5
**NERVE, OPTIC**								
Submitted	5	5	5	5	5	5	5	5
No Visible Lesions	5	5	5	5	5	5	5	5
**NERVE, SCIATIC**								
Submitted	5	5	5	5	5	5	5	5
No Visible Lesions	5	5	5	5	5	5	5	5
**NERVE, TIBIAL**								
Submitted	5	5	5	5	5	5	5	5

Appendix 18

Table 1

Summary of Macroscopic Pathology: Day 29

5550008

Removal Reason(s): RECOVERY EUTHANASIA	Male				Female			
Summary: Incidence	0 vg Group 1	0.36x 10E12 vg Group 2	1.1x 10E12 vg Group 3	3.3x 10E12 vg Group 4	0 vg Group 1	0.36x 10E12 vg Group 2	1.1x 10E12 vg Group 3	3.3x 10E12 vg Group 4
Number of Animals:	5	5	5	5	5	5	5	5
**NERVE, TIBIAL (Continued...)**								
No Visible Lesions	5	5	5	5	5	5	5	5
**NERVE ROOT, DORSAL, CERVICAL**								
Submitted	5	5	5	5	5	5	5	5
No Visible Lesions	5	5	5	5	5	5	5	5
**NERVE ROOT, DORSAL, LUMBAR**								
Submitted	5	5	5	5	5	5	5	5
No Visible Lesions	5	5	5	5	5	5	5	5
**NERVE ROOT, DORSAL, THORACIC**								
Submitted	5	5	5	5	5	5	5	5
No Visible Lesions	5	5	5	5	5	5	5	5
**NERVE ROOT, VENTRAL, CERVICAL**								
Submitted	5	5	5	5	5	5	5	5
No Visible Lesions	5	5	5	5	5	5	5	5
**NERVE ROOT, VENTRAL, LUMBAR**								
Submitted	5	5	5	5	5	5	5	5
No Visible Lesions	5	5	5	5	5	5	5	5
**NERVE ROOT, VENTRAL, THORACIC**								
Submitted	5	5	5	5	5	5	5	5
No Visible Lesions	5	5	5	5	5	5	5	5
**OVARY**								
Submitted	.	.	.	.	5	5	5	5
No Visible Lesions	.	.	.	.	5	5	4	5
Cyst, pale	.	.	.	.	0	0	1	0
**OVIDUCT**								
Submitted	.	.	.	.	5	5	5	5
No Visible Lesions	.	.	.	.	5	5	5	5

Appendix 18

Table 1

Summary of Macroscopic Pathology: Day 29

5550008

Removal Reason(s): RECOVERY EUTHANASIA	Male				Female			
Summary: Incidence	0 vg Group 1	0.36x 10E12 vg Group 2	1.1x 10E12 vg Group 3	3.3x 10E12 vg Group 4	0 vg Group 1	0.36x 10E12 vg Group 2	1.1x 10E12 vg Group 3	3.3x 10E12 vg Group 4
Number of Animals:	5	5	5	5	5	5	5	5
**PANCREAS**								
Submitted	5	5	5	5	5	5	5	5
No Visible Lesions	5	5	5	5	5	5	5	5
**SITE, INJECTION**								
Submitted	5	5	5	5	5	5	5	5
No Visible Lesions	5	5	5	5	5	5	5	5
**SKIN**								
Submitted	5	5	5	5	5	5	5	5
No Visible Lesions	5	5	5	5	5	5	5	5
**SMALL INTESTINE, DUODENUM**								
Submitted	5	5	5	5	5	5	5	5
No Visible Lesions	5	5	5	5	5	5	5	5
**SMALL INTESTINE, ILEUM**								
Submitted	5	5	5	5	5	5	5	5
No Visible Lesions	5	5	5	5	5	5	5	5
**SMALL INTESTINE, JEJUNUM**								
Submitted	5	5	5	5	5	5	5	5
No Visible Lesions	5	5	5	5	5	5	5	5
**SPINAL CORD, CERVICAL**								
Submitted	5	5	5	5	5	5	5	5
No Visible Lesions	5	5	5	5	5	5	5	5
**SPINAL CORD, LUMBAR**								
Submitted	5	5	5	5	5	5	5	5
No Visible Lesions	5	5	5	5	5	5	5	5
**SPINAL CORD, THORACIC**								
Submitted	5	5	5	5	5	5	5	5
No Visible Lesions	5	5	5	5	5	5	5	5
**SPLEEN**								
Submitted	5	5	5	5	5	5	5	5
No Visible Lesions	5	5	5	5	5	5	5	5

Appendix 18

Table 1

Summary of Macroscopic Pathology: Day 29

5550008

Removal Reason(s): RECOVERY EUTHANASIA	Male				Female			
Summary: Incidence	0 vg Group 1	0.36x 10E12 vg Group 2	1.1x 10E12 vg Group 3	3.3x 10E12 vg Group 4	0 vg Group 1	0.36x 10E12 vg Group 2	1.1x 10E12 vg Group 3	3.3x 10E12 vg Group 4
Number of Animals:	5	5	5	5	5	5	5	5
**STOMACH**								
Submitted	5	5	5	5	5	5	5	5
No Visible Lesions	5	5	5	5	5	5	5	5
**SUBCUTIS**								
Submitted	0	0	1	0	.	.	.	.
Focus, dark	.	.	1	.	.	.	.	.
**TESTIS**								
Submitted	5	5	5	5	.	.	.	.
No Visible Lesions	5	5	4	5	.	.	.	.
Small	0	0	1	0	.	.	.	.
Abnormal consistency; soft	0	0	1	0	.	.	.	.
**THYMUS**								
Submitted	5	5	5	5	5	5	5	5
No Visible Lesions	5	5	5	3	4	3	5	5
Focus, dark	0	0	0	2	0	2	0	0
Discoloration, mottled	0	0	0	0	1	0	0	0
**TONGUE**								
Submitted	5	5	5	5	5	5	5	5
No Visible Lesions	5	5	5	5	5	5	5	5
**TRACHEA**								
Submitted	5	5	5	5	5	5	5	5
No Visible Lesions	5	5	5	5	5	5	5	5
**URETER**								
Submitted	5	5	5	5	5	5	5	5
No Visible Lesions	5	5	5	5	5	5	5	5
**URINARY BLADDER**								
Submitted	5	5	5	5	5	5	5	5
No Visible Lesions	5	5	5	5	5	5	5	5
**UTERUS**								
Submitted	.	.	.	.	5	5	5	5
No Visible Lesions	.	.	.	.	5	5	5	5
**VAGINA**								
Submitted	.	.	.	.	5	5	5	5

Appendix 18

Table 1

Summary of Macroscopic Pathology: Day 29

5550008

Removal Reason(s): RECOVERY EUTHANASIA	Male				Female			
Summary: Incidence	0 vg Group 1	0.36x 10E12 vg Group 2	1.1x 10E12 vg Group 3	3.3x 10E12 vg Group 4	0 vg Group 1	0.36x 10E12 vg Group 2	1.1x 10E12 vg Group 3	3.3x 10E12 vg Group 4
Number of Animals:	5	5	5	5	5	5	5	5
**VAGINA (Continued...)**								
No Visible Lesions	.	.	.	.	5	5	5	5

Appendix 18

Table 1

Summary of Macroscopic Pathology: Day 91

5550008

Removal Reason(s): RECOVERY EUTHANASIA	Male				Female			
Summary: Incidence	0 vg Group 1	0.36x 10E12 vg Group 2	1.1x 10E12 vg Group 3	3.3x 10E12 vg Group 4	0 vg Group 1	0.36x 10E12 vg Group 2	1.1x 10E12 vg Group 3	3.3x 10E12 vg Group 4
Number of Animals:	5	5	5	5	5	5	5	5
**ARTERY, AORTA**								
Submitted	5	5	5	5	5	5	5	5
No Visible Lesions	5	5	5	5	5	5	5	5
**BODY CAVITY, NASAL**								
Submitted	5	5	5	5	5	5	5	5
No Visible Lesions	5	5	5	5	5	5	5	5
**BONE MARROW, STERNUM**								
Submitted	5	5	5	5	5	5	5	5
No Visible Lesions	5	5	5	5	5	5	5	5
**BONE, FEMUR**								
Submitted	5	5	5	5	5	5	5	5
No Visible Lesions	5	5	5	5	5	5	5	5
**BONE, STERNUM**								
Submitted	5	5	5	5	5	5	5	5
No Visible Lesions	5	5	5	5	5	5	5	5
**BRAIN**								
Submitted	5	5	5	5	5	5	5	5
No Visible Lesions	5	5	5	5	5	5	5	5
**CERVIX**								
Submitted	.	.	.	.	5	5	5	5
No Visible Lesions	.	.	.	.	5	5	5	5
**EPIDIDYMIS**								
Submitted	5	5	5	5	.	.	.	.
No Visible Lesions	5	5	4	4	.	.	.	.
Small	0	0	0	1	.	.	.	.
Focus, raised	0	0	1	0	.	.	.	.
**ESOPHAGUS**								
Submitted	5	5	5	5	5	5	5	5
No Visible Lesions	5	5	5	5	5	5	5	5
**EYE**								
Submitted	5	5	5	5	5	5	5	5
No Visible Lesions	5	5	5	5	5	5	5	5

Appendix 18

Table 1

Summary of Macroscopic Pathology: Day 91

5550008

Removal Reason(s): RECOVERY EUTHANASIA	Male				Female			
Summary: Incidence	0 vg Group 1	0.36x 10E12 vg Group 2	1.1x 10E12 vg Group 3	3.3x 10E12 vg Group 4	0 vg Group 1	0.36x 10E12 vg Group 2	1.1x 10E12 vg Group 3	3.3x 10E12 vg Group 4
Number of Animals:	5	5	5	5	5	5	5	5
**GALT**								
Submitted	5	5	5	5	5	5	5	5
No Visible Lesions	5	5	5	5	5	5	5	5
**GANGLION, DORSAL ROOT, CERVICAL**								
Submitted	5	5	5	5	5	5	5	5
No Visible Lesions	5	5	5	5	5	5	5	5
**GANGLION, DORSAL ROOT, LUMBAR**								
Submitted	5	5	5	5	5	5	5	5
No Visible Lesions	5	5	5	5	5	5	5	5
**GANGLION, DORSAL ROOT, THORACIC**								
Submitted	5	5	5	5	5	5	5	5
No Visible Lesions	5	5	5	5	5	5	5	5
**GLAND, ADRENAL**								
Submitted	5	5	5	5	5	5	5	5
No Visible Lesions	5	5	5	5	5	5	4	5
Enlargement	0	0	0	0	0	0	1	0
**GLAND, CLITORAL**								
Submitted	.	.	.	.	5	5	5	5
No Visible Lesions	.	.	.	.	5	5	5	5
**GLAND, HARDERIAN**								
Submitted	5	5	5	5	5	5	5	5
No Visible Lesions	5	5	5	5	5	5	5	5
**GLAND, LACRIMAL**								
Submitted	5	5	5	5	5	5	5	5
No Visible Lesions	5	5	5	5	5	5	5	5
**GLAND, MAMMARY**								
Submitted	5	5	5	5	5	5	5	5
No Visible Lesions	5	5	5	5	5	5	5	5
**GLAND, PARATHYROID**								
Submitted	5	5	5	5	5	5	5	5
No Visible Lesions	5	5	5	5	5	5	5	5

Appendix 18

Table 1

Summary of Macroscopic Pathology: Day 91

5550008

Removal Reason(s): RECOVERY EUTHANASIA	Male				Female			
Summary: Incidence	0 vg Group 1	0.36x 10E12 vg Group 2	1.1x 10E12 vg Group 3	3.3x 10E12 vg Group 4	0 vg Group 1	0.36x 10E12 vg Group 2	1.1x 10E12 vg Group 3	3.3x 10E12 vg Group 4
Number of Animals:	5	5	5	5	5	5	5	5
**GLAND, PITUITARY**								
Submitted	5	5	5	5	5	5	5	5
No Visible Lesions	5	5	5	5	4	5	4	3
Enlargement	0	0	0	0	1	0	1	2
**GLAND, PREPUTIAL**								
Submitted	5	5	5	5	.	.	.	.
No Visible Lesions	5	5	5	5	.	.	.	.
**GLAND, PROSTATE**								
Submitted	5	5	5	5	.	.	.	.
No Visible Lesions	5	5	5	5	.	.	.	.
**GLAND, SALIVARY, MANDIBULAR**								
Submitted	5	5	5	5	5	5	5	5
No Visible Lesions	5	5	5	5	5	5	5	5
**GLAND, SALIVARY, PAROTID**								
Submitted	5	5	5	5	5	5	5	5
No Visible Lesions	5	5	5	5	5	5	5	5
**GLAND, SALIVARY, SUBLINGUAL**								
Submitted	5	5	5	5	5	5	5	5
No Visible Lesions	5	5	5	5	5	5	5	5
**GLAND, SEMINAL VESICLE**								
Submitted	5	5	5	5	.	.	.	.
No Visible Lesions	5	5	5	4	.	.	.	.
Small	0	0	0	1	.	.	.	.
**GLAND, THYROID**								
Submitted	5	5	5	5	5	5	5	5
No Visible Lesions	5	5	5	5	5	5	5	4
Small	0	0	0	0	0	0	0	1
**GLAND, ZYMBALS**								
Submitted	5	5	5	5	5	5	5	5
No Visible Lesions	5	5	5	5	5	5	5	5

Appendix 18

Table 1

Summary of Macroscopic Pathology: Day 91

5550008

Removal Reason(s): RECOVERY EUTHANASIA	Male				Female			
Summary: Incidence	0 vg Group 1	0.36x 10E12 vg Group 2	1.1x 10E12 vg Group 3	3.3x 10E12 vg Group 4	0 vg Group 1	0.36x 10E12 vg Group 2	1.1x 10E12 vg Group 3	3.3x 10E12 vg Group 4
Number of Animals:	5	5	5	5	5	5	5	5
**HEART**								
Submitted	5	5	5	5	5	5	5	5
No Visible Lesions	5	5	5	5	5	5	5	5
**JOINT, FEMOROTIBIAL**								
Submitted	5	5	5	5	5	5	5	5
No Visible Lesions	5	5	5	5	5	5	5	5
**KIDNEY**								
Submitted	5	5	5	5	5	5	5	5
No Visible Lesions	5	5	4	5	5	5	5	5
Focus, dark	0	0	1	0	0	0	0	0
**LARGE INTESTINE, CECUM**								
Submitted	5	5	5	5	5	5	5	5
No Visible Lesions	5	5	5	5	5	5	5	5
**LARGE INTESTINE, COLON**								
Submitted	5	5	5	5	5	5	5	5
No Visible Lesions	5	5	5	5	5	5	5	5
**LARGE INTESTINE, RECTUM**								
Submitted	5	5	5	5	5	5	5	5
No Visible Lesions	5	5	5	5	5	5	5	5
**LARYNX**								
Submitted	5	5	5	5	5	5	5	5
No Visible Lesions	5	5	5	5	5	5	5	5
**LIVER**								
Submitted	5	5	5	5	5	5	5	5
No Visible Lesions	1	4	2	2	5	3	3	4
Focus, pale	4	1	3	3	0	2	2	0
Focus, raised	0	0	0	0	0	0	0	1
**LUNG**								
Submitted	5	5	5	5	5	5	5	5
No Visible Lesions	4	5	5	5	5	4	5	5
Focus, pale	0	0	0	0	0	1	0	0
Focus, dark	1	0	0	0	0	0	0	0

Appendix 18

Table 1

Summary of Macroscopic Pathology: Day 91

5550008

Removal Reason(s): RECOVERY EUTHANASIA	Male				Female			
Summary: Incidence	0 vg Group 1	0.36x 10E12 vg Group 2	1.1x 10E12 vg Group 3	3.3x 10E12 vg Group 4	0 vg Group 1	0.36x 10E12 vg Group 2	1.1x 10E12 vg Group 3	3.3x 10E12 vg Group 4
Number of Animals:	5	5	5	5	5	5	5	5
**LYMPH NODE**								
Submitted	0	1	0	0	1	1	0	0
Discoloration, mottled	.	0	.	.	1	1	.	.
Enlargement	.	1	.	.	0	0	.	.
**LYMPH NODE, CERVICAL**								
Submitted	5	5	5	5	5	5	5	5
No Visible Lesions	5	5	4	5	5	4	4	5
Discoloration, dark	0	0	1	0	0	1	0	0
Enlargement	0	0	0	0	0	0	1	0
**LYMPH NODE, ILIAC**								
Submitted	5	5	5	5	5	5	5	5
No Visible Lesions	4	5	4	5	5	5	5	5
Discoloration, dark	1	0	0	0	0	0	0	0
Focus, dark	0	0	1	0	0	0	0	0
**LYMPH NODE, MANDIBULAR**								
Submitted	5	5	5	5	5	5	5	5
No Visible Lesions	5	5	4	4	5	4	4	4
Enlargement	0	0	1	1	0	0	1	1
Discoloration, dark	0	0	0	0	0	1	0	0
**LYMPH NODE, MESENTERIC**								
Submitted	5	5	5	5	5	5	5	5
No Visible Lesions	5	5	5	5	5	5	5	4
Enlargement	0	0	0	0	0	0	0	1
**MUSCLE, BICEPS FEMORIS**								
Submitted	5	5	5	5	5	5	5	5
No Visible Lesions	5	5	5	5	5	5	5	5
**MUSCLE, GASTROCNEMIUS**								
Submitted	5	5	5	5	5	5	5	5
No Visible Lesions	5	5	5	5	5	5	5	5
**NERVE, OPTIC**								
Submitted	5	5	5	5	5	5	5	5
No Visible Lesions	5	5	5	5	5	5	5	5

Appendix 18
Table 1

Summary of Macroscopic Pathology: Day 91

5550008

Removal Reason(s): RECOVERY EUTHANASIA	Male				Female			
Summary: Incidence	0 vg Group 1	0.36x 10E12 vg Group 2	1.1x 10E12 vg Group 3	3.3x 10E12 vg Group 4	0 vg Group 1	0.36x 10E12 vg Group 2	1.1x 10E12 vg Group 3	3.3x 10E12 vg Group 4
Number of Animals:	5	5	5	5	5	5	5	5
**NERVE, SCIATIC**								
Submitted	5	5	5	5	5	5	5	5
No Visible Lesions	5	5	5	5	5	5	5	5
**NERVE, TIBIAL**								
Submitted	5	5	5	5	5	5	5	5
No Visible Lesions	5	5	5	5	5	5	5	5
**NERVE ROOT, DORSAL, CERVICAL**								
Submitted	5	5	5	5	5	5	5	5
No Visible Lesions	5	5	5	5	5	5	5	5
**NERVE ROOT, DORSAL, LUMBAR**								
Submitted	5	5	5	5	5	5	5	5
No Visible Lesions	5	5	5	5	5	5	5	5
**NERVE ROOT, DORSAL, THORACIC**								
Submitted	5	5	5	5	5	5	5	5
No Visible Lesions	5	5	5	5	5	5	5	5
**NERVE ROOT, VENTRAL, CERVICAL**								
Submitted	5	5	5	5	5	5	5	5
No Visible Lesions	5	5	5	5	5	5	5	5
**NERVE ROOT, VENTRAL, LUMBAR**								
Submitted	5	5	5	5	5	5	5	5
No Visible Lesions	5	5	5	5	5	5	5	5
**NERVE ROOT, VENTRAL, THORACIC**								
Submitted	5	5	5	5	5	5	5	5
No Visible Lesions	5	5	5	5	5	5	5	5
**OVARY**								
Submitted	.	.	.	.	5	5	5	5
No Visible Lesions	.	.	.	.	5	5	5	5

Appendix 18

Table 1

Summary of Macroscopic Pathology: Day 91

5550008

Removal Reason(s): RECOVERY EUTHANASIA	Male				Female			
Summary: Incidence	0 vg Group 1	0.36x 10E12 vg Group 2	1.1x 10E12 vg Group 3	3.3x 10E12 vg Group 4	0 vg Group 1	0.36x 10E12 vg Group 2	1.1x 10E12 vg Group 3	3.3x 10E12 vg Group 4
Number of Animals:	5	5	5	5	5	5	5	5
**OVIDUCT**								
Submitted	.	.	.	.	5	5	5	5
No Visible Lesions	.	.	.	.	5	5	5	5
**PANCREAS**								
Submitted	5	5	5	5	5	5	5	5
No Visible Lesions	5	5	5	5	5	5	5	5
**SITE, INJECTION**								
Submitted	5	5	5	5	5	5	5	5
No Visible Lesions	5	5	5	5	5	5	5	5
**SKIN**								
Submitted	5	5	5	5	5	5	5	5
No Visible Lesions	5	5	5	5	5	5	5	5
**SMALL INTESTINE, DUODENUM**								
Submitted	5	5	5	5	5	5	5	5
No Visible Lesions	5	5	5	5	5	5	5	5
**SMALL INTESTINE, ILEUM**								
Submitted	5	5	5	5	5	5	5	5
No Visible Lesions	5	5	5	5	5	5	5	5
**SMALL INTESTINE, JEJUNUM**								
Submitted	5	5	5	5	5	5	5	5
No Visible Lesions	5	4	5	5	5	5	5	5
Diverticulum	0	1	0	0	0	0	0	0
**SPINAL CORD, CERVICAL**								
Submitted	5	5	5	5	5	5	5	5
No Visible Lesions	5	5	5	5	5	5	5	5
**SPINAL CORD, LUMBAR**								
Submitted	5	5	5	5	5	5	5	5
No Visible Lesions	5	5	5	5	5	5	5	5
**SPINAL CORD, THORACIC**								
Submitted	5	5	5	5	5	5	5	5
No Visible Lesions	5	5	5	5	5	5	5	5

Appendix 18

Table 1

Summary of Macroscopic Pathology: Day 91

5550008

Removal Reason(s): RECOVERY EUTHANASIA	Male				Female			
Summary: Incidence	0 vg Group 1	0.36x 10E12 vg Group 2	1.1x 10E12 vg Group 3	3.3x 10E12 vg Group 4	0 vg Group 1	0.36x 10E12 vg Group 2	1.1x 10E12 vg Group 3	3.3x 10E12 vg Group 4
Number of Animals:	5	5	5	5	5	5	5	5
**SPLEEN**								
Submitted	5	5	5	5	5	5	5	5
No Visible Lesions	5	5	5	5	5	5	5	5
**STOMACH**								
Submitted	5	5	5	5	5	5	5	5
No Visible Lesions	5	4	5	4	5	5	5	5
Focus, depressed	0	1	0	1	0	0	0	0
**TESTIS**								
Submitted	5	5	5	5	.	.	.	.
No Visible Lesions	5	5	5	4	.	.	.	.
Small	0	0	0	1	.	.	.	.
**THYMUS**								
Submitted	5	5	5	5	5	5	5	5
No Visible Lesions	4	5	4	5	4	5	5	4
Focus, dark	1	0	0	0	1	0	0	1
Discoloration, mottled	0	0	1	0	0	0	0	0
**TONGUE**								
Submitted	5	5	5	5	5	5	5	5
No Visible Lesions	5	5	4	5	5	5	5	5
Focus, dark	0	0	1	0	0	0	0	0
**TRACHEA**								
Submitted	5	5	5	5	5	5	5	5
No Visible Lesions	5	5	5	5	5	5	5	5
**URETER**								
Submitted	5	5	5	5	5	5	5	5
No Visible Lesions	5	5	5	5	5	5	5	5
**URINARY BLADDER**								
Submitted	5	5	5	5	5	5	5	5
No Visible Lesions	5	5	5	5	5	5	5	5
**UTERUS**								
Submitted	.	.	.	.	5	5	5	5
No Visible Lesions	.	.	.	.	5	5	5	5

Appendix 18

Table 1

Summary of Macroscopic Pathology: Day 91

5550008

Removal Reason(s): RECOVERY EUTHANASIA	Male				Female			
Summary: Incidence	0 vg Group 1	0.36x 10E12 vg Group 2	1.1x 10E12 vg Group 3	3.3x 10E12 vg Group 4	0 vg Group 1	0.36x 10E12 vg Group 2	1.1x 10E12 vg Group 3	3.3x 10E12 vg Group 4
Number of Animals:	5	5	5	5	5	5	5	5
**VAGINA**								
Submitted	.	.	.	.	4	5	5	5
No Visible Lesions	.	.	.	.	4	5	5	5
Not Examined: Lost During Necropsy.	.	.	.	.	1	0	0	0
**WHOLE ANIMAL**								
Decreased adipose tissue	.	.	.	.	.	.	.	1

Appendix 18

Table 2

Summary of Absolute Organ Weights Explanation Page

Abbreviation	Description
Lt	Left
Rt	Right

Note: This is a comprehensive list of abbreviations. All of the abbreviations listed may not be applicable to this report.

Dosing Information

Dosing information is abbreviated on various data outputs; the following represents the dosing information for this study.

Group No.	Test Material	Dose Level (vg)
1	Reference Item	0
2	AAV9/AP4M1	3.6E11
3	AAV9/AP4M1	1.1E12
4	AAV9/AP4M1	3.3E12

Appendix 18

Table 2

Summary of Absolute Organ Weights: Day 8

5550008

Sex: Male / Day(s) Relative to Start Date		0 vg Group 1	0.36x 10E12 vg Group 2	1.1x 10E12 vg Group 3	3.3x 10E12 vg Group 4
Terminal Body Weight (g) [G]	Mean	244.3	243.6	232.4	213.2
	SD	16.5	38.6	23.5	28.4
	N	4	5	5	5
	%Diff	-	-0.3	-4.9	-12.7
Brain Weight (g) [G1]	Mean	1.9155	1.9416	1.9154	1.8622
	SD	0.0592	0.1069	0.0730	0.0830
	N	4	5	5	5
	%Diff	-	1.3626	-0.0052	-2.7826
Epididymis Weight (g) [G1]	Mean	0.4753	0.5048	0.4786	0.4364
	SD	0.0855	0.1054	0.0818	0.0581
	N	4	5	5	5
	%Diff	-	6.2178	0.7049	-8.1746
Gland, Adrenal Weight (g) [G1]	Mean	0.04175	0.04358	0.03934	0.03684
	SD	0.00203	0.00721	0.00820	0.00477
	N	4	5	5	5
	%Diff	-	4.38323	-5.77246	-11.76048
Gland, Pituitary Weight (g) [G1]	Mean	0.01020	0.01078	0.00888	0.00906
	SD	0.00179	0.00191	0.00213	0.00205
	N	4	5	5	5
	%Diff	-	5.68627	-12.94118	-11.17647
Gland, Prostate Weight (g) [G2]	Mean	0.4430	0.4720	0.4892	0.4274
	SD	0.0493	0.0728	0.0572	0.1393
	N	4	5	5	5
	%Diff	-	6.5463	10.4289	-3.5214
Thyroid/Parathyroid Weight (g) [G1]	Mean	0.01173	0.01068	0.00894	0.01192
	SD	0.00302	0.00235	0.00091	0.00043
	N	4	5	5	5
	%Diff	-	-8.91258	-23.75267	1.66311
Heart Weight (g) [G1]	Mean	0.9050	0.9848	0.9634	0.8496
	SD	0.0373	0.1897	0.1465	0.0948
	N	4	5	5	5
	%Diff	-	8.8177	6.4530	-6.1215

[G] - Anova & Dunnett
[G1] - Anova & Dunnett
[G2] - Kruskal-Wallis & Dunn

Appendix 18

Table 2

Summary of Absolute Organ Weights: Day 8

5550008

Sex: Male<br>Day(s) Relative to Start Date		0<br>vg<br>Group 1	0.36x<br>10E12<br>vg<br>Group 2	1.1x<br>10E12<br>vg<br>Group 3	3.3x<br>10E12<br>vg<br>Group 4
Kidney Weight (g) [G]	Mean	2.1315	2.1116	1.9834	1.8522
	SD	0.1625	0.4313	0.2094	0.2820
	N	4	5	5	5
	%Diff	-	-0.9336	-6.9482	-13.1034
Liver Weight (g) [G]	Mean	8.1058	8.1104	7.7578	6.9066
	SD	0.2924	1.4426	0.7657	1.0128
	N	4	5	5	5
	%Diff	-	0.0574	-4.2926	-14.7938
Spleen Weight (g) [G1]	Mean	0.6613	0.5812	0.5558	0.5352
	SD	0.0791	0.1216	0.0635	0.1291
	N	4	5	5	5
	%Diff	-	-12.1059	-15.9471	-19.0624
Testis Weight (g) [G1]	Mean	2.8945	2.8030	2.7476	2.6360
	SD	0.0437	0.3400	0.1505	0.2506
	N	4	5	5	5
	%Diff	-	-3.1612	-5.0751	-8.9307
Thymus Weight (g) [G]	Mean	0.5508	0.5498	0.5524	0.5060
	SD	0.0523	0.0949	0.0738	0.1478
	N	4	5	5	5
	%Diff	-	-0.1725	0.2996	-8.1253

[G] - Anova & Dunnett
[G1] - Kruskal-Wallis & Dunn

Appendix 18

Table 2

Summary of Absolute Organ Weights: Day 8

5550008

Sex: Female<br>Day(s) Relative to Start Date		0<br>vg<br>Group 1	0.36x<br>10E12<br>vg<br>Group 2	1.1x<br>10E12<br>vg<br>Group 3	3.3x<br>10E12<br>vg<br>Group 4
Terminal Body Weight (g) [G]	Mean	166.0	181.0	179.8	167.0
	SD	6.4	19.9	6.6	11.0
	N	5	5	5	5
	%Diff	-	9.0	8.3	0.6
Brain Weight (g) [G1]	Mean	1.7774	1.7720	1.8272	1.7364
	SD	0.0928	0.0929	0.0691	0.1985
	N	5	5	5	5
	%Diff	-	-0.3038	2.8018	-2.3067
Gland, Adrenal Weight (g) [G1]	Mean	0.05500	0.05410	0.05878	0.04936
	SD	0.00834	0.00654	0.01161	0.00615
	N	5	5	5	5
	%Diff	-	-1.63636	6.87273	-10.25455
Gland, Pituitary Weight (g) [G1]	Mean	0.01328	0.01266	0.01310	0.01248
	SD	0.00127	0.00271	0.00262	0.00195
	N	5	5	5	5
	%Diff	-	-4.66867	-1.35542	-6.02410
Thyroid/Parathyroid Weight (g) [G1]	Mean	0.00860	0.00816	0.00858	0.00730
	SD	0.00159	0.00153	0.00147	0.00133
	N	5	5	5	5
	%Diff	-	-5.11628	-0.23256	-15.11628
Heart Weight (g) [G1]	Mean	0.6746	0.7142	0.7222	0.6724
	SD	0.0279	0.0786	0.0581	0.0726
	N	5	5	5	5
	%Diff	-	5.8701	7.0560	-0.3261
Kidney Weight (g) [G2]	Mean	1.4852	1.5000	1.5788	1.3930
	SD	0.0371	0.2102	0.0752	0.0795
	N	5	5	5	5
	%Diff	-	0.9965	6.3022	-6.2079
Liver Weight (g) [G1]	Mean	5.8422	5.9552	5.5008	5.4646
	SD	0.5901	0.7982	0.4991	0.5229
	N	5	5	5	5
	%Diff	-	1.9342	-5.8437	-6.4633

[G] - Kruskal-Wallis & Dunn
[G1] - Anova & Dunnett
[G2] - Kruskal-Wallis & Dunn

Appendix 18

Table 2

Summary of Absolute Organ Weights: Day 8

5550008

Sex: Female<br>Day(s) Relative to Start Date		0 vg Group 1	0.36x 10E12 vg Group 2	1.1x 10E12 vg Group 3	3.3x 10E12 vg Group 4
Ovary Weight (g) [G]	Mean	0.0712	0.0700	0.0806	0.0662
	SD	0.0068	0.0157	0.0092	0.0108
	N	5	5	5	5
	%Diff	-	-1.6854	13.2022	-7.0225
Spleen Weight (g) [G]	Mean	0.3942	0.4378	0.4262	0.3556
	SD	0.0293	0.0874	0.0422	0.0401
	N	5	5	5	5
	%Diff	-	11.0604	8.1177	-9.7920
Thymus Weight (g) [G]	Mean	0.4192	0.4676	0.3594	0.3598
	SD	0.0505	0.0434	0.0335	0.0920
	N	5	5	5	5
	%Diff	-	11.5458	-14.2653	-14.1698
Uterus/Cervix (g) [G1]	Mean	0.4692	0.5944	0.5638	0.6600
	SD	0.0801	0.3516	0.1829	0.2758
	N	5	5	5	5
	%Diff	-	26.6837	20.1620	40.6650

[G] - Anova & Dunnett
[G1] - Kruskal-Wallis & Dunn

Appendix 18

Table 2

Summary of Absolute Organ Weights: Day 29

5550008

Sex: Male<br>Day(s) Relative to Start Date		0<br>vg<br>Group 1	0.36x<br>10E12<br>vg<br>Group 2	1.1x<br>10E12<br>vg<br>Group 3	3.3x<br>10E12<br>vg<br>Group 4
Terminal Body Weight (g) [G]	Mean	368.8	369.0	356.0	399.8
	SD	23.7	15.7	25.4	71.9
	N	5	5	5	5
	%Diff	-	0.1	-3.5	8.4
Brain Weight (g) [G1]	Mean	2.0286	2.1434	2.0150	2.0596
	SD	0.1068	0.0560	0.0725	0.0890
	N	5	5	5	5
	%Diff	-	5.6591	-0.6704	1.5281
Epididymis Weight (g) [G1]	Mean	0.9342	0.9658	0.9378	0.9480
	SD	0.0888	0.0681	0.1401	0.1265
	N	5	5	5	5
	%Diff	-	3.3826	0.3854	1.4772
Gland, Adrenal Weight (g) [G2]	Mean	0.05752	0.05672	0.05328	0.06024
	SD	0.00790	0.00504	0.00516	0.01662
	N	5	5	5	5
	%Diff	-	-1.39082	-7.37135	4.72879
Gland, Pituitary Weight (g) [G1]	Mean	0.01188	0.01174	0.01260	0.01474
	SD	0.00392	0.00298	0.00092	0.00291
	N	5	5	5	5
	%Diff	-	-1.17845	6.06061	24.07407
Gland, Prostate Weight (g) [G1]	Mean	0.9208	0.9394	0.8682	0.8604
	SD	0.1928	0.1184	0.2333	0.2304
	N	5	5	5	5
	%Diff	-	2.0200	-5.7124	-6.5595
Thyroid/Parathyroid Weight (g) [G1]	Mean	0.01546	0.01562	0.01316	0.01684
	SD	0.00346	0.00565	0.00303	0.00600
	N	5	5	5	5
	%Diff	-	1.03493	-14.87710	8.92626
Heart Weight (g) [G2]	Mean	1.2262	1.3714	1.2648	1.3504
	SD	0.0997	0.0797	0.0888	0.1690
	N	5	5	5	5
	%Diff	-	11.8415	3.1479	10.1289

[G] - Kruskal-Wallis & Dunn
[G1] - Anova & Dunnett
[G2] - Kruskal-Wallis & Dunn

Appendix 18

Table 2

Summary of Absolute Organ Weights: Day 29

5550008

Sex: Male<br>Day(s) Relative to Start Date		0<br>vg<br>Group 1	0.36x<br>10E12<br>vg<br>Group 2	1.1x<br>10E12<br>vg<br>Group 3	3.3x<br>10E12<br>vg<br>Group 4
Kidney Weight (g) [G]	Mean	2.6012	2.6598	2.4050	2.9332
	SD	0.0977	0.1108	0.1467	0.3363
	N	5	5	5	5
	%Diff	-	2.2528	-7.5427	12.7633
Liver Weight (g) [G]	Mean	9.7726	10.8282	10.1248	12.2260
	SD	0.5281	0.9457	0.7371	2.6804
	N	5	5	5	5
	%Diff	-	10.8016	3.6040	25.1049
Spleen Weight (g) [G1]	Mean	0.8230	0.7544	0.6714	0.8324
	SD	0.2267	0.0784	0.0379	0.2119
	N	5	5	5	5
	%Diff	-	-8.3354	-18.4204	1.1422
Testis Weight (g) [G1]	Mean	3.3422	3.3910	3.2744	3.5246
	SD	0.4718	0.3017	0.6169	0.2840
	N	5	5	5	5
	%Diff	-	1.4601	-2.0286	5.4575
Thymus Weight (g) [G1]	Mean	0.5618	0.5598	0.5028	0.5734
	SD	0.1144	0.1630	0.0418	0.2274
	N	5	5	5	5
	%Diff	-	-0.3560	-10.5020	2.0648

[G] - Kruskal-Wallis & Dunn
[G1] - Anova & Dunnett

Appendix 18

Table 2

Summary of Absolute Organ Weights: Day 29

5550008

Sex: Female<br>Day(s) Relative to Start Date		0<br>vg<br>Group 1	0.36x<br>10E12<br>vg<br>Group 2	1.1x<br>10E12<br>vg<br>Group 3	3.3x<br>10E12<br>vg<br>Group 4
Terminal Body Weight (g) [G]	Mean	200.2	232.2	210.4	215.2
	SD	18.6	18.1	18.1	25.2
	N	5	5	5	5
	%Diff	-	16.0	5.1	7.5
Brain Weight (g) [G1]	Mean	1.8264	1.9330	1.8680	1.8308
	SD	0.0976	0.0907	0.0686	0.0763
	N	5	5	5	5
	%Diff	-	5.8366	2.2777	0.2409
Gland, Adrenal Weight (g) [G1]	Mean	0.05444	0.06326	0.06036	0.05880
	SD	0.00514	0.00477	0.01069	0.00930
	N	5	5	5	5
	%Diff	-	16.20132	10.87436	8.00882
Gland, Pituitary Weight (g) [G1]	Mean	0.01292	0.01608	0.01482	0.01542
	SD	0.00377	0.00220	0.00108	0.00172
	N	5	5	5	5
	%Diff	-	24.45820	14.70588	19.34985
Thyroid/Parathyroid Weight (g) [G1]	Mean	0.01140	0.01186	0.01288	0.01142
	SD	0.00189	0.00147	0.00236	0.00210
	N	5	5	5	5
	%Diff	-	4.03509	12.98246	0.17544
Heart Weight (g) [G1]	Mean	0.7410	0.8604	0.7854	0.8442
	SD	0.0735	0.0758	0.0497	0.0832
	N	5	5	5	5
	%Diff	-	16.1134	5.9919	13.9271
Kidney Weight (g) [G1]	Mean	1.4000	1.6344	1.4734	1.5468
	SD	0.1572	0.1496	0.2124	0.1145
	N	5	5	5	5
	%Diff	-	16.7429	5.2429	10.4857
Liver Weight (g) [G1]	Mean	5.4676	6.6616 **	6.2192	6.3630
	SD	0.5550	0.4067	0.5451	0.6673
	N	5	5	5	5
	%Diff	-	21.8377	13.7464	16.3765

[G] - Anova & Dunnett
[G1] - Anova & Dunnett: ** = $p \leq 0.01$

Appendix 18

Table 2

Summary of Absolute Organ Weights: Day 29

5550008

Sex: Female<br>Day(s) Relative to Start Date		0 vg<br>Group 1	0.36x 10E12 vg<br>Group 2	1.1x 10E12 vg<br>Group 3	3.3x 10E12 vg<br>Group 4
Ovary Weight (g) [G]	Mean	0.0802	0.0998	0.1088	0.0944
	SD	0.0135	0.0090	0.0386	0.0198
	N	5	5	5	5
	%Diff	-	24.4389	35.6608	17.7057
Spleen Weight (g) [G]	Mean	0.4246	0.4978	0.4604	0.4666
	SD	0.0437	0.1225	0.0946	0.0798
	N	5	5	5	5
	%Diff	-	17.2398	8.4315	9.8917
Thymus Weight (g) [G]	Mean	0.3400	0.4162	0.3986	0.4120
	SD	0.0676	0.0748	0.1258	0.1465
	N	5	5	5	5
	%Diff	-	22.4118	17.2353	21.1765
Uterus/Cervix (g) [G]	Mean	0.8168	0.4990 *	0.5448 *	0.4318 **
	SD	0.2967	0.1107	0.0713	0.0503
	N	5	5	5	5
	%Diff	-	-38.9079	-33.3007	-47.1352

[G] - Anova & Dunnett: * = p ≤ 0.05; ** = p ≤ 0.01

Appendix 18

Table 2

Summary of Absolute Organ Weights: Day 91

5550008

Sex: Male<br>Day(s) Relative to Start Date		0<br>vg<br>Group 1	0.36x<br>10E12<br>vg<br>Group 2	1.1x<br>10E12<br>vg<br>Group 3	3.3x<br>10E12<br>vg<br>Group 4
Terminal Body Weight (g) [G]	Mean	613.2	619.2	578.4	510.4 *
	SD	75.8	33.7	67.2	51.5
	N	5	5	5	5
	%Diff	-	1.0	-5.7	-16.8
Brain Weight (g) [G1]	Mean	2.2010	2.2454	2.2470	2.2136
	SD	0.0413	0.1007	0.0880	0.0630
	N	5	5	5	5
	%Diff	-	2.0173	2.0900	0.5725
Epididymis Weight (g) [G2]	Mean	1.4210	1.3378	1.3326	1.1770
	SD	0.0717	0.2636	0.0376	0.2267
	N	5	5	5	5
	%Diff	-	-5.8550	-6.2210	-17.1710
Gland, Adrenal Weight (g) [G1]	Mean	0.04782	0.05778	0.05042	0.05202
	SD	0.00970	0.00828	0.00730	0.00826
	N	5	5	5	5
	%Diff	-	20.82811	5.43706	8.78294
Gland, Pituitary Weight (g) [G1]	Mean	0.01704	0.01590	0.01572	0.01400
	SD	0.00317	0.00260	0.00206	0.00204
	N	5	5	5	5
	%Diff	-	-6.69014	-7.74648	-17.84038
Gland, Prostate Weight (g) [G1]	Mean	1.4924	1.4838	1.3474	1.5584
	SD	0.1761	0.1195	0.3360	0.2504
	N	5	5	5	5
	%Diff	-	-0.5763	-9.7159	4.4224
Thyroid/Parathyroid Weight (g) [G1]	Mean	0.02228	0.02062	0.01894	0.01820
	SD	0.00578	0.00440	0.00254	0.00406
	N	5	5	5	5
	%Diff	-	-7.45063	-14.99102	-18.31239
Heart Weight (g) [G1]	Mean	1.7454	1.7360	1.6658	1.5144
	SD	0.2442	0.0962	0.2474	0.1417
	N	5	5	5	5
	%Diff	-	-0.5386	-4.5606	-13.2348

[G] - Anova & Dunnett: * = p ≤ 0.05
[G1] - Anova & Dunnett
[G2] - Kruskal-Wallis & Dunn

Appendix 18

Table 2

Summary of Absolute Organ Weights: Day 91

5550008

Sex: Male<br>Day(s) Relative to Start Date		0 vg<br>Group 1	0.36x 10E12 vg<br>Group 2	1.1x 10E12 vg<br>Group 3	3.3x 10E12 vg<br>Group 4
Kidney Weight (g) [G]	Mean	3.2748	3.1788	3.0068	2.9108
	SD	0.3966	0.2214	0.2981	0.4117
	N	5	5	5	5
	%Diff	-	-2.9315	-8.1837	-11.1152
Liver Weight (g) [G]	Mean	15.2410	14.3168	13.3344	11.9126
	SD	4.1853	0.8773	2.0717	1.5645
	N	5	5	5	5
	%Diff	-	-6.0639	-12.5097	-21.8385
Spleen Weight (g) [G]	Mean	0.9404	0.9320	0.8344	0.8470
	SD	0.0809	0.1475	0.0857	0.1900
	N	5	5	5	5
	%Diff	-	-0.8932	-11.2718	-9.9319
Testis Weight (g) [G1]	Mean	3.8728	3.7756	3.7296	3.2066
	SD	0.3141	0.4625	0.2820	0.7677
	N	5	5	5	5
	%Diff	-	-2.5098	-3.6976	-17.2020
Thymus Weight (g) [G]	Mean	0.2740	0.2826	0.3334	0.2730
	SD	0.0531	0.0435	0.0959	0.0831
	N	5	5	5	5
	%Diff	-	3.1387	21.6788	-0.3650

[G] - Anova & Dunnett
[G1] - Kruskal-Wallis & Dunn

Appendix 18

Table 2

Summary of Absolute Organ Weights: Day 91

5550008

Sex: Female<br>Day(s) Relative to Start Date		0 vg<br>Group 1	0.36x 10E12 vg<br>Group 2	1.1x 10E12 vg<br>Group 3	3.3x 10E12 vg<br>Group 4
Terminal Body Weight (g) [G]	Mean	269.0	288.6	283.0	266.8
	SD	23.9	32.3	49.1	27.1
	N	5	5	5	5
	%Diff	-	7.3	5.2	-0.8
Brain Weight (g) [G1]	Mean	1.9784	1.9500	2.0202	1.9430
	SD	0.0996	0.1011	0.0848	0.0691
	N	5	4	5	5
	%Diff	-	-1.4355	2.1128	-1.7893
Gland, Adrenal Weight (g) [G1]	Mean	0.06708	0.06182	0.05724	0.05802
	SD	0.00850	0.00768	0.01146	0.00832
	N	5	5	5	5
	%Diff	-	-7.84138	-14.66905	-13.50626
Gland, Pituitary Weight (g) [G1]	Mean	0.01802	0.01858	0.01984	0.02198
	SD	0.00298	0.00288	0.00339	0.00514
	N	5	5	5	5
	%Diff	-	3.10766	10.09989	21.97558
Thyroid/Parathyroid Weight (g) [G1]	Mean	0.01302	0.01454	0.01360	0.01150
	SD	0.00247	0.00400	0.00155	0.00302
	N	5	5	5	5
	%Diff	-	11.67435	4.45469	-11.67435
Heart Weight (g) [G1]	Mean	0.9174	0.9412	0.9882	0.9556
	SD	0.1019	0.0481	0.1725	0.0926
	N	5	5	5	5
	%Diff	-	2.5943	7.7175	4.1639
Kidney Weight (g) [G1]	Mean	1.6450	1.7714	1.6606	1.6822
	SD	0.1879	0.2059	0.2446	0.1289
	N	5	5	5	5
	%Diff	-	7.6839	0.9483	2.2614
Liver Weight (g) [G1]	Mean	6.4214	6.9626	6.9188	6.7382
	SD	0.7062	0.8436	0.8980	0.6006
	N	5	5	5	5
	%Diff	-	8.4281	7.7460	4.9335

[G] - Anova & Dunnett
[G1] - Anova & Dunnett

Appendix 18

Table 2

Summary of Absolute Organ Weights: Day 91

5550008

Sex: Female<br>Day(s) Relative to Start Date		0<br>vg<br>Group 1	0.36x<br>10E12<br>vg<br>Group 2	1.1x<br>10E12<br>vg<br>Group 3	3.3x<br>10E12<br>vg<br>Group 4
Ovary Weight (g) [G]	Mean	0.0816	0.0872	0.0844	0.0670
	SD	0.0078	0.0094	0.0258	0.0122
	N	5	5	5	5
	%Diff	-	6.8627	3.4314	-17.8922
Spleen Weight (g) [G]	Mean	0.5638	0.4670	0.5022	0.5046
	SD	0.0732	0.0726	0.0703	0.0753
	N	5	5	5	5
	%Diff	-	-17.1692	-10.9259	-10.5002
Thymus Weight (g) [G]	Mean	0.2334	0.1780	0.2248	0.2174
	SD	0.0400	0.0129	0.0496	0.0447
	N	5	5	5	5
	%Diff	-	-23.7361	-3.6847	-6.8552
Uterus/Cervix (g) [G1]	Mean	0.5794	0.6534	1.1830	0.8834
	SD	0.1195	0.2510	0.9220	0.2718
	N	5	5	5	5
	%Diff	-	12.7718	104.1767	52.4681

[G] - Anova & Dunnett
[G1] - Kruskal-Wallis & Dunn

Appendix 18
Table 3

Summary of Organ Weights Relative to Body Weight Explanation Page

Dosing Information

Dosing information is abbreviated on various data outputs; the following represents the dosing information for this study.

Group No.	Test Material	Dose Level (vg)
1	Reference Item	0
2	AAV9/AP4M1	3.6E11
3	AAV9/AP4M1	1.1E12
4	AAV9/AP4M1	3.3E12

Appendix 18

Table 3

Summary of Organ Weights Relative to Body Weight: Day 8

5550008

Sex: Male<br>Day(s) Relative to Start Date		0 vg Group 1	0.36x 10E12 vg Group 2	1.1x 10E12 vg Group 3	3.3x 10E12 vg Group 4
Brain (%) [G]	Mean	0.78687	0.81115	0.82884	0.88630
	SD	0.05627	0.11668	0.05997	0.12577
	N	4	5	5	5
	%Diff	-	3.08517	5.33379	12.63526
Epididymis (%) [G]	Mean	0.19388	0.20791	0.20538	0.20710
	SD	0.02590	0.03665	0.02508	0.03600
	N	4	5	5	5
	%Diff	-	7.23415	5.92932	6.81807
Gland, Adrenal (%) [G]	Mean	0.01716	0.01792	0.01688	0.01753
	SD	0.00166	0.00139	0.00252	0.00327
	N	4	5	5	5
	%Diff	-	4.40669	-1.67899	2.13459
Gland, Pituitary (%) [G]	Mean	0.00418	0.00442	0.00378	0.00422
	SD	0.00070	0.00020	0.00059	0.00042
	N	4	5	5	5
	%Diff	-	5.72845	-9.46368	0.93758
Gland, Prostate (%) [G]	Mean	0.18217	0.19737	0.21068	0.20048
	SD	0.02615	0.04018	0.01762	0.06204
	N	4	5	5	5
	%Diff	-	8.34549	15.65491	10.05273
Thyroid/Parathyroid (%) [G]	Mean	0.00486	0.00455	0.00388	0.00566
	SD	0.00152	0.00169	0.00059	0.00065
	N	4	5	5	5
	%Diff	-	-6.39195	-20.07331	16.51440
Heart (%) [G]	Mean	0.37170	0.40297	0.41306	0.39955
	SD	0.02718	0.02409	0.02521	0.01817
	N	4	5	5	5
	%Diff	-	8.41371	11.12841	7.49392
Kidney (%) [G]	Mean	0.87338	0.86244	0.85354	0.86806
	SD	0.05203	0.05209	0.03033	0.07045
	N	4	5	5	5
	%Diff	-	-1.25193	-2.27192	-0.60896

[G] - Anova & Dunnett

Appendix 18

Table 3

Summary of Organ Weights Relative to Body Weight: Day 8

5550008

Sex: Male<br>Day(s) Relative to Start Date		0<br>vg<br>Group 1	0.36x<br>10E12<br>vg<br>Group 2	1.1x<br>10E12<br>vg<br>Group 3	3.3x<br>10E12<br>vg<br>Group 4
Liver (%) [G]	Mean	3.32619	3.32647	3.34273	3.23770
	SD	0.17586	0.18987	0.17423	0.22354
	N	4	5	5	5
	%Diff	-	0.00859	0.49730	-2.66028
Spleen (%) [G1]	Mean	0.27015	0.23822	0.23981	0.25117
	SD	0.01794	0.02941	0.02492	0.05495
	N	4	5	5	5
	%Diff	-	-11.82195	-11.23397	-7.02713
Testis (%) [G]	Mean	1.18898	1.16749	1.18979	1.24753
	SD	0.08066	0.17103	0.10854	0.15103
	N	4	5	5	5
	%Diff	-	-1.80761	0.06797	4.92395
Thymus (%) [G]	Mean	0.22718	0.22855	0.24127	0.23564
	SD	0.03577	0.03911	0.05242	0.04905
	N	4	5	5	5
	%Diff	-	0.60396	6.20323	3.72145

[G] - Anova & Dunnett
[G1] - Kruskal-Wallis & Dunn

Appendix 18

Table 3

Summary of Organ Weights Relative to Body Weight: Day 8

5550008

Sex: Female<br>Day(s) Relative to Start Date		0<br>vg<br>Group 1	0.36x<br>10E12<br>vg<br>Group 2	1.1x<br>10E12<br>vg<br>Group 3	3.3x<br>10E12<br>vg<br>Group 4
Brain (%) [G]	Mean	1.07236	0.98544	1.01666	1.03929
	SD	0.07689	0.08044	0.03437	0.09383
	N	5	5	5	5
	%Diff	-	-8.10535	-5.19440	-3.08419
Gland, Adrenal (%) [G1]	Mean	0.03312	0.02990	0.03268	0.02950
	SD	0.00472	0.00185	0.00621	0.00241
	N	5	5	5	5
	%Diff	-	-9.74384	-1.33253	-10.92666
Gland, Pituitary (%) [G]	Mean	0.00800	0.00705	0.00729	0.00745
	SD	0.00062	0.00155	0.00142	0.00079
	N	5	5	5	5
	%Diff	-	-11.83780	-8.84676	-6.87209
Thyroid/Parathyroid (%) [G]	Mean	0.00517	0.00455	0.00478	0.00438
	SD	0.00087	0.00103	0.00084	0.00073
	N	5	5	5	5
	%Diff	-	-12.04405	-7.62046	-15.39124
Heart (%) [G]	Mean	0.40667	0.39509	0.40182	0.40197
	SD	0.01764	0.02300	0.03104	0.02284
	N	5	5	5	5
	%Diff	-	-2.84774	-1.19225	-1.15543
Kidney (%) [G]	Mean	0.89575	0.82736*	0.87859	0.83511
	SD	0.04050	0.04365	0.04352	0.03514
	N	5	5	5	5
	%Diff	-	-7.63545	-1.91527	-6.76964
Liver (%) [G]	Mean	3.51743	3.29617	3.05983	3.26747
	SD	0.29761	0.33945	0.26592	0.10496
	N	5	5	5	5
	%Diff	-	-6.29024	-13.00953	-7.10644
Ovary (%) [G]	Mean	0.04295	0.03834	0.04476	0.03965
	SD	0.00446	0.00530	0.00393	0.00631
	N	5	5	5	5
	%Diff	-	-10.73315	4.21607	-7.66813

[G] - Anova & Dunnett: * = $p \leq 0.05$
[G1] - Kruskal-Wallis & Dunn

Appendix 18

Table 3

Summary of Organ Weights Relative to Body Weight: Day 8

5550008

Sex: Female<br><br>Day(s) Relative to Start Date		0<br>vg<br><br>Group 1	0.36x<br>10E12<br>vg<br>Group 2	1.1x<br>10E12<br>vg<br>Group 3	3.3x<br>10E12<br>vg<br>Group 4
Spleen (%) [G]	Mean	0.23795	0.24025	0.23765	0.21257
	SD	0.02246	0.02431	0.02881	0.01337
	N	5	5	5	5
	%Diff	-	0.96631	-0.12257	-10.66611
Thymus (%) [G]	Mean	0.25374	0.26024	0.20010	0.21477
	SD	0.04098	0.03072	0.02052	0.05223
	N	5	5	5	5
	%Diff	-	2.56065	-21.13948	-15.35944
Uterus/Cervix (%) - [G1]	Mean	0.28285	0.32979	0.31157	0.39723
	SD	0.04838	0.19101	0.09394	0.16547
	N	5	5	5	5
	%Diff	-	16.59435	10.15483	40.43978

[G] - Anova & Dunnett
[G1] - Kruskal-Wallis & Dunn

Appendix 18

Table 3

Summary of Organ Weights Relative to Body Weight: Day 29

5550008

Sex: Male<br>Day(s) Relative to Start Date		0 vg<br>Group 1	0.36x 10E12 vg<br>Group 2	1.1x 10E12 vg<br>Group 3	3.3x 10E12 vg<br>Group 4
Brain (%) [G]	Mean	0.55114	0.58178	0.56805	0.52553
	SD	0.03306	0.03019	0.04030	0.07310
	N	5	5	5	5
	%Diff	-	5.55838	3.06875	-4.64681
Epididymis (%) [G1]	Mean	0.25335	0.26255	0.26225	0.23982
	SD	0.01974	0.02742	0.02248	0.02796
	N	5	5	5	5
	%Diff	-	3.63104	3.51312	-5.34103
Gland, Adrenal (%) [G1]	Mean	0.01555	0.01543	0.01500	0.01494
	SD	0.00134	0.00192	0.00143	0.00206
	N	5	5	5	5
	%Diff	-	-0.75524	-3.53103	-3.91024
Gland, Pituitary (%) [G1]	Mean	0.00319	0.00320	0.00354	0.00369
	SD	0.00096	0.00086	0.00014	0.00032
	N	5	5	5	5
	%Diff	-	0.30649	10.95428	15.57443
Gland, Prostate (%) [G1]	Mean	0.24858	0.25424	0.24278	0.21275
	SD	0.04489	0.02707	0.05658	0.02184
	N	5	5	5	5
	%Diff	-	2.27533	-2.33340	-14.41268
Thyroid/Parathyroid (%) [G1]	Mean	0.00419	0.00423	0.00367	0.00418
	SD	0.00086	0.00151	0.00066	0.00117
	N	5	5	5	5
	%Diff	-	0.93917	-12.25349	-0.11841
Heart (%) [G1]	Mean	0.33348	0.37266	0.35547	0.34045
	SD	0.03371	0.03374	0.01228	0.02037
	N	5	5	5	5
	%Diff	-	11.74638	6.59297	2.09020
Kidney (%) [G1]	Mean	0.70657	0.72169	0.67734	0.74139
	SD	0.02985	0.03838	0.04905	0.06176
	N	5	5	5	5
	%Diff	-	2.14015	-4.13655	4.92869

[G] - Kruskal-Wallis & Dunn
[G1] - Anova & Dunnett

Appendix 18

Table 3

Summary of Organ Weights Relative to Body Weight: Day 29

5550008

Sex: Male<br>Day(s) Relative to Start Date		0<br>vg<br>Group 1	0.36x<br>10E12<br>vg<br>Group 2	1.1x<br>10E12<br>vg<br>Group 3	3.3x<br>10E12<br>vg<br>Group 4
Liver (%) [G]	Mean	2.65564	2.93556	2.84894	3.04447*
	SD	0.17570	0.24346	0.19736	0.17322
	N	5	5	5	5
	%Diff	-	10.54059	7.27882	14.64148
Spleen (%) [G]	Mean	0.22123	0.20503	0.18898	0.20858
	SD	0.04856	0.02579	0.01018	0.03337
	N	5	5	5	5
	%Diff	-	-7.32001	-14.57865	-5.71768
Testis (%) [G]	Mean	0.90764	0.92044	0.91420	0.89476
	SD	0.12788	0.09008	0.12613	0.09592
	N	5	5	5	5
	%Diff	-	1.40934	0.72187	-1.41991
Thymus (%) [G]	Mean	0.15302	0.15164	0.14181	0.14157
	SD	0.03353	0.04394	0.01612	0.03761
	N	5	5	5	5
	%Diff	-	-0.90040	-7.32403	-7.47793

[G] - Anova & Dunnett: * = $p \leq 0.05$

Appendix 18

Table 3

Summary of Organ Weights Relative to Body Weight: Day 29

5550008

Sex: Female<br>Day(s) Relative to Start Date		0<br>vg<br>Group 1	0.36x<br>10E12<br>vg<br>Group 2	1.1x<br>10E12<br>vg<br>Group 3	3.3x<br>10E12<br>vg<br>Group 4
Brain (%) [G]	Mean	0.91532	0.83656	0.89251	0.85744
	SD	0.04686	0.07493	0.07707	0.07573
	N	5	5	5	5
	%Diff	-	-8.60524	-2.49206	-6.32310
Gland, Adrenal (%) [G]	Mean	0.02721	0.02732	0.02860	0.02746
	SD	0.00129	0.00208	0.00343	0.00426
	N	5	5	5	5
	%Diff	-	0.38948	5.10231	0.90730
Gland, Pituitary (%) [G]	Mean	0.00645	0.00700	0.00709	0.00721
	SD	0.00189	0.00139	0.00086	0.00084
	N	5	5	5	5
	%Diff	-	8.54283	9.85817	11.73858
Thyroid/Parathyroid (%) [G]	Mean	0.00568	0.00513	0.00618	0.00539
	SD	0.00063	0.00076	0.00137	0.00133
	N	5	5	5	5
	%Diff	-	-9.56462	8.95154	-5.09303
Heart (%) [G]	Mean	0.37012	0.37057	0.37405	0.39305
	SD	0.01260	0.01539	0.01704	0.01325
	N	5	5	5	5
	%Diff	-	0.12175	1.06234	6.19670
Kidney (%) [G]	Mean	0.69907	0.70480	0.69838	0.72192
	SD	0.03690	0.05258	0.05828	0.03678
	N	5	5	5	5
	%Diff	-	0.82053	-0.09918	3.26836
Liver (%) [G]	Mean	2.73684	2.87770	2.96018	2.96240
	SD	0.22587	0.20132	0.20120	0.12397
	N	5	5	5	5
	%Diff	-	5.14681	8.16034	8.24144
Ovary (%) [G]	Mean	0.04033	0.04320	0.05090	0.04359
	SD	0.00742	0.00520	0.01346	0.00497
	N	5	5	5	5
	%Diff	-	7.09337	26.18478	8.08145

[G] - Anova & Dunnett

Appendix 18

Table 3

Summary of Organ Weights Relative to Body Weight: Day 29

5550008

Sex: Female<br>Day(s) Relative to Start Date		0<br>vg<br>Group 1	0.36x<br>10E12<br>vg<br>Group 2	1.1x<br>10E12<br>vg<br>Group 3	3.3x<br>10E12<br>vg<br>Group 4
Spleen (%) [G]	Mean	0.21260	0.21340	0.21741	0.21587
	SD	0.01845	0.04262	0.02723	0.01367
	N	5	5	5	5
	%Diff	-	0.37761	2.26186	1.53879
Thymus (%) [G]	Mean	0.16882	0.17966	0.18817	0.18830
	SD	0.01930	0.03222	0.05253	0.05052
	N	5	5	5	5
	%Diff	-	6.42352	11.46181	11.53984
Uterus/Cervix (%) - [G]	Mean	0.40827	0.21708**	0.26001*	0.20452**
	SD	0.13426	0.05816	0.03712	0.04589
	N	5	5	5	5
	%Diff	-	-46.82974	-36.31425	-49.90564

[G] - Anova & Dunnett: * = p ≤ 0.05; ** = p ≤ 0.01

Appendix 18
Table 3

Summary of Organ Weights Relative to Body Weight: Day 91

5550008

Sex: Male<br>Day(s) Relative to Start Date		0<br>vg<br>Group 1	0.36x<br>10E12<br>vg<br>Group 2	1.1x<br>10E12<br>vg<br>Group 3	3.3x<br>10E12<br>vg<br>Group 4
Brain (%) [G]	Mean	0.36330	0.36395	0.39156	0.43706*
	SD	0.04427	0.03257	0.03429	0.04302
	N	5	5	5	5
	%Diff	-	0.17971	7.77897	20.30426
Epididymis (%) [G1]	Mean	0.23362	0.21533	0.23259	0.23456
	SD	0.02078	0.03841	0.02469	0.05934
	N	5	5	5	5
	%Diff	-	-7.82694	-0.43898	0.40283
Gland, Adrenal (%) [G]	Mean	0.00778	0.00937	0.00880	0.01019
	SD	0.00096	0.00166	0.00152	0.00117
	N	5	5	5	5
	%Diff	-	20.50968	13.10082	31.01288
Gland, Pituitary (%) [G]	Mean	0.00277	0.00256	0.00272	0.00277
	SD	0.00023	0.00035	0.00016	0.00051
	N	5	5	5	5
	%Diff	-	-7.35428	-1.75062	0.01156
Gland, Prostate (%) [G]	Mean	0.24496	0.23971	0.23128	0.30405*
	SD	0.03222	0.01527	0.03775	0.02325
	N	5	5	5	5
	%Diff	-	-2.14626	-5.58724	24.11950
Thyroid/Parathyroid (%) [G]	Mean	0.00361	0.00332	0.00328	0.00356
	SD	0.00072	0.00063	0.00031	0.00068
	N	5	5	5	5
	%Diff	-	-8.12325	-9.15280	-1.37755
Heart (%) [G]	Mean	0.28471	0.28137	0.28821	0.29752
	SD	0.01832	0.02655	0.02861	0.02048
	N	5	5	5	5
	%Diff	-	-1.17550	1.22834	4.49820
Kidney (%) [G]	Mean	0.53496	0.51532	0.52151	0.56898
	SD	0.03637	0.05382	0.03388	0.03397
	N	5	5	5	5
	%Diff	-	-3.67262	-2.51477	6.35934

[G] - Anova & Dunnett: * = $p \leq 0.05$
[G1] - Kruskal-Wallis & Dunn

Appendix 18

Table 3

Summary of Organ Weights Relative to Body Weight: Day 91

5550008

Sex: Male<br>Day(s) Relative to Start Date		0<br>vg<br>Group 1	0.36x<br>10E12<br>vg<br>Group 2	1.1x<br>10E12<br>vg<br>Group 3	3.3x<br>10E12<br>vg<br>Group 4
Liver (%) [G]	Mean	2.45512	2.31429	2.29852	2.32905
	SD	0.34677	0.12838	0.12475	0.10084
	N	5	5	5	5
	%Diff	-	-5.73637	-6.37844	-5.13495
Spleen (%) [G]	Mean	0.15469	0.15025	0.14513	0.16558
	SD	0.01877	0.02002	0.01656	0.02936
	N	5	5	5	5
	%Diff	-	-2.87401	-6.18151	7.03623
Testis (%) [G1]	Mean	0.63646	0.60837	0.64810	0.64271
	SD	0.06896	0.05253	0.04563	0.20134
	N	5	5	5	5
	%Diff	-	-4.41211	1.82993	0.98289
Thymus (%) [G]	Mean	0.04449	0.04553	0.05866	0.05362
	SD	0.00459	0.00527	0.02059	0.01557
	N	5	5	5	5
	%Diff	-	2.32302	31.82540	20.49726

[G] - Anova & Dunnett
[G1] - Kruskal-Wallis & Dunn

Appendix 18

Table 3

Summary of Organ Weights Relative to Body Weight: Day 91

5550008

Sex: Female<br>Day(s) Relative to Start Date		0<br>vg<br>Group 1	0.36x<br>10E12<br>vg<br>Group 2	1.1x<br>10E12<br>vg<br>Group 3	3.3x<br>10E12<br>vg<br>Group 4
Brain (%) [G]	Mean	0.73880	0.68207	0.72799	0.73337
	SD	0.05748	0.09500	0.10982	0.06552
	N	5	4	5	5
	%Diff	-	-7.67918	-1.46316	-0.73444
Gland, Adrenal (%) [G]	Mean	0.02502	0.02166	0.02077	0.02191
	SD	0.00329	0.00386	0.00581	0.00350
	N	5	5	5	5
	%Diff	-	-13.43939	-17.00130	-12.42224
Gland, Pituitary (%) [G]	Mean	0.00669	0.00653	0.00722	0.00831
	SD	0.00091	0.00141	0.00191	0.00226
	N	5	5	5	5
	%Diff	-	-2.35374	7.84513	24.25478
Thyroid/Parathyroid (%) [G]	Mean	0.00483	0.00496	0.00495	0.00435
	SD	0.00069	0.00086	0.00113	0.00123
	N	5	5	5	5
	%Diff	-	2.72432	2.40557	-10.08307
Heart (%) [G]	Mean	0.34145	0.32809	0.34939	0.35906
	SD	0.02729	0.02345	0.01613	0.02666
	N	5	5	5	5
	%Diff	-	-3.91365	2.32610	5.15620
Kidney (%) [G]	Mean	0.61097	0.61701	0.59040	0.63696
	SD	0.03205	0.07017	0.05996	0.09325
	N	5	5	5	5
	%Diff	-	0.98753	-3.36780	4.25342
Liver (%) [G]	Mean	2.38385	2.42734	2.45915	2.53855
	SD	0.06051	0.30958	0.15128	0.27150
	N	5	5	5	5
	%Diff	-	1.82451	3.15882	6.48955
Ovary (%) [G]	Mean	0.03035	0.03072	0.02937	0.02536
	SD	0.00147	0.00599	0.00444	0.00587
	N	5	5	5	5
	%Diff	-	1.21828	-3.22263	-16.45001

[G] - Anova & Dunnett

Appendix 18

Table 3

Summary of Organ Weights Relative to Body Weight: Day 91

5550008

Sex: Female<br><br>Day(s) Relative to Start Date		0<br>vg<br><br>Group 1	0.36x<br>10E12<br>vg<br>Group 2	1.1x<br>10E12<br>vg<br>Group 3	3.3x<br>10E12<br>vg<br>Group 4
Spleen (%) [G]	Mean	0.20926	0.16402	0.17869	0.18949
	SD	0.01523	0.03574	0.01831	0.02473
	N	5	5	5	5
	%Diff	-	-21.61950	-14.60663	-9.44627
Thymus (%) [G]	Mean	0.08740	0.06216	0.08016	0.08224
	SD	0.01706	0.00674	0.01704	0.01824
	N	5	5	5	5
	%Diff	-	-28.88399	-8.28979	-5.90716
Uterus/Cervix (%) - [G1]	Mean	0.21757	0.23136	0.44022	0.33447
	SD	0.05374	0.10218	0.36258	0.11650
	N	5	5	5	5
	%Diff	-	6.34092	102.33940	53.73154

[G] - Anova & Dunnett
[G1] - Kruskal-Wallis & Dunn

Appendix 18

Table 4

Summary of Organ Weights Relative to Brain Weight Explanation Page

Dosing Information

Dosing information is abbreviated on various data outputs; the following represents the dosing information for this study.

Group No.	Test Material	Dose Level (vg)
1	Reference Item	0
2	AAV9/AP4M1	3.6E11
3	AAV9/AP4M1	1.1E12
4	AAV9/AP4M1	3.3E12

Appendix 18

Table 4

Summary of Organ Weights Relative to Brain Weight: Day 8

5550008

Sex: Male<br>Day(s) Relative to Start Date		0<br>vg<br>Group 1	0.36x<br>10E12<br>vg<br>Group 2	1.1x<br>10E12<br>vg<br>Group 3	3.3x<br>10E12<br>vg<br>Group 4
Epididymis (%) [G]	Mean	24.84005	25.99324	24.95273	23.39693
	SD	4.63717	5.39963	3.91780	2.45126
	N	4	5	5	5
	%Diff	-	4.64245	0.45360	-5.80966
Gland, Adrenal (%) [G]	Mean	2.18324	2.23653	2.04537	1.98198
	SD	0.16728	0.27871	0.35735	0.28122
	N	4	5	5	5
	%Diff	-	2.44066	-6.31495	-9.21856
Gland, Pituitary (%) [G]	Mean	0.53332	0.55314	0.46129	0.48802
	SD	0.09661	0.07736	0.09725	0.11915
	N	4	5	5	5
	%Diff	-	3.71517	-13.50640	-8.49531
Gland, Prostate (%) [G1]	Mean	23.20135	24.44343	25.53396	22.92219
	SD	3.26988	4.41393	2.79097	7.28072
	N	4	5	5	5
	%Diff	-	5.35351	10.05379	-1.20320
Thyroid/Parathyroid (%) [G]	Mean	0.61536	0.55154	0.46792	0.64053
	SD	0.17224	0.13023	0.05901	0.02057
	N	4	5	5	5
	%Diff	-	-10.37171	-23.95906	4.08984
Heart (%) [G]	Mean	47.24147	50.66173	50.14296	45.70426
	SD	1.03649	9.16372	5.91146	5.68141
	N	4	5	5	5
	%Diff	-	7.23994	6.14184	-3.25395
Kidney (%) [G]	Mean	111.20673	108.35969	103.38464	99.74355
	SD	6.34445	18.98609	7.96264	16.93372
	N	4	5	5	5
	%Diff	-	-2.56013	-7.03382	-10.30799
Liver (%) [G]	Mean	423.45866	416.06248	404.66498	371.96570
	SD	19.78695	56.94768	31.75607	61.50159
	N	4	5	5	5
	%Diff	-	-1.74661	-4.43814	-12.16009

[G] - Anova & Dunnett
[G1] - Kruskal-Wallis & Dunn

Appendix 18

Table 4

Summary of Organ Weights Relative to Brain Weight: Day 8

5550008

Sex: Male<br>Day(s) Relative to Start Date		0<br>vg<br>Group 1	0.36x<br>10E12<br>vg<br>Group 2	1.1x<br>10E12<br>vg<br>Group 3	3.3x<br>10E12<br>vg<br>Group 4
Spleen (%) [G]	Mean	34.54905	29.83942	29.03492	28.93533
	SD	4.38827	5.48685	3.37558	7.71579
	N	4	5	5	5
	%Diff	-	-13.63173	-15.96029	-16.24857
Testis (%) [G1]	Mean	151.25676	144.27198	143.50020	141.57379
	SD	6.55215	14.56049	6.93916	12.51579
	N	4	5	5	5
	%Diff	-	-4.61783	-5.12807	-6.40168
Thymus (%) [G1]	Mean	28.79032	28.42170	28.90854	27.41831
	SD	3.09941	5.27105	4.31696	8.76817
	N	4	5	5	5
	%Diff	-	-1.28034	0.41063	-4.76550

[G] - Kruskal-Wallis & Dunn
[G1] - Anova & Dunnett

Appendix 18

Table 4

Summary of Organ Weights Relative to Brain Weight: Day 8

5550008

Sex: Female<br>Day(s) Relative to Start Date		0<br>vg<br>Group 1	0.36x<br>10E12<br>vg<br>Group 2	1.1x<br>10E12<br>vg<br>Group 3	3.3x<br>10E12<br>vg<br>Group 4
Gland, Adrenal (%) [G]	Mean	3.09401	3.05102	3.20460	2.85356
	SD	0.44517	0.32279	0.52886	0.29049
	N	5	5	5	5
	%Diff	-	-1.38946	3.57419	-7.77152
Gland, Pituitary (%) [G]	Mean	0.74740	0.71962	0.71614	0.72552
	SD	0.06348	0.16977	0.13395	0.13549
	N	5	5	5	5
	%Diff	-	-3.71649	-4.18219	-2.92700
Thyroid/Parathyroid (%) [G]	Mean	0.48580	0.46154	0.47078	0.42393
	SD	0.09743	0.09235	0.08659	0.07965
	N	5	5	5	5
	%Diff	-	-4.99518	-3.09208	-12.73618
Heart (%) [G]	Mean	38.04107	40.29359	39.49665	39.04975
	SD	2.61168	3.84226	2.24951	5.40002
	N	5	5	5	5
	%Diff	-	5.92128	3.82635	2.65156
Kidney (%) [G]	Mean	83.81667	84.55287	86.56724	81.09152
	SD	6.24333	10.07360	6.43590	10.66770
	N	5	5	5	5
	%Diff	-	0.87835	3.28165	-3.25133
Liver (%) [G]	Mean	329.29199	335.82130	301.70892	316.59347
	SD	35.87642	39.68648	33.46541	31.66406
	N	5	5	5	5
	%Diff	-	1.98283	-8.37648	-3.85631
Ovary (%) [G]	Mean	4.00078	3.93661	4.41220	3.81614
	SD	0.24036	0.79793	0.47370	0.47853
	N	5	5	5	5
	%Diff	-	-1.60397	10.28351	-4.61508
Spleen (%) [G]	Mean	22.28342	24.61655	23.41834	20.66264
	SD	2.77096	4.15401	3.16107	3.03926
	N	5	5	5	5
	%Diff	-	10.47024	5.09311	-7.27350

[G] - Anova & Dunnett

Appendix 18

Table 4

Summary of Organ Weights Relative to Brain Weight: Day 8

5550008

Sex: Female<br>Day(s) Relative to Start Date		0<br>vg<br>Group 1	0.36x<br>10E12<br>vg<br>Group 2	1.1x<br>10E12<br>vg<br>Group 3	3.3x<br>10E12<br>vg<br>Group 4
Thymus (%) [G]	Mean	23.59668	26.47273	19.71716	20.79388
	SD	2.64746	3.09421	2.26377	5.03247
	N	5	5	5	5
	%Diff	-	12.18836	-16.44095	-11.87794
Uterus/Cervix (%) [G1]	Mean	26.61909	33.69947	30.61836	38.71469
	SD	6.03191	20.06196	9.13230	16.73239
	N	5	5	5	5
	%Diff	-	26.59888	15.02407	45.43958

[G] - Anova & Dunnett
[G1] - Kruskal-Wallis & Dunn

Appendix 18

Table 4

Summary of Organ Weights Relative to Brain Weight: Day 29

5550008

Sex: Male<br>Day(s) Relative to Start Date		0<br>vg<br>Group 1	0.36x<br>10E12<br>vg<br>Group 2	1.1x<br>10E12<br>vg<br>Group 3	3.3x<br>10E12<br>vg<br>Group 4
Epididymis (%) [G]	Mean	46.18653	45.08385	46.50278	45.90599
	SD	5.37341	3.37286	6.44599	4.39149
	N	5	5	5	5
	%Diff	-	-2.38744	0.68473	-0.60740
Gland, Adrenal (%) [G]	Mean	2.83929	2.64614	2.64531	2.90808
	SD	0.39598	0.22306	0.24649	0.70188
	N	5	5	5	5
	%Diff	-	-6.80279	-6.83203	2.42304
Gland, Pituitary (%) [G]	Mean	0.58674	0.54598	0.62548	0.71383
	SD	0.19174	0.13155	0.04359	0.12213
	N	5	5	5	5
	%Diff	-	-6.94766	6.60210	21.65945
Gland, Prostate (%) [G]	Mean	45.53849	43.89305	43.10926	41.50379
	SD	9.95997	6.11683	11.60675	9.69773
	N	5	5	5	5
	%Diff	-	-3.61330	-5.33446	-8.85999
Thyroid/Parathyroid (%) [G]	Mean	0.76086	0.73263	0.65207	0.81700
	SD	0.16480	0.27833	0.14093	0.28388
	N	5	5	5	5
	%Diff	-	-3.71086	-14.29904	7.37786
Heart (%) [G]	Mean	60.50294	63.98574	62.85176	65.41607
	SD	4.85938	3.44125	5.34559	5.82874
	N	5	5	5	5
	%Diff	-	5.75641	3.88216	8.12049
Kidney (%) [G1]	Mean	128.41119	124.05847	119.48944	142.14047
	SD	6.19195	2.30146	8.67339	11.21038
	N	5	5	5	5
	%Diff	-	-3.38967	-6.94779	10.69166
Liver (%) [G1]	Mean	481.72468	505.16332	502.67391	591.02302
	SD	4.52933	41.33360	36.11434	111.02542
	N	5	5	5	5
	%Diff	-	4.86557	4.34880	22.68896

[G] - Anova & Dunnett
[G1] - Kruskal-Wallis & Dunn

Appendix 18

Table 4

Summary of Organ Weights Relative to Brain Weight: Day 29

5550008

Sex: Male<br>Day(s) Relative to Start Date		0<br>vg<br>Group 1	0.36x<br>10E12<br>vg<br>Group 2	1.1x<br>10E12<br>vg<br>Group 3	3.3x<br>10E12<br>vg<br>Group 4
Spleen (%) [G]	Mean	40.41195	35.17934	33.33110	40.33720
	SD	9.65059	3.31197	1.71542	9.52155
	N	5	5	5	5
	%Diff	-	-12.94816	-17.52168	-0.18497
Testis (%) [G]	Mean	164.76497	158.06823	162.41825	171.00068
	SD	21.66895	11.19531	29.77155	8.68178
	N	5	5	5	5
	%Diff	-	-4.06442	-1.42428	3.78461
Thymus (%) [G]	Mean	27.80436	26.15467	24.95260	27.71034
	SD	6.33180	7.67050	1.89406	10.38596
	N	5	5	5	5
	%Diff	-	-5.93321	-10.25652	-0.33816

[G] - Anova & Dunnett

Appendix 18

Table 4

Summary of Organ Weights Relative to Brain Weight: Day 29

5550008

Sex: Female<br>Day(s) Relative to Start Date		0<br>vg<br>Group 1	0.36x<br>10E12<br>vg<br>Group 2	1.1x<br>10E12<br>vg<br>Group 3	3.3x<br>10E12<br>vg<br>Group 4
Gland, Adrenal (%) [G]	Mean	2.97801	3.27505	3.23710	3.20600
	SD	0.18383	0.24498	0.58784	0.44953
	N	5	5	5	5
	%Diff	-	9.97432	8.70014	7.65576
Gland, Pituitary (%) [G]	Mean	0.70280	0.83049	0.79278	0.84226
	SD	0.19129	0.09342	0.03697	0.08587
	N	5	5	5	5
	%Diff	-	18.16797	12.80264	19.84324
Thyroid/Parathyroid (%) [G]	Mean	0.62350	0.61403	0.69378	0.62588
	SD	0.09285	0.07638	0.15221	0.12740
	N	5	5	5	5
	%Diff	-	-1.51812	11.27170	0.38191
Heart (%) [G]	Mean	40.53297	44.60902	42.06941	46.06831
	SD	2.71379	4.71631	2.64678	3.44561
	N	5	5	5	5
	%Diff	-	10.05612	3.79057	13.65639
Kidney (%) [G]	Mean	76.53034	84.52259	79.05724	84.45384
	SD	5.69031	6.15547	12.31461	4.32156
	N	5	5	5	5
	%Diff	-	10.44325	3.30182	10.35341
Liver (%) [G]	Mean	298.99933	344.99011*	333.38817	347.10634*
	SD	19.80150	22.96042	32.58696	26.42712
	N	5	5	5	5
	%Diff	-	15.38157	11.50131	16.08934
Ovary (%) [G]	Mean	4.40011	5.16266	5.83809	5.13475
	SD	0.74480	0.39697	2.06207	0.90755
	N	5	5	5	5
	%Diff	-	17.33020	32.68034	16.69579
Spleen (%) [G]	Mean	23.24076	25.89725	24.68375	25.40919
	SD	1.87657	7.09218	5.11116	3.54392
	N	5	5	5	5
	%Diff	-	11.43029	6.20888	9.33028

[G] - Anova & Dunnett: * = p ≤ 0.05

Appendix 18

Table 4

Summary of Organ Weights Relative to Brain Weight: Day 29

5550008

Sex: Female<br>Day(s) Relative to Start Date		0<br>vg<br>Group 1	0.36x<br>10E12<br>vg<br>Group 2	1.1x<br>10E12<br>vg<br>Group 3	3.3x<br>10E12<br>vg<br>Group 4
Thymus (%) [G]	Mean	18.55444	21.45450	21.40878	22.30586
	SD	3.05027	3.03572	7.02284	7.22695
	N	5	5	5	5
	%Diff	-	15.63001	15.38359	20.21845
Uterus/Cervix (%) [G]	Mean	44.80779	25.77754**	29.18589*	23.64033**
	SD	15.76880	5.40263	3.83873	3.12721
	N	5	5	5	5
	%Diff	-	-42.47086	-34.86424	-47.24058

[G] - Anova & Dunnett: * = $p \leq 0.05$; ** = $p \leq 0.01$

Appendix 18

Table 4

Summary of Organ Weights Relative to Brain Weight: Day 91

5550008

Sex: Male<br>Day(s) Relative to Start Date		0<br>vg<br>Group 1	0.36x<br>10E12<br>vg<br>Group 2	1.1x<br>10E12<br>vg<br>Group 3	3.3x<br>10E12<br>vg<br>Group 4
Epididymis (%) [G]	Mean	64.60538	59.72309	59.36962	53.26965
	SD	4.02403	12.20263	2.58582	10.70159
	N	5	5	5	5
	%Diff	-	-7.55711	-8.10422	-17.54611
Gland, Adrenal (%) [G]	Mean	2.17896	2.57759	2.24505	2.34634
	SD	0.48362	0.38375	0.31854	0.34099
	N	5	5	5	5
	%Diff	-	18.29460	3.03324	7.68206
Gland, Pituitary (%) [G]	Mean	0.77560	0.71278	0.69918	0.63467
	SD	0.15487	0.14271	0.08321	0.10644
	N	5	5	5	5
	%Diff	-	-8.10023	-9.85398	-18.17044
Gland, Prostate (%) [G]	Mean	67.88979	66.32542	59.89755	70.37981
	SD	8.84162	7.71339	14.09134	11.01002
	N	5	5	5	5
	%Diff	-	-2.30428	-11.77238	3.66774
Thyroid/Parathyroid (%) [G]	Mean	1.01400	0.91907	0.84342	0.82361
	SD	0.27185	0.19387	0.11202	0.19352
	N	5	5	5	5
	%Diff	-	-9.36230	-16.82248	-18.77661
Heart (%) [G]	Mean	79.41513	77.42304	74.04360	68.48678
	SD	12.20310	5.22943	9.63360	7.06802
	N	5	5	5	5
	%Diff	-	-2.50845	-6.76386	-13.76104
Kidney (%) [G]	Mean	148.94232	141.71415	133.81911	131.37395
	SD	19.60805	10.61806	12.22615	17.04571
	N	5	5	5	5
	%Diff	-	-4.85300	-10.15374	-11.79542
Liver (%) [G]	Mean	694.62163	639.55552	592.25074	537.88266
	SD	203.25274	61.29031	78.81906	66.55983
	N	5	5	5	5
	%Diff	-	-7.92750	-14.73765	-22.56466

[G] - Anova & Dunnett

Appendix 18

Table 4

Summary of Organ Weights Relative to Brain Weight: Day 91

5550008

Sex: Male<br>Day(s) Relative to Start Date		0<br>vg<br>Group 1	0.36x<br>10E12<br>vg<br>Group 2	1.1x<br>10E12<br>vg<br>Group 3	3.3x<br>10E12<br>vg<br>Group 4
Spleen (%) [G]	Mean	42.74320	41.56811	37.11702	38.22608
	SD	3.83836	6.84035	3.29334	8.40004
	N	5	5	5	5
	%Diff	-	-2.74919	-13.16275	-10.56804
Testis (%) [G1]	Mean	176.17435	168.69540	166.04679	145.07191
	SD	16.94994	24.51628	11.67239	35.28302
	N	5	5	5	5
	%Diff	-	-4.24520	-5.74860	-17.65436
Thymus (%) [G]	Mean	12.46737	12.65277	14.83697	12.36173
	SD	2.55987	2.40867	4.28095	3.91904
	N	5	5	5	5
	%Diff	-	1.48709	19.00641	-0.84732

[G] - Anova & Dunnett
[G1] - Kruskal-Wallis & Dunn

Appendix 18

Table 4

Summary of Organ Weights Relative to Brain Weight: Day 91

5550008

Sex: Female<br>Day(s) Relative to Start Date		0<br>vg<br>Group 1	0.36x<br>10E12<br>vg<br>Group 2	1.1x<br>10E12<br>vg<br>Group 3	3.3x<br>10E12<br>vg<br>Group 4
Gland, Adrenal (%) [G]	Mean	3.40037	3.22135	2.84061	2.97873
	SD	0.49994	0.37703	0.61619	0.33837
	N	5	4	5	5
	%Diff	-	-5.26475	-16.46172	-12.39982
Gland, Pituitary (%) [G]	Mean	0.91433	0.98922	0.98293	1.12945
	SD	0.17120	0.17534	0.17518	0.25808
	N	5	4	5	5
	%Diff	-	8.19036	7.50175	23.52705
Thyroid/Parathyroid (%) [G1]	Mean	0.65637	0.74121	0.67565	0.59413
	SD	0.10333	0.23722	0.09600	0.16325
	N	5	4	5	5
	%Diff	-	12.92525	2.93657	-9.48248
Heart (%) [G]	Mean	46.30958	48.28805	48.91725	49.12577
	SD	3.57404	3.55903	8.02675	3.62680
	N	5	4	5	5
	%Diff	-	4.27227	5.63095	6.08124
Kidney (%) [G]	Mean	83.06909	92.86003	82.44388	86.74325
	SD	7.34261	7.17369	13.48201	8.33949
	N	5	4	5	5
	%Diff	-	11.78650	-0.75264	4.42301
Liver (%) [G]	Mean	324.40904	365.30750	342.99358	347.20317
	SD	29.09383	27.26498	46.13571	34.59812
	N	5	4	5	5
	%Diff	-	12.60706	5.72874	7.02635
Ovary (%) [G]	Mean	4.12687	4.53685	4.18007	3.45199
	SD	0.36639	0.72045	1.22998	0.65577
	N	5	4	5	5
	%Diff	-	9.93439	1.28895	-16.35328
Spleen (%) [G]	Mean	28.49385	25.60187	24.93686	25.93544
	SD	3.33900	2.51511	3.95253	3.41074
	N	5	4	5	5
	%Diff	-	-10.14947	-12.48335	-8.97881

[G] - Anova & Dunnett
[G1] - Kruskal-Wallis & Dunn

Appendix 18

Table 4

Summary of Organ Weights Relative to Brain Weight: Day 91

5550008

Sex: Female<br>Day(s) Relative to Start Date		0<br>vg<br>Group 1	0.36x<br>10E12<br>vg<br>Group 2	1.1x<br>10E12<br>vg<br>Group 3	3.3x<br>10E12<br>vg<br>Group 4
Thymus (%) [G]	Mean	11.82739	8.91803	11.16538	11.15282
	SD	2.10703	0.71786	2.70712	2.02875
	N	5	4	5	5
	%Diff	-	-24.59849	-5.59733	-5.70348
Uterus/Cervix (%) [G1]	Mean	29.57864	34.69306	59.17636	45.75602
	SD	7.78973	13.67028	47.71003	15.24553
	N	5	4	5	5
	%Diff	-	17.29093	100.06453	54.69281

[G] - Anova & Dunnett
[G1] - Kruskal-Wallis & Dunn

Appendix 18

Table 5

Summary of Microscopic Pathology Explanation Page

Abbreviation	Description
GALT	Gut Associated Lymphoid Tissue
SS	Special Stain

Note: This is a comprehensive list of abbreviations. All of the abbreviations listed may not be applicable to this report.

Dosing Information

Dosing information is abbreviated on various data outputs; the following represents the dosing information for this study:

Group No.	Test Material	Dose Level (vg)
1	Reference Item	0
2	AAV9/AP4M1	3.6E11
3	AAV9/AP4M1	1.1E12
4	AAV9/AP4M1	3.3E12

Appendix 18

Table 5

Summary of Microscopic Pathology: Day 8

5550008

Removal Reason(s): TERMINAL EUTHANASIA	Male				Female			
Summary: Incidence	0 vg Group 1	0.36x 10E12 vg Group 2	1.1x 10E12 vg Group 3	3.3x 10E12 vg Group 4	0 vg Group 1	0.36x 10E12 vg Group 2	1.1x 10E12 vg Group 3	3.3x 10E12 vg Group 4
Number of Animals:	4	5	5	5	5	5	5	5
**BRAIN**								
Examined	4	5	5	5	5	5	5	5
No Visible Lesions	4	5	5	5	5	4	5	5
Infiltration, mononuclear cell; meninges	0	0	0	0	0	1	0	0
.... minimal	0	0	0	0	0	1	0	0
**CERVIX**								
Examined	.	.	.	.	5	5	5	5
No Visible Lesions	.	.	.	.	5	5	5	5
**EPIDIDYMIS**								
Examined	4	5	5	5	.	.	.	.
No Visible Lesions	3	4	3	2	.	.	.	.
Infiltration, mononuclear cell	1	0	0	0	.	.	.	.
.... minimal	1	0	0	0	.	.	.	.
Peripuberty	0	1	2	3	.	.	.	.
**EYE**								
Examined	4	5	5	5	5	5	5	5
No Visible Lesions	4	5	5	5	4	5	5	4
Dysplasia; retina	0	0	0	0	1	0	0	1
.... minimal	0	0	0	0	0	0	0	1
.... mild	0	0	0	0	1	0	0	0
**GANGLION, DORSAL ROOT, CERVICAL**								
Examined	4	5	5	5	5	5	5	5
No Visible Lesions	4	5	5	5	5	5	5	5
**GANGLION, DORSAL ROOT, LUMBAR**								
Examined	4	5	5	5	5	5	5	5
No Visible Lesions	4	5	3	0	5	4	4	2
Infiltration, mononuclear cell	0	0	2	5	0	1	1	3
.... minimal	0	0	2	5	0	1	1	3
**GANGLION, DORSAL ROOT, THORACIC**								

Appendix 18
Table 5

Summary of Microscopic Pathology: Day 8

5550008

Removal Reason(s): TERMINAL EUTHANASIA	Male				Female			
Summary: Incidence	0 vg Group 1	0.36x 10E12 vg Group 2	1.1x 10E12 vg Group 3	3.3x 10E12 vg Group 4	0 vg Group 1	0.36x 10E12 vg Group 2	1.1x 10E12 vg Group 3	3.3x 10E12 vg Group 4
Number of Animals:	4	5	5	5	5	5	5	5
**GANGLION, DORSAL ROOT, THORACIC (Continued...)**								
Examined	4	5	5	5	5	5	5	5
No Visible Lesions	4	5	5	5	5	5	5	5
**GLAND, ADRENAL**								
Examined	4	5	5	5	5	5	5	5
No Visible Lesions	4	3	5	5	5	5	5	5
Vacuolation; cortical	0	2	0	0	0	0	0	0
.... minimal	0	1	0	0	0	0	0	0
.... mild	0	1	0	0	0	0	0	0
**GLAND, PARATHYROID**								
Examined	4	5	5	5	5	5	5	4
No Visible Lesions	4	5	5	5	5	5	5	4
Not Examined: Not Present In Section.	.	.	.	.	0	0	0	1
**GLAND, PITUITARY**								
Examined	4	5	5	5	5	5	5	5
No Visible Lesions	4	5	5	4	5	5	5	5
Congestion	0	0	0	1	0	0	0	0
.... moderate	0	0	0	1	0	0	0	0
**GLAND, PROSTATE**								
Examined	4	5	5	5	.	.	.	.
No Visible Lesions	4	5	5	5	.	.	.	.
**GLAND, THYROID**								
Examined	4	5	5	5	5	5	5	5
No Visible Lesions	4	5	5	5	5	5	5	5
**HEART**								
Examined	4	4	5	5	5	5	5	5
No Visible Lesions	3	4	5	5	5	5	4	5
Not Examined: Not Present In Section.	0	1	0	0	.	.	.	.
Infiltration, mononuclear cell/degeneration, myofiber	1	0	0	0	0	0	0	0
.... minimal	1	0	0	0	0	0	0	0

Appendix 18

Table 5

Summary of Microscopic Pathology: Day 8

5550008

Removal Reason(s): TERMINAL EUTHANASIA	Male				Female			
Summary: Incidence	0 vg Group 1	0.36x 10E12 vg Group 2	1.1x 10E12 vg Group 3	3.3x 10E12 vg Group 4	0 vg Group 1	0.36x 10E12 vg Group 2	1.1x 10E12 vg Group 3	3.3x 10E12 vg Group 4
Number of Animals:	4	5	5	5	5	5	5	5
**HEART (Continued...)**								
Cyst; atrioventricular valve	0	0	0	0	0	0	1	0
**KIDNEY**								
Examined	4	5	5	5	5	5	5	5
No Visible Lesions	1	1	4	5	4	3	4	2
Infiltration, mononuclear cell	2	2	0	0	1	1	1	1
.... minimal	2	2	0	0	1	1	1	1
Dilatation; tubular	0	2	0	0	0	0	0	1
.... mild	0	2	0	0	0	0	0	1
Chronic progressive nephropathy	2	2	1	0	0	2	1	2
.... minimal	2	2	1	0	0	2	1	2
**LIVER**								
Examined	4	5	5	5	5	5	5	5
No Visible Lesions	0	3	0	1	1	2	4	3
Vacuolation; hepatocellular, periportal	0	1	0	2	3	2	0	1
.... minimal	0	1	0	2	2	2	0	1
.... mild	0	0	0	0	1	0	0	0
Tension lipidosis	2	1	1	1	1	0	0	0
.... minimal	1	1	1	1	1	0	0	0
.... mild	1	0	0	0	0	0	0	0
Infiltration, mixed cell	3	0	2	1	1	0	1	0
.... minimal	3	0	2	1	1	0	1	0
Hyperplasia; bile duct	1	0	0	0	0	0	0	0
.... minimal	1	0	0	0	0	0	0	0
Infiltration, mononuclear cell	1	0	0	1	0	0	0	0
.... minimal	1	0	0	1	0	0	0	0
Infiltration, mononuclear cell; periportal	0	1	1	1	2	1	0	1
.... minimal	0	1	1	1	2	1	0	1
Extramedullary hematopoiesis	0	0	1	0	0	0	0	0
.... minimal	0	0	1	0	0	0	0	0

Appendix 18

Table 5

Summary of Microscopic Pathology: Day 8

5550008

Removal Reason(s): TERMINAL EUTHANASIA	Male				Female			
Summary: Incidence	0 vg Group 1	0.36x 10E12 vg Group 2	1.1x 10E12 vg Group 3	3.3x 10E12 vg Group 4	0 vg Group 1	0.36x 10E12 vg Group 2	1.1x 10E12 vg Group 3	3.3x 10E12 vg Group 4
Number of Animals:	4	5	5	5	5	5	5	5
**LUNG**								
Examined	4	5	5	5	5	5	5	5
No Visible Lesions	4	5	5	4	5	5	5	5
Hemorrhage; chronic	0	0	0	1	0	0	0	0
.... mild	0	0	0	1	0	0	0	0
**LYMPH NODE**								
Examined	.	.	.	.	0	1	0	1
Hemorrhage; acute	.	.	.	.	.	1	.	1
.... mild	.	.	.	.	.	0	.	1
.... moderate	.	.	.	.	.	1	.	0
**LYMPH NODE, CERVICAL**								
Examined	4	5	5	5	5	5	5	5
No Visible Lesions	4	3	3	4	5	4	5	5
Hemorrhage; acute	0	2	2	1	0	1	0	0
.... minimal	0	0	1	0	0	0	0	0
.... mild	0	2	1	1	0	1	0	0
**LYMPH NODE, ILIAC**								
Examined	4	5	5	5	5	4	5	4
No Visible Lesions	4	4	5	5	5	4	5	4
Not Examined: Not Present In Section.	.	.	.	.	0	1	0	1
Cellularity, increased; lymphoid	0	1	0	0	0	0	0	0
.... mild	0	1	0	0	0	0	0	0
**LYMPH NODE, MANDIBULAR**								
Examined	4	5	5	5	5	5	5	5
No Visible Lesions	4	5	3	5	5	4	5	4
Hemorrhage; acute	0	0	2	0	0	1	0	1
.... mild	0	0	2	0	0	1	0	1
**LYMPH NODE, MESENTERIC**								
Examined	4	5	5	5	5	5	5	5
No Visible Lesions	4	5	5	5	5	5	5	5

Appendix 18

Table 5

Summary of Microscopic Pathology: Day 8

5550008

Removal Reason(s): TERMINAL EUTHANASIA	Male				Female			
Summary: Incidence	0 vg Group 1	0.36x 10E12 vg Group 2	1.1x 10E12 vg Group 3	3.3x 10E12 vg Group 4	0 vg Group 1	0.36x 10E12 vg Group 2	1.1x 10E12 vg Group 3	3.3x 10E12 vg Group 4
Number of Animals:	4	5	5	5	5	5	5	5
**MUSCLE, BICEPS FEMORIS**								
Examined	4	5	5	5	5	5	5	5
No Visible Lesions	4	5	5	5	5	5	5	5
**MUSCLE, GASTROCNEMIUS**								
Examined	4	5	5	5	5	5	5	5
No Visible Lesions	4	5	5	5	5	5	5	5
**NERVE, OPTIC**								
Examined	2	5	3	3	5	4	5	5
No Visible Lesions	2	5	3	3	5	4	5	5
Not Examined: Not Present In Section.	2	0	2	2	0	1	0	0
**NERVE, SCIATIC**								
Examined	4	5	5	5	5	5	5	5
No Visible Lesions	4	5	4	4	5	5	5	4
Degeneration; axonal	0	0	1	1	0	0	0	1
.... minimal	0	0	1	1	0	0	0	1
**NERVE, TIBIAL**								
Examined	4	5	5	5	5	5	5	5
No Visible Lesions	4	4	3	3	5	5	5	4
Degeneration; axonal	0	1	2	2	0	0	0	1
.... minimal	0	1	2	2	0	0	0	1
**OVARY**								
Examined	.	.	.	.	5	5	5	5
No Visible Lesions	.	.	.	.	5	5	5	5
**SITE, INJECTION**								
Examined	4	5	5	5	5	5	5	5
No Visible Lesions	0	2	2	3	1	1	3	3
Infiltration, mononuclear cell	0	0	0	0	0	1	0	0
.... minimal	0	0	0	0	0	1	0	0
Infiltration, mononuclear cell; cauda equina	0	0	0	1	0	1	0	0
.... minimal	0	0	0	1	0	1	0	0
Infiltration, mononuclear cell; leptomeninges	0	1	2	1	3	2	1	1

Summary of Microscopic Pathology: Day 8

5550008

Removal Reason(s): TERMINAL EUTHANASIA	Male				Female			
Summary: Incidence	0 vg Group 1	0.36x 10E12 vg Group 2	1.1x 10E12 vg Group 3	3.3x 10E12 vg Group 4	0 vg Group 1	0.36x 10E12 vg Group 2	1.1x 10E12 vg Group 3	3.3x 10E12 vg Group 4
Number of Animals:	4	5	5	5	5	5	5	5
**SITE, INJECTION (Continued...)**								
.... minimal	0	1	2	1	3	2	1	1
Degeneration; axonal, cauda equina	4	2	2	1	2	2	1	1
.... minimal	4	2	2	1	2	2	1	1
**SITE, SURGICAL**								
Examined	.	.	.	.	0	1	0	1
Hemorrhage; acute	.	.	.	.	.	1	.	1
.... mild	.	.	.	.	.	1	.	1
Fibroplasia	.	.	.	.	.	0	.	1
.... moderate	.	.	.	.	.	0	.	1
Hyperplasia; epidermal	.	.	.	.	.	0	.	1
.... minimal	.	.	.	.	.	0	.	1
**SPINAL CORD, CERVICAL**								
Examined	4	5	5	5	5	5	5	5
No Visible Lesions	4	4	5	5	5	5	5	5
Hyperplasia; leptomeninges	0	1	0	0	0	0	0	0
.... minimal	0	1	0	0	0	0	0	0
**SPINAL CORD, LUMBAR**								
Examined	4	5	5	5	5	5	5	5
No Visible Lesions	4	5	5	5	4	5	5	5
Infiltration, mononuclear cell	0	0	0	0	1	0	0	0
.... minimal	0	0	0	0	1	0	0	0
**SPINAL CORD, THORACIC**								
Examined	4	5	5	5	5	5	5	5
No Visible Lesions	4	5	5	5	5	5	5	5
**SPLEEN**								
Examined	4	5	5	5	5	5	5	5
No Visible Lesions	4	5	5	5	5	5	5	5
**SUBCUTIS**								
Examined	0	0	1	0	.	.	.	.
Hemorrhage; acute	.	.	1	.	.	.	.	.

Appendix 18

Table 5

Summary of Microscopic Pathology: Day 8

5550008

Removal Reason(s): TERMINAL EUTHANASIA	Male				Female			
Summary: Incidence	0 vg Group 1	0.36x 10E12 vg Group 2	1.1x 10E12 vg Group 3	3.3x 10E12 vg Group 4	0 vg Group 1	0.36x 10E12 vg Group 2	1.1x 10E12 vg Group 3	3.3x 10E12 vg Group 4
Number of Animals:	4	5	5	5	5	5	5	5
**SUBCUTIS (Continued...)**								
.... mild	.	.	1	.	.	.	.	.
Fibroplasia	.	.	1	.	.	.	.	.
.... mild	.	.	1	.	.	.	.	.
Infiltration, mononuclear cell	.	.	1	.	.	.	.	.
.... mild	.	.	1	.	.	.	.	.
Fibrosis	.	.	1	.	.	.	.	.
.... mild	.	.	1	.	.	.	.	.
**TESTIS**								
Examined	4	5	5	5	.	.	.	.
No Visible Lesions	4	4	3	2	.	.	.	.
Peripuberty	0	1	2	3	.	.	.	.
**THYMUS**								
Examined	4	5	5	5	5	5	5	5
No Visible Lesions	4	5	5	5	5	4	5	5
Hemorrhage; acute	0	0	0	0	0	1	0	0
.... mild	0	0	0	0	0	1	0	0
**UTERUS**								
Examined	.	.	.	.	5	5	5	5
No Visible Lesions	.	.	.	.	5	5	5	4
Cyst	.	.	.	.	0	0	0	1
**NERVE ROOT, VENTRAL, CERVICAL**								
Examined	4	5	5	5	5	5	5	5
No Visible Lesions	4	5	5	5	5	5	5	5
**NERVE ROOT, VENTRAL, THORACIC**								
Examined	4	5	5	5	5	5	5	5
No Visible Lesions	4	5	5	5	5	5	5	5
**NERVE ROOT, VENTRAL, LUMBAR**								
Examined	4	5	5	5	5	5	5	5
No Visible Lesions	4	5	5	5	5	5	5	5

Appendix 18

Table 5

Summary of Microscopic Pathology: Day 8

5550008

Removal Reason(s): TERMINAL EUTHANASIA	Male				Female			
Summary: Incidence	0 vg Group 1	0.36x 10E12 vg Group 2	1.1x 10E12 vg Group 3	3.3x 10E12 vg Group 4	0 vg Group 1	0.36x 10E12 vg Group 2	1.1x 10E12 vg Group 3	3.3x 10E12 vg Group 4
Number of Animals:	4	5	5	5	5	5	5	5
**NERVE ROOT, DORSAL, CERVICAL**								
Examined	4	5	5	5	5	5	5	5
No Visible Lesions	4	5	5	5	5	5	5	5
**NERVE ROOT, DORSAL, LUMBAR**								
Examined	4	5	5	5	5	5	5	5
No Visible Lesions	3	5	2	4	5	5	5	5
Degeneration; axonal	1	0	2	1	0	0	0	0
.... minimal	1	0	2	1	0	0	0	0
Infiltration, mononuclear cell; leptomeninges	0	0	1	0	0	0	0	0
.... minimal	0	0	1	0	0	0	0	0
**NERVE ROOT, DORSAL, THORACIC**								
Examined	4	5	5	5	5	5	5	5
No Visible Lesions	4	5	5	5	5	5	5	5

Appendix 18

Table 5

Summary of Microscopic Pathology: Day 29

5550008

Removal Reason(s): RECOVERY EUTHANASIA	Male				Female			
Summary: Incidence	0 vg Group 1	0.36x 10E12 vg Group 2	1.1x 10E12 vg Group 3	3.3x 10E12 vg Group 4	0 vg Group 1	0.36x 10E12 vg Group 2	1.1x 10E12 vg Group 3	3.3x 10E12 vg Group 4
Number of Animals:	5	5	5	5	5	5	5	5
**BRAIN**								
Examined	5	5	5	5	5	5	5	5
No Visible Lesions	5	5	5	5	5	5	5	5
**CERVIX**								
Examined	.	.	.	.	5	5	5	5
No Visible Lesions	.	.	.	.	5	5	5	5
**EPIDIDYMIS**								
Examined	5	5	5	5	.	.	.	.
No Visible Lesions	3	4	3	2	.	.	.	.
Cellular debris	0	1	1	1	.	.	.	.
.... minimal	0	1	0	1	.	.	.	.
.... moderate	0	0	1	0	.	.	.	.
Infiltration, mononuclear cell	2	0	1	2	.	.	.	.
.... minimal	2	0	1	2	.	.	.	.
Sperm, decreased	0	0	1	0	.	.	.	.
.... moderate	0	0	1	0	.	.	.	.
**EYE**								
Examined	5	5	5	5	5	5	5	5
No Visible Lesions	5	5	5	5	5	5	5	5
**GANGLION, DORSAL ROOT, CERVICAL**								
Examined	5	5	5	5	5	5	5	5
No Visible Lesions	5	5	5	5	5	5	5	4
Degeneration; axonal	0	0	0	0	0	0	0	1
.... minimal	0	0	0	0	0	0	0	1
Infiltration, mononuclear cell	0	0	0	0	0	0	0	1
.... minimal	0	0	0	0	0	0	0	1
**GANGLION, DORSAL ROOT, LUMBAR**								
Examined	5	5	5	5	5	5	5	5
No Visible Lesions	5	5	1	1	5	5	2	0
Infiltration, mononuclear cell	0	0	4	4	0	0	3	5
.... minimal	0	0	4	3	0	0	2	3

Appendix 18

Table 5

Summary of Microscopic Pathology: Day 29

5550008

Removal Reason(s): RECOVERY EUTHANASIA	Male				Female			
Summary: Incidence	0 vg Group 1	0.36x 10E12 vg Group 2	1.1x 10E12 vg Group 3	3.3x 10E12 vg Group 4	0 vg Group 1	0.36x 10E12 vg Group 2	1.1x 10E12 vg Group 3	3.3x 10E12 vg Group 4
Number of Animals:	5	5	5	5	5	5	5	5
**GANGLION, DORSAL ROOT, LUMBAR (Continued...)**								
.... mild	0	0	0	1	0	0	1	2
Degeneration; axonal	0	0	2	4	0	0	2	5
.... minimal	0	0	2	1	0	0	2	4
.... mild	0	0	0	3	0	0	0	1
Degeneration; neuronal	0	0	3	3	0	0	1	3
.... minimal	0	0	3	2	0	0	1	3
.... mild	0	0	0	1	0	0	0	0
**GANGLION, DORSAL ROOT, THORACIC**								
Examined	5	5	5	5	5	5	5	5
No Visible Lesions	5	5	5	5	5	5	5	3
Degeneration; axonal	0	0	0	0	0	0	0	1
.... minimal	0	0	0	0	0	0	0	1
Infiltration, mononuclear cell	0	0	0	0	0	0	0	1
.... minimal	0	0	0	0	0	0	0	1
**GLAND, ADRENAL**								
Examined	5	5	5	5	5	5	5	5
No Visible Lesions	4	4	5	2	4	5	5	5
Vacuolation; cortical	1	1	0	3	1	0	0	0
.... minimal	1	1	0	3	0	0	0	0
.... mild	0	0	0	0	1	0	0	0
Infiltration, mononuclear cell	0	0	0	1	0	0	0	0
.... minimal	0	0	0	1	0	0	0	0
**GLAND, PARATHYROID**								
Examined	5	4	4	5	5	4	5	3
No Visible Lesions	5	4	4	5	5	4	5	3
Not Examined: Not Present In Section.	0	1	1	0	0	1	0	2
**GLAND, PITUITARY**								
Examined	5	5	5	5	5	5	5	5
No Visible Lesions	5	5	5	5	5	5	5	5

Appendix 18

Table 5

Summary of Microscopic Pathology: Day 29

5550008

Removal Reason(s): RECOVERY EUTHANASIA	Male				Female			
Summary: Incidence	0 vg Group 1	0.36x 10E12 vg Group 2	1.1x 10E12 vg Group 3	3.3x 10E12 vg Group 4	0 vg Group 1	0.36x 10E12 vg Group 2	1.1x 10E12 vg Group 3	3.3x 10E12 vg Group 4
Number of Animals:	5	5	5	5	5	5	5	5
**GLAND, PROSTATE**								
Examined	5	5	5	5	.	.	.	.
No Visible Lesions	4	4	4	4	.	.	.	.
Vacuolation; neuronal, ganglion	0	1	0	0	.	.	.	.
.... minimal	0	1	0	0	.	.	.	.
Infiltration, mononuclear cell	1	0	1	1	.	.	.	.
.... minimal	1	0	1	1	.	.	.	.
**GLAND, THYROID**								
Examined	5	5	5	5	5	5	5	5
No Visible Lesions	5	5	5	5	5	5	5	5
**HEART**								
Examined	5	5	5	5	5	5	5	5
No Visible Lesions	5	5	5	4	5	5	5	4
Infiltration, mononuclear cell/degeneration, myofiber	0	0	0	0	0	0	0	1
.... minimal	0	0	0	0	0	0	0	1
Fibrosis	0	0	0	1	0	0	0	0
.... minimal	0	0	0	1	0	0	0	0
**KIDNEY**								
Examined	5	5	5	5	5	5	5	5
No Visible Lesions	1	0	0	0	2	3	4	0
Cast; hyaline	0	0	2	0	0	1	1	1
.... minimal	0	0	2	0	0	1	1	1
Infiltration, mononuclear cell	3	3	3	5	1	0	0	4
.... minimal	3	3	3	5	1	0	0	4
Dilatation; tubular	1	1	0	0	1	2	0	1
.... minimal	1	1	0	0	1	1	0	1
.... mild	0	0	0	0	0	1	0	0
Chronic progressive nephropathy	2	2	3	4	2	1	0	2
.... minimal	2	1	3	4	2	1	0	2
.... mild	0	1	0	0	0	0	0	0

Appendix 18

Table 5

Summary of Microscopic Pathology: Day 29

5550008

Removal Reason(s): RECOVERY EUTHANASIA	Male				Female			
Summary: Incidence	0 vg Group 1	0.36x 10E12 vg Group 2	1.1x 10E12 vg Group 3	3.3x 10E12 vg Group 4	0 vg Group 1	0.36x 10E12 vg Group 2	1.1x 10E12 vg Group 3	3.3x 10E12 vg Group 4
Number of Animals:	5	5	5	5	5	5	5	5
**LIVER**								
Examined	5	5	5	5	5	5	5	5
No Visible Lesions	0	0	1	0	0	0	1	0
Vacuolation; hepatocellular, periportal	2	1	2	1	0	3	1	2
.... minimal	2	1	2	1	0	3	1	2
Tension lipidosis	1	0	2	1	1	2	1	1
.... minimal	1	0	0	1	1	2	0	1
.... mild	0	0	2	0	0	0	1	0
Infiltration, mixed cell	4	4	3	5	5	4	3	5
.... minimal	4	4	3	5	4	4	3	5
.... mild	0	0	0	0	1	0	0	0
Infiltration, mononuclear cell; periportal	3	1	2	0	0	1	0	1
.... minimal	2	0	2	0	0	1	0	1
.... mild	1	1	0	0	0	0	0	0
Extramedullary hematopoiesis	0	0	0	1	0	0	0	0
.... minimal	0	0	0	1	0	0	0	0
**LUNG**								
Examined	5	5	5	5	5	5	5	5
No Visible Lesions	5	5	5	3	5	5	5	5
Hemorrhage; chronic	0	0	0	1	0	0	0	0
.... minimal	0	0	0	1	0	0	0	0
Pigmented macrophage	0	0	0	1	0	0	0	0
.... minimal	0	0	0	1	0	0	0	0
**LYMPH NODE**								
Examined	0	1	0	1	1	0	2	1
No Visible Lesions	.	0	.	0	0	.	1	0
Hemorrhage; acute	.	1	.	0	1	.	1	1
.... minimal	.	0	.	0	0	.	1	0
.... mild	.	1	.	0	1	.	0	1
Pigmented macrophage	.	0	.	1	0	.	1	0
.... mild	.	0	.	1	0	.	1	0

Appendix 18

Table 5

Summary of Microscopic Pathology: Day 29

5550008

Removal Reason(s): RECOVERY EUTHANASIA	Male				Female			
Summary: Incidence	0 vg Group 1	0.36x 10E12 vg Group 2	1.1x 10E12 vg Group 3	3.3x 10E12 vg Group 4	0 vg Group 1	0.36x 10E12 vg Group 2	1.1x 10E12 vg Group 3	3.3x 10E12 vg Group 4
Number of Animals:	5	5	5	5	5	5	5	5
**LYMPH NODE, CERVICAL**								
Examined	5	5	5	5	5	5	5	5
No Visible Lesions	5	5	5	5	5	5	4	5
Hemorrhage; acute	0	0	0	0	0	0	1	0
.... mild	0	0	0	0	0	0	1	0
**LYMPH NODE, ILIAC**								
Examined	5	4	5	5	4	5	5	5
No Visible Lesions	5	4	5	5	4	4	5	5
Not Examined: Not Present In Section.	0	1	0	0	1	0	0	0
Hemorrhage; acute	0	0	0	0	0	1	0	0
.... mild	0	0	0	0	0	1	0	0
**LYMPH NODE, MANDIBULAR**								
Examined	4	5	5	5	5	5	5	5
No Visible Lesions	4	4	4	5	5	5	4	5
Not Examined: Not Present In Section.	1	0	0	0	.	.	.	.
Hemorrhage; acute	0	0	0	0	0	0	1	0
.... mild	0	0	0	0	0	0	1	0
Plasmacytosis	0	1	1	0	0	0	0	0
.... mild	0	1	1	0	0	0	0	0
Cellularity, increased; lymphoid	0	0	1	0	0	0	0	0
.... mild	0	0	1	0	0	0	0	0
**LYMPH NODE, MESENTERIC**								
Examined	5	5	5	5	5	5	5	5
No Visible Lesions	5	5	5	5	5	5	5	5
**MUSCLE, BICEPS FEMORIS**								
Examined	5	5	5	5	5	5	5	5
No Visible Lesions	5	5	5	5	5	5	5	5
**MUSCLE, GASTROCNEMIUS**								
Examined	5	5	5	5	5	5	5	5
No Visible Lesions	5	5	5	5	5	5	5	5

Appendix 18

Table 5

Summary of Microscopic Pathology: Day 29

5550008

Removal Reason(s): RECOVERY EUTHANASIA	Male				Female			
Summary: Incidence	0 vg Group 1	0.36x 10E12 vg Group 2	1.1x 10E12 vg Group 3	3.3x 10E12 vg Group 4	0 vg Group 1	0.36x 10E12 vg Group 2	1.1x 10E12 vg Group 3	3.3x 10E12 vg Group 4
Number of Animals:	5	5	5	5	5	5	5	5
**NERVE, OPTIC**								
Examined	5	5	5	4	5	5	5	4
No Visible Lesions	5	5	5	4	5	5	5	4
Not Examined: Not Present In Section.	0	0	0	1	0	0	0	1
**NERVE, SCIATIC**								
Examined	5	5	5	5	5	5	5	5
No Visible Lesions	5	5	0	0	5	5	0	0
Degeneration; axonal	0	0	5	5	0	0	5	5
.... minimal	0	0	4	4	0	0	5	2
.... mild	0	0	1	1	0	0	0	3
**NERVE, TIBIAL**								
Examined	5	5	5	5	5	5	5	5
No Visible Lesions	5	5	1	1	5	5	1	0
Degeneration; axonal	0	0	4	4	0	0	4	5
.... minimal	0	0	3	3	0	0	4	3
.... mild	0	0	1	1	0	0	0	2
**OVARY**								
Examined	.	.	.	.	5	5	5	5
No Visible Lesions	.	.	.	.	5	5	4	5
Cyst	.	.	.	.	0	0	1	0
**SITE, INJECTION**								
Examined	5	5	5	5	5	5	5	5
No Visible Lesions	5	2	2	1	3	1	1	0
Infiltration, mononuclear cell; cauda equina	0	0	0	0	1	0	0	0
.... minimal	0	0	0	0	1	0	0	0
Infiltration, mononuclear cell; leptomeninges	0	2	1	4	1	4	2	4
.... minimal	0	2	1	3	1	4	2	4
.... mild	0	0	0	1	0	0	0	0
Degeneration; axonal, cauda equina	0	2	3	4	0	0	3	5
.... minimal	0	2	3	2	0	0	2	0

Appendix 18

Table 5

Summary of Microscopic Pathology: Day 29

5550008

Removal Reason(s): RECOVERY EUTHANASIA	Male				Female			
Summary: Incidence	0 vg Group 1	0.36x 10E12 vg Group 2	1.1x 10E12 vg Group 3	3.3x 10E12 vg Group 4	0 vg Group 1	0.36x 10E12 vg Group 2	1.1x 10E12 vg Group 3	3.3x 10E12 vg Group 4
Number of Animals:	5	5	5	5	5	5	5	5
**SITE, INJECTION (Continued...)**								
.... mild	0	0	0	2	0	0	1	3
.... moderate	0	0	0	0	0	0	0	2
**SPINAL CORD, CERVICAL**								
Examined	5	5	5	5	5	5	5	5
No Visible Lesions	5	5	5	5	5	4	5	5
Infiltration, mononuclear cell; leptomeninges	0	0	0	0	0	1	0	0
.... minimal	0	0	0	0	0	1	0	0
**SPINAL CORD, LUMBAR**								
Examined	5	5	5	5	5	5	5	5
No Visible Lesions	5	5	5	5	5	5	5	4
Infiltration, mononuclear cell; leptomeninges	0	0	0	0	0	0	0	1
.... minimal	0	0	0	0	0	0	0	1
**SPINAL CORD, THORACIC**								
Examined	5	5	5	5	5	5	5	5
No Visible Lesions	5	5	5	4	5	4	5	3
Infiltration, mononuclear cell; adipose tissue	0	0	0	1	0	1	0	2
.... minimal	0	0	0	0	0	1	0	2
.... mild	0	0	0	1	0	0	0	0
**SPLEEN**								
Examined	5	5	5	5	5	5	5	5
No Visible Lesions	5	5	5	5	5	5	5	5
**SUBCUTIS**								
Examined	0	0	1	0	.	.	.	.
Hemorrhage; acute	.	.	1	.	.	.	.	.
.... minimal	.	.	1	.	.	.	.	.
Fibroplasia	.	.	1	.	.	.	.	.
.... mild	.	.	1	.	.	.	.	.

Appendix 18

Table 5

Summary of Microscopic Pathology: Day 29

5550008

Removal Reason(s): RECOVERY EUTHANASIA<br>Summary: Incidence	Male				Female			
	0 vg Group 1	0.36x 10E12 vg Group 2	1.1x 10E12 vg Group 3	3.3x 10E12 vg Group 4	0 vg Group 1	0.36x 10E12 vg Group 2	1.1x 10E12 vg Group 3	3.3x 10E12 vg Group 4
Number of Animals:	5	5	5	5	5	5	5	5
**TESTIS**								
Examined	5	5	5	5	.	.	.	.
No Visible Lesions	5	3	4	5	.	.	.	.
Degeneration/atrophy; seminiferous tubule	0	1	1	0	.	.	.	.
.... mild	0	1	0	0	.	.	.	.
.... marked	0	0	1	0	.	.	.	.
Cellular debris	0	1	0	0	.	.	.	.
.... minimal	0	1	0	0	.	.	.	.
**THYMUS**								
Examined	5	5	5	5	5	5	5	5
No Visible Lesions	5	5	5	5	5	5	5	5
**UTERUS**								
Examined	.	.	.	.	5	5	5	5
No Visible Lesions	.	.	.	.	5	5	5	5
**NERVE ROOT, VENTRAL, CERVICAL**								
Examined	5	5	5	5	5	5	5	5
No Visible Lesions	5	5	5	5	5	5	5	5
**NERVE ROOT, VENTRAL, THORACIC**								
Examined	5	5	5	5	5	5	5	5
No Visible Lesions	5	5	5	5	5	5	5	5
**NERVE ROOT, VENTRAL, LUMBAR**								
Examined	5	5	5	5	5	5	5	5
No Visible Lesions	5	5	5	5	5	5	5	5
**NERVE ROOT, DORSAL, CERVICAL**								
Examined	5	5	5	5	5	5	5	5
No Visible Lesions	5	5	5	5	5	5	5	5
**NERVE ROOT, DORSAL, LUMBAR**								
Examined	5	5	5	5	5	5	5	5

Appendix 18

Table 5

Summary of Microscopic Pathology: Day 29

5550008

Removal Reason(s): RECOVERY EUTHANASIA	Male				Female			
Summary: Incidence	0 vg Group 1	0.36x 10E12 vg Group 2	1.1x 10E12 vg Group 3	3.3x 10E12 vg Group 4	0 vg Group 1	0.36x 10E12 vg Group 2	1.1x 10E12 vg Group 3	3.3x 10E12 vg Group 4
Number of Animals:	5	5	5	5	5	5	5	5
**NERVE ROOT, DORSAL, LUMBAR (Continued...)**								
No Visible Lesions	5	4	1	1	5	5	1	0
Degeneration; axonal	0	1	4	4	0	0	4	5
.... minimal	0	1	3	1	0	0	3	1
.... mild	0	0	1	3	0	0	1	3
.... moderate	0	0	0	0	0	0	0	1
**NERVE ROOT, DORSAL, THORACIC**								
Examined	5	5	5	5	5	5	5	5
No Visible Lesions	5	5	5	5	5	5	5	5

Appendix 18

Table 5

Summary of Microscopic Pathology: Day 91

5550008

Removal Reason(s): RECOVERY EUTHANASIA	Male				Female			
Summary: Incidence	0 vg Group 1	0.36x 10E12 vg Group 2	1.1x 10E12 vg Group 3	3.3x 10E12 vg Group 4	0 vg Group 1	0.36x 10E12 vg Group 2	1.1x 10E12 vg Group 3	3.3x 10E12 vg Group 4
Number of Animals:	5	5	5	5	5	5	5	5
**BRAIN**								
Examined	5	5	5	5	5	5	5	5
No Visible Lesions	5	5	5	5	5	5	5	5
**CERVIX**								
Examined	.	.	.	.	5	5	5	5
No Visible Lesions	.	.	.	.	5	5	5	5
**EPIDIDYMIS**								
Examined	5	5	5	5	.	.	.	.
No Visible Lesions	5	5	3	3	.	.	.	.
Cellular debris	0	0	0	1	.	.	.	.
.... moderate	0	0	0	1	.	.	.	.
Sperm granuloma	0	0	1	0	.	.	.	.
.... minimal	0	0	1	0	.	.	.	.
Infiltration, mononuclear cell	0	0	1	0	.	.	.	.
.... minimal	0	0	1	0	.	.	.	.
Peripuberty	0	0	0	1	.	.	.	.
Sperm, decreased	0	0	0	1	.	.	.	.
.... severe	0	0	0	1	.	.	.	.
**EYE**								
Examined	5	5	5	5	5	5	5	5
No Visible Lesions	5	5	5	5	5	5	5	5
**GANGLION, DORSAL ROOT, CERVICAL**								
Examined	5	5	5	5	5	5	5	5
No Visible Lesions	5	5	5	5	5	5	5	5
**GANGLION, DORSAL ROOT, LUMBAR**								
Examined	5	5	5	5	5	5	5	5
No Visible Lesions	5	5	1	2	5	4	1	2
Infiltration, mononuclear cell	0	0	2	3	0	1	2	2
.... minimal	0	0	2	2	0	1	2	2
.... mild	0	0	0	1	0	0	0	0
Degeneration; axonal	0	0	0	1	0	0	2	1

Appendix 18

Table 5

Summary of Microscopic Pathology: Day 91

5550008

Removal Reason(s): RECOVERY EUTHANASIA	Male				Female			
Summary: Incidence	0 vg Group 1	0.36x 10E12 vg Group 2	1.1x 10E12 vg Group 3	3.3x 10E12 vg Group 4	0 vg Group 1	0.36x 10E12 vg Group 2	1.1x 10E12 vg Group 3	3.3x 10E12 vg Group 4
Number of Animals:	5	5	5	5	5	5	5	5
**GANGLION, DORSAL ROOT, LUMBAR (Continued...)**								
.... minimal	0	0	0	1	0	0	2	1
Degeneration; neuronal	0	0	2	3	0	0	1	2
.... minimal	0	0	2	3	0	0	1	2
**GANGLION, DORSAL ROOT, THORACIC**								
Examined	5	5	5	5	5	5	5	5
No Visible Lesions	5	5	5	5	5	5	5	5
**GLAND, ADRENAL**								
Examined	5	5	5	5	5	5	5	5
No Visible Lesions	2	4	4	3	5	5	5	5
Vacuolation; cortical	3	1	1	2	0	0	0	0
.... minimal	1	1	1	1	0	0	0	0
.... mild	2	0	0	1	0	0	0	0
**GLAND, PARATHYROID**								
Examined	4	5	5	5	3	5	3	5
No Visible Lesions	4	5	5	5	3	5	3	5
Not Examined: Not Present In Section.	1	0	0	0	2	0	2	0
**GLAND, PITUITARY**								
Examined	5	5	5	5	5	5	5	5
No Visible Lesions	5	5	5	5	5	5	4	4
Hypertrophy/hyperplasia; pars distalis	0	0	0	0	0	0	1	1
.... mild	0	0	0	0	0	0	1	1
**GLAND, PROSTATE**								
Examined	5	5	5	5	.	.	.	.
No Visible Lesions	1	1	3	4	.	.	.	.
Infiltration, mononuclear cell	4	4	2	1	.	.	.	.
.... minimal	4	2	1	1	.	.	.	.
.... mild	0	2	0	0	.	.	.	.
.... moderate	0	0	1	0	.	.	.	.

Appendix 18

Table 5

Summary of Microscopic Pathology: Day 91

5550008

Removal Reason(s): RECOVERY EUTHANASIA	Male				Female			
Summary: Incidence	0 vg Group 1	0.36x 10E12 vg Group 2	1.1x 10E12 vg Group 3	3.3x 10E12 vg Group 4	0 vg Group 1	0.36x 10E12 vg Group 2	1.1x 10E12 vg Group 3	3.3x 10E12 vg Group 4
Number of Animals:	5	5	5	5	5	5	5	5
**GLAND, PROSTATE (Continued...)**								
Inflammation, mixed cell	0	0	1	0	.	.	.	.
.... mild	0	0	1	0	.	.	.	.
**GLAND, SEMINAL VESICLE**								
Examined	0	0	0	1	.	.	.	.
No Visible Lesions	.	.	.	1	.	.	.	.
**GLAND, THYROID**								
Examined	5	5	5	5	5	5	5	5
No Visible Lesions	5	4	5	5	5	5	5	5
Hypertrophy; follicle	0	1	0	0	0	0	0	0
.... moderate	0	1	0	0	0	0	0	0
**HEART**								
Examined	5	5	5	5	5	5	5	5
No Visible Lesions	0	3	3	5	4	4	5	5
Infiltration, mononuclear cell/degeneration, myofiber	5	2	2	0	1	1	0	0
.... minimal	5	2	2	0	1	1	0	0
**KIDNEY**								
Examined	5	5	5	5	5	5	5	5
No Visible Lesions	1	1	2	1	4	2	3	4
Infiltration, mononuclear cell	2	3	2	4	0	1	2	0
.... minimal	2	3	2	4	0	1	2	0
Dilatation; tubular	0	1	1	0	0	2	0	1
.... minimal	0	1	0	0	0	0	0	1
.... mild	0	0	1	0	0	2	0	0
Chronic progressive nephropathy	2	3	2	1	1	1	1	0
.... minimal	2	2	2	1	1	1	1	0
.... mild	0	1	0	0	0	0	0	0
**LIVER**								
Examined	5	5	5	5	5	5	5	5
No Visible Lesions	1	0	0	0	0	0	0	0
Necrosis	0	0	1	0	0	0	0	0

Appendix 18

Table 5

Summary of Microscopic Pathology: Day 91

5550008

Removal Reason(s): RECOVERY EUTHANASIA	Male				Female			
Summary: Incidence	0 vg Group 1	0.36x 10E12 vg Group 2	1.1x 10E12 vg Group 3	3.3x 10E12 vg Group 4	0 vg Group 1	0.36x 10E12 vg Group 2	1.1x 10E12 vg Group 3	3.3x 10E12 vg Group 4
Number of Animals:	5	5	5	5	5	5	5	5
**LIVER (Continued...)**								
.... minimal	0	0	1	0	0	0	0	0
Vacuolation; hepatocellular	0	1	0	0	0	0	0	0
.... minimal	0	1	0	0	0	0	0	0
Vacuolation; hepatocellular, periportal	0	3	1	2	1	1	1	2
.... minimal	0	2	1	2	1	1	1	2
.... mild	0	1	0	0	0	0	0	0
Tension lipidosis	1	2	2	1	0	1	0	0
.... minimal	1	2	0	0	0	0	0	0
.... mild	0	0	2	1	0	1	0	0
Infiltration, mixed cell	2	3	2	2	5	5	5	5
.... minimal	2	3	2	2	5	5	5	5
Infiltration, mononuclear cell	2	0	1	1	0	0	0	0
.... minimal	2	0	1	1	0	0	0	0
Infiltration, mononuclear cell; periportal	1	2	1	2	1	0	1	1
.... minimal	1	2	1	2	1	0	1	1
**LUNG**								
Examined	5	5	5	5	5	5	5	5
No Visible Lesions	4	5	5	5	5	5	5	5
Hemorrhage; acute	1	0	0	0	0	0	0	0
.... minimal	1	0	0	0	0	0	0	0
**LYMPH NODE**								
Examined	.	.	.	.	1	0	0	0
Hemorrhage; acute	.	.	.	.	1	.	.	.
.... minimal	.	.	.	.	1	.	.	.
**LYMPH NODE, CERVICAL**								
Examined	5	5	5	5	5	5	5	5
No Visible Lesions	5	5	4	5	5	3	5	5
Hemorrhage; acute	0	0	1	0	0	2	0	0
.... minimal	0	0	1	0	0	0	0	0
.... mild	0	0	0	0	0	2	0	0

Appendix 18

Table 5

Summary of Microscopic Pathology: Day 91

5550008

Removal Reason(s): RECOVERY EUTHANASIA	Male				Female			
Summary: Incidence	0 vg Group 1	0.36x 10E12 vg Group 2	1.1x 10E12 vg Group 3	3.3x 10E12 vg Group 4	0 vg Group 1	0.36x 10E12 vg Group 2	1.1x 10E12 vg Group 3	3.3x 10E12 vg Group 4
Number of Animals:	5	5	5	5	5	5	5	5
**LYMPH NODE, ILIAC**								
Examined	5	5	4	5	5	5	5	5
No Visible Lesions	4	5	4	5	4	5	5	5
Not Examined: Not Present In Section.	0	0	1	0	.	.	.	.
Hemorrhage; acute	1	0	0	0	1	0	0	0
.... mild	1	0	0	0	1	0	0	0
**LYMPH NODE, MANDIBULAR**								
Examined	5	5	5	5	5	5	5	5
No Visible Lesions	5	5	5	4	5	4	4	3
Hemorrhage; acute	0	0	0	0	0	1	0	0
.... moderate	0	0	0	0	0	1	0	0
Plasmacytosis	0	0	0	0	0	0	0	2
.... mild	0	0	0	0	0	0	0	2
Cellularity, increased; lymphoid	0	0	0	1	0	0	1	1
.... mild	0	0	0	1	0	0	1	0
.... moderate	0	0	0	0	0	0	0	1
**LYMPH NODE, MESENTERIC**								
Examined	5	5	5	5	5	5	5	5
No Visible Lesions	5	5	5	5	5	5	5	4
Cellularity, increased; lymphoid	0	0	0	0	0	0	0	1
.... mild	0	0	0	0	0	0	0	1
**MUSCLE, BICEPS FEMORIS**								
Examined	5	5	5	5	5	5	5	5
No Visible Lesions	5	5	5	5	5	5	5	5
**MUSCLE, GASTROCNEMIUS**								
Examined	5	5	5	5	5	5	5	5
No Visible Lesions	5	5	5	5	5	5	5	5
**NERVE, OPTIC**								
Examined	5	5	5	4	5	5	5	5
No Visible Lesions	5	5	5	4	5	5	5	5

Appendix 18
Table 5

Summary of Microscopic Pathology: Day 91

5550008

Removal Reason(s): RECOVERY EUTHANASIA	Male				Female			
Summary: Incidence	0 vg Group 1	0.36x 10E12 vg Group 2	1.1x 10E12 vg Group 3	3.3x 10E12 vg Group 4	0 vg Group 1	0.36x 10E12 vg Group 2	1.1x 10E12 vg Group 3	3.3x 10E12 vg Group 4
Number of Animals:	5	5	5	5	5	5	5	5
**NERVE, OPTIC (Continued...)**								
Not Examined: Not Present In Section.	0	0	0	1	.	.	.	.
**NERVE, SCIATIC**								
Examined	5	4	5	5	5	5	5	5
No Visible Lesions	5	2	2	1	5	4	0	0
Degeneration; axonal	0	2	3	4	0	1	5	5
.... minimal	0	2	3	4	0	1	5	5
**NERVE, TIBIAL**								
Examined	5	5	5	5	5	5	5	5
No Visible Lesions	5	3	3	1	5	3	1	0
Degeneration; axonal	0	2	2	4	0	2	4	5
.... minimal	0	2	2	4	0	2	4	5
**OVARY**								
Examined	.	.	.	.	5	5	5	5
No Visible Lesions	.	.	.	.	5	5	5	5
**SITE, INJECTION**								
Examined	5	5	5	5	5	5	5	5
No Visible Lesions	4	4	2	1	4	1	0	0
Infiltration, mononuclear cell	0	0	1	0	0	0	0	0
.... minimal	0	0	1	0	0	0	0	0
Infiltration, mononuclear cell; leptomeninges	1	1	2	1	1	4	4	4
.... minimal	1	1	2	1	1	4	4	4
Degeneration; axonal, cauda equina	0	0	0	4	0	0	3	4
.... minimal	0	0	0	4	0	0	3	4
**SMALL INTESTINE, JEJUNUM**								
Examined	0	1	0	0	.	.	.	.
No Visible Lesions	.	1	.	.	.	.	.	.
**SPINAL CORD, CERVICAL**								
Examined	5	5	5	5	5	5	5	5
No Visible Lesions	5	5	5	5	5	5	5	5

Appendix 18

Table 5

Summary of Microscopic Pathology: Day 91

5550008

Removal Reason(s): RECOVERY EUTHANASIA	Male				Female			
Summary: Incidence	0 vg Group 1	0.36x 10E12 vg Group 2	1.1x 10E12 vg Group 3	3.3x 10E12 vg Group 4	0 vg Group 1	0.36x 10E12 vg Group 2	1.1x 10E12 vg Group 3	3.3x 10E12 vg Group 4
Number of Animals:	5	5	5	5	5	5	5	5
**SPINAL CORD, LUMBAR**								
Examined	5	5	5	5	5	5	5	5
No Visible Lesions	5	5	5	5	5	5	3	5
Infiltration, mononuclear cell; leptomeninges	0	0	0	0	0	0	2	0
.... minimal	0	0	0	0	0	0	2	0
**SPINAL CORD, THORACIC**								
Examined	5	5	5	5	5	5	5	5
No Visible Lesions	5	5	5	5	5	5	4	3
Infiltration, mononuclear cell; adipose tissue	0	0	0	0	0	0	1	2
.... minimal	0	0	0	0	0	0	1	2
**SPLEEN**								
Examined	5	5	5	5	5	5	5	5
No Visible Lesions	5	5	5	5	5	5	5	5
**STOMACH**								
Examined	0	1	0	0	.	.	.	.
No Visible Lesions	.	1	.	.	.	.	.	.
**TESTIS**								
Examined	5	5	5	5	.	.	.	.
No Visible Lesions	5	5	5	3	.	.	.	.
Vacuolation; sertoli cell	0	0	0	1	.	.	.	.
.... minimal	0	0	0	1	.	.	.	.
Peripuberty	0	0	0	1	.	.	.	.
Degeneration/atrophy; seminiferous tubule	0	0	0	1	.	.	.	.
.... moderate	0	0	0	1	.	.	.	.
**THYMUS**								
Examined	5	5	5	5	5	5	5	5
No Visible Lesions	5	5	5	5	5	5	5	5
**TONGUE**								
Examined	0	0	1	0	.	.	.	.
No Visible Lesions	.	.	1	.	.	.	.	.

Appendix 18

Table 5

Summary of Microscopic Pathology: Day 91

5550008

Removal Reason(s): RECOVERY EUTHANASIA	Male				Female			
Summary: Incidence	0 vg Group 1	0.36x 10E12 vg Group 2	1.1x 10E12 vg Group 3	3.3x 10E12 vg Group 4	0 vg Group 1	0.36x 10E12 vg Group 2	1.1x 10E12 vg Group 3	3.3x 10E12 vg Group 4
Number of Animals:	5	5	5	5	5	5	5	5
**UTERUS**								
Examined	.	.	.	.	5	5	5	5
No Visible Lesions	.	.	.	.	5	5	5	5
**NERVE ROOT, VENTRAL, CERVICAL**								
Examined	5	5	5	5	5	5	5	5
No Visible Lesions	5	5	5	5	5	5	5	5
**NERVE ROOT, VENTRAL, THORACIC**								
Examined	5	5	5	5	5	5	5	5
No Visible Lesions	5	5	5	5	5	5	5	5
**NERVE ROOT, VENTRAL, LUMBAR**								
Examined	5	5	5	5	5	5	5	5
No Visible Lesions	5	5	5	5	5	5	5	5
**NERVE ROOT, DORSAL, CERVICAL**								
Examined	5	5	5	5	5	5	5	5
No Visible Lesions	5	5	5	5	5	5	5	5
**NERVE ROOT, DORSAL, LUMBAR**								
Examined	5	5	5	5	5	5	5	5
No Visible Lesions	5	4	1	1	5	3	0	0
Degeneration; axonal	0	1	4	4	0	2	5	5
.... minimal	0	1	4	2	0	2	5	1
.... mild	0	0	0	2	0	0	0	4
**NERVE ROOT, DORSAL, THORACIC**								
Examined	5	5	5	5	5	5	5	5
No Visible Lesions	5	5	5	5	5	5	5	5

Individual Absolute Organ Weights Explanation Page

Abbreviation	Description
Wt	Weight
FC	Flag comment
I	Macroscopic pathology – Included in mean
RC	Result comment

Note: This is a comprehensive list of abbreviations. All of the abbreviations listed may not be applicable to this report.

Dosing Information

Dosing information is abbreviated on various data outputs; the following represents the dosing information for this study:

Group No.	Test Material	Dose Level (vg)
1	Reference Item	0
2	AAV9/AP4M1	3.6E11
3	AAV9/AP4M1	1.1E12
4	AAV9/AP4M1	3.3E12

Appendix 18

Appendix 1

Individual Absolute Organ Weights: Day 8

5550008

Sex: Male Day(s) Relative to Start Date

0<br>vg<br>Group 1	Organ Weight (Absolute)						
	Terminal Body Wt (g)	Brain (g)	Epididymis (g)	Gland Adrenal (g)	Gland Pituitary (g)	Gland Prostate (g)	Thyroid/ Parathyroid (g)
	-	-	-	-	-	-	-
1001	242 [a]	1.950	0.388	0.0395	0.0122	0.401	0.0116
1002	224 [a]	1.869	0.418	0.0440	0.0093	0.491	0.0155
1014	264 [a]	1.862	0.565	0.0428	0.0111	0.480	0.0117
1015	247	1.981	0.530	0.0407	0.0082	0.400	0.0081
Mean	244.3	1.9155	0.4753	0.04175	0.01020	0.4430	0.01173
SD	16.5	0.0592	0.0855	0.00203	0.00179	0.0493	0.00302
N	4	4	4	4	4	4	4

[a] [RC:Value Confirmed]

Appendix 18

Appendix 1

Individual Absolute Organ Weights: Day 8

5550008

Sex: Male Day(s) Relative to Start Date

0	Organ Weight (Absolute)					
vg	Heart	Kidney	Liver	Spleen	Testis	Thymus
Group 1	(g)	(g)	(g)	(g)	(g)	(g)
	-	-	-	-	-	-
1001	0.950	2.169	8.401	0.696	2.836	0.582
1002	0.870	1.911	7.801	0.564	2.918	0.608
1014	0.879	2.144	8.306	0.748	2.936	0.511
1015	0.921	2.302	7.915	0.637	2.888	0.502
Mean	0.9050	2.1315	8.1058	0.6613	2.8945	0.5508
SD	0.0373	0.1625	0.2924	0.0791	0.0437	0.0523
N	4	4	4	4	4	4

Appendix 18
Appendix 1

Individual Absolute Organ Weights: Day 8

5550008
Sex: Male Day(s) Relative to Start Date

0.36x 10E12 vg Group 2	Organ Weight (Absolute)						
	Terminal Body Wt (g)	Brain (g)	Epididymis (g)	Gland Adrenal (g)	Gland Pituitary (g)	Gland Prostate (g)	Thyroid/ Parathyroid (g)
	-	-	-	-	-	-	-
2004	188	1.858	0.340 [b]	0.0330	0.0083	0.409	0.0142 [c]
2009	281 [a]	1.942	0.470	0.0447	0.0124	0.520	0.0111
2010	272 [a]	2.125	0.544	0.0530	0.0129	0.382	0.0110
2014	256	1.886	0.614	0.0453	0.0107	0.499	0.0090
2015	221	1.897	0.556	0.0419	0.0096	0.550	0.0081
Mean	243.6	1.9416	0.5048	0.04358	0.01078	0.4720	0.01068
SD	38.6	0.1069	0.1054	0.00721	0.00191	0.0728	0.00235
N	5	5	5	5	5	5	5
%Diff	-0.3	1.3626	6.2178	4.38323	5.68627	6.5463	-8.91258

[a] [RC:Value Confirmed]
[b] [RC:Value confirmed.]
[c] [RC:weighed after approximately 20 minutes in formalin]

Appendix 18

Appendix 1

Individual Absolute Organ Weights: Day 8

5550008

Sex: Male Day(s) Relative to Start Date

0.36x 10E12 vg Group 2	Organ Weight (Absolute)					
	Heart (g)	Kidney (g)	Liver (g)	Spleen (g)	Testis (g)	Thymus (g)
	-	-	-	-	-	-
2004	0.728	1.563	6.389	0.482	2.467	0.504
2009	1.243	2.610	9.165	0.640	2.464	0.713
2010	1.036	2.465	9.857	0.698	3.201	0.469
2014	1.019	2.069	8.125	0.664	3.079	0.525
2015	0.898	1.851	7.016	0.422	2.804	0.538
Mean	0.9848	2.1116	8.1104	0.5812	2.8030	0.5498
SD	0.1897	0.4313	1.4426	0.1216	0.3400	0.0949
N	5	5	5	5	5	5
%Diff	8.8177	-0.9336	0.0574	-12.1059	-3.1612	-0.1725

Appendix 18

Appendix 1

Individual Absolute Organ Weights: Day 8

5550008

Sex: Male Day(s) Relative to Start Date

1.1x 10E12 vg Group 3	Organ Weight (Absolute)						
	Terminal Body Wt (g)	Brain (g)	Epididymis (g)	Gland Adrenal (g)	Gland Pituitary (g)	Gland Prostate (g)	Thyroid/ Parathyroid (g)
	-	-	-	-	-	-	-
3001	211	1.812	0.496	0.0316	0.0067	0.498	0.0102
3002	244	1.932	0.472	0.0349	0.0110	0.521	0.0092
3003	204	1.877	0.346 [a]	0.0371	0.0069	0.389	0.0081
3012	245	1.955	0.513	0.0402	0.0087	0.528	0.0080
3013	258	2.001	0.566	0.0529	0.0111	0.510	0.0092
Mean	232.4	1.9154	0.4786	0.03934	0.00888	0.4892	0.00894
SD	23.5	0.0730	0.0818	0.00820	0.00213	0.0572	0.00091
N	5	5	5	5	5	5	5
%Diff	-4.9	-0.0052	0.7049	-5.77246	-12.94118	10.4289	-23.75267

[a] [RC:Value confirmed.]

Appendix 18

Appendix 1

Individual Absolute Organ Weights: Day 8

5550008

Sex: Male Day(s) Relative to Start Date

1.1x 10E12 vg Group 3	Organ Weight (Absolute)					
	Heart (g)	Kidney (g)	Liver (g)	Spleen (g)	Testis (g)	Thymus (g)
	-	-	-	-	-	-
3001	0.836	1.752	7.174	0.583	2.660	0.519
3002	0.958	2.168	8.760	0.609	2.866	0.590
3003	0.808	1.759	6.878	0.448	2.709	0.663
3012	1.063	2.136	7.744	0.586	2.568	0.480
3013	1.152	2.102	8.233	0.553	2.935	0.510
Mean	0.9634	1.9834	7.7578	0.5558	2.7476	0.5524
SD	0.1465	0.2094	0.7657	0.0635	0.1505	0.0738
N	5	5	5	5	5	5
%Diff	6.4530	-6.9482	-4.2926	-15.9471	-5.0751	0.2996

Appendix 18

Appendix 1

Individual Absolute Organ Weights: Day 8

5550008

Sex: Male Day(s) Relative to Start Date

3.3x 10E12 vg Group 4	Organ Weight (Absolute)						
	Terminal Body Wt (g)	Brain (g)	Epididymis (g)	Gland Adrenal (g)	Gland Pituitary (g)	Gland Prostate (g)	Thyroid/ Parathyroid (g)
	-	-	-	-	-	-	-
4001	213	1.801	0.421	0.0432	0.0080	0.520	0.0116
4002	206	1.852	0.363 [a]	0.0300	0.0082	0.226	0.0121
4006	207	2.004	0.524	0.0379	0.0090	0.561	0.0125
4007	181	1.851	0.427	0.0377	0.0075	0.343	0.0114
4008	259	1.803	0.447	0.0354	0.0126	0.487	0.0120
Mean	213.2	1.8622	0.4364	0.03684	0.00906	0.4274	0.01192
SD	28.4	0.0830	0.0581	0.00477	0.00205	0.1393	0.00043
N	5	5	5	5	5	5	5
%Diff	-12.7	-2.7826	-8.1746	-11.76048	-11.17647	-3.5214	1.66311

[a] [RC:Value confirmed.]

Appendix 18

Appendix 1

Individual Absolute Organ Weights: Day 8

5550008

Sex: Male Day(s) Relative to Start Date

3.3x 10E12 vg Group 4	Organ Weight (Absolute)					
	Heart (g)	Kidney (g)	Liver (g)	Spleen (g)	Testis (g)	Thymus (g)
	-	-	-	-	-	-
4001	0.853	1.947	7.061	0.651	2.330	0.475
4002	0.798	1.946	7.303	0.649	2.481	0.543
4006	0.884	1.832	6.747	0.409	2.861	0.324
4007	0.730	1.390	5.329	0.386	2.588	0.459
4008	0.983	2.146	8.093	0.581	2.920	0.729
Mean	0.8496	1.8522	6.9066	0.5352	2.6360	0.5060
SD	0.0948	0.2820	1.0128	0.1291	0.2506	0.1478
N	5	5	5	5	5	5
%Diff	-6.1215	-13.1034	-14.7938	-19.0624	-8.9307	-8.1253

Individual Absolute Organ Weights: Day 8

5550008
Sex: Female Day(s) Relative to Start Date

0<br>vg<br>Group 1	Organ Weight (Absolute)						
	Terminal Body Wt (g)	Brain (g)	Gland Adrenal (g)	Gland Pituitary (g)	Thyroid/ Parathyroid (g)	Heart (g)	Kidney (g)
	-	-	-	-	-	-	-
1501	171 [a]	1.767	0.0535	0.0150	0.0092	0.643	1.524
1502	166 [a]	1.636	0.0458	0.0120	0.0080	0.680	1.517
1505	169	1.883	0.0552	0.0142	0.0072	0.694	1.432
1506	155	1.833	0.0520	0.0124	0.0075	0.649	1.478
1507	169	1.768	0.0685	0.0128	0.0111	0.707	1.475
Mean	166.0	1.7774	0.05500	0.01328	0.00860	0.6746	1.4852
SD	6.4	0.0928	0.00834	0.00127	0.00159	0.0279	0.0371
N	5	5	5	5	5	5	5

[a] [RC:Value Confirmed]

Appendix 18

Appendix 1

Individual Absolute Organ Weights: Day 8

5550008

Sex: Female Day(s) Relative to Start Date

0<br>vg<br>Group 1	Organ Weight (Absolute)<br>Liver<br>(g)	 Ovary<br>(g)	 Spleen<br>(g)	 Thymus<br>(g)	 Uterus/<br>Cervix<br>(g)
	-	-	-	-	-
1501	5.981	0.068	0.367	0.356	0.426
1502	5.373	0.061	0.442	0.410	0.611
1505	5.705	0.077	0.375	0.421	0.450
1506	5.360	0.073	0.398	0.497	0.421
1507	6.792	0.077	0.389	0.412	0.438
Mean	5.8422	0.0712	0.3942	0.4192	0.4692
SD	0.5901	0.0068	0.0293	0.0505	0.0801
N	5	5	5	5	5

Appendix 18

Appendix 1

Individual Absolute Organ Weights: Day 8

5550008

Sex: Female Day(s) Relative to Start Date

0.36x 10E12 vg Group 2	Organ Weight (Absolute)						
	Terminal Body Wt (g)	Brain (g)	Gland Adrenal (g)	Gland Pituitary (g)	Thyroid/ Parathyroid (g)	Heart (g)	Kidney (g)
	-	-	-	-	-	-	-
2505	155	1.675	0.0441	0.0126	0.0064 [b]	0.651	1.325
2506	167	1.728	0.0514	0.0123	0.0106	0.611	1.279
2510	191 [a]	1.915	0.0567	0.0084	0.0077	0.746	1.550
2511	205 [a]	1.808	0.0574	0.0147	0.0078	0.784	1.805
2512	187 [a]	1.734	0.0609	0.0153	0.0083	0.779	1.541
Mean	181.0	1.7720	0.05410	0.01266	0.00816	0.7142	1.5000
SD	19.9	0.0929	0.00654	0.00271	0.00153	0.0786	0.2102
N	5	5	5	5	5	5	5
%Diff	9.0	-0.3038	-1.63636	-4.66867	-5.11628	5.8701	0.9965

[a] [RC:Value Confirmed]
[b] [RC:Value confirmed.]

Appendix 18

Appendix 1

Individual Absolute Organ Weights: Day 8

5550008

Sex: Female Day(s) Relative to Start Date

0.36x 10E12 vg Group 2	Organ Weight (Absolute)				
	Liver (g)	Ovary (g)	Spleen (g)	Thymus (g)	Uterus/ Cervix (g)
	-	-	-	-	-
2505	5.473	0.055	0.363	0.451	0.847 [a]
2506	4.864	0.051	0.346	0.441	0.336
2510	6.584	0.078	0.486	0.432	0.389
2511	6.051	0.084	0.557	0.474	1.087 [b]
2512	6.804	0.082	0.437	0.540	0.313
Mean	5.9552	0.0700	0.4378	0.4676	0.5944
SD	0.7982	0.0157	0.0874	0.0434	0.3516
N	5	5	5	5	5
%Diff	1.9342	-1.6854	11.0604	11.5458	26.6837

[a] [RC:Estrus.]
[b] [RC:estrus]

Appendix 18

Appendix 1

Individual Absolute Organ Weights: Day 8

5550008

Sex: Female Day(s) Relative to Start Date

1.1x 10E12 vg Group 3	Organ Weight (Absolute)						
	Terminal Body Wt (g)	Brain (g)	Gland Adrenal (g)	Gland Pituitary (g)	Thyroid/ Parathyroid (g)	Heart (g)	Kidney (g)
	-	-	-	-	-	-	-
3507	187 [a]	1.835	0.0488	0.0105	0.0099	0.680	1.630
3508	175 [a]	1.738	0.0498	0.0112	0.0097	0.652	1.659
3511	187	1.901	0.0733	0.0167	0.0068 [b]	0.801	1.593
3512	176	1.884	0.0693	0.0122	0.0093	0.743	1.469
3513	174	1.778	0.0527	0.0149	0.0072	0.735	1.543
Mean	179.8	1.8272	0.05878	0.01310	0.00858	0.7222	1.5788
SD	6.6	0.0691	0.01161	0.00262	0.00147	0.0581	0.0752
N	5	5	5	5	5	5	5
%Diff	8.3	2.8018	6.87273	-1.35542	-0.23256	7.0560	6.3022

[a] [RC:Value Confirmed]
[b] [RC:Value confirmed.]

Appendix 18

Appendix 1

Individual Absolute Organ Weights: Day 8

5550008

Sex: Female Day(s) Relative to Start Date

1.1x 10E12 vg Group 3	Organ Weight (Absolute)				
	Liver (g)	Ovary (g)	Spleen (g)	Thymus (g)	Uterus/ Cervix (g)
	-	-	-	-	-
3507	5.589	0.084	0.440	0.364	0.723
3508	6.056	0.079	0.477	0.354	0.357
3511	5.837	0.094	0.367	0.363	0.696
3512	4.802	0.069	0.403	0.311	0.671 [a]
3513	5.220	0.077	0.444	0.405	0.372
Mean	5.5008	0.0806	0.4262	0.3594	0.5638
SD	0.4991	0.0092	0.0422	0.0335	0.1829
N	5	5	5	5	5
%Diff	-5.8437	13.2022	8.1177	-14.2653	20.1620

[a] [RC:estrus]

Appendix 18

Appendix 1

Individual Absolute Organ Weights: Day 8

5550008

Sex: Female Day(s) Relative to Start Date

3.3x 10E12 vg Group 4	Organ Weight (Absolute)						
	Terminal Body Wt (g)	Brain (g)	Gland Adrenal (g)	Gland Pituitary (g)	Thyroid/ Parathyroid (g)	Heart (g)	Kidney (g)
	-	-	-	-	-	-	-
4601	179 [a]	1.895	0.0577	0.0137	0.0094	0.711	1.393
4502	157	1.744	0.0495	0.0101	0.0068 I	0.581	1.313
4508	160 [a]	1.756	0.0424	0.0109	0.0075	0.634	1.341
4509	179 [a]	1.883	0.0526	0.0148	0.0058 [b]	0.771	1.520
4510	160 [a]	1.404 [b]	0.0446	0.0129	0.0070	0.665	1.398
Mean	167.0	1.7364	0.04936	0.01248	0.00730	0.6724	1.3930
SD	11.0	0.1985	0.00615	0.00195	0.00133	0.0726	0.0795
N	5	5	5	5	5	5	5
%Diff	0.6	-2.3067	-10.25455	-6.02410	-15.11628	-0.3261	-6.2079

I = Include
[a] [RC:Value Confirmed]
[b] [RC:Value confirmed.]

Appendix 18
Appendix 1

Individual Absolute Organ Weights: Day 8

5550008
Sex: Female Day(s) Relative to Start Date

3.3x 10E12 vg Group 4	Organ Weight (Absolute)				
	Liver (g)	Ovary (g)	Spleen (g)	Thymus (g)	Uterus/ Cervix (g)
	-	-	-	-	-
4601	6.150	0.073	0.374	0.383	0.340
4502	5.051	0.057	0.312	0.219	0.858 [a]
4508	5.075	0.080	0.324	0.454	0.406
4509	5.907	0.067	0.412	0.418	0.965 I
4510	5.140	0.054	0.356	0.325	0.731 [a]
Mean	5.4646	0.0662	0.3556	0.3598	0.6600
SD	0.5229	0.0108	0.0401	0.0920	0.2758
N	5	5	5	5	5
%Diff	-6.4633	-7.0225	-9.7920	-14.1698	40.6650

I = Include
[a] [RC:estrus]

Appendix 18
Appendix 1

Individual Absolute Organ Weights: Day 29

5550008
Sex: Male Day(s) Relative to Start Date

0<br>vg<br>Group 1	Organ Weight (Absolute)						
	Terminal Body Wt (g)	Brain (g)	Epididymis (g)	Gland Adrenal (g)	Gland Pituitary (g)	Gland Prostate (g)	Thyroid/ Parathyroid (g)
	-	-	-	-	-	-	-
1003	349 [a]	1.967	0.941	0.0543	0.0131	0.956	0.0156
1004	345 [a]	2.069	0.781	0.0472	0.0053	0.583	0.0153
1008	365	1.939	1.005	0.0553	0.0115	0.979	0.0099
1009	384	1.970	0.963	0.0668	0.0145	1.048	0.0191
1010	401	2.198	0.981	0.0640	0.0150	1.038	0.0174
Mean	368.8	2.0286	0.9342	0.05752	0.01188	0.9208	0.01546
SD	23.7	0.1068	0.0888	0.00790	0.00392	0.1928	0.00346
N	5	5	5	5	5	5	5

[a] [RC:VALUE CONFIRMED]

Individual Absolute Organ Weights: Day 29

5550008
Sex: Male Day(s) Relative to Start Date

0	Organ Weight (Absolute)					
vg	Heart	Kidney	Liver	Spleen	Testis	Thymus
Group 1	(g)	(g)	(g)	(g)	(g)	(g)
	-	-	-	-	-	-
1003	1.348	2.591	9.412	0.778	3.808	0.496
1004	1.162	2.524	10.120	0.549	2.905	0.557
1008	1.176	2.490	9.290	0.817	3.249	0.754
1009	1.127	2.697	9.516	0.791	2.889	0.458
1010	1.318	2.704	10.525	1.180	3.860	0.544
Mean	1.2262	2.6012	9.7726	0.8230	3.3422	0.5618
SD	0.0997	0.0977	0.5281	0.2267	0.4718	0.1144
N	5	5	5	5	5	5

Appendix 18

Appendix 1

Individual Absolute Organ Weights: Day 29

5550008

Sex: Male Day(s) Relative to Start Date

0.36x 10E12 vg Group 2	Organ Weight (Absolute)						
	Terminal Body Wt (g)	Brain (g)	Epididymis (g)	Gland Adrenal (g)	Gland Pituitary (g)	Gland Prostate (g)	Thyroid/ Parathyroid (g)
	-	-	-	-	-	-	-
2001	380 [a]	2.207	0.894	0.0523	0.0145	0.922	0.0120
2002	368 [a]	2.185	1.001	0.0614	0.0106	0.967	0.0137
2011	356	2.128	1.062	0.0607	0.0137	0.994	0.0220
2012	389	2.063	0.912	0.0504	0.0071	1.065	0.0211
2013	352	2.134	0.960	0.0588	0.0128	0.749	0.0093
Mean	369.0	2.1434	0.9658	0.05672	0.01174	0.9394	0.01562
SD	15.7	0.0560	0.0681	0.00504	0.00298	0.1184	0.00565
N	5	5	5	5	5	5	5
%Diff	0.1	5.6591	3.3826	-1.39082	-1.17845	2.0200	1.03493

[a] [RC:VALUE CONFIRMED]

Appendix 18

Appendix 1

Individual Absolute Organ Weights: Day 29

5550008

Sex: Male Day(s) Relative to Start Date

0.36x 10E12 vg Group 2	Organ Weight (Absolute)					
	Heart (g)	Kidney (g)	Liver (g)	Spleen (g)	Testis (g)	Thymus (g)
	-	-	-	-	-	-
2001	1.404	2.822	10.725	0.717	3.820	0.419
2002	1.329	2.719	12.354	0.861	3.322	0.757
2011	1.478	2.598	9.844	0.811	3.550	0.635
2012	1.266	2.543	10.904	0.673	3.044	0.623
2013	1.380	2.617	10.314	0.710	3.219	0.365
Mean	1.3714	2.6598	10.8282	0.7544	3.3910	0.5598
SD	0.0797	0.1108	0.9457	0.0784	0.3017	0.1630
N	5	5	5	5	5	5
%Diff	11.8415	2.2528	10.8016	-8.3354	1.4601	-0.3560

Appendix 18

Appendix 1

Individual Absolute Organ Weights: Day 29

5550008

Sex: Male Day(s) Relative to Start Date

1.1x 10E12 vg Group 3	Organ Weight (Absolute)						
	Terminal Body Wt (g)	Brain (g)	Epididymis (g)	Gland Adrenal (g)	Gland Pituitary (g)	Gland Prostate (g)	Thyroid/ Parathyroid (g)
	-	-	-	-	-	-	-
3004	337 [a]	1.982	0.857	0.0519	0.0126	0.607	0.0110
3005	377 [a]	1.927	1.008	0.0522	0.0130	1.092	0.0148
3009	372	2.067	1.028	0.0623	0.0127	0.700	0.0122
3010	373	2.109	1.065	0.0494	0.0136	1.129	0.0176
3011	321	1.990	0.731 I	0.0506	0.0111	0.813	0.0102
Mean	356.0	2.0150	0.9378	0.05328	0.01260	0.8682	0.01316
SD	25.4	0.0725	0.1401	0.00516	0.00092	0.2333	0.00303
N	5	5	5	5	5	5	5
%Diff	-3.5	-0.6704	0.3854	-7.37135	6.06061	-5.7124	-14.87710

I = Include

[a] [RC:Value Confirmed]

Appendix 18

Appendix 1

Individual Absolute Organ Weights: Day 29

5550008

Sex: Male Day(s) Relative to Start Date

1.1x 10E12 vg Group 3	Organ Weight (Absolute)					
	Heart (g)	Kidney (g)	Liver (g)	Spleen (g)	Testis (g)	Thymus (g)
	-	-	-	-	-	-
3004	1.253	2.207	8.877	0.661	3.242	0.437
3005	1.372	2.509	10.466	0.666	3.587	0.488
3009	1.299	2.574	10.796	0.733	3.730	0.529
3010	1.272	2.319	10.346	0.668	3.594	0.518
3011	1.128	2.416	10.139	0.629	2.219 I	0.542
Mean	1.2648	2.4050	10.1248	0.6714	3.2744	0.5028
SD	0.0888	0.1467	0.7371	0.0379	0.6169	0.0418
N	5	5	5	5	5	5
%Diff	3.1479	-7.5427	3.6040	-18.4204	-2.0286	-10.5020

I = Include

Appendix 18

Appendix 1

Individual Absolute Organ Weights: Day 29

5550008

Sex: Male Day(s) Relative to Start Date

3.3x 10E12 vg Group 4	Organ Weight (Absolute)						
	Terminal Body Wt (g)	Brain (g)	Epididymis (g)	Gland Adrenal (g)	Gland Pituitary (g)	Gland Prostate (g)	Thyroid/ Parathyroid (g)
	-	-	-	-	-	-	-
4003	357 [a]	2.005	0.839	0.0455	0.0134	0.701	0.0116
4004	487 [a]	2.117	0.963	0.0684	0.0168	1.168	0.0255 I
4005	332 [a]	1.932	0.824	0.0501	0.0132	0.615	0.0186
4012	355	2.096	0.977	0.0516	0.0116	0.792	0.0107
4013	468	2.148	1.137	0.0856	0.0187	1.026	0.0178
Mean	399.8	2.0596	0.9480	0.06024	0.01474	0.8604	0.01684
SD	71.9	0.0890	0.1265	0.01662	0.00291	0.2304	0.00600
N	5	5	5	5	5	5	5
%Diff	8.4	1.5281	1.4772	4.72879	24.07407	-6.5595	8.92626

I = Include
[a] [RC:Value Confirmed]

Appendix 18

Appendix 1

Individual Absolute Organ Weights: Day 29

5550008

Sex: Male Day(s) Relative to Start Date

3.3x 10E12 vg Group 4	Organ Weight (Absolute)					
	Heart (g)	Kidney (g)	Liver (g)	Spleen (g)	Testis (g)	Thymus (g)
	-	-	-	-	-	-
4003	1.228	2.742	10.777	0.707	3.260	0.373
4004	1.510	3.104	16.186	1.210	3.702	0.966
4005	1.183	2.589	9.567	0.745	3.320	0.497
4012	1.279	2.798	10.908	0.757	3.409	0.515
4013	1.552	3.433	13.692	0.743	3.932	0.516
Mean	1.3504	2.9332	12.2260	0.8324	3.5246	0.5734
SD	0.1690	0.3363	2.6804	0.2119	0.2840	0.2274
N	5	5	5	5	5	5
%Diff	10.1289	12.7633	25.1049	1.1422	5.4575	2.0648

Appendix 18
Appendix 1

Individual Absolute Organ Weights: Day 29

5550008
Sex: Female Day(s) Relative to Start Date

0 vg Group 1	Terminal Body Wt (g)	Organ Weight (Absolute) Brain (g)	Gland Adrenal (g)	Gland Pituitary (g)	Thyroid/ Parathyroid (g)	Heart (g)	Kidney (g)
	-	-	-	-	-	-	-
1508	182	1.678	0.0508	0.0070	0.0114	0.690	1.230
1509	204	1.880	0.0571	0.0131	0.0118	0.782	1.473
1513	224 [a]	1.906	0.0571	0.0123	0.0138	0.838	1.633
1514	210	1.892	0.0598	0.0169	0.0115	0.743	1.355
1515	181	1.776	0.0474	0.0153	0.0085	0.652	1.309
Mean	200.2	1.8264	0.05444	0.01292	0.01140	0.7410	1.4000
SD	18.6	0.0976	0.00514	0.00377	0.00189	0.0735	0.1572
N	5	5	5	5	5	5	5

[a] [RC:VALUE CONFIRMED]

Appendix 18

Appendix 1

Individual Absolute Organ Weights: Day 29

5550008

Sex: Female Day(s) Relative to Start Date

0	Organ Weight (Absolute)				
vg	Liver	Ovary	Spleen	Thymus	Uterus/ Cervix
Group 1	(g)	(g)	(g)	(g)	(g)
	-	-	-	-	-
1508	4.842	0.079	0.383	0.297	0.838 [a]
1509	6.178	0.066	0.455	0.329	0.819 [a]
1513	5.398	0.073	0.482	0.455	1.236 [a]
1514	5.867	0.102	0.384	0.334	0.398
1515	5.053	0.081	0.419	0.285	0.793
Mean	5.4676	0.0802	0.4246	0.3400	0.8168
SD	0.5550	0.0135	0.0437	0.0676	0.2967
N	5	5	5	5	5

[a] [RC:estrus]

Appendix 18

Appendix 1

Individual Absolute Organ Weights: Day 29

5550008

Sex: Female Day(s) Relative to Start Date

0.36x 10E12 vg Group 2	Organ Weight (Absolute)						
	Terminal Body Wt (g)	Brain (g)	Gland Adrenal (g)	Gland Pituitary (g)	Thyroid/ Parathyroid (g)	Heart (g)	Kidney (g)
	-	-	-	-	-	-	-
2501	248 [a]	1.908	0.0589	0.0142	0.0106	0.968	1.740
2502	249 [a]	1.858	0.0687	0.0135	0.0119	0.889	1.631
2507	221 [a]	1.908	0.0611	0.0163	0.0140	0.845	1.442
2508	236 [a]	2.091	0.0681	0.0184	0.0124	0.839	1.816
2509	207 [a]	1.900	0.0595	0.0180	0.0104	0.761	1.543
Mean	232.2	1.9330	0.06326	0.01608	0.01186	0.8604	1.6344
SD	18.1	0.0907	0.00477	0.00220	0.00147	0.0758	0.1496
N	5	5	5	5	5	5	5
%Diff	16.0	5.8366	16.20132	24.45820	4.03509	16.1134	16.7429

[a] [RC:VALUE CONFIRMED]

Appendix 18

Appendix 1

Individual Absolute Organ Weights: Day 29

5550008

Sex: Female Day(s) Relative to Start Date

0.36x 10E12 vg Group 2	Organ Weight (Absolute)				
	Liver (g)	Ovary (g)	Spleen (g)	Thymus (g)	Uterus/ Cervix (g)
	-	-	-	-	-
2501	6.300	0.091	0.503	0.381	0.398
2502	7.166	0.104	0.701	0.386	0.507
2507	6.461	0.090	0.376	0.454	0.376
2508	7.033	0.111	0.448	0.526	0.595
2509	6.348	0.103	0.461	0.334	0.619
Mean	6.6616	0.0998	0.4978	0.4162	0.4990
SD	0.4067	0.0090	0.1225	0.0748	0.1107
N	5	5	5	5	5
%Diff	21.8377	24.4389	17.2398	22.4118	-38.9079

Appendix 18

Appendix 1

Individual Absolute Organ Weights: Day 29

5550008

Sex: Female Day(s) Relative to Start Date

1.1x 10E12 vg Group 3	Terminal Body Wt (g)	Brain (g)	Gland Adrenal (g)	Gland Pituitary (g)	Thyroid/ Parathyroid (g)	Heart (g)	Kidney (g)
	-	-	-	-	-	-	-
3504	206 [a]	1.802	0.0595	0.0145	0.0156	0.791	1.593
3505	193 [a]	1.927	0.0567	0.0163	0.0120	0.728	1.205
3506	208 [a]	1.925	0.0475	0.0150	0.0109	0.820	1.374
3514	204	1.786	0.0611	0.0133	0.0152	0.743	1.434
3615	241	1.900	0.0770	0.0150	0.0107	0.845	1.761
Mean	210.4	1.8680	0.06036	0.01482	0.01288	0.7854	1.4734
SD	18.1	0.0686	0.01069	0.00108	0.00236	0.0497	0.2124
N	5	5	5	5	5	5	5
%Diff	5.1	2.2777	10.87436	14.70588	12.98246	5.9919	5.2429

Organ Weight (Absolute)

[a] [RC:VALUE CONFIRMED]

Appendix 18

Appendix 1

Individual Absolute Organ Weights: Day 29

5550008

Sex: Female Day(s) Relative to Start Date

1.1x 10E12 vg Group 3	Organ Weight (Absolute)				
	Liver (g)	Ovary (g)	Spleen (g)	Thymus (g)	Uterus/ Cervix (g)
	-	-	-	-	-
3504	6.666	0.113	0.483	0.553	0.600
3505	5.448	0.074	0.389	0.304	0.475
3506	6.461	0.082	0.389	0.318	0.637 [a]
3514	5.853	0.103	0.426	0.300	0.485
3615	6.668	0.172 I	0.615	0.518	0.527
Mean	6.2192	0.1088	0.4604	0.3986	0.5448
SD	0.5451	0.0386	0.0946	0.1258	0.0713
N	5	5	5	5	5
%Diff	13.7464	35.6608	8.4315	17.2353	-33.3007

I = Include

[a] [RC:Estrus]

Appendix 18

Appendix 1

Individual Absolute Organ Weights: Day 29

5550008

Sex: Female Day(s) Relative to Start Date

3.3x 10E12 vg Group 4	Organ Weight (Absolute)						
	Terminal Body Wt (g)	Brain (g)	Gland Adrenal (g)	Gland Pituitary (g)	Thyroid/ Parathyroid (g)	Heart (g)	Kidney (g)
	-	-	-	-	-	-	-
4505	194 [a]	1.711	0.0471	0.0152	0.0132	0.788	1.477
4506	239 [a]	1.898	0.0637	0.0159	0.0120	0.901	1.693
4507	187 [a]	1.815	0.0558	0.0152	0.0124	0.730	1.418
4511	214	1.835	0.0717	0.0130	0.0078	0.871	1.509
4512	242	1.895	0.0557	0.0178	0.0117	0.931	1.637
Mean	215.2	1.8308	0.05880	0.01542	0.01142	0.8442	1.5468
SD	25.2	0.0763	0.00930	0.00172	0.00210	0.0832	0.1145
N	5	5	5	5	5	5	5
%Diff	7.5	0.2409	8.00882	19.34985	0.17544	13.9271	10.4857

[a] [RC:VALUE CONFIRMED]

Appendix 18

Appendix 1

Individual Absolute Organ Weights: Day 29

5550008

Sex: Female Day(s) Relative to Start Date

3.3x 10E12 vg Group 4	Organ Weight (Absolute)				
	Liver (g)	Ovary (g)	Spleen (g)	Thymus (g)	Uterus/ Cervix (g)
	-	-	-	-	-
4505	5.907	0.078	0.412	0.245	0.422
4506	7.078	0.125	0.558	0.631	0.415
4507	5.791	0.079	0.388	0.317	0.520
4511	5.934	0.087	0.428	0.433	0.406
4512	7.105	0.103	0.547	0.434	0.396
Mean	6.3630	0.0944	0.4666	0.4120	0.4318
SD	0.6673	0.0198	0.0798	0.1465	0.0503
N	5	5	5	5	5
%Diff	16.3765	17.7057	9.8917	21.1765	-47.1352

Appendix 18
Appendix 1

Individual Absolute Organ Weights: Day 91

5550008
Sex: Male Day(s) Relative to Start Date

0<br>vg<br>Group 1	Organ Weight (Absolute)						
	Terminal Body Wt (g)	Brain (g)	Epididymis (g)	Gland Adrenal (g)	Gland Pituitary (g)	Gland Prostate (g)	Thyroid/ Parathyroid (g)
	-	-	-	-	-	-	-
1005	568	2.194	1.403	0.0443	0.0135	1.289	0.0147
1006	543	2.200	1.352	0.0474	0.0154	1.640	0.0201
1007	739	2.144	1.479	0.0646	0.0219	1.710	0.0280
1011	616 [a]	2.207	1.512	0.0417	0.0180	1.396	0.0282 [b]
1012	600	2.260	1.359	0.0411	0.0164	1.427	0.0204
Mean	613.2	2.2010	1.4210	0.04782	0.01704	1.4924	0.02228
SD	75.8	0.0413	0.0717	0.00970	0.00317	0.1761	0.00578
N	5	5	5	5	5	5	5

[a] [RC:Value Confirmed]
[b] [RC:Thyroid weighed after dropped in formalin for about 5 minutes.]

Appendix 18

Appendix 1

Individual Absolute Organ Weights: Day 91

5550008

Sex: Male Day(s) Relative to Start Date

0	Organ Weight (Absolute)					
vg	Heart	Kidney	Liver	Spleen	Testis	Thymus
Group 1	(g)	(g)	(g)	(g)	(g)	(g)
	-	-	-	-	-	-
1005	1.610	2.745	13.097	0.817	4.182	0.213
1006	1.621	3.106	12.581	0.964	3.615	0.253
1007	2.158	3.805	22.638	0.965	4.168	0.356
1011	1.562	3.470	13.547	1.037	3.906	0.260
1012	1.776	3.248	14.342	0.919	3.493	0.288
Mean	1.7454	3.2748	15.2410	0.9404	3.8728	0.2740
SD	0.2442	0.3966	4.1853	0.0809	0.3141	0.0531
N	5	5	5	5	5	5

Appendix 18

Appendix 1

Individual Absolute Organ Weights: Day 91

5550008

Sex: Male Day(s) Relative to Start Date

0.36x 10E12 vg Group 2	Organ Weight (Absolute)						
	Terminal Body Wt (g)	Brain (g)	Epididymis (g)	Gland Adrenal (g)	Gland Pituitary (g)	Gland Prostate (g)	Thyroid/ Parathyroid (g)
	-	-	-	-	-	-	-
2103	610	2.397	1.487	0.0542	0.0125	1.353	0.0257
2005	656	2.141	1.425	0.0587	0.0174	1.493	0.0238
2006	605	2.234	1.495	0.0476	0.0146	1.452	0.0169
2007	650	2.173	1.411	0.0581	0.0193	1.677	0.0213
2008	575	2.282	0.871	0.0703	0.0157	1.444	0.0154
Mean	619.2	2.2454	1.3378	0.05778	0.01590	1.4838	0.02062
SD	33.7	0.1007	0.2636	0.00828	0.00260	0.1195	0.00440
N	5	5	5	5	5	5	5
%Diff	1.0	2.0173	-5.8550	20.82811	-6.69014	-0.5763	-7.45063

Appendix 18

Appendix 1

Individual Absolute Organ Weights: Day 91

5550008

Sex: Male Day(s) Relative to Start Date

0.36x 10E12 vg Group 2	Organ Weight (Absolute)					
	Heart (g)	Kidney (g)	Liver (g)	Spleen (g)	Testis (g)	Thymus (g)
	-	-	-	-	-	-
2103	1.677	3.119	13.408	1.046	3.890	0.259
2005	1.608	2.818	14.240	0.925	4.188	0.357
2006	1.859	3.295	14.999	0.766	3.883	0.249
2007	1.766	3.366	15.400	1.111	3.938	0.284
2008	1.770	3.296	13.537	0.812	2.979	0.264
Mean	1.7360	3.1788	14.3168	0.9320	3.7756	0.2826
SD	0.0962	0.2214	0.8773	0.1475	0.4625	0.0435
N	5	5	5	5	5	5
%Diff	-0.5386	-2.9315	-6.0639	-0.8932	-2.5098	3.1387

Appendix 18

Appendix 1

Individual Absolute Organ Weights: Day 91

5550008

Sex: Male Day(s) Relative to Start Date

1.1x 10E12 vg Group 3	Organ Weight (Absolute)						
	Terminal Body Wt (g)	Brain (g)	Epididymis (g)	Gland Adrenal (g)	Gland Pituitary (g)	Gland Prostate (g)	Thyroid/ Parathyroid (g)
	-	-	-	-	-	-	-
3006	527	2.229	1.341	0.0564	0.0145	1.012	0.0171
3007	672	2.325	1.392	0.0593	0.0179	1.875	0.0210
3008	580	2.333	1.292	0.0450	0.0145	1.130	0.0167
3014	503	2.117	1.323 I	0.0492	0.0137	1.279	0.0176
3015	610	2.231	1.315	0.0422	0.0180	1.441	0.0223
Mean	578.4	2.2470	1.3326	0.05042	0.01572	1.3474	0.01894
SD	67.2	0.0880	0.0376	0.00730	0.00206	0.3360	0.00254
N	5	5	5	5	5	5	5
%Diff	-5.7	2.0900	-6.2210	5.43706	-7.74648	-9.7159	-14.99102

I = Include

Appendix 18

Appendix 1

Individual Absolute Organ Weights: Day 91

5550008

Sex: Male Day(s) Relative to Start Date

1.1x 10E12 vg Group 3	Organ Weight (Absolute)					
	Heart (g)	Kidney (g)	Liver (g)	Spleen (g)	Testis (g)	Thymus (g)
	-	-	-	-	-	-
3006	1.731	2.891	12.476	0.913	3.652	0.499
3007	2.042	3.286	16.221	0.934	4.099	0.294
3008	1.477	2.789	12.537	0.783	3.458	0.323
3014	1.414	2.706	10.888	0.735	3.494	0.253
3015	1.665	3.362	14.550	0.807	3.945	0.298
Mean	1.6658	3.0068	13.3344	0.8344	3.7296	0.3334
SD	0.2474	0.2981	2.0717	0.0857	0.2820	0.0959
N	5	5	5	5	5	5
%Diff	-4.5606	-8.1837	-12.5097	-11.2718	-3.6976	21.6788

Appendix 18

Appendix 1

Individual Absolute Organ Weights: Day 91

5550008

Sex: Male Day(s) Relative to Start Date

3.3x 10E12 vg Group 4	Organ Weight (Absolute)						
	Terminal Body Wt (g)	Brain (g)	Epididymis (g)	Gland Adrenal (g)	Gland Pituitary (g)	Gland Prostate (g)	Thyroid/ Parathyroid (g)
	-	-	-	-	-	-	-
4009	558	2.262	0.869	0.0578	0.0136	1.847	0.0170
4010	430	2.147	1.236	0.0444	0.0150	1.218	0.0139
4011	521	2.164	1.413	0.0427	0.0155	1.700	0.0248
4014	494	2.294	1.344	0.0536	0.0106	1.396	0.0167
4015	549	2.201	1.023 I	0.0616	0.0153	1.631	0.0186
Mean	510.4	2.2136	1.1770	0.05202	0.01400	1.5584	0.01820
SD	51.5	0.0630	0.2267	0.00826	0.00204	0.2504	0.00406
N	5	5	5	5	5	5	5
%Diff	-16.8	0.5725	-17.1710	8.78294	-17.84038	4.4224	-18.31239

I = Include

Appendix 18
Appendix 1

Individual Absolute Organ Weights: Day 91

5550008
Sex: Male Day(s) Relative to Start Date

3.3x 10E12 vg Group 4	Organ Weight (Absolute)					
	Heart (g)	Kidney (g)	Liver (g)	Spleen (g)	Testis (g)	Thymus (g)
	-	-	-	-	-	-
4009	1.508	3.513	13.863	0.813	2.313	0.209
4010	1.364	2.386	9.712	0.677	3.646	0.207
4011	1.619	2.898	12.391	0.713	3.683	0.411
4014	1.390	2.741	11.132	0.876	3.950	0.276
4015	1.691	3.016	12.465	1.156	2.441 I	0.262
Mean	1.5144	2.9108	11.9126	0.8470	3.2066	0.2730
SD	0.1417	0.4117	1.5645	0.1900	0.7677	0.0831
N	5	5	5	5	5	5
%Diff	-13.2348	-11.1152	-21.8385	-9.9319	-17.2020	-0.3650

I = Include

Appendix 18

Appendix 1

Individual Absolute Organ Weights: Day 91

5550008

Sex: Female Day(s) Relative to Start Date

0	Organ Weight (Absolute)						
vg<br><br>Group 1	Terminal Body Wt (g)	Brain (g)	Gland Adrenal (g)	Gland Pituitary (g)	Thyroid/ Parathyroid (g)	Heart (g)	Kidney (g)
	-	-	-	-	-	-	-
1503	275	2.061	0.0754	0.0159	0.0124	0.919	1.771
1504	283 [a]	1.967	0.0620	0.0193 I	0.0116	0.870	1.599
1510	256 [a]	1.827	0.0755	0.0206	0.0117	0.837	1.570
1511	296 [a]	2.076	0.0665	0.0204	0.0174	1.092	1.885
1512	235	1.961	0.0560	0.0139	0.0120	0.869	1.400
Mean	269.0	1.9784	0.06708	0.01802	0.01302	0.9174	1.6450
SD	23.9	0.0996	0.00850	0.00298	0.00247	0.1019	0.1879
N	5	5	5	5	5	5	5

I = Include

[a] [RC:Value Confirmed]

Appendix 18

Appendix 1

Individual Absolute Organ Weights: Day 91

5550008

Sex: Female Day(s) Relative to Start Date

0	Organ Weight (Absolute)				
vg	Liver	Ovary	Spleen	Thymus	Uterus/ Cervix
Group 1	(g)	(g)	(g)	(g)	(g)
	-	-	-	-	-
1503	6.429	0.082	0.595	0.188	0.491
1504	6.823	0.080	0.540	0.198	0.571
1510	6.005	0.082	0.569	0.248	0.787
1511	7.336	0.093	0.657	0.285	0.523
1512	5.514	0.071	0.458	0.248	0.525
Mean	6.4214	0.0816	0.5638	0.2334	0.5794
SD	0.7062	0.0078	0.0732	0.0400	0.1195
N	5	5	5	5	5

Appendix 18

Appendix 1

Individual Absolute Organ Weights: Day 91

5550008

Sex: Female Day(s) Relative to Start Date

0.36x 10E12 vg Group 2	Organ Weight (Absolute)						
	Terminal Body Wt (g)	Brain (g)	Gland Adrenal (g)	Gland Pituitary (g)	Thyroid/ Parathyroid (g)	Heart (g)	Kidney (g)
	-	-	-	-	-	-	-
2503	249 [a]	1.954	0.0569	0.0182	0.0092	0.861	1.750
2504	331 [a]	2.048	0.0626	0.0157	0.0195	0.991	2.120
2613	285 [a]	OA [b]	0.0577	0.0161	0.0150	0.946	1.602
2614	309	1.810	0.0570	0.0204	0.0168	0.955	1.636
2615	269	1.988	0.0749	0.0225	0.0122	0.953	1.749
Mean	288.6	1.9500	0.06182	0.01858	0.01454	0.9412	1.7714
SD	32.3	0.1011	0.00768	0.00288	0.00400	0.0481	0.2059
N	5	4	5	5	5	5	5
%Diff	7.3	-1.4355	-7.84138	3.10766	11.67435	2.5943	7.6839

[a] [RC:Value Confirmed]
[b] [RC:Weight not taken before frozen samples]

Appendix 18

Appendix 1

Individual Absolute Organ Weights: Day 91

5550008

Sex: Female Day(s) Relative to Start Date

0.36x 10E12 vg Group 2	Organ Weight (Absolute)				
	Liver (g)	Ovary (g)	Spleen (g)	Thymus (g)	Uterus/ Cervix (g)
	-	-	-	-	-
2503	6.784	0.094	0.521	0.160	0.571
2504	8.275	0.072	0.456	0.175	0.586
2613	6.254	0.084	0.345	0.196	0.547
2614	6.250	0.094	0.510	0.178	0.468
2615	7.250	0.092	0.503	0.181	1.095
Mean	6.9626	0.0872	0.4670	0.1780	0.6534
SD	0.8436	0.0094	0.0726	0.0129	0.2510
N	5	5	5	5	5
%Diff	8.4281	6.8627	-17.1692	-23.7361	12.7718

Appendix 18

Appendix 1

Individual Absolute Organ Weights: Day 91

5550008

Sex: Female Day(s) Relative to Start Date

1.1x 10E12 vg Group 3	Organ Weight (Absolute)						
	Terminal Body Wt (g)	Brain (g)	Gland Adrenal (g)	Gland Pituitary (g)	Thyroid/ Parathyroid (g)	Heart (g)	Kidney (g)
	-	-	-	-	-	-	-
3501	262	1.948	0.0762 I	0.0245 I	0.0156	0.952	1.678
3502	367	2.104	0.0546	0.0185	0.0118	1.277	1.928
3503	241	2.097	0.0586	0.0207	0.0143	0.864	1.379
3509	264	2.034	0.0488	0.0203	0.0123	0.851	1.449
3510	281	1.918	0.0480	0.0152	0.0140	0.997	1.869
Mean	283.0	2.0202	0.05724	0.01984	0.01360	0.9882	1.6606
SD	49.1	0.0848	0.01146	0.00339	0.00155	0.1725	0.2446
N	5	5	5	5	5	5	5
%Diff	5.2	2.1128	-14.66905	10.09989	4.45469	7.7175	0.9483

I = Include

Appendix 18

Appendix 1

Individual Absolute Organ Weights: Day 91

5550008

Sex: Female Day(s) Relative to Start Date

1.1x 10E12 vg Group 3	Organ Weight (Absolute)				
	Liver (g)	Ovary (g)	Spleen (g)	Thymus (g)	Uterus/ Cervix (g)
	-	-	-	-	-
3501	6.545	0.085	0.491	0.167	2.667 [a]
3502	8.197	0.125	0.570	0.241	0.593
3503	5.959	0.056	0.403	0.193	0.722
3509	6.432	0.070	0.477	0.226	1.488 [a]
3510	7.461	0.086	0.570	0.297	0.445
Mean	6.9188	0.0844	0.5022	0.2248	1.1830
SD	0.8980	0.0258	0.0703	0.0496	0.9220
N	5	5	5	5	5
%Diff	7.7460	3.4314	-10.9259	-3.6847	104.1767

[a] [RC:Estrus]

Appendix 18

Appendix 1

Individual Absolute Organ Weights: Day 91

5550008

Sex: Female Day(s) Relative to Start Date

3.3x 10E12 vg Group 4	Organ Weight (Absolute)						
	Terminal Body Wt (g)	Brain (g)	Gland Adrenal (g)	Gland Pituitary (g)	Thyroid/ Parathyroid (g)	Heart (g)	Kidney (g)
	-	-	-	-	-	-	-
4503	279	2.019	0.0696	0.0243 I	0.0073 I	1.066	1.500
4504	237	1.878	0.0529	0.0153	0.0095	0.813	1.829
4513	274	2.017	0.0622	0.0230	0.0126	0.987	1.768
4514	302	1.900	0.0481	0.0187	0.0135	0.976	1.696
4515	242	1.901	0.0573	0.0286 I	0.0146	0.936	1.618
Mean	266.8	1.9430	0.05802	0.02198	0.01150	0.9556	1.6822
SD	27.1	0.0691	0.00832	0.00514	0.00302	0.0926	0.1289
N	5	5	5	5	5	5	5
%Diff	-0.8	-1.7893	-13.50626	21.97558	-11.67435	4.1639	2.2614

I = Include

Appendix 18

Appendix 1

Individual Absolute Organ Weights: Day 91

5550008

Sex: Female Day(s) Relative to Start Date

3.3x 10E12 vg Group 4	Organ Weight (Absolute)				
	Liver (g)	Ovary (g)	Spleen (g)	Thymus (g)	Uterus/ Cervix (g)
	-	-	-	-	-
4503	6.799	0.064	0.488	0.272	0.665
4504	5.900	0.083	0.486	0.229	1.246 [a]
4513	6.400 I	0.073	0.618	0.242	0.780
4514	7.298	0.065	0.521	0.163	1.092
4515	7.294	0.050	0.410	0.181	0.634
Mean	6.7382	0.0670	0.5046	0.2174	0.8834
SD	0.6006	0.0122	0.0753	0.0447	0.2718
N	5	5	5	5	5
%Diff	4.9335	-17.8922	-10.5002	-6.8552	52.4681

I = Include

[a] [RC:Estrus]

Individual Organ Weights Relative to Body Weight Explanation Page

Abbreviation	Description
BW	Body Weight

Note: This is a comprehensive list of abbreviations. All of the abbreviations listed may not be applicable to this report.

Dosing Information

Dosing information is abbreviated on various data outputs; the following represents the dosing information for this study:

Group No.	Test Material	Dose Level (vg)
1	Reference Item	0
2	AAV9/AP4M1	3.6E11
3	AAV9/AP4M1	1.1E12
4	AAV9/AP4M1	3.3E12

Appendix 18

Appendix 2

Individual Organ Weights Relative to Body Weight: Day 8

5550008

Sex: Male Day(s) Relative to Start Date

0	Organ Weight (BW)						
vg<br>Group 1	Brain<br>(%)	Epididymis<br>(%)	Gland Adrenal<br>(%)	Gland Pituitary<br>(%)	Gland Prostate<br>(%)	Thyroid/ Parathyroid<br>(%)	Heart<br>(%)
	-	-	-	-	-	-	-
1001	0.8058	0.1603	0.0163	0.0050	0.1657	0.0048	0.3926
1002	0.8344	0.1866	0.0196	0.0042	0.2192	0.0069	0.3884
1014	0.7053	0.2140	0.0162	0.0042	0.1818	0.0044	0.3330
1015	0.8020	0.2146	0.0165	0.0033	0.1619	0.0033	0.3729
Mean	0.78687	0.19388	0.01716	0.00418	0.18217	0.00486	0.37170
SD	0.05627	0.02590	0.00166	0.00070	0.02615	0.00152	0.02718
N	4	4	4	4	4	4	4

Appendix 18

Appendix 2

Individual Organ Weights Relative to Body Weight: Day 8

5550008

Sex: Male Day(s) Relative to Start Date

0	Organ Weight (BW)				
vg	Kidney	Liver	Spleen	Testis	Thymus
Group 1	(%)	(%)	(%)	(%)	(%)
	-	-	-	-	-
1001	0.8963	3.4715	0.2876	1.1719	0.2405
1002	0.8531	3.4826	0.2518	1.3027	0.2714
1014	0.8121	3.1462	0.2833	1.1121	0.1936
1015	0.9320	3.2045	0.2579	1.1692	0.2032
Mean	0.87338	3.32619	0.27015	1.18898	0.22718
SD	0.05203	0.17586	0.01794	0.08066	0.03577
N	4	4	4	4	4

Appendix 18

Appendix 2

Individual Organ Weights Relative to Body Weight: Day 8

5550008

Sex: Male Day(s) Relative to Start Date

0.36x 10E12 vg Group 2	Organ Weight (BW)						
	Brain (%)	Epididymis (%)	Gland Adrenal (%)	Gland Pituitary (%)	Gland Prostate (%)	Thyroid/ Parathyroid (%)	Heart (%)
	-	-	-	-	-	-	-
2004	0.9883	0.1809	0.0176	0.0044	0.2176	0.0076	0.3872
2009	0.6911	0.1673	0.0159	0.0044	0.1851	0.0040	0.4423
2010	0.7813	0.2000	0.0195	0.0047	0.1404	0.0040	0.3809
2014	0.7367	0.2398	0.0177	0.0042	0.1949	0.0035	0.3980
2015	0.8584	0.2516	0.0190	0.0043	0.2489	0.0037	0.4063
Mean	0.81115	0.20791	0.01792	0.00442	0.19737	0.00455	0.40297
SD	0.11668	0.03665	0.00139	0.00020	0.04018	0.00169	0.02409
N	5	5	5	5	5	5	5
%Diff	3.08517	7.23415	4.40669	5.72845	8.34549	-6.39195	8.41371

Appendix 18

Appendix 2

Individual Organ Weights Relative to Body Weight: Day 8

5550008

Sex: Male Day(s) Relative to Start Date

0.36x 10E12 vg Group 2	Organ Weight (BW)				
	Kidney (%)	Liver (%)	Spleen (%)	Testis (%)	Thymus (%)
	-	-	-	-	-
2004	0.8314	3.3984	0.2564	1.3122	0.2681
2009	0.9288	3.2616	0.2278	0.8769	0.2537
2010	0.9063	3.6239	0.2566	1.1768	0.1724
2014	0.8082	3.1738	0.2594	1.2027	0.2051
2015	0.8376	3.1747	0.1910	1.2688	0.2434
Mean	0.86244	3.32647	0.23822	1.16749	0.22855
SD	0.05209	0.18987	0.02941	0.17103	0.03911
N	5	5	5	5	5
%Diff	-1.25193	0.00859	-11.82195	-1.80761	0.60396

Appendix 18

Appendix 2

Individual Organ Weights Relative to Body Weight: Day 8

5550008

Sex: Male Day(s) Relative to Start Date

1.1x 10E12 vg Group 3	Organ Weight (BW)						
	Brain (%)	Epididymis (%)	Gland Adrenal (%)	Gland Pituitary (%)	Gland Prostate (%)	Thyroid/ Parathyroid (%)	Heart (%)
	-	-	-	-	-	-	-
3001	0.8588	0.2351	0.0150	0.0032	0.2360	0.0048	0.3962
3002	0.7918	0.1934	0.0143	0.0045	0.2135	0.0038	0.3926
3003	0.9201	0.1696	0.0182	0.0034	0.1907	0.0040	0.3961
3012	0.7980	0.2094	0.0164	0.0036	0.2155	0.0033	0.4339
3013	0.7756	0.2194	0.0205	0.0043	0.1977	0.0036	0.4465
Mean	0.82884	0.20538	0.01688	0.00378	0.21068	0.00388	0.41306
SD	0.05997	0.02508	0.00252	0.00059	0.01762	0.00059	0.02521
N	5	5	5	5	5	5	5
%Diff	5.33379	5.92932	-1.67899	-9.46368	15.65491	-20.07331	11.12841

Appendix 18

Appendix 2

Individual Organ Weights Relative to Body Weight: Day 8

5550008

Sex: Male Day(s) Relative to Start Date

1.1x 10E12 vg Group 3	Organ Weight (BW)				
	Kidney (%)	Liver (%)	Spleen (%)	Testis (%)	Thymus (%)
	-	-	-	-	-
3001	0.8303	3.4000	0.2763	1.2607	0.2460
3002	0.8885	3.5902	0.2496	1.1746	0.2418
3003	0.8623	3.3716	0.2196	1.3279	0.3250
3012	0.8718	3.1608	0.2392	1.0482	0.1959
3013	0.8147	3.1911	0.2143	1.1376	0.1977
Mean	0.85354	3.34273	0.23981	1.18979	0.24127
SD	0.03033	0.17423	0.02492	0.10854	0.05242
N	5	5	5	5	5
%Diff	-2.27192	0.49730	-11.23397	0.06797	6.20323

Individual Organ Weights Relative to Body Weight: Day 8

5550008

Sex: Male Day(s) Relative to Start Date

3.3x 10E12 vg Group 4	Organ Weight (BW)						
	Brain (%)	Epididymis (%)	Gland Adrenal (%)	Gland Pituitary (%)	Gland Prostate (%)	Thyroid/ Parathyroid (%)	Heart (%)
	-	-	-	-	-	-	-
4001	0.8455	0.1977	0.0203	0.0038	0.2441	0.0054	0.4005
4002	0.8990	0.1762	0.0146	0.0040	0.1097	0.0059	0.3874
4006	0.9681	0.2531	0.0183	0.0043	0.2710	0.0060	0.4271
4007	1.0227	0.2359	0.0208	0.0041	0.1895	0.0063	0.4033
4008	0.6961	0.1726	0.0137	0.0049	0.1880	0.0046	0.3795
Mean	0.88630	0.20710	0.01753	0.00422	0.20048	0.00566	0.39955
SD	0.12577	0.03600	0.00327	0.00042	0.06204	0.00065	0.01817
N	5	5	5	5	5	5	5
%Diff	12.63526	6.81807	2.13459	0.93758	10.05273	16.51440	7.49392

Appendix 18

Appendix 2

Individual Organ Weights Relative to Body Weight: Day 8

5550008

Sex: Male Day(s) Relative to Start Date

3.3x 10E12 vg Group 4	Organ Weight (BW)				
	Kidney (%)	Liver (%)	Spleen (%)	Testis (%)	Thymus (%)
	-	-	-	-	-
4001	0.9141	3.3150	0.3056	1.0939	0.2230
4002	0.9447	3.5451	0.3150	1.2044	0.2636
4006	0.8850	3.2594	0.1976	1.3821	0.1565
4007	0.7680	2.9442	0.2133	1.4298	0.2536
4008	0.8286	3.1247	0.2243	1.1274	0.2815
Mean	0.86806	3.23770	0.25117	1.24753	0.23564
SD	0.07045	0.22354	0.05495	0.15103	0.04905
N	5	5	5	5	5
%Diff	-0.60896	-2.66028	-7.02713	4.92395	3.72145

Appendix 18

Appendix 2

Individual Organ Weights Relative to Body Weight: Day 8

5550008

Sex: Female Day(s) Relative to Start Date

0	Organ Weight (BW)						
vg<br><br>Group 1	Brain<br><br>(%)	Gland Adrenal<br>(%)	Gland Pituitary<br>(%)	Thyroid/ Parathyroid<br>(%)	Heart<br><br>(%)	Kidney<br><br>(%)	Liver<br><br>(%)
	-	-	-	-	-	-	-
1501	1.0333	0.0313	0.0088	0.0054	0.3760	0.8912	3.4977
1502	0.9855	0.0276	0.0072	0.0048	0.4096	0.9139	3.2367
1505	1.1142	0.0327	0.0084	0.0043	0.4107	0.8473	3.3757
1506	1.1826	0.0335	0.0080	0.0048	0.4187	0.9535	3.4581
1507	1.0462	0.0405	0.0076	0.0066	0.4183	0.8728	4.0189
Mean	1.07236	0.03312	0.00800	0.00517	0.40667	0.89575	3.51743
SD	0.07689	0.00472	0.00062	0.00087	0.01764	0.04050	0.29761
N	5	5	5	5	5	5	5

Appendix 18

Appendix 2

Individual Organ Weights Relative to Body Weight: Day 8

5550008

Sex: Female Day(s) Relative to Start Date

0	Organ Weight (BW)			
vg<br>Group 1	Ovary<br>(%)	Spleen<br>(%)	Thymus<br>(%)	Uterus/ Cervix<br>(%)
	-	-	-	-
1501	0.0398	0.2146	0.2082	0.2491
1502	0.0367	0.2663	0.2470	0.3681
1505	0.0456	0.2219	0.2491	0.2663
1506	0.0471	0.2568	0.3206	0.2716
1507	0.0456	0.2302	0.2438	0.2592
Mean	0.04295	0.23795	0.25374	0.28285
SD	0.00446	0.02246	0.04098	0.04838
N	5	5	5	5

Appendix 18

Appendix 2

Individual Organ Weights Relative to Body Weight: Day 8

5550008

Sex: Female Day(s) Relative to Start Date

0.36x 10E12 vg Group 2	Organ Weight (BW)						
	Brain (%)	Gland Adrenal (%)	Gland Pituitary (%)	Thyroid/ Parathyroid (%)	Heart (%)	Kidney (%)	Liver (%)
	-	-	-	-	-	-	-
2505	1.0806	0.0285	0.0081	0.0041	0.4200	0.8548	3.5310
2506	1.0347	0.0308	0.0074	0.0063	0.3659	0.7659	2.9126
2510	1.0026	0.0297	0.0044	0.0040	0.3906	0.8115	3.4471
2511	0.8820	0.0280	0.0072	0.0038	0.3824	0.8805	2.9517
2512	0.9273	0.0326	0.0082	0.0044	0.4166	0.8241	3.6385
Mean	0.98544	0.02990	0.00705	0.00455	0.39509	0.82736	3.29617
SD	0.08044	0.00185	0.00155	0.00103	0.02300	0.04365	0.33945
N	5	5	5	5	5	5	5
%Diff	-8.10535	-9.74384	-11.83780	-12.04405	-2.84774	-7.63545	-6.29024

Appendix 18

Appendix 2

Individual Organ Weights Relative to Body Weight: Day 8

5550008

Sex: Female Day(s) Relative to Start Date

0.36x 10E12 vg Group 2	Organ Weight (BW)			
	Ovary (%)	Spleen (%)	Thymus (%)	Uterus/ Cervix (%)
	-	-	-	-
2505	0.0355	0.2342	0.2910	0.5465
2506	0.0305	0.2072	0.2641	0.2012
2510	0.0408	0.2545	0.2262	0.2037
2511	0.0410	0.2717	0.2312	0.5302
2512	0.0439	0.2337	0.2888	0.1674
Mean	0.03834	0.24025	0.26024	0.32979
SD	0.00530	0.02431	0.03072	0.19101
N	5	5	5	5
%Diff	-10.73315	0.96631	2.56065	16.59435

Appendix 18

Appendix 2

Individual Organ Weights Relative to Body Weight: Day 8

5550008

Sex: Female Day(s) Relative to Start Date

1.1x 10E12 vg Group 3	Organ Weight (BW)						
	Brain (%)	Gland Adrenal (%)	Gland Pituitary (%)	Thyroid/ Parathyroid (%)	Heart (%)	Kidney (%)	Liver (%)
	-	-	-	-	-	-	-
3507	0.9813	0.0261	0.0056	0.0053	0.3636	0.8717	2.9888
3508	0.9931	0.0285	0.0064	0.0055	0.3726	0.9480	3.4606
3511	1.0166	0.0392	0.0089	0.0036	0.4283	0.8519	3.1214
3512	1.0705	0.0394	0.0069	0.0053	0.4222	0.8347	2.7284
3513	1.0218	0.0303	0.0086	0.0041	0.4224	0.8868	3.0000
Mean	1.01666	0.03268	0.00729	0.00478	0.40182	0.87859	3.05983
SD	0.03437	0.00621	0.00142	0.00084	0.03104	0.04352	0.26592
N	5	5	5	5	5	5	5
%Diff	-5.19440	-1.33253	-8.84676	-7.62046	-1.19225	-1.91527	-13.00953

Appendix 18

Appendix 2

Individual Organ Weights Relative to Body Weight: Day 8

5550008

Sex: Female Day(s) Relative to Start Date

1.1x 10E12 vg Group 3	Organ Weight (BW)			
	Ovary (%)	Spleen (%)	Thymus (%)	Uterus/ Cervix (%)
	-	-	-	-
3507	0.0449	0.2353	0.1947	0.3866
3508	0.0451	0.2726	0.2023	0.2040
3511	0.0503	0.1963	0.1941	0.3722
3512	0.0392	0.2290	0.1767	0.3813
3513	0.0443	0.2552	0.2328	0.2138
Mean	0.04476	0.23765	0.20010	0.31157
SD	0.00393	0.02881	0.02052	0.09394
N	5	5	5	5
%Diff	4.21607	-0.12257	-21.13948	10.15483

Appendix 18
Appendix 2

Individual Organ Weights Relative to Body Weight: Day 8

5550008
Sex: Female Day(s) Relative to Start Date

3.3x 10E12 vg Group 4	Organ Weight (BW)						
	Brain (%)	Gland Adrenal (%)	Gland Pituitary (%)	Thyroid/ Parathyroid (%)	Heart (%)	Kidney (%)	Liver (%)
	-	-	-	-	-	-	-
4601	1.0587	0.0322	0.0077	0.0053	0.3972	0.7782	3.4358
4502	1.1108	0.0315	0.0064	0.0043	0.3701	0.8363	3.2172
4508	1.0975	0.0265	0.0068	0.0047	0.3963	0.8381	3.1719
4509	1.0520	0.0294	0.0083	0.0032	0.4307	0.8492	3.3000
4510	0.8775	0.0279	0.0081	0.0044	0.4156	0.8738	3.2125
Mean	1.03929	0.02950	0.00745	0.00438	0.40197	0.83511	3.26747
SD	0.09383	0.00241	0.00079	0.00073	0.02284	0.03514	0.10496
N	5	5	5	5	5	5	5
%Diff	-3.08419	-10.92666	-6.87209	-15.39124	-1.15543	-6.76964	-7.10644

Appendix 18

Appendix 2

Individual Organ Weights Relative to Body Weight: Day 8

5550008

Sex: Female Day(s) Relative to Start Date

3.3x 10E12 vg Group 4	Organ Weight (BW)			
	Ovary (%)	Spleen (%)	Thymus (%)	Uterus/ Cervix (%)
	-	-	-	-
4601	0.0408	0.2089	0.2140	0.1899
4502	0.0363	0.1987	0.1395	0.5465
4508	0.0500	0.2025	0.2838	0.2538
4509	0.0374	0.2302	0.2335	0.5391
4510	0.0338	0.2225	0.2031	0.4569
Mean	0.03965	0.21257	0.21477	0.39723
SD	0.00631	0.01337	0.05223	0.16547
N	5	5	5	5
%Diff	-7.66813	-10.66611	-15.35944	40.43978

Appendix 18

Appendix 2

Individual Organ Weights Relative to Body Weight: Day 29

5550008

Sex: Male Day(s) Relative to Start Date

0	Organ Weight (BW)						
vg<br>Group 1	Brain<br>(%)	Epididymis<br>(%)	Gland Adrenal<br>(%)	Gland Pituitary<br>(%)	Gland Prostate<br>(%)	Thyroid/ Parathyroid<br>(%)	Heart<br>(%)
	-	-	-	-	-	-	-
1003	0.5636	0.2696	0.0156	0.0038	0.2739	0.0045	0.3862
1004	0.5997	0.2264	0.0137	0.0015	0.1690	0.0044	0.3368
1008	0.5312	0.2753	0.0152	0.0032	0.2682	0.0027	0.3222
1009	0.5130	0.2508	0.0174	0.0038	0.2729	0.0050	0.2935
1010	0.5481	0.2446	0.0160	0.0037	0.2589	0.0043	0.3287
Mean	0.55114	0.25335	0.01555	0.00319	0.24858	0.00419	0.33348
SD	0.03306	0.01974	0.00134	0.00096	0.04489	0.00086	0.03371
N	5	5	5	5	5	5	5

Appendix 18

Appendix 2

Individual Organ Weights Relative to Body Weight: Day 29

5550008

Sex: Male Day(s) Relative to Start Date

0	Organ Weight (BW)				
vg	Kidney	Liver	Spleen	Testis	Thymus
Group 1	(%)	(%)	(%)	(%)	(%)
	-	-	-	-	-
1003	0.7424	2.6968	0.2229	1.0911	0.1421
1004	0.7316	2.9333	0.1591	0.8420	0.1614
1008	0.6822	2.5452	0.2238	0.8901	0.2066
1009	0.7023	2.4781	0.2060	0.7523	0.1193
1010	0.6743	2.6247	0.2943	0.9626	0.1357
Mean	0.70657	2.65564	0.22123	0.90764	0.15302
SD	0.02985	0.17570	0.04856	0.12788	0.03353
N	5	5	5	5	5

Appendix 18

Appendix 2

Individual Organ Weights Relative to Body Weight: Day 29

5550008

Sex: Male Day(s) Relative to Start Date

0.36x 10E12 vg Group 2	Organ Weight (BW)						
	Brain (%)	Epididymis (%)	Gland Adrenal (%)	Gland Pituitary (%)	Gland Prostate (%)	Thyroid/ Parathyroid (%)	Heart (%)
	-	-	-	-	-	-	-
2001	0.5808	0.2353	0.0138	0.0038	0.2426	0.0032	0.3695
2002	0.5938	0.2720	0.0167	0.0029	0.2628	0.0037	0.3611
2011	0.5978	0.2983	0.0171	0.0038	0.2792	0.0062	0.4152
2012	0.5303	0.2344	0.0130	0.0018	0.2738	0.0054	0.3254
2013	0.6063	0.2727	0.0167	0.0036	0.2128	0.0026	0.3920
Mean	0.58178	0.26255	0.01543	0.00320	0.25424	0.00423	0.37266
SD	0.03019	0.02742	0.00192	0.00086	0.02707	0.00151	0.03374
N	5	5	5	5	5	5	5
%Diff	5.55838	3.63104	-0.75524	0.30649	2.27533	0.93917	11.74638

Appendix 18

Appendix 2

Individual Organ Weights Relative to Body Weight: Day 29

5550008

Sex: Male Day(s) Relative to Start Date

0.36x 10E12 vg Group 2	Organ Weight (BW)				
	Kidney (%)	Liver (%)	Spleen (%)	Testis (%)	Thymus (%)
	-	-	-	-	-
2001	0.7426	2.8224	0.1887	1.0053	0.1103
2002	0.7389	3.3571	0.2340	0.9027	0.2057
2011	0.7298	2.7652	0.2278	0.9972	0.1784
2012	0.6537	2.8031	0.1730	0.7825	0.1602
2013	0.7435	2.9301	0.2017	0.9145	0.1037
Mean	0.72169	2.93556	0.20503	0.92044	0.15164
SD	0.03838	0.24346	0.02579	0.09008	0.04394
N	5	5	5	5	5
%Diff	2.14015	10.54059	-7.32001	1.40934	-0.90040

Appendix 18

Appendix 2

Individual Organ Weights Relative to Body Weight: Day 29

5550008

Sex: Male Day(s) Relative to Start Date

1.1x 10E12 vg Group 3	Organ Weight (BW)						
	Brain (%)	Epididymis (%)	Gland Adrenal (%)	Gland Pituitary (%)	Gland Prostate (%)	Thyroid/ Parathyroid (%)	Heart (%)
	-	-	-	-	-	-	-
3004	0.5881	0.2543	0.0154	0.0037	0.1801	0.0033	0.3718
3005	0.5111	0.2674	0.0138	0.0034	0.2897	0.0039	0.3639
3009	0.5556	0.2763	0.0167	0.0034	0.1882	0.0033	0.3492
3010	0.5654	0.2855	0.0132	0.0036	0.3027	0.0047	0.3410
3011	0.6199	0.2277	0.0158	0.0035	0.2533	0.0032	0.3514
Mean	0.56805	0.26225	0.01500	0.00354	0.24278	0.00367	0.35547
SD	0.04030	0.02248	0.00143	0.00014	0.05658	0.00066	0.01228
N	5	5	5	5	5	5	5
%Diff	3.06875	3.51312	-3.53103	10.95428	-2.33340	-12.25349	6.59297

Appendix 18

Appendix 2

Individual Organ Weights Relative to Body Weight: Day 29

5550008

Sex: Male Day(s) Relative to Start Date

1.1x 10E12 vg Group 3	Organ Weight (BW)				
	Kidney (%)	Liver (%)	Spleen (%)	Testis (%)	Thymus (%)
	-	-	-	-	-
3004	0.6549	2.6341	0.1961	0.9620	0.1297
3005	0.6655	2.7761	0.1767	0.9515	0.1294
3009	0.6919	2.9022	0.1970	1.0027	0.1422
3010	0.6217	2.7737	0.1791	0.9635	0.1389
3011	0.7526	3.1586	0.1960	0.6913	0.1688
Mean	0.67734	2.84894	0.18898	0.91420	0.14181
SD	0.04905	0.19736	0.01018	0.12613	0.01612
N	5	5	5	5	5
%Diff	-4.13655	7.27882	-14.57865	0.72187	-7.32403

Appendix 18
Appendix 2

Individual Organ Weights Relative to Body Weight: Day 29

5550008
Sex: Male Day(s) Relative to Start Date

3.3x 10E12 vg Group 4	Organ Weight (BW)						
	Brain (%)	Epididymis (%)	Gland Adrenal (%)	Gland Pituitary (%)	Gland Prostate (%)	Thyroid/ Parathyroid (%)	Heart (%)
	-	-	-	-	-	-	-
4003	0.5616	0.2350	0.0127	0.0038	0.1964	0.0032	0.3440
4004	0.4347	0.1977	0.0140	0.0034	0.2398	0.0052	0.3101
4005	0.5819	0.2482	0.0151	0.0040	0.1852	0.0056	0.3563
4012	0.5904	0.2752	0.0145	0.0033	0.2231	0.0030	0.3603
4013	0.4590	0.2429	0.0183	0.0040	0.2192	0.0038	0.3316
Mean	0.52553	0.23982	0.01494	0.00369	0.21275	0.00418	0.34045
SD	0.07310	0.02796	0.00206	0.00032	0.02184	0.00117	0.02037
N	5	5	5	5	5	5	5
%Diff	-4.64681	-5.34103	-3.91024	15.57443	-14.41268	-0.11841	2.09020

Appendix 18

Appendix 2

Individual Organ Weights Relative to Body Weight: Day 29

5550008

Sex: Male Day(s) Relative to Start Date

3.3x 10E12 vg Group 4	Organ Weight (BW)				
	Kidney (%)	Liver (%)	Spleen (%)	Testis (%)	Thymus (%)
	-	-	-	-	-
4003	0.7681	3.0188	0.1980	0.9132	0.1045
4004	0.6374	3.3236	0.2485	0.7602	0.1984
4005	0.7798	2.8816	0.2244	1.0000	0.1497
4012	0.7882	3.0727	0.2132	0.9603	0.1451
4013	0.7335	2.9256	0.1588	0.8402	0.1103
Mean	0.74139	3.04447	0.20858	0.89476	0.14157
SD	0.06176	0.17322	0.03337	0.09592	0.03761
N	5	5	5	5	5
%Diff	4.92869	14.64148	-5.71768	-1.41991	-7.47793

Appendix 18

Appendix 2

Individual Organ Weights Relative to Body Weight: Day 29

5550008

Sex: Female Day(s) Relative to Start Date

0	Organ Weight (BW)						
vg<br><br>Group 1	Brain<br><br>(%)	Gland Adrenal<br>(%)	Gland Pituitary<br>(%)	Thyroid/ Parathyroid<br>(%)	Heart<br><br>(%)	Kidney<br><br>(%)	Liver<br><br>(%)
	-	-	-	-	-	-	-
1508	0.9220	0.0279	0.0038	0.0063	0.3791	0.6758	2.6604
1509	0.9216	0.0280	0.0064	0.0058	0.3833	0.7221	3.0284
1513	0.8509	0.0255	0.0055	0.0062	0.3741	0.7290	2.4098
1514	0.9010	0.0285	0.0080	0.0055	0.3538	0.6452	2.7938
1515	0.9812	0.0262	0.0085	0.0047	0.3602	0.7232	2.7917
Mean	0.91532	0.02721	0.00645	0.00568	0.37012	0.69907	2.73684
SD	0.04686	0.00129	0.00189	0.00063	0.01260	0.03690	0.22587
N	5	5	5	5	5	5	5

Appendix 18

Appendix 2

Individual Organ Weights Relative to Body Weight: Day 29

5550008

Sex: Female Day(s) Relative to Start Date

0<br>vg<br>Group 1	Organ Weight (BW)<br>Ovary<br>(%)	 Spleen<br>(%)	 Thymus<br>(%)	 Uterus/<br>Cervix<br>(%)
	-	-	-	-
1508	0.0434	0.2104	0.1632	0.4604
1509	0.0324	0.2230	0.1613	0.4015
1513	0.0326	0.2152	0.2031	0.5518
1514	0.0486	0.1829	0.1590	0.1895
1515	0.0448	0.2315	0.1575	0.4381
Mean	0.04033	0.21260	0.16882	0.40827
SD	0.00742	0.01845	0.01930	0.13426
N	5	5	5	5

Appendix 18

Appendix 2

Individual Organ Weights Relative to Body Weight: Day 29

5550008

Sex: Female Day(s) Relative to Start Date

0.36x 10E12 vg Group 2	Organ Weight (BW)						
	Brain (%)	Gland Adrenal (%)	Gland Pituitary (%)	Thyroid/ Parathyroid (%)	Heart (%)	Kidney (%)	Liver (%)
	-	-	-	-	-	-	-
2501	0.7694	0.0238	0.0057	0.0043	0.3903	0.7016	2.5403
2502	0.7462	0.0276	0.0054	0.0048	0.3570	0.6550	2.8779
2507	0.8633	0.0276	0.0074	0.0063	0.3824	0.6525	2.9235
2508	0.8860	0.0289	0.0078	0.0053	0.3555	0.7695	2.9801
2509	0.9179	0.0287	0.0087	0.0050	0.3676	0.7454	3.0667
Mean	0.83656	0.02732	0.00700	0.00513	0.37057	0.70480	2.87770
SD	0.07493	0.00208	0.00139	0.00076	0.01539	0.05258	0.20132
N	5	5	5	5	5	5	5
%Diff	-8.60524	0.38948	8.54283	-9.56462	0.12175	0.82053	5.14681

Appendix 18
Appendix 2

Individual Organ Weights Relative to Body Weight: Day 29

5550008
Sex: Female Day(s) Relative to Start Date

0.36x 10E12 vg Group 2	Organ Weight (BW)			
	Ovary (%)	Spleen (%)	Thymus (%)	Uterus/ Cervix (%)
	-	-	-	-
2501	0.0367	0.2028	0.1536	0.1605
2502	0.0418	0.2815	0.1550	0.2036
2507	0.0407	0.1701	0.2054	0.1701
2508	0.0470	0.1898	0.2229	0.2521
2509	0.0498	0.2227	0.1614	0.2990
Mean	0.04320	0.21340	0.17966	0.21708
SD	0.00520	0.04262	0.03222	0.05816
N	5	5	5	5
%Diff	7.09337	0.37761	6.42352	-46.82974

Appendix 18

Appendix 2

Individual Organ Weights Relative to Body Weight: Day 29

5550008

Sex: Female Day(s) Relative to Start Date

1.1x 10E12 vg Group 3	Organ Weight (BW)						
	Brain (%)	Gland Adrenal (%)	Gland Pituitary (%)	Thyroid/ Parathyroid (%)	Heart (%)	Kidney (%)	Liver (%)
	-	-	-	-	-	-	-
3504	0.8748	0.0289	0.0070	0.0076	0.3840	0.7733	3.2359
3505	0.9984	0.0294	0.0084	0.0062	0.3772	0.6244	2.8228
3506	0.9255	0.0228	0.0072	0.0052	0.3942	0.6606	3.1063
3514	0.8755	0.0300	0.0065	0.0075	0.3642	0.7029	2.8691
3615	0.7884	0.0320	0.0062	0.0044	0.3506	0.7307	2.7668
Mean	0.89251	0.02860	0.00709	0.00618	0.37405	0.69838	2.96018
SD	0.07707	0.00343	0.00086	0.00137	0.01704	0.05828	0.20120
N	5	5	5	5	5	5	5
%Diff	-2.49206	5.10231	9.85817	8.95154	1.06234	-0.09918	8.16034

Appendix 18

Appendix 2

Individual Organ Weights Relative to Body Weight: Day 29

5550008

Sex: Female Day(s) Relative to Start Date

1.1x 10E12 vg Group 3	Organ Weight (BW)			
	Ovary (%)	Spleen (%)	Thymus (%)	Uterus/ Cervix (%)
	-	-	-	-
3504	0.0549	0.2345	0.2684	0.2913
3505	0.0383	0.2016	0.1575	0.2461
3506	0.0394	0.1870	0.1529	0.3063
3514	0.0505	0.2088	0.1471	0.2377
3615	0.0714	0.2552	0.2149	0.2187
Mean	0.05090	0.21741	0.18817	0.26001
SD	0.01346	0.02723	0.05253	0.03712
N	5	5	5	5
%Diff	26.18478	2.26186	11.46181	-36.31425

Appendix 18

Appendix 2

Individual Organ Weights Relative to Body Weight: Day 29

5550008

Sex: Female Day(s) Relative to Start Date

3.3x 10E12 vg Group 4	Organ Weight (BW)						
	Brain (%)	Gland Adrenal (%)	Gland Pituitary (%)	Thyroid/ Parathyroid (%)	Heart (%)	Kidney (%)	Liver (%)
	-	-	-	-	-	-	-
4505	0.8820	0.0243	0.0078	0.0068	0.4062	0.7613	3.0448
4506	0.7941	0.0267	0.0067	0.0050	0.3770	0.7084	2.9615
4507	0.9706	0.0298	0.0081	0.0066	0.3904	0.7583	3.0968
4511	0.8575	0.0335	0.0061	0.0036	0.4070	0.7051	2.7729
4512	0.7831	0.0230	0.0074	0.0048	0.3847	0.6764	2.9360
Mean	0.85744	0.02746	0.00721	0.00539	0.39305	0.72192	2.96240
SD	0.07573	0.00426	0.00084	0.00133	0.01325	0.03678	0.12397
N	5	5	5	5	5	5	5
%Diff	-6.32310	0.90730	11.73858	-5.09303	6.19670	3.26836	8.24144

Appendix 18

Appendix 2

Individual Organ Weights Relative to Body Weight: Day 29

5550008

Sex: Female Day(s) Relative to Start Date

3.3x 10E12 vg Group 4	Organ Weight (BW)			
	Ovary (%)	Spleen (%)	Thymus (%)	Uterus/ Cervix (%)
	-	-	-	-
4505	0.0402	0.2124	0.1263	0.2175
4506	0.0523	0.2335	0.2640	0.1736
4507	0.0422	0.2075	0.1695	0.2781
4511	0.0407	0.2000	0.2023	0.1897
4512	0.0426	0.2260	0.1793	0.1636
Mean	0.04359	0.21587	0.18830	0.20452
SD	0.00497	0.01367	0.05052	0.04589
N	5	5	5	5
%Diff	8.08145	1.53879	11.53984	-49.90564

Appendix 18

Appendix 2

Individual Organ Weights Relative to Body Weight: Day 91

5550008

Sex: Male Day(s) Relative to Start Date

0 vg Group 1	Organ Weight (BW) Brain (%)	Epididymis (%)	Gland Adrenal (%)	Gland Pituitary (%)	Gland Prostate (%)	Thyroid/ Parathyroid (%)	Heart (%)
	-	-	-	-	-	-	-
1005	0.3863	0.2470	0.0078	0.0024	0.2269	0.0026	0.2835
1006	0.4052	0.2490	0.0087	0.0028	0.3020	0.0037	0.2985
1007	0.2901	0.2001	0.0087	0.0030	0.2314	0.0038	0.2920
1011	0.3583	0.2455	0.0068	0.0029	0.2266	0.0046	0.2536
1012	0.3767	0.2265	0.0069	0.0027	0.2378	0.0034	0.2960
Mean	0.36330	0.23362	0.00778	0.00277	0.24496	0.00361	0.28471
SD	0.04427	0.02078	0.00096	0.00023	0.03222	0.00072	0.01832
N	5	5	5	5	5	5	5

Appendix 18

Appendix 2

Individual Organ Weights Relative to Body Weight: Day 91

5550008

Sex: Male Day(s) Relative to Start Date

0	Organ Weight (BW)				
vg	Kidney	Liver	Spleen	Testis	Thymus
Group 1	(%)	(%)	(%)	(%)	(%)
	-	-	-	-	-
1005	0.4833	2.3058	0.1438	0.7363	0.0375
1006	0.5720	2.3169	0.1775	0.6657	0.0466
1007	0.5149	3.0633	0.1306	0.5640	0.0482
1011	0.5633	2.1992	0.1683	0.6341	0.0422
1012	0.5413	2.3903	0.1532	0.5822	0.0480
Mean	0.53496	2.45512	0.15469	0.63646	0.04449
SD	0.03637	0.34677	0.01877	0.06896	0.00459
N	5	5	5	5	5

Appendix 18

Appendix 2

Individual Organ Weights Relative to Body Weight: Day 91

5550008

Sex: Male Day(s) Relative to Start Date

0.36x 10E12 vg Group 2	Organ Weight (BW)						
	Brain (%)	Epididymis (%)	Gland Adrenal (%)	Gland Pituitary (%)	Gland Prostate (%)	Thyroid/ Parathyroid (%)	Heart (%)
	-	-	-	-	-	-	-
2103	0.3930	0.2438	0.0089	0.0020	0.2218	0.0042	0.2749
2005	0.3264	0.2172	0.0089	0.0027	0.2276	0.0036	0.2451
2006	0.3693	0.2471	0.0079	0.0024	0.2400	0.0028	0.3073
2007	0.3343	0.2171	0.0089	0.0030	0.2580	0.0033	0.2717
2008	0.3969	0.1515	0.0122	0.0027	0.2511	0.0027	0.3078
Mean	0.36395	0.21533	0.00937	0.00256	0.23971	0.00332	0.28137
SD	0.03257	0.03841	0.00166	0.00035	0.01527	0.00063	0.02655
N	5	5	5	5	5	5	5
%Diff	0.17971	-7.82694	20.50968	-7.35428	-2.14626	-8.12325	-1.17550

Appendix 18

Appendix 2

Individual Organ Weights Relative to Body Weight: Day 91

5550008

Sex: Male Day(s) Relative to Start Date

0.36x 10E12 vg Group 2	Organ Weight (BW)				
	Kidney (%)	Liver (%)	Spleen (%)	Testis (%)	Thymus (%)
	-	-	-	-	-
2103	0.5113	2.1980	0.1715	0.6377	0.0425
2005	0.4296	2.1707	0.1410	0.6384	0.0544
2006	0.5446	2.4792	0.1266	0.6418	0.0412
2007	0.5178	2.3692	0.1709	0.6058	0.0437
2008	0.5732	2.3543	0.1412	0.5181	0.0459
Mean	0.51532	2.31429	0.15025	0.60837	0.04553
SD	0.05382	0.12838	0.02002	0.05253	0.00527
N	5	5	5	5	5
%Diff	-3.67262	-5.73637	-2.87401	-4.41211	2.32302

Appendix 18
Appendix 2

Individual Organ Weights Relative to Body Weight: Day 91

5550008
Sex: Male Day(s) Relative to Start Date

1.1x 10E12 vg Group 3	Organ Weight (BW)						
	Brain (%)	Epididymis (%)	Gland Adrenal (%)	Gland Pituitary (%)	Gland Prostate (%)	Thyroid/ Parathyroid (%)	Heart (%)
	-	-	-	-	-	-	-
3006	0.4230	0.2545	0.0107	0.0028	0.1920	0.0032	0.3285
3007	0.3460	0.2071	0.0088	0.0027	0.2790	0.0031	0.3039
3008	0.4022	0.2228	0.0078	0.0025	0.1948	0.0029	0.2547
3014	0.4209	0.2630	0.0098	0.0027	0.2543	0.0035	0.2811
3015	0.3657	0.2156	0.0069	0.0030	0.2362	0.0037	0.2730
Mean	0.39156	0.23259	0.00880	0.00272	0.23128	0.00328	0.28821
SD	0.03429	0.02469	0.00152	0.00016	0.03775	0.00031	0.02861
N	5	5	5	5	5	5	5
%Diff	7.77897	-0.43898	13.10082	-1.75062	-5.58724	-9.15280	1.22834

Appendix 18

Appendix 2

Individual Organ Weights Relative to Body Weight: Day 91

5550008

Sex: Male Day(s) Relative to Start Date

1.1x 10E12 vg Group 3	Organ Weight (BW)				
	Kidney (%)	Liver (%)	Spleen (%)	Testis (%)	Thymus (%)
	-	-	-	-	-
3006	0.5486	2.3674	0.1732	0.6930	0.0947
3007	0.4890	2.4138	0.1390	0.6100	0.0438
3008	0.4809	2.1616	0.1350	0.5962	0.0557
3014	0.5380	2.1646	0.1461	0.6946	0.0503
3015	0.5511	2.3852	0.1323	0.6467	0.0489
Mean	0.52151	2.29852	0.14513	0.64810	0.05866
SD	0.03388	0.12475	0.01656	0.04563	0.02059
N	5	5	5	5	5
%Diff	-2.51477	-6.37844	-6.18151	1.82993	31.82540

Appendix 18

Appendix 2

Individual Organ Weights Relative to Body Weight: Day 91

5550008

Sex: Male Day(s) Relative to Start Date

3.3x 10E12 vg Group 4	Organ Weight (BW)						
	Brain (%)	Epididymis (%)	Gland Adrenal (%)	Gland Pituitary (%)	Gland Prostate (%)	Thyroid/ Parathyroid (%)	Heart (%)
	-	-	-	-	-	-	-
4009	0.4054	0.1557	0.0104	0.0024	0.3310	0.0030	0.2703
4010	0.4993	0.2874	0.0103	0.0035	0.2833	0.0032	0.3172
4011	0.4154	0.2712	0.0082	0.0030	0.3263	0.0048	0.3107
4014	0.4644	0.2721	0.0109	0.0021	0.2826	0.0034	0.2814
4015	0.4009	0.1863	0.0112	0.0028	0.2971	0.0034	0.3080
Mean	0.43706	0.23456	0.01019	0.00277	0.30405	0.00356	0.29752
SD	0.04302	0.05934	0.00117	0.00051	0.02325	0.00068	0.02048
N	5	5	5	5	5	5	5
%Diff	20.30426	0.40283	31.01288	0.01156	24.11950	-1.37755	4.49820

Appendix 18

Appendix 2

Individual Organ Weights Relative to Body Weight: Day 91

5550008

Sex: Male Day(s) Relative to Start Date

3.3x 10E12 vg Group 4	Organ Weight (BW)				
	Kidney (%)	Liver (%)	Spleen (%)	Testis (%)	Thymus (%)
	-	-	-	-	-
4009	0.6296	2.4844	0.1457	0.4145	0.0375
4010	0.5549	2.2586	0.1574	0.8479	0.0481
4011	0.5562	2.3783	0.1369	0.7069	0.0789
4014	0.5549	2.2534	0.1773	0.7996	0.0559
4015	0.5494	2.2705	0.2106	0.4446	0.0477
Mean	0.56898	2.32905	0.16558	0.64271	0.05362
SD	0.03397	0.10084	0.02936	0.20134	0.01557
N	5	5	5	5	5
%Diff	6.35934	-5.13495	7.03623	0.98289	20.49726

Appendix 18

Appendix 2

Individual Organ Weights Relative to Body Weight: Day 91

5550008

Sex: Female Day(s) Relative to Start Date

0	Organ Weight (BW)						
vg<br><br>Group 1	Brain<br><br>(%)	Gland Adrenal<br>(%)	Gland Pituitary<br>(%)	Thyroid/ Parathyroid<br>(%)	Heart<br><br>(%)	Kidney<br><br>(%)	Liver<br><br>(%)
	-	-	-	-	-	-	-
1503	0.7495	0.0274	0.0058	0.0045	0.3342	0.6440	2.3378
1504	0.6951	0.0219	0.0068	0.0041	0.3074	0.5650	2.4110
1510	0.7137	0.0295	0.0080	0.0046	0.3270	0.6133	2.3457
1511	0.7014	0.0225	0.0069	0.0059	0.3689	0.6368	2.4784
1512	0.8345	0.0238	0.0059	0.0051	0.3698	0.5957	2.3464
Mean	0.73880	0.02502	0.00669	0.00483	0.34145	0.61097	2.38385
SD	0.05748	0.00329	0.00091	0.00069	0.02729	0.03205	0.06051
N	5	5	5	5	5	5	5

Appendix 18

Appendix 2

Individual Organ Weights Relative to Body Weight: Day 91

5550008

Sex: Female Day(s) Relative to Start Date

0	Organ Weight (BW)			
vg<br>Group 1	Ovary<br>(%)	Spleen<br>(%)	Thymus<br>(%)	Uterus/<br>Cervix<br>(%)
	-	-	-	-
1503	0.0298	0.2164	0.0684	0.1785
1504	0.0283	0.1908	0.0700	0.2018
1510	0.0320	0.2223	0.0969	0.3074
1511	0.0314	0.2220	0.0963	0.1767
1512	0.0302	0.1949	0.1055	0.2234
Mean	0.03035	0.20926	0.08740	0.21757
SD	0.00147	0.01523	0.01706	0.05374
N	5	5	5	5

Appendix 18

Appendix 2

Individual Organ Weights Relative to Body Weight: Day 91

5550008

Sex: Female Day(s) Relative to Start Date

0.36x 10E12 vg Group 2	Organ Weight (BW)						
	Brain (%)	Gland Adrenal (%)	Gland Pituitary (%)	Thyroid/ Parathyroid (%)	Heart (%)	Kidney (%)	Liver (%)
	-	-	-	-	-	-	-
2503	0.7847	0.0229	0.0073	0.0037	0.3458	0.7028	2.7245
2504	0.6187	0.0189	0.0047	0.0059	0.2994	0.6405	2.5000
2613	-	0.0202	0.0056	0.0053	0.3319	0.5621	2.1944
2614	0.5858	0.0184	0.0066	0.0054	0.3091	0.5294	2.0227
2615	0.7390	0.0278	0.0084	0.0045	0.3543	0.6502	2.6952
Mean	0.68207	0.02166	0.00653	0.00496	0.32809	0.61701	2.42734
SD	0.09500	0.00386	0.00141	0.00086	0.02345	0.07017	0.30958
N	4	5	5	5	5	5	5
%Diff	-7.67918	-13.43939	-2.35374	2.72432	-3.91365	0.98753	1.82451

Appendix 18
Appendix 2

Individual Organ Weights Relative to Body Weight: Day 91

5550008
Sex: Female Day(s) Relative to Start Date

0.36x 10E12 vg Group 2	Organ Weight (BW)			
	Ovary (%)	Spleen (%)	Thymus (%)	Uterus/ Cervix (%)
	-	-	-	-
2503	0.0378	0.2092	0.0643	0.2293
2504	0.0218	0.1378	0.0529	0.1770
2613	0.0295	0.1211	0.0688	0.1919
2614	0.0304	0.1650	0.0576	0.1515
2615	0.0342	0.1870	0.0673	0.4071
Mean	0.03072	0.16402	0.06216	0.23136
SD	0.00599	0.03574	0.00674	0.10218
N	5	5	5	5
%Diff	1.21828	-21.61950	-28.88399	6.34092

Appendix 18

Appendix 2

Individual Organ Weights Relative to Body Weight: Day 91

5550008

Sex: Female Day(s) Relative to Start Date

1.1x 10E12 vg Group 3	Organ Weight (BW)						
	Brain (%)	Gland Adrenal (%)	Gland Pituitary (%)	Thyroid/ Parathyroid (%)	Heart (%)	Kidney (%)	Liver (%)
	-	-	-	-	-	-	-
3501	0.7435	0.0291	0.0094	0.0060	0.3634	0.6405	2.4981
3502	0.5733	0.0149	0.0050	0.0032	0.3480	0.5253	2.2335
3503	0.8701	0.0243	0.0086	0.0059	0.3585	0.5722	2.4726
3509	0.7705	0.0185	0.0077	0.0047	0.3223	0.5489	2.4364
3510	0.6826	0.0171	0.0054	0.0050	0.3548	0.6651	2.6552
Mean	0.72799	0.02077	0.00722	0.00495	0.34939	0.59040	2.45915
SD	0.10982	0.00581	0.00191	0.00113	0.01613	0.05996	0.15128
N	5	5	5	5	5	5	5
%Diff	-1.46316	-17.00130	7.84513	2.40557	2.32610	-3.36780	3.15882

Appendix 18

Appendix 2

Individual Organ Weights Relative to Body Weight: Day 91

5550008

Sex: Female Day(s) Relative to Start Date

1.1x 10E12 vg Group 3	Organ Weight (BW)			
	Ovary (%)	Spleen (%)	Thymus (%)	Uterus/ Cervix (%)
	-	-	-	-
3501	0.0324	0.1874	0.0637	1.0179
3502	0.0341	0.1553	0.0657	0.1616
3503	0.0232	0.1672	0.0801	0.2996
3509	0.0265	0.1807	0.0856	0.5636
3510	0.0306	0.2028	0.1057	0.1584
Mean	0.02937	0.17869	0.08016	0.44022
SD	0.00444	0.01831	0.01704	0.36258
N	5	5	5	5
%Diff	-3.22263	-14.60663	-8.28979	102.33940

Appendix 18

Appendix 2

Individual Organ Weights Relative to Body Weight: Day 91

5550008

Sex: Female Day(s) Relative to Start Date

3.3x 10E12 vg Group 4	Organ Weight (BW)						
	Brain (%)	Gland Adrenal (%)	Gland Pituitary (%)	Thyroid/ Parathyroid (%)	Heart (%)	Kidney (%)	Liver (%)
	-	-	-	-	-	-	-
4503	0.7237	0.0249	0.0087	0.0026	0.3821	0.5376	2.4369
4504	0.7924	0.0223	0.0065	0.0040	0.3430	0.7717	2.4895
4513	0.7361	0.0227	0.0084	0.0046	0.3602	0.6453	2.3358
4514	0.6291	0.0159	0.0062	0.0045	0.3232	0.5616	2.4166
4515	0.7855	0.0237	0.0118	0.0060	0.3868	0.6686	3.0140
Mean	0.73337	0.02191	0.00831	0.00435	0.35906	0.63696	2.53855
SD	0.06552	0.00350	0.00226	0.00123	0.02666	0.09325	0.27150
N	5	5	5	5	5	5	5
%Diff	-0.73444	-12.42224	24.25478	-10.08307	5.15620	4.25342	6.48955

Appendix 18
Appendix 2

Individual Organ Weights Relative to Body Weight: Day 91

5550008
Sex: Female Day(s) Relative to Start Date

3.3x 10E12 vg Group 4	Organ Weight (BW)			
	Ovary (%)	Spleen (%)	Thymus (%)	Uterus/ Cervix (%)
	-	-	-	-
4503	0.0229	0.1749	0.0975	0.2384
4504	0.0350	0.2051	0.0966	0.5257
4513	0.0266	0.2255	0.0883	0.2847
4514	0.0215	0.1725	0.0540	0.3616
4515	0.0207	0.1694	0.0748	0.2620
Mean	0.02536	0.18949	0.08224	0.33447
SD	0.00587	0.02473	0.01824	0.11650
N	5	5	5	5
%Diff	-16.45001	-9.44627	-5.90716	53.73154

Individual Organ Weights Relative to Brain Weight Explanation Page

Dosing Information

Dosing information is abbreviated on various data outputs; the following represents the dosing information for this study:

Group No.	Test Material	Dose Level (vg)
1	Reference Item	0
2	AAV9/AP4M1	3.6E11
3	AAV9/AP4M1	1.1E12
4	AAV9/AP4M1	3.3E12

Appendix 18

Appendix 3

Individual Organ Weights Relative to Brain Weight: Day 8

5550008

Sex: Male Day(s) Relative to Start Date

0<br>vg<br>Group 1	Organ Weight/Brain Ratio<br>Epididymis<br>(%)	Gland Adrenal<br>(%)	Gland Pituitary<br>(%)	Gland Prostate<br>(%)	Thyroid/ Parathyroid<br>(%)	Heart<br>(%)	Kidney<br>(%)
	-	-	-	-	-	-	-
1001	19.8974	2.0256	0.6256	20.5641	0.5949	48.7179	111.2308
1002	22.3649	2.3542	0.4976	26.2707	0.8293	46.5490	102.2472
1014	30.3437	2.2986	0.5961	25.7787	0.6284	47.2073	115.1450
1015	26.7542	2.0545	0.4139	20.1918	0.4089	46.4917	116.2039
Mean	24.84005	2.18324	0.53332	23.20135	0.61536	47.24147	111.20673
SD	4.63717	0.16728	0.09661	3.26988	0.17224	1.03649	6.34445
N	4	4	4	4	4	4	4

Appendix 18

Appendix 3

Individual Organ Weights Relative to Brain Weight: Day 8

5550008

Sex: Male Day(s) Relative to Start Date

0	Organ Weight/Brain Ratio			
vg	Liver	Spleen	Testis	Thymus
Group 1	(%)	(%)	(%)	(%)
	-	-	-	-
1001	430.8205	35.6923	145.4359	29.8462
1002	417.3890	30.1766	156.1263	32.5308
1014	446.0795	40.1719	157.6799	27.4436
1015	399.5457	32.1555	145.7850	25.3407
Mean	423.45866	34.54905	151.25676	28.79032
SD	19.78695	4.38827	6.55215	3.09941
N	4	4	4	4

Appendix 18

Appendix 3

Individual Organ Weights Relative to Brain Weight: Day 8

5550008

Sex: Male Day(s) Relative to Start Date

0.36x 10E12 vg Group 2	Organ Weight/Brain Ratio						
	Epididymis (%)	Gland Adrenal (%)	Gland Pituitary (%)	Gland Prostate (%)	Thyroid/ Parathyroid (%)	Heart (%)	Kidney (%)
	-	-	-	-	-	-	-
2004	18.2992	1.7761	0.4467	22.0129	0.7643	39.1819	84.1227
2009	24.2019	2.3018	0.6385	26.7765	0.5716	64.0062	134.3975
2010	25.6000	2.4941	0.6071	17.9765	0.5176	48.7529	116.0000
2014	32.5557	2.4019	0.5673	26.4581	0.4772	54.0297	109.7031
2015	29.3094	2.2088	0.5061	28.9931	0.4270	47.3379	97.5751
Mean	25.99324	2.23653	0.55314	24.44343	0.55154	50.66173	108.35969
SD	5.39963	0.27871	0.07736	4.41393	0.13023	9.16372	18.98609
N	5	5	5	5	5	5	5
%Diff	4.64245	2.44066	3.71517	5.35351	-10.37171	7.23994	-2.56013

Appendix 18

Appendix 3

Individual Organ Weights Relative to Brain Weight: Day 8

5550008

Sex: Male Day(s) Relative to Start Date

0.36x 10E12 vg Group 2	Organ Weight/Brain Ratio			
	Liver (%)	Spleen (%)	Testis (%)	Thymus (%)
	-	-	-	-
2004	343.8644	25.9419	132.7772	27.1259
2009	471.9361	32.9557	126.8795	36.7147
2010	463.8588	32.8471	150.6353	22.0706
2014	430.8059	35.2068	163.2556	27.8367
2015	369.8471	22.2457	147.8123	28.3606
Mean	416.06248	29.83942	144.27198	28.42170
SD	56.94768	5.48685	14.56049	5.27105
N	5	5	5	5
%Diff	-1.74661	-13.63173	-4.61783	-1.28034

Appendix 18

Appendix 3

Individual Organ Weights Relative to Brain Weight: Day 8

5550008

Sex: Male Day(s) Relative to Start Date

1.1x 10E12 vg Group 3	Organ Weight/Brain Ratio						
	Epididymis (%)	Gland Adrenal (%)	Gland Pituitary (%)	Gland Prostate (%)	Thyroid/ Parathyroid (%)	Heart (%)	Kidney (%)
	-	-	-	-	-	-	-
3001	27.3731	1.7439	0.3698	27.4834	0.5629	46.1369	96.6887
3002	24.4306	1.8064	0.5694	26.9669	0.4762	49.5859	112.2153
3003	18.4337	1.9766	0.3676	20.7246	0.4315	43.0474	93.7134
3012	26.2404	2.0563	0.4450	27.0077	0.4092	54.3734	109.2583
3013	28.2859	2.6437	0.5547	25.4873	0.4598	57.5712	105.0475
Mean	24.95273	2.04537	0.46129	25.53396	0.46792	50.14296	103.38464
SD	3.91780	0.35735	0.09725	2.79097	0.05901	5.91146	7.96264
N	5	5	5	5	5	5	5
%Diff	0.45360	-6.31495	-13.50640	10.05379	-23.95906	6.14184	-7.03382

Appendix 18

Appendix 3

Individual Organ Weights Relative to Brain Weight: Day 8

5550008

Sex: Male Day(s) Relative to Start Date

1.1x 10E12 vg Group 3	Organ Weight/Brain Ratio			
	Liver (%)	Spleen (%)	Testis (%)	Thymus (%)
	-	-	-	-
3001	395.9161	32.1744	146.7991	28.6424
3002	453.4161	31.5217	148.3437	30.5383
3003	366.4358	23.8679	144.3261	35.3223
3012	396.1125	29.9744	131.3555	24.5524
3013	411.4443	27.6362	146.6767	25.4873
Mean	404.66498	29.03492	143.50020	28.90854
SD	31.75607	3.37558	6.93916	4.31696
N	5	5	5	5
%Diff	-4.43814	-15.96029	-5.12807	0.41063

Appendix 18

Appendix 3

Individual Organ Weights Relative to Brain Weight: Day 8

5550008

Sex: Male Day(s) Relative to Start Date

3.3x 10E12 vg Group 4	Organ Weight/Brain Ratio						
	Epididymis (%)	Gland Adrenal (%)	Gland Pituitary (%)	Gland Prostate (%)	Thyroid/ Parathyroid (%)	Heart (%)	Kidney (%)
	-	-	-	-	-	-	-
4001	23.3759	2.3987	0.4442	28.8728	0.6441	47.3626	108.1066
4002	19.6004	1.6199	0.4428	12.2030	0.6533	43.0886	105.0756
4006	26.1477	1.8912	0.4491	27.9940	0.6238	44.1118	91.4172
4007	23.0686	2.0367	0.4052	18.5305	0.6159	39.4381	75.0945
4008	24.7920	1.9634	0.6988	27.0105	0.6656	54.5202	119.0238
Mean	23.39693	1.98198	0.48802	22.92219	0.64053	45.70426	99.74355
SD	2.45126	0.28122	0.11915	7.28072	0.02057	5.68141	16.93372
N	5	5	5	5	5	5	5
%Diff	-5.80966	-9.21856	-8.49531	-1.20320	4.08984	-3.25395	-10.30799

Appendix 18

Appendix 3

Individual Organ Weights Relative to Brain Weight: Day 8

5550008

Sex: Male Day(s) Relative to Start Date

3.3x 10E12 vg Group 4	Organ Weight/Brain Ratio			
	Liver (%)	Spleen (%)	Testis (%)	Thymus (%)
	-	-	-	-
4001	392.0600	36.1466	129.3726	26.3742
4002	394.3305	35.0432	133.9633	29.3197
4006	336.6766	20.4092	142.7645	16.1677
4007	287.8984	20.8536	139.8163	24.7974
4008	448.8630	32.2241	161.9523	40.4326
Mean	371.96570	28.93533	141.57379	27.41831
SD	61.50159	7.71579	12.51579	8.76817
N	5	5	5	5
%Diff	-12.16009	-16.24857	-6.40168	-4.76550

Appendix 18

Appendix 3

Individual Organ Weights Relative to Brain Weight: Day 8

5550008

Sex: Female Day(s) Relative to Start Date

0<br>vg<br>Group 1	Organ Weight/Brain Ratio<br>Gland<br>Adrenal<br>(%)	Gland<br>Pituitary<br>(%)	Thyroid/<br>Parathyroid<br>(%)	Heart<br><br>(%)	Kidney<br><br>(%)	Liver<br><br>(%)	Ovary<br><br>(%)
	-	-	-	-	-	-	-
1501	3.0277	0.8489	0.5207	36.3894	86.2479	338.4833	3.8483
1502	2.7995	0.7335	0.4890	41.5648	92.7262	328.4230	3.7286
1505	2.9315	0.7541	0.3824	36.8561	76.0489	302.9740	4.0892
1506	2.8369	0.6765	0.4092	35.4064	80.6328	292.4168	3.9825
1507	3.8744	0.7240	0.6278	39.9887	83.4276	384.1629	4.3552
Mean	3.09401	0.74740	0.48580	38.04107	83.81667	329.29199	4.00078
SD	0.44517	0.06348	0.09743	2.61168	6.24333	35.87642	0.24036
N	5	5	5	5	5	5	5

Appendix 18

Appendix 3

Individual Organ Weights Relative to Brain Weight: Day 8

5550008

Sex: Female Day(s) Relative to Start Date

0	Organ Weight/Brain Ratio		
vg<br><br>Group 1	Spleen<br><br>(%)	Thymus<br><br>(%)	Uterus/<br>Cervix<br>(%)
	-	-	-
1501	20.7697	20.1471	24.1087
1502	27.0171	25.0611	37.3472
1505	19.9150	22.3579	23.8980
1506	21.7130	27.1140	22.9678
1507	22.0023	23.3032	24.7738
Mean	22.28342	23.59668	26.61909
SD	2.77096	2.64746	6.03191
N	5	5	5

Appendix 18

Appendix 3

Individual Organ Weights Relative to Brain Weight: Day 8

5550008

Sex: Female Day(s) Relative to Start Date

0.36x 10E12 vg Group 2	Organ Weight/Brain Ratio						
	Gland Adrenal (%)	Gland Pituitary (%)	Thyroid/ Parathyroid (%)	Heart (%)	Kidney (%)	Liver (%)	Ovary (%)
	-	-	-	-	-	-	-
2505	2.6328	0.7522	0.3821	38.8657	79.1045	326.7463	3.2836
2506	2.9745	0.7118	0.6134	35.3588	74.0162	281.4815	2.9514
2510	2.9608	0.4386	0.4021	38.9556	80.9399	343.8120	4.0731
2511	3.1748	0.8131	0.4314	43.3628	99.8341	334.6792	4.6460
2512	3.5121	0.8824	0.4787	44.9250	88.8697	392.3875	4.7290
Mean	3.05102	0.71962	0.46154	40.29359	84.55287	335.82130	3.93661
SD	0.32279	0.16977	0.09235	3.84226	10.07360	39.68648	0.79793
N	5	5	5	5	5	5	5
%Diff	-1.38946	-3.71649	-4.99518	5.92128	0.87835	1.98283	-1.60397

Appendix 18

Appendix 3

Individual Organ Weights Relative to Brain Weight: Day 8

5550008

Sex: Female Day(s) Relative to Start Date

0.36x 10E12 vg Group 2	Organ Weight/Brain Ratio		
	Spleen (%)	Thymus (%)	Uterus/ Cervix (%)
	-	-	-
2505	21.6716	26.9254	50.5672
2506	20.0231	25.5208	19.4444
2510	25.3786	22.5587	20.3133
2511	30.8075	26.2168	60.1217
2512	25.2018	31.1419	18.0507
Mean	24.61655	26.47273	33.69947
SD	4.15401	3.09421	20.06196
N	5	5	5
%Diff	10.47024	12.18836	26.59888

Appendix 18

Appendix 3

Individual Organ Weights Relative to Brain Weight: Day 8

5550008

Sex: Female Day(s) Relative to Start Date

1.1x 10E12 vg Group 3	Organ Weight/Brain Ratio						
	Gland Adrenal (%)	Gland Pituitary (%)	Thyroid/ Parathyroid (%)	Heart (%)	Kidney (%)	Liver (%)	Ovary (%)
	-	-	-	-	-	-	-
3507	2.6594	0.5722	0.5395	37.0572	88.8283	304.5777	4.5777
3508	2.8654	0.6444	0.5581	37.5144	95.4545	348.4465	4.5455
3511	3.8559	0.8785	0.3577	42.1357	83.7980	307.0489	4.9448
3512	3.6783	0.6476	0.4936	39.4374	77.9724	254.8832	3.6624
3513	2.9640	0.8380	0.4049	41.3386	86.7829	293.5883	4.3307
Mean	3.20460	0.71614	0.47078	39.49665	86.56724	301.70892	4.41220
SD	0.52886	0.13395	0.08659	2.24951	6.43590	33.46541	0.47370
N	5	5	5	5	5	5	5
%Diff	3.57419	-4.18219	-3.09208	3.82635	3.28165	-8.37648	10.28351

Appendix 18

Appendix 3

Individual Organ Weights Relative to Brain Weight: Day 8

5550008

Sex: Female Day(s) Relative to Start Date

1.1x 10E12 vg Group 3	Organ Weight/Brain Ratio		
	Spleen (%)	Thymus (%)	Uterus/ Cervix (%)
	-	-	-
3507	23.9782	19.8365	39.4005
3508	27.4453	20.3682	20.5409
3511	19.3056	19.0952	36.6123
3512	21.3907	16.5074	35.6157
3513	24.9719	22.7784	20.9224
Mean	23.41834	19.71716	30.61836
SD	3.16107	2.26377	9.13230
N	5	5	5
%Diff	5.09311	-16.44095	15.02407

Appendix 18

Appendix 3

Individual Organ Weights Relative to Brain Weight: Day 8

5550008

Sex: Female Day(s) Relative to Start Date

3.3x 10E12 vg Group 4	Organ Weight/Brain Ratio						
	Gland Adrenal (%)	Gland Pituitary (%)	Thyroid/ Parathyroid (%)	Heart (%)	Kidney (%)	Liver (%)	Ovary (%)
	-	-	-	-	-	-	-
4601	3.0449	0.7230	0.4960	37.5198	73.5092	324.5383	3.8522
4502	2.8383	0.5791	0.3899	33.3142	75.2867	289.6216	3.2683
4508	2.4146	0.6207	0.4271	36.1048	76.3667	289.0091	4.5558
4509	2.7934	0.7860	0.3080	40.9453	80.7223	313.7015	3.5582
4510	3.1766	0.9188	0.4986	47.3647	99.5726	366.0969	3.8462
Mean	2.85356	0.72552	0.42393	39.04975	81.09152	316.59347	3.81614
SD	0.29049	0.13549	0.07965	5.40002	10.66770	31.66406	0.47853
N	5	5	5	5	5	5	5
%Diff	-7.77152	-2.92700	-12.73618	2.65156	-3.25133	-3.85631	-4.61508

Appendix 18

Appendix 3

Individual Organ Weights Relative to Brain Weight: Day 8

5550008

Sex: Female Day(s) Relative to Start Date

3.3x 10E12 vg Group 4	Organ Weight/Brain Ratio		
	Spleen (%)	Thymus (%)	Uterus/ Cervix (%)
	-	-	-
4601	19.7361	20.2111	17.9420
4502	17.8899	12.5573	49.1972
4508	18.4510	25.8542	23.1207
4509	21.8800	22.1986	51.2480
4510	25.3561	23.1481	52.0655
Mean	20.66264	20.79388	38.71469
SD	3.03926	5.03247	16.73239
N	5	5	5
%Diff	-7.27350	-11.87794	45.43958

Appendix 18

Appendix 3

Individual Organ Weights Relative to Brain Weight: Day 29

5550008

Sex: Male Day(s) Relative to Start Date

0	Organ Weight/Brain Ratio						
vg<br>Group 1	Epididymis<br>(%)	Gland Adrenal<br>(%)	Gland Pituitary<br>(%)	Gland Prostate<br>(%)	Thyroid/ Parathyroid<br>(%)	Heart<br>(%)	Kidney<br>(%)
	-	-	-	-	-	-	-
1003	47.8393	2.7605	0.6660	48.6019	0.7931	68.5308	131.7234
1004	37.7477	2.2813	0.2562	28.1779	0.7395	56.1624	121.9913
1008	51.8308	2.8520	0.5931	50.4899	0.5106	60.6498	128.4167
1009	48.8832	3.3909	0.7360	53.1980	0.9695	57.2081	136.9036
1010	44.6315	2.9117	0.6824	47.2247	0.7916	59.9636	123.0209
Mean	46.18653	2.83929	0.58674	45.53849	0.76086	60.50294	128.41119
SD	5.37341	0.39598	0.19174	9.95997	0.16480	4.85938	6.19195
N	5	5	5	5	5	5	5

Appendix 18

Appendix 3

Individual Organ Weights Relative to Brain Weight: Day 29

5550008

Sex: Male Day(s) Relative to Start Date

0<br>vg<br>Group 1	Organ Weight/Brain Ratio<br>Liver<br>(%)<br>-	 Spleen<br>(%)<br>-	 Testis<br>(%)<br>-	 Thymus<br>(%)<br>-
1003	478.4952	39.5526	193.5943	25.2161
1004	489.1252	26.5346	140.4060	26.9212
1008	479.1129	42.1351	167.5606	38.8860
1009	483.0457	40.1523	146.6497	23.2487
1010	478.8444	53.6852	175.6142	24.7498
Mean	481.72468	40.41195	164.76497	27.80436
SD	4.52933	9.65059	21.66895	6.33180
N	5	5	5	5

Appendix 18

Appendix 3

Individual Organ Weights Relative to Brain Weight: Day 29

5550008

Sex: Male Day(s) Relative to Start Date

0.36x 10E12 vg Group 2	Organ Weight/Brain Ratio						
	Epididymis (%)	Gland Adrenal (%)	Gland Pituitary (%)	Gland Prostate (%)	Thyroid/ Parathyroid (%)	Heart (%)	Kidney (%)
	-	-	-	-	-	-	-
2001	40.5075	2.3697	0.6570	41.7762	0.5437	63.6158	127.8659
2002	45.8124	2.8101	0.4851	44.2563	0.6270	60.8238	124.4394
2011	49.9060	2.8524	0.6438	46.7105	1.0338	69.4549	122.0865
2012	44.2075	2.4430	0.3442	51.6238	1.0228	61.3669	123.2671
2013	44.9859	2.7554	0.5998	35.0984	0.4358	64.6673	122.6336
Mean	45.08385	2.64614	0.54598	43.89305	0.73263	63.98574	124.05847
SD	3.37286	0.22306	0.13155	6.11683	0.27833	3.44125	2.30146
N	5	5	5	5	5	5	5
%Diff	-2.38744	-6.80279	-6.94766	-3.61330	-3.71086	5.75641	-3.38967

Appendix 18

Appendix 3

Individual Organ Weights Relative to Brain Weight: Day 29

5550008

Sex: Male   Day(s) Relative to Start Date

0.36x 10E12 vg Group 2	Organ Weight/Brain Ratio			
	Liver (%)	Spleen (%)	Testis (%)	Thymus (%)
	-	-	-	-
2001	485.9538	32.4875	173.0856	18.9850
2002	565.4005	39.4050	152.0366	34.6453
2011	462.5940	38.1109	166.8233	29.8402
2012	528.5507	32.6224	147.5521	30.1987
2013	483.3177	33.2709	150.8435	17.1040
Mean	505.16332	35.17934	158.06823	26.15467
SD	41.33360	3.31197	11.19531	7.67050
N	5	5	5	5
%Diff	4.86557	-12.94816	-4.06442	-5.93321

Appendix 18

Appendix 3

Individual Organ Weights Relative to Brain Weight: Day 29

5550008

Sex: Male Day(s) Relative to Start Date

1.1x 10E12 vg Group 3	Organ Weight/Brain Ratio						
	Epididymis (%)	Gland Adrenal (%)	Gland Pituitary (%)	Gland Prostate (%)	Thyroid/ Parathyroid (%)	Heart (%)	Kidney (%)
	-	-	-	-	-	-	-
3004	43.2392	2.6186	0.6357	30.6256	0.5550	63.2190	111.3522
3005	52.3093	2.7089	0.6746	56.6684	0.7680	71.1988	130.2024
3009	49.7339	3.0140	0.6144	33.8655	0.5902	62.8447	124.5283
3010	50.4979	2.3423	0.6449	53.5325	0.8345	60.3129	109.9573
3011	36.7337	2.5427	0.5578	40.8543	0.5126	56.6834	121.4070
Mean	46.50278	2.64531	0.62548	43.10926	0.65207	62.85176	119.48944
SD	6.44599	0.24649	0.04359	11.60675	0.14093	5.34559	8.67339
N	5	5	5	5	5	5	5
%Diff	0.68473	-6.83203	6.60210	-5.33446	-14.29904	3.88216	-6.94779

Appendix 18

Appendix 3

Individual Organ Weights Relative to Brain Weight: Day 29

5550008

Sex: Male Day(s) Relative to Start Date

1.1x 10E12 vg Group 3	Organ Weight/Brain Ratio			
	Liver (%)	Spleen (%)	Testis (%)	Thymus (%)
	-	-	-	-
3004	447.8809	33.3502	163.5721	22.0484
3005	543.1240	34.5615	186.1443	25.3243
3009	522.3029	35.4620	180.4548	25.5926
3010	490.5642	31.6738	170.4125	24.5614
3011	509.4975	31.6080	111.5075	27.2362
Mean	502.67391	33.33110	162.41825	24.95260
SD	36.11434	1.71542	29.77155	1.89406
N	5	5	5	5
%Diff	4.34880	-17.52168	-1.42428	-10.25652

Appendix 18

Appendix 3

Individual Organ Weights Relative to Brain Weight: Day 29

5550008

Sex: Male Day(s) Relative to Start Date

3.3x 10E12 vg Group 4	Organ Weight/Brain Ratio						
	Epididymis (%)	Gland Adrenal (%)	Gland Pituitary (%)	Gland Prostate (%)	Thyroid/ Parathyroid (%)	Heart (%)	Kidney (%)
	-	-	-	-	-	-	-
4003	41.8454	2.2693	0.6683	34.9626	0.5786	61.2469	136.7581
4004	45.4889	3.2310	0.7936	55.1724	1.2045	71.3274	146.6226
4005	42.6501	2.5932	0.6832	31.8323	0.9627	61.2319	134.0062
4012	46.6126	2.4618	0.5534	37.7863	0.5105	61.0210	133.4924
4013	52.9330	3.9851	0.8706	47.7654	0.8287	72.2533	159.8231
Mean	45.90599	2.90808	0.71383	41.50379	0.81700	65.41607	142.14047
SD	4.39149	0.70188	0.12213	9.69773	0.28388	5.82874	11.21038
N	5	5	5	5	5	5	5
%Diff	-0.60740	2.42304	21.65945	-8.85999	7.37786	8.12049	10.69166

Appendix 18

Appendix 3

Individual Organ Weights Relative to Brain Weight: Day 29

5550008

Sex: Male Day(s) Relative to Start Date

3.3x 10E12 vg Group 4	Organ Weight/Brain Ratio			
	Liver (%)	Spleen (%)	Testis (%)	Thymus (%)
	-	-	-	-
4003	537.5062	35.2618	162.5935	18.6035
4004	764.5725	57.1564	174.8701	45.6306
4005	495.1863	38.5611	171.8427	25.7246
4012	520.4198	36.1164	162.6431	24.5706
4013	637.4302	34.5903	183.0540	24.0223
Mean	591.02302	40.33720	171.00068	27.71034
SD	111.02542	9.52155	8.68178	10.38596
N	5	5	5	5
%Diff	22.68896	-0.18497	3.78461	-0.33816

Appendix 18

Appendix 3

Individual Organ Weights Relative to Brain Weight: Day 29

5550008

Sex: Female Day(s) Relative to Start Date

0	Organ Weight/Brain Ratio						
vg<br>Group 1	Gland Adrenal (%)	Gland Pituitary (%)	Thyroid/ Parathyroid (%)	Heart (%)	Kidney (%)	Liver (%)	Ovary (%)
	-	-	-	-	-	-	-
1508	3.0274	0.4172	0.6794	41.1204	73.3015	288.5578	4.7080
1509	3.0372	0.6968	0.6277	41.5957	78.3511	328.6170	3.5106
1513	2.9958	0.6453	0.7240	43.9664	85.6768	283.2109	3.8300
1514	3.1607	0.8932	0.6078	39.2706	71.6173	310.0951	5.3911
1515	2.6689	0.8615	0.4786	36.7117	73.7050	284.5158	4.5608
Mean	2.97801	0.70280	0.62350	40.53297	76.53034	298.99933	4.40011
SD	0.18383	0.19129	0.09285	2.71379	5.69031	19.80150	0.74480
N	5	5	5	5	5	5	5

Appendix 18

Appendix 3

Individual Organ Weights Relative to Brain Weight: Day 29

5550008

Sex: Female Day(s) Relative to Start Date

0	Organ Weight/Brain Ratio		
vg<br>Group 1	Spleen<br>(%)	Thymus<br>(%)	Uterus/<br>Cervix<br>(%)
	-	-	-
1508	22.8248	17.6996	49.9404
1509	24.2021	17.5000	43.5638
1513	25.2886	23.8720	64.8478
1514	20.2960	17.6533	21.0359
1515	23.5923	16.0473	44.6509
Mean	23.24076	18.55444	44.80779
SD	1.87657	3.05027	15.76880
N	5	5	5

Appendix 18

Appendix 3

Individual Organ Weights Relative to Brain Weight: Day 29

5550008

Sex: Female Day(s) Relative to Start Date

0.36x 10E12 vg Group 2	Organ Weight/Brain Ratio						
	Gland Adrenal (%)	Gland Pituitary (%)	Thyroid/ Parathyroid (%)	Heart (%)	Kidney (%)	Liver (%)	Ovary (%)
	-	-	-	-	-	-	-
2501	3.0870	0.7442	0.5556	50.7338	91.1950	330.1887	4.7694
2502	3.6975	0.7266	0.6405	47.8471	87.7826	385.6835	5.5974
2507	3.2023	0.8543	0.7338	44.2872	75.5765	338.6268	4.7170
2508	3.2568	0.8800	0.5930	40.1243	86.8484	336.3462	5.3085
2509	3.1316	0.9474	0.5474	40.0526	81.2105	334.1053	5.4211
Mean	3.27505	0.83049	0.61403	44.60902	84.52259	344.99011	5.16266
SD	0.24498	0.09342	0.07638	4.71631	6.15547	22.96042	0.39697
N	5	5	5	5	5	5	5
%Diff	9.97432	18.16797	-1.51812	10.05612	10.44325	15.38157	17.33020

Appendix 18

Appendix 3

Individual Organ Weights Relative to Brain Weight: Day 29

5550008

Sex: Female Day(s) Relative to Start Date

0.36x 10E12 vg Group 2	Organ Weight/Brain Ratio		
	Spleen (%)	Thymus (%)	Uterus/ Cervix (%)
	-	-	-
2501	26.3627	19.9686	20.8595
2502	37.7287	20.7750	27.2874
2507	19.7065	23.7945	19.7065
2508	21.4252	25.1554	28.4553
2509	24.2632	17.5789	32.5789
Mean	25.89725	21.45450	25.77754
SD	7.09218	3.03572	5.40263
N	5	5	5
%Diff	11.43029	15.63001	-42.47086

Appendix 18

Appendix 3

Individual Organ Weights Relative to Brain Weight: Day 29

5550008

Sex: Female Day(s) Relative to Start Date

1.1x 10E12 vg Group 3	Organ Weight/Brain Ratio						
	Gland Adrenal (%)	Gland Pituitary (%)	Thyroid/ Parathyroid (%)	Heart (%)	Kidney (%)	Liver (%)	Ovary (%)
	-	-	-	-	-	-	-
3504	3.3019	0.8047	0.8657	43.8957	88.4018	369.9223	6.2708
3505	2.9424	0.8459	0.6227	37.7789	62.5324	282.7193	3.8402
3506	2.4675	0.7792	0.5662	42.5974	71.3766	335.6364	4.2597
3514	3.4211	0.7447	0.8511	41.6013	80.2912	327.7156	5.7671
3615	4.0526	0.7895	0.5632	44.4737	92.6842	350.9474	9.0526
Mean	3.23710	0.79278	0.69378	42.06941	79.05724	333.38817	5.83809
SD	0.58784	0.03697	0.15221	2.64678	12.31461	32.58696	2.06207
N	5	5	5	5	5	5	5
%Diff	8.70014	12.80264	11.27170	3.79057	3.30182	11.50131	32.68034

Appendix 18

Appendix 3

Individual Organ Weights Relative to Brain Weight: Day 29

5550008

Sex: Female Day(s) Relative to Start Date

1.1x 10E12 vg Group 3	Organ Weight/Brain Ratio		
	Spleen (%)	Thymus (%)	Uterus/ Cervix (%)
	-	-	-
3504	26.8036	30.6881	33.2963
3505	20.1868	15.7758	24.6497
3506	20.2078	16.5195	33.0909
3514	23.8522	16.7973	27.1557
3615	32.3684	27.2632	27.7368
Mean	24.68375	21.40878	29.18589
SD	5.11116	7.02284	3.83873
N	5	5	5
%Diff	6.20888	15.38359	-34.86424

Appendix 18

Appendix 3

Individual Organ Weights Relative to Brain Weight: Day 29

5550008

Sex: Female Day(s) Relative to Start Date

3.3x 10E12 vg Group 4	Organ Weight/Brain Ratio						
	Gland Adrenal (%)	Gland Pituitary (%)	Thyroid/ Parathyroid (%)	Heart (%)	Kidney (%)	Liver (%)	Ovary (%)
	-	-	-	-	-	-	-
4505	2.7528	0.8884	0.7715	46.0549	86.3238	345.2367	4.5587
4506	3.3562	0.8377	0.6322	47.4710	89.1992	372.9189	6.5859
4507	3.0744	0.8375	0.6832	40.2204	78.1267	319.0634	4.3526
4511	3.9074	0.7084	0.4251	47.4659	82.2343	323.3787	4.7411
4512	2.9393	0.9393	0.6174	49.1293	86.3852	374.9340	5.4354
Mean	3.20600	0.84226	0.62588	46.06831	84.45384	347.10634	5.13475
SD	0.44953	0.08587	0.12740	3.44561	4.32156	26.42712	0.90755
N	5	5	5	5	5	5	5
%Diff	7.65576	19.84324	0.38191	13.65639	10.35341	16.08934	16.69579

Appendix 18

Appendix 3

Individual Organ Weights Relative to Brain Weight: Day 29

5550008

Sex: Female Day(s) Relative to Start Date

3.3x 10E12 vg Group 4	Organ Weight/Brain Ratio		
	Spleen (%)	Thymus (%)	Uterus/ Cervix (%)
	-	-	-
4505	24.0795	14.3191	24.6639
4506	29.3994	33.2455	21.8651
4507	21.3774	17.4656	28.6501
4511	23.3243	23.5967	22.1253
4512	28.8654	22.9024	20.8971
Mean	25.40919	22.30586	23.64033
SD	3.54392	7.22695	3.12721
N	5	5	5
%Diff	9.33028	20.21845	-47.24058

Appendix 18

Appendix 3

Individual Organ Weights Relative to Brain Weight: Day 91

5550008

Sex: Male Day(s) Relative to Start Date

0	Organ Weight/Brain Ratio						
vg<br><br>Group 1	Epididymis<br><br>(%)	Gland<br>Adrenal<br>(%)	Gland<br>Pituitary<br>(%)	Gland<br>Prostate<br>(%)	Thyroid/<br>Parathyroid<br>(%)	Heart<br><br>(%)	Kidney<br><br>(%)
	-	-	-	-	-	-	-
1005	63.9471	2.0191	0.6153	58.7511	0.6700	73.3820	125.1139
1006	61.4545	2.1545	0.7000	74.5455	0.9136	73.6818	141.1818
1007	68.9832	3.0131	1.0215	79.7575	1.3060	100.6530	177.4720
1011	68.5093	1.8894	0.8156	63.2533	1.2778	70.7748	157.2270
1012	60.1327	1.8186	0.7257	63.1416	0.9027	78.5841	143.7168
Mean	64.60538	2.17896	0.77560	67.88979	1.01400	79.41513	148.94232
SD	4.02403	0.48362	0.15487	8.84162	0.27185	12.20310	19.60805
N	5	5	5	5	5	5	5

Appendix 18

Appendix 3

Individual Organ Weights Relative to Brain Weight: Day 91

5550008

Sex: Male Day(s) Relative to Start Date

0<br>vg<br>Group 1	Organ Weight/Brain Ratio<br>Liver<br>(%)<br>-	 Spleen<br>(%)<br>-	 Testis<br>(%)<br>-	 Thymus<br>(%)<br>-
1005	596.9462	37.2379	190.6108	9.7083
1006	571.8636	43.8182	164.3182	11.5000
1007	1055.8769	45.0093	194.4030	16.6045
1011	613.8197	46.9869	176.9823	11.7807
1012	634.6018	40.6637	154.5575	12.7434
Mean	694.62163	42.74320	176.17435	12.46737
SD	203.25274	3.83836	16.94994	2.55987
N	5	5	5	5

Appendix 18

Appendix 3

Individual Organ Weights Relative to Brain Weight: Day 91

5550008

Sex: Male Day(s) Relative to Start Date

0.36x 10E12 vg Group 2	Organ Weight/Brain Ratio						
	Epididymis (%)	Gland Adrenal (%)	Gland Pituitary (%)	Gland Prostate (%)	Thyroid/ Parathyroid (%)	Heart (%)	Kidney (%)
	-	-	-	-	-	-	-
2103	62.0359	2.2612	0.5215	56.4456	1.0722	69.9625	130.1210
2005	66.5577	2.7417	0.8127	69.7338	1.1116	75.1051	131.6207
2006	66.9203	2.1307	0.6535	64.9955	0.7565	83.2140	147.4933
2007	64.9333	2.6737	0.8882	77.1744	0.9802	81.2701	154.9011
2008	38.1683	3.0806	0.6880	63.2778	0.6748	77.5635	144.4347
Mean	59.72309	2.57759	0.71278	66.32542	0.91907	77.42304	141.71415
SD	12.20263	0.38375	0.14271	7.71339	0.19387	5.22943	10.61806
N	5	5	5	5	5	5	5
%Diff	-7.55711	18.29460	-8.10023	-2.30428	-9.36230	-2.50845	-4.85300

Appendix 18

Appendix 3

Individual Organ Weights Relative to Brain Weight: Day 91

5550008

Sex: Male Day(s) Relative to Start Date

0.36x 10E12 vg Group 2	Organ Weight/Brain Ratio			
	Liver (%)	Spleen (%)	Testis (%)	Thymus (%)
	-	-	-	-
2103	559.3659	43.6379	162.2862	10.8052
2005	665.1098	43.2041	195.6095	16.6745
2006	671.3966	34.2883	173.8138	11.1459
2007	708.6977	51.1275	181.2241	13.0695
2008	593.2077	35.5828	130.5434	11.5688
Mean	639.55552	41.56811	168.69540	12.65277
SD	61.29031	6.84035	24.51628	2.40867
N	5	5	5	5
%Diff	-7.92750	-2.74919	-4.24520	1.48709

Appendix 18

Appendix 3

Individual Organ Weights Relative to Brain Weight: Day 91

5550008

Sex: Male Day(s) Relative to Start Date

1.1x 10E12 vg Group 3	Organ Weight/Brain Ratio						
	Epididymis (%)	Gland Adrenal (%)	Gland Pituitary (%)	Gland Prostate (%)	Thyroid/ Parathyroid (%)	Heart (%)	Kidney (%)
	-	-	-	-	-	-	-
3006	60.1615	2.5303	0.6505	45.4015	0.7672	77.6581	129.6994
3007	59.8710	2.5505	0.7699	80.6452	0.9032	87.8280	141.3333
3008	55.3793	1.9288	0.6215	48.4355	0.7158	63.3090	119.5456
3014	62.4941	2.3240	0.6471	60.4157	0.8314	66.7926	127.8224
3015	58.9422	1.8915	0.8068	64.5899	0.9996	74.6302	150.6948
Mean	59.36962	2.24505	0.69918	59.89755	0.84342	74.04360	133.81911
SD	2.58582	0.31854	0.08321	14.09134	0.11202	9.63360	12.22615
N	5	5	5	5	5	5	5
%Diff	-8.10422	3.03324	-9.85398	-11.77238	-16.82248	-6.76386	-10.15374

Appendix 18

Appendix 3

Individual Organ Weights Relative to Brain Weight: Day 91

5550008

Sex: Male Day(s) Relative to Start Date

1.1x 10E12 vg Group 3	Organ Weight/Brain Ratio			
	Liver (%)	Spleen (%)	Testis (%)	Thymus (%)
	-	-	-	-
3006	559.7129	40.9601	163.8403	22.3867
3007	697.6774	40.1720	176.3011	12.6452
3008	537.3768	33.5619	148.2212	13.8448
3014	514.3127	34.7189	165.0449	11.9509
3015	652.1739	36.1721	176.8265	13.3572
Mean	592.25074	37.11702	166.04679	14.83697
SD	78.81906	3.29334	11.67239	4.28095
N	5	5	5	5
%Diff	-14.73765	-13.16275	-5.74860	19.00641

Appendix 18

Appendix 3

Individual Organ Weights Relative to Brain Weight: Day 91

5550008

Sex: Male Day(s) Relative to Start Date

3.3x 10E12 vg Group 4	Organ Weight/Brain Ratio						
	Epididymis (%)	Gland Adrenal (%)	Gland Pituitary (%)	Gland Prostate (%)	Thyroid/ Parathyroid (%)	Heart (%)	Kidney (%)
	-	-	-	-	-	-	-
4009	38.4173	2.5553	0.6012	81.6534	0.7515	66.6667	155.3050
4010	57.5687	2.0680	0.6986	56.7303	0.6474	63.5305	111.1318
4011	65.2957	1.9732	0.7163	78.5582	1.1460	74.8152	133.9187
4014	58.5876	2.3365	0.4621	60.8544	0.7280	60.5929	119.4856
4015	46.4789	2.7987	0.6951	74.1027	0.8451	76.8287	137.0286
Mean	53.26965	2.34634	0.63467	70.37981	0.82361	68.48678	131.37395
SD	10.70159	0.34099	0.10644	11.01002	0.19352	7.06802	17.04571
N	5	5	5	5	5	5	5
%Diff	-17.54611	7.68206	-18.17044	3.66774	-18.77661	-13.76104	-11.79542

Appendix 18

Appendix 3

Individual Organ Weights Relative to Brain Weight: Day 91

5550008

Sex: Male Day(s) Relative to Start Date

3.3x 10E12 vg Group 4	Organ Weight/Brain Ratio			
	Liver (%)	Spleen (%)	Testis (%)	Thymus (%)
	-	-	-	-
4009	612.8647	35.9416	102.2546	9.2396
4010	452.3521	31.5324	169.8184	9.6414
4011	572.5970	32.9482	170.1941	18.9926
4014	485.2659	38.1866	172.1883	12.0314
4015	566.3335	52.5216	110.9041	11.9037
Mean	537.88266	38.22608	145.07191	12.36173
SD	66.55983	8.40004	35.28302	3.91904
N	5	5	5	5
%Diff	-22.56466	-10.56804	-17.65436	-0.84732

Appendix 18

Appendix 3

Individual Organ Weights Relative to Brain Weight: Day 91

5550008

Sex: Female Day(s) Relative to Start Date

0	Organ Weight/Brain Ratio						
vg<br><br>Group 1	Gland<br>Adrenal<br>(%)	Gland<br>Pituitary<br>(%)	Thyroid/<br>Parathyroid<br>(%)	Heart<br><br>(%)	Kidney<br><br>(%)	Liver<br><br>(%)	Ovary<br><br>(%)
	-	-	-	-	-	-	-
1503	3.6584	0.7715	0.6016	44.5900	85.9292	311.9360	3.9787
1504	3.1520	0.9812	0.5897	44.2298	81.2913	346.8734	4.0671
1510	4.1325	1.1275	0.6404	45.8128	85.9332	328.6809	4.4882
1511	3.2033	0.9827	0.8382	52.6012	90.7996	353.3719	4.4798
1512	2.8557	0.7088	0.6119	44.3141	71.3921	281.1831	3.6206
Mean	3.40037	0.91433	0.65637	46.30958	83.06909	324.40904	4.12687
SD	0.49994	0.17120	0.10333	3.57404	7.34261	29.09383	0.36639
N	5	5	5	5	5	5	5

Appendix 18

Appendix 3

Individual Organ Weights Relative to Brain Weight: Day 91

5550008

Sex: Female Day(s) Relative to Start Date

0	Organ Weight/Brain Ratio		
vg	Spleen	Thymus	Uterus/ Cervix
Group 1	(%)	(%)	(%)
	-	-	-
1503	28.8695	9.1218	23.8234
1504	27.4530	10.0661	29.0290
1510	31.1440	13.5742	43.0761
1511	31.6474	13.7283	25.1927
1512	23.3554	12.6466	26.7721
Mean	28.49385	11.82739	29.57864
SD	3.33900	2.10703	7.78973
N	5	5	5

Appendix 18

Appendix 3

Individual Organ Weights Relative to Brain Weight: Day 91

5550008

Sex: Female Day(s) Relative to Start Date

0.36x 10E12 vg Group 2	Organ Weight/Brain Ratio						
	Gland Adrenal (%)	Gland Pituitary (%)	Thyroid/ Parathyroid (%)	Heart (%)	Kidney (%)	Liver (%)	Ovary (%)
	-	-	-	-	-	-	-
2503	2.9120	0.9314	0.4708	44.0635	89.5599	347.1853	4.8106
2504	3.0566	0.7666	0.9521	48.3887	103.5156	404.0527	3.5156
2614	3.1492	1.1271	0.9282	52.7624	90.3867	345.3039	5.1934
2615	3.7676	1.1318	0.6137	47.9376	87.9779	364.6881	4.6278
Mean	3.22135	0.98922	0.74121	48.28805	92.86003	365.30750	4.53685
SD	0.37703	0.17534	0.23722	3.55903	7.17369	27.26498	0.72045
N	4	4	4	4	4	4	4
%Diff	-5.26475	8.19036	12.92525	4.27227	11.78650	12.60706	9.93439

Appendix 18

Appendix 3

Individual Organ Weights Relative to Brain Weight: Day 91

5550008

Sex: Female Day(s) Relative to Start Date

0.36x 10E12 vg Group 2	Organ Weight/Brain Ratio		
	Spleen (%)	Thymus (%)	Uterus/ Cervix (%)
	-	-	-
2503	26.6633	8.1883	29.2221
2504	22.2656	8.5449	28.6133
2614	28.1768	9.8343	25.8564
2615	25.3018	9.1046	55.0805
Mean	25.60187	8.91803	34.69306
SD	2.51511	0.71786	13.67028
N	4	4	4
%Diff	-10.14947	-24.59849	17.29093

Appendix 18

Appendix 3

Individual Organ Weights Relative to Brain Weight: Day 91

5550008

Sex: Female Day(s) Relative to Start Date

1.1x 10E12 vg Group 3	Organ Weight/Brain Ratio						
	Gland Adrenal (%)	Gland Pituitary (%)	Thyroid/ Parathyroid (%)	Heart (%)	Kidney (%)	Liver (%)	Ovary (%)
	-	-	-	-	-	-	-
3501	3.9117	1.2577	0.8008	48.8706	86.1396	335.9856	4.3634
3502	2.5951	0.8793	0.5608	60.6939	91.6350	389.5913	5.9411
3503	2.7945	0.9871	0.6819	41.2017	65.7606	284.1679	2.6705
3509	2.3992	0.9980	0.6047	41.8387	71.2389	316.2242	3.4415
3510	2.5026	0.7925	0.7299	51.9812	97.4453	388.9990	4.4838
Mean	2.84061	0.98293	0.67565	48.91725	82.44388	342.99358	4.18007
SD	0.61619	0.17518	0.09600	8.02675	13.48201	46.13571	1.22998
N	5	5	5	5	5	5	5
%Diff	-16.46172	7.50175	2.93657	5.63095	-0.75264	5.72874	1.28895

Appendix 18

Appendix 3

Individual Organ Weights Relative to Brain Weight: Day 91

5550008

Sex: Female Day(s) Relative to Start Date

1.1x 10E12 vg Group 3	Organ Weight/Brain Ratio		
	Spleen (%)	Thymus (%)	Uterus/ Cervix (%)
	-	-	-
3501	25.2053	8.5729	136.9097
3502	27.0913	11.4544	28.1844
3503	19.2179	9.2036	34.4301
3509	23.4513	11.1111	73.1563
3510	29.7185	15.4849	23.2013
Mean	24.93686	11.16538	59.17636
SD	3.95253	2.70712	47.71003
N	5	5	5
%Diff	-12.48335	-5.59733	100.06453

Appendix 18

Appendix 3

Individual Organ Weights Relative to Brain Weight: Day 91

5550008

Sex: Female Day(s) Relative to Start Date

3.3x 10E12 vg Group 4	Organ Weight/Brain Ratio						
	Gland Adrenal (%)	Gland Pituitary (%)	Thyroid/ Parathyroid (%)	Heart (%)	Kidney (%)	Liver (%)	Ovary (%)
	-	-	-	-	-	-	-
4503	3.4473	1.2036	0.3616	52.7984	74.2942	336.7509	3.1699
4504	2.8168	0.8147	0.5059	43.2907	97.3908	314.1640	4.4196
4513	3.0838	1.1403	0.6247	48.9341	87.6549	317.3029	3.6192
4514	2.5316	0.9842	0.7105	51.3684	89.2632	384.1053	3.4211
4515	3.0142	1.5045	0.7680	49.2372	85.1131	383.6928	2.6302
Mean	2.97873	1.12945	0.59413	49.12577	86.74325	347.20317	3.45199
SD	0.33837	0.25808	0.16325	3.62680	8.33949	34.59812	0.65577
N	5	5	5	5	5	5	5
%Diff	-12.39982	23.52705	-9.48248	6.08124	4.42301	7.02635	-16.35328

Appendix 18

Appendix 3

Individual Organ Weights Relative to Brain Weight: Day 91

5550008

Sex: Female Day(s) Relative to Start Date

3.3x 10E12 vg Group 4	Organ Weight/Brain Ratio		
	Spleen (%)	Thymus (%)	Uterus/ Cervix (%)
	-	-	-
4503	24.1704	13.4720	32.9371
4504	25.8786	12.1938	66.3472
4513	30.6396	11.9980	38.6713
4514	27.4211	8.5789	57.4737
4515	21.5676	9.5213	33.3509
Mean	25.93544	11.15282	45.75602
SD	3.41074	2.02875	15.24553
N	5	5	5
%Diff	-8.97881	-5.70348	54.69281

Appendix 18

Appendix 4

Individual Macroscopic and Microscopic Pathology:

5550008

Animal:	1001	Group:	1	Sex:	Male
Species:	Rat	Strain:	Sprague Dawley		
		Dose:	0 vg		
		Removal Reason:	Terminal Euthanasia		
		Study Day (Week) of Death:	8 (2)		

Gross Pathology Animal Details:

No animal details found

Gross Pathology Observations [Correlation]:

LIVER : Focus, pale : (Comment) 1 fissure right medial (TGL)

Any remaining study plan required tissues, which have been examined, have no visible lesions

Gross Pathology - The following Tissues were Not Examined:

None

Histopathology Animal Details:

No animal details found

Histopathology Observations [Correlation]:

LIVER : Tension lipidosis; mild [LIVER : Focus, pale : (Comment) 1 fissure right medial (G)]
LIVER : Infiltration, mixed cell; minimal
SITE, INJECTION : Degeneration; axonal, minimal, cauda equina

Histopathology - The following Tissues were Within Normal Limits:

BRAIN; EPIDIDYMIS; EYE; GANGLION, DORSAL ROOT, CERVICAL; GANGLION, DORSAL ROOT, LUMBAR; GANGLION, DORSAL ROOT, THORACIC; GLAND, ADRENAL; GLAND, PARATHYROID; GLAND, PITUITARY; GLAND, PROSTATE; GLAND, THYROID; HEART; KIDNEY; LUNG; LYMPH NODE, CERVICAL; LYMPH NODE, ILIAC; LYMPH NODE, MANDIBULAR; LYMPH NODE, MESENTERIC; MUSCLE, BICEPS FEMORIS; MUSCLE, GASTROCNEMIUS; NERVE, OPTIC; NERVE, SCIATIC; NERVE, TIBIAL; SPINAL CORD, CERVICAL; SPINAL CORD, LUMBAR; SPINAL CORD, THORACIC; SPLEEN; TESTIS; THYMUS; NERVE ROOT, VENTRAL, CERVICAL; NERVE ROOT, VENTRAL, THORACIC; NERVE ROOT, VENTRAL, LUMBAR; NERVE ROOT, DORSAL, CERVICAL; NERVE ROOT, DORSAL, LUMBAR; NERVE ROOT, DORSAL, THORACIC

Histopathology - The following Tissues were Not Examined:

None

Appendix 18

Appendix 4

Individual Macroscopic and Microscopic Pathology:

5550008

Animal: 1002	Group: 1	Sex: Male
Species: Rat	Strain: Sprague Dawley<br>Dose: 0 vg<br>Removal Reason: Terminal Euthanasia<br>Study Day (Week) of Death: 8 (2)	

Gross Pathology Animal Details:

No animal details found

Gross Pathology Observations [Correlation]:

No observations found

Any remaining study plan required tissues, which have been examined, have no visible lesions

Gross Pathology - The following Tissues were Not Examined:

None

Histopathology Animal Details:

No animal details found

Histopathology Observations [Correlation]:

EPIDIDYMIS : Infiltration, mononuclear cell; minimal
HEART : Infiltration, mononuclear cell/degeneration, myofiber; minimal
KIDNEY : Infiltration, mononuclear cell; minimal
LIVER : Infiltration, mixed cell; minimal
SITE, INJECTION : Degeneration; axonal, minimal, cauda equina
NERVE ROOT, DORSAL, LUMBAR : Degeneration; axonal, minimal

Histopathology - The following Tissues were Within Normal Limits:

BRAIN; EYE; GANGLION, DORSAL ROOT, CERVICAL; GANGLION, DORSAL ROOT, LUMBAR; GANGLION, DORSAL ROOT, THORACIC; GLAND, ADRENAL; GLAND, PARATHYROID; GLAND, PITUITARY; GLAND, PROSTATE; GLAND, THYROID; LUNG; LYMPH NODE, CERVICAL; LYMPH NODE, ILIAC; LYMPH NODE, MANDIBULAR; LYMPH NODE, MESENTERIC; MUSCLE, BICEPS FEMORIS; MUSCLE, GASTROCNEMIUS; NERVE, SCIATIC; NERVE, TIBIAL; SPINAL CORD, CERVICAL; SPINAL CORD, LUMBAR; SPINAL CORD, THORACIC; SPLEEN; TESTIS; THYMUS; NERVE ROOT, VENTRAL, CERVICAL; NERVE ROOT, VENTRAL, THORACIC; NERVE ROOT, VENTRAL, LUMBAR; NERVE ROOT, DORSAL, CERVICAL; NERVE ROOT, DORSAL, THORACIC

Histopathology - The following Tissues were Not Examined:

NERVE, OPTIC - Not Present In Section.

Appendix 18

Appendix 4

Individual Macroscopic and Microscopic Pathology:

5550008

Animal:	1003	Group:	1	Sex:	Male
Species:	Rat	Strain:	Sprague Dawley		
		Dose:	0 vg		
		Removal Reason: Recovery Euthanasia			
		Study Day (Week) of Death: 29 (5)			

Gross Pathology Animal Details:

No animal details found

Gross Pathology Observations [Correlation]:

LIVER : Focus, pale : (Comment) 1, fissure, right medial (TGL)

Any remaining study plan required tissues, which have been examined, have no visible lesions

Gross Pathology - The following Tissues were Not Examined:

None

Histopathology Animal Details:

No animal details found

Histopathology Observations [Correlation]:

EPIDIDYMIS : Infiltration, mononuclear cell; minimal
KIDNEY : Infiltration, mononuclear cell; minimal
KIDNEY : Chronic progressive nephropathy; minimal
LIVER : Vacuolation; hepatocellular, periportal, minimal
LIVER : Tension lipidosis; minimal [LIVER : Focus, pale : (Comment) 1, fissure, right medial (G)]
LIVER : Infiltration, mixed cell; minimal
LIVER : Infiltration, mononuclear cell; periportal, minimal

Histopathology - The following Tissues were Within Normal Limits:

BRAIN; EYE; GANGLION, DORSAL ROOT, CERVICAL; GANGLION, DORSAL ROOT, LUMBAR; GANGLION, DORSAL ROOT, THORACIC; GLAND, ADRENAL; GLAND, PARATHYROID; GLAND, PITUITARY; GLAND, PROSTATE; GLAND, THYROID; HEART; LUNG; LYMPH NODE, CERVICAL; LYMPH NODE, ILIAC; LYMPH NODE, MANDIBULAR; LYMPH NODE, MESENTERIC; MUSCLE, BICEPS FEMORIS; MUSCLE, GASTROCNEMIUS; NERVE, OPTIC; NERVE, SCIATIC; NERVE, TIBIAL; SITE, INJECTION; SPINAL CORD, CERVICAL; SPINAL CORD, LUMBAR; SPINAL CORD, THORACIC; SPLEEN; TESTIS; THYMUS; NERVE ROOT, VENTRAL, CERVICAL; NERVE ROOT, VENTRAL, THORACIC; NERVE ROOT, VENTRAL, LUMBAR; NERVE ROOT, DORSAL, CERVICAL; NERVE ROOT, DORSAL, LUMBAR; NERVE ROOT, DORSAL, THORACIC

Histopathology - The following Tissues were Not Examined:

None

Individual Macroscopic and Microscopic Pathology:

5550008

Animal:	1004	Group:	1	Sex:	Male
Species:	Rat	Strain:	Sprague Dawley		
		Dose:	0 vg		
		Removal Reason:	Recovery Euthanasia		
		Study Day (Week) of Death:	29 (5)		

Gross Pathology Animal Details:

No animal details found

Gross Pathology Observations [Correlation]:

No observations found

Any remaining study plan required tissues, which have been examined, have no visible lesions

Gross Pathology - The following Tissues were Not Examined:

None

Histopathology Animal Details:

No animal details found

Histopathology Observations [Correlation]:

EPIDIDYMIS : Infiltration, mononuclear cell; minimal
GLAND, ADRENAL : (Note) Only Cortex And One Medulla Available For Evaluation.
GLAND, PITUITARY : (Note) Only Pars Distalis Available For Evaluation.
KIDNEY : Infiltration, mononuclear cell; minimal
KIDNEY : Chronic progressive nephropathy; minimal
LIVER : Infiltration, mixed cell; minimal

Histopathology - The following Tissues were Within Normal Limits:

BRAIN; EYE; GANGLION, DORSAL ROOT, CERVICAL; GANGLION, DORSAL ROOT, LUMBAR; GANGLION, DORSAL ROOT, THORACIC; GLAND, ADRENAL; GLAND, PARATHYROID; GLAND, PITUITARY; GLAND, PROSTATE; GLAND, THYROID; HEART; LUNG; LYMPH NODE, CERVICAL; LYMPH NODE, ILIAC; LYMPH NODE, MESENTERIC; MUSCLE, BICEPS FEMORIS; MUSCLE, GASTROCNEMIUS; NERVE, OPTIC; NERVE, SCIATIC; NERVE, TIBIAL; SITE, INJECTION; SPINAL CORD, CERVICAL; SPINAL CORD, LUMBAR; SPINAL CORD, THORACIC; SPLEEN; TESTIS; THYMUS; NERVE ROOT, VENTRAL, CERVICAL; NERVE ROOT, VENTRAL, THORACIC; NERVE ROOT, VENTRAL, LUMBAR; NERVE ROOT, DORSAL, CERVICAL; NERVE ROOT, DORSAL, LUMBAR; NERVE ROOT, DORSAL, THORACIC

Histopathology - The following Tissues were Not Examined:

LYMPH NODE, MANDIBULAR - Not Present In Section.

Appendix 18

Appendix 4

Individual Macroscopic and Microscopic Pathology:

5550008

Animal:	1005	Group:	1	Sex:	Male
Species:	Rat	Strain:	Sprague Dawley		
		Dose:	0 vg		
		Removal Reason:	Recovery Euthanasia		
		Study Day (Week) of Death:	91 (13)		

Gross Pathology Animal Details:

No animal details found

Gross Pathology Observations [Correlation]:

LYMPH NODE, ILIAC : Discoloration, dark : (Comment) left. (TGL)

Any remaining study plan required tissues, which have been examined, have no visible lesions

Gross Pathology - The following Tissues were Not Examined:

None

Histopathology Animal Details:

No animal details found

Histopathology Observations [Correlation]:

GLAND, PROSTATE : Infiltration, mononuclear cell; minimal
HEART : Infiltration, mononuclear cell/degeneration, myofiber; minimal
KIDNEY : Infiltration, mononuclear cell; minimal
LIVER : Infiltration, mixed cell; minimal
LIVER : Infiltration, mononuclear cell; periportal, minimal
LYMPH NODE, ILIAC : Hemorrhage; acute, mild : (Comment) with some hemosiderophages [LYMPH NODE, ILIAC : Discoloration, dark : (Comment) left. (G)]

Histopathology - The following Tissues were Within Normal Limits:

BRAIN; EPIDIDYMIS; EYE; GANGLION, DORSAL ROOT, CERVICAL; GANGLION, DORSAL ROOT, LUMBAR; GANGLION, DORSAL ROOT, THORACIC; GLAND, ADRENAL; GLAND, PARATHYROID; GLAND, PITUITARY; GLAND, THYROID; LUNG; LYMPH NODE, CERVICAL; LYMPH NODE, MANDIBULAR; LYMPH NODE, MESENTERIC; MUSCLE, BICEPS FEMORIS; MUSCLE, GASTROCNEMIUS; NERVE, OPTIC; NERVE, SCIATIC; NERVE, TIBIAL; SITE, INJECTION; SPINAL CORD, CERVICAL; SPINAL CORD, LUMBAR; SPINAL CORD, THORACIC; SPLEEN; TESTIS; THYMUS; NERVE ROOT, VENTRAL, CERVICAL; NERVE ROOT, VENTRAL, THORACIC; NERVE ROOT, VENTRAL, LUMBAR; NERVE ROOT, DORSAL, CERVICAL; NERVE ROOT, DORSAL, LUMBAR; NERVE ROOT, DORSAL, THORACIC

Histopathology - The following Tissues were Not Examined:

None

Individual Macroscopic and Microscopic Pathology:

5550008

Animal: 1006	Group: 1	Sex: Male
Species: Rat	Strain: Sprague Dawley	
	Dose: 0 vg	
	Removal Reason: Recovery Euthanasia	
	Study Day (Week) of Death: 91 (13)	

Gross Pathology Animal Details:

No animal details found

Gross Pathology Observations [Correlation]:

GENERAL OBSERVATIONS : (Comment) ID chip found in abdominal cavity, adjacent to kidney left
LIVER : Focus, pale : (Comment) 2, fissure, left medial (TGL)
LUNG : Focus, dark : (Comment) 3 right middle (TGL)
THYMUS : Focus, dark : (Comment) 1, right (TGL)

Any remaining study plan required tissues, which have been examined, have no visible lesions

Gross Pathology - The following Tissues were Not Examined:

None

Histopathology Animal Details:

No animal details found

Histopathology Observations [Correlation]:

GLAND, ADRENAL : Vacuolation; cortical, mild
GLAND, PITUITARY : (Note) Only Pars Distalis Available For Evaluation.
GLAND, PROSTATE : Infiltration, mononuclear cell; minimal
HEART : Infiltration, mononuclear cell/degeneration, myofiber; minimal : (Comment) with some pigmented macrophages
KIDNEY : Chronic progressive nephropathy; minimal
LIVER : Tension lipidosis; minimal [LIVER : Focus, pale : (Comment) 2, fissure, left medial (G)]
LIVER : Infiltration, mononuclear cell; minimal
LUNG : Hemorrhage; acute, minimal [LUNG : Focus, dark : (Comment) 3 right middle (G)]
NO CORRELATE : No correlating lesion [THYMUS : Focus, dark : (Comment) 1, right (G)]

Histopathology - The following Tissues were Within Normal Limits:

BRAIN; EPIDIDYMIS; EYE; GANGLION, DORSAL ROOT, CERVICAL; GANGLION, DORSAL ROOT, LUMBAR; GANGLION, DORSAL ROOT, THORACIC; GLAND, PITUITARY; GLAND, THYROID; LYMPH NODE, CERVICAL; LYMPH NODE, ILIAC; LYMPH NODE, MANDIBULAR; LYMPH NODE, MESENTERIC; MUSCLE, BICEPS FEMORIS; MUSCLE, GASTROCNEMIUS; NERVE, OPTIC; NERVE, SCIATIC; NERVE, TIBIAL; SITE, INJECTION; SPINAL CORD, CERVICAL; SPINAL CORD, LUMBAR; SPINAL CORD, THORACIC; SPLEEN; TESTIS; THYMUS; NERVE ROOT, VENTRAL, CERVICAL; NERVE ROOT, VENTRAL, THORACIC; NERVE ROOT, VENTRAL, LUMBAR; NERVE ROOT, DORSAL, CERVICAL; NERVE ROOT, DORSAL, LUMBAR; NERVE ROOT, DORSAL, THORACIC

Histopathology - The following Tissues were Not Examined:

GLAND, PARATHYROID - Not Present In Section.

Appendix 18

Appendix 4

Individual Macroscopic and Microscopic Pathology:

5550008

Animal:	1007	Group:	1	Sex:	Male
Species:	Rat	Strain:	Sprague Dawley		
		Dose:	0 vg		
		Removal Reason: Recovery Euthanasia			
		Study Day (Week) of Death: 91 (13)			

Gross Pathology Animal Details:

No animal details found

Gross Pathology Observations [Correlation]:

LIVER : Focus, pale : (Comment) 1, fissure, right medial. (TGL)

Any remaining study plan required tissues, which have been examined, have no visible lesions

Gross Pathology - The following Tissues were Not Examined:

None

Histopathology Animal Details:

No animal details found

Histopathology Observations [Correlation]:

GLAND, ADRENAL : Vacuolation; cortical, minimal
GLAND, PITUITARY : (Note) Only Pars Distalis Available For Evaluation.
GLAND, PROSTATE : Infiltration, mononuclear cell; minimal
HEART : Infiltration, mononuclear cell/degeneration, myofiber; minimal
KIDNEY : Chronic progressive nephropathy; minimal
LIVER : Infiltration, mixed cell; minimal
SITE, INJECTION : Infiltration, mononuclear cell; minimal, leptomeninges
NO CORRELATE : No correlating lesion [LIVER : Focus, pale : (Comment) 1, fissure, right medial. (G)]

Histopathology - The following Tissues were Within Normal Limits:

BRAIN; EPIDIDYMIS; EYE; GANGLION, DORSAL ROOT, CERVICAL; GANGLION, DORSAL ROOT, LUMBAR; GANGLION, DORSAL ROOT, THORACIC; GLAND, PARATHYROID; GLAND, PITUITARY; GLAND, THYROID; LUNG; LYMPH NODE, CERVICAL; LYMPH NODE, ILIAC; LYMPH NODE, MANDIBULAR; LYMPH NODE, MESENTERIC; MUSCLE, BICEPS FEMORIS; MUSCLE, GASTROCNEMIUS; NERVE, OPTIC; NERVE, SCIATIC; NERVE, TIBIAL; SPINAL CORD, CERVICAL; SPINAL CORD, LUMBAR; SPINAL CORD, THORACIC; SPLEEN; TESTIS; THYMUS; NERVE ROOT, VENTRAL, CERVICAL; NERVE ROOT, VENTRAL, THORACIC; NERVE ROOT, VENTRAL, LUMBAR; NERVE ROOT, DORSAL, CERVICAL; NERVE ROOT, DORSAL, LUMBAR; NERVE ROOT, DORSAL, THORACIC

Histopathology - The following Tissues were Not Examined:

None

Appendix 18

Appendix 4

Individual Macroscopic and Microscopic Pathology:

5550008

Animal:	1008	Group:	1	Sex:	Male
Species:	Rat	Strain:	Sprague Dawley		
		Dose:	0 vg		
		Removal Reason:	Recovery Euthanasia		
		Study Day (Week) of Death:	29 (5)		

Gross Pathology Animal Details:

No animal details found

Gross Pathology Observations [Correlation]:

No observations found

Any remaining study plan required tissues, which have been examined, have no visible lesions

Gross Pathology - The following Tissues were Not Examined:

None

Histopathology Animal Details:

No animal details found

Histopathology Observations [Correlation]:

GLAND, PARATHYROID : (Note) One Of A Pair Available For Evaluation.
KIDNEY : Dilatation; tubular, minimal : (Comment) filled with hyaline material
LIVER : Vacuolation; hepatocellular, periportal, minimal
LIVER : Infiltration, mixed cell; minimal

Histopathology - The following Tissues were Within Normal Limits:

BRAIN; EPIDIDYMIS; EYE; GANGLION, DORSAL ROOT, CERVICAL; GANGLION, DORSAL ROOT, LUMBAR; GANGLION, DORSAL ROOT, THORACIC; GLAND, ADRENAL; GLAND, PARATHYROID; GLAND, PITUITARY; GLAND, PROSTATE; GLAND, THYROID; HEART; LUNG; LYMPH NODE, CERVICAL; LYMPH NODE, ILIAC; LYMPH NODE, MANDIBULAR; LYMPH NODE, MESENTERIC; MUSCLE, BICEPS FEMORIS; MUSCLE, GASTROCNEMIUS; NERVE, OPTIC; NERVE, SCIATIC; NERVE, TIBIAL; SITE, INJECTION; SPINAL CORD, CERVICAL; SPINAL CORD, LUMBAR; SPINAL CORD, THORACIC; SPLEEN; TESTIS; THYMUS; NERVE ROOT, VENTRAL, CERVICAL; NERVE ROOT, VENTRAL, THORACIC; NERVE ROOT, VENTRAL, LUMBAR; NERVE ROOT, DORSAL, CERVICAL; NERVE ROOT, DORSAL, LUMBAR; NERVE ROOT, DORSAL, THORACIC

Histopathology - The following Tissues were Not Examined:

None

Individual Macroscopic and Microscopic Pathology:

5550008

Animal: 1009	Group: 1	Sex: Male
Species: Rat	Strain: Sprague Dawley	
	Dose: 0 vg	
	Removal Reason: Recovery Euthanasia	
	Study Day (Week) of Death: 29 (5)	

Gross Pathology Animal Details:

No animal details found

Gross Pathology Observations [Correlation]:

No observations found

Any remaining study plan required tissues, which have been examined, have no visible lesions

Gross Pathology - The following Tissues were Not Examined:

None

Histopathology Animal Details:

No animal details found

Histopathology Observations [Correlation]:

KIDNEY : Infiltration, mononuclear cell; minimal
LIVER : Infiltration, mononuclear cell; periportal, minimal

Histopathology - The following Tissues were Within Normal Limits:

BRAIN; EPIDIDYMIS; EYE; GANGLION, DORSAL ROOT, CERVICAL; GANGLION, DORSAL ROOT, LUMBAR; GANGLION, DORSAL ROOT, THORACIC; GLAND, ADRENAL; GLAND, PARATHYROID; GLAND, PITUITARY; GLAND, PROSTATE; GLAND, THYROID; HEART; LUNG; LYMPH NODE, CERVICAL; LYMPH NODE, ILIAC; LYMPH NODE, MANDIBULAR; LYMPH NODE, MESENTERIC; MUSCLE, BICEPS FEMORIS; MUSCLE, GASTROCNEMIUS; NERVE, OPTIC; NERVE, SCIATIC; NERVE, TIBIAL; SITE, INJECTION; SPINAL CORD, CERVICAL; SPINAL CORD, LUMBAR; SPINAL CORD, THORACIC; SPLEEN; TESTIS; THYMUS; NERVE ROOT, VENTRAL, CERVICAL; NERVE ROOT, VENTRAL, THORACIC; NERVE ROOT, VENTRAL, LUMBAR; NERVE ROOT, DORSAL, CERVICAL; NERVE ROOT, DORSAL, LUMBAR; NERVE ROOT, DORSAL, THORACIC

Histopathology - The following Tissues were Not Examined:

None

Appendix 18

Appendix 4

Individual Macroscopic and Microscopic Pathology:

5550008

Animal: 1010	Group: 1	Sex: Male
Species: Rat	Strain: Sprague Dawley	
	Dose: 0 vg	
	Removal Reason: Recovery Euthanasia	
	Study Day (Week) of Death: 29 (5)	

Gross Pathology Animal Details:

No animal details found

Gross Pathology Observations [Correlation]:

No observations found

Any remaining study plan required tissues, which have been examined, have no visible lesions

Gross Pathology - The following Tissues were Not Examined:

None

Histopathology Animal Details:

No animal details found

Histopathology Observations [Correlation]:

GLAND, ADRENAL : Vacuolation; cortical, minimal
GLAND, PROSTATE : Infiltration, mononuclear cell; minimal
LIVER : Infiltration, mixed cell; minimal
LIVER : Infiltration, mononuclear cell; periportal, mild

Histopathology - The following Tissues were Within Normal Limits:

BRAIN; EPIDIDYMIS; EYE; GANGLION, DORSAL ROOT, CERVICAL; GANGLION, DORSAL ROOT, LUMBAR; GANGLION, DORSAL ROOT, THORACIC; GLAND, PARATHYROID; GLAND, PITUITARY; GLAND, THYROID; HEART; KIDNEY; LUNG; LYMPH NODE, CERVICAL; LYMPH NODE, ILIAC; LYMPH NODE, MANDIBULAR; LYMPH NODE, MESENTERIC; MUSCLE, BICEPS FEMORIS; MUSCLE, GASTROCNEMIUS; NERVE, OPTIC; NERVE, SCIATIC; NERVE, TIBIAL; SITE, INJECTION; SPINAL CORD, CERVICAL; SPINAL CORD, LUMBAR; SPINAL CORD, THORACIC; SPLEEN; TESTIS; THYMUS; NERVE ROOT, VENTRAL, CERVICAL; NERVE ROOT, VENTRAL, THORACIC; NERVE ROOT, VENTRAL, LUMBAR; NERVE ROOT, DORSAL, CERVICAL; NERVE ROOT, DORSAL, LUMBAR; NERVE ROOT, DORSAL, THORACIC

Histopathology - The following Tissues were Not Examined:

None

Appendix 18

Appendix 4

Individual Macroscopic and Microscopic Pathology:

5550008

Animal: 1011	Group: 1	Sex: Male
Species: Rat	Strain: Sprague Dawley<br>Dose: 0 vg<br>Removal Reason: Recovery Euthanasia<br>Study Day (Week) of Death: 91 (13)	

Gross Pathology Animal Details:

No animal details found

Gross Pathology Observations [Correlation]:

GENERAL OBSERVATIONS : (Comment) Liver lesion submitted cass G.
LIVER : Focus, pale : (Comment) 1, fissure, right medial, near hilus right lateral. (TGL)

Any remaining study plan required tissues, which have been examined, have no visible lesions

Gross Pathology - The following Tissues were Not Examined:

None

Histopathology Animal Details:

No animal details found

Histopathology Observations [Correlation]:

GLAND, PITUITARY : (Note) Only Pars Distalis Available For Evaluation.
GLAND, PROSTATE : Infiltration, mononuclear cell; minimal
HEART : Infiltration, mononuclear cell/degeneration, myofiber; minimal
LIVER : Infiltration, mononuclear cell; minimal
NO CORRELATE : No correlating lesion [LIVER : Focus, pale : (Comment) 1, fissure, right medial, near hilus right lateral. (G)]

Histopathology - The following Tissues were Within Normal Limits:

BRAIN; EPIDIDYMIS; EYE; GANGLION, DORSAL ROOT, CERVICAL; GANGLION, DORSAL ROOT, LUMBAR; GANGLION, DORSAL ROOT, THORACIC; GLAND, ADRENAL; GLAND, PARATHYROID; GLAND, PITUITARY; GLAND, THYROID; KIDNEY; LUNG; LYMPH NODE, CERVICAL; LYMPH NODE, ILIAC; LYMPH NODE, MANDIBULAR; LYMPH NODE, MESENTERIC; MUSCLE, BICEPS FEMORIS; MUSCLE, GASTROCNEMIUS; NERVE, OPTIC; NERVE, SCIATIC; NERVE, TIBIAL; SITE, INJECTION; SPINAL CORD, CERVICAL; SPINAL CORD, LUMBAR; SPINAL CORD, THORACIC; SPLEEN; TESTIS; THYMUS; NERVE ROOT, VENTRAL, CERVICAL; NERVE ROOT, VENTRAL, THORACIC; NERVE ROOT, VENTRAL, LUMBAR; NERVE ROOT, DORSAL, CERVICAL; NERVE ROOT, DORSAL, LUMBAR; NERVE ROOT, DORSAL, THORACIC

Histopathology - The following Tissues were Not Examined:

None

Individual Macroscopic and Microscopic Pathology:

5550008

Animal:	1012	Group:	1	Sex:	Male
Species:	Rat	Strain:	Sprague Dawley		
		Dose:	0 vg		
		Removal Reason:	Recovery Euthanasia		
		Study Day (Week) of Death:	91 (13)		

Gross Pathology Animal Details:

No animal details found

Gross Pathology Observations [Correlation]:

LIVER : Focus, pale : (Comment) 1, fissure, medial (TGL)

Any remaining study plan required tissues, which have been examined, have no visible lesions

Gross Pathology - The following Tissues were Not Examined:

None

Histopathology Animal Details:

No animal details found

Histopathology Observations [Correlation]:

GLAND, ADRENAL : Vacuolation; cortical, mild
HEART : Infiltration, mononuclear cell/degeneration, myofiber; minimal
KIDNEY : Infiltration, mononuclear cell; minimal
NO CORRELATE : No correlating lesion [LIVER : Focus, pale : (Comment) 1, fissure, medial (G)]

Histopathology - The following Tissues were Within Normal Limits:

BRAIN; EPIDIDYMIS; EYE; GANGLION, DORSAL ROOT, CERVICAL; GANGLION, DORSAL ROOT, LUMBAR; GANGLION, DORSAL ROOT, THORACIC; GLAND, PARATHYROID; GLAND, PITUITARY; GLAND, PROSTATE; GLAND, THYROID; LIVER; LUNG; LYMPH NODE, CERVICAL; LYMPH NODE, ILIAC; LYMPH NODE, MANDIBULAR; LYMPH NODE, MESENTERIC; MUSCLE, BICEPS FEMORIS; MUSCLE, GASTROCNEMIUS; NERVE, OPTIC; NERVE, SCIATIC; NERVE, TIBIAL; SITE, INJECTION; SPINAL CORD, CERVICAL; SPINAL CORD, LUMBAR; SPINAL CORD, THORACIC; SPLEEN; TESTIS; THYMUS; NERVE ROOT, VENTRAL, CERVICAL; NERVE ROOT, VENTRAL, THORACIC; NERVE ROOT, VENTRAL, LUMBAR; NERVE ROOT, DORSAL, CERVICAL; NERVE ROOT, DORSAL, LUMBAR; NERVE ROOT, DORSAL, THORACIC

Histopathology - The following Tissues were Not Examined:

None

Appendix 18

Appendix 4

Individual Macroscopic and Microscopic Pathology:

5550008

Animal: 1013	Group: 1	Sex: Male
Species: Rat	Strain: Sprague Dawley	
	Dose: 0 vg	
	Removal Reason: Unscheduled Euthanasia	
	Study Day (Week) of Death: 7 (1)	

Gross Pathology Animal Details:

No animal details found

Gross Pathology Observations [Correlation]:

GENERAL OBSERVATIONS : (Comment) histology section of spleen submitted cass C; histology section of urinary bladder submitted cass D; jejunum lesion extra section submitted cass C. Reason for sacrifice: poor and/or deteriorating condition, other: backbone prominent, weak, thin, limited usage 3 all limbs, DEH3, abnormal respiratory rate increased.
LARGE INTESTINE, CECUM : Abnormal content; dark : (Comment) firm
LARGE INTESTINE, COLON : Abnormal content; dark : (Comment) firm
SMALL INTESTINE, JEJUNUM : Abnormal content; dark : (Comment) proximal
SPLEEN : Small (TGL)
STOMACH : Focus, dark : (Comment) >10, mucosa glandular (TGL)

Any remaining study plan required tissues, which have been examined, have no visible lesions

Gross Pathology - The following Tissues were Not Examined:

None

Histopathology Animal Details:

Cause of Death: Undetermined

Histopathology Observations [Correlation]:

KIDNEY : Dilatation; tubular, mild
LIVER : Tension lipidosis; minimal
SITE, INJECTION : Infiltration, mononuclear cell; minimal, cauda equina
THYMUS : (Note) Postmortem Change Present.
THYMUS : Cellularity, decreased; lymphoid, moderate
NO CORRELATE : No correlating lesion [SPLEEN : Small (G) | STOMACH : Focus, dark : (Comment) >10, mucosa glandular (G)]

Histopathology - The following Tissues were Within Normal Limits:

BRAIN; EPIDIDYMIS; EYE; GANGLION, DORSAL ROOT, CERVICAL; GANGLION, DORSAL ROOT, LUMBAR; GANGLION, DORSAL ROOT, THORACIC; GLAND, ADRENAL; GLAND, PARATHYROID; GLAND, PITUITARY; GLAND, PROSTATE; GLAND, THYROID; HEART; LARGE INTESTINE, CECUM; LARGE INTESTINE, COLON; LUNG; LYMPH NODE, CERVICAL; LYMPH NODE, ILIAC; LYMPH NODE, MANDIBULAR; LYMPH NODE, MESENTERIC; MUSCLE, BICEPS FEMORIS; MUSCLE, GASTROCNEMIUS; NERVE, OPTIC; NERVE, SCIATIC; NERVE, TIBIAL; SMALL INTESTINE, JEJUNUM; SPINAL CORD, CERVICAL; SPINAL CORD, LUMBAR; SPINAL CORD, THORACIC; SPLEEN; STOMACH; TESTIS; NERVE ROOT, VENTRAL, CERVICAL; NERVE ROOT, VENTRAL, THORACIC; NERVE ROOT, VENTRAL, LUMBAR; NERVE ROOT, DORSAL, CERVICAL; NERVE ROOT, DORSAL, LUMBAR; NERVE ROOT, DORSAL, THORACIC

Histopathology - The following Tissues were Not Examined:

Appendix 18

Appendix 4

Individual Macroscopic and Microscopic Pathology:

5550008

Animal: 1013 (Continued)	Group: 1	Sex: Male
None		

Individual Macroscopic and Microscopic Pathology:

5550008

Animal:	1014	Group:	1	Sex:	Male
Species:	Rat	Strain:	Sprague Dawley		
		Dose:	0 vg		
		Removal Reason:	Terminal Euthanasia		
		Study Day (Week) of Death:	8 (2)		

Gross Pathology Animal Details:

No animal details found

Gross Pathology Observations [Correlation]:

No observations found

Any remaining study plan required tissues, which have been examined, have no visible lesions

Gross Pathology - The following Tissues were Not Examined:

None

Histopathology Animal Details:

No animal details found

Histopathology Observations [Correlation]:

KIDNEY : Chronic progressive nephropathy; minimal
LIVER : Hyperplasia; minimal, bile duct
LIVER : Infiltration, mononuclear cell; minimal : (Comment) periportal
SITE, INJECTION : Degeneration; axonal, minimal, cauda equina

Histopathology - The following Tissues were Within Normal Limits:

BRAIN; EPIDIDYMIS; EYE; GANGLION, DORSAL ROOT, CERVICAL; GANGLION, DORSAL ROOT, LUMBAR; GANGLION, DORSAL ROOT, THORACIC; GLAND, ADRENAL; GLAND, PARATHYROID; GLAND, PITUITARY; GLAND, PROSTATE; GLAND, THYROID; HEART; LUNG; LYMPH NODE, CERVICAL; LYMPH NODE, ILIAC; LYMPH NODE, MANDIBULAR; LYMPH NODE, MESENTERIC; MUSCLE, BICEPS FEMORIS; MUSCLE, GASTROCNEMIUS; NERVE, SCIATIC; NERVE, TIBIAL; SPINAL CORD, CERVICAL; SPINAL CORD, LUMBAR; SPINAL CORD, THORACIC; SPLEEN; TESTIS; THYMUS; NERVE ROOT, VENTRAL, CERVICAL; NERVE ROOT, VENTRAL, THORACIC; NERVE ROOT, VENTRAL, LUMBAR; NERVE ROOT, DORSAL, CERVICAL; NERVE ROOT, DORSAL, LUMBAR; NERVE ROOT, DORSAL, THORACIC

Histopathology - The following Tissues were Not Examined:

NERVE, OPTIC - Not Present In Section.

Appendix 18

Appendix 4

Individual Macroscopic and Microscopic Pathology:

5550008

Animal: 1015	Group: 1	Sex: Male
Species: Rat	Strain: Sprague Dawley	
	Dose: 0 vg	
	Removal Reason: Terminal Euthanasia	
	Study Day (Week) of Death: 8 (2)	

Gross Pathology Animal Details:

No animal details found

Gross Pathology Observations [Correlation]:

THYMUS : Focus, dark : (Comment) >10. (TGL)

Any remaining study plan required tissues, which have been examined, have no visible lesions

Gross Pathology - The following Tissues were Not Examined:

None

Histopathology Animal Details:

No animal details found

Histopathology Observations [Correlation]:

KIDNEY : Infiltration, mononuclear cell; minimal
KIDNEY : Chronic progressive nephropathy; minimal
LIVER : Tension lipidosis; minimal
LIVER : Infiltration, mixed cell; minimal
SITE, INJECTION : Degeneration; axonal, minimal, cauda equina
NO CORRELATE : No correlating lesion [THYMUS : Focus, dark : (Comment) >10. (G)]

Histopathology - The following Tissues were Within Normal Limits:

BRAIN; EPIDIDYMIS; EYE; GANGLION, DORSAL ROOT, CERVICAL; GANGLION, DORSAL ROOT, LUMBAR; GANGLION, DORSAL ROOT, THORACIC; GLAND, ADRENAL; GLAND, PARATHYROID; GLAND, PITUITARY; GLAND, PROSTATE; GLAND, THYROID; HEART; LUNG; LYMPH NODE, CERVICAL; LYMPH NODE, ILIAC; LYMPH NODE, MANDIBULAR; LYMPH NODE, MESENTERIC; MUSCLE, BICEPS FEMORIS; MUSCLE, GASTROCNEMIUS; NERVE, OPTIC; NERVE, SCIATIC; NERVE, TIBIAL; SPINAL CORD, CERVICAL; SPINAL CORD, LUMBAR; SPINAL CORD, THORACIC; SPLEEN; TESTIS; THYMUS; NERVE ROOT, VENTRAL, CERVICAL; NERVE ROOT, VENTRAL, THORACIC; NERVE ROOT, VENTRAL, LUMBAR; NERVE ROOT, DORSAL, CERVICAL; NERVE ROOT, DORSAL, LUMBAR; NERVE ROOT, DORSAL, THORACIC

Histopathology - The following Tissues were Not Examined:

None

Appendix 18

Appendix 4

Individual Macroscopic and Microscopic Pathology:

5550008

Animal: 1501	Group: 1	Sex: Female
Species: Rat	Strain: Sprague Dawley	
	Dose: 0 vg	
	Removal Reason: Terminal Euthanasia	
	Study Day (Week) of Death: 8 (2)	

Gross Pathology Animal Details:

No animal details found

Gross Pathology Observations [Correlation]:

No observations found

Any remaining study plan required tissues, which have been examined, have no visible lesions

Gross Pathology - The following Tissues were Not Examined:

None

Histopathology Animal Details:

No animal details found

Histopathology Observations [Correlation]:

LIVER : Vacuolation; hepatocellular, periportal, minimal
SITE, INJECTION : Infiltration, mononuclear cell; minimal, leptomeninges
SITE, INJECTION : Degeneration; axonal, minimal, cauda equina

Histopathology - The following Tissues were Within Normal Limits:

BRAIN; CERVIX; EYE; GANGLION, DORSAL ROOT, CERVICAL; GANGLION, DORSAL ROOT, LUMBAR; GANGLION, DORSAL ROOT, THORACIC; GLAND, ADRENAL; GLAND, PARATHYROID; GLAND, PITUITARY; GLAND, THYROID; HEART; KIDNEY; LUNG; LYMPH NODE, CERVICAL; LYMPH NODE, ILIAC; LYMPH NODE, MANDIBULAR; LYMPH NODE, MESENTERIC; MUSCLE, BICEPS FEMORIS; MUSCLE, GASTROCNEMIUS; NERVE, OPTIC; NERVE, SCIATIC; NERVE, TIBIAL; OVARY; SPINAL CORD, CERVICAL; SPINAL CORD, LUMBAR; SPINAL CORD, THORACIC; SPLEEN; THYMUS; UTERUS; NERVE ROOT, VENTRAL, CERVICAL; NERVE ROOT, VENTRAL, THORACIC; NERVE ROOT, VENTRAL, LUMBAR; NERVE ROOT, DORSAL, CERVICAL; NERVE ROOT, DORSAL, LUMBAR; NERVE ROOT, DORSAL, THORACIC

Histopathology - The following Tissues were Not Examined:

None

Appendix 18

Appendix 4

Individual Macroscopic and Microscopic Pathology:

5550008

Animal:	1502	Group:	1	Sex:	Female
Species:	Rat	Strain:	Sprague Dawley		
		Dose:	0 vg		
		Removal Reason:	Terminal Euthanasia		
		Study Day (Week) of Death:	8 (2)		

Gross Pathology Animal Details:

No animal details found

Gross Pathology Observations [Correlation]:

LIVER : Focus, pale : (Comment) 1, fissure, right medial (TGL)

Any remaining study plan required tissues, which have been examined, have no visible lesions

Gross Pathology - The following Tissues were Not Examined:

None

Histopathology Animal Details:

No animal details found

Histopathology Observations [Correlation]:

EYE : Dysplasia; mild, retina
GLAND, PARATHYROID : (Note) One Of A Pair Available For Evaluation.
GLAND, THYROID : (Note) One Of A Pair Available For Evaluation.
LIVER : Vacuolation; hepatocellular, periportal, minimal
SITE, INJECTION : Infiltration, mononuclear cell; minimal, leptomeninges
SPINAL CORD, LUMBAR : Infiltration, mononuclear cell; minimal
NO CORRELATE : No correlating lesion [LIVER : Focus, pale : (Comment) 1, fissure, right medial (G)]

Histopathology - The following Tissues were Within Normal Limits:

BRAIN; CERVIX; GANGLION, DORSAL ROOT, CERVICAL; GANGLION, DORSAL ROOT, LUMBAR; GANGLION, DORSAL ROOT, THORACIC; GLAND, ADRENAL; GLAND, PARATHYROID; GLAND, PITUITARY; GLAND, THYROID; HEART; KIDNEY; LUNG; LYMPH NODE, CERVICAL; LYMPH NODE, ILIAC; LYMPH NODE, MANDIBULAR; LYMPH NODE, MESENTERIC; MUSCLE, BICEPS FEMORIS; MUSCLE, GASTROCNEMIUS; NERVE, OPTIC; NERVE, SCIATIC; NERVE, TIBIAL; OVARY; SPINAL CORD, CERVICAL; SPINAL CORD, THORACIC; SPLEEN; THYMUS; UTERUS; NERVE ROOT, VENTRAL, CERVICAL; NERVE ROOT, VENTRAL, THORACIC; NERVE ROOT, VENTRAL, LUMBAR; NERVE ROOT, DORSAL, CERVICAL; NERVE ROOT, DORSAL, LUMBAR; NERVE ROOT, DORSAL, THORACIC

Histopathology - The following Tissues were Not Examined:

None

Appendix 18

Appendix 4

Individual Macroscopic and Microscopic Pathology:

5550008

Animal:	1503	Group:	1	Sex:	Female
Species:	Rat	Strain:	Sprague Dawley		
		Dose:	0 vg		
		Removal Reason:	Recovery Euthanasia		
		Study Day (Week) of Death:	91 (13)		

Gross Pathology Animal Details:

No animal details found

Gross Pathology Observations [Correlation]:

GENERAL OBSERVATIONS : (Comment) Lymph node lesion submitted with pancreas
LYMPH NODE : Discoloration, mottled : (Comment) pancreatic (TGL)

Any remaining study plan required tissues, which have been examined, have no visible lesions

Gross Pathology - The following Tissues were Not Examined:

None

Histopathology Animal Details:

No animal details found

Histopathology Observations [Correlation]:

LIVER : Infiltration, mixed cell; minimal
LYMPH NODE : Hemorrhage; acute, minimal [LYMPH NODE : Discoloration, mottled : (Comment) pancreatic (G)]

Histopathology - The following Tissues were Within Normal Limits:

BRAIN; CERVIX; EYE; GANGLION, DORSAL ROOT, CERVICAL; GANGLION, DORSAL ROOT, LUMBAR; GANGLION, DORSAL ROOT, THORACIC; GLAND, ADRENAL; GLAND, PITUITARY; GLAND, THYROID; HEART; KIDNEY; LUNG; LYMPH NODE, CERVICAL; LYMPH NODE, ILIAC; LYMPH NODE, MANDIBULAR; LYMPH NODE, MESENTERIC; MUSCLE, BICEPS FEMORIS; MUSCLE, GASTROCNEMIUS; NERVE, OPTIC; NERVE, SCIATIC; NERVE, TIBIAL; OVARY; SITE, INJECTION; SPINAL CORD, CERVICAL; SPINAL CORD, LUMBAR; SPINAL CORD, THORACIC; SPLEEN; THYMUS; UTERUS; NERVE ROOT, VENTRAL, CERVICAL; NERVE ROOT, VENTRAL, THORACIC; NERVE ROOT, VENTRAL, LUMBAR; NERVE ROOT, DORSAL, CERVICAL; NERVE ROOT, DORSAL, LUMBAR; NERVE ROOT, DORSAL, THORACIC

Histopathology - The following Tissues were Not Examined:

GLAND, PARATHYROID - Not Present In Section.

Individual Macroscopic and Microscopic Pathology:

5550008

Animal:	1504	Group:	1	Sex:	Female
Species:	Rat	Strain:	Sprague Dawley		
		Dose:	0 vg		
		Removal Reason:	Recovery Euthanasia		
		Study Day (Week) of Death:	91 (13)		

Gross Pathology Animal Details:

No animal details found

Gross Pathology Observations [Correlation]:

GLAND, PITUITARY : Enlargement (TGL)

Any remaining study plan required tissues, which have been examined, have no visible lesions

Gross Pathology - The following Tissues were Not Examined:

VAGINA - Lost During Necropsy.

Histopathology Animal Details:

No animal details found

Histopathology Observations [Correlation]:

LIVER : Infiltration, mixed cell; minimal
SITE, INJECTION : Infiltration, mononuclear cell; minimal, leptomeninges
NO CORRELATE : No correlating lesion [GLAND, PITUITARY : Enlargement (G)]

Histopathology - The following Tissues were Within Normal Limits:

BRAIN; CERVIX; EYE; GANGLION, DORSAL ROOT, CERVICAL; GANGLION, DORSAL ROOT, LUMBAR; GANGLION, DORSAL ROOT, THORACIC; GLAND, ADRENAL; GLAND, PARATHYROID; GLAND, PITUITARY; GLAND, THYROID; HEART; KIDNEY; LUNG; LYMPH NODE, CERVICAL; LYMPH NODE, ILIAC; LYMPH NODE, MANDIBULAR; LYMPH NODE, MESENTERIC; MUSCLE, BICEPS FEMORIS; MUSCLE, GASTROCNEMIUS; NERVE, OPTIC; NERVE, SCIATIC; NERVE, TIBIAL; OVARY; SPINAL CORD, CERVICAL; SPINAL CORD, LUMBAR; SPINAL CORD, THORACIC; SPLEEN; THYMUS; UTERUS; NERVE ROOT, VENTRAL, CERVICAL; NERVE ROOT, VENTRAL, THORACIC; NERVE ROOT, VENTRAL, LUMBAR; NERVE ROOT, DORSAL, CERVICAL; NERVE ROOT, DORSAL, LUMBAR; NERVE ROOT, DORSAL, THORACIC

Histopathology - The following Tissues were Not Examined:

None

Individual Macroscopic and Microscopic Pathology:

5550008

Animal:	1505	Group:	1	Sex:	Female
Species:	Rat	Strain:	Sprague Dawley		
		Dose:	0 vg		
		Removal Reason:	Terminal Euthanasia		
		Study Day (Week) of Death:	8 (2)		

Gross Pathology Animal Details:

No animal details found

Gross Pathology Observations [Correlation]:

LIVER : Focus, pale : (Comment) 1, fissure, right medial. (TGL)

Any remaining study plan required tissues, which have been examined, have no visible lesions

Gross Pathology - The following Tissues were Not Examined:

None

Histopathology Animal Details:

No animal details found

Histopathology Observations [Correlation]:

KIDNEY : Infiltration, mononuclear cell; minimal
LIVER : Tension lipidosis; minimal [LIVER : Focus, pale : (Comment) 1, fissure, right medial. (G)]
LIVER : Infiltration, mixed cell; minimal
LIVER : Infiltration, mononuclear cell; periportal, minimal
SITE, INJECTION : Infiltration, mononuclear cell; minimal, leptomeninges

Histopathology - The following Tissues were Within Normal Limits:

BRAIN; CERVIX; EYE; GANGLION, DORSAL ROOT, CERVICAL; GANGLION, DORSAL ROOT, LUMBAR; GANGLION, DORSAL ROOT, THORACIC; GLAND, ADRENAL; GLAND, PARATHYROID; GLAND, PITUITARY; GLAND, THYROID; HEART; LUNG; LYMPH NODE, CERVICAL; LYMPH NODE, ILIAC; LYMPH NODE, MANDIBULAR; LYMPH NODE, MESENTERIC; MUSCLE, BICEPS FEMORIS; MUSCLE, GASTROCNEMIUS; NERVE, OPTIC; NERVE, SCIATIC; NERVE, TIBIAL; OVARY; SPINAL CORD, CERVICAL; SPINAL CORD, LUMBAR; SPINAL CORD, THORACIC; SPLEEN; THYMUS; UTERUS; NERVE ROOT, VENTRAL, CERVICAL; NERVE ROOT, VENTRAL, THORACIC; NERVE ROOT, VENTRAL, LUMBAR; NERVE ROOT, DORSAL, CERVICAL; NERVE ROOT, DORSAL, LUMBAR; NERVE ROOT, DORSAL, THORACIC

Histopathology - The following Tissues were Not Examined:

None

Appendix 18

Appendix 4

Individual Macroscopic and Microscopic Pathology:

5550008

Animal:	1506	Group:	1	Sex:	Female
Species:	Rat	Strain:	Sprague Dawley		
		Dose:	0 vg		
		Removal Reason:	Terminal Euthanasia		
		Study Day (Week) of Death:	8 (2)		

Gross Pathology Animal Details:

No animal details found

Gross Pathology Observations [Correlation]:

THYMUS : Focus, dark : (Comment) 1, right (TGL)

Any remaining study plan required tissues, which have been examined, have no visible lesions

Gross Pathology - The following Tissues were Not Examined:

None

Histopathology Animal Details:

No animal details found

Histopathology Observations [Correlation]:

GLAND, PITUITARY : (Note) Only Pars Distalis Available For Evaluation.
LIVER : Vacuolation; hepatocellular, periportal, mild
LIVER : Infiltration, mononuclear cell; periportal, minimal
NO CORRELATE : No correlating lesion [THYMUS : Focus, dark : (Comment) 1, right (G)]

Histopathology - The following Tissues were Within Normal Limits:

BRAIN; CERVIX; EYE; GANGLION, DORSAL ROOT, CERVICAL; GANGLION, DORSAL ROOT, LUMBAR; GANGLION, DORSAL ROOT, THORACIC; GLAND, ADRENAL; GLAND, PARATHYROID; GLAND, PITUITARY; GLAND, THYROID; HEART; KIDNEY; LUNG; LYMPH NODE, CERVICAL; LYMPH NODE, ILIAC; LYMPH NODE, MANDIBULAR; LYMPH NODE, MESENTERIC; MUSCLE, BICEPS FEMORIS; MUSCLE, GASTROCNEMIUS; NERVE, OPTIC; NERVE, SCIATIC; NERVE, TIBIAL; OVARY; SITE, INJECTION; SPINAL CORD, CERVICAL; SPINAL CORD, LUMBAR; SPINAL CORD, THORACIC; SPLEEN; THYMUS; UTERUS; NERVE ROOT, VENTRAL, CERVICAL; NERVE ROOT, VENTRAL, THORACIC; NERVE ROOT, VENTRAL, LUMBAR; NERVE ROOT, DORSAL, CERVICAL; NERVE ROOT, DORSAL, LUMBAR; NERVE ROOT, DORSAL, THORACIC

Histopathology - The following Tissues were Not Examined:

None

Appendix 18

Appendix 4

Individual Macroscopic and Microscopic Pathology:

5550008

Animal: 1507	Group: 1	Sex: Female
Species: Rat	Strain: Sprague Dawley	
	Dose: 0 vg	
	Removal Reason: Terminal Euthanasia	
	Study Day (Week) of Death: 8 (2)	

Gross Pathology Animal Details:

No animal details found

Gross Pathology Observations [Correlation]:

No observations found

Any remaining study plan required tissues, which have been examined, have no visible lesions

Gross Pathology - The following Tissues were Not Examined:

None

Histopathology Animal Details:

No animal details found

Histopathology Observations [Correlation]:

GLAND, ADRENAL : (Note) One Of A Pair Available For Evaluation.
GLAND, PARATHYROID : (Note) One Of A Pair Available For Evaluation.
SITE, INJECTION : Degeneration; axonal, minimal, cauda equina

Histopathology - The following Tissues were Within Normal Limits:

BRAIN; CERVIX; EYE; GANGLION, DORSAL ROOT, CERVICAL; GANGLION, DORSAL ROOT, LUMBAR; GANGLION, DORSAL ROOT, THORACIC; GLAND, ADRENAL; GLAND, PARATHYROID; GLAND, PITUITARY; GLAND, THYROID; HEART; KIDNEY; LIVER; LUNG; LYMPH NODE, CERVICAL; LYMPH NODE, ILIAC; LYMPH NODE, MANDIBULAR; LYMPH NODE, MESENTERIC; MUSCLE, BICEPS FEMORIS; MUSCLE, GASTROCNEMIUS; NERVE, OPTIC; NERVE, SCIATIC; NERVE, TIBIAL; OVARY; SPINAL CORD, CERVICAL; SPINAL CORD, LUMBAR; SPINAL CORD, THORACIC; SPLEEN; THYMUS; UTERUS; NERVE ROOT, VENTRAL, CERVICAL; NERVE ROOT, VENTRAL, THORACIC; NERVE ROOT, VENTRAL, LUMBAR; NERVE ROOT, DORSAL, CERVICAL; NERVE ROOT, DORSAL, LUMBAR; NERVE ROOT, DORSAL, THORACIC

Histopathology - The following Tissues were Not Examined:

None

Individual Macroscopic and Microscopic Pathology:

5550008

Animal: 1508	Group: 1		Sex: Female
Species: Rat	Strain: Sprague Dawley		
	Dose: 0 vg		
	Removal Reason: Recovery Euthanasia		
	Study Day (Week) of Death: 29 (5)		

Gross Pathology Animal Details:

No animal details found

Gross Pathology Observations [Correlation]:

LYMPH NODE, ILIAC : (Comment) Left lost at necropsy.
THYMUS : Discoloration, mottled : (Comment) right (TGL)

Any remaining study plan required tissues, which have been examined, have no visible lesions

Gross Pathology - The following Tissues were Not Examined:

None

Histopathology Animal Details:

No animal details found

Histopathology Observations [Correlation]:

KIDNEY : Chronic progressive nephropathy; minimal
LIVER : Infiltration, mixed cell; minimal
NO CORRELATE : No correlating lesion [THYMUS : Discoloration, mottled : (Comment) right (G)]

Histopathology - The following Tissues were Within Normal Limits:

BRAIN; CERVIX; EYE; GANGLION, DORSAL ROOT, CERVICAL; GANGLION, DORSAL ROOT, LUMBAR; GANGLION, DORSAL ROOT, THORACIC; GLAND, ADRENAL; GLAND, PARATHYROID; GLAND, PITUITARY; GLAND, THYROID; HEART; LUNG; LYMPH NODE, CERVICAL; LYMPH NODE, MANDIBULAR; LYMPH NODE, MESENTERIC; MUSCLE, BICEPS FEMORIS; MUSCLE, GASTROCNEMIUS; NERVE, OPTIC; NERVE, SCIATIC; NERVE, TIBIAL; OVARY; SITE, INJECTION; SPINAL CORD, CERVICAL; SPINAL CORD, LUMBAR; SPINAL CORD, THORACIC; SPLEEN; THYMUS; UTERUS; NERVE ROOT, VENTRAL, CERVICAL; NERVE ROOT, VENTRAL, THORACIC; NERVE ROOT, VENTRAL, LUMBAR; NERVE ROOT, DORSAL, CERVICAL; NERVE ROOT, DORSAL, LUMBAR; NERVE ROOT, DORSAL, THORACIC

Histopathology - The following Tissues were Not Examined:

LYMPH NODE, ILIAC - Not Present In Section.

Appendix 18

Appendix 4

Individual Macroscopic and Microscopic Pathology:

5550008

Animal:	1509	Group:	1	Sex:	Female
Species:	Rat	Strain:	Sprague Dawley		
		Dose:	0 vg		
		Removal Reason:	Recovery Euthanasia		
		Study Day (Week) of Death:	29 (5)		

Gross Pathology Animal Details:

No animal details found

Gross Pathology Observations [Correlation]:

GENERAL OBSERVATIONS : (Comment) Pituitary submitted in two pieces; Optic nerve left submitted in cassette C; Lymph node lesion submitted with pancreas.
LYMPH NODE : Focus, dark : (Comment) 1, pancreatic (TGL)

Any remaining study plan required tissues, which have been examined, have no visible lesions

Gross Pathology - The following Tissues were Not Examined:

None

Histopathology Animal Details:

No animal details found

Histopathology Observations [Correlation]:

GLAND, PITUITARY : (Note) Only Pars Distalis Available For Evaluation.
KIDNEY : Dilatation; tubular, minimal
LIVER : Infiltration, mixed cell; mild
LYMPH NODE : Hemorrhage; acute, mild : (Comment) with hemosiderophages [LYMPH NODE : Focus, dark : (Comment) 1, pancreatic (G)]
SITE, INJECTION : Infiltration, mononuclear cell; minimal, cauda equina

Histopathology - The following Tissues were Within Normal Limits:

BRAIN; CERVIX; EYE; GANGLION, DORSAL ROOT, CERVICAL; GANGLION, DORSAL ROOT, LUMBAR; GANGLION, DORSAL ROOT, THORACIC; GLAND, ADRENAL; GLAND, PARATHYROID; GLAND, PITUITARY; GLAND, THYROID; HEART; LUNG; LYMPH NODE, CERVICAL; LYMPH NODE, ILIAC; LYMPH NODE, MANDIBULAR; LYMPH NODE, MESENTERIC; MUSCLE, BICEPS FEMORIS; MUSCLE, GASTROCNEMIUS; NERVE, OPTIC; NERVE, SCIATIC; NERVE, TIBIAL; OVARY; SPINAL CORD, CERVICAL; SPINAL CORD, LUMBAR; SPINAL CORD, THORACIC; SPLEEN; THYMUS; UTERUS; NERVE ROOT, VENTRAL, CERVICAL; NERVE ROOT, VENTRAL, THORACIC; NERVE ROOT, VENTRAL, LUMBAR; NERVE ROOT, DORSAL, CERVICAL; NERVE ROOT, DORSAL, LUMBAR; NERVE ROOT, DORSAL, THORACIC

Histopathology - The following Tissues were Not Examined:

None

Appendix 18

Appendix 4

Individual Macroscopic and Microscopic Pathology:

5550008

Animal:	1510	Group:	1	Sex:	Female
Species:	Rat	Strain:	Sprague Dawley		
		Dose:	0 vg		
		Removal Reason: Recovery Euthanasia			
		Study Day (Week) of Death: 91 (13)			

Gross Pathology Animal Details:

No animal details found

Gross Pathology Observations [Correlation]:

THYMUS : Focus, dark : (Comment) 3, left (TGL)

Any remaining study plan required tissues, which have been examined, have no visible lesions

Gross Pathology - The following Tissues were Not Examined:

None

Histopathology Animal Details:

No animal details found

Histopathology Observations [Correlation]:

GLAND, PITUITARY : (Note) Only Pars Distalis Available For Evaluation.
LIVER : Infiltration, mixed cell; minimal
LYMPH NODE, ILIAC : Hemorrhage; acute, mild : (Comment) with some hemosiderophages
NO CORRELATE : No correlating lesion [THYMUS : Focus, dark : (Comment) 3, left (G)]

Histopathology - The following Tissues were Within Normal Limits:

BRAIN; CERVIX; EYE; GANGLION, DORSAL ROOT, CERVICAL; GANGLION, DORSAL ROOT, LUMBAR; GANGLION, DORSAL ROOT, THORACIC; GLAND, ADRENAL; GLAND, PITUITARY; GLAND, THYROID; HEART; KIDNEY; LUNG; LYMPH NODE, CERVICAL; LYMPH NODE, MANDIBULAR; LYMPH NODE, MESENTERIC; MUSCLE, BICEPS FEMORIS; MUSCLE, GASTROCNEMIUS; NERVE, OPTIC; NERVE, SCIATIC; NERVE, TIBIAL; OVARY; SITE, INJECTION; SPINAL CORD, CERVICAL; SPINAL CORD, LUMBAR; SPINAL CORD, THORACIC; SPLEEN; THYMUS; UTERUS; NERVE ROOT, VENTRAL, CERVICAL; NERVE ROOT, VENTRAL, THORACIC; NERVE ROOT, VENTRAL, LUMBAR; NERVE ROOT, DORSAL, CERVICAL; NERVE ROOT, DORSAL, LUMBAR; NERVE ROOT, DORSAL, THORACIC

Histopathology - The following Tissues were Not Examined:

GLAND, PARATHYROID - Not Present In Section.

Individual Macroscopic and Microscopic Pathology:

5550008

Animal:	1511	Group:	1	Sex:	Female
Species:	Rat	Strain:	Sprague Dawley		
		Dose:	0 vg		
		Removal Reason:	Recovery Euthanasia		
		Study Day (Week) of Death:	91 (13)		

Gross Pathology Animal Details:

No animal details found

Gross Pathology Observations [Correlation]:

No observations found

Any remaining study plan required tissues, which have been examined, have no visible lesions

Gross Pathology - The following Tissues were Not Examined:

None

Histopathology Animal Details:

No animal details found

Histopathology Observations [Correlation]:

GLAND, PARATHYROID : (Note) One Of A Pair Available For Evaluation.
HEART : Infiltration, mononuclear cell/degeneration, myofiber; minimal
LIVER : Vacuolation; hepatocellular, periportal, minimal
LIVER : Infiltration, mixed cell; minimal

Histopathology - The following Tissues were Within Normal Limits:

BRAIN; CERVIX; EYE; GANGLION, DORSAL ROOT, CERVICAL; GANGLION, DORSAL ROOT, LUMBAR; GANGLION, DORSAL ROOT, THORACIC; GLAND, ADRENAL; GLAND, PARATHYROID; GLAND, PITUITARY; GLAND, THYROID; KIDNEY; LUNG; LYMPH NODE, CERVICAL; LYMPH NODE, ILIAC; LYMPH NODE, MANDIBULAR; LYMPH NODE, MESENTERIC; MUSCLE, BICEPS FEMORIS; MUSCLE, GASTROCNEMIUS; NERVE, OPTIC; NERVE, SCIATIC; NERVE, TIBIAL; OVARY; SITE, INJECTION; SPINAL CORD, CERVICAL; SPINAL CORD, LUMBAR; SPINAL CORD, THORACIC; SPLEEN; THYMUS; UTERUS; NERVE ROOT, VENTRAL, CERVICAL; NERVE ROOT, VENTRAL, THORACIC; NERVE ROOT, VENTRAL, LUMBAR; NERVE ROOT, DORSAL, CERVICAL; NERVE ROOT, DORSAL, LUMBAR; NERVE ROOT, DORSAL, THORACIC

Histopathology - The following Tissues were Not Examined:

None

Appendix 18

Appendix 4

Individual Macroscopic and Microscopic Pathology:

5550008

Animal:	1512	Group:	1	Sex:	Female
Species:	Rat	Strain:	Sprague Dawley		
		Dose:	0 vg		
		Removal Reason:	Recovery Euthanasia		
		Study Day (Week) of Death:	91 (13)		

Gross Pathology Animal Details:

No animal details found

Gross Pathology Observations [Correlation]:

No observations found

Any remaining study plan required tissues, which have been examined, have no visible lesions

Gross Pathology - The following Tissues were Not Examined:

None

Histopathology Animal Details:

No animal details found

Histopathology Observations [Correlation]:

GLAND, PARATHYROID : (Note) One Of A Pair Available For Evaluation.
KIDNEY : Chronic progressive nephropathy; minimal
LIVER : Infiltration, mixed cell; minimal
LIVER : Infiltration, mononuclear cell; periportal, minimal

Histopathology - The following Tissues were Within Normal Limits:

BRAIN; CERVIX; EYE; GANGLION, DORSAL ROOT, CERVICAL; GANGLION, DORSAL ROOT, LUMBAR; GANGLION, DORSAL ROOT, THORACIC; GLAND, ADRENAL; GLAND, PARATHYROID; GLAND, PITUITARY; GLAND, THYROID; HEART; LUNG; LYMPH NODE, CERVICAL; LYMPH NODE, ILIAC; LYMPH NODE, MANDIBULAR; LYMPH NODE, MESENTERIC; MUSCLE, BICEPS FEMORIS; MUSCLE, GASTROCNEMIUS; NERVE, OPTIC; NERVE, SCIATIC; NERVE, TIBIAL; OVARY; SITE, INJECTION; SPINAL CORD, CERVICAL; SPINAL CORD, LUMBAR; SPINAL CORD, THORACIC; SPLEEN; THYMUS; UTERUS; NERVE ROOT, VENTRAL, CERVICAL; NERVE ROOT, VENTRAL, THORACIC; NERVE ROOT, VENTRAL, LUMBAR; NERVE ROOT, DORSAL, CERVICAL; NERVE ROOT, DORSAL, LUMBAR; NERVE ROOT, DORSAL, THORACIC

Histopathology - The following Tissues were Not Examined:

None

Appendix 18

Appendix 4

Individual Macroscopic and Microscopic Pathology:

5550008

Animal:	1513	Group:	1	Sex:	Female
Species:	Rat	Strain:	Sprague Dawley		
		Dose:	0 vg		
		Removal Reason:	Recovery Euthanasia		
		Study Day (Week) of Death:	29 (5)		

Gross Pathology Animal Details:

No animal details found

Gross Pathology Observations [Correlation]:

No observations found

Any remaining study plan required tissues, which have been examined, have no visible lesions

Gross Pathology - The following Tissues were Not Examined:

None

Histopathology Animal Details:

No animal details found

Histopathology Observations [Correlation]:

GLAND, ADRENAL : Vacuolation; cortical, mild
GLAND, PITUITARY : (Note) Only Pars Distalis Available For Evaluation.
KIDNEY : Infiltration, mononuclear cell; minimal
KIDNEY : Chronic progressive nephropathy; minimal
LIVER : Infiltration, mixed cell; minimal
SITE, INJECTION : Infiltration, mononuclear cell; minimal, leptomeninges

Histopathology - The following Tissues were Within Normal Limits:

BRAIN; CERVIX; EYE; GANGLION, DORSAL ROOT, CERVICAL; GANGLION, DORSAL ROOT, LUMBAR; GANGLION, DORSAL ROOT, THORACIC; GLAND, PARATHYROID; GLAND, PITUITARY; GLAND, THYROID; HEART; LUNG; LYMPH NODE, CERVICAL; LYMPH NODE, ILIAC; LYMPH NODE, MANDIBULAR; LYMPH NODE, MESENTERIC; MUSCLE, BICEPS FEMORIS; MUSCLE, GASTROCNEMIUS; NERVE, OPTIC; NERVE, SCIATIC; NERVE, TIBIAL; OVARY; SPINAL CORD, CERVICAL; SPINAL CORD, LUMBAR; SPINAL CORD, THORACIC; SPLEEN; THYMUS; UTERUS; NERVE ROOT, VENTRAL, CERVICAL; NERVE ROOT, VENTRAL, THORACIC; NERVE ROOT, VENTRAL, LUMBAR; NERVE ROOT, DORSAL, CERVICAL; NERVE ROOT, DORSAL, LUMBAR; NERVE ROOT, DORSAL, THORACIC

Histopathology - The following Tissues were Not Examined:

None

Individual Macroscopic and Microscopic Pathology:

5550008

Animal: 1514	Group: 1	Sex: Female
Species: Rat	Strain: Sprague Dawley<br>Dose: 0 vg<br>Removal Reason: Recovery Euthanasia<br>Study Day (Week) of Death: 29 (5)	

Gross Pathology Animal Details:

No animal details found

Gross Pathology Observations [Correlation]:

GENERAL OBSERVATIONS : (Comment) Optic nerve submitted in cassette C.
GLAND, THYROID : Focus, dark : (Comment) 1, left (TGL)

Any remaining study plan required tissues, which have been examined, have no visible lesions

Gross Pathology - The following Tissues were Not Examined:

None

Histopathology Animal Details:

No animal details found

Histopathology Observations [Correlation]:

GLAND, PARATHYROID : (Note) One Of A Pair Available For Evaluation.
LIVER : Tension lipidosis; minimal
LIVER : Infiltration, mixed cell; minimal
NO CORRELATE : No correlating lesion [GLAND, THYROID : Focus, dark : (Comment) 1, left (G)]

Histopathology - The following Tissues were Within Normal Limits:

BRAIN; CERVIX; EYE; GANGLION, DORSAL ROOT, CERVICAL; GANGLION, DORSAL ROOT, LUMBAR; GANGLION, DORSAL ROOT, THORACIC; GLAND, ADRENAL; GLAND, PARATHYROID; GLAND, PITUITARY; GLAND, THYROID; HEART; KIDNEY; LUNG; LYMPH NODE, CERVICAL; LYMPH NODE, ILIAC; LYMPH NODE, MANDIBULAR; LYMPH NODE, MESENTERIC; MUSCLE, BICEPS FEMORIS; MUSCLE, GASTROCNEMIUS; NERVE, OPTIC; NERVE, SCIATIC; NERVE, TIBIAL; OVARY; SITE, INJECTION; SPINAL CORD, CERVICAL; SPINAL CORD, LUMBAR; SPINAL CORD, THORACIC; SPLEEN; THYMUS; UTERUS; NERVE ROOT, VENTRAL, CERVICAL; NERVE ROOT, VENTRAL, THORACIC; NERVE ROOT, VENTRAL, LUMBAR; NERVE ROOT, DORSAL, CERVICAL; NERVE ROOT, DORSAL, LUMBAR; NERVE ROOT, DORSAL, THORACIC

Histopathology - The following Tissues were Not Examined:

None

Appendix 18

Appendix 4

Individual Macroscopic and Microscopic Pathology:

5550008

Animal:	1515	Group:	1	Sex:	Female
Species:	Rat	Strain:	Sprague Dawley		
		Dose:	0 vg		
		Removal Reason:	Recovery Euthanasia		
		Study Day (Week) of Death:	29 (5)		

Gross Pathology Animal Details:

No animal details found

Gross Pathology Observations [Correlation]:

No observations found

Any remaining study plan required tissues, which have been examined, have no visible lesions

Gross Pathology - The following Tissues were Not Examined:

None

Histopathology Animal Details:

No animal details found

Histopathology Observations [Correlation]:

GLAND, PARATHYROID : (Note) One Of A Pair Available For Evaluation.
LIVER : Infiltration, mixed cell; minimal

Histopathology - The following Tissues were Within Normal Limits:

BRAIN; CERVIX; EYE; GANGLION, DORSAL ROOT, CERVICAL; GANGLION, DORSAL ROOT, LUMBAR; GANGLION, DORSAL ROOT, THORACIC; GLAND, ADRENAL; GLAND, PARATHYROID; GLAND, PITUITARY; GLAND, THYROID; HEART; KIDNEY; LUNG; LYMPH NODE, CERVICAL; LYMPH NODE, ILIAC; LYMPH NODE, MANDIBULAR; LYMPH NODE, MESENTERIC; MUSCLE, BICEPS FEMORIS; MUSCLE, GASTROCNEMIUS; NERVE, OPTIC; NERVE, SCIATIC; NERVE, TIBIAL; OVARY; SITE, INJECTION; SPINAL CORD, CERVICAL; SPINAL CORD, LUMBAR; SPINAL CORD, THORACIC; SPLEEN; THYMUS; UTERUS; NERVE ROOT, VENTRAL, CERVICAL; NERVE ROOT, VENTRAL, THORACIC; NERVE ROOT, VENTRAL, LUMBAR; NERVE ROOT, DORSAL, CERVICAL; NERVE ROOT, DORSAL, LUMBAR; NERVE ROOT, DORSAL, THORACIC

Histopathology - The following Tissues were Not Examined:

None

Individual Macroscopic and Microscopic Pathology:

5550008

Animal:	2001	Group:	2	Sex:	Male
Species:	Rat	Strain:	Sprague Dawley		
		Dose:	0.36x 10E12 vg		
		Removal Reason: Recovery Euthanasia			
		Study Day (Week) of Death: 29 (5)			

Gross Pathology Animal Details:

No animal details found

Gross Pathology Observations [Correlation]:

LYMPH NODE, MANDIBULAR : Focus, dark : (Comment) 1, left (TGL)

Any remaining study plan required tissues, which have been examined, have no visible lesions

Gross Pathology - The following Tissues were Not Examined:

None

Histopathology Animal Details:

No animal details found

Histopathology Observations [Correlation]:

GLAND, ADRENAL : Vacuolation; cortical, minimal
GLAND, PARATHYROID : (Note) One Of A Pair Available For Evaluation.
KIDNEY : Infiltration, mononuclear cell; minimal
LIVER : Infiltration, mixed cell; minimal
LYMPH NODE, MANDIBULAR : Plasmacytosis; mild [LYMPH NODE, MANDIBULAR : Focus, dark : (Comment) 1, left (G)]

Histopathology - The following Tissues were Within Normal Limits:

BRAIN; EPIDIDYMIS; EYE; GANGLION, DORSAL ROOT, CERVICAL; GANGLION, DORSAL ROOT, LUMBAR; GANGLION, DORSAL ROOT, THORACIC; GLAND, PARATHYROID; GLAND, PITUITARY; GLAND, PROSTATE; GLAND, THYROID; HEART; LUNG; LYMPH NODE, CERVICAL; LYMPH NODE, ILIAC; LYMPH NODE, MESENTERIC; MUSCLE, BICEPS FEMORIS; MUSCLE, GASTROCNEMIUS; NERVE, OPTIC; NERVE, SCIATIC; NERVE, TIBIAL; SITE, INJECTION; SPINAL CORD, CERVICAL; SPINAL CORD, LUMBAR; SPINAL CORD, THORACIC; SPLEEN; TESTIS; THYMUS; NERVE ROOT, VENTRAL, CERVICAL; NERVE ROOT, VENTRAL, THORACIC; NERVE ROOT, VENTRAL, LUMBAR; NERVE ROOT, DORSAL, CERVICAL; NERVE ROOT, DORSAL, LUMBAR; NERVE ROOT, DORSAL, THORACIC

Histopathology - The following Tissues were Not Examined:

None

Individual Macroscopic and Microscopic Pathology:

5550008

Animal: 2002	Group: 2		Sex: Male
Species: Rat	Strain: Sprague Dawley		
	Dose: 0.36x 10E12 vg		
	Removal Reason: Recovery Euthanasia		
	Study Day (Week) of Death: 29 (5)		

Gross Pathology Animal Details:

No animal details found

Gross Pathology Observations [Correlation]:

GENERAL OBSERVATIONS : (Comment) Lymph node lesion submitted in cassette E
LYMPH NODE : Discoloration, mottled : (Comment) renal, left (TGL)

Any remaining study plan required tissues, which have been examined, have no visible lesions

Gross Pathology - The following Tissues were Not Examined:

None

Histopathology Animal Details:

No animal details found

Histopathology Observations [Correlation]:

GLAND, PARATHYROID : (Note) One Of A Pair Available For Evaluation.
KIDNEY : (Note) Only Cortex Available For Evaluation.
KIDNEY : Dilatation; tubular, minimal
LIVER : Infiltration, mononuclear cell; periportal, mild
LYMPH NODE : Hemorrhage; acute, mild : (Comment) with some hemosiderophages [LYMPH NODE : Discoloration, mottled : (Comment) renal, left (G)]
SITE, INJECTION : Infiltration, mononuclear cell; minimal, leptomeninges
TESTIS : Cellular debris; minimal

Histopathology - The following Tissues were Within Normal Limits:

BRAIN; EPIDIDYMIS; EYE; GANGLION, DORSAL ROOT, CERVICAL; GANGLION, DORSAL ROOT, LUMBAR; GANGLION, DORSAL ROOT, THORACIC; GLAND, ADRENAL; GLAND, PARATHYROID; GLAND, PITUITARY; GLAND, PROSTATE; GLAND, THYROID; HEART; LUNG; LYMPH NODE, CERVICAL; LYMPH NODE, MANDIBULAR; LYMPH NODE, MESENTERIC; MUSCLE, BICEPS FEMORIS; MUSCLE, GASTROCNEMIUS; NERVE, OPTIC; NERVE, SCIATIC; NERVE, TIBIAL; SPINAL CORD, CERVICAL; SPINAL CORD, LUMBAR; SPINAL CORD, THORACIC; SPLEEN; THYMUS; NERVE ROOT, VENTRAL, CERVICAL; NERVE ROOT, VENTRAL, THORACIC; NERVE ROOT, VENTRAL, LUMBAR; NERVE ROOT, DORSAL, CERVICAL; NERVE ROOT, DORSAL, LUMBAR; NERVE ROOT, DORSAL, THORACIC

Histopathology - The following Tissues were Not Examined:

LYMPH NODE, ILIAC - Not Present In Section.

Appendix 18

Appendix 4

Individual Macroscopic and Microscopic Pathology:

5550008

Animal: 2103	Group: 2	Sex: Male
Species: Rat	Strain: Sprague Dawley<br>Dose: 0.36x 10E12 vg<br>Removal Reason: Recovery Euthanasia<br>Study Day (Week) of Death: 91 (13)	

Gross Pathology Animal Details:

No animal details found

Gross Pathology Observations [Correlation]:

No observations found

Any remaining study plan required tissues, which have been examined, have no visible lesions

Gross Pathology - The following Tissues were Not Examined:

None

Histopathology Animal Details:

No animal details found

Histopathology Observations [Correlation]:

GLAND, PROSTATE : Infiltration, mononuclear cell; mild
KIDNEY : Infiltration, mononuclear cell; minimal
KIDNEY : Chronic progressive nephropathy; minimal
LIVER : Tension lipidosis; minimal
LIVER : Infiltration, mixed cell; minimal
SITE, INJECTION : Infiltration, mononuclear cell; minimal, leptomeninges

Histopathology - The following Tissues were Within Normal Limits:

BRAIN; EPIDIDYMIS; EYE; GANGLION, DORSAL ROOT, CERVICAL; GANGLION, DORSAL ROOT, LUMBAR; GANGLION, DORSAL ROOT, THORACIC; GLAND, ADRENAL; GLAND, PARATHYROID; GLAND, PITUITARY; GLAND, THYROID; HEART; LUNG; LYMPH NODE, CERVICAL; LYMPH NODE, ILIAC; LYMPH NODE, MANDIBULAR; LYMPH NODE, MESENTERIC; MUSCLE, BICEPS FEMORIS; MUSCLE, GASTROCNEMIUS; NERVE, OPTIC; NERVE, SCIATIC; NERVE, TIBIAL; SPINAL CORD, CERVICAL; SPINAL CORD, LUMBAR; SPINAL CORD, THORACIC; SPLEEN; TESTIS; THYMUS; NERVE ROOT, VENTRAL, CERVICAL; NERVE ROOT, VENTRAL, THORACIC; NERVE ROOT, VENTRAL, LUMBAR; NERVE ROOT, DORSAL, CERVICAL; NERVE ROOT, DORSAL, LUMBAR; NERVE ROOT, DORSAL, THORACIC

Histopathology - The following Tissues were Not Examined:

None

Individual Macroscopic and Microscopic Pathology:

5550008

Animal:	2004	Group:	2	Sex:	Male
Species:	Rat	Strain:	Sprague Dawley		
		Dose:	0.36x 10E12 vg		
		Removal Reason:	Terminal Euthanasia		
		Study Day (Week) of Death:	8 (2)		

Gross Pathology Animal Details:

No animal details found

Gross Pathology Observations [Correlation]:

LYMPH NODE, ILIAC : Enlargement : (Comment) left (TGL)

Any remaining study plan required tissues, which have been examined, have no visible lesions

Gross Pathology - The following Tissues were Not Examined:

None

Histopathology Animal Details:

No animal details found

Histopathology Observations [Correlation]:

LYMPH NODE, ILIAC : Cellularity, increased; lymphoid, mild [LYMPH NODE, ILIAC : Enlargement : (Comment) left (G)]
SITE, INJECTION : Degeneration; axonal, minimal, cauda equina

Histopathology - The following Tissues were Within Normal Limits:

BRAIN; EPIDIDYMIS; EYE; GANGLION, DORSAL ROOT, CERVICAL; GANGLION, DORSAL ROOT, LUMBAR; GANGLION, DORSAL ROOT, THORACIC; GLAND, ADRENAL; GLAND, PARATHYROID; GLAND, PITUITARY; GLAND, PROSTATE; GLAND, THYROID; HEART; KIDNEY; LIVER; LUNG; LYMPH NODE, CERVICAL; LYMPH NODE, MANDIBULAR; LYMPH NODE, MESENTERIC; MUSCLE, BICEPS FEMORIS; MUSCLE, GASTROCNEMIUS; NERVE, OPTIC; NERVE, SCIATIC; NERVE, TIBIAL; SPINAL CORD, CERVICAL; SPINAL CORD, LUMBAR; SPINAL CORD, THORACIC; SPLEEN; TESTIS; THYMUS; NERVE ROOT, VENTRAL, CERVICAL; NERVE ROOT, VENTRAL, THORACIC; NERVE ROOT, VENTRAL, LUMBAR; NERVE ROOT, DORSAL, CERVICAL; NERVE ROOT, DORSAL, LUMBAR; NERVE ROOT, DORSAL, THORACIC

Histopathology - The following Tissues were Not Examined:

None

Individual Macroscopic and Microscopic Pathology:

5550008

Animal:	2005	Group:	2	Sex:	Male
Species:	Rat	Strain:	Sprague Dawley		
		Dose:	0.36x 10E12 vg		
		Removal Reason: Recovery Euthanasia			
		Study Day (Week) of Death: 91 (13)			

Gross Pathology Animal Details:

No animal details found

Gross Pathology Observations [Correlation]:

GENERAL OBSERVATIONS : (Comment) Lymph node lesion submitted with pancreas.
LYMPH NODE : Enlargement : (Comment) pancreatic (TGL)

Any remaining study plan required tissues, which have been examined, have no visible lesions

Gross Pathology - The following Tissues were Not Examined:

None

Histopathology Animal Details:

No animal details found

Histopathology Observations [Correlation]:

GLAND, PROSTATE : Infiltration, mononuclear cell; minimal
HEART : Infiltration, mononuclear cell/degeneration, myofiber; minimal
KIDNEY : Infiltration, mononuclear cell; minimal
KIDNEY : Chronic progressive nephropathy; mild
LIVER : Vacuolation; hepatocellular, periportal, minimal
LIVER : Infiltration, mixed cell; minimal
LIVER : Infiltration, mononuclear cell; periportal, minimal
NO CORRELATE : No correlating lesion [LYMPH NODE : Enlargement : (Comment) pancreatic (G)]

Histopathology - The following Tissues were Within Normal Limits:

BRAIN; EPIDIDYMIS; EYE; GANGLION, DORSAL ROOT, CERVICAL; GANGLION, DORSAL ROOT, LUMBAR; GANGLION, DORSAL ROOT, THORACIC; GLAND, ADRENAL; GLAND, PARATHYROID; GLAND, PITUITARY; GLAND, THYROID; LUNG; LYMPH NODE, CERVICAL; LYMPH NODE, ILIAC; LYMPH NODE, MANDIBULAR; LYMPH NODE, MESENTERIC; MUSCLE, BICEPS FEMORIS; MUSCLE, GASTROCNEMIUS; NERVE, OPTIC; NERVE, SCIATIC; NERVE, TIBIAL; SITE, INJECTION; SPINAL CORD, CERVICAL; SPINAL CORD, LUMBAR; SPINAL CORD, THORACIC; SPLEEN; TESTIS; THYMUS; NERVE ROOT, VENTRAL, CERVICAL; NERVE ROOT, VENTRAL, THORACIC; NERVE ROOT, VENTRAL, LUMBAR; NERVE ROOT, DORSAL, CERVICAL; NERVE ROOT, DORSAL, LUMBAR; NERVE ROOT, DORSAL, THORACIC

Histopathology - The following Tissues were Not Examined:

None

Appendix 18

Appendix 4

Individual Macroscopic and Microscopic Pathology:

5550008

Animal: 2006	Group: 2	Sex: Male
Species: Rat	Strain: Sprague Dawley<br>Dose: 0.36x 10E12 vg<br>Removal Reason: Recovery Euthanasia<br>Study Day (Week) of Death: 91 (13)	

Gross Pathology Animal Details:

No animal details found

Gross Pathology Observations [Correlation]:

GENERAL OBSERVATIONS : (Comment) Jejunum lesion submitted in cass D
SMALL INTESTINE, JEJUNUM : Diverticulum : (Comment) 1, caudal (TGL)

Any remaining study plan required tissues, which have been examined, have no visible lesions

Gross Pathology - The following Tissues were Not Examined:

None

Histopathology Animal Details:

No animal details found

Histopathology Observations [Correlation]:

GLAND, ADRENAL : Vacuolation; cortical, minimal
HEART : Infiltration, mononuclear cell/degeneration, myofiber; minimal
LIVER : Vacuolation; hepatocellular, minimal : (Comment) random multifocal, mostly macrovesicular
NERVE, SCIATIC : Degeneration; axonal, minimal
NERVE, TIBIAL : Degeneration; axonal, minimal
NO CORRELATE : No correlating lesion [SMALL INTESTINE, JEJUNUM : Diverticulum : (Comment) 1, caudal (G)]
NERVE ROOT, DORSAL, LUMBAR : Degeneration; axonal, minimal

Histopathology - The following Tissues were Within Normal Limits:

BRAIN; EPIDIDYMIS; EYE; GANGLION, DORSAL ROOT, CERVICAL; GANGLION, DORSAL ROOT, LUMBAR; GANGLION, DORSAL ROOT, THORACIC; GLAND, PARATHYROID; GLAND, PITUITARY; GLAND, PROSTATE; GLAND, THYROID; KIDNEY; LUNG; LYMPH NODE, CERVICAL; LYMPH NODE, ILIAC; LYMPH NODE, MANDIBULAR; LYMPH NODE, MESENTERIC; MUSCLE, BICEPS FEMORIS; MUSCLE, GASTROCNEMIUS; NERVE, OPTIC; SITE, INJECTION; SMALL INTESTINE, JEJUNUM; SPINAL CORD, CERVICAL; SPINAL CORD, LUMBAR; SPINAL CORD, THORACIC; SPLEEN; TESTIS; THYMUS; NERVE ROOT, VENTRAL, CERVICAL; NERVE ROOT, VENTRAL, THORACIC; NERVE ROOT, VENTRAL, LUMBAR; NERVE ROOT, DORSAL, CERVICAL; NERVE ROOT, DORSAL, THORACIC

Histopathology - The following Tissues were Not Examined:

None

Individual Macroscopic and Microscopic Pathology:

5550008

Animal:	2007	Group:	2	Sex:	Male
Species:	Rat	Strain:	Sprague Dawley		
		Dose:	0.36x 10E12 vg		
		Removal Reason: Recovery Euthanasia			
		Study Day (Week) of Death: 91 (13)			

Gross Pathology Animal Details:

No animal details found

Gross Pathology Observations [Correlation]:

No observations found

Any remaining study plan required tissues, which have been examined, have no visible lesions

Gross Pathology - The following Tissues were Not Examined:

None

Histopathology Animal Details:

No animal details found

Histopathology Observations [Correlation]:

GLAND, PROSTATE : Infiltration, mononuclear cell; minimal
KIDNEY : Chronic progressive nephropathy; minimal
LIVER : Vacuolation; hepatocellular, periportal, minimal
LIVER : Infiltration, mononuclear cell; periportal, minimal
NERVE, SCIATIC : Degeneration; axonal, minimal
NERVE, TIBIAL : Degeneration; axonal, minimal

Histopathology - The following Tissues were Within Normal Limits:

BRAIN; EPIDIDYMIS; EYE; GANGLION, DORSAL ROOT, CERVICAL; GANGLION, DORSAL ROOT, LUMBAR; GANGLION, DORSAL ROOT, THORACIC; GLAND, ADRENAL; GLAND, PARATHYROID; GLAND, PITUITARY; GLAND, THYROID; HEART; LUNG; LYMPH NODE, CERVICAL; LYMPH NODE, ILIAC; LYMPH NODE, MANDIBULAR; LYMPH NODE, MESENTERIC; MUSCLE, BICEPS FEMORIS; MUSCLE, GASTROCNEMIUS; NERVE, OPTIC; SITE, INJECTION; SPINAL CORD, CERVICAL; SPINAL CORD, LUMBAR; SPINAL CORD, THORACIC; SPLEEN; TESTIS; THYMUS; NERVE ROOT, VENTRAL, CERVICAL; NERVE ROOT, VENTRAL, THORACIC; NERVE ROOT, VENTRAL, LUMBAR; NERVE ROOT, DORSAL, CERVICAL; NERVE ROOT, DORSAL, LUMBAR; NERVE ROOT, DORSAL, THORACIC

Histopathology - The following Tissues were Not Examined:

None

Appendix 18

Appendix 4

Individual Macroscopic and Microscopic Pathology:

5550008

Animal:	2008	Group:	2	Sex:	Male
Species:	Rat	Strain:	Sprague Dawley		
		Dose:	0.36x 10E12 vg		
		Removal Reason: Recovery Euthanasia			
		Study Day (Week) of Death: 91 (13)			

Gross Pathology Animal Details:

No animal details found

Gross Pathology Observations [Correlation]:

LIVER : Focus, pale : (Comment) 1, fissure, right medial (TGL)
STOMACH : Focus, depressed : (Comment) dark, 1, mucosa, glandular (TGL)

Any remaining study plan required tissues, which have been examined, have no visible lesions

Gross Pathology - The following Tissues were Not Examined:

None

Histopathology Animal Details:

No animal details found

Histopathology Observations [Correlation]:

GLAND, PROSTATE : Infiltration, mononuclear cell; mild
GLAND, THYROID : Hypertrophy; bilateral, moderate, follicle
KIDNEY : Infiltration, mononuclear cell; minimal
KIDNEY : Dilatation; tubular, minimal : (Comment) with hyaline eosinophilic material in dilated tubules (two, medullary region)
LIVER : Vacuolation; hepatocellular, periportal, mild
LIVER : Tension lipidosis; minimal [LIVER : Focus, pale : (Comment) 1, fissure, right medial (G)]
LIVER : Infiltration, mixed cell; minimal
NO CORRELATE : No correlating lesion [STOMACH : Focus, depressed : (Comment) dark, 1, mucosa, glandular (G)]

Histopathology - The following Tissues were Within Normal Limits:

BRAIN; EPIDIDYMIS; EYE; GANGLION, DORSAL ROOT, CERVICAL; GANGLION, DORSAL ROOT, LUMBAR; GANGLION, DORSAL ROOT, THORACIC; GLAND, ADRENAL; GLAND, PARATHYROID; GLAND, PITUITARY; HEART; LUNG; LYMPH NODE, CERVICAL; LYMPH NODE, ILIAC; LYMPH NODE, MANDIBULAR; LYMPH NODE, MESENTERIC; MUSCLE, BICEPS FEMORIS; MUSCLE, GASTROCNEMIUS; NERVE, OPTIC; NERVE, SCIATIC; NERVE, TIBIAL; SITE, INJECTION; SPINAL CORD, CERVICAL; SPINAL CORD, LUMBAR; SPINAL CORD, THORACIC; SPLEEN; STOMACH; TESTIS; THYMUS; NERVE ROOT, VENTRAL, CERVICAL; NERVE ROOT, VENTRAL, THORACIC; NERVE ROOT, VENTRAL, LUMBAR; NERVE ROOT, DORSAL, CERVICAL; NERVE ROOT, DORSAL, LUMBAR; NERVE ROOT, DORSAL, THORACIC

Histopathology - The following Tissues were Not Examined:

None

Appendix 18

Appendix 4

Individual Macroscopic and Microscopic Pathology:

5550008

Animal:	2009	Group:	2	Sex:	Male
Species:	Rat	Strain:	Sprague Dawley		
		Dose:	0.36x 10E12 vg		
		Removal Reason:	Terminal Euthanasia		
		Study Day (Week) of Death:	8 (2)		

Gross Pathology Animal Details:

No animal details found

Gross Pathology Observations [Correlation]:

No observations found

Any remaining study plan required tissues, which have been examined, have no visible lesions

Gross Pathology - The following Tissues were Not Examined:

None

Histopathology Animal Details:

No animal details found

Histopathology Observations [Correlation]:

KIDNEY : Infiltration, mononuclear cell; minimal
KIDNEY : Chronic progressive nephropathy; minimal
LIVER : Infiltration, mononuclear cell; periportal, minimal
NERVE, TIBIAL : Degeneration; axonal, minimal

Histopathology - The following Tissues were Within Normal Limits:

BRAIN; EPIDIDYMIS; EYE; GANGLION, DORSAL ROOT, CERVICAL; GANGLION, DORSAL ROOT, LUMBAR; GANGLION, DORSAL ROOT, THORACIC; GLAND, ADRENAL; GLAND, PARATHYROID; GLAND, PITUITARY; GLAND, PROSTATE; GLAND, THYROID; HEART; LUNG; LYMPH NODE, CERVICAL; LYMPH NODE, ILIAC; LYMPH NODE, MANDIBULAR; LYMPH NODE, MESENTERIC; MUSCLE, BICEPS FEMORIS; MUSCLE, GASTROCNEMIUS; NERVE, OPTIC; NERVE, SCIATIC; SITE, INJECTION; SPINAL CORD, CERVICAL; SPINAL CORD, LUMBAR; SPINAL CORD, THORACIC; SPLEEN; TESTIS; THYMUS; NERVE ROOT, VENTRAL, CERVICAL; NERVE ROOT, VENTRAL, THORACIC; NERVE ROOT, VENTRAL, LUMBAR; NERVE ROOT, DORSAL, CERVICAL; NERVE ROOT, DORSAL, LUMBAR; NERVE ROOT, DORSAL, THORACIC

Histopathology - The following Tissues were Not Examined:

None

Individual Macroscopic and Microscopic Pathology:

5550008

Animal: 2010	Group: 2	Sex: Male
Species: Rat	Strain: Sprague Dawley<br>Dose: 0.36x 10E12 vg<br>Removal Reason: Terminal Euthanasia<br>Study Day (Week) of Death: 8 (2)	

Gross Pathology Animal Details:

No animal details found

Gross Pathology Observations [Correlation]:

THYMUS : Focus, dark : (Comment) 4 (TGL)

Any remaining study plan required tissues, which have been examined, have no visible lesions

Gross Pathology - The following Tissues were Not Examined:

None

Histopathology Animal Details:

No animal details found

Histopathology Observations [Correlation]:

EPIDIDYMIS : Peripuberty
GLAND, ADRENAL : Vacuolation; cortical, mild
GLAND, PARATHYROID : (Note) One Of A Pair Available For Evaluation.
KIDNEY : Dilatation; tubular, mild : (Comment) with fibrosis and mononuclear cell infiltration
LYMPH NODE, CERVICAL : Hemorrhage; acute, mild
SITE, INJECTION : Degeneration; axonal, minimal, cauda equina
TESTIS : Peripuberty
NO CORRELATE : No correlating lesion [THYMUS : Focus, dark : (Comment) 4 (G)]

Histopathology - The following Tissues were Within Normal Limits:

BRAIN; EYE; GANGLION, DORSAL ROOT, CERVICAL; GANGLION, DORSAL ROOT, LUMBAR; GANGLION, DORSAL ROOT, THORACIC; GLAND, PARATHYROID; GLAND, PITUITARY; GLAND, PROSTATE; GLAND, THYROID; HEART; LIVER; LUNG; LYMPH NODE, ILIAC; LYMPH NODE, MANDIBULAR; LYMPH NODE, MESENTERIC; MUSCLE, BICEPS FEMORIS; MUSCLE, GASTROCNEMIUS; NERVE, OPTIC; NERVE, SCIATIC; NERVE, TIBIAL; SPINAL CORD, CERVICAL; SPINAL CORD, LUMBAR; SPINAL CORD, THORACIC; SPLEEN; THYMUS; NERVE ROOT, VENTRAL, CERVICAL; NERVE ROOT, VENTRAL, THORACIC; NERVE ROOT, VENTRAL, LUMBAR; NERVE ROOT, DORSAL, CERVICAL; NERVE ROOT, DORSAL, LUMBAR; NERVE ROOT, DORSAL, THORACIC

Histopathology - The following Tissues were Not Examined:

None

Appendix 18

Appendix 4

Individual Macroscopic and Microscopic Pathology:

5550008

Animal: 2011	Group: 2	Sex: Male
Species: Rat	Strain: Sprague Dawley	
	Dose: 0.36x 10E12 vg	
	Removal Reason: Recovery Euthanasia	
	Study Day (Week) of Death: 29 (5)	

Gross Pathology Animal Details:

No animal details found

Gross Pathology Observations [Correlation]:

GLAND, PITUITARY : Discoloration, dark (TGL)

Any remaining study plan required tissues, which have been examined, have no visible lesions

Gross Pathology - The following Tissues were Not Examined:

None

Histopathology Animal Details:

No animal details found

Histopathology Observations [Correlation]:

GLAND, PROSTATE : Vacuolation; neuronal, minimal, ganglion
KIDNEY : Chronic progressive nephropathy; minimal
LIVER : Vacuolation; hepatocellular, periportal, minimal
LIVER : Infiltration, mixed cell; minimal
SITE, INJECTION : Infiltration, mononuclear cell; minimal, leptomeninges
SITE, INJECTION : Degeneration; axonal, minimal, cauda equina
NO CORRELATE : No correlating lesion [GLAND, PITUITARY : Discoloration, dark (G)]

Histopathology - The following Tissues were Within Normal Limits:

BRAIN; EPIDIDYMIS; EYE; GANGLION, DORSAL ROOT, CERVICAL; GANGLION, DORSAL ROOT, LUMBAR; GANGLION, DORSAL ROOT, THORACIC; GLAND, ADRENAL; GLAND, PITUITARY; GLAND, THYROID; HEART; LUNG; LYMPH NODE, CERVICAL; LYMPH NODE, ILIAC; LYMPH NODE, MANDIBULAR; LYMPH NODE, MESENTERIC; MUSCLE, BICEPS FEMORIS; MUSCLE, GASTROCNEMIUS; NERVE, OPTIC; NERVE, SCIATIC; NERVE, TIBIAL; SPINAL CORD, CERVICAL; SPINAL CORD, LUMBAR; SPINAL CORD, THORACIC; SPLEEN; TESTIS; THYMUS; NERVE ROOT, VENTRAL, CERVICAL; NERVE ROOT, VENTRAL, THORACIC; NERVE ROOT, VENTRAL, LUMBAR; NERVE ROOT, DORSAL, CERVICAL; NERVE ROOT, DORSAL, LUMBAR; NERVE ROOT, DORSAL, THORACIC

Histopathology - The following Tissues were Not Examined:

GLAND, PARATHYROID - Not Present In Section.

Appendix 18

Appendix 4

Individual Macroscopic and Microscopic Pathology:

5550008

Animal: 2012	Group: 2	Sex: Male
Species: Rat	Strain: Sprague Dawley	
	Dose: 0.36x 10E12 vg	
	Removal Reason: Recovery Euthanasia	
	Study Day (Week) of Death: 29 (5)	

Gross Pathology Animal Details:

No animal details found

Gross Pathology Observations [Correlation]:

GENERAL OBSERVATIONS : (Comment) Pituitary submitted in two pieces.

Any remaining study plan required tissues, which have been examined, have no visible lesions

Gross Pathology - The following Tissues were Not Examined:

None

Histopathology Animal Details:

No animal details found

Histopathology Observations [Correlation]:

EPIDIDYMIS : Cellular debris; bilateral, minimal
GLAND, PITUITARY : (Note) Only Pars Distalis Available For Evaluation.
KIDNEY : Infiltration, mononuclear cell; minimal
LIVER : Infiltration, mixed cell; minimal
SITE, INJECTION : Degeneration; axonal, minimal, cauda equina
TESTIS : Degeneration/atrophy; unilateral, mild, seminiferous tubule
NERVE ROOT, DORSAL, LUMBAR : Degeneration; axonal, minimal

Histopathology - The following Tissues were Within Normal Limits:

BRAIN; EYE; GANGLION, DORSAL ROOT, CERVICAL; GANGLION, DORSAL ROOT, LUMBAR; GANGLION, DORSAL ROOT, THORACIC; GLAND, ADRENAL; GLAND, PARATHYROID; GLAND, PITUITARY; GLAND, PROSTATE; GLAND, THYROID; HEART; LUNG; LYMPH NODE, CERVICAL; LYMPH NODE, ILIAC; LYMPH NODE, MANDIBULAR; LYMPH NODE, MESENTERIC; MUSCLE, BICEPS FEMORIS; MUSCLE, GASTROCNEMIUS; NERVE, OPTIC; NERVE, SCIATIC; NERVE, TIBIAL; SPINAL CORD, CERVICAL; SPINAL CORD, LUMBAR; SPINAL CORD, THORACIC; SPLEEN; THYMUS; NERVE ROOT, VENTRAL, CERVICAL; NERVE ROOT, VENTRAL, THORACIC; NERVE ROOT, VENTRAL, LUMBAR; NERVE ROOT, DORSAL, CERVICAL; NERVE ROOT, DORSAL, THORACIC

Histopathology - The following Tissues were Not Examined:

None

Individual Macroscopic and Microscopic Pathology:

5550008

Animal: 2013	Group: 2	Sex: Male
Species: Rat	Strain: Sprague Dawley<br>Dose: 0.36x 10E12 vg<br>Removal Reason: Recovery Euthanasia<br>Study Day (Week) of Death: 29 (5)	

Gross Pathology Animal Details:

No animal details found

Gross Pathology Observations [Correlation]:

No observations found

Any remaining study plan required tissues, which have been examined, have no visible lesions

Gross Pathology - The following Tissues were Not Examined:

None

Histopathology Animal Details:

No animal details found

Histopathology Observations [Correlation]:

GLAND, PARATHYROID : (Note) One Of A Pair Available For Evaluation.
GLAND, PITUITARY : (Note) Only Pars Distalis Available For Evaluation.
KIDNEY : Infiltration, mononuclear cell; minimal
KIDNEY : Chronic progressive nephropathy; mild
LIVER : Infiltration, mixed cell; minimal

Histopathology - The following Tissues were Within Normal Limits:

BRAIN; EPIDIDYMIS; EYE; GANGLION, DORSAL ROOT, CERVICAL; GANGLION, DORSAL ROOT, LUMBAR; GANGLION, DORSAL ROOT, THORACIC; GLAND, ADRENAL; GLAND, PARATHYROID; GLAND, PITUITARY; GLAND, PROSTATE; GLAND, THYROID; HEART; LUNG; LYMPH NODE, CERVICAL; LYMPH NODE, ILIAC; LYMPH NODE, MANDIBULAR; LYMPH NODE, MESENTERIC; MUSCLE, BICEPS FEMORIS; MUSCLE, GASTROCNEMIUS; NERVE, OPTIC; NERVE, SCIATIC; NERVE, TIBIAL; SITE, INJECTION; SPINAL CORD, CERVICAL; SPINAL CORD, LUMBAR; SPINAL CORD, THORACIC; SPLEEN; TESTIS; THYMUS; NERVE ROOT, VENTRAL, CERVICAL; NERVE ROOT, VENTRAL, THORACIC; NERVE ROOT, VENTRAL, LUMBAR; NERVE ROOT, DORSAL, CERVICAL; NERVE ROOT, DORSAL, LUMBAR; NERVE ROOT, DORSAL, THORACIC

Histopathology - The following Tissues were Not Examined:

None

Appendix 18

Appendix 4

Individual Macroscopic and Microscopic Pathology:

5550008

Animal: 2014	Group: 2	Sex: Male
Species: Rat	Strain: Sprague Dawley<br>Dose: 0.36x 10E12 vg<br>Removal Reason: Terminal Euthanasia<br>Study Day (Week) of Death: 8 (2)	

Gross Pathology Animal Details:

No animal details found

Gross Pathology Observations [Correlation]:

LIVER : Focus, pale : (Comment) 1, fissure, right medial. (TGL)
THYMUS : Focus, dark : (Comment) >10. (TGL)

Any remaining study plan required tissues, which have been examined, have no visible lesions

Gross Pathology - The following Tissues were Not Examined:

None

Histopathology Animal Details:

No animal details found

Histopathology Observations [Correlation]:

KIDNEY : Dilatation; tubular, mild : (Comment) with peritubular fibrosis
LIVER : Vacuolation; hepatocellular, periportal, minimal
LIVER : Tension lipidosis; minimal [LIVER : Focus, pale : (Comment) 1, fissure, right medial. (G)]
NO CORRELATE : No correlating lesion [THYMUS : Focus, dark : (Comment) >10. (G)]

Histopathology - The following Tissues were Within Normal Limits:

BRAIN; EPIDIDYMIS; EYE; GANGLION, DORSAL ROOT, CERVICAL; GANGLION, DORSAL ROOT, LUMBAR; GANGLION, DORSAL ROOT, THORACIC; GLAND, ADRENAL; GLAND, PARATHYROID; GLAND, PITUITARY; GLAND, PROSTATE; GLAND, THYROID; HEART; LUNG; LYMPH NODE, CERVICAL; LYMPH NODE, ILIAC; LYMPH NODE, MANDIBULAR; LYMPH NODE, MESENTERIC; MUSCLE, BICEPS FEMORIS; MUSCLE, GASTROCNEMIUS; NERVE, OPTIC; NERVE, SCIATIC; NERVE, TIBIAL; SITE, INJECTION; SPINAL CORD, CERVICAL; SPINAL CORD, LUMBAR; SPINAL CORD, THORACIC; SPLEEN; TESTIS; THYMUS; NERVE ROOT, VENTRAL, CERVICAL; NERVE ROOT, VENTRAL, THORACIC; NERVE ROOT, VENTRAL, LUMBAR; NERVE ROOT, DORSAL, CERVICAL; NERVE ROOT, DORSAL, LUMBAR; NERVE ROOT, DORSAL, THORACIC

Histopathology - The following Tissues were Not Examined:

None

Appendix 18

Appendix 4

Individual Macroscopic and Microscopic Pathology:

5550008

Animal: 2015	Group: 2	Sex: Male
Species: Rat	Strain: Sprague Dawley<br>Dose: 0.36x 10E12 vg<br>Removal Reason: Terminal Euthanasia<br>Study Day (Week) of Death: 8 (2)	

Gross Pathology Animal Details:

No animal details found

Gross Pathology Observations [Correlation]:

GENERAL OBSERVATIONS : (Comment) Right lymph node deep cervical not submitted for histopathological evaluation (frozen).
LYMPH NODE, CERVICAL : Focus, dark : (Comment) 1, bilateral (TGL)

Any remaining study plan required tissues, which have been examined, have no visible lesions

Gross Pathology - The following Tissues were Not Examined:

None

Histopathology Animal Details:

No animal details found

Histopathology Observations [Correlation]:

GLAND, ADRENAL : Vacuolation; cortical, minimal
GLAND, PARATHYROID : (Note) One Of A Pair Available For Evaluation.
KIDNEY : Infiltration, mononuclear cell; minimal
KIDNEY : Chronic progressive nephropathy; minimal
LYMPH NODE, CERVICAL : Hemorrhage; acute, mild [LYMPH NODE, CERVICAL : Focus, dark : (Comment) 1, bilateral (G)]
SITE, INJECTION : Infiltration, mononuclear cell; minimal, leptomeninges
SPINAL CORD, CERVICAL : Hyperplasia; minimal, leptomeninges

Histopathology - The following Tissues were Within Normal Limits:

BRAIN; EPIDIDYMIS; EYE; GANGLION, DORSAL ROOT, CERVICAL; GANGLION, DORSAL ROOT, LUMBAR; GANGLION, DORSAL ROOT, THORACIC; GLAND, PARATHYROID; GLAND, PITUITARY; GLAND, PROSTATE; GLAND, THYROID; LIVER; LUNG; LYMPH NODE, ILIAC; LYMPH NODE, MANDIBULAR; LYMPH NODE, MESENTERIC; MUSCLE, BICEPS FEMORIS; MUSCLE, GASTROCNEMIUS; NERVE, OPTIC; NERVE, SCIATIC; NERVE, TIBIAL; SPINAL CORD, LUMBAR; SPINAL CORD, THORACIC; SPLEEN; TESTIS; THYMUS; NERVE ROOT, VENTRAL, CERVICAL; NERVE ROOT, VENTRAL, THORACIC; NERVE ROOT, VENTRAL, LUMBAR; NERVE ROOT, DORSAL, CERVICAL; NERVE ROOT, DORSAL, LUMBAR; NERVE ROOT, DORSAL, THORACIC

Histopathology - The following Tissues were Not Examined:

HEART - Not Present In Section.

Individual Macroscopic and Microscopic Pathology:

5550008

Animal:	2501	Group:	2	Sex:	Female
Species:	Rat	Strain:	Sprague Dawley		
		Dose:	0.36x 10E12 vg		
		Removal Reason: Recovery Euthanasia			
		Study Day (Week) of Death: 29 (5)			

Gross Pathology Animal Details:

No animal details found

Gross Pathology Observations [Correlation]:

GENERAL OBSERVATIONS : (Comment) pituitary submitted in two pieces
LYMPH NODE, ILIAC : Discoloration, mottled : (Comment) left (TGL)
THYMUS : Focus, dark : (Comment) >10 (TGL)

Any remaining study plan required tissues, which have been examined, have no visible lesions

Gross Pathology - The following Tissues were Not Examined:

None

Histopathology Animal Details:

No animal details found

Histopathology Observations [Correlation]:

LIVER : Infiltration, mononuclear cell; periportal, minimal
LYMPH NODE, ILIAC : Hemorrhage; acute, mild [LYMPH NODE, ILIAC : Discoloration, mottled : (Comment) left (G)]
SITE, INJECTION : Infiltration, mononuclear cell; minimal, leptomeninges
SPINAL CORD, THORACIC : Infiltration, mononuclear cell; minimal, adipose tissue : (Comment) and skeletal muscle adjacent to vertebra
NO CORRELATE : No correlating lesion [THYMUS : Focus, dark : (Comment) >10 (G)]

Histopathology - The following Tissues were Within Normal Limits:

BRAIN; CERVIX; EYE; GANGLION, DORSAL ROOT, CERVICAL; GANGLION, DORSAL ROOT, LUMBAR; GANGLION, DORSAL ROOT, THORACIC; GLAND, ADRENAL; GLAND, PITUITARY; GLAND, THYROID; HEART; KIDNEY; LUNG; LYMPH NODE, CERVICAL; LYMPH NODE, MANDIBULAR; LYMPH NODE, MESENTERIC; MUSCLE, BICEPS FEMORIS; MUSCLE, GASTROCNEMIUS; NERVE, OPTIC; NERVE, SCIATIC; NERVE, TIBIAL; OVARY; SPINAL CORD, CERVICAL; SPINAL CORD, LUMBAR; SPLEEN; THYMUS; UTERUS; NERVE ROOT, VENTRAL, CERVICAL; NERVE ROOT, VENTRAL, THORACIC; NERVE ROOT, VENTRAL, LUMBAR; NERVE ROOT, DORSAL, CERVICAL; NERVE ROOT, DORSAL, LUMBAR; NERVE ROOT, DORSAL, THORACIC

Histopathology - The following Tissues were Not Examined:

GLAND, PARATHYROID - Not Present In Section.

Appendix 18

Appendix 4

Individual Macroscopic and Microscopic Pathology:

5550008

Animal:	2502	Group:	2	Sex:	Female
Species:	Rat	Strain:	Sprague Dawley		
		Dose:	0.36x 10E12 vg		
		Removal Reason: Recovery Euthanasia			
		Study Day (Week) of Death: 29 (5)			

Gross Pathology Animal Details:

No animal details found

Gross Pathology Observations [Correlation]:

LYMPH NODE, MANDIBULAR : Enlargement : (Comment) right (TGL)

Any remaining study plan required tissues, which have been examined, have no visible lesions

Gross Pathology - The following Tissues were Not Examined:

None

Histopathology Animal Details:

No animal details found

Histopathology Observations [Correlation]:

GLAND, PARATHYROID : (Note) One Of A Pair Available For Evaluation.
KIDNEY : Dilatation; tubular, minimal
KIDNEY : Chronic progressive nephropathy; minimal
LIVER : Vacuolation; hepatocellular, periportal, minimal
LIVER : Tension lipidosis; minimal
LIVER : Infiltration, mixed cell; minimal
SITE, INJECTION : Infiltration, mononuclear cell; minimal, leptomeninges
NO CORRELATE : No correlating lesion [LYMPH NODE, MANDIBULAR : Enlargement : (Comment) right (G)]

Histopathology - The following Tissues were Within Normal Limits:

BRAIN; CERVIX; EYE; GANGLION, DORSAL ROOT, CERVICAL; GANGLION, DORSAL ROOT, LUMBAR; GANGLION, DORSAL ROOT, THORACIC; GLAND, ADRENAL; GLAND, PARATHYROID; GLAND, PITUITARY; GLAND, THYROID; HEART; LUNG; LYMPH NODE, CERVICAL; LYMPH NODE, ILIAC; LYMPH NODE, MANDIBULAR; LYMPH NODE, MESENTERIC; MUSCLE, BICEPS FEMORIS; MUSCLE, GASTROCNEMIUS; NERVE, OPTIC; NERVE, SCIATIC; NERVE, TIBIAL; OVARY; SPINAL CORD, CERVICAL; SPINAL CORD, LUMBAR; SPINAL CORD, THORACIC; SPLEEN; THYMUS; UTERUS; NERVE ROOT, VENTRAL, CERVICAL; NERVE ROOT, VENTRAL, THORACIC; NERVE ROOT, VENTRAL, LUMBAR; NERVE ROOT, DORSAL, CERVICAL; NERVE ROOT, DORSAL, LUMBAR; NERVE ROOT, DORSAL, THORACIC

Histopathology - The following Tissues were Not Examined:

None

Individual Macroscopic and Microscopic Pathology:

5550008

Animal: 2503	Group: 2	Sex: Female
Species: Rat	Strain: Sprague Dawley<br>Dose: 0.36x 10E12 vg<br>Removal Reason: Recovery Euthanasia<br>Study Day (Week) of Death: 91 (13)	

Gross Pathology Animal Details:

No animal details found

Gross Pathology Observations [Correlation]:

GENERAL OBSERVATIONS : (Comment) Lymph node lesion submitted with pancreas
LYMPH NODE : Discoloration, mottled : (Comment) Pancreatic (TGL)

Any remaining study plan required tissues, which have been examined, have no visible lesions

Gross Pathology - The following Tissues were Not Examined:

None

Histopathology Animal Details:

No animal details found

Histopathology Observations [Correlation]:

GANGLION, DORSAL ROOT, LUMBAR : Infiltration, mononuclear cell; minimal
GLAND, PARATHYROID : (Note) One Of A Pair Available For Evaluation.
GLAND, PITUITARY : (Note) Only Pars Distalis Available For Evaluation.
KIDNEY : Dilatation; tubular, mild : (Comment) with hyaline material
LIVER : Infiltration, mixed cell; minimal
NERVE, SCIATIC : Degeneration; axonal, minimal
NERVE, TIBIAL : Degeneration; axonal, minimal
SITE, INJECTION : Infiltration, mononuclear cell; minimal, leptomeninges
NO CORRELATE : No correlating lesion [LYMPH NODE : Discoloration, mottled : (Comment) Pancreatic (G)]
NERVE ROOT, DORSAL, LUMBAR : Degeneration; axonal, minimal

Histopathology - The following Tissues were Within Normal Limits:

BRAIN; CERVIX; EYE; GANGLION, DORSAL ROOT, CERVICAL; GANGLION, DORSAL ROOT, THORACIC; GLAND, ADRENAL; GLAND, PARATHYROID; GLAND, PITUITARY; GLAND, THYROID; HEART; LUNG; LYMPH NODE; LYMPH NODE, CERVICAL; LYMPH NODE, ILIAC; LYMPH NODE, MANDIBULAR; LYMPH NODE, MESENTERIC; MUSCLE, BICEPS FEMORIS; MUSCLE, GASTROCNEMIUS; NERVE, OPTIC; OVARY; SPINAL CORD, CERVICAL; SPINAL CORD, LUMBAR; SPINAL CORD, THORACIC; SPLEEN; THYMUS; UTERUS; NERVE ROOT, VENTRAL, CERVICAL; NERVE ROOT, VENTRAL, THORACIC; NERVE ROOT, VENTRAL, LUMBAR; NERVE ROOT, DORSAL, CERVICAL; NERVE ROOT, DORSAL, THORACIC

Histopathology - The following Tissues were Not Examined:

None

Individual Macroscopic and Microscopic Pathology:

5550008

Animal: 2504	Group: 2	Sex: Female
Species: Rat	Strain: Sprague Dawley	
	Dose: 0.36x 10E12 vg	
	Removal Reason: Recovery Euthanasia	
	Study Day (Week) of Death: 91 (13)	

Gross Pathology Animal Details:

No animal details found

Gross Pathology Observations [Correlation]:

GENERAL OBSERVATIONS : (Comment) Liver lesion submitted cassette C. Lymph node deep cervical left submitted as representative section of lesion.
LIVER : Focus, pale : (Comment) 1, near hilus, right lateral (TGL)
LYMPH NODE, CERVICAL : Discoloration, dark : (Comment) bilateral (TGL)
LYMPH NODE, MANDIBULAR : Discoloration, dark : (Comment) bilateral (TGL)

Any remaining study plan required tissues, which have been examined, have no visible lesions

Gross Pathology - The following Tissues were Not Examined:

None

Histopathology Animal Details:

No animal details found

Histopathology Observations [Correlation]:

GLAND, PARATHYROID : (Note) One Of A Pair Available For Evaluation.
KIDNEY : Dilatation; tubular, mild : (Comment) with hyaline eosinophilic material
LIVER : Infiltration, mixed cell; minimal
LYMPH NODE, CERVICAL : Hemorrhage; acute, mild [LYMPH NODE, CERVICAL : Discoloration, dark : (Comment) bilateral (G)]
LYMPH NODE, MANDIBULAR : Hemorrhage; acute, moderate [LYMPH NODE, MANDIBULAR : Discoloration, dark : (Comment) bilateral (G)]
NO CORRELATE : No correlating lesion [LIVER : Focus, pale : (Comment) 1, near hilus, right lateral (G)]

Histopathology - The following Tissues were Within Normal Limits:

BRAIN; CERVIX; EYE; GANGLION, DORSAL ROOT, CERVICAL; GANGLION, DORSAL ROOT, LUMBAR; GANGLION, DORSAL ROOT, THORACIC; GLAND, ADRENAL; GLAND, PARATHYROID; GLAND, PITUITARY; GLAND, THYROID; HEART; LUNG; LYMPH NODE, ILIAC; LYMPH NODE, MESENTERIC; MUSCLE, BICEPS FEMORIS; MUSCLE, GASTROCNEMIUS; NERVE, OPTIC; NERVE, SCIATIC; NERVE, TIBIAL; OVARY; SITE, INJECTION; SPINAL CORD, CERVICAL; SPINAL CORD, LUMBAR; SPINAL CORD, THORACIC; SPLEEN; THYMUS; UTERUS; NERVE ROOT, VENTRAL, CERVICAL; NERVE ROOT, VENTRAL, THORACIC; NERVE ROOT, VENTRAL, LUMBAR; NERVE ROOT, DORSAL, CERVICAL; NERVE ROOT, DORSAL, LUMBAR; NERVE ROOT, DORSAL, THORACIC

Histopathology - The following Tissues were Not Examined:

None

Appendix 18

Appendix 4

Individual Macroscopic and Microscopic Pathology:

5550008

Animal:	2505	Group:	2	Sex:	Female
Species:	Rat	Strain:	Sprague Dawley		
		Dose:	0.36x 10E12 vg		
		Removal Reason:	Terminal Euthanasia		
		Study Day (Week) of Death:	8 (2)		

Gross Pathology Animal Details:

No animal details found

Gross Pathology Observations [Correlation]:

THYMUS : Focus, dark : (Comment) >10. (TGL)

Any remaining study plan required tissues, which have been examined, have no visible lesions

Gross Pathology - The following Tissues were Not Examined:

None

Histopathology Animal Details:

No animal details found

Histopathology Observations [Correlation]:

BRAIN : Infiltration, mononuclear cell; minimal, meninges : (Comment) slide 3, cerebellum
GLAND, PARATHYROID : (Note) One Of A Pair Available For Evaluation.
KIDNEY : Chronic progressive nephropathy; minimal
LIVER : Vacuolation; hepatocellular, periportal, minimal
SITE, INJECTION : Infiltration, mononuclear cell; minimal, cauda equina
SITE, INJECTION : Degeneration; axonal, minimal, cauda equina
THYMUS : Hemorrhage; acute, mild [THYMUS : Focus, dark : (Comment) >10. (G)]

Histopathology - The following Tissues were Within Normal Limits:

CERVIX; EYE; GANGLION, DORSAL ROOT, CERVICAL; GANGLION, DORSAL ROOT, LUMBAR; GANGLION, DORSAL ROOT, THORACIC; GLAND, ADRENAL; GLAND, PARATHYROID; GLAND, PITUITARY; GLAND, THYROID; HEART; LUNG; LYMPH NODE, CERVICAL; LYMPH NODE, ILIAC; LYMPH NODE, MANDIBULAR; LYMPH NODE, MESENTERIC; MUSCLE, BICEPS FEMORIS; MUSCLE, GASTROCNEMIUS; NERVE, OPTIC; NERVE, SCIATIC; NERVE, TIBIAL; OVARY; SPINAL CORD, CERVICAL; SPINAL CORD, LUMBAR; SPINAL CORD, THORACIC; SPLEEN; UTERUS; NERVE ROOT, VENTRAL, CERVICAL; NERVE ROOT, VENTRAL, THORACIC; NERVE ROOT, VENTRAL, LUMBAR; NERVE ROOT, DORSAL, CERVICAL; NERVE ROOT, DORSAL, LUMBAR; NERVE ROOT, DORSAL, THORACIC

Histopathology - The following Tissues were Not Examined:

None

Individual Macroscopic and Microscopic Pathology:

5550008

Animal: 2506	Group: 2	Sex: Female
Species: Rat	Strain: Sprague Dawley<br>Dose: 0.36x 10E12 vg<br>Removal Reason: Terminal Euthanasia<br>Study Day (Week) of Death: 8 (2)	

Gross Pathology Animal Details:

No animal details found

Gross Pathology Observations [Correlation]:

No observations found

Any remaining study plan required tissues, which have been examined, have no visible lesions

Gross Pathology - The following Tissues were Not Examined:

None

Histopathology Animal Details:

No animal details found

Histopathology Observations [Correlation]:

GLAND, PARATHYROID : (Note) One Of A Pair Available For Evaluation.
LIVER : Vacuolation; hepatocellular, periportal, minimal
SITE, INJECTION : Infiltration, mononuclear cell; minimal, leptomeninges
SITE, INJECTION : Degeneration; axonal, minimal, cauda equina

Histopathology - The following Tissues were Within Normal Limits:

BRAIN; CERVIX; EYE; GANGLION, DORSAL ROOT, CERVICAL; GANGLION, DORSAL ROOT, LUMBAR; GANGLION, DORSAL ROOT, THORACIC; GLAND, ADRENAL; GLAND, PARATHYROID; GLAND, PITUITARY; GLAND, THYROID; HEART; KIDNEY; LUNG; LYMPH NODE, CERVICAL; LYMPH NODE, ILIAC; LYMPH NODE, MANDIBULAR; LYMPH NODE, MESENTERIC; MUSCLE, BICEPS FEMORIS; MUSCLE, GASTROCNEMIUS; NERVE, OPTIC; NERVE, SCIATIC; NERVE, TIBIAL; OVARY; SPINAL CORD, CERVICAL; SPINAL CORD, LUMBAR; SPINAL CORD, THORACIC; SPLEEN; THYMUS; UTERUS; NERVE ROOT, VENTRAL, CERVICAL; NERVE ROOT, VENTRAL, THORACIC; NERVE ROOT, VENTRAL, LUMBAR; NERVE ROOT, DORSAL, CERVICAL; NERVE ROOT, DORSAL, LUMBAR; NERVE ROOT, DORSAL, THORACIC

Histopathology - The following Tissues were Not Examined:

None

Appendix 18

Appendix 4

Individual Macroscopic and Microscopic Pathology:

5550008

Animal:	2507	Group:	2	Sex:	Female
Species:	Rat	Strain:	Sprague Dawley		
		Dose:	0.36x 10E12 vg		
		Removal Reason: Recovery Euthanasia			
		Study Day (Week) of Death: 29 (5)			

Gross Pathology Animal Details:

No animal details found

Gross Pathology Observations [Correlation]:

THYMUS : Focus, dark : (Comment) 4, left (TGL)

Any remaining study plan required tissues, which have been examined, have no visible lesions

Gross Pathology - The following Tissues were Not Examined:

None

Histopathology Animal Details:

No animal details found

Histopathology Observations [Correlation]:

GLAND, PITUITARY : (Note) Only Pars Distalis Available For Evaluation.
LIVER : Vacuolation; hepatocellular, periportal, minimal
LIVER : Infiltration, mixed cell; minimal
SITE, INJECTION : Infiltration, mononuclear cell; minimal, leptomeninges
SPINAL CORD, CERVICAL : Infiltration, mononuclear cell; minimal, leptomeninges
NO CORRELATE : No correlating lesion [THYMUS : Focus, dark : (Comment) 4, left (G)]

Histopathology - The following Tissues were Within Normal Limits:

BRAIN; CERVIX; EYE; GANGLION, DORSAL ROOT, CERVICAL; GANGLION, DORSAL ROOT, LUMBAR; GANGLION, DORSAL ROOT, THORACIC; GLAND, ADRENAL; GLAND, PARATHYROID; GLAND, PITUITARY; GLAND, THYROID; HEART; KIDNEY; LUNG; LYMPH NODE, CERVICAL; LYMPH NODE, ILIAC; LYMPH NODE, MANDIBULAR; LYMPH NODE, MESENTERIC; MUSCLE, BICEPS FEMORIS; MUSCLE, GASTROCNEMIUS; NERVE, OPTIC; NERVE, SCIATIC; NERVE, TIBIAL; OVARY; SPINAL CORD, LUMBAR; SPINAL CORD, THORACIC; SPLEEN; THYMUS; UTERUS; NERVE ROOT, VENTRAL, CERVICAL; NERVE ROOT, VENTRAL, THORACIC; NERVE ROOT, VENTRAL, LUMBAR; NERVE ROOT, DORSAL, CERVICAL; NERVE ROOT, DORSAL, LUMBAR; NERVE ROOT, DORSAL, THORACIC

Histopathology - The following Tissues were Not Examined:

None

Individual Macroscopic and Microscopic Pathology:

5550008

Animal: 2508	Group: 2		Sex: Female
Species: Rat	Strain: Sprague Dawley		
	Dose: 0.36x 10E12 vg		
	Removal Reason: Recovery Euthanasia		
	Study Day (Week) of Death: 29 (5)		

Gross Pathology Animal Details:

No animal details found

Gross Pathology Observations [Correlation]:

GENERAL OBSERVATIONS : (Comment) Optic nerve left submitted in cassette C
LIVER : Focus, pale : (Comment) 1, fissure, left medial (TGL)

Any remaining study plan required tissues, which have been examined, have no visible lesions

Gross Pathology - The following Tissues were Not Examined:

None

Histopathology Animal Details:

No animal details found

Histopathology Observations [Correlation]:

KIDNEY : Cast; hyaline, minimal
KIDNEY : Dilatation; tubular, mild : (Comment) with hyaline eosinophilic material
LIVER : Tension lipidosis; minimal [LIVER : Focus, pale : (Comment) 1, fissure, left medial (G)]
LIVER : Infiltration, mixed cell; minimal
SITE, INJECTION : Infiltration, mononuclear cell; minimal, leptomeninges

Histopathology - The following Tissues were Within Normal Limits:

BRAIN; CERVIX; EYE; GANGLION, DORSAL ROOT, CERVICAL; GANGLION, DORSAL ROOT, LUMBAR; GANGLION, DORSAL ROOT, THORACIC; GLAND, ADRENAL; GLAND, PARATHYROID; GLAND, PITUITARY; GLAND, THYROID; HEART; LUNG; LYMPH NODE, CERVICAL; LYMPH NODE, ILIAC; LYMPH NODE, MANDIBULAR; LYMPH NODE, MESENTERIC; MUSCLE, BICEPS FEMORIS; MUSCLE, GASTROCNEMIUS; NERVE, OPTIC; NERVE, SCIATIC; NERVE, TIBIAL; OVARY; SPINAL CORD, CERVICAL; SPINAL CORD, LUMBAR; SPINAL CORD, THORACIC; SPLEEN; THYMUS; UTERUS; NERVE ROOT, VENTRAL, CERVICAL; NERVE ROOT, VENTRAL, THORACIC; NERVE ROOT, VENTRAL, LUMBAR; NERVE ROOT, DORSAL, CERVICAL; NERVE ROOT, DORSAL, LUMBAR; NERVE ROOT, DORSAL, THORACIC

Histopathology - The following Tissues were Not Examined:

None

Appendix 18

Appendix 4

Individual Macroscopic and Microscopic Pathology:

5550008

Animal: 2509	Group: 2		Sex: Female
Species: Rat	Strain: Sprague Dawley		
	Dose: 0.36x 10E12 vg		
	Removal Reason: Recovery Euthanasia		
	Study Day (Week) of Death: 29 (5)		

Gross Pathology Animal Details:

No animal details found

Gross Pathology Observations [Correlation]:

No observations found

Any remaining study plan required tissues, which have been examined, have no visible lesions

Gross Pathology - The following Tissues were Not Examined:

None

Histopathology Animal Details:

No animal details found

Histopathology Observations [Correlation]:

GLAND, PARATHYROID : (Note) One Of A Pair Available For Evaluation.
LIVER : Vacuolation; hepatocellular, periportal, minimal
LIVER : Infiltration, mixed cell; minimal

Histopathology - The following Tissues were Within Normal Limits:

BRAIN; CERVIX; EYE; GANGLION, DORSAL ROOT, CERVICAL; GANGLION, DORSAL ROOT, LUMBAR; GANGLION, DORSAL ROOT, THORACIC; GLAND, ADRENAL; GLAND, PARATHYROID; GLAND, PITUITARY; GLAND, THYROID; HEART; KIDNEY; LUNG; LYMPH NODE, CERVICAL; LYMPH NODE, ILIAC; LYMPH NODE, MANDIBULAR; LYMPH NODE, MESENTERIC; MUSCLE, BICEPS FEMORIS; MUSCLE, GASTROCNEMIUS; NERVE, OPTIC; NERVE, SCIATIC; NERVE, TIBIAL; OVARY; SITE, INJECTION; SPINAL CORD, CERVICAL; SPINAL CORD, LUMBAR; SPINAL CORD, THORACIC; SPLEEN; THYMUS; UTERUS; NERVE ROOT, VENTRAL, CERVICAL; NERVE ROOT, VENTRAL, THORACIC; NERVE ROOT, VENTRAL, LUMBAR; NERVE ROOT, DORSAL, CERVICAL; NERVE ROOT, DORSAL, LUMBAR; NERVE ROOT, DORSAL, THORACIC

Histopathology - The following Tissues were Not Examined:

None

Individual Macroscopic and Microscopic Pathology:

5550008

Animal:	2510	Group:	2	Sex:	Female
Species:	Rat	Strain:	Sprague Dawley		
		Dose:	0.36x 10E12 vg		
		Removal Reason: Terminal Euthanasia			
		Study Day (Week) of Death: 8 (2)			

Gross Pathology Animal Details:

No animal details found

Gross Pathology Observations [Correlation]:

GENERAL OBSERVATIONS : (Comment) Site surgical lesion submitted on plastic in bag. Right lymph node deep cervical not submitted for histopathological evaluation (frozen).
LYMPH NODE, CERVICAL : Discoloration, mottled : (Comment) bilateral. (TGL)
SITE, SURGICAL : Focus, dark : (Comment) >10, subcutis. (TGL)

Any remaining study plan required tissues, which have been examined, have no visible lesions

Gross Pathology - The following Tissues were Not Examined:

None

Histopathology Animal Details:

No animal details found

Histopathology Observations [Correlation]:

KIDNEY : Infiltration, mononuclear cell; minimal
KIDNEY : Chronic progressive nephropathy; minimal
LYMPH NODE, CERVICAL : Hemorrhage; acute, mild [LYMPH NODE, CERVICAL : Discoloration, mottled : (Comment) bilateral. (G)]
LYMPH NODE, MANDIBULAR : Hemorrhage; acute, mild
SITE, SURGICAL : Hemorrhage; acute, regionally extensive, mild [SITE, SURGICAL : Focus, dark : (Comment) >10, subcutis. (G)]

Histopathology - The following Tissues were Within Normal Limits:

BRAIN; CERVIX; EYE; GANGLION, DORSAL ROOT, CERVICAL; GANGLION, DORSAL ROOT, LUMBAR; GANGLION, DORSAL ROOT, THORACIC; GLAND, ADRENAL; GLAND, PARATHYROID; GLAND, PITUITARY; GLAND, THYROID; HEART; LIVER; LUNG; LYMPH NODE, MESENTERIC; MUSCLE, BICEPS FEMORIS; MUSCLE, GASTROCNEMIUS; NERVE, OPTIC; NERVE, SCIATIC; NERVE, TIBIAL; OVARY; SITE, INJECTION; SPINAL CORD, CERVICAL; SPINAL CORD, LUMBAR; SPINAL CORD, THORACIC; SPLEEN; THYMUS; UTERUS; NERVE ROOT, VENTRAL, CERVICAL; NERVE ROOT, VENTRAL, THORACIC; NERVE ROOT, VENTRAL, LUMBAR; NERVE ROOT, DORSAL, CERVICAL; NERVE ROOT, DORSAL, LUMBAR; NERVE ROOT, DORSAL, THORACIC

Histopathology - The following Tissues were Not Examined:

LYMPH NODE, ILIAC - Not Present In Section.

Appendix 18

Appendix 4

Individual Macroscopic and Microscopic Pathology:

5550008

Animal:	2511	Group:	2	Sex:	Female
Species:	Rat	Strain:	Sprague Dawley		
		Dose:	0.36x 10E12 vg		
		Removal Reason:	Terminal Euthanasia		
		Study Day (Week) of Death:	8 (2)		

Gross Pathology Animal Details:

No animal details found

Gross Pathology Observations [Correlation]:

GENERAL OBSERVATIONS : (Comment) lymph node lesion submitted cass E
LYMPH NODE : Discoloration, mottled : (Comment) renal, left (TGL)

Any remaining study plan required tissues, which have been examined, have no visible lesions

Gross Pathology - The following Tissues were Not Examined:

None

Histopathology Animal Details:

No animal details found

Histopathology Observations [Correlation]:

LYMPH NODE : Hemorrhage; acute, moderate : (Comment) with hemosiderophages [LYMPH NODE : Discoloration, mottled : (Comment) renal, left (G)]
SITE, INJECTION : Infiltration, mononuclear cell; minimal : (Comment) Fibrous ligament dorsally (ligamentum flavum)

Histopathology - The following Tissues were Within Normal Limits:

BRAIN; CERVIX; EYE; GANGLION, DORSAL ROOT, CERVICAL; GANGLION, DORSAL ROOT, LUMBAR; GANGLION, DORSAL ROOT, THORACIC; GLAND, ADRENAL; GLAND, PARATHYROID; GLAND, PITUITARY; GLAND, THYROID; HEART; KIDNEY; LIVER; LUNG; LYMPH NODE, CERVICAL; LYMPH NODE, ILIAC; LYMPH NODE, MANDIBULAR; LYMPH NODE, MESENTERIC; MUSCLE, BICEPS FEMORIS; MUSCLE, GASTROCNEMIUS; NERVE, OPTIC; NERVE, SCIATIC; NERVE, TIBIAL; OVARY; SPINAL CORD, CERVICAL; SPINAL CORD, LUMBAR; SPINAL CORD, THORACIC; SPLEEN; THYMUS; UTERUS; NERVE ROOT, VENTRAL, CERVICAL; NERVE ROOT, VENTRAL, THORACIC; NERVE ROOT, VENTRAL, LUMBAR; NERVE ROOT, DORSAL, CERVICAL; NERVE ROOT, DORSAL, LUMBAR; NERVE ROOT, DORSAL, THORACIC

Histopathology - The following Tissues were Not Examined:

None

Appendix 18

Appendix 4

Individual Macroscopic and Microscopic Pathology:

5550008

Animal: 2512	Group: 2	Sex: Female
Species: Rat	Strain: Sprague Dawley<br>Dose: 0.36x 10E12 vg<br>Removal Reason: Terminal Euthanasia<br>Study Day (Week) of Death: 8 (2)	

Gross Pathology Animal Details:

No animal details found

Gross Pathology Observations [Correlation]:

No observations found

Any remaining study plan required tissues, which have been examined, have no visible lesions

Gross Pathology - The following Tissues were Not Examined:

None

Histopathology Animal Details:

No animal details found

Histopathology Observations [Correlation]:

GANGLION, DORSAL ROOT, LUMBAR : Infiltration, mononuclear cell; minimal
LIVER : Infiltration, mononuclear cell; periportal, minimal
SITE, INJECTION : Infiltration, mononuclear cell; minimal, leptomeninges

Histopathology - The following Tissues were Within Normal Limits:

BRAIN; CERVIX; EYE; GANGLION, DORSAL ROOT, CERVICAL; GANGLION, DORSAL ROOT, THORACIC; GLAND, ADRENAL; GLAND, PARATHYROID; GLAND, PITUITARY; GLAND, THYROID; HEART; KIDNEY; LUNG; LYMPH NODE, CERVICAL; LYMPH NODE, ILIAC; LYMPH NODE, MANDIBULAR; LYMPH NODE, MESENTERIC; MUSCLE, BICEPS FEMORIS; MUSCLE, GASTROCNEMIUS; NERVE, SCIATIC; NERVE, TIBIAL; OVARY; SPINAL CORD, CERVICAL; SPINAL CORD, LUMBAR; SPINAL CORD, THORACIC; SPLEEN; THYMUS; UTERUS; NERVE ROOT, VENTRAL, CERVICAL; NERVE ROOT, VENTRAL, THORACIC; NERVE ROOT, VENTRAL, LUMBAR; NERVE ROOT, DORSAL, CERVICAL; NERVE ROOT, DORSAL, LUMBAR; NERVE ROOT, DORSAL, THORACIC

Histopathology - The following Tissues were Not Examined:

NERVE, OPTIC - Not Present In Section.

Individual Macroscopic and Microscopic Pathology:

5550008

Animal:	2613	Group:	2	Sex:	Female
Species:	Rat	Strain:	Sprague Dawley		
		Dose:	0.36x 10E12 vg		
		Removal Reason:	Recovery Euthanasia		
		Study Day (Week) of Death:	91 (13)		

Gross Pathology Animal Details:

No animal details found

Gross Pathology Observations [Correlation]:

LIVER : Focus, pale : (Comment) 1, fissure, right medial. (TGL)
LUNG : Focus, pale : (Comment) 4, right caudal. (TGL)

Any remaining study plan required tissues, which have been examined, have no visible lesions

Gross Pathology - The following Tissues were Not Examined:

None

Histopathology Animal Details:

No animal details found

Histopathology Observations [Correlation]:

GLAND, PITUITARY : (Note) Only Pars Distalis Available For Evaluation.
HEART : Infiltration, mononuclear cell/degeneration, myofiber; minimal
KIDNEY : Infiltration, mononuclear cell; minimal
KIDNEY : Chronic progressive nephropathy; minimal
LIVER : Tension lipidosis; mild [LIVER : Focus, pale : (Comment) 1, fissure, right medial. (G)]
LIVER : Infiltration, mixed cell; minimal
LYMPH NODE, CERVICAL : Hemorrhage; acute, mild
SITE, INJECTION : Infiltration, mononuclear cell; minimal, leptomeninges
NO CORRELATE : No correlating lesion [LUNG : Focus, pale : (Comment) 4, right caudal. (G)]

Histopathology - The following Tissues were Within Normal Limits:

BRAIN; CERVIX; EYE; GANGLION, DORSAL ROOT, CERVICAL; GANGLION, DORSAL ROOT, LUMBAR; GANGLION, DORSAL ROOT, THORACIC; GLAND, ADRENAL; GLAND, PARATHYROID; GLAND, PITUITARY; GLAND, THYROID; LUNG; LYMPH NODE, ILIAC; LYMPH NODE, MANDIBULAR; LYMPH NODE, MESENTERIC; MUSCLE, BICEPS FEMORIS; MUSCLE, GASTROCNEMIUS; NERVE, OPTIC; NERVE, SCIATIC; NERVE, TIBIAL; OVARY; SPINAL CORD, CERVICAL; SPINAL CORD, LUMBAR; SPINAL CORD, THORACIC; SPLEEN; THYMUS; UTERUS; NERVE ROOT, VENTRAL, CERVICAL; NERVE ROOT, VENTRAL, THORACIC; NERVE ROOT, VENTRAL, LUMBAR; NERVE ROOT, DORSAL, CERVICAL; NERVE ROOT, DORSAL, LUMBAR; NERVE ROOT, DORSAL, THORACIC

Histopathology - The following Tissues were Not Examined:

None

Appendix 18

Appendix 4

Individual Macroscopic and Microscopic Pathology:

5550008

Animal:	2614	Group:	2	Sex:	Female
Species:	Rat	Strain:	Sprague Dawley		
		Dose:	0.36x 10E12 vg		
		Removal Reason:	Recovery Euthanasia		
		Study Day (Week) of Death:	91 (13)		

Gross Pathology Animal Details:

No animal details found

Gross Pathology Observations [Correlation]:

No observations found

Any remaining study plan required tissues, which have been examined, have no visible lesions

Gross Pathology - The following Tissues were Not Examined:

None

Histopathology Animal Details:

No animal details found

Histopathology Observations [Correlation]:

GLAND, PARATHYROID : (Note) One Of A Pair Available For Evaluation.
GLAND, PITUITARY : (Note) Only Pars Distalis Available For Evaluation.
LIVER : Vacuolation; hepatocellular, periportal, minimal
LIVER : Infiltration, mixed cell; minimal
SITE, INJECTION : Infiltration, mononuclear cell; minimal, leptomeninges

Histopathology - The following Tissues were Within Normal Limits:

BRAIN; CERVIX; EYE; GANGLION, DORSAL ROOT, CERVICAL; GANGLION, DORSAL ROOT, LUMBAR; GANGLION, DORSAL ROOT, THORACIC; GLAND, ADRENAL; GLAND, PARATHYROID; GLAND, PITUITARY; GLAND, THYROID; HEART; KIDNEY; LUNG; LYMPH NODE, CERVICAL; LYMPH NODE, ILIAC; LYMPH NODE, MANDIBULAR; LYMPH NODE, MESENTERIC; MUSCLE, BICEPS FEMORIS; MUSCLE, GASTROCNEMIUS; NERVE, OPTIC; NERVE, SCIATIC; NERVE, TIBIAL; OVARY; SPINAL CORD, CERVICAL; SPINAL CORD, LUMBAR; SPINAL CORD, THORACIC; SPLEEN; THYMUS; UTERUS; NERVE ROOT, VENTRAL, CERVICAL; NERVE ROOT, VENTRAL, THORACIC; NERVE ROOT, VENTRAL, LUMBAR; NERVE ROOT, DORSAL, CERVICAL; NERVE ROOT, DORSAL, LUMBAR; NERVE ROOT, DORSAL, THORACIC

Histopathology - The following Tissues were Not Examined:

None

Appendix 18

Appendix 4

Individual Macroscopic and Microscopic Pathology:

5550008

Animal: 2615	Group: 2	Sex: Female
Species: Rat	Strain: Sprague Dawley<br>Dose: 0.36x 10E12 vg<br>Removal Reason: Recovery Euthanasia<br>Study Day (Week) of Death: 91 (13)	

Gross Pathology Animal Details:

No animal details found

Gross Pathology Observations [Correlation]:

No observations found

Any remaining study plan required tissues, which have been examined, have no visible lesions

Gross Pathology - The following Tissues were Not Examined:

None

Histopathology Animal Details:

No animal details found

Histopathology Observations [Correlation]:

GLAND, ADRENAL : (Note) Only Cortex And One Medulla Available For Evaluation.
GLAND, PARATHYROID : (Note) One Of A Pair Available For Evaluation.
LIVER : Infiltration, mixed cell; minimal
NERVE, TIBIAL : Degeneration; axonal, minimal
SITE, INJECTION : Infiltration, mononuclear cell; minimal, leptomeninges
NERVE ROOT, DORSAL, LUMBAR : Degeneration; axonal, minimal

Histopathology - The following Tissues were Within Normal Limits:

BRAIN; CERVIX; EYE; GANGLION, DORSAL ROOT, CERVICAL; GANGLION, DORSAL ROOT, LUMBAR; GANGLION, DORSAL ROOT, THORACIC; GLAND, ADRENAL; GLAND, PARATHYROID; GLAND, PITUITARY; GLAND, THYROID; HEART; KIDNEY; LUNG; LYMPH NODE, CERVICAL; LYMPH NODE, ILIAC; LYMPH NODE, MANDIBULAR; LYMPH NODE, MESENTERIC; MUSCLE, BICEPS FEMORIS; MUSCLE, GASTROCNEMIUS; NERVE, OPTIC; NERVE, SCIATIC; OVARY; SPINAL CORD, CERVICAL; SPINAL CORD, LUMBAR; SPINAL CORD, THORACIC; SPLEEN; THYMUS; UTERUS; NERVE ROOT, VENTRAL, CERVICAL; NERVE ROOT, VENTRAL, THORACIC; NERVE ROOT, VENTRAL, LUMBAR; NERVE ROOT, DORSAL, CERVICAL; NERVE ROOT, DORSAL, THORACIC

Histopathology - The following Tissues were Not Examined:

None

Individual Macroscopic and Microscopic Pathology:

5550008

Animal: 3001	Group: 3	Sex: Male
Species: Rat	Strain: Sprague Dawley<br>Dose: 1.1x 10E12 vg<br>Removal Reason: Terminal Euthanasia<br>Study Day (Week) of Death: 8 (2)	

Gross Pathology Animal Details:

No animal details found

Gross Pathology Observations [Correlation]:

No observations found

Any remaining study plan required tissues, which have been examined, have no visible lesions

Gross Pathology - The following Tissues were Not Examined:

None

Histopathology Animal Details:

No animal details found

Histopathology Observations [Correlation]:

EPIDIDYMIS : Peripuberty
GANGLION, DORSAL ROOT, LUMBAR : Infiltration, mononuclear cell; minimal
GLAND, PARATHYROID : (Note) One Of A Pair Available For Evaluation.
GLAND, PITUITARY : (Note) Only Pars Distalis Available For Evaluation.
LIVER : Infiltration, mononuclear cell; periportal, minimal
NERVE, TIBIAL : Degeneration; axonal, minimal
SITE, INJECTION : Infiltration, mononuclear cell; minimal, leptomeninges
TESTIS : Peripuberty
NERVE ROOT, DORSAL, LUMBAR : Infiltration, mononuclear cell; minimal, leptomeninges

Histopathology - The following Tissues were Within Normal Limits:

BRAIN; EYE; GANGLION, DORSAL ROOT, CERVICAL; GANGLION, DORSAL ROOT, THORACIC; GLAND, ADRENAL; GLAND, PARATHYROID; GLAND, PITUITARY; GLAND, PROSTATE; GLAND, THYROID; HEART; KIDNEY; LUNG; LYMPH NODE, CERVICAL; LYMPH NODE, ILIAC; LYMPH NODE, MANDIBULAR; LYMPH NODE, MESENTERIC; MUSCLE, BICEPS FEMORIS; MUSCLE, GASTROCNEMIUS; NERVE, SCIATIC; SPINAL CORD, CERVICAL; SPINAL CORD, LUMBAR; SPINAL CORD, THORACIC; SPLEEN; THYMUS; NERVE ROOT, VENTRAL, CERVICAL; NERVE ROOT, VENTRAL, THORACIC; NERVE ROOT, VENTRAL, LUMBAR; NERVE ROOT, DORSAL, CERVICAL; NERVE ROOT, DORSAL, THORACIC

Histopathology - The following Tissues were Not Examined:

NERVE, OPTIC - Not Present In Section.

Individual Macroscopic and Microscopic Pathology:

5550008

Animal:	3002	Group:	3	Sex:	Male
Species:	Rat	Strain:	Sprague Dawley		
		Dose:	1.1x 10E12 vg		
		Removal Reason: Terminal Euthanasia			
		Study Day (Week) of Death: 8 (2)			

Gross Pathology Animal Details:

No animal details found

Gross Pathology Observations [Correlation]:

No observations found

Any remaining study plan required tissues, which have been examined, have no visible lesions

Gross Pathology - The following Tissues were Not Examined:

None

Histopathology Animal Details:

No animal details found

Histopathology Observations [Correlation]:

EPIDIDYMIS : Peripuberty
GLAND, PITUITARY : (Note) Only Pars Distalis Available For Evaluation.
KIDNEY : Chronic progressive nephropathy; minimal
LIVER : Infiltration, mixed cell; minimal
NERVE, SCIATIC : Degeneration; axonal, minimal
TESTIS : Peripuberty

Histopathology - The following Tissues were Within Normal Limits:

BRAIN; EYE; GANGLION, DORSAL ROOT, CERVICAL; GANGLION, DORSAL ROOT, LUMBAR; GANGLION, DORSAL ROOT, THORACIC; GLAND, ADRENAL; GLAND, PARATHYROID; GLAND, PITUITARY; GLAND, PROSTATE; GLAND, THYROID; HEART; LUNG; LYMPH NODE, CERVICAL; LYMPH NODE, ILIAC; LYMPH NODE, MANDIBULAR; LYMPH NODE, MESENTERIC; MUSCLE, BICEPS FEMORIS; MUSCLE, GASTROCNEMIUS; NERVE, OPTIC; NERVE, TIBIAL; SITE, INJECTION; SPINAL CORD, CERVICAL; SPINAL CORD, LUMBAR; SPINAL CORD, THORACIC; SPLEEN; THYMUS; NERVE ROOT, VENTRAL, CERVICAL; NERVE ROOT, VENTRAL, THORACIC; NERVE ROOT, VENTRAL, LUMBAR; NERVE ROOT, DORSAL, CERVICAL; NERVE ROOT, DORSAL, LUMBAR; NERVE ROOT, DORSAL, THORACIC

Histopathology - The following Tissues were Not Examined:

None

Individual Macroscopic and Microscopic Pathology:

5550008

Animal:	3003	Group:	3	Sex:	Male
Species:	Rat	Strain:	Sprague Dawley		
		Dose:	1.1x 10E12 vg		
		Removal Reason: Terminal Euthanasia			
		Study Day (Week) of Death: 8 (2)			

Gross Pathology Animal Details:

No animal details found

Gross Pathology Observations [Correlation]:

GENERAL OBSERVATIONS : (Comment) skin lesion submitted on plastic.
LIVER : Focus, pale : (Comment) 1, fissure, right medial (TGL)
LYMPH NODE, MANDIBULAR : Focus, dark : (Comment) >10, left (TGL)
SUBCUTIS : Focus, dark : (Comment) 1, extending into muscle, dorsal thoracic (TGL)

Any remaining study plan required tissues, which have been examined, have no visible lesions

Gross Pathology - The following Tissues were Not Examined:

None

Histopathology Animal Details:

No animal details found

Histopathology Observations [Correlation]:

LIVER : Tension lipidosis; minimal [LIVER : Focus, pale : (Comment) 1, fissure, right medial (G)]
LYMPH NODE, MANDIBULAR : Hemorrhage; acute, mild [LYMPH NODE, MANDIBULAR : Focus, dark : (Comment) >10, left (G)]
NERVE, TIBIAL : Degeneration; axonal, minimal
SITE, INJECTION : Infiltration, mononuclear cell; minimal, leptomeninges
SITE, INJECTION : Degeneration; axonal, minimal, cauda equina
SUBCUTIS : Hemorrhage; acute, mild [SUBCUTIS : Focus, dark : (Comment) 1, extending into muscle, dorsal thoracic (G)]
SUBCUTIS : Fibroplasia; regionally extensive, mild : (Comment) subcutis, with seroma formation
SUBCUTIS : Infiltration, mononuclear cell; mild : (Comment) subcutis
SUBCUTIS : Fibrosis; focal, mild : (Comment) dermis, with epidermal hyperplasia overlying
NO CORRELATE : No correlating lesion

Histopathology - The following Tissues were Within Normal Limits:

BRAIN; EPIDIDYMIS; EYE; GANGLION, DORSAL ROOT, CERVICAL; GANGLION, DORSAL ROOT, LUMBAR; GANGLION, DORSAL ROOT, THORACIC; GLAND, ADRENAL; GLAND, PARATHYROID; GLAND, PITUITARY; GLAND, PROSTATE; GLAND, THYROID; HEART; KIDNEY; LUNG; LYMPH NODE, CERVICAL; LYMPH NODE, ILIAC; LYMPH NODE, MESENTERIC; MUSCLE, BICEPS FEMORIS; MUSCLE, GASTROCNEMIUS; NERVE, OPTIC; NERVE, SCIATIC; SPINAL CORD, CERVICAL; SPINAL CORD, LUMBAR; SPINAL CORD, THORACIC; SPLEEN; TESTIS; THYMUS; NERVE ROOT, VENTRAL, CERVICAL; NERVE ROOT, VENTRAL, THORACIC; NERVE ROOT, VENTRAL, LUMBAR; NERVE ROOT, DORSAL, CERVICAL; NERVE ROOT, DORSAL, LUMBAR; NERVE ROOT, DORSAL, THORACIC

Histopathology - The following Tissues were Not Examined:

None

Individual Macroscopic and Microscopic Pathology:

5550008

Animal:	3004	Group:	3	Sex:	Male
Species:	Rat	Strain:	Sprague Dawley		
		Dose:	1.1x 10E12 vg		
		Removal Reason: Recovery Euthanasia			
		Study Day (Week) of Death: 29 (5)			

Gross Pathology Animal Details:

No animal details found

Gross Pathology Observations [Correlation]:

GENERAL OBSERVATIONS : (Comment) liver lesion submitted cass B; brain left submitted in two pieces
LIVER : Focus, pale : (Comment) 1, fissure, right medial, near hilus, right lateral (TGL)

Any remaining study plan required tissues, which have been examined, have no visible lesions

Gross Pathology - The following Tissues were Not Examined:

None

Histopathology Animal Details:

No animal details found

Histopathology Observations [Correlation]:

GLAND, PARATHYROID : (Note) One Of A Pair Available For Evaluation.
KIDNEY : Cast; hyaline, minimal
KIDNEY : Infiltration, mononuclear cell; minimal
KIDNEY : Chronic progressive nephropathy; minimal
LIVER : Tension lipidosis; mild [LIVER : Focus, pale : (Comment) 1, fissure, right medial, near hilus, right lateral (G)]
LIVER : Infiltration, mixed cell; minimal
LIVER : Infiltration, mononuclear cell; periportal, minimal
NERVE, SCIATIC : Degeneration; axonal, minimal
NERVE, TIBIAL : Degeneration; axonal, minimal

Histopathology - The following Tissues were Within Normal Limits:

BRAIN; EPIDIDYMIS; EYE; GANGLION, DORSAL ROOT, CERVICAL; GANGLION, DORSAL ROOT, LUMBAR; GANGLION, DORSAL ROOT, THORACIC; GLAND, ADRENAL; GLAND, PARATHYROID; GLAND, PITUITARY; GLAND, PROSTATE; GLAND, THYROID; HEART; LUNG; LYMPH NODE, CERVICAL; LYMPH NODE, ILIAC; LYMPH NODE, MANDIBULAR; LYMPH NODE, MESENTERIC; MUSCLE, BICEPS FEMORIS; MUSCLE, GASTROCNEMIUS; NERVE, OPTIC; SITE, INJECTION; SPINAL CORD, CERVICAL; SPINAL CORD, LUMBAR; SPINAL CORD, THORACIC; SPLEEN; TESTIS; THYMUS; NERVE ROOT, VENTRAL, CERVICAL; NERVE ROOT, VENTRAL, THORACIC; NERVE ROOT, VENTRAL, LUMBAR; NERVE ROOT, DORSAL, CERVICAL; NERVE ROOT, DORSAL, LUMBAR; NERVE ROOT, DORSAL, THORACIC

Histopathology - The following Tissues were Not Examined:

None

Individual Macroscopic and Microscopic Pathology:

5550008

Animal:	3005	Group:	3	Sex:	Male
Species:	Rat	Strain:	Sprague Dawley		
		Dose:	1.1x 10E12 vg		
		Removal Reason: Recovery Euthanasia			
		Study Day (Week) of Death: 29 (5)			

Gross Pathology Animal Details:

No animal details found

Gross Pathology Observations [Correlation]:

LYMPH NODE, MANDIBULAR : Focus, dark : (Comment) 1, left (TGL)
LYMPH NODE, MANDIBULAR : Enlargement : (Comment) left (TGL)

Any remaining study plan required tissues, which have been examined, have no visible lesions

Gross Pathology - The following Tissues were Not Examined:

None

Histopathology Animal Details:

No animal details found

Histopathology Observations [Correlation]:

EPIDIDYMIS : Infiltration, mononuclear cell; minimal
GANGLION, DORSAL ROOT, LUMBAR : Infiltration, mononuclear cell; minimal
GANGLION, DORSAL ROOT, LUMBAR : Degeneration; neuronal, minimal
GLAND, PARATHYROID : (Note) One Of A Pair Available For Evaluation.
GLAND, PITUITARY : (Note) Only Pars Distalis Available For Evaluation.
KIDNEY : Infiltration, mononuclear cell; minimal
KIDNEY : Chronic progressive nephropathy; minimal
LIVER : Vacuolation; hepatocellular, periportal, minimal
LYMPH NODE, MANDIBULAR : Plasmacytosis; mild [LYMPH NODE, MANDIBULAR : Focus, dark : (Comment) 1, left (G)]
LYMPH NODE, MANDIBULAR : Cellularity, increased; lymphoid, mild [LYMPH NODE, MANDIBULAR : Enlargement : (Comment) left (G)]
NERVE, SCIATIC : Degeneration; axonal, minimal
SITE, INJECTION : Degeneration; axonal, minimal, cauda equina
NERVE ROOT, DORSAL, LUMBAR : Degeneration; axonal, minimal

Histopathology - The following Tissues were Within Normal Limits:

BRAIN; EYE; GANGLION, DORSAL ROOT, CERVICAL; GANGLION, DORSAL ROOT, THORACIC; GLAND, ADRENAL; GLAND, PARATHYROID; GLAND, PITUITARY; GLAND, PROSTATE; GLAND, THYROID; HEART; LUNG; LYMPH NODE, CERVICAL; LYMPH NODE, ILIAC; LYMPH NODE, MESENTERIC; MUSCLE, BICEPS FEMORIS; MUSCLE, GASTROCNEMIUS; NERVE, OPTIC; NERVE, TIBIAL; SPINAL CORD, CERVICAL; SPINAL CORD, LUMBAR; SPINAL CORD, THORACIC; SPLEEN; TESTIS; THYMUS; NERVE ROOT, VENTRAL, CERVICAL; NERVE ROOT, VENTRAL, THORACIC; NERVE ROOT, VENTRAL, LUMBAR; NERVE ROOT, DORSAL, CERVICAL; NERVE ROOT, DORSAL, THORACIC

Histopathology - The following Tissues were Not Examined:

None

Individual Macroscopic and Microscopic Pathology:

5550008

Animal:	3006	Group:	3	Sex:	Male
Species:	Rat	Strain:	Sprague Dawley		
		Dose:	1.1x 10E12 vg		
		Removal Reason: Recovery Euthanasia			
		Study Day (Week) of Death: 91 (13)			

Gross Pathology Animal Details:

No animal details found

Gross Pathology Observations [Correlation]:

LIVER : Focus, pale : (Comment) 1, fissure, right medial. (TGL)
LYMPH NODE, CERVICAL : Discoloration, dark : (Comment) left (TGL)

Any remaining study plan required tissues, which have been examined, have no visible lesions

Gross Pathology - The following Tissues were Not Examined:

None

Histopathology Animal Details:

No animal details found

Histopathology Observations [Correlation]:

EPIDIDYMIS : Infiltration, mononuclear cell; minimal
GANGLION, DORSAL ROOT, LUMBAR : Infiltration, mononuclear cell; minimal
GLAND, PROSTATE : Infiltration, mononuclear cell; moderate
GLAND, PROSTATE : Inflammation, mixed cell; focal, mild : (Comment) glandular/acinar, with acinar atrophy and cellular debris in lumen
LIVER : Tension lipidosis; mild [LIVER : Focus, pale : (Comment) 1, fissure, right medial. (G)]
LIVER : Infiltration, mononuclear cell; minimal
LYMPH NODE, CERVICAL : Hemorrhage; acute, minimal [LYMPH NODE, CERVICAL : Discoloration, dark : (Comment) left (G)]
NERVE, SCIATIC : Degeneration; axonal, minimal
NERVE ROOT, DORSAL, LUMBAR : Degeneration; axonal, minimal

Histopathology - The following Tissues were Within Normal Limits:

BRAIN; EYE; GANGLION, DORSAL ROOT, CERVICAL; GANGLION, DORSAL ROOT, THORACIC; GLAND, ADRENAL; GLAND, PARATHYROID; GLAND, PITUITARY; GLAND, THYROID; HEART; KIDNEY; LUNG; LYMPH NODE, MANDIBULAR; LYMPH NODE, MESENTERIC; MUSCLE, BICEPS FEMORIS; MUSCLE, GASTROCNEMIUS; NERVE, OPTIC; NERVE, TIBIAL; SITE, INJECTION; SPINAL CORD, CERVICAL; SPINAL CORD, LUMBAR; SPINAL CORD, THORACIC; SPLEEN; TESTIS; THYMUS; NERVE ROOT, VENTRAL, CERVICAL; NERVE ROOT, VENTRAL, THORACIC; NERVE ROOT, VENTRAL, LUMBAR; NERVE ROOT, DORSAL, CERVICAL; NERVE ROOT, DORSAL, THORACIC

Histopathology - The following Tissues were Not Examined:

LYMPH NODE, ILIAC - Not Present In Section.

Individual Macroscopic and Microscopic Pathology:

5550008

Animal: 3007	Group: 3	Sex: Male
Species: Rat	Strain: Sprague Dawley	
	Dose: 1.1x 10E12 vg	
	Removal Reason: Recovery Euthanasia	
	Study Day (Week) of Death: 91 (13)	

Gross Pathology Animal Details:

No animal details found

Gross Pathology Observations [Correlation]:

KIDNEY : Focus, dark : (Comment) 1, left (TGL)
LYMPH NODE, ILIAC : Focus, dark : (Comment) 2, left (TGL)

Any remaining study plan required tissues, which have been examined, have no visible lesions

Gross Pathology - The following Tissues were Not Examined:

None

Histopathology Animal Details:

No animal details found

Histopathology Observations [Correlation]:

GANGLION, DORSAL ROOT, LUMBAR : Degeneration; neuronal, minimal
GLAND, PROSTATE : Infiltration, mononuclear cell; minimal
KIDNEY : Dilatation; tubular, mild : (Comment) filled with hyaline material and cellular debris [KIDNEY : Focus, dark : (Comment) 1, left (G)]
KIDNEY : Chronic progressive nephropathy; minimal
LIVER : Vacuolation; hepatocellular, periportal, minimal
LIVER : Infiltration, mixed cell; minimal
NERVE, TIBIAL : Degeneration; axonal, minimal
SITE, INJECTION : Infiltration, mononuclear cell; minimal, leptomeninges
NO CORRELATE : No correlating lesion [LYMPH NODE, ILIAC : Focus, dark : (Comment) 2, left (G)]

Histopathology - The following Tissues were Within Normal Limits:

BRAIN; EPIDIDYMIS; EYE; GANGLION, DORSAL ROOT, CERVICAL; GANGLION, DORSAL ROOT, THORACIC; GLAND, ADRENAL; GLAND, PARATHYROID; GLAND, PITUITARY; GLAND, THYROID; HEART; LUNG; LYMPH NODE, CERVICAL; LYMPH NODE, ILIAC; LYMPH NODE, MANDIBULAR; LYMPH NODE, MESENTERIC; MUSCLE, BICEPS FEMORIS; MUSCLE, GASTROCNEMIUS; NERVE, OPTIC; NERVE, SCIATIC; SPINAL CORD, CERVICAL; SPINAL CORD, LUMBAR; SPINAL CORD, THORACIC; SPLEEN; TESTIS; THYMUS; NERVE ROOT, VENTRAL, CERVICAL; NERVE ROOT, VENTRAL, THORACIC; NERVE ROOT, VENTRAL, LUMBAR; NERVE ROOT, DORSAL, CERVICAL; NERVE ROOT, DORSAL, LUMBAR; NERVE ROOT, DORSAL, THORACIC

Histopathology - The following Tissues were Not Examined:

None

Appendix 18

Appendix 4

Individual Macroscopic and Microscopic Pathology:

5550008

Animal:	3008	Group:	3	Sex:	Male
Species:	Rat	Strain:	Sprague Dawley		
		Dose:	1.1x 10E12 vg		
		Removal Reason: Recovery Euthanasia			
		Study Day (Week) of Death: 91 (13)			

Gross Pathology Animal Details:

No animal details found

Gross Pathology Observations [Correlation]:

GENERAL OBSERVATIONS : (Comment) Liver lesion submitted cass A.
LIVER : Focus, pale : (Comment) 1, right lateral near hilus (TGL)

Any remaining study plan required tissues, which have been examined, have no visible lesions

Gross Pathology - The following Tissues were Not Examined:

None

Histopathology Animal Details:

No animal details found

Histopathology Observations [Correlation]:

GANGLION, DORSAL ROOT, LUMBAR : Infiltration, mononuclear cell; minimal
GLAND, ADRENAL : (Note) Only Cortex And One Medulla Available For Evaluation.
HEART : Infiltration, mononuclear cell/degeneration, myofiber; minimal
LIVER : Necrosis; focal, minimal
LIVER : Tension lipidosis; mild [LIVER : Focus, pale : (Comment) 1, right lateral near hilus (G)]
NERVE, SCIATIC : Degeneration; axonal, minimal
SITE, INJECTION : Infiltration, mononuclear cell; minimal
NERVE ROOT, DORSAL, LUMBAR : Degeneration; axonal, minimal

Histopathology - The following Tissues were Within Normal Limits:

BRAIN; EPIDIDYMIS; EYE; GANGLION, DORSAL ROOT, CERVICAL; GANGLION, DORSAL ROOT, THORACIC; GLAND, ADRENAL; GLAND, PARATHYROID; GLAND, PITUITARY; GLAND, PROSTATE; GLAND, THYROID; KIDNEY; LUNG; LYMPH NODE, CERVICAL; LYMPH NODE, ILIAC; LYMPH NODE, MANDIBULAR; LYMPH NODE, MESENTERIC; MUSCLE, BICEPS FEMORIS; MUSCLE, GASTROCNEMIUS; NERVE, OPTIC; NERVE, TIBIAL; SPINAL CORD, CERVICAL; SPINAL CORD, LUMBAR; SPINAL CORD, THORACIC; SPLEEN; TESTIS; THYMUS; NERVE ROOT, VENTRAL, CERVICAL; NERVE ROOT, VENTRAL, THORACIC; NERVE ROOT, VENTRAL, LUMBAR; NERVE ROOT, DORSAL, CERVICAL; NERVE ROOT, DORSAL, THORACIC

Histopathology - The following Tissues were Not Examined:

None

Individual Macroscopic and Microscopic Pathology:

5550008

Animal:	3009	Group:	3	Sex:	Male
Species:	Rat	Strain:	Sprague Dawley		
		Dose:	1.1x 10E12 vg		
		Removal Reason: Recovery Euthanasia			
		Study Day (Week) of Death: 29 (5)			

Gross Pathology Animal Details:

No animal details found

Gross Pathology Observations [Correlation]:

LIVER : Focus, pale : (Comment) 1, fissure, right medial (TGL)

Any remaining study plan required tissues, which have been examined, have no visible lesions

Gross Pathology - The following Tissues were Not Examined:

None

Histopathology Animal Details:

No animal details found

Histopathology Observations [Correlation]:

GANGLION, DORSAL ROOT, LUMBAR : Infiltration, mononuclear cell; minimal
GANGLION, DORSAL ROOT, LUMBAR : Degeneration; axonal, minimal
GLAND, PARATHYROID : (Note) One Of A Pair Available For Evaluation.
GLAND, PROSTATE : Infiltration, mononuclear cell; minimal
KIDNEY : Infiltration, mononuclear cell; minimal
LIVER : Vacuolation; hepatocellular, periportal, minimal
LIVER : Tension lipidosis; mild [LIVER : Focus, pale : (Comment) 1, fissure, right medial (G)]
LIVER : Infiltration, mixed cell; minimal
NERVE, SCIATIC : Degeneration; axonal, minimal
NERVE, TIBIAL : Degeneration; axonal, minimal
SITE, INJECTION : Infiltration, mononuclear cell; minimal, leptomeninges
SITE, INJECTION : Degeneration; axonal, minimal, cauda equina
NERVE ROOT, DORSAL, LUMBAR : Degeneration; axonal, minimal

Histopathology - The following Tissues were Within Normal Limits:

BRAIN; EPIDIDYMIS; EYE; GANGLION, DORSAL ROOT, CERVICAL; GANGLION, DORSAL ROOT, THORACIC; GLAND, ADRENAL; GLAND, PARATHYROID; GLAND, PITUITARY; GLAND, THYROID; HEART; LUNG; LYMPH NODE, CERVICAL; LYMPH NODE, ILIAC; LYMPH NODE, MANDIBULAR; LYMPH NODE, MESENTERIC; MUSCLE, BICEPS FEMORIS; MUSCLE, GASTROCNEMIUS; NERVE, OPTIC; SPINAL CORD, CERVICAL; SPINAL CORD, LUMBAR; SPINAL CORD, THORACIC; SPLEEN; TESTIS; THYMUS; NERVE ROOT, VENTRAL, CERVICAL; NERVE ROOT, VENTRAL, THORACIC; NERVE ROOT, VENTRAL, LUMBAR; NERVE ROOT, DORSAL, CERVICAL; NERVE ROOT, DORSAL, THORACIC

Histopathology - The following Tissues were Not Examined:

None

Individual Macroscopic and Microscopic Pathology:

5550008

Animal: 3010	Group: 3	Sex: Male
Species: Rat	Strain: Sprague Dawley<br>Dose: 1.1x 10E12 vg<br>Removal Reason: Recovery Euthanasia<br>Study Day (Week) of Death: 29 (5)	

Gross Pathology Animal Details:

No animal details found

Gross Pathology Observations [Correlation]:

GENERAL OBSERVATIONS : (Comment) Subcutis lesion submitted on plastic in bag 1.
SUBCUTIS : Focus, dark : (Comment) 2, lumbar (TGL)

Any remaining study plan required tissues, which have been examined, have no visible lesions

Gross Pathology - The following Tissues were Not Examined:

None

Histopathology Animal Details:

No animal details found

Histopathology Observations [Correlation]:

GANGLION, DORSAL ROOT, LUMBAR : Infiltration, mononuclear cell; minimal
GANGLION, DORSAL ROOT, LUMBAR : Degeneration; axonal, minimal
GANGLION, DORSAL ROOT, LUMBAR : Degeneration; neuronal, minimal
KIDNEY : Cast; hyaline, minimal
LIVER : Infiltration, mixed cell; minimal
LIVER : Infiltration, mononuclear cell; periportal, minimal
NERVE, SCIATIC : Degeneration; axonal, mild
NERVE, TIBIAL : Degeneration; axonal, mild
SITE, INJECTION : Degeneration; axonal, minimal, cauda equina
SUBCUTIS : Hemorrhage; acute, minimal [SUBCUTIS : Focus, dark : (Comment) 2, lumbar (G)]
SUBCUTIS : Fibroplasia; regionally extensive, mild : (Comment) with seroma formation and attendant mononuclear cell infiltration (suture knot granuloma also in section)
NERVE ROOT, DORSAL, LUMBAR : Degeneration; axonal, mild

Histopathology - The following Tissues were Within Normal Limits:

BRAIN; EPIDIDYMIS; EYE; GANGLION, DORSAL ROOT, CERVICAL; GANGLION, DORSAL ROOT, THORACIC; GLAND, ADRENAL; GLAND, PITUITARY; GLAND, PROSTATE; GLAND, THYROID; HEART; LUNG; LYMPH NODE, CERVICAL; LYMPH NODE, ILIAC; LYMPH NODE, MANDIBULAR; LYMPH NODE, MESENTERIC; MUSCLE, BICEPS FEMORIS; MUSCLE, GASTROCNEMIUS; NERVE, OPTIC; SPINAL CORD, CERVICAL; SPINAL CORD, LUMBAR; SPINAL CORD, THORACIC; SPLEEN; TESTIS; THYMUS; NERVE ROOT, VENTRAL, CERVICAL; NERVE ROOT, VENTRAL, THORACIC; NERVE ROOT, VENTRAL, LUMBAR; NERVE ROOT, DORSAL, CERVICAL; NERVE ROOT, DORSAL, THORACIC

Histopathology - The following Tissues were Not Examined:

GLAND, PARATHYROID - Not Present In Section.

Individual Macroscopic and Microscopic Pathology:

5550008

Animal: 3011	Group: 3	Sex: Male
Species: Rat	Strain: Sprague Dawley<br>Dose: 1.1x 10E12 vg<br>Removal Reason: Recovery Euthanasia<br>Study Day (Week) of Death: 29 (5)	

Gross Pathology Animal Details:

No animal details found

Gross Pathology Observations [Correlation]:

GENERAL OBSERVATIONS : (Comment) epididymis left in two pieces
EPIDIDYMIS : Small : (Comment) left (TGL)
TESTIS : Small : (Comment) left (TGL)
TESTIS : Abnormal consistency; soft : (Comment) left (TGL)

Any remaining study plan required tissues, which have been examined, have no visible lesions

Gross Pathology - The following Tissues were Not Examined:

None

Histopathology Animal Details:

No animal details found

Histopathology Observations [Correlation]:

EPIDIDYMIS : Cellular debris; unilateral, moderate
EPIDIDYMIS : Sperm, decreased; unilateral, moderate [EPIDIDYMIS : Small : (Comment) left (G)]
GANGLION, DORSAL ROOT, LUMBAR : Infiltration, mononuclear cell; minimal
GANGLION, DORSAL ROOT, LUMBAR : Degeneration; neuronal, minimal
GLAND, PITUITARY : (Note) Only Pars Distalis Available For Evaluation.
KIDNEY : Chronic progressive nephropathy; minimal
NERVE, SCIATIC : Degeneration; axonal, minimal
NERVE, TIBIAL : Degeneration; axonal, minimal
TESTIS : Degeneration/atrophy; unilateral, marked, seminiferous tubule [TESTIS : Abnormal consistency; soft : (Comment) left (G) | TESTIS : Small : (Comment) left (G)]
NERVE ROOT, DORSAL, LUMBAR : Degeneration; axonal, minimal

Histopathology - The following Tissues were Within Normal Limits:

BRAIN; EYE; GANGLION, DORSAL ROOT, CERVICAL; GANGLION, DORSAL ROOT, THORACIC; GLAND, ADRENAL; GLAND, PARATHYROID; GLAND, PITUITARY; GLAND, PROSTATE; GLAND, THYROID; HEART; LIVER; LUNG; LYMPH NODE, CERVICAL; LYMPH NODE, ILIAC; LYMPH NODE, MANDIBULAR; LYMPH NODE, MESENTERIC; MUSCLE, BICEPS FEMORIS; MUSCLE, GASTROCNEMIUS; NERVE, OPTIC; SITE, INJECTION; SPINAL CORD, CERVICAL; SPINAL CORD, LUMBAR; SPINAL CORD, THORACIC; SPLEEN; THYMUS; NERVE ROOT, VENTRAL, CERVICAL; NERVE ROOT, VENTRAL, THORACIC; NERVE ROOT, VENTRAL, LUMBAR; NERVE ROOT, DORSAL, CERVICAL; NERVE ROOT, DORSAL, THORACIC

Histopathology - The following Tissues were Not Examined:

None

Appendix 18

Appendix 4

Individual Macroscopic and Microscopic Pathology:

5550008

Animal:	3012	Group:	3	Sex:	Male
Species:	Rat	Strain:	Sprague Dawley		
		Dose:	1.1x 10E12 vg		
		Removal Reason:	Terminal Euthanasia		
		Study Day (Week) of Death:	8 (2)		

Gross Pathology Animal Details:

No animal details found

Gross Pathology Observations [Correlation]:

GENERAL OBSERVATIONS : (Comment) Right lymph node deep cervical not submitted for histopathological evaluation (frozen).
LIVER : Focus, pale : (Comment) 1, fissure, right medial. (TGL)
LYMPH NODE, CERVICAL : Focus, dark : (Comment) >10, bilateral. (TGL)

Any remaining study plan required tissues, which have been examined, have no visible lesions

Gross Pathology - The following Tissues were Not Examined:

None

Histopathology Animal Details:

No animal details found

Histopathology Observations [Correlation]:

LIVER : Infiltration, mixed cell; minimal
LYMPH NODE, CERVICAL : Hemorrhage; acute, mild [LYMPH NODE, CERVICAL : Focus, dark : (Comment) >10, bilateral. (G)]
SITE, INJECTION : Degeneration; axonal, minimal, cauda equina
NO CORRELATE : No correlating lesion [LIVER : Focus, pale : (Comment) 1, fissure, right medial. (G)]
NERVE ROOT, DORSAL, LUMBAR : Degeneration; axonal, minimal

Histopathology - The following Tissues were Within Normal Limits:

BRAIN; EPIDIDYMIS; EYE; GANGLION, DORSAL ROOT, CERVICAL; GANGLION, DORSAL ROOT, LUMBAR; GANGLION, DORSAL ROOT, THORACIC; GLAND, ADRENAL; GLAND, PARATHYROID; GLAND, PITUITARY; GLAND, PROSTATE; GLAND, THYROID; HEART; KIDNEY; LUNG; LYMPH NODE, ILIAC; LYMPH NODE, MANDIBULAR; LYMPH NODE, MESENTERIC; MUSCLE, BICEPS FEMORIS; MUSCLE, GASTROCNEMIUS; NERVE, OPTIC; NERVE, SCIATIC; NERVE, TIBIAL; SPINAL CORD, CERVICAL; SPINAL CORD, LUMBAR; SPINAL CORD, THORACIC; SPLEEN; TESTIS; THYMUS; NERVE ROOT, VENTRAL, CERVICAL; NERVE ROOT, VENTRAL, THORACIC; NERVE ROOT, VENTRAL, LUMBAR; NERVE ROOT, DORSAL, CERVICAL; NERVE ROOT, DORSAL, THORACIC

Histopathology - The following Tissues were Not Examined:

None

Individual Macroscopic and Microscopic Pathology:

5550008

Animal: 3013	Group: 3	Sex: Male
Species: Rat	Strain: Sprague Dawley<br>Dose: 1.1x 10E12 vg<br>Removal Reason: Terminal Euthanasia<br>Study Day (Week) of Death: 8 (2)	

Gross Pathology Animal Details:

No animal details found

Gross Pathology Observations [Correlation]:

GENERAL OBSERVATIONS : (Comment) Eye left damaged. Right lymph node deep cervical not submitted for histopathological evaluation (frozen).
LYMPH NODE, CERVICAL : Focus, dark : (Comment) 1, bilateral (TGL)
LYMPH NODE, MANDIBULAR : Discoloration, mottled : (Comment) bilateral (TGL)

Any remaining study plan required tissues, which have been examined, have no visible lesions

Gross Pathology - The following Tissues were Not Examined:

None

Histopathology Animal Details:

No animal details found

Histopathology Observations [Correlation]:

GANGLION, DORSAL ROOT, LUMBAR : Infiltration, mononuclear cell; minimal
GLAND, PARATHYROID : (Note) One Of A Pair Available For Evaluation.
GLAND, THYROID : (Note) One Of A Pair Available For Evaluation.
LIVER : Extramedullary hematopoiesis; minimal
LYMPH NODE, CERVICAL : Hemorrhage; acute, minimal [LYMPH NODE, CERVICAL : Focus, dark : (Comment) 1, bilateral (G)]
LYMPH NODE, MANDIBULAR : Hemorrhage; acute, mild [LYMPH NODE, MANDIBULAR : Discoloration, mottled : (Comment) bilateral (G)]
NERVE ROOT, DORSAL, LUMBAR : Degeneration; axonal, minimal

Histopathology - The following Tissues were Within Normal Limits:

BRAIN; EPIDIDYMIS; EYE; GANGLION, DORSAL ROOT, CERVICAL; GANGLION, DORSAL ROOT, THORACIC; GLAND, ADRENAL; GLAND, PARATHYROID; GLAND, PITUITARY; GLAND, PROSTATE; GLAND, THYROID; HEART; KIDNEY; LUNG; LYMPH NODE, ILIAC; LYMPH NODE, MESENTERIC; MUSCLE, BICEPS FEMORIS; MUSCLE, GASTROCNEMIUS; NERVE, SCIATIC; NERVE, TIBIAL; SITE, INJECTION; SPINAL CORD, CERVICAL; SPINAL CORD, LUMBAR; SPINAL CORD, THORACIC; SPLEEN; TESTIS; THYMUS; NERVE ROOT, VENTRAL, CERVICAL; NERVE ROOT, VENTRAL, THORACIC; NERVE ROOT, VENTRAL, LUMBAR; NERVE ROOT, DORSAL, CERVICAL; NERVE ROOT, DORSAL, THORACIC

Histopathology - The following Tissues were Not Examined:

NERVE, OPTIC - Not Present In Section.

Appendix 18

Appendix 4

Individual Macroscopic and Microscopic Pathology:

5550008

Animal:	3014	Group:	3	Sex:	Male
Species:	Rat	Strain:	Sprague Dawley		
		Dose:	1.1x 10E12 vg		
		Removal Reason: Recovery Euthanasia			
		Study Day (Week) of Death: 91 (13)			

Gross Pathology Animal Details:

No animal details found

Gross Pathology Observations [Correlation]:

EPIDIDYMIS : Focus, raised : (Comment) pale, soft, 3, body, right, cut surface: material, pale, thick. (TGL)
LIVER : Focus, pale : (Comment) 1, fissure, right medial. (TGL)

Any remaining study plan required tissues, which have been examined, have no visible lesions

Gross Pathology - The following Tissues were Not Examined:

None

Histopathology Animal Details:

No animal details found

Histopathology Observations [Correlation]:

EPIDIDYMIS : Sperm granuloma; unilateral, minimal [EPIDIDYMIS : Focus, raised : (Comment) pale, soft, 3, body, right, cut surface: material, pale, thick. (G)]
GLAND, PARATHYROID : (Note) One Of A Pair Available For Evaluation.
HEART : Infiltration, mononuclear cell/degeneration, myofiber; minimal
KIDNEY : Infiltration, mononuclear cell; minimal
KIDNEY : Chronic progressive nephropathy; minimal
LIVER : Infiltration, mixed cell; minimal
NO CORRELATE : No correlating lesion [LIVER : Focus, pale : (Comment) 1, fissure, right medial. (G)]
NERVE ROOT, DORSAL, LUMBAR : Degeneration; axonal, minimal

Histopathology - The following Tissues were Within Normal Limits:

BRAIN; EYE; GANGLION, DORSAL ROOT, CERVICAL; GANGLION, DORSAL ROOT, LUMBAR; GANGLION, DORSAL ROOT, THORACIC; GLAND, ADRENAL; GLAND, PARATHYROID; GLAND, PITUITARY; GLAND, PROSTATE; GLAND, THYROID; LUNG; LYMPH NODE, CERVICAL; LYMPH NODE, ILIAC; LYMPH NODE, MANDIBULAR; LYMPH NODE, MESENTERIC; MUSCLE, BICEPS FEMORIS; MUSCLE, GASTROCNEMIUS; NERVE, OPTIC; NERVE, SCIATIC; NERVE, TIBIAL; SITE, INJECTION; SPINAL CORD, CERVICAL; SPINAL CORD, LUMBAR; SPINAL CORD, THORACIC; SPLEEN; TESTIS; THYMUS; NERVE ROOT, VENTRAL, CERVICAL; NERVE ROOT, VENTRAL, THORACIC; NERVE ROOT, VENTRAL, LUMBAR; NERVE ROOT, DORSAL, CERVICAL; NERVE ROOT, DORSAL, THORACIC

Histopathology - The following Tissues were Not Examined:

None

Individual Macroscopic and Microscopic Pathology:

5550008

Animal: 3015	Group: 3	Sex: Male
Species: Rat	Strain: Sprague Dawley	
	Dose: 1.1x 10E12 vg	
	Removal Reason: Recovery Euthanasia	
	Study Day (Week) of Death: 91 (13)	

Gross Pathology Animal Details:

No animal details found

Gross Pathology Observations [Correlation]:

GENERAL OBSERVATIONS : (Comment) Optic nerve left submitted in cass C
LYMPH NODE, MANDIBULAR : Enlargement : (Comment) left (TGL)
THYMUS : Discoloration, mottled : (Comment) left (TGL)
TONGUE : Focus, dark : (Comment) 1, tip (TGL)

Any remaining study plan required tissues, which have been examined, have no visible lesions

Gross Pathology - The following Tissues were Not Examined:

None

Histopathology Animal Details:

No animal details found

Histopathology Observations [Correlation]:

GANGLION, DORSAL ROOT, LUMBAR : Degeneration; neuronal, minimal
GLAND, ADRENAL : Vacuolation; cortical, minimal
GLAND, PITUITARY : (Note) Only Pars Distalis Available For Evaluation.
KIDNEY : Infiltration, mononuclear cell; minimal
LIVER : Infiltration, mononuclear cell; periportal, minimal
NERVE, SCIATIC : Degeneration; axonal, minimal
NERVE, TIBIAL : Degeneration; axonal, minimal
SITE, INJECTION : Infiltration, mononuclear cell; minimal, leptomeninges
NO CORRELATE : No correlating lesion [LYMPH NODE, MANDIBULAR : Enlargement : (Comment) left (G) | THYMUS : Discoloration, mottled : (Comment) left (G) | TONGUE : Focus, dark : (Comment) 1, tip (G)]
NERVE ROOT, DORSAL, LUMBAR : Degeneration; axonal, minimal

Histopathology - The following Tissues were Within Normal Limits:

BRAIN; EPIDIDYMIS; EYE; GANGLION, DORSAL ROOT, CERVICAL; GANGLION, DORSAL ROOT, THORACIC; GLAND, PARATHYROID; GLAND, PITUITARY; GLAND, PROSTATE; GLAND, THYROID; HEART; LUNG; LYMPH NODE, CERVICAL; LYMPH NODE, ILIAC; LYMPH NODE, MANDIBULAR; LYMPH NODE, MESENTERIC; MUSCLE, BICEPS FEMORIS; MUSCLE, GASTROCNEMIUS; NERVE, OPTIC; SPINAL CORD, CERVICAL; SPINAL CORD, LUMBAR; SPINAL CORD, THORACIC; SPLEEN; TESTIS; THYMUS; TONGUE; NERVE ROOT, VENTRAL, CERVICAL; NERVE ROOT, VENTRAL, THORACIC; NERVE ROOT, VENTRAL, LUMBAR; NERVE ROOT, DORSAL, CERVICAL; NERVE ROOT, DORSAL, THORACIC

Histopathology - The following Tissues were Not Examined:

None

Individual Macroscopic and Microscopic Pathology:

5550008

Animal: 3501	Group: 3	Sex: Female
Species: Rat	Strain: Sprague Dawley<br>Dose: 1.1x 10E12 vg<br>Removal Reason: Recovery Euthanasia<br>Study Day (Week) of Death: 91 (13)	

Gross Pathology Animal Details:

No animal details found

Gross Pathology Observations [Correlation]:

GLAND, ADRENAL : Enlargement : (Comment) left (TGL)
GLAND, PITUITARY : Enlargement (TGL)

Any remaining study plan required tissues, which have been examined, have no visible lesions

Gross Pathology - The following Tissues were Not Examined:

None

Histopathology Animal Details:

No animal details found

Histopathology Observations [Correlation]:

GANGLION, DORSAL ROOT, LUMBAR : Infiltration, mononuclear cell; minimal
GANGLION, DORSAL ROOT, LUMBAR : Degeneration; axonal, minimal
GLAND, PITUITARY : (Note) Only Pars Distalis Available For Evaluation.
GLAND, PITUITARY : Hypertrophy/hyperplasia; diffuse, mild, pars distalis [GLAND, PITUITARY : Enlargement (G)]
KIDNEY : Infiltration, mononuclear cell; minimal
LIVER : Infiltration, mixed cell; minimal
NERVE, SCIATIC : Degeneration; axonal, minimal
SITE, INJECTION : Infiltration, mononuclear cell; minimal, leptomeninges
SITE, INJECTION : Degeneration; axonal, minimal, cauda equina
NO CORRELATE : No correlating lesion [GLAND, ADRENAL : Enlargement : (Comment) left (G)]
NERVE ROOT, DORSAL, LUMBAR : Degeneration; axonal, minimal

Histopathology - The following Tissues were Within Normal Limits:

BRAIN; CERVIX; EYE; GANGLION, DORSAL ROOT, CERVICAL; GANGLION, DORSAL ROOT, THORACIC; GLAND, ADRENAL; GLAND, THYROID; HEART; LUNG; LYMPH NODE, CERVICAL; LYMPH NODE, ILIAC; LYMPH NODE, MANDIBULAR; LYMPH NODE, MESENTERIC; MUSCLE, BICEPS FEMORIS; MUSCLE, GASTROCNEMIUS; NERVE, OPTIC; NERVE, TIBIAL; OVARY; SPINAL CORD, CERVICAL; SPINAL CORD, LUMBAR; SPINAL CORD, THORACIC; SPLEEN; THYMUS; UTERUS; NERVE ROOT, VENTRAL, CERVICAL; NERVE ROOT, VENTRAL, THORACIC; NERVE ROOT, VENTRAL, LUMBAR; NERVE ROOT, DORSAL, CERVICAL; NERVE ROOT, DORSAL, THORACIC

Histopathology - The following Tissues were Not Examined:

GLAND, PARATHYROID - Not Present In Section.

Individual Macroscopic and Microscopic Pathology:

5550008

Animal:	3502	Group:	3	Sex:	Female
Species:	Rat	Strain:	Sprague Dawley		
		Dose:	1.1x 10E12 vg		
		Removal Reason: Recovery Euthanasia			
		Study Day (Week) of Death: 91 (13)			

Gross Pathology Animal Details:

No animal details found

Gross Pathology Observations [Correlation]:

GENERAL OBSERVATIONS : (Comment) Optic nerve left submitted in cassette C. Lymph node deep cervical lesion submitted with left as representative section.
LIVER : Focus, pale : (Comment) 1, fissure, right medial (TGL)
LYMPH NODE, CERVICAL : Enlargement : (Comment) bilateral (TGL)
LYMPH NODE, MANDIBULAR : Enlargement : (Comment) left (TGL)
SITE, INJECTION : (Comment) puncture can be seen ventrally at L4

Any remaining study plan required tissues, which have been examined, have no visible lesions

Gross Pathology - The following Tissues were Not Examined:

None

Histopathology Animal Details:

No animal details found

Histopathology Observations [Correlation]:

GANGLION, DORSAL ROOT, LUMBAR : Degeneration; neuronal, minimal
GLAND, PARATHYROID : (Note) One Of A Pair Available For Evaluation.
GLAND, THYROID : (Note) One Of A Pair Available For Evaluation.
KIDNEY : Infiltration, mononuclear cell; minimal
KIDNEY : Chronic progressive nephropathy; minimal
LIVER : Infiltration, mixed cell; minimal
LYMPH NODE, MANDIBULAR : Cellularity, increased; lymphoid, mild [LYMPH NODE, MANDIBULAR : Enlargement : (Comment) left (G)]
NERVE, SCIATIC : Degeneration; axonal, minimal
NERVE, TIBIAL : Degeneration; axonal, minimal
SITE, INJECTION : Infiltration, mononuclear cell; minimal, leptomeninges
SPINAL CORD, LUMBAR : Infiltration, mononuclear cell; minimal, leptomeninges
NO CORRELATE : No correlating lesion [LIVER : Focus, pale : (Comment) 1, fissure, right medial (G) | LYMPH NODE, CERVICAL : Enlargement : (Comment) bilateral (G)]
NERVE ROOT, DORSAL, LUMBAR : Degeneration; axonal, minimal

Histopathology - The following Tissues were Within Normal Limits:

BRAIN; CERVIX; EYE; GANGLION, DORSAL ROOT, CERVICAL; GANGLION, DORSAL ROOT, THORACIC; GLAND, ADRENAL; GLAND, PARATHYROID; GLAND, PITUITARY; GLAND, THYROID; HEART; LUNG; LYMPH NODE, CERVICAL; LYMPH NODE, ILIAC; LYMPH NODE, MESENTERIC; MUSCLE, BICEPS FEMORIS; MUSCLE, GASTROCNEMIUS; NERVE, OPTIC; OVARY; SPINAL CORD, CERVICAL; SPINAL CORD, THORACIC; SPLEEN; THYMUS; UTERUS; NERVE ROOT, VENTRAL, CERVICAL; NERVE ROOT, VENTRAL, THORACIC; NERVE ROOT, VENTRAL, LUMBAR; NERVE ROOT, DORSAL, CERVICAL; NERVE ROOT, DORSAL, THORACIC

Appendix 18

Appendix 4

Individual Macroscopic and Microscopic Pathology:

5550008

Animal: 3502 (Continued)	Group: 3	Sex: Female

Histopathology - The following Tissues were Not Examined:

None

Individual Macroscopic and Microscopic Pathology:

5550008

Animal: 3503	Group: 3	Sex: Female
Species: Rat	Strain: Sprague Dawley<br>Dose: 1.1x 10E12 vg<br>Removal Reason: Recovery Euthanasia<br>Study Day (Week) of Death: 91 (13)	

Gross Pathology Animal Details:

No animal details found

Gross Pathology Observations [Correlation]:

No observations found

Any remaining study plan required tissues, which have been examined, have no visible lesions

Gross Pathology - The following Tissues were Not Examined:

None

Histopathology Animal Details:

No animal details found

Histopathology Observations [Correlation]:

GANGLION, DORSAL ROOT, LUMBAR : Infiltration, mononuclear cell; minimal
GLAND, PARATHYROID : (Note) One Of A Pair Available For Evaluation.
LIVER : Infiltration, mixed cell; minimal
NERVE, SCIATIC : Degeneration; axonal, minimal
NERVE, TIBIAL : Degeneration; axonal, minimal
SITE, INJECTION : Degeneration; axonal, minimal, cauda equina
SPINAL CORD, LUMBAR : Infiltration, mononuclear cell; minimal, leptomeninges
NERVE ROOT, DORSAL, LUMBAR : Degeneration; axonal, minimal

Histopathology - The following Tissues were Within Normal Limits:

BRAIN; CERVIX; EYE; GANGLION, DORSAL ROOT, CERVICAL; GANGLION, DORSAL ROOT, THORACIC; GLAND, ADRENAL; GLAND, PARATHYROID; GLAND, PITUITARY; GLAND, THYROID; HEART; KIDNEY; LUNG; LYMPH NODE, CERVICAL; LYMPH NODE, ILIAC; LYMPH NODE, MANDIBULAR; LYMPH NODE, MESENTERIC; MUSCLE, BICEPS FEMORIS; MUSCLE, GASTROCNEMIUS; NERVE, OPTIC; OVARY; SPINAL CORD, CERVICAL; SPINAL CORD, THORACIC; SPLEEN; THYMUS; UTERUS; NERVE ROOT, VENTRAL, CERVICAL; NERVE ROOT, VENTRAL, THORACIC; NERVE ROOT, VENTRAL, LUMBAR; NERVE ROOT, DORSAL, CERVICAL; NERVE ROOT, DORSAL, THORACIC

Histopathology - The following Tissues were Not Examined:

None

Individual Macroscopic and Microscopic Pathology:

5550008

Animal:	3504	Group:	3	Sex:	Female
Species:	Rat	Strain:	Sprague Dawley		
		Dose:	1.1x 10E12 vg		
		Removal Reason: Recovery Euthanasia			
		Study Day (Week) of Death: 29 (5)			

Gross Pathology Animal Details:

No animal details found

Gross Pathology Observations [Correlation]:

LIVER : Focus, pale : (Comment) 1, fissure, right medial (TGL)

Any remaining study plan required tissues, which have been examined, have no visible lesions

Gross Pathology - The following Tissues were Not Examined:

None

Histopathology Animal Details:

No animal details found

Histopathology Observations [Correlation]:

GANGLION, DORSAL ROOT, LUMBAR : Infiltration, mononuclear cell; minimal
GLAND, PARATHYROID : (Note) One Of A Pair Available For Evaluation.
LIVER : Tension lipidosis; mild [LIVER : Focus, pale : (Comment) 1, fissure, right medial (G)]
NERVE, SCIATIC : Degeneration; axonal, minimal
NERVE, TIBIAL : Degeneration; axonal, minimal
SITE, INJECTION : Degeneration; axonal, mild, cauda equina
NERVE ROOT, DORSAL, LUMBAR : Degeneration; axonal, minimal

Histopathology - The following Tissues were Within Normal Limits:

BRAIN; CERVIX; EYE; GANGLION, DORSAL ROOT, CERVICAL; GANGLION, DORSAL ROOT, THORACIC; GLAND, ADRENAL; GLAND, PARATHYROID; GLAND, PITUITARY; GLAND, THYROID; HEART; KIDNEY; LUNG; LYMPH NODE, CERVICAL; LYMPH NODE, ILIAC; LYMPH NODE, MANDIBULAR; LYMPH NODE, MESENTERIC; MUSCLE, BICEPS FEMORIS; MUSCLE, GASTROCNEMIUS; NERVE, OPTIC; OVARY; SPINAL CORD, CERVICAL; SPINAL CORD, LUMBAR; SPINAL CORD, THORACIC; SPLEEN; THYMUS; UTERUS; NERVE ROOT, VENTRAL, CERVICAL; NERVE ROOT, VENTRAL, THORACIC; NERVE ROOT, VENTRAL, LUMBAR; NERVE ROOT, DORSAL, CERVICAL; NERVE ROOT, DORSAL, THORACIC

Histopathology - The following Tissues were Not Examined:

None

Appendix 18

Appendix 4

Individual Macroscopic and Microscopic Pathology:

5550008

Animal:	3505	Group:	3	Sex:	Female
Species:	Rat	Strain:	Sprague Dawley		
		Dose:	1.1x 10E12 vg		
		Removal Reason:	Recovery Euthanasia		
		Study Day (Week) of Death:	29 (5)		

Gross Pathology Animal Details:

No animal details found

Gross Pathology Observations [Correlation]:

GENERAL OBSERVATIONS : (Comment) oviduct left submitted in two pieces

Any remaining study plan required tissues, which have been examined, have no visible lesions

Gross Pathology - The following Tissues were Not Examined:

None

Histopathology Animal Details:

No animal details found

Histopathology Observations [Correlation]:

GLAND, PARATHYROID : (Note) One Of A Pair Available For Evaluation.
LIVER : Infiltration, mixed cell; minimal
NERVE, SCIATIC : Degeneration; axonal, minimal
NERVE, TIBIAL : Degeneration; axonal, minimal
SITE, INJECTION : Infiltration, mononuclear cell; minimal, leptomeninges
NERVE ROOT, DORSAL, LUMBAR : Degeneration; axonal, minimal

Histopathology - The following Tissues were Within Normal Limits:

BRAIN; CERVIX; EYE; GANGLION, DORSAL ROOT, CERVICAL; GANGLION, DORSAL ROOT, LUMBAR; GANGLION, DORSAL ROOT, THORACIC; GLAND, ADRENAL; GLAND, PARATHYROID; GLAND, PITUITARY; GLAND, THYROID; HEART; KIDNEY; LUNG; LYMPH NODE, CERVICAL; LYMPH NODE, ILIAC; LYMPH NODE, MANDIBULAR; LYMPH NODE, MESENTERIC; MUSCLE, BICEPS FEMORIS; MUSCLE, GASTROCNEMIUS; NERVE, OPTIC; OVARY; SPINAL CORD, CERVICAL; SPINAL CORD, LUMBAR; SPINAL CORD, THORACIC; SPLEEN; THYMUS; UTERUS; NERVE ROOT, VENTRAL, CERVICAL; NERVE ROOT, VENTRAL, THORACIC; NERVE ROOT, VENTRAL, LUMBAR; NERVE ROOT, DORSAL, CERVICAL; NERVE ROOT, DORSAL, THORACIC

Histopathology - The following Tissues were Not Examined:

None

Individual Macroscopic and Microscopic Pathology:

5550008

Animal: 3506	Group: 3	Sex: Female
Species: Rat	Strain: Sprague Dawley<br>Dose: 1.1x 10E12 vg<br>Removal Reason: Recovery Euthanasia<br>Study Day (Week) of Death: 29 (5)	

Gross Pathology Animal Details:

No animal details found

Gross Pathology Observations [Correlation]:

GENERAL OBSERVATIONS : (Comment) Lymph node deep cervical lesion representative section submitted with left side
LYMPH NODE, CERVICAL : Focus, dark : (Comment) 1 to 3, bilateral (TGL)
LYMPH NODE, MANDIBULAR : Focus, dark : (Comment) 1, left (TGL)

Any remaining study plan required tissues, which have been examined, have no visible lesions

Gross Pathology - The following Tissues were Not Examined:

None

Histopathology Animal Details:

No animal details found

Histopathology Observations [Correlation]:

GLAND, PITUITARY : (Note) Only Pars Distalis Available For Evaluation.
LYMPH NODE, CERVICAL : Hemorrhage; acute, mild [LYMPH NODE, CERVICAL : Focus, dark : (Comment) 1 to 3, bilateral (G)]
LYMPH NODE, MANDIBULAR : Hemorrhage; acute, mild [LYMPH NODE, MANDIBULAR : Focus, dark : (Comment) 1, left (G)]
NERVE, SCIATIC : Degeneration; axonal, minimal
NERVE, TIBIAL : Degeneration; axonal, minimal

Histopathology - The following Tissues were Within Normal Limits:

BRAIN; CERVIX; EYE; GANGLION, DORSAL ROOT, CERVICAL; GANGLION, DORSAL ROOT, LUMBAR; GANGLION, DORSAL ROOT, THORACIC; GLAND, ADRENAL; GLAND, PARATHYROID; GLAND, PITUITARY; GLAND, THYROID; HEART; KIDNEY; LIVER; LUNG; LYMPH NODE, ILIAC; LYMPH NODE, MESENTERIC; MUSCLE, BICEPS FEMORIS; MUSCLE, GASTROCNEMIUS; NERVE, OPTIC; OVARY; SITE, INJECTION; SPINAL CORD, CERVICAL; SPINAL CORD, LUMBAR; SPINAL CORD, THORACIC; SPLEEN; THYMUS; UTERUS; NERVE ROOT, VENTRAL, CERVICAL; NERVE ROOT, VENTRAL, THORACIC; NERVE ROOT, VENTRAL, LUMBAR; NERVE ROOT, DORSAL, CERVICAL; NERVE ROOT, DORSAL, LUMBAR; NERVE ROOT, DORSAL, THORACIC

Histopathology - The following Tissues were Not Examined:

None

Individual Macroscopic and Microscopic Pathology:

5550008

Animal: 3507	Group: 3	Sex: Female
Species: Rat	Strain: Sprague Dawley	
	Dose: 1.1x 10E12 vg	
	Removal Reason: Terminal Euthanasia	
	Study Day (Week) of Death: 8 (2)	

Gross Pathology Animal Details:

No animal details found

Gross Pathology Observations [Correlation]:

No observations found

Any remaining study plan required tissues, which have been examined, have no visible lesions

Gross Pathology - The following Tissues were Not Examined:

None

Histopathology Animal Details:

No animal details found

Histopathology Observations [Correlation]:

GANGLION, DORSAL ROOT, LUMBAR : Infiltration, mononuclear cell; minimal
GLAND, PARATHYROID : (Note) One Of A Pair Available For Evaluation.

Histopathology - The following Tissues were Within Normal Limits:

BRAIN; CERVIX; EYE; GANGLION, DORSAL ROOT, CERVICAL; GANGLION, DORSAL ROOT, THORACIC; GLAND, ADRENAL; GLAND, PARATHYROID; GLAND, PITUITARY; GLAND, THYROID; HEART; KIDNEY; LIVER; LUNG; LYMPH NODE, CERVICAL; LYMPH NODE, ILIAC; LYMPH NODE, MANDIBULAR; LYMPH NODE, MESENTERIC; MUSCLE, BICEPS FEMORIS; MUSCLE, GASTROCNEMIUS; NERVE, OPTIC; NERVE, SCIATIC; NERVE, TIBIAL; OVARY; SITE, INJECTION; SPINAL CORD, CERVICAL; SPINAL CORD, LUMBAR; SPINAL CORD, THORACIC; SPLEEN; THYMUS; UTERUS; NERVE ROOT, VENTRAL, CERVICAL; NERVE ROOT, VENTRAL, THORACIC; NERVE ROOT, VENTRAL, LUMBAR; NERVE ROOT, DORSAL, CERVICAL; NERVE ROOT, DORSAL, LUMBAR; NERVE ROOT, DORSAL, THORACIC

Histopathology - The following Tissues were Not Examined:

None

Appendix 18

Appendix 4

Individual Macroscopic and Microscopic Pathology:

5550008

Animal:	3508	Group:	3	Sex:	Female
Species:	Rat	Strain:	Sprague Dawley		
		Dose:	1.1x 10E12 vg		
		Removal Reason: Terminal Euthanasia			
		Study Day (Week) of Death: 8 (2)			

Gross Pathology Animal Details:

No animal details found

Gross Pathology Observations [Correlation]:

No observations found

Any remaining study plan required tissues, which have been examined, have no visible lesions

Gross Pathology - The following Tissues were Not Examined:

None

Histopathology Animal Details:

No animal details found

Histopathology Observations [Correlation]:

GLAND, PARATHYROID : (Note) One Of A Pair Available For Evaluation.

Histopathology - The following Tissues were Within Normal Limits:

BRAIN; CERVIX; EYE; GANGLION, DORSAL ROOT, CERVICAL; GANGLION, DORSAL ROOT, LUMBAR; GANGLION, DORSAL ROOT, THORACIC; GLAND, ADRENAL; GLAND, PARATHYROID; GLAND, PITUITARY; GLAND, THYROID; HEART; KIDNEY; LIVER; LUNG; LYMPH NODE, CERVICAL; LYMPH NODE, ILIAC; LYMPH NODE, MANDIBULAR; LYMPH NODE, MESENTERIC; MUSCLE, BICEPS FEMORIS; MUSCLE, GASTROCNEMIUS; NERVE, OPTIC; NERVE, SCIATIC; NERVE, TIBIAL; OVARY; SITE, INJECTION; SPINAL CORD, CERVICAL; SPINAL CORD, LUMBAR; SPINAL CORD, THORACIC; SPLEEN; THYMUS; UTERUS; NERVE ROOT, VENTRAL, CERVICAL; NERVE ROOT, VENTRAL, THORACIC; NERVE ROOT, VENTRAL, LUMBAR; NERVE ROOT, DORSAL, CERVICAL; NERVE ROOT, DORSAL, LUMBAR; NERVE ROOT, DORSAL, THORACIC

Histopathology - The following Tissues were Not Examined:

None

Appendix 18

Appendix 4

Individual Macroscopic and Microscopic Pathology:

5550008

Animal: 3509	Group: 3		Sex: Female
Species: Rat	Strain: Sprague Dawley		
	Dose: 1.1x 10E12 vg		
	Removal Reason: Recovery Euthanasia		
	Study Day (Week) of Death: 91 (13)		

Gross Pathology Animal Details:

No animal details found

Gross Pathology Observations [Correlation]:

LIVER : Focus, pale : (Comment) 1, fissure, right medial. (TGL)

Any remaining study plan required tissues, which have been examined, have no visible lesions

Gross Pathology - The following Tissues were Not Examined:

None

Histopathology Animal Details:

No animal details found

Histopathology Observations [Correlation]:

LIVER : Infiltration, mixed cell; minimal
NERVE, SCIATIC : Degeneration; axonal, minimal
NERVE, TIBIAL : Degeneration; axonal, minimal
SITE, INJECTION : Infiltration, mononuclear cell; minimal, leptomeninges
NO CORRELATE : No correlating lesion [LIVER : Focus, pale : (Comment) 1, fissure, right medial. (G)]
NERVE ROOT, DORSAL, LUMBAR : Degeneration; axonal, minimal

Histopathology - The following Tissues were Within Normal Limits:

BRAIN; CERVIX; EYE; GANGLION, DORSAL ROOT, CERVICAL; GANGLION, DORSAL ROOT, LUMBAR; GANGLION, DORSAL ROOT, THORACIC; GLAND, ADRENAL; GLAND, PITUITARY; GLAND, THYROID; HEART; KIDNEY; LUNG; LYMPH NODE, CERVICAL; LYMPH NODE, ILIAC; LYMPH NODE, MANDIBULAR; LYMPH NODE, MESENTERIC; MUSCLE, BICEPS FEMORIS; MUSCLE, GASTROCNEMIUS; NERVE, OPTIC; OVARY; SPINAL CORD, CERVICAL; SPINAL CORD, LUMBAR; SPINAL CORD, THORACIC; SPLEEN; THYMUS; UTERUS; NERVE ROOT, VENTRAL, CERVICAL; NERVE ROOT, VENTRAL, THORACIC; NERVE ROOT, VENTRAL, LUMBAR; NERVE ROOT, DORSAL, CERVICAL; NERVE ROOT, DORSAL, THORACIC

Histopathology - The following Tissues were Not Examined:

GLAND, PARATHYROID - Not Present In Section.

Individual Macroscopic and Microscopic Pathology:

5550008

Animal:	3510	Group:	3	Sex:	Female
Species:	Rat	Strain:	Sprague Dawley		
		Dose:	1.1x 10E12 vg		
		Removal Reason: Recovery Euthanasia			
		Study Day (Week) of Death: 91 (13)			

Gross Pathology Animal Details:

No animal details found

Gross Pathology Observations [Correlation]:

GENERAL OBSERVATIONS : (Comment) Optic nerve left submitted in cass C

Any remaining study plan required tissues, which have been examined, have no visible lesions

Gross Pathology - The following Tissues were Not Examined:

None

Histopathology Animal Details:

No animal details found

Histopathology Observations [Correlation]:

GANGLION, DORSAL ROOT, LUMBAR : Degeneration; axonal, minimal
GLAND, ADRENAL : (Note) Only Cortex And One Medulla Available For Evaluation.
LIVER : Vacuolation; hepatocellular, periportal, minimal
LIVER : Infiltration, mixed cell; minimal
LIVER : Infiltration, mononuclear cell; periportal, minimal
NERVE, SCIATIC : Degeneration; axonal, minimal
NERVE, TIBIAL : Degeneration; axonal, minimal
SITE, INJECTION : Infiltration, mononuclear cell; minimal, leptomeninges
SITE, INJECTION : Degeneration; axonal, minimal, cauda equina
SPINAL CORD, THORACIC : Infiltration, mononuclear cell; minimal, adipose tissue
NERVE ROOT, DORSAL, LUMBAR : Degeneration; axonal, minimal

Histopathology - The following Tissues were Within Normal Limits:

BRAIN; CERVIX; EYE; GANGLION, DORSAL ROOT, CERVICAL; GANGLION, DORSAL ROOT, THORACIC; GLAND, ADRENAL; GLAND, PARATHYROID; GLAND, PITUITARY; GLAND, THYROID; HEART; KIDNEY; LUNG; LYMPH NODE, CERVICAL; LYMPH NODE, ILIAC; LYMPH NODE, MANDIBULAR; LYMPH NODE, MESENTERIC; MUSCLE, BICEPS FEMORIS; MUSCLE, GASTROCNEMIUS; NERVE, OPTIC; OVARY; SPINAL CORD, CERVICAL; SPINAL CORD, LUMBAR; SPLEEN; THYMUS; UTERUS; NERVE ROOT, VENTRAL, CERVICAL; NERVE ROOT, VENTRAL, THORACIC; NERVE ROOT, VENTRAL, LUMBAR; NERVE ROOT, DORSAL, CERVICAL; NERVE ROOT, DORSAL, THORACIC

Histopathology - The following Tissues were Not Examined:

None

Appendix 18

Appendix 4

Individual Macroscopic and Microscopic Pathology:

5550008

Animal:	3511	Group:	3	Sex:	Female
Species:	Rat	Strain:	Sprague Dawley		
		Dose:	1.1x 10E12 vg		
		Removal Reason:	Terminal Euthanasia		
		Study Day (Week) of Death:	8 (2)		

Gross Pathology Animal Details:

No animal details found

Gross Pathology Observations [Correlation]:

No observations found

Any remaining study plan required tissues, which have been examined, have no visible lesions

Gross Pathology - The following Tissues were Not Examined:

None

Histopathology Animal Details:

No animal details found

Histopathology Observations [Correlation]:

GLAND, PARATHYROID : (Note) One Of A Pair Available For Evaluation.
GLAND, THYROID : (Note) One Of A Pair Available For Evaluation.
KIDNEY : Infiltration, mononuclear cell; minimal
KIDNEY : Chronic progressive nephropathy; minimal
LIVER : Infiltration, mixed cell; minimal
SITE, INJECTION : Infiltration, mononuclear cell; minimal, leptomeninges

Histopathology - The following Tissues were Within Normal Limits:

BRAIN; CERVIX; EYE; GANGLION, DORSAL ROOT, CERVICAL; GANGLION, DORSAL ROOT, LUMBAR; GANGLION, DORSAL ROOT, THORACIC; GLAND, ADRENAL; GLAND, PARATHYROID; GLAND, PITUITARY; GLAND, THYROID; HEART; LUNG; LYMPH NODE, CERVICAL; LYMPH NODE, ILIAC; LYMPH NODE, MANDIBULAR; LYMPH NODE, MESENTERIC; MUSCLE, BICEPS FEMORIS; MUSCLE, GASTROCNEMIUS; NERVE, OPTIC; NERVE, SCIATIC; NERVE, TIBIAL; OVARY; SPINAL CORD, CERVICAL; SPINAL CORD, LUMBAR; SPINAL CORD, THORACIC; SPLEEN; THYMUS; UTERUS; NERVE ROOT, VENTRAL, CERVICAL; NERVE ROOT, VENTRAL, THORACIC; NERVE ROOT, VENTRAL, LUMBAR; NERVE ROOT, DORSAL, CERVICAL; NERVE ROOT, DORSAL, LUMBAR; NERVE ROOT, DORSAL, THORACIC

Histopathology - The following Tissues were Not Examined:

None

Individual Macroscopic and Microscopic Pathology:

5550008

Animal: 3512	Group: 3	Sex: Female
Species: Rat	Strain: Sprague Dawley	
	Dose: 1.1x 10E12 vg	
	Removal Reason: Terminal Euthanasia	
	Study Day (Week) of Death: 8 (2)	

Gross Pathology Animal Details:

No animal details found

Gross Pathology Observations [Correlation]:

THYMUS : Focus, dark : (Comment) >10, left (TGL)

Any remaining study plan required tissues, which have been examined, have no visible lesions

Gross Pathology - The following Tissues were Not Examined:

None

Histopathology Animal Details:

No animal details found

Histopathology Observations [Correlation]:

HEART : Cyst; atrioventricular valve : (Comment) lymphocyst on atrial surface of right AV valve
NO CORRELATE : No correlating lesion [THYMUS : Focus, dark : (Comment) >10, left (G)]

Histopathology - The following Tissues were Within Normal Limits:

BRAIN; CERVIX; EYE; GANGLION, DORSAL ROOT, CERVICAL; GANGLION, DORSAL ROOT, LUMBAR; GANGLION, DORSAL ROOT, THORACIC; GLAND, ADRENAL; GLAND, PARATHYROID; GLAND, PITUITARY; GLAND, THYROID; KIDNEY; LIVER; LUNG; LYMPH NODE, CERVICAL; LYMPH NODE, ILIAC; LYMPH NODE, MANDIBULAR; LYMPH NODE, MESENTERIC; MUSCLE, BICEPS FEMORIS; MUSCLE, GASTROCNEMIUS; NERVE, OPTIC; NERVE, SCIATIC; NERVE, TIBIAL; OVARY; SITE, INJECTION; SPINAL CORD, CERVICAL; SPINAL CORD, LUMBAR; SPINAL CORD, THORACIC; SPLEEN; THYMUS; UTERUS; NERVE ROOT, VENTRAL, CERVICAL; NERVE ROOT, VENTRAL, THORACIC; NERVE ROOT, VENTRAL, LUMBAR; NERVE ROOT, DORSAL, CERVICAL; NERVE ROOT, DORSAL, LUMBAR; NERVE ROOT, DORSAL, THORACIC

Histopathology - The following Tissues were Not Examined:

None

Individual Macroscopic and Microscopic Pathology:

5550008

Animal: 3513	Group: 3	Sex: Female
Species: Rat	Strain: Sprague Dawley	
	Dose: 1.1x 10E12 vg	
	Removal Reason: Terminal Euthanasia	
	Study Day (Week) of Death: 8 (2)	

Gross Pathology Animal Details:

No animal details found

Gross Pathology Observations [Correlation]:

GENERAL OBSERVATIONS : (Comment) liver lesion submitted cass B
LIVER : Focus, pale : (Comment) 1, near hilus, right medial (TGL)

Any remaining study plan required tissues, which have been examined, have no visible lesions

Gross Pathology - The following Tissues were Not Examined:

None

Histopathology Animal Details:

No animal details found

Histopathology Observations [Correlation]:

GLAND, PARATHYROID : (Note) One Of A Pair Available For Evaluation.
SITE, INJECTION : Degeneration; axonal, minimal, cauda equina
NO CORRELATE : No correlating lesion [LIVER : Focus, pale : (Comment) 1, near hilus, right medial (G)]

Histopathology - The following Tissues were Within Normal Limits:

BRAIN; CERVIX; EYE; GANGLION, DORSAL ROOT, CERVICAL; GANGLION, DORSAL ROOT, LUMBAR; GANGLION, DORSAL ROOT, THORACIC; GLAND, ADRENAL; GLAND, PARATHYROID; GLAND, PITUITARY; GLAND, THYROID; HEART; KIDNEY; LIVER; LUNG; LYMPH NODE, CERVICAL; LYMPH NODE, ILIAC; LYMPH NODE, MANDIBULAR; LYMPH NODE, MESENTERIC; MUSCLE, BICEPS FEMORIS; MUSCLE, GASTROCNEMIUS; NERVE, OPTIC; NERVE, SCIATIC; NERVE, TIBIAL; OVARY; SPINAL CORD, CERVICAL; SPINAL CORD, LUMBAR; SPINAL CORD, THORACIC; SPLEEN; THYMUS; UTERUS; NERVE ROOT, VENTRAL, CERVICAL; NERVE ROOT, VENTRAL, THORACIC; NERVE ROOT, VENTRAL, LUMBAR; NERVE ROOT, DORSAL, CERVICAL; NERVE ROOT, DORSAL, LUMBAR; NERVE ROOT, DORSAL, THORACIC

Histopathology - The following Tissues were Not Examined:

None

Appendix 18

Appendix 4

Individual Macroscopic and Microscopic Pathology:

5550008

Animal:	3514	Group:	3	Sex:	Female
Species:	Rat	Strain:	Sprague Dawley		
		Dose:	1.1x 10E12 vg		
		Removal Reason: Recovery Euthanasia			
		Study Day (Week) of Death: 29 (5)			

Gross Pathology Animal Details:

No animal details found

Gross Pathology Observations [Correlation]:

GENERAL OBSERVATIONS : (Comment) lymph node popliteal lesion submitted cass E
LYMPH NODE : Enlargement : (Comment) popliteal right (TGL)

Any remaining study plan required tissues, which have been examined, have no visible lesions

Gross Pathology - The following Tissues were Not Examined:

None

Histopathology Animal Details:

No animal details found

Histopathology Observations [Correlation]:

GANGLION, DORSAL ROOT, LUMBAR : Infiltration, mononuclear cell; mild
GANGLION, DORSAL ROOT, LUMBAR : Degeneration; axonal, minimal
LIVER : Infiltration, mixed cell; minimal
NERVE, SCIATIC : Degeneration; axonal, minimal
NERVE, TIBIAL : Degeneration; axonal, minimal
SITE, INJECTION : Degeneration; axonal, minimal, cauda equina
NO CORRELATE : No correlating lesion [LYMPH NODE : Enlargement : (Comment) popliteal right (G)]
NERVE ROOT, DORSAL, LUMBAR : Degeneration; axonal, mild

Histopathology - The following Tissues were Within Normal Limits:

BRAIN; CERVIX; EYE; GANGLION, DORSAL ROOT, CERVICAL; GANGLION, DORSAL ROOT, THORACIC; GLAND, ADRENAL; GLAND, PARATHYROID; GLAND, PITUITARY; GLAND, THYROID; HEART; KIDNEY; LUNG; LYMPH NODE; LYMPH NODE, CERVICAL; LYMPH NODE, ILIAC; LYMPH NODE, MANDIBULAR; LYMPH NODE, MESENTERIC; MUSCLE, BICEPS FEMORIS; MUSCLE, GASTROCNEMIUS; NERVE, OPTIC; OVARY; SPINAL CORD, CERVICAL; SPINAL CORD, LUMBAR; SPINAL CORD, THORACIC; SPLEEN; THYMUS; UTERUS; NERVE ROOT, VENTRAL, CERVICAL; NERVE ROOT, VENTRAL, THORACIC; NERVE ROOT, VENTRAL, LUMBAR; NERVE ROOT, DORSAL, CERVICAL; NERVE ROOT, DORSAL, THORACIC

Histopathology - The following Tissues were Not Examined:

None

Individual Macroscopic and Microscopic Pathology:

5550008

Animal: 3615	Group: 3	Sex: Female
Species: Rat	Strain: Sprague Dawley<br>Dose: 1.1x 10E12 vg<br>Removal Reason: Recovery Euthanasia<br>Study Day (Week) of Death: 29 (5)	

Gross Pathology Animal Details:

No animal details found

Gross Pathology Observations [Correlation]:

GENERAL OBSERVATIONS : (Comment) Lymph node renal lesion submitted in cassette E; Lymph node lesion mediastinal submitted in cassette H; Oviduct left submitted with ovary left; Pituitary submitted in two pieces.
LYMPH NODE : Discoloration, mottled : (Comment) renal, left (TGL)
LYMPH NODE : Focus, dark : (Comment) 1, mediastinal (TGL)
LYMPH NODE : Enlargement : (Comment) renal, left (TGL)
OVARY : Cyst, pale : (Comment) 1, left (TGL)

Any remaining study plan required tissues, which have been examined, have no visible lesions

Gross Pathology - The following Tissues were Not Examined:

None

Histopathology Animal Details:

No animal details found

Histopathology Observations [Correlation]:

GANGLION, DORSAL ROOT, LUMBAR : Infiltration, mononuclear cell; minimal
GANGLION, DORSAL ROOT, LUMBAR : Degeneration; axonal, minimal
GANGLION, DORSAL ROOT, LUMBAR : Degeneration; neuronal, minimal
GLAND, PARATHYROID : (Note) One Of A Pair Available For Evaluation.
KIDNEY : Cast; hyaline, minimal
LIVER : Vacuolation; hepatocellular, periportal, minimal
LIVER : Infiltration, mixed cell; minimal
LYMPH NODE : Hemorrhage; acute, minimal
LYMPH NODE : Pigmented macrophage; mild [LYMPH NODE : Discoloration, mottled : (Comment) renal, left (G) | LYMPH NODE : Focus, dark : (Comment) 1, mediastinal (G)]
NERVE, SCIATIC : Degeneration; axonal, minimal
OVARY : Cyst [OVARY : Cyst, pale : (Comment) 1, left (G)]
SITE, INJECTION : Infiltration, mononuclear cell; minimal, leptomeninges
SITE, INJECTION : Degeneration; axonal, minimal, cauda equina
NO CORRELATE : No correlating lesion [LYMPH NODE : Enlargement : (Comment) renal, left (G)]
NERVE ROOT, DORSAL, LUMBAR : Degeneration; axonal, minimal

Histopathology - The following Tissues were Within Normal Limits:

Appendix 18

Appendix 4

Individual Macroscopic and Microscopic Pathology:

5550008

Animal: 3615 (Continued)	Group: 3	Sex: Female

BRAIN; CERVIX; EYE; GANGLION, DORSAL ROOT, CERVICAL; GANGLION, DORSAL ROOT, THORACIC; GLAND, ADRENAL; GLAND, PARATHYROID; GLAND, PITUITARY; GLAND, THYROID; HEART; LUNG; LYMPH NODE, CERVICAL; LYMPH NODE, ILIAC; LYMPH NODE, MANDIBULAR; LYMPH NODE, MESENTERIC; MUSCLE, BICEPS FEMORIS; MUSCLE, GASTROCNEMIUS; NERVE, OPTIC; NERVE, TIBIAL; SPINAL CORD, CERVICAL; SPINAL CORD, LUMBAR; SPINAL CORD, THORACIC; SPLEEN; THYMUS; UTERUS; NERVE ROOT, VENTRAL, CERVICAL; NERVE ROOT, VENTRAL, THORACIC; NERVE ROOT, VENTRAL, LUMBAR; NERVE ROOT, DORSAL, CERVICAL; NERVE ROOT, DORSAL, THORACIC

Histopathology - The following Tissues were Not Examined:

None

Appendix 18

Appendix 4

Individual Macroscopic and Microscopic Pathology:

5550008

Animal:	4001	Group:	4	Sex:	Male
Species:	Rat	Strain:	Sprague Dawley		
		Dose:	3.3x 10E12 vg		
		Removal Reason:	Terminal Euthanasia		
		Study Day (Week) of Death:	8 (2)		

Gross Pathology Animal Details:

No animal details found

Gross Pathology Observations [Correlation]:

GLAND, PITUITARY : Discoloration, dark (TGL)

Any remaining study plan required tissues, which have been examined, have no visible lesions

Gross Pathology - The following Tissues were Not Examined:

None

Histopathology Animal Details:

No animal details found

Histopathology Observations [Correlation]:

GANGLION, DORSAL ROOT, LUMBAR : Infiltration, mononuclear cell; minimal
LIVER : Tension lipidosis; minimal
LIVER : Infiltration, mixed cell; minimal
NERVE, TIBIAL : Degeneration; axonal, minimal
SITE, INJECTION : Infiltration, mononuclear cell; minimal, cauda equina
SITE, INJECTION : Infiltration, mononuclear cell; minimal, leptomeninges
NO CORRELATE : No correlating lesion [GLAND, PITUITARY : Discoloration, dark (G)]

Histopathology - The following Tissues were Within Normal Limits:

BRAIN; EPIDIDYMIS; EYE; GANGLION, DORSAL ROOT, CERVICAL; GANGLION, DORSAL ROOT, THORACIC; GLAND, ADRENAL; GLAND, PARATHYROID; GLAND, PITUITARY; GLAND, PROSTATE; GLAND, THYROID; HEART; KIDNEY; LUNG; LYMPH NODE, CERVICAL; LYMPH NODE, ILIAC; LYMPH NODE, MANDIBULAR; LYMPH NODE, MESENTERIC; MUSCLE, BICEPS FEMORIS; MUSCLE, GASTROCNEMIUS; NERVE, SCIATIC; SPINAL CORD, CERVICAL; SPINAL CORD, LUMBAR; SPINAL CORD, THORACIC; SPLEEN; TESTIS; THYMUS; NERVE ROOT, VENTRAL, CERVICAL; NERVE ROOT, VENTRAL, THORACIC; NERVE ROOT, VENTRAL, LUMBAR; NERVE ROOT, DORSAL, CERVICAL; NERVE ROOT, DORSAL, LUMBAR; NERVE ROOT, DORSAL, THORACIC

Histopathology - The following Tissues were Not Examined:

NERVE, OPTIC - Not Present In Section.

Individual Macroscopic and Microscopic Pathology:

5550008

Animal:	4002	Group:	4	Sex:	Male
Species:	Rat	Strain:	Sprague Dawley		
		Dose:	3.3x 10E12 vg		
		Removal Reason: Terminal Euthanasia			
		Study Day (Week) of Death: 8 (2)			

Gross Pathology Animal Details:

No animal details found

Gross Pathology Observations [Correlation]:

No observations found

Any remaining study plan required tissues, which have been examined, have no visible lesions

Gross Pathology - The following Tissues were Not Examined:

None

Histopathology Animal Details:

No animal details found

Histopathology Observations [Correlation]:

GANGLION, DORSAL ROOT, LUMBAR : Infiltration, mononuclear cell; minimal
GLAND, PARATHYROID : (Note) One Of A Pair Available For Evaluation.
LIVER : Vacuolation; hepatocellular, periportal, minimal

Histopathology - The following Tissues were Within Normal Limits:

BRAIN; EPIDIDYMIS; EYE; GANGLION, DORSAL ROOT, CERVICAL; GANGLION, DORSAL ROOT, THORACIC; GLAND, ADRENAL; GLAND, PARATHYROID; GLAND, PITUITARY; GLAND, PROSTATE; GLAND, THYROID; HEART; KIDNEY; LUNG; LYMPH NODE, CERVICAL; LYMPH NODE, ILIAC; LYMPH NODE, MANDIBULAR; LYMPH NODE, MESENTERIC; MUSCLE, BICEPS FEMORIS; MUSCLE, GASTROCNEMIUS; NERVE, OPTIC; NERVE, SCIATIC; NERVE, TIBIAL; SITE, INJECTION; SPINAL CORD, CERVICAL; SPINAL CORD, LUMBAR; SPINAL CORD, THORACIC; SPLEEN; TESTIS; THYMUS; NERVE ROOT, VENTRAL, CERVICAL; NERVE ROOT, VENTRAL, THORACIC; NERVE ROOT, VENTRAL, LUMBAR; NERVE ROOT, DORSAL, CERVICAL; NERVE ROOT, DORSAL, LUMBAR; NERVE ROOT, DORSAL, THORACIC

Histopathology - The following Tissues were Not Examined:

None

Individual Macroscopic and Microscopic Pathology:

5550008

Animal: 4003	Group: 4	Sex: Male
Species: Rat	Strain: Sprague Dawley	
	Dose: 3.3x 10E12 vg	
	Removal Reason: Recovery Euthanasia	
	Study Day (Week) of Death: 29 (5)	

Gross Pathology Animal Details:

No animal details found

Gross Pathology Observations [Correlation]:

No observations found

Any remaining study plan required tissues, which have been examined, have no visible lesions

Gross Pathology - The following Tissues were Not Examined:

None

Histopathology Animal Details:

No animal details found

Histopathology Observations [Correlation]:

EPIDIDYMIS : Infiltration, mononuclear cell; minimal
GANGLION, DORSAL ROOT, LUMBAR : Infiltration, mononuclear cell; minimal
GANGLION, DORSAL ROOT, LUMBAR : Degeneration; axonal, mild
GANGLION, DORSAL ROOT, LUMBAR : Degeneration; neuronal, minimal
GLAND, PARATHYROID : (Note) One Of A Pair Available For Evaluation.
GLAND, PROSTATE : Infiltration, mononuclear cell; minimal
KIDNEY : Infiltration, mononuclear cell; minimal
LIVER : Infiltration, mixed cell; minimal
LUNG : Pigmented macrophage; focal, minimal
NERVE, SCIATIC : Degeneration; axonal, minimal
NERVE, TIBIAL : Degeneration; axonal, minimal
SITE, INJECTION : Infiltration, mononuclear cell; mild, leptomeninges
SITE, INJECTION : Degeneration; axonal, mild, cauda equina
NERVE ROOT, DORSAL, LUMBAR : Degeneration; axonal, mild

Histopathology - The following Tissues were Within Normal Limits:

BRAIN; EYE; GANGLION, DORSAL ROOT, CERVICAL; GANGLION, DORSAL ROOT, THORACIC; GLAND, ADRENAL; GLAND, PARATHYROID; GLAND, PITUITARY; GLAND, THYROID; HEART; LYMPH NODE, CERVICAL; LYMPH NODE, ILIAC; LYMPH NODE, MANDIBULAR; LYMPH NODE, MESENTERIC; MUSCLE, BICEPS FEMORIS; MUSCLE, GASTROCNEMIUS; NERVE, OPTIC; SPINAL CORD, CERVICAL; SPINAL CORD, LUMBAR; SPINAL CORD, THORACIC; SPLEEN; TESTIS; THYMUS; NERVE ROOT, VENTRAL, CERVICAL; NERVE ROOT, VENTRAL, THORACIC; NERVE ROOT, VENTRAL, LUMBAR; NERVE ROOT, DORSAL, CERVICAL; NERVE ROOT, DORSAL, THORACIC

Histopathology - The following Tissues were Not Examined:

None

Individual Macroscopic and Microscopic Pathology:

5550008

Animal: 4004	Group: 4	Sex: Male
Species: Rat	Strain: Sprague Dawley<br>Dose: 3.3x 10E12 vg<br>Removal Reason: Recovery Euthanasia<br>Study Day (Week) of Death: 29 (5)	

Gross Pathology Animal Details:

No animal details found

Gross Pathology Observations [Correlation]:

GENERAL OBSERVATIONS : (Comment) Optic nerve left submitted in cassette B
GLAND, THYROID : Enlargement : (Comment) side(s) not recorded at necropsy (TGL)
THYMUS : Focus, dark : (Comment) 1, left (TGL)

Any remaining study plan required tissues, which have been examined, have no visible lesions

Gross Pathology - The following Tissues were Not Examined:

None

Histopathology Animal Details:

No animal details found

Histopathology Observations [Correlation]:

EPIDIDYMIS : Infiltration, mononuclear cell; minimal
GANGLION, DORSAL ROOT, LUMBAR : Infiltration, mononuclear cell; mild
GANGLION, DORSAL ROOT, LUMBAR : Degeneration; axonal, mild
GANGLION, DORSAL ROOT, LUMBAR : Degeneration; neuronal, mild
GLAND, ADRENAL : Vacuolation; cortical, minimal
GLAND, ADRENAL : Infiltration, mononuclear cell; minimal
GLAND, PARATHYROID : (Note) One Of A Pair Available For Evaluation.
GLAND, PITUITARY : (Note) Only Pars Distalis Available For Evaluation.
KIDNEY : Infiltration, mononuclear cell; minimal
KIDNEY : Chronic progressive nephropathy; minimal
LIVER : Infiltration, mixed cell; minimal
NERVE, SCIATIC : Degeneration; axonal, minimal
SITE, INJECTION : Infiltration, mononuclear cell; minimal, leptomeninges
SITE, INJECTION : Degeneration; axonal, minimal, cauda equina
NO CORRELATE : No correlating lesion [GLAND, THYROID : Enlargement : (Comment) side(s) not recorded at necropsy (G) | THYMUS : Focus, dark : (Comment) 1, left (G)]
NERVE ROOT, DORSAL, LUMBAR : Degeneration; axonal, mild

Histopathology - The following Tissues were Within Normal Limits:

BRAIN; EYE; GANGLION, DORSAL ROOT, CERVICAL; GANGLION, DORSAL ROOT, THORACIC; GLAND, PARATHYROID; GLAND, PITUITARY; GLAND, PROSTATE; GLAND, THYROID; HEART; LUNG; LYMPH NODE, CERVICAL; LYMPH NODE, ILIAC; LYMPH NODE, MANDIBULAR; LYMPH NODE, MESENTERIC; MUSCLE, BICEPS FEMORIS; MUSCLE, GASTROCNEMIUS; NERVE, OPTIC; NERVE, TIBIAL; SPINAL CORD, CERVICAL; SPINAL CORD, LUMBAR; SPINAL CORD, THORACIC; SPLEEN; TESTIS; THYMUS; NERVE ROOT, VENTRAL, CERVICAL; NERVE ROOT, VENTRAL, THORACIC; NERVE ROOT, VENTRAL, LUMBAR; NERVE ROOT, DORSAL, CERVICAL; NERVE ROOT, DORSAL, THORACIC

Appendix 18

Appendix 4

Individual Macroscopic and Microscopic Pathology:

5550008

Animal: 4004 (Continued)	Group: 4	Sex: Male

Histopathology - The following Tissues were Not Examined:

None

Individual Macroscopic and Microscopic Pathology:

5550008

Animal:	4005	Group:	4	Sex:	Male
Species:	Rat	Strain:	Sprague Dawley		
		Dose:	3.3x 10E12 vg		
		Removal Reason: Recovery Euthanasia			
		Study Day (Week) of Death: 29 (5)			

Gross Pathology Animal Details:

No animal details found

Gross Pathology Observations [Correlation]:

GENERAL OBSERVATIONS : (Comment) Lymph node renal submitted cass E
LYMPH NODE : Discoloration, dark : (Comment) renal, left (TGL)

Any remaining study plan required tissues, which have been examined, have no visible lesions

Gross Pathology - The following Tissues were Not Examined:

None

Histopathology Animal Details:

No animal details found

Histopathology Observations [Correlation]:

GLAND, ADRENAL : Vacuolation; cortical, minimal
KIDNEY : Infiltration, mononuclear cell; minimal
KIDNEY : Chronic progressive nephropathy; minimal
LIVER : Infiltration, mixed cell; minimal
LYMPH NODE : Pigmented macrophage; mild [LYMPH NODE : Discoloration, dark : (Comment) renal, left (G)]
NERVE, SCIATIC : Degeneration; axonal, minimal
NERVE, TIBIAL : Degeneration; axonal, minimal

Histopathology - The following Tissues were Within Normal Limits:

BRAIN; EPIDIDYMIS; EYE; GANGLION, DORSAL ROOT, CERVICAL; GANGLION, DORSAL ROOT, LUMBAR; GANGLION, DORSAL ROOT, THORACIC; GLAND, PARATHYROID; GLAND, PITUITARY; GLAND, PROSTATE; GLAND, THYROID; HEART; LUNG; LYMPH NODE, CERVICAL; LYMPH NODE, ILIAC; LYMPH NODE, MANDIBULAR; LYMPH NODE, MESENTERIC; MUSCLE, BICEPS FEMORIS; MUSCLE, GASTROCNEMIUS; NERVE, OPTIC; SITE, INJECTION; SPINAL CORD, CERVICAL; SPINAL CORD, LUMBAR; SPINAL CORD, THORACIC; SPLEEN; TESTIS; THYMUS; NERVE ROOT, VENTRAL, CERVICAL; NERVE ROOT, VENTRAL, THORACIC; NERVE ROOT, VENTRAL, LUMBAR; NERVE ROOT, DORSAL, CERVICAL; NERVE ROOT, DORSAL, LUMBAR; NERVE ROOT, DORSAL, THORACIC

Histopathology - The following Tissues were Not Examined:

None

Individual Macroscopic and Microscopic Pathology:

5550008

Animal: 4006	Group: 4	Sex: Male
Species: Rat	Strain: Sprague Dawley<br>Dose: 3.3x 10E12 vg<br>Removal Reason: Terminal Euthanasia<br>Study Day (Week) of Death: 8 (2)	

Gross Pathology Animal Details:

No animal details found

Gross Pathology Observations [Correlation]:

GENERAL OBSERVATIONS : (Comment) Right lymph node deep cervical not submitted for histopathological evaluation (frozen).
LYMPH NODE, CERVICAL : Discoloration, dark : (Comment) bilateral. (TGL)
LYMPH NODE, MANDIBULAR : Discoloration, mottled : (Comment) bilateral. (TGL)
THYMUS : Focus, dark : (Comment) >10. (TGL)

Any remaining study plan required tissues, which have been examined, have no visible lesions

Gross Pathology - The following Tissues were Not Examined:

None

Histopathology Animal Details:

No animal details found

Histopathology Observations [Correlation]:

EPIDIDYMIS : Peripuberty
GANGLION, DORSAL ROOT, LUMBAR : Infiltration, mononuclear cell; minimal
LYMPH NODE, CERVICAL : Hemorrhage; acute, mild [LYMPH NODE, CERVICAL : Discoloration, dark : (Comment) bilateral. (G)]
NERVE, SCIATIC : Degeneration; axonal, minimal
NERVE, TIBIAL : Degeneration; axonal, minimal
SITE, INJECTION : Degeneration; axonal, minimal, cauda equina
TESTIS : Peripuberty
NO CORRELATE : No correlating lesion [LYMPH NODE, MANDIBULAR : Discoloration, mottled : (Comment) bilateral. (G) | THYMUS : Focus, dark : (Comment) >10. (G)]
NERVE ROOT, DORSAL, LUMBAR : Degeneration; axonal, minimal

Histopathology - The following Tissues were Within Normal Limits:

BRAIN; EYE; GANGLION, DORSAL ROOT, CERVICAL; GANGLION, DORSAL ROOT, THORACIC; GLAND, ADRENAL; GLAND, PARATHYROID; GLAND, PITUITARY; GLAND, PROSTATE; GLAND, THYROID; HEART; KIDNEY; LIVER; LUNG; LYMPH NODE, ILIAC; LYMPH NODE, MANDIBULAR; LYMPH NODE, MESENTERIC; MUSCLE, BICEPS FEMORIS; MUSCLE, GASTROCNEMIUS; NERVE, OPTIC; SPINAL CORD, CERVICAL; SPINAL CORD, LUMBAR; SPINAL CORD, THORACIC; SPLEEN; THYMUS; NERVE ROOT, VENTRAL, CERVICAL; NERVE ROOT, VENTRAL, THORACIC; NERVE ROOT, VENTRAL, LUMBAR; NERVE ROOT, DORSAL, CERVICAL; NERVE ROOT, DORSAL, THORACIC

Histopathology - The following Tissues were Not Examined:

None

Appendix 18

Appendix 4

Individual Macroscopic and Microscopic Pathology:

5550008

Animal:	4007	Group:	4	Sex:	Male
Species:	Rat	Strain:	Sprague Dawley		
		Dose:	3.3x 10E12 vg		
		Removal Reason: Terminal Euthanasia			
		Study Day (Week) of Death: 8 (2)			

Gross Pathology Animal Details:

No animal details found

Gross Pathology Observations [Correlation]:

HEART : Focus, depressed : (Comment) dark, 1, ventricle right, epicardium. (TGL)
LUNG : Discoloration, dark : (Comment) right accessory, right caudal. (TGL)
THYMUS : Focus, dark : (Comment) >10. (TGL)

Any remaining study plan required tissues, which have been examined, have no visible lesions

Gross Pathology - The following Tissues were Not Examined:

None

Histopathology Animal Details:

No animal details found

Histopathology Observations [Correlation]:

EPIDIDYMIS : Peripuberty
GANGLION, DORSAL ROOT, LUMBAR : Infiltration, mononuclear cell; minimal
GLAND, PARATHYROID : (Note) One Of A Pair Available For Evaluation.
GLAND, PITUITARY : (Note) Only Pars Distalis Available For Evaluation.
LIVER : Infiltration, mononuclear cell; minimal
LUNG : Hemorrhage; chronic, mild [LUNG : Discoloration, dark : (Comment) right accessory, right caudal. (G)]
TESTIS : Peripuberty
NO CORRELATE : No correlating lesion [THYMUS : Focus, dark : (Comment) >10. (G) | HEART : Focus, depressed : (Comment) dark, 1, ventricle right, epicardium. (G)]

Histopathology - The following Tissues were Within Normal Limits:

BRAIN; EYE; GANGLION, DORSAL ROOT, CERVICAL; GANGLION, DORSAL ROOT, THORACIC; GLAND, ADRENAL; GLAND, PARATHYROID; GLAND, PITUITARY; GLAND, PROSTATE; GLAND, THYROID; HEART; KIDNEY; LYMPH NODE, CERVICAL; LYMPH NODE, ILIAC; LYMPH NODE, MANDIBULAR; LYMPH NODE, MESENTERIC; MUSCLE, BICEPS FEMORIS; MUSCLE, GASTROCNEMIUS; NERVE, SCIATIC; NERVE, TIBIAL; SITE, INJECTION; SPINAL CORD, CERVICAL; SPINAL CORD, LUMBAR; SPINAL CORD, THORACIC; SPLEEN; THYMUS; NERVE ROOT, VENTRAL, CERVICAL; NERVE ROOT, VENTRAL, THORACIC; NERVE ROOT, VENTRAL, LUMBAR; NERVE ROOT, DORSAL, CERVICAL; NERVE ROOT, DORSAL, LUMBAR; NERVE ROOT, DORSAL, THORACIC

Histopathology - The following Tissues were Not Examined:

NERVE, OPTIC - Not Present In Section.

Appendix 18

Appendix 4

Individual Macroscopic and Microscopic Pathology:

5550008

Animal: 4008	Group: 4	Sex: Male
Species: Rat	Strain: Sprague Dawley<br>Dose: 3.3x 10E12 vg<br>Removal Reason: Terminal Euthanasia<br>Study Day (Week) of Death: 8 (2)	

Gross Pathology Animal Details:

No animal details found

Gross Pathology Observations [Correlation]:

GLAND, PITUITARY : Discoloration, dark (TGL)
THYMUS : Focus, dark : (Comment) 1, left (TGL)

Any remaining study plan required tissues, which have been examined, have no visible lesions

Gross Pathology - The following Tissues were Not Examined:

None

Histopathology Animal Details:

No animal details found

Histopathology Observations [Correlation]:

EPIDIDYMIS : Peripuberty
GANGLION, DORSAL ROOT, LUMBAR : Infiltration, mononuclear cell; minimal
GLAND, PARATHYROID : (Note) One Of A Pair Available For Evaluation.
GLAND, PITUITARY : (Note) Only Pars Distalis Available For Evaluation.
GLAND, PITUITARY : Congestion; moderate [GLAND, PITUITARY : Discoloration, dark (G)]
LIVER : Vacuolation; hepatocellular, periportal, minimal
LIVER : Infiltration, mononuclear cell; periportal, minimal
TESTIS : Peripuberty
NO CORRELATE : No correlating lesion [THYMUS : Focus, dark : (Comment) 1, left (G)]

Histopathology - The following Tissues were Within Normal Limits:

BRAIN; EYE; GANGLION, DORSAL ROOT, CERVICAL; GANGLION, DORSAL ROOT, THORACIC; GLAND, ADRENAL; GLAND, PARATHYROID; GLAND, PROSTATE; GLAND, THYROID; HEART; KIDNEY; LUNG; LYMPH NODE, CERVICAL; LYMPH NODE, ILIAC; LYMPH NODE, MANDIBULAR; LYMPH NODE, MESENTERIC; MUSCLE, BICEPS FEMORIS; MUSCLE, GASTROCNEMIUS; NERVE, OPTIC; NERVE, SCIATIC; NERVE, TIBIAL; SITE, INJECTION; SPINAL CORD, CERVICAL; SPINAL CORD, LUMBAR; SPINAL CORD, THORACIC; SPLEEN; THYMUS; NERVE ROOT, VENTRAL, CERVICAL; NERVE ROOT, VENTRAL, THORACIC; NERVE ROOT, VENTRAL, LUMBAR; NERVE ROOT, DORSAL, CERVICAL; NERVE ROOT, DORSAL, LUMBAR; NERVE ROOT, DORSAL, THORACIC

Histopathology - The following Tissues were Not Examined:

None

Appendix 18

Appendix 4

Individual Macroscopic and Microscopic Pathology:

5550008

Animal:	4009	Group:	4	Sex:	Male
Species:	Rat	Strain:	Sprague Dawley		
		Dose:	3.3x 10E12 vg		
		Removal Reason: Recovery Euthanasia			
		Study Day (Week) of Death: 91 (13)			

Gross Pathology Animal Details:

No animal details found

Gross Pathology Observations [Correlation]:

GENERAL OBSERVATIONS : (Comment) Pituitary submitted in 2 pieces.
LIVER : Focus, pale : (Comment) 1, fissure, right medial. (TGL)

Any remaining study plan required tissues, which have been examined, have no visible lesions

Gross Pathology - The following Tissues were Not Examined:

None

Histopathology Animal Details:

No animal details found

Histopathology Observations [Correlation]:

EPIDIDYMIS : Peripuberty
GLAND, ADRENAL : Vacuolation; cortical, minimal
KIDNEY : Infiltration, mononuclear cell; minimal
LIVER : Vacuolation; hepatocellular, periportal, minimal
LIVER : Tension lipidosis; mild [LIVER : Focus, pale : (Comment) 1, fissure, right medial. (G)]
LIVER : Infiltration, mononuclear cell; minimal
NERVE, SCIATIC : Degeneration; axonal, minimal
NERVE, TIBIAL : Degeneration; axonal, minimal
SITE, INJECTION : Degeneration; axonal, minimal, cauda equina
TESTIS : Vacuolation; minimal, sertoli cell
TESTIS : Peripuberty
NERVE ROOT, DORSAL, LUMBAR : Degeneration; axonal, minimal

Histopathology - The following Tissues were Within Normal Limits:

BRAIN; EYE; GANGLION, DORSAL ROOT, CERVICAL; GANGLION, DORSAL ROOT, LUMBAR; GANGLION, DORSAL ROOT, THORACIC; GLAND, PARATHYROID; GLAND, PITUITARY; GLAND, PROSTATE; GLAND, THYROID; HEART; LUNG; LYMPH NODE, CERVICAL; LYMPH NODE, ILIAC; LYMPH NODE, MANDIBULAR; LYMPH NODE, MESENTERIC; MUSCLE, BICEPS FEMORIS; MUSCLE, GASTROCNEMIUS; SPINAL CORD, CERVICAL; SPINAL CORD, LUMBAR; SPINAL CORD, THORACIC; SPLEEN; THYMUS; NERVE ROOT, VENTRAL, CERVICAL; NERVE ROOT, VENTRAL, THORACIC; NERVE ROOT, VENTRAL, LUMBAR; NERVE ROOT, DORSAL, CERVICAL; NERVE ROOT, DORSAL, THORACIC

Histopathology - The following Tissues were Not Examined:

NERVE, OPTIC - Not Present In Section.

Appendix 18
Appendix 4

Individual Macroscopic and Microscopic Pathology:

5550008

Animal: 4010	Group: 4	Sex: Male
Species: Rat	Strain: Sprague Dawley<br>Dose: 3.3x 10E12 vg<br>Removal Reason: Recovery Euthanasia<br>Study Day (Week) of Death: 91 (13)	

Gross Pathology Animal Details:

No animal details found

Gross Pathology Observations [Correlation]:

GENERAL OBSERVATIONS : (Comment) Liver lesion submitted in cass C; Brain submitted in two pieces
LIVER : Focus, pale : (Comment) 1, near hilus, right lateral (TGL)
STOMACH : Focus, depressed : (Comment) dark,1, mucosa, glandular (TGL)

Any remaining study plan required tissues, which have been examined, have no visible lesions

Gross Pathology - The following Tissues were Not Examined:

None

Histopathology Animal Details:

No animal details found

Histopathology Observations [Correlation]:

GANGLION, DORSAL ROOT, LUMBAR : Infiltration, mononuclear cell; minimal
GANGLION, DORSAL ROOT, LUMBAR : Degeneration; axonal, minimal
GANGLION, DORSAL ROOT, LUMBAR : Degeneration; neuronal, minimal
GLAND, PARATHYROID : (Note) One Of A Pair Available For Evaluation.
LIVER : Infiltration, mononuclear cell; periportal, minimal
NERVE, SCIATIC : Degeneration; axonal, minimal
NERVE, TIBIAL : Degeneration; axonal, minimal
SITE, INJECTION : Degeneration; axonal, minimal, cauda equina
NO CORRELATE : No correlating lesion [STOMACH : Focus, depressed : (Comment) dark,1, mucosa, glandular (G) | LIVER : Focus, pale : (Comment) 1, near hilus, right lateral (G)]
NERVE ROOT, DORSAL, LUMBAR : Degeneration; axonal, mild

Histopathology - The following Tissues were Within Normal Limits:

BRAIN; EPIDIDYMIS; EYE; GANGLION, DORSAL ROOT, CERVICAL; GANGLION, DORSAL ROOT, THORACIC; GLAND, ADRENAL; GLAND, PARATHYROID; GLAND, PITUITARY; GLAND, PROSTATE; GLAND, THYROID; HEART; KIDNEY; LUNG; LYMPH NODE, CERVICAL; LYMPH NODE, ILIAC; LYMPH NODE, MANDIBULAR; LYMPH NODE, MESENTERIC; MUSCLE, BICEPS FEMORIS; MUSCLE, GASTROCNEMIUS; NERVE, OPTIC; SPINAL CORD, CERVICAL; SPINAL CORD, LUMBAR; SPINAL CORD, THORACIC; SPLEEN; TESTIS; THYMUS; NERVE ROOT, VENTRAL, CERVICAL; NERVE ROOT, VENTRAL, THORACIC; NERVE ROOT, VENTRAL, LUMBAR; NERVE ROOT, DORSAL, CERVICAL; NERVE ROOT, DORSAL, THORACIC

Histopathology - The following Tissues were Not Examined:

None

Appendix 18

Appendix 4

Individual Macroscopic and Microscopic Pathology:

5550008

Animal:	4011	Group:	4	Sex:	Male
Species:	Rat	Strain:	Sprague Dawley		
		Dose:	3.3x 10E12 vg		
		Removal Reason: Recovery Euthanasia			
		Study Day (Week) of Death: 91 (13)			

Gross Pathology Animal Details:

No animal details found

Gross Pathology Observations [Correlation]:

No observations found

Any remaining study plan required tissues, which have been examined, have no visible lesions

Gross Pathology - The following Tissues were Not Examined:

None

Histopathology Animal Details:

No animal details found

Histopathology Observations [Correlation]:

GANGLION, DORSAL ROOT, LUMBAR : Infiltration, mononuclear cell; minimal
GANGLION, DORSAL ROOT, LUMBAR : Degeneration; neuronal, minimal
KIDNEY : Infiltration, mononuclear cell; minimal
KIDNEY : Chronic progressive nephropathy; minimal
LIVER : Infiltration, mixed cell; minimal
NERVE, SCIATIC : Degeneration; axonal, minimal
NERVE, TIBIAL : Degeneration; axonal, minimal
SITE, INJECTION : Degeneration; axonal, minimal, cauda equina
NERVE ROOT, DORSAL, LUMBAR : Degeneration; axonal, mild

Histopathology - The following Tissues were Within Normal Limits:

BRAIN; EPIDIDYMIS; EYE; GANGLION, DORSAL ROOT, CERVICAL; GANGLION, DORSAL ROOT, THORACIC; GLAND, ADRENAL; GLAND, PARATHYROID; GLAND, PITUITARY; GLAND, PROSTATE; GLAND, THYROID; HEART; LUNG; LYMPH NODE, CERVICAL; LYMPH NODE, ILIAC; LYMPH NODE, MANDIBULAR; LYMPH NODE, MESENTERIC; MUSCLE, BICEPS FEMORIS; MUSCLE, GASTROCNEMIUS; NERVE, OPTIC; SPINAL CORD, CERVICAL; SPINAL CORD, LUMBAR; SPINAL CORD, THORACIC; SPLEEN; TESTIS; THYMUS; NERVE ROOT, VENTRAL, CERVICAL; NERVE ROOT, VENTRAL, THORACIC; NERVE ROOT, VENTRAL, LUMBAR; NERVE ROOT, DORSAL, CERVICAL; NERVE ROOT, DORSAL, THORACIC

Histopathology - The following Tissues were Not Examined:

None

Appendix 18

Appendix 4

Individual Macroscopic and Microscopic Pathology:

5550008

Animal: 4012	Group: 4	Sex: Male
Species: Rat	Strain: Sprague Dawley	
	Dose: 3.3x 10E12 vg	
	Removal Reason: Recovery Euthanasia	
	Study Day (Week) of Death: 29 (5)	

Gross Pathology Animal Details:

No animal details found

Gross Pathology Observations [Correlation]:

GENERAL OBSERVATIONS : (Comment) liver lesion submitted cass B
LIVER : Focus, pale : (Comment) 2, near hilus, right lateral (TGL)

Any remaining study plan required tissues, which have been examined, have no visible lesions

Gross Pathology - The following Tissues were Not Examined:

None

Histopathology Animal Details:

No animal details found

Histopathology Observations [Correlation]:

EPIDIDYMIS : Cellular debris; bilateral, minimal
GANGLION, DORSAL ROOT, LUMBAR : Infiltration, mononuclear cell; minimal
GANGLION, DORSAL ROOT, LUMBAR : Degeneration; axonal, mild
GLAND, PARATHYROID : (Note) One Of A Pair Available For Evaluation.
KIDNEY : Infiltration, mononuclear cell; minimal
KIDNEY : Chronic progressive nephropathy; minimal
LIVER : Tension lipidosis; minimal [LIVER : Focus, pale : (Comment) 2, near hilus, right lateral (G)]
LIVER : Infiltration, mixed cell; minimal
LIVER : Extramedullary hematopoiesis; minimal
NERVE, SCIATIC : Degeneration; axonal, mild
NERVE, TIBIAL : Degeneration; axonal, mild
SITE, INJECTION : Infiltration, mononuclear cell; minimal, leptomeninges
SITE, INJECTION : Degeneration; axonal, mild, cauda equina
SPINAL CORD, THORACIC : Infiltration, mononuclear cell; mild, adipose tissue
NERVE ROOT, DORSAL, LUMBAR : Degeneration; axonal, mild

Histopathology - The following Tissues were Within Normal Limits:

BRAIN; EYE; GANGLION, DORSAL ROOT, CERVICAL; GANGLION, DORSAL ROOT, THORACIC; GLAND, ADRENAL; GLAND, PARATHYROID; GLAND, PITUITARY; GLAND, PROSTATE; GLAND, THYROID; HEART; LUNG; LYMPH NODE, CERVICAL; LYMPH NODE, ILIAC; LYMPH NODE, MANDIBULAR; LYMPH NODE, MESENTERIC; MUSCLE, BICEPS FEMORIS; MUSCLE, GASTROCNEMIUS; NERVE, OPTIC; SPINAL CORD, CERVICAL; SPINAL CORD, LUMBAR; SPLEEN; TESTIS; THYMUS; NERVE ROOT, VENTRAL, CERVICAL; NERVE ROOT, VENTRAL, THORACIC; NERVE ROOT, VENTRAL, LUMBAR; NERVE ROOT, DORSAL, CERVICAL; NERVE ROOT, DORSAL, THORACIC

Histopathology - The following Tissues were Not Examined:

None

Individual Macroscopic and Microscopic Pathology:

5550008

Animal: 4013	Group: 4	Sex: Male
Species: Rat	Strain: Sprague Dawley	
	Dose: 3.3x 10E12 vg	
	Removal Reason: Recovery Euthanasia	
	Study Day (Week) of Death: 29 (5)	

Gross Pathology Animal Details:

No animal details found

Gross Pathology Observations [Correlation]:

GENERAL OBSERVATIONS : (Comment) Pituitary submitted in two pieces.
THYMUS : Focus, dark : (Comment) >10 (TGL)

Any remaining study plan required tissues, which have been examined, have no visible lesions

Gross Pathology - The following Tissues were Not Examined:

None

Histopathology Animal Details:

No animal details found

Histopathology Observations [Correlation]:

GANGLION, DORSAL ROOT, LUMBAR : Infiltration, mononuclear cell; minimal
GANGLION, DORSAL ROOT, LUMBAR : Degeneration; axonal, minimal
GANGLION, DORSAL ROOT, LUMBAR : Degeneration; neuronal, minimal
GLAND, ADRENAL : Vacuolation; cortical, minimal
GLAND, PITUITARY : (Note) Only Pars Distalis Available For Evaluation.
HEART : Fibrosis; focal, minimal
KIDNEY : Infiltration, mononuclear cell; minimal
KIDNEY : Chronic progressive nephropathy; minimal
LIVER : Vacuolation; hepatocellular, periportal, minimal
LIVER : Infiltration, mixed cell; minimal
LUNG : Hemorrhage; chronic, minimal
NERVE, SCIATIC : Degeneration; axonal, minimal
NERVE, TIBIAL : Degeneration; axonal, minimal
SITE, INJECTION : Infiltration, mononuclear cell; minimal, leptomeninges
SITE, INJECTION : Degeneration; axonal, minimal, cauda equina
NO CORRELATE : No correlating lesion [THYMUS : Focus, dark : (Comment) >10 (G)]
NERVE ROOT, DORSAL, LUMBAR : Degeneration; axonal, minimal

Histopathology - The following Tissues were Within Normal Limits:

BRAIN; EPIDIDYMIS; EYE; GANGLION, DORSAL ROOT, CERVICAL; GANGLION, DORSAL ROOT, THORACIC; GLAND, PARATHYROID; GLAND, PITUITARY; GLAND, PROSTATE; GLAND, THYROID; LYMPH NODE, CERVICAL; LYMPH NODE, ILIAC; LYMPH NODE, MANDIBULAR; LYMPH NODE, MESENTERIC; MUSCLE, BICEPS FEMORIS; MUSCLE, GASTROCNEMIUS; SPINAL CORD, CERVICAL; SPINAL CORD, LUMBAR; SPINAL CORD, THORACIC; SPLEEN; TESTIS; THYMUS; NERVE ROOT, VENTRAL, CERVICAL; NERVE ROOT, VENTRAL, THORACIC; NERVE ROOT, VENTRAL, LUMBAR; NERVE ROOT, DORSAL, CERVICAL; NERVE ROOT, DORSAL, THORACIC

Individual Macroscopic and Microscopic Pathology:

5550008

Animal: 4013 (Continued)	Group: 4	Sex: Male

Histopathology - The following Tissues were Not Examined:

NERVE, OPTIC - Not Present In Section.

Appendix 18

Appendix 4

Individual Macroscopic and Microscopic Pathology:

5550008

Animal: 4014	Group: 4	Sex: Male
Species: Rat	Strain: Sprague Dawley<br>Dose: 3.3x 10E12 vg<br>Removal Reason: Recovery Euthanasia<br>Study Day (Week) of Death: 91 (13)	

Gross Pathology Animal Details:

No animal details found

Gross Pathology Observations [Correlation]:

GENERAL OBSERVATIONS : (Comment) Liver lesion submitted cass A;
GLAND, SEMINAL VESICLE : Small : (Comment) left. (TGL)
LIVER : Focus, pale : (Comment) 3, right lateral, near hilus. (TGL)

Any remaining study plan required tissues, which have been examined, have no visible lesions

Gross Pathology - The following Tissues were Not Examined:

None

Histopathology Animal Details:

No animal details found

Histopathology Observations [Correlation]:

GANGLION, DORSAL ROOT, LUMBAR : Infiltration, mononuclear cell; mild
GANGLION, DORSAL ROOT, LUMBAR : Degeneration; neuronal, minimal
GLAND, ADRENAL : Vacuolation; cortical, mild
KIDNEY : Infiltration, mononuclear cell; minimal
LIVER : Infiltration, mixed cell; minimal
NERVE, SCIATIC : Degeneration; axonal, minimal
NERVE, TIBIAL : Degeneration; axonal, minimal
SITE, INJECTION : Infiltration, mononuclear cell; minimal, leptomeninges
SITE, INJECTION : Degeneration; axonal, minimal, cauda equina
NO CORRELATE : No correlating lesion [GLAND, SEMINAL VESICLE : Small : (Comment) left. (G) | LIVER : Focus, pale : (Comment) 3, right lateral, near hilus. (G)]
NERVE ROOT, DORSAL, LUMBAR : Degeneration; axonal, minimal

Histopathology - The following Tissues were Within Normal Limits:

BRAIN; EPIDIDYMIS; EYE; GANGLION, DORSAL ROOT, CERVICAL; GANGLION, DORSAL ROOT, THORACIC; GLAND, PARATHYROID; GLAND, PITUITARY; GLAND, PROSTATE; GLAND, SEMINAL VESICLE; GLAND, THYROID; HEART; LUNG; LYMPH NODE, CERVICAL; LYMPH NODE, ILIAC; LYMPH NODE, MANDIBULAR; LYMPH NODE, MESENTERIC; MUSCLE, BICEPS FEMORIS; MUSCLE, GASTROCNEMIUS; NERVE, OPTIC; SPINAL CORD, CERVICAL; SPINAL CORD, LUMBAR; SPINAL CORD, THORACIC; SPLEEN; TESTIS; THYMUS; NERVE ROOT, VENTRAL, CERVICAL; NERVE ROOT, VENTRAL, THORACIC; NERVE ROOT, VENTRAL, LUMBAR; NERVE ROOT, DORSAL, CERVICAL; NERVE ROOT, DORSAL, THORACIC

Histopathology - The following Tissues were Not Examined:

None

Appendix 18

Appendix 4

Individual Macroscopic and Microscopic Pathology:

5550008

Animal: 4015	Group: 4	Sex: Male
Species: Rat	Strain: Sprague Dawley<br>Dose: 3.3x 10E12 vg<br>Removal Reason: Recovery Euthanasia<br>Study Day (Week) of Death: 91 (13)	

Palpable Mass Details:

Mass 1; Inguinal, Left; Soft,Subcutaneous,Movable,Normal surface; 45-49 mm

Gross Pathology Animal Details:

No animal details found

Gross Pathology Observations [Correlation]:

GENERAL OBSERVATIONS : (Comment) lymph node mandibular lesion right submitted with salivary gland mandibular right
EPIDIDYMIS : (Comment) Testis and epididymis left found subcutaneously in inguinal region left, may correspond to mass 1
EPIDIDYMIS : Small : (Comment) left (TGL)
LYMPH NODE, MANDIBULAR : Enlargement : (Comment) bilateral (TGL)
TESTIS : (Comment) Testis and epididymis left found subcutaneously in inguinal region left, may correspond to mass 1.
TESTIS : Small : (Comment) left (TGL)
NO CORRELATE : No correlating lesion [Mass 1; Inguinal, Left; Soft,Subcutaneous,Movable,Normal surface; 45-49 mm (M)]

Any remaining study plan required tissues, which have been examined, have no visible lesions

Gross Pathology - The following Tissues were Not Examined:

None

Histopathology Animal Details:

No animal details found

Histopathology Observations [Correlation]:

EPIDIDYMIS : Cellular debris; unilateral, moderate
EPIDIDYMIS : Sperm, decreased; unilateral, severe [EPIDIDYMIS : Small : (Comment) left (G)]
GLAND, PROSTATE : Infiltration, mononuclear cell; minimal
KIDNEY : Infiltration, mononuclear cell; minimal
LIVER : Vacuolation; hepatocellular, periportal, minimal
LIVER : Infiltration, mononuclear cell; periportal, minimal
LYMPH NODE, MANDIBULAR : Cellularity, increased; lymphoid, mild [LYMPH NODE, MANDIBULAR : Enlargement : (Comment) bilateral (G)]
TESTIS : Degeneration/atrophy; unilateral, moderate, seminiferous tubule [TESTIS : Small : (Comment) left (G)]

Histopathology - The following Tissues were Within Normal Limits:

Individual Macroscopic and Microscopic Pathology:

5550008

Animal: 4015 (Continued)	Group: 4	Sex: Male

BRAIN; EYE; GANGLION, DORSAL ROOT, CERVICAL; GANGLION, DORSAL ROOT, LUMBAR; GANGLION, DORSAL ROOT, THORACIC; GLAND, ADRENAL; GLAND, PARATHYROID; GLAND, PITUITARY; GLAND, THYROID; HEART; LUNG; LYMPH NODE, CERVICAL; LYMPH NODE, ILIAC; LYMPH NODE, MESENTERIC; MUSCLE, BICEPS FEMORIS; MUSCLE, GASTROCNEMIUS; NERVE, OPTIC; NERVE, SCIATIC; NERVE, TIBIAL; SITE, INJECTION; SPINAL CORD, CERVICAL; SPINAL CORD, LUMBAR; SPINAL CORD, THORACIC; SPLEEN; THYMUS; NERVE ROOT, VENTRAL, CERVICAL; NERVE ROOT, VENTRAL, THORACIC; NERVE ROOT, VENTRAL, LUMBAR; NERVE ROOT, DORSAL, CERVICAL; NERVE ROOT, DORSAL, LUMBAR; NERVE ROOT, DORSAL, THORACIC

Histopathology - The following Tissues were Not Examined:

None

Individual Macroscopic and Microscopic Pathology:

5550008

Animal: 4601	Group: 4	Sex: Female
Species: Rat	Strain: Sprague Dawley	
	Dose: 3.3x 10E12 vg	
	Removal Reason: Terminal Euthanasia	
	Study Day (Week) of Death: 8 (2)	

Gross Pathology Animal Details:

No animal details found

Gross Pathology Observations [Correlation]:

No observations found

Any remaining study plan required tissues, which have been examined, have no visible lesions

Gross Pathology - The following Tissues were Not Examined:

None

Histopathology Animal Details:

No animal details found

Histopathology Observations [Correlation]:

GANGLION, DORSAL ROOT, LUMBAR : Infiltration, mononuclear cell; minimal
GLAND, PARATHYROID : (Note) One Of A Pair Available For Evaluation.
GLAND, THYROID : (Note) One Of A Pair Available For Evaluation.
NERVE, TIBIAL : Degeneration; axonal, minimal

Histopathology - The following Tissues were Within Normal Limits:

BRAIN; CERVIX; EYE; GANGLION, DORSAL ROOT, CERVICAL; GANGLION, DORSAL ROOT, THORACIC; GLAND, ADRENAL; GLAND, PARATHYROID; GLAND, PITUITARY; GLAND, THYROID; HEART; KIDNEY; LIVER; LUNG; LYMPH NODE, CERVICAL; LYMPH NODE, MANDIBULAR; LYMPH NODE, MESENTERIC; MUSCLE, BICEPS FEMORIS; MUSCLE, GASTROCNEMIUS; NERVE, OPTIC; NERVE, SCIATIC; OVARY; SITE, INJECTION; SPINAL CORD, CERVICAL; SPINAL CORD, LUMBAR; SPINAL CORD, THORACIC; SPLEEN; THYMUS; UTERUS; NERVE ROOT, VENTRAL, CERVICAL; NERVE ROOT, VENTRAL, THORACIC; NERVE ROOT, VENTRAL, LUMBAR; NERVE ROOT, DORSAL, CERVICAL; NERVE ROOT, DORSAL, LUMBAR; NERVE ROOT, DORSAL, THORACIC

Histopathology - The following Tissues were Not Examined:

LYMPH NODE, ILIAC - Not Present In Section.

Appendix 18

Appendix 4

Individual Macroscopic and Microscopic Pathology:

5550008

Animal: 4502	Group: 4	Sex: Female
Species: Rat	Strain: Sprague Dawley	
	Dose: 3.3x 10E12 vg	
	Removal Reason: Terminal Euthanasia	
	Study Day (Week) of Death: 8 (2)	

Gross Pathology Animal Details:

No animal details found

Gross Pathology Observations [Correlation]:

GLAND, THYROID : Small : (Comment) bilateral (TGL)

Any remaining study plan required tissues, which have been examined, have no visible lesions

Gross Pathology - The following Tissues were Not Examined:

None

Histopathology Animal Details:

No animal details found

Histopathology Observations [Correlation]:

GANGLION, DORSAL ROOT, LUMBAR : Infiltration, mononuclear cell; minimal
GLAND, PARATHYROID : (Note) One Of A Pair Available For Evaluation.
GLAND, PITUITARY : (Note) One Of A Pair Available For Evaluation.
LIVER : Infiltration, mononuclear cell; periportal, minimal
NERVE, SCIATIC : Degeneration; axonal, minimal
SITE, INJECTION : Degeneration; axonal, minimal, cauda equina
NO CORRELATE : No correlating lesion [GLAND, THYROID : Small : (Comment) bilateral (G)]

Histopathology - The following Tissues were Within Normal Limits:

BRAIN; CERVIX; EYE; GANGLION, DORSAL ROOT, CERVICAL; GANGLION, DORSAL ROOT, THORACIC; GLAND, ADRENAL; GLAND, PARATHYROID; GLAND, PITUITARY; GLAND, THYROID; HEART; KIDNEY; LUNG; LYMPH NODE, CERVICAL; LYMPH NODE, ILIAC; LYMPH NODE, MANDIBULAR; LYMPH NODE, MESENTERIC; MUSCLE, BICEPS FEMORIS; MUSCLE, GASTROCNEMIUS; NERVE, OPTIC; NERVE, TIBIAL; OVARY; SPINAL CORD, CERVICAL; SPINAL CORD, LUMBAR; SPINAL CORD, THORACIC; SPLEEN; THYMUS; UTERUS; NERVE ROOT, VENTRAL, CERVICAL; NERVE ROOT, VENTRAL, THORACIC; NERVE ROOT, VENTRAL, LUMBAR; NERVE ROOT, DORSAL, CERVICAL; NERVE ROOT, DORSAL, LUMBAR; NERVE ROOT, DORSAL, THORACIC

Histopathology - The following Tissues were Not Examined:

None

Appendix 18

Appendix 4

Individual Macroscopic and Microscopic Pathology:

5550008

Animal: 4503	Group: 4	Sex: Female
Species: Rat	Strain: Sprague Dawley	
	Dose: 3.3x 10E12 vg	
	Removal Reason: Recovery Euthanasia	
	Study Day (Week) of Death: 91 (13)	

Gross Pathology Animal Details:

No animal details found

Gross Pathology Observations [Correlation]:

GLAND, PITUITARY : Enlargement (TGL)
GLAND, THYROID : Small : (Comment) bilateral (TGL)
LYMPH NODE, MESENTERIC : Enlargement (TGL)

Any remaining study plan required tissues, which have been examined, have no visible lesions

Gross Pathology - The following Tissues were Not Examined:

None

Histopathology Animal Details:

No animal details found

Histopathology Observations [Correlation]:

GANGLION, DORSAL ROOT, LUMBAR : Infiltration, mononuclear cell; minimal
GANGLION, DORSAL ROOT, LUMBAR : Degeneration; neuronal, minimal
GLAND, PARATHYROID : (Note) One Of A Pair Available For Evaluation.
GLAND, PITUITARY : Hypertrophy/hyperplasia; diffuse, mild, pars distalis [GLAND, PITUITARY : Enlargement (G)]
LIVER : Vacuolation; hepatocellular, periportal, minimal
LIVER : Infiltration, mixed cell; minimal
LYMPH NODE, MESENTERIC : Cellularity, increased; lymphoid, mild [LYMPH NODE, MESENTERIC : Enlargement (G)]
NERVE, SCIATIC : Degeneration; axonal, minimal
NERVE, TIBIAL : Degeneration; axonal, minimal
SITE, INJECTION : Infiltration, mononuclear cell; minimal, leptomeninges
SITE, INJECTION : Degeneration; axonal, minimal, cauda equina
SPINAL CORD, THORACIC : Infiltration, mononuclear cell; minimal, adipose tissue
NO CORRELATE : No correlating lesion [GLAND, THYROID : Small : (Comment) bilateral (G)]
NERVE ROOT, DORSAL, LUMBAR : Degeneration; axonal, mild

Histopathology - The following Tissues were Within Normal Limits:

BRAIN; CERVIX; EYE; GANGLION, DORSAL ROOT, CERVICAL; GANGLION, DORSAL ROOT, THORACIC; GLAND, ADRENAL; GLAND, PARATHYROID; GLAND, THYROID; HEART; KIDNEY; LUNG; LYMPH NODE, CERVICAL; LYMPH NODE, ILIAC; LYMPH NODE, MANDIBULAR; MUSCLE, BICEPS FEMORIS; MUSCLE, GASTROCNEMIUS; NERVE, OPTIC; OVARY; SPINAL CORD, CERVICAL; SPINAL CORD, LUMBAR; SPLEEN; THYMUS; UTERUS; NERVE ROOT, VENTRAL, CERVICAL; NERVE ROOT, VENTRAL, THORACIC; NERVE ROOT, VENTRAL, LUMBAR; NERVE ROOT, DORSAL, CERVICAL; NERVE ROOT, DORSAL, THORACIC

Histopathology - The following Tissues were Not Examined:

Appendix 18

Appendix 4

Individual Macroscopic and Microscopic Pathology:

5550008

Animal: 4503 (Continued)	Group: 4	Sex: Female
None		

Individual Macroscopic and Microscopic Pathology:

5550008

Animal: 4504	Group: 4	Sex: Female
Species: Rat	Strain: Sprague Dawley	
	Dose: 3.3x 10E12 vg	
	Removal Reason: Recovery Euthanasia	
	Study Day (Week) of Death: 91 (13)	

Gross Pathology Animal Details:

No animal details found

Gross Pathology Observations [Correlation]:

GENERAL OBSERVATIONS : (Comment) Thymus lesion submitted with general histology section
LYMPH NODE, MANDIBULAR : Enlargement : (Comment) bilateral (TGL)
THYMUS : Focus, dark : (Comment) 5 (TGL)
WHOLE ANIMAL : Decreased adipose tissue

Any remaining study plan required tissues, which have been examined, have no visible lesions

Gross Pathology - The following Tissues were Not Examined:

None

Histopathology Animal Details:

No animal details found

Histopathology Observations [Correlation]:

GANGLION, DORSAL ROOT, LUMBAR : Infiltration, mononuclear cell; minimal
GANGLION, DORSAL ROOT, LUMBAR : Degeneration; neuronal, minimal
KIDNEY : Dilatation; tubular, minimal
LIVER : Infiltration, mixed cell; minimal
LYMPH NODE, MANDIBULAR : Plasmacytosis; mild
LYMPH NODE, MANDIBULAR : Cellularity, increased; lymphoid, moderate [LYMPH NODE, MANDIBULAR : Enlargement : (Comment) bilateral (G)]
NERVE, SCIATIC : Degeneration; axonal, minimal
NERVE, TIBIAL : Degeneration; axonal, minimal
SITE, INJECTION : Infiltration, mononuclear cell; minimal, leptomeninges
SITE, INJECTION : Degeneration; axonal, minimal, cauda equina
SPINAL CORD, THORACIC : Infiltration, mononuclear cell; minimal, adipose tissue
NO CORRELATE : No correlating lesion [THYMUS : Focus, dark : (Comment) 5 (G)]
NERVE ROOT, DORSAL, LUMBAR : Degeneration; axonal, mild

Histopathology - The following Tissues were Within Normal Limits:

BRAIN; CERVIX; EYE; GANGLION, DORSAL ROOT, CERVICAL; GANGLION, DORSAL ROOT, THORACIC; GLAND, ADRENAL; GLAND, PARATHYROID; GLAND, PITUITARY; GLAND, THYROID; HEART; LUNG; LYMPH NODE, CERVICAL; LYMPH NODE, ILIAC; LYMPH NODE, MESENTERIC; MUSCLE, BICEPS FEMORIS; MUSCLE, GASTROCNEMIUS; NERVE, OPTIC; OVARY; SPINAL CORD, CERVICAL; SPINAL CORD, LUMBAR; SPLEEN; THYMUS; UTERUS; NERVE ROOT, VENTRAL, CERVICAL; NERVE ROOT, VENTRAL, THORACIC; NERVE ROOT, VENTRAL, LUMBAR; NERVE ROOT, DORSAL, CERVICAL; NERVE ROOT, DORSAL, THORACIC

Histopathology - The following Tissues were Not Examined:

None

Appendix 18

Appendix 4

Individual Macroscopic and Microscopic Pathology:

5550008

Animal: 4505	Group: 4	Sex: Female
Species: Rat	Strain: Sprague Dawley<br>Dose: 3.3x 10E12 vg<br>Removal Reason: Recovery Euthanasia<br>Study Day (Week) of Death: 29 (5)	

Gross Pathology Animal Details:

No animal details found

Gross Pathology Observations [Correlation]:

No observations found

Any remaining study plan required tissues, which have been examined, have no visible lesions

Gross Pathology - The following Tissues were Not Examined:

None

Histopathology Animal Details:

No animal details found

Histopathology Observations [Correlation]:

GANGLION, DORSAL ROOT, LUMBAR : Infiltration, mononuclear cell; mild
GANGLION, DORSAL ROOT, LUMBAR : Degeneration; axonal, minimal
KIDNEY : Infiltration, mononuclear cell; minimal
LIVER : Infiltration, mixed cell; minimal
NERVE, SCIATIC : Degeneration; axonal, minimal
NERVE, TIBIAL : Degeneration; axonal, minimal
SITE, INJECTION : Infiltration, mononuclear cell; minimal, leptomeninges
SITE, INJECTION : Degeneration; axonal, mild, cauda equina
NERVE ROOT, DORSAL, LUMBAR : Degeneration; axonal, mild

Histopathology - The following Tissues were Within Normal Limits:

BRAIN; CERVIX; EYE; GANGLION, DORSAL ROOT, CERVICAL; GANGLION, DORSAL ROOT, THORACIC; GLAND, ADRENAL; GLAND, PITUITARY; GLAND, THYROID; HEART; LUNG; LYMPH NODE, CERVICAL; LYMPH NODE, ILIAC; LYMPH NODE, MANDIBULAR; LYMPH NODE, MESENTERIC; MUSCLE, BICEPS FEMORIS; MUSCLE, GASTROCNEMIUS; OVARY; SPINAL CORD, CERVICAL; SPINAL CORD, LUMBAR; SPINAL CORD, THORACIC; SPLEEN; THYMUS; UTERUS; NERVE ROOT, VENTRAL, CERVICAL; NERVE ROOT, VENTRAL, THORACIC; NERVE ROOT, VENTRAL, LUMBAR; NERVE ROOT, DORSAL, CERVICAL; NERVE ROOT, DORSAL, THORACIC

Histopathology - The following Tissues were Not Examined:

GLAND, PARATHYROID - Not Present In Section.
NERVE, OPTIC - Not Present In Section.

Individual Macroscopic and Microscopic Pathology:

5550008

Animal: 4506	Group: 4	Sex: Female
Species: Rat	Strain: Sprague Dawley<br>Dose: 3.3x 10E12 vg<br>Removal Reason: Recovery Euthanasia<br>Study Day (Week) of Death: 29 (5)	

Gross Pathology Animal Details:

No animal details found

Gross Pathology Observations [Correlation]:

No observations found

Any remaining study plan required tissues, which have been examined, have no visible lesions

Gross Pathology - The following Tissues were Not Examined:

None

Histopathology Animal Details:

No animal details found

Histopathology Observations [Correlation]:

GANGLION, DORSAL ROOT, LUMBAR : Infiltration, mononuclear cell; minimal
GANGLION, DORSAL ROOT, LUMBAR : Degeneration; axonal, minimal
GANGLION, DORSAL ROOT, LUMBAR : Degeneration; neuronal, minimal
GLAND, PITUITARY : (Note) Only Pars Distalis Available For Evaluation.
KIDNEY : Chronic progressive nephropathy; minimal
LIVER : Vacuolation; hepatocellular, periportal, minimal
LIVER : Infiltration, mixed cell; minimal
LIVER : Infiltration, mononuclear cell; periportal, minimal
NERVE, SCIATIC : Degeneration; axonal, mild
NERVE, TIBIAL : Degeneration; axonal, mild
SITE, INJECTION : Degeneration; axonal, mild, cauda equina
SPINAL CORD, LUMBAR : Infiltration, mononuclear cell; minimal, leptomeninges
NERVE ROOT, DORSAL, LUMBAR : Degeneration; axonal, moderate

Histopathology - The following Tissues were Within Normal Limits:

BRAIN; CERVIX; EYE; GANGLION, DORSAL ROOT, CERVICAL; GANGLION, DORSAL ROOT, THORACIC; GLAND, ADRENAL; GLAND, PARATHYROID; GLAND, PITUITARY; GLAND, THYROID; HEART; LUNG; LYMPH NODE, CERVICAL; LYMPH NODE, ILIAC; LYMPH NODE, MANDIBULAR; LYMPH NODE, MESENTERIC; MUSCLE, BICEPS FEMORIS; MUSCLE, GASTROCNEMIUS; NERVE, OPTIC; OVARY; SPINAL CORD, CERVICAL; SPINAL CORD, THORACIC; SPLEEN; THYMUS; UTERUS; NERVE ROOT, VENTRAL, CERVICAL; NERVE ROOT, VENTRAL, THORACIC; NERVE ROOT, VENTRAL, LUMBAR; NERVE ROOT, DORSAL, CERVICAL; NERVE ROOT, DORSAL, THORACIC

Histopathology - The following Tissues were Not Examined:

None

Individual Macroscopic and Microscopic Pathology:

5550008

Animal: 4507	Group: 4	Sex: Female
Species: Rat	Strain: Sprague Dawley<br>Dose: 3.3x 10E12 vg<br>Removal Reason: Recovery Euthanasia<br>Study Day (Week) of Death: 29 (5)	

Gross Pathology Animal Details:

No animal details found

Gross Pathology Observations [Correlation]:

No observations found

Any remaining study plan required tissues, which have been examined, have no visible lesions

Gross Pathology - The following Tissues were Not Examined:

None

Histopathology Animal Details:

No animal details found

Histopathology Observations [Correlation]:

GANGLION, DORSAL ROOT, LUMBAR : Infiltration, mononuclear cell; minimal
GANGLION, DORSAL ROOT, LUMBAR : Degeneration; axonal, minimal
GANGLION, DORSAL ROOT, THORACIC : Infiltration, mononuclear cell; minimal
KIDNEY : Infiltration, mononuclear cell; minimal
KIDNEY : Chronic progressive nephropathy; minimal
LIVER : Infiltration, mixed cell; minimal
NERVE, SCIATIC : Degeneration; axonal, mild
NERVE, TIBIAL : Degeneration; axonal, minimal
SITE, INJECTION : Infiltration, mononuclear cell; minimal, leptomeninges
SITE, INJECTION : Degeneration; axonal, mild, cauda equina
SPINAL CORD, THORACIC : Infiltration, mononuclear cell; minimal, adipose tissue
NERVE ROOT, DORSAL, LUMBAR : Degeneration; axonal, minimal

Histopathology - The following Tissues were Within Normal Limits:

BRAIN; CERVIX; EYE; GANGLION, DORSAL ROOT, CERVICAL; GLAND, ADRENAL; GLAND, PITUITARY; GLAND, THYROID; HEART; LUNG; LYMPH NODE, CERVICAL; LYMPH NODE, ILIAC; LYMPH NODE, MANDIBULAR; LYMPH NODE, MESENTERIC; MUSCLE, BICEPS FEMORIS; MUSCLE, GASTROCNEMIUS; NERVE, OPTIC; OVARY; SPINAL CORD, CERVICAL; SPINAL CORD, LUMBAR; SPLEEN; THYMUS; UTERUS; NERVE ROOT, VENTRAL, CERVICAL; NERVE ROOT, VENTRAL, THORACIC; NERVE ROOT, VENTRAL, LUMBAR; NERVE ROOT, DORSAL, CERVICAL; NERVE ROOT, DORSAL, THORACIC

Histopathology - The following Tissues were Not Examined:

GLAND, PARATHYROID - Not Present In Section.

Individual Macroscopic and Microscopic Pathology:

5550008

Animal:	4508	Group:	4	Sex:	Female
Species:	Rat	Strain:	Sprague Dawley		
		Dose:	3.3x 10E12 vg		
		Removal Reason:	Terminal Euthanasia		
		Study Day (Week) of Death:	8 (2)		

Gross Pathology Animal Details:

No animal details found

Gross Pathology Observations [Correlation]:

No observations found

Any remaining study plan required tissues, which have been examined, have no visible lesions

Gross Pathology - The following Tissues were Not Examined:

None

Histopathology Animal Details:

No animal details found

Histopathology Observations [Correlation]:

EYE : Dysplasia; minimal, retina
GLAND, PARATHYROID : (Note) One Of A Pair Available For Evaluation.
KIDNEY : Infiltration, mononuclear cell; minimal
KIDNEY : Chronic progressive nephropathy; minimal

Histopathology - The following Tissues were Within Normal Limits:

BRAIN; CERVIX; GANGLION, DORSAL ROOT, CERVICAL; GANGLION, DORSAL ROOT, LUMBAR; GANGLION, DORSAL ROOT, THORACIC; GLAND, ADRENAL; GLAND, PARATHYROID; GLAND, PITUITARY; GLAND, THYROID; HEART; LIVER; LUNG; LYMPH NODE, CERVICAL; LYMPH NODE, ILIAC; LYMPH NODE, MANDIBULAR; LYMPH NODE, MESENTERIC; MUSCLE, BICEPS FEMORIS; MUSCLE, GASTROCNEMIUS; NERVE, OPTIC; NERVE, SCIATIC; NERVE, TIBIAL; OVARY; SITE, INJECTION; SPINAL CORD, CERVICAL; SPINAL CORD, LUMBAR; SPINAL CORD, THORACIC; SPLEEN; THYMUS; UTERUS; NERVE ROOT, VENTRAL, CERVICAL; NERVE ROOT, VENTRAL, THORACIC; NERVE ROOT, VENTRAL, LUMBAR; NERVE ROOT, DORSAL, CERVICAL; NERVE ROOT, DORSAL, LUMBAR; NERVE ROOT, DORSAL, THORACIC

Histopathology - The following Tissues were Not Examined:

None

Individual Macroscopic and Microscopic Pathology:

5550008

Animal: 4509	Group: 4	Sex: Female
Species: Rat	Strain: Sprague Dawley<br>Dose: 3.3x 10E12 vg<br>Removal Reason: Terminal Euthanasia<br>Study Day (Week) of Death: 8 (2)	

Gross Pathology Animal Details:

No animal details found

Gross Pathology Observations [Correlation]:

GENERAL OBSERVATIONS : (Comment) Site surgical lesion submitted on plastic in bag 1;
SITE, SURGICAL : Focus, dark : (Comment) >10, subcutis. (TGL)
UTERUS : Cyst, pale : (Comment) 2, serosa, horn, right. (TGL)

Any remaining study plan required tissues, which have been examined, have no visible lesions

Gross Pathology - The following Tissues were Not Examined:

None

Histopathology Animal Details:

No animal details found

Histopathology Observations [Correlation]:

GANGLION, DORSAL ROOT, LUMBAR : Infiltration, mononuclear cell; minimal
KIDNEY : Dilatation; tubular, mild : (Comment) with peripheral fibrosis
LIVER : Vacuolation; hepatocellular, periportal, minimal
SITE, SURGICAL : Hemorrhage; acute, regionally extensive, mild [SITE, SURGICAL : Focus, dark : (Comment) >10, subcutis. (G)]
SITE, SURGICAL : Fibroplasia; regionally extensive, moderate : (Comment) seroma formation in subcutis, with mononuclear cell infiltration, fibrin/proteinaceous material accumulation, and acute hemorrhage
SITE, SURGICAL : Hyperplasia; epidermal, focal, minimal
UTERUS : Cyst [UTERUS : Cyst, pale : (Comment) 2, serosa, horn, right. (G)]

Histopathology - The following Tissues were Within Normal Limits:

BRAIN; CERVIX; EYE; GANGLION, DORSAL ROOT, CERVICAL; GANGLION, DORSAL ROOT, THORACIC; GLAND, ADRENAL; GLAND, PITUITARY; GLAND, THYROID; HEART; LUNG; LYMPH NODE, CERVICAL; LYMPH NODE, ILIAC; LYMPH NODE, MANDIBULAR; LYMPH NODE, MESENTERIC; MUSCLE, BICEPS FEMORIS; MUSCLE, GASTROCNEMIUS; NERVE, OPTIC; NERVE, SCIATIC; NERVE, TIBIAL; OVARY; SITE, INJECTION; SPINAL CORD, CERVICAL; SPINAL CORD, LUMBAR; SPINAL CORD, THORACIC; SPLEEN; THYMUS; NERVE ROOT, VENTRAL, CERVICAL; NERVE ROOT, VENTRAL, THORACIC; NERVE ROOT, VENTRAL, LUMBAR; NERVE ROOT, DORSAL, CERVICAL; NERVE ROOT, DORSAL, LUMBAR; NERVE ROOT, DORSAL, THORACIC

Histopathology - The following Tissues were Not Examined:

GLAND, PARATHYROID - Not Present In Section.

Appendix 18

Appendix 4

Individual Macroscopic and Microscopic Pathology:

5550008

Animal: 4510	Group: 4	Sex: Female
Species: Rat	Strain: Sprague Dawley<br>Dose: 3.3x 10E12 vg<br>Removal Reason: Terminal Euthanasia<br>Study Day (Week) of Death: 8 (2)	

Gross Pathology Animal Details:

No animal details found

Gross Pathology Observations [Correlation]:

GENERAL OBSERVATIONS : (Comment) lymph node lesion renal right cass E, left cass F, lymph node mediastinal submitted cass G
LYMPH NODE : Discoloration, mottled : (Comment) renal, bilateral; mediastinal (TGL)
LYMPH NODE, MANDIBULAR : Focus, dark : (Comment) 2, right (TGL)
THYMUS : Discoloration, mottled (TGL)

Any remaining study plan required tissues, which have been examined, have no visible lesions

Gross Pathology - The following Tissues were Not Examined:

None

Histopathology Animal Details:

No animal details found

Histopathology Observations [Correlation]:

KIDNEY : (Note) Only Cortex Available For Evaluation.
KIDNEY : Chronic progressive nephropathy; minimal
LYMPH NODE : Hemorrhage; acute, mild [LYMPH NODE : Discoloration, mottled : (Comment) renal, bilateral; mediastinal (G)]
LYMPH NODE, MANDIBULAR : Hemorrhage; acute, mild [LYMPH NODE, MANDIBULAR : Focus, dark : (Comment) 2, right (G)]
SITE, INJECTION : Infiltration, mononuclear cell; minimal, leptomeninges
NO CORRELATE : No correlating lesion [THYMUS : Discoloration, mottled (G)]

Histopathology - The following Tissues were Within Normal Limits:

BRAIN; CERVIX; EYE; GANGLION, DORSAL ROOT, CERVICAL; GANGLION, DORSAL ROOT, LUMBAR; GANGLION, DORSAL ROOT, THORACIC; GLAND, ADRENAL; GLAND, PARATHYROID; GLAND, PITUITARY; GLAND, THYROID; HEART; LIVER; LUNG; LYMPH NODE, CERVICAL; LYMPH NODE, ILIAC; LYMPH NODE, MESENTERIC; MUSCLE, BICEPS FEMORIS; MUSCLE, GASTROCNEMIUS; NERVE, OPTIC; NERVE, SCIATIC; NERVE, TIBIAL; OVARY; SPINAL CORD, CERVICAL; SPINAL CORD, LUMBAR; SPINAL CORD, THORACIC; SPLEEN; THYMUS; UTERUS; NERVE ROOT, VENTRAL, CERVICAL; NERVE ROOT, VENTRAL, THORACIC; NERVE ROOT, VENTRAL, LUMBAR; NERVE ROOT, DORSAL, CERVICAL; NERVE ROOT, DORSAL, LUMBAR; NERVE ROOT, DORSAL, THORACIC

Histopathology - The following Tissues were Not Examined:

None

Appendix 18

Appendix 4

Individual Macroscopic and Microscopic Pathology:

5550008

Animal:	4511	Group:	4	Sex:	Female
Species:	Rat	Strain:	Sprague Dawley		
		Dose:	3.3x 10E12 vg		
		Removal Reason: Recovery Euthanasia			
		Study Day (Week) of Death: 29 (5)			

Gross Pathology Animal Details:

No animal details found

Gross Pathology Observations [Correlation]:

GENERAL OBSERVATIONS : (Comment) Lymph node lesion submitted in cassette E.
LYMPH NODE : Discoloration, mottled : (Comment) mediastinal (TGL)

Any remaining study plan required tissues, which have been examined, have no visible lesions

Gross Pathology - The following Tissues were Not Examined:

None

Histopathology Animal Details:

No animal details found

Histopathology Observations [Correlation]:

GANGLION, DORSAL ROOT, CERVICAL : Degeneration; axonal, minimal
GANGLION, DORSAL ROOT, CERVICAL : Infiltration, mononuclear cell; minimal
GANGLION, DORSAL ROOT, LUMBAR : Infiltration, mononuclear cell; mild
GANGLION, DORSAL ROOT, LUMBAR : Degeneration; axonal, mild
GANGLION, DORSAL ROOT, LUMBAR : Degeneration; neuronal, minimal
GANGLION, DORSAL ROOT, THORACIC : Degeneration; axonal, minimal
GLAND, PARATHYROID : (Note) One Of A Pair Available For Evaluation.
HEART : Infiltration, mononuclear cell/degeneration, myofiber; minimal
KIDNEY : Cast; hyaline, minimal
KIDNEY : Infiltration, mononuclear cell; minimal
KIDNEY : Dilatation; tubular, minimal : (Comment) with hyaline eosinophilic material
LIVER : Vacuolation; hepatocellular, periportal, minimal
LIVER : Infiltration, mixed cell; minimal
LYMPH NODE : Hemorrhage; acute, mild [LYMPH NODE : Discoloration, mottled : (Comment) mediastinal (G)]
NERVE, SCIATIC : Degeneration; axonal, mild
NERVE, TIBIAL : Degeneration; axonal, mild
SITE, INJECTION : Infiltration, mononuclear cell; minimal, leptomeninges
SITE, INJECTION : Degeneration; axonal, moderate, cauda equina
SPINAL CORD, THORACIC : Infiltration, mononuclear cell; minimal, adipose tissue
NERVE ROOT, DORSAL, LUMBAR : Degeneration; axonal, mild

Histopathology - The following Tissues were Within Normal Limits:

Appendix 18

Appendix 4

Individual Macroscopic and Microscopic Pathology:

5550008

Animal: 4511 (Continued) Group: 4 Sex: Female

BRAIN; CERVIX; EYE; GLAND, ADRENAL; GLAND, PARATHYROID; GLAND, PITUITARY; GLAND, THYROID; LUNG; LYMPH NODE, CERVICAL; LYMPH NODE, ILIAC; LYMPH NODE, MANDIBULAR; LYMPH NODE, MESENTERIC; MUSCLE, BICEPS FEMORIS; MUSCLE, GASTROCNEMIUS; NERVE, OPTIC; OVARY; SPINAL CORD, CERVICAL; SPINAL CORD, LUMBAR; SPLEEN; THYMUS; UTERUS; NERVE ROOT, VENTRAL, CERVICAL; NERVE ROOT, VENTRAL, THORACIC; NERVE ROOT, VENTRAL, LUMBAR; NERVE ROOT, DORSAL, CERVICAL; NERVE ROOT, DORSAL, THORACIC

Histopathology - The following Tissues were Not Examined:

None

Individual Macroscopic and Microscopic Pathology:

5550008

Animal:	4512	Group:	4	Sex:	Female
Species:	Rat	Strain:	Sprague Dawley		
		Dose:	3.3x 10E12 vg		
		Removal Reason: Recovery Euthanasia			
		Study Day (Week) of Death: 29 (5)			

Gross Pathology Animal Details:

No animal details found

Gross Pathology Observations [Correlation]:

GENERAL OBSERVATIONS : (Comment) Liver lesion submitted cass B
LIVER : Focus, pale : (Comment) 1, near hilus, right lateral (TGL)

Any remaining study plan required tissues, which have been examined, have no visible lesions

Gross Pathology - The following Tissues were Not Examined:

None

Histopathology Animal Details:

No animal details found

Histopathology Observations [Correlation]:

GANGLION, DORSAL ROOT, LUMBAR : Infiltration, mononuclear cell; minimal
GANGLION, DORSAL ROOT, LUMBAR : Degeneration; axonal, minimal
GANGLION, DORSAL ROOT, LUMBAR : Degeneration; neuronal, minimal
GLAND, PARATHYROID : (Note) One Of A Pair Available For Evaluation.
GLAND, PITUITARY : (Note) Only Pars Distalis Available For Evaluation.
KIDNEY : Infiltration, mononuclear cell; minimal
LIVER : Tension lipidosis; minimal [LIVER : Focus, pale : (Comment) 1, near hilus, right lateral (G)]
LIVER : Infiltration, mixed cell; minimal
NERVE, SCIATIC : Degeneration; axonal, minimal
NERVE, TIBIAL : Degeneration; axonal, minimal
SITE, INJECTION : Infiltration, mononuclear cell; minimal, leptomeninges
SITE, INJECTION : Degeneration; axonal, moderate, cauda equina
NERVE ROOT, DORSAL, LUMBAR : Degeneration; axonal, mild

Histopathology - The following Tissues were Within Normal Limits:

BRAIN; CERVIX; EYE; GANGLION, DORSAL ROOT, CERVICAL; GANGLION, DORSAL ROOT, THORACIC; GLAND, ADRENAL; GLAND, PARATHYROID; GLAND, PITUITARY; GLAND, THYROID; HEART; LUNG; LYMPH NODE, CERVICAL; LYMPH NODE, ILIAC; LYMPH NODE, MANDIBULAR; LYMPH NODE, MESENTERIC; MUSCLE, BICEPS FEMORIS; MUSCLE, GASTROCNEMIUS; NERVE, OPTIC; OVARY; SPINAL CORD, CERVICAL; SPINAL CORD, LUMBAR; SPINAL CORD, THORACIC; SPLEEN; THYMUS; UTERUS; NERVE ROOT, VENTRAL, CERVICAL; NERVE ROOT, VENTRAL, THORACIC; NERVE ROOT, VENTRAL, LUMBAR; NERVE ROOT, DORSAL, CERVICAL; NERVE ROOT, DORSAL, THORACIC

Histopathology - The following Tissues were Not Examined:

None

Individual Macroscopic and Microscopic Pathology:

5550008

Animal: 4513	Group: 4	Sex: Female
Species: Rat	Strain: Sprague Dawley<br>Dose: 3.3x 10E12 vg<br>Removal Reason: Recovery Euthanasia<br>Study Day (Week) of Death: 91 (13)	

Gross Pathology Animal Details:

No animal details found

Gross Pathology Observations [Correlation]:

LIVER : Focus, raised : (Comment) pale, firm, 1, fissure, right medial (TGL)

Any remaining study plan required tissues, which have been examined, have no visible lesions

Gross Pathology - The following Tissues were Not Examined:

None

Histopathology Animal Details:

No animal details found

Histopathology Observations [Correlation]:

LIVER : Vacuolation; hepatocellular, periportal, minimal
LIVER : Infiltration, mixed cell; minimal
NERVE, SCIATIC : Degeneration; axonal, minimal
NERVE, TIBIAL : Degeneration; axonal, minimal
SITE, INJECTION : Infiltration, mononuclear cell; minimal, leptomeninges
SITE, INJECTION : Degeneration; axonal, minimal, cauda equina
NO CORRELATE : No correlating lesion [LIVER : Focus, raised : (Comment) pale, firm, 1, fissure, right medial (G)]
NERVE ROOT, DORSAL, LUMBAR : Degeneration; axonal, mild

Histopathology - The following Tissues were Within Normal Limits:

BRAIN; CERVIX; EYE; GANGLION, DORSAL ROOT, CERVICAL; GANGLION, DORSAL ROOT, LUMBAR; GANGLION, DORSAL ROOT, THORACIC; GLAND, ADRENAL; GLAND, PARATHYROID; GLAND, PITUITARY; GLAND, THYROID; HEART; KIDNEY; LUNG; LYMPH NODE, CERVICAL; LYMPH NODE, ILIAC; LYMPH NODE, MANDIBULAR; LYMPH NODE, MESENTERIC; MUSCLE, BICEPS FEMORIS; MUSCLE, GASTROCNEMIUS; NERVE, OPTIC; OVARY; SPINAL CORD, CERVICAL; SPINAL CORD, LUMBAR; SPINAL CORD, THORACIC; SPLEEN; THYMUS; UTERUS; NERVE ROOT, VENTRAL, CERVICAL; NERVE ROOT, VENTRAL, THORACIC; NERVE ROOT, VENTRAL, LUMBAR; NERVE ROOT, DORSAL, CERVICAL; NERVE ROOT, DORSAL, THORACIC

Histopathology - The following Tissues were Not Examined:

None

Appendix 18

Appendix 4

Individual Macroscopic and Microscopic Pathology:

5550008

Animal:	4514	Group:	4	Sex:	Female
Species:	Rat	Strain:	Sprague Dawley		
		Dose:	3.3x 10E12 vg		
		Removal Reason: Recovery Euthanasia			
		Study Day (Week) of Death: 91 (13)			

Gross Pathology Animal Details:

No animal details found

Gross Pathology Observations [Correlation]:

No observations found

Any remaining study plan required tissues, which have been examined, have no visible lesions

Gross Pathology - The following Tissues were Not Examined:

None

Histopathology Animal Details:

No animal details found

Histopathology Observations [Correlation]:

GANGLION, DORSAL ROOT, LUMBAR : Degeneration; axonal, minimal
GLAND, PITUITARY : (Note) Only Pars Distalis Available For Evaluation.
LIVER : Infiltration, mixed cell; minimal
LIVER : Infiltration, mononuclear cell; periportal, minimal
NERVE, SCIATIC : Degeneration; axonal, minimal
NERVE, TIBIAL : Degeneration; axonal, minimal
SITE, INJECTION : Degeneration; axonal, minimal, cauda equina
NERVE ROOT, DORSAL, LUMBAR : Degeneration; axonal, mild

Histopathology - The following Tissues were Within Normal Limits:

BRAIN; CERVIX; EYE; GANGLION, DORSAL ROOT, CERVICAL; GANGLION, DORSAL ROOT, THORACIC; GLAND, ADRENAL; GLAND, PARATHYROID; GLAND, PITUITARY; GLAND, THYROID; HEART; KIDNEY; LUNG; LYMPH NODE, CERVICAL; LYMPH NODE, ILIAC; LYMPH NODE, MANDIBULAR; LYMPH NODE, MESENTERIC; MUSCLE, BICEPS FEMORIS; MUSCLE, GASTROCNEMIUS; NERVE, OPTIC; OVARY; SPINAL CORD, CERVICAL; SPINAL CORD, LUMBAR; SPINAL CORD, THORACIC; SPLEEN; THYMUS; UTERUS; NERVE ROOT, VENTRAL, CERVICAL; NERVE ROOT, VENTRAL, THORACIC; NERVE ROOT, VENTRAL, LUMBAR; NERVE ROOT, DORSAL, CERVICAL; NERVE ROOT, DORSAL, THORACIC

Histopathology - The following Tissues were Not Examined:

None

Appendix 18

Appendix 4

Individual Macroscopic and Microscopic Pathology:

5550008

Animal:	4515	Group:	4	Sex:	Female
Species:	Rat	Strain:	Sprague Dawley		
		Dose:	3.3x 10E12 vg		
		Removal Reason: Recovery Euthanasia			
		Study Day (Week) of Death: 91 (13)			

Gross Pathology Animal Details:

No animal details found

Gross Pathology Observations [Correlation]:

GLAND, PITUITARY : Enlargement (TGL)

Any remaining study plan required tissues, which have been examined, have no visible lesions

Gross Pathology - The following Tissues were Not Examined:

None

Histopathology Animal Details:

No animal details found

Histopathology Observations [Correlation]:

GLAND, PITUITARY : (Note) Only Pars Distalis Available For Evaluation.
LIVER : Infiltration, mixed cell; minimal
LYMPH NODE, MANDIBULAR : Plasmacytosis; mild
NERVE, SCIATIC : Degeneration; axonal, minimal
NERVE, TIBIAL : Degeneration; axonal, minimal
SITE, INJECTION : Infiltration, mononuclear cell; minimal, leptomeninges
NO CORRELATE : No correlating lesion [GLAND, PITUITARY : Enlargement (G)]
NERVE ROOT, DORSAL, LUMBAR : Degeneration; axonal, minimal

Histopathology - The following Tissues were Within Normal Limits:

BRAIN; CERVIX; EYE; GANGLION, DORSAL ROOT, CERVICAL; GANGLION, DORSAL ROOT, LUMBAR; GANGLION, DORSAL ROOT, THORACIC; GLAND, ADRENAL; GLAND, PARATHYROID; GLAND, PITUITARY; GLAND, THYROID; HEART; KIDNEY; LUNG; LYMPH NODE, CERVICAL; LYMPH NODE, ILIAC; LYMPH NODE, MESENTERIC; MUSCLE, BICEPS FEMORIS; MUSCLE, GASTROCNEMIUS; NERVE, OPTIC; OVARY; SPINAL CORD, CERVICAL; SPINAL CORD, LUMBAR; SPINAL CORD, THORACIC; SPLEEN; THYMUS; UTERUS; NERVE ROOT, VENTRAL, CERVICAL; NERVE ROOT, VENTRAL, THORACIC; NERVE ROOT, VENTRAL, LUMBAR; NERVE ROOT, DORSAL, CERVICAL; NERVE ROOT, DORSAL, THORACIC

Histopathology - The following Tissues were Not Examined:

None

SIGNATURE(S) FOR DOCUMENT: 5550008 - 5550008 Pathology Final Report Path report

**QA Approval:**	I approve the Quality Assurance Statement for this report.
Name:	**Mendez Santos, Joshua**
Mendez Santos, Joshua	14-Jan-2022 13:28:18 (UTC+00:00)
Electronically Signed in M-Files	Timestamp

**Principal Investigator:**	I approve this document.
Name:	**O'Brien, Maureen**
O'Brien, Maureen	14-Jan-2022 14:06:15 (UTC+00:00)
Electronically Signed in M-Files	Timestamp

Appendix 19

charles river

PATHOLOGY PEER REVIEW CERTIFICATE

Test Facility Study No. 5550008

Sponsor Reference No. UTSW.Gray-003

A Single-Dose Toxicity Study of AAV9/AP4M1 by Intrathecal Injection in Rats

1. PURPOSE

The purpose of this pathology peer review was to assess the overall quality and consistency of the microscopic data and determine the validity of the study pathologist's conclusions. A pathology peer review for this study was performed as follows for this study:

2. METHODS

1. Review of protocol and amendments, pathology report, histology records, clinical observations, organ weight data, clinical pathology data.
2. Review of the DRG, nerves, and spinal cord from all animals in all groups to verify the effect levels.

3. RESULTS

Any differences of opinion were resolved and agreement on terminology and diagnoses was achieved. The histopathology tables and corresponding narrative in the pathology report reflect the diagnoses and conclusions agreed to by the peer review pathologist.

Melissa M. Schutten, DVM, PhD, DACVP
Peer Review Pathologist

SIGNATURE(S) FOR DOCUMENT: 5550008 General Toxicology Final Report

**QA Approval:**	I approve the Quality Assurance Statement for this report.
Name:	**Huang, Bo**
Huang, Bo	10-Mar-2022 14:14:14 (UTC+00:00)
Electronically Signed in M-Files	Timestamp

**Study Director Approval:**	I approve this Report.
Name:	**Cinquino, Stefania**
Cinquino, Stefania	10-Mar-2022 14:22:24 (UTC+00:00)
Electronically Signed in M-Files	Timestamp
