# Supplementary material for: Intrathecal AAV9/AP4M1 gene therapy for hereditary spastic paraplegia 50 shows safety and efficacy in preclinical studies
Source: J Clin Invest. 2023 May 15;133(10):e164575. doi: 10.1172/JCI164575 (PMC10178841; doi:10.1172/JCI164575)
Supplement: Supplemental data set 1 [file jci-133-164575-s058.pdf]

REPORT

Study Title<br>A Single Dose Toxicity Study of AAV9/*AP4M1* Vectors Administered by Intrathecal Injection in WT C57BL/6J Mice	Study Number<br>Study #05
Test Article/Equipment or Process Name:<br>Melpida	Report Date<br>05/19/2021
Key Words<br>AAV9, AP4M1, intrathecal, toxicity, histology, in vivo, dose-ranging	
Distribution<br><Enter Text Here>	Contributors<br>Xin Chen, Thomas Dong, Yuhui Hu, Steven Gray

Post Approval:

Functional Area	Print Name	Signature	Date
Author	Xin Chen	Xinchen	5/19/21
Reviewer			
Reviewer			
Approval	Steven Gray	[signature]	12/8/2021
Approval			

CONTENTS

1. Introduction.......4
2. Materials and methods.......4
Study Design.......4
Table 1. Summary of the non-GLP cohorts.......4
3. Results and Discussion.......5
IT AAV9/*AP4M1* dose-dependently increased *hAP4M1opt* mRNA in all brain regions. 5
IT AAV9/*AP4M1* caused no elevation of serum toxicity panel in most of the WT mice.......8
IT AAV9/*AP4M1* caused no effects on body weight in male or female mice.......10
IT AAV9/*AP4M1* caused no death in WT mice.......11
IT AAV9/*AP4M1* caused no clinical signs or adverse events in WT mice.......11
Histopathology.......11
4. Conclusions.......12
5. Appendix.......12
UNC-VC Vector Certificate of Analysis.......12
Summary Report- SPG50 1-month post injection (21-016) February 9, 2021.......15
Summary Report- SPG50 5-month post injection (21-017) February 10, 2021.......15
Summary Report- SPG50 12-months post injection (21-004) February 5, 2021.......16
Table 2. Individual body weight (g).......19

List of Figures

Figure 1. AAV9/*AP4M1* dose-dependently increased *hAP4M1opt* mRNA in brain regions of WT mice at 1-month post injection. 5
Figure 2. AAV9/*AP4M1* dose-dependently increased *hAP4M1opt* mRNA in brain regions of WT mice at 5-month post injection.......6
Figure 3. AAV9/*AP4M1* dose-dependently increased *hAP4M1opt* mRNA in brain regions of WT mice at 12-month post injection. 7
Figure 4. AAV9/*AP4M1* caused no elevation of serum toxicity panel 1-month post injection in all mice except 1 male mouse which received low dose. 9
Figure 5. AAV9/*AP4M1* caused no elevation of serum toxicity panel 5-months post injection in all mice except 1 female mouse which received vehicle. 9
Figure 6. AAV9/*AP4M1* caused no elevation of serum toxicity panel 12-months post injection in all mice except 2 male mice which received high dose. 10
Figure 7. AAV9/*AP4M1* caused no effects on body weight in male or female mice.......10
Figure 8. AAV9/*AP4M1* dose-dependently increased *hAP4M1opt* mRNA in livers of WT mice at 12-month post injection.......11

List of Tables

Table 1. Summary of the non-GLP cohorts.......4
Table 2. Individual body weight (g).......19

ABBREVIATIONS

AAV9	adeno-associated virus serotype 9
ALB	Albumin
AP4M1	adaptor-related protein complex AP-4, μ4
AST	Aspartate transaminase
BUN	Blood urea nitrogen
CK	Creatine kinase
GLP	Good Laboratory Practice
IACUC	Institutional Animal Care and Use Committee
IT	Intrathecal
SPG50	Spastic paraplegia 50
TBIL	Total bilirubin
UNC-VC	University of North Carolina – Vector Core
UTSW	University of Texas Southwestern
vg	vector genome
WT	Wild-type

1. INTRODUCTION

The objective of this study was to characterize the toxicity and gene expression of scAAV9/UsP-*hAP4M1opt*-BGHpA (AAV9/*AP4M1* or Melpida) following a single intrathecal (IT) injection in wild type (WT) C57BL/6J mice. The AAV9/*AP4M1* is being developed for treatment of Spastic Paraplegia 50 (SPG50).

2. MATERIALS AND METHODS

Study Design

WT C57BL/6J mice from Jackson Laboratories were assigned to the study as indicated in Table 1.

Table 1. Summary of the non-GLP cohorts

Group	Route*	Age (weeks)	Body weight (g)**		Dose (vg/mouse)	Vector manufacturer	Endpoint (12 months post injection)
			Male (n=10)	Female (n=10)			
1	IT	7	19.9±0.4	15.7±0.4	Vehicle	UNC-VC	Body weights, Clinical signs, Adverse events, Mortality, and Histopathology
2			21.3±0.5	15.7±0.4	1.25×10^{11} (Low)		
3			21.5±0.3	15.3±0.4	5×10^{11} (High)		

GLP, Good Laboratory Practice; IT, intrathecal; UNC-VC, University of North Carolina – Vector Core; vg, vector genome
*IT injections were via lumbar puncture, a 5 µL dose in vehicle (350mM phosphate-buffered saline, 5% sorbitol).
**Mean ± standard error

The non-GLP studies presented in Table 1 were designed to identify any long-term safety issues of the experimental therapy. The mice were randomized to different groups and injected IT with 5 µL of vehicle or different doses of AAV9/*AP4M1* vectors. The AAV9/*AP4M1* vectors were made by UNC-VC (University of North Carolina - Vector Core). Sodium dodecyl sulfate–polyacrylamide gel electrophoresis and silver stain analysis showed no visible contaminating protein in the UNC-VC lot. Scanning Electron Microscope showed 88% full capsid in this product (see appendix).

Mice were monitored for changes in body weight, clinical signs, adverse events, and mortality following the treatment. All mice were weighed weekly for the first month and then monthly thereafter. Any clinical signs or adverse events including neurological symptoms were investigated, evaluated, and recorded. Appropriate supportive or therapeutic interventions were offered per Institutional Animal Care and Use Committee (IACUC) and veterinary guidance. 1-month post injection, 6 mice (3 males and 3 females) from each group and 5-month post injection, 4 mice (2 males and 2 females) from each group were euthanized. All mouse brains were used for *AP4M1* mRNA expression by RNAscope and mouse serum were used to check serum toxicity panel including Aspartate transaminase (AST), Total bilirubin (TBIL), Albumin (ALB), Creatine Kinase (CK), and Blood Urea Nitrogen (BUN). Blood and tissue samples were collected from mice that are euthanized for humane reasons. Where possible, a detailed necropsy was performed to investigate or identify the reason for the ailment by a trained technician or veterinary staff. Terminal serum and tissue samples at 12 months following the treatment were collected for serum toxicity panel and histopathological assessment, respectively. The histopathological evaluations on collected tissue samples were performed and reported by Dr. Mary Wight-Carter, DVM, DACVP, Veterinary Pathologist at Animal Research Center, University of Texas Southwestern (UTSW) Medical Center.

Quantitative data were tested for normal distribution (Shapiro-Wilk normality test) and homogeneity of variance (Brown-Forsythe test). Data sets that passed these two tests were analyzed using one-way ANOVA

with α set at 0.05 with Holm-Sidak correction for multiple comparisons. Data sets that did not pass tests for normality or homogeneity of variance were analyzed using Kruskal-Wallis test with α set at 0.05. All pair-wise comparisons were made, with Dunn's correction for multiple comparisons.

3. RESULTS AND DISCUSSION

IT AAV9/*AP4M1* dose-dependently increased *hAP4M1opt* mRNA in all brain regions.

Animals receiving AAV9/*AP4M1* had detectable levels of *hAP4M1opt* mRNA in all brain regions assessed (Figure 1-3). While the low dose animals had detectable levels of mRNA, which were not significantly higher than the controls, the high dose group had significantly higher mRNA levels than control animals. Moreover, *hAP4M1opt* mRNA expression sustained up to 12-month post injection (Figure 1-3).

Either high (5×10^{11} vg/mouse) or low (1.25×10^{11} vg/mouse) dose of AAV9/*AP4M1* vector was administered IT to WT mice at the age of 7 weeks old. At 1-month (Figure 1), 5-months (Figure 2), and 12-months (Figure 3) post injection, mouse brains were harvested for RNAscope staining to detect *hAP4M1opt* mRNA. Histology images were digitized with a ScanScope slide scanner and analyzed using custom analysis settings in HALO™ Image Analysis Platform (A, scale bar, 100μm). Results in panel B were presented as % area staining positive for *hAP4M1opt* mRNA by tissue region (mean ± SEM, n=6/group, n=4/group and n=10/group for Figure 1, 2, and 3, respectively). ***P<0.001, **p<0.01, and *p<0.05 compared to mice treated with vehicle.

Figure 1. AAV9/*AP4M1* dose-dependently increased *hAP4M1opt* mRNA in brain regions of WT mice at 1-month post injection.

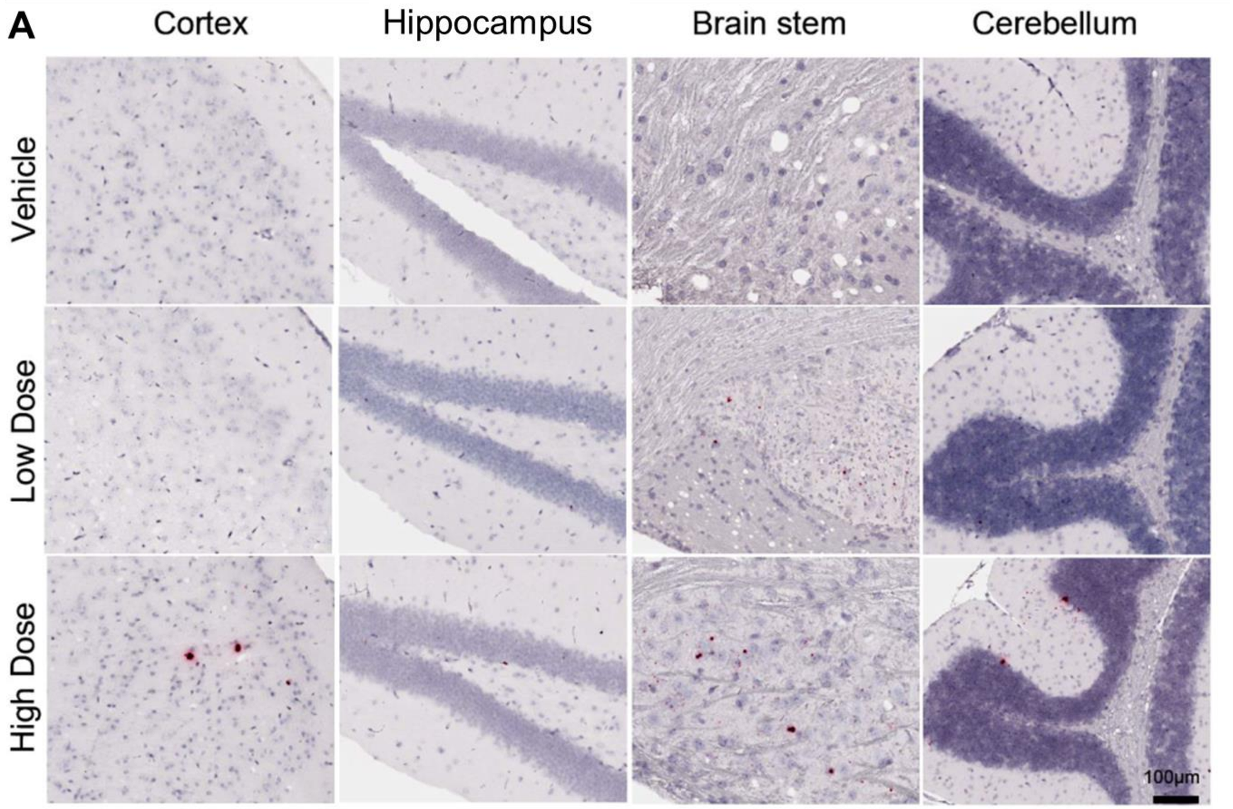

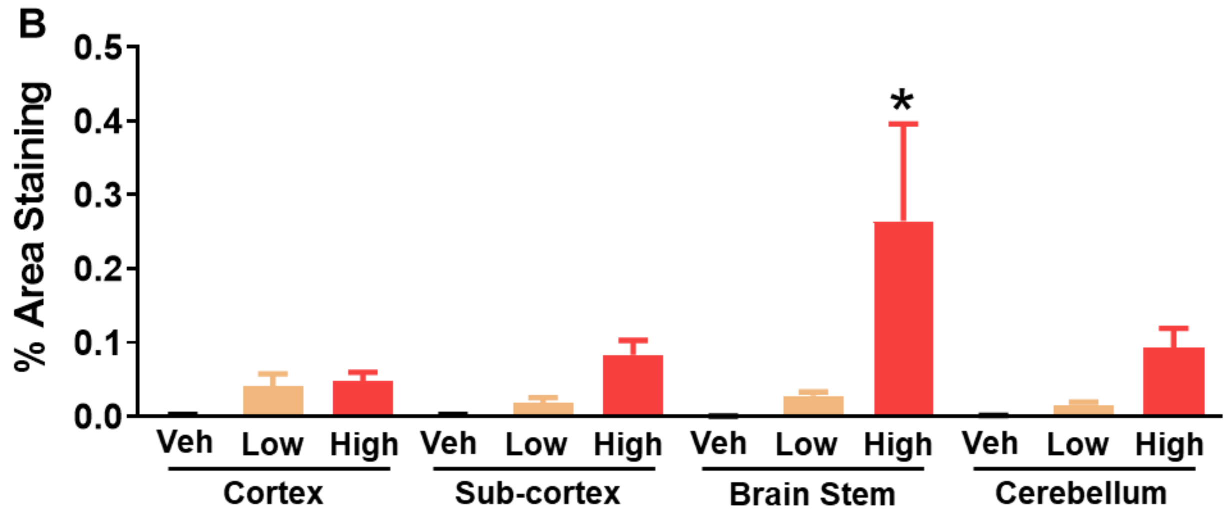

Figure 2. AAV9/*AP4M1* dose-dependently increased *hAP4M1opt* mRNA in brain regions of WT mice at 5-month post injection.

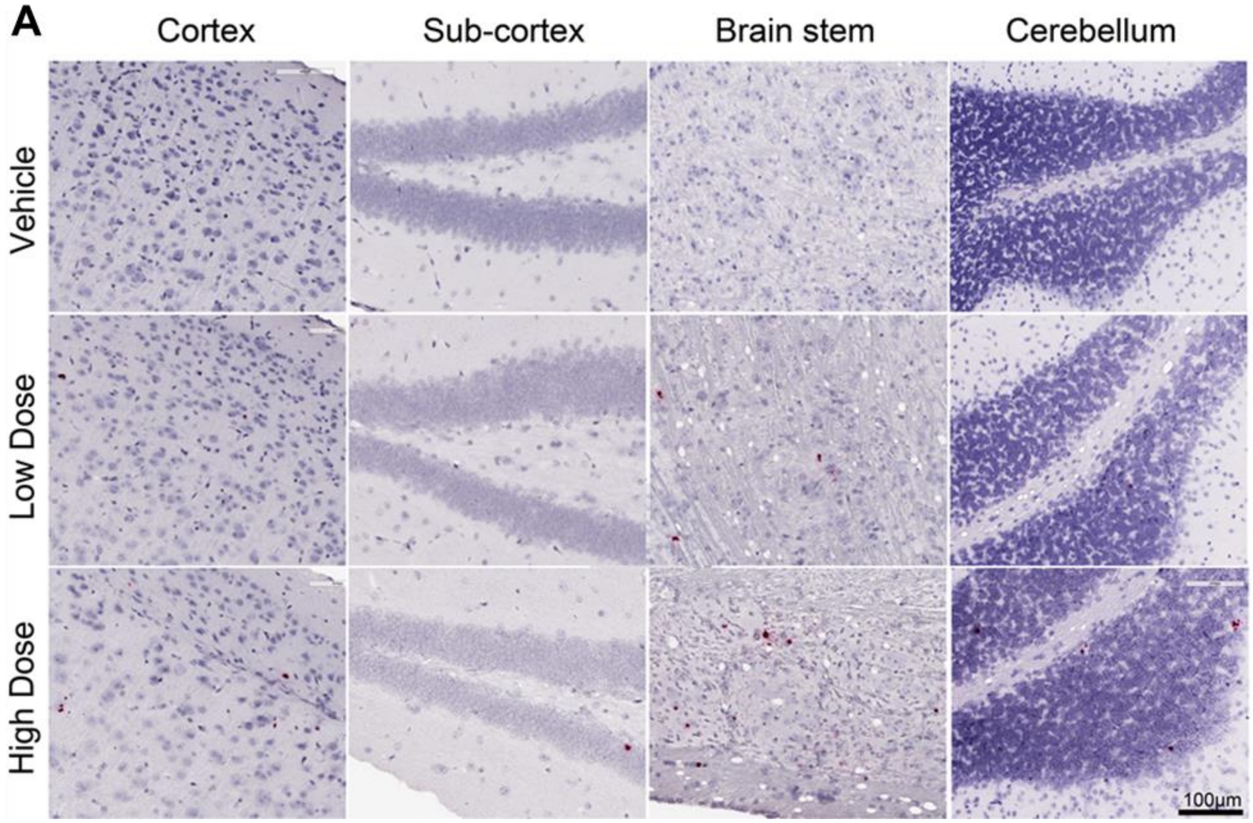

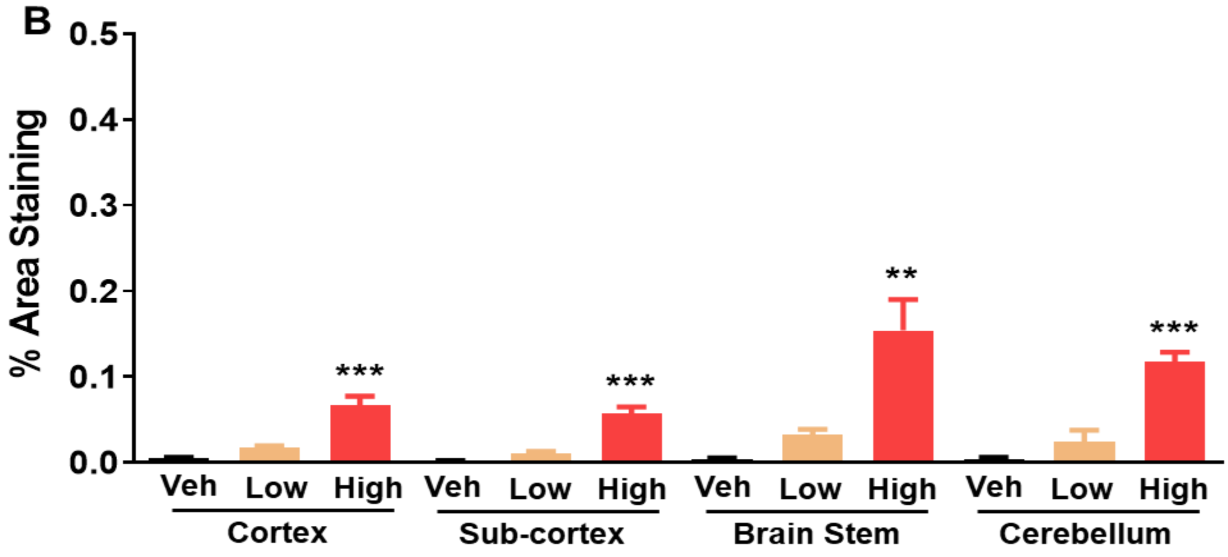

Figure 3. **AAV9/*AP4M1* dose-dependently increased *hAP4M1opt* mRNA in brain regions of WT mice at 12-month post injection.**

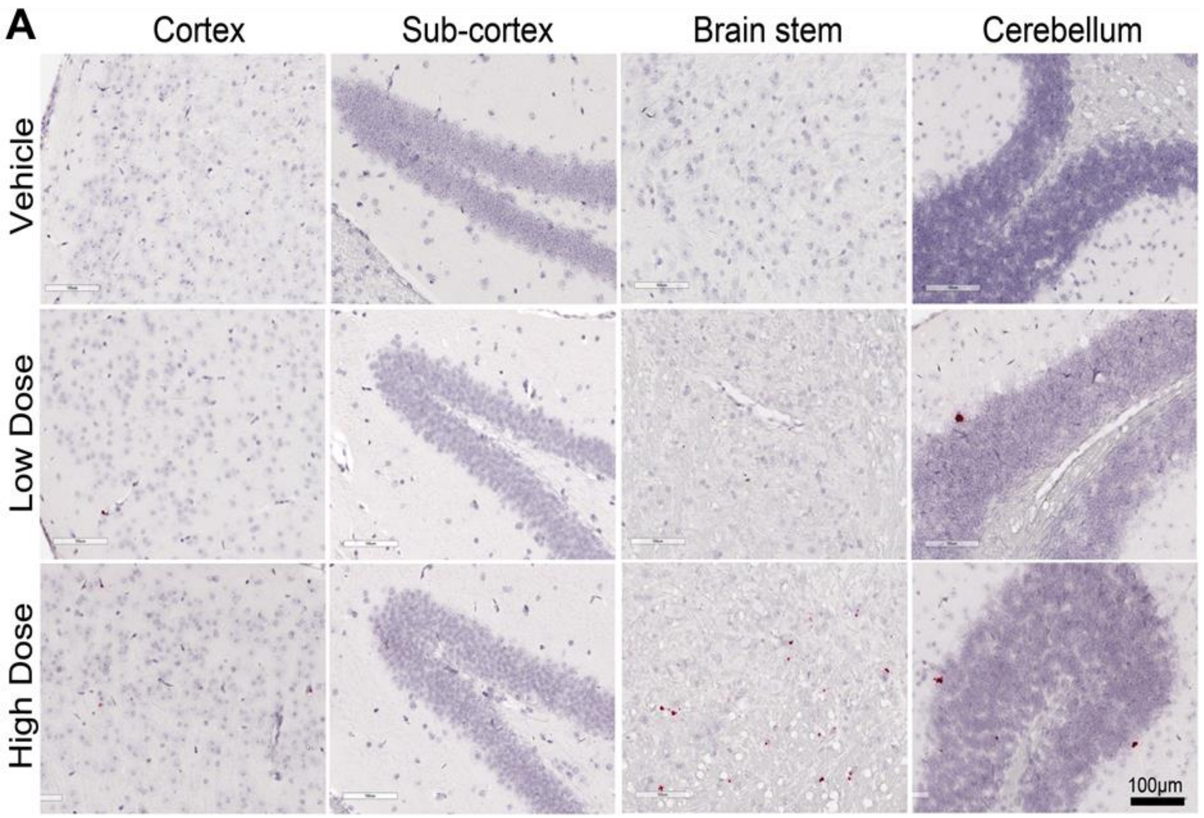

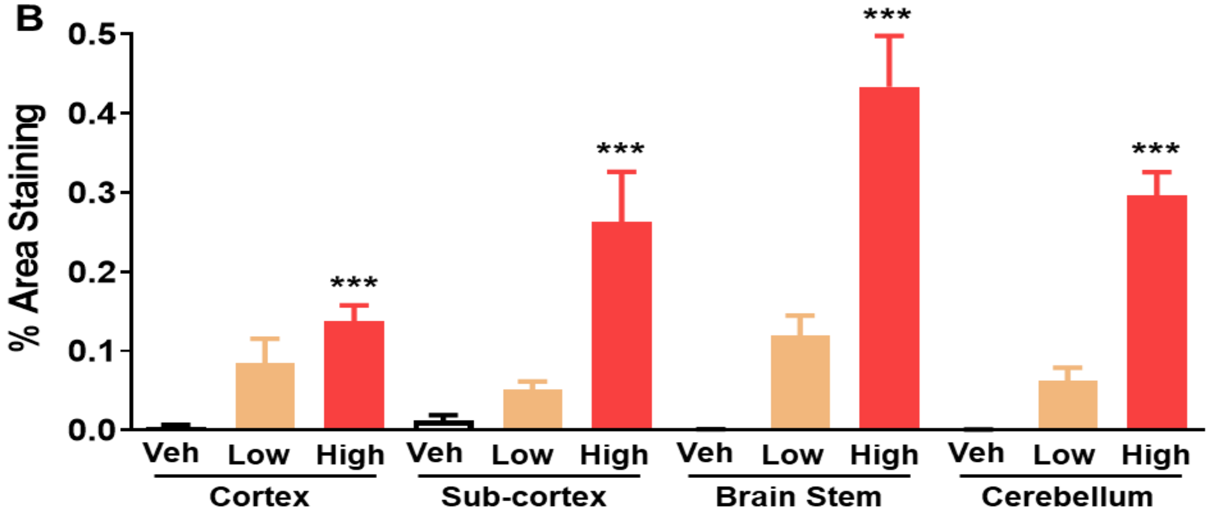

IT AAV9/*AP4M1* caused no elevation of serum toxicity panel in most of the WT mice

Animals receiving AAV9/*AP4M1* had normal levels of serum toxicity panel including AST, TBIL, ALB, CK, and BUN 1-month post injection in all except 1 male mouse (Figure 4). In this male mouse which received low dose 1.25×10^{11} vg/mouse, AST and TBIL reached 293 U/L and 0.9 mg/dL, respectively. At 5-month post injection (Figure 5), serum toxicity panel were normal in all except 1 female mouse. In this female mouse which received vehicle, AST and TBIL reached 180 U/L and 0.76 mg/dL, respectively. At 12-month post injection (Figure 6), serum toxicity panel were also normal in all except 2 male mice which received high dose 5×10^{11} vg/mouse. In one male mouse, AST, TBIL, and BUN reached 187 U/L, 0.72 mg/dL, and 124 mg/dL, respectively. In the other male mouse, AST reached 283 U/L. In conclusion, our results suggest that most of the WT mice tolerate AAV9/*AP4M*1 well, however there is an occasional toxicity to the liver and kidney across groups.

Either high (5×10^{11} vg/mouse) or low (1.25×10^{11} vg/mouse) dose of AAV9/*AP4M1* vector was administered IT to WT mice at the age of 7 weeks old. At 1-month (Figure 4), 5-months (Figure 5), and 12-months (Figure 6) post injection, mouse serum was collected for serum chemistry. Results are presented as mean ± SEM, n=6/group, n=4/group and n=10/group for Figure 4, 5, and 6, respectively. No significant difference was found between any groups.

Figure 4. **AAV9/*AP4M1* caused no elevation of serum toxicity panel 1-month post injection in all mice except 1 male mouse which received low dose.**

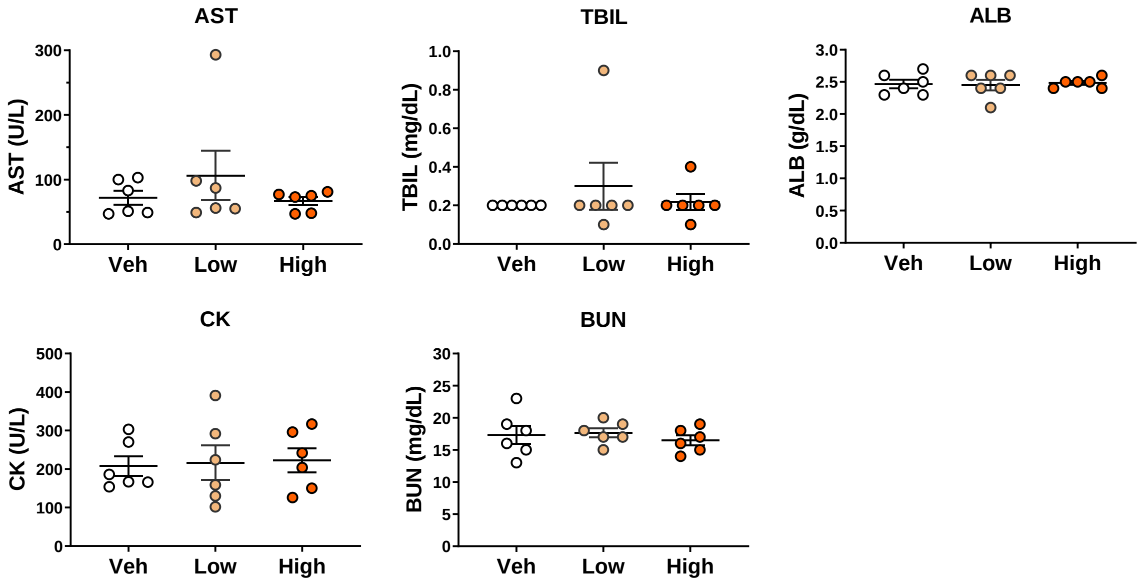

Figure 5. **AAV9/*AP4M1* caused no elevation of serum toxicity panel 5-months post injection in all mice except 1 female mouse which received vehicle.**

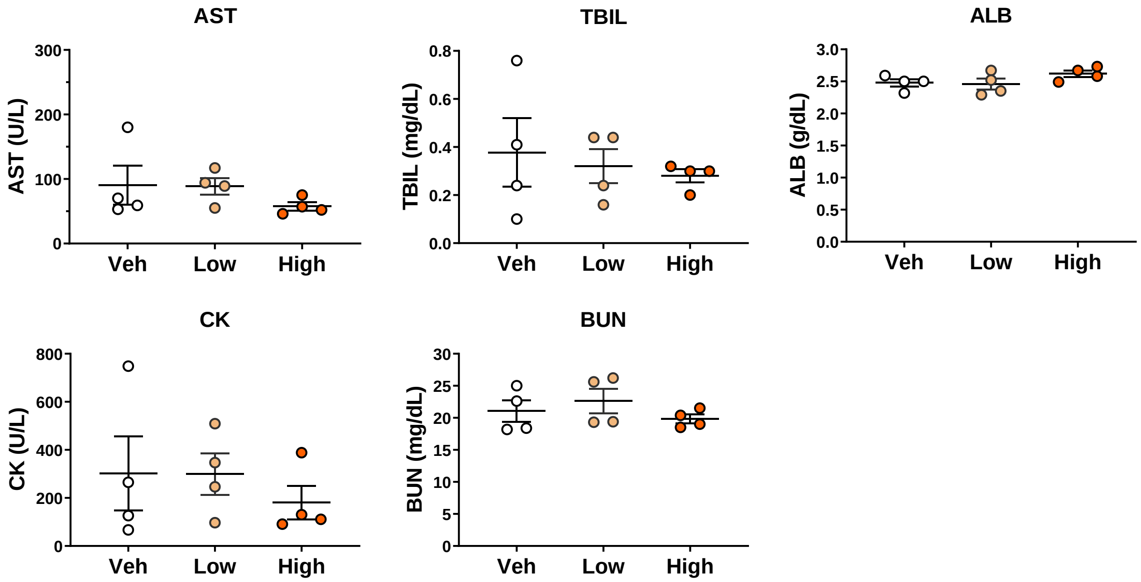

Figure 6. **AAV9/*AP4M1* caused no elevation of serum toxicity panel 12-months post injection in all mice except 2 male mice which received high dose.**

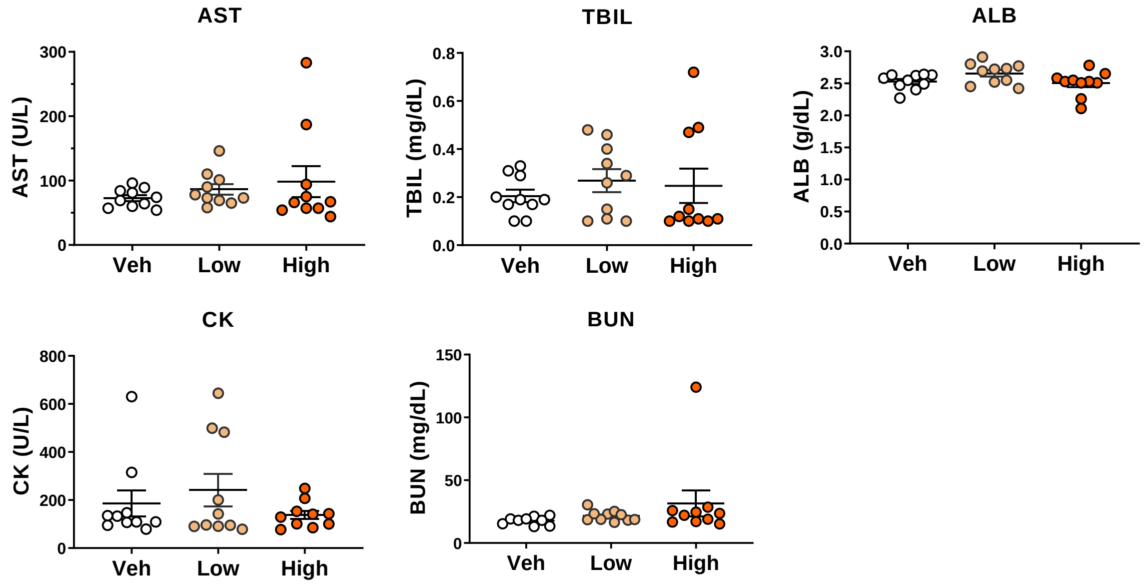

IT AAV9/*AP4M1* caused no effects on body weight in male or female mice

Body weight was monitored to assess the overall health of the animals. There was no significant difference in body weight between groups within male or female mice at any point of assessment (Figure 7 and Table 2), suggesting that doses up to 5×10^{11} vg/mouse were well tolerated in the WT C57BL/6J mice up to 12 months following the treatment.

Either high (5×10^{11} vg/mouse) or low (1.25×10^{11} vg/mouse) dose of AAV9/*AP4M1* vector was administered IT to WT mice at the age of 7 weeks old. Mice were weighed weekly for the first month following the treatment and then monthly thereafter.

Figure 7. **AAV9/*AP4M1* caused no effects on body weight in male or female mice.**

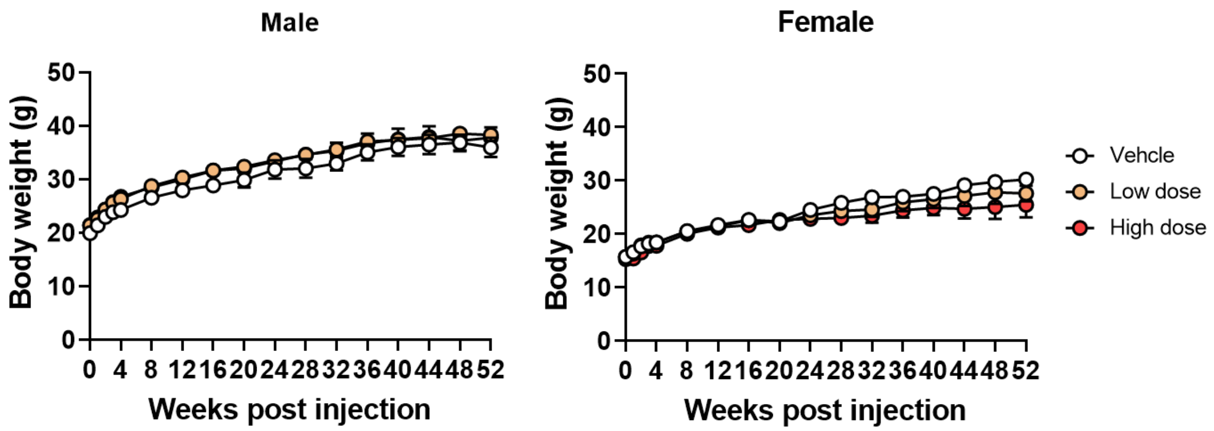

IT AAV9/*AP4M1* caused no death in WT mice

There were no obvious clinical signs of morbidity in the adult WT mice dosed with AAV9/*AP4M1* at doses up to 5×10^{11} vg/mouse (Table 1). There was no unexpected death in this study over 1-year post injection of AAV9/*AP4M1*. All mice survived to the end of the experiment, further indicating that doses up to 5×10^{11} vg/mouse were well tolerated in WT C57BL/6J mice up to 12 months following the treatment.

IT AAV9/*AP4M1* caused no clinical signs or adverse events in WT mice

No outward signs of toxicity were noted over the duration of the study.

Histopathology

At 1-month, 5-months, and 12-months post injection, 6, 4, and 10 mice/group were anesthetized, respectively and perfused with phosphate-buffered saline containing 1 U/mL heparin and main tissues/organs were harvested. No obvious abnormalities were noticed except for some granular appearance in the liver of three mice (2 males and 1 female) which were treated with high dose at 12-month post injection. All tissues/organs collected were fixed in 10% formalin for 24 hours and then transferred to 70% ethanol. Animals receiving AAV9/*AP4M1* had detectable levels of *hAP4M1opt* mRNA in livers (Figure 8). While the low dose animals had no obvious effect on liver morphology, the high dose group had more abnormal morphology than control animals. The histopathological evaluations on all collected tissue samples were performed and reported by Dr. Mary Wight-Carter, DVM, DACVP, Veterinary Pathologist (Appendix for Summary Reports- SPG50). Dr. Wight-Carter concluded that 1) all microscopic changes present in the mice harvested at 1-month and 5-months post injection were considered variations on normal microanatomy for mice this age, and 2) the tumors and increased number of inflammatory cell infiltrates and degenerative lesions seen in the mice harvested at 12-months post injection were expected in mice as they age.

Figure 8. AAV9/*AP4M1* dose-dependently increased *hAP4M1opt* mRNA in livers of WT mice at 12-month post injection.

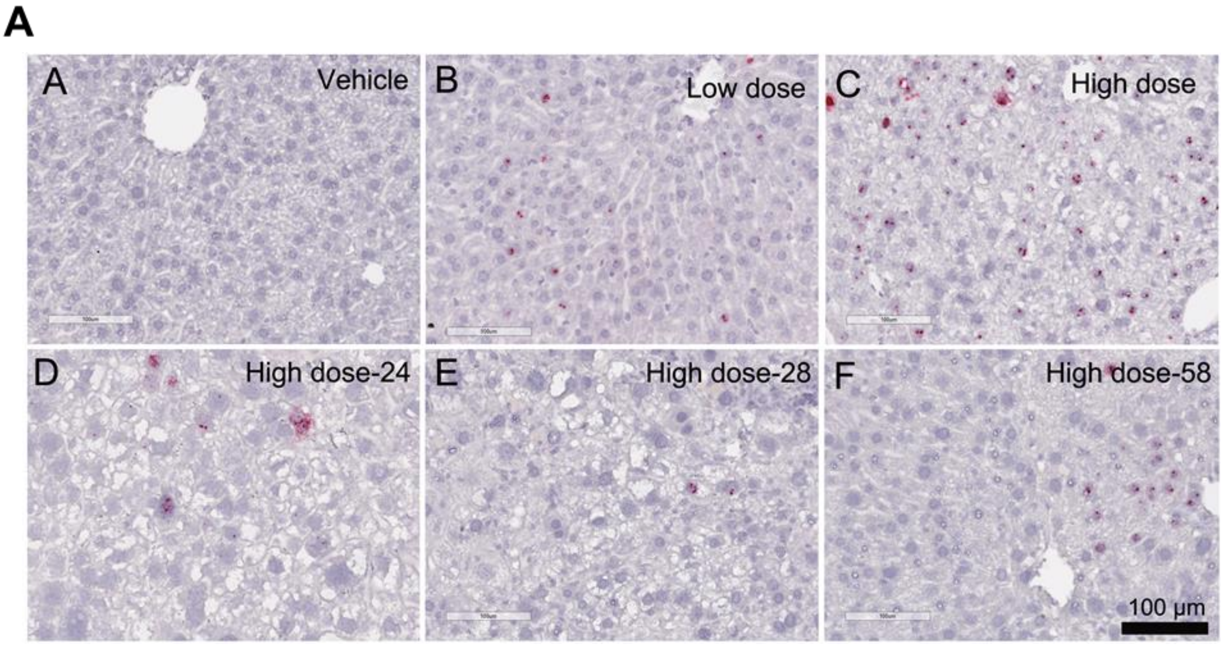

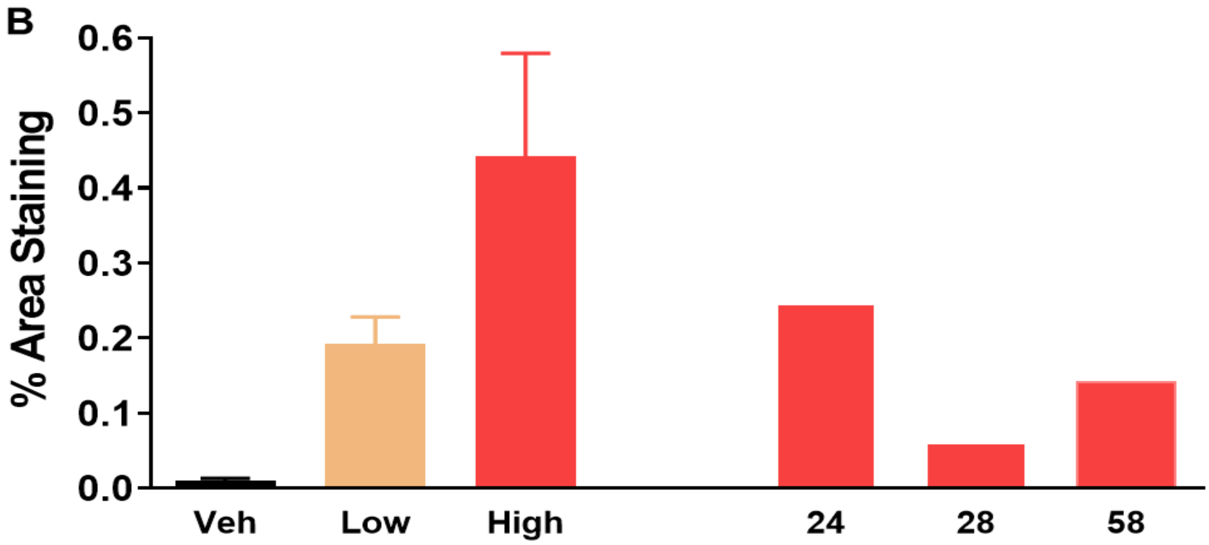

4. CONCLUSIONS

IT AAV9/*AP4M1* doses up to 5×10^{11} vg/mouse was safe and well tolerated in WT mice in general. There was some potential toxicity. For example, 2 male mice which received 5×10^{11} vg/mouse had abnormal liver tox panel at 12-months post injection. The same 2 male (20%) mice plus 1 female (10%) mouse had granular appearance in the liver during necropsy, which was diagnosed as hepatocellular adenomas by microscopic examination. It is important to mention that the hepatocellular adenomas are expected in mice as they age, for example NTP Historical Controls_B6 reported that the incident rate of hepatocellular adenomas reaches as high as 51% in males and 18% in females.

5. APPENDIX

UNC-VC Vector Certificate of Analysis

UNIVERSITY OF NORTH CAROLINA VECTOR CORE

Quality Control Summary

**Lot #**	**LAV112-conc**	**Name**	**AP4M1**

Test by qPCR

Test #	Titer, vg/mL	Analyst	Date	File
1	1.03E+14	PZ	11/21/2019	20191121-1007-ghbh-pz

PAGE analysis

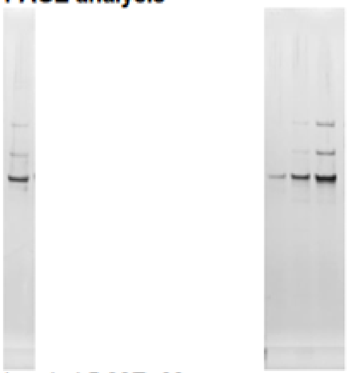

Loaded 5.00E+09 vg 4980E std 2e9vg 5e9vg 1e10vg
Calculated 5.20E+09 vg

Analyst	Ping Zhang
Date	11/19/2019
Reference #	20191119-silver

UNIVERSITY OF NORTH CAROLINA
VECTOR CORE

SEM

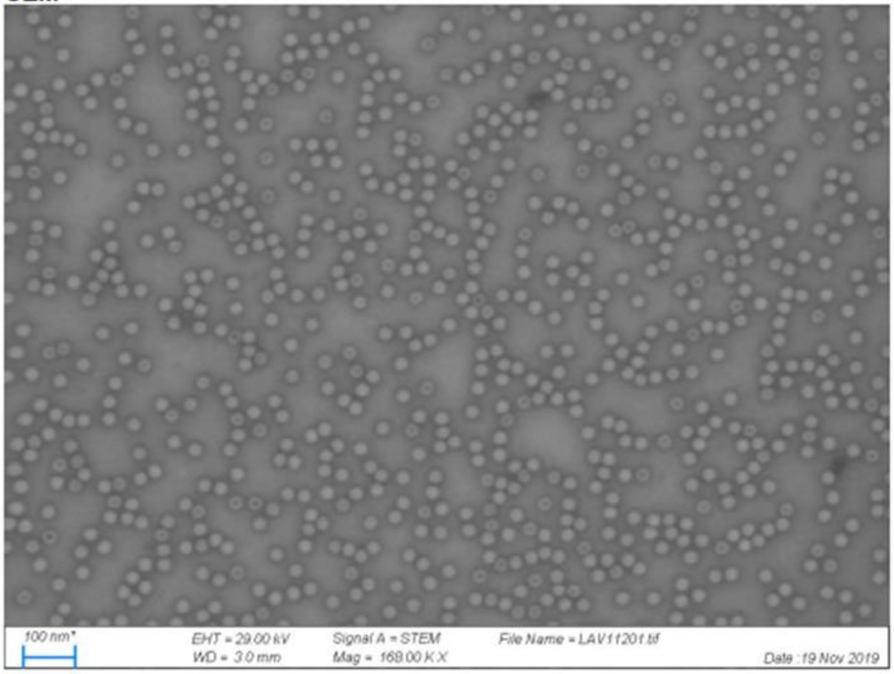

88% full

Analyst	Ping Zhang
Date	11/19/2019
Reference #	20191119-LAV112-02

Summary Report- SPG50 1-month post injection (21-016) February 9, 2021

Tissues were fixed in 10% neutral buffered formalin for 1 day, stored in 70% ethanol, trimmed into tissue cassettes and sent for processing to IDEXX laboratories. Hematoxylin and Eosin stained slides were produced from the cassettes. Tissues and the corresponding slides were labeled with the following ID's: WT-3, WT-6, WT-7, WT- 13, WT-16, WT-17, WT-23, WT-26, WT-27, WT-33, WT-36, WT-37, WT- 43, WT-47, WT-53, WT-56, and WT-57. Brain, heart, liver, lung, gonad, spleen, kidney, and spinal cord were submitted for all animals except for the following instances. There was no ovary present for WT-37 and WT-56. They both had small sections of uterus or oviduct, which were microscopically normal. There was no gonad or section of reproductive tract present for WT-6. The spleen was not present for WT-47. The cerebrum, cerebellum and olfactory bulb were all microscopically normal. There were no abnormalities found in the spinal cord of all mice.

The testes for WT-3, WT-7, WT- 13, WT-16, WT-17, WT-23, WT-26, and WT-27 were microscopically normal. Ovaries were present for WT-33, WT-36, WT-43, WT-47, WT-46, WT-53, and WT-57. All of the ovaries that were present were normal and the structures within the ovaries were consistent with various points in the estrus cycle.

The heart and lung were present for all the animals. These tissues were normal in all instances.

The kidneys of mouse ID WT-3, WT-6, WT-7, WT-13, WT-16, WT-17, WT-23, WT-26, WT-27, WT-37, WT-43, WT-46, WT-47, WT-53, WT-56, and WT-57 contained no abnormalities. The kidney of WT-33 had a single epithelial lined cyst. Single cysts may occur occasionally in mice as a congenital lesion. The renal medulla had mild dilation of a few tubules in kidney of WT-36. The tubule dilation seen in these kidneys was mild simple dilation and was most likely due to a focal cellular cast. This lesion is not uncommon in mice.

The livers of mouse ID WT-6, WT-7, WT-13, WT-16, WT-23, WT-36, WT-37, and WT-43 contained no microscopic abnormalities. Animal numbers WT-3, WT-26, WT-27, WT- 33, WT-46, WT-47, WT-53, WT-56, and WT57 had multifocal areas of extramedullary hematopoiesis present in the liver parenchyma. This is a common finding in mice of this age. The liver of WT-17 contained multifocal mitotic figures within the hepatocytes. This is consistent with hepatocellular hyperplasia. There was no evidence of active hepatocellular degeneration as a cause for the hyperplasia and hyperplasia is considered a spontaneous event in a young mouse.

The spleen of all mice had variable amounts of extramedullary hematopoiesis. The spleens of mouse ID WT- 7 and WT- 16 contained a few scattered macrophages with intracytoplasmic hemosiderin. This is considered normal in mice. It occurs more frequently as mice age.

All microscopic changes present in these mice are considered variations on normal microanatomy for mice this age.

Mary Wight-Carter DVM, DACVP

Summary Report- SPG50 5-month post injection (21-017) February 10, 2021

Tissues were fixed in 10% neutral buffered formalin for 1 day, stored in 70% ethanol, trimmed into tissue cassettes and sent for processing to IDEXX laboratories. Hematoxylin and Eosin stained slides were produced from the cassettes. Tissues and the corresponding slides were labeled with the following ID's: WT-1, WT-2, WT-11, WT- 12, WT-21, WT-22, WT-31, WT-32, WT- 41, WT-42, WT-51, and WT-52. Brain, heart, liver, lung, gonad, spleen, kidney, and spinal cord were submitted for all animals.

The cerebrum, cerebellum and olfactory bulb were all microscopically normal. There were no abnormalities found in the spinal cord of all mice.

The testes for WT-1, WT-2, WT- 11, WT-12, WT-21, and WT-22 were microscopically normal. Ovaries were present for WT-31, WT-32, WT-41, WT-42, WT-51, and WT-52. All of the ovaries were normal and the structures within the ovaries were consistent with various points in the estrus cycle.

The heart and lung were present for all the animals. These tissues were normal in all instances except the lung of WT-31. There were a few perivascular infiltrates with small numbers of lymphocytes and plasma cells in the lungs of WT-31. These inflammatory infiltrates are commonly seen in the lungs of adult mice.

The kidneys of mouse ID WT-1, WT-2, WT-11, WT-12, WT-41, WT-42, WT-51 and WT-52 contained no abnormalities. The renal medulla had mild dilation of a few tubules in kidney of WT-32. The tubule dilation seen in this kidney was mild simple dilation and was most likely due to a focal cellular cast. This lesion is not uncommon in mice. There were focal perivascular infiltrates with small numbers of lymphocytes and plasma cells in kidneys of WT-21, WT-22 and WT-31. The inflammatory infiltrates are considered incidental findings as they occur occasionally in adult mice.

The livers of mouse ID WT-1, WT-11, WT-12, and WT-52 contained no microscopic abnormalities. Animal numbers WT-2, WT-21, WT-22, WT- 31, WT-32, WT-41, WT-42, and WT-51 had multifocal areas of extramedullary hematopoiesis present in the liver parenchyma. This is a common finding in mice of this age.

The spleen of all mice had variable amounts of extramedullary hematopoiesis. The spleens of mouse ID WT-1, WT-2, WT-11, WT-12, WT-21, WT-22, WT-31, WT-32, WT-41, WT-42, WT-51 and WT-52 contained a few scattered macrophages with intracytoplasmic hemosiderin. This is considered normal in adult mice.

All microscopic changes present in these mice are considered variations on normal microanatomy for mice this age.

Mary Wight-Carter DVM, DACVP

Summary Report- SPG50 12-months post injection (21-004) February 5, 2021

Tissues were fixed in 10% neutral buffered formalin for 1 day, stored in 70% ethanol, trimmed into tissue cassettes, and sent for processing to IDEXX laboratories. Hematoxylin and Eosin stained slides were produced from the cassettes. Tissues and the corresponding slides were labeled with the following ID's: WT-4, WT-5, WT-8, WT-9, WT- 10, WT-14, WT-15, WT-18, WT-19, WT-20, WT-24, WT-25, WT-28, WT-29, WT-30, WT-34, WT-35, WT-38, WT-39, WT-40, WT-44, WT-45, WT-48, WT-49, WT-50, WT-54, WT-55, WT-58, WT-59, and WT-60. Brain, heart, triceps muscle, liver, lung, gonad, spleen, kidney, sciatic nerve, cervical and lumbar spinal cord were submitted for all animals except for the following instances. There was no ovary present for WT-38 and WT-45. They both had small sections of uterus or oviduct, which were microscopically normal. There was no gonad or section of reproductive tract present for WT-35, WT-39, WT- 50, and WT-55. The sciatic nerve was not present for animal ID WT-18, WT-19 WT-38, WT-39, WT-44, WT-48, WT-49, WT-50, and WT-59. The triceps muscle was not present for animal ID WT-9 and WT-60. The brain was not present for WT-4.

The cerebrum, cerebellum and olfactory bulb were all microscopically normal. There were no abnormalities found in the cervical and lumbar cord of all mice.

The testes for WT-4, WT-5, WT-8, WT-9, WT-10, WT-14, WT-15 WT-18, WT-19, WT-20, WT-25, WT-28, and WT-29 were microscopically normal. A few seminiferous tubules of animal ID WT-30 had multiple variably sized vacuoles that replaced various levels of the seminiferous epithelium. There was no evidence of accompanying germ cell degeneration. Since there were a very few tubules affected and there was no accompanying degeneration, it suggests that this was an incidental finding. Animal ID WT-24 had a few mineralized seminiferous tubules. This dystrophic mineralization occurs where there is focal degeneration. Testicular degeneration occurs in approximately 20% of aged male mice and these lesions were most likely early signs of testicular degeneration in this mouse.

Ovaries were present for WT-34, WT-44, WT-48, WT-49, WT-54, WT-58, and WT-59. All of the ovaries that were present were normal and the structures within the ovaries were consistent with various points in the estrus cycle.

The heart of animal ID WT-20 had two myofibers with mineralization. This can be found sporadically in aged mice. The heart of WT-54 had a medium sized artery at the base of the heart had intramural infiltrates with small numbers of neutrophils, lymphocytes, and plasma cells. This inflammatory reaction can be seen in adult mice due to injury of the arteries from regular physiologic stresses. It is considered an incidental finding. The hearts and triceps muscles were microscopically normal in the remainder of the animals.

The kidneys of mouse ID WT-4, WT-14, WT-24, WT-49, WT-59, and WT-60 contained no abnormalities. The renal medulla had mild to moderate dilation of a few tubules of kidneys of WT-18, WT-29, WT- 34, WT- 35, WT- 38, WT-40, WT- 45, WT-48, WT-54, and WT-55. The tubule dilation seen in these kidneys was mild simple dilation and was most likely due to a focal cellular cast. This lesion is not uncommon in mice. There was mild nephritis composed of interstitial fibrosis, tubular degeneration, and infiltrates with small to moderate numbers of lymphocytes in animal ID WT-5, WT-8, WT-9, WT-10, WT-15, WT-18, WT-20, and WT 29. There were mild perivascular infiltrates with small to moderate numbers of lymphocytes and plasma cells in kidneys of WT-19, WT- 25, WT-28, WT-30, WT-35, WT-39, WT-40, WT-44, and WT-50. The kidney of WT-58 had a few mineralized tubules, which mildly dilated the tubules. The above-described lesions are considered incidental findings as they occur occasionally in adult or aged mice and typically are more frequent in male mice.

The livers of mouse ID WT-4, WT-8, WT-9, WT-14, WT-15, WT-19, WT-25, WT-29, WT-34, and WT-50 contained no microscopic abnormalities. The liver of WT-9 and WT-45 contained mild perivascular infiltrates with lymphocytes and plasma cells with no corresponding fibrosis or hepatocellular necrosis. Mild perivascular infiltrates are a common finding in mice and increase in incidence as the mice age. Liver from WT-39 contained clusters of lymphoid hyperplasia. This was most likely a physiologic response to gut related immune stimulation. This is considered an incidental finding. The livers of WT-30, WT-38, WT-40, WT-44, WT-45, WT-48, and WT-59 contained multifocal infiltrates with small numbers of mixed inflammatory cell infiltrates with hepatocellular necrosis (micro-abscess). Areas with 1-2 cell hepatocyte necrosis accompanied by inflammatory cells can occur spontaneously in the mouse liver with increased incidence as the mice age. Animal numbers WT-5, WT-10 WT-35, WT-49, WT-54, and WT-60 had multifocal areas of extramedullary hematopoiesis present in the liver parenchyma. This is less common in rodents as they age and typically occurs in response to increased hematopoietic demand. Multifocal hepatocytes throughout the livers from WT-18, WT-20, and WT-55 had round variably sized intracytoplasmic vacuoles that are morphologically consistent with lipidosis. The lipidosis was mild and can occur as a normal finding depending on the metabolic status of the mouse. The livers of mouse ID WT-24, WT-28, and WT-58 contained mild compression of normal parenchyma by hepatocellular adenomas. These tumors occur spontaneously in mice with an increased frequency in aged mice.

The lungs of mouse ID WT-4, WT-5, WT-8, WT-10, WT-14, WT-15, WT-18, WT-19, WT-20, WT-24, WT-25, WT-28, WT-29, WT-30 WT-34, WT-45, WT-48, WT-49, WT-50, WT-55, WT-58, and WT-59

contained no abnormalities. The lungs from the following mice had mild to moderate perivascular infiltrates with lymphocytes and plasma cells: WT-35, WT-38, WT-39, WT-40, WT-44, and WT-54. The lungs from WT-9, WT-54, and WT-60 contained mild peribronchiolar infiltrates with lymphocytes and plasma cells. These inflammatory infiltrates are commonly seen in the lungs of adult mice. Mild interstitial pneumonia was present in the lungs of mouse ID WT-54. This can occasionally occur in older mice due to aspiration of excessive amounts of dust or other materials from their environment.

The spleen of all mice had variable amounts of extramedullary hematopoiesis and hemosiderin within the macrophages of the red pulp. This is considered normal in adult mice.

The sciatic nerves of mouse ID WT-4, WT-5, WT-8, WT-9, WT-15, WT-20, WT-24, WT-25, WT-28, WT-30, WT-45, WT-54, WT-55, and WT-60 contained no microscopic abnormalities. There were a few scattered mast cells present in the sciatic nerve of these mice: WT-10, WT-14, WT-29 WT-34, WT-35, and WT-58. This is an incidental finding in mice.

The tumors and increased number of inflammatory cell infiltrates and degenerative lesions seen in these mice are expected in mice as they age.

Mary Wight-Carter DVM, DACVP

Table 2. Individual body weight (g)

Mouse ID	Sex	IT	0 Week	1 Week	2 Weeks	3 Weeks	4 Weeks	8 Weeks	12 Weeks	16 Weeks	20 Weeks
WT-1	M	Vehicle	19.4	20.4	21.9	22.8	23.8	26.3	27.7	29.7	29.3
WT-2	M	Vehicle	18.2	20.1	21.6	22.9	24.0	25.5	25.7	26.0	26.3
WT-3	M	Vehicle	20.6	22.9	24.7	26.2	26.4				
WT-4	M	Vehicle	17.4	19.6	21.8	22.5	22.9	24.8	25.2	25.9	26.4
WT-5	M	Vehicle	19.7	20.1	21.1	21.2	21.6	23.3	24.5	25.0	26.7
WT-6	M	Vehicle	19.1	20.1	21.8	22.1	21.6				
WT-7	M	Vehicle	20.5	21.7	23.1	23.9	24.1				
WT-8	M	Vehicle	20.8	22.7	23.6	24.9	25.2	27.6	30.8	31.5	32.3
WT-9	M	Vehicle	21.3	22.7	25.1	25.3	25.6	27.4	29.5	31.0	32.7
WT-10	M	Vehicle	22.3	23.5	25.2	26.6	27.6	31.1	32.3	32.9	35.4
WT-11	M	1.25E+11	21.6	22.8	24.6	25.6	26.9	28.1	29.1	30.4	30.2
WT-12	M	1.25E+11	21.6	21.7	23.7	25.3	26.8	28.6	30.8	32.8	32.8
WT-13	M	1.25E+11	21.4	21.8	23.5	24.4	23.4				
WT-14	M	1.25E+11	21.7	22.7	25.1	26.4	26.8	28.0	29.8	31.5	32.7
WT-15	M	1.25E+11	21.8	23.6	24.3	27.2	28.2	29.2	30.1	31.4	32.3
WT-16	M	1.25E+11	20.7	22.6	24.9	25.5	25.2				
WT-17	M	1.25E+11	19.6	22.3	23.6	24.8	24.9				
WT-18	M	1.25E+11	19.4	20.3	22.1	23.8	25.5	28.6	30.1	31.7	33.2
WT-19	M	1.25E+11	25.1	25.7	26.8	26.9	27.6	28.5	30.3	31.0	31.4
WT-20	M	1.25E+11	20.5	21.8	24.4	26.3	27.9	30.1	32.1	32.7	33.9
WT-21	M	5.00E+11	20.6	22.1	23.5	24.5	26.0	28.3	28.6	30.3	31.0
WT-22	M	5.00E+11	21.6	23.2	25.1	26.4	28.5	30.3	31.0	33.0	33.0
WT-23	M	5.00E+11	22.8	24.6	26.1	28.3	28.1				
WT-24	M	5.00E+11	21.4	22.2	24.8	25.9	27.5	30.1	31.0	31.9	32.5
WT-25	M	5.00E+11	22.1	23.9	25.1	26.4	27.7	29.1	31.1	33.7	33.1
WT-26	M	5.00E+11	22.9	23.6	24.8	26.5	26.4				
WT-27	M	5.00E+11	22.5	23.4	25.7	26.8	26.8				
WT-28	M	5.00E+11	19.5	21.4	21.8	23.4	24.1	26.8	28.1	28.9	29.4
WT-29	M	5.00E+11	20.9	22.6	23.6	24.6	26.0	26.7	29.1	31.0	31.2
WT-30	M	5.00E+11	20.2	21.6	23.2	24.6	26.0	28.1	30.9	32.5	33.8
WT-31	F	Vehicle	14.6	15.4	16.1	16.5	17.4	19.1	20.4	21.2	19.0
WT-32	F	Vehicle	15.9	16.7	18.1	18.6	19.3	20.1	21.4	22.3	22.0
WT-33	F	Vehicle	13.7	15.1	16.3	16.3	16.2				
WT-34	F	Vehicle	14.9	15.6	16.6	17.8	18.0	20.6	21.4	21.8	21.6

WT-35	F	Vehicle	15.9	16.9	18.1	19.3	19.8	21.1	22.4	25.1	24.7
WT-36	F	Vehicle	15.7	16.4	17.7	18.7	18.2				
WT-37	F	Vehicle	18.4	19.6	19.7	19.9	18.0				
WT-38	F	Vehicle	16.4	16.8	18.8	18.7	20.0	21.9	22.6	23.3	24.0
WT-39	F	Vehicle	16.2	16.9	18.2	18.5	18.8	20.2	22.1	22.4	22.9
WT-40	F	Vehicle	15.4	16.7	17.3	18.2	18.7	20.9	21.5	22.5	22.3
WT-41	F	1.25E+11	15.7	16.3	19.1	18.2	18.8	19.0	20.0	20.7	19.0
WT-42	F	1.25E+11	16.1	17.4	19.1	19.2	20.6	22.4	23.5	26.2	23.0
WT-43	F	1.25E+11	15.4	16.3	18.1	18.3	16.0				
WT-44	F	1.25E+11	17.6	18.1	20.2	21.2	22.0	22.8	23.5	25.2	24.7
WT-45	F	1.25E+11	13.4	14.1	15.3	15.9	17.2	17.8	18.6	20.2	20.2
WT-46	F	1.25E+11	14.8	14.9	15.8	16.2	15.0				
WT-47	F	1.25E+11	16.6	17.1	18.7	19.3	17.0				
WT-48	F	1.25E+11	16.9	17.5	18.4	20.2	20.6	21.5	23.2	22.6	22.6
WT-49	F	1.25E+11	15.7	16.8	17.9	18.6	19.5	20.6	21.9	22.3	22.7
WT-50	F	1.25E+11	15.2	15.1	16.4	16.7	17.6	19.4	19.5	20.9	22.2
WT-51	F	5.00E+11	14.1	15.1	15.8	16.8	17.6	19.4	19.7	20.2	21.0
WT-52	F	5.00E+11	15.6	16.2	17.8	18.6	19.1	21.1	22.6	23.7	25.0
WT-53	F	5.00E+11	16.9	16.5	18.2	19.8	20.0				
WT-54	F	5.00E+11	14.9	14.8	15.7	16.5	16.2	18.1	19.8	19.8	20.6
WT-55	F	5.00E+11	17.7	17.2	18.2	19.8	20.0	22.5	23.3	24.4	25.6
WT-56	F	5.00E+11	14.5	14.9	16.1	18.4	17.0				
WT-57	F	5.00E+11	14.3	13.6	13.7	13.9	13.0				
WT-58	F	5.00E+11	16.6	16.9	18.2	19.5	20.0	21.8	23.3	23.4	23.6
WT-59	F	5.00E+11	14.3	14.9	16.2	17.3	17.3	18.3	19.6	20.0	20.7
WT-60	F	5.00E+11	14.1	14.4	15.8	17.2	18.0	19.2	20.3	20.3	21.9

Mouse ID	Sex	IT	24 Weeks	28 Weeks	32 Weeks	36 Weeks	40 Weeks	44 Weeks	48 Weeks	52 Weeks
WT-1	M	Vehicle								
WT-2	M	Vehicle								
WT-3	M	Vehicle								
WT-4	M	Vehicle	26.7	26.7	28.3	30.0	30.5	30.4	31.4	29.2
WT-5	M	Vehicle	29.2	29.1	32.1	33.2	33.8	34.3	36.2	35.2
WT-6	M	Vehicle								
WT-7	M	Vehicle								

WT-8	M	Vehicle	34.2	35.1	35.0	37.6	39.0	39.7	39.0	38.5
WT-9	M	Vehicle	32.8	33.5	33.6	36.5	38.5	38.2	38.3	38.1
WT-10	M	Vehicle	36.1	35.6	35.6	37.9	38.3	39.7	39.4	38.6
WT-11	M	1.25E+11								
WT-12	M	1.25E+11								
WT-13	M	1.25E+11								
WT-14	M	1.25E+11	34.0	34.6	36.2	36.9	37.5	38.1	38.4	37.6
WT-15	M	1.25E+11	33.5	34.9	36.0	36.3	36.7	38.2	38.3	37.9
WT-16	M	1.25E+11								
WT-17	M	1.25E+11								
WT-18	M	1.25E+11	34.3	34.7	36.3	38.2	38.3	38.2	39.8	38.5
WT-19	M	1.25E+11	31.4	32.0	32.4	34.9	34.5	34.3	35.4	35.6
WT-20	M	1.25E+11	34.6	36.6	37.0	39.0	39.7	39.7	40.8	41.7
WT-21	M	5.00E+11								
WT-22	M	5.00E+11								
WT-23	M	5.00E+11								
WT-24	M	5.00E+11	33.9	35.0	35.5	36.4	36.4	36.7	36.2	36.8
WT-25	M	5.00E+11	35.1	36.4	37.7	40.0	41.4	41.3	39.7	38.9
WT-26	M	5.00E+11								
WT-27	M	5.00E+11								
WT-28	M	5.00E+11	28.6	30.0	29.6	29.9	30.1	30.5	29.9	30.8
WT-29	M	5.00E+11	33.7	35.0	36.1	38.1	39.3	40.2	40.1	41.6
WT-30	M	5.00E+11	35.5	36.6	37.7	39.2	39.9	40.7	40.1	40.7
WT-31	F	Vehicle								
WT-32	F	Vehicle								
WT-33	F	Vehicle								
WT-34	F	Vehicle	22.7	22.9	23.4	23.8	24.6	25.8	26.4	25.9
WT-35	F	Vehicle	25.6	27.5	30.1	28.1	26.9	31.4	31.2	30.1
WT-36	F	Vehicle								

WT-37	F	Vehicle								
WT-38	F	Vehicle	24.2	25.6	26.2	29.4	31.4	32.7	32.3	33.1
WT-39	F	Vehicle	25.0	27.8	27.5	26.9	27.2	27.8	29.8	31.7
WT-40	F	Vehicle	25.2	25.5	27.2	26.6	27.6	28.0	29.2	30.4
WT-41	F	1.25E+11								
WT-42	F	1.25E+11								
WT-43	F	1.25E+11								
WT-44	F	1.25E+11	26.4	27.1	27.6	29.5	30.9	31.2	33.2	31.3
WT-45	F	1.25E+11	20.4	20.9	20.9	21.5	22.1	22.2	22.2	23.8
WT-46	F	1.25E+11								
WT-47	F	1.25E+11								
WT-48	F	1.25E+11	24.9	26.5	26.8	29.2	28.2	31.1	30.2	28.5
WT-49	F	1.25E+11	24.5	24.9	25.9	26.8	27.9	27.7	30.1	29.9
WT-50	F	1.25E+11	21.4	22.1	21.8	22.7	23.5	23.5	23.3	24.6
WT-51	F	5.00E+11								
WT-52	F	5.00E+11								
WT-53	F	5.00E+11								
WT-54	F	5.00E+11	21.4	21.4	22.0	22.7	22.3	20.7	20.3	19.9
WT-55	F	5.00E+11	25.6	27.3	28.6	29.5	29.8	31.4	33.4	33.9
WT-56	F	5.00E+11								
WT-57	F	5.00E+11								
WT-58	F	5.00E+11	23.7	23.9	23.0	24.3	25.2	24.7	24.8	25.9
WT-59	F	5.00E+11	21.6	20.9	21.7	22.1	22.7	23.4	23.8	24.5
WT-60	F	5.00E+11	21.9	21.6	21.8	23.6	24.5	23.5	23.1	23.2
