# Supplementary material for: Intrathecal AAV9/AP4M1 gene therapy for hereditary spastic paraplegia 50 shows safety and efficacy in preclinical studies
Source: J Clin Invest. 2023 May 15;133(10):e164575. doi: 10.1172/JCI164575 (PMC10178841; doi:10.1172/JCI164575)
Supplement: Supplemental data set 2 [file jci-133-164575-s059.pdf]

charles river

FINAL REPORT

Test Facility Study No. 5550014

Sponsor Reference No. UTSW-Gray-SPG50-004

A Single-Dose Study of AAV9/AP4M1 by Intrathecal Injection in Immunosuppressed Monkeys

GLP

SPONSOR:
University of Texas Southwestern Medical Center
5323 Harry Hines Blvd
Dallas, TX 75390-9056
USA

TEST FACILITY:
Charles River Laboratories Montreal ULC
Senneville Site (CR-SEN)
22022 Transcanadienne
Senneville, QC H9X 3R3
Canada

TABLE OF CONTENTS

LIST OF APPENDICES 3
QUALITY ASSURANCE STATEMENT 4
COMPLIANCE STATEMENT AND REPORT APPROVAL 6
1. RESPONSIBLE PERSONNEL 7
2. SUMMARY 8
3. INTRODUCTION 10
4. MATERIALS AND METHODS 10
4.1. Test Material Identification 10
4.2. Immunosuppression Material Identification 11
4.3. Stability 11
4.4. Study Design 11
4.5. Deviations 12
5. COMPUTERIZED SYSTEMS 14
6. RETENTION OF RECORDS, SAMPLES, AND SPECIMENS 15
7. RESULTS 16
7.1. Mortality 16
7.2. Clinical Observations 16
7.3. Body Weights and Body Weight Gains 16
7.4. Food Evaluation 16
7.5. Neurological Evaluation 16
7.6. Nerve Conductivity Velocity 16
7.7. Hematology 18
7.8. Coagulation 18
7.9. Clinical Chemistry 18
7.10. Urinalysis 19
7.11. Cytokine Analysis 19
7.12. Tissue Biodistribution, Gene Expression, and Splenocyte Analysis 19
7.13. Macroscopic Pathology 21
7.14. Organ Weights 21
7.15. Microscopic Evaluations 22
8. CONCLUSION 26
9. REFERENCES 27

LIST OF APPENDICES

Appendix 1 Amended Study Plan and Study Plan 28
Appendix 2 Test and Reference Item Characterization 108
Appendix 3 Individual Mortality 118
Appendix 4 Individual Clinical Observations 123
Appendix 5 Individual Body Weights 147
Appendix 6 Individual Body Weight Gains 162
Appendix 7 Individual Neurological Examinations 177
Appendix 8 SOP - Neurological Examinations 202
Appendix 9 Nerve Conduction Velocity 218
Appendix 10 Clinical Pathology Report 233
Appendix 11 Biomarker Cytokines Analysis Interpretative Report 310
Appendix 12 Bioanalysis Report 400
Appendix 13 Pathology Report 427
Appendix 14 Pathology Peer Review 488

QUALITY ASSURANCE STATEMENT

Study Number: 5550014

This Study has been audited by Quality Assurance in accordance with the applicable Good Laboratory Practice regulations. Reports were submitted in accordance with SOPs as follows:

		Dates Findings Submitted to:	
**Date(s) of Audit**	**Phase(s) Audited**	**Study Director**	**Test Facility Management**
12Jul2021	Study Plan Amendment 01	12Jul2021	12Jul2021
12Jul2021	Dose Preparation	12Jul2021	12Jul2021
12Jul2021	Final Study Plan	12Jul2021	12Jul2021
13Jul2021	Dose Administration	14Jul2021	14Jul2021
20Aug2021	Study Plan Amendment 02	20Aug2021	20Aug2021
24Sep2021	Study Plan Amendment 03	24Sep2021	24Sep2021
14Oct2021	Neurological Exam	14Oct2021	14Oct2021
14Oct2021	Study Plan Amendment 04	14Oct2021	14Oct2021
20Oct2021	Study Plan Amendment 05	20Oct2021	20Oct2021
06Nov2021	Data Review - Bioanalysis & Immunology	06Nov2021	06Nov2021
17Nov2021 - 18Nov2021	Data Review - Bioanalysis & Immunology	22Nov2021	22Nov2021
17Nov2021 - 18Nov2021	Report Preparation	22Nov2021	22Nov2021
17Nov2021 - 19Nov2021	Phase Report - Biomarkers	22Nov2021	22Nov2021
18Nov2021	Data Review - Sample Management	22Nov2021	22Nov2021
22Nov2021 - 24Nov2021	Data Review - Animal Care	24Nov2021	24Nov2021
22Nov2021 - 24Nov2021	Data Review - Clinical Pathology	25Nov2021	25Nov2021
22Nov2021 - 24Nov2021	Data Review - Formulations	24Nov2021	24Nov2021
22Nov2021 - 24Nov2021	Data Review - Sample Management	24Nov2021	24Nov2021
22Nov2021 - 24Nov2021	Data Review - Technical Operations	24Nov2021	24Nov2021
22Nov2021 - 24Nov2021	Data Review - Veterinary Services	24Nov2021	24Nov2021
24Nov2021	Report Preparation	24Nov2021	24Nov2021
24Nov2021	Data Review - Necropsy	24Nov2021	24Nov2021
24Nov2021	Data Review - Histology	24Nov2021	24Nov2021
24Nov2021	Report Preparation	24Nov2021	24Nov2021

QUALITY ASSURANCE STATEMENT

Study Number: 5550014

This Study has been audited by Quality Assurance in accordance with the applicable Good Laboratory Practice regulations. Reports were submitted in accordance with SOPs as follows:

		Dates Findings Submitted to:	
**Date(s) of Audit**	**Phase(s) Audited**	**Study Director**	**Test Facility Management**
24Nov2021	Data Review - Shipping/Receiving	25Nov2021	25Nov2021
25Nov2021	Phase Report - Clinical Pathology	26Nov2021	26Nov2021
26Nov2021	Phase Report - Pathology	26Nov2021	26Nov2021
29Nov2021	Report - Materials and Methods	30Nov2021	30Nov2021
29Nov2021 - 30Nov2021	Report - Results	30Nov2021	30Nov2021
29Nov2021	Phase Report - Deviation Log	30Nov2021	30Nov2021
29Dec2021	Study Plan Amendment 06	29Dec2021	29Dec2021
22Feb2022	Final Phase Report - Biomarkers	22Feb2022	22Feb2022
23Feb2022 - 24Feb2022	Final Report	24Feb2022	24Feb2022

In addition to the above-mentioned audits, process-based and/or routine facility inspections were also conducted during the course of this study. Inspection findings, if any, specific to this study were reported by Quality Assurance to the Study Director and Test Facility Management and listed as a Phase Audit on this Quality Assurance Statement.

The Final Report has been reviewed to assure that it accurately describes the materials and methods, and that the reported results accurately reflect the raw data.

All electronic signatures appear at the end of the document upon finalization.

COMPLIANCE STATEMENT AND REPORT APPROVAL

The study was performed in accordance with the OECD Principles of Good Laboratory Practice and as accepted by Regulatory Authorities throughout the European Union, United States of America (FDA), Japan (MHLW), and other countries that are signatories to the OECD Mutual Acceptance of Data Agreement.

Exceptions from the above regulations are listed below.

- Characterization of the test and reference items was performed by the Sponsor subcontractor according to established SOPs, controls, and approved test methodologies to ensure integrity and validity of the results generated; these analyses were not conducted in compliance with the GLP or GMP regulations.
- Stability testing of the supplied test and reference items was not performed in this study although required by the Study Plan. This had no significant impact due to the known high degree of stability of AAV9 vector under the conditions of this study. (Bee JS, 2022, Gruntman, 2015)
- Concentration and homogeneity of the test and reference item formulations were not determined in this study.
- Tissue bioanalysis, gene expression, and splenocyte analysis was performed by the Sponsor according to established SOPs, controls, and approved test methodologies to ensure integrity and validity of the results generated; these analyses were not conducted in compliance with the GLP regulations.
- Nerve conduction velocity (NCV) assessment was performed using scientifically sound but non-validated methodologies.

This study was conducted in accordance with the procedures described herein. All deviations authorized/acknowledged by the Study Director are documented in the study records. The report represents an accurate and complete record of the results obtained.

There were no deviations from the above regulations that affected the overall integrity of the study or the interpretation of the study results and conclusions.

All electronic signatures appear at the end of the document upon finalization.

1. RESPONSIBLE PERSONNEL

Role/Phase	Quality Assurance Program (QAP)	Name	Contact Information
Study Director	Charles River	Stefania Cinquino, BSc<br>(from study initiation to 11 Jul 2021 and from 10 Aug 2021 to end)<br><br>Julie Douville, PhD<br>(from 12 Jul 2021 to 09 Aug 2021)	Address as cited for Test Facility
Test Facility Management	Charles River	Julie Douville, PhD<br>(from study initiation to 11 Jul 2021 and from 10 Aug 2021 to end)<br><br>Kevin Norton, MSc, DSP<br>(from 12 Jul 2021 to 09 Aug 2021)	Address as cited for Test Facility
Test Facility QAP	Charles River	Nooshin Davani, BSc, RQAP-GLP	Address as cited for Test Facility
**Individual Scientist (IS)**			
Nerve Conduction Velocity	Charles River	Nataliya Sadekova, MSc, DSP	Address as cited for Test Facility
Clinical Pathology	Charles River	Florence Poitout, DVM, DACVP, DECVCP	Address as cited for Test Facility
Cytokines	Charles River	Hycham Harouchi, BSc	Address as cited for Test Facility
Immuno-toxicology Splenocyte Preparation[a]	Charles River	Philippe Rousseau, PhD	Address as cited for Test Facility
Pathology	Charles River	Elaine Debien, DVM, DES, MSc, DACVP	Address as cited for Test Facility
**Principal Investigator (PI)**			
Tissue Biodistribution Analysis (Bioanalysis)[b]	N/A	Steven Gray, PhD	University of Texas Southwestern Medical Center Dallas, TX, USA
Tissue Gene expression Analysis[b]	N/A		
Splenocyte Analysis[b]	N/A		

[a] No formal report for this phase.
[b] Sponsor.

2. SUMMARY

The objectives of this study were to determine the potential toxicity, biodistribution, and gene expression of AAV9/AP4M1, for the treatment of Spastic Paraplegia Type 50 (SPG50) caused by the AP4M1 gene mutation, when given by a single intrathecal injection to monkeys, to evaluate the potential reversibility and/or progression of any findings, and to determine the potential impact of immunosuppressant (IMS) on the toxicity profile of this AAV9 vector.

The study design was as follows:

Text Table 1
Experimental Design

Group No.	Test Material	Dose Level (vg)	Dose Volume (mL)	Dose Concentration (vg/mL)	No. of Animals Main Study	
					Males	Females
1	Reference Item[a]	0	1	0	1	1
2	AAV9/AP4M1	$8.4x10^{13}$	1.55	$5.43x10^{13}$	-	2
3	AAV9/AP4M1	$1.68x10^{14}$	3.10	$5.43x10^{13}$	1	1

[a] PBS containing 5% D-sorbitol and 0.001% Pluronic F-68.

The following parameters and endpoints were evaluated in this study: mortality, clinical observations, body weights, appetence, neurological examinations, nerve conduction velocity (NCV) evaluation, clinical pathology parameters (hematology, coagulation, clinical chemistry, and urinalysis), cytokines analysis, tissue bioanalysis and gene expression, splenocyte analysis, organ weights, and macroscopic and microscopic examinations.

There was no mortality during the course of the study.

There were no AAV9/AP4M1-related clinical observations, or effects on body weights, appetence, hematology, clinical chemistry, or urinalysis parameters, IL-6, IL-10, TNF-a, MCP-1, and IP-10 levels, macroscopic findings, or organ weights.

Clinical pathology results revealed equivocal mild increases in fibrinogen concentration in both animals administered $1.68x10^{14}$ vg on Days 52 and 94. These increases may have been associated with microscopic necrosis/inflammation observed in the biceps femoris at injection sites, however due to the higher magnitude observed in AAV9/AP4M1-dosed animals, this change remains of an uncertain relationship to AAV9/AP4M1.

There were AAV9/AP4M1-related increases in IL-8 noted in females at $\geq 8.4x10^{13}$ vg. Due to its low magnitude, this change was considered nonadverse.

Administration of AAV9/AP4M1 at $1.68x10^{14}$ vg resulted in notable microscopic findings consisting of an increase in the severity of degeneration (axonal and neuronal) in multiple tissues of the central and peripheral nervous system in animals at $1.68x10^{14}$ vg, which manifested into neurological findings. Degenerative changes were confirmed with Fluoro-Jade B (FJB), and associated with IBA-1, and/or GFAP staining, which were performed on the lumbar spinal cord (including injection site) and lumbar dorsal root ganglion (DRG) sections. Due to the nature of these findings, the axonal and neuronal degeneration were considered adverse. The remaining microscopic changes (perivascular vacuolation of macrophages and mononuclear cell infiltration) at $\geq 8.4x10^{13}$ vg were considered nonadverse due to the their low severity and that they were generally not directly associated with the neuronal/axonal degeneration.

At the injection site, axonal degeneration of the cauda equina was seen at $1.68x10^{14}$ vg in both sexes, while axonal degeneration of the dorsal funiculus and perivascular vacuolation of macrophages were seen in the male only. Perivascular infiltrates of mononuclear cells were noted in females at $\geq 8.4x10^{13}$ vg. In the spinal cord, axonal degeneration of the dorsal funiculus was seen in all three segments and considered AAV9/AP4M1-related at $1.68x10^{14}$ vg in both sexes.

In the lumbar dorsal root ganglion (DRG), mononuclear cell infiltrates occurred at $\geq 8.4x10^{13}$ vg in both sexes, and neuronal degeneration was noted at $1.68x10^{14}$ vg in both sexes. In the dorsal lumbar nerve root, there was axonal degeneration at $1.68x10^{14}$ vg in both sexes, and mononuclear cell infiltrates in females at $\geq 8.4x10^{13}$ vg. Mononuclear cell infiltrates also occurred in the dorsal thoracic nerve root in the male at $1.68x10^{14}$ vg.

In the brain, axonal degeneration of the medulla oblongata was noted in the male at $1.68x10^{14}$ vg, and an increase in the severity of meningeal mononuclear cell infiltrates in both females at $8.4x10^{13}$ vg.

In the trigeminal ganglion, there was axonal degeneration and mononuclear cell infiltrates in the male at $1.68x10^{14}$ vg.

In the peripheral nerves (sciatic, sural, and tibial), there was an increase in severity of axonal degeneration at $1.68x10^{14}$ vg in both sexes, most likely exacerbated by the edema/inflammation associated with experimental procedures (repetitive intramuscular injections). The degeneration in the nerves correlated with a decrease in nerve conduction velocity and response amplitude of the sural nerve noted on Day 45, 77 and 92; these changes were more prominent in the male, which was also noted with neurological findings of abnormal general attitude and motor function and decreased proprioceptive positioning in the left hindlimb on Day 94.

AAV9/AP4M1 resulted in delivery of AP4M1 vector DNA across the central nervous system (CNS) and peripheral organs. The AP4M1 vector DNA was widely detected at high levels in multiple brain regions. In the peripheral organs, even higher amounts of AP4M1 DNA persisted in the liver and to a lesser extent in other organs tested. Consistent with this AP4M1 DNA biodistribution data, AP4M1 transgene expression was also widely detected at high level in multiple CNS and peripheral tissues.

In conclusion, administration of AAV9/AP4M1 by a single intrathecal injection in monkeys at $8.4x10^{13}$ and $1.68x10^{14}$ vg was well tolerated. Adverse findings at $1.68x10^{14}$ vg included axonal or neuronal degeneration noted microscopically in the spinal cord (including the injection site), lumbar DRG, dorsal nerve roots, brain, trigeminal ganglion, and peripheral nerves (sciatic, sural, and tibial), with associated decreases in nerve conduction velocity and neurological effects. Based on these results, the no-observed-adverse-effect level (NOAEL) was considered to be $8.4x10^{13}$ vg.

Furthermore, it was concluded that AAV9 delivered intrathecally can achieve broad distribution as well as expression across the nervous system and peripheral organs without generating any immune response under the immunosuppressant regimen, although the level of gene transfer in the brain is sub-saturating with a minority of cells receiving the transgene.

3. INTRODUCTION

The objectives of this study were to determine the potential toxicity, biodistribution, and gene expression of AAV9/AP4M1, for the treatment of Spastic Paraplegia Type 50 (SPG50) caused by the AP4M1 gene mutation, when given by a single intrathecal injection to monkeys, to evaluate the potential reversibility and/or progression of any findings, and to determine the potential impact of immunosuppressant (IMS) on the toxicity profile of this AAV9 vector.

Study Initiation Date:	29 Jun 2021
Initiation of Dosing:	13 Jul 2021
Completion of In-life:	14 Oct 2021
Experimental Starting Date:	30 Jun 2021
Experimental Completion Date:	Signature date of the Pathology Report

4. MATERIALS AND METHODS

The study was conducted in accordance with the Study Plan and last Amended Study Plan presented in Appendix 1 with the following requirements and exceptions provided in the sections below.

4.1. Test Material Identification

Text Table 2
Test Item Identification

	Test Item
**Identification:**	AAV9/AP4M1 (Appendix 2)
**Alternate Identification:**	rAAV9.AP4M1
**Batch No.:**	T-GEMINIS-033
**Expiration/Retest Date:**	Not available
**Physical Description:**	Colorless, clear to slightly opalescent, free of visible particles
**Concentration (Based on ddPCR Results):**	5.43E13 vg/mL
**Storage Conditions:**	Set to maintain -80ºC
**Provided by:**	Sponsor

Text Table 3
Test Material Identification

	Reference Item/Vehicle
**Identification:**	PBS containing 5% D-sorbitol and 0.001% Pluronic F-68 (Appendix 2)
**Alternate Identification:**	1XdPBS, 5% sorbitol, 0.001% pluronico FMR-T-0043.01
**Batch No.:**	2021-02-09/01
**Expiration/Retest Date:**	Not available
**Storage Conditions:**	Set to maintain -80ºC
**Provided by:**	Sponsor

4.2. Immunosuppression Material Identification

Text Table 4
Immunosuppression (IMS) Identification

	IMS 1	IMS 2	IMS 3
**Identification:**	Methylprednisolone succinate	Methylprednisolone acetate	Rapamycin
**Alternate Identification:**	Solu-Medrol	Depo-Medrol	N/A
**Physical Description:**	Liquid	Liquid	Powder
**Concentration:**	125 mg/mL	40 mg/mL	N/A
**Storage Conditions (Temperature Set to Maintain):**	21°C<br>Protected from light	21°C	-20°C
**Provided by:**	Test Facility	Test Facility	Test Facility
**Supplier:**	McKesson Canada	Zoetis Canada or Pfizer	Toronto Research Chemicals or Alfa Aesar

N/A = Not applicable.

Text Table 5
Immunosuppressants Vehicle Identification

	Vehicle for Solu-Medrol	Vehicle Components for Rapamycin		
**Identification:**	0.9% sodium chloride injection, USP	N,N-Dimethylacetamide	Tween-80	Polyethylene Glycol
**Storage Conditions (Temperature Set to Maintain):**	21°C	21°C	21°C	21°C
**Provided by:**	Test Facility	Test Facility		

4.3. Stability

For the time periods covered by the parameters of this study, stability was demonstrated for all samples related to cytokine samples.

4.4. Study Design

Text Table 6
Experimental Design

Group No.	Test Material	Dose Level (vg)	Dose Volume (mL)	Dose Concentration (vg/mL)	Animal Numbers Main Study	
					Males	Females
1	Reference Item[a]	0	1	0	1201	1701
2	AAV9/AP4M1	8.4×10^{13}	1.55	5.43×10^{13}	-	2701, 2702
3	AAV9/AP4M1	1.68×10^{14}	3.10	5.43×10^{13}	3201	3701

[a] PBS containing 5% D-sorbitol and 0.001% Pluronic F-68.

Text Table 7
Additional Study Plan Deliverables

**Animal Source:**	Charles River Laboratories, Frederick, MD, USA
**Age at Initiation of Dosing**	3 to 4 years old
**Body Weight Range at Initiation of Dosing**	3.1 – 3.2 kg males<br>2.6 – 3.4 kg females
**Supplemental Diet**	Animals received half of their morning and afternoon pellets in juice and an extra portion of fruit and vegetables on Days -2 to 2.
**Additional Enrichment**	Additional enrichment items were provided throughout the study
**Number of Acclimation days**	13

None of the following study events had an adverse impact on the study or the interpretation of the data unless otherwise indicated.

Text Table 8
Study Events

**Event**
• On Days 28 to 35, Female No. 2702 received the antibiotic trimethoprim sulfamethoxazole (TMS) orally once daily due to redness, swelling, and a skin lesion with discharge on the left hindlimb. A swab of the lesion was sent to Idexx laboratories for bacterial testing which came back positive for staphylococcus aureus and leclercia.<br>• On occasion, reflux and/or spillage was noted during the rapamycin administration for some animals. Since these occurrences were observed on occasion for animals in each group and were considered minor, they did not have an impact on the overall immunosuppressant exposure of the animals.

4.5. Deviations

All deviations that occurred during the study have been authorized/acknowledged by the Study Director, assessed for impact, and documented in the study records. All Study Plan deviations and any SOP deviations that could have impacted the quality or integrity of the Study are listed below.

None of the deviations were considered to have impacted the overall integrity of the study or the interpretation of the study results and conclusions.

Text Table 9
Deviations

**Formulations and Dosing**		
**Study Plan Section No.**	**Deviation**	**Impact**
7.1	The Solu-Medrol supplier indicated in the Study Plan was Pfizer, however it was received from McKesson Canada.	The Solu-Medrol used on study was from an appropriate supplier.
7.1	Tween 80 and Rapamycin were not stored protected from light, although indicated in the Study Plan.	It is not a requirement to have stored these individual components protected from light, however, the rapamycin solution was stored in amber glass vials as intended.

Formulations and Dosing		
**Study Plan Section No.**	**Deviation**	**Impact**
10.3.1	On Day 1, the 0.5-mL Abbocath® flush with 0.9% sodium chloride was not administered to Male No. 3201 following the immunosuppression administration of methylprednisolone succinate.	Based on the relatively minimal dead volume of the Abbocath® (approximately 0.3 mL), this had no impact as the animal received within 20% of its intended dose volume.
**Husbandry**		
**Study Plan Section No.**	**Deviation**	**Impact**
8.	The origin of the spare animal and Female No. 3701 was Chinese instead of Vietnamese.	This had no impact on the study objectives as these animals were considered suitable for use on the study.
8.3	As per study plan, at least 2 weeks should have been allowed between animal transfer and the start of dosing, however, 13 days of acclimation was provided.	The acclimation provided to the animals was considered sufficient.
8.4	Animals in Groups 1 and 3 were randomly assigned to treatment groups instead of being assigned as pre-established pairs using a computerized based randomization procedure, as indicated in the Study Plan.	The animal identification list assigning these animals was signed by the study director prior to placement in groups. Furthermore, bodyweights were evenly distributed among groups and all animals were considered suitable for use.
**In-life Observations, Measurements, and Evaluations**		
**Study Plan Section No.**	**Deviation**	**Impact**
11.	General in-life assessments were not performed on the spare animal aside from bodyweight and detailed examinations until the animal was released. Additionally, on Days 35 and 94, the bodyweight measurement (Day 35 only) or detailed examination were not performed.	No impact as the spare was not used on the study.
11.	On occasion, the afternoon mortality check was not performed.	There was no impact on the welfare of the animals; animals were verified at other occasions on the same day and the following day.
11.	On Day 13, the food evaluation was not performed.	Sufficient data were collected on the study to allow for potential effects on appetence.
**Postmortem and Pathology**		
**Study Plan Section No.**	**Deviation**	**Impact**
14.5	There was no weight recorded for the thymus of Female No. 1701. Furthermore, the tissue for this animal was lost following fixation.	No impact on pathology evaluation as the organ weight change present in the thymus was seen across dose groups, including the control male, and was considered not related to the administration of AAV9/AP4M1.
15.1	The tongue was omitted in the list of tissues to undergo histology processing; therefore the tissue was not prepared to slide for any animal.	No impact on pathology evaluation as this tissue was not to be examined microscopically.

Formulations and Dosing		
**Study Plan Section No.**	**Deviation**	**Impact**
**Retention and Disposition of Records, Samples, and Specimens**		
**Study Plan Section No.**	**Deviation**	**Impact**
21	The prestudy/predose serum and CSF samples will be shipped to the Sponsor following finalization of the study, as the CITES permit renewals will not be received prior to scheduled finalization. Furthermore, Watson cannot be archived prior to finalization until samples are shipped. Watson and any documentation generated will be archived following sample shipment and confirmation of receipt.	No impact as samples will nonetheless be shipped and any records archived.
**SOP**		
**Deviation**		**Impact**
The dose volume was not adjusted to compensate for the dead volume of the spinal needle (0.1 mL for pencil point type needles), as per SOP.		Based on the minimal volume difference, animals received their intended dose volume.
**Planned Deviations**		
**Study Plan Section No.**	**Deviation**	**Impact**
10.3	Due to the animal transfer date and start of dosing, a planned deviation was issued for the administration of rapamycin to be started on Day -13, however, a discrepancy was noted in this deviation and should have stated Day -12 instead. Furthermore, the spare animal was also administered rapamycin.	The duration of rapamycin treatment was considered sufficient to allow for immunosuppressant effect; it was started on the intended day. In addition, the spare animal was treated should any replacements be needed.
13.3	A planned deviation was issued to address the discrepancy in the storage of the prestudy CSF samples; samples were to be collected on dry ice instead of wet ice.	Dry ice was the appropriate storage condition for this type of sample.

5. COMPUTERIZED SYSTEMS

Critical computerized systems used in the study are listed below or presented in the appropriate Phase Report. All computerized systems used in the conduct of this study have been validated; when a particular system has not satisfied all requirements, appropriate administrative and procedural controls were implemented to assure the quality and integrity of data.

Text Table 10
Critical Computerized Systems

System Name	Version No.	Description of Data Collected and/or Analyzed
Provantis®	10	In-life; clinical pathology; postmortem; Test Material receipt, accountability, and/or formulation activities.
M-Files®	21	Reporting and collection of 21 CFR Part 11 compliant signature
DocuSign™	Part 11	Collection of 21 CFR Part 11 compliant signature
eInfotree	7	Excel Module for data collection with 21 CFR Part 11 compliance requirements, security, audit trail, and electronic signatures.
Acq*Knowledge*	4	Electrophysiological recording
Softmax Pro GxP	5.4.6	Cytokine data collection
Bio Plex Manager (Bio-Rad)	6.1	Cytokine data collection
Watson LIMS	7.6.1 HF3	Cytokine data analysis and sample management
SRS (CR-SEN in-house application built with SAS and SAS system for Windows)	1	Statistical analyses of cytokines
Mesa Laboratories AmegaView CMS	v3 Build 1209.08	Continuous Monitoring System. Monitoring of standalone fridges, freezers, incubators, and selected laboratories to measure temperature, relative humidity, and CO_2, as appropriate
Johnson Controls Metasys	MVE 7	Building Automation System. Control of HVAC and other building systems, as well as temperature/humidity control and trending in selected laboratories and animal rooms

6. RETENTION OF RECORDS, SAMPLES, AND SPECIMENS

All study-specific raw data, documentation, Study Plan, samples, specimens, and final reports from this study were archived at the Test Facility by no later than the date of Final Report issue. At least 1 year after issue of the Draft Report, the Sponsor will be contacted.

Electronic data generated by the Test Facility were archived as noted above, except that the data collected using Provantis®, reporting files stored on M-Files®, and deviations were archived at the Charles River Laboratories facility location in Wilmington, MA.

All records, retained samples and specimens, and reports generated from phases or segments performed by Sponsor will be archived by the Sponsor. Details regarding the retention of the materials were provided to the Study Director.

Disposition of residual/retained analytical samples was as described in the table below.

Text Table 11
Disposition of Residual/Retained Samples

Sample Type	Disposition
Remaining dose formulations	Archived by the Sponsor
Additional serum	Archived by the Sponsor
Cytokine	Discarded
Splenocytes	Archived by the Sponsor
Bioanalytical (serum and CSF)	To be archived by the Sponsor[a]
Tissues (biodistribution and gene expression)	Archived by the Sponsor

[a] Samples will be archived following shipment of the samples to the Sponsor (after finalization of the study; refer to Deviations for further details).

7. RESULTS

7.1. Mortality

(Appendix 3)

There were no unscheduled deaths during the study.

7.2. Clinical Observations

(Appendix 4)

There were no AAV9/AP4M1-related clinical observations.

Clinical observations including, but not limited to, hunched posture, erected fur, tremors, decreased muscle tone, and decrease activity, were not due to the administration of AAV9/AP4M1 due to their low incidence, transient or sporadic nature, or similar incidence in the control group.

7.3. Body Weights and Body Weight Gains

(Appendix 5 and Appendix 6)

There were no AAV9/AP4M1-related effect on body weights or body weight gains.

Any variations were within the range of the concurrent control group data, lacked a dose relationship, and/or were considered to be due to biological variation.

7.4. Food Evaluation

(Appendix 4)

There was no AAV9/AP4M1-related effect on appetite.

7.5. Neurological Evaluation

(Appendix 7 and Appendix 8)

AAV9/AP4M1-related neurological changes were noted on Day 94 for Male No. 3201 and consisted of abnormal general attitude and motor function (slight tremors in the hindlimbs) and decreased proprioceptive positioning in the left hindlimb.

7.6. Nerve Conductivity Velocity

(Appendix 9)

Intrathecal administration of AAV9/AP4M1 at 1.68×10^{14} vg resulted in a slight decrease in mean sural nerve conduction velocity of 11% on Day 45, relative to the control group, accompanied by a decrease in mean response amplitude of 27%. Male No. 3201 exhibited the most significant change with sural nerve conduction velocity (NCV) of 44 m/sec compared to a mean of 52 m/sec in control animals.

On Days 77 and 92, a significant decrease in mean sural nerve conduction velocity of 26% and 28%, respectively, was noted at 1.68×10^{14} vg. The changes in conduction velocity were accompanied by associated decreases in mean sural nerve response amplitude which was reduced by 69% on Day 77 and by 74% on Day 92. Male No. 3201 was the most impacted, exhibiting the

most pronounced changes with sural nerve conduction velocity of 34 m/sec (35% change relative to mean control value) on Day 77 and 32 m/sec (39%) on Day 92 compared to 52-53 m/sec in control animals. The most pronounced decrease in response amplitude was also noted in Male No. 3201, particularly on Day 92, during which an amplitude of 1.2 µV was noted compared to a mean amplitude of 10.2 µV in control animals.

Individual sural NCV values are presented in the following graphs.

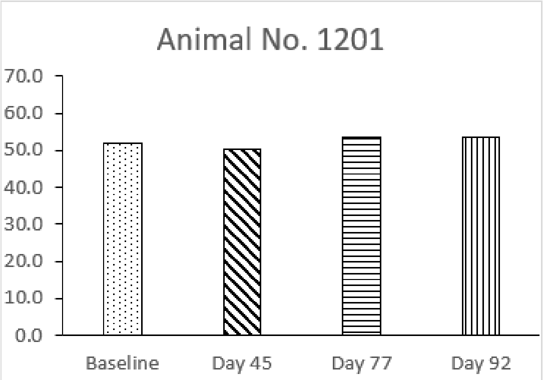

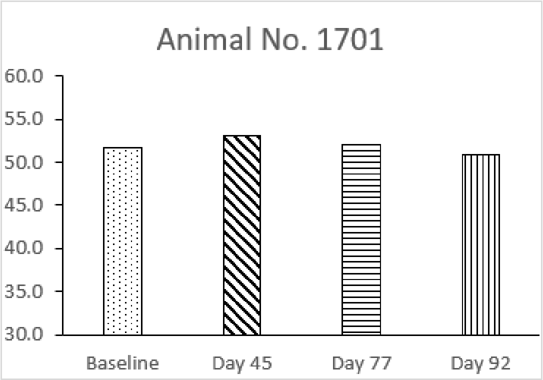

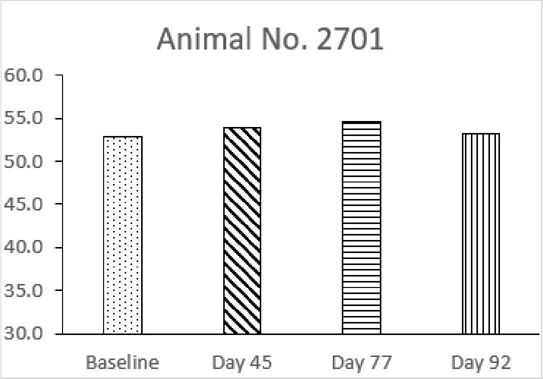

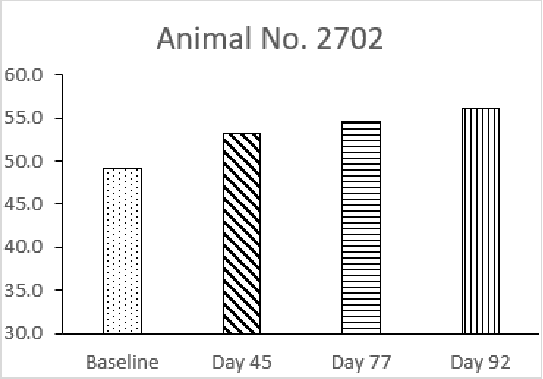

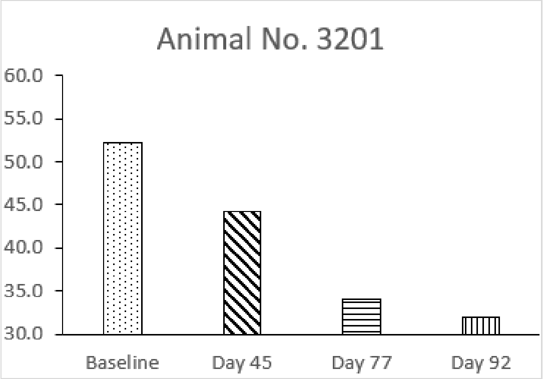

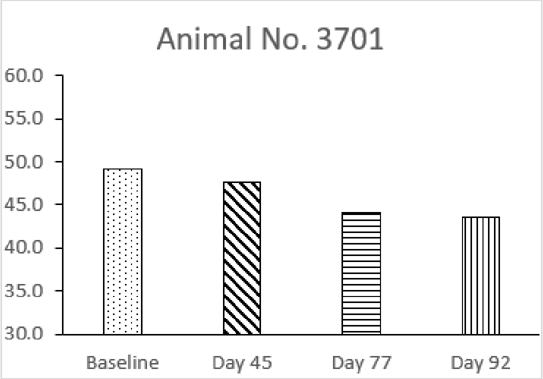

No changes in peroneal nerve conduction velocity or amplitude and no changes in the onset latency of the cauda equina were noted up to Day 92. In addition, there were no changes in conduction velocity or amplitude for the sural nerve at $8.4x10^{13}$ vg.

7.7. Hematology

(Appendix 10)

No AAV9/AP4M1-related hematology changes were noted at any dose level.

On Day 24 only, one female administered AAV9/AP4M1 at 8.4×10^{13} vg (No. 2702) presented mild increases in neutrophil (1.56x, individual value compared to respective pretreatment) and monocyte (3.77x) counts, correlating with changes in coagulation and clinical chemistry parameters. Similar changes in neutrophil and monocyte counts were observed in other animals including one control female (No. 1701) at other timepoints.

All differences in hematology parameters were considered not AAV9/AP4M1-related based on their small magnitude, inconsistent direction, absence of a dose response, general overlap of individual values with the range of control and baseline values, and/or were of a magnitude of change commonly observed in monkeys under similar study conditions.

7.8. Coagulation

(Appendix 10)

On Day 24 only, one female administered AAV9/AP4M1 at 8.4×10^{13} vg (No. 2702) presented a transient increase in fibrinogen concentration (2.11x individual value compared to pretreatment), correlating with changes in hematology and clinical chemistry parameters.

A gradual increase in fibrinogen concentration was observed in all individual animals, including control animals between Day 2 and Day 94 compared to their respective pretreatment values. On Days 52 and 94, the male and female at 1.68×10^{14} vg (Nos. 3201 and 3701) presented mild increases in fibrinogen concentration (1.82x to 2.55x, individual values compared to respective pretreatment) that were of higher magnitude than the increases observed in control animals (1.15x to 1.47x, individual values compared to pretreatment). These increases were not associated with relevant changes in globulins or leukocytes. They occurred several weeks after the administration of AAV9/AP4M1 and may have been associated with the microscopic necrosis/inflammation observed in the biceps femoris at the immunosuppressant injection sites; however, they remain of an uncertain relationship to AAV9/AP4M1.

Remaining differences in coagulation parameters were considered not AAV9/AP4M1-related based on their small magnitude, inconsistent direction, absence of a dose response, general overlap of individual values with the range of control and baseline values, and/or were of a magnitude of change commonly observed in monkeys under similar study conditions.

7.9. Clinical Chemistry

(Appendix 10)

No AAV9/AP4M1-related clinical chemistry changes were noted at any dose level.

On Day 24 only, one female administered at 8.4×10^{13} vg (No. 2702) presented transient mild increases in globulins (1.32x, individual values compared to pretreatment), triglycerides (4.31x), and decreases in albumin (0.81x) and albumin/globulin ratio (0.57x). These changes correlated with increases in neutrophil and monocyte counts, and an increase in fibrinogen concentration. They were supportive of inflammation and were possibly related to a moderate skin lesion of the left hindlimb observed on Day 28 in this animal. These changes were not observed on Day 52 or

Day 94, after resolution of the skin lesion. This individual had no particular microscopic findings observed at the end of the study; therefore these changes were unlikely AAV9/AP4M1-related.

Remaining differences in clinical chemistry parameters were considered not AAV9/AP4M1-related based on their small magnitude, inconsistent direction, absence of a dose response, general overlap of individual values with the range of control and baseline values, and/or were of a magnitude of change commonly observed in monkeys under similar study conditions.

7.10. Urinalysis

(Appendix 10)

No AAV9/AP4M1-related urinalysis changes were noted at any dose level.

All differences in urinalysis parameters were considered not AAV9/AP4M1-related based on their small magnitude, inconsistent direction, absence of a dose response, general overlap of individual values with the range of control and baseline values, and/or were of a magnitude of change commonly observed in monkeys under similar study conditions.

7.11. Cytokine Analysis

(Appendix 11)

All females administered AAV9/AP4M1 showed increases in IL-8 levels. Increases were noted at 30 minutes postdose (1.8X and 2.7X, respectively) as well as 24 hours postdose (2.9X and 1.2X, respectively). Female No. 2702 showed a 1.6X and 1.5X increase at 4 and 24 hours postdose, respectively. Those increases of low magnitude were considered AAV9/AP4M1-related due to their high incidence with the females. There was no effect in the male administered 1.68×10^{14} vg.

No AAV9/AP4M1-related effect could be observed for IL-6, IL-10, TNF-a, MCP-1, and IP-10.

7.12. Tissue Biodistribution, Gene Expression, and Splenocyte Analysis

(Appendix 12)

Intrathecal delivery of AAV9/AP4M1 vector resulted in delivery of the AP4M1 vector DNA across the central nervous system and peripheral organs. The AP4M1 vector DNA was widely detected at high levels in multiple brain regions. In the peripheral organs, even higher amounts of AP4M1 DNA persisted in the liver and to a lesser extent in other organs tested. AP4M1 vector biodistribution results are shown in Text Figure 1.

Consistent with this AP4M1 DNA biodistribution data, AP4M1 transgene expression was also widely detected at high level in multiple central nervous system (CNS) and peripheral tissues (Text Figure 2). Collectively, IT delivery of AAV9/AP4M1 results in broad AP4M1 biodistribution and expression across the body of NHPs.

Text Figure 1
AP4M1 Tissue Biodistribution on Day 94

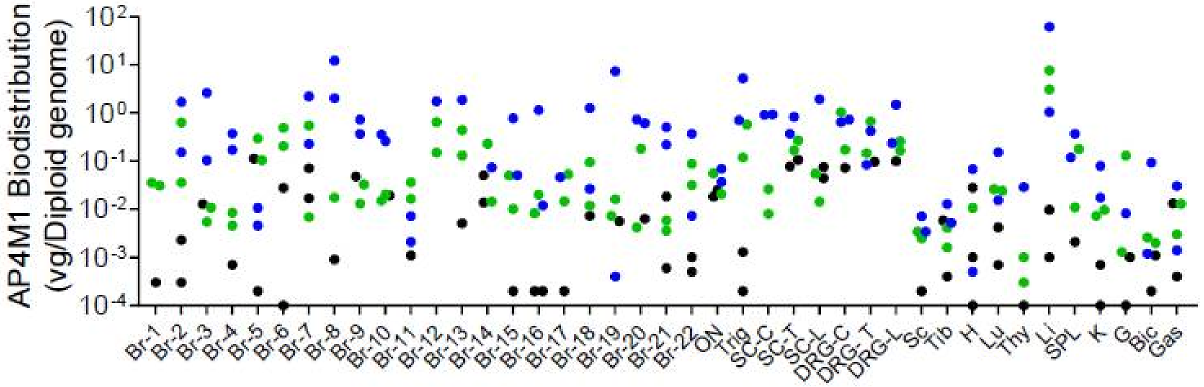

0 vg = black dots; 8.4×10^{13} vg = green dots; 1.68×10^{14} vg = blue dots;
Br-1, Br-2 = Frontal Cortex; Br-3, Br-4 = Striatum; Br-5, Br-6 = Parietal Cortex; Br-7, Br-8 = Temporal Cortex; Br-9, Br-10 = Hippocampus; Br-11, Br-12 = Thalamus; Br-13, Br-14 = Pons; Br-15, Br-16 = Midbrain; Br-17, Br-18 = Medulla; Br-19, Br-20 = Cerebellum; Br-21, Br-22 = Occipital Cortex; ON = Optic Nerve; Trig = Trigeminal Ganglion; SC-C = Cervical Spinal Cord; SC-T = Thoracic Spinal Cord; SC-L = Lumbar Spinal Cord; DRG-C = Cervical Dorsal Root Ganglion (DRG); DRG-T = Thoracic DRG; DRG-L = Lumbar DRG; Sc = Sciatic Nerve; Tib = Tibial Nerve; H = Heart; Lu = Lung; Thy = Thymus; Li = Liver; SPL = Spleen; K = Kidney; G = Gonad; Bic = Biceps Femoris; Gas = Gastrocnemius

Text Figure 2
AP4M1 Tissue Expression on Day 94

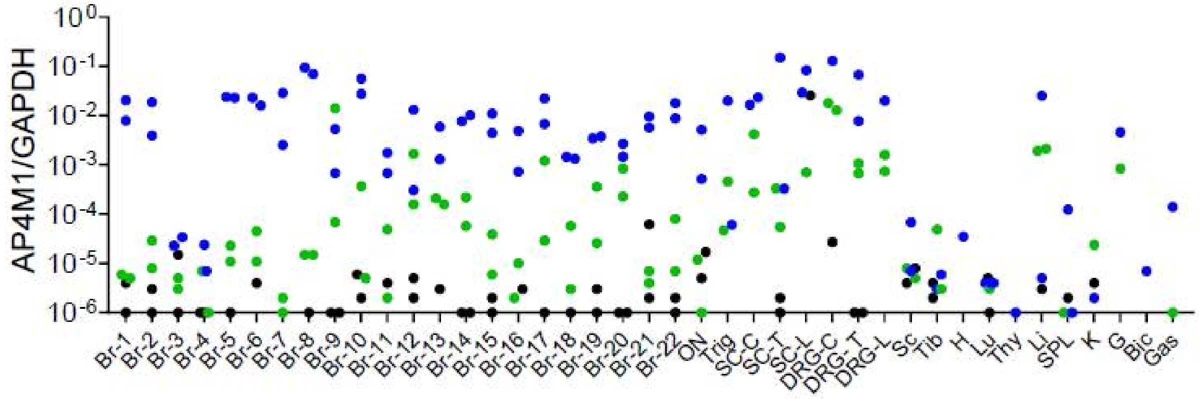

0 vg = black dots; 8.4×10^{13} vg = green dots; 1.68×10^{14} vg = blue dots;
Br-1, Br-2 = Frontal Cortex; Br-3, Br-4 = Striatum; Br-5, Br-6 = Parietal Cortex; Br-7, Br-8 = Temporal Cortex; Br-9, Br-10 = Hippocampus; Br-11, Br-12 = Thalamus; Br-13, Br-14 = Pons; Br-15, Br-16 = Midbrain; Br-17, Br-18 = Medulla; Br-19, Br-20 = Cerebellum; Br-21, Br-22 = Occipital Cortex; ON = Optic Nerve; Trig = Trigeminal Ganglion; SC-C = Cervical Spinal Cord; SC-T = Thoracic Spinal Cord; SC-L = Lumbar Spinal Cord; DRG-C = Cervical Dorsal Root Ganglion (DRG); DRG-T = Thoracic DRG; DRG-L = Lumbar DRG; Sc = Sciatic Nerve; Tib = Tibial Nerve; H = Heart; Lu = Lung; Thy = Thymus; Li = Liver; SPL = Spleen; K = Kidney; G = Gonad; Bic = Biceps Femoris; Gas = Gastrocnemius

The AAV9/AP4M1 vector generated no detectable T-cell immune response to either AAV9 or the human AP4M1 protein, under the immunosuppressant regimen, as shown in Text Figure 3.

Text Figure 3
Immune Response of Splenocytes on Day 94

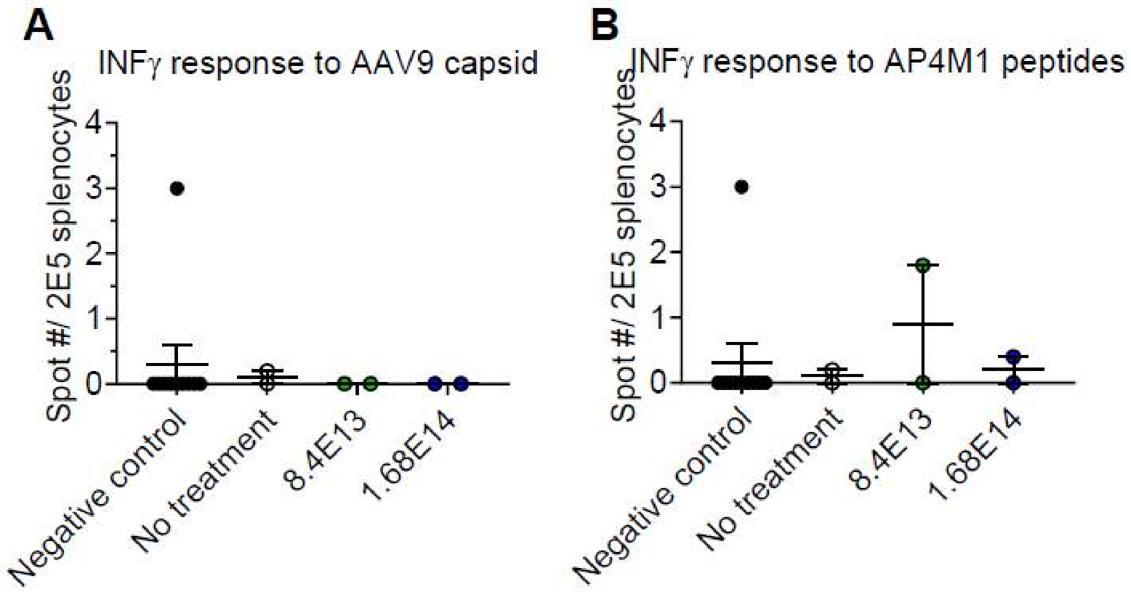

7.13. Macroscopic Pathology

(Appendix 13)

No AAV9/AP4M1-related gross findings were noted. The gross findings observed were considered incidental, related to experimental procedures/immunosuppressant administration, of the nature commonly observed in this strain and age of monkeys, and/or were of similar incidence in control and treated animals and, therefore, were considered unrelated to the administration of AAV9/AP4M1.

These included the small thymuses that were observed with a similar incidence across dose groups, including controls, and considered secondary to the administration of immunosuppressants and unrelated to the administration of AAV9/AP4M1. Furthermore, the macroscopic findings noted in the biceps femoris (swelling, thickness, dark/pale foci, and/or mottled discoloration) were seen with a similar incidence across dose groups, including controls, and were considered secondary to the repetitive procedure of intramuscular injection in the thigh, and unrelated to the administration of AAV9/AP4M1.

7.14. Organ Weights

(Appendix 13)

No AAV9/AP4M1-related organ weight changes were noted. There were isolated organ weight values that were different from their respective controls. There were, however, no patterns, trends, or correlating data to suggest these values were toxicologically relevant. Thus, the organ weight differences observed were considered incidental, related to experimental procedures/immunosuppressant administration, and/or related to difference of sexual maturity and unrelated to the administration of AAV9/AP4M1.

These included the decreases in thymus weight (absolute or relative to body and brain weight), when compared to organ weight historical data at the Test Facility, that were observed with a

similar magnitude across dose groups, including the control, and were considered secondary to the administration of immunosuppressants, and unrelated to the administration of AAV9/AP4M1.

7.15. Microscopic Evaluations

(Appendix 13)

AAV9/AP4M1-related microscopic findings occurred in the spinal cord (all segments, including the injection site), lumbar dorsal root ganglion (DRG), lumbar and thoracic dorsal nerve roots, brain, trigeminal ganglion, and peripheral nerves (sciatic, sural, and tibial), and are summarized in the following table.

Text Table 12
Summary of Microscopic Findings (H&E) – Scheduled Euthanasia (Day 94)

	Males		Females		
**Group**	**1**	**3**	**1**	**2**	**3**
**Dose (vg)**	**0**	**$1.68x10^{14}$**	**0**	**$8.4x10^{13}$**	**$1.68x10^{14}$**
**No. Animals per Group**	**1**	**1**	**1**	**2**	**1**
**Injection site (No. Examined)**	1	1	1	2	1
Degeneration; axonal, cauda equina	(0)[a]	(1)	(0)	(0)	(1)
Moderate	-	0	-	-	1
Marked	-	1	-	-	0
Degeneration; axonal, dorsal funiculus	(0)	(1)	(0)	(0)	(0)
Minimal	-	1	-	-	-
Vacuolation; perivascular, macrophage	(0)	(1)	(0)	(0)	(0)
Mild	-	1	-	-	-
Infiltration, mononuclear cell; perivascular	(0)	(0)	(0)	(1)	(1)
Minimal	-	-	-	1	1
**Spinal cord, lumbar (No. Examined)**	1	1	1	2	1
Degeneration; axonal, dorsal funiculus	(0)	(1)	(1)	(1)	(1)
Minimal	-	0	1	1	0
Mild	-	0	0	0	1
Moderate	-	1	0	0	0
**Spinal cord, thoracic (No. Examined)**	1	1	1	2	1
Degeneration; axonal, dorsal funiculus	(0)	(1)	(0)	(1)	(1)
Minimal	-	0	-	1	0
Mild	-	0	-	0	1
Moderate	-	1	-	0	0
**Spinal cord, cervical (No. Examined)**	1	1	1	2	1
Degeneration; axonal, dorsal funiculus	(0)	(1)	(0)	(1)	(1)
Minimal	-	0	-	1	0
Mild	-	1	-	0	1
**DRG, lumbar (No. Examined)**	1	1	1	2	1
Infiltration, mononuclear cell	(0)	(1)	(0)	(2)	(1)
Minimal	-	1	-	2	1
Degeneration; neuronal	(0)	(1)	(0)	(0)	(1)
Minimal	-	1	-	-	1
**Nerve root, dorsal, lumbar (No. Examined)**	1	1	1	2	1
Degeneration; axonal	(0)	(1)	(0)	(0)	(1)
Minimal	-	1	-	-	1
Infiltration, mononuclear cell	(0)	(0)	(0)	(1)	(1)
Minimal	-	-	-	1	1

	Males		Females		
**Group**	**1**	**3**	**1**	**2**	**3**
**Dose (vg)**	**0**	**1.68×10^{14}**	**0**	**8.4×10^{13}**	**1.68×10^{14}**
**No. Animals per Group**	**1**	**1**	**1**	**2**	**1**
**Nerve root, dorsal, thoracic (No. Examined)**	1	1	1	2	1
Infiltration, mononuclear cell	(0)	(1)	(0)	(0)	(0)
Minimal	-	1	-	-	-
**Brain (No. Examined)**	1	1	1	2	1
Degeneration; axonal, medulla oblongata	(0)	(1)	(0)	(0)	(0)
Minimal	-	1	-	-	-
Infiltration, mononuclear cell; meninges	(1)	(1)	(0)	(2)	(1)
Minimal	1	1	-	0	1
Mild	0	0	-	2	0
**Ganglion, trigeminal (No. Examined)**	1	1	1	2	1
Degeneration; axonal	(0)	(1)	(0)	(0)	(0)
Minimal	-	1	-	-	-
Infiltration, mononuclear cell	(0)	(1)	(0)	(0)	(0)
Minimal	-	1	-	-	-
**Nerve, sciatic (No. Examined)**	1	1	1	2	1
Degeneration; axonal	(1)	(1)	(1)	(2)	(1)
Minimal	1	0	0	0	0
Mild	0	1	1	2	0
Moderate	0	0	0	0	1
**Nerve, sural (No. Examined)**	1	1	1	2	1
Degeneration; axonal	(0)	(1)	(0)	(2)	(1)
Minimal	-	0	-	2	0
Mild	-	1	-	0	1
**Nerve, tibial (No. Examined)**	1	1	1	2	1
Degeneration; axonal	(1)	(1)	(1)	(2)	(1)
Minimal	1	0	1	1	0
Mild	0	1	0	1	1

[a] Numbers in parentheses represent the number of animals with the finding.

At the injection site, which was located at the level of the caudal lumbar spinal cord (target L5-L6), there was moderate to marked axonal degeneration of the nerve roots of the cauda equina in both animals given 1.68×10^{14} vg. The axonal degeneration consisted of vacuolation of the axonal sheath, swelling of the axon, and/or internal amorphous eosinophilic debris with or without macrophages (digestion chambers). In the male at 1.68×10^{14} vg, there was additional minimal axonal degeneration of the dorsal funiculus, as well as mild perivascular vacuolation of macrophages. Minimal perivascular infiltrates of mononuclear cells were also noted in 2 females at $\geq 8.4 \times 10^{13}$ vg. These infiltrates were mainly composed of lymphocytes, with fewer macrophages. In general, the incidence and severity of axonal degeneration and/or mononuclear cell infiltrates at the injection site paralleled the incidence and severity of positive reaction to Fluoro-Jade B fluorescent stain (indicating degenerated axons), and increased intensity of IBA-1 (microglial cells) and/or GFAP (astrocytes) stains in the affected animals (Text Table 12).

In the lumbar spinal cord, there was an increase in the severity of axonal degeneration in the dorsal funiculus in both male and female at 1.68×10^{14} vg (mild to moderate), when compared to control animals. Positivity to Fluoro-Jade B stain and increased intensity of IBA-1 and/or GFAP stains reflected well this change in both animals at 1.68×10^{14} vg. Additionally, based on Fluoro-Jade B stain evaluation, minimal axonal degeneration was observed in the lateral and ventral funiculi of the female at 1.68×10^{14} vg.

In the thoracic and cervical spinal cord, there was mild or moderate axonal degeneration in the dorsal funiculus at 1.68×10^{14} vg in both sexes. Minimal severity of axonal degeneration seen in one female at 8.4×10^{13} vg was considered not related to AAV9/AP4M1 given the similar incidence and severity of axonal degeneration noted in the spinal cord (lumbar) of the control female.

In the lumbar dorsal root ganglion, minimal mononuclear cell infiltrates occurred in all AAV9/AP4M1-dosed animals at $\geq 8.4 \times 10^{13}$ vg, and correlated with minimal increased intensity of IBA-1 in 2 females at $\geq 8.4 \times 10^{13}$ vg. Minimal neuronal degeneration was also noticed in the lumbar DRG in both male and female at 1.68×10^{14} vg. This latter change was characterized by the effacement/loss of rare neuronal cell bodies with presence of glial and/or mononuclear cells. Lumbar DRG findings were coupled with minimal axonal degeneration of the dorsal lumbar nerve root in both animals at 1.68×10^{14} vg, as well as minimal mononuclear cell infiltrates in females at $\geq 8.4 \times 10^{13}$ vg, both changes correlating with Fluoro-Jade B stain positivity and/or increased intensity of IBA-1 and/or GFAP in these animals. Minimal mononuclear cell infiltrates were also seen in the dorsal thoracic nerve root in the male at 1.68×10^{14} vg.

In the brain, there was minimal axonal degeneration at the periphery of the medulla oblongata in the male at 1.68×10^{14} vg, and an increase in the severity of meningeal mononuclear cell infiltrates in both females at 8.4×10^{13} vg (mild), when compared to control animals.

In the trigeminal ganglion, there was minimal axonal degeneration and mononuclear cell infiltrates in the male at 1.68×10^{14} vg.

In the peripheral nerves (sciatic, sural, and tibial), there was an increase in severity of axonal degeneration at 1.68x1014 vg in both sexes, compared to controls. This change was generally coupled with the presence of edema with or without mixed cell infiltrates dissecting through the nerve fibers and considered likely secondary to the inflammatory changes seen in the nearby biceps femoris (procedural-related changes, see below). Thus, the increase in severity of axonal degeneration seen in peripheral nerves was considered probably related to AAV9/AP4M1, but likely exacerbated by the edema/inflammation associated with experimental procedures (repetitive intramuscular injections).

Other microscopic findings observed were considered incidental, related to experimental procedures/immunosuppressant administration, of the nature commonly observed in this strain and age of monkeys, and/or were of similar incidence and severity in control and treated animals and, therefore, were considered unrelated to the administration of AAV9/AP4M1.

These included minimal to mild decreased lymphoid cellularity in the cervical and iliac lymph nodes and/or spleen that were seen with a similar incidence and severity in all groups, including controls, and considered secondary to the administration of immunosuppressants and unrelated to the administration of AAV9/AP4M1.

There was minimal axonal degeneration in the spinal cord and minimal mononuclear cell infiltrates in the brain (perivascular) and meninges, that were seen in several animals, including controls, and that were considered secondary to the procedure of intrathecal injection and unrelated to the administration of AAV9/AP4M1.

In the biceps femoris, microscopic findings of edema, necrosis/inflammation, myofiber degeneration/atrophy, and fibrosis (with gross correlates of swelling, thickness, dark/pale foci, and/or mottled discoloration) occurred with a similar incidence and severity across dose groups, including control animals. These findings were all considered secondary to the repetitive procedure of intramuscular injection of the immunosuppressant in the thigh, and unrelated to the administration of AAV9/AP4M1.

In the iliac lymph nodes, minimal or mild sinus histiocytosis was also noted with a similar incidence and severity across dose groups, including controls, and was considered to be a physiological response to the inflammatory changes in the biceps femoris.

8. CONCLUSION

In conclusion, administration of AAV9/AP4M1 by a single intrathecal injection in monkeys at $8.4x10^{13}$ and $1.68x10^{14}$ vg was well tolerated. Adverse findings at $1.68x10^{14}$ vg included axonal or neuronal degeneration noted microscopically in the spinal cord (including the injection site), lumbar dorsal root ganglion (DRG), dorsal nerve roots, brain, trigeminal ganglion, and peripheral nerves (sciatic, sural, and tibial), with associated decreases in nerve conduction velocity and neurological effects. Based on these results, the no-observed-adverse-effect level (NOAEL) was considered to be $8.4x10^{13}$ vg.

Furthermore, it was concluded that AAV9 delivered intrathecally can achieve broad distribution as well as expression across the nervous system and peripheral organs without generating any immune response under the immunosuppressant regimen, although the level of gene transfer in the brain is sub-saturating with a minority of cells receiving the transgene.

9. REFERENCES

Bee JS, Zhang Y, Phillippi MK, et al. Impact of Time Out of Intended Storage and Freeze-thaw Rates on the Stability of Adeno-associated Virus 8 and 9, Journal of Pharmaceutical Sciences, 111(2) (2022).

Gruntman AM, Su L, Su Q, et al. Stability and Compatibility of Recombinant Adeno-Associated Virus Under Conditions Commonly Encountered in Human Gene Therapy Trials, Human Gene Therapy Methods, 26(2): 71–76 (2015).

STUDY PLAN AMENDMENT 6

Test Facility Study No. 5550014

Sponsor Reference No. UTSW-Gray-SPG50-004

A Single-Dose Study of AAV9/AP4M1 by Intrathecal Injection in Immunosuppressed Monkeys

GLP

SPONSOR:
University of Texas Southwestern Medical Center
5323 Harry Hines Blvd
Dallas, TX 75390-9056
USA

TEST FACILITY:
Charles River Laboratories Montreal ULC
Senneville Site (CR-SEN)
22022 Transcanadienne
Senneville, QC H9X 3R3
Canada

Appendix 1

SUMMARY OF CHANGES AND JUSTIFICATIONS

Study Plan effective date: 29 Jun 2021

Note: When applicable, additions are indicated in bold underlined text and deletions are indicated in bold strikethrough text in the affected sections of the document.

Item or Section(s)	Justification
**Amendment 1**	**Date: 12 Jul 2021**
Title page,<br>4. RESPONSIBLE PERSONNEL,<br>18. REGULATORY COMPLIANCE,<br>19. QUALITY ASSURANCE – *NEW SECTION*	As requested by the agency, this study will be performed as per GLP; appropriate details were added throughout the document.
2. PROPOSED STUDY SCHEDULE	To correct the date of animal transfer to reflect the actual date animals were transferred onto the study.<br>An audited comprehensive Draft report will be provided; report name and dates are updated.
4. RESPONSIBLE PERSONNEL	Due to the absence of Stefania Cinquino, a new Study Director will be assigned effective immediately. The test facility management was also updated.<br>To assign the IS for cytokines and splenocyte preparation.<br>The bioanalytical portion of the study will be performed non-GLP, therefore, the exception was added.
7.1. Immunosuppression Material	Due to the availability of the methylprednisolone acetate, the 40 mg/mL concentration will be used in this study.<br>The supplier and storage of Methylprednisolone acetate was updated.
7.3. Immunosuppressant Preparation Details	Methylprednisolone acetate will be used as is; section updated.<br>The storage of Methylprednisolone acetate was updated.
10.1. Administration of Test and Reference Item	Due to the dose volume, particularly in Group 3, 1 mL of CSF will be removed prior to dosing for all groups.
10.2. Administration of Anti-Inflammatory	To update the number of days of meloxicam administration, as per CRL-SEN SOP.
10.3. Administration of Immunosuppressant Material	The dose volume and concentration of methylprednisolone acetate was updated based on the concentration ordered.<br>The days of rapamycin administration were updated based on the actual day the animals were transferred onto the study, which are considered to be a sufficient number of days for immunosuppression.<br>Due to possible animal replacements; the spare will be administered rapamycin; section updated.<br>To specify that the rapamycin administration will be performed at approximately the same time each day; section updated.
10.3.1. Methylprednisolone Succinate,<br>10.3.2. Methylprednisolone Acetate	To remove reference to warming the immunosuppressants at ambient temperature as these are stored at room temperature.

Appendix 1

Item or Section(s)	Justification
10.3.3. Rapamycin	The days of rapamycin administration were updated based on the actual day the animals were transferred onto the study, which are considered to be a sufficient number of days for immunosuppression.<br>To specify that the rapamycin administration will be performed at approximately the same time each day; section updated.
13.3. Bioanalytical Sample Analysis	To include details for the collection and processing of CSF samples
14.7. Tissue Collection for Bioanalysis	Section revised to:<br>include animals which are pre-terminally euthanized, to list tissue exceptions where 3 samples are not possible due to the size of the tissue, include the optic nerve which was inadvertently omitted, include PCR techniques for tissue collection, and remove the need for sectioning each punch as duplicate samples are already being provided (right and left hemispheres).
14.8. Tissue Collection for Bioanalysis – *NEW SECTION*	Section was added to detail the regions of the brain to be collected and to which punch and slice numbers these correspond.
15.3. Pathology Peer Review	To correct the section referenced.
22. REPORTING	A comprehensive Draft report will be provided; section updated.
23.2. Justification of Route and Dose Levels	To include reference to the rats study conducted at CRL-SEN.
23.3 Guidelines for Study	To include appropriate guidelines for the study design.
ATTACHMENT A	To remove the side for brain collection; as the brain is being sectioned.
ATTACHMENT B	To correct the sections referenced.
ATTACHMENT D	To include a placeholder for the brain slicing diagram
ATTACHMENT E	To include a placeholder for the spinal cord sampling diagram
**Amendment 2**	**Date: 10 Aug 2021**
2. PROPOSED STUDY SCHEDULE	Based on feedback from the regulatory agency, the study duration is extended to 94 days. The study dates and reporting schedule are adjusted accordingly.
4. RESPONSIBLE PERSONNEL	Due to their return, the originally assigned Study Director will assume the Study Director responsibilities effective immediately.<br>Due to workload, the pathologist will be reassigned.
8.2. Animal Identification	To include an alternate method of identification.
10.3. Administration of Immunosuppressant Material, 10.3.2. Methylprednisolone Acetate, 10.3.3. Rapamycin	The duration of treatment with each agent is updated based on the extended duration of the study.
11.1. Nerve Conductivity Velocity (NCV)	The occasions are adjusted based on the extended study duration, and to specify that the postdose evaluations will be performed in a blinded manner.
12.1. Sample Collection	To include an additional clinical pathology occasion based on the extended study duration.
13.2. Cytokine Sample Analysis	To remove the KC-like as this cytokine is not validated for monkeys. Additionally, to include the details for IP-10 analysis as this cytokine is analyzed with a separate assay.
14. TERMINAL PROCEDURES	Based on feedback from the regulatory agency, the study duration is extended to 94 days.; necropsy day is adjusted accordingly.
14.7. Tissue Collection for Bioanalysis	Tissues for bioanalysis will also be collected from animals found dead; section updated accordingly.

Appendix 1

Item or Section(s)	Justification
14.8. Tissue Collection for Bioanalysis	The slice numbers indicated were updated to match the brain sampling diagram in Attachment D. In addition, the regions of the brain were re-ordered according to the slice number in which they are located.
15.3. Pathology Peer Review	As the pathologist was re-assigned (previously the peer review pathologist), the peer review pathologist was re-assigned as well.
17. COMPUTERIZED SYSTEMS	To include the software to be used for cytokine analysis.
18. REGULATORY COMPLIANCE	To specify that the nerve conduction velocity assessment is performed outside of GLP.
ATTACHMENT A	To clarify the sampling for the DRGs and spinal cord segments.
ATTACHMENT B	To clarify the sampling for the brain.
ATTACHMENT D	To include the brain sampling diagram.
ATTACHMENT E	To include the spinal cord sampling diagram.
**Amendment 3**	**Date: 24 Sep 2021**
2. PROPOSED STUDY SCHEDULE	To include an inlife summary memo.
7.1. Immunosuppression Material	To include an additional supplier for rapamycin.
11.1. Nerve Conductivity Velocity (NCV)	A potential effect on NCV was noted on Day 45, however, due to the limited number of animals, an additional occasion will be added to confirm the potential effect.
ATTACHMENT D	The punch numbers were updated based on the changes made in Amendment 2.
ATTACHMENT E	Due to formatting issues when converting the amendment to PDF, the spinal cord diagram became unreadable. The diagram is being re-amended.
**Amendment 4**	**Date: 12 Oct 2021**
4. RESPONSIBLE PERSONNEL	To include the PI for tissue gene expression.<br>To include an IS for clinical pathology.
14. TERMINAL PROCEDURES	The tissues for microscopic evaluation was updated as per Attachment A.
14.9. Splenocyte Sample Collection Analysis	To correct the analytical procedure.<br>To correct the referenced attachment.
15.2. Microscopic Evaluation	Based on the microscopic findings noted in the previously conducted rat study with the same test item, IHC will be performed on selected tissues.
ATTACHMENT A	Based on the microscopic findings noted in the previously conducted rat study with the same test item, additional tissues were added for microscopic evaluation.
ATTACHMENT C	To include the shipping details of the splenocytes.
**Amendment 5**	**Date: 20 Oct 2021**
4. RESPONSIBLE PERSONNEL	To include an IS for clinical pathology.<br>To include an PI for splenocyte analysis.
14.9. Splenocyte Sample Collection Analysis	To include the method for splenocyte analysis.
16. STATISTICAL ANALYSIS	To include the mean and standard deviation for NCV data.
18. REGULATORY COMPLIANCE	Tissue and splenocyte analysis will not be conducted as per GLP; exception was included.
ATTACHMENT A	To defined the abbreviation “DRG”.

Appendix 1

Item or Section(s)	Justification
**Amendment 6**	**Date: 22 Dec 2021**
14.7. Tissue Collection for Histopathology, Biodistribution, and Gene Expression,<br>14.8. Tissue Collection for Histopathology, Biodistribution, and Gene Expression	To rename the titles of these sections to accurately capture for which analyses the samples were collected.
15.3. Pathology Peer Review	To remove reference of the slides being shipped as the pathology peer review was done by a Test Facility pathologist.
21. RETENTION AND DISPOSITION OF RECORDS, SAMPLES, AND SPECIMENS	To include the disposition of all samples collected on study.
ATTACHMENT A	To remove reference to macroscopic abnormalities as these were not to be processed and evaluated microscopically.

TABLE OF CONTENTS

SUMMARY OF CHANGES AND JUSTIFICATIONS 2
1. OBJECTIVE 8
2. PROPOSED STUDY SCHEDULE 8
3. SPONSOR 8
4. RESPONSIBLE PERSONNEL 9
5. TEST MATERIALS 11
6. DOSE FORMULATION AND ANALYSIS 12
7. IMMUNOSUPPRESSION FORMULATION 12
8. TEST SYSTEM 13
9. HUSBANDRY 15
10. EXPERIMENTAL DESIGN 16
11. IN-LIFE PROCEDURES, OBSERVATIONS, AND MEASUREMENTS 19
12. CLINICAL PATHOLOGY 20
13. CYTOKINES COLLECTION AND ANALYSIS 22
14. TERMINAL PROCEDURES 23
15. MICROSCOPIC EVALUATION 27
16. STATISTICAL ANALYSIS 28
17. COMPUTERIZED SYSTEMS 29
18. REGULATORY COMPLIANCE 30
19. QUALITY ASSURANCE 30
20. AMENDMENTS AND DEVIATIONS 30
21. RETENTION AND DISPOSITION OF RECORDS, SAMPLES, AND SPECIMENS 31
- STUDY CLASSIFICATION 31
22. REPORTING 32
23. JUSTIFICATIONS AND GUIDELINES 32
24. ANIMAL WELFARE 33
25. REFERENCES 33
TEST FACILITY APPROVAL 34
SPONSOR APPROVAL 35

Appendix 1

ATTACHMENT A 36
ATTACHMENT B 39
ATTACHMENT C 41
ATTACHMENT E..46

Appendix 1

1. OBJECTIVE

The objectives of this study are to determine the potential toxicity, biodistribution, and gene expression of AAV9/AP4M1, for the treatment of Spastic Paraplegia Type 50 (SPG50) caused by the AP4M1 gene mutation, when given by a single intrathecal injection to monkeys, to evaluate the potential reversibility and/or progression of any findings, and to determine the potential impact of immunosuppressant (IMS) on the toxicity profile of this AAV9 vector.

2. PROPOSED STUDY SCHEDULE

Proposed study dates are listed below. Actual dates will be included in the Final Report.

Experimental Starting Date:	02 Jul 2021<br>(First date of study-specific data collection)
Experimental Completion Date:	09 May 2022<br>(Last date on which data are collected)
Animal Transfer:	30 Jun 2021
Initiation of Dosing:	13 Jul 2021
Completion of In-life:	14 Oct 2021<br>(Last date of necropsy)
In-life Summary Memo:	29 Oct 2021<br>(2 weeks following completion of in-life)
Audited Draft Report:	09 Dec 2021<br>(10 weeks following completion of in-life)
Final Report:	09 Apr 2022<br>(Expected date of Study Director signature of report. Target to be within 6 months of issue of Draft Report)

3. SPONSOR

Role/Phase	Name	Contact Information
Sponsor Representative	Steven Gray, PhD	University of Texas Southwestern Medical Center<br>5323 Harry Hines Blvd<br>University of Texas Southwestern Medical Center<br>Dallas, TX 75390<br>Tel: 214.648.0670<br>E-mail: steven.gray@UTsouthwestern.edu
Alternate Sponsor Representative	Roxana Ploski	University of Texas Southwestern Medical Center<br>5323 Harry Hines Blvd<br>University of Texas Southwestern Medical Center<br>Dallas, TX 75390<br>Tel: 214.648.9828<br>E-mail: roxana.ploski@UTsouthwestern.edu

Role/Phase	Name	Contact Information
Alternate Study Contact	Terry Pirovolakis	CureSPG50<br>6 Topham Road<br>Toronto, ON M4B 3K2<br>Canada<br>Tel:416.625.1933<br>E-mail: tpirovol@hotmail.com

4. RESPONSIBLE PERSONNEL

Role/Phase	Quality Assurance Program (QAP)	Name	Contact Information
Study Director	Charles River	Stefania Cinquino, BSc	Address as cited for Test Facility<br>Tel: 514.630.8200, ext 2151<br>E-mail: stefania.cinquino@crl.com
Test Facility Management	Charles River	Julie Douville, PhD	Address as cited for Test Facility<br>Tel: 514.630.8200, ext 8309<br>E-mail: julie.douville@crl.com
Test Facility QAP	Charles River	Nooshin Davani, BSc, RQAP-GLP	Address as cited for Test Facility<br>Tel: 514.630.8200, ext 2605<br>E-mail: nooshin.davani@crl.com
**Individual Scientist (IS)**			
Nerve Conduction Velocity	Charles River	Nataliya Sadekova, MSc, DSP	Address as cited for Test Facility<br>Tel: 514.630.8200, ext 2334<br>E-mail: nataliya.sadekova@crl.com
Cytokines	Charles River	Hycham Harouchi, BSc	Address as cited for Test Facility<br>Tel: 514.630.8200, ext 8847<br>E-mail: hycham.harouchi@crl.com
Pathology	Charles River	Elaine Debien, DVM, DES, MSc, DACVP	Address as cited for Test Facility<br>Tel: 819.346.8200, ext 7016<br>E-mail: elaine.debien@crl.com
Immuno-toxicology Splenocyte Preparation	Charles River	Philippe Rousseau, PhD	Address as cited for Test Facility<br>Tel: (514) 630-8200, ext 2154<br>E-mail: philippe.tousseau@crl.com
Clinical Pathology	Charles River	Florence Poitout, DVM, DACVP, DECVCP	Address as cited for Test Facility<br>Tel: 514.630.8200, ext 2201<br>E-mail: florence.poitout@crl.com
**Principal Investigator (PI)**			
Tissue Biodistribution Analysis (Bioanalysis) [a]	N/A	Steven Gray, PhD	University of Texas Southwestern Medical Center<br>5323 Harry Hines Blvd<br>University of Texas Southwestern Medical Center<br>Dallas, TX 75390<br>Tel: 214-648-0670<br>E-mail:<br>steven.gray@UTsouthwestern.edu

Appendix 1

**Role/Phase**	Quality Assurance Program (QAP)	Name	Contact Information
Tissue Gene expression Analysis [a]	Test Site	Steven Gray, PhD	University of Texas Southwestern Medical Center<br>5323 Harry Hines Blvd<br>University of Texas Southwestern Medical Center<br>Dallas, TX 75390<br>Tel: 214-648-0670<br>E-mail:<br>steven.gray@UTsouthwestern.edu
Splenocyte Analysis [a]	Test Site	Steven Gray, PhD	University of Texas Southwestern Medical Center<br>5323 Harry Hines Blvd<br>University of Texas Southwestern Medical Center<br>Dallas, TX 75390<br>Tel: 214-648-0670<br>E-mail:<br>steven.gray@UTsouthwestern.edu

[a] Sponsor.

Each IS and PI is required to report any deviations or other circumstances that could affect the quality or integrity of the study to the Study Director in a timely manner for authorization/acknowledgement. Each IS and PI, with the exception of those listed below, will provide a report addressing their assigned phase of the study, which will be included as an appendix to the Final Report.

The IS phase report will include the following:

- A listing of critical computerized systems used in the conduct and/or interpretation of the assigned study phase

The IS for the Immunotoxicology Splenocyte Preparation phase will not provide a formal report.

The PI phase report will include the following:

- The archive site for all records, samples, specimens and reports generated from the phase or segment (alternatively, details regarding the retention of the materials may be provided to the Study Director for inclusion in the Final Report)
- A listing of critical computerized systems used in the conduct and/or interpretation of the assigned study phase

5. TEST MATERIALS

5.1. Test and Reference Item Characterization

A Certificate of Analysis or equivalent documentation will be provided for inclusion in the Final Report (if available).

5.2. Test Item Identification

Test Item Identification

	Test Item
**Identification:**	AAV9/AP4M1
**Alternate Identification:**	rAAV9.AP4M1
**Batch/Lot No.:**	T-GEMINIS-033
**Expiration/Retest Date:**	22 Dec 2021 (1-year stability time point)<br>Stability concomitant to the study
**Physical Description:**	Colorless, clear to slightly opalescent, free of visible particles
**Concentration:**<br>**(based on ddPCR results)**	5.43E13 vg/mL
**Storage Conditions (temperature set to maintain):**	-80ºC<br>(upon thawing of a vial, it may be stored at 4ºC and used on a subsequent dosing day)
**Provided by:**	Sponsor

5.3. Reference Item/Vehicle Identification

Reference Item/Vehicle Identification

	Reference Item
**Identification:**	PBS containing 5% D-sorbitol and 0.001% pluronic F-68
**Storage Conditions (temperature set to maintain):**	-80°C
**Provided by:**	Sponsor

5.4. Test and Reference Item Inventory and Disposition

Records of the receipt, distribution, storage, and disposition of test materials will be maintained. All unused Sponsor-supplied bulk test materials, with the exception of reserve samples, will be returned to the Sponsor following issuance of the Draft Report unless otherwise requested (documentation will be retained in the study record). An earlier shipment of these materials may also be requested and authorized by the Study Director and Sponsor. See Shipment of Samples and Study Records (Attachment C) for shipping details.

5.5. Safety

The safety precautions for the test item and dose formulations will be documented in a Test Material Safety Data Sheet (TMSDS) based on the SDS or similar document.

6. DOSE FORMULATION AND ANALYSIS

6.1. Preparation of Formulations

Dose formulations will be divided into aliquots, where required, and dispensed on each dosing occasion.

Preparation Details

Dose Formulation	Frequency of Preparation	Storage Conditions (temperature set to maintain)
Reference Item	Used as received	4°C
Test Item	At least once	4°C

Any residual volumes from each dosing occasion will be will be shipped back to the Sponsor; see Attachment C. Residual volumes will be archived by the Sponsor and will be kept for potential future analysis. Analysis, if conducted, will be added by amendment.

6.2. Preparation Details

Dosing formulations will be prepared under a laminar flow hood using clean procedures.

Dosing formulations will be prepared based on Sponsor's instructions at appropriate concentrations to meet dose level requirements.

7. IMMUNOSUPPRESSION FORMULATION

7.1. Immunosuppression Material

Immunosuppression (IMS) Identification

	IMS 1	IMS 2	IMS 3
**Identification:**	Methylprednisolone succinate	Methylprednisolone acetate	Rapamycin
**Alternate Identification:**	Solu-Medrol	Depo-Medrol	-
**Physical Description:**	Liquid	Liquid	Powder
**Concentration:**	125 mg/mL	40 mg/mL	N/A
**Storage Conditions (temperature set to maintain):**	21°C Protected from light	21°C	-20°C Protected from light
**Provided by:**	Test Facility	Test Facility	Test Facility
**Supplier:**	Pfizer	Zoetis Canada or Pfizer	Toronto Research Chemicals or Alfa Aesar

N/A = Not applicable

7.2. Immunosuppression Vehicle

Immunosuppressants Vehicle Identification

	Vehicle for Solu-Medrol	Vehicle Components for Rapamycin		
**Identification:**	0.9% sodium chloride injection, USP	N,N-Dimethylacetamide	Tween-80	Polyethylene Glycol
**Storage Conditions (temperature set to maintain):**	21°C	21°C	21°C Protected from light	21°C
**Provided by:**	Test Facility	Test Facility	Test Facility	Test Facility

7.3. Immunosuppressant Preparation Details

IMS dose formulations will be divided into aliquots, where required, and dispensed on each dosing occasion.

Preparation Details

Dose Formulation	Frequency of Preparation	Storage Conditions (temperature set to maintain)
Methylprednisolone succinate	At least weekly	21°C protected from light
Methylprednisolone acetate	Used as is	21°C
Rapamycin	At least weekly	4°C Protected from light (in amber glass vials)

IMS dosing formulations will be prepared based on Sponsor's instructions at appropriate concentrations to meet dose level requirements.

7.4. Sample Collection and Analysis

Samples for test or reference item dose formulation and/or IMS dose formulation analysis will not be collected by the Test Facility.

8. TEST SYSTEM

Species:	Monkey
Strain:	Cynomolgus
Condition:	Purpose-bred, naïve
Source:	CR-SEN Colony, original source will be documented in the Final Report
Continent of Origin:	Vietnam
Number of Males to be Assigned:	2

Number of Females to be Assigned:	4 (plus 1 alternate)
Target Age at the Initiation of Dosing:	2 to 4 years of age
Target Weight at the Initiation of Dosing:	1.5 to 6 kg

The actual age and weight of the animals at the initiation of dosing will be listed in the Final Report.

8.1. Animal Screening

Method:	All animals used on study will have documentation to confirm one negative tuberculosis (TB) test. Additional TB testing may be performed as required.

8.2. Animal Identification

Method:	Subcutaneously implanted electronic identification chip or tattoo.

8.3. Environmental Acclimation

Method:	At least 2 weeks will be allowed between animal transfer and the start of dosing in order to accustom the animals to the laboratory environment.

8.4. Selection, Assignment, Replacement, and Disposition of Animals

Selection:	Pre-established pairs will be assigned to groups using a computerized-based randomization procedure. Males and females will be randomized separately.
Replacement:	Before the initiation of dosing, any assigned animals considered unsuitable for use in the study will be replaced by alternate animals. After initiation of dosing, study animals may be replaced during the replacement period with alternate animals in the event of accidental injury, non-test item-related health issues, or similar circumstances. The alternate animals may be used as replacements on the study within 2 days. General in-life assessments will include alternate animals until released from study.
Disposition:	The disposition of all animals will be documented in the study records.

9. HUSBANDRY

9.1. Housing

Housing:	Group-housed (up to 3 animals of the same sex and same dosing group together).
Caging:	Stainless steel cages with stainless steel mesh floor
Cage Identification:	Color-coded cage card indicating study, group, animal/tattoo number(s), and sex.

Housing set-up is as described in the *Guide for the Care and Use of Laboratory Animals* (National Research Council, 2011) Animals will be separated during designated procedures/activities or will be separated as required for monitoring and/or health purposes, as deemed appropriate by Study Director and/or Clinical Veterinarian.

9.2. Animal Enrichment

Psychological/ Environmental Enrichment:	Animals will be socially housed and will be provided with items such as perches, floor enrichment devices, foraging devices and/or suspended devices, except during study procedures/activities. Additional enrichment, such as music, natural sounds or color videos films will also be provided. Each animal will be offered food supplements (such as certified treats, fruit/vegetables and/or Foraging Crumbles™).

9.3. Environmental Conditions

The targeted conditions for animal room environment will be as follows:

Temperature:	23 ±3°C
Humidity:	30% to 70%
Light Cycle:	12 hours light and 12 hours dark (except during designated procedures)

9.4. Food

Diet:	Lab Diet Certified Primate Diet 5048
Type:	Pellets
Frequency/Ration:	Twice daily, except during designated procedures. The chow will be provided in amounts appropriate for the size and age of the animals.
Analysis:	Results of analysis for nutritional components and environmental contaminants are provided by the supplier and are kept on file at the Test Facility. It is considered that there are no known contaminants in the feed that would interfere with the objectives of the study.

9.5. Water

Type: Municipal tap water, treated by reverse osmosis and ultraviolet irradiation.

Frequency/Ration: Freely available to each animal via an automatic watering system (except during designated procedures).

Analysis: Periodic analysis of the water is performed, and results of these analyses are kept on file at the Test Facility. It is considered that there are no known contaminants in the water that would interfere with the outcome of the study.

9.6. Veterinary Care

Veterinary care will be available throughout the course of the study and animals will be examined by the veterinary staff as warranted by clinical signs or other changes. In the event that animals show signs of illness or distress, the responsible veterinarian may make initial recommendations about treatment of the animal(s) and/or alteration of study procedures, which must be approved by the Study Director or Scientific designate. Treatment of the animal(s) for minor injuries or ailments may be approved without prior consultation with the Sponsor representative when such treatment does not impact fulfillment of the study objectives. If the condition of the animal(s) warrants significant therapeutic intervention or alterations in study procedures, the Sponsor representative will be contacted, when possible, to discuss appropriate action. If the condition of the animal(s) is such that emergency measures must be taken, the Study Director (or Scientific designate) and/or veterinarian will attempt to consult with the Sponsor representative prior to responding to the medical crisis, but the Study Director (or Scientific designate) and/or veterinarian has authority to act immediately at his/her discretion to alleviate suffering. The Sponsor representative will be fully informed of any such events.

10. EXPERIMENTAL DESIGN

Experimental Design

Group No.	Test Material	Dose Level (vg)	Dose Volume (mL)	Dose Concentration (vg/mL)	No. of Animals Main Study Males	No. of Animals Main Study Females
1	Reference Item	0	1	0	1	1
2	AAV9/AP4M1	8.4×10^{13}	1.55	5.43×10^{13}	-	2
3	AAV9/AP4M1	1.68×10^{14}	3.10	5.43×10^{13}	1	1

10.1. Administration of Test and Reference Item

Dose Route: Percutaneous intrathecal injection (slow bolus, target rate of 1 mL/min) at the lumbar level (target L5-L6 space, L4-L5 may also be used if necessary)

Frequency: Once

Duration:	1 day
Method:	The first day of dosing will be designated as Day 1.<br>Animals will be food deprived at least 6 hours prior to dosing. Animals will be anesthetized with a ketamine/dexmedetomidine/glycopyrrolate cocktail. Tracheal intubation will be performed, oxygen will be provided and anesthesia will then be maintained using isoflurane, if required.<br>Prior to dose administration, a 1-mL CSF sample will be withdrawn, as per Section 13.3.<br>Dose formulations will be allowed to warm up to room temperature for at least 30 minutes prior to dosing.<br>Percutaneous intrathecal injection will be performed using atraumatic spinal needle (e.g. B Braun, 25 Gauge, 1 inch); actual needle will be recorded in the raw data. Animals will remain in a Trendelenberg position for at least 15 minutes after the dosing is completed prior to the administration of the reversal agent (atipamezole), when required.

10.2. Administration of Anti-Inflammatory

On days of dose administration and/or CSF collections, animals will receive an anti-inflammatory (Meloxicam) subcutaneously prior to dosing/CSF collection and then orally daily, starting the morning after dosing/CSF collection, for a total of 3 doses.

10.3. Administration of Immunosuppressant Material

Immunosuppressant (IMS) Information

Group No.	Test Material	Study Day	Method	Dose Level (mg/kg/dose)	Dose Volume (mL/kg)	Dose Concentration (mg/mL)	Frequency
1 to 3[a]	Methylprednisolone succinate	1	IV infusion for 30 min	10	0.5	20	Once on Day 1 only
	Methylprednisolone acetate	1 to 93	IM	1	0.025 (25 µL/kg)	40	SID; first dose morning of surgery
	Rapamycin	-12 to 93	IM	0.01	0.067	0.15	BID, 12 hr ± 3 hrs apart[b]

min = minute; IV = intravenous; IM = intramuscular; SID = once daily; BID = twice daily
[a] Including spares for the rapamycin administration.
[b] At approximately the same time each day.

10.3.1. Methylprednisolone Succinate

Dose Route: Intravenous infusion into a lateral tail vein (or alternate peripheral vein if necessary) using an infusion pump connected to a temporary indwelling catheter (Abbocath®)

Following the end of infusion, the Abbocath® will be flushed with 0.5 mL of 0.9% sodium chloride for injection.

Frequency: Once on Day 1 for 30 minutes, prior to dosing (within 4 hours of dosing)

Infusion Rate: 1 mL/kg/h

Method: The animals will be temporarily restrained for dose administration and will not be sedated.

10.3.2. Methylprednisolone Acetate

Dose Route: Intramuscular injection into the lateral compartment of the thigh

Duration: From Days 1 to Day 93

Frequency: Once daily; first dose the morning of dosing (within 4 hours of dosing)

Method: The animals will be temporarily restrained for dose administration and will not be sedated.

10.3.3. Rapamycin

Dose Route: Intramuscular injection into the lateral compartment of the thigh

Duration:	From Days -12 to Day 93
Frequency:	Twice daily; 12 hr ± 3 hrs apart (at approximately the same time each day)
Method:	Dose formulations will be allowed to warm up at ambient temperature for at least 30 minutes prior to dosing, as appropriate. The animals will be temporarily restrained for dose administration and will not be sedated.

11. IN-LIFE PROCEDURES, OBSERVATIONS, AND MEASUREMENTS

General In-life Assessments – Main Study Animals

Parameter	Frequency	Comments
**Mortality/ Moribundity Checks**[a]	Twice daily (morning and afternoon) starting upon arrival through termination.	Animals will be observed within their cage unless removal is necessary for identification or confirmation of possible findings.
**Detailed Clinical Observations**[a]	Weekly starting Day -1, and at least every 2 weeks during the prestudy period.	Animals are removed from the cage
**Individual Body Weights**[a]	Weekly starting Day -1, and at least every 2 weeks during the prestudy period.	Fasted weight on the day of necropsy. No terminal body weights collected from animals found dead or preterminally euthanized.
**Food Evaluation**[a]	Once daily; from at least Week -1 and throughout the study	Qualitative
**Neurological Examinations**[a]	Once pretreatment, on Day 1 at 4 to 6 hours postdose, on Days 2, 7 and 28, and at the end of the observation period.	Assessment for general attitude and motor function, postural reactions, and cranial and spinal nerve functions; performed on unanesthetized animals.

[a] Minimum required frequency for this parameter indicated

11.1. Nerve Conductivity Velocity (NCV)

Frequency: Once prestudy and on Days 45, 77, and 92; to be performed blinded on Days 45, 77, and 92.

Anesthesia: An intramuscular injection of ketamine, glycopyrrolate and dexmedetomidine will be administered following an appropriate fasting period. Following the completion of the assessment, a reversal agent, atipamezole, will be administered if required.

Evaluation: Peroneal motor: NCV and Amplitude
Sural sensory: NCV and Amplitude
Cauda equina: Onset latency

12. CLINICAL PATHOLOGY

12.1. Sample Collection

Clinical Pathology Sample Collection – Main Study Animals

Group Nos.	Occasion/ Time Point	Hematology	Coagulation	Clinical Chemistry	Urinalysis	Additional Serum
All animals	Pretreatment	X	X	X	X	X
1 to 3	Day 2	X	X	X	X	X
1 to 3	Week 1	X	X	X	X	X
1 to 3	Week 2	-	-	X	-	X
1 to 3	Week 3	-	-	X	-	X
1 to 3	Week 4	X	X	X	X	X
1 to 3	Week 8	X	X	X	X	X
1 to 3	End of recovery	X	X	X	X	X
Unscheduled euthanasia (when possible)		X	X	X	-	X
**Overnight Fasting:**		-	-	Yes	Yes	-
**Method/Comments:**		Venipuncture	Venipuncture	Venipuncture	morning collection while water deprived	Venipuncture
**Target Volume (mL)[a]:**		0.7	1.2	0.7	As available	1.2
**Anticoagulant:**		EDTA	Sodium citrate	None, in SST	-	None, in SST
**Special Requirements:**		-	-	-	-	-
**Processing:**		None	Plasma	Serum	-	Serum

X = Sample to be collected; - = Not applicable; SST = Serum separator tube.
[a] Additional samples may be obtained (e.g., due to clotting of non-serum samples) if permissible sampling frequency and volume are not exceeded.

12.2. Hematology

Hematology Parameters

Red blood cell count	Platelet count
Hemoglobin concentration	White blood cell count
Hematocrit	Neutrophil count (absolute)
Mean corpuscular volume	Lymphocyte count (absolute)
Red blood cell distribution width	Monocyte count (absolute)
Mean corpuscular hemoglobin concentration	Eosinophil count (absolute)
Mean corpuscular hemoglobin	Basophil count (absolute)
Reticulocyte count (absolute)	Large unstained cells (absolute)

A blood smear will be prepared from each hematology sample and may be examined for confirmation of hematology results.

12.3. Coagulation

Coagulation Parameters

Activated partial thromboplastin time	Prothrombin time
Fibrinogen	Sample quality

12.4. Clinical Chemistry

Clinical Chemistry Parameters

Alanine aminotransferase	Total protein
Aspartate aminotransferase	Albumin
Alkaline phosphatase	Globulin
Gamma-glutamyltransferase	Albumin/globulin ratio
Creatine kinase	Glucose
Total bilirubin[a]	Cholesterol
Urea nitrogen	Triglycerides
Creatinine	Sodium
Calcium	Potassium
Phosphorus	Chloride
	Sample quality

[a] When total bilirubin is > 1.0 mg/dL, direct bilirubin will also be measured and indirect bilirubin will be calculated.

12.5. Urinalysis

Urinalysis Parameters

Color	Protein[a]
Appearance/Clarity	Glucose[a]
Specific gravity	Bilirubin
Volume	Ketones
pH	Blood

[a] Semi-quantitative measurement

12.6. Additional Serum Sample Processing

The samples will be centrifuged and the resultant serum will be separated, transferred to 2 uniquely labeled polypropylene tubes, and frozen immediately over dry ice or in a freezer set to maintain -80°C within 2 hours of collection.

Samples will be shipped to the Sponsor for archival. Analysis, if conducted, will be added by amendment.

12.7. Bone Marrow Smear Evaluation

Bone marrow smears will be collected and prepared as described in Attachment A and may be examined for confirmation of results.

13. CYTOKINES COLLECTION AND ANALYSIS

Cytokines Sample Collection

Group Nos.	Time Postdose on Day 1			
	Predose	30 min	4 hr	24 hr
1 to 3	X	X	X	X
Unscheduled euthanasia (when possible)	X			

**Method/Comments:**	Venipuncture
**Target Volume[a]:**	0.5
**Anticoagulant:**	K_2EDTA
**Special Requirements:**	-
**Processing:**	Plasma

X = Sample to be collected; min = Minute; hr = Hour.

[a] Additional blood samples may be obtained (e.g., due to sample quality) if permissible sampling frequency and blood volume are not exceeded.

13.1. Cytokine Sample Processing

The samples will be centrifuged and the resultant plasma will be separated, transferred to 2 uniquely labeled polypropylene tubes (target 1 x 75 µL + leftover), and frozen immediately over dry ice or in a freezer set to maintain -80°C within 2 hours of collection.

13.2. Cytokine Sample Analysis

Analysis for cytokines (IL-6, IL-8, IL-10, MCP-1, and TNF-α) will be conducted using a multiplex bead-based method (Luminex). The procedures to be followed during the course of this study along with the assay acceptance criteria will be detailed in CR-SEN analytical procedure AP.BMK.mkpCyt.xx, in which “xx” denotes the version number. The cytokine method was validated under CR-SEN Study No. 3600083, however some cytokines have method limitations when used together in a multiplex panel. These limitations will be taken into consideration and discussed in the interpretative phase report.

IP-10 will also be analyzed using an ELISA assay. The procedures to be followed during the course of this study along with the assay acceptance criteria will be detailed in CR-SEN analytical procedure AP.BMK.mkpIP10.xx, in which “xx” denotes the version number. The

ELISA method was validated under CR-SEN Study No. 3600698. The results from this analysis will also be included in the interpretative report for the cytokines analysis.

13.3. Bioanalytical Sample Analysis

Bioanalytical Sample Collection - Blood

**Group Nos.**	**Prestudy**
1 to 3	X
**Method/Comments:**	Venipuncture
**Target Volume[a]:**	1
**Anticoagulant:**	None, in SST
**Special Requirements:**	None
**Processing:**	Serum

X = Sample to be collected.
[a] Additional blood samples may be obtained (e.g., due to sample quality) if permissible sampling frequency and blood volume are not exceeded.

The samples will be centrifuged and the resultant serum will be separated, transferred to 2 uniquely labeled polypropylene tubes, and frozen immediately over dry ice or in a freezer set to maintain -80°C.

Bioanalytical Sample Collection - CSF

Group Nos.	Predose
1 to 3	X
Target Volume (mL):	1[a]
At least 6 hours Fasting:	Yes
Anticoagulant:	None, in polypropylene tubes
Special Requirements:	Placed on wet ice after collection

X = Sample to be collected.
[a] Split into 2 approximately equal aliquots

The CSF samples will be placed on dry ice and then transferred in a freezer set to maintain -80°C.

Samples will be kept for possible future analysis of anti-AAV9 antibodies using a cell-based neutralizing antibody assay.

14. TERMINAL PROCEDURES

Terminal procedures are summarized in the following tables:

Replaced, Found Dead, and Unscheduled Euthanasia Animals

Animals	Necropsy Procedures			Histology Processing	Microscopic Evaluation
	Necropsy	Tissue Collection	Organ Weights		
Animals replaced prestudy	X	Standard Diagnostic List	-	-	-
Animals replaced after dosing start	X	Full List[a]	-	-	-
Unscheduled deaths after dosing start	X	Full List[a]	-	Full List[a]	Full List[a]

Main Study Animals

Group No.	Scheduled Euthanasia Day	Necropsy Procedures			Histology Processing	Microscopic Evaluation
		Necropsy	Tissue Collection	Organ Weights		
1	94	X	Full List[a]	Full List[a]	Full List[a]	Select Tissues[b]
2						
3						

X = Procedure to be conducted; - = Not applicable.

[a] See Tissue Weighing, Collection, Processing and Evaluation table in Attachment A for list of tissues applicable to each procedure.

[b] Brain, spinal cord (cervical, thoracic, lumbar), dorsal root ganglia (cervical, thoracic, lumbar), trigeminal ganglion, draining lymph nodes (deep cervical and iliac), liver, spleen, kidney, heart, skeletal muscle (gastrocnemius and biceps femoris), nerves (optic, sciatic, sural, and tibial).

14.1. Method of Euthanasia

Exsanguination by incision of the axillary or femoral arteries following anesthesia by intravenous injection of sodium pentobarbital, unless deemed inappropriate by the Study Director and/or the clinical veterinarian. A sedative, ketamine HCl for injection, USP will be administered by intramuscular injection before animals are transported from the animal room to the necropsy area.

14.2. Unscheduled Euthanasia

Main Study animals to be euthanized for humane reasons before the scheduled time will undergo sample collection for evaluation of clinical pathology parameters, cytokine analysis, and bioanalysis, if possible as specified in Section 12.1, 13, and 14.7.

Tissues from animal replaced after the start of dosing will be retained (as per Tissue Collection and Preservation section) and any data generated will not be included in the report unless deemed appropriate by the Study Director.

14.3. Scheduled Euthanasia

Main Study animals surviving until scheduled euthanasia will be food deprived overnight. When possible, the animals will be euthanized rotating across dose groups such that similar numbers of animals from each group, including controls, will be necropsied throughout the day.

14.4. Necropsy

Animals as detailed in the Terminal Procedures table will be subjected to a complete necropsy examination, which will include evaluation of the carcass and musculoskeletal system; all external surfaces and orifices; cranial cavity and external surfaces of the brain; and thoracic, abdominal, and pelvic cavities with their associated organs and tissues. Necropsy examinations will be performed by qualified personnel with appropriate training and experience in animal anatomy and gross pathology. A veterinary pathologist, or other suitably qualified person, will be available for consultation.

Images may be generated for illustration of or consultation on gross observations. These images will not be used for data generation or interpretation, and will not be archived or included in the final report.

14.5. Organ Weights

The organs detailed in the Terminal Procedures and Tissue Weighing, Collection, Processing and Evaluation (Attachment A) tables will be weighed at necropsy. Paired organs will be weighed together. In the event of gross abnormalities, in addition to the combined weight, the weight of each organ of a pair may be taken and entered as a tissue comment. Organ weight as a percent of body weight (using the terminal body weight) and organ weight as a percent of brain weight will be calculated.

14.6. Tissue Collection and Preservation

Representative samples of tissues will be collected and preserved in 10% neutral buffered formalin, except for tissues requiring alternate fixatives as defined by standard operating procedures, as detailed in the Terminal Procedures and Tissue Weighing, Collection, Processing and Evaluation (Attachment A) tables. Additional tissue samples may be collected to elucidate abnormal findings.

14.7. Tissue Collection for Histopathology, Biodistribution, and Gene Expression ~~Bioanalysis~~

For all animals, 3 samples of 4x4x4 mm each as applicable, except for testis/ovary, nerve optic and trigeminal ganglion where 1 sample will be collected and DRG where 2 samples/segment will be collected where possible, of the spinal cord (cervical, lumbar, and thoracic), dorsal root ganglia (cervical, lumbar, and thoracic), liver, spleen, kidney, lung, skeletal muscles (gastrocnemius and biceps femoris), optic nerve, sciatic nerve, tibial nerve, trigeminal ganglion, testis/ovary, thymus, and heart will be collected, weighed, placed in PCR clean polypropylene tubes, flash frozen in liquid nitrogen, placed on dry ice, and transferred to a freezer set to maintain -80°C until shipment. Clean removal techniques will be used.

For all animals, the brain (cerebellum, hippocampus, cortex [frontal, parietal, occipital, and temporal lobes], striatum, thalamus, midbrain, pons, medulla) of each animal in all groups will be sectioned at 4-mm coronal slice thickness (see Attachment B) using clean removal technique.

The second slice and every other slice thereafter will be fixed in 10% neutral buffered formalin for histopathological evaluation. The third slice and every other slice thereafter will be used to collect tissue punches (8 x 8 x 4 mm, when possible) for bioanalysis (BA) according to the table in Section 14.8. The sampling of brain slices for histopathology and BA analysis is summarized in Attachment D. All punches for BA will be weighed (before freezing). Tissue punches for BA will be placed in PCR clean vials, flash frozen in liquid nitrogen, placed on dry ice and stored at -80°C until analysis.

When possible, the order of tissue collection will be as follows: spleen, liver, kidney, brain then remaining tissues.

Tissues for histopathology will be collected prior to sample collection for any other parameters.

The samples will be shipped to the University of Texas Southwestern Medical Center, see Attachment B. The bioanalytical laboratory will be notified before shipment of the samples. Samples will be stored at the bioanalytical laboratory in a freezer set to maintain -60ºC or below until analysis.

Samples will be analyzed for vector genome biodistribution and transgene expression by qPCR. All analytical work will be conducted by the Sponsor, using an analytical method developed and qualified by that laboratory.

14.8. Tissue Collection for Histopathology, Biodistribution, and Gene Expression ~~Bioanalysis~~

Tissue Collection - Brain

Slice #	Punch #	Description	Hemisphere
3	1	Frontal Cortex	Right
3	2	Frontal Cortex	Left
7	3	Striatum	Right
7	4	Striatum	Left
9	5	Parietal Cortex	Right
9	6	Parietal Cortex	Left
9	7	Temporal cortex	Right
9	8	Temporal cortex	Left
9	9	Hippocampus	Right
9	10	Hippocampus	Left
9	11	Thalamus	Right
9	12	Thalamus	Left
11	13	Pons	Right
11	14	Pons	Left
11	15	Midbrain	Right
11	16	Midbrain	Left
13	17	Medulla	Right
13	18	Medulla	Left
15	19	Cerebellum	Right
15	20	Cerebellum	Left
15	21	Occipital Cortex	Right

15	22	Occipital Cortex	Left

Slice #, Punch # and Description based on Attachment B and D.

14.9. Splenocyte Sample Collection Analysis

The spleen will be collected using cell culture clean procedures from all animals at scheduled necropsy. Dissected spleens, a sample of approximately 1x1cm, weighed will be placed into prechilled tubes containing RPMI media and stored at 2°C to 8°C or on wet ice before processing to splenocytes. Splenocytes will then be cryopreserved/frozen until analysis.

Samples will be processed at ambient temperature according to standard CR-SEN SOP and analytical procedure AP.5550014.SPL.xx (where xx denote the version of the procedure), except that all washes will be performed with RPMI-1640 Media and samples will be frozen as follows:

Prepare a sufficient number of 2 mL cryovials to hold the cells at 2e7 cells/mL. Open the vials and add 100 µL of Hybridoma-Grade DMSO (Freezing media is 90% heat-inactivated FBS and 10 % DMSO). Add sufficient heat-inactivated FBS to the cell pellet to put the cells at 2e7/mL when added to the DMSO. Gently resuspend the pellet via pipetting and then add to the tubes containing DMSO. The act of addition and higher density of FBS will mix the FBS and DMSO yielding an evenly distributed freezing media. 1-2 gentle inversions can be used to assure even mixing. If adequate splenocytes are available, 3 aliquots of cells at 2e7/mL will be prepared for each animal. Splenocytes will be stored in the vapor phase of liquid nitrogen until shipped to the Sponsor on dry ice. See Attachment C for shipping details.

The splenocytes will be analyzed for T-cell responses against AAV9 and AP4M1 using an ELISpot assay. All analytical work will be conducted by the Sponsor, using an analytical method developed and qualified by that laboratory.

15. MICROSCOPIC EVALUATION

15.1. Histology

Tissues in the Terminal Procedures and Tissue Weighing, Collection, Processing and Evaluation (Attachment A) tables from animals identified in the Terminal Procedures table will be embedded in paraffin, sectioned, mounted on glass slides, and stained with hematoxylin and eosin.

15.2. Microscopic Evaluation

Tissues as detailed in the Terminal Procedures and Tissue Weighing, Collection, Processing and Evaluation (Attachment A) tables will be evaluated histopathologically by a veterinary pathologist with training and experience in laboratory animal pathology.

IBA-1, GFAP (glial fibrillary acidic protein) and Fluoro-Jade B staining will be performed on the lumbar spinal cord and the lumbar DRGs for all animals at scheduled and unscheduled termination.

Immunohistochemistry (IHC) will be performed on the lumbar spinal cord and the lumbar DRGs for all animals at scheduled and unscheduled termination. These slides will be stained for IBA-1, GFAP (glial fibrillary acidic protein) and Fluoro-Jade B and used to identify microglial cells (IBA-1) and astrocytes (GFAP). Immunohistochemistry staining for IBA-1 and GFAP will be detailed in staining procedures HISP.5550014.IBA1.xx and HISP.5550014.GFAP.xx respectively (where xx denotes the version number).

Special stains may be used at the discretion of the pathologist to further characterize lesions and changes identified during routine evaluation of individual animals. Any special stains will be documented in the individual animal data. Any additional stains or evaluations, if deemed necessary by the pathologist, may be added by study plan amendment following discussion with the Study Director and in consultation the Sponsor. Efforts will be made to evaluate all study plan-required tissues microscopically; however, it is not always feasible for every study plan-required tissue to be present on every slide. Study plan-required tissues that are not examined will be documented in the histopathology data and the impact of these missing tissues on the study will be documented in the pathology report.

Images may be generated for illustration of or consultation on histological observations. These images will not be used for data generation or interpretation, and will not be archived or included in the Final Report.

15.3. Pathology Peer Review

A pathology peer review, will be conducted by:

Peer Review Pathologist:	Andre-Jean Lambert, DVM, DES, Vet Pathol, DACVP<br>Charles River Laboratories Montreal ULCSenneville Site (CR-SEN)<br>Address as cited for Test Facility<br>Tel: 514.630.8200, ext 2659<br>E-mail: andre-jean.lambert@crl.com

~~**Histopathology slides will be shipped to the pathologist, see Attachment C for shipping details.**~~

The peer review statement or equivalent documentation will be included as an appendix to the Final Report.

16. STATISTICAL ANALYSIS

Data will be presented as individual values by animal, except for the following.

Means and standard deviations (sexes pooled) will be calculated for NCV data.

16.1. Constructed Variables

Body Weight Changes:	Calculated between each scheduled interval as well as between the following intervals: from beginning to end of each phase.

Organ Weight Relative to Body Weight:	Calculated against the terminal body weight for scheduled intervals.
Organ Weight Relative to Brain Weight:	Calculated against the brain weight for scheduled intervals.

Additional or alternative body weight intervals may be evaluated to elucidate study results at the discretion of the Study Director.

17. COMPUTERIZED SYSTEMS

The following critical computerized systems may be used in the study. The actual computerized systems will be documented in the study data and/or the Final Report.

Critical Computerized Systems

System Name	Description of Data Collected and/or Analyzed
Provantis®	In-life; clinical pathology; postmortem; Test Material receipt, accountability and/or formulation activities
Deviation Information Library	Deviations
Share Document Management System	Reporting
M-Files®	Reporting and collection of 21 CFR Part 11 compliant signature
eInfotree	Excel Module for collection of 21 CFR Part 11 compliance requirements, security, audit trail, and electronic signatures
DocuSign™	Collection of 21 CFR Part 11 compliant signature
SRS (CR-SEN in-house application built with SAS) and/or in-house reporting software Nevis 2012 (using SAS)	Cytokines
Mesa Laboratories AmegaView CMS	Continuous Monitoring System. Monitoring of standalone fridges, freezers, incubators, and selected laboratories to measure temperature, relative humidity, and CO_2, as appropriate
Johnson Controls Metasys	Building Automation System. Control of HVAC and other building systems, as well as temperature/humidity control and trending in selected laboratories and animal rooms
Watson LIMS	Sample management and data regression for cytokines assessment
Bio Plex Manager (Luminex)	Data collection for Cytokines
Softmax Pro GxP	Data collection for Cytokines
StatLia	Data collection for Cytokines
Acq*Knowledge*	Electrophysiological recording for nerve conduction velocity
Excel	Tabulated data entry for nerve conduction velocity
SRS (CR-SEN in-house application built with SAS)	Table formatting for nerve conduction velocity

Data for parameters not required by the Study Plan, which are automatically generated by analytical devices used will be retained on file but not reported. Statistical analysis results that

are generated by the program but are not required by the Study Plan and/or are not scientifically relevant will be retained on file but will not be included in the tabulations.

18. REGULATORY COMPLIANCE

The study will be performed in accordance with the OECD Principles of Good Laboratory Practice and as accepted by Regulatory Authorities throughout the European Union, United States of America (FDA), Japan (MHLW), and other countries that are signatories to the OECD Mutual Acceptance of Data Agreement.

Exceptions to GLPs include the following study elements:

- Characterization of the test and reference items will be/were performed by the Sponsor or Sponsor subcontractor according to established SOPs, controls, and approved test methodologies to ensure integrity and validity of the results generated; these analyses will not be/were not conducted in compliance with the GLP or GMP regulations.
- Stability testing of the supplied test and reference items will be/was performed by the Sponsor or Sponsor subcontractor according to established SOPs, controls, and approved test methodologies to ensure integrity and validity of the results generated; these analyses will not be/were not conducted in compliance with the GLP or GMP regulations.
- Concentration and homogeneity of the test and reference item formulations will not be determined in this study.
- Tissue bioanalysis, gene expression, and splenocyte analysis will not be conducted in compliance with GLP regulations.
- Nerve conduction velocity (NCV) will be performed using scientifically sound but non-validated methodologies.

19. QUALITY ASSURANCE

19.1. Test Facility

The Test Facility Quality Assurance Program (QAP) will monitor the study to assure the facilities, equipment, personnel, methods, practices, records, and controls are in conformance with Good Laboratory Practice regulations. The QAP will review the Study Plan, conduct inspections at intervals adequate to assure the integrity of the study, and audit the Final Report to assure that it accurately describes the methods and standard operating procedures and that the reported results accurately reflect the raw data of the study.

20. AMENDMENTS AND DEVIATIONS

Changes to the approved Study Plan shall be made in the form of an amendment, which will be signed and dated by the Study Director. Every reasonable effort will be made to discuss any necessary study plan changes in advance with the Sponsor. The Study Director will notify the Sponsor of deviations that may result in a significant impact on the study as soon as possible.

21. RETENTION AND DISPOSITION OF RECORDS, SAMPLES, AND SPECIMENS

All study-specific raw data, electronic data, documentation, study plan (and amendments, if any), retained samples and specimens, and interim (if applicable) and final reports will be archived by no later than the date of final report issue. All materials generated by Charles River from this study will be transferred to a Charles River archive. At least 1 year after issue of the Draft Report, the Sponsor will be contacted.

Disposition of residual/retained analytical samples will be as described in the table below.

Disposition of Residual/Retained Samples

Sample Type	Disposition	Schedule
**Cytokine**	**Discard**	**Samples will be maintained for a maximum of 6 months following issuance of the first full Draft Report after which samples will be managed as defined in the 'Disposition' column as requested and authorized by the Study Director and in consultation with the Sponsor.**
**Splenocytes**		
**Additional Serum**	Archived by the Sponsor	Samples to be archived by the Sponsor
Bioanalytical (**blood, CSF, and** tissues)		
Frozen tissues		

Records to be maintained will include, but will not be limited to, documentation and data for the following:

- Deviations, study plan, and study plan amendments
- Study schedule
- Study-related correspondence
- Test system receipt, health, and husbandry
- Test and reference item receipt, identification, preparation, and analysis
- In-life measurements and observations
- Clinical pathology sample collection and evaluation
- Cytokine sample collection and evaluation
- Gross and microscopic observations and related data
- Organ weight measurements
- Statistical analysis results

- **STUDY CLASSIFICATION**

Study Category:	Toxicology
Study Type:	Single Dose Toxicity
Study Design:	Parallel
Primary Treatment CAS Registry Number:	Not Available

Primary Treatment Unique Ingredient ID:	Not Available
Class of Compound:	AAV9 Vector
Administration Dose Form:	Solution

22. REPORTING

A comprehensive Draft Report will be prepared following completion of the study and will be finalized following consultation with the Sponsor. The report will include a summary of the experimental methods and all information necessary to provide a complete and accurate description of the results and any circumstances that may have affected the quality or integrity of the study.

The Sponsor will receive an electronic version of the Draft and Final Report provided in Adobe Acrobat PDF format (hyperlinked and searchable) along with a Microsoft Word version of the text. The PDF document will be created from native electronic files to the extent possible, including text and tables generated by the Test Facility. Report components not available in native electronic files and/or original signature pages will be scanned and converted to PDF image files for incorporation.

Reports should be finalized within 6 months of issue of the Draft Report. If the Sponsor has not provided comments to the report within 6 months of draft issue, the report will be finalized by the Test Facility unless other arrangements are made by the Sponsor.

23. JUSTIFICATIONS AND GUIDELINES

23.1. Justification of Test System and Number of Animals

At this time, studies in laboratory animals provide the best available basis for extrapolation to humans and are required to support regulatory submissions. Acceptable models that do not use live animals currently do not exist.

The cynomolgus monkey was chosen as the animal model for this study as it is an accepted nonrodent species for nonclinical toxicity testing by regulatory agencies.

The total number of animals to be used in this study is considered to be the minimum required to properly characterize the effects of the test item. This study has been designed such that it does not require an unnecessary number of animals to accomplish its objectives.

23.2. Justification of Route and Dose Levels

The intrathecal route of exposure was selected because this is the intended route of human exposure.

The dose levels were selected based on information provided by the Sponsors' prior studies with AAV9/AP4M1 in mice and rats (CRL-SEN Study No. 5550008), as well as prior pharmacology for other similar AAV9 vectors in mice, rats, pigs, and nonhuman primates. Based on those prior

pharmacology studies, the chosen low dose is anticipated to provide efficacy in ongoing mouse studies which should translate to humans. No clear dose-limiting toxicities were observed at doses equivalent to the low or high dose in mice. At one year postdose, 3 out of 10 mice receiving the high dose had hepatocellular adenoma, which could have been caused by the test item. The low dose in this NHP study corresponds to a nearly maximum feasible dose (MFD) due to limitations on injection volume and test item concentration, and this is the target dose for human translation. A 2x higher dose is achieved using a higher injection volume, and is included to evaluate a possible safety margin. None of the doses are expected to generate more than a minimal to moderate toxic effect. If any adverse effects are observed, the range of doses in the study design is an attempt to produce a graded response.

23.3. Guidelines for Study

The design of this study was based on the study objective(s) and the overall product development strategy for the test item, and on the following study design guidelines.

- ICH Harmonised Tripartite Guideline S6 (R1). *Preclinical Safety Evaluation of Biotechnology-Derived Pharmaceuticals.*
- Japanese Guidelines for Nonclinical Studies of Drugs Manual (1995). *Guidelines for Toxicity Studies of Drugs (Chapter 2, Single Dose Toxicity Study).*

24. ANIMAL WELFARE

The Study Plan and any amendment(s) or procedures involving the care and use of animals in this study will be reviewed and approved by CR-SEN Institutional Animal Care and Use Committee (IACUC). During the study, the care and use of animals will be conducted with guidance from the USA National Research Council and the Canadian Council on Animal Care (CCAC).

25. REFERENCES

Office of Laboratory Animal Welfare. *Public Health Services Policy on Humane Care and Use of Laboratory Animals*. Bethesda, MD: National Institutes of Health. Current edition.

Appendix 1

TEST FACILITY APPROVAL

All electronic signatures appear at the end of the document upon finalization.

SPONSOR APPROVAL

The Study Plan Amendment was approved by the Sponsor by e-mail on the date designated below. The correspondence giving approval will be archived, as appropriate with other Sponsor communications.

21 Dec 2021

Date of Sponsor Approval

ATTACHMENT A

Tissue Weighing, Collection, Processing, and Evaluation Table

Organ	Weigh	Macroscopic Evaluation and Collection	Histology Processing	Microscopic Evaluation	Bioanalysis
Animal ID	-	X	-	-	-
Artery, aorta	-	X	X	-	-
Bone marrow, sternum	-	X	X	-	-
Bone marrow smear	-	X[a]	-	-	-
Bone, femur, right	-	X	X	-	-
Bone, sternum	-	X	X	-	-
Brain	X	X	X	X	X (cerebellum, hippocampus, cortex (frontal, parietal, occipital, and temporal lobes), striatum, thalamus, midbrain, pons, medulla)
Epididymis	X (2)	X (2)	X (2)	-	-
Esophagus	-	X	X	-	-
Eye	-	X (2)	X (2)	-	-
Gallbladder	- [b]	X	X	-	-
Ganglion, dorsal root (DRG), (cervical, lumbar, and thoracic with dorsal and ventral nerve roots)[f]	-	X (2)	X (2)	X (2)	Refer to ATTACHMENT E
Ganglion, trigeminal	-	X	X (left)	X (left)	X (right)
Gland, adrenal	X (2)	X (2)	X (2)	-	-
Gland, lacrimal	-	X (2)	-	-	-
Gland, mammary	-	X	X	-	-
Gland, parathyroid	-[c]	X (2)	X (2)	-	-
Gland, pituitary	X	X	X	-	-
Gland, prostate	X	X	X	-	-
Gland, salivary, submandibular	-	X (2)	X (1)	-	-
Gland, salivary, sublingual	-	X (2)	-	-	-
Gland salivary, parotid	-	X (2)	-	-	-
Gland, seminal vesicle	-	X (2)	X (2)	-	-
Gland, thyroid	X (2)	X (2)	X (2)	-	-
Gut-associated lymphoid tissue[d]	-	X	X	-	-
Heart	X	X	X	X	X
Joint, femorotibial, right	-	X	X	-	-
Kidney	X (2)	X (2)	X (2)	X (2)	X (right)
Large intestine, cecum	-	X	X	-	-
Large intestine, colon	-	X	X	-	-
Large intestine, rectum	-	X	X	-	-

Appendix 1

Organ	Weigh	Macroscopic Evaluation and Collection	Histology Processing	Microscopic Evaluation	Bioanalysis
Liver	X	X	X	X	X (left lateral)
Lung	-	X	X	-	X
Lymph node(s) draining administration site(s): deep cervical and iliac	-	X (2)	X (2)	X (2)	-
Lymph node, mandibular	-	X (2)	X (1)	-	-
Lymph node, mesenteric	-	X	X	-	-
Muscle, skeletal, gastrocnemius	-	X (2)	X (2)	X (2)	X (1, right)
Muscle, skeletal, biceps femoris	-	X (2)	X (2)	X (2)	X (1, right)
Nerve, optic	-	X (2)	X (2)	X (2)	X (1, right)
Nerve, sciatic	-	X (2)	X (2)	X (2)	X (1, right)
Nerve, sural	-	X (2)	X (2)	X (2)	-
Nerve, tibial	-	X (2)	X (2)	X (2)	X (1, right)
Ovary	X (2)	X (2)	X (2)	-	X (1, right)
Oviduct	-	X (2)	-	-	-
Pancreas	-	X	X	-	-
Skin	-	X	X	-	-
Small intestine, duodenum	-	X	X	-	-
Small intestine, ileum	-	X	X	-	-
Small intestine, jejunum	-	X	X	-	-
Spinal cord (cervical, lumbar, and thoracic, including injection site)[e]	-	X	X	X	X<br>Refer to ATTACHMENT E
Spleen	X	X	X	X	X
Stomach	-	X	X	-	-
Testis	X (2)	X (2)	X (2)	-	X (1, right)
Thymus	X	X	X	-	X
Tongue	-	X	X	-	-
Trachea	-	X	X	-	-
Ureter	-	X (2)	-	-	-
Urinary bladder	-	X	X	-	-
Uterus/Cervix	X	X	X	-	-
Vagina	-	X	X	-	-

Appendix 1

Organ	Weigh	Macroscopic Evaluation and Collection	Histology Processing	Microscopic Evaluation	Bioanalysis

X = Procedure to be conducted. - = Not applicable. (1) = one side. (2) = both sides.

~~**Macroscopic abnormalities in the organs listed and in other organs will be sampled at necropsy, processed for histology and examined microscopically.**~~

[a] Bone marrow smears will be collected from the 5th to 7th rib at scheduled and unscheduled necropsies (for possible examination). Smears will not be collected from animals that are found dead or from animals that were euthanized moribund and then stored in the refrigerator prior to necropsy. Bone marrow smears are allowed to air dry and are not fixed in formalin.

[b] Weigh with liver.

[c] Weigh with gland, thyroid.

[d] From small intestine: Peyer's patch or solitary lymphoid follicle.

[e] Transverse and/or oblique sections.

[f] Nerve roots will be examined if present in section of dorsal root ganglia.

Appendix 1

ATTACHMENT B

Brief Collection Procedure

1. The brain matrix to be used will be chilled in a saline ice bath. Following collection of the brain, it will be placed in the matrix in the ice bath for approximately 10 minutes to firm up the tissue prior to processing
2. The brain slicing will be performed as per diagram below:

Slice number

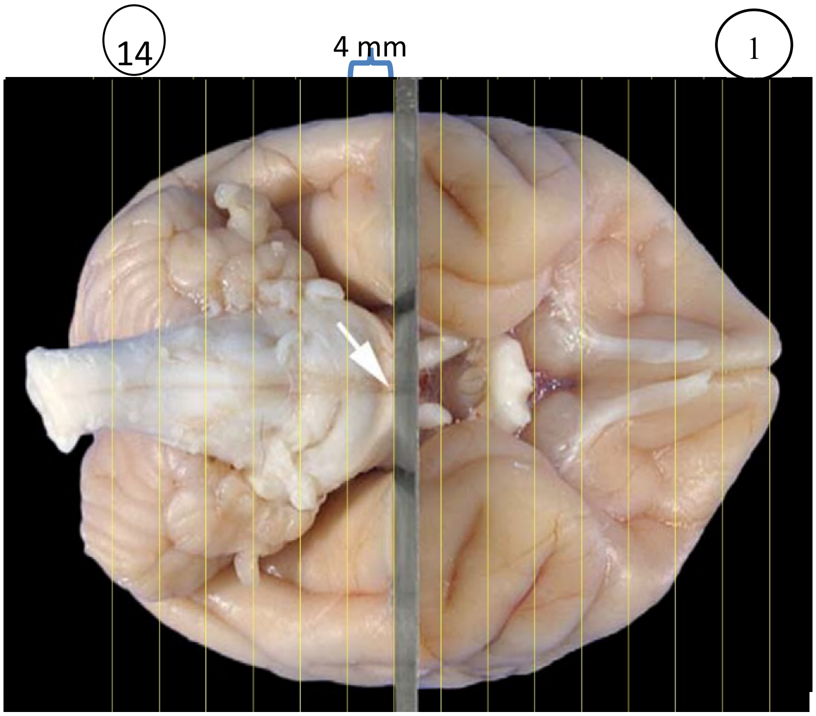

NOTE: The initial razor cut should be consistent for all specimens (i.e. start from rostral to pons), with all subsequent slices taken 4mm apart. The starting location for slicing will be recorded with in the study data.

3. Following complete sectioning of the brain within the matrix, the brain will be carefully removed and each individual brain slab will be laid out flat in consecutive order, with the caudal face of each slab oriented against the table/bench (rostral face of each slice will be face up).
4. Each brain slab will then be cut sagitally, to separate the hemispheres Right and Left.
5. Each hemisphere from alternative slabs will then be punched as per Sections 14.7 and 14.8 and Attachment D, and the remaining tissue from both hemispheres will be frozen flat indirectly on dry ice, then placed flat into appropriately sized cassettes labeled with group number, animal number and brain section number and stored at -80°C. Alternate slabs will be subdivided and

placed into appropriately sized cassettes labeled with group number, animal number and brain section number and placed in 10% NBF.

Appendix 1

ATTACHMENT C

Shipment of Samples and Study Records

Matrix	Purpose	Day/ Week/ Aliquot	Proposed Shipment Date	Conditions for Shipment	Recipient/Address
Bulk test item<br>Residual Dose Formulations	Return to Sponsor	Not applicable	Following completion of dosing	Dry ice	Attention to:<br>Steven Gray, PhD<br>University of Texas Southwestern Medical Center<br>NA2.508<br>5323 Harry Hines Blvd<br>University of Texas Southwestern Medical Center<br>Dallas, TX 75390<br>Tel: 214-648-0670<br>E-mail: steven.gray@UTSouthwestern.edu
Additional Serum	Archival	Not applicable	Following the last collection	Dry ice	Attention to:<br>Juan A. Rodriguez<br>University of Texas Southwestern 5901 Forest Park Rd,<br>NA2.508 Receiving – North Campus Dock<br>Dallas, TX 75390<br>Tel: 281-380-5165 E-mail: Juan.Rodriguez@UTSouthwestern
Tissues	Bioanalysis	Not applicable	Within 2 weeks from collection	Dry ice	Attention to:<br>Juan A. Rodriguez<br>University of Texas Southwestern 5901 Forest Park Rd,<br>NA2.508 Receiving – North Campus Dock<br>Dallas, TX 75390<br>Tel: 281-380-5165 E-mail: Juan.Rodriguez@UTSouthwestern
Splenocytes	Tissue gene expression Analysis	Not applicable	Following the last collection	Dry ice	Attention to:<br>Steven Gray, PhD<br>University of Texas Southwestern Medical Center<br>NA2.508<br>5323 Harry Hines Blvd<br>University of Texas Southwestern Medical Center<br>Dallas, TX 75390<br>Tel: 214-648-0670<br>E-mail: steven.gray@UTSouthwestern.edu

Alternate shipping details may be provided thereafter and will be documented in study correspondence.

Appendix 1

ATTACHMENT D

Brain Collection Procedure

BRAIN SAMPLING FOR HISTOLOGY AND BIOANALYSIS

From the left hemisphere of the brain, 12 sections (white rectangles) will be selected for histopathology. From both hemispheres, samples for bioanalysis (dashed rectangles) will be collected as described in Section 14.8. The following images are to be used as a guideline only, and slight variations may occur.

Abbreviation list: P.C.: Parietal Cortex; T.C.: Temporal Cortex; Thal.: Thalamus; Hipp.: Hippocampus.

Appendix 1

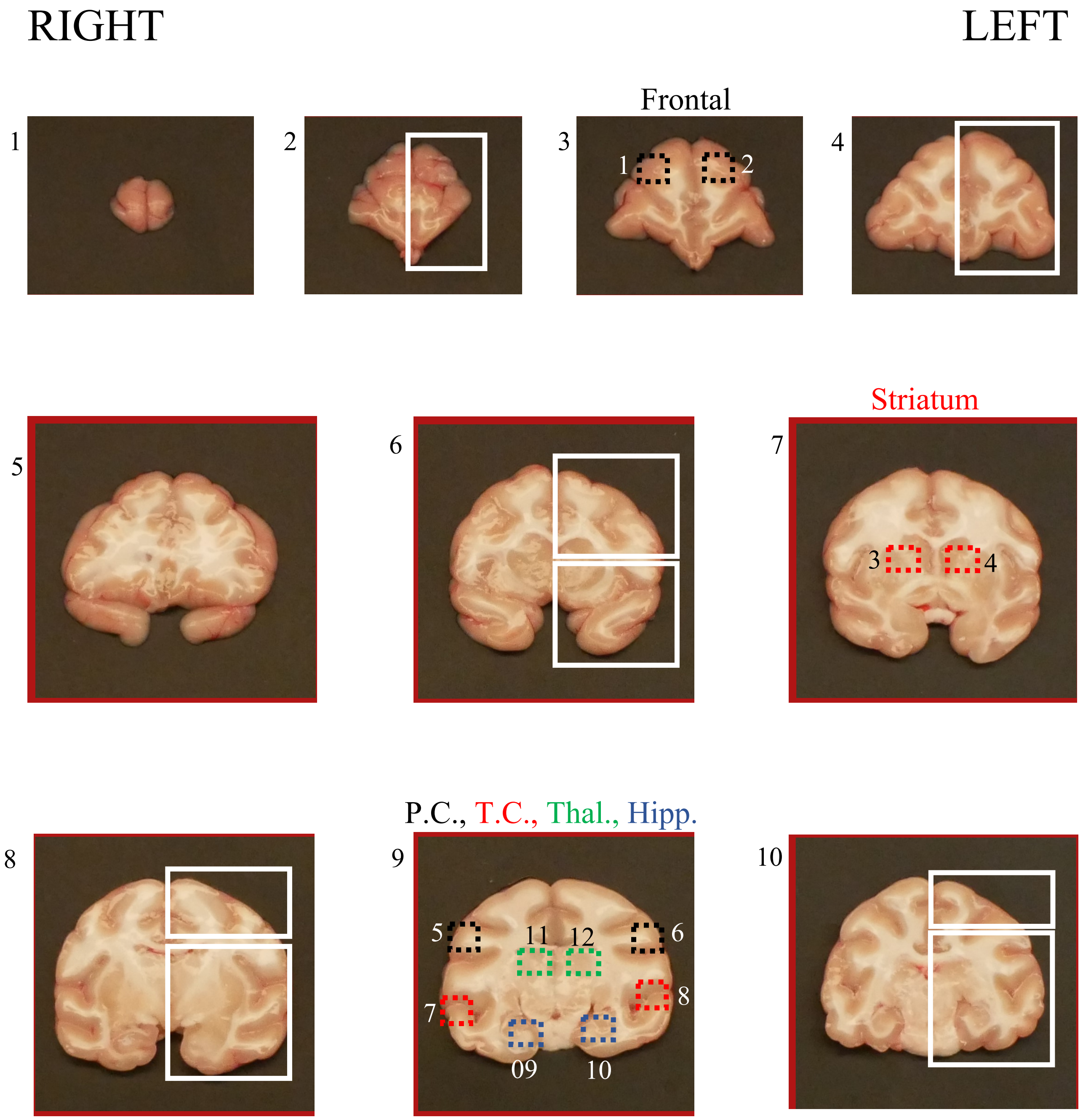
RIGHT
LEFT
Frontal
1
2
3
4
1
2
5
6
Striatum
7
3
4
P.C., T.C., Thal., Hipp.
8
9
10
5
11
12
6
7
8
09
10

Appendix 1

Pons, Midbrain

Medulla

11
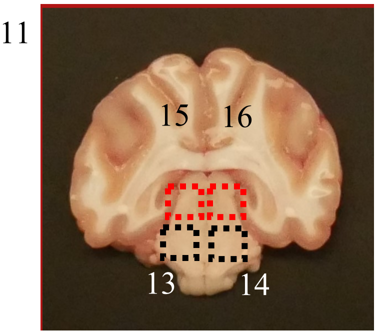

12
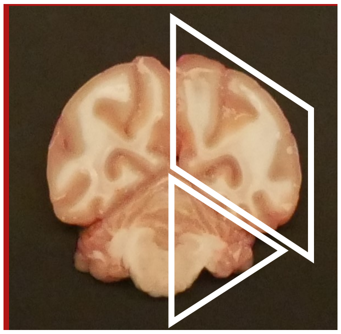

13
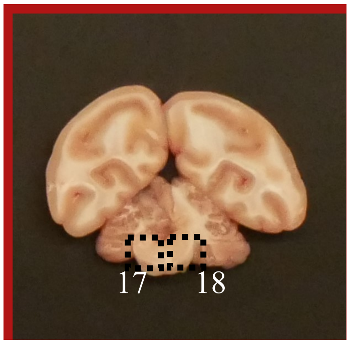

Occipital, Cerebellum

14
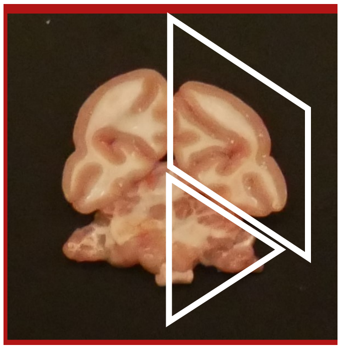

15
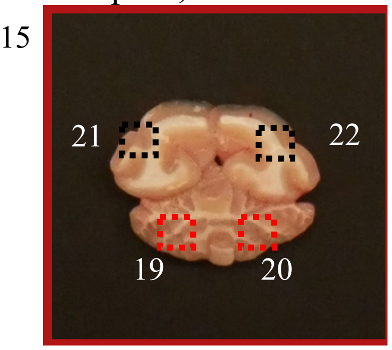

16
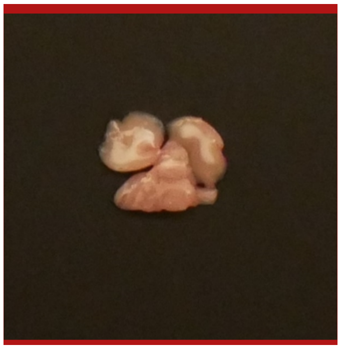

ATTACHMENT E

SPINAL CORD COLLECTION PROCEDURE

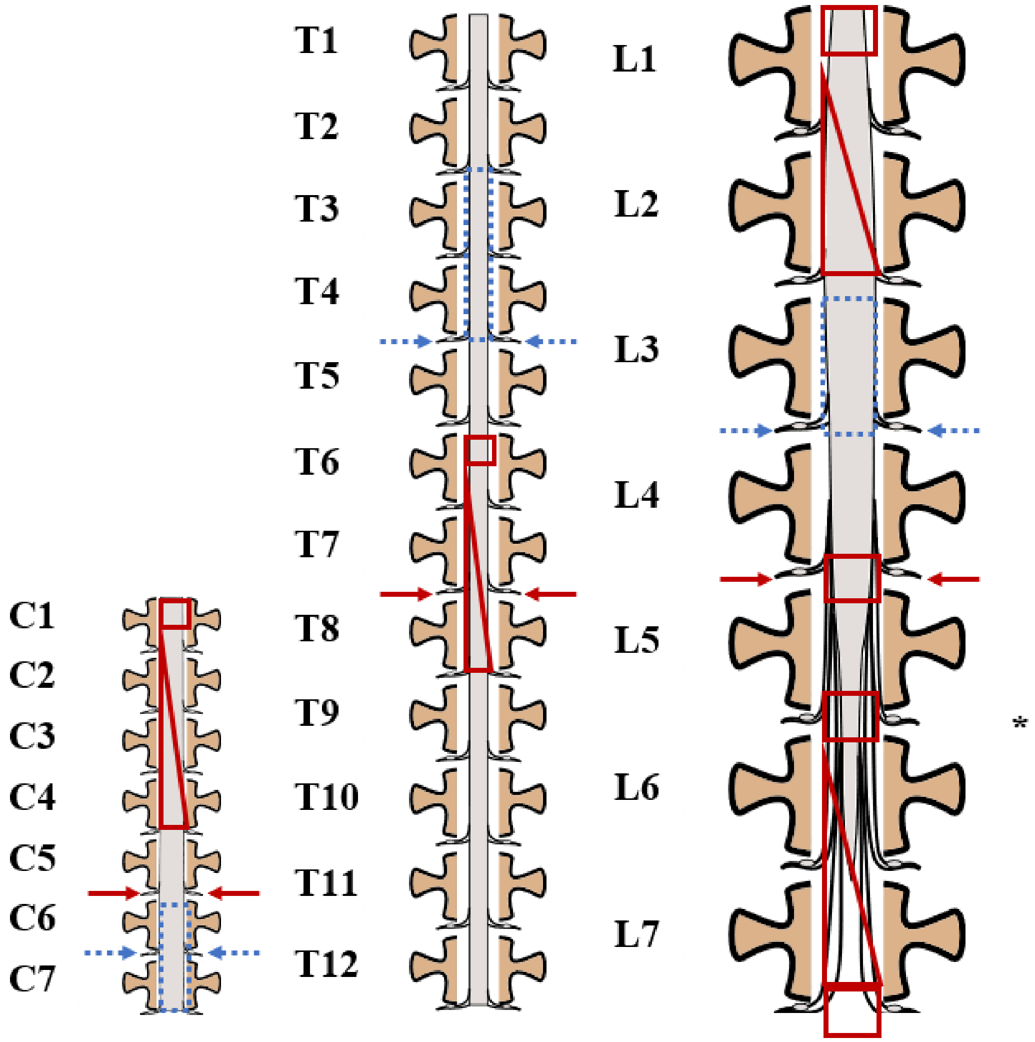

By convention, the last lumbar vertebra is considered to be L7. Red rectangles and arrows represent histology sections for the spinal cord and DRGs respectively, while dashed blue rectangles and arrows represent bioanalysis samples. Levels for the DRGs are given as a guideline and may slightly vary. * = Injection site.

SIGNATURE(S) FOR DOCUMENT: 5550014 - Study Plan Amendment 6

**Study Director Approval:**	I approve this document.		
Name:	**Cinquino, Stefania**		
	Cinquino, Stefania	22-Dec-2021 12:38:29 (UTC+00:00)	
Electronically Signed in	M-Files	Timestamp	

Appendix 1

FINAL STUDY PLAN

Test Facility Study No. 5550014

Sponsor Reference No. UTSW-Gray-SPG50-004

A Single-Dose Study of AAV9/AP4M1 by Intrathecal Injection in Immunosuppressed Monkeys

Non-GLP

SPONSOR:
University of Texas Southwestern Medical Center
5323 Harry Hines Blvd
Dallas, TX 75390-9056
USA

TEST FACILITY:
Charles River Laboratories Montreal ULC
Senneville Site (CR-SEN)
22022 Transcanadienne
Senneville, QC H9X 3R3
Canada

TABLE OF CONTENTS

1. OBJECTIVE....3
2. PROPOSED STUDY SCHEDULE....3
3. SPONSOR....3
4. RESPONSIBLE PERSONNEL....4
5. TEST MATERIALS....5
6. DOSE FORMULATION AND ANALYSIS....6
7. IMMUNOSUPPRESSION FORMULATION....7
8. TEST SYSTEM....8
9. HUSBANDRY....9
10. EXPERIMENTAL DESIGN....11
11. IN-LIFE PROCEDURES, OBSERVATIONS, AND MEASUREMENTS....13
12. CLINICAL PATHOLOGY....14
13. CYTOKINES COLLECTION AND ANALYSIS....16
14. TERMINAL PROCEDURES....17
15. MICROSCOPIC EVALUATION....20
16. STATISTICAL ANALYSIS....21
17. COMPUTERIZED SYSTEMS....21
18. REGULATORY COMPLIANCE....22
19. AMENDMENTS AND DEVIATIONS....22
20. RETENTION AND DISPOSITION OF RECORDS, SAMPLES, AND SPECIMENS....23
• STUDY CLASSIFICATION....23
21. REPORTING....24
22. JUSTIFICATIONS AND GUIDELINES....24
23. ANIMAL WELFARE....25
24. REFERENCES....25
TEST FACILITY APPROVAL....26
SPONSOR APPROVAL....27
ATTACHMENT A....28
ATTACHMENT B....31

1. OBJECTIVE

The objectives of this study are to determine the potential toxicity, biodistribution, and gene expression of AAV9/AP4M1, for the treatment of Spastic Paraplegia Type 50 (SPG50) caused by the AP4M1 gene mutation, when given by a single intrathecal injection to monkeys, to evaluate the potential reversibility and/or progression of any findings, and to determine the potential impact of immunosuppressant (IMS) on the toxicity profile of this AAV9 vector.

2. PROPOSED STUDY SCHEDULE

Proposed study dates are listed below. Actual dates will be included in the Final Report.

Experimental Starting Date:	02 Jul 2021<br>(First date of study-specific data collection)
Experimental Completion Date:	09 May 2022<br>(Last date on which data are collected)
Animal Transfer:	28 Jun 2021
Initiation of Dosing:	13 Jul 2021
Completion of In-life:	13 Sep 2021<br>(Last date of necropsy)
Abbreviated Draft Report:	09 Nov 2021<br>(8 weeks following completion of in-life)
Final Report:	09 May 2022<br>(Expected date of Study Director signature of report. Target to be within 6 months of issue of Draft Report)

3. SPONSOR

Role/Phase	Name	Contact Information
Sponsor Representative	Steven Gray, PhD	University of Texas Southwestern Medical Center<br>5323 Harry Hines Blvd<br>University of Texas Southwestern Medical Center<br>Dallas, TX 75390<br>Tel: 214.648.0670<br>E-mail: steven.gray@UTsouthwestern.edu
Alternate Sponsor Representative	Roxana Ploski	University of Texas Southwestern Medical Center<br>5323 Harry Hines Blvd<br>University of Texas Southwestern Medical Center<br>Dallas, TX 75390<br>Tel: 214.648.9828<br>E-mail: roxana.ploski@UTsouthwestern.edu

Appendix 1

Role/Phase	Name	Contact Information
Alternate Study Contact	Terry Pirovolakis	CureSPG50<br>6 Topham Road<br>Toronto, ON M4B 3K2<br>Canada<br>Tel:416.625.1933<br>E-mail: tpirovol@hotmail.com

4. RESPONSIBLE PERSONNEL

<table>
<tr><th colspan="2">Role/Phase</th><th>Name</th><th>Contact Information</th></tr>
<tr><td colspan="2">Study Director</td><td>Stefania Cinquino, BSc</td><td>Address as cited for Test Facility
Tel: 514.630.8200, ext 2151
E-mail: stefania.cinquino@crl.com</td></tr>
<tr><td colspan="2">Test Facility Management</td><td>Julie Douville, PhD</td><td>Address as cited for Test Facility
Tel: 514.630.8200, ext 8309
E-mail: julie.douville@crl.com</td></tr>
<tr><th colspan="4">Individual Scientist (IS)</th></tr>
<tr><td colspan="2">Nerve Conduction Velocity</td><td>Nataliya Sadekova, MSc, DSP</td><td>Address as cited for Test Facility
Tel: 514.630.8200, ext 2334
E-mail: nataliya.sadekova@crl.com</td></tr>
<tr><td colspan="2">Cytokines</td><td>Name, Credentials</td><td>Address as cited for Test Facility
Tel:
E-mail:</td></tr>
<tr><td colspan="2">Pathology</td><td>Ryan Schafbuch, BSc, DVM, MS, DACVP</td><td>Address as cited for Test Facility
Tel: 514.630.8200, ext 8322
E-mail: ryan.schafbuch@crl.com</td></tr>
<tr><td colspan="2">Immuno-toxicology Splenocyte Preparation</td><td>Name, Credentials</td><td>Address as cited for Test Facility
Tel:
E-mail:</td></tr>
<tr><th colspan="4">Principal Investigator (PI)</th></tr>
<tr><td>Tissue Biodistribution Analysis (Bioanalysis) [a]</td><td>Test Site</td><td>Steven Gray, PhD</td><td>University of Texas Southwestern Medical Center
5323 Harry Hines Blvd
University of Texas Southwestern Medical Center
Dallas, TX 75390
Tel: 214-648-0670
E-mail:
steven.gray@UTsouthwestern.edu</td></tr>
</table>

[a] Sponsor.

Each IS and PI is required to report any deviations or other circumstances that could affect the quality or integrity of the study to the Study Director in a timely manner for authorization/acknowledgement. Each IS and PI, with the exception of those listed below, will provide a report addressing their assigned phase of the study, which will be included as an appendix to the Final Report.

The IS phase report will include the following:

- A listing of critical computerized systems used in the conduct and/or interpretation of the assigned study phase

The IS for the Immunotoxicology Splenocyte Preparation phase will not provide a formal report.

The PI phase report will include the following:

- The archive site for all records, samples, specimens and reports generated from the phase or segment (alternatively, details regarding the retention of the materials may be provided to the Study Director for inclusion in the Final Report)
- A listing of critical computerized systems used in the conduct and/or interpretation of the assigned study phase

5. TEST MATERIALS

5.1. Test and Reference Item Characterization

A Certificate of Analysis or equivalent documentation will be provided for inclusion in the Final Report (if available).

5.2. Test Item Identification

Test Item Identification

	**Test Item**
**Identification:**	AAV9/AP4M1
**Alternate Identification:**	rAAV9.AP4M1
**Batch/Lot No.:**	T-GEMINIS-033
**Expiration/Retest Date:**	22 Dec 2021 (1-year stability time point)<br>Stability concomitant to the study
**Physical Description:**	Colorless, clear to slightly opalescent, free of visible particles
**Concentration:**<br>**(based on ddPCR results)**	5.43E13 vg/mL
**Storage Conditions (temperature set to maintain):**	-80ºC<br>(upon thawing of a vial, it may be stored at 4ºC and used on a subsequent dosing day)
**Provided by:**	Sponsor

5.3. Reference Item/Vehicle Identification

Reference Item/Vehicle Identification

	**Reference Item**
**Identification:**	PBS containing 5% D-sorbitol and 0.001% pluronic F-68
**Storage Conditions (temperature set to maintain):**	-80°C
**Provided by:**	Sponsor

5.4. Test and Reference Item Inventory and Disposition

Records of the receipt, distribution, storage, and disposition of test materials will be maintained. All unused Sponsor-supplied bulk test materials, with the exception of reserve samples, will be returned to the Sponsor following issuance of the Draft Report unless otherwise requested (documentation will be retained in the study record). An earlier shipment of these materials may also be requested and authorized by the Study Director and Sponsor. See Shipment of Samples and Study Records (Attachment C) for shipping details.

5.5. Safety

The safety precautions for the test item and dose formulations will be documented in a Test Material Safety Data Sheet (TMSDS) based on the SDS or similar document.

6. DOSE FORMULATION AND ANALYSIS

6.1. Preparation of Formulations

Dose formulations will be divided into aliquots, where required, and dispensed on each dosing occasion.

Preparation Details

Dose Formulation	Frequency of Preparation	Storage Conditions (temperature set to maintain)
Reference Item	Used as received	4°C
Test Item	At least once	4°C

Any residual volumes from each dosing occasion will be will be shipped back to the Sponsor; see Attachment C. Residual volumes will be archived by the Sponsor and will be kept for potential future analysis. Analysis, if conducted, will be added by amendment.

6.2. Preparation Details

Dosing formulations will be prepared under a laminar flow hood using clean procedures.

Dosing formulations will be prepared based on Sponsor's instructions at appropriate concentrations to meet dose level requirements.

7. IMMUNOSUPPRESSION FORMULATION

7.1. Immunosuppression Material

Immunosuppression (IMS) Identification

	IMS 1	IMS 2	IMS 3
**Identification:**	Methylprednisolone succinate	Methylprednisolone acetate	Rapamycin
**Alternate Identification:**	Solu-Medrol	Depo-Medrol	-
**Physical Description:**	Liquid	Liquid	Powder
**Concentration:**	125 mg/mL	20 mg/mL	N/A
**Storage Conditions (temperature set to maintain):**	21°C Protected from light	21°C Protected from light	-20°C Protected from light
**Provided by:**	Test Facility	Test Facility	Test Facility
**Supplier:**	Pfizer	Pfizer	Toronto Research Chemicals

N/A = Not applicable

7.2. Immunosuppression Vehicle

Immunosuppressants Vehicle Identification

	Vehicle for Solu-Medrol	Vehicle Components for Rapamycin		
**Identification:**	0.9% sodium chloride injection, USP	N,N-Dimethylacetamide	Tween-80	Polyethylene Glycol
**Storage Conditions (temperature set to maintain):**	21°C	21°C	21°C Protected from light	21°C
**Provided by:**	Test Facility	Test Facility	Test Facility	Test Facility

7.3. Immunosuppressant Preparation Details

IMS dose formulations will be divided into aliquots, where required, and dispensed on each dosing occasion.

Preparation Details

Dose Formulation	Frequency of Preparation	Storage Conditions (temperature set to maintain)
Methylprednisolone succinate	At least weekly	21°C<br>protected from light
Methylprednisolone acetate	At least weekly	21°C<br>protected from light
Rapamycin	At least weekly	4°C<br>Protected from light (in amber glass vials)

IMS dosing formulations will be prepared based on Sponsor's instructions at appropriate concentrations to meet dose level requirements.

7.4. Sample Collection and Analysis

Samples for test or reference item dose formulation and/or IMS dose formulation analysis will not be collected by the Test Facility.

8. TEST SYSTEM

Species:	Monkey
Strain:	Cynomolgus
Condition:	Purpose-bred, naïve
Source:	CR-SEN Colony, original source will be documented in the Final Report
Continent of Origin:	Vietnam
Number of Males to be Assigned:	2
Number of Females to be Assigned:	4 (plus 1 alternate)
Target Age at the Initiation of Dosing:	2 to 4 years of age
Target Weight at the Initiation of Dosing:	1.5 to 6 kg

The actual age and weight of the animals at the initiation of dosing will be listed in the Final Report.

8.1. Animal Screening

Method:	All animals used on study will have documentation to confirm one negative tuberculosis (TB) test. Additional TB testing may be performed as required.

8.2. Animal Identification

Method:	Subcutaneously implanted electronic identification chip.

8.3. Environmental Acclimation

Method:	At least 2 weeks will be allowed between animal transfer and the start of dosing in order to accustom the animals to the laboratory environment.

8.4. Selection, Assignment, Replacement, and Disposition of Animals

Selection:	Pre-established pairs will be assigned to groups using a computerized-based randomization procedure. Males and females will be randomized separately.
Replacement:	Before the initiation of dosing, any assigned animals considered unsuitable for use in the study will be replaced by alternate animals. After initiation of dosing, study animals may be replaced during the replacement period with alternate animals in the event of accidental injury, non-test item-related health issues, or similar circumstances. The alternate animals may be used as replacements on the study within 2 days. General in-life assessments will include alternate animals until released from study.
Disposition:	The disposition of all animals will be documented in the study records.

9. HUSBANDRY

9.1. Housing

Housing:	Group-housed (up to 3 animals of the same sex and same dosing group together).
Caging:	Stainless steel cages with stainless steel mesh floor
Cage Identification:	Color-coded cage card indicating study, group, animal/tattoo number(s), and sex.

Housing set-up is as described in the *Guide for the Care and Use of Laboratory Animals* (National Research Council, 2011) Animals will be separated during designated procedures/activities or will be separated as required for monitoring and/or health purposes, as deemed appropriate by Study Director and/or Clinical Veterinarian.

9.2. Animal Enrichment

Psychological/ Environmental Enrichment:	Animals will be socially housed and will be provided with items such as perches, floor enrichment devices, foraging devices and/or suspended devices, except during study procedures/activities. Additional enrichment, such as music, natural sounds or color videos films will also be provided. Each animal will be offered food supplements (such as certified treats, fruit/vegetables and/or Foraging Crumbles™).

9.3. Environmental Conditions

The targeted conditions for animal room environment will be as follows:

Temperature:	23 ±3°C
Humidity:	30% to 70%
Light Cycle:	12 hours light and 12 hours dark (except during designated procedures)

9.4. Food

Diet:	Lab Diet Certified Primate Diet 5048
Type:	Pellets
Frequency/Ration:	Twice daily, except during designated procedures. The chow will be provided in amounts appropriate for the size and age of the animals.
Analysis:	Results of analysis for nutritional components and environmental contaminants are provided by the supplier and are kept on file at the Test Facility. It is considered that there are no known contaminants in the feed that would interfere with the objectives of the study.

9.5. Water

Type:	Municipal tap water, treated by reverse osmosis and ultraviolet irradiation.
Frequency/Ration:	Freely available to each animal via an automatic watering system (except during designated procedures).
Analysis:	Periodic analysis of the water is performed, and results of these analyses are kept on file at the Test Facility. It is considered that there are no known contaminants in the water that would interfere with the outcome of the study.

9.6. Veterinary Care

Veterinary care will be available throughout the course of the study and animals will be examined by the veterinary staff as warranted by clinical signs or other changes. In the event that

animals show signs of illness or distress, the responsible veterinarian may make initial recommendations about treatment of the animal(s) and/or alteration of study procedures, which must be approved by the Study Director or Scientific designate. Treatment of the animal(s) for minor injuries or ailments may be approved without prior consultation with the Sponsor representative when such treatment does not impact fulfillment of the study objectives. If the condition of the animal(s) warrants significant therapeutic intervention or alterations in study procedures, the Sponsor representative will be contacted, when possible, to discuss appropriate action. If the condition of the animal(s) is such that emergency measures must be taken, the Study Director (or Scientific designate) and/or veterinarian will attempt to consult with the Sponsor representative prior to responding to the medical crisis, but the Study Director (or Scientific designate) and/or veterinarian has authority to act immediately at his/her discretion to alleviate suffering. The Sponsor representative will be fully informed of any such events.

10. EXPERIMENTAL DESIGN

Experimental Design

Group No.	Test Material	Dose Level (vg)	Dose Volume (mL)	Dose Concentration (vg/mL)	No. of Animals Main Study Males	No. of Animals Main Study Females
1	Reference Item	0	1	0	1	1
2	AAV9/AP4M1	8.4×10^{13}	1.55	5.43×10^{13}	-	2
3	AAV9/AP4M1	1.68×10^{14}	3.10	5.43×10^{13}	1	1

10.1. Administration of Test and Reference Item

Dose Route: Percutaneous intrathecal injection (slow bolus, target rate of 1 mL/min) at the lumbar level (target L5-L6 space, L4-L5 may also be used if necessary)

Frequency: Once

Duration: 1 day

Method: The first day of dosing will be designated as Day 1.

Animals will be food deprived at least 6 hours prior to dosing. Animals will be anesthetized with a ketamine/dexmedetomidine/glycopyrrolate cocktail. Tracheal intubation will be performed, oxygen will be provided and anesthesia will then be maintained using isoflurane, if required.

Dose formulations will be allowed to warm up to room temperature for at least 30 minutes prior to dosing.

Percutaneous intrathecal injection will be performed using atraumatic spinal needle (e.g. B Braun, 25 Gauge, 1 inch); actual needle will be recorded in the raw data. Animals will remain in a Trendelenberg

position for at least 15 minutes after the dosing is completed prior to the administration of the reversal agent (atipamezole), when required.

10.2. Administration of Anti-Inflammatory

On days of dose administration and/or CSF collections, animals will receive an anti-inflammatory (Meloxicam) subcutaneously prior to dosing/CSF collection and then orally daily, starting the morning after dosing/CSF collection, for a total of 2 doses.

10.3. Administration of Immunosuppressant Material

Immunosuppressant (IMS) Information

Group No.	Test Material	Study Day	Method	Dose Level (mg/kg/dose)	Dose Volume (mL/kg)	Dose Concentration (mg/mL)	Frequency
1 to 3	Methylprednisolone succinate	1	IV infusion for 30 min	10	0.5	20	Once on Day 1 only
	Methylprednisolone acetate	1 to 62	IM	1	0.05	20	SID; first dose morning of surgery
	Rapamycin	-14 to 62	IM	0.01	0.067	0.15	BID, 12 hr ± 3 hrs apart

min = minute; IV = intravenous; IM = intramuscular; SID = once daily; BID = twice daily

10.3.1. Methylprednisolone Succinate

Dose Route: Intravenous infusion into a lateral tail vein (or alternate peripheral vein if necessary) using an infusion pump connected to a temporary indwelling catheter (Abbocath®)

Following the end of infusion, the Abbocath® will be flushed with 0.5 mL of 0.9% sodium chloride for injection.

Frequency: Once on Day 1 for 30 minutes, prior to dosing (within 4 hours of dosing)

Infusion Rate: 1 mL/kg/h

Method: Dose formulations will be allowed to warm up at ambient temperature for at least 30 minutes prior to dosing, as appropriate. The animals will be temporarily restrained for dose administration and will not be sedated.

10.3.2. Methylprednisolone Acetate

Dose Route: Intramuscular injection into the lateral compartment of the thigh

Duration: From Days 1 to Day 62

Frequency: Once daily; first dose the morning of dosing (within 4 hours of dosing)

Method: Dose formulations will be allowed to warm up at ambient temperature for at least 30 minutes prior to dosing, as appropriate. The animals will be temporarily restrained for dose administration and will not be sedated.

10.3.3. Rapamycin

Dose Route: Intramuscular injection into the lateral compartment of the thigh

Duration: From Days -14 to Day 62

Frequency: Twice daily; 12 hr ± 3 hrs apart

Method: Dose formulations will be allowed to warm up at ambient temperature for at least 30 minutes prior to dosing, as appropriate. The animals will be temporarily restrained for dose administration and will not be sedated.

11. IN-LIFE PROCEDURES, OBSERVATIONS, AND MEASUREMENTS

General In-life Assessments – Main Study Animals

Parameter	Frequency	Comments
**Mortality/ Moribundity Checks**[a]	Twice daily (morning and afternoon) starting upon arrival through termination.	Animals will be observed within their cage unless removal is necessary for identification or confirmation of possible findings.
**Detailed Clinical Observations**[a]	Weekly starting Day -1, and at least every 2 weeks during the prestudy period.	Animals are removed from the cage
**Individual Body Weights**[a]	Weekly starting Day -1, and at least every 2 weeks during the prestudy period.	Fasted weight on the day of necropsy. No terminal body weights collected from animals found dead or preterminally euthanized.
**Food Evaluation**[a]	Once daily; from at least Week -1 and throughout the study	Qualitative
**Neurological Examinations**[a]	Once pretreatment, on Day 1 at 4 to 6 hours postdose, on Days 2, 7 and 28, and at the end of the observation period.	Assessment for general attitude and motor function, postural reactions, and cranial and spinal nerve functions; performed on unanesthetized animals.

[a] Minimum required frequency for this parameter indicated

11.1. Nerve Conductivity Velocity (NCV)

Frequency: Once prestudy and on Days 28 and 56.

Anesthesia: An intramuscular injection of ketamine, glycopyrrolate and dexmedetomidine will be administered following an appropriate fasting period. Following the completion of the assessment, a reversal agent, atipamezole, will be administered if required.

Evaluation: Peroneal motor: NCV and Amplitude
Sural sensory: NCV and Amplitude
Cauda equina: Onset latency

12. CLINICAL PATHOLOGY

12.1. Sample Collection

Clinical Pathology Sample Collection – Main Study Animals

Group Nos.	Occasion/ Time Point	Hematology	Coagulation	Clinical Chemistry	Urinalysis	Additional Serum
All animals	Pretreatment	X	X	X	X	X
1 to 3	Day 2	X	X	X	X	X
1 to 3	Week 1	X	X	X	X	X
1 to 3	Week 2	-	-	X	-	X
1 to 3	Week 3	-	-	X	-	X
1 to 3	Week 4	X	X	X	X	X
1 to 3	End of recovery	X	X	X	X	X
Unscheduled euthanasia (when possible)		X	X	X	-	X
**Overnight Fasting:**		-	-	Yes	Yes	-
**Method/Comments:**		Venipuncture	Venipuncture	Venipuncture	morning collection while water deprived	Venipuncture
**Target Volume (mL)[a]:**		0.7	1.2	0.7	As available	1.2
**Anticoagulant:**		EDTA	Sodium citrate	None, in SST	-	None, in SST
**Special Requirements:**		-	-	-	-	-
**Processing:**		None	Plasma	Serum	-	Serum

X = Sample to be collected; - = Not applicable; SST = Serum separator tube.
[a] Additional samples may be obtained (e.g., due to clotting of non-serum samples) if permissible sampling frequency and volume are not exceeded.

12.2. Hematology

Hematology Parameters

Red blood cell count<br>Hemoglobin concentration<br>Hematocrit<br>Mean corpuscular volume<br>Red blood cell distribution width<br>Mean corpuscular hemoglobin concentration<br>Mean corpuscular hemoglobin<br>Reticulocyte count (absolute)	Platelet count<br>White blood cell count<br>Neutrophil count (absolute)<br>Lymphocyte count (absolute)<br>Monocyte count (absolute)<br>Eosinophil count (absolute)<br>Basophil count (absolute)<br>Large unstained cells (absolute)

A blood smear will be prepared from each hematology sample and may be examined for confirmation of hematology results.

12.3. Coagulation

Coagulation Parameters

Activated partial thromboplastin time<br>Fibrinogen	Prothrombin time<br>Sample quality

12.4. Clinical Chemistry

Clinical Chemistry Parameters

Alanine aminotransferase<br>Aspartate aminotransferase<br>Alkaline phosphatase<br>Gamma-glutamyltransferase<br>Creatine kinase<br>Total bilirubin[a]<br>Urea nitrogen<br>Creatinine<br>Calcium<br>Phosphorus	Total protein<br>Albumin<br>Globulin<br>Albumin/globulin ratio<br>Glucose<br>Cholesterol<br>Triglycerides<br>Sodium<br>Potassium<br>Chloride<br>Sample quality

[a] When total bilirubin is > 1.0 mg/dL, direct bilirubin will also be measured and indirect bilirubin will be calculated.

12.5. Urinalysis

Urinalysis Parameters

Color<br>Appearance/Clarity<br>Specific gravity<br>Volume<br>pH	Protein[a]<br>Glucose[a]<br>Bilirubin<br>Ketones<br>Blood

[a] Semi-quantitative measurement

12.6. Additional Serum Sample Processing

The samples will be centrifuged and the resultant serum will be separated, transferred to 2 uniquely labeled polypropylene tubes, and frozen immediately over dry ice or in a freezer set to maintain -80°C within 2 hours of collection.

Samples will be shipped to the Sponsor for archival. Analysis, if conducted, will be added by amendment.

12.7. Bone Marrow Smear Evaluation

Bone marrow smears will be collected and prepared as described in Attachment A and may be examined for confirmation of results.

13. CYTOKINES COLLECTION AND ANALYSIS

Cytokines Sample Collection

Group Nos.	Time Postdose on Day 1			
	Predose	30 min	4 hr	24 hr
1 to 3	X	X	X	X
Unscheduled euthanasia (when possible)	X			

**Method/Comments:**	Venipuncture
**Target Volume[a]:**	0.5
**Anticoagulant:**	K_2EDTA
**Special Requirements:**	-
**Processing:**	Plasma

X = Sample to be collected; min = Minute; hr = Hour.

[a] Additional blood samples may be obtained (e.g., due to sample quality) if permissible sampling frequency and blood volume are not exceeded.

13.1. Cytokine Sample Processing

The samples will be centrifuged and the resultant plasma will be separated, transferred to 2 uniquely labeled polypropylene tubes (target 1 x 75 µL + leftover), and frozen immediately over dry ice or in a freezer set to maintain -80°C within 2 hours of collection.

13.2. Cytokine Sample Analysis

Analysis for cytokines (IL-6, IL-8, IL-10, IP-10, KC-like, MCP-1, and TNF-α) will be conducted using a multiplex bead-based method (Luminex). The procedures to be followed during the course of this study along with the assay acceptance criteria will be detailed in CR-SEN analytical procedure AP.BMK.mkpCyt.xx, in which “xx” denotes the version number. The cytokine method was validated under CR-SEN Study No. 3600083, however some cytokines have method limitations when used together in a multiplex panel. These limitations will be taken into consideration and discussed in the interpretative phase report.

13.3. Bioanalytical Sample Analysis

Bioanalytical Sample Collection

Group Nos.	Prestudy
1 to 3	X

**Method/Comments:**	Venipuncture
**Target Volume[a]:**	1
**Anticoagulant:**	None, in SST
**Special Requirements:**	None
**Processing:**	Serum

X = Sample to be collected.
[a] Additional blood samples may be obtained (e.g., due to sample quality) if permissible sampling frequency and blood volume are not exceeded.

The samples will be centrifuged and the resultant serum will be separated, transferred to 2 uniquely labeled polypropylene tubes, and frozen immediately over dry ice or in a freezer set to maintain -80°C.

Samples will be kept for possible future analysis of anti-AAV9 antibodies using a cell-based neutralizing antibody assay.

14. TERMINAL PROCEDURES

Terminal procedures are summarized in the following tables:

Replaced, Found Dead, and Unscheduled Euthanasia Animals

Animals	Necropsy Procedures			Histology Processing	Microscopic Evaluation
	Necropsy	Tissue Collection	Organ Weights		
Animals replaced prestudy	X	Standard Diagnostic List	-	-	-
Animals replaced after dosing start	X	Full List[a]	-	-	-
Unscheduled deaths after dosing start	X	Full List[a]	-	Full List[a]	Full List[a]

Main Study Animals

Group No.	Scheduled Euthanasia Day	Necropsy Procedures			Histology Processing	Microscopic Evaluation
		Necropsy	Tissue Collection	Organ Weights		
1	63	X	Full List[a]	Full List[a]	Full List[a]	Select Tissues[b]
2						
3						

X = Procedure to be conducted; - = Not applicable.
[a] See Tissue Weighing, Collection, Processing and Evaluation table in Attachment A for list of tissues applicable to each procedure.
[b] Brain, spinal cord (cervical, thoracic, lumbar), dorsal root ganglia (cervical, thoracic, lumbar), liver, spleen, lung, kidney, and heart.

14.1. Method of Euthanasia

Exsanguination by incision of the axillary or femoral arteries following anesthesia by intravenous injection of sodium pentobarbital, unless deemed inappropriate by the Study Director and/or the clinical veterinarian. A sedative, ketamine HCl for injection, USP will be administered by intramuscular injection before animals are transported from the animal room to the necropsy area.

14.2. Unscheduled Euthanasia

Main Study animals to be euthanized for humane reasons before the scheduled time will undergo sample collection for evaluation of clinical pathology parameters, cytokine analysis, and bioanalysis, if possible as specified in Section 12.1, 13, and 14.7.

Tissues from animal replaced after the start of dosing will be retained (as per Tissue Collection and Preservation section) and any data generated will not be included in the report unless deemed appropriate by the Study Director.

14.3. Scheduled Euthanasia

Main Study animals surviving until scheduled euthanasia will be food deprived overnight. When possible, the animals will be euthanized rotating across dose groups such that similar numbers of animals from each group, including controls, will be necropsied throughout the day.

14.4. Necropsy

Animals as detailed in the Terminal Procedures table will be subjected to a complete necropsy examination, which will include evaluation of the carcass and musculoskeletal system; all external surfaces and orifices; cranial cavity and external surfaces of the brain; and thoracic, abdominal, and pelvic cavities with their associated organs and tissues. Necropsy examinations will be performed by qualified personnel with appropriate training and experience in animal anatomy and gross pathology. A veterinary pathologist, or other suitably qualified person, will be available for consultation.

Images may be generated for illustration of or consultation on gross observations. These images will not be used for data generation or interpretation, and will not be archived or included in the final report.

14.5. Organ Weights

The organs detailed in the Terminal Procedures and Tissue Weighing, Collection, Processing and Evaluation (Attachment A) tables will be weighed at necropsy. Paired organs will be weighed together. In the event of gross abnormalities, in addition to the combined weight, the weight of each organ of a pair may be taken and entered as a tissue comment. Organ weight as a percent of body weight (using the terminal body weight) and organ weight as a percent of brain weight will be calculated.

14.6. Tissue Collection and Preservation

Representative samples of tissues will be collected and preserved in 10% neutral buffered formalin, except for tissues requiring alternate fixatives as defined by standard operating procedures, as detailed in the Terminal Procedures and Tissue Weighing, Collection, Processing and Evaluation (Attachment A) tables. Additional tissue samples may be collected to elucidate abnormal findings.

14.7. Tissue Collection for Bioanalysis

3 samples of 4x4x4 mm each, except for DRG where 1 sample/segment will be collected) of the spinal cord (cervical, lumbar, and thoracic), dorsal root ganglia (cervical, lumbar, and thoracic), liver, spleen, kidney, lung, skeletal muscles (gastrocnemius and biceps femoris), sciatic nerve, tibial nerve, trigeminal ganglia, testis/ovaries, thymus, and heart will be collected, weighed, placed in polypropylene tubes on dry ice, and transferred to a freezer set to maintain -80°C until shipment. Clean removal techniques will be used.

At scheduled euthanasia, the brain (cerebellum, hippocampus, cortex [frontal, parietal, occipital, and temporal lobes], striatum, thalamus, midbrain, pons, medulla) of each animal in all groups will be sectioned at 4-mm coronal slice thickness (see Attachment B) using clean removal technique. The second slice and every other slice thereafter will be fixed in 10% neutral buffered formalin for histopathological evaluation. The third slice and every other slice thereafter will be used to collect tissue punches (8 x 8 x 4 mm, when possible) for bioanalysis (BA) according to the table in Section 14.8. The sampling of brain slices for histopathology and BA analysis is summarized in Attachment D. Each punch will be sectioned in half (sagittal section relative to brain), as to provide duplicate samples of each structure. All punches for BA will be weighed (before freezing). Tissue punches for BA will be placed in PCR clean vials, flash frozen in liquid nitrogen and stored at -80°C until analysis. For animals found dead or euthanized early, the whole brain will be fixed in formalin for histopathology.

When possible, the order of tissue collection will be as follows: spleen, liver, kidney, brain then remaining tissues.

Tissues for histopathology will be collected prior to sample collection for any other parameters.

The samples will be shipped to the University of Texas Southwestern Medical Center, see Attachment B. The bioanalytical laboratory will be notified before shipment of the samples. Samples will be stored at the bioanalytical laboratory in a freezer set to maintain -60ºC or below until analysis.

Samples will be analyzed for vector genome biodistribution and transgene expression by qPCR. All analytical work will be conducted by the Sponsor, using an analytical method developed and qualified by that laboratory.

14.8. Splenocyte Sample Collection Analysis

The spleen will be collected using cell culture clean procedures from all animals at scheduled necropsy. Dissected spleens, a sample of approximately 1x1cm, weighed will be placed into prechilled tubes containing RPMI media and stored at 2°C to 8°C or on wet ice before processing to splenocytes. Splenocytes will then be cryopreserved/frozen until analysis.

Samples will be processed at ambient temperature according to standard CR-SEN SOP and analytical procedure AP.5550015.SPL.xx (where xx denote the version of the procedure), except that all washes will be performed with RPMI-1640 Media and samples will be frozen as follows:

Prepare a sufficient number of 2 mL cryovials to hold the cells at 2e7 cells/mL. Open the vials and add 100 µL of Hybridoma-Grade DMSO (Freezing media is 90% heat-inactivated FBS and 10 % DMSO). Add sufficient heat-inactivated FBS to the cell pellet to put the cells at 2e7/mL when added to the DMSO. Gently resuspend the pellet via pipetting and then add to the tubes containing DMSO. The act of addition and higher density of FBS will mix the FBS and DMSO yielding an evenly distributed freezing media. 1-2 gentle inversions can be used to assure even mixing. If adequate splenocytes are available, 3 aliquots of cells at 2e7/mL will be prepared for each animal. Splenocytes will be stored in the vapor phase of liquid nitrogen until shipped to the Sponsor on dry ice. See Attachment B for shipping details.

The splenocytes will be analyzed for T-cell responses against AAV9 and AP4M1. All analytical work will be conducted by the Sponsor, using an analytical method developed and qualified by that laboratory.

15. MICROSCOPIC EVALUATION

15.1. Histology

Tissues in the Terminal Procedures and Tissue Weighing, Collection, Processing and Evaluation (Attachment A) tables from animals identified in the Terminal Procedures table will be embedded in paraffin, sectioned, mounted on glass slides, and stained with hematoxylin and eosin.

15.2. Microscopic Evaluation

Tissues as detailed in the Terminal Procedures and Tissue Weighing, Collection, Processing and Evaluation (Attachment A) tables will be evaluated histopathologically by a veterinary pathologist with training and experience in laboratory animal pathology.

Special stains may be used at the discretion of the pathologist to further characterize lesions and changes identified during routine evaluation of individual animals. Any special stains will be documented in the individual animal data. Any additional stains or evaluations, if deemed necessary by the pathologist, may be added by study plan amendment following discussion with the Study Director and in consultation the Sponsor. Efforts will be made to evaluate all study plan-required tissues microscopically; however, it is not always feasible for every study plan-required tissue to be present on every slide. Study plan-required tissues that are not examined

will be documented in the histopathology data and the impact of these missing tissues on the study will be documented in the pathology report.

Images may be generated for illustration of or consultation on histological observations. These images will not be used for data generation or interpretation, and will not be archived or included in the Final Report.

15.3. Pathology Peer Review

A pathology peer review, will be conducted by:

Peer Review Pathologist:	Elaine Debien, DVM, DES, MSc, DACVP<br>Charles River Laboratories Montreal ULC<br>Senneville Site (CR-SEN)<br>Address as cited for Test Facility<br>Tel: 819.346.8200, ext 7016<br>E-mail: elaine.debien@crl.com

Histopathology slides will be shipped to the pathologist, see Attachment B for shipping details.

The peer review statement or equivalent documentation will be included as an appendix to the Final Report.

16. STATISTICAL ANALYSIS

Data will be presented as individual values by animal.

16.1. Constructed Variables

Body Weight Changes:	Calculated between each scheduled interval as well as between the following intervals: from beginning to end of each phase.
Organ Weight Relative to Body Weight:	Calculated against the terminal body weight for scheduled intervals.
Organ Weight Relative to Brain Weight:	Calculated against the brain weight for scheduled intervals.

Additional or alternative body weight intervals may be evaluated to elucidate study results at the discretion of the Study Director.

17. COMPUTERIZED SYSTEMS

The following critical computerized systems may be used in the study. The actual computerized systems will be documented in the study data and/or the Final Report.

Critical Computerized Systems

System Name	Description of Data Collected and/or Analyzed
Provantis®	In-life; clinical pathology; postmortem; Test Material receipt, accountability and/or formulation activities
Deviation Information Library	Deviations
Share Document Management System	Reporting
M-Files®	Reporting and collection of 21 CFR Part 11 compliant signature
eInfotree	Excel Module for collection of 21 CFR Part 11 compliance requirements, security, audit trail, and electronic signatures
DocuSign™	Collection of 21 CFR Part 11 compliant signature
SRS (CR-SEN in-house application built with SAS) and/or in-house reporting software Nevis 2012 (using SAS)	Cytokines
Mesa Laboratories AmegaView CMS	Continuous Monitoring System. Monitoring of standalone fridges, freezers, incubators, and selected laboratories to measure temperature, relative humidity, and CO_2, as appropriate
Johnson Controls Metasys	Building Automation System. Control of HVAC and other building systems, as well as temperature/humidity control and trending in selected laboratories and animal rooms
Watson LIMS	Sample management and data regression for cytokines assessment
Bio Plex Manager (Luminex)	Data collection for Cytokines
Acq*Knowledge*	Electrophysiological recording for nerve conduction velocity
Excel	Tabulated data entry for nerve conduction velocity
SRS (CR-SEN in-house application built with SAS)	Table formatting for nerve conduction velocity

Data for parameters not required by the Study Plan, which are automatically generated by analytical devices used will be retained on file but not reported. Statistical analysis results that are generated by the program but are not required by the Study Plan and/or are not scientifically relevant will be retained on file but will not be included in the tabulations.

18. REGULATORY COMPLIANCE

This study is not within the scope of regulations governing the conduct of nonclinical laboratory studies and is not intended to comply with such regulations.

19. AMENDMENTS AND DEVIATIONS

Changes to the approved Study Plan shall be made in the form of an amendment, which will be signed and dated by the Study Director. Every reasonable effort will be made to discuss any necessary study plan changes in advance with the Sponsor. The Study Director will notify the Sponsor of deviations that may result in a significant impact on the study as soon as possible.

20. RETENTION AND DISPOSITION OF RECORDS, SAMPLES, AND SPECIMENS

All study-specific raw data, electronic data, documentation, study plan (and amendments, if any), retained samples and specimens, and interim (if applicable) and final reports will be archived by no later than the date of final report issue. All materials generated by Charles River from this study will be transferred to a Charles River archive. At least 1 year after issue of the Draft Report, the Sponsor will be contacted.

Disposition of residual/retained analytical samples will be as described in the table below.

Disposition of Residual/Retained Samples

Sample Type	Disposition	Schedule
Bioanalytical (tissues)	Archived by the Sponsor	Samples to be archived by the Sponsor
Frozen tissues		

Records to be maintained will include, but will not be limited to, documentation and data for the following:

- Deviations, study plan, and study plan amendments
- Study schedule
- Study-related correspondence
- Test system receipt, health, and husbandry
- Test and reference item receipt, identification, preparation, and analysis
- In-life measurements and observations
- Clinical pathology sample collection and evaluation
- Cytokine sample collection and evaluation
- Gross and microscopic observations and related data
- Organ weight measurements
- Statistical analysis results

- **STUDY CLASSIFICATION**

Study Category:	Toxicology
Study Type:	Single Dose Toxicity
Study Design:	Parallel
Primary Treatment CAS Registry Number:	Not Available
Primary Treatment Unique Ingredient ID:	Not Available
Class of Compound:	AAV9 Vector
Administration Dose Form:	Solution

21. REPORTING

An abbreviated Draft Report will be prepared following completion of the study and will be finalized following consultation with the Sponsor. The report will include a summary of the experimental methods and all information necessary to provide a complete and accurate description of the results and any circumstances that may have affected the quality or integrity of the study.

The Sponsor will receive an electronic version of the Draft and Final Report provided in Adobe Acrobat PDF format (hyperlinked and searchable) along with a Microsoft Word version of the text. The PDF document will be created from native electronic files to the extent possible, including text and tables generated by the Test Facility. Report components not available in native electronic files and/or original signature pages will be scanned and converted to PDF image files for incorporation.

Reports should be finalized within 6 months of issue of the Draft Report. If the Sponsor has not provided comments to the report within 6 months of draft issue, the report will be finalized by the Test Facility unless other arrangements are made by the Sponsor.

22. JUSTIFICATIONS AND GUIDELINES

22.1. Justification of Test System and Number of Animals

At this time, studies in laboratory animals provide the best available basis for extrapolation to humans and are required to support regulatory submissions. Acceptable models that do not use live animals currently do not exist.

The cynomolgus monkey was chosen as the animal model for this study as it is an accepted nonrodent species for nonclinical toxicity testing by regulatory agencies.

The total number of animals to be used in this study is considered to be the minimum required to properly characterize the effects of the test item. This study has been designed such that it does not require an unnecessary number of animals to accomplish its objectives.

22.2. Justification of Route and Dose Levels

The intrathecal route of exposure was selected because this is the intended route of human exposure.

The dose levels were selected based on information provided by the Sponsors' prior studies with AAV9/AP4M1 in mice, as well as prior pharmacology for other similar AAV9 vectors in mice, rats, pigs, and nonhuman primates. Based on those prior pharmacology studies, the chosen low dose is anticipated to provide efficacy in ongoing mouse studies which should translate to humans. No clear dose-limiting toxicities were observed at doses equivalent to the low or high dose in mice. At one year postdose, 3 out of 10 mice receiving the high dose had hepatocellular adenoma, which could have been caused by the test item. The low dose in this NHP study corresponds to a nearly maximum feasible dose (MFD) due to limitations on injection volume

and test item concentration, and this is the target dose for human translation. A 2x higher dose is achieved using a higher injection volume, and is included to evaluate a possible safety margin. None of the doses are expected to generate more than a minimal to moderate toxic effect. If any adverse effects are observed, the range of doses in the study design is an attempt to produce a graded response.

22.3. Guidelines for Study

The design of this study was based on the study objective(s) and the overall product development strategy for the test item.

23. ANIMAL WELFARE

The Study Plan and any amendment(s) or procedures involving the care and use of animals in this study will be reviewed and approved by CR-SEN Institutional Animal Care and Use Committee (IACUC). During the study, the care and use of animals will be conducted with guidance from the USA National Research Council and the Canadian Council on Animal Care (CCAC).

24. REFERENCES

Office of Laboratory Animal Welfare. *Public Health Services Policy on Humane Care and Use of Laboratory Animals*. Bethesda, MD: National Institutes of Health. Current edition.

TEST FACILITY APPROVAL

All electronic signatures appear at the end of the document upon finalization.

Appendix 1

SPONSOR APPROVAL

The Study Plan was approved by the Sponsor by e-mail on the date designated below. The correspondence giving approval will be archived, as appropriate with other Sponsor communications.

29 Jun 2021

Date of Sponsor Approval

ATTACHMENT A

Tissue Weighing, Collection, Processing, and Evaluation Table

Organ	Weigh	Macroscopic Evaluation and Collection	Histology Processing	Microscopic Evaluation	Bioanalysis
Animal ID	-	X	-	-	-
Artery, aorta	-	X	X	-	-
Bone marrow, sternum	-	X	X	-	-
Bone marrow smear	-	X[a]	-	-	-
Bone, femur, right	-	X	X	-	-
Bone, sternum	-	X	X	-	-
Brain	X	X	X	X (left)	X (right) (cerebellum, hippocampus, cortex (frontal, parietal, occipital, and temporal lobes), striatum, thalamus, midbrain, pons, medulla)
Epididymis	X (2)	X (2)	X (2)	-	-
Esophagus	-	X	X	-	-
Eye	-	X (2)	X (2)	-	-
Gallbladder	- [b]	X	X	-	-
Ganglion, dorsal root, (cervical, lumbar, and thoracic with dorsal and ventral nerve roots)[f]	-	X (2)	X (2)	X (2)	X – DRG only (2) (C6-C7, T1-T2, L4)
Ganglion, trigeminal	-	X	X (left)	X (left)	X (right)
Gland, adrenal	X (2)	X (2)	X (2)	-	-
Gland, lacrimal	-	X (2)	-	-	-
Gland, mammary	-	X	X	-	-
Gland, parathyroid	-[c]	X (2)	X (2)	-	-
Gland, pituitary	X	X	X	-	-
Gland, prostate	X	X	X	-	-
Gland, salivary, submandibular	-	X (2)	X (1)	-	-
Gland, salivary, sublingual	-	X (2)	-	-	-
Gland salivary, parotid	-	X (2)	-	-	-
Gland, seminal vesicle	-	X (2)	X (2)	-	-
Gland, thyroid	X (2)	X (2)	X (2)	-	-
Gut-associated lymphoid tissue[d]	-	X	X	-	-
Heart	X	X	X	X	X
Joint, femorotibial, right	-	X	X	-	-
Kidney	X (2)	X (2)	X (2)	X (2)	X (right)
Large intestine, cecum	-	X	X	-	-
Large intestine, colon	-	X	X	-	-

Appendix 1

Organ	Weigh	Macroscopic Evaluation and Collection	Histology Processing	Microscopic Evaluation	Bioanalysis
Large intestine, rectum	-	X	X	-	-
Liver	X	X	X	X	X (left lateral)
Lung	-	X	X	-	X
Lymph node(s) draining administration site(s): deep cervical and iliac	-	X (2)	X (2)	X (2)	-
Lymph node, mandibular	-	X (2)	X (1)	-	-
Lymph node, mesenteric	-	X	X	-	-
Muscle, skeletal, gastrocnemius	-	X (2)	X (1)	-	X (1, right)
Muscle, skeletal, biceps femoris	-	X (2)	-	-	X (1, right)
Nerve, optic	-	X (2)	X (2)	-	X (1, right)
Nerve, sciatic	-	X (2)	X (1)	X (1)	X (1, right)
Nerve, tibial	-	X (2)	-	-	X (1, right)
Ovary	X (2)	X (2)	X (2)	-	X (1, right)
Oviduct	-	X (2)	-	-	-
Pancreas	-	X	X	-	-
Skin	-	X	X	-	-
Small intestine, duodenum	-	X	X	-	-
Small intestine, ileum	-	X	X	-	-
Small intestine, jejunum	-	X	X	-	-
Spinal cord (cervical, lumbar, and thoracic, including injection site)[e]	-	X	X	X	X (C6-C7, T9-T12, L4)
Spleen	X	X	X	X	X
Stomach	-	X	X	-	-
Testis	X (2)	X (2)	X (2)	-	X (1, right)
Thymus	X	X	X	-	X
Tongue	-	X	X	-	-
Trachea	-	X	X	-	-
Ureter	-	X (2)	-	-	-
Urinary bladder	-	X	X	-	-
Uterus/Cervix	X	X	X	-	-
Vagina	-	X	X	-	-

Appendix 1

Organ	Weigh	Macroscopic Evaluation and Collection	Histology Processing	Microscopic Evaluation	Bioanalysis

X = Procedure to be conducted. - = Not applicable. (1) = one side. (2) = both sides.

Macroscopic abnormalities in the organs listed and in other organs will be sampled at necropsy, processed for histology and examined microscopically.

[a] Bone marrow smears will be collected from the 5th to 7th rib at scheduled and unscheduled necropsies (for possible examination). Smears will not be collected from animals that are found dead or from animals that were euthanized moribund and then stored in the refrigerator prior to necropsy. Bone marrow smears are allowed to air dry and are not fixed in formalin.

[b] Weigh with liver.

[c] Weigh with gland, thyroid.

[d] From small intestine: Peyer’s patch or solitary lymphoid follicle.

[e] Transverse and/or oblique sections.

[f] Nerve roots will be examined if present in section of dorsal root ganglia.

ATTACHMENT B

Brain Collection Procedure

1. The brain matrix to be used will be chilled in a saline ice bath. Following collection of the brain, it will be placed in the matrix in the ice bath for approximately 10 minutes to firm up the tissue prior to processing
2. The brain slicing will be performed as per diagram below:

Slice number

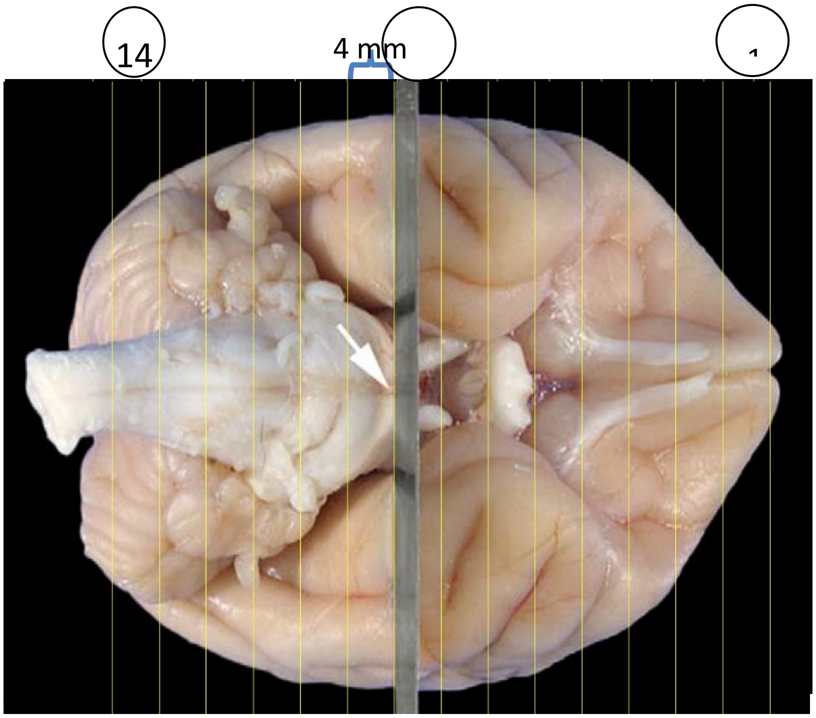

NOTE: The initial razor cut should be consistent for all specimens (i.e. start from rostral to pons), with all subsequent slices taken 4mm apart. The starting location for slicing will be recorded with in the study data.

3. Following complete sectioning of the brain within the matrix, the brain will be carefully removed and each individual brain slab will be laid out flat in consecutive order, with the caudal face of each slab oriented against the table/bench (rostral face of each slice will be face up).
4. Each brain slab will then be cut sagitally, to separate the hemispheres Right and Left.
5. The hemisphere will then be punched as per Sections 15.7, 15.8, 15.9 and Attachment D, and the remaining tissue from both hemispheres will be frozen flat indirectly on dry ice, then placed flat into appropriately sized cassettes labeled with group number, animal number and brain section number and stored at -80°C or placed in 10% NBF.

Appendix 1

ATTACHMENT C

Shipment of Samples and Study Records

Matrix	Purpose	Day/ Week/ Aliquot	Proposed Shipment Date	Conditions for Shipment	Recipient/Address
Bulk test item<br>Residual Dose Formulations	Return to Sponsor	Not applicable	Following completion of dosing	Dry ice	Attention to:<br>Steven Gray, PhD<br>University of Texas Southwestern Medical Center<br>NA2.508<br>5323 Harry Hines Blvd<br>University of Texas Southwestern Medical Center<br>Dallas, TX 75390<br>Tel: 214-648-0670<br>E-mail: steven.gray@UTSouthwestern.edu
Additional Serum	Archival	Not applicable	Following the last collection	Dry ice	Attention to:<br>Juan A. Rodriguez<br>University of Texas Southwestern 5901 Forest Park Rd,<br>NA2.508 Receiving – North Campus Dock<br>Dallas, TX 75390<br>Tel: 281-380-5165 E-mail:<br>Juan.Rodriguez@UTSouthwestern
Tissues	Bioanalysis	Not applicable	Within 2 weeks from collection	Dry ice	Attention to:<br>Juan A. Rodriguez<br>University of Texas Southwestern 5901 Forest Park Rd,<br>NA2.508 Receiving – North Campus Dock<br>Dallas, TX 75390<br>Tel: 281-380-5165 E-mail:<br>Juan.Rodriguez@UTSouthwestern

Alternate shipping details may be provided thereafter and will be documented in study correspondence.

Appendix 1

Signatures:

Testing Facility Management Approval: I approve this document.

Name: **Parente, Carmela**

Parente, Carmela

29-Jun-2021 13:34:47 (UTC+00:00)

Electronically Signed in M-Files

Timestamp

Study Director Approval: I approve this document.

Name: **Cinquino, Stefania**

Cinquino, Stefania

29-Jun-2021 13:35:35 (UTC+00:00)

Electronically Signed in M-Files

Timestamp

Appendix 2

Certificate Of Analysis

Research grade - Not for human use.

Client identification: CURE SPG50

Product Name and type of product: rAAV9, sterile suspension of rAAV9 vector carrying the AP4M1 therapeutic gene.

Batch Number: T-GEMINIS-033

Quality grade: Research grade

Production process: 50 Liters batch

Manufacturing date: 22 December 2020

Formulation buffer (FMR-T-0043): MilliQ Water, 1X dPBS, 5% D-Sorbitol, 0.001% Pluronic

Vials Shipped: 34 x 0.5 ml vialed in polypropylene Cryotubes

Shipment Date: 19 January 2021

Transport conditions: Dry ice

Storage conditions: ≤-60ºC

For Research Use Only

Performed by:	Paz López (QC Coordinator)	Date: 29-APRIL-2021
Reviewed by	Sandy Douthe (QC Manager)	Date: 29 APRIL 2021
Reviewed by	Begoña Ortega (QA Technician)	Date: 29-APRIL 2021

Parque Tecnológico de Guipuzcoa - Paseo Mikeletegui, 83, 2ª Planta
20009 San Sebastián, Spain - Tel: +34 943 477 733 - viralgenvc.com

Appendix 2

VIRALGEN

Product: rAAV9-AP4M1
Batch Number: T-GEMINIS-033

rAAV Purified Bulk / Bulk Drug Substance assays:

ASSAY	METHOD	TEST SITE	SPECIFICATION	RESULT
**SAFETY ASSAYS**				
Replication competent AAV	PT/133 & PT/025 Infection of permissive cell line/Rep2 qPCR (based on ITRqPCR titration)	Genosafe	Report result	Not Detected <10 rcAAV in $1x10^{11}$ vg
**STRENGHT ASSAYS**				
Vector genome titer (vg/mL)	PNT-CC-005 ITR qPCR	Viralgen	report results	$1.71x10^{14}$
Vector genome titer (vg/ml)	PNT-CC-049 ITRddPCR	Viralgen	report results	$5.17x10^{13}$
**PURITY ASSAYS**				
General Purity	PNT-CC-012 SDS-Page/ silver stain	Viralgen	Report result	Detection VP1, VP2 and VP3. Presence of extra bands between 150-250 KDa
Residual Host Cell Protein (ng/mL)	PNT-CC-023 HEK293 ELISA Assay	Viralgen	Report result	<100
Residual Host Cell DNA (pg/mL with and without DNase)	PNT-CC-033 18S qPCR (123 and 254 bp amplicons)	Viralgen	Report result	123 bp: $6.86x10^6$ (+DNase I) $7.38x10^6$ (-DNase I) 254 bp: $5.02x10^6$ (+DNAseI) $5.21x10^6$ (-DNase I)
Residual Host Cell DNA (pg/mL, without DNase)	SP-M.8303 E1A qPCR	SGS-Vitrology	Report result	$2.6x10^6$
Residual Plasmid DNA (copies/mL with and without DNase)	PNT-CC-014 KanaR qPCR	Viralgen	Report result	$9.61x10^{11}$ (+Dnase I) $1.26x10^{12}$ (-Dnase I)
Full/Empty particles ratio	V6725 CryoTEM	Vironova	>50% full	Filled: 84% Empty: 9% Uncertain: 7%

For Research Use Only

Parque Tecnológico de Guipuzcoa - Paseo Mikeletegui, 83, 2ª Planta
20009 San Sebastián, Spain - Tel: +34 943 477 733 - viralgenvc.com

Appendix 2

VIRALGEN

Product: rAAV9-AP4M1
Batch Number: T-GEMINIS-033

ASSAY	METHOD	TEST SITE	SPECIFICATION	RESULT
**PURITY ASSAYS**				
Aggregation	V0149 & V0692 nsTEM	Vironova	Report result	92% of the individual particles at <40nm
Residual Chemical (Transfection reagent)	Refer to DMF	Docuchem	Report result	<25.13 ppm >12.57 ppm
Residual Chemical (Lysis reagent)	Refer to DMF		Report result	<LOD (1 ppm)
Residual Chemical (Clarifying reagent)	Refer to DMF		Report result	<1.03 ppm >0.21 ppm
Residual Chemical (Iodixanol)	HPLC		Report result	<3.12 ppm >1.04 ppm
Residual Chemical (Antifoam)	Refer to DMF		Report result	<LOD (5 ppm)
Residual Affinity Ligand (ng/mL)	PNT-CC-037 ELISA	Viralgen	Report result	511.3
**IDENTITY ASSAYS**				
Protein Identity	PNT-CC-003 SDS-PAGE/ Western Blot	Viralgen	Detection of VP1, VP2 & VP3	Detection of VP1, VP2 and VP3
Genome identity	PNT-143 Sequencing (Sanger)	Secugen	100% conform to sequence of reference	Conform

For Research Use Only

Parque Tecnológico de Guipuzcoa - Paseo Mikeletegui, 83, 2ª Planta
20009 San Sebastián, Spain - Tel: +34 943 477 733 - viralgenvc.com

Appendix 2

VIRALGEN

Product: rAAV9-AP4M1
Batch Number: T-GEMINIS-033

rAAV Final Product / Drug Product assays:

ASSAY	METHOD	TEST SITE	SPECIFICATION	RESULT
**SAFETY ASSAYS**				
Sterility	LTMI-009	Biolab S.L	No growth	No growth
Endotoxin (EU/ml)	PNT-CC-015 Kinetic chromogenic	Viralgen	<0.2	<0.051
[1]Mycoplasma	PNT-CC-001 PCR end point	Viralgen	Negative	Negative
**STRENGHT ASSAYS**				
Vector genome titer (vg/mL)	PNT-CC-005 ITR qPCR	Viralgen	$0.75x10^{14}$-$4x10^{14}$ Target $1.70x10^{14}$	$2.17x10^{14}$
Vector genome titer (vg/ml)	PNT-CC-049 ITRddPCR	Viralgen	Report Result	$5.43x10^{13}$
Infectious titer (TCID50/mL)	PNT-CC-004 TCID50/ITRqPCR	Viralgen	Report result	$1.02x10^{10}$
vg/TCID50 (ITRqPCR) ratio	N/A	Viralgen	Report result	21237.16
**QUALITY ASSAYS**				
Osmolality (mOsm/Kg)	PNT-CC-016 Freezing point	Viralgen	587 +/-50	570
pH	PNT-CC-044 Potentiometry	Viralgen	7.4±0.4	7.21
Appearence	PNT-CC-017 Visual inspection	Viralgen	Colorless, clear to slightly opalescent, free of visible particles	Not done because vialed in polypropylene cryovials
Particles size distribution	PNT-CC-053 DLS	Viralgen	Report Results	99-99.5% of particulate volume between 23.98-25.25 nm in mean diameter

[1] Mycoplasma assay performed in the transfection pool.

For Research Use

Parque Tecnológico de Guipuzcoa - Paseo Mikeletegui, 83, 2ª Planta
20009 San Sebastián, Spain - Tel: +34 943 477 733 - viralgenvc.com

Appendix 2

Product: rAAV9-AP4M1
Batch Number: T-GEMINIS-033

1. Test Information: General purity and protein identity by SDS-PAGE/Silver staining and Western Blot analysis

According to the standard operating procedure PNT-CC-012_Pureza del vector por SDS_PAGE_TINCION DE PLATA, and the PNT-CC-003_Identificación de proteínas por Western Blot.

1.1 Gel Properties: 10% Acrylamide gel

1.2. SDS-PAGE Results:

SDS-PAGE/Silver staining
5x10^9 vg/well (based on ITRqPCR titer result)

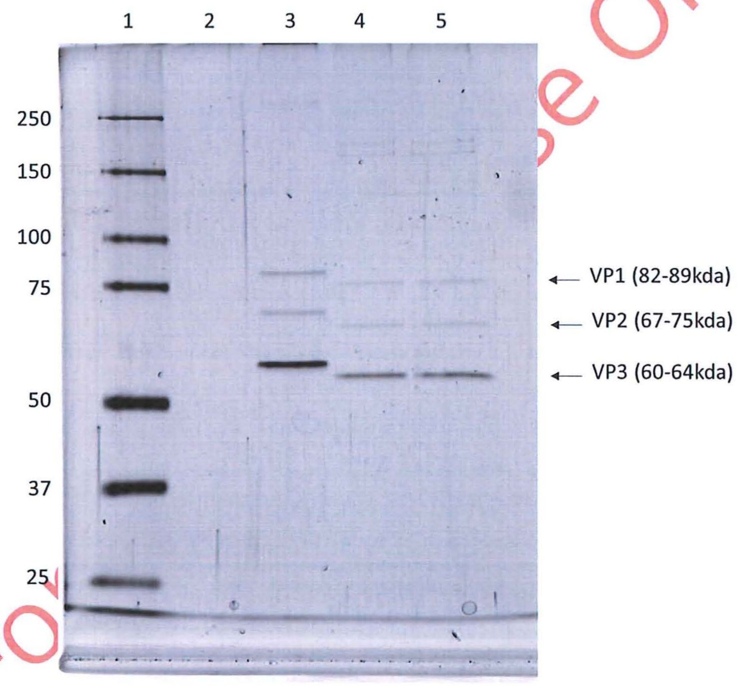

Lane 1: Ladder
Lane 2: Negative Control. Formulation Buffer FMR-T-0043
Lane 3: rAAV8 Standard vector ATCC VR-1816
Lane 4: T-GEMINIS-033 BDS
Lane 5: T-GEMINIS-033 BDS

Presence of extra-bands between 150-250 KDa. An investigational analysis was done and the extra bands were identified as encapsidated (or not accessible to DNase) DNA.

Parque Tecnológico de Guipuzcoa - Paseo Mikeletegui, 83, 2ª Planta
20009 San Sebastián, Spain - Tel: +34 943 477 733 - viralgenvc.com

Appendix 2

1.3. SDS-PAGE/Western blot Results:

**SDS-Page/Western Blot Results**<br>5x10^9 vg/well (based on ITRqPCR titer result)
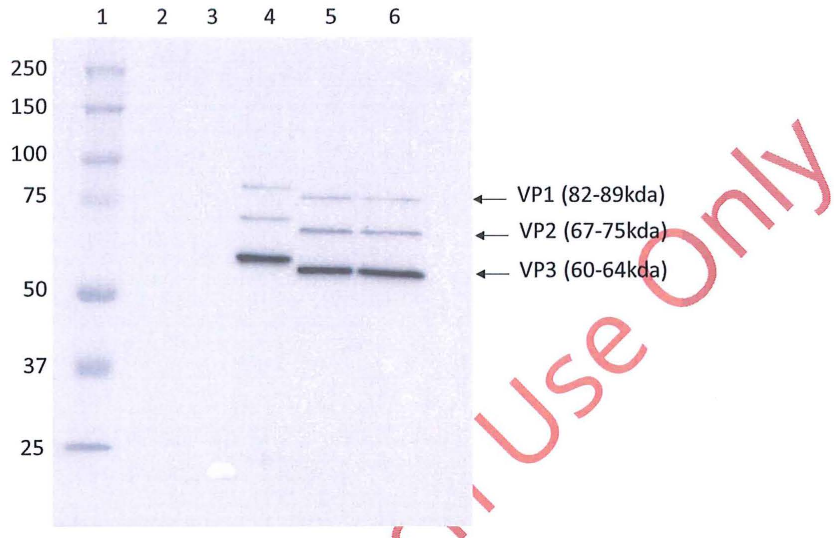
Lane 1: Ladder.<br>Lane 2: Empty<br>Lane 3: Negative Control. Formulation buffer FMT-T-0043<br>Lane 4: rAAV8 Standard vector ATCC VR-1816<br>Lane 5: T-GEMINIS-033 BDS<br>Lane 6: T-GEMINIS-033 BDS

For Research Use Only

Parque Tecnológico de Guipuzcoa - Paseo Mikeletegui, 83, 2ª Planta
20009 San Sebastián, Spain - Tel: +34 943 477 733 - viralgenvc.com

Appendix 2

Product Name: FMR-T-0043

Batch Number:2021-02-09/01

Certificate Of Analysis

Research grade - Not for human use

Name and type of product: FMR-T-0043/ Formulation buffer

Batch Number:2021-02-09/01

Date of manufacture: 9Th February 2021 (vialing date).

Composition:1xdPBS,5% sorbitol and 0.001% pluronic

Quality grade: Research grade

Primary container: Polypropilene Cryotubes 1,2 ml

Batch size: 30 vials containing 0,5 ml of buffer

FOR RESEARCH USE ONLY

Name and address of the manufacturer:

VIRALGEN - Parque Tecnológico de Gipuzkoa - Paseo Mikeletegi 83, 2ª Planta.

CP20009 San Sebastián, SPAIN. Tel: +34 943 477 733.

Appendix 2

Product Name: FMR-T-0043

Batch Number:2021-02-09/01

VIRALGEN

TEST	METHOD	TEST SITE	RESULT
(1)Bioburden	PNT-CC-026 Filtration	Viralgen	<1CFU/10 ml
(1)Endotoxins (EU/mL)	PNT-CC-015 Kinetic chromogenic	Viralgen	<0.050
(2)pH	Potenciometry	Viralgen	7.35
(2)Conductivity (mS/cm)	Conductimetry	Viralgen	14.08

(1) Assays done at Viralgen Vector Core Quality Control laboratories

(2) Assays done at the time of buffer preparation by the downstream Process team following the formulation record procedure described on FMR-T-0043

Performed by: Paz López (QC Deputy Director) **Date:** 05th January 2022

PLM

FOR RESEARCH USE ONLY

Parque Tecnológico de Gipuzkoa. Paseo Mikeletegi 83, 2ª Planta. CP20009 San Sebastián, SPAIN. Tel: +34 943 477 733
viralgenvc.com

Individual In-life Data Explanation Page

Individual Mortality

Abbreviation	Description	Abbreviation	Description
-	Not scheduled to be performed / Not seen	Path	Pathology
AM SIR	Signs of ill health or reaction to treatment check in the morning	PM SIR	Signs of ill health or reaction to treatment check in the afternoon
NR	Not recorded		

Individual Clinical Observations

Abbreviation	Description	Abbreviation	Description
./-	Not scheduled to be performed / Not seen / Dead	PAM	Detailed examination in the afternoon
AM_S	Signs of ill health or reaction to treatment check in the morning	Part	Particles
CAM	Cage side observation in the morning	PM_S	Signs of ill health or reaction to treatment check in the afternoon
Cp #	Cage side observation post dose	pr #/PR #/Pre	Observation predose
CPM	Cage side observation in the afternoon	(PT)	Permanent
Cpr	Cage side observation predose	Sev Not App	Severity not applicable
CSO	Cage side observation	Sev Not Rec	Severity not recorded
DAM	Detailed examination in the morning	SIRT	Signs of ill health or reaction to treatment
DE/D	Detailed examination	U #/Up #	Unscheduled observation post dose
DuRx	Observation during dosing	UDu #	Unscheduled observation during dosing
DW	Detailed examination weekly	Un #/Unsc #	Unscheduled observation
Fev	Food evaluation	Upr #	Unscheduled observation predose
IM	Intramuscular	Vet	Anything observed by veterinary staff
Inj	Injection	(VET)	Symptom recorded by veterinary staff
Inter	Internal	w/	with
OTHR	Other	#	Number to avoid using the same timeslot/animal/day
p #/ P #	Observation post dose		

Note: Only animals with findings are presented in this appendix.

Individual Body Weights

Abbreviation	Description	Abbreviation	Description
./-	Not scheduled to be performed/dead	OA	Omitted activity
< or >	Out of range	RC	Result comment
FC	Flag comment	TERR	Technical error
NT	Not taken	UPTD	Unable to perform due to technical difficulty

Individual Body Weight Gains

Abbreviation	Description	Abbreviation	Description
./-	Not scheduled to be performed/dead	OA	Omitted activity
< or >	Out of range	RC	Result comment
FC	Flag comment	TERR	Technical error
NT	Not taken	UPTD	Unable to perform due to technical difficulty

Individual Neurological Examinations Explanation Page

Abbreviation	Description	Abbreviation	Description
./-	Not scheduled to be performed	TERR	Technical error
NHP	Non-human primate	TNR	Test not reported
OA	Omitted activity	R	Right
Gen Att	General Attitude	RC	Result comment
Lat Pos	Lateral recumbency position	RF	Right Forelimb
L	Left	RH/hr	Right Hindlimb
LF	Left Forelimb	Reac / Rea	Reactions
LH/hl	Left Hindlimb	Ref	Reflex
Mot Function	Motor Function	Tact	Tactile
Not Eval	Not evaluated	Vis	Visual
Proprio Pos	Proprioceptive Positioning	Wheelbarrow	Wheel barrowing
hl	Hand left	hr	Hand right
DE	Detailed examination	4-6H	Observation 4 to 6 hours post dose

Note: This is a comprehensive list of abbreviations. All of the abbreviations listed may not be applicable to this report.

Dosing Information

Dosing information is abbreviated on various data outputs; the following represents the dosing information for this study.

Group No.	Test Material	Dose Level (vg)
1	Reference Item	0
2	AAV9/AP4M1	$8.4x10^{13}$
3	AAV9/AP4M1	$1.68x10^{14}$

Appendix 3

Individual Mortality

5550014

Sex: Male Day(s): - Relative to Start Date

0<br>vg<br><br>Group 1	Day of Death	Removal Date	Path Removal Reason
1201	94	14-Oct-2021	TERM

TE/TERM = Terminal Euthanasia FD = Found Dead UE/UNSC = Unscheduled Euthanasia
REL = Released
AD/ACCD = Accidental death
IE/INTM = Interim Euthanasia REC = Recovery Euthanasia

Appendix 3

Individual Mortality

5550014

Sex: Male Day(s): - Relative to Start Date

1.68 X10E14 vg Group 3	Day of Death	Removal Date	Path Removal Reason
3201	94	14-Oct-2021	TERM

TE/TERM = Terminal Euthanasia FD = Found Dead UE/UNSC = Unscheduled Euthanasia
REL = Released
AD/ACCD = Accidental death
IE/INTM = Interim Euthanasia REC = Recovery Euthanasia

Appendix 3

Individual Mortality

5550014

Sex: Female Day(s): - Relative to Start Date

0<br>vg<br><br>Group 1	Day of Death	Removal Date	Path Removal Reason
1701	94	14-Oct-2021	TERM

TE/TERM = Terminal Euthanasia FD = Found Dead UE/UNSC = Unscheduled Euthanasia
REL = Released
AD/ACCD = Accidental death
IE/INTM = Interim Euthanasia REC = Recovery Euthanasia

Appendix 3

Individual Mortality

5550014

Sex: Female Day(s): - Relative to Start Date

8.4 X10E13 vg Group 2	Day of Death	Removal Date	Path Removal Reason
2701	94	14-Oct-2021	TERM
2702	94	14-Oct-2021	TERM

TE/TERM = Terminal Euthanasia FD = Found Dead UE/UNSC = Unscheduled Euthanasia
REL = Released
AD/ACCD = Accidental death
IE/INTM = Interim Euthanasia REC = Recovery Euthanasia

Appendix 3

Individual Mortality

5550014

Sex: Female Day(s): - Relative to Start Date

1.68 X10E14 vg Group 3	Day of Death	Removal Date	Path Removal Reason
3701	94	14-Oct-2021	TERM

TE/TERM = Terminal Euthanasia FD = Found Dead UE/UNSC = Unscheduled Euthanasia
REL = Released
AD/ACCD = Accidental death
IE/INTM = Interim Euthanasia REC = Recovery Euthanasia

Appendix 4

Individual Clinical Observations

5550014

0<br>vg<br>Group 1<br>Sex: Male	Observation Type: All Types	Day(s) Relative to Start Date						
		-8<br>DE	-1<br>DE	7<br>DE	14<br>DE	21<br>DE	25<br>AM_S	28<br>DE
1201	Hunched Posture	.	.	.	.	.	.	.
	Fur, Thin Cover, Dorsal Aspect Generalized	.	.	.	.	.	.	.
	Fur, Thin Cover, Hindlimb, Left	.	.	.	.	.	.	.
	Fur, Thin Cover, Hindlimb, Right	.	.	.	.	.	.	.
	Fur, Thin Cover, Tail	.	.	.	.	.	.	.
	Skin, Dry, Tail	X	X	X	X	X	.	X
	Skin, Discolored, Hindlimb, Left, Red	.	.	.	.	.	.	X
	Skin, Discolored, Inguinal, Left, Red	.	X	X	X	X	.	.
	Skin, Discolored, Inguinal, Right, Red	.	.	.	.	.	.	X
	Skin, Scab, Tail	.	.	.	.	X	.	X
	Reduced Appetite, Severity Not Applicable	.	.	.	.	.	X	.
	Skin, Bruise, Inguinal, Right, Slight	.	.	X	X	.	.	.

X=Present

Appendix 4

Individual Clinical Observations

5550014

0<br>vg<br>Group 1<br>Sex: Male	Observation Type: All Types	Day(s) Relative to Start Date						
		35<br>DE	42<br>DE	48<br>PM_S	49<br>DE	56<br>DE	63<br>DE	70<br>DE
1201	Hunched Posture	.	.	X	.	.	.	.
	Fur, Thin Cover, Dorsal Aspect Generalized	.	.	.	.	.	X	X
	Fur, Thin Cover, Hindlimb, Left	.	.	.	X	X	X	X
	Fur, Thin Cover, Hindlimb, Right	.	.	.	.	.	.	.
	Fur, Thin Cover, Tail	.	.	.	X	X	X	X
	Skin, Dry, Tail	X	X	.	X	X	X	X
	Skin, Discolored, Hindlimb, Left, Red	.	.	.	.	.	.	.
	Skin, Discolored, Inguinal, Left, Red	.	.	.	.	.	.	.
	Skin, Discolored, Inguinal, Right, Red	.	.	.	.	.	.	.
	Skin, Scab, Tail	X	.	.	.	.	.	.
	Reduced Appetite, Severity Not Applicable	.	.	.	.	.	.	.
	Skin, Bruise, Inguinal, Right, Slight	.	.	.	.	.	.	.

X=Present

Appendix 4

Individual Clinical Observations

5550014

0<br>vg<br>Group 1<br>Sex: Male	Observation Type: All Types	Day(s) Relative to Start Date						
		77<br>DE	79<br>Unsc	84<br>DE	86<br>Unsc	88<br>Unsc	91<br>DE	94<br>DE
1201	Hunched Posture	.	.	.	.	X	.	.
	Fur, Thin Cover, Dorsal Aspect Generalized	X	X	X	X	.	X	.
	Fur, Thin Cover, Hindlimb, Left	X	X	X	X	.	X	.
	Fur, Thin Cover, Hindlimb, Right	.	.	.	.	.	.	X
	Fur, Thin Cover, Tail	X	X	X	X	.	X	X
	Skin, Dry, Tail	X	X	X	X	.	X	X
	Skin, Discolored, Hindlimb, Left, Red	.	.	.	.	.	.	.
	Skin, Discolored, Inguinal, Left, Red	.	.	.	.	.	.	.
	Skin, Discolored, Inguinal, Right, Red	.	.	.	.	.	.	.
	Skin, Scab, Tail	.	.	X	X	.	X	X
	Reduced Appetite, Severity Not Applicable	.	.	.	.	X	.	.
	Skin, Bruise, Inguinal, Right, Slight	.	.	.	.	.	.	.

X=Present

Individual Clinical Observations

5550014

1.68 X10E14 vg Group 3 Sex: Male	Observation Type: All Types	Day(s) Relative to Start Date						
		-8 DE	-1 DE	1 DuRx	7 DE	14 DE	21 DE	28 DE
3201	Tremors, Slight	.	.	X	.	.	.	.
	Muscle Tone, Forelimb, Left, Decreased	.	.	.	.	.	.	.
	Muscle Tone, Hindlimb, Right, Decreased	.	.	.	.	.	.	.
	Hunched Posture	.	.	.	.	.	.	.
	Fur, Erected	.	.	X	.	.	.	.
	Fur, Staining, Abdominal, Yellow	.	.	.	.	.	.	.
	Fur, Thin Cover, Dorsal Aspect Generalized	.	.	.	.	.	.	.
	Fur, Thin Cover, Hindlimb, Left	X	X	.	X	X	X	X
	Fur, Thin Cover, Hindlimb, Right	X	X	.	X	X	X	X
	Fur, Thin Cover, Lumbar	X	X	.	.	.	.	.
	Fur, Thin Cover, Tail	.	.	.	.	.	.	.
	Skin, Discolored, Abdominal, Yellow	X	X	.	.	.	.	.
	Skin, Discolored, Hindlimb, Left, Red	.	X	.	X	X	.	.
	Skin, Discolored, Inguinal, Left, Yellow	X	X	.	.	.	.	.
	Skin, Discolored, Inguinal, Right, Red	.	.	.	X	X	.	.
	Skin, Discolored, Inguinal, Right, Yellow	X	X	.	.	.	.	.
	Skin, Scab, Hindlimb, Right	.	.	.	X	X	X	X
	Skin, Scab, Tail	.	.	.	.	X	X	X
	Reduced Appetite, Severity Not Applicable	.	.	.	.	.	.	.
	Material Present, Foamy, White, Severe	.	.	.	.	.	.	.

X=Present

Appendix 4

Individual Clinical Observations

5550014

1.68 X10E14 vg Group 3 Sex: Male	Observation Type: All Types	Day(s) Relative to Start Date						
		35 DE	42 DE	48 PM_S	49 DE	56 DE	63 DE	70 DE
3201	Tremors, Slight	.	.	.	.	.	.	.
	Muscle Tone, Forelimb, Left, Decreased	.	.	.	.	.	.	.
	Muscle Tone, Hindlimb, Right, Decreased	.	.	.	.	.	.	.
	Hunched Posture	.	.	X	.	.	.	.
	Fur, Erected	.	.	X	.	.	.	.
	Fur, Staining, Abdominal, Yellow	.	.	.	.	.	.	.
	Fur, Thin Cover, Dorsal Aspect Generalized	.	.	.	X	X	X	X
	Fur, Thin Cover, Hindlimb, Left	X	X	.	X	X	X	X
	Fur, Thin Cover, Hindlimb, Right	X	X	.	X	X	X	X
	Fur, Thin Cover, Lumbar	.	.	.	.	.	.	.
	Fur, Thin Cover, Tail	.	.	.	X	X	X	X
	Skin, Discolored, Abdominal, Yellow	.	.	.	.	.	.	.
	Skin, Discolored, Hindlimb, Left, Red	.	.	.	.	.	.	X
	Skin, Discolored, Inguinal, Left, Yellow	.	.	.	.	.	.	.
	Skin, Discolored, Inguinal, Right, Red	.	.	.	.	.	.	.
	Skin, Discolored, Inguinal, Right, Yellow	.	.	.	.	.	.	.
	Skin, Scab, Hindlimb, Right	.	.	.	.	.	.	.
	Skin, Scab, Tail	X	X	.	X	X	X	X
	Reduced Appetite, Severity Not Applicable	.	.	.	.	.	.	.
	Material Present, Foamy, White, Severe	.	.	.	.	.	.	.

X=Present

Appendix 4

Individual Clinical Observations

5550014

1.68 X10E14 vg Group 3 Sex: Male	Observation Type: All Types	Day(s) Relative to Start Date						
		77 DE	78 Unsc	79 Unsc	84 DE	86 Unsc	88 Unsc	91 DE
3201	Tremors, Slight	.	.	.	.	.	.	.
	Muscle Tone, Forelimb, Left, Decreased	.	.	X	.	.	.	.
	Muscle Tone, Hindlimb, Right, Decreased	.	.	.	.	X	.	.
	Hunched Posture	.	.	.	.	.	X	.
	Fur, Erected	.	.	.	.	.	.	.
	Fur, Staining, Abdominal, Yellow	X	.	X	X	X	.	X
	Fur, Thin Cover, Dorsal Aspect Generalized	X	.	X	X	X	.	X
	Fur, Thin Cover, Hindlimb, Left	X	.	X	X	X	.	X
	Fur, Thin Cover, Hindlimb, Right	X	.	X	X	X	.	X
	Fur, Thin Cover, Lumbar	.	.	.	.	.	.	.
	Fur, Thin Cover, Tail	X	.	X	X	X	.	X
	Skin, Discolored, Abdominal, Yellow	.	.	.	.	.	.	.
	Skin, Discolored, Hindlimb, Left, Red	X	.	X	X	X	.	X
	Skin, Discolored, Inguinal, Left, Yellow	.	.	.	.	.	.	.
	Skin, Discolored, Inguinal, Right, Red	.	.	.	.	.	.	.
	Skin, Discolored, Inguinal, Right, Yellow	.	.	.	.	.	.	.
	Skin, Scab, Hindlimb, Right	.	.	.	.	.	.	.
	Skin, Scab, Tail	X	.	X	X	X	.	X
	Reduced Appetite, Severity Not Applicable	.	.	.	.	.	X	.
	Material Present, Foamy, White, Severe	.	X	.	.	.	.	.

X=Present

Appendix 4

Individual Clinical Observations

5550014

1.68<br>X10E14<br>vg<br>Group 3<br>Sex: Male	Observation Type: All Types	Day(s) Relative to Start Date<br>94<br>DE						
3201	Tremors, Slight	.						
	Muscle Tone, Forelimb, Left, Decreased	.						
	Muscle Tone, Hindlimb, Right, Decreased	.						
	Hunched Posture	.						
	Fur, Erected	.						
	Fur, Staining, Abdominal, Yellow	X						
	Fur, Thin Cover, Dorsal Aspect Generalized	.						
	Fur, Thin Cover, Hindlimb, Left	.						
	Fur, Thin Cover, Hindlimb, Right	X						
	Fur, Thin Cover, Lumbar	.						
	Fur, Thin Cover, Tail	X						
	Skin, Discolored, Abdominal, Yellow	.						
	Skin, Discolored, Hindlimb, Left, Red	X						
	Skin, Discolored, Inguinal, Left, Yellow	.						
	Skin, Discolored, Inguinal, Right, Red	.						
	Skin, Discolored, Inguinal, Right, Yellow	.						
	Skin, Scab, Hindlimb, Right	.						
	Skin, Scab, Tail	X						
	Reduced Appetite, Severity Not Applicable	.						
	Material Present, Foamy, White, Severe	.						

X=Present

Appendix 4

Individual Clinical Observations

5550014

0<br>vg<br>Group 1<br>Sex: Female	Observation Type: All Types	Day(s) Relative to Start Date						
		-8<br>DE	-1<br>DE	1<br>DuRx	1<br>Unsc	1<br>Up	7<br>DE	14<br>DE
1701	Tremors, Slight	.	.	X	.	.	.	.
	Fur, Erected	.	.	X	.	.	.	.
	Fur, Thin Cover, Dorsal Aspect Generalized	X	X	.	X	X	X	X
	Fur, Thin Cover, Hindlimb, Left	X	X	.	X	X	X	X
	Fur, Thin Cover, Hindlimb, Right	X	X	.	X	X	X	X
	Fur, Thin Cover, Tail	.	.	.	.	.	.	.
	Skin, Dry, Tail	X	X	.	X	X	X	X
	Skin, Discolored, Hindlimb, Left, Red	.	.	.	.	X	X	X
	Skin, Discolored, Muzzle, Red	.	.	.	.	.	.	X
	Sexual Skin	X	X	.	X	X	X	X
	Swollen, Hindlimb, Left, Moderate, Soft	.	.	.	.	X	.	.
	Discharge, Color, Muzzle, Red	.	.	.	X	X	.	.

X=Present

Appendix 4

Individual Clinical Observations

5550014

0<br>vg<br>Group 1<br>Sex: Female	Observation Type: All Types	Day(s) Relative to Start Date						
		21<br>DE	28<br>DE	35<br>DE	42<br>DE	49<br>DE	56<br>DE	63<br>DE
1701	Tremors, Slight	.	.	.	.	.	.	.
	Fur, Erected	.	.	.	.	.	.	.
	Fur, Thin Cover, Dorsal Aspect Generalized	X	X	X	X	X	X	X
	Fur, Thin Cover, Hindlimb, Left	X	X	X	X	X	X	X
	Fur, Thin Cover, Hindlimb, Right	X	X	X	X	X	X	X
	Fur, Thin Cover, Tail	.	.	.	.	X	X	X
	Skin, Dry, Tail	X	X	X	X	X	X	X
	Skin, Discolored, Hindlimb, Left, Red	.	.	.	.	.	.	.
	Skin, Discolored, Muzzle, Red	X	.	.	.	.	.	.
	Sexual Skin	.	.	.	.	.	X	X
	Swollen, Hindlimb, Left, Moderate, Soft	.	.	.	.	.	.	.
	Discharge, Color, Muzzle, Red	.	.	.	.	.	.	.

X=Present

Appendix 4

Individual Clinical Observations

5550014

0<br>vg<br>Group 1<br>Sex: Female	Observation Type: All Types	Day(s) Relative to Start Date						
		70<br>DE	77<br>DE	79<br>Unsc	84<br>DE	86<br>Unsc	91<br>DE	94<br>DE
1701	Tremors, Slight	.	.	.	.	.	.	.
	Fur, Erected	.	.	.	.	.	.	.
	Fur, Thin Cover, Dorsal Aspect Generalized	X	X	X	X	X	X	.
	Fur, Thin Cover, Hindlimb, Left	X	X	X	X	X	X	.
	Fur, Thin Cover, Hindlimb, Right	X	X	X	X	X	X	X
	Fur, Thin Cover, Tail	X	X	X	X	X	X	X
	Skin, Dry, Tail	X	X	X	X	X	X	X
	Skin, Discolored, Hindlimb, Left, Red	X	X	X	X	X	X	X
	Skin, Discolored, Muzzle, Red	.	.	.	.	.	.	.
	Sexual Skin	X	X	X	X	X	X	.
	Swollen, Hindlimb, Left, Moderate, Soft	.	.	.	.	.	.	.
	Discharge, Color, Muzzle, Red	.	.	.	.	.	.	.

X=Present

Appendix 4

Individual Clinical Observations

5550014

8.4<br>X10E13<br>vg<br>Group 2<br>Sex: Female	Observation Type: All Types	Day(s) Relative to Start Date<br>-10<br>AM_S	-8<br>DE	-1<br>DE	1<br>Up	7<br>DE	14<br>DE	21<br>DE
2701	Muscle Tone, Forelimb, Left, Decreased	.	.	.	.	.	.	.
	Muscle Tone, Hindlimb, Right, Decreased	.	.	.	.	.	.	.
	Fur, Thin Cover, Cranium	.	.	.	.	.	.	.
	Fur, Thin Cover, Dorsal Aspect Generalized	.	X	X	.	X	X	X
	Fur, Thin Cover, Forelimb, Left	.	X	X	.	X	X	X
	Fur, Thin Cover, Forelimb, Right	.	X	X	.	X	X	X
	Fur, Thin Cover, Hindlimb, Left	.	X	X	.	X	X	X
	Fur, Thin Cover, Hindlimb, Right	.	X	X	.	X	X	X
	Fur, Thin Cover, Tail	.	.	.	.	.	.	.
	Skin, Dry, Tail	.	X	X	.	X	X	X
	Skin, Discolored, Cranium, Red	.	.	X	.	.	.	.
	Skin, Discolored, Periorbital, Left, Purple, & Red	.	X	.	.	.	.	.
	Skin, Scab, Abdominal	.	X	X	.	X	X	X
	Skin, Scab, Hindlimb, Right	.	X	X	.	.	.	.
	Skin, Scab, Lumbar	.	X	X	.	X	X	X
	Skin, Scab, Tail	.	.	.	.	X	X	X
	Sexual Skin	.	X	X	.	X	X	X
	Thin	.	.	.	.	.	.	.
	Feces, Abnormal Consistency, Soft, Slight Group Housed	.	.	.	.	.	.	.
	Reduced Appetite, Severity Not Applicable	.	.	.	.	.	.	.
	Material Present, Dry, Red, Moderate Group Housed	.	.	.	.	.	.	.
2702 !	Fur, Thin Cover, Dorsal Aspect Generalized	.	.	.	.	.	.	.
	Fur, Thin Cover, Hindlimb, Left	.	.	.	.	.	.	.
	Fur, Thin Cover, Tail	.	.	.	.	.	.	.

!=Result comment recorded against 1 or more clinical observations. X=Present

Appendix 4

Individual Clinical Observations

5550014

8.4<br>X10E13<br>vg<br>Group 2<br>Sex: Female	Observation Type: All Types	Day(s) Relative to Start Date<br>-10<br>AM_S	 -8<br>DE	 -1<br>DE	 1<br>Up	 7<br>DE	 14<br>DE	 21<br>DE
2702 !	Skin, Lesion, Hindlimb, Left, Moderate	.	.	.	.	.	.	.
	Skin, Dry, Tail	.	.	.	.	.	.	.
	Skin, Discolored, Hindlimb, Left, Red	.	.	.	X	X	X	.
	Skin, Discolored, Inguinal, Left, Red	.	X	X	.	.	X	.
	Skin, Discolored, Muzzle, Red	.	.	.	.	X	X	X
	Skin, Scab, Hindlimb, Left	.	.	.	.	.	.	.
	Skin, Scab, Tail	.	.	.	.	.	.	.
	Sexual Skin	.	X	X	X	X	X	X
	Swollen, Hindlimb, Left, Moderate, Soft	.	.	.	X	.	.	.
	Swollen, Hindlimb, Left, Slight, Soft	.	.	.	.	.	.	.
	Feces, Abnormal Consistency, Soft, Slight Group Housed	.	.	.	.	.	.	.
	Discharge, Color, Site Not Recorded, White	.	.	.	.	.	.	.
	Reduced Appetite, Severity Not Applicable	X	.	.	.	.	.	.
	Material Present, Dry, Red, Moderate Group Housed	.	.	.	.	.	.	.

!=Result comment recorded against 1 or more clinical observations. X=Present

Individual Clinical Observations

5550014

8.4<br>X10E13<br>vg<br>Group 2<br>Sex: Female	Observation Type: All Types	Day(s) Relative to Start Date<br>25<br>AM_S	28<br>DE	28<br>PM_S	30<br>Unsc	35<br>DE	42<br>DE	49<br>DE
2701	Muscle Tone, Forelimb, Left, Decreased	.	.	.	.	.	.	.
	Muscle Tone, Hindlimb, Right, Decreased	.	.	.	.	.	.	.
	Fur, Thin Cover, Cranium	.	.	.	.	.	.	.
	Fur, Thin Cover, Dorsal Aspect Generalized	.	X	.	.	X	X	X
	Fur, Thin Cover, Forelimb, Left	.	X	.	.	X	X	X
	Fur, Thin Cover, Forelimb, Right	.	X	.	.	X	X	X
	Fur, Thin Cover, Hindlimb, Left	.	X	.	.	X	X	X
	Fur, Thin Cover, Hindlimb, Right	.	X	.	.	X	X	X
	Fur, Thin Cover, Tail	.	.	.	.	.	.	X
	Skin, Dry, Tail	.	X	.	.	X	X	X
	Skin, Discolored, Cranium, Red	.	.	.	.	.	.	.
	Skin, Discolored, Periorbital, Left, Purple, & Red	.	.	.	.	.	.	.
	Skin, Scab, Abdominal	.	.	.	.	.	.	.
	Skin, Scab, Hindlimb, Right	.	.	.	.	.	.	.
	Skin, Scab, Lumbar	.	X	.	.	X	.	.
	Skin, Scab, Tail	.	X	.	.	X	.	.
	Sexual Skin	.	X	.	.	X	X	X
	Thin	.	.	.	.	.	.	.
	Feces, Abnormal Consistency, Soft, Slight Group Housed	.	.	X	.	.	.	.
	Reduced Appetite, Severity Not Applicable	X	.	.	.	.	.	.
	Material Present, Dry, Red, Moderate Group Housed	.	.	.	.	.	.	.
2702	Fur, Thin Cover, Dorsal Aspect Generalized	.	.	.	.	.	.	X
	Fur, Thin Cover, Hindlimb, Left	.	.	.	.	.	.	.
	Fur, Thin Cover, Tail	.	.	.	.	.	.	X

X=Present

Appendix 4

Individual Clinical Observations

5550014

8.4<br>X10E13<br>vg<br>Group 2<br>Sex: Female	Observation Type: All Types	Day(s) Relative to Start Date						
		25<br>AM_S	28<br>DE	28<br>PM_S	30<br>Unsc	35<br>DE	42<br>DE	49<br>DE
2702	Skin, Lesion, Hindlimb, Left, Moderate	.	.	X	.	.	.	.
	Skin, Dry, Tail	.	.	.	.	.	.	.
	Skin, Discolored, Hindlimb, Left, Red	.	X	.	X	X	X	X
	Skin, Discolored, Inguinal, Left, Red	.	.	.	.	.	.	.
	Skin, Discolored, Muzzle, Red	.	.	.	.	.	.	.
	Skin, Scab, Hindlimb, Left	.	.	.	.	X	.	.
	Skin, Scab, Tail	.	.	.	.	.	.	.
	Sexual Skin	.	X	.	X	X	X	X
	Swollen, Hindlimb, Left, Moderate, Soft	.	.	.	X	X	.	.
	Swollen, Hindlimb, Left, Slight, Soft	.	X	.	.	.	.	.
	Feces, Abnormal Consistency, Soft, Slight Group Housed	.	.	X	.	.	.	.
	Discharge, Color, Site Not Recorded, White	.	X	.	X	.	.	.
	Reduced Appetite, Severity Not Applicable	X	.	.	.	.	.	.
	Material Present, Dry, Red, Moderate Group Housed	.	.	.	.	.	.	.

X=Present

Appendix 4

Individual Clinical Observations

5550014

8.4<br>X10E13<br>vg<br>Group 2<br>Sex: Female	Observation Type: All Types	Day(s) Relative to Start Date						
		56<br>DE	63<br>DE	67<br>AM_S	70<br>DE	77<br>DE	79<br>Unsc	84<br>DE
2701	Muscle Tone, Forelimb, Left, Decreased	.	.	.	.	.	.	.
	Muscle Tone, Hindlimb, Right, Decreased	.	.	.	.	.	.	.
	Fur, Thin Cover, Cranium	.	X	.	X	X	X	X
	Fur, Thin Cover, Dorsal Aspect Generalized	X	X	.	X	X	X	X
	Fur, Thin Cover, Forelimb, Left	X	X	.	X	X	X	X
	Fur, Thin Cover, Forelimb, Right	X	X	.	X	X	X	X
	Fur, Thin Cover, Hindlimb, Left	X	X	.	X	X	X	X
	Fur, Thin Cover, Hindlimb, Right	X	X	.	X	X	X	X
	Fur, Thin Cover, Tail	X	X	.	X	X	X	X
	Skin, Dry, Tail	X	X	.	X	X	X	X
	Skin, Discolored, Cranium, Red	.	.	.	.	.	.	.
	Skin, Discolored, Periorbital, Left, Purple, & Red	.	.	.	.	.	.	.
	Skin, Scab, Abdominal	.	.	.	.	.	.	.
	Skin, Scab, Hindlimb, Right	.	.	.	.	.	.	.
	Skin, Scab, Lumbar	.	.	.	.	.	.	.
	Skin, Scab, Tail	X	X	.	X	X	X	X
	Sexual Skin	X	X	.	X	X	X	X
	Thin	.	.	.	.	.	X	X
	Feces, Abnormal Consistency, Soft, Slight Group Housed	.	.	.	.	.	.	.
	Reduced Appetite, Severity Not Applicable	.	.	.	.	.	.	.
	Material Present, Dry, Red, Moderate Group Housed	.	.	X	.	.	.	.
2702	Fur, Thin Cover, Dorsal Aspect Generalized	X	X	.	X	X	X	X
	Fur, Thin Cover, Hindlimb, Left	.	.	.	X	X	X	X
	Fur, Thin Cover, Tail	X	X	.	X	X	X	X

X=Present

Appendix 4

Individual Clinical Observations

5550014

8.4<br>X10E13<br>vg<br>Group 2<br>Sex: Female	Observation Type: All Types	Day(s) Relative to Start Date						
		56<br>DE	63<br>DE	67<br>AM_S	70<br>DE	77<br>DE	79<br>Unsc	84<br>DE
2702	Skin, Lesion, Hindlimb, Left, Moderate	.	.	.	.	.	.	.
	Skin, Dry, Tail	X	X	.	X	X	X	X
	Skin, Discolored, Hindlimb, Left, Red	X	X	.	X	X	X	X
	Skin, Discolored, Inguinal, Left, Red	.	.	.	.	.	.	.
	Skin, Discolored, Muzzle, Red	.	.	.	.	.	.	.
	Skin, Scab, Hindlimb, Left	.	.	.	.	.	.	.
	Skin, Scab, Tail	.	.	.	.	.	.	X
	Sexual Skin	X	X	.	X	X	X	X
	Swollen, Hindlimb, Left, Moderate, Soft	.	.	.	.	.	.	.
	Swollen, Hindlimb, Left, Slight, Soft	.	.	.	.	.	.	.
	Feces, Abnormal Consistency, Soft, Slight Group Housed	.	.	.	.	.	.	.
	Discharge, Color, Site Not Recorded, White	.	.	.	.	.	.	.
	Reduced Appetite, Severity Not Applicable	.	.	.	.	.	.	.
	Material Present, Dry, Red, Moderate Group Housed	.	.	X	.	.	.	.

X=Present

Appendix 4

Individual Clinical Observations

5550014

8.4<br>X10E13<br>vg<br>Group 2<br>Sex: Female	Observation Type: All Types	Day(s) Relative to Start Date						
		86<br>Unsc	91<br>DE	94<br>DE				
2701	Muscle Tone, Forelimb, Left, Decreased	X	X	.				
	Muscle Tone, Hindlimb, Right, Decreased	X	X	.				
	Fur, Thin Cover, Cranium	X	X	.				
	Fur, Thin Cover, Dorsal Aspect Generalized	X	X	.				
	Fur, Thin Cover, Forelimb, Left	X	X	.				
	Fur, Thin Cover, Forelimb, Right	X	X	.				
	Fur, Thin Cover, Hindlimb, Left	X	X	.				
	Fur, Thin Cover, Hindlimb, Right	X	X	X				
	Fur, Thin Cover, Tail	X	X	X				
	Skin, Dry, Tail	X	X	X				
	Skin, Discolored, Cranium, Red	.	.	.				
	Skin, Discolored, Periorbital, Left, Purple, & Red	.	.	.				
	Skin, Scab, Abdominal	.	.	.				
	Skin, Scab, Hindlimb, Right	.	.	.				
	Skin, Scab, Lumbar	.	.	.				
	Skin, Scab, Tail	X	X	X				
	Sexual Skin	X	X	.				
	Thin	X	X	X				
	Feces, Abnormal Consistency, Soft, Slight Group Housed	.	.	.				
	Reduced Appetite, Severity Not Applicable	.	.	.				
	Material Present, Dry, Red, Moderate Group Housed	.	.	.				
2702	Fur, Thin Cover, Dorsal Aspect Generalized	X	X	.				
	Fur, Thin Cover, Hindlimb, Left	X	X	.				
	Fur, Thin Cover, Tail	X	X	X				

X=Present

Appendix 4

Individual Clinical Observations

5550014

8.4<br>X10E13<br>vg<br>Group 2<br>Sex: Female	Observation Type: All Types	Day(s) Relative to Start Date						
		86<br>Unsc	91<br>DE	94<br>DE				
2702	Skin, Lesion, Hindlimb, Left, Moderate	.	.	.				
	Skin, Dry, Tail	X	X	X				
	Skin, Discolored, Hindlimb, Left, Red	X	X	X				
	Skin, Discolored, Inguinal, Left, Red	.	.	.				
	Skin, Discolored, Muzzle, Red	.	.	.				
	Skin, Scab, Hindlimb, Left	.	.	.				
	Skin, Scab, Tail	X	X	.				
	Sexual Skin	X	X	.				
	Swollen, Hindlimb, Left, Moderate, Soft	.	.	.				
	Swollen, Hindlimb, Left, Slight, Soft	.	.	.				
	Feces, Abnormal Consistency, Soft, Slight Group Housed	.	.	.				
	Discharge, Color, Site Not Recorded, White	.	.	.				
	Reduced Appetite, Severity Not Applicable	.	.	.				
	Material Present, Dry, Red, Moderate Group Housed	.	.	.				

X=Present

Appendix 4

Individual Clinical Observations

5550014

Comment Information

Group	Sex	Animal	Day	Observation Type	Comment
2: 8.4X10E13 vg	Female	2702	1 (Up)	All Types	SWOLLEN AFTER IM
2: 8.4X10E13 vg	Female	2702	7 (DE)	All Types	SWOLLEN AFTER IM

Appendix 4

Individual Clinical Observations

5550014

1.68 X10E14 vg Group 3 Sex: Female	Observation Type: All Types	Day(s) Relative to Start Date -11 Upr	-8 DE	-1 DE	1 DuRx	1 UDu	1 Up	7 DE
3701 !	Retching	X	.	.	.	.	.	.
	Tremors (Localized), Hindlimb, Right, Slight	.	.	.	.	.	X	.
	Tremors, Moderate	.	.	.	X	.	.	.
	Tremors, Slight	X	.	.	.	X	.	.
	Muscle Tone, Hindlimb, Left, Increased	.	.	.	.	.	X	.
	Muscle Tone, Hindlimb, Right, Increased	.	.	.	.	.	X	.
	Muscle Tone, Site Not Recorded, Increased	.	.	.	.	.	X	.
	Hunched Posture	.	.	.	.	.	.	.
	Fur, Erected	.	.	.	X	.	.	.
	Fur, Thin Cover, Dorsal Aspect Generalized	.	.	.	.	.	.	.
	Fur, Thin Cover, Hindlimb, Left	.	.	.	.	.	.	.
	Fur, Thin Cover, Hindlimb, Right	.	.	.	.	.	.	X
	Fur, Thin Cover, Tail	.	.	.	.	.	.	.
	Skin, Dry, Tail	.	X	X	.	.	.	X
	Skin, Discolored, Hindlimb, Left, Red	.	.	.	.	.	.	.
	Skin, Discolored, Periorbital, Left, Red	.	.	X	.	.	.	X
	Skin, Scab, Hindlimb, Left	.	.	.	.	.	.	.
	Skin, Scab, Tail	.	.	.	.	.	.	X
	Sexual Skin	.	X	X	.	.	.	X
	Swollen, Hindlimb, Left, Slight, Firm	.	.	.	.	.	.	.
	Sneezing	.	.	.	.	.	.	.
	Activity Decreased	.	.	.	.	.	.	.
	Skin, Bruise, Inguinal, Left, Slight	.	.	.	.	.	.	X

!=Result comment recorded against 1 or more clinical observations. X=Present

Appendix 4

Individual Clinical Observations

5550014

1.68 X10E14 vg Group 3 Sex: Female	Observation Type: All Types	Day(s) Relative to Start Date						
		14 DE	21 DE	28 DE	35 DE	42 DE	49 DE	56 DE
3701	Retching	.	.	.	.	.	.	.
	Tremors (Localized), Hindlimb, Right, Slight	.	.	.	.	.	.	.
	Tremors, Moderate	.	.	.	.	.	.	.
	Tremors, Slight	.	.	.	.	.	.	.
	Muscle Tone, Hindlimb, Left, Increased	.	.	.	.	.	.	.
	Muscle Tone, Hindlimb, Right, Increased	.	.	.	.	.	.	.
	Muscle Tone, Site Not Recorded, Increased	.	.	.	.	.	.	.
	Hunched Posture	.	.	.	.	.	.	.
	Fur, Erected	.	.	.	.	.	.	.
	Fur, Thin Cover, Dorsal Aspect Generalized	.	.	.	.	.	.	X
	Fur, Thin Cover, Hindlimb, Left	.	.	.	.	.	X	X
	Fur, Thin Cover, Hindlimb, Right	X	X	X	X	X	X	X
	Fur, Thin Cover, Tail	.	.	.	.	.	X	X
	Skin, Dry, Tail	X	X	X	X	X	X	X
	Skin, Discolored, Hindlimb, Left, Red	.	.	.	.	.	.	.
	Skin, Discolored, Periorbital, Left, Red	X	.	.	.	.	.	.
	Skin, Scab, Hindlimb, Left	.	.	.	.	.	.	.
	Skin, Scab, Tail	X	X	X	X	.	.	.
	Sexual Skin	X	X	X	X	X	X	X
	Swollen, Hindlimb, Left, Slight, Firm	.	.	.	.	.	.	.
	Sneezing	.	.	.	.	.	.	.
	Activity Decreased	.	.	.	.	.	.	.
	Skin, Bruise, Inguinal, Left, Slight	X	.	.	.	.	.	.

X=Present

Appendix 4

Individual Clinical Observations

5550014

1.68 X10E14 vg Group 3 Sex: Female	Observation Type: All Types	Day(s) Relative to Start Date						
		57 AM_S	61 AM_S	63 DE	70 DE	77 DE	79 Unsc	84 DE
3701	Retching	.	.	.	.	.	.	.
	Tremors (Localized), Hindlimb, Right, Slight	X	X	.	.	.	.	.
	Tremors, Moderate	.	.	.	.	.	.	.
	Tremors, Slight	.	.	.	.	.	.	.
	Muscle Tone, Hindlimb, Left, Increased	.	.	.	.	.	.	.
	Muscle Tone, Hindlimb, Right, Increased	.	.	.	.	.	.	.
	Muscle Tone, Site Not Recorded, Increased	.	.	.	.	.	.	.
	Hunched Posture	X	X	.	.	.	.	.
	Fur, Erected	.	.	.	.	.	.	.
	Fur, Thin Cover, Dorsal Aspect Generalized	.	.	X	X	X	X	X
	Fur, Thin Cover, Hindlimb, Left	.	.	X	X	X	X	X
	Fur, Thin Cover, Hindlimb, Right	.	.	X	X	X	X	X
	Fur, Thin Cover, Tail	.	.	X	X	X	X	X
	Skin, Dry, Tail	.	.	X	X	X	X	X
	Skin, Discolored, Hindlimb, Left, Red	.	.	.	.	.	.	.
	Skin, Discolored, Periorbital, Left, Red	.	.	.	.	.	.	.
	Skin, Scab, Hindlimb, Left	.	.	.	.	.	.	X
	Skin, Scab, Tail	.	.	.	.	.	.	X
	Sexual Skin	.	.	X	X	X	X	X
	Swollen, Hindlimb, Left, Slight, Firm	.	.	.	.	.	.	.
	Sneezing	.	X	.	.	.	.	.
	Activity Decreased	X	X	.	.	.	.	.
	Skin, Bruise, Inguinal, Left, Slight	.	.	.	.	.	.	.

X=Present

Appendix 4

Individual Clinical Observations

5550014

1.68<br>X10E14<br>vg<br>Group 3<br>Sex: Female	Observation Type: All Types	Day(s) Relative to Start Date<br>86<br>Unsc	 89<br>Unsc	 91<br>DE	 94<br>DE			
3701	Retching	.	.	.	.			
	Tremors (Localized), Hindlimb, Right, Slight	.	.	.	.			
	Tremors, Moderate	.	.	.	.			
	Tremors, Slight	.	.	.	.			
	Muscle Tone, Hindlimb, Left, Increased	.	.	.	.			
	Muscle Tone, Hindlimb, Right, Increased	.	.	.	.			
	Muscle Tone, Site Not Recorded, Increased	.	.	.	.			
	Hunched Posture	.	.	.	.			
	Fur, Erected	.	.	.	.			
	Fur, Thin Cover, Dorsal Aspect Generalized	X	X	X	.			
	Fur, Thin Cover, Hindlimb, Left	X	X	X	.			
	Fur, Thin Cover, Hindlimb, Right	X	X	X	X			
	Fur, Thin Cover, Tail	X	X	X	X			
	Skin, Dry, Tail	X	X	X	X			
	Skin, Discolored, Hindlimb, Left, Red	.	X	X	X			
	Skin, Discolored, Periorbital, Left, Red	.	.	.	.			
	Skin, Scab, Hindlimb, Left	X	X	X	.			
	Skin, Scab, Tail	X	X	X	X			
	Sexual Skin	X	X	X	X			
	Swollen, Hindlimb, Left, Slight, Firm	.	X	.	.			
	Sneezing	.	.	.	.			
	Activity Decreased	.	.	.	.			
	Skin, Bruise, Inguinal, Left, Slight	.	.	.	.			

X=Present

Appendix 4

Individual Clinical Observations

5550014

Comment Information

Group	Sex	Animal	Day	Observation Type	Comment
3: 1.68X10E14 vg	Female	3701	1 (Up)	All Types	MUSCLE TONE INCREASED TAIL

Appendix 5

Individual Body Weights

5550014

Sex: Male Bodyweight (kg)

0<br>vg<br>Group 1	Day(s) Relative to Start Date<br>-13	-8	-1	7	14	21	28
1201	3.0	3.0	3.2	3.0	3.1	3.0	3.0

Appendix 5

Individual Body Weights

5550014
Sex: Male Bodyweight (kg)

0<br>vg<br>Group 1	Day(s) Relative to Start Date						
	35	42	49	56	63	70	77
1201	3.0	3.1	3.0	3.0	3.1	3.1	3.1

Appendix 5

Individual Body Weights

5550014

Sex: Male Bodyweight (kg)

0<br>vg<br>Group 1	Day(s) Relative to Start Date		
	84	91	94
1201	3.1	3.1	3.1

Appendix 5

Individual Body Weights

5550014
Sex: Male Bodyweight (kg)

1.68 X10E14 vg Group 3	Day(s) Relative to Start Date						
	-13	-8	-1	7	14	21	28
3201	2.9	2.9	3.1	3.1	3.1	2.9	2.9

Appendix 5

Individual Body Weights

5550014
Sex: Male Bodyweight (kg)

1.68 X10E14 vg Group 3	Day(s) Relative to Start Date						
	35	42	49	56	63	70	77
3201	2.9	3.0	3.0	3.0	3.0	3.1	3.0

Appendix 5

Individual Body Weights

5550014
Sex: Male Bodyweight (kg)

1.68 X10E14 vg Group 3	Day(s) Relative to Start Date		
	84	91	94
3201	3.1	3.0	2.9

Appendix 5

Individual Body Weights

5550014
Sex: Female Bodyweight (kg)

0<br>vg<br>Group 1	Day(s) Relative to Start Date<br>-13	-8	-1	7	14	21	28
1701	2.9	2.7 [a]	3.0 [a]	2.7	2.9	2.7	2.7

[a] [RC:Value Confirmed]

Appendix 5

Individual Body Weights

5550014
Sex: Female Bodyweight (kg)

0<br>vg<br>Group 1	Day(s) Relative to Start Date<br>35	42	49	56	63	70	77
1701	2.7	2.7	2.7	2.7	2.8	2.8	2.7

Appendix 5

Individual Body Weights

5550014
Sex: Female Bodyweight (kg)

0<br>vg<br>Group 1	Day(s) Relative to Start Date<br>84	91	94
1701	2.7	2.7	2.6

Appendix 5

Individual Body Weights

5550014
Sex: Female Bodyweight (kg)

8.4 X10E13 vg Group 2	Day(s) Relative to Start Date						
	-13	-8	-1	7	14	21	28
2701	2.7	2.6	2.6	2.4	2.6	2.4 [a]	2.5
2702	3.5	3.4	3.4	3.4	3.5	3.3	3.2

[a] [RC:2.4 kg weight verified]

Appendix 5

Individual Body Weights

5550014
Sex: Female Bodyweight (kg)

8.4 X10E13 vg Group 2	Day(s) Relative to Start Date						
	35	42	49	56	63	70	77
2701	2.5	2.6	2.5	2.4	2.6	2.8	2.6 [a]
2702	3.2	3.3	3.4	3.3	3.5	3.6	3.5

[a] [RC:2.6 kg weight verified]

Appendix 5

Individual Body Weights

5550014
Sex: Female Bodyweight (kg)

8.4 X10E13 vg Group 2	Day(s) Relative to Start Date		
	84	91	94
2701	2.5	2.5	2.4
2702	3.5	3.4	3.3

Appendix 5

Individual Body Weights

5550014
Sex: Female Bodyweight (kg)

1.68 X10E14 vg Group 3	Day(s) Relative to Start Date						
	-13	-8	-1	7	14	21	28
3701	2.8	2.8	2.9	2.7	2.9	2.6 [a]	2.6

[a] [RC:2.6 kg weight verified]

Appendix 5

Individual Body Weights

5550014
Sex: Female Bodyweight (kg)

1.68 X10E14 vg Group 3	Day(s) Relative to Start Date						
	35	42	49	56	63	70	77
3701	2.6	2.8	2.7	2.6	2.8	2.9	2.9

Appendix 5

Individual Body Weights

5550014

Sex: Female Bodyweight (kg)

1.68 X10E14 vg Group 3	Day(s) Relative to Start Date		
	84	91	94
3701	2.9	2.8	2.8

Appendix 6

Individual Body Weight Gains (kg)

5550014
Sex: Male Bodyweight Gain (Interval)

0<br>vg<br>Group 1	Day(s) Relative to Start Date						
	-13 → -8	-8 → -1	-1 → 7	7 → 14	14 → 21	21 → 28	28 → 35
1201	0.0	0.2	-0.2	0.1	-0.1	0.0	0.0

Appendix 6

Individual Body Weight Gains (kg)

5550014
Sex: Male Bodyweight Gain (Interval)

0<br>vg<br>Group 1	Day(s) Relative to Start Date 35 → 42	42 → 49	49 → 56	56 → 63	63 → 70	70 → 77	77 → 84
1201	0.1	-0.1	0.0	0.1	0.0	0.0	0.0

Appendix 6

Individual Body Weight Gains (kg)

5550014

Sex: Male Bodyweight Gain (Interval)

0<br>vg<br>Group 1	Day(s) Relative to Start Date		
	84 → 91	-1 → 91	91 → 94
1201	0.0	-0.1	0.0

Appendix 6

Individual Body Weight Gains (kg)

5550014
Sex: Male Bodyweight Gain (Interval)

1.68 X10E14 vg Group 3	Day(s) Relative to Start Date						
	-13 → -8	-8 → -1	-1 → 7	7 → 14	14 → 21	21 → 28	28 → 35
3201	0.0	0.2	0.0	0.0	-0.2	0.0	0.0

Appendix 6

Individual Body Weight Gains (kg)

5550014
Sex: Male Bodyweight Gain (Interval)

1.68 X10E14 vg Group 3	Day(s) Relative to Start Date						
	35 → 42	42 → 49	49 → 56	56 → 63	63 → 70	70 → 77	77 → 84
3201	0.1	0.0	0.0	0.0	0.1	-0.1	0.1

Appendix 6

Individual Body Weight Gains (kg)

5550014

Sex: Male Bodyweight Gain (Interval)

1.68 X10E14 vg Group 3	Day(s) Relative to Start Date		
	84 → 91	-1 → 91	91 → 94
3201	-0.1	-0.1	-0.1

Appendix 6

Individual Body Weight Gains (kg)

5550014
Sex: Female Bodyweight Gain (Interval)

0<br>vg<br>Group 1	Day(s) Relative to Start Date						
	-13 → -8	-8 → -1	-1 → 7	7 → 14	14 → 21	21 → 28	28 → 35
1701	-0.2	0.3	-0.3	0.2	-0.2	0.0	0.0

Appendix 6

Individual Body Weight Gains (kg)

5550014

Sex: Female Bodyweight Gain (Interval)

0<br>vg<br><br>Group 1	Day(s) Relative<br>to Start Date						
	35 → 42	42 → 49	49 → 56	56 → 63	63 → 70	70 → 77	77 → 84
1701	0.0	0.0	0.0	0.1	0.0	-0.1	0.0

Appendix 6

Individual Body Weight Gains (kg)

5550014

Sex: Female Bodyweight Gain (Interval)

0<br>vg<br>Group 1	Day(s) Relative to Start Date<br>84 → 91	-1 → 91	91 → 94
1701	0.0	-0.3	-0.1

Appendix 6

Individual Body Weight Gains (kg)

5550014

Sex: Female Bodyweight Gain (Interval)

8.4 X10E13 vg Group 2	Day(s) Relative to Start Date						
	-13 → -8	-8 → -1	-1 → 7	7 → 14	14 → 21	21 → 28	28 → 35
2701	-0.1	0.0	-0.2	0.2	-0.2	0.1	0.0
2702	-0.1	0.0	0.0	0.1	-0.2	-0.1	0.0

Appendix 6

Individual Body Weight Gains (kg)

5550014

Sex: Female Bodyweight Gain (Interval)

8.4 X10E13 vg Group 2	Day(s) Relative to Start Date						
	35 → 42	42 → 49	49 → 56	56 → 63	63 → 70	70 → 77	77 → 84
2701	0.1	-0.1	-0.1	0.2	0.2	-0.2	-0.1
2702	0.1	0.1	-0.1	0.2	0.1	-0.1	0.0

Appendix 6

Individual Body Weight Gains (kg)

5550014

Sex: Female Bodyweight Gain (Interval)

8.4 X10E13 vg Group 2	Day(s) Relative to Start Date		
	84 → 91	-1 → 91	91 → 94
2701	0.0	-0.1	-0.1
2702	-0.1	0.0	-0.1

Appendix 6

Individual Body Weight Gains (kg)

5550014
Sex: Female Bodyweight Gain (Interval)

1.68 X10E14 vg Group 3	Day(s) Relative to Start Date						
	-13 → -8	-8 → -1	-1 → 7	7 → 14	14 → 21	21 → 28	28 → 35
3701	0.0	0.1	-0.2	0.2	-0.3	0.0	0.0

Appendix 6

Individual Body Weight Gains (kg)

5550014

Sex: Female   Bodyweight Gain (Interval)

1.68 X10E14 vg Group 3	Day(s) Relative to Start Date						
	35 → 42	42 → 49	49 → 56	56 → 63	63 → 70	70 → 77	77 → 84
3701	0.2	-0.1	-0.1	0.2	0.1	0.0	0.0

Appendix 6

Individual Body Weight Gains (kg)

5550014

Sex: Female Bodyweight Gain (Interval)

1.68 X10E14 vg Group 3	Day(s) Relative to Start Date		
	84 → 91	-1 → 91	91 → 94
3701	-0.1	-0.1	0.0

Appendix 7

Individual Neurological Examinations

5550014

Sex: Male

0<br>vg<br>Group 1	Day(s) Relative to Start Date	Neuro (NHP)<br>Gen Att/Mot Function	Proprio Pos L Hindlimb	Proprio Pos R Hindlimb	Placing Reac Visual LH	Placing Reac Visual RH	Placing Reac Tactile LH
1201	-7	Normal	Normal	Normal	Normal	Normal	Normal
	1 (4-6H)	Normal	Normal	Normal	Normal	Normal	Normal
	2	Normal	Normal	Normal	Normal	Normal	Normal
	7 (DE)	Normal	Normal	Normal	Normal	Normal	Normal
	28 (DE)	Normal	Normal	Normal	Normal	Normal	Normal
	94	Normal	Normal	Normal	Normal	Normal	Normal

Appendix 7

Individual Neurological Examinations

5550014

Sex: Male

0<br>vg<br>Group 1	Day(s) Relative to Start Date	Neuro (NHP)					
		Placing Reac Tactile RH	Head Movement	Pupil Symmetry	Head Symmetry	Eye Position Left	Eye Position Right
1201	-7	Normal	Normal	Normal	Normal	Normal	Normal
	1 (4-6H)	Normal	Normal	Normal	Normal	Normal	Normal
	2	Normal	Normal	Normal	Normal	Normal	Normal
	7 (DE)	Normal	Normal	Normal	Normal	Normal	Normal
	28 (DE)	Normal	Normal	Normal	Normal	Normal	Normal
	94	Normal	Normal	Normal	Normal	Normal	Normal

Appendix 7

Individual Neurological Examinations

5550014
Sex: Male

0<br>vg<br>Group 1	Day(s) Relative to Start Date	Neuro (NHP)					
		Palpebral Reflex Left	Palpebral Reflex Right	Menace Reflex Left	Menace Reflex Right	Head Muscle Tone	Pupillary Light Ref L
1201	-7	Normal	Normal	Normal	Normal	Normal	Normal
	1 (4-6H)	Normal	Normal	Normal	Normal	Normal	Normal
	2	Normal	Normal	Normal	Normal	Normal	Normal
	7 (DE)	Normal	Normal	Normal	Normal	Normal	Normal
	28 (DE)	Normal	Normal	Normal	Normal	Normal	Normal
	94	Normal	Normal	Normal	Normal	Normal	Normal

Appendix 7

Individual Neurological Examinations

5550014

Sex: Male

0<br>vg<br>Group 1	Day(s) Relative to Start Date	Neuro (NHP)<br>Pupillary Light Ref R	Vestibular Nystagmus	Muscle Tone L Hindlimb	Muscle Tone R Hindlimb	Flexor Reflex LH	Flexor Reflex RH
1201	-7	Normal	Normal	Normal	Normal	Normal	Normal
	1 (4-6H)	Normal	Normal	Normal	Normal	Normal	Normal
	2	Normal	Normal	Normal	Normal	Normal	Normal
	7 (DE)	Normal	Normal	Normal	Normal	Normal	Normal
	28 (DE)	Normal	Normal	Normal	Normal	Normal	Normal
	94	Normal	Normal	Normal	Normal	Normal	Normal

Appendix 7

Individual Neurological Examinations

5550014
Sex: Male

0 vg Group 1	Day(s) Relative to Start Date	Neuro (NHP) Flexor Reflex LF	Flexor Reflex RF	Perineal Reflex	Jaw Tone
1201	-7	Normal	Normal	Normal	Normal
	1 (4-6H)	Normal	Normal	Normal	Normal
	2	Normal	Normal	Normal	Normal
	7 (DE)	Normal	Normal	Normal	Normal
	28 (DE)	Normal	Normal	Normal	Normal
	94	Normal	Normal	Normal	Normal

Appendix 7

Individual Neurological Examinations

5550014

Sex: Male

1.68 X10E14 vg Group 3	Day(s) Relative to Start Date	Neuro (NHP)					
		Gen Att/Mot Function	Proprio Pos L Hindlimb	Proprio Pos R Hindlimb	Placing Reac Visual LH	Placing Reac Visual RH	Placing Reac Tactile LH
3201	-7	Normal	Normal	Normal	Normal	Normal	Normal
	1 (4-6H)	Normal	Normal	Normal	Normal	Normal	Normal
	2	Normal	Normal	Normal	Normal	Normal	Normal
	7 (DE)	Normal	Normal	Normal	Normal	Normal	Normal
	28 (DE)	Normal	Normal	Normal	Normal	Normal	Normal
	94	Abnormal [a]	Decreased	Normal	Normal	Normal	Normal

[a] [RC:tremors slight hl, hr]

Appendix 7

Individual Neurological Examinations

5550014
Sex: Male

1.68 X10E14 vg Group 3	Day(s) Relative to Start Date	Neuro (NHP)					
		Placing Reac Tactile RH	Head Movement	Pupil Symmetry	Head Symmetry	Eye Position Left	Eye Position Right
3201	-7	Normal	Normal	Normal	Normal	Normal	Normal
	1 (4-6H)	Normal	Normal	Normal	Normal	Normal	Normal
	2	Normal	Normal	Normal	Normal	Normal	Normal
	7 (DE)	Normal	Normal	Normal	Normal	Normal	Normal
	28 (DE)	Normal	Normal	Normal	Normal	Normal	Normal
	94	Normal	Normal	Normal	Normal	Normal	Normal

Appendix 7

Individual Neurological Examinations

5550014
Sex: Male

1.68 X10E14 vg Group 3	Day(s) Relative to Start Date	Neuro (NHP) Palpebral Reflex Left	Palpebral Reflex Right	Menace Reflex Left	Menace Reflex Right	Head Muscle Tone	Pupillary Light Ref L
3201	-7	Normal	Normal	Normal	Normal	Normal	Normal
	1 (4-6H)	Normal	Normal	Normal	Normal	Normal	Normal
	2	Normal	Normal	Normal	Normal	Normal	Normal
	7 (DE)	Normal	Normal	Normal	Normal	Normal	Normal
	28 (DE)	Normal	Normal	Normal	Normal	Normal	Normal
	94	Normal	Normal	Normal	Normal	Normal	Normal

Appendix 7

Individual Neurological Examinations

5550014

Sex: Male

1.68 X10E14 vg Group 3	Day(s) Relative to Start Date	Neuro (NHP) Pupillary Light Ref R	Vestibular Nystagmus	Muscle Tone L Hindlimb	Muscle Tone R Hindlimb	Flexor Reflex LH	Flexor Reflex RH
3201	-7	Normal	Normal	Normal	Normal	Normal	Normal
	1 (4-6H)	Normal	Normal	Normal	Normal	Normal	Normal
	2	Normal	Normal	Normal	Normal	Normal	Normal
	7 (DE)	Normal	Normal	Normal	Normal	Normal	Normal
	28 (DE)	Normal	Normal	Normal	Normal	Normal	Normal
	94	Normal	Normal	Normal	Normal	Normal	Normal

Appendix 7

Individual Neurological Examinations

5550014

Sex: Male

1.68 X10E14 vg Group 3	Day(s) Relative to Start Date	Neuro (NHP)			
		Flexor Reflex LF	Flexor Reflex RF	Perineal Reflex	Jaw Tone
3201	-7	Normal	Normal	Normal	Normal
	1 (4-6H)	Normal	Normal	Normal	Normal
	2	Normal	Normal	Normal	Normal
	7 (DE)	Normal	Normal	Normal	Normal
	28 (DE)	Normal	Normal	Normal	Normal
	94	Normal	Normal	Normal	Normal

Appendix 7

Individual Neurological Examinations

5550014
Sex: Female

0<br>vg<br>Group 1	Day(s) Relative to Start Date	Neuro (NHP)<br>Gen Att/Mot Function	Proprio Pos L Hindlimb	Proprio Pos R Hindlimb	Placing Reac Visual LH	Placing Reac Visual RH	Placing Reac Tactile LH
1701	-7	Normal	Normal	Normal	Normal	Normal	Normal
	1 (4-6H)	Normal	Normal	Normal	Normal	Normal	Normal
	2	Normal	Normal	Normal	Normal	Normal	Normal
	7 (DE)	Normal	Normal	Normal	Normal	Normal	Normal
	28 (DE)	Normal	Normal	Normal	Normal	Normal	Normal
	94	Normal	Normal	Normal	Normal	Normal	Normal

Appendix 7

Individual Neurological Examinations

5550014
Sex: Female

0<br>vg<br>Group 1	Day(s) Relative to Start Date	Neuro (NHP)<br>Placing Reac Tactile RH	Head Movement	Pupil Symmetry	Head Symmetry	Eye Position Left	Eye Position Right
1701	-7	Normal	Normal	Normal	Normal	Normal	Normal
	1 (4-6H)	Normal	Normal	Normal	Normal	Normal	Normal
	2	Normal	Normal	Normal	Normal	Normal	Normal
	7 (DE)	Normal	Normal	Normal	Normal	Normal	Normal
	28 (DE)	Normal	Normal	Normal	Normal	Normal	Normal
	94	Normal	Normal	Normal	Normal	Normal	Normal

Appendix 7

Individual Neurological Examinations

5550014

Sex: Female

0<br>vg<br>Group 1	Day(s) Relative to Start Date	Neuro (NHP)<br>Palpebral Reflex Left	Palpebral Reflex Right	Menace Reflex Left	Menace Reflex Right	Head Muscle Tone	Pupillary Light Ref L
1701	-7	Normal	Normal	Normal	Normal	Normal	Normal
	1 (4-6H)	Normal	Normal	Normal	Normal	Normal	Normal
	2	Normal	Normal	Normal	Normal	Normal	Normal
	7 (DE)	Normal	Normal	Normal	Normal	Normal	Normal
	28 (DE)	Normal	Normal	Normal	Normal	Normal	Normal
	94	Normal	Normal	Normal	Normal	Normal	Normal

Appendix 7

Individual Neurological Examinations

5550014

Sex: Female

0<br>vg<br>Group 1	Day(s) Relative to Start Date	Neuro (NHP)					
		Pupillary Light Ref R	Vestibular Nystagmus	Muscle Tone L Hindlimb	Muscle Tone R Hindlimb	Flexor Reflex LH	Flexor Reflex RH
1701	-7	Normal	Normal	Normal	Normal	Normal	Normal
	1 (4-6H)	Normal	Normal	Normal	Normal	Normal	Normal
	2	Normal	Normal	Normal	Normal	Normal	Normal
	7 (DE)	Normal	Normal	Normal	Normal	Normal	Normal
	28 (DE)	Normal	Normal	Normal	Normal	Normal	Normal
	94	Normal	Normal	Normal	Normal	Normal	Normal

Appendix 7

Individual Neurological Examinations

5550014

Sex: Female

0<br>vg<br>Group 1	Day(s) Relative to Start Date	Neuro (NHP)<br>Flexor Reflex LF	Flexor Reflex RF	Perineal Reflex	Jaw Tone
1701	-7	Normal	Normal	Normal	Normal
	1 (4-6H)	Normal	Normal	Normal	Normal
	2	Normal	Normal	Normal	Normal
	7 (DE)	Normal	Normal	Normal	Normal
	28 (DE)	Normal	Normal	Normal	Normal
	94	Normal	Normal	Normal	Normal

Appendix 7

Individual Neurological Examinations

5550014

Sex: Female

8.4<br>X10E13<br>vg<br>Group 2	Day(s) Relative to Start Date	Neuro (NHP)<br>Gen Att/Mot Function	Proprio Pos L Hindlimb	Proprio Pos R Hindlimb	Placing Reac Visual LH	Placing Reac Visual RH	Placing Reac Tactile LH
2701	-7	Normal	Normal	Normal	Normal	Normal	Normal
	1 (4-6H)	Normal	Normal	Normal	Normal	Normal	Normal
	2	Normal	Normal	Normal	Normal	Normal	Normal
	7 (DE)	Normal	Normal	Normal	Normal	Normal	Normal
	28 (DE)	Normal	Normal	Normal	Normal	Normal	Normal
	94	Normal	Normal	Normal	Normal	Normal	Normal
2702	-7	Normal	Normal	Normal	Normal	Normal	Normal
	1 (4-6H)	Normal	Normal	Normal	Normal	Normal	Normal
	2	Normal	Normal	Normal	Normal	Normal	Normal
	7 (DE)	Normal	Normal	Normal	Normal	Normal	Normal
	28 (DE)	Normal	Normal	Normal	Normal	Normal	Normal
	94	Normal	Normal	Normal	Normal	Normal	Normal

Appendix 7

Individual Neurological Examinations

5550014
Sex: Female

8.4 X10E13 vg Group 2	Day(s) Relative to Start Date	Neuro (NHP)					
		Placing Reac Tactile RH	Head Movement	Pupil Symmetry	Head Symmetry	Eye Position Left	Eye Position Right
2701	-7	Normal	Normal	Normal	Normal	Normal	Normal
	1 (4-6H)	Normal	Normal	Normal	Normal	Normal	Normal
	2	Normal	Normal	Normal	Normal	Normal	Normal
	7 (DE)	Normal	Normal	Normal	Normal	Normal	Normal
	28 (DE)	Normal	Normal	Normal	Normal	Normal	Normal
	94	Normal	Normal	Normal	Normal	Normal	Normal
2702	-7	Normal	Normal	Normal	Normal	Normal	Normal
	1 (4-6H)	Normal	Normal	Normal	Normal	Normal	Normal
	2	Normal	Normal	Normal	Normal	Normal	Normal
	7 (DE)	Normal	Normal	Normal	Normal	Normal	Normal
	28 (DE)	Normal	Normal	Normal	Normal	Normal	Normal
	94	Normal	Normal	Normal	Normal	Normal	Normal

Appendix 7

Individual Neurological Examinations

5550014
Sex: Female

8.4 X10E13 vg Group 2	Day(s) Relative to Start Date	Neuro (NHP) Palpebral Reflex Left	Palpebral Reflex Right	Menace Reflex Left	Menace Reflex Right	Head Muscle Tone	Pupillary Light Ref L
2701	-7	Normal	Normal	Normal	Normal	Normal	Normal
	1 (4-6H)	Normal	Normal	Normal	Normal	Normal	Normal
	2	Normal	Normal	Normal	Normal	Normal	Normal
	7 (DE)	Normal	Normal	Normal	Normal	Normal	Normal
	28 (DE)	Normal	Normal	Normal	Normal	Normal	Normal
	94	Normal	Normal	Normal	Normal	Normal	Normal
2702	-7	Normal	Normal	Normal	Normal	Normal	Normal
	1 (4-6H)	Normal	Normal	Normal	Normal	Normal	Normal
	2	Normal	Normal	Normal	Normal	Normal	Normal
	7 (DE)	Normal	Normal	Normal	Normal	Normal	Normal
	28 (DE)	Normal	Normal	Normal	Normal	Normal	Normal
	94	Normal	Normal	Normal	Normal	Normal	Normal

Appendix 7

Individual Neurological Examinations

5550014

Sex: Female

8.4 X10E13 vg Group 2	Day(s) Relative to Start Date	Neuro (NHP)					
		Pupillary Light Ref R	Vestibular Nystagmus	Muscle Tone L Hindlimb	Muscle Tone R Hindlimb	Flexor Reflex LH	Flexor Reflex RH
2701	-7	Normal	Normal	Normal	Normal	Normal	Normal
	1 (4-6H)	Normal	Normal	Normal	Normal	Normal	Normal
	2	Normal	Normal	Normal	Normal	Normal	Normal
	7 (DE)	Normal	Normal	Normal	Normal	Normal	Normal
	28 (DE)	Normal	Normal	Normal	Normal	Normal	Normal
	94	Normal	Normal	Normal	Normal	Normal	Normal
2702	-7	Normal	Normal	Normal	Normal	Normal	Normal
	1 (4-6H)	Normal	Normal	Normal	Normal	Normal	Normal
	2	Normal	Normal	Normal	Normal	Normal	Normal
	7 (DE)	Normal	Normal	Normal	Normal	Normal	Normal
	28 (DE)	Normal	Normal	Normal	Normal	Normal	Normal
	94	Normal	Normal	Normal	Normal	Normal	Normal

Appendix 7

Individual Neurological Examinations

5550014
Sex: Female

8.4 X10E13 vg Group 2	Day(s) Relative to Start Date	Neuro (NHP) Flexor Reflex LF	Flexor Reflex RF	Perineal Reflex	Jaw Tone
2701	-7	Normal	Normal	Normal	Normal
	1 (4-6H)	Normal	Normal	Normal	Normal
	2	Normal	Normal	Normal	Normal
	7 (DE)	Normal	Normal	Normal	Normal
	28 (DE)	Normal	Normal	Normal	Normal
	94	Normal	Normal	Normal	Normal
2702	-7	Normal	Normal	Normal	Normal
	1 (4-6H)	Normal	Normal	Normal	Normal
	2	Normal	Normal	Normal	Normal
	7 (DE)	Normal	Normal	Normal	Normal
	28 (DE)	Normal	Normal	Normal	Normal
	94	Normal	Normal	Normal	Normal

Appendix 7

Individual Neurological Examinations

5550014

Sex: Female

1.68 X10E14 vg Group 3	Day(s) Relative to Start Date	Neuro (NHP) Gen Att/Mot Function	Proprio Pos L Hindlimb	Proprio Pos R Hindlimb	Placing Reac Visual LH	Placing Reac Visual RH	Placing Reac Tactile LH
3701	-7	Normal	Normal	Normal	Normal	Normal	Normal
	1 (4-6H)	Normal	Normal	Normal	Normal	Normal	Normal
	2	Normal	Normal	Normal	Normal	Normal	Normal
	7 (DE)	Normal	Normal	Normal	Normal	Normal	Normal
	28 (DE)	Normal	Normal	Normal	Normal	Normal	Normal
	94	Normal	Normal	Normal	Normal	Normal	Normal

Appendix 7

Individual Neurological Examinations

5550014

Sex: Female

1.68 X10E14 vg Group 3	Day(s) Relative to Start Date	Neuro (NHP) Placing Reac Tactile RH	Head Movement	Pupil Symmetry	Head Symmetry	Eye Position Left	Eye Position Right
3701	-7	Normal	Normal	Normal	Normal	Normal	Normal
	1 (4-6H)	Normal	Normal	Normal	Normal	Normal	Normal
	2	Normal	Normal	Normal	Normal	Normal	Normal
	7 (DE)	Normal	Normal	Normal	Normal	Normal	Normal
	28 (DE)	Normal	Normal	Normal	Normal	Normal	Normal
	94	Normal	Normal	Normal	Normal	Normal	Normal

Appendix 7

Individual Neurological Examinations

5550014
Sex: Female

1.68 X10E14 vg Group 3	Day(s) Relative to Start Date	Neuro (NHP)					
		Palpebral Reflex Left	Palpebral Reflex Right	Menace Reflex Left	Menace Reflex Right	Head Muscle Tone	Pupillary Light Ref L
3701	-7	Normal	Normal	Normal	Normal	Normal	Normal
	1 (4-6H)	Normal	Normal	Normal	Normal	Normal	Normal
	2	Normal	Normal	Normal	Normal	Normal	Normal
	7 (DE)	Normal	Normal	Normal	Normal	Normal	Normal
	28 (DE)	Normal	Normal	Normal	Normal	Normal	Normal
	94	Normal	Normal	Normal	Normal	Normal	Normal

Appendix 7

Individual Neurological Examinations

5550014

Sex: Female

1.68 X10E14 vg Group 3	Day(s) Relative to Start Date	Neuro (NHP)					
		Pupillary Light Ref R	Vestibular Nystagmus	Muscle Tone L Hindlimb	Muscle Tone R Hindlimb	Flexor Reflex LH	Flexor Reflex RH
3701	-7	Normal	Normal	Normal	Normal	Normal	Normal
	1 (4-6H)	Normal	Normal	Normal	Normal	Normal	Normal
	2	Normal	Normal	Normal	Normal	Normal	Normal
	7 (DE)	Normal	Normal	Normal	Normal	Normal	Normal
	28 (DE)	Normal	Normal	Normal	Normal	Normal	Normal
	94	Normal	Normal	Normal	Normal	Normal	Normal

Appendix 7

Individual Neurological Examinations

5550014
Sex: Female

1.68 X10E14 vg Group 3	Day(s) Relative to Start Date	Neuro (NHP)			
		Flexor Reflex LF	Flexor Reflex RF	Perineal Reflex	Jaw Tone
3701	-7	Normal	Normal	Normal	Normal
	1 (4-6H)	Normal	Normal	Normal	Normal
	2	Normal	Normal	Normal	Normal
	7 (DE)	Normal	Normal	Normal	Normal
	28 (DE)	Normal	Normal	Normal	Normal
	94	Normal	Normal	Normal	Normal

Appendix 8

DocuSign Envelope ID: 6741D4CA-D260-42D8-B645-F9F63978E090

charles river

Title: NEUROLOGICAL ASSESSMENTS - NON-RODENTS	SOP Number: TB08-03-01	Effective Date: 28-Apr-2021
	Version Number: 9.0	Site: CR-SEN/SHB

Management Approval:
DocuSigned by:
Signer Name: Julie Douville
Signing Reason: I approve this document
Signing Time: 16-Apr-2021 | 11:40:06 EDT
B2DF78DE59474E8493719FA388399F90

1.0 Purpose

To outline the method of evaluating the neurological status of non-rodent animals. Covers the list of possible tests to be performed for an evaluation of the nervous system of dogs, cats, swine and NHP. This SOP could be used as a complete list from which the appropriate tests to be done on a given study can be selected (study specific required tests would be indicated in the Study Plan).

2.0 Scope

This SOP applies to all trained personnel performing neurological assessments on non-rodent animals.

3.0 Responsibilities

3.1 The Toxicology Technician and Clinical Veterinarian staff is responsible for proper execution of this procedure.

3.2 The Research Associate / Team Leader (or delegate) is responsible for preparing a master form of Appendix 1 or Appendix 5 (as appropriate) for the tests to be performed for a given study, based on the Study Plan and/or SOP.

3.3 The Study Director (or delegate) is responsible for approving the master form that will be used for the data collection on the study.

4.0 Definitions/ Abbreviations

NHP: Non-Human Primates

5.0 Materials

5.1 Required Forms (as appropriate)

Appendix 1 - Neurological Assessment Results Issue Date: 28-Apr-2021 (TB08-03-01)
Appendix 2 - Neurological Assessment and Observational Battery Exam Comment Sheet Issue Date: 21-Mar-2016 (TB08-03-01)
Appendix 5 - Observational Battery – Non-Human Primates Issue Date: 21-Mar-2016 (TB08-03-01)
Appendix 6 - Neurological Assessment – Non-Rodents - Legend Issue Date: 21-Mar-2016 (TB08-03-01)

Title:<br><br>**NEUROLOGICAL ASSESSMENTS - NON-RODENTS**	SOP Number:<br>TB08-03-01	Effective Date:<br>28-Apr-2021
	Version Number:<br>9.0	Site: CR-SEN-SHB

5.2 Required Material

Gauze
Forceps
Light pen
Rubber neurology hammer
Provantis PC with computer cart

6.0 <u>Procedure</u>

6.1 The master form (Appendix 1 or Appendix 5) will be prepared with the Study Number and the tests to be performed for the study. "NE – Not Evaluated" will be entered for the tests that are not applicable or not to be performed. The master form must be approved by the Study Director (or delegate) prior to the first use on study.

6.2 The approved master form will be kept in the Raw Data book and will be used to prepare the sheets for the offline recording, or a copy will be given to the technician as a guideline for online recording.

6.3 If the neurological assessment is performed offline prior to the randomization, Appendix 1 will be prepared using the animals' Arrival Number. Following the randomization, if the animal is assigned to a study group, the animal number will be added on the form.

6.4 When possible, staff performing the neurological assessment should be the same at each occurrence as to reduce variances in scoring.

6.5 The result of each test to be performed is recorded in Provantis, on Appendix 1 or Appendix 5 by the Technician or the Clinical Veterinarian. When performed online, a positive entry will be made for all tests; NE - Not Evaluated will be entered for the tests that are not required by the Study Plan or SOP.

6.6 Any results scored as abnormal are detailed in a comment in Provantis when performed online, or on Appendix 2 when performed offline. Abbreviations can be used but are restricted to those listed in the Appendix 6 for the identification of tests and sites. The inclusion of these abbreviations in the glossary does not exclude the use of the unabbreviated terms. Comments should also be used to describe what aspect of the result is abnormal, e.g. left or right when a single test is used for both left and right.

6.7 For any test where a single score entered represents two observations (e.g. left and right), all observations must be normal to record "1". If one observation is not normal, the appropriate score is entered and a comment entered, identifying the side, limb, etc.

6.8 The results are reviewed by the Research Associate / Team Leader (or delegate), the Study Director (or delegate) or the Clinical Veterinarian, or other appropriately trained personnel.

6.9 The neurological examination can consist of one or more of the following tests depending on the Study Plan requirements.

6.10 Scoring is recorded as per specific test and is defined as follows:

Scoring	
1	Normal
2	Abnormal (details added in comment)
3	Decreased
4	Increased
5	None
NE	Not Evaluated

7.0 General Attitude and Motor Function (dogs, cats, swine and NHP)

7.1 Except for NHP which are only examined in their cage, the animal's general behavior and awareness of the environment is assessed in both the cage and the examination area. For the Motor Function, limb movements and position are assessed by reference to their rate, range, force and direction. All steps are recorded as one entry.

Scoring	
1	Normal
2	Abnormal (details added in comment)
NE	Not Evaluated

8.0 Postural Reactions

8.1 Proprioceptive Positioning (dogs, cats, swine and NHP (NHP: hindlimbs only))

8.1.1 Each limb is flexed so the dorsal surface of the paw touches a solid horizontal surface (do not put the weight of the animal on the paw). The animal should return its paw to its normal position. The test is performed up to 3 times if required. The most often recorded response will be considered the score for this test. For chair restrained NHP, the test is performed while observing the animal cage side prior to placement in chair, as appropriate.

Scoring	
1	Normal
3	Decreased
5	None
NE	Not Evaluated

8.2 Extensor Thrust (dogs, cats and swine)

8.2.1 The animal is lifted off the ground and then lowered back to the ground, allowing the rear feet to touch the ground first. The animal should extend the limb in an attempt to touch the surface. The test is performed for left and right hindlimbs.

Scoring	
1	Normal
3	Decreased
4	Increased
NE	Not Evaluated

8.3 Placing Reactions-Visual (dogs, cats, swine and NHP (NHP: hindlimbs only))

8.3.1 The animal is carried toward a table top and observed if it reaches out to support itself as it approaches the table. This is done by supporting all limbs except one (then each limb is tested). For chair restrained NHP, the test is performed while observing the animal cage side prior to placement in chair, as appropriate.

Scoring	
1	Normal
3	Decreased
5	None
NE	Not Evaluated

8.4 Placing Reactions-Tactile (dogs, cats, swine and NHP (NHP: hindlimbs only; not conducted on chaired NHP))

8.4.1 The same tests as above (step 8.3) are done but the eyes of the animals are covered.

Scoring	
1	Normal
3	Decreased
5	None
NE	Not Evaluated

8.5 Righting Reaction (dogs, cats and swine)

8.5.1 With the animal placed in a lateral recumbency position, observe the animal's righting ability - this will be scored individually for each lateral aspects of the animal.

Scoring	
1	Normal
3	Decreased
5	None
NE	Not Evaluated

8.6 Eye Tracking (dogs, 5 weeks and older)

8.6.1 In a normal standing position, the eyes should track an object with head movement.

Scoring	
1	Normal (gaze pursuit with head movement)
5	None (does not follow target, unable to get attention)
NE	Not Evaluated

9.0 <u>Cranial Nerves</u>

9.1 Head Movement (dogs, cats, swine and NHP)

9.1.1 Observe the head for any evidence of head tilt, or involuntary movements.

Scoring	
1	Normal
2	Abnormal (details added in a comment)
NE	Not Evaluated

9.2 Head Symmetry (dogs, cats, swine and NHP)

9.2.1 Observe the head for any evidence of facial muscle asymmetry.

Scoring	
1	Normal
2	Abnormal (details added in comment)
NE	Not Evaluated

9.3 Head Muscle Tone (dogs, cats, swine and NHP (not conducted on chaired NHP))

9.3.1 Palpate the muscles of the head for tone and/or atrophy.

Scoring	
1	Normal
3	Decreased
4	Increased
5	None
NE	Not Evaluated

DocuSign Envelope ID: 6741D4CA-D260-42D8-8645-F9F63978E090

9.4 Menace Reflex (Eye Reactions) (dogs, cats, swine and NHP). For chaired NHP, the test is done for both eyes at once using one hand.

9.4.1 With one eye of the animal covered, threaten the opposite eye with a menacing gesture of the hand, being careful to avoid striking the animal or stimulating the area with air currents, test each side. The animal should close its eye.

Scoring	
1	Normal
3	Decreased
4	Increased
5	None
NE	Not Evaluated

9.5 Jaw Tone (dogs, cats and NHP)

9.5.1 Palpate the muscles of the jaw for tone and/or atrophy. This can also be done via observation of mastication and/or vocalization.

Scoring	
1	Normal
3	Decreased
4	Increased
5	None
NE	Not Evaluated

9.6 Pupil Symmetry (dogs, cats, swine and NHP)

9.6.1 Observe the symmetry of the pupil's position.

Scoring	
1	Normal
2	Abnormal (details added in comment)
NE	Not Evaluated

9.7 Eye Position (dogs, cats, swine and NHP (not conducted on chaired NHP))

9.7.1 The nose will be elevated when the animal is in the normal standing position. The eyes should remain in the middle of the palpebral fissure.

Scoring	
1	Normal
2	Abnormal (details added in comment)
NE	Not Evaluated

DocuSign Envelope ID: 6741D4CA-D260-42D8-8645-F9F63978E090

9.8 Palpebral Reflex (dogs, cats, swine and NHP (not conducted on chaired NHP))

9.8.1 The periphery of the cornea will be gently touched with the edge of a clean gauze pad for each animal. The animal should blink its eyelids including the nictitating membrane if applicable.

Scoring	
1	Normal
3	Decreased
5	None
NE	Not Evaluated

9.9 Pupillary Light Reflex (dogs, cats, swine and NHP (not conducted on chaired NHP))

9.9.1 In a dimly lit room, if possible, a bright focal light source will be directed from the temporal or lateral aspect of the eye through the pupil towards the retina.

- The direct light reflex will consist of observing the pupil for degree, speed and symmetry of constriction.

9.9.2 Each eye will be checked for the direct reflex.

9.9.3 In a normally lit room, the response may be tested by shading both eyes with the hand prior to exposure to the focal light source. The activity is entered as a single score.

Scoring	
1	Normal
2	Abnormal (details added in comment)
5	None
NE	Not Evaluated

9.10 Vestibular Nystagmus (dogs, cats, swine and NHP (not conducted on chaired NHP))

- Move the head from side to side to generate normal vestibular nystagmus. The eyes should follow the direction of the head in sudden movements.

Scoring	
1	Normal
2	Abnormal (details added in comment)
NE	Not Evaluated

Title: NEUROLOGICAL ASSESSMENTS - NON-RODENTS	SOP Number: TB08-03-01	Effective Date: 28-Apr-2021
	Version Number: 9.0	Site: CR-SEN-SHB

10.0 Spinal Nerves

10.1 Muscle Tone (dogs, cats, swine and NHP (NHP: hindlimbs only))

10.1.1 Using gentle manipulation of each limb, the degree of resistance is determined. A normal animal should have a slight resistance.

Scoring	
1	Normal
3	Decreased
4	Increased
5	None
NE	Not Evaluated

10.2 Patellar Reflexes (dogs, cats and swine)

10.2.1 With the animal in a relaxed position (lateral recumbency for cats and dogs) place the upper hindlimb in a semi flexed position. The patellar tendon below the knee is lightly tapped with a rubber neurology hammer. The stifle joint should quickly extend.

Scoring	
1	Normal
3	Decreased
4	Increased
5	None
NE	Not Evaluated

10.3 Flexor Reflex (dogs, cats, swine and NHP (chaired NHP: hindlimbs only)

10.3.1 The base of the nail (one) of each limb is quickly and lightly pinched with a haemostat. The animal should react with motion or retract its limb.

Scoring	
1	Normal
3	Decreased
4	Increased
5	None
NE	Not Evaluated

10.4 Panniculus Reflex (dogs, cats and swine)

10.4.1 Starting in the cranial region of the thorax and going caudally, gently stimulate the different areas of the skin with a forcep. The cutaneous trunci muscle should contract making the skin of the area move. The evaluations will be performed for each side of the body (right and left). A single score is entered for both assessments.

Scoring	
1	Normal
2	Abnormal (details added in comment)
NE	Not Evaluated

10.5 Perineal Reflex (dogs, cats, swine and NHP)

10.5.1 The anus is gently stimulated with a forcep. The normal reflex will make the anal sphincter contract and/or the tail flex.

Scoring	
1	Normal
5	None
NE	Not Evaluated

11.0 <u>Additional Optional Assessments</u>

Note:
- The following assessments are additional assessments when required by Study Plan.

11.1 Postural Reactions

11.1.1 Hemihopping and Hemistanding (dogs, cats and swine)

- The limbs on one side are held off the ground while the animal is forced to walk sideways on its two remaining limbs. A normal animal should maintain itself straight. Both sides are tested. A single score is entered for both sides.

Scoring	
1	Normal
3	Decreased
5	None
NE	Not Evaluated

11.1.2 Wheel Barrowing (dogs, cats and swine)

- Thoracic or pelvic limbs are held off the ground while the animal is moved forwards (holding pelvic limbs) and then backwards (holding thoracic limbs) on its two remaining limbs. A normal animal should support itself during the test. A single score is entered for both tests.

Scoring	
1	Normal
3	Decreased
5	None
NE	Not Evaluated

11.1.3 Hopping (dogs, cats and swine)

- All limbs except one are supported, making the animal hop on that limb. A normal animal should support itself on its limb. A single score is entered for all limbs.

Scoring	
1	Normal
3	Decreased
5	None
NE	Not Evaluated

11.2 Cranial Nerves

11.2.1 Tongue Test (dogs, cats and swine)

- Pull the tongue and observe its size (symmetry), movement and strength.

Scoring	
1	Normal
2	Abnormal (details added in comment)
NE	Not Evaluated

11.2.2 Pharynx Test (dogs, cats and swine)

- Probe the pharynx with a finger and verify if animal is able to swallow or gag.

Scoring	
1	Normal
3	Decreased
5	None
NE	Not Evaluated

Title:<br><br>**NEUROLOGICAL ASSESSMENTS - NON-RODENTS**	SOP Number:<br>TB08-03-01	Effective Date:<br>28-Apr-2021
	Version Number:<br>9.0	Site: CR-SEN-SHB

12.0 Non-Human Primate Specific Battery

12.1 General Notes

12.1.1 When possible, staff performing the observational battery should be the same at each occurrence as to reduce variance in response by the animal and in scoring. The testing Technician should be blind to the study groups and not be familiar with study animals in order to not be biased.

12.1.2 The animal cage card indicating the study group number will be removed and replaced with the animal arrival number. The study Team Leader or delegate will fill in the animal study number on Appendix 1 once the evaluation is completed.

12.1.3 Observations will occur in random order throughout the room.

12.1.4 During the evaluation, outside disturbances should be kept to a minimum. It is recommended to post a door sheet stating “Testing in Progress”. In addition, the room door windows should be covered in order to minimize disturbances.

12.1.5 The observational battery, (OB) should be performed between 1-3 hours post feeding.

12.1.6 Animal separation (if not already performed) and cage tray rinse is performed a minimum of 30 minutes prior to observations. The cage/room wash should not be performed within 2 hours before observations. Should excessive cage or self-licking be observed, indicate the time of the tray or cage wash on the appropriate Appendix.

12.1.7 The results are recorded on the appropriate Appendix by the Technician or the Clinical Veterinarian. Values in parentheses represent “Normal” or “None”.

12.1.8 When the letter “C” is entered as a value or is separated by a comma after a score, and a check (✓) is made in the Comments box, the details of the observation will be recorded on the Comment Sheet (Appendix 2). Otherwise if only, a check (✓) is placed in the comment section this will indicate a general comment. In the “Test” column of the appendix, indicate the test number as found in the SOP. E.g. Arousal level = 7.4. If a comment is not test specific, the Test box is to be crossed out.

12.2 Body Position

Scoring	
1	Lying down - Sternal
2	Lying down - Lateral
3	Sitting
4	Quadrupedal standing
5	Hanging or bipedal standing
6	Climbing
7	Hunched posture
C	Other: refer to Comment sheet

12.3 Arousal level

Scoring	
1	None: resting or sleeping
2	Slight movement
0	Normal movement
3	Vigorous, constant movement
4	Sudden, erratic movement
C	Other: refer to Comment sheet

12.4 Locomotion

12.4.1 In the case where the animal has not moved during the observation period, the Technician is to stimulate the animal to move in order to assess locomotion. The locomotion is scored as follows (e.g..: S1).

Scoring	
0	Normal
1	Abnormal gait
2	Limited usage of limb(s) (indicate limb(s)): LF, RF, LH, RH
3	Animal did not move
S	Animal stimulated to move

12.5 Tremors: involuntary trembling motions of body, head or limbs

Scoring	
0	None
1	Slight
2	Moderate
3	Severe
B	Body
H	Head
L*	Limb (s) (*indicate limb(s)) LF, RF, LH, RH

12.6 Twitches: sudden involuntary jerk of limbs

Scoring	
0	None
1	Slight
2	Moderate
3	Severe

DocuSign Envelope ID: 6741D4CA-D260-42D8-8645-F9F63978E090

12.7 Convulsions

Scoring	
0	None
**Clonic-Type convulsions**: persistent contractions and relaxation of voluntary muscles	
CL	Clonic: a violent contraction and relaxation of muscles of the head and/or limbs
CH	Chomping: clonus of jaws, “chewing” motion
**Tonic –Type convulsions**: persistent contraction of voluntary muscles	
T	Tonic: sustained extension or limbs
OP	Opisthotonos: head and body arched backwards
EM	Emprosthotonos: head and body extended forward

DocuSign Envelope ID: 6741D4CA-D260-42D8-8645-F9F63978E090

12.8 Bizarre/Stereotypic Behavior

12.8.1 Additional detail or clarification may be provided in the Comments (Appendix 2)

Scoring	
0	None
**If present, record description and severity**	
L	Compulsive licking
R	Retropulsion: backward walking
P	Polidipsia (excessive drinking)
BO	Biting objects: frequently bites objects or cage
CG	Circling
EP	Eye poking
HG	Hand/Foot gesture, holding or maintaining hand/foot in an atypical manner
HS	Head search: stereotypic turning from side to side
SB	Self-biting: bites limbs or body
SP	Stereotypic pacing
SW	Staring at cage wall, self or other object
C	Other: description recorded on Comment sheet
**Severity**	
1	Slight (occasional)
2	Moderate (about half the time of observation)
3	Severe (over half the time of the observation)

12.8.2 Facial Observations

Scoring	
1	Symmetrical
2	Asymmetrical

12.9 Vocalization

Scoring	
0	None
1	Slight (Less than half the time of observation)
2	Moderate (More than half the time of observation)
3	Severe (Non-stop during observation)

12.10 Palpebral Closure

Scoring	
0	Normal (both eyes open)
1	½ closed
2	Completely closed

Title: NEUROLOGICAL ASSESSMENTS - NON-RODENTS	SOP Number: TB08-03-01	Effective Date: 28-Apr-2021
	Version Number: 9.0	Site: CR-SEN-SHB

12.11 Respiratory Rate/Pattern

Scoring	
0	Normal
1	Decreased
2	Increased
L	Labored
D	Dyspneic
G	Gasping
A	Abnormal sounds

12.12 Vomiting: Must see animal vomit

Scoring	
0	None
R	Retching
#	Record number of times vomited
M	Material in tray

12.13 Defecation: Must see animal defecate

Scoring	
0	None
#	Record number of stools
D	Diarrhea
M	Material in tray

12.14 Urination: Must see animal urinate

Scoring	
0	None
1	Normal urination
2	Severe/Polyuria

12.15 Lacrimation

Scoring	
0	None
1	Present (both eyes)
2	Left eye only
3	Right eye only

DocuSign Envelope ID: 6741D4CA-D260-42D8-B645-F9F63978E090

12.16 Salivation

Scoring	
0	None
1	Slight
2	Moderate
3	Severe

13.0 References

N/A

14.0 Revision History

Version	Date	Reason For Revision
9.0	28-Apr-2021	Section 6.0 clarified to indicate that a master form needs to be approved at the beginning of the study.<br>Minor typographical/formatting fixed throughout the document.<br>Section 9.10 added Vestibular Nystagmus (and removed from Section 11.2.1)<br>Appendix 1 Vestibular Nystagmus added as a default parameter.

FINAL REPORT

Study Phase: Nerve Conduction Velocity

Test Facility Study No. 5550014

Sponsor Reference No. UTSW-Gray-SPG50-004

GLP

TEST FACILITY:
Charles River Laboratories Montreal ULC
Senneville Site (CR-SEN)

TABLE OF CONTENTS

LIST OF APPENDICES 3
1. INTRODUCTION 4
2. MATERIALS AND METHODS 4
2.1. Experimental Design 4
2.2. Nerve Conduction Velocity (NCV) 4
2.3. Systems 4
2.4. Statistical Analysis 5
3. RESULTS AND DISCUSSION 5
4. CONCLUSION 8
5. REPORT APPROVAL 9

LIST OF APPENDICES

Appendix 1 Individual and Summary of Nerve Conduction Velocity (NCV) 11

1. INTRODUCTION

This report presents the nerve conduction velocity summary findings in monkeys assigned to Study No. 5550014: *A Single-Dose Study of AAV9/AP4M1 by Intrathecal Injection in Immunosuppressed Monkeys*. The objectives of this study were to determine the potential toxicity, biodistribution, and gene expression of AAV9/AP4M1, for the treatment of Spastic Paraplegia Type 50 (SPG50) caused by the AP4M1 gene mutation, when given by a single intrathecal injection to monkeys, to evaluate the potential reversibility and/or progression of any findings, and to determine the potential impact of immunosuppressant (IMS) on the toxicity profile of this AAV9 vector.

For the work detailed in this report, the phase start date was 05 Jul 2021, and the phase completion date was 12 Oct 2021.

2. MATERIALS AND METHODS

2.1. Experimental Design

Experimental design applicable to nerve conduction velocity evaluations are summarized in Text Table 1.

Text Table 1
Experimental Design

Group No.	Test Material	Dose Level (vg)	Dose Volume (mL)	Dose Concentration (vg/mL)	Animal Nos. Main Study	
					Males	Females
1	Reference Item	0	1	0	1201	1701
2	AAV9/AP4M1	8.4×10^{13}	1.55	5.43×10^{13}	-	2701-2702
3	AAV9/AP4M1	1.68×10^{14}	3.10	5.43×10^{13}	3201	3701

2.2. Nerve Conduction Velocity (NCV)

Frequency: Once prestudy and on Days 45, 77, and 92.

Anesthesia: An intramuscular injection of ketamine, glycopyrrolate and dexmedetomidine was administered following an appropriate fasting period. Following the completion of the assessment, a reversal agent, atipamezole, was administered.

Evaluation:
Peroneal motor: NCV and Amplitude
Sural sensory: NCV and Amplitude
Cauda equina: Onset latency

2.3. Systems

Critical computerized systems used in this study phase are listed below.

Text Table 2
Computerized Systems

System Name	Version No.	Description of Data Collected and/or Analyzed
Acq*Knowledge*	4.4	Electrophysiological recording
Excel	2016	Tabulated data entry

2.4. Statistical Analysis

The data was presented as individual and mean values along with standard deviation for each measurement.

3. RESULTS AND DISCUSSION

Prior to initiation of dosing, all measurements for the peroneal motor nerve, sural sensory nerve and the cauda equina were comparable with concurrent control assessments.

Following a single intrathecal administration of AAV9/AP4M1 at $8.4x10^{13}$ and $1.68x10^{14}$ vg, no changes in peroneal nerve conduction velocity or amplitude and no changes in the onset latency of the cauda equina were noted up to Day 92. In addition, there were no changes in conduction velocity or amplitude for the sural nerve at $8.4x10^{13}$ vg. All measurements were comparable to the concurrent control.

Intrathecal administration of AAV9/AP4M1 at $1.68x10^{14}$ vg resulted in a slight decrease in sural nerve conduction velocity of 11% on Day 45, relative to the control group, accompanied by a decrease in response amplitude of 27%. Animal no. 3201 exhibited the most significant change with nerve conduction velocity of 44 m/sec compared to 52 m/sec in control animals.

Intrathecal administration of AAV9/AP4M1 at $1.68x10^{14}$ vg resulted in significant decreases in sural nerve conduction velocity of 26% on Day 77 and of 28% on Day 92, relative to control. The changes in conduction velocity were accompanied by associated decreases in sural nerve response amplitude which was reduced by 69% on Day 77 and by 74% on Day 92.
Animal no. 3201 was the most impacted animal exhibiting the most pronounced changes with nerve conduction velocity of 34 m/sec (35% change relative to control) on Day 77 and 32 m/sec (39% change relative to control) on Day 92 compared to 52-53 m/sec in control animals. The most pronounced decrease in response amplitude was also noted in Animal no. 3201, especially on Day 92, during which an amplitude of 1.2 µV was noted compared to a mean amplitude of 10.2 µV in control animals.

The changes in sural nerve conduction velocity are presented in figures below:

Appendix 9

Text Figures 1-6
Individual Sural Nerve Conduction Velocity across occasions

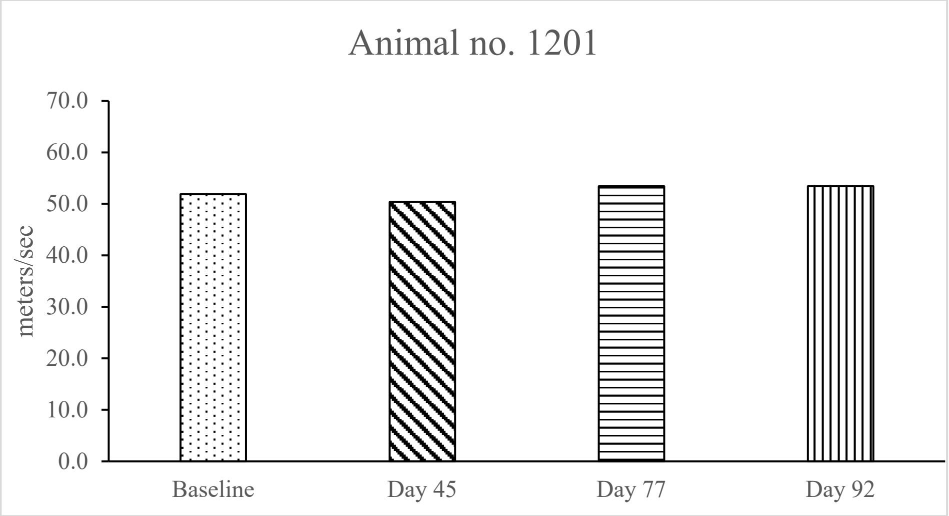

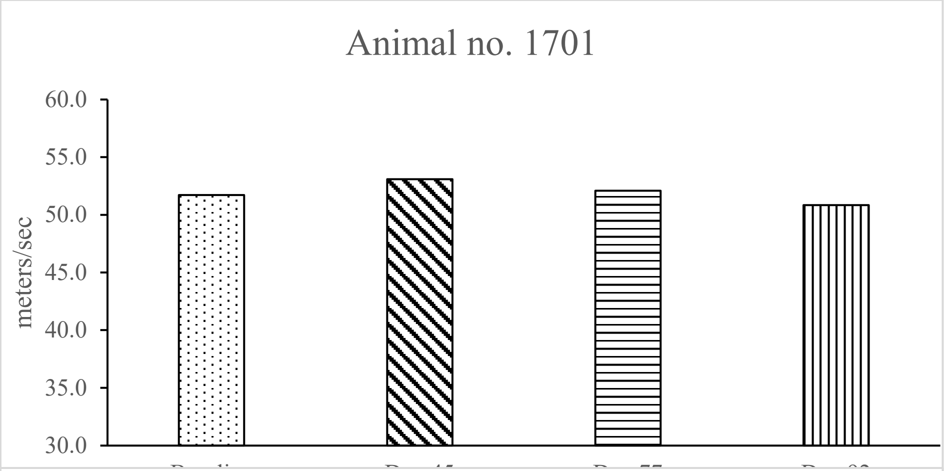

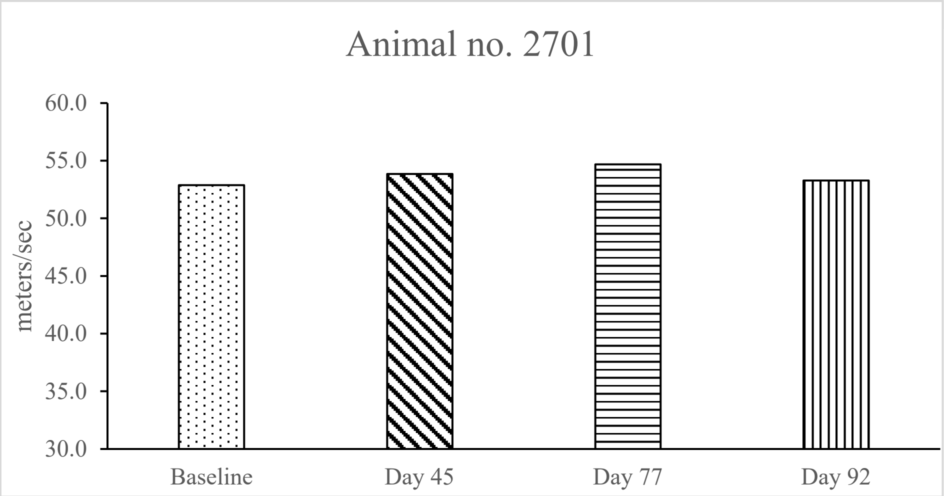

Appendix 9

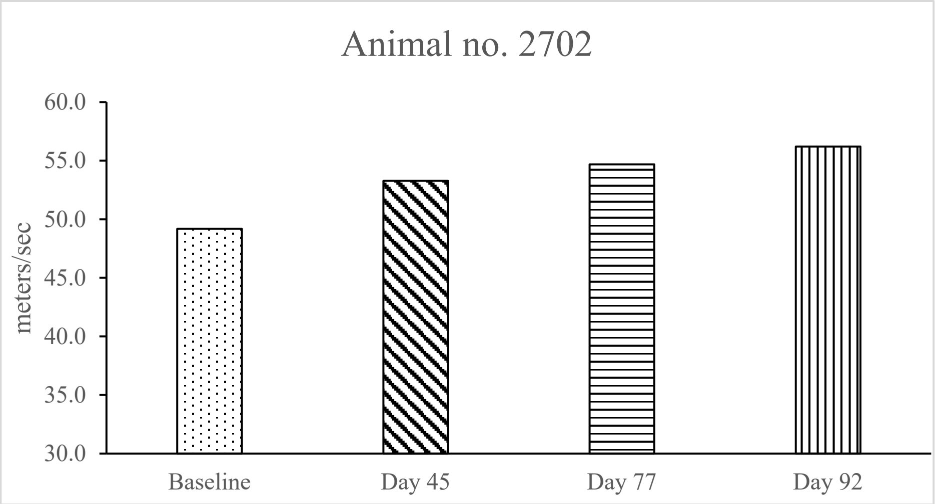
Animal no. 2702
60.0
55.0
50.0
45.0
40.0
35.0
30.0
meters/sec
Baseline
Day 45
Day 77
Day 92

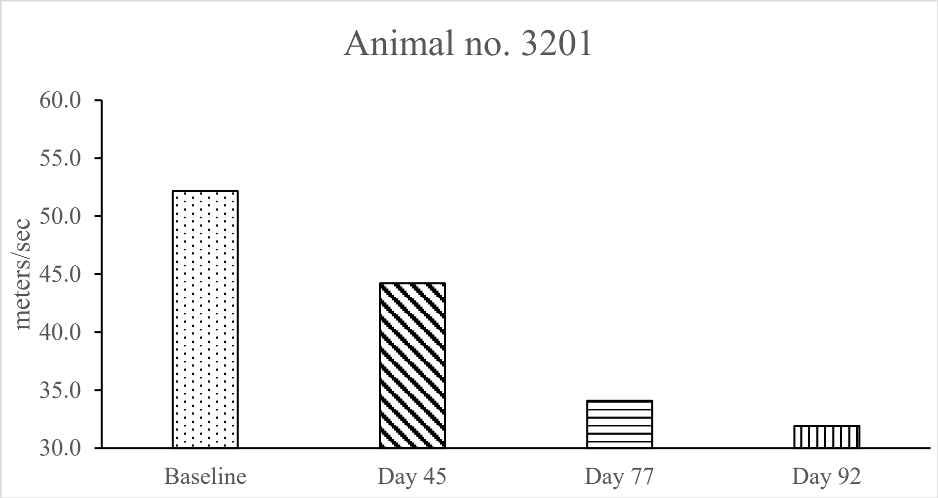
Animal no. 3201
60.0
55.0
50.0
45.0
40.0
35.0
30.0
meters/sec
Baseline
Day 45
Day 77
Day 92

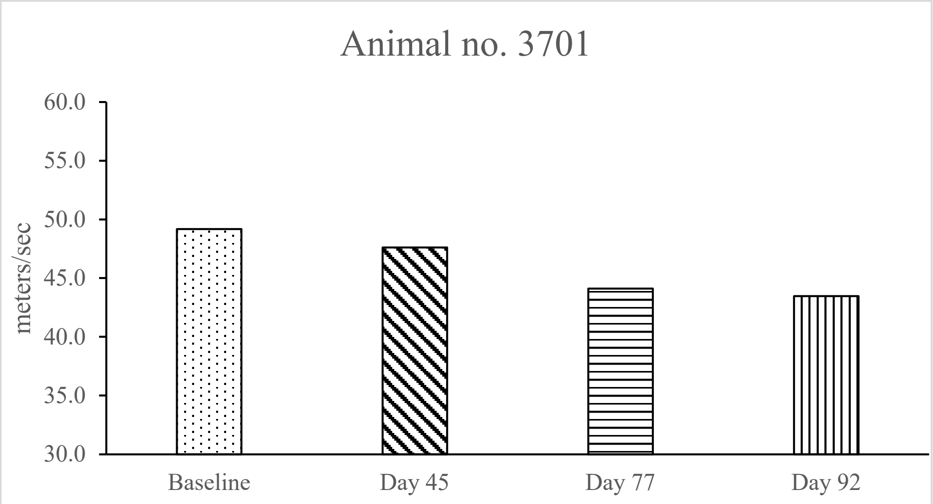
Animal no. 3701
60.0
55.0
50.0
45.0
40.0
35.0
30.0
meters/sec
Baseline
Day 45
Day 77
Day 92

4. CONCLUSION

Intrathecal administration of AAV9/AP4M1 at 1.68×10^{14} vg resulted in a decrease in nerve conduction velocity and response amplitude of the sural nerve on Day 45, 77 and 92 with no deficits noted in peroneal or cauda equina nerves. Intrathecal administration of AAV9/AP4M1 at 8.4×10^{13} vg resulted in no deficits in any of the nerves assessed.

5. REPORT APPROVAL

All electronic signatures appear at the end of the document upon finalization.

Individual Nerve Conduction Velocity (NCV) Explanation Page

Abbreviation	Description	Abbreviation	Description
./-	Not scheduled to be performed/dead	M	Male
Diff	Difference	NR	Not recorded
F	Female	Temp	Temperature
Lat	Latency	X	Excluded from mean

Note: This is a comprehensive list of abbreviations. All of the abbreviations listed may not be applicable to this report.

Dosing Information

Dosing information is abbreviated on various data outputs; the following represents the dosing information for this study.

Group No.	Test Material	Dose Level (vg)
1	Reference Item	0
2	AAV9/AP4M1	$8.4x10^{13}$
3	AAV9/AP4M1	$1.68x10^{14}$

Appendix 9

Appendix 1

Individual and Summary of Nerve Conduction Velocity (NCV)

Prestudy

Group 1	Peroneal Motor Response - Left							Sural Nerve Response - Left				Cauda Equina	Temp
Animal ID/ Sex	Proximal on. Lat (msec)	Proximal Amplitude (mV)	Distal on. Lat (msec)	Distal Amplitude (mV)	Diff	Distance (mm)	NCV (m/sec)	Onset Latency (msec)	Amplitude (µV)	Distance (mm)	NCV (m/sec)	Onset Latency (msec)	(°C)
1201 M	4.5	17.8	2.8	16.9	1.7	110	65.7	1.1	23.6	55	51.9	2.8	37.0
1701 F	5.1	20.3	3.3	19.5	1.8	105	57.5	1.2	10.7	60	51.7	2.9	36.2
Mean	4.8	19.0	3.0	18.2	1.8	107.5	61.6	1.1	17.2	57.5	51.8	2.8	36.6
SD	0.5	1.7	0.4	1.9	0.1	3.5	5.8	0.1	9.1	3.5	0.1	0.1	0.6
N	2	2	2	2	2	2	2	2	2	2	2	2	2

Group 2	Peroneal Motor Response - Left							Sural Nerve Response - Left				Cauda Equina	Temp
Animal ID/ Sex	Proximal on. Lat (msec)	Proximal Amplitude (mV)	Distal on. Lat (msec)	Distal Amplitude (mV)	Diff	Distance (mm)	NCV (m/sec)	Onset Latency (msec)	Amplitude (µV)	Distance (mm)	NCV (m/sec)	Onset Latency (msec)	(°C)
2701 F	4.3	19.8	2.6	17.6	1.8	108	60.8	1.0	16.9	55	52.9	2.6	37.4
2702 F	4.1	20.3	2.3	19.1	1.8	110	60.3	1.2	24.2	60	49.2	2.9	38.2
Mean	4.2	20.1	2.4	18.4	1.8	109	60.6	1.1	20.6	58	51.0	2.7	37.8
SD	0.1	0.3	0.2	1.1	0.0	1.4	0.4	0.1	5.2	3.5	2.6	0.2	0.6
N	2	2	2	2	2	2	2	2	2	2	2	2	2

Group 3	Peroneal Motor Response - Left							Sural Nerve Response - Left				Cauda Equina	Temp
Animal ID/ Sex	Proximal on. Lat (msec)	Proximal Amplitude (mV)	Distal on. Lat (msec)	Distal Amplitude (mV)	Diff	Distance (mm)	NCV (m/sec)	Onset Latency (msec)	Amplitude (µV)	Distance (mm)	NCV (m/sec)	Onset Latency (msec)	(°C)
3201 M	4.5	19.8	2.6	0.0	1.9	112	59.7	1.2	19.0	60	52.2	2.8	38.2
3701 F	5.1	19.7	3.1	0.0	2.0	118	58.3	1.2	21.6	60	49.2	2.8	37.9
Mean	4.8	19.7	2.8	0.0	2.0	115.0	59.0	1.2	20.3	60.0	50.7	2.8	38.1
SD	0.4	0.1	0.3	0.0	0.1	4.2	1.0	0.0	1.8	0.0	2.1	0.0	0.2
N	2	2	2	2	2	2	2	2	2	2	2	2	2

Appendix 9

Appendix 1

Individual and Summary of Nerve Conduction Velocity (NCV)

Day 45

Group 1	Peroneal Motor Response - Left							Sural Nerve Response - Left				Cauda Equina	Temp
Animal ID/ Sex	Proximal on. Lat (msec)	Proximal Amplitude (mV)	Distal on. Lat (msec)	Distal Amplitude (mV)	Diff	Distance (mm)	NCV (m/sec)	Onset Latency (msec)	Amplitude (μV)	Distance (mm)	NCV (m/sec)	Onset Latency (msec)	(°C)
1201 M	4.6	7.1	2.7	8.5	1.9	115	61.3	1.4	27.0	70	50.4	3.0	37.4
1701 F	4.6	20.0	3.1	15.2	1.5	90	60.0	1.1	12.6	60	53.1	2.9	37.5
Mean	4.6	13.5	2.9	11.9	1.7	102.5	60.7	1.3	19.8	65.0	51.7	2.9	37.5
SD	0.0	9.2	0.3	4.8	0.3	17.7	0.9	0.2	10.2	7.1	1.9	0.0	0.1
N	2	2	2	2	2	2	2	2	2	2	2	2	2

Group 2	Peroneal Motor Response - Left							Sural Nerve Response - Left				Cauda Equina	Temp
Animal ID/ Sex	Proximal on. Lat (msec)	Proximal Amplitude (mV)	Distal on. Lat (msec)	Distal Amplitude (mV)	Diff	Distance (mm)	NCV (m/sec)	Onset Latency (msec)	Amplitude (μV)	Distance (mm)	NCV (m/sec)	Onset Latency (msec)	(°C)
2701 F	4.6	17.6	3.0	18.4	1.6	100	61.5	1.3	22.7	70	53.8	2.7	37.0
2702 F	4.4	17.9	2.4	19.1	2.1	120	58.5	1.2	26.5	65	53.3	2.9	37.5
Mean	4.5	17.7	2.7	18.7	1.8	110	60.0	1.3	24.6	68	53.6	2.8	37.3
SD	0.1	0.2	0.4	0.5	0.3	14.1	2.1	0.1	2.7	3.5	0.4	0.2	0.4
N	2	2	2	2	2	2	2	2	2	2	2	2	2

Group 3	Peroneal Motor Response - Left							Sural Nerve Response - Left				Cauda Equina	Temp
Animal ID/ Sex	Proximal on. Lat (msec)	Proximal Amplitude (mV)	Distal on. Lat (msec)	Distal Amplitude (mV)	Diff	Distance (mm)	NCV (m/sec)	Onset Latency (msec)	Amplitude (μV)	Distance (mm)	NCV (m/sec)	Onset Latency (msec)	(°C)
3201 M	4.5	15.2	2.5	18.2	2.0	115	57.5	1.5	16.0	65	44.2	3.3	37.2
3701 F	4.4	17.6	2.5	15.6	1.9	110	59.5	1.3	13.0	60	47.6	3.0	36.5
Mean	4.5	16.4	2.5	16.9	1.9	112.5	58.5	1.4	14.5	62.5	45.9	3.1	36.9
SD	0.1	1.7	0.0	1.9	0.1	3.5	1.4	0.1	2.1	3.5	2.4	0.2	0.5
N	2	2	2	2	2	2	2	2	2	2	2	2	2

Appendix 9

Appendix 1

Individual and Summary of Nerve Conduction Velocity (NCV)

Day 77

Group 1	Peroneal Motor Response - Left							Sural Nerve Response - Left				Cauda Equina	Temp
Animal ID/ Sex	Proximal on. Lat (msec)	Proximal Amplitude (mV)	Distal on. Lat (msec)	Distal Amplitude (mV)	Diff	Distance (mm)	NCV (m/sec)	Onset Latency (msec)	Amplitude (µV)	Distance (mm)	NCV (m/sec)	Onset Latency (msec)	(°C)
1201 M	4.3	11.5	2.5	12.9	1.8	115	65.7	1.3	14.2	70	53.4	2.9	37.1
1701 F	4.9	10.6	2.9	16.9	2.0	110	55.7	1.2	14.5	62	52.1	2.8	37.0
Mean	4.6	11.0	2.7	14.9	1.9	112.5	60.7	1.3	14.4	66.0	52.8	2.8	37.1
SD	0.4	0.7	0.3	2.8	0.2	3.5	7.1	0.1	0.2	5.7	0.9	0.1	0.1
N	2	2	2	2	2	2	2	2	2	2	2	2	2

Group 2	Peroneal Motor Response - Left							Sural Nerve Response - Left				Cauda Equina	Temp
Animal ID/ Sex	Proximal on. Lat (msec)	Proximal Amplitude (mV)	Distal on. Lat (msec)	Distal Amplitude (mV)	Diff	Distance (mm)	NCV (m/sec)	Onset Latency (msec)	Amplitude (µV)	Distance (mm)	NCV (m/sec)	Onset Latency (msec)	(°C)
2701 F	4.3	19.8	2.4	19.7	1.9	110	58.7	1.3	14.7	70	54.7	2.7	37.9
2702 F	4.2	19.7	2.4	19.6	1.9	105	56.8	1.3	20.4	70	54.7	2.8	36.8
Mean	4.2	19.7	2.4	19.7	1.9	108	57.7	1.3	17.6	70	54.7	2.7	37.4
SD	0.1	0.0	0.0	0.1	0.0	3.5	1.4	0.0	4.0	0.0	0.0	0.0	0.8
N	2	2	2	2	2	2	2	2	2	2	2	2	2

Group 3	Peroneal Motor Response - Left							Sural Nerve Response - Left				Cauda Equina	Temp
Animal ID/ Sex	Proximal on. Lat (msec)	Proximal Amplitude (mV)	Distal on. Lat (msec)	Distal Amplitude (mV)	Diff	Distance (mm)	NCV (m/sec)	Onset Latency (msec)	Amplitude (µV)	Distance (mm)	NCV (m/sec)	Onset Latency (msec)	(°C)
3201 M	4.4	15.6	2.5	18.5	1.9	110	57.9	1.8	5.0	60	34.1	2.9	38.1
3701 F	4.3	16.7	2.5	15.4	1.8	100	56.3	1.4	4.0	60	44.1	2.6	37.3
Mean	4.3	16.2	2.5	17.0	1.8	105.0	57.1	1.6	4.5	60.0	39.1	2.8	37.7
SD	0.1	0.8	0.0	2.2	0.1	7.1	1.1	0.3	0.7	0.0	7.1	0.2	0.6
N	2	2	2	2	2	2	2	2	2	2	2	2	2

Appendix 9

Appendix 1

Individual and Summary of Nerve Conduction Velocity (NCV)

Day 92

Group 1	Peroneal Motor Response - Left							Sural Nerve Response - Left				Cauda Equina	Temp
Animal ID/ Sex	Proximal on. Lat (msec)	Proximal Amplitude (mV)	Distal on. Lat (msec)	Distal Amplitude (mV)	Diff	Distance (mm)	NCV (m/sec)	Onset Latency (msec)	Amplitude (µV)	Distance (mm)	NCV (m/sec)	Onset Latency (msec)	(°C)
1201 M	4.9	12.8	2.8	11.3	2.1	115	55.4	1.3	12.1	70	53.4	2.8	38.0
1701 F	4.5	10.5	2.5	10.6	1.9	110	57.1	1.2	8.3	60	50.8	2.9	36.9
Mean	4.7	11.6	2.7	11.0	2.0	112.5	56.3	1.2	10.2	65.0	52.1	2.9	37.5
SD	0.3	1.6	0.2	0.5	0.1	3.5	1.2	0.1	2.7	7.1	1.8	0.1	0.8
N	2	2	2	2	2	2	2	2	2	2	2	2	2

Group 2	Peroneal Motor Response - Left							Sural Nerve Response - Left				Cauda Equina	Temp
Animal ID/ Sex	Proximal on. Lat (msec)	Proximal Amplitude (mV)	Distal on. Lat (msec)	Distal Amplitude (mV)	Diff	Distance (mm)	NCV (m/sec)	Onset Latency (msec)	Amplitude (µV)	Distance (mm)	NCV (m/sec)	Onset Latency (msec)	(°C)
2701 F	4.3	20.7	2.7	17.1	1.6	110	68.8	1.2	12.6	65	53.3	2.6	37.5
2702 F	4.1	22.4	2.1	19.2	1.9	105	54.5	1.2	11.1	68	56.2	2.7	38.2
Mean	4.2	21.5	2.4	18.1	1.8	108	61.6	1.2	11.9	67	54.7	2.6	37.9
SD	0.1	1.2	0.4	1.5	0.2	3.5	10.0	0.0	1.1	2.1	2.1	0.1	0.5
N	2	2	2	2	2	2	2	2	2	2	2	2	2

Group 3	Peroneal Motor Response - Left							Sural Nerve Response - Left				Cauda Equina	Temp
Animal ID/ Sex	Proximal on. Lat (msec)	Proximal Amplitude (mV)	Distal on. Lat (msec)	Distal Amplitude (mV)	Diff	Distance (mm)	NCV (m/sec)	Onset Latency (msec)	Amplitude (µV)	Distance (mm)	NCV (m/sec)	Onset Latency (msec)	(°C)
3201 M	4.6	13.4	2.7	14.6	1.9	110	57.1	1.9	1.2	60	31.9	3.1	36.6
3701 F	4.4	13.2	2.4	15.0	2.1	120	57.8	1.4	4.3	60	43.5	2.8	37.3
Mean	4.5	13.3	2.5	14.8	2.0	115.0	57.5	1.6	2.7	60.0	37.7	2.9	37.0
SD	0.1	0.1	0.2	0.3	0.1	7.1	0.5	0.4	2.1	0.0	8.2	0.2	0.5
N	2	2	2	2	2	2	2	2	2	2	2	2	2

Appendix 9

SIGNATURE(S) FOR DOCUMENT: 5550014 - 5550014 Other Final Report Nerve Conduction Velocity

**Individual Scientist:**	I approve this document.
Name:	**Sadekova, Nataliya**
Sadekova, Nataliya	03-Mar-2022 19:48:36 (UTC+00:00)
Electronically Signed in M-Files	Timestamp

Appendix 10

FINAL REPORT

Study Phase: Clinical Pathology

Test Facility Study No. 5550014

Sponsor Reference No. UTSW-Gray-SPG50-004

GLP

TEST FACILITY:
Charles River Laboratories Montreal ULC
Senneville Site (CR-SEN)

TABLE OF CONTENTS

LIST OF APPENDICES 3
1. SUMMARY 4
2. INTRODUCTION 5
3. MATERIALS AND METHODS 5
3.1. Experimental Design 5
3.2. Computerized Systems 5
4. RESULTS AND DISCUSSIONS 6
4.1. Hematology 6
4.2. Coagulation 6
4.3. Clinical Chemistry 7
4.4. Urinalysis 7
5. CONCLUSIONS 8
6. REPORT APPROVAL 9

LIST OF APPENDICES

Appendix 1 Individual Hematology Values 17
Appendix 2 Individual Coagulation Values 42
Appendix 3 Individual Clinical Chemistry Values 47
Appendix 4 Individual Urinalysis Values 67

1. SUMMARY

The objectives of this study were to determine the potential toxicity, biodistribution, and gene expression of AAV9/AP4M1, for the treatment of Spastic Paraplegia Type 50 (SPG50) caused by the AP4M1 gene mutation, when given by a single intrathecal injection to monkeys, to evaluate the potential reversibility and/or progression of any findings, and to determine the potential impact of immunosuppressant (IMS) on the toxicity profile of this AAV9 vector.

Three groups of monkeys were administered AAV9/AP4M1 via a single percutaneous intrathecal injection at doses of 0 (Reference), 8.4×10^{13}, or 1.68×10^{14} vg. Blood and urine samples were collected for the evaluation of clinical pathology parameters (hematology, coagulation, clinical chemistry and urinalysis). Animals designated for the terminal necropsy (1/sex in Groups 1 and 3 and 2 females in Group 2) were euthanized on Day 94.

No AAV9/AP4M1-related hematology, clinical chemistry and urinalysis changes were noted at any dose level.

Equivocal mild increases in fibrinogen concentration were noted in male and female administered AAV9/AP4M1 at 1.68×10^{14} vg on Days 52 and 94 (1.82x to 2.55x, individual values compared to respective pretreatment), of higher magnitude than the increases observed in control animals (1.15x to 1.47x). These increases were not associated with relevant changes in globulins or leukocytes and may have been associated with the microscopic necrosis/inflammation observed in the biceps femoris at injection sites and remain of an uncertain relationship to AAV9/AP4M1.

On Day 24 only, one female administered AAV9/AP4M1 at 8.4×10^{13} vg presented transient mild increases in neutrophil (1.56x, individual value compared to respective Day -8 pretreatment) and monocyte (3.77x) counts, a mild increase in fibrinogen concentration (2.11x), mild increases in globulins (1.32x), triglycerides (4.31x), and mild decreases in albumin (0.81x) and albumin/globulin ratio (0.57x). These changes were supportive of inflammation and were possibly related to a moderate skin lesion of the left hindlimb observed on Day 28 in this animal. They were not observed on Day 52 or Day 94, after resolution of the skin lesion. This individual had no particular microscopic findings observed at the end of the study; hence these changes were unlikely AAV9/AP4M1-related.

2. INTRODUCTION

This report presents the clinical pathology findings in monkeys assigned to Study No. 5550014. The objectives of this study were to determine the potential toxicity, biodistribution, and gene expression of AAV9/AP4M1, for the treatment of Spastic Paraplegia Type 50 (SPG50) caused by the AP4M1 gene mutation, when given by a single intrathecal injection to monkeys, to evaluate the potential reversibility and/or progression of any findings, and to determine the potential impact of immunosuppressant (IMS) on the toxicity profile of this AAV9 vector.

This study phase was started on 05 Jul 2021 and completed on 14 Oct 2021.

3. MATERIALS AND METHODS

3.1. Experimental Design

Experimental procedures applicable to clinical pathology are summarized in Text Table 1.

Text Table 1
Experimental Design

Group No.	Test Material	Dose Level (vg)	Dose Volume (mL)	Dose Concentration (vg/mL)	No. of Animals Main Study	
					Males	Females
1	Reference Item	0	1	0	1	1
2	AAV9/AP4M1	8.4×10^{13}	1.55	5.43×10^{13}	-	2
3	AAV9/AP4M1	1.68×10^{14}	3.10	5.43×10^{13}	1	1

Text Table 2
Clinical Pathology Evaluation

Group Nos.	Occasion/ Time Point	Hematology	Coagulation	Clinical Chemistry	Urinalysis
All animals	Pretreatment	X	X	X	X
1 to 3	Day 2	X	X	X	X
1 to 3	Week 1	X	X	X	X
1 to 3	Week 2	-	-	X	-
1 to 3	Week 3	-	-	X	-
1 to 3	Week 4	X	X	X	X
1 to 3	Week 8	X	X	X	X
1 to 3	End of recovery	X	X	X	X

X = Sample collected; - = Not applicable;

3.2. Computerized Systems

Critical computerized systems used in this study phase are listed in Text Table 3.

Text Table 3
Computerized Systems

System Name	Version No.	Description of Data Collected and/or Analyzed
Provantis®	10	Hematology, coagulation, clinical chemistry and urinalysis.<br>Analysis of numerical in-life data
Advia Multispecies System	6.9.0 MS	Hematology parameters
Cobas 6000 c501 Analyzer	06-01	Clinical chemistry parameters (serum)
STA Compact Stago Analyzer	108.06	Coagulation parameters
Clinitek NOVUS	1.3.3	Urinalysis parameters
M-Files®	21	Reporting and collection of 21 CFR Part 11 compliant signature

4. RESULTS AND DISCUSSIONS

For the purpose of this report, treated animals' values were compared to baseline values and control values. Fold change (x) in clinical pathology parameters were determined by comparing the AAV9/AP4M1 individual value to the respective baseline individual value unless otherwise noted.

4.1. Hematology

(Appendix 1)

No AAV9/AP4M1-related hematology changes were noted at any dose level.

On Day 24 only, one female administered AAV9/AP4M1 at $8.4x10^{13}$ vg (No. 2702) presented mild increases in neutrophil (1.56x, individual value compared to respective Day-8 pretreatment) and monocyte (3.77x) counts, correlating with changes in coagulation and clinical chemistry parameters. Similar changes in neutrophil and monocyte counts were observed in other animals including one control female (No. 1701) at other timepoints.

All differences in hematology parameters were not considered AAV9/AP4M1-related based on their small magnitude, inconsistent direction, absence of a dose response, general overlap of individual values with the range of control and baseline values, and/or were of a magnitude of change commonly observed in monkeys under similar study conditions.

4.2. Coagulation

(Appendix 2)

On Day 24 only, one female administered AAV9/AP4M1 at $8.4x10^{13}$ vg (No. 2702) presented a transient mild increase in fibrinogen concentration (2.11x individual value compared to Day -3 pretreatment), correlating with changes in hematology and clinical chemistry parameters.

A gradual increase in fibrinogen concentration was observed in all individual animals, including control animals between Day 2 and Day 94 compared to their respective Day -8 (Day -3 for Animal No. 1201) pretreatment values. On Days 52 and 94, male and female administered AAV9/AP4M1 at $1.68x10^{14}$ vg (Nos. 3201 and 3701) presented mild increases in fibrinogen

concentration (1.82x to 2.55x, individual values compared to respective pretreatment) that were of higher magnitude than the increases observed in control animals (1.15x to 1.47x, individual values compared to pretreatment). These increases were not associated with relevant changes in globulins or leukocytes. They occurred several weeks after the administration of AAV9/AP4M1 and may have been associated with the microscopic necrosis/inflammation observed in the biceps femoris at injection sites, they remain of an uncertain relationship to AAV9/AP4M1.

Remaining differences in coagulation parameters were not considered AAV9/AP4M1-related based on their small magnitude, inconsistent direction, absence of a dose response, general overlap of individual values with the range of control and baseline values, and/or were of a magnitude of change commonly observed in monkeys under similar study conditions.

4.3. Clinical Chemistry

(Appendix 3)

No AAV9/AP4M1-related clinical chemistry changes were noted at any dose level.

On Day 24 only, one female administered AAV9/AP4M1 at 8.4×10^{13} vg (No. 2702) presented transient mild increases in globulins (1.32x, individual values compared to Day -8 pretreatment), triglycerides (4.31x), and decreases in albumin (0.81x) and albumin/globulin ratio (0.57x). These changes correlated with increases in neutrophil and monocyte counts, and an increase in fibrinogen concentration. They were supportive of inflammation and were possibly related to a moderate skin lesion of the left hindlimb observed on Day 28 in this animal. These changes were not observed on Day 52 or Day 94, after resolution of the skin lesion. This individual had no particular microscopic findings observed at the end of the study; hence these changes were unlikely AAV9/AP4M1-related.

Remaining differences in clinical chemistry parameters were not considered AAV9/AP4M1-related based on their small magnitude, inconsistent direction, absence of a dose response, general overlap of individual values with the range of control and baseline values, and/or were of a magnitude of change commonly observed in monkeys under similar study conditions.

4.4. Urinalysis

(Appendix 4)

No AAV9/AP4M1-related urinalysis changes were noted at any dose level.

All differences in urinalysis parameters were not considered AAV9/AP4M1-related based on their small magnitude, inconsistent direction, absence of a dose response, general overlap of individual values with the range of control and baseline values, and/or were of a magnitude of change commonly observed in monkeys under similar study conditions.

5. CONCLUSIONS

Administration of AAV9/AP4M1 to monkeys when given by a single intrathecal injection at $8.4x10^{13}$ or $1.68x10^{14}$ vg elicited equivocal mild increases in fibrinogen concentration in male and female administered AAV9/AP4M1 at $1.68x10^{14}$ vg on Days 52 and 94. These increases may have been associated with microscopic necrosis/inflammation observed in the biceps femoris at injection sites and remain of an uncertain relationship to AAV9/AP4M1. No AAV9/AP4M1-related hematology, clinical chemistry or urinalysis changes were noted at any dose level.

On Day 24 only, one female administered AAV9/AP4M1 at $8.4x10^{13}$ vg presented transient mild increases in neutrophil and monocyte counts, fibrinogen concentration, globulins and triglycerides, and mild decreases in albumin and albumin/globulin ratio. These changes were supportive of inflammation and were possibly related to a moderate skin lesion of the left hindlimb observed on Day 28 in this animal. Changes were not observed on Day 52 or Day 94, after resolution of the skin lesion. This individual had no particular microscopic findings observed at the end of the study; hence these changes were unlikely AAV9/AP4M1-related.

6. REPORT APPROVAL

All electronic signatures appear at the end of the document upon finalization.

Appendix 10

Individual Clinical Pathology Explanation Page

Hematology

ADVIA Analyzer
Analyzed Parameter Descriptions

Parameter	Abbreviation	Units	Methodology
Hematocrit	HCT	%	Calculated
Hemoglobin	HGB	g/dL	Colorimetric
Mean Corpuscular Hemoglobin	MCH	pg	Calculated
Mean Corpuscular Hemoglobin Concentration	MCHC	g/dL	Calculated
Mean Corpuscular Volume	MCV	fL	Calculated
Mean Platelet Volume	MPV	fL	Calculated
Platelet Distribution Width	PDW	%	Calculated
Platelet Count	PLT	$x10^3/\mu L$	Light scatter
Red Blood Cell Count	RBC	$x10^6/\mu L$	Light scatter
Red Blood Cell Distribution Width	RDW	%	Calculated
Reticulocytes	RETIC	$x10^9/L$	Calculated
Reticulocytes Percent	RETIC	%	Light scatter
White Blood Cell Count	WBC	$x10^3/\mu L$	Light scatter
White Blood Cell Differential Count			
Neutrophils Percent	NEUT	%	Light scatter
Lymphocytes Percent	LYMPH	%	Light scatter
Monocytes Percent	MONO	%	Light scatter
Eosinophils Percent	EOS	%	Light scatter
Basophils Percent	BASO	%	Light scatter
Large Unstained Cells Percent	LUC	%	Light scatter
Neutrophils	NEUT	$x10^3/\mu L$	Calculated
Lymphocytes	LYMPH	$x10^3/\mu L$	Calculated
Monocytes	MONO	$x10^3/\mu L$	Calculated
Eosinophils	EOS	$x10^3/\mu L$	Calculated
Basophils	BASO	$x10^3/\mu L$	Calculated
Large Unstained Cells	LUC	$x10^3/\mu L$	Calculated

Manual and Visual
Analyzed Parameter Descriptions

Parameter	Abbreviation	Units	Methodology
White Blood Cell Differential Count		% and/or $x10^3/\mu L$	Microscopic enumeration (100 white cells)
- Immature Neutrophils Count	IMM NEUT		
- Immature Neutrophils Percent	IMM NEUT		
- Immature Cells Percent	IMM CELL		
- Immature Cells Count	IMM CELL		
- Large Platelets	LPLT		
- Neutrophils Band Form	NEUT BAND		

Appendix 10

- Neutrophils Band Form Percent	NEUT BAND		
- Packed Cell Volume	PCV		
- Neutrophils	NEUT		
- Lymphocytes	LYMPH		
- Monocytes	MONO		
- Eosinophils	EOS		
- Basophils	BASO		
Others			
Bone Marrow Stain		None	Manual, Wright-Giemsa stain
Bone Marrow Slide Fixation		None	Manual, Fixative
- Nucleated Red Blood Cells/100 Leukocytes	RBCNUCLE	#/100 WBC	Microscopic enumeration (100 white cells) Reported as Number but not included in WBC Differential
CELL MORPHOLOGY			
- Cytoplasmic Basophilia Neutrophil	CYTO BASO NEUT	1+ (Minimal) 2+ (Mild)	Microscopic Examination
- Polychromasia	POLY	3+ (Moderate)	
- Anisocytosis	ANISO	4+ (Marked)	
- Hypochromasia	HYPO		
- Reactive Lymphocytes	REACTIVE LYMPH		
- Megakaryocytes	MEGAK		
- Smudge Cells	SMUDGE CELL		
- Microcytes	MICROCYTES		
- Macrocytes	MACROCYTES		
- Poikilocytosis	POIK		
- Rouleaux Formation	ROULEAUX		
- Agglutination	AGGL		
- Red Blood Cell Clumping	RBC Clumping		
- Acanthocytes	ACAN		
- Codocytes	CODO		
- Dacryocytes	DACR		
- Platelet Clumps	PLATELET CLUMPS		
- Eccentrocytes	ECCENTCY		
- Schistocytes	SCHZ		
- Spherocytes	SPHR		
- Stomatocytes	STOM		
- Howell Jolly Bodies	HJB		
- Basophilic Stippling	BASO STIP		
- Echinocytes	ECHINO		
- Vacuolated Neutrophils	VAC NEUT		
- Vacuolated Lymphocyted	VAC LYM		
- Döhle Bodies	DOHLE BODY		
- Degenerated Cells	DEG CELL		
- Ovalocytes	OVAL		
- Large Platelets Alpha	LARGE PLATELETS		

- Immature Neutrophils Morphology	IMM NEUT MORPH		
- Heinz Bodies	HEINZ BODY		
- Plasmodium	PLASMOD		
- Kurloff Cell	KURL		
- Burr Cells	BURR		
- Neutrophils Band Form Morphology	NEUT BAND MORPH		
- Nuclear Swelling	NUC SWELL NEUT		
- Red Blood Cell Morphology	RBC MORPH		
- White Blood Cell Morphology	WBC MORPH		
- Toxic Granulation	TOXG		
- Platelet Morphology	PLT MORPH		
Heinz Bodies Percent	HEINZ BODY	%	Microscopic examination. Methyl violet in physiological saline
Reticulocyte Percent	RETIC	%	Microscopic enumeration, new methylene blue stain

Aerospray Automated Slide Stainer
Analyzed Parameter Descriptions

Parameter	Abbreviation	Units	Methodology
White Blood Cell Differential Stain		None	2 parts aqueous stain (Eosin-Thiazin)

Midas III Slide Stainer
Analyzed Parameter Descriptions

Parameter	Abbreviation	Units	Methodology
White Blood Cell Differential Stain		None	Wright-Giemsa stain
Bone Marrow Stain		None	Wright-Giemsa stain
Bone Marrow Slide Fixation		None	Fixative

Coagulation

START 4 Compact Stago Analyzer
Analyzed Parameter Descriptions

Parameter	Abbreviation	Units	Methodology
Activated Partial Thromboplastin Time	APTT	sec	Viscosity
Fibrinogen	FIB	mg/dL	Viscosity
Prothrombin Time	PT	sec	Viscosity

STA Compact Stago Analyzer
Analyzed Parameter Descriptions

Parameter	Abbreviation	Units	Methodology
Activated Partial Thromboplastin Time	APTT	sec	Viscosity
Fibrinogen	FIB	mg/dL	Viscosity

Prothrombin Time	PT	sec	Viscosity

Plasma Appearance
(Reported as SAMQ Coagulation)

Analyzed Parameter Descriptions

Parameter	Abbreviation	Degree is graded as	Methodology
Normal sample	N	Normal	Manual and visual
Hemolyzed sample	H	+ = slight (pale/light red)<br>++ = moderate (red)<br>+++ = severe (dark red)	Manual and visual
Lipemic sample	L	+ = slight (cloudy)<br>++ = moderate (turbid)<br>+++ = severe (lactescent)	Manual and visual
Icterus sample	I	+ = slight (dark yellow)<br>++ = moderate (very dark yellow)<br>+++ = severe (dark yellow-green)	Manual and visual

Clinical Chemistry

Cobas 6000 Analyzer

Analyzed Parameter Descriptions

Parameter	Abbreviation	Units	Methodology
Alanine Aminotransferase	ALT	U/L	ALT IFCC UV
Albumin	ALB	g/dL	Bromcresol green colorimetric
Alkaline Phosphatase	ALP	U/L	ALP IFCC liquid colorimetric
Amylase	AMYL	U/L	Enzymatic colorimetric
Aspartate Aminotransferase	AST	U/L	AST IFCC UV
Calcium (Alternate)	CA	mg/dL	O-cresolphthalein complexone colorimetric
Cholesterol	CHOL	mg/dL	CHOD-PAP enzymatic colorimetric
Creatinine	CREAT	mg/dL	Jaffe kinetic colorimetric. Rate-blanked and compensated
Creatine Kinase	CK	U/L	NAC activated UV
C-Reactive Protein	CRP	mg/L	Immunoturbidimetric
C-Reactive Protein High Sensitivity	CRP HS	mg/L	Immunoturbidimetric
Direct Bilirubin	DBIL	mg/dL	Jendrassik colorimetric
GAMMA-Glutamyl Transferase	GGT	U/L	Nitro-Anilide, Glycylglycine; enzymatic colorimetric
Glutamate Dehydrogenase	GLDH	U/L	Kinetic UV
Glucose	GLUC	mg/dL	Hexokinase UV
Iron	FE	µg/dL	Colorimetric
Lactate	LACT	mg/dL	Enzymatic colorimetric
Magnesium	MG	mg/dL	Colorimetric
Phosphorus	PHOS	mg/dL	Molybdate UV
Sodium, Potassium, Chloride (SI)	NA,K,CL	mmol/L	Indirect measurement (Ion selective electrode)
Total Bilirubin	TBIL	mg/dL	DPD colorimetric
Total Protein	TPROT	g/dL	Biuret colorimetric

Triglycerides	TRIG	mg/dL	GPO-PAP enzymatic colorimetric
Urea Nitrogen	UREAN	mg/dL	Urease kinetic UV

Calculations

Analyzed Parameter Descriptions

Parameter	Abbreviation	Units	Calculation
Albumin/Globulin ratio	A/G	ratio	Albumin / Globulin
Globulin	GLOB	g/dL	Total Protein - Albumin
Indirect Bilirubin	IBIL	mg/dL	Total Bilirubin - Direct Bilirubin
Urea Nitrogen / Creatinine ratio	UREAN/CREAT	None	Urea Nitrogen / Creatinine

Serum Appearance (Reported as SAMQ)

Analyzed Parameter Descriptions

Parameter	Abbreviation	Key to Results (Code)	Methodology
Normal sample	N	Normal	Manual and visual
Hemolyzed sample	H	+ = slight (pale/light red)<br>++ = moderate (red)<br>+++ = severe (dark red)	Manual and visual
Lipemic sample	L	+ = slight (cloudy)<br>++ = moderate (turbid)<br>+++ = severe (lactescent)	Manual and visual
Icterus sample	I	+ = slight (dark yellow)<br>++ = moderate (very dark yellow)<br>+++ = severe (dark yellow-green)	Manual and visual

Urinalysis

Clinitek

Analyzed Parameter Descriptions

Parameter	Abbreviation	Key to Results (Code)<br>Macroscopic Urinalysis	Methodology
Urine Bilirubin	BIL	Negative (Neg), 1+ (small), 2+ (Moderate), 3+ (Large)	Automated reflectance spectrophotometer
Urine Blood	BLD	Negative (Neg), 1+ (Trace), 2+ (Small), 3+ (Moderate), 4+ (Large)	Automated reflectance spectrophotometer
Urine Color	COLOR	Co = Colorless, LY = Light Yellow, DY = Dark Yellow, Or = Orange, Re= Red, Br = Brown, Gr = Green, Ot = Other (color is identified)	Automated reflectance spectrophotometer
Urine Clarity	CLARITY	Clr = Clear, Cld = Cloudy, Tur= Turbid	Automated reflectance spectrophotometer
Urine Glucose Alpha	GLUC	Negative (Neg), 1+ (5.5 mmol/L), 2+ (14 mmol/L) ,3+ (28 mmol/L), 4+ (≥55 mmol/L)	Automated reflectance spectrophotometer
Urine Ketones	KET	Negative (Neg), 1+ (Trace), 2+ (1.5 mmol/L), 3+ (3.9 mmol /L), 4+ (≥ 7.8	Automated reflectance spectrophotometer

		mmol /L)	
Urine Nitrite	NIT	Negative (Neg) (-), Positive (Pos) (+)	Automated reflectance spectrophotometer
Urine pH	URINE pH	4.5, 5.0, 5.5, 6.0, 6.5, 7.0, 7.5, 8.0, 8.5, 9.0, ≥ 9.0	Automated reflectance spectrophotometer
Urine Protein Alpha	PROT	Negative (Neg), 1+ (Trace), 2+ (0.3g/L), 3+ (1.0 g/L), 4+ (≥3.0 g/L)	Automated reflectance spectrophotometer
Urine Specific Gravity	SPECIFIC GRAVITY	1.000 to 1.099 (>=1.099)	Automated reflectance spectrophotometer
Urine Urobilinogen	UROBIL	Negative (Neg), 1+ (16µmol/L), 2+ (33 µmol/L), 3+ (66 µmol/L), 4+ (≥ 131 µmol/L)	Automated reflectance spectrophotometer

Manual and Visual

Analyzed Parameter Descriptions

Parameter	Abbreviation	Key to Results (Code)/Unit	Methodology
Urine Bilirubin	BIL	Negative (Neg), 1+ (small), 2+ (Moderate), 3+ (Large)	Manual Results – Multistix strip
Urine Blood	BLD	Negative (Neg), 1+ (Trace), 2+ (Small), 3+ (Moderate), 4+ (Large)	Manual Results – Multistix strip
Urine Color	COLOR	Co = Colorless, LY = Light Yellow, DY = Dark Yellow, Or = Orange, Re= Red, Br = Brown, Gr = Green, Ot = Other (color is identified)	Manual and visual
Urine Clarity	CLARITY	Clr = Clear, Cld = Cloudy, Tur= Turbid	Manual and visual
Urine Glucose Alpha	GLUC	Negative (Neg), 1+ (5.5 mmol/L), 2+ (14 mmol/L) 3+ (28 mmol/L), 4+ (55 mmol/L or ≥ 111 mmol/L)	Manual Results – Multistix strip
Urine Ketones	KET	Negative (Neg), 1+ (0.5 mmol/L), 2+ (1.5 mmol/L), 3+ (4.0 mmol /L), 4+ (8 mmol /L or 16 mmol/L)	Manual Results – Multistix strip
Urine Nitrite	NIT	Negative (Neg) (-), Positive (Pos) (+)	Manual Results – Multistix strip
Urine pH	URINE pH	4.5, 5.0, 5.5, 6.0, 6.5, 7.0, 7.5, 8.0, 8.5	Manual Results – Multistix strip
Urine Protein Alpha	PROT	Negative (Neg), 1+ (Trace), 2+ (0.3g/L), 3+ (1.0 g/L), 4+ (≥3.0 g/L or ≥ 20.0 g/L)	Manual Results – Multistix strip
Urine Specific Gravity	SPECIFIC GRAVITY	None	Manual Results – Refractive index using the Refractometer
Urine Urobilinogen	UROBIL	Neg (3.2 µmol/L), 1+ (16 µmol/L), 2+ (33 µmol/L), 3+ (66 µmol/L), 4+ (131 µmol/L)	Manual Results – Multistix strip
Urine Volume	VOLUME	mL	Manual and visual

Appendix 10

Other Abbreviations

Abbreviation	Description	Abbreviation	Description
./-	Not required for veterinary monitoring / Not scheduled to be performed / No findings / Not evaluated / Dead	NT	Not taken
ADQ	Adequate	OA	Omitted activity
CLOT	Sample clotted	QNS	Quantity not sufficient
COM	Contaminated with organic material	RC	Result comment
COMM	Comment added	SC	Sample comment
DEC	Decreased	SNR	Sample not received
FC	Flag comment	TNR	Test not reported
GFC	Gross fecal contamination present	TTSM	Sample was analyzed 3 times (original, 1st and 2nd repeats), values not comparable (not reported)
INC	Increased	U/#	U/collection period
LIF	Laboratory Investigation Form	Unsc	Unscheduled
LLOQ	Less than lower limit of quantitation	UPTD	Unable to perform due to technical difficulty
MDIFF	Manual differential	UTD	Unable to determine
mg/#	mg/collection period	UTDH	Unable to determine due to marked hemolysis
NAF	No abnormal findings	UTDL	Unable to determine due to marked lipemia
NC	Not calculable	UTDM	Unable to determine, not confirmed by microscopy
NCD	No clot detected	UTDR	Unable to determine, results not reproducible
NSCH	Not scheduled to be performed	Vet	Collection for veterinary monitoring

Note: This is a comprehensive list of systems, parameters and/or abbreviations. Everything listed above may not be applicable to this report.

Note: Additional morphology for flagged samples may be reported if applicable.

Dosing Information

Dosing information is abbreviated on various data outputs; the following represents the dosing information for this study.

Group No.	Test Material	Dose Level (vg)
1	Reference Item	0
2	AAV9/AP4M1	$8.4x10^{13}$
3	AAV9/AP4M1	$1.68x10^{14}$

Appendix 10

Appendix 1

Individual Hematology Values

5550014

Sex: Male

0<br>vg<br>Group 1	Day(s) Relative to Start Date	Reporting Hematology<br>WBC (10^3/uL)	NEUT (10^3/uL)	LYMPH (10^3/uL)	MONO (10^3/uL)
1201	-8	12.31	8.30	3.22	0.58
	2	8.92	5.55	2.93	0.38
	6	7.36	3.68	3.24	0.29
	24	9.89	6.18	2.94	0.66
	52	11.27	6.27	4.17	0.70
	94	11.06	6.28	4.16	0.50

Appendix 10

Appendix 1

Individual Hematology Values

5550014

Sex: Male

0<br>vg<br>Group 1	Day(s) Relative to Start Date	Reporting Hematology<br>EOS (10^3/uL)	BASO (10^3/uL)	LUC (10^3/uL)	RBC (10^6/uL)
1201	-8	0.11	0.06	0.04	6.27
	2	0.03	0.01	0.02	5.95
	6	0.15	0.00	MDIFF	5.81
	24	0.06	0.02	0.03	5.95
	52	0.06	0.04	0.04	6.31
	94	0.04	0.03	0.04	6.85

Appendix 10

Appendix 1

Individual Hematology Values

5550014

Sex: Male

0<br>vg<br>Group 1	Day(s) Relative to Start Date	Reporting Hematology<br>HGB (g/dL)	HCT (%)	MCV (fL)	MCH (pg)
1201	-8	14.5	45.7	72.8	23.1
	2	13.8	45.0	75.6	23.1
	6	13.4	43.9	75.5	23.0
	24	13.3	42.2	71.0	22.3
	52	14.2	45.8	72.6	22.4
	94	14.8	49.6	72.5	21.6

Appendix 10

Appendix 1

Individual Hematology Values

5550014

Sex: Male

0<br>vg<br>Group 1	Day(s) Relative to Start Date	Reporting Hematology<br>MCHC (g/dL)	RDW (%)	PLT (10^3/uL)	RETIC (10^9/L)
1201	-8	31.7	12.6	305	49.2
	2	30.5	12.0	603	52.4
	6	30.5	11.3	492	86.0
	24	31.5	11.2	382	83.2
	52	30.9	11.8	449	64.3
	94	29.9	11.8	391	76.1

Appendix 10

Appendix 1

Individual Hematology Values

5550014

Sex: Male

0<br>vg<br>Group 1	Day(s) Relative to Start Date	Reporting Hematology<br>ANISO	PLATELET CLUMPS	WBC MORPH
1201	-8	-	-	-
	2	-	-	-
	6	1+	4+	NAF
	24	-	-	-
	52	-	-	-
	94	-	-	-

Appendix 10

Appendix 1

Individual Hematology Values

5550014

Sex: Male

1.68 X10E14 vg Group 3	Day(s) Relative to Start Date	Reporting Hematology WBC (10^3/uL)	NEUT (10^3/uL)	LYMPH (10^3/uL)	MONO (10^3/uL)
3201	-8	13.03	4.78	7.72	0.17
	2	11.72	6.82	4.42	0.28
	6	10.82	4.23	5.92	0.32
	24	12.50	6.67	5.27	0.40
	52	14.37	8.37	5.36	0.46
	94	16.67	10.45	5.71	0.38

Appendix 10

Appendix 1

Individual Hematology Values

5550014

Sex: Male

1.68 X10E14 vg Group 3	Day(s) Relative to Start Date	Reporting Hematology EOS (10^3/uL)	BASO (10^3/uL)	LUC (10^3/uL)	RBC (10^6/uL)
3201	-8	0.26	0.07	0.04	5.93
	2	0.15	0.02	0.03	5.23
	6	0.24	0.04	0.07	5.34
	24	0.08	0.02	0.07	5.38
	52	0.05	0.07	0.06	5.22
	94	0.05	0.04	0.04	5.74

Appendix 10

Appendix 1

Individual Hematology Values

5550014

Sex: Male

1.68 X10E14 vg Group 3	Day(s) Relative to Start Date	Reporting Hematology HGB (g/dL)	HCT (%)	MCV (fL)	MCH (pg)
3201	-8	14.7	45.8	77.3	24.8
	2	13.1	40.8	77.9	25.0
	6	13.3	41.5	77.7	24.9
	24	13.0	41.0	76.1	24.2
	52	12.8	39.9	76.4	24.5
	94	13.8	42.9	74.8	24.0

Appendix 10

Appendix 1

Individual Hematology Values

5550014

Sex: Male

1.68 X10E14 vg Group 3	Day(s) Relative to Start Date	Reporting Hematology MCHC (g/dL)	RDW (%)	PLT (10^3/uL)	RETIC (10^9/L)
3201	-8	32.0	13.2	311	66.6
	2	32.2	12.4	309	57.6
	6	32.1	12.1	381	47.0
	24	31.8	11.9	309	41.5
	52	32.1	12.7	336	71.3
	94	32.1	12.2	332	73.1

Appendix 10

Appendix 1

Individual Hematology Values

5550014

Sex: Male

1.68 X10E14 vg Group 3	Day(s) Relative to Start Date	Reporting Hematology ANISO	PLATELET CLUMPS	WBC MORPH
3201	-8	-	-	-
	2	-	-	-
	6	-	-	-
	24	-	-	-
	52	-	-	-
	94	-	-	-

Appendix 10

Appendix 1

Individual Hematology Values

5550014

Sex: Female

0<br>vg<br>Group 1	Day(s) Relative to Start Date	Reporting Hematology<br>WBC (10^3/uL)	NEUT (10^3/uL)	LYMPH (10^3/uL)	MONO (10^3/uL)
1701	-8	12.60	6.04	5.96	0.33
	2	16.37	12.26	3.33	0.68
	6	11.33	6.43	4.08	0.52
	24	8.73	4.86	3.47	0.27
	52	21.63	14.83	5.30	1.10
	94	12.83	7.18	5.02	0.36

Appendix 10

Appendix 1

Individual Hematology Values

5550014

Sex: Female

0<br>vg<br>Group 1	Day(s) Relative to Start Date	Reporting Hematology<br>EOS (10^3/uL)	BASO (10^3/uL)	LUC (10^3/uL)	RBC (10^6/uL)
1701	-8	0.16	0.07	0.05	5.07
	2	0.04	0.02	0.04	4.34
	6	0.20	0.02	0.07	4.39
	24	0.10	0.01	0.02	5.01
	52	0.21	0.12	0.07	5.51
	94	0.22	0.02	0.02	5.48

Appendix 10

Appendix 1

Individual Hematology Values

5550014

Sex: Female

0<br>vg<br>Group 1	Day(s) Relative to Start Date	Reporting Hematology<br>HGB (g/dL)	HCT (%)	MCV (fL)	MCH (pg)
1701	-8	12.5	40.0	79.0	24.7
	2	10.8	33.4	77.0	24.8
	6	10.8	35.6	81.2	24.6
	24	12.4	40.0	79.8	24.7
	52	13.5	44.7	81.2	24.5
	94	12.9	43.7	79.7	23.6

Appendix 10

Appendix 1

Individual Hematology Values

5550014

Sex: Female

0 vg Group 1	Day(s) Relative to Start Date	Reporting Hematology MCHC (g/dL)	RDW (%)	PLT (10^3/uL)	RETIC (10^9/L)
1701	-8	31.3	12.9	484	81.5
	2	32.2	12.2	509	64.9
	6	30.3	11.9	574	110.2
	24	31.0	11.7	505	77.9
	52	30.2	11.9	455	76.2
	94	29.6	11.8	465	87.2

Appendix 10

Appendix 1

Individual Hematology Values

5550014

Sex: Female

0<br>vg<br>Group 1	Day(s) Relative to Start Date	Reporting Hematology<br>ANISO	 PLATELET CLUMPS	 WBC MORPH
1701	-8	-	-	-
	2	-	-	-
	6	-	-	-
	24	-	-	-
	52	-	-	-
	94	-	-	-

Appendix 10

Appendix 1

Individual Hematology Values

5550014

Sex: Female

8.4 X10E13 vg Group 2	Day(s) Relative to Start Date	Reporting Hematology WBC (10^3/uL)	NEUT (10^3/uL)	LYMPH (10^3/uL)	MONO (10^3/uL)
2701	-8	9.86	5.22	3.98	0.42
	2	13.64	9.08	3.73	0.69
	6	10.67	5.35	4.28	0.81
	24	11.05	6.46	3.88	0.52
	52	20.44	13.81	5.13	1.00
	94	18.73	11.11	6.38	0.85
2702	-8	13.72	9.72	3.50	0.35
	2	16.06	12.04	3.05	0.88
	6	13.75	9.53	3.37	0.67
	24	19.77	15.19	3.01	1.32
	52	16.71	9.97	5.86	0.71
	94	12.22	6.76	4.95	0.44

Appendix 10

Appendix 1

Individual Hematology Values

5550014

Sex: Female

8.4 X10E13 vg Group 2	Day(s) Relative to Start Date	Reporting Hematology EOS (10^3/uL)	BASO (10^3/uL)	LUC (10^3/uL)	RBC (10^6/uL)
2701	-8	0.12	0.05	0.07	5.39
	2	0.05	0.02	0.07	4.67
	6	0.12	0.01	0.10	4.84
	24	0.14	0.01	0.05	5.11
	52	0.37	0.06	0.08	4.87
	94	0.27	0.04	0.08	5.68
2702	-8	0.04	0.05	0.06	5.16
	2	0.05	0.01	0.02	4.63
	6	0.05	0.02	0.12	4.76
	24	0.02	0.01	0.23	4.29
	52	0.08	0.05	0.04	4.94
	94	0.03	0.03	0.03	5.61

Appendix 10

Appendix 1

Individual Hematology Values

5550014

Sex: Female

8.4 X10E13 vg Group 2	Day(s) Relative to Start Date	Reporting Hematology HGB (g/dL)	HCT (%)	MCV (fL)	MCH (pg)
2701	-8	12.8	40.6	75.4	23.8
	2	11.2	35.1	75.2	24.0
	6	11.6	37.1	76.5	24.0
	24	11.9	37.6	73.7	23.4
	52	12.1	34.3	70.4	24.9
	94	12.9	40.5	71.3	22.8
2702	-8	12.4	41.1	79.7	24.0
	2	11.3	36.3	78.4	24.5
	6	11.6	38.0	79.7	24.3
	24	9.9	34.3	79.9	23.2
	52	11.8	38.1	77.1	23.9
	94	13.1	42.7	76.2	23.3

Appendix 10

Appendix 1

Individual Hematology Values

5550014

Sex: Female

8.4 X10E13 vg Group 2	Day(s) Relative to Start Date	Reporting Hematology MCHC (g/dL)	RDW (%)	PLT (10^3/uL)	RETIC (10^9/L)
2701	-8	31.6	11.9	401	64.6
	2	31.9	10.9	448	59.7
	6	31.3	10.8	561	113.6
	24	31.7	10.8	446	52.9
	52	35.3	11.4	401	59.4
	94	31.9	10.8	410	42.8
2702	-8	30.1	13.0	350	54.8
	2	31.3	11.9	373	51.8
	6	30.4	11.9	459	80.8
	24	29.0	11.4	409	77.1
	52	31.0	13.5	375	92.4
	94	30.6	13.0	367	73.4

Appendix 10

Appendix 1

Individual Hematology Values

5550014

Sex: Female

8.4 X10E13 vg Group 2	Day(s) Relative to Start Date	Reporting Hematology ANISO	PLATELET CLUMPS	WBC MORPH
2701	-8	-	-	-
	2	-	-	-
	6	-	-	-
	24	-	-	-
	52	-	-	-
	94	-	-	-
2702	-8	-	-	-
	2	-	-	-
	6	-	-	-
	24	-	-	-
	52	-	-	-
	94	-	-	-

Appendix 10

Appendix 1

Individual Hematology Values

5550014

Sex: Female

1.68 X10E14 vg Group 3	Day(s) Relative to Start Date	Reporting Hematology WBC (10^3/uL)	NEUT (10^3/uL)	LYMPH (10^3/uL)	MONO (10^3/uL)
3701	-8	14.84	9.42	4.85	0.26
	2	17.08	12.92	3.52	0.43
	6	18.00	11.27	5.71	0.57
	24	12.71	8.54	3.72	0.32
	52	21.57	15.94	4.87	0.47
	94	18.86	13.17	4.73	0.57

Appendix 10

Appendix 1

Individual Hematology Values

5550014

Sex: Female

1.68 X10E14 vg Group 3	Day(s) Relative to Start Date	Reporting Hematology EOS (10^3/uL)	BASO (10^3/uL)	LUC (10^3/uL)	RBC (10^6/uL)
3701	-8	0.19	0.08	0.04	5.14
	2	0.15	0.01	0.04	4.68
	6	0.33	0.05	0.07	4.55
	24	0.08	0.00	0.04	4.72
	52	0.20	0.04	0.03	4.61
	94	0.32	0.02	0.04	5.32

Appendix 10

Appendix 1

Individual Hematology Values

5550014

Sex: Female

1.68 X10E14 vg Group 3	Day(s) Relative to Start Date	Reporting Hematology HGB (g/dL)	HCT (%)	MCV (fL)	MCH (pg)
3701	-8	12.8	40.6	79.0	24.8
	2	11.7	36.2	77.3	25.1
	6	11.0	35.6	78.2	24.2
	24	11.6	37.3	79.1	24.6
	52	11.4	37.2	80.6	24.6
	94	12.5	41.8	78.5	23.5

Appendix 10

Appendix 1

Individual Hematology Values

5550014

Sex: Female

1.68 X10E14 vg Group 3	Day(s) Relative to Start Date	Reporting Hematology MCHC (g/dL)	RDW (%)	PLT (10^3/uL)	RETIC (10^9/L)
3701	-8	31.4	13.7	297	47.7
	2	32.4	13.2	417	44.6
	6	30.9	13.1	437	90.5
	24	31.1	13.1	379	76.1
	52	30.6	13.0	387	84.4
	94	29.9	13.3	330	73.2

Appendix 10

Appendix 1

Individual Hematology Values

5550014

Sex: Female

1.68 X10E14 vg Group 3	Day(s) Relative to Start Date	Reporting Hematology ANISO	PLATELET CLUMPS	WBC MORPH
3701	-8	-	-	-
	2	-	-	-
	6	-	-	-
	24	-	-	-
	52	-	-	-
	94	-	-	-

Appendix 10

Appendix 2

Individual Coagulation Values

5550014

Sex: Male

0<br>vg<br>Group 1	Day(s) Relative to Start Date	Reporting Coagulation<br>PT (sec)	APTT (sec)	FIB (mg/dL)	SAMQ Coagulation
1201	-3	10.9	22.7	327	L+
	2	11.3	20.2	373	N
	7	10.5	21.0	379	N
	24	10.4	23.0	344	N
	52	10.7	21.9	398	N
	94	10.5	21.0	481	N

Appendix 10

Appendix 2

Individual Coagulation Values

5550014

Sex: Male

1.68 X10E14 vg Group 3	Day(s) Relative to Start Date	Reporting Coagulation PT (sec)	APTT (sec)	FIB (mg/dL)	SAMQ Coagulation
3201	-8	11.1	21.6	232	N
	2	11.5	19.3	370	N
	6	10.9	20.5	368	N
	24	10.1	20.8	420	N
	52	10.7	20.1	484	N
	94	10.7	19.4	592	L+

Appendix 10

Appendix 2

Individual Coagulation Values

5550014

Sex: Female

0<br>vg<br>Group 1	Day(s) Relative to Start Date	Reporting Coagulation<br>PT (sec)	APTT (sec)	FIB (mg/dL)	SAMQ Coagulation
1701	-8	11.2	23.0	316	N
	2	12.1	22.1	373	N
	6	11.4	22.4	439	N
	24	10.9	22.7	310	N
	52	10.7	21.4	362	N
	94	11.0	21.9	409	N

Appendix 10

Appendix 2

Individual Coagulation Values

5550014

Sex: Female

8.4 X10E13 vg Group 2	Day(s) Relative to Start Date	Reporting Coagulation PT (sec)	APTT (sec)	FIB (mg/dL)	SAMQ Coagulation
2701	-8	10.8	19.1	312	N
	2	11.6	18.3	386	N
	6	11.1	19.2	420	N
	24	10.5	19.4	300	N
	52	11.2	19.5	344	N
	94	10.9	17.9	468	H+
2702	-3	10.7	21.2	389	N
	2	11.6	21.0	461	N
	6	11.4	21.6	439	N
	24	11.6	23.7	819	N
	53	10.5	20.2	402	L++
	94	10.5	20.5	427	N

Appendix 10

Appendix 2

Individual Coagulation Values

5550014

Sex: Female

1.68 X10E14 vg Group 3	Day(s) Relative to Start Date	Reporting Coagulation PT (sec)	APTT (sec)	FIB (mg/dL)	SAMQ Coagulation
3701	-8	11.4	21.8	280	N
	2	11.7	20.7	402	N
	6	11.6	23.0	461	N
	24	11.5	24.2	443	N
	52	12.0	22.3	510	N
	94	11.1	21.5	614	L+

Appendix 10

Appendix 3

Individual Clinical Chemistry Values

5550014

Sex: Male

0 vg Group 1	Day(s) Relative to Start Date	Reporting Biochemistry AST (U/L)	ALT (U/L)	ALP (U/L)	GGT (U/L)	CK (U/L)	TBIL (mg/dL)
1201	-8	54	67	444	88	715	0.15
	2	94	95	469	98	3197	0.20
	6	68	93	411	90	1691	0.20
	10	77	83	385	86	1662	0.10
	17	57	77	402	86	762	0.17
	24	41	67	339	73	444	0.10
	52	43	68	275	68	577	0.10
	94	53	60	248	71	2625	0.09

Appendix 10

Appendix 3

Individual Clinical Chemistry Values

5550014

Sex: Male

0<br>vg<br>Group 1	Day(s) Relative to Start Date	Reporting Biochemistry<br>UREAN (mg/dL)	CREAT (mg/dL)	GLUC (mg/dL)	CHOL (mg/dL)	TRIG (mg/dL)	TPROT (g/dL)
1201	-8	23	0.6	96	143	64	7.2
	2	19	0.9	147	136	28	7.8
	6	20	0.8	125	150	48	7.6
	10	24	0.6	99	148	51	7.9
	17	18	0.7	103	169	34	7.7
	24	19	0.6	100	131	85	7.4
	52	17	0.7	118	129	93	7.7
	94	16	0.9	190	135	60	8.3

Individual Clinical Chemistry Values

5550014
Sex: Male

0<br>vg<br>Group 1	Day(s) Relative to Start Date	Reporting Biochemistry ALB (g/dL)	GLOB (g/dL)	A/G (ratio)	CA (mg/dL)	PHOS (mg/dL)	NA (mmol/L)
1201	-8	4.5	2.7	1.7	9.0	4.7	145
	2	4.5	3.3	1.4	9.4	5.8	153
	6	4.6	3.0	1.5	9.5	5.7	151
	10	4.6	3.3	1.4	9.8	6.1	147
	17	4.7	3.0	1.6	9.8	6.1	145
	24	4.4	3.0	1.5	9.3	5.2	146
	52	4.7	3.0	1.6	9.5	5.5	150
	94	4.8	3.5	1.4	9.9	5.7	150

Appendix 10

Appendix 3

Individual Clinical Chemistry Values

5550014

Sex: Male

0<br>vg<br>Group 1	Day(s) Relative to Start Date	Reporting Biochemistry<br>K<br>(mmol/L)	CL<br>(mmol/L)	SAMQ
1201	-8	4.9	103	N
	2	5.8	104	H+
	6	5.0	104	N
	10	6.0	99	N
	17	4.9	100	N
	24	4.2	102	N
	52	4.5	98	N
	94	4.5	99	N

Individual Clinical Chemistry Values

5550014
Sex: Male

1.68 X10E14 vg Group 3	Day(s) Relative to Start Date	Reporting Biochemistry AST (U/L)	ALT (U/L)	ALP (U/L)	GGT (U/L)	CK (U/L)	TBIL (mg/dL)
3201	-8	63	61	607	98	955	0.15
	2	83	93	544	93	1490	0.14
	6	73	90	533	84	932	0.11
	10	52	67	463	75	277	0.08
	17	57	67	460	70	360	0.13
	24	44	54	394	59	245	0.05
	52	62	54	245	49	1259	0.11
	94	41	48	159	48	579	0.04

Appendix 10

Appendix 3

Individual Clinical Chemistry Values

5550014

Sex: Male

1.68 X10E14 vg Group 3	Day(s) Relative to Start Date	Reporting Biochemistry UREAN (mg/dL)	CREAT (mg/dL)	GLUC (mg/dL)	CHOL (mg/dL)	TRIG (mg/dL)	TPROT (g/dL)
3201	-8	24	0.6	71	112	60	7.1
	2	27	0.7	79	114	31	7.6
	6	23	0.6	76	130	71	7.1
	10	24	0.5	70	138	106	7.3
	17	21	0.6	67	163	50	7.5
	24	26	0.6	63	129	149	7.4
	52	23	0.7	69	131	107	7.6
	94	20	0.8	90	114	106	7.8

Appendix 10

Appendix 3

Individual Clinical Chemistry Values

5550014

Sex: Male

1.68 X10E14 vg Group 3	Day(s) Relative to Start Date	Reporting Biochemistry					
		ALB (g/dL)	GLOB (g/dL)	A/G (ratio)	CA (mg/dL)	PHOS (mg/dL)	NA (mmol/L)
3201	-8	4.5	2.6	1.7	9.0	5.8	144
	2	4.5	3.1	1.5	9.0	5.3	150
	6	4.5	2.6	1.7	9.5	6.2	148
	10	4.6	2.7	1.7	9.8	6.9	144
	17	4.7	2.8	1.7	9.9	6.6	149
	24	4.6	2.8	1.6	9.6	5.3	147
	52	4.6	3.0	1.5	9.7	5.2	147
	94	4.6	3.2	1.4	9.6	4.7	146

Appendix 10

Appendix 3

Individual Clinical Chemistry Values

5550014

Sex: Male

1.68 X10E14 vg Group 3	Day(s) Relative to Start Date	Reporting Biochemistry K (mmol/L)	CL (mmol/L)	SAMQ
3201	-8	4.4	106	N
	2	4.4	111	N
	6	4.6	109	N
	10	5.1	106	N
	17	4.8	104	N
	24	4.4	104	N
	52	4.5	103	N
	94	3.6	103	N

Appendix 10

Appendix 3

Individual Clinical Chemistry Values

5550014

Sex: Female

0 vg Group 1	Day(s) Relative to Start Date	Reporting Biochemistry AST (U/L)	ALT (U/L)	ALP (U/L)	GGT (U/L)	CK (U/L)	TBIL (mg/dL)
1701	-8	105	102	447	36	2177	0.06
	2	147	159	440	36	2806	0.07
	6	124	159	425	40	963	0.10
	10	123	180	437	55	1813	0.06
	17	69	136	477	64	375	0.10
	24	76	140	387	51	385	0.05
	52	131	120	312	34	1403	0.05
	94	52	84	251	34	432	0.08

Appendix 10

Appendix 3

Individual Clinical Chemistry Values

5550014

Sex: Female

0<br>vg<br>Group 1	Day(s) Relative to Start Date	Reporting Biochemistry<br>UREAN (mg/dL)	CREAT (mg/dL)	GLUC (mg/dL)	CHOL (mg/dL)	TRIG (mg/dL)	TPROT (g/dL)
1701	-8	20	0.7	105	138	48	6.8
	2	22	0.8	87	132	20	7.0
	6	22	0.7	80	143	48	6.7
	10	21	0.6	93	139	96	7.1
	17	24	0.7	74	171	37	7.5
	24	22	0.6	80	153	151	7.2
	52	26	0.7	93	133	104	7.1
	94	14	0.8	120	152	56	7.4

Appendix 10

Appendix 3

Individual Clinical Chemistry Values

5550014

Sex: Female

0 vg Group 1	Day(s) Relative to Start Date	Reporting Biochemistry ALB (g/dL)	GLOB (g/dL)	A/G (ratio)	CA (mg/dL)	PHOS (mg/dL)	NA (mmol/L)
1701	-8	4.1	2.7	1.5	9.1	5.0	145
	2	3.9	3.1	1.3	8.9	4.9	145
	6	3.9	2.8	1.4	9.2	5.1	146
	10	4.1	3.0	1.4	9.1	5.4	146
	17	4.3	3.2	1.3	9.4	5.6	147
	24	4.3	2.9	1.5	9.5	4.9	147
	52	4.2	2.9	1.4	9.5	3.8	149
	94	4.3	3.1	1.4	9.5	5.1	149

Appendix 10

Appendix 3

Individual Clinical Chemistry Values

5550014

Sex: Female

0<br>vg<br>Group 1	Day(s) Relative to Start Date	Reporting Biochemistry<br>K (mmol/L)	CL (mmol/L)	SAMQ
1701	-8	4.7	106	N
	2	4.6	109	H+
	6	4.6	106	N
	10	4.4	100	N
	17	4.8	104	N
	24	5.2	107	N
	52	4.5	104	N
	94	4.0	103	N

Appendix 10

Appendix 3

Individual Clinical Chemistry Values

5550014

Sex: Female

8.4 X10E13 vg Group 2	Day(s) Relative to Start Date	AST (U/L)	ALT (U/L)	ALP (U/L)	GGT (U/L)	CK (U/L)	TBIL (mg/dL)
2701	-8	68	96	250	57	923	0.13
	2	116	156	305	60	995	0.12
	6	50	108	279	63	280	0.09
	10	58	93	262	60	345	0.10
	17	40	64	251	60	369	0.11
	24	40	71	204	58	184	0.06
	52	50	84	181	58	450	0.06
	94	50	62	172	51	673	0.05
2702	-8	60	96	252	45	529	0.14
	2	153	166	292	45	2369	0.11
	6	53	118	369	45	804	0.10
	10	46	97	372	44	273	0.04
	17	36	64	553	43	215	0.08
	24	35	49	746	41	263	0.04
	52	40	76	289	38	600	0.03
	94	42	64	156	38	744	0.06

Appendix 10

Appendix 3

Individual Clinical Chemistry Values

5550014

Sex: Female

8.4 X10E13 vg Group 2	Day(s) Relative to Start Date	UREAN (mg/dL)	CREAT (mg/dL)	GLUC (mg/dL)	CHOL (mg/dL)	TRIG (mg/dL)	TPROT (g/dL)
		Reporting Biochemistry					
2701	-8	21	0.6	89	160	30	7.2
	2	17	0.8	90	138	25	7.1
	6	21	0.6	75	155	40	7.2
	10	23	0.6	70	150	33	7.2
	17	21	0.6	71	173	29	7.2
	24	19	0.5	72	154	69	7.1
	52	17	0.5	82	144	57	6.9
	94	17	0.6	111	155	77	7.4
2702	-8	23	0.8	63	155	49	7.3
	2	23	0.8	111	142	37	7.3
	6	22	0.8	72	161	55	7.2
	10	19	0.6	90	171	99	7.5
	17	21	0.7	90	169	69	7.3
	24	20	0.7	89	137	211	7.5
	52	19	0.7	88	124	129	7.7
	94	16	0.8	108	121	89	7.7

Appendix 10

Appendix 3

Individual Clinical Chemistry Values

5550014

Sex: Female

8.4 X10E13 vg Group 2	Day(s) Relative to Start Date	Reporting Biochemistry ALB (g/dL)	GLOB (g/dL)	A/G (ratio)	CA (mg/dL)	PHOS (mg/dL)	NA (mmol/L)
2701	-8	4.5	2.7	1.7	9.4	4.5	145
	2	4.0	3.1	1.3	9.3	3.8	144
	6	4.3	2.9	1.5	9.6	4.6	147
	10	4.4	2.8	1.6	9.7	4.5	143
	17	4.3	2.9	1.5	9.7	4.8	146
	24	4.2	2.9	1.4	9.6	4.5	146
	52	4.3	2.6	1.7	9.4	4.0	144
	94	4.1	3.3	1.2	9.3	4.2	145
2702	-8	4.2	3.1	1.4	9.2	3.8	146
	2	3.9	3.4	1.1	9.3	3.2	145
	6	4.0	3.2	1.3	9.3	3.8	148
	10	4.0	3.5	1.1	9.4	4.1	146
	17	3.7	3.6	1.0	9.5	3.4	147
	24	3.4	4.1	0.8	9.6	4.0	147
	52	3.9	3.8	1.0	9.4	4.1	148
	94	4.2	3.5	1.2	9.4	4.1	145

Appendix 10

Appendix 3

Individual Clinical Chemistry Values

5550014

Sex: Female

8.4 X10E13 vg Group 2	Day(s) Relative to Start Date	Reporting Biochemistry K (mmol/L)	CL (mmol/L)	SAMQ
2701	-8	4.5	102	N
	2	4.1	104	N
	6	4.5	105	N
	10	5.6	104	N
	17	4.5	104	N
	24	4.4	104	N
	52	4.6	102	N
	94	4.3	103	H+
2702	-8	4.2	107	N
	2	4.1	106	H+
	6	4.6	110	N
	10	5.0	103	N
	17	4.9	106	N
	24	4.7	103	N
	52	4.7	105	N
	94	5.0	108	N

Appendix 10

Appendix 3

Individual Clinical Chemistry Values

5550014

Sex: Female

1.68 X10E14 vg Group 3	Day(s) Relative to Start Date	Reporting Biochemistry AST (U/L)	ALT (U/L)	ALP (U/L)	GGT (U/L)	CK (U/L)	TBIL (mg/dL)
3701	-8	69	52	214	51	426	0.11
	2	77	64	229	64	497	0.09
	6	68	50	225	56	1026	0.10
	10	51	44	218	53	202	0.03
	17	75	58	207	46	434	0.13
	24	45	45	183	51	196	0.08
	52	56	46	198	49	283	0.05
	94	40	44	182	42	249	0.05

Individual Clinical Chemistry Values

5550014

Sex: Female

1.68 X10E14 vg Group 3	Day(s) Relative to Start Date	Reporting Biochemistry					
		UREAN (mg/dL)	CREAT (mg/dL)	GLUC (mg/dL)	CHOL (mg/dL)	TRIG (mg/dL)	TPROT (g/dL)
3701	-8	18	0.5	50	187	83	7.4
	2	16	0.6	108	172	39	7.6
	6	14	0.5	66	171	49	7.1
	10	12	0.5	64	162	72	7.2
	17	19	0.6	70	168	61	7.4
	24	15	0.5	70	141	97	7.3
	52	10	0.5	64	110	109	7.2
	94	10	0.6	117	114	63	7.4

Appendix 10

Appendix 3

Individual Clinical Chemistry Values

5550014

Sex: Female

1.68 X10E14 vg Group 3	Day(s) Relative to Start Date	ALB (g/dL)	GLOB (g/dL)	A/G (ratio)	CA (mg/dL)	PHOS (mg/dL)	NA (mmol/L)
		Reporting Biochemistry					
3701	-8	4.5	2.9	1.6	9.2	3.9	145
	2	4.1	3.5	1.2	9.2	3.6	144
	6	4.1	3.0	1.4	9.1	4.2	146
	10	4.1	3.1	1.3	9.2	4.5	143
	17	4.2	3.2	1.3	9.3	4.6	143
	24	4.4	2.9	1.5	9.1	3.6	144
	52	4.1	3.1	1.3	9.1	3.9	147
	94	3.9	3.5	1.1	9.2	3.8	145

Appendix 10

Appendix 3

Individual Clinical Chemistry Values

5550014

Sex: Female

1.68 X10E14 vg Group 3	Day(s) Relative to Start Date	Reporting Biochemistry K (mmol/L)	CL (mmol/L)	SAMQ
3701	-8	4.2	107	N
	2	3.9	107	N
	6	4.6	109	N
	10	4.5	104	N
	17	4.8	106	N
	24	3.9	106	N
	52	4.2	103	N
	94	4.4	107	N

Appendix 10

Appendix 4

Individual Urinalysis Values

5550014

Sex: Male

0 vg Group 1	Day(s) Relative to Start Date	Reporting Urinalysis VOLUME (mL)	COLOR	CLARITY	GLUC	BIL	KET
1201	-8	6.0	LY	Cld	Neg	Neg	Neg
	2	16.0	LY	Clr	Neg	Neg	2+
	6	25.0	LY	Cld	Neg	Neg	Neg
	24	18.0	LY	Cld	Neg	Neg	Neg
	52	10.0	LY	Cld	Neg	Neg	Neg
	94	7.3	LY	Cld	Neg	Neg	Neg

Appendix 10

Appendix 4

Individual Urinalysis Values

5550014

Sex: Male

0<br>vg<br>Group 1	Day(s) Relative to Start Date	Reporting Urinalysis<br>SPECIFIC GRAVITY	BLD	URINE pH	PROT
1201	-8	1.016	1+	9.0 [a]	Neg
	2	1.027	Neg	7.0	Neg
	6	1.012	Neg	9.0 [a]	Neg
	24	1.012	Neg	9.0 [a]	Neg
	52	1.018	Neg	9.0 [a]	Neg
	94	1.008	Neg	9.0 [a]	Neg

[a] [RC:Assigned value above the reportable range]

Appendix 10

Appendix 4

Individual Urinalysis Values

5550014

Sex: Male

1.68 X10E14 vg Group 3	Day(s) Relative to Start Date	Reporting Urinalysis					
		VOLUME (mL)	COLOR	CLARITY	GLUC	BIL	KET
3201	-8	5.0	LY	Clr	Neg	Neg	Neg
	2	11.0	LY	Clr	Neg	Neg	Neg
	6	18.0	LY	Clr	Neg	Neg	Neg
	24	6.2	LY	Tur	Neg	Neg	Neg
	52	2.4	LY	Cld	Neg	Neg	Neg
	94	8.4	LY	Tur	Neg	Neg	Neg

Appendix 10

Appendix 4

Individual Urinalysis Values

5550014

Sex: Male

1.68 X10E14 vg Group 3	Day(s) Relative to Start Date	Reporting Urinalysis			
		SPECIFIC GRAVITY	BLD	URINE pH	PROT
3201	-8	1.010	Neg	9.0 [a]	Neg
	2	1.030	Neg	8.0	Neg
	6	1.014	Neg	8.5	Neg
	24	1.027	Neg	9.0 [a]	Neg
	52	1.030	Neg	9.0 [a]	Neg
	94	1.029	Neg	9.0 [a]	Neg

[a] [RC:Assigned value above the reportable range]

Appendix 10

Appendix 4

Individual Urinalysis Values

5550014

Sex: Female

0<br>vg<br>Group 1	Day(s) Relative to Start Date	Reporting Urinalysis<br>VOLUME (mL)	COLOR	CLARITY	GLUC	BIL	KET
1701	-8	4.0	LY	Cld	Neg	Neg	Neg
	2	9.6	LY	Clr	Neg	Neg	Neg
	6	15.0	LY	Clr	Neg	Neg	Neg
	24	1.4	LY	Clr	Neg	Neg	Neg
	52	2.2	LY	Cld	Neg	Neg	Neg
	94	5.2	LY	Clr	Neg	Neg	Neg

Appendix 10

Appendix 4

Individual Urinalysis Values

5550014

Sex: Female

0<br>vg<br>Group 1	Day(s) Relative to Start Date	Reporting Urinalysis<br>SPECIFIC GRAVITY	BLD	URINE pH	PROT
1701	-8	1.016	3+	9.0 [a]	1+
	2	1.012	Neg	9.0 [a]	Neg
	6	1.015	1+	9.0 [a]	Neg
	24	1.009	1+	9.0 [a]	Neg
	52	1.008	1+	8.5	Neg
	94	1.009	1+	9.0 [a]	Neg

[a] [RC:Assigned value above the reportable range]

Appendix 10

Appendix 4

Individual Urinalysis Values

5550014

Sex: Female

8.4 X10E13 vg Group 2	Day(s) Relative to Start Date	Reporting Urinalysis					
		VOLUME (mL)	COLOR	CLARITY	GLUC	BIL	KET
2701	-8	8.0	LY	Cld	Neg	Neg	Neg
	2	2.8	LY	Cld	Neg	Neg	Neg
	6	12.0	LY	Clr	Neg	Neg	Neg
	24	4.4	LY	Cld	Neg	Neg	Neg
	52	6.4	LY	Tur	Neg	Neg	Neg
	94	5.7	LY	Cld	Neg	Neg	Neg
2702	-8	9.0	LY	Cld	Neg	Neg	1+
	2	9.2	LY	Cld	Neg	Neg	1+
	6	1.6	LY	Cld	Neg	Neg	Neg
	24	2.0	LY	Tur	Neg	Neg	Neg
	52	4.0	LY	Clr	Neg	Neg	Neg
	94	4.7	LY	Cld	Neg	Neg	Neg

Appendix 10

Appendix 4

Individual Urinalysis Values

5550014

Sex: Female

8.4 X10E13 vg Group 2	Day(s) Relative to Start Date	Reporting Urinalysis SPECIFIC GRAVITY	BLD	URINE pH	PROT
2701	-8	1.024	Neg	8.5	Neg
	2	1.020	3+	9.0 [a]	Neg
	6	1.016	Neg	9.0 [a]	Neg
	24	1.012	Neg	9.0 [a]	Neg
	52	1.033	Neg	9.0 [a]	1+
	94	1.008	Neg	9.0 [a]	Neg
2702	-8	1.009	2+	9.0 [a]	Neg
	2	1.014	1+	9.0 [a]	Neg
	6	1.024	1+	9.0 [a]	2+
	24	1.020	2+	9.0 [a]	2+
	52	1.007	Neg	9.0 [a]	Neg
	94	1.012	Neg	9.0 [a]	Neg

[a] [RC:Assigned value above the reportable range]

Appendix 10

Appendix 4

Individual Urinalysis Values

5550014

Sex: Female

1.68 X10E14 vg Group 3	Day(s) Relative to Start Date	Reporting Urinalysis					
		VOLUME (mL)	COLOR	CLARITY	GLUC	BIL	KET
3701	-8	30.0	LY	Tur	Neg	Neg	Neg
	2	21.6	LY	Clr	Neg	Neg	1+
	6	12.0	LY	Tur	Neg	Neg	Neg
	24	4.4	LY	Cld	Neg	Neg	Neg
	52	13.0	LY	Cld	Neg	Neg	Neg
	94	12.7	LY	Clr	Neg	Neg	Neg

Appendix 10

Appendix 4

Individual Urinalysis Values

5550014

Sex: Female

1.68 X10E14 vg Group 3	Day(s) Relative to Start Date	Reporting Urinalysis SPECIFIC GRAVITY	BLD	URINE pH	PROT
3701	-8	1.019	3+	9.0 [a]	Neg
	2	1.034	Neg	5.5	Neg
	6	1.019	1+	7.5	Neg
	24	1.006	Neg	9.0 [a]	Neg
	52	1.007	3+	9.0 [a]	Neg
	94	1.006	1+	8.5	Neg

[a] [RC:Assigned value above the reportable range]

Appendix 10

SIGNATURE(S) FOR DOCUMENT: 5550014 - 5550014 Clinical Pathology Final Report

**Principal Investigator:**	I approve this document.
Name:	**Poitout, Florence**
Poitout, Florence	03-Mar-2022 19:49:00 (UTC+00:00)
Electronically Signed in M-Files	Timestamp

Appendix 11

FINAL REPORT

Study Phase: Biomarker Cytokines Analysis Interpretative Report

Test Facility Study No. 5550014

Sponsor Reference No. UTSW-Gray-SPG50-004

GLP

TEST FACILITY:
Charles River Laboratories Montreal ULC
Senneville Site (CR-SEN)

TABLE OF CONTENTS

LIST OF TABLES....3
LIST OF APPENDICES....4
1. LIST OF ABBREVIATIONS....5
2. INTRODUCTION....6
3. EXPERIMENTAL PROCEDURES....6
3.1. Materials and Methods....6
3.2. Computerized Systems....6
4. RESULTS AND DISCUSSIONS....7
4.1. Standards and Quality Control Samples for the multiplex and IP-10....7
4.2. Study Sample....7
4.3. Definition of Normal Ranges....8
4.4. Cytokine Results....8
5. CONCLUSION....10
6. REPORT APPROVAL....11

LIST OF TABLES

Table 1 Summary of Fold Changes and Statistical Tests of Cytokines 12

LIST OF APPENDICES

Appendix 1 Deviations....................18
Appendix 2 Individual Cytokine Values....................21
Appendix 3 AP.BMK.mkpCYT.12....................25
Appendix 4 AP.BMK.mkpIP10.09....................70

1. LIST OF ABBREVIATIONS

This section provides abbreviations of terms and concepts that may be commonly used throughout this report.

MCP1	Monocyte chemoattractant protein-1
QC	Quality Control
tQC	Trending Quality Control (Endogenous QC)
IP-10	Interferon gamma-induced protein 10
IL	Interleukin
LLOQ	Lower Limit of Quantitation
TNFα	Tumor necrosis factor alpha
ULOQ	Upper Limit of Quantitation

2. INTRODUCTION

This report describes the biomarker evaluation of cytokines in monkey plasma samples from Study No. 5550014 entitled "*A Single-Dose Study of AAV9/AP4M1 by Intrathecal Injection in Immunosuppressed Monkeys*".

For the work detailed in this report, the experimental start and end dates were 08 Oct 2021, and 30 Oct 2021, respectively.

Animals were assigned to 3 groups (1/sex/group for Groups 1 and 3 and 2 females in Group 2) and administered AAV9/AP4M1 or Reference Item/Vehicle by intrathecal injection once. Group 1 animals were administered Reference Item. Animals in Groups 2 and 3 were administered $8.4x10^{13}$ and $1.68x10^{14}$ of AAV9/AP4M1, respectively. Samples for cytokines were collected from all animals on Day 1 (pre-dose, 30 min, 4 hours and 24 hours postdose).

3. EXPERIMENTAL PROCEDURES

3.1. Materials and Methods

The methodology and materials used for the biomarker analyses were detailed in their respective analytical procedures listed in the table below:

Biomarker	Analytical Procedure No.	Validation Study Number
Multiplex (Il-6, IL-8, IL-10, TNF-a, MCP-1)	AP.BMK.mkpCYT.12	3600083
IP-10	AP.BMK.mkpIP10.09	3800698

3.2. Computerized Systems

Critical computerized systems used in this study phase are listed below (see Text Table 1).

Text Table 1
Computerized Systems

System Name	Version No.	Description of Data Collected and/or Analyzed
Softmax Pro GxP	5.4.6	Data collection
Bio Plex Manager (Bio-Rad)	6.1	Data collection
Watson LIMS	7.6.1 HF3	Data analysis and sample management
eInfotree	7.6 or higher	Excel module for collection of 21 CFR Part 11 compliance requirements, security, audit trail and electronic signatures
Deviation Information Library	2.1	Recording of deviation reports
M-files®	21	Reporting and collection of 21 CFR Part 11 compliant signature
SRS (CR-SEN in-house application built in SAS)	1.4	Statistical analysis
Mesa Laboratories AmegaView CMS	v3.0 Build 1209.08	Continuous Monitoring System. Monitoring of standalone fridges, freezers, incubators, and selected laboratories to measure temperature, relative humidity, and CO_2, as appropriate
Johnson Controls Metasys	MVE 7.0	Building Automation System. Control of HVAC and other building systems, as well as temperature/humidity control and trending in selected laboratories and animal rooms

4. RESULTS AND DISCUSSIONS

4.1. Standards and Quality Control Samples for the multiplex and IP-10

Standard, QC preparation and acceptance criteria are described in the latest version of the analytical procedure (Appendix 3 and Appendix 4). Standard curve and QC specifications are presented in Text Table 2 and in Text Table 3.

Text Table 2
Multiplex Standard Curve and Quality Controls Specifications

<table>
<tr><th>Cytokine</th><th>Range of the Curve (pg/mL)</th><th>LLOQ (pg/mL)</th><th>ULOQ (pg/mL)</th><th>QC1 (pg/mL)</th><th>QC2 (pg/mL)</th><th>QC3 (pg/mL)</th><th>tQC (pg/mL)</th></tr>
<tr><td>IL-6</td><td>37.5 to 6000.00*</td><td>37.50</td><td>4800.00</td><td rowspan="3">100.00</td><td rowspan="3">1000.00</td><td rowspan="3">3200.00</td><td>1103.25</td></tr>
<tr><td>IL-8</td><td rowspan="2">37.5 to 6000.00</td><td rowspan="2">37.50</td><td rowspan="2">6000.00</td><td>1143.54</td></tr>
<tr><td>TNF-a</td><td>776.20</td></tr>
<tr><td>IL-10</td><td>187.50 to 30000.00</td><td>187.50</td><td>30000.00</td><td>500.00</td><td>5000.00</td><td>16000.00</td><td>4122.64</td></tr>
<tr><td>MCP-1</td><td>37.5 to 2400.00</td><td>37.50</td><td>2400.00</td><td>100.00</td><td>1000.00[a]</td><td>1600.00</td><td>746.71</td></tr>
</table>

* Standard above the ULOQ is an accessory standard used to better define the upper portion of the curve.
[a] For MCP-1, the QC2 concentration was set at 1000.00 pg/mL instead of between 240.00 to 960.00 pg/mL. The QC2 concentration was first set at 1000.00 pg/mL because the first ULOQ tested was at 6000.00 pg/mL. Due to a plateau observed at the upper end of the standard curve, the ULOQ was decreased to 2400.00 pg/mL and the QC2 (1000.00 pg/mL) was kept. A difference of 40 pg/mL is not considered to have an impact on the validation or study sample analysis and the QC2 still monitors the day-to-day variability that could be observed in the middle part of the curve.

Text Table 3
IP-10 Standard Curve and Quality Controls Specifications

Range of the Curve (pg/mL)	LLOQ (pg/mL)	ULOQ (pg/mL)	QC1 (pg/mL)	QC2 (pg/mL)	QC3 (pg/mL)	tQC (pg/mL)
15.00 to 750.00*	30.00	750.00	70.00	250.00	500.00	181.45

* Standard below the LLOQ is an accessory standard used to better define the lower portion of the curve.

A total of two multiplex assays were performed and they both met the method acceptance criteria. All acceptable results were reported from these assays.

A total of two IP-10 assays were performed and one of the two assays did not meet the method acceptance criteria. All results were reported from the assay that met the acceptance criteria.

4.2. Study Sample

All study samples collected on Day 1 were analyzed within the validated ambient room temperature, 4°C, freeze/thaw and long-term stabilities for all the cytokines tested.

For IP-10 only, the following samples obtained results below the LLOQ in the initial analysis performed diluted 2-fold but there was insufficient sample volume to repeat the analysis using undiluted samples.

Subject	Nominal Time
1701	Day 1 4 hours postdose
1701	Day 1 24 hours postdose
2702	Day 1 30 minutes postdose
2702	Day 1 4 hours postdose
3701	Day 1 4 hours postdose

Those samples will be noted on the results table and the calculation value will be determined as LLOQ/2.

Any residual cytokines samples were maintained for a maximum of 6 months following issuance of the first full Draft Report after which samples were Discarded as requested and authorized by the Study Director and in consultation with the Sponsor.

4.3. Definition of Normal Ranges

The upper limit of the normal range of concentrations was defined as the overall baseline mean (predose Day 1, values for all animals in all groups, males and females calculated separately) plus 2 standard deviations.

Fold change indicates the ratio of the measured concentrations over the upper limit of the normal range of concentration for increases and the ratio of the lower limit of the normal range of concentration over the measured concentrations for decreases. Incidence indicates the number of individual animals per group with a fold change higher or equal to 1.1-fold. Throughout the discussion, the fold increases will be expressed as "X" for example: 1.1X being equivalent to 1.1-fold increase.

Individual animal data were compared to the upper limit of the normal range, as applicable, for any observed trends (time or dose related changes). For individual animals, if a value was above this value, this increase was considered to indicate a potential AAV9/AP4M1-related effect.

The upper limit of normal range of concentrations are presented in Text Table 4.

Text Table 4
Upper Limit of the Normal Range of Concentrations (pg/mL)

	IL-6	IL-8	IL-10	TNF-α	MCP-1	IP-10
Males	37.50	380.48	187.50	706.22	222.79	317.36
Females	37.50	135.36	187.50	249.76	164.09	192.36

4.4. Cytokine Results

The cytokines results are presented in Table 1 and Appendix 2 as well as Appendix 3 and Appendix 4.

All samples tested for IL-6 and IL-10 were below the LLOQ, therefore the administration of AAV9/AP4M1 was considered not to have an impact on the concentrations of those cytokines.

For TNF-α and IP-10, some quantifiable results were obtained at the predose time point as well as postdose. No increases above the upper limit of normal range could be observed, therefore the

administration of AAV9/AP4M1 was considered not to have an impact on the concentrations of those cytokines

For IL-8, all the females administered AAV9/AP4M1 obtained increases. Animal Nos. 2701 and 3701 obtained increases at 30 minutes postdose (1.8X and 2.7X, respectively) as well as 24 hours postdose (2.9X and 1.2X, respectively). Animal No. 2702 obtained 1.6X increase at 4 hours postdose and 1.5X at 24 hours postdose. Those increases of low magnitude were considered AAV9/AP4M1-related due to their high incidence with the females. The dosed male did not obtain any increase.

For MCP-1, most of the results were quantifiable and only Female No. 2702 obtained an increase of 1.4X at 24 hours postdose. Due to the low magnitude and incidence of increases, the treatment with AAV9/AP4M1 was considered not to have an impact on the levels of MCP-1.

5. CONCLUSION

All samples collected for the cytokines analyses were analyzed using validated immunoassay methods. Based on the acceptable performance of the standards and QCs during sample analysis, it is concluded that the concentration values reported for the study samples are valid. All samples collected during the course of this study were analyzed within validated stabilities.

No AAV9/AP4M1-related effect could be observed for Il-6, IL-10, TNF-a, MCP-1, and IP-10.

For IL-8, some AAV9/AP4M1-related increases could be observed for the females only. Despite the high incidence of the increases, their low magnitudes suggest a limited biological significance.

6. REPORT APPROVAL

All electronic signatures appear at the end of the document upon finalization.

Appendix 11

Table 1

Summary of Fold Changes and Statistical Tests of Cytokines

			Group 1 0 vg				Group 2 8.4x10E13 vg				Group 3 1.68x10E14 vg			
Analyte	Gender	Nominal Time	N	Inc	Minimum Fold	Maximum Fold	N	Inc	Minimum Fold	Maximum Fold	N	Inc	Minimum Fold	Maximum Fold
mkpIL6	Male	D1 Predose	1	0	0.0	0.0					1	0	0.0	0.0
		D1M30	1	0	0.0	0.0					1	0	0.0	0.0
		D1H4	1	0	0.0	0.0					1	0	0.0	0.0
		D1H24	1	0	0.0	0.0					1	0	0.0	0.0
	Female	D1 Predose	1	0	0.0	0.0	2	0	0.0	0.0	1	0	0.0	0.0
		D1M30	1	0	0.0	0.0	2	0	0.0	0.0	1	0	0.0	0.0
		D1H4	1	0	0.0	0.0	2	0	0.0	0.0	1	0	0.0	0.0
		D1H24	1	0	0.0	0.0	2	0	0.0	0.0	1	0	0.0	0.0

Inc= Incidence

Appendix 11

Table 1

Summary of Fold Changes and Statistical Tests of Cytokines

			Group 1 0 vg				Group 2 8.4x10E13 vg				Group 3 1.68x10E14 vg			
Analyte	Gender	Nominal Time	N	Inc	Minimum Fold	Maximum Fold	N	Inc	Minimum Fold	Maximum Fold	N	Inc	Minimum Fold	Maximum Fold
mkpIL8	Male	D1 Predose	1	0	0.0	0.0					1	0	0.0	0.0
		D1M30	1	0	0.0	0.0					1	0	0.0	0.0
		D1H4	1	0	0.0	0.0					1	0	0.0	0.0
		D1H24	1	0	0.0	0.0					1	0	0.0	0.0
	Female	D1 Predose	1	0	0.0	0.0	2	0	0.0	0.0	1	0	0.0	0.0
		D1M30	1	0	0.0	0.0	2	1	1.8	1.8	1	1	2.7	2.7
		D1H4	1	0	0.0	0.0	2	1	1.6	1.6	1	0	0.0	0.0
		D1H24	1	0	0.0	0.0	2	2	1.5	2.9	1	1	1.2	1.2

Inc= Incidence

Appendix 11

Table 1

Summary of Fold Changes and Statistical Tests of Cytokines

			Group 1 0 vg				Group 2 8.4x10E13 vg				Group 3 1.68x10E14 vg			
Analyte	Gender	Nominal Time	N	Inc	Minimum Fold	Maximum Fold	N	Inc	Minimum Fold	Maximum Fold	N	Inc	Minimum Fold	Maximum Fold
mkpIL10	Male	D1 Predose	1	0	0.0	0.0					1	0	0.0	0.0
		D1M30	1	0	0.0	0.0					1	0	0.0	0.0
		D1H4	1	0	0.0	0.0					1	0	0.0	0.0
		D1H24	1	0	0.0	0.0					1	0	0.0	0.0
	Female	D1 Predose	1	0	0.0	0.0	2	0	0.0	0.0	1	0	0.0	0.0
		D1M30	1	0	0.0	0.0	2	0	0.0	0.0	1	0	0.0	0.0
		D1H4	1	0	0.0	0.0	2	0	0.0	0.0	1	0	0.0	0.0
		D1H24	1	0	0.0	0.0	2	0	0.0	0.0	1	0	0.0	0.0

Inc= Incidence

Appendix 11

Table 1

Summary of Fold Changes and Statistical Tests of Cytokines

			Group 1 0 vg				Group 2 8.4x10E13 vg				Group 3 1.68x10E14 vg			
Analyte	Gender	Nominal Time	N	Inc	Minimum Fold	Maximum Fold	N	Inc	Minimum Fold	Maximum Fold	N	Inc	Minimum Fold	Maximum Fold
mkpMCP1	Male	D1 Predose	1	0	0.0	0.0					1	0	0.0	0.0
		D1M30	1	0	0.0	0.0					1	0	0.0	0.0
		D1H4	1	0	0.0	0.0					1	0	0.0	0.0
		D1H24	1	0	0.0	0.0					1	0	0.0	0.0
	Female	D1 Predose	1	0	0.0	0.0	2	0	0.0	0.0	1	0	0.0	0.0
		D1M30	1	0	0.0	0.0	2	0	0.0	0.0	1	0	0.0	0.0
		D1H4	1	0	0.0	0.0	2	0	0.0	0.0	1	0	0.0	0.0
		D1H24	1	0	0.0	0.0	2	1	1.4	1.4	1	0	0.0	0.0

Inc= Incidence

Appendix 11

Table 1

Summary of Fold Changes and Statistical Tests of Cytokines

			Group 1 0 vg				Group 2 8.4x10E13 vg				Group 3 1.68x10E14 vg			
Analyte	Gender	Nominal Time	N	Inc	Minimum Fold	Maximum Fold	N	Inc	Minimum Fold	Maximum Fold	N	Inc	Minimum Fold	Maximum Fold
mkpTNFA	Male	D1 Predose	1	0	0.0	0.0					1	0	0.0	0.0
		D1M30	1	0	0.0	0.0					1	0	0.0	0.0
		D1H4	1	0	0.0	0.0					1	0	0.0	0.0
		D1H24	1	0	0.0	0.0					1	0	0.0	0.0
	Female	D1 Predose	1	0	0.0	0.0	2	0	0.0	0.0	1	0	0.0	0.0
		D1M30	1	0	0.0	0.0	2	0	0.0	0.0	1	0	0.0	0.0
		D1H4	1	0	0.0	0.0	2	0	0.0	0.0	1	0	0.0	0.0
		D1H24	1	0	0.0	0.0	2	0	0.0	0.0	1	0	0.0	0.0

Inc= Incidence

Appendix 11

Table 1

Summary of Fold Changes and Statistical Tests of Cytokines

			Group 1 0 vg				Group 2 8.4x10E13 vg				Group 3 1.68x10E14 vg			
Analyte	Gender	Nominal Time	N	Inc	Minimum Fold	Maximum Fold	N	Inc	Minimum Fold	Maximum Fold	N	Inc	Minimum Fold	Maximum Fold
mkpIP10	Male	D1 Predose	1	0	0.0	0.0					1	0	0.0	0.0
		D1M30	1	0	0.0	0.0					1	0	0.0	0.0
		D1H4	1	0	0.0	0.0					1	0	0.0	0.0
		D1H24	1	0	0.0	0.0					1	0	0.0	0.0
	Female	D1 Predose	1	0	0.0	0.0	2	0	0.0	0.0	1	0	0.0	0.0
		D1M30	1	0	0.0	0.0	2	0	0.0	0.0	1	0	0.0	0.0
		D1H4	1	0	0.0	0.0	2	0	0.0	0.0	1	0	0.0	0.0
		D1H24	1	0	0.0	0.0	2	0	0.0	0.0	1	0	0.0	0.0

Inc= Incidence

Appendix 11

Appendix 1

DEVIATIONS

All deviations that occurred during this study phase have been acknowledged by the Study Director, assessed for impact, and documented in the study records. None of the deviations were considered to have impacted the overall integrity of this study phase or the interpretation of the study phase results and conclusions.

Individual Cytokine Values Explanation Page

Abbreviation	Description	Abbreviation	Description
./--	No findings / Dead	QNS	Quantity not sufficient
CLOT	Sample clotted	SNR	Sample not received
NC	Not calculable	SNC	Sample not collected
NR	Not reported, result out of acceptance criteria	TNR	Test not reported
X	Excluded from mean	PD	Post Dose
a	Insufficient volume for a repeat analysis at a lower dilution, LLOQ/2 (taking into consideration the dilution factor) will be reported	b	Result reported in singlicate. Second replicate is <LLOQ. Singlicate result within threshold, as per Analytical Procedure quantifiable result to be reported.

Note: This is a comprehensive list of abbreviations. All of the abbreviations listed may not be applicable to this report.

Note:

For IL-6, IL-8, TNF-α and MCP-1

Lower Limit of Quantitation (LLOQ) = 37.50 pg/mL (75.00 pg/mL when taking the dilution factor into account), <LLOQ was assigned as 75.00/2 (37.50 pg/mL) for calculation purposes.

For IL-10
Lower Limit of Quantitation (LLOQ) = 187.50 pg/mL (375.00 pg/mL when taking the dilution factor into account), <LLOQ was assigned as 375.00/2 (187.50 pg/mL) for calculation purposes.

For IP-10
Lower Limit of Quantitation (LLOQ) = 30.00 pg/mL (60.00 pg/mL when taking the dilution factor into account), <LLOQ was assigned as 60.00/2 (30.00 pg/mL) for calculation purposes.

The upper limit of the normal range of concentrations was defined as:
The overall baseline mean (predose/pretreatment values for all animals* in all groups)** + 2 standard deviations

Fold change was reported as:
The ratio of the measured Cytokine concentration/upper limit of the normal range of concentrations.

The fold change was calculated for each sample.
Incidence of cytokine elevations was reported as:
The number of individual animals* per group with a fold change ≥ 1.1-fold.

*Calculations were done separately for females and males.

**If predose values were not available, values from all animals of the non-treated group(s) were used to generate the overall baseline mean.

Dosing Information
Dosing information is abbreviated on various data outputs; the following represents the dosing information for this study.

Group No.	Test Material	Dose Level (vg)
1	Reference Item	0
2	AAV9/AP4M1	$8.4x10^{13}$
3	AAV9/AP4M1	$1.68x10^{14}$

Individual Cytokine Values

Males

Group 1 - Reference Item Group 3 - AAV9/AP4M1 1.68x10[14] vg

Group	Animal Number	Occasion	IL-6 pg/mL	Incidence	Fold Change	IL-8 pg/mL	Incidence	Fold Change	IL-10 pg/mL	Incidence	Fold Change
1	1201	Day 1 - Predose	< 75.00	0	1.0	216.67	0	0.6	< 375.00	0	1.0
		Day 1 - 30 min PD	< 75.00	0	1.0	83.31	0	0.2	< 375.00	0	1.0
		Day 1 - 4 hours PD	< 75.00	0	1.0	118.23	0	0.3	< 375.00	0	1.0
		Day 1 - 24 hours PD	< 75.00	0	1.0	149.38	0	0.4	< 375.00	0	1.0
3	3201	Day 1 - Predose	< 75.00	0	1.0	< 75.00	0	0.1	< 375.00	0	1.0
		Day 1 - 30 min PD	< 75.00	0	1.0	87.31	0	0.2	< 375.00	0	1.0
		Day 1 - 4 hours PD	< 75.00	0	1.0	92.50	0	0.2	< 375.00	0	1.0
		Day 1 - 24 hours PD	< 75.00	0	1.0	< 75.00	0	0.1	< 375.00	0	1.0

Individual Cytokine Values

Males

Group 1 - Reference Item

Group 3 - AAV9/AP4M1 1.68x10^{14} vg

Group	Animal Number	Occasion	MCP-1 pg/mL	Incidence	Fold Change	TNF-α pg/mL	Incidence	Fold Change	IP-10 pg/mL	Incidence	Fold Change
1	1201	Day 1 - Predose	106.24	0	0.5	386.84	0	0.5	94.08	0	0.3
		Day 1 - 30 min PD	87.33	0	0.4	< 75.00	0	0.1	62.70	0	0.2
		Day 1 - 4 hours PD	91.81	0	0.4	< 75.00	0	0.1	60.57 b	0	0.2
		Day 1 - 24 hours PD	151.26	0	0.7	< 75.00	0	0.1	72.48	0	0.2
3	3201	Day 1 - Predose	167.13	0	0.8	< 75.00	0	0.1	210.72	0	0.7
		Day 1 - 30 min PD	86.74	0	0.4	< 75.00	0	0.1	134.55	0	0.4
		Day 1 - 4 hours PD	< 75.00	0	0.2	< 75.00	0	0.1	98.95	0	0.3
		Day 1 - 24 hours PD	75.01	0	0.3	146.87	0	0.2	91.94	0	0.3

Appendix 11

Appendix 2

Individual Cytokine Values

Females

Group 1 - Reference Item
Group 2 - AAV9/AP4M1 8.4x10[13] vg
Group 3 - AAV9/AP4M1 1.68x10[14] vg

Group	Animal Number	Occasion	IL-6 pg/mL	Incidence	Fold Change	IL-8 pg/mL	Incidence	Fold Change	IL-10 pg/mL	Incidence	Fold Change
1	1701	Day 1 - Predose	< 75.00	0	1.0	115.79	0	0.9	< 375.00	0	1.0
		Day 1 - 30 min PD	< 75.00	0	1.0	< 75.00	0	0.3	< 375.00	0	1.0
		Day 1 - 4 hours PD	< 75.00	0	1.0	< 75.00	0	0.3	< 375.00	0	1.0
		Day 1 - 24 hours PD	< 75.00	0	1.0	< 75.00	0	0.3	< 375.00	0	1.0
2	2701	Day 1 - Predose	< 75.00	0	1.0	< 75.00	0	0.3	< 375.00	0	1.0
		Day 1 - 30 min PD	< 75.00	0	1.0	242.78	1	1.8	< 375.00	0	1.0
		Day 1 - 4 hours PD	< 75.00	0	1.0	< 75.00	0	0.3	< 375.00	0	1.0
		Day 1 - 24 hours PD	< 75.00	0	1.0	395.17	1	2.9	< 375.00	0	1.0
	2702	Day 1 - Predose	< 75.00	0	1.0	< 75.00	0	0.3	< 375.00	0	1.0
		Day 1 - 30 min PD	< 75.00	0	1.0	< 75.00	0	0.3	< 375.00	0	1.0
		Day 1 - 4 hours PD	< 75.00	0	1.0	212.91	1	1.6	< 375.00	0	1.0
		Day 1 - 24 hours PD	< 75.00	0	1.0	196.56	1	1.5	< 375.00	0	1.0
3	3701	Day 1 - Predose	< 75.00	0	1.0	< 75.00	0	0.3	< 375.00	0	1.0
		Day 1 - 30 min PD	< 75.00	0	1.0	362.46	1	2.7	< 375.00	0	1.0
		Day 1 - 4 hours PD	< 75.00	0	1.0	91.49	0	0.7	< 375.00	0	1.0
		Day 1 - 24 hours PD	< 75.00	0	1.0	160.17	1	1.2	< 375.00	0	1.0

Individual Cytokine Values

Females

Group 1 - Reference Item
Group 2 - AAV9/AP4M1 8.4x10[13] vg
Group 3 - AAV9/AP4M1 1.68x10[14] vg

Group	Animal Number	Occasion	MCP-1 pg/mL	Incidence	Fold Change	TNF-α pg/mL	Incidence	Fold Change	IP-10 pg/mL	Incidence	Fold Change
1	1701	Day 1 - Predose	112.32	0	0.7	207.31	0	0.8	92.61	0	0.5
		Day 1 - 30 min PD	82.04	0	0.5	< 75.00	0	0.2	61.76	0	0.3
		Day 1 - 4 hours PD	76.09	0	0.5	< 75.00	0	0.2	< 60.00 a	0	0.2
		Day 1 - 24 hours PD	85.03	0	0.5	< 75.00	0	0.2	< 60.00 a	0	0.2
2	2701	Day 1 - Predose	139.02	0	0.8	< 75.00	0	0.2	170.20	0	0.9
		Day 1 - 30 min PD	93.56	0	0.6	< 75.00	0	0.2	106.58	0	0.6
		Day 1 - 4 hours PD	< 75.00	0	0.2	< 75.00	0	0.2	74.60	0	0.4
		Day 1 - 24 hours PD	128.61	0	0.8	80.93	0	0.3	125.76	0	0.7
	2702	Day 1 - Predose	149.59	0	0.9	< 75.00	0	0.2	77.43	0	0.4
		Day 1 - 30 min PD	144.25	0	0.9	< 75.00	0	0.2	< 60.00 a	0	0.2
		Day 1 - 4 hours PD	115.44	0	0.7	< 75.00	0	0.2	< 60.00 a	0	0.2
		Day 1 - 24 hours PD	237.71	1	1.4	< 75.00	0	0.2	103.43	0	0.5
3	3701	Day 1 - Predose	123.32	0	0.8	< 75.00	0	0.2	99.34	0	0.5
		Day 1 - 30 min PD	103.24	0	0.6	< 75.00	0	0.2	75.71	0	0.4
		Day 1 - 4 hours PD	< 75.00	0	0.2	< 75.00	0	0.2	< 60.00 a	0	0.2
		Day 1 - 24 hours PD	100.14	0	0.6	< 75.00	0	0.2	61.67	0	0.3

Appendix 11

Appendix 3

charles river

Title: **LUMINEX METHOD FOR THE QUANTITATIVE DETECTION OF IL-1β, IL-1RA, IL-6, IL-10, IL-12/23 (p40), IL-15, IL-18, IFN-γ, TNF-α, G-CSF, MCP-1, MIP-1β, GM-CSF, IL-2, IL-4, IL-5, IL-8, IL-13 AND IL-17A IN CYNOMOLGUS MONKEY PLASMA USING THE MAGNETIC BEADS**	AP Number: AP.BMK.mkpCYT.12	Effective Date: Signature of AP
	CR-SEN/SHB	Supersedes: 19-Mar-2019
Prepared by: Marc-André Roy Scientist I, Immunology (A) Stephanie C.		Date: 12-Nov-2020
Verified by: Sonia Ménard Research Scientist I, Biomarkers [signature]		Date: 12 Nov 2020
Management Approval: Annie St-Pierre Senior Research Scientist II, Biomarkers [signature]		Date: 12 Nov 2020

1.0 Purpose

To describe a method to determine the concentration of IL-1β, IL-1RA, IL-6, IL-10, IL-12/23 (p40), IL-15, IL-18, IFN-γ, TNF-α, G-CSF, MCP-1,MIP-1β, GM-CSF, IL-2, IL-4, IL-5, IL-8, IL-13 and IL-17A in cynomolgus monkey plasma by Luminex.

2.0 Scope

This procedure applies to Luminex assays undertaken in the Biomarkers department.

3.0 Responsibilities

All staff performing this assay are responsible for compliance with this analytical procedure.

4.0 Required forms

Appendix 1	Cytokine Multiplex Spiking Sheet (Example of document)
Appendix 2a	Cytokine Multiplex Assay Sheet (Plate washer)
Appendix 2b	Cytokine Multiplex Assay Sheet (Manual wash)
Appendix 3	Daily Solution preparation Sheet (Example of document) **Note: Appendix # 2 of CACI-001 can be used as well.**
Appendix 4	Solution preparation Sheet (Example of document) **Note: Appendix # 1 of CACI-001 can be used as well.**
Appendix 5	Assay Instructions Sheet (Example of document)
Appendix 6	Cytokine Multiplex Spiking Preparation of tQC (Example of document)
Appendix 7	Sample Analysis Instruction Sheet (Example of spreadsheet). **Note: this appendix is to be used when assay includes sample(s) not analyzed for all cytokines stated in Assay Instructions Sheet (Appendix 5).**

(A) Stéphanie Martin signed on behalf of Marc-André Roy. SMA/12-Nov-2020

AP Number: AP.BMK.mkpCYT.12	Effective Date: Signature of AP	Supersedes: 19-Mar-2019	Page 2 of 18 pages

5.0 Materials/Equipment/Reagents

Materials can be substituted provided the same specifications are met.

The procedure may require other general laboratory supplies commonly used in Laboratory Sciences.

If the lot# of a reagent is recorded, the recording of the inventory # is not critical, since it is considered to be for information or reference purposes only. The only situation that would make the inventory # critical is for reagents of the same lot that have an expiry date based on the receipt date.

5.1 Materials/Equipment

5.1.1 Non Disposable/Instrumentation
Centrifuge
Pipettes (multi-channel, micro)
Vortex mixer
Bio Plex Suspension Array
Bio Plex Manager™ Software
Watson Laboratory Information Management System (LIMS)
Automatic plate washer for Magnetic beads (Bio-Plex PRO II units)
Handheld magnetic washer
Orbital plate shaker (orbit 4mm)
Titer plate shaker
Serologic pipettes
Sonicating bath
Waterbath

5.1.2 Disposable
Aluminum foil
Tubes (polypropylene, various caps, various size)
Ultra-pure water (UPW)
Pipette tips (various sizes)
Non-sterile solution basins
Parafilm
Absorbent paper
Auto-sticker plate sealers
96-well plate, polypropylene, (used for pre-loading).

5.2 Kit Components

Notes:

i. Concentration of kit components / reagents can vary from lot to lot. If the kit lot changes, but the critical reagents (Non-Human Primate Cytokine Standard, Serum Matrix, Non-Human Primate Cytokines Detection Antibodies, Streptavidin-Phycoerythrin or Non-Human Primate Cytokine Antibody-Immobilized Beads) are the same as the kit reagents already qualified (from another kit lot), then the qualification of the new critical reagents is not required.

ii. When possible, the same kit lot number should be used for the entire study to prevent any

Appendix 11

Appendix 3

AP Number: AP.BMK.mkpCYT.12	Effective Date: Signature of AP	Supersedes: 19-Mar-2019	Page 3 of 18 pages

variability in the results. New kit lots should be qualified as per SOP BMK-004 prior to use. In the event where the same lot of kit cannot be used throughout the study and lot-to-lot variability is present, this should be considered in the interpretation of the results.

iii. All kit reagents should be stored in a refrigerator set to maintain 4°C until the expiration date printed on the label.

iv. All kit reagents should be brought to ambient room temperature (RT) prior to use.

Non-Human Primate Cytokine Kit
(Millipore cat# PRCYTOMAG-40K-XX, where XX denotes the number of cytokines included in the panel, refer to Appendix #5 and appropriate Study plan for the actual list of cytokines to be analyzed)

Reagents provided	Catalogue No.	Quantity	Use
Non-Human Primate Cytokine Standard	MXPR8040*	1 vial	Lyophilized
Non-Human Primate Cytokine Quality Controls 1 and 2	MXPR6040	2 vials	**Do Not Use**
Serum Matrix	MXPRSM*	1 vial	Lyophilized
96-well Plate	N/Ap**	1 plate	**Ready to use**
Assay Buffer	L-AB	1 Bottle (30mL)	**Ready to use**
Wash Buffer (10X)	L-WB	1 Bottle (60mL)	To be diluted
Non-Human Primate Cytokines Detection Antibodies	MXPR1040-2	1 Bottle (3.2mL)	**Ready to use**
Streptavidin-Phycoerythrin	L-SAPE4	1 Bottle (3.2mL)	**Ready to use**
Bead diluent	LBD	1 Bottle (3.5mL)	To be diluted
Mixing Bottle	N/Ap	1 Bottle	**Ready to use**

*1 additional vial of Non-Human Primate Cytokine Standard should be ordered per kit. 3 additional vials of Serum Matrix should be ordered per kit.
**Note that lot number/expiry is not always provided by supplier, as the loading plate is not a pre-coated plate and the plate is included in the kit, there is no need to have a prompt to record the lot and expiry date.

Non-Human Primate Cytokine Antibody-Immobilized Beads
Refer to Appendix #5 and appropriate Study plan for the list of cytokines to be analyzed

Bead/Analyte name	Catalogue No. (50X concentration, 90µL each)
Anti-IL-1β	HCYIL1B-MAG
Anti- IL-1RA	HIL1RA-MAG
Anti-IL-6	HCYIL6-MAG
Anti-IL-10	PRIL10-MAG
Anti- IL-12/23 (p40)	PRIL12P40-MAG
Anti-IL-15	HIL15-MAG
Anti-IL-18	PRIL18-MAG
Anti-TNF-α	PRTNFA-MAG
Anti-IFN-γ	PRIFNG-MAG

AP Number: AP.BMK.mkpCYT.12	Effective Date: Signature of AP	Supersedes: 19-Mar-2019	Page 4 of 18 pages

Bead/Analyte name	Catalogue No. (50X concentration, 90µL each)
Anti-MCP-1	HCYMCP1-MAG
Anti-MIP-1β	HMIP1B-MAG
Anti-G-CSF	HGCSF-MAG
Anti-GM-CSF	PRGMCSF-MAG
Anti-IL-2	PRIL2-MAG
Anti-IL-4	HIL4-MAG
Anti-IL-5	HIL5-MAG
Anti-IL-13	PRIL13-MAG
Anti-IL-17A	HIL17-MAG
Anti-IL-8	HCYIL8-MAG

5.3 Matrix(ces) (used to prepare trending QC)

Species: Cynomolgus Monkey plasma
Anticoagulant: EDTA
Supplier: Charles River or BioIVT
Lot number: to be documented in raw data
Storage: in a freezer set to maintain -20°C or -80°C (as appropriate)
Expiry: SIP (Stability in progress). Refer to validation box.

5.4 Other reagents

- Sheath fluid (Luminex Catalogue no. 40-50000 or Bio-Rad Catalogue no. 171-000055)

6.0 Preparation of Assay Reagents

Notes:

i. Volumes described for any preparation may be scaled up or down proportionally as long as the target concentration is not changed. All changes must be documented in raw data.
ii. Bring all reagents to ambient RT before use.
iii. For the preparation of daily solutions, pipettes should be recorded on Appendix #2.

6.1 Preparation of kit reagents

6.1.1 Wash buffer cytokines (code: **mkpCYT WB**)

Record the preparation on appendix 4

- Add 540 mL of UPW to an appropriate container.
- Add the contents of the Wash Buffer Concentrate (10X) bottle (60 mL per bottle) and mix well by inversion.
- Store in a refrigerator set to maintain 4°C for a maximum of 1 month.
- Use at ambient RT during the assay.

6.1.2 Antibody-Immobilized Beads Working Solution **(code: ABWS)**

Note: if not all cytokines are needed to be analyzed, Bead Diluent should be used to replace the missing antibody-bead volume. The total volume of the solution needs to be 3.000 mL. Dilution 1/50.

AP Number: AP.BMK.mkpCYT.12	Effective Date: Signature of AP	Supersedes: 19-Mar-2019	Page 5 of 18 pages

Record the preparation on Appendix #3

- Sonicate the antibody-bead bottles for 30 seconds and then vortex for 1 minute. **Refer to Appendix #5 for the list of cytokines to be analyzed.**
- For example: If 19 cytokines: Add 1.860 mL of Bead Diluent to the mixing bottle and 60 µL from each antibody bead tube to the mixing bottle; vortex well.
- Each well requires 25 µL of the diluted beads.
- This solution is prepared on the day of the assay.

6.1.3 Preparation Serum Matrix (code: **mkpSM**)

Record the preparation on appendix 1

- Reconstitute 1 vial of Lyophilized Serum Matrix with 1mL of UPW
- Vortex well.
- Allow at least 10 minutes for complete reconstitution.
- Leftover reconstituted Serum Matrix may be stored in a freezer set to maintain -20°C for up to 1 month. Discard if remaining volume is not needed or insufficient for subsequent assays.

6.2 Preparation of the Non-Human Primate Cytokine Standards and Quality Control Samples

Notes:

i. All changes should be documented.
ii. Preparation is performed at ambient RT.

6.2.1 Reconstitution of the Non-Human Primate Cytokine Standard (code: **Stock**)

- Reconstitute the Non-Human Primate Cytokine Standard vial with 250 µL of UPW.
- Invert the vial several times to mix; vortex the vial for 10 seconds.
- Allow the vial to sit for at least 5 minutes for complete reconstitution and then transfer as soon as possible into a polypropylene tube and promptly start the spiking.
- The standard stock should be used to prepare the Standards and QCs within **one hour of the reconstitution finish time**.
- Record preparation on Appendix 1.

6.2.2 Preparation of standards and QCs

- Standards are prepared using the standard stock (Code: Stock) as the high standard, and doing serial dilutions using the Serum matrix (Code: SM) as a diluent.
- The diluted standards should be prepared on the day of the assay and used **(refer to the start time of the incubation with the beads)** within **one hour of finish time**.
- Record preparation on Appendix 1.
- The following range should be kept for the preparation of the QCs except for the MCP-1 QC2
  - Low (QC1): Less than or equal to 3 times the LLOQ (but >LLOQ).
  - Medium (QC2) *: 10 - 40% of the ULOQ.
  - High (QC3): 50 - 80% of the ULOQ.

* During validation: For MCP-1, the QC2 concentration was set at 1000.00 pg/mL instead of between 240.00 to 960.00 pg/mL. The QC2 concentration was first set at 1000.00

Appendix 11

Appendix 3

pg/mL because the first ULOQ tested was at 6000.00 pg/mL. Due to a plateau observed at the upper end of the standard curve, the ULOQ was decreased to 2400.00 pg/mL and the QC2 (1000.00 pg/mL) was kept. A difference of 40 pg/mL is not considered to have an impact on the validation or study sample analysis and the QC2 still monitors the day-to-day variability that could be observed in the middle part of the curve.

7.0 Preparation of trending QC (tQC) using diluted matrix(ces) or spiked matrix(ces) using Standard Stock

The selected lot of monkey plasma must be used diluted at least 1/2 with Serum matrix (the tQC can be matrix(ces) with appropriate endogenous concentration or if endogenous concentration cannot be found for all the cytokines tested, the tQC will be a matrix(ces) diluted 2-fold and then spiked at appropriate concentration). Refer to BMK-004 for preparation details.

- Aliquot the chosen lot(s) neat into appropriately labeled polypropylene tubes and store in a freezer set to maintain -20°C or -80°C (as appropriate). **The expiry date on the label of the neat matrix(ces) should be SIP (stability in progress).**
- Stability of tQC samples will be monitored and deemed acceptable based on the assay performance when analyzed using a qualified lot of kit.
- Diluted or spiked tQC samples should be prepared on wet ice on the day of the assay.
- Record preparation of tQC samples on Appendix #6.

Validation Summary Table:

**Based on validation Study 3600083**	
**Parameter**	**Results**
**Validated range**	Refer to table on Appendix #5
**Dilution in Serum Matrix diluent**	Based on Parallelism:<br>IL-1β, IL-1RA and IL-8= 2 to 64-fold<br>MIP-1β=2 to 16-fold<br>IL-6*, IL-10*, TNF-a, IFN-γ = 2 to 4-fold<br>IL-12/23(p40), IL-13, MCP-1*, G-CSF = 2-fold<br>Based on the Linearity of dilution :<br>IL-2= 5 to 128-fold but MRD set at 2-fold<br>IL-4= 4 to 64-fold but MRD set at 2-fold<br>IL-5= 2 to 256-fold<br>IL-15 and IFN-γ= 2 to 198.77-fold<br>IL-17A= 8 to 256-fold but MRD set at 2-fold<br>GM-CSF and IL-18= no conclusive results; MRD 2-fold<br>***The appropriate dilution range should be determined based on expected IL-6, IL-10, and MCP-1 values. Samples can be diluted using one of the 2 options listed below but options cannot be combined within the same study.**<br>**Option 1:** Sample can be diluted as stated above<br>**Option 2:** Samples can be diluted from 8 to 64-fold for IL-6 and IL-10 and 8 to 32-fold for MCP-1.

Appendix 11

Appendix 3

AP Number: AP.BMK.mkpCYT.12	Effective Date: Signature of AP	Supersedes: 19-Mar-2019	Page 7 of 18 pages

**Based on validation Study 3600083**	
**Parameter**	**Results**
**Short term matrix stability (at ambient RT)**	IL-1β, IL-2, IL-4, IL-5, IL-12/23(p40), IL-13, IL-15, IL-17A, IFN-γ, GM-CSF= 6 hrs 57 min<br>IL-1RA, IL-6, IL-8, IL-10, MIP-1β, G-CSF= 6 hrs 30 min<br>TNF-α= 6 hrs 7 min<br>IL-18 and MCP-1= 5 hrs 51 min
**Short term matrix stability (at 4°C/wet ice)**	IL-1β, IL-2, IL-4, IL-5, IL-12/23(p40), IL-13, IL-15, IL-17A, IFN-γ, GM-CSF, TNF-α ans IL-18= 24 hrs 7 min<br>IL-1RA, IL-6, IL-8, IL-10, MIP-1β, G-CSF= 24 hrs 2 min<br>MCP-1= 24 hrs 25 min
**Freeze-thaw matrix stability at -20°C**	IL-1β, IL-5, IL-13, IL-15, GM-CSF, IL-1RA, IL-6, IL-8, IL-10, MIP-1β, TNF-α, GM-CSF and IL-18= 4 Freeze-Thaw cycles<br>IL-2, IL-4, IL-12/23(p40), IL-17A, IFN-γ and MCP-1= 5 Freeze-Thaw cycles
**Freeze-thaw matrix stability at -80°C**	IL-1β, IL-4, IL-12/23(p40), IL-13, IL-5, IL-15, IL-17A, IFN-γ, GM-CSF, IL-1RA, IL-6, IL-8, IL-10, MIP-1β, TNF-α, G-CSF and IL-18= 4 Freeze-Thaw cycles<br>IL-2, and MCP-1= 5 Freeze-Thaw cycles
**Long term matrix stability at -20°C**	IL-1β, IL-2, IL-4, IL-5, IL-12/23(p40), IL-13, IL-15, IL-17A, IFN-γ, GM-CSF, IL-1RA, IL-6, IL-8, IL-10, MIP-1β, TNF-α and G-CSF = 90 days<br>IL-18= 29 days<br>MCP-1= 92 days
**Long term matrix stability at -80 °C**	IL-2, IL-4, IL-5, IL-12/23(p40), IL-13, IL-15, IL-17A, GM-CSF, IL-8, IL-10, MIP-1β, G-CSF = 90 days<br>IL-18= 88 days<br>IL-1RA, TNF-α, IFN-γ= 173 days<br>IL-6= 195 days<br>IL-1β= 264 days<br>MCP-1= 92 days

8.0 <u>Assay procedure</u>

Notes:

i. **Refer to Appendix #5 for the Cytokine Multiplex Assay Sheet to be used for the assay procedure (Appendix 2a or Appendix 2b).**

ii. Pipettes used for the assay procedure, preparation of standards, QCs, tQCs, diluted samples and daily solutions will be recorded on Appendix # 2. Pipettes used for sample dilution can also be recorded on the dilution sheet.

iii. *QC samples prepared in Serum Matrix and tQC should be loaded n=2 (one set before and one set after samples) or more if needed for kit qualification.*

iv. Samples should be transported from Sample Management on dry ice. Record in-process sample storage in Appendix #2.

v. Study samples should be analyzed in duplicate at the same time.

vi. The study samples will be diluted at least 2-fold using the Serum Matrix (minimal required dilution (MRD)).

vii. Dilution of samples should be performed on wet ice, refer to Appendix #5 for instructions.

AP Number: AP.BMK.mkpCYT.12	Effective Date: Signature of AP	Supersedes: 19-Mar-2019	Page 8 of 18 pages

viii. All solutions added for incubation at ambient RT should be brought to ambient RT prior to use.
ix. For all incubation steps, the plate should be covered with aluminium foil sealer in order to be protected from light.
x. Refer to Appendix 2a (plate washer) or 2b (manual wash) for the multiplex assay procedure. The automatic plate washer should be the first option for washing steps, however, if there is no plate washer in the laboratory, manual wash, using handheld magnetic washer, can be done (refer to appendix #5).
xi. If the reading of the plate needs to be stopped due to a reading malfunction, repeat the last wash step, add 150 µL of Sheath fluid in all wells of the plate. Incubate the plate protected from light on a plate shaker set at 600 rpm at ambient RT for at least 5 minutes and re-read the entire plate. **This should be documented in the raw data**.

9.0 Plate Washer for magnetic beads verification and settings (Appendix 2a)

- Daily Maintenance should be performed as per SOP CAE-213.
- Wash program should be ran as specified in the SOP using program «LUM MAG»

Plate mode		
BPlex-Mag		
**Cycle 1 (8 parameters):**		
C1 P1 Soak<br>Time 1 min 0s<br>Shake OFF	C1 P4 Soak<br>Time 1 min 0s<br>Shake OFF	C1 P7 Soak<br>Time 1 min 0s<br>Shake OFF
C1 P2 Aspirate<br>Crosswise (cross) aspirate (Asp) YES<br>Position (pos): bottom<br>Time: 4s<br>H-Speed 10mm/s<br>Asp Rate 2	C1 P5 Aspirate<br>Crosswise (cross) aspirate (Asp) YES<br>Position (pos): bottom<br>Time: 4s<br>H-Speed 10mm/s<br>Asp Rate 2	C1 P8 Aspirate<br>Crosswise (cross) aspirate (Asp) YES<br>Position (pos): bottom<br>Time: 4s<br>H-Speed 10mm/s<br>Asp Rate 2
C1 P3 Dispense<br>Z-pos Overflow<br>Volume 200 µL<br>Channel 1<br>Flow (FL) rate 200 µL/s	C1 P6 Dispense<br>Z-pos Overflow<br>Volume 200 µL<br>Channel 1<br>Flow (FL) rate 200 µL/s	

10.0 Plate Washer for magnetic beads verification and settings (Appendix 2b)

- Procedure to empty the wells with handheld magnetic washer is detailed in the Appendix 2b.

11.0 Preparation of the Bio-Plex Suspension Array (Luminex) Protocol

Note: The different items (STD's, QC's or Samples) loaded on the Bio-Plex plate layout should always be set up in replicates of 2. In the case where only one well was used on the assay plate or if a sample is not loaded, Bio-Plex Manager should still be configured with 2 replicates. An appropriate note in the raw data will indicate to the Scientist that only one well was loaded or if the sample was not loaded and the required replicates will be deactivated accordingly after importing the data into Watson.

11.1.1 The luminex should be calibrated as follows:

- Perform a warm up of the Bio-Plex Suspension assay System.

Appendix 11

Appendix 3

AP Number: AP.BMK.mkpCYT.12	Effective Date: Signature of AP	Supersedes: 19-Mar-2019	Page 9 of 18 pages

- **If the Bio-Plex Suspension assay System is no longer warm when the analyst is ready to perform the start-up, this step should be repeated (note that Bio-Plex manager will prompt the user if the instrument needs a warm up before start up).**
- Perform a start-up of the Bio-Plex Suspension assay System.
- Perform a calibration of the Bio-Plex Suspension assay System as described below.

11.1.2 Unclog before reading plate

- This procedure is required before reading a Luminex plate. It is not needed for the first plate to be read **on a given day** if the calibration passes successfully.
- If the unclog fails, the needle should be sonicated, and the unclog repeated.
- If the repeat is successful, the plate can be read and the calibration does not need to be repeated

11.1.3 The BioPlex calibration kit contains two bottles, Bio-Plex cal 1 (red) and Bio-Plex cal 2 (green).

- In the luminex calibration window, enter information related to Bio-Plex cal 1 (i.e. DD target, CL1 target, CL2 target) written on the back of the red bottle.
- In the luminex calibration window, enter information related to Bio-Plex cal 2 written on the back of the green bottle. For the cytokines luminex enter **only the Low RP1 target value**. The High RP1 target value should not be used.

11.1.4 Parameters of the Protocol should be set as follows:

- In section #2."Select Analytes", select the panel "Millipore Non-Human Primate Cytokine Bead Panel". If the panel "Millipore Non-Human Primate Cytokine Bead Panel" is not in the selection of the drop-down menu, create it and add the cytokines and their defined region as described in the kit insert. The cytokine ID should be entered as stated in bold below.

- Transfer the following cytokine in the "Selected" column: ***(Please note that the cytokine ID should be typed in Bioplex exactly as below):***

Refer to Appendix #5 for the list of cytokines to be analyzed.

**mkpIL1B (Region 46)**	**mkpIL6 (Region 57)**
**mkpIL1RA (Region 42)**	**mkpIFNG (Region 20)**
**mkpIL10 (Region 35)**	**mkpTNFA (Region 72)**
**mkpIL18 (Region 78)**	**mkpIL12-23 (Region 74)**
**mkpIL15 (Region 37)**	**mkpGCSF (Region 18)**
**mkpMIP1B (Region 73)**	**mkpMCP1 (Region 67)**
**mkpGMCSF (Region 14)**	**mkpIL2 (Region 33)**
**mkpIL4 (Region 53)**	**mkpIL5 (Region 55)**
**mkpIL8 (Region 63)**	**mkpIL13 (Region 44)**
**mkpIL17A (Region 39)**	

- In section #3."Format Plate", format the plate sequence as per Watson's plate sequence.
- In section #7."Run Protocol", **set the beads at 50 per region**. (At least 30 beads should be acquired to consider the reading reliable)
- In the "Advanced Settings", set sample size at 100 µL.
- The doublet discriminator Gate range setting (DD gates) is set between **5 000 and 25 000**.
- The reporter PMT is set as default.

AP Number: AP.BMK.mkpCYT.12	Effective Date: Signature of AP	Supersedes: 19-Mar-2019	Page 10 of 18 pages

12.0 <u>Exporting data to Watson LIMS</u>

Notes:

i. *The assay should first be setup with these settings: Assay Type should be Multi Analyte Plate and the Instrument Type and Instrument Interface should be Bio-Rad Bio-Plex.*

ii. *For Watson analysis, Analyte info in Master assay should be set as follow:*

**Analyte Identification (also refer to Section 10.1.4)**				
IL-1β= mkpIL1B	IL-1RA= mkpIL1RA	IL-2=mkpIL2	IL-4= mkpIL4	IL-10= mkpIL10
IL-12/23(p40)= mkpIL12-23	TNF-α= mkpTNFA	IL-8= mkpIL8	IFN-γ= mkpIFNG	IL-15= mkpIL15
G-CSF= mkpGCSF	GM-CSF= mkpGMCSF	IL-5= mkpIL5	IL-13= mkpIL13	IL-6= mkpIL6
IL-17A= mkpIL17A	IL-18= mkpIL18	MCP-1= mkpMCP1	MIP-1β= mkpMIP1B	

Details (Watson Master Assay settings)	
Reduction type	5 PL (MARQUARDT)
Analyte Info	
Concentration units	pg/mL
Weighting Factor	1/Y**2
Decimal Places	6
Regression Type	5 PL (MARQUARDT)

12.1 <u>Export data to Watson LIMS as follows:</u>

- First make sure the required Bio-Plex data file is opened; the original unformatted file must be used for exporting.
- Click on the “Table Options” menu, and select “Show Replicates” (or click the equivalent taskbar button).
- Then, click on “File”, “Export Table” (or click the equivalent taskbar button).
- From the “Table Export Options” window, select the following options: Export Format: “Table per Analyte”, Export Source: “All Analytes”, Export Destination: “Text File (Tab Delimited).”
- Do NOT tick the Exclude checkboxes. Click “OK”, type a filename that represents the study/reference number and run (e.g.: STUDYNO_mkpCYT - xx, where “xx” denotes the assay number) and <u>refer</u> to SOP CAE-147 for exporting.

AP Number: AP.BMK.mkpCYT.12	Effective Date: Signature of AP	Supersedes: 19-Mar-2019	Page 11 of 18 pages

13.0 <u>Preparation of the Bio-Plex Manager printout</u>

Note:

i. *Due to a known issue with the Bio-Plex software, some of the pages on the Luminex printouts have the following information omitted at the bottom of the page: -1- low bead #. The above omission has no impact upon the integrity of the study.*

ii. *At least the first page of each analyte printout should be signed and dated. The pagination of each analyte printout is indicated at the bottom right of each page as "x/y", where "x" denotes the current page number and "y" denotes the total number of pages.*

iii. *When the printouts are reprinted, the "signed/by and Document ID" does not appears due to software limitations. Refer to original printouts when this situation occurs.*

13.1.1 When preparing the Bio-Plex Manager Protocol, the sample dilution should always be set at 1 regardless of the actual dilution factors. The final concentration will be adjusted in Watson using the appropriate dilution factor.

13.1.2 Once the assay plate has been read, the columns of the Bio-Plex Manager printout should be adjusted as follows prior to printing:

Type	Well	FI	FI - Bkgd	Bead Count	Sampling Error

13.1.3 Calculation

The five parameters logistic (5PL Marquardt) model is used to fit the sigmoid calibration curve. A logarithmic sigmoid calibration curve is obtained by plotting the Fluorescence Intensity (FI) against the concentration.
Concentrations of analytes in the test samples are determined by computer interpolation from the plot of the calibration curve.

Appendix 11

Appendix 3

AP Number: AP.BMK.mkpCYT.12	Effective Date: Signature of AP	Supersedes: 19-Mar-2019	Page 12 of 18 pages

14.0 Assay Acceptance Criteria

14.1 Standard Curve Acceptance Criteria

14.1.1 For all cytokines: Mean (FI) Blank < Mean (FI) LLOQ

Note: The Bio-Plex printout should be used in order to evaluate the FI (FI= Fluorescence Intensity)

14.1.2 The calibration curve should contain at least six non-zero standards, after removal of rejected standards (as described below). If the LLOQ and/or the ULOQ are removed, then the next lowest and/or next highest standard becomes the LLOQ and ULOQ, respectively. Any samples reported as > the new ULOQ or as < the new LLOQ should be repeated.

- Percent nominal calculated using the mean concentrations should be within 75% - 125% of their theoretical value for all standard except for LLOQ and ULOQ which should be within 70% - 130% (accessory standard are not included).
- A standard that meets the acceptance criteria but tends to bias the standard curve causing the curve to fail, thus having a negative impact on the overall standard curve or the other working standards, may be partially or fully masked.

If the criteria mentioned above are not met, a standard should be fully or partially removed (i.e. the minimal reprocessing):

- 8- and 9-points standard curve: Up to a maximum of i) two full points, or ii) one full point and two partial points, or iii) three partial points may be removed from a calibration curve. Two adjacent standards cannot be fully deleted in a curve.
- 7-point standard curve: Up to a maximum of i) one full point and one partial point, or ii) two partial points may be removed from a calibration curve.
- 6-point-standard curve: Up to a maximum of i) two partial points may be removed from a calibration curve.
- If the accessory standards have a negative impact on the overall back-calculation of the individual standards they can be partially or fully masked.

14.2 Acceptance Criteria for QC samples prepared in Serum matrix and tQC

Percent theoretical is calculated using the mean concentration and should be within 75% - 125% for quality control (QC and tQC) samples. The precision (%CV) between QC sample duplicate concentrations should be ≤ 20%, except if the QC value was reported in singlicate (refer to section 13.3).

14.3 Run Acceptance Criteria

A run is accepted if the following three criteria are met:

14.3.1 The curve must meet the acceptance criteria (mentioned in section 14.1).

14.3.2 At least 67% of the quality control (QC) samples must meet the acceptance criteria (mentioned in section 14.2), with 50% accepted quality control (QC) samples at each level.

14.3.3 With the addition of a “Sampling Error” column on the Luminex printouts certain results may be called into question when errors occur. See the table below for a description of the errors:

AP Number: AP.BMK.mkpCYT.12	Effective Date: Signature of AP	Supersedes: 19-Mar-2019	Page 13 of 18 pages

Error Code:	Indicates:
1	Low bead number detected in the well
2	Aggregated beads detected in the well
3	Bead classification efficiency problem detected in the well
4	Region selection problem detected in the well
5	Platform temperature problem detected

For this study, errors that can potentially compromise the quality of the data appear in this column (error codes 1, 3 and 4); please refer to the following list:

1) if both replicates for a standard, QC or test sample have sampling errors the standard, QC or test sample is to be rejected.
2) if one replicate of a blank, a standard or quality control has a sampling error the error can be masked and the value reported as a singlicate.

The above list requires that a minimum of 30 beads be read for the result to be considered reliable. Please note that for a low bead error, the error might not be applicable to all analytes. Only the affected analytes will be addressed. Error code 5 (platform temperature problem detected) appears if a variation of ± 2°C occurs during reading. If this happens, impact on the data should be evaluated and documented. If aggregation of the beads occurs causing the error code 2 to appear but does not impede acquisition of a minimum of 30 beads or does not cause bead classification or region selection problems, it will not be considered as causing reliability issues with the obtained results.

14.4 Acceptance criteria for Study samples

14.4.1 The mean duplicate concentration will be reported.

14.4.2 The %CV between the duplicate concentrations should be ≤ 20%. A sample must be repeated if the %CV between the duplicate concentrations is >20%.

14.4.3 if one replicate for a sample has a sampling error, the sample will be repeated or if insufficient volume remains only the singlicate value will be reported and flagged as such in the report.

14.4.4 Watson cannot calculate the % CV between singlicates if one singlicate is within the curve range, and the other singlicate is <LLOQ when the sample is analyzed at the lowest validated dilution or >ULOQ when analyzed at the highest validated dilution. Therefore, LLOQ and ULOQ thresholds have been defined, that represent 20.4% CV with the LLOQ and ULOQ, respectively. If one singlicate is not within the curve range and the other singlicate is > LLOQ threshold or <ULOQ threshold, the sample should be repeated. If the quantifiable is ≤ LLOQ threshold or ≥ ULOQ threshold, the quantifiable singlicate is reported. ***The specific LLOQ and ULOQ thresholds will be defined in Appendix # 5.***

14.4.5 Any study sample with concentration below LLOQ will be reported as < LLOQ (including dilution factor). The value of the LLOQ should be recorded in the footnote of the report table.

14.4.6 "Y/2" (where "Y" is the LLOQ concentration multiplied by the dilution factor) will be used for the calculation of the mean and standard deviation (SD) for values for which the measured concentration was <LLOQ, and will be mentioned as a footnote as well.

Appendix 11

Appendix 3

AP Number: AP.BMK.mkpCYT.12	Effective Date: Signature of AP	Supersedes: 19-Mar-2019	Page 14 of 18 pages

14.4.7 Any sample with a concentration >ULOQ should be further diluted with Serum Matrix and re-analyzed (as applicable).The final reported value should be the observed concentration multiplied by the dilution factor.

14.4.8 In the event that a sample may not be reanalyzed at a higher dilution factor due to validation or volume limitation, "X" (where X is the assay ULOQ concentration multiplied by the dilution factor at which the sample was analyzed) will be reported and flagged appropriately. "X" will be used for the calculation of the mean and standard deviation (SD).

14.4.9 The acceptance criteria defined for a multiplex assay are to be verified for each individual cytokine and sample. As such, samples may need to be reanalyzed for one or more cytokines but not necessarily for the entire panel. However, due to the multiplexing nature of the assay, it is not always feasible to repeat analysis for a specific cytokine(s) without generating data for the other cytokines in the panel. Therefore, when repeating samples for specific cytokine(s), one analytical run will be created in Watson and results for all analytes will be generated for every samples analyzed in the assay. However, samples where re-analysis for a specific cytokine(s) is not required will be deactivated (or masked) in Watson. The results will be kept in the raw data but not included in the report. Appendix #7, listing the samples and cytokines to be re-analyzed will be issued and signed by a Scientist or designee, prior to the analysis being performed. It should be noted that the Appendix #7 should be used only when sample(s) do not need to be analyzed for all cytokines stated in Appendix #5.

14.5 Reporting:

14.5.1 Concentrations in pg/mL, mean and SD should be rounded to two decimal places and percentage to one decimal place. Note that due to a software limitation, all means and SDs will be reported to 1 more decimal place and percentages will be reported with no decimal places when generated by the SRS software.

14.5.2 The upper limit of the normal range of concentrations will be defined as:

The overall baseline mean (predose/pretreament values for all animals* in all groups)** + 2 standard deviations.

Fold change will be reported as:

- The ratio of the measured Cytokine concentration/ upper limit of the normal range of concentrations.

- The fold change should be calculated for each sample.

Incidence of Cytokine elevations will be reported as:

The number of individual animals* per group with a fold change ≥ 1.1-fold.

Based on the nature of the study or specific sponsor requirement the upper limit of the normal range of concentrations, fold change and/or incidence might be calculated differently, and should be documented in the study specific study plans.

*Calculations will be done separately for females and males.

**If predose values are not available, values from all animals of the non-treated group(s) will be used to generate the overall baseline mean.

AP Number: AP.BMK.mkpCYT.12	Effective Date: Signature of AP	Supersedes: 19-Mar-2019	Page 15 of 18 pages

15.0 Revision History

Version	Date	Reason For Revision
1	23 Mar 2016	New AP
2	15 Apr 2016	- Clarified the amount of reagents to order in section 5.2.<br>- Updated the volumes to prepare the Wash buffer.<br>- Added IL-5 and removed IL-12 in the Validation Summary Table for Freeze-thaw matrix stability at -80°C.<br>- The "divided by 2" was removed from the last line of section 13.4.3.
3	28 Sep 2016	- Charles River logo was updated.<br>- LTS for IL-1RA, IFN-γ, TNF-a, IL-6, FT for IL-2, and all stabilities for IL-18 and MCP-1 were updated.<br>- Parallelism and linearity section for IL-1b, IFN-γ, IL-6, IL-8, IL-10 and MCP-1 was updated<br>- Wash buffer name code was changed.<br>- Appendices 2 and 4 were updated to reflect changes on wash buffer name code.<br>- Appendix 5 was updated to add a comment for GM-CSF.<br>- Appendix 2 was updated to update the step of the addition of the assay buffer.<br>- Section13.5.2 was updated to reflect appropriate interpretation (upper limit of normal range instead of normal range) (if predose or pretreatment not available the mean of the control group will be used)
4	17 Nov 2016	- Section 5.2: Clarification on the amount of reagent to order<br>- Section 8: Correction of the reference to the appendix for the in-process sample storage.<br>- Section 13.5.2: Fold change will be calculated for each sample.
5	07 Sep 2017	**Validation Box, Freeze-thaw matrix stability at -80°C:**<br>- IL-13 was added to the list of cytokines that can be submitted to 4 freeze-thaw cycles in a freezer set to maintain -80°C.<br>- GM-CF was corrected to GM-CSF.<br>**Throughout the document:** CR-MTL as updated to CR MTL, and minor typos were corrected.<br>- **Appendix 2, In Process Sample Storage:** N/Ap was added to the first box, to account for kit qualifications, endochecks and spikechecks, where no samples from Watson are used.<br>- **Appendix 2, Scientific Data Review :** N/Ap was added for "samples to repeat" for all cytokines, to account for kit qualifications, endochecks and spikechecks, where no samples from Watson are used.

AP Number: AP.BMK.mkpCYT.12	Effective Date: Signature of AP	Supersedes: 19-Mar-2019	Page 16 of 18 pages

		- **Appendix 5**: Added a prompt to enter the assay in which the kit lot and the standard were qualified.
6	26 Jan 2018	- Section 5.2 updated the amount of extra Non-Human Primate Cytokine Standard and serum matrix to be ordered per kit, clarified the critical reagents and added reagent volumes and catalogue numbers by bead<br>- Section 6.0: Added the appropriate appendix for the data recording<br>- Section 7.0: IL-1β was removed from the list of cytokines with a 90-day LTS since the LTS for IL-1 β has been extended to 264 days in a freezer set to maintain -80°C.<br>- Section 7.0, 8.0 and Appendix #5: Sample dilution updated from RT to wet ice.<br>- Section 10.1.2: corrected typo from f to if.<br>- Appendix 2: Updated sample handling from RT to wet ice, added brackets where missing, updated wash Buffer code from mkpCytWB to mkpCYTWB.<br>- Appendix 4: Separated the Storage location and expiry date prompts, update wash buffer code to mkpCYTWB<br>- Minor wording updates and reorganization
7	03 Apr 2018	- To clarify the example in section 6.1.2.<br>- To clarify the reconstitution of the Serum Matrix (Section 6.1.3) and to update the code of the solution.<br>- To remove the linearity of dilution for IL-1b because parallelism was performed under the validation and was obtained up to MRD (2-fold).<br>- To update the Data review section in Appendix 02. IL-17A should be mentioned instead of IL-17.<br>- To update appendices 01 and 02 for the code of the serum matrix.
8	24 Jul 2018	- Section 5.3: statement that matrices may have been stored at a different temperature was removed since both -20°C and -80°C are stated as possible storage temperatures.<br>- Section 9. Wording for maintenance of the plate washer was removed and replaced by a reference to the appropriate SOP.<br>- Sections 10.1.4 and 11.0 were updated with the SEND terminology.<br>- Section 13: the appropriate action in the events of beads errors in samples wells was moved from the run acceptance criteria to the samples acceptance criteria.<br>- Section 13.4.3 describing: If the mean concentration of a sample is lower than the lower limit of quantitation (LLOQ) and the %CV of the duplicate results is >20% […] was removed, since Watson cannot calculate the %CV when one of the singlicates is <LLOQ.<br>- Section 13.4 was updated to include the description of the ULOQ threshold.

Appendix 11

Appendix 3

AP Number: AP.BMK.mkpCYT.12	Effective Date: Signature of AP	Supersedes: 19-Mar-2019	Page 17 of 18 pages

		- Appendix #2: Sample dilution step of the assay instruction: N/Ap was added as a possibility to account for the absence of samples in a spikecheck.<br>- Appendix #5 was updated to include a space to enter the values of the ULOQ thresholds and to remove the check box for analysis on wet ice.
9	06 Dec 2018	- Addition of the Appendix 7, to define which analyte will be measured for sample repeats.<br>- Section 1.0: Correction of the name of MIP-1β<br>- Section 4.0: Add reference of the Appendix #7.<br>- Section 5.2: Increase to 3 the number of serum matrix vials to order by kit, two vials were not sufficient.<br>- Section 5.3: Update supplier name for BioIVT.<br>- Section 8.0: rewording for clarification<br>- Section 10.0: clarification of the note if sample was not loaded<br>- Section 13.4.8: Add paragraph for explanation of which cytokines results will be reported for sample repeats.<br>- Section 13.5.2: Definition of incidence of cytokine elevation was updated. The number of individual animals per group with a concentration upper to the limit of normal range was updated for a concentration ≥ 1.1-fold change.<br>- Appendix #3: transfer the sentence from the appendix #2, for the preparation / homogenization of the antibody-beads.<br>- Appendix #7: Add the correct symbol for cytokines names, change the entree format for records of date and time.<br>- Minor typographical errors corrected without changes in procedure.
10	11 Dec 2018	- Addition of special characters, which were missing on the title of the previous version.
11	19-Mar-2019	- Page 1 header: logo updated.<br>- Section 4.0: Example of Document was removed from next to Appendix #2, since this appendix is not subject to change.<br>- Section 5.1.1 Volumetric pipettes was changed to serologic pipettes.<br>- Section 13.1.2: clarification was added.<br>- Appendix #3: A prompt was added to record the sonicating and the vortexing of the beads prior to preparation of the beads mix. Supplier column removed since all reagents are from the kit.<br>- Appendix #4: Supplier column removed.<br>- Appendix #5: Correction of the storage temperature of the tQC (80°C was corrected to -80°C)<br>- Minor typos and inconsistencies were corrected throughout the document, without changes in the procedure.

Appendix 11

Appendix 3

12	Signature of AP	- Pages numbers were added to the header instead of footers in order to harmonize with other APs.<br>- Updated the section 5.1.1 to add the handheld magnetic washer and orbital plate shaker (orbit 4 mm).<br>- Updated the section 5.3 for the supplier to use when ordering matrix.<br>- Clarify the section 6.2 for the end time for the reconstitution of the standard stock and the STDs/QCs preparation.<br>- Manual wash using handheld magnetic washer added. Therefore, section 8 point i and section 9 were updated and section10 was added. These sections were updated/added to allow the use of manual wash.<br>- Updated Appendix #1, formula now refer to calculated concentration instead of target concentration.<br>- Updated Appendix #2a title to include plate washer.<br>- A new Appendix #2b was created for the assay using the manual wash.<br>- Updated Appendix #5 for the use of plate washer or manual wash.<br>- Minor typos and inconsistencies were corrected throughout the document, without changes in the procedure.

Appendix 11

Appendix 3

Cytokine Multiplex Spiking Sheet

Study/Reference no/Assay ID: ____
Reference material: Non-Human primate Cytokine Standard
Manufacturer: Millipore
Catalogue No.: MXPR8040

Table 1. Serum Matrix Lot number ____ Inventory # : ____

Sample Identification	Batch assigned	# of reconstituted vial	Volume of UPW added to each vial (mL)		Time left on bench after reconstitution (at least 10 minutes)		Transfer to polypropylene tube	Volume used from each vial to pool (mL)		Total Volume (mL)
Serum Matrix cat # MXPRSM	mkpSM-	4	1.000	( )( )( )( )	Start:	Finish:	( )	0.950	( ) ( ) ( )( )	3.800

Reconstituted serum Matrix Stored ☐ Location (if applicable) ____ Discarded after use ☐

Table 2. Standard stock Lot number ____ Inventory # : ____

Sample Identification	# of reconstituted vial	Volume of UPW added to each vial (mL). Vial inverted several times to mix, and vortex for 10 seconds		Time left on bench after reconstitution (at least 5 minutes)		Transfer to polypropylene tube	Volume used from each vial to pool (mL)		Total Volume (mL)
Non-Human primate Standard Cytokine (Stock)	2	0.250	( ) ( )	Start:	Finish:	( )	0.225	( ) ( )	0.450

Comments: **Standards and QCs should be used for the preparation of STDs and QCs within one hour of reconstitution finish time.**

Table 3. Preparation of Standards and QCs

	Target Concentration (pg/mL)				Stock Concentration (pg/mL)				Stock		Serum Matrix		Calculated Concentration (pg/mL)			
Sample Identification	IL-1β, IL-1RA, IL-6, IL-12/23 (p40), IL-15, TNF-α, IFN-γ, G-CSF, MCP-1, MIP-1β, GM-CSF, IL-2, IL-5, IL-8, IL-13 and IL-17A	IL-10, Il-18	IL-4	Stock Identification	IL-1β, IL-1RA, IL-6, IL-12/23 (p40), IL-15, TNF-α, IFN-γ, G-CSF, MCP-1, MIP-1β, GM-CSF, IL-2, IL-5, IL-8, IL-13 and IL-17A	IL-10, IL-18	IL-4	Total Volume (µL)	Volume (µL)	Performed (√)	Volume (µL)	Performed (√)	IL-1β, IL-1RA, IL-6, IL-12/23 (p40), IL-15, TNF-α, IFN-γ, G-CSF, MCP-1, MIP-1β, GM-CSF, IL-2, IL-5, IL-8, IL-13 and IL-17A	IL-10, IL-18	IL-4	End time of preparation of STDs and QCs
**Stock**	10000.00	50000.00	20000.00	Stock	10000.00	50000.00	20000.00	N/Ap	N/Ap	N/Ap	N/Ap	N/Ap	10000.00	50000.00	20000.00	
STD 9 (ULOQ2)*	6000.00	30000.00	12000.00	Stock	10000.00	50000.00	20000.00	350	210	( )	140	( )	6000.00	30000.00	12000.00	
STD 8 *	4800.00	24000.00	9600.00	STD 9 (ULOQ2)*	6000.00	30000.00	12000.00	205	164	( )	41	( )	4800.00	24000.00	9600.00	
STD 7 (ULOQ1)	2400.00	12000.00	4800.00	STD 8 *	4800.00	24000.00	9600.00	260	130	( )	130	( )	2400.00	12000.00	4800.00	
STD 6	1200.00	6000.00	2400.00	STD 7 (ULOQ1)	2400.00	12000.00	4800.00	160	80	( )	80	( )	1200.00	6000.00	2400.00	
STD 5	600.00	3000.00	1200.00	STD 6	1200.00	6000.00	2400.00	166	83	( )	83	( )	600.00	3000.00	1200.00	
STD 4	300.00	1500.00	600.00	STD 5	600.00	3000.00	1200.00	180	90	( )	90	( )	300.00	1500.00	600.00	
STD 3	150.00	750.00	300.00	STD 4	300.00	1500.00	600.00	210	105	( )	105	( )	150.00	750.00	300.00	
STD 2 (LLOQ2)	75.00	375.00	150.00	STD 3	150.00	750.00	300.00	270	135	( )	135	( )	75.00	375.00	150.00	
STD 1 (LLOQ1)	37.50	187.50	75.00	STD 2 (LLOQ2)	75.00	375.00	150.00	180	90	( )	90	( )	37.50	187.50	75.00	
STD 0	0.00	0.00	0.00	N/Ap	N/Ap	N/Ap	N/Ap	75	N/Ap	N/Ap	75	( )	0.00	0.00	0.00	
QC 3	3200.00	16000.00	6400.00	Stock	10000.00	50000.00	20000.00	325	104	( )	221	( )	3200.00	16000.00	6400.00	
QC3A	1600.00	8000.00	3200.00	**QC 3**	3200.00	16000.00	6400.00	176	88	( )	88	( )	1600.00	8000.00	3200.00	
QC 2	1000.00	5000.00	2000.00	**QC 3**	3200.00	16000.00	6400.00	240	75	( )	165	( )	1000.00	5000.00	2000.00	
QC 1	200.00	1000.00	400.00	**QC 2**	1000.00	5000.00	2000.00	175	35	( )	140	( )	200.00	1000.00	400.00	
QC 1A	100.00	500.00	200.00	**QC 2**	1000.00	5000.00	2000.00	170	17	( )	153	( )	100.00	500.00	200.00	

Comments: *= not applied for MCP-1 (refer to Appendix #5)

Spiking sheet verified by/ date: ____ Calculations verified by/ date: ____

Spiking performed by/ date: ____ Reviewed by/ date: ____

Appendix 1 (AP.BMK.mkpCyt.12)

Appendix 11

Appendix 3

Cytokine Multiplex Assay sheet (Plate washer)

Study/Reference No: ____________ Assay I.D.: ____________

Page: 1 of 10

Reagents/ Working Solutions					
Name	***Batch or Lot #***	***Inventory number***		***Expiry Date***	***Entered by /Date***
		Assay ID:	Assay ID: or ☐N/Ap		
Non-Human primate Cytokine kit					
Non Human primate Detection Antibodies					
Assay Buffer					
Bead diluent					
Streptavidin-Phycoerythrin					
Antibody-Immobilized Beads Working solution	ABWS-	N/Ap	N/Ap		
Wash Buffer Cytokines	mkpCYT WB-				
Serum matrix	mkpSM-	N/Ap	N/Ap		
Sheath fluid					
UPW	N/Ap	N/Ap	N/Ap		

PLATE SEQUENCE (Printed from Watson)

In-Process Sample Storage	Performed (√)		Start time	Performed by / Date
**Assay ID: or N/Ap ☐**				
Samples transported from Sample Management	Dry Ice	( )	N/Ap	
Samples thawed	Wet ice	( )		
Samples placed in temporary storage after use and until returned to Sample Management	Dry Ice	( )		
**In-Process Sample Storage**	**Performed (√)**		**Start time**	**Performed by / Date**
**Assay ID: or N/Ap ☐**				
Samples transported from Sample Management	Dry Ice	( )	N/Ap	
Samples thawed	Wet ice	( )		
Samples placed in temporary storage after use and until returned to Sample Management	Dry Ice	( )		

Appendix 2a (AP.BMK.mkpCyt.12)

Appendix 11

Appendix 3

Cytokine Multiplex Assay sheet (Plate washer)

Study/Reference No: ______________ Assay I.D.: ______________

Page: 2 of 10

INSTRUMENTS		
Name	***ID***	***Entered by / Date***
Plate washer for magnetic beads		
Pipettes		
Multichannel pipette		
Sonicating bath		
Titer plate shaker set at 600 rpm		
Bio-Plex Suspension Array		

Comments: ______________

Appendix 2a (AP.BMK.mkpCyt.12)

Appendix 11

Appendix 3

Cytokine Multiplex Assay sheet (Plate washer)

Study/Reference No: ______________ Assay I.D.: ______________

Page: 3 of 10

Steps	*Time / Performed (√)* Assay ID:	*Time / Performed (√)* Assay ID: or N/Ap ☐	*Performed by / Date*
Allow reagents to reach ambient RT before starting the assay.	( )	( )	
**Plate Preparation:** add 200 µL of Assay Buffer into each well of the microtiter plate. Cover the filter plate with an aluminum foil-wrapped plate cover and mix on a plate shaker set at 600 rpm at ambient RT for at least 10 minutes.	Start:<br>Finish:	Start:<br>Finish:	
Prepare Standards and QCs at ambient RT and tQCs on wet ice, in polypropylene tubes.	( )	( )	
Prepare diluted samples and keep on wet ice until loading as per Appendix #5	( ) or N/Ap ( )	( )	
Remove Assay buffer by decanting manually and blot dry on absorbent papers.	( )	( )	
**Load:** Add 25 µL of each Standard, QCs and samples (if applicable) into the appropriate wells (preloading can be performed if desired).	Preload( ) or N/Ap ( )<br>Load ( )	Preload( ) or N/Ap ( )<br>Load ( )	
**Load:** Add 25 µL of Assay Buffer in all wells.	( )	( )	
Sonicate for 30 seconds and vortex for 1 minute each bead vial used in the assay.<br>Prepare the Antibody-Immobilized Beads Working solution (code: ABWS).	( )	( )	
**Beads:** Vortex beads bottle and add 25 µL of beads to each well.	( )	( )	
Cover the plate with an aluminum foil plate cover (protected from light), and incubate on a plate shaker set at 600 rpm at ambient RT for 2 hours (± 5 minutes).	Start:<br>Finish:	Start:<br>Finish:	
**Wash:** Wash the plate using Wash Buffer cytokines (Code: mkpCYT WB) and using the LUM MAG program as per recommendations in the AP.	( )	( )	
**Capture:** Add 25 µL of detection antibodies into each well.	( )	( )	
Cover the plate with an aluminum foil plate cover (protected from light), and incubate 1 hour (± 2 minutes) on a plate shaker set at 600 rpm at ambient RT. **DO NOT ASPIRATE AFTER INCUBATION.**	Start:<br>Finish:	Start:<br>Finish:	
**Detection:** Add 25 µL of streptavidin-phycoerythrin to each well.	( )	( )	
Cover the plate with an aluminum foil plate cover (protected from light) and incubate on a plate shaker set at 600 rpm at ambient RT for 30 minutes (± 1 minute).	Start:<br>Finish:	Start:<br>Finish:	

Appendix 2a (AP.BMK.mkpCyt.12)

Appendix 11

Appendix 3

Cytokine Multiplex Assay sheet (Plate washer)

Study/Reference No: ____________ Assay I.D.: ____________

Page: 4 of 10

**Steps**	***Time / Performed (√)***		***Time / Performed (√)***		***Performed by / Date***
	Assay ID:		Assay ID: or N/Ap ☐		
**Wash:** Wash the plate using Wash Buffer cytokines (Code: mkpCyt WB) and using the LUM MAG program as per recommendations in the AP.	( )		( )		
Add **150 µL** of Sheath fluid to all wells. Cover the filter plate with an aluminum foil-wrapped plate cover and resuspend the beads by shaking on a plate shaker set at 600 rpm at ambient RT for at least 5 minutes.	Start:		Start:		
	Finish:		Finish:		
Before reading the plate, always perform prime. Unclog is also needed, except if this is the first plate of the day after the calibration.	Prime ( ) Unclog ( ) or N/Ap ( )		Prime ( ) Unclog ( ) or N/Ap ( )		
If unclog fails, Luminex needle sonicated and unclog repeated	Yes ( )	N/Ap ( )	Yes ( )	N/Ap ( )	
**Read:** Read plate on the Bio-Plex Suspension Assay System.	( )		( )		
Wash between plates was performed	Yes ( )	N/Ap ( )	Yes ( )	N/Ap ( )	
Instrument shutdown was performed	Yes ( )	N/Ap ( )	Yes ( )	N/Ap ( )	

Appendix 2a (AP.BMK.mkpCyt.12)

Cytokine Multiplex Assay sheet (Plate washer)

Study/Reference No: ______________________ Assay I.D.: ______________________

Page: 5 of 10

SCIENTIFIC DATA REVIEW

Assay ID: ______________________

Performed by (init./date):______________________

	**IL-1β or ☐ N/Ap**	**IL-1RA or ☐ N/Ap**	**IL-2 or ☐ N/Ap**	**IL-4 or ☐ N/Ap**	**IL-5 or ☐ N/Ap**	**IL-6 or ☐ N/Ap**	**IL-8 or ☐ N/Ap**	**IL-10 or ☐ N/Ap**
Mean of (FI) Blank < Mean of (FI) LLOQ	Yes or No	Yes or No	Yes or No	Yes or No	Yes or No	Yes or No	Yes or No	Yes or No
Number of working standards within ±25% of the theoretical values (±30% for LLOQ and ULOQ):	/	/	/	/	/	/	/	/
QC samples								
Number of QC1A within acceptance criteria*:	/	/	/	/	/	/	/	/
Number of QC1 within acceptance criteria*:	/	/	/	/	/	/	/	/
Number of QC2 within acceptance criteria*:	/	/	/	/	/	/	/	/
Number of QC3A within acceptance criteria*:	/	/	/	/	/	/	/	/
Number of QC3 within acceptance criteria*:	/	/	/	/	/	/	/	/
Number of tQC within acceptance criteria*:	/	/	/	/	/	/	/	/
Number of beads acquired ≥ 30 in all wells:	Yes or No	Yes or No	Yes or No	Yes or No	Yes or No	Yes or No	Yes or No	Yes or No
Assay is acceptable:	Yes or No	Yes or No	Yes or No	Yes or No	Yes or No	Yes or No	Yes or No	Yes or No
Samples to repeat:	Yes or No or N/Ap	Yes or No or N/Ap	Yes or No or N/Ap	Yes or No or N/Ap	Yes or No or N/Ap	Yes or No or N/Ap	Yes or No or N/Ap	Yes or No or N/Ap

*Concentration within 75-125% of theoretical, %CV ≤ 20% between duplicates.

Appendix 2a (AP.BMK.mkpCyt.12)

Cytokine Multiplex Assay sheet (Plate washer)

Study/Reference No: ______________ Assay I.D.: ______________

Page: 6 of 10

SCIENTIFIC DATA REVIEW

Assay ID: ______________

Performed by (init./date): ______________

	**IL-12/23 (p40) or ☐ N/Ap**	**IL-13 or ☐ N/Ap**	**IL-15 or ☐ N/Ap**	**IL-17A or ☐ N/Ap**	**IL-18 or ☐ N/Ap**	**IFN-γ or ☐ N/Ap**	**TNF-α or ☐ N/Ap**	**MCP-1 or ☐ N/Ap**
Mean of (FI) Blank < Mean of (FI) LLOQ	Yes or No	Yes or No	Yes or No	Yes or No	Yes or No	Yes or No	Yes or No	Yes or No
Number of working standards within ±25% of the theoretical values (±30% for LLOQ and ULOQ):	/	/	/	/	/	/	/	/
QC samples								
Number of QC1A within acceptance criteria*:	/	/	/	/	/	/	/	/
Number of QC1 within acceptance criteria*:	/	/	/	/	/	/	/	/
Number of QC2 within acceptance criteria*:	/	/	/	/	/	/	/	/
Number of QC3A within acceptance criteria*:	/	/	/	/	/	/	/	/
Number of QC3 within acceptance criteria*:	/	/	/	/	/	/	/	/
Number of tQC within acceptance criteria*:	/	/	/	/	/	/	/	/
Number of beads acquired ≥ 30 in all wells:	Yes or No	Yes or No	Yes or No	Yes or No	Yes or No	Yes or No	Yes or No	Yes or No
Assay is acceptable:	Yes or No	Yes or No	Yes or No	Yes or No	Yes or No	Yes or No	Yes or No	Yes or No
Samples to repeat:	Yes or No or N/Ap	Yes or No or N/Ap	Yes or No or N/Ap	Yes or No or N/Ap	Yes or No or N/Ap	Yes or No or N/Ap	Yes or No or N/Ap	Yes or No or N/Ap

*Concentration within 75-125% of theoretical, %CV ≤ 20% between duplicates.

Appendix 2a (AP.BMK.mkpCyt.12)

Appendix 11

Appendix 3

Cytokine Multiplex Assay sheet (Plate washer)

Study/Reference No: ______________________ Assay I.D.: ______________________

Page: 7 of 10

SCIENTIFIC DATA REVIEW

Assay ID: ______________________

Performed by (init./date):______________________

	MIP-1β or ☐ N/Ap	G-CSF or ☐ N/Ap	GM-CSF or ☐ N/Ap
Mean of (FI) Blank < Mean of (FI) LLOQ	Yes or No	Yes or No	Yes or No
Number of working standards within ±25% of the theoretical values (±30% for LLOQ and ULOQ):	/	/	/
QC samples			
Number of QC1A within acceptance criteria*:	/	/	/
Number of QC1 within acceptance criteria*:	/	/	/
Number of QC2 within acceptance criteria*:	/	/	/
Number of QC3A within acceptance criteria*:	/	/	/
Number of QC3 within acceptance criteria*:	/	/	/
Number of tQC within acceptance criteria*:	/	/	/
Number of beads acquired ≥ 30 in all wells:	Yes or No	Yes or No	Yes or No
Assay is acceptable:	Yes or No	Yes or No	Yes or No
Samples to repeat:	Yes or No or N/Ap	Yes or No or N/Ap	Yes or No or N/Ap

*Concentration within 75-125% of theoretical, %CV ≤ 20% between duplicates.

Appendix 2a (AP.BMK.mkpCyt.12)

Appendix 11

Appendix 3

Cytokine Multiplex Assay sheet (Plate washer)

Study/Reference No: ______________________

Assay I.D.: ______________________

Page: 8 of 10

SCIENTIFIC DATA REVIEW

Assay ID: ______________ or ☐ N/Ap

Performed by (init./date): ______________________

	IL-1β or ☐ N/Ap	IL-1RA or ☐ N/Ap	IL-2 or ☐ N/Ap	IL-4 or ☐ N/Ap	IL-5 or ☐ N/Ap	IL-6 or ☐ N/Ap	IL-8 or ☐ N/Ap	IL-10 or ☐ N/Ap
Mean of (FI) Blank < Mean of (FI) LLOQ	Yes or No	Yes or No	Yes or No	Yes or No	Yes or No	Yes or No	Yes or No	Yes or No
Number of working standards within ±25% of the theoretical values (±30% for LLOQ and ULOQ):	/	/	/	/	/	/	/	/
QC samples								
Number of QC1A within acceptance criteria*:	/	/	/	/	/	/	/	/
Number of QC1 within acceptance criteria*:	/	/	/	/	/	/	/	/
Number of QC2 within acceptance criteria*:	/	/	/	/	/	/	/	/
Number of QC3A within acceptance criteria*:	/	/	/	/	/	/	/	/
Number of QC3 within acceptance criteria*:	/	/	/	/	/	/	/	/
Number of tQC within acceptance criteria*:	/	/	/	/	/	/	/	/
Number of beads acquired ≥ 30 in all wells:	Yes or No	Yes or No	Yes or No	Yes or No	Yes or No	Yes or No	Yes or No	Yes or No
Assay is acceptable:	Yes or No	Yes or No	Yes or No	Yes or No	Yes or No	Yes or No	Yes or No	Yes or No
Samples to repeat:	Yes or No or N/Ap	Yes or No or N/Ap	Yes or No or N/Ap	Yes or No or N/Ap	Yes or No or N/Ap	Yes or No or N/Ap	Yes or No or N/Ap	Yes or No or N/Ap

*Concentration within 75-125% of theoretical, %CV ≤ 20% between duplicates.

Appendix 2a (AP.BMK.mkpCyt.12)

Appendix 11

Appendix 3

Cytokine Multiplex Assay sheet (Plate washer)

Study/Reference No: ______________________ Assay I.D.: ______________________

Page: 9 of 10

SCIENTIFIC DATA REVIEW

Assay ID: ______________________ **or ☐ N/Ap**

Performed by (init./date):______________________

	**IL-12/23 (p40) or ☐ N/Ap**	**IL-13 or ☐ N/Ap**	**IL-15 or ☐ N/Ap**	**IL-17A or ☐ N/Ap**	**IL-18 or ☐ N/Ap**	**IFN-γ or ☐ N/Ap**	**TNF-α or ☐ N/Ap**	**MCP-1 or ☐ N/Ap**
Mean of (FI) Blank < Mean of (FI) LLOQ	Yes or No	Yes or No	Yes or No	Yes or No	Yes or No	Yes or No	Yes or No	Yes or No
Number of working standards within ±25% of the theoretical values (±30% for LLOQ and ULOQ):	/	/	/	/	/	/	/	/
QC samples								
Number of QC1A within acceptance criteria*:	/	/	/	/	/	/	/	/
Number of QC1 within acceptance criteria*:	/	/	/	/	/	/	/	/
Number of QC2 within acceptance criteria*:	/	/	/	/	/	/	/	/
Number of QC3A within acceptance criteria*:	/	/	/	/	/	/	/	/
Number of QC3 within acceptance criteria*:	/	/	/	/	/	/	/	/
Number of tQC within acceptance criteria*:	/	/	/	/	/	/	/	/
Number of beads acquired ≥ 30 in all wells:	Yes or No	Yes or No	Yes or No	Yes or No	Yes or No	Yes or No	Yes or No	Yes or No
Assay is acceptable:	Yes or No	Yes or No	Yes or No	Yes or No	Yes or No	Yes or No	Yes or No	Yes or No
Samples to repeat:	Yes or No or N/Ap	Yes or No or N/Ap	Yes or No or N/Ap	Yes or No or N/Ap	Yes or No or N/Ap	Yes or No or N/Ap	Yes or No or N/Ap	Yes or No or N/Ap

*Concentration within 75-125% of theoretical, %CV ≤ 20% between duplicates.

Appendix 2a (AP.BMK.mkpCyt.12)

Appendix 11

Appendix 3

Cytokine Multiplex Assay sheet (Plate washer)

Study/Reference No: ______________________ Assay I.D.: ______________________

Page: 10 of 10

SCIENTIFIC DATA REVIEW

Assay ID: ____________________ **or ☐ N/Ap**

Performed by (init./date):__________________________

	**MIP-1β or ☐ N/Ap**	**G-CSF or ☐ N/Ap**	**GM-CSF or ☐ N/Ap**
Mean of (FI) Blank < Mean of (FI) LLOQ	Yes or No	Yes or No	Yes or No
Number of working standards within ±25% of the theoretical values (±30% for LLOQ and ULOQ):	/	/	/
QC samples			
Number of QC1A within acceptance criteria*:	/	/	/
Number of QC1 within acceptance criteria*:	/	/	/
Number of QC2 within acceptance criteria*:	/	/	/
Number of QC3A within acceptance criteria*:	/	/	/
Number of QC3 within acceptance criteria*:	/	/	/
Number of tQC within acceptance criteria*:	/	/	/
Number of beads acquired ≥ 30 in all wells:	Yes or No	Yes or No	Yes or No
Assay is acceptable:	Yes or No	Yes or No	Yes or No
Samples to repeat:	Yes or No or N/Ap	Yes or No or N/Ap	Yes or No or N/Ap

*Concentration within 75-125% of theoretical, %CV ≤ 20% between duplicates.

Appendix #2 Reviewed by/date: ______________________

Appendix 2a (AP.BMK.mkpCyt.12)

Appendix 11

Appendix 3

Cytokine Multiplex Assay sheet (Manual wash)

Study/Reference No: ____________ Assay I.D.: ____________

Page: 1 of 10

Reagents/ Working Solutions					
Name	***Batch or Lot #***	***Inventory number***		***Expiry Date***	***Entered by /Date***
		Assay ID:	Assay ID: or ☐N/Ap		
Non-Human primate Cytokine kit					
Non Human primate Detection Antibodies					
Assay Buffer					
Bead diluent					
Streptavidin-Phycoerythrin					
Antibody-Immobilized Beads Working solution	ABWS-	N/Ap	N/Ap		
Wash Buffer Cytokines	mkpCYT WB-				
Serum matrix	mkpSM-	N/Ap	N/Ap		
Sheath fluid					
UPW	N/Ap	N/Ap	N/Ap		

PLATE SEQUENCE (Printed from Watson)

**In-Process Sample Storage**	**Performed (√)**		**Start time**	**Performed by / Date**
**Assay ID: or N/Ap ☐**				
Samples transported from Sample Management	Dry Ice	( )	N/Ap	
Samples thawed	Wet ice	( )		
Samples placed in temporary storage after use and until returned to Sample Management	Dry Ice	( )		
**In-Process Sample Storage**	**Performed (√)**		**Start time**	**Performed by / Date**
**Assay ID: or N/Ap ☐**				
Samples transported from Sample Management	Dry Ice	( )	N/Ap	
Samples thawed	Wet ice	( )		
Samples placed in temporary storage after use and until returned to Sample Management	Dry Ice	( )		

Appendix 2b (AP.BMK.mkpCyt.12)

Appendix 11

Appendix 3

Cytokine Multiplex Assay sheet (Manual wash)

Study/Reference No: ______________ Assay I.D.: ______________

Page: 2 of 10

INSTRUMENTS		
Name	***ID***	***Entered by / Date***
Pipettes		
Multichannel pipette		
Sonicating bath		
Titer plate shaker set at 600 rpm		
Orbital plate shaker (orbit: 4 mm) set at 600 rpm		
Bio-Plex Suspension Array		

Comments: ______________

Appendix 2b (AP.BMK.mkpCyt.12)

Appendix 11

Appendix 3

Cytokine Multiplex Assay sheet (Manual wash)

Study/Reference No: ____________ Assay I.D.: ____________

Page: 3 of 10

Steps	*Time / Performed (√)* Assay ID:	*Time / Performed (√)* Assay ID: or N/Ap ☐	*Performed by / Date*
Allow reagents to reach ambient RT before starting the assay.	( )	( )	
**Plate Preparation:** add 200 µL of Assay Buffer into each well of the microtiter plate. Cover the filter plate with an aluminum foil-wrapped plate cover and mix on a plate shaker set at 600 rpm at ambient RT for at least 10 minutes.	Start:<br>Finish:	Start:<br>Finish:	
Prepare Standards and QCs at ambient RT and tQCs on wet ice, in polypropylene tubes.	( )	( )	
Prepare diluted samples and keep on wet ice until loading as per Appendix #5	( ) or N/Ap ( )	( )	
Remove Assay buffer by decanting manually and blot dry on absorbent papers.	( )	( )	
**Load:** Add 25 µL of each Standard, QCs and samples (if applicable) into the appropriate wells (preloading can be performed if desired).	Preload( ) or N/Ap ( )<br>Load ( )	Preload( ) or N/Ap ( )<br>Load ( )	
**Load:** Add 25 µL of Assay Buffer in all wells.	( )	( )	
Sonicate for 30 seconds and vortex for 1 minute each bead vial used in the assay.<br>Prepare the Antibody-Immobilized Beads Working solution (code: ABWS).	( )	( )	
**Beads:** Vortex beads bottle and add 25 µL of beads to each well.	( )	( )	
Cover the plate with an aluminum foil plate cover (protected from light), and incubate on a plate shaker set at 600 rpm at ambient RT for 2 hours (± 5 minutes).	Start:<br>Finish:	Start:<br>Finish:	
**Manual Wash:** Rest plate on magnet for at least 60 seconds. Remove well contents by gently decanting the plate and gently tapping on absorbent paper. Remove plate from magnet, add 200µL/well of Wash Buffer (Code: **mkpCYT WB**) and shake for approx. 30 seconds on a plate shaker set at 600 rpm.<br>Repeat the washing steps 2 times (total of 3 wash). Plate should not be allowed to dry.	1st wash ( )<br>2nd wash ( )<br>3rd wash ( )	1st wash ( )<br>2nd wash ( )<br>3rd wash ( )	
**Capture:** Add 25 µL of detection antibodies into each well.	( )	( )	
Cover the plate with an aluminum foil plate cover (protected from light), and incubate 1 hour (± 2 minutes) on a plate shaker set at 600 rpm at ambient RT. **DO NOT EMPTY THE WELL AFTER INCUBATION.**	Start:<br>Finish:	Start:<br>Finish:	
**Detection:** Add 25 µL of streptavidin-phycoerythrin to each well.	( )	( )	
Cover the plate with an aluminum foil plate cover (protected from light) and incubate on a plate shaker set at 600 rpm at ambient RT for 30 minutes (± 1 minute).	Start:<br>Finish:	Start:<br>Finish:	

Appendix 2b (AP.BMK.mkpCyt.12)

Appendix 11

Appendix 3

Cytokine Multiplex Assay sheet (Manual wash)

Study/Reference No: ____________ Assay I.D.: ____________

Page: 4 of 10

Steps	*Time / Performed (√)*		*Time / Performed (√)*		*Performed by / Date*
	Assay ID:		Assay ID: or N/Ap ☐		
**Manual Wash:** Rest plate on magnet for at least 60 seconds. Remove well contents by gently decanting the plate and gently tapping on absorbent paper. Remove plate from magnet, add 200µL/well of Wash Buffer (Code: **mkpCYT WB**) and shake for approx. 30 seconds on a plate shaker set at 600 rpm. Repeat the washing steps 2 times (total of 3 wash). Plate should not be allowed to dry.	1st wash ( )<br>2nd wash ( )<br>3rd wash ( )		1st wash ( )<br>2nd wash ( )<br>3rd wash ( )		
Add **150 µL** of Sheath fluid to all wells. Cover the filter plate with an aluminum foil-wrapped plate cover and resuspend the beads by shaking on a plate shaker set at 600 rpm at ambient RT for at least 5 minutes.	Start:<br>Finish:		Start:<br>Finish:		
Before reading the plate, always perform prime. Unclog is also needed, <u>except</u> if this is the first plate of the day after the calibration.	Prime ( )<br>Unclog ( ) or N/Ap ( )		Prime ( )<br>Unclog ( ) or N/Ap ( )		
If unclog fails, Luminex needle sonicated and unclog repeated	Yes ( )	N/Ap ( )	Yes ( )	N/Ap ( )	
**Read:** Read plate on the Bio-Plex Suspension Assay System.	( )		( )		
Wash between plates was performed	Yes ( )	N/Ap ( )	Yes ( )	N/Ap ( )	
Instrument shutdown was performed	Yes ( )	N/Ap ( )	Yes ( )	N/Ap ( )	

Appendix 2b (AP.BMK.mkpCyt.12)

Appendix 11

Appendix 3

Cytokine Multiplex Assay sheet (Manual wash)

Study/Reference No: ______________________ Assay I.D.: ______________________

Page: 5 of 10

SCIENTIFIC DATA REVIEW

Assay ID: ______________________

Performed by (init./date): ______________________

	IL-1β or ☐ N/Ap	IL-1RA or ☐ N/Ap	IL-2 or ☐ N/Ap	IL-4 or ☐ N/Ap	IL-5 or ☐ N/Ap	IL-6 or ☐ N/Ap	IL-8 or ☐ N/Ap	IL-10 or ☐ N/Ap
Mean of (FI) Blank < Mean of (FI) LLOQ	Yes or No	Yes or No	Yes or No	Yes or No	Yes or No	Yes or No	Yes or No	Yes or No
Number of working standards within ±25% of the theoretical values (±30% for LLOQ and ULOQ):	/	/	/	/	/	/	/	/
QC samples								
Number of QC1A within acceptance criteria*:	/	/	/	/	/	/	/	/
Number of QC1 within acceptance criteria*:	/	/	/	/	/	/	/	/
Number of QC2 within acceptance criteria*:	/	/	/	/	/	/	/	/
Number of QC3A within acceptance criteria*:	/	/	/	/	/	/	/	/
Number of QC3 within acceptance criteria*:	/	/	/	/	/	/	/	/
Number of tQC within acceptance criteria*:	/	/	/	/	/	/	/	/
Number of beads acquired ≥ 30 in all wells:	Yes or No	Yes or No	Yes or No	Yes or No	Yes or No	Yes or No	Yes or No	Yes or No
Assay is acceptable:	Yes or No	Yes or No	Yes or No	Yes or No	Yes or No	Yes or No	Yes or No	Yes or No
Samples to repeat:	Yes or No or N/Ap	Yes or No or N/Ap	Yes or No or N/Ap	Yes or No or N/Ap	Yes or No or N/Ap	Yes or No or N/Ap	Yes or No or N/Ap	Yes or No or N/Ap

*Concentration within 75-125% of theoretical, %CV ≤ 20% between duplicates.

Appendix 2b (AP.BMK.mkpCyt.12)

Appendix 11

Appendix 3

Cytokine Multiplex Assay sheet (Manual wash)

Study/Reference No: ____________________ Assay I.D.: ____________________

Page: 6 of 10

SCIENTIFIC DATA REVIEW

Assay ID: ____________________

Performed by (init./date):____________________

	IL-12/23 (p40) or ☐ N/Ap	IL-13 or ☐ N/Ap	IL-15 or ☐ N/Ap	IL-17A or ☐ N/Ap	IL-18 or ☐ N/Ap	IFN-γ or ☐ N/Ap	TNF-α or ☐ N/Ap	MCP-1 or ☐ N/Ap
Mean of (FI) Blank < Mean of (FI) LLOQ	Yes or No	Yes or No	Yes or No	Yes or No	Yes or No	Yes or No	Yes or No	Yes or No
Number of working standards within ±25% of the theoretical values (±30% for LLOQ and ULOQ):	/	/	/	/	/	/	/	/
QC samples								
Number of QC1A within acceptance criteria*:	/	/	/	/	/	/	/	/
Number of QC1 within acceptance criteria*:	/	/	/	/	/	/	/	/
Number of QC2 within acceptance criteria*:	/	/	/	/	/	/	/	/
Number of QC3A within acceptance criteria*:	/	/	/	/	/	/	/	/
Number of QC3 within acceptance criteria*:	/	/	/	/	/	/	/	/
Number of tQC within acceptance criteria*:	/	/	/	/	/	/	/	/
Number of beads acquired ≥ 30 in all wells:	Yes or No	Yes or No	Yes or No	Yes or No	Yes or No	Yes or No	Yes or No	Yes or No
Assay is acceptable:	Yes or No	Yes or No	Yes or No	Yes or No	Yes or No	Yes or No	Yes or No	Yes or No
Samples to repeat:	Yes or No or N/Ap	Yes or No or N/Ap	Yes or No or N/Ap	Yes or No or N/Ap	Yes or No or N/Ap	Yes or No or N/Ap	Yes or No or N/Ap	Yes or No or N/Ap

*Concentration within 75-125% of theoretical, %CV ≤ 20% between duplicates.

Appendix 2b (AP.BMK.mkpCyt.12)

Appendix 11

Appendix 3

Cytokine Multiplex Assay sheet (Manual wash)

Study/Reference No: ______________________ Assay I.D.: ______________________

Page: 7 of 10

SCIENTIFIC DATA REVIEW

Assay ID: ______________________

Performed by (init./date): ______________________

	MIP-1β or ☐ N/Ap	G-CSF or ☐ N/Ap	GM-CSF or ☐ N/Ap
Mean of (FI) Blank < Mean of (FI) LLOQ	Yes or No	Yes or No	Yes or No
Number of working standards within ±25% of the theoretical values (±30% for LLOQ and ULOQ):	/	/	/
QC samples			
Number of QC1A within acceptance criteria*:	/	/	/
Number of QC1 within acceptance criteria*:	/	/	/
Number of QC2 within acceptance criteria*:	/	/	/
Number of QC3A within acceptance criteria*:	/	/	/
Number of QC3 within acceptance criteria*:	/	/	/
Number of tQC within acceptance criteria*:	/	/	/
Number of beads acquired ≥ 30 in all wells:	Yes or No	Yes or No	Yes or No
Assay is acceptable:	Yes or No	Yes or No	Yes or No
Samples to repeat:	Yes or No or N/Ap	Yes or No or N/Ap	Yes or No or N/Ap

*Concentration within 75-125% of theoretical, %CV ≤ 20% between duplicates.

Appendix 2b (AP.BMK.mkpCyt.12)

Appendix 11

Appendix 3

Cytokine Multiplex Assay sheet (Manual wash)

Study/Reference No: ______________________ Assay I.D.: ______________________

Page: 8 of 10

SCIENTIFIC DATA REVIEW

Assay ID: ______________or ☐ N/Ap

Performed by (init./date):______________________

	IL-1β or ☐ N/Ap	IL-1RA or ☐ N/Ap	IL-2 or ☐ N/Ap	IL-4 or ☐ N/Ap	IL-5 or ☐ N/Ap	IL-6 or ☐ N/Ap	IL-8 or ☐ N/Ap	IL-10 or ☐ N/Ap
Mean of (FI) Blank < Mean of (FI) LLOQ	Yes or No	Yes or No	Yes or No	Yes or No	Yes or No	Yes or No	Yes or No	Yes or No
Number of working standards within ±25% of the theoretical values (±30% for LLOQ and ULOQ):	/	/	/	/	/	/	/	/
QC samples								
Number of QC1A within acceptance criteria*:	/	/	/	/	/	/	/	/
Number of QC1 within acceptance criteria*:	/	/	/	/	/	/	/	/
Number of QC2 within acceptance criteria*:	/	/	/	/	/	/	/	/
Number of QC3A within acceptance criteria*:	/	/	/	/	/	/	/	/
Number of QC3 within acceptance criteria*:	/	/	/	/	/	/	/	/
Number of tQC within acceptance criteria*:	/	/	/	/	/	/	/	/
Number of beads acquired ≥ 30 in all wells:	Yes or No	Yes or No	Yes or No	Yes or No	Yes or No	Yes or No	Yes or No	Yes or No
Assay is acceptable:	Yes or No	Yes or No	Yes or No	Yes or No	Yes or No	Yes or No	Yes or No	Yes or No
Samples to repeat:	Yes or No or N/Ap	Yes or No or N/Ap	Yes or No or N/Ap	Yes or No or N/Ap	Yes or No or N/Ap	Yes or No or N/Ap	Yes or No or N/Ap	Yes or No or N/Ap

*Concentration within 75-125% of theoretical, %CV ≤ 20% between duplicates.

Appendix 2b (AP.BMK.mkpCyt.12)

Appendix 11

Appendix 3

Cytokine Multiplex Assay sheet (Manual wash)

Study/Reference No: ______________________ Assay I.D.: ______________________

Page: 9 of 10

SCIENTIFIC DATA REVIEW

Assay ID: ____________________ **or ☐ N/Ap**

Performed by (init./date):____________________

	**IL-12/23 (p40) or ☐ N/Ap**	**IL-13 or ☐ N/Ap**	**IL-15 or ☐ N/Ap**	**IL-17A or ☐ N/Ap**	**IL-18 or ☐ N/Ap**	**IFN-γ or ☐ N/Ap**	**TNF-α or ☐ N/Ap**	**MCP-1 or ☐ N/Ap**
Mean of (FI) Blank < Mean of (FI) LLOQ	Yes or No	Yes or No	Yes or No	Yes or No	Yes or No	Yes or No	Yes or No	Yes or No
Number of working standards within ±25% of the theoretical values (±30% for LLOQ and ULOQ):	/	/	/	/	/	/	/	/
QC samples								
Number of QC1A within acceptance criteria*:	/	/	/	/	/	/	/	/
Number of QC1 within acceptance criteria*:	/	/	/	/	/	/	/	/
Number of QC2 within acceptance criteria*:	/	/	/	/	/	/	/	/
Number of QC3A within acceptance criteria*:	/	/	/	/	/	/	/	/
Number of QC3 within acceptance criteria*:	/	/	/	/	/	/	/	/
Number of tQC within acceptance criteria*:	/	/	/	/	/	/	/	/
Number of beads acquired ≥ 30 in all wells:	Yes or No	Yes or No	Yes or No	Yes or No	Yes or No	Yes or No	Yes or No	Yes or No
Assay is acceptable:	Yes or No	Yes or No	Yes or No	Yes or No	Yes or No	Yes or No	Yes or No	Yes or No
Samples to repeat:	Yes or No or N/Ap	Yes or No or N/Ap	Yes or No or N/Ap	Yes or No or N/Ap	Yes or No or N/Ap	Yes or No or N/Ap	Yes or No or N/Ap	Yes or No or N/Ap

*Concentration within 75-125% of theoretical, %CV ≤ 20% between duplicates.

Appendix 2b (AP.BMK.mkpCyt.12)

Appendix 11

Appendix 3

Cytokine Multiplex Assay sheet (Manual wash)

Study/Reference No: ______________________ Assay I.D.: ______________________

Page: 10 of 10

SCIENTIFIC DATA REVIEW

Assay ID: ____________ **or ☐ N/Ap**

Performed by (init./date):____________________

	**MIP-1β or ☐ N/Ap**	**G-CSF or ☐ N/Ap**	**GM-CSF or ☐ N/Ap**
Mean of (FI) Blank < Mean of (FI) LLOQ	Yes or No	Yes or No	Yes or No
Number of working standards within ±25% of the theoretical values (±30% for LLOQ and ULOQ):	/	/	/
QC samples			
Number of QC1A within acceptance criteria*:	/	/	/
Number of QC1 within acceptance criteria*:	/	/	/
Number of QC2 within acceptance criteria*:	/	/	/
Number of QC3A within acceptance criteria*:	/	/	/
Number of QC3 within acceptance criteria*:	/	/	/
Number of tQC within acceptance criteria*:	/	/	/
Number of beads acquired ≥ 30 in all wells:	Yes or No	Yes or No	Yes or No
Assay is acceptable:	Yes or No	Yes or No	Yes or No
Samples to repeat:	Yes or No or N/Ap	Yes or No or N/Ap	Yes or No or N/Ap

*Concentration within 75-125% of theoretical, %CV ≤ 20% between duplicates.

Appendix #2 Reviewed by/date: ____________________

Appendix 2b (AP.BMK.mkpCyt.12)

Appendix 11

Appendix 3

Daily Solution Preparation Sheet

Study/Reference no: ________________ Assay ID: ________________________

Preparation of: **Antibody-Immobilized Beads Working Solution (code: ABWS)**

Note: if not all cytokines are needed to be analyzed, Bead Diluent should be used to replace the missing antibody-bead volume. The total volume of the solution needs to be 3.000 mL. (Dilution 1/50)

Batch #	Reagents**	Lot/Batch no	Inventory Number	*Calculated Volume units (µL)	Actual Volume units (µL)	Performed by & Date	*Calculations verified by/date:
	Bead Diluent						
	IL-1β Beads						
	IL-1RA Beads						
	IL-6 Beads						
	IL-10 Beads						
	IL-12/23 (p40) Beads						
	IL-15 Beads						
	IL-18 Beads						
	IFN-γ Beads						
	TNF-α Beads						
	MCP-1 Beads						
	MIP-1β Beads						
	G-CSF Beads						
	GM-CSF						
	IL-2						
	IL-4						
	IL-5						
	IL-8						
	IL-13						
	IL-17A						
				Total volume:			

* Applicable only when the volumes are scaled up or down.

** Note: the antibody-beads bottles should be sonicated for 30 seconds and then vortex for 1 minute prior to the preparation of the ABWS (refer to AP).

Reviewed by/date: ________________

Appendix #3 (AP.BMK.mkpCyt.12)

Appendix 11

Appendix 3

Solution Preparation Sheet

Study/Reference no: ____________

Preparation **Wash Buffer Cytokines (Code: mkpCYTWB)**

Storage Location: ____________ or discarded after use ☐ Solution Expiration date: ________

Batch no	Reagents	Lot no	Expiry date	Inventory Number	*Calculated Volume units	Actual Volume units	Prepared by & Date	*Calculations verified by/date:
mkpCYTWB/	Wash Buffer Concentrate (10X)							
	Ultra Pure Water	N/Ap		N/Ap				
				Total volume:				

*Applicable only when the volumes are scaled up or down

Comments ____________

Reviewed by/Date: ____________

Appendix #4 (AP.BMK.mkpCyt.12)

Appendix 11

Appendix 3

Assay Instructions Sheet

Study/Reference No.:_______________________

Assay ID:_____________________

Lot of Kit and lot of STD stock to be used:________________________

Kit and STD qualified in assay ID:_______________________

- Analyze samples on wet ice and diluted as per provided dilution sheet

Table 1 The following tQC should be use for the assay

QC Identification	Monkey Plasma lot#	Location in a freezer set to maintain -80°C or -20°C	tQC concentration	Source assay and study ID	Source Assay Date[a]
tQC					

[a] Refer to AP for QC samples stability. **The expiry date on the label of the neat matrix should be SIP.**
tQC is to be prepared as per Appendix #6 and/or Dilution sheet.

Table 2 Cytokine concentrations

	Cytokine Concentrations (pg/mL)				
Standards ID	IL-1β, IL-1RA, IL-6, IL-15, IFN-γ, TNF-α, MIP-1β, IL-2, IL-5, IL-8, IL-13 and IL-17A	MCP-1	IL-12/23 (p40), G-CSF, GM-CSF[f]	IL-10, IL-18	IL-4
**STD 9 (ULOQ2)**	**6000.00**	6000.00[b]	**6000.00**	**30000.00**	**12000.00**
STD 8	4800.00	4800.00[b]	4800.00	24000.00	9600.00
**STD 7 (ULOQ1)**	2400.00	**2400.00**	2400.00	12000.00	4800.00
STD 6	1200.00	1200.00	1200.00	6000.00	2400.00
STD 5	600.00	600.00	600.00	3000.00	1200.00
STD 4	300.00	300.00	300.00	1500.00	600.00
STD 3	150.00	150.00	150.00	750.00	300.00
**STD 2 (LLOQ2)**	75.00	75.00	**75.00**	375.00	**150.00**
**STD 1 (LLOQ1)**	**37.50**	**37.50**	37.50[d]	**187.50**	75.00[d]
STD 0	0.00	0.00	0.00	0.00	0.00
QCs ID					
QC3	**3200.00**	3200.00	**3200.00**	**16000.00**	**6400.00**
QC3A	1600.00	**1600.00**	1600.00	8000.00	3200.00
QC2	**1000.00**	**1000.00[e]**	**1000.00**	**5000.00**	**2000.00**
QC1	200.00	200.00	**200.00**	1000.00	**400.00[c]**
QC1A	**100.00**	**100.00**	100.00	**500.00**	200.00
LLOQ Threshold (pg/mL)					
ULOQ Threshold (pg/mL)					

b) Should not be included in the standard curve setting for MCP-1.
c) For IL-4, LLOQ set at 75.00 and 150.00 pg/mL and QC1A set at 200.00 pg/mL failed to meet the acceptance criteria, for study samples analysis, range of the curve will be 150.00 to 12000.00 pg/mL but all samples <400.00 pg/mL will have to be reported as <LLOQ (LLOQ= 400.0 pg/mL).
d) Accessory standard

Appendix 11

Appendix 3

Assay Instructions Sheet

Study/Reference No.:______________________

e) Out of the range requested by the validation Study plan, refer to note added in section 6.2.2 Preparation of standards and QCs

f) For GM-CSF, if selected as a Single plex, low QC1 and LLOQ should be increased as failure of the low QC during the precision and relative accuracy assessment was high compared to the other cytokines.
Bold concentrations reflect the appropriate levels for each cytokine (LLOQ, ULOQ and QCs)

Table 3 List of cytokines to be analyzed

IL-1β ☐	IL-15 ☐
IL-1RA ☐	IL-17A ☐
IL-2 ☐	IL-18 ☐
IL-4 ☐	IFN-γ ☐
IL-5 ☐	TNF-α ☐
IL-6☐	MCP-1 ☐
IL-8☐	MIP-1β ☐
IL-10☐	G-CSF ☐
IL-12/23 (p40)☐	GM-CSF ☐
IL-13 ☐	

- Assay should be performed using the automatic plate washer (Appendix #2a) ☐ ***or*** manual handheld magnetic washer (Appendix #2b) ☐

Comments: ____
____
____

Verified by/date: ______________________

Reviewed by/date: ______________________

Appendix 11

Appendix 3

Cytokine Multiplex Spiking Sheet Preparation of tQC

Study/Reference no/Assay ID: ____________________

	Target Concentration (pg/mL)				Stock Concentration (pg/mL)				Stock		2-fold diluted Lot # XXXXXX[a]		Calculated Concentration (pg/mL)		
Sample Identification	IL-1β, IL-1RA, IL-6, IL-12/23 (p40), IL-15, TNF-α, IFN-γ, G-CSF, MCP-1, MIP-1β, GM-CSF, IL-2, IL-5, IL-8, IL-13 and IL-17A	IL-10, Il-18	IL-4	Stock Identification	IL-1β, IL-1RA, IL-6, IL-12/23 (p40), IL-15, TNF-α, IFN-γ, G-CSF, MCP-1, MIP-1β, GM-CSF, IL-2, IL-5, IL-8, IL-13 and IL-17A	IL-10, IL-18	IL-4	Total Volume (µL)	Volume (µL)	Performed (√)	Volume (µL)	Performed (√)	IL-1β, IL-1RA, IL-6, IL-12/23 (p40), IL-15, TNF-α, IFN-γ, G-CSF, MCP-1, MIP-1β, GM-CSF, IL-2, IL-5, IL-8, IL-13 and IL-17A	IL-10, IL-18	IL-4
**Stock[b]**	**10000.00**	**50000.00**	**20000.00**	Stock b	10000.00	50000.00	20000.00	N/Ap	N/Ap	N/Ap	N/Ap	N/Ap	10000.00	50000.00	20000.00
QC Stock	2500.00	12500.00	5000.00	Stock b	10000.00	50000.00	20000.00	200	50	( )	150	( )	2500.00	12500.00	5000.00
tQC	1250.00	6250.00	2500.00	QC Stock	2500.00	12500.00	5000.00	350	175	( )	175	( )	1250.00	6250.00	2500.00

Comments: a= Refer to dilution sheet for the preparation of the 2-fold dilution.
b= Refer to Appendix #1

Verified by/ date: ____________________ Spiking performed by/ date: ____________________

Calculations verified by/ date: ____________________ Reviewed by/ date: ____________________

Appendix 6 (AP.BMK.mkpCyt.12)

Appendix 11

Appendix 3

Sample Analysis Instruction Sheet

Study/Reference no: ______________

Assay ID: ______________

	Cytokine(s) to be analyzed for each sample ID																		
Sample ID	IL-1β	IL-1RA	IL-2	IL-4	IL-5	IL-6	IL-8	IL-10	IL-12/23 (p40)	IL-13	IL-15	IL-17A	IL-18	IFN-γ	TNF-α	MCP-1	MIP-1β	G-CSF	GM-CSF

✓ = To be analyzed

Comment(s): ____

Verified by / Date: ____________________ Reviewed by / Date : ____________________

Time: ____________________

Appendix #7 (AP.BMK.mkpCyt.12)

Appendix 11

Appendix 4

charles river

Title: **QUANTITATION OF IP-10 (CXCL10) IN CYNOMOLGUS MONKEY PLASMA BY AN ENZYME LINKED IMMUNOSORBENT ASSAY (ELISA)**	AP Number: AP.BMK.mkpIP10.09	Effective Date: Signature of AP
	CR SEN/SHB	Supersedes: 30 Jun 2021
Approved by: Marie-Eve Rodrigue, Senior Research Scientist I, Biomarkers — MERodrigue		Date: 05 Aug 2021
Verified by: Sonia Ménard, Research Scientist II, Biomarkers — [signature]		Date: 05 Aug 2021
Authorized by: Julie Fontaine, Senior Research Scientist I, Biomarkers — Julie Fontaine		Date: 05 Aug 2021

1.0 Purpose

The purpose of this assay is to determine the concentration of IP-10 (or alternately known as CXCL10) in Cynomolgus monkey plasma by ELISA.

2.0 Scope

This procedure applies to IP-10 in Cynomolgus plasma assays undertaken in the Biomarkers department

3.0 Responsibilities

All staff performing this assay are responsible for compliance with this analytical procedure.

4.0 Required forms

Appendix #1: Plasma IP-10 ELISA Assay sheet

Appendix #2: Plasma IP-10 ELISA Spiking Sheet (Kit Standard) *(Example of spreadsheet)*

Appendix #3: IP-10 in Monkey Plasma Assay Instructions *(Example of document)*

Appendix #4: Plasma IP-10 ELISA Spiking Sheet (Monkey Reference Standard)*(Example of spreadsheet)*

5.0 Materials/Equipment/Reagents

Notes:

- *The procedure may require other general laboratory supplies commonly used in laboratory science. Materials can be substituted (with the exception of the kit components or the monkey reference standard) provided the same specifications are met.*
- *If the lot # of a reagent is recorded, the recording of the inventory # is not critical, since it is considered to be for information or reference purposes only. The only situation that would make the inventory # critical is for reagents that have an expiry date based on the receipt date.*

5.1 Materials/Equipment

5.1.1 Non Disposable/Instrumentation
Repeater pipette
Pipettes (multi-channel, micro)
Vortex mixer
Spectrophotometer plate reader
SOFTmax PRO GxP software
Watson Laboratory Information Management System (LIMS)
Analytical balance

5.1.2 Disposable
Aluminum foil
Tubes (polypropylene, various caps, various size)
Ultra pure water (UPW)
Pipette tips (various size)
Non-sterile solution basins
Parafilm
Eppendorf combitips (various capacity)
Absorbent paper
Auto-sticker plate sealers
96 wells plate, U-bottom, polypropylene (used for pre-loading)
0.2 or 0.22 µm filters

5.2 Kit Components

Notes:

i. Concentrations of kit components/ reagents may vary from lot to lot.
ii. When possible, the same kit/Monkey Reference Standard lot should be used for the entire study to reduce any potential assay variability due to change of lot. Any new kit lot should be qualified as per S.O.P. BMK-004 prior to use. In the event where the same lot of kit cannot be used throughout the study and lot to lot variability is present, this should be considered in the interpretation of the results.
ii. All kit reagents should be stored in a refrigerator set to maintain 4°C.

AP Number: AP.BMK.mkpIP10.09	Effective Date: Signature of AP	Supersedes: 30 Jun 2021

Quantikine Human CXCL10/IP-10, cat # SIP100 (R&D Systems)

- IP-10 Microplate, 6 units (Part 890834)
- IP-10 conjugate, (Part 890835), 6 x 21 mL
- IP-10 standard: To be reconstituted at 5000 pg/mL, (Part 890836), 6 vials
- Assay diluent RD1-56, (Part 895102), 6 x 17 mL
- Calibrator diluent RD5K, (Part 895119), 6 x 21 mL **(Do not use)**
- Calibrator diluent RD6Q, (Part 895128), 6 x 21 mL
- Wash buffer concentrate 25X, (Part 895003), 6 x 21 mL **(Do not use)**
- Color reagent A, (part 895000), 6 x 12.5 mL
- Color reagent B, (part 895001), 6 x 12.5 mL
- Stop solution (2N Sulfuric acid), (part 895032), 6 x 6 mL

Note: Catalog number SIP100 contains the equivalent of 6 kits. This catalog number can be substituted by DIP100 (1 kit) or PDIP100 (50 kits).Note that when PDIP100 is used, the IP-10 standard should be used for more than one assay.

5.3 Monkey Reference Standard

Reagents	Supplier	Catalogue #
Recombinant rRhIP-10/ CXCL10 (5µg)	Bio Basic	RC322-21

5.4 Other reagents

Reagents	Supplier	Catalogue #
TBS-Tween buffer pouches (pH 8.0)	Sigma	T9039
1X PBS, pH 7.2	Gibco	20012
Bovine serum albumin (BSA)	Sigma	A6003

5.5 Endogenous Matrix (used to prepare trending QC)

Identity: Cynomolgus monkey plasma
Anticoagulant: EDTA
Storage: In a freezer set to maintain -20°C or -80°C
Expiry Date: SIP (stability in progress)

6.0 Preparation of Assay Reagents

Notes:

i. Volumes described for any preparation may be scaled up or down proportionally as long as the target concentration is not changed. All changes must be documented in raw data.
ii. Bring all reagents to ambient RT before use.
iii. Complete Appendix #1, #2 or #4 of this AP or Appendix #1 or #2 of SOP CACI-001 whichever is applicable, for each solution preparation.

AP Number: AP.BMK.mkpIP10.09	Effective Date: Signature of AP	Supersedes: 30 Jun 2021

6.1 Preparation of IP-10 Standards and Quality Control (QC) Samples

Note: Standard curve should be prepared by using the monkey reference standard (Bio Basic Cat# RC322-21). However, the Human IP-10 standard provided in the kit can also be used. The Standard to be used will be documented on Appendix #3.

6.1.1 Reconstitution of Recombinant rRhIP-10/CXCL10 Standard (code: rRhIP-10 STD) using the monkey reference standard

- Once thawed, quick spin the original vial of Recombinant rRhIP-10/CXCL10 (5µg) to allow the content to be at the bottom of the vial.
- Reconstitute 1 Recombinant rRhIP-10/CXCL10 vial (from Bio Basic) at a final concentration of 10 µg/mL (add 500 µL of 0.1%BSA to a vial of 5 µg of Recombinant rRhIP-10/CXCL10) at ambient RT.
- Aliquot the solution (recommended volume 20 µL per aliquot) into appropriately labeled microtubes and store in a freezer set to maintain -80°C with SIP as the expiry date.
- Record preparation on Appendix #4.

6.1.2 Preparation of IP-10 Stock (code: **mkpIP10-Std**) using the Human IP-10 standard from the Kit

(reconstituted at 5000.00 pg/mL)

- Please refer to Appendix #2 for the preparation of IP-10 Stock
- Add UPW (refer to the vial label for the volume) to the lyophilized vial IP-10 standard. Mix by repeated inversion.
- Incubate the vial for at least 15 minutes (but no more than 30 minutes) at ambient RT to allow proper reconstitution. Prepare the STD curve and quality control (QC) samples immediately after the end of reconstitution.
- Leftover reconstituted IP-10 Stock may be aliquoted (as per instructions given on Appendix #3) immediately after the end of reconstitution and stored in a freezer set to maintain -20˚C for up to 1 month. Discard if remaining volume not needed or insufficient for subsequent assays.
- On instances when a frozen aliquot is used to prepare the STD curve and QCs, it should be thawed immediately prior to use.

6.1.3 Preparation of Standard (STD) and QC samples

Notes:

i. Refer to Appendix #2 or Appendix #4, as applicable, for the preparation of standards and QC samples

ii. Standards and QCs are prepared at ambient RT on the day of the assay.

iii. Refer to BMK-004 for target QC concentration ranges.

6.2 Preparation of solutions

6.2.1 IP-10 Wash Buffer (1X TBS/ 0.05% Tween-20, pH 8.0) (code:**mkpIP10-WB**)

- Dissolve one TBS-Tween buffer pouch into 1L of UPW.
- Mix well.
- Store at ambient RT for a maximum of 1 month.

AP Number: AP.BMK.mkpIP10.09	Effective Date: Signature of AP	Supersedes: 30 Jun 2021

6.2.2 Substrate Solution IP-10 (code: **SS mkpIP-10**)

- Mix 11.5 mL of Color Reagent A and B (equal volumes) to an appropriate container.
- Protect this solution from light with aluminium foil.
- Mix by repeated inversion.
- Expiration: **To be used within 15 minutes of preparation.**

6.2.3 PBS/0.1% BSA (code: 0.1%BSA)

- In an appropriate container, add 0.50g of BSA to 500 mL of 1X PBS pH 7.2.
- Mix thoroughly until completely dissolved.
- Filter using a 0.2 or 0.22 µm filter system.
- Store in a refrigerator set to maintain 4°C for up to 2 weeks.

7.0 Preparation of trending QC (tQC)

The tQC will be prepared with a monkey plasma lot neat or diluted up to 256-fold in Calibrator diluent RD6Q. Refer to BMK-004 for preparation details.

- Aliquot the chosen lot(s) neat into appropriately labelled polypropylene tubes and store in a freezer set to maintain -20°C or -80°C. The expiry date on the label of the neat matrix should be SIP.
- Stability of the tQC sample(s) will be monitored and deemed acceptable based on the assay performance when analyzed using a qualified lot of kit.
- tQC is prepared on the day of the assay at ambient RT.

8.0 Validation Table

Parameter	Results	Based on Validation Study #
**Validated range using the Monkey Reference Standard***	LLOQ = 9.38 pg/mL<br>ULOQ = 600.00 pg/mL	**3800698**
**Validated range using the kit Human Reference Standard**	LLOQ = 30.00 pg/mL<br>ULOQ = 750.00 pg/mL	
**Dilution using the Monkey Reference Standard***	Samples may be analyzed neat or diluted up to 8-Fold with Calibrator Diluent RD6Q	
**Dilution using the kit Human Reference Standard**	Samples may be analyzed neat or diluted up to 256-Fold with Calibrator Diluent RD6Q	
**Short term matrix stability**	**26 hours and 26 minutes at ambient RT and in a refrigerator set to maintain 4°C**	

AP Number: AP.BMK.mkpIP10.09	Effective Date: Signature of AP	Supersedes: 30 Jun 2021

**Long term matrix stability**	**203 days in freezers set to maintain -20°C and -80°C**	
**Freeze-thaw matrix stability**	4 Freeze/thaw cycles in freezers set to maintain -20°C and -80°C.	
*A standard curve using the monkey reference standard should be used in priority. However, the human reference standard from the kit can also be used with the validated range and dilution listed above.		

9.0 Procedure

9.1 ELISA Procedure

Notes:

i. Assay reagents and samples should be brought to ambient RT before starting the assay.

ii. Standards, QCs (QC1, QC2, QC3 and tQC, one set before and one set after the study samples) and study samples should be analyzed in duplicate. (Please note that for qualification purposes, QCs can be loaded n=3).

iii. Study samples should be analyzed in batches so that samples from each group and sex (if applicable) are represented in a single assay (unless otherwise stated in advance prior to analysis). This will not be applicable to repeat analysis.

iv. On the first occasion, samples should be analyzed diluted 3-fold (when using the monkey reference standard) and 2-fold when using the human standard from the kit, unless different levels of IP-10 are expected.

v. Samples should be transported from Sample Management on dry ice and thawed at ambient RT. Record in-process sample storage on Appendix #1.

vi. Pipettes used for the assay procedure, preparation of standards and QCs and daily solutions will be recorded on Appendix # 1. Pipettes for dilution of study samples will be recorded on the dilution sheet or on Appendix #1 (both are appropriate).

vii. Please note that for the manual washing step only, multi-channel pipette can be used beyond the calibrated range as the exact volume is not critical for the washing of the assay plate.

viii. Please refer to Appendix #1 for the assay procedure.

AP Number: AP.BMK.mkpIP10.09	Effective Date: Signature of AP	Supersedes: 30 Jun 2021

10.0 <u>Calculation of Data</u>

Data capture of the output from the plate reader is performed using SOFTmax PRO GxP software. The blank is not subtracted in SOFTmax PRO.

SoftMax Pro GxP Settings		
**Settings**	**Display**	**Options**
Wavelength	Endpoint<br>Lm1 450 nm<br>Lm2 540 nm	Wavelength:<br>Lm1: 450 nm<br>Lm2: 540 nm
PathCheck	N/Ap	Off
Automix & blanking	Automix: Off	Before First Read should be unchecked<br>Pre-Read Plate should be unchecked
Auto Calibrate	On or Once	AutoCalibrate should be ticked
Column Wavelength Priority	Column Priority	Select column priority
Assay Plate Type	N/Ap	96 Well Standard clrbtm
Wells to Read	N/Ap	Read entire plate
Carriage Speed	C.Speed: Normal	Select Normal
AutoRead	N/Ap	AutoRead should be unchecked
Speed Read	N/Ap	Speed read should be unchecked
∑ Reduction		
∑ Reduction	Wavelength Combination:<br>!Lm1-!Lm2	Select: !Lm1-!Lm2<br>Data Mode: Absorbance<br>Use plate blank should be unchecked
∑ Reduction		
Raw / ∑ Reduction	N/Ap	Select ∑ Reduced

Data will be reduced using LIMS. NSB/Standard 0 (0.00 pg/mL) should not be included as a calibrator of the standard curve. The assay should first be setup with these settings: Assay Type should be ELISA and the Instrument Type and Instrument Interface should be Softmax Pro (Reduced Data). The individual ODs of each standard are used to fit the standard curve using the following parameters:

AP Number: AP.BMK.mkpIP10.09	Effective Date: Signature of AP	Supersedes: 30 Jun 2021

Watson Master Assay settings:

Analyte ID: mkpIP10	
Details	
Reduction type	Logistic (Auto Estimate)
Configuring Standards	Mean of replicate raw data should be unchecked
Configuring QCs	Mean of replicate raw data should be unchecked<br>Number of serial dilutions to use should be 2<br>Select mean concs
Configuring others	Mean of replicate raw data should be unchecked<br>Number of serial dilutions to use should be 2<br>Select mean concs
Analyte Info	
Concentration units	pg/mL
Weighting Factor	1/Y
Decimal Places	6
Regression Type	Logistic (Auto Estimate)
Titer-Plate Mapping	
Titer-Plate Details	Number of plate per Analytical Run should be 1.<br>STD 0 (0.00 pg/mL) should be loaded as NSB.<br>NSB should be ignored.

11.0 <u>Assay Acceptance Criteria</u>

11.1 Blank Evaluation

Blank (STD 0) Evaluation	Action to take
Both Blank wells OD < Mean OD of LLOQ	No action to be taken
One of the 2 Blank wells OD > Mean OD of LLOQ	Investigation to be provided to discuss the impact on the Data (Accept or Reject the assay)
Both Blank wells OD > Mean OD of LLOQ	

11.2 Calibration Curve

- The mean back-calculated values for all working calibrators should be within ±25% of their theoretical values).
- A maximum of i) two full points, or ii) one full point and two partial points, or iii) three partial points can be masked from a STD curve with 8 or more working STDs. Two consecutive STD levels cannot be fully masked.

AP Number: AP.BMK.mkpIP10.09	Effective Date: Signature of AP	Supersedes: 30 Jun 2021

- A maximum of i) one full point and one partial point, or ii) two partial points can be masked from a STD curve with 7 working STDs.
- A maximum of two partial points will be masked from a STD curve with 6 working STDs.
- This does not apply to the accessory standard, which is an additional standard with a concentration below the LLOQ to help define the curve at the lower end. If the accessory standard has a negative impact on the overall back-calculation of the individual standards, it can be partially masked.
- If the standard curve fails to meet the acceptance criteria, the partial deletion of standard that meet acceptance criteria may be performed if it allows the improvement of the curve fit and remaining standards.
- The curve must contain at least 6 working standards following deletion of standards.
- If the LLOQ and/or the ULOQ are removed, then the next lowest and/or next highest working calibrator becomes the new LLOQ and ULOQ, respectively. Any sample measured as greater than the new ULOQ or as below the new LLOQ should be repeated.

11.3 Quality Controls (QCs in Calibrator Diluent RD6Q and tQC)

- Mean back calculated concentrations should be within 75% - 125% of their theoretical concentrations and the % difference should be within ±25%.

11.4 Run Acceptance Criteria

- A run is accepted if the calibration standard curve meets the acceptance criteria mentioned in section 11.2 and if at least 67% of the QC samples meet the acceptance criteria mentioned in section 11.3, with at least 50% of the QCs (QC1 to 3 and tQC samples) accepted at each level.

11.5 Acceptance Criteria for Study Samples

- The % difference between singlicate values should be within ±25%. A sample should be repeated if this criteria is not met.
- Watson cannot calculate the % difference between singlicates if one singlicate is within the curve range, and the other singlicate is <LLOQ or >ULOQ. Therefore, LLOQ and ULOQ thresholds have been defined, that represent 25.4% difference with the LLOQ and ULOQ, respectively. If one singlicate is outside the curve range and the other singlicate is ≥ LLOQ threshold or ≤ULOQ threshold, the sample should be repeated. If the quantifiable singlicate is ≤ LLOQ threshold (when analysed neat) or ≥ ULOQ threshold (at the highest dilution factor), the quantifiable singlicate is reported. Refer to Appendix #3 for the LLOQ and ULOQ threshold concentrations.
- "< Y" (where y is the assay LLOQ concentration multiplied by the dilution factor at which the sample was analyzed, if applicable) will be reported for study samples with concentrations below the LLOQ. The value of the LLOQ should be recorded in the footnote of the report table.
- "Y/2" will be used for the calculation of the mean and standard deviation (SD) for values for which the measured concentration was <LLOQ, and will be mentioned as a footnote as well.
- Any samples with concentration > ULOQ should be diluted up to 256-fold with Calibrator diluent RD6Q and re-analyzed. The final reported value should be observed

AP Number: AP.BMK.mkpIP10.09	Effective Date: Signature of AP	Supersedes: 30 Jun 2021

concentration multiplied by the dilution factor.

- In the event where the study sample may not be re-analyzed at a higher dilution factor due to validation or volume limitation, ">X" (where X is the assay ULOQ concentration multiplied by the dilution factor at which the sample was analyzed) will be reported. "X" will be used for the calculation of the mean and SD, and will be mentioned as a footnote as well.

11.6 Reported Results

- Results, group mean and SD will be reported in pg/mL with two decimal places. Percentages will be reported at one decimal place. Due to a software limitation, all means and SDs will be reported to 1 more decimal place and percentages will be reported with no decimal places when generated by the SRS software.

- The upper limit of the normal range of concentrations will be defined as:
  The overall baseline mean (predose/pretreatment values for all animals* in all groups)** + 2 standard deviations.

- Fold change will be reported as:
  The ratio of the measured IP-10 concentration/upper limit of the normal range of concentrations.
  The fold change should be calculated for each sample

- Incidence of IP-10 elevations will be reported:
  The number of individual animals* per group with a fold change ≥1.1-fold.

*Calculations will be done separately for females and males.
**If predose values are not available, values from all animals of the non-treated group(s) will be used to generate the overall baseline mean.

Based on the nature of the study or specific sponsor requirement the upper limit of the normal range of concentrations, fold change and/or incidence might be calculated differently, and should be documented in appropriate study plans.

12.0 Revision History

Version	Date	Reason For Revision
1	07-Oct-2016	New AP.
2	17-Mar-2017	- Charles River logo was updated and dash removed from abbreviated name.<br>- Wording in section 6.1.2 for QC preparation was updated to reference SOP BMK-004.<br>- Section 10.5 was added to include definition for upper limit of the normal concentration range and to provide instructions on how to report incidence of IP-10 elevation and calculate fold change.
3	01-May-2017	-Section 6.1.1 Add the possibility of aliquoting and storing the IP-10 stock to account for differences in packaging of the kits. (Appendix #2

AP Number: AP.BMK.mkpIP10.09	Effective Date: Signature of AP	Supersedes: 30 Jun 2021

		was modified accordingly, details below).<br>This change was recommended by the kit manufacturer based on the stability of the IP-10 standard stock, and thus has no impact.<br>-Minor formatting in Appendices #1 and #2.
4	22-Jun-2017	**Appendix #1:**<br>-Add a step to bring assay reagents to RT before starting the assay in accordance with other APs<br>-reduce the volumes for manual washes to 350 µL<br>-add a prompt to record the expiry date of UPW on the reagents/working solutions table.<br>**Appendix #2:**<br>Standard stock information:<br>-Moved the prompt to record the batch ID of the reconstituted standard from Table 2 to "Standard Stock" section.<br>-Changed the wording: Aliquot expiry date to batch expiry date;<br>-"Not needed" was changed to "N/Ap"<br>**Table 2**:<br>-Modified to enter a generic name for the Stock used to prepare the STD 8 and QC3 instead of a prompt to record the batch ID.
5	11-Aug-2017	**Section 6.1.1**: Volume of aliquots was changed to be left undefined, based on possible differences in provided volumes.<br>**Qualification box:** maximum dilution was updated to 512-fold based on recent qualification assays.<br>**Appendix 1**: Clarifications were added to the procedure.
6	04-Jun-2018	**Section 1.0:** Modified to remove the statement that the AP should only be used to support non-GLP work. Method has been validated and the AP can now support GLP work.<br>Section 7.0: Modified to add the possibility to store the tQC in a freezer set to maintain -20°C.<br>**Validation box:**<br>Validation study number was added to replace the non-GLP qualification study number in the previous AP version.<br>ULOQ was changed from 1000.00 to 750.00 pg/mL<br>Dilution range was updated.<br>Stability results were added.<br>**Section 9.0:** Watson Master Assay Settings were modified to add the SEND terminology.<br>**Section 10.1:** Acceptance criteria of the standard curve were updated to reflect the new 6-point curve (previously 7-point) and the change in acceptance criteria of LLOQ and ULOQ (previously ±30% of theoretical, decreased to ±25%).<br>**Section 10.2:** Acceptance criteria of the QCs were modified

AP Number: AP.BMK.mkpIP10.09	Effective Date: Signature of AP	Supersedes: 30 Jun 2021

		(previously ±30% of theoretical and 30% difference between singlicates, decreased to ±25% theoretical and ±25% difference between singlicates).<br><br>**Section 10.4:**<br>The maximum dilution was updated in the acceptance criteria.<br>Modified to add a ULOQ threshold.<br>**Appendix #1:**<br>Suggested volume of pre-load was decreased from 200 to 180 µL.<br>Data review section modified to reflect the change in acceptance criteria of the curve and QCs (section 10.1 and 10.2 of AP)<br>Minor formatting of the tables was performed.<br>**Appendix #2**:<br>Minor formatting changes.<br><br>**Appendix #3**:<br>Standard curve and QC details were removed, since the curve range is stated in the AP, and the actual standards and concentrations are stated on Appendix #2.<br>Minor formatting changes of the appendix.
7	11-Jan 2019	**Section 5.4:** The name of Bioreclamation as a supplier for monkey plasma was changed to BioIVT, and "other commercial suppliers" was added as a possible source for monkey plasma.<br>**Validation box:** updated to include the results of the long term stability.<br>**Section 10.5**: was modified to update the definition of the incidence of IP-10 elevations and to include reference to study plans in the event where a different definition of fold-change and elevation is used.<br>Minor changes in wording throughout the document, without changes in the procedure.
8	30 Jun 2021	Signature box: to update the signature reasons to reflect ST01-01-31<br>Section 5.1, 5.3, 5.4, 6.1, 6.2 and 7.2, and Appendix #4 were added/updated because a monkey reference standard should be used to prepared the standards and QCs and therefore reagents, and instructions for the preparation of the standard and QC were added.<br>Section 5.5: Suppliers were removed as any supplier is acceptable. Instruction to record lot number in the raw data was removed as recording of raw data is covered by ST01-02-01<br>Section 10.0: To remove the automix from the SoftMaxPro settings, as this was considered a better practice by instrument specialists. This change has no impact as the plate is mixed manually before reading. SoftMax Pro instruction box was updated for clarity with no change to the processes (other than the removal of automix). Other minor

Appendix 11

Appendix 4

AP Number: AP.BMK.mkpIP10.09	Effective Date: Signature of AP	Supersedes: 30 Jun 2021

		updates were done to standardize with other APs.<br>Section 11.1 and Appendix #1: Blank evaluation criteria was added.<br>Section 11.2: The reprocessing of the calibration curve with 7 or more working standards added since the curve may have more than 6 working standards. In addition, the outcome if the LLOQ or ULOQ is removed was added.<br>Section 11.3: the % difference formula was removed because it is define in the SOP<br>Appendix #1: Incubation end time replaced by execution time of the washing step because the end time of the incubation correspond to the time the plate is washed.<br>Appendix #3: Addition of the option for the standard to be used in the preparation of the standard curve<br>Minor typos and inconsistencies corrected throughout the document, with no change to the procedure.
9	Signature of AP	Section 8.0: To correct a typographical error in the validation study number

Appendix 11

Appendix 4

Plasma IP-10 ELISA Assay Sheet

Study/reference No: ____________ Assay I.D.: ____________

Page. 1 of 4

Reagents/ Working Solutions						
Name	*Batch or Lot #* *(as applicable)*	*Inventory # (if applicable)* Assay ID:	Assay ID: *Or N/Ap* ☐	Assay ID: *Or N/Ap* ☐	*Expiry Date*	*Entered by /Date*
Human CXCL10/IP-10 kit						
IP-10 Microplate						
Assay diluent RD1-56						
IP-10 Conjugate						
Calibrator Diluent RD6Q						
Stop solution						
Recombinant rRhIP-10/CXCL10 Standard	rRhIP-10 STD- *Or N/Ap* ☐	N/Ap				
IP-10 Stock (from kit)	mkpIP10-Std- *Or N/Ap* ☐	N/Ap				
IP-10 Wash Buffer	mkpIP10-WB-	N/Ap				
UPW		N/Ap				

Working Solutions Preparation Assay ID:__________						
**Batch no**	**Chemical ID**	*Lot#*	*Inventory#*	*Volume (mL)*	*Total vol. (mL)*	**Performed by /Date**
**Preparation of: Substrate Solution IP-10 (code SS mkpIP-10)**						
SS mkpIP-10-	Color Reagent A					
	Color Reagent B					
Time of preparation: ____________	(*Note*: It should be used within 15 min of preparation)					

Appendix #1 (AP.BMK.mkpIP10.09)

Plasma IP-10 ELISA Assay Sheet

Study/reference No: ____________________ Assay I.D.: ____________________

Page. 2 of 4

Working Solutions Preparation Assay ID:________ Or N/Ap ☐						
**Batch no**	Chemical ID	***Lot#***	***Inventory#***	***Volume (mL)***	***Total vol. (mL)***	**Performed by /Date**
**Preparation of: Substrate Solution IP-10 (code SS mkpIP-10)**						
SS mkpIP-10-	Color Reagent A					
	Color Reagent B					
Time of preparation: ______________ (*Note*: It should be used within 15 min of preparation)						

Working Solutions Preparation Assay ID:________ Or N/Ap ☐						
**Batch no**	**Chemical ID**	***Lot#***	***Inventory#***	***Volume (mL)***	***Total vol. (mL)***	**Performed by /Date**
**Preparation of: Substrate Solution IP-10 (code SS mkpIP-10)**						
SS mkpIP-10-	Color Reagent A					
	Color Reagent B					
Time of preparation: ______________ (*Note*: It should be used within 15 min of preparation)						

In-Process Sample Storage				
**Assay ID:** *Or N/Ap* ☐	**Performed (√)**		**Start Time**	**Performed by / Date**
Samples transported from Sample Management.	On dry ice	( )	N/Ap	
Samples thawed, diluted, and kept until loading:	Ambient RT	( )		
Samples placed in temporary storage after use and until returned to Sample Management	On dry ice	( )		
**In-Process Sample Storage**				
**Assay ID:** *Or N/Ap* ☐	**Performed (√)**		**Start Time**	**Performed by / Date**
Samples transported from Sample Management.	On dry ice	( )	N/Ap	
Samples thawed, diluted, and kept until loading:	Ambient RT	( )		
Samples placed in temporary storage after use and until returned to Sample Management	On dry ice	( )		
**In-Process Sample Storage**				
**Assay ID:** *Or N/Ap* ☐	**Performed (√)**		**Start Time**	**Performed by / Date**
Samples transported from Sample Management.	On dry ice	( )	N/Ap	
Samples thawed, diluted, and kept until loading:	Ambient RT	( )		
Samples placed in temporary storage after use and until returned to Sample Management	On dry ice	( )		

INSTRUMENTS		
Name	***ID***	***Entered by / Date***
Pipettes		
Multichannel pipettes		

Appendix #1 (AP.BMK.mkpIP10.09)

Appendix 11

Appendix 4

Plasma IP-10 ELISA Assay Sheet

Study/reference No: ____________ Assay I.D.: ____________

Page. 3 of 4

ASSAY				
Steps	*Time /Performed (√)*			*Performed by / Date*
	Assay ID:	*Assay ID:* *Or N/Ap* ☐	*Assay ID:* *Or N/Ap* ☐	
**Bring all assay reagents (except the frozen aliquot of the mkplP10-STD*) to ambient RT** before starting the assay **. Color Reagent A and Color Reagent B should be covered with aluminum foil to avoid light exposure, and should only be mixed prior to use.	( )	( )	( )	
**Pre-Load**: add standards, QCs and study samples (one replicate) into a non-coated 96 well plate. Suggested volume is 180 µL. Note that this step can be skipped if insufficient sample volume is available.	( ) or N/Ap ( )	( ) or N/Ap ( )	( ) or N/Ap ( )	
Before loading, add 75 µL of assay diluent RD1-56 into each well of the **assay plate**.	( )	( )	( )	
**Load:** Pipet 75 µL/well of standards, QC samples and study samples in duplicate from the non-coated plate to the appropriate wells of the assay plate. Incubate assay plate for **2 hrs (± 5 min)** at ambient RT.	Start:	Start:	Start:	
**Wash** the plate **manually 4 times with 350 µL** of **mkplP10-WB**, and blot the assay plate on an absorbent paper.	Time:	Time:	Time	
**Capture/ detection:** Add 200 µL of IP-10 conjugate into each well. Incubate **for 2 hrs (± 5 min**) at ambient RT.	Start:	Start:	Start:	
**Wash** the plate **manually 4 times with 350 µL** of **mkplP10-WB** and blot the assay plate on an absorbent paper.	Time:	Time:	Time:	
Just prior to the substrate step prepare the Substrate solution IP-10 (code SS mkpIP-10) protected from light (**to be used within 15 minutes of preparation**).	( )	( )	( )	
**Substrate**: Add 200 µL of Substrate Solution IP-10 into each well. Cover the assay plate with a plate sealer and aluminum foil and incubate in the **DARK** for **30 minutes (± 1 minute)** at ambient RT.	Start:	Start:	Start:	
**Stop**: Add 50 µL of Stop Solution. The color in the wells should change from blue to yellow. If the color in the wells is green or the color change does not appear uniform, gently tap the plate to ensure thorough mixing.	Time:	Time:	Time:	
**Read** the plate with a spectrophotometer set at 450-540 nm. The plate should be read as soon as possible, within 30 minutes of Stop Solution addition and protected from light if not read immediately after addition of the Stop Solution.	( )	( )	( )	

*** If used, the frozen aliquot of the mkplP-10-STD should be brought to ambient RT just prior to use.**

**** In the event where the kit is packaged in bulk, only the appropriate volume should be brought to ambient RT prior to use, and the leftover should be returned to storage as soon as possible.**

Appendix #1 (AP.BMK.mkplP10.09)

Plasma IP-10 ELISA Assay Sheet

Study/reference No: ____________ Assay I.D.: ____________

Page. 4 of 4

Data Review	Assay ID: ______	Assay ID: ______ Or N/AP ☐	Assay ID: ______ Or N/AP ☐
Number of working calibrators in curve within ±25% of the theoretical value	/	/	/
Number of QC1 within acceptance criteria*:	/	/	/
Number of QC2 within acceptance criteria* :	/	/	/
Number of QC3 within acceptance criteria* :	/	/	/
Number of tQC within acceptance criteria* :	/	/	/
Data review performed by/Date: ____________			

* % difference between singlicate values should be within ±25% and the percent (%) theoretical calculated using the mean concentration should be within 75% - 125%.

Scientific Review	Assay ID: ______	Assay ID: ______ Or N/AP ☐	Assay ID: ______ Or N/AP ☐
Standard Curve Blank (STD 0) evaluated:	Yes or No	Yes or No	Yes or No
Investigation to be provided for blank evaluation:	Yes or No	Yes or No	Yes or No
Assay is acceptable:	Yes or No	Yes or No	Yes or No
Sample(s) to repeat:	Yes or No or N/Ap	Yes or No	Yes or No
Scientific review performed by/Date: ____________			

Appendix #1 Reviewed by: ____________ Date: ____________

Appendix #1 (AP.BMK.mkpIP10.09)

Appendix 11

Appendix 4

Plasma IP-10 ELISA Spiking Sheet (Kit Standard)

Study/Reference no ______________________ Assay ID: ______________

Reference standard: IP-10 standard

Part. no. 890836

Supplier: R&D system

IP-10 Standard (**Stock**) Freshly reconstituted ( ) Fill Table 1 below

Frozen aliquot ( ) Table 1 N/Ap ( )

Batch ID: Refer to Appendix #1 Batch Expiry date: Refer to Appendix #1

Table 1. Preparation of Standard stock

Stock ID/Batch ID	IP-10 standard lot number	Volume of UPW added to each vial (µL)[A]		Concentration (pg/mL)	Mix by inversion	Time left on bench after reconstitution (At least 15 minutes but no more than 30 minutes)		Expiry date of lyophilized standard	Vial label
IP-10 Standard (**Stock**)[B] / mkpIP10-Std-			( )	5000.00	( )	Start:	Finish:		

[A]Enter volume manually as per vial label and stick the label in the appropriate space.

[B]The Standard reconstituted stock was aliquoted: No ( ) Yes ( ) Freezer ID (if stored): ____________ Expiry date of reconstituted standard: ____________

Table 2. Preparation of Standards and Quality control samples

Sample ID	Target Concentration (pg/mL)	Stock ID	Stock Concentration (pg/mL)	Total Volume (µL)	Stock		**Calibrator diluent RD6Q**		Final Concentration (pg/mL)
					Volume (µL)	Performed (√)	Volume (µL)	Performed (√)	
STD 8 (not loaded)	1000.00	Stock	5000.00	725	145	( )	580	( )	1000.00
**STD 7 (ULOQ)**	750.00	STD 8 (not loaded)	1000.00	700	525	( )	175	( )	750.00
STD 6	500.00	STD 7 (ULOQ)	750.00	600	400	( )	200	( )	500.00
STD 5	250.00	STD 6	500.00	550	275	( )	275	( )	250.00
STD 4	125.00	STD 5	250.00	550	275	( )	275	( )	125.00
STD 3	60.00	STD 4	125.00	550	264	( )	286	( )	60.00
**STD 2 (LLOQ)**	30.00	STD 3	60.00	400	200	( )	200	( )	30.00
STD 1	15.00	STD 2 (LLOQ)	30.00	250	125	( )	125	( )	15.00
STD 0	0.00	N/ap	0.00	250	N/Ap	(----)	250	( )	0.00
QC3	450.00	Stock	5000.00	600	54	( )	546	( )	450.00
QC2	250.00	QC3	450.00	560	311	( )	249	( )	249.91
QC1	70.00	QC2	250.00	450	126	( )	324	( )	70.00

Calibrator Diluent RD6Q lot: Refer to Appendix #1

Comments: ____

Spiking sheet verified by/Date: ______________ Performed by/Date: ______________

Calculations verified by/Date: ______________ Reviewed by/Date: ______________

Appendix #2 (AP.BMK.mkpIP10.09)

IP-10 in Monkey Plasma Assay Instructions

Study/Reference no. : ________________ Assay ID: ________________

Kit lot: ________________ Kit Qualification Assay ID: ________________

The following Reference Standard should be used for the preparation of the standard curves:

Reference Material to use	Lot/Batch ID	Location	Appendix to use
☐ Human IP-10 standard from the kit (Part #890836)			
☐ Reconstituted IP-10 Standard from the kit			
☐ Monkey reference standard (Bio Basic Cat# RC322-21)			
☐ Reconstituted Monkey reference standard (rRhIP-10 STD)			

The following tQC should be used for the assay.

QC Identification	Monkey Plasma lot#	Dilution	Location in a freezer set to maintain -20°C or -80°C	tQC concentration (pg/mL)	Source assay and study ID	Source assay date[a]
tQC						

[a] Refer to AP for tQC samples stability. **The expiry date on the label of the neat matrix should be SIP.**

- LLOQ Threshold:_________ pg/mL
- ULOQ Threshold:_________ pg/mL
- Analyze study samples : ☐ Neat
  ☐ as per provided dilution sheet.

Comment(s):
____
____
____
____

Verified by/date: ______________________

Reviewed by/date: ______________________

Appendix # 3 (AP.BMK.mkpIP10.09)

Appendix 11

Appendix 4

Plasma IP-10 ELISA Spiking Sheet (Monkey Reference Standard)

Study/Reference No: ______________ Assay ID: ______________

Reference standard: Recombinant rRhIP-10/ CXCL10 (5µg)

Catalogue number: RC322-21

Supplier: Bio Basic

Monkey Reference Standard (Stock) — **Freshly reconstituted** ( ) Fill Table 1 below; **Frozen aliquot** ( ) Table 1 N/Ap — Batch ID used: Refer to Appendix 1 — Batch Expiry date: Refer to Appendix 1

Table 1. Preparation of Monkey Reference Standard stock

Stock	Recombinant rRhIP-10/ CXCL10 (5µg/vial)			Batch assigned	# of reconstituted vial	Volume of 0.1%BSA to add to each vial		Final Concentration
	Vial lot#	Inventory #	Expiry date			Volume (µL)	Performed (√)	(µg/mL)
Recombinant rRhIP-10/CXCL10 Standard (code: rRhIP-10 STD)				rRhIP-10 STD-	1	500	( )	10.00

Quick spin: ( ) **Centrifuge ID:** ______________

0.1%BSA batch: ______________ **0.1%BSA expiry date:** ______________

Aliquoting : **# aliquots of** ______________ **Expiry date:** ______________ **Storage:** ______________

Box ID: ______________

Place aliquot label here

Table 2: Preparation of Standards and Quality control samples

Sample ID	Stock ID	Stock Concentration (pg/mL)	Total Volume (µL)	Stock		Calibrator diluent RD6Q		Final Concentration (pg/mL)
				Volume (µL)	Performed (√)	Volume (µL)	Performed (√)	
Stock 1	rRhIP-10 STD	10000000.00	200	10	( )	190	( )	500000.00
Stock 2	Stock 1	500000.00	1000	20	( )	980	( )	10000.00
**STD 9**	Stock 2	10000.00	1000	120	( )	880	( )	1200.00
STD 8 (ULOQ)	STD 9	1200.00	1200	600	( )	600	( )	600.00
**STD 7**	STD 8 (ULOQ)	600.00	600	300	( )	300	( )	300.00
STD 6	STD 7	300.00	600	300	( )	300	( )	150.00
STD 5	STD 6	150.00	600	300	( )	300	( )	75.00
STD 4	STD 5	75.00	700	350	( )	350	( )	37.50
STD 3	STD 4	37.50	800	400	( )	400	( )	18.75
**STD 2 (LLOQ)**	STD 3	18.75	1000	500	( )	500	( )	9.38
STD 1	STD 2 (LLOQ)	9.38	300	100	( )	200	( )	3.13
STD 0	N/Ap	N/Ap	250	N/Ap	(---)	250	( )	0.00
QC3	Stock 2	10000.00	2000	80	( )	1920	( )	400.00
QC2	QC3	400.00	925	200	( )	725	( )	86.49
QC1	QC2	86.49	900	250	( )	650	( )	24.02

Calibrator Diluent RD6Q lot: Refer to Appendix #1

Comments: ______________

Spiking sheet Verified by/Date: ______________ Performed by/Date: ______________

Calculations verified by/Date: ______________ Reviewed by/Date: ______________

Appendix #4 (AP.BMK.mkpIP10.09)

SIGNATURE(S) FOR DOCUMENT: 5550014 - 5550014 Biomarker Final Report Cytokines

**Principal Investigator:**	I approve this document.
Name:	**Harouchi, Hycham**
Harouchi, Hycham	04-Mar-2022 06:06:28 (UTC+00:00)
Electronically Signed in M-Files	Timestamp

Appendix 12

REPORT

AAV9/*AP4M1* study in NHPs: Biodistribution, Expression, and Immune Response

Appendix 12

Work conducted at University of Texas Southwestern Medical Center in the laboratory of Dr. Steven Gray

Personnel and Role

- Xin Chen: cDNA synthesis, data compilation, and report preparation.
- Kathryn McMillan: Genomic DNA and total RNA preparation, ELISpot assay, and report preparation.
- Sandra Unorji and Melissa Hyatt: qPCR assay
- Yang Yu: qPCR assay/analysis and report preparation.
- Steven Gray: Supervisor and report approval.

I certify that, to the best of my knowledge, the information in this report is correct and a true representation of the work carried out.

Xin Chen	Date
K. McMillan	1/14/2022
Kathryn McMillan	Date
Sandra Unorji	01/14/2022
Sandra Unorji	Date
Melissa Hyatt<br>Digitally signed by Melissa Hyatt<br>DN: cn=Melissa Hyatt, c=US, o=UT Southwestern Medical Center, ou=AAV Vector Core, email=melissa.hyatt@utsouthwestern.edu<br>Date: 2022.01.14 09:41:00 -06'00'	
Melissa Hyatt	Date
Yang Yu	Date
Steven Gray	Date

Table of Contents

1. OBJECTIVE 4
2. ABBREVIATIONS....4
3. MATERIALS AND METHODS 4
4. RESULTS AND DISCUSSION 5
5. CONCLUSIONS....8
6. REFERENCES....8
7. APPENDICES....20

List of Tables and Figures

Table 1. Experimental Design 5
Table 3. Raw data vg/NHP diploid genome.... 20
Table 4. Raw data of expression: copies of AP4M1 per copy of GAPDH 22
Table 5. Raw data of immune response: Spot# in ELISpot plate.... 23

Figure 1. AP4M1 biodistribution in WT NHPs.... 6
Figure 2. AP4M1 expression in WT NHPs. 7
Figure 3. ELISpot assay.... 8

1. OBJECTIVE

The objective for this study was to characterize the biodistribution, expression, and immune response of self-complementary Adeno-Associated Virus serotype 9 (scAAV9)/UsP-*hAP4M1opt*-BGHpA (AAV9/*AP4M1*), following a single lumbar intrathecal (IT) injection in wild-type (WT) non-human primates (NHPs).

2. ABBREVIATIONS

AAV	Adeno-Associated Virus
AP4M1	Adaptor-related protein complex AP-4, μ4
BGH	Bovine growth hormone
DNA	Deoxyribonucleic acid
ELISpot	Enzyme-linked immunospot
INFγ	Interferon γ
IT	Lumbar intrathecal
NHP	Non-human primate
PBS	Phosphate-buffered saline
PMA	Phorbol 12-myristate 13-acetate
qPCR	Quantitative PCR
RNA	Ribonucleic acid
Sc	Self-complementary
UsP	Promoter
vg	Vector genomes
WT	Wild type

3. MATERIALS AND METHODS

3.1 AAV9/*AP4M1* Production

AAV9/*AP4M1* in Phosphate-buffered saline (PBS), 5% Sorbitol, pH 7.4 containing 0.001% F-68 was produced by Viralgen in Spain. The final vector product (lot # T-GEMINIS-033) was prepared with a titer of 5.43×10^{13} vector genomes (vg) /mL (COA in section 7. APPENDICES).

3.2 Animal Studies

Animal study was performed by Charles River Laboratories, Inc. Mattawan MI. Male and female NHPs were randomized into cohorts and dosed as shown in Table 1. At the initiation of dosing, the animals assigned to study were 2 to 4 years of age and weighed between 1.5 to 6 kg. AAV9/*AP4M1* vector was injected IT once on day 1 in each animal, in a volume of 1, 1.55, or 3.1 mL, at the infusion rate of 1 mL/min, and a final dose of 0, 8.4×10^{13}, or 1.68×10^{14} vg/NHP. NHPs were sacrificed on day 94. Tissues were collected frozen, and lymphocytes were prepared, and then sent to Dr. Gray's laboratory on dry ice for further analysis.

Table 1. Experimental Design

Group No.	Test Material	Dose Level (vg/animal)	Dose Concentration (vg/mL)	Dose Volume (mL)	Animal Numbers	
					Main Study	
					Male	Female
1	Vehicle	0	0	1	1201	1701
2	AAV9/*AP4M1*	8.4×10^{13}	5.43×10^{13}	1.55	-	2701, 2702
3	AAV9/*AP4M1*	1.68×10^{14}	5.43×10^{13}	3.10	3201	3701

3.3 Biodistribution and Expression Analysis by qPCR

Total genomic DNA and RNA were purified from tissue samples collected at necropsy day 94, using a Qiagen Qiacube HT kit. cDNA was synthesized from purified RNA and qPCR was used to determine the quantity of AAV9/*AP4M1* vector DNA biodistribution and *AP4M1* RNA transgene expression. Qualification of the qPCR assay is attached in the section 6. REFERENCES.

3.4 Immune response by ELISpot

Both peptide library pools of AAV9 capsid and AP4M1 protein were purchased from Mimotopes, Victoria, Australia. The peptides libraries were comprised of 10-mers with a 5 amino acid offset. The AAV9 capsid library pool contained 147 peptides and the AP4M1 protein library pool contained 89 peptides. Both pools were stored at -80°C before use. ELISpot assays were performed using Human IFN-γ 96-well White Precoated CTL-TrueBlue substrate (Fisher, NC1787696). Briefly, splenocytes were thawed, washed, and resuspended in cRMPI-1640 medium for counting. 2×10^5 splenocytes in 100 uL of cRPMI-1640 medium were plated into each well of an ELISpot plate. 100 uL of cRPMI-1640 medium containing the AAV9 capsid pool or the AP4M1 protein pool at 1 µg/well was then added to the wells. The controls included cells with no peptide, cells stimulated with a mixture of Phorbol 12-myristate 13-acetate (PMA) and Ionomycin (Invitrogen, 00-4970-93), medium with human INFγ (Fisher, 50-813-413), or medium only. The splenocytes were incubated for 48 hours in a humidified 37°C CO_2 incubator. The detailed steps of the ELISpot assay are attached in the references.

4. RESULTS AND DISCUSSION

A study was conducted in WT NHPs with each animal receiving a single IT injection of AAV9/*AP4M1* vector at a dose of 0, 8.4×10^{13}, or 1.68×10^{14} total vg. Genomic DNA was purified from the samples collected at necropsy day 94. *AP4M1* vector biodistribution was quantified by qPCR and provided in Figure 1. IT delivery of AAV9/*AP4M1* vector results in delivery of *AP4M1* vector DNA across the central nervous system and peripheral organs. The *AP4M1* vector DNA is widely detected at high level in multiple brain regions. In the peripheral organs, even higher amounts of *AP4M1* DNA persist in liver and to the less extent in other organs tested. Consistent with this *AP4M1* DNA biodistribution data, *AP4M1* transgene expression is also widely detected at high level in multiple CNS and peripheral tissues (Figure 2). Collectively, IT delivery of AAV9/*AP4M1* results in broad *AP4M1* biodistribution and expression across the body of NHPs.

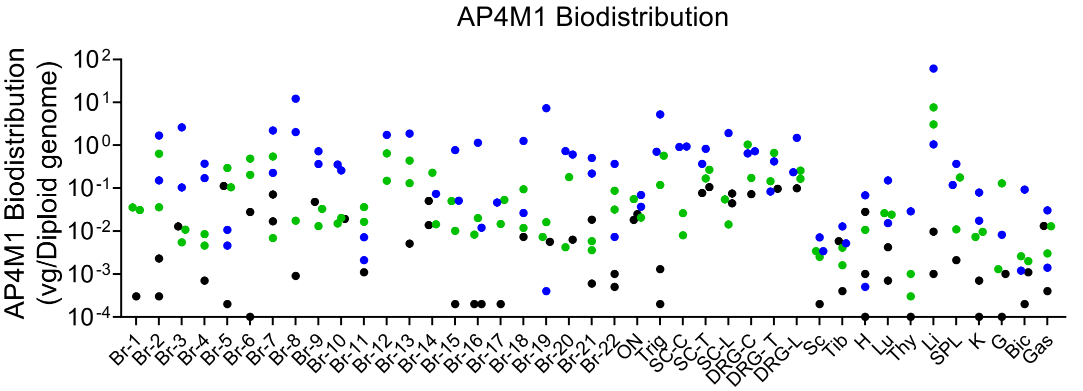

Figure 1. ***AP4M1* biodistribution in WT NHPs.**
WT NHPs received a single IT injection of AAV9/*AP4M1* at a dose of 0 (black dots), 8.4×10^{13} (green dots), or 1.68×10^{14} (blue dots) vg. Genomic DNA was purified from the samples collected at necropsy day 94, and *AP4M1* vector biodistribution across the central nervous and peripheral organs was quantified by qPCR. Results were presented as Mean±SEM. Br, Brain; ON, Optic Nerve; Trig, Trigeminal G; SC-C, Spinal Cord-Cervical; SC-T, Spinal Cord-Thoracic; SC-Lumbar, Spinal Cord-Lumbar; DRG-C, Dorsal Root Ganglion-Cervical; DRG-T, Dorsal Root Ganglion-Thoracic; DRG-L, Dorsal Root Ganglion-Lumbar; Sc, Sciatic Nerve; Tib, Tibia Nerve; H, Heart; Lu, Lung; Thy, Thymus; Li, Liver; SPL, Spleen; K, Kidney; G, Gonad; Bic, Biceps Femoris; Gas, gastrocnemius.

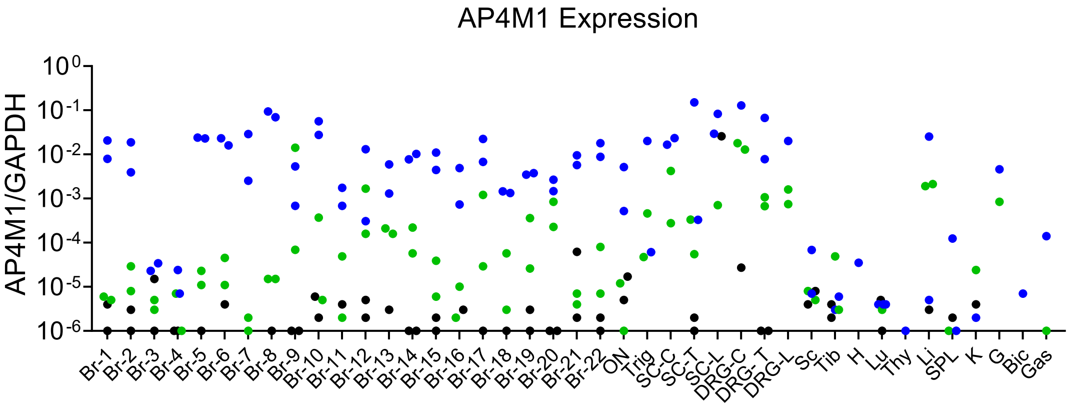

Figure 2. ***AP4M1* expression in WT NHPs.**

WT NHPs received a single IT injection of AAV9/*AP4M1* at a dose of 0 (black dots), 8.4×10^{13} (green dots), or 1.68×10^{14} (blue dots) vg. Total RNA was purified from the samples collected at necropsy day 94, cDNA was synthesized, and *AP4M1* expression across the central nervous and peripheral organs was quantified by qPCR. Results were presented as Mean ± SEM. Br, Brain; ON, Optic Nerve; Trig, Trigeminal G; SC-C, Spinal Cord-Cervical; SC-T, Spinal Cord-Thoracic; SC-Lumbar, Spinal Cord-Lumbar; DRG-C, Dorsal Root Ganglion-Cervical; DRG-T, Dorsal Root Ganglion-Thoracic; DRG-L, Dorsal Root Ganglion-Lumbar; Sc, Sciatic Nerve; Tib, Tibia Nerve; H, Heart; Lu, Lung; Thy, Thymus; Li, Liver; SPL, Spleen; K, Kidney; G, Gonad; Bic, Biceps Femoris; Gas, gastrocnemius.

Immune responses to AAV and/or transgene remain a challenge that can confound the safety and efficacy of AAV-mediated gene transfer. This study evaluated the T-cell INFγ immune response to the AAV9/*AP4M1* vector in WT NHPs. Specifically, splenocytes from WT NHPs IT injected with AAV9/*AP4M1* vectors 94 days prior were plated and treated *in vitro* with either AAV9 capsid or AP4M1 protein peptide pools for 2 days along with both negative (no peptide) and positive (PMA + Ionomycin) controls.

While the negative control has 0 spots (Figure 3), the positive controls have many spots (Table 5). Panels A and B in Figure 3 show the INFγ response to the AAV9 capsid pool and AP4M1 protein pool, respectively. None of the vehicle, low (8.4×10^{13} vg) or high (1.68×10^{14} vg) dose of AAV9/*AP4M1* vector generated any increase of the spot numbers compared to negative control, indicating that the AAV9/*AP4M1* vector generated no detectable T-cell immune response in WT NHPs, to either AAV9 or the human AP4M1 protein, under the immunosuppressant protocol.

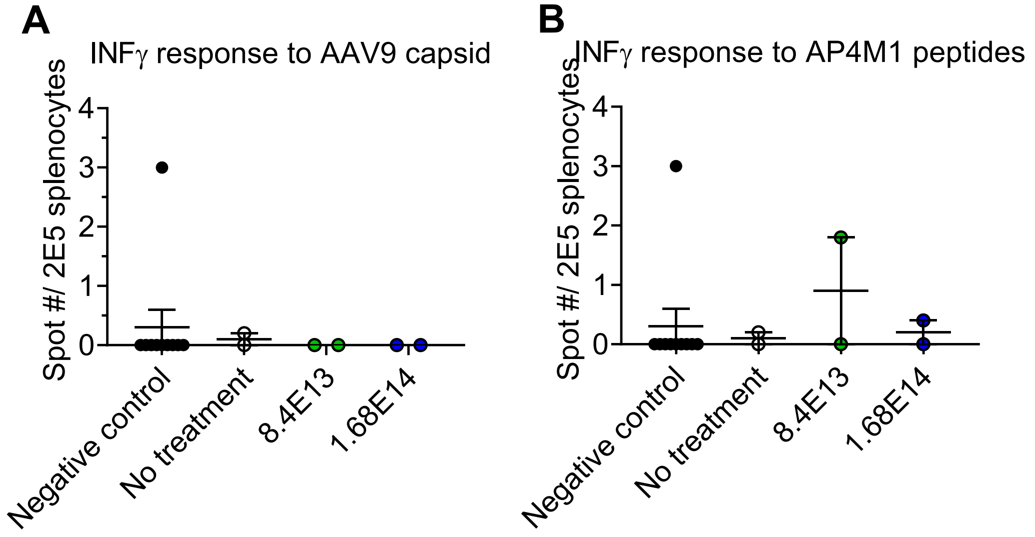

Figure 3. ELISpot assay

Spot numbers in each well were blindly counted with a specialized automated ELISpot reader. Each data point represents the mean value of duplicate assays for the negative control or the mean value of quintuplicate assays from an individual animal specimen, with lines representing the mean measurement ± SEM.

5. CONCLUSIONS

The AAV9 vector genome DNA biodistribution pattern should be dependent on the capsid, regardless of the DNA cargo that it carries, which is further supported by the similar pattern of DNA biodistribution from this study using AAV9/*AP4M1* as that expected for AAV9. The exceptions to this would be if an immune response led to viral clearance, or if there was toxicity against the expressed transgene that led to cell death and loss of viral genomes. In this study, it was concluded that AAV9 delivered IT can achieve broad distribution as well as expression across the nervous system and peripheral organs without generating any immune response under the immunosuppressant protocol, although the level of gene transfer in the brain is sub-saturating with a minority of cells receiving the transgene. Thus, this study is considered to portray the normal biodistribution and expression pattern expected for an AAV9 vector in NHPs. These results do not suggest any loss of vector due to cellular toxicity.

6. REFERENCES

6.1 Standard Operating Procedure

6.1.1 Non-Human Primate Samples for DNA and RNA Extraction

Prepared by Kathryn McMillan, UTSW Medical Center
Version: 02-02-2021

1. Exposure Reporting: Immediately report any exposure incident to your Principal Investigator, Safety Investigator, and/or call the University Employee Occupational Health Clinic. The specimen involved in the exposure/injury should be secured at <-60° C (unless whole blood then 2-6° C) so that it can be used for confirmation purposes.
   a. University Employee Occupational Health Clinic: 8:00am – 5:00pm 214-645-5300
   b. Environment Health and Safety: 214-645-1317. Ask for the Biological Safety Officer.
   c. Emergency: 911.
2. All manipulations of non-human primate samples must be performed in the biological safety cabinet (BSC) designated for non-human primate work in NA2.510B. Signs will be posted at the entrance to the hood area in NA2.510B and on the actual hood where work with the non-human primate samples is under progress. The sign with say "Do Not Enter, Monkey Sample Work in Progress."
3. Surfaces of all materials removed from the BSC (this includes hands and pipette man) must be sprayed with Vesphene disinfectant and be free of infectious agents. The sash of the BSC should be raised and the BSC operated for at least 15 minutes before work begins.
4. Double gloves must be worn at all times while working with non-human primate samples or anything in this BSC. Disposable lab coat or disposable tyvek sleeves, face mask and goggles must be worn at all times when working with all non-human primate samples.

5. Sharps use and generation of aerosols will be minimized. No glass is allowed to be used at any time for non-human primate sample manipulations. This includes glass pipettes for aspiration. Use only plastic serological pipettes and barrier pipette tips instead.
6. In case of spills inside the BSC: contaminated surfaces will be soaked with Vesphene disinfectant and covered with a paper towel for 30 minutes. The towels will be removed and placed in a biohazard waste bag. The hood will be completely wiped down with Vesphene, dried with paper towels, then wiped down with 70% ethanol and allowed to air dry. For spills outside the BSC, contaminated surfaces will be soaked with 10% bleach and after a 30-minute contact time, dried with paper towels and handled as biohazardous waste.
7. All non-human primate non-liquid waste (plates, tubes, pipettes, tips, etc.) will be placed in a plastic bin containing a solution of 10% bleach. Interior surfaces of tips, pipettes, plates, tubes, etc. must be completely exposed to 10% bleach solution either by drawing bleach solution into the pipette or container, or by completely submerging items individually and inspecting for contact with bleach. Waste will be left in the bleach solution for at least 20 minutes, emptied of all liquid, and placed in a small biohazard waste bag in a secondary container in the BSC. This bag will be tied up and disinfected before it is removed from the hood. Biohazard waste will be taken to the autoclave room (NA2.218) and immediately autoclaved by the lab worker generating the waste.
8. Non-human primate sample liquid material (or solutions coming in contact with non-human primate samples) may be aspirated into waste flask for inactivation. Flask must contain a volume of undiluted bleach prior to use equal to 10% of the volume of the flask. When finished aspirating, tubing must be cleaned by aspiration of a 10% bleach solution followed by aspiration of 70% ethanol (leaving residual bleach in tubing will destroy it). Let the bleach have a minimum 30-minute contact time with the solution in the flask before pouring the solution down the sink. You must wear PPE during this time.
9. Non-human primate samples still considered infectious that are removed from BSC will only be transported to the designated incubator in NA2.510, the -80 C freezer in NA2.510 or other approved laboratory spaces (as indicated on the Lab Safety Plan Schedule F), using secondary containment from all exits from NA2.510.
10. The following method will inactivate the non-human primate samples to be used for work outside of the BSC:

    On dry ice, frozen tissue samples will be placed in a round-bottomed tube in an S-block (from Qiagen) containing a 5- or 7-mm stainless steel bead and incubated on dry ice for 15 minutes. Each S-Block is then moved to room temperature and 0.6 mL of RLT buffer with beta-mercaptoethanol added (from Qiagen All prep Kit) will be added to each tube, then the lid is closed. The tube containing sample and buffer will be placed in a Qiagen TissueLyser LT inside the BSC, and the tissue will be lysed according to the manufacturer's instructions. Following complete lysis of the tissue, the samples are considered safe for use outside of BSC and are no longer considered BSL2. Care should be taken to ensure that the samples are completely lysed.

11. Removal of waste materials from the cabinet:
    a. Following a contact time of 30 minutes, bleach tray and aspirator waste flask materials are disposed down the drain with plenty of water.

b. Chemicals used for inactivating agent may be flushed down the drain with plenty of water and/or aspirated, followed by bleach and 70% ethanol.
c. The autoclave waste bag will be loosely tied with autoclave tape, and an X placed over the biohazard sign. The bag will be immediately autoclaved, then disposed of as non-hazardous trash.

12. When finished working in BSC, wipe down all surfaces with Vesphene (i.e. all materials and internal walls of the BSC). When dry, spray everything with 70% ethanol and leave to air dry. BSC is never to be turned off and the sash should remain open to the protective level at all times. UV light is not a recognized disinfectant.
13. When finished disinfecting all surfaces after working with non-human primate samples, remove your outer gloves, then your lab coat and mask, then your inner gloves. Dispose gloves and any disposable PPE in the Biohazard waste.
14. Wash hands inside NA2.510B when finished working. Wash hands in the main lab once you have left NA2.510B.

6.1.2 DNA Extraction from Animal Tissues using QIAamp 96 DNA QIAcube HT Kit (5) (Qiagen, 51331).

DNA extraction was performed per manufacture recommendation except that the buffer used was not RTL buffer but ATL buffer. Prepared ATL + Proteinase K at (33mL ATL + 3 mL ProK). Used 400uL for non-CNS tissues and 300uL for CNS tissues. After 2 rounds of Tissuelyser II, incubated overnight and transferred 200uL to fresh S-block for extraction. Considered safe outside of BSL-2 post incubation.

6.1.3 Total RNA Extraction from Animal Tissues using RNeasy 96 QIAcube HT Kit (5) (Qiagen, 74171).

RNA extraction was performed per manufacture recommendation except that buffer was Qiazol reagent, 750mL. Homogenized twice, incubated at room temperature for 5 min, added 150uL chloroform, shaken vigorously. Spun at top speed for 1min, then pulled top layer (300uL) into new S-block for extraction. Considered safe outside of BSL-2 after chloroform step.

6.1.4 cDNA Synthesis for qPCR with RT2 HT First Strand Kit (Qiagen, 330411)

cDNA synthesis was performed per manufacture recommendation.

6.1.5 Quantification of *AP4M1* in monkey gDNA samples.

Prepared by Yang Yu, UTSW Medical Center
Version: **09-06-2021**

A. Overview
This protocol is designed to use quantitative PCR (qPCR) to determine the double-stranded copies of the *AP4M1* DNA present in a purified genomic DNA sample. This SOP has been optimized and validated for use with monkey genomic DNA. The total amount of sample DNA (host genomes) is determined by SYBR green qPCR analysis with primers specific to Monkey GADPH, and the copies of h*AP4M1*opt DNA within each sample is determined by SYBR green qPCR analysis with primers specific for h*AP4M1*opt.

B. Quantification of *hAP4M1* DNA in gDNA sample

1. *Make plasmid DNA standards*

Dilute linearized plasmid DNA (pTRS-UsP-*hAP4M1opt*-BGHpA) to 1.59×10^9 double-stranded copies/µL stock in 10 mM Tris-EDTA buffer pH 8 (Invitrogen Cat# 9858) in 1.5 mL siliconized tubes (Fisher Cat# 02681331). Make a first dilution 1:200 to 7.95×10^6 copies following by serial 1:10 dilutions to 7.95 copies/µL. 2 µL of each dilution are loaded to each reaction. All dilutions are prepared with 10 mM Tris prepared from 1M Tris pH 8 (Invitrogen Cat# AM9855G) with UltraPure DNase/RNase-free distilled water (Invitrogen Cat# 10977-015). Vortex and spin briefly in every step.

1.59×10^7 copies/reaction	2 µL of 1.59×10^9 copies/µL stock + 398 µL of 10 mM Tris
10 µL ↓	
1.59×10^6 copies/reaction	+ 90 µL of 10 mM Tris
10 µL ↓	
1.59×10^5 copies/reaction	+ 90 µL of 10 mM Tris
10 µL ↓	
1.59×10^4 copies/reaction	+ 90 µL of 10 mM Tris
10 µL ↓	
1.59×10^3 copies/reaction	+ 90 µL of 10 mM Tris
10 µL ↓	
1.59×10^2 copies/reaction	+ 90 µL of 10 mM Tris
10 µL ↓	
15.9 copies/reaction	

2. *Prepare the qPCR SYBR master reactions*
   1) Set up gDNA samples as follows.

2× SYBR master mix	10 µL (Roche Cat# 04887352001)
Forward Primer (20 µM)	0.5 µL (CCCTGGGCGAAGGAACTATC, IDT, Inc)
Reverse Primer (20 µM)	0.5 µL (CACAGCCTCGGTCTGAATGA, IDT, Inc)
H_2O	7 µL (Teknova Cat# W3440)

   a. Pipet 18 µL of master mix into each well intended for gDNA samples.
   b. Add 2 µL of sample gDNA to the well.

   2) Set up standard plasmid DNA as follows.

2× SYBR mater mix	10 µL (Roche Cat# 04887352001)
Forward Primer (20 µM)	0.5 µL (CCCTGGGCGAAGGAACTATC, IDT, Inc)
Reverse Primer (20 µM)	0.5 µL (CACAGCCTCGGTCTGAATGA, IDT, Inc)
H_2O	7 µL (Teknova Cat# W3440)

   a. Pipet 18 µL of master mix into each well intended for standard curve samples.
   b. Add 2 µL of plasmid DNA standard to the appropriate well.
   c. Add H_2O as no template control.
   d. Seal the plate with the seal for qPCR usage. (Roche Cat# 04-729-749-001)
   e. Spin down the plate @ 1800 rpm for 10 sec.
   f. Cycle in the Roche LightCycler480.

C. Quantification of Monkey *GAPDH*

1. *Make genomic DNA standards*
   1) Measure the monkey liver gDNA concentration using the LVis plate with the CLARIOstar plate reader (BMG LABTECH). Use the concentration of 58.075 ng/µL as the highest dilution stock.

2) Make 6 serial 1:4 dilutions with 10 mM Tris from 58.075 ng /µL to 0.0142 ng/µL.

2. *Preparation of gDNA samples for qPCR run*
   1) Mix samples by pipetting up and down, then spin at 1800 rpm for 10 sec.

3. *Prepare the qPCR SYBR master reactions*
   1) Set up standard gDNA and gDNA samples as follows.
      2× SYBR mater mix 10 µL (Roche Cat# 04887352001)
      Forward Primer (20 µM) 0.25 µL (GGCCTCCAAGGAGTAAGACC, Sigma)
      Reverse Primer (20 µM) 0.25 µL (TCTCTTCCTCTTGTGCTCTCG, Sigma)
      H_2O 7.5 µL (PCR water Teknova Cat# W3440)
      a. Pipet 18 µL of master mix into each well intended for gDNA samples.
      b. Add 2 µL of gDNA standard or gDNA samples
      c. Add H_2O as no template control.
      d. Seal the plate with the seal for qPCR usage. (Roche Cat# 04-729-749-001)
      e. Spin down the plate @ 1800 rpm for 10 sec.
      f. Cycle in the Roche LightCycler480.

D. Running Cycles

hAP4M1opt

	**Target (^{0}C)**	**Time (hh:mm:ss)**	**Cycle**	**Acquisition Mode**	**Ramp Rate**	**Acquisitions**	**Detect Mode**
Denature	95	0:10:00	1	none	4.8		SyBr green I/HRM Dye
Amplification	95	0:00:10	55	none	4.8		
	60	0:00:10		none	2.5		
	72	0:00:10		single	4.8		
Melt	95	0:00:05	1	none	4.8		
	65	0:01:00		none	2.5		
	95			continuous	0.11	5/^{0}C	
Cool	40	0:00:10	1	none	2.5		

Monkey *GAPDH*

	**Target (^{0}C)**	**Time (hh:mm:ss)**	**Cycle**	**Acquisition Mode**	**Ramp Rate**	**Acquisitions**	**Detect Mode**
Denature	95	0:10:00	1	none	4.8		SyBr green I/HRM Dye
Amplification	95	0:00:10	45	none	4.8		
	60	0:00:10		none	2.5		
	72	0:00:10		single	4.8		
Melt	95	0:00:05	1	none	4.8		
	65	0:01:00		none	2.5		
	95			continuous	0.11	5/^{0}C	
Cool	40	0:00:10	1	none	2.5		

E. Analysis
Use Ab Quant/ 2nd derivative max in LightCycler 480 v1.5 software to calculate the number of genomes per sample relative to the plasmid DNA standard curve or the amount of host DNA in ng relative to the monkey gDNA standard. Use Tm calling as a quality control to check whether the specific product was amplified.

Calculation of number of copies of viral genome/µL

$$= \frac{Copy\ of\ virus\ genome\ as\ double\ strand\ DNA\ relative\ to\ the\ copy\ number\ of\ plasmid\ DNA\ standard}{2}$$

- *Calculation of genome copies and amount (µg) of host DNA relative to the ng amount of host DNA standard*

This calculation assumes that the average weight of a bp of a double strand DNA is 620 g/mol, thus the molecular weight of DNA of 3 billion bps in a haploid cell is 1.85×10^{12} g/mol. The quantity of DNA in each cell contains (1.85×10^{12} g/mol)×(1 mole/6.022×10^{23} molecules)×2=6 pg of diploid DNA, so 1 pg of DNA contains 0.167 double strand copies=0.334 single-stranded copies of DNA, and 1 ng=334 single-stranded copies.

Number of monkey GAPDH genome copies = $\frac{ng*334\ (single\ stranded\ copies=genome)}{dilution\ \ factor*2}$

Conversion from ng/µL to µg/µL amount of host DNA= ng/µL of host DNA ×1000

- *Calculation of number of copies of Viral genomes normalized to number of copies of genome host*

$$= \frac{Copies\ of\ viral\ genome/\mu L}{Copies\ of\ genome\ DNA\ in\ host/\mu L}$$

- Calculation of number of copies of Viral genomes normalized to amount of host DNA host (µg)

$$= \frac{Copies\ of\ viral\ genome/\mu L}{amount\ of\ DNA\ in\ host\ (\mu g)/\mu L}$$

6.1.6 Quantification of AP4M1 in monkey cDNA samples.
Prepared by Yang Yu, UTSW Medical Center
Version: **09-06-2021**

A. Overview
This protocol is designed to use quantitative PCR (qPCR) to determine the single-stranded copies of the *AP4M1* cDNA present in a synthesized cDNA sample. This SOP has been optimized and validated for use with monkey cDNA. The total amount of sample cDNA (host genomes) is determined by SYBR green qPCR analysis with primers specific to Monkey GADPH, and the copies of hAP4M1opt cDNA within each sample is determined by SYBR green qPCR analysis with primers specific for hAP4M1opt.

B. Quantification of hAP4M1 DNA in cDNA sample

1. *Make plasmid DNA standards*

Dilute linearized plasmid DNA (pTRS-UsP-*hAP4M1opt*-BGHpA) to 1.59×10^9 double-stranded copies/ µL stock in 10 mM Tris-EDTA buffer pH 8 (Invitrogen Cat# 9858) in 1.5 mL siliconized tubes (Fisher Cat# 02681331). Make a first dilution 1:200 to 7.95×10^6 copies/µL following by serial 1:10 dilutions to 7.95copies/µL. 2 µL of each dilution are loaded to each reaction. All dilutions are prepared with 10 mM Tris prepared from 1M Tris pH 8 (Invitrogen Cat# AM9855G) with UltraPure DNase/RNase-free distilled water (Invitrogen Cat# 10977-015). Vortex and spin briefly in every step.

1.59×10^7 copies/reaction 10 µL of 1.59×10^9 copies/µL stock + 90 µL of 10 mM Tris
10 µL
1.59×10^6 copies/reaction + 90 µL of 10 mM Tris
10 µL
1.59×10^5 copies/reaction + 90 µL of 10 mM Tris
10 µL
1.59×10^4 copies/reaction + 90 µL of 10 mM Tris
10 µL
1.59×10^3 copies/reaction + 90 µL of 10 mM Tris
10 µL
1.59×10^2 copies/reaction + 90 µL of 10 mM Tris
10 µL
15.9copies/reaction

2. *Prepare the qPCR SYBR master reactions*
   1) Set up cDNA samples as follows.

2× SYBR master mix	10 µL (Roche Cat# 04887352001)
Forward Primer (20 µM)	0.5 µL (CCCTGGGCGAAGGAACTATC, IDT, Inc)
Reverse Primer (20 µM)	0.5 µL (CACAGCCTCGGTCTGAATGA, IDT, Inc)
H_2O	7 µL (Teknova Cat# W3440)

   a. Pipet 18 µL of master mix into each well intended for cDNA samples.
   b. Add 2 µL of sample cDNA to the well.

   2) Set up standard plasmid DNA as follows.

2× SYBR mater mix	10 µL (Roche Cat# 04887352001)
Forward Primer (20 µM)	0.5 µL (CCCTGGGCGAAGGAACTATC, IDT, Inc)
Reverse Primer (20 µM)	0.5 µL (CACAGCCTCGGTCTGAATGA, IDT, Inc)
H_2O	7 µL (Teknova Cat# W3440)

   g. Pipet 18 µL of master mix into each well intended for standard curve samples.
   h. Add 2 µL of plasmid DNA standard to the appropriate well.
   i. Add H_2O as no template control.
   j. Seal the plate with the seal for qPCR usage. (Roche Cat# 04-729-749-001)
   k. Spin down the plate @ 1800 rpm for 10 sec.
   l. Cycle in the Roche LightCycler480.

C. Quantification of Monkey GAPDH

1. *Make genomic DNA standards*
   1) Measure the monkey liver gDNA concentration using the LVis plate with the CLARIOstar plate reader (BMG LABTECH). Use the concentration of 58.075ng/µL as the highest dilution stock.
   2) Make 6 serial 1:4 dilutions with 10 mM Tris from 58.075ng /µL to 0.0142ng/µL.

2. *Preparation of cDNA samples for qPCR run*
   1) Mix samples by pipetting up and down, then spin at 1800 rpm for 10 sec.

3. *Prepare the qPCR SYBR master reactions*

1) Set up standard gDNA and cDNA samples as follows.
   2× SYBR mater mix 10 µL (Roche Cat# 04887352001)
   Forward Primer (20 µM) 0.25 µL (GGCCTCCAAGGAGTAAGACC, Sigma)
   Reverse Primer (20 µM) 0.25 µL (TCTCTTCCTCTTGTGCTCTCG, Sigma)
   H_2O 7.5 µL (PCR water Teknova Cat# W3440)
   a. Pipet 18 µL of master mix into each well intended for cDNA samples.
   b. Add 2 µL of gDNA standard or cDNA samples
   c. Add H_2O as no template control.
   d. Seal the plate with the seal for qPCR usage. (Roche Cat#04-729-692-001)
   e. Spin down the plate @ 1800 rpm for 10 sec.
   f. Cycle in the Roche LightCycler480.

D. Running Cycles

hAP4M1opt

	**Target (^{0}C)**	**Time (hh:mm:ss)**	**Cycle**	**Acquisition Mode**	**Ramp Rate**	**Acquisitions**	**Detect Mode**
Denature	95	0:10:00	1	none	4.8		SyBr green I/HRM Dye
Amplification	95	0:00:10	55	none	4.8		
	60	0:00:10		none	2.5		
	72	0:00:10		single	4.8		
Melt	95	0:00:05	1	none	4.8		
	65	0:01:00		none	2.5		
	95			continuous	0.11	5/^{0}C	
Cool	40	0:00:10	1	none	2.5		

Monkey GAPDH

	**Target (^{0}C)**	**Time (hh:mm:ss)**	**Cycle**	**Acquisition Mode**	**Ramp Rate**	**Acquisitions**	**Detect Mode**
Denature	95	0:10:00	1	none	4.8		SyBr green I/HRM Dye
Amplification	95	0:00:10	45	none	4.8		
	60	0:00:10		none	2.5		
	72	0:00:10		single	4.8		
Melt	95	0:00:05	1	none	4.8		
	65	0:01:00		none	2.5		
	95			continuous	0.11	5/^{0}C	
Cool	40	0:00:10	1	none	2.5		

E. Analysis

Use Ab Quant/ 2nd derivative max in LightCycler 480 v 1.5 software to calculate the number of genomes per sample relative to the plasmid DNA standard curve or the amount of host cDNA in ng relative to the monkey gDNA standard. Use Tm calling as a quality control to check whether the specific product was amplified.

Calculation of number of copies of viral genome/µL

= Copy of virus genome as single strand cDNA relative to the copy number of plasmid DNA standard

- *Calculation of genome copies and amount (µg) of host cDNA relative to the ng amount of host DNA standard*

This calculation assumes that the average weight of a bp of a double strand DNA is 620 g/mol, thus the molecular weight of DNA of 3 billion bps in a haploid cell is 1.85×10^{12} g/mol. The quantity of DNA in each cell contains (1.85×10^{12} g/mol)×(1 mole/6.022×10^{23} molecules)×2=6 pg of diploid DNA, so 1 pg of DNA contains 0.167 double strand copies=0.334 single-stranded copies of DNA, and 1 ng=334 single-stranded copies of DNA.

$$\text{Number of monkey GAPDH genome copies} = \frac{ng*334\ (single\ stranded\ copies=genome)}{dilution\ \ factor*2}$$

Conversion from ng/µL to µg/µL amount of host DNA= ng/µL of host DNA ×1000

- *Calculation of number of copies of Viral genomes normalized to number of copies of genome host*

$$=\frac{Copies\ of\ viral\ genome/\mu L}{Copies\ of\ genome\ DNA\ in\ host/\mu L}$$

- Calculation of number of copies of Viral genomes normalized to amount of host DNA host (µg)

$$=\frac{Copies\ of\ viral\ genome/\mu L}{amount\ of\ DNA\ in\ host\ (\mu g)/\mu L}$$

6.1.7 Biodistribution qPCR Validation Studies.

Prepared by Yang Yu, UTSW Medical Center
Version: **01-13-2022**

A. Validation of pTRS-UsP-hAP4M1opt-BGHpA detection in no matrix.

Overview

The plasmid, pTRS-UsP-hAP4M1opt-BGHpA, was used as a template in 2 SYBR reactions using the hAP4M1opt primer set 2 to validate PCR efficiency and lower detection threshold.

Plasmid dilutions in no matrix

The plasmid was diluted from 1.59×10^7 copies to 1 copy, and 2 replicates of serial dilution were used in 2 independent runs (4 replicates total). The 2nd derivative max and fit points algorithm were applied to the analysis. T_m analysis was used to determine PCR product purity, which is also confirmed by using agarose gel electrophoresis. Among all samples, 100% of replicates at or above 1 copy per reaction were successfully detected. Conservatively, we set the lower limit of detection (LLOD) at 4 copies of the plasmid per reaction. One out of 4 replicates amplified in the negative control, and it was detected as 1.36 copies, which is below the LLOD (4 copies) that was used for data analysis. The PCR amplification efficiency was between 1.916 and 1.935 using the 2nd derivative max algorithm.

The variability (range) of the lower limit of quantitation (LLOQ) for detection of the AP4M1opt plasmid sequence was 11.1 to 20.8 copies detected with an input of 15.9 copies,3.69 to 8.75 copies detected at 8 copies level, 2.2 to 8.47 copies at 4 copies,1.06 to 4.78 at 2 copies, and 0.984 to 2.22 copies at 1 copy level in SYBR reaction with AP4M1opt primer set 2.

Results

Our qPCR validation studies detected the plasmid pTRS-UsP-hAP4M1opt-BGHpA, LLOD, and LLOQ when plasmid DNA alone was measured. Overall, our LLOD was 100% successful in the detection of 4 copies of AP4M1opt per reaction.

B. Validation of pTRS-UsP-hAP4M1opt-BGHpA in a monkey genomic DNA matrix.

Overview

The plasmid, pTRS-UsP-hAP4M1opt-BGHpA, together with a matrix of monkey genomic DNA, were used as a template in 2 SYBR reactions using the hAP4M1opt primer set 2 to validate PCR efficiency and lower detection threshold.

Plasmid dilutions in gDNA matrix

The plasmid was diluted from 1.59×10^7 copies to 1 copy, and 2 replicates of serial dilution were used in 2 independent runs (4 replicates total). All the dilutions were carried out in a matrix of 100 ng monkey genomic DNA. The 2nd derivative max and fit points algorithm were applied to the analysis. T_m analysis was used to determine PCR product purity, which is also confirmed by using agarose gel electrophoresis. For these results, minor background amplification was detected above a background signal. Among all reactions, 100% of replicates at or above 1 copy per reaction were successfully detected. Conservatively, the LLOD was set at 4 copies of plasmid per reaction. The efficiency was between 1.938 and 1.946 using the 2nd derivative max algorithm.

The variability (range) of the LLOQ for detection of the AP4M1opt plasmid sequence in the matrix of 100 ng monkey genomic DNA was 10.2 to 18.9 copies detected with an input of 15.9 copies, 5.31 to 6.93 copies detected at 8 copies level, 3.32 to 9.03 copies at 4 copies, 1.65 to 14.5 at 2 copies, and 0.0437 to 4.92 copies at 1 copy level in SYBR reaction with AP4M1opt primer set 2.

Results

Our qPCR validation studies detected the plasmid pTRS-UsP-hAP4M1opt-BGHpA at comparable efficiencies, LLOD, and LLOQ regardless of whether plasmid DNA alone was measured, or the plasmid DNA was detected in a matrix of monkey genomic DNA. In the data analysis of the actual samples that were run, any values below the threshold were considered as too low to call and thus excluded. Overall, our LLOD was 100% successful in the detection of 4 copies of AP4M1opt sequence in 100 ng of monkey genomic DNA (40 copies/ug), which conforms to the Food and Drug Administration (FDA) guidelines on conducting vector genome biodistribution studies. This plasmid is our production plasmid to make the proposed clinical vector

scAAV9/AP4M1opt. We conclude that these PCR conditions can be used to detect the copies of AP4M1opt viral genome in monkey genomic DNA samples.

6.1.8 ELISpot Using CTL Test Kits.
Prepared by Kathryn McMillan, UTSW Medical Center
Version:**1.2**

A. DAY 0 Protocol (Sterile conditions)

1. Prepare Capture Solution: 40uL of anti-INFγ capture solution is added to 10 mL of Diluent A.
2. 80uL of prepared solution is added to each well
3. Incubate plate overnight at 4°C in a fridge

B. DAY 1 Protocol (Sterile conditions)
Plate setting

	1	2	3	4	5	6	7	8	9	10	11	12
A	Sample 1					Sample 9					Cells-No Peptide Control	
B	Sample 2					Sample 10						
C	Sample 3					Sample 11					Cells + Stim. Control	
D	Sample 4					Sample 12						
E	Sample 5					Sample 13					No cells + IFNγ Control	
F	Sample 6					Sample 14						
G	Sample 7					Sample 15					No cells Control	
H	Sample 8					Sample 16						

Wells with peptide Add DMSO to make sure same conc. of DMSO with other wells

All work done in designated NHP hood or in NHP designated equipment

1. On dry ice, pull out frozen splenocytes/lymphocytes samples
2. Thaw the cells one at a time in water bath setup in 37°C incubator
3. Prepare 15ml tube with enough media to do a 1:10 dilution of your sample
   a. ex. 1ml of frozen cell + 9ml of media
4. Centrifuge at 330g for 5min at room temperature
5. Aspirate the media and resuspend pellet with 1ml of new media
   a. Helpful to set pipette to 250uL for resuspension
6. Prepare cell suspensions for counting: 10ul Cells + 90ul Trypan Blue in round bottom plates. Load 10uL into hemocytometer and count:
   a. Ex. 1

Cells Counted:	Current Conc. :	Desired Conc. :	Total Volume:
42	4.2E6/mL	2.0E6/mL	2.1mL

   b. Ex. 2

Cells Counted:	Current Conc. :	Desired Conc. :	Total Volume:
15	1.5E6/mL	2.0E6/mL	0.75mL

   c. In Ex. 1, you should add enough media to cell suspension for a total volume of 2.1 mL

d. In Ex. 2, you should repeat steps 4 and 5 and resuspend in the calculated total volume ≤1mL.

7. Prepare Peptide mix
    a. Peptide Pool: 10 mL media + calculated peptide from stock. Desired: 1ug/100uL/well
    b. Ex. Peptide pool conc: 1.4mg/mL
        i. 0.1mg / 1.4mg/mL × 1000 = 71.4 ul stock peptide in 10ml media
    c. Calculate DMSO conc. of peptide mix:
    d. Ex.: 71.4uL stock/ 10,000ul ×100= 0.714% DMSO (match this % in all samples)
        i. Ex.: 71.4uL stock/ 10,000ul ×100= 0.714% DMSO (match this % in all samples)
8. Prepare Positive/Negative controls:
    a. Cells + No Peptide group: 400ul of media + (0.714% DMSO from step 7)
        i. Ex: 400uL × 0.714% = 2.86uL DMSO
    b. Cells + Stim. group: 400ul of media + (0.714% DMSO from step 7) + 0.8ul of P+I Stimulation Cocktail
        i. Ex: 400uL × 0.714% = 2.86uL DMSO
    c. No cells + IFNγ group: 400ul media + 10uL IFNγ aliquot prepped ahead of time
    d. No cells group: 400ul of media

9. Aspirate the D0 Capture solution from the plate
10. Load 150uL 1× D-PBS, aspirate off.
11. Load 100uL of cells into plates according to layout. Pool remaining cell suspensions for control wells
12. To Cells-No Peptide control wells, add 100uL of cell pool + 100uL of prepared control from step 8a
13. To Cells + Stim. control wells, add 100uL of cell pool + 100uL of prepared control from step 8b
14. To No Cells + IFNγ control wells, add 100uL of IFNγ solution from step 8c + 100uL of peptide mix
15. To No Cells control wells, add 100uL media + 100uL of peptide mix
16. Add 100ul of Peptide mix into remaining experimental wells. ALL WELLS SHOULD BE AT 200uL
17. Wrap in foil, incubate it for 24hr at 37°C with 5-7% CO_2

C. Day 2 Protocol (Sterile conditions)

<u>*****All work done in designated NHP hood or in NHP designated equipment*****</u>

1. Prepare Wash Solutions for the day: 1x PBS, distilled water and 1× Tween-PBS 0.05%.
2. Prepare Detection Solution by diluting Detection Antibody according to your specific protocol.
    a. Given protocol: 40 ul of anti-INFγ Biotinylated Antibody + 10mL Diluent B
    b. Prep just before use. Can hold final wash in wells to prepare, then aspirate and load
3. Wash plate two times with PBS and then two times with 0.05% Tween-PBS, 200μl/well each time.
    a. Plate is considered non-hazardous after PBS-T washes since the Tween-20 will disrupt the viral envelope
4. Add 80μl/well Detection Solution. Incubate at RT, 2h.
5. Prepare Tertiary Solution by diluting the Tertiary Antibody according to your specific protocol.
    a. Given protocol: 10uL Streptavidin supplied by kit + 10mL of diluent C
    b. Prep just before use. Can hold final wash in wells to prepare, then aspirate and load
6. Wash plate three times with 0.05% Tween-PBS, 200μl/well.
7. Add 80μl/well of Strep-AP Solution. Incubate at RT, 30min.
8. Prepare Developer Solution according to your specific protocol.

   a. Given protocol: To 10mL of Diluent Blue, add 160uL of S1, mix by inversion 4×. Add 160uL S2, mix by inversion 4×. Add 93uL S3, mix by inversion. Wrap tube in foil to protect from light. **SOLUTION ORDER IS CRITICAL!**
   b. Prep just before use. Can hold final wash in wells to prepare, then aspirate and load
9. Wash plate two times with 0.05% Tween-PBS, and then two times with distilled water, 200µl/well each time.
10. Add Developer Solution, 80µl/well. Incubate at RT, 15 min.
11. Stop reaction by gently rinsing membrane with tap water, decant, and repeat three times. Remove protective underdrain of the plate and rinse back of plate with tap water. Air dry plate for 2 hours face-down in running hood or on paper towels for 24 hours on bench top.
12. Scan and count plate.

7. APPENDICES

7.1 Raw Data

Table 2. Raw data vg/NHP diploid genome

	Br-1	Br-2	Br-3	Br-4	Br-5	Br-6	Br-7	Br-8	Br-9	Br-10
1201		0.0023		0.0007	0.1129	0.0279	0.0716		0.0478	0.0192
1701	0.0003	0.0003	0.0128		0.0002	0.0001	0.0168	0.0009		
2701	0.0308	0.0359	0.0055	0.0046	0.2968	0.4889	0.0069		0.0131	0.0151
2702	0.0357	0.6364	0.0108	0.0085	0.1047	0.2050	0.5469	0.0174	0.0328	0.0202
3201		1.6917	0.1040	0.3734	0.0046		0.2270	2.0207	0.3665	0.3546
3701		0.1520	2.6114	0.1710	0.0107		2.2089	12.1331	0.7280	0.2603

	Br-11	Br-12	Br-13	Br-14	Br-15	Br-16	Br-17	Br-18	Br-19	Br-20
1201			0.0051	0.0507	0.0002	0.0002		0.0073	0.0056	0.0063
1701	0.0011			0.0138		0.0002	0.0002		0.0000	
2701	0.0362	0.6462	0.1302	0.2286	0.0503	0.0201	0.0535	0.0944	0.0073	0.1813
2702	0.0164	0.1489	0.4404	0.0144	0.0101	0.0083	0.0146	0.0119	0.0162	0.0042
3201	0.0021	1.7471	1.8765	0.0739	0.0511	0.0120		1.2634	7.3115	0.7319
3701	0.0072				0.7706	1.1426	0.0465	0.0264	0.0004	0.6108

	Br-21	Br-22	ON	Trig	SC-C	SC-T	SC-L	DRG-C	DRG- T	DRG-L
1201	0.0185	0.0005	0.0248	0.0013		0.0770	0.0442			
1701	0.0006	0.0010	0.0183	0.0002		0.1061	0.0749	0.0728	0.0967	0.0992
2701	0.0058	0.0317	0.0558	0.5743	0.0080	0.1685	0.0143	1.0445	0.6618	0.2565
2702	0.0036	0.0876	0.0207	0.1183	0.0262	0.2680	0.0548	0.1739	0.1457	0.1660
3201	0.5047	0.0073	0.0369	5.2388	0.9126	0.3686	1.9214	0.7248	0.0837	1.4973
3701	0.2193	0.3703	0.0695	0.7046	0.9402	0.8284		0.6463	0.4195	0.2361

	Sc	Tib	H	Lu	Thy	Li	SPL	K	G	Bic	Gas

1201	0.0002	0.0058	0.0010	0.0007	0.0000	0.0097		0.0007	0.0000	0.0002	0.0132
1701		0.0004	0.0001	0.0042	0.0001	0.0010	0.0021	0.0001	0.0001	0.0011	0.0004
2701	0.0025	0.0016	0.0107	0.0261	0.0003	3.0719	0.0110	0.0073	0.0013	0.0020	0.0130
2702	0.0034	0.0041	0.0282	0.0241	0.0010	7.6226	0.1780	0.0096	0.1291	0.0026	0.0030
3201	0.0071	0.0128	0.0685	0.1519	0.0289	61.4790	0.1192	0.0789	0.0082	0.0924	0.0305
3701	0.0034	0.0052	0.0005	0.0153	0.0000	1.0524	0.3672	0.0175	0.0010	0.0012	0.0014

Table 3. Raw data of expression: copies of AP4M1 per copy of GAPDH

	Br-1	Br-2	Br-3	Br-4	Br-5	Br-6	Br-7	Br-8	Br-9	Br-10
1201	0.00000	0.00000	0.00000	0.00000	0.00000	0.00000	0.00000	0.00000	0.00000	0.00001
1701	0.00000	0.00000	0.00002	0.00000	0.00000	0.00000	0.00000	0.00000	0.00000	0.00000
2701	0.00001	0.00003	0.00000	0.00000	0.00001	0.00005	0.00000	0.00002	0.01416	0.00037
2702	0.00001	0.00001	0.00001	0.00001	0.00002	0.00001	0.00000	0.00002	0.00007	0.00001
3201	0.00795	0.00394	0.00003	0.00002	0.02296	0.01594	0.00253	0.09336	0.00534	0.02763
3701	0.02072	0.01881	0.00002	0.00001	0.02398	0.02323	0.02872	0.06968	0.00068	0.05626

	Br-11	Br-12	Br-13	Br-14	Br-15	Br-16	Br-17	Br-18	Br-19	Br-20
1201	0.00000	0.00001	0.00000	0.00000	0.00000	0.00000	0.00000	0.00000	0.00000	0.00000
1701	0.00000	0.00000	0.00000	0.00000	0.00000	0.00000	0.00000	0.00000	0.00000	0.00000
2701	0.00005	0.00016	0.00021	0.00022	0.00001	0.00000	0.00003	0.00000	0.00003	0.00023
2702	0.00000	0.00168	0.00016	0.00006	0.00004	0.00001	0.00121	0.00006	0.00036	0.00084
3201	0.00175	0.01307	0.00590	0.00775	0.00442	0.00488	0.00673	0.00146	0.00347	0.00267
3701	0.00068	0.00031	0.00130	0.01024	0.01096	0.00073	0.02231	0.00133	0.00375	0.00146

	Br-21	Br-22	ON	Trig	SC-C	SC-T	SC-L	DRG-C	DRG- T	DRG-L
1201	0.00006	0.00000	0.00002	0.00000	0.00000	0.00000	0.02568	0.00003	0.00000	0.00000
1701	0.00000	0.00000	0.00001	0.00000	0.00000	0.00000	0.00000	0.00000	0.00000	0.00000
2701	0.00000	0.00008	0.00000	0.00046	0.00420	0.00033	0.00000	0.01284	0.00067	0.00075
2702	0.00001	0.00001	0.00001	0.00005	0.00028	0.00006	0.00071	0.01797	0.00107	0.00160
3201	0.00953	0.00883	0.00516	0.02011	0.02327	0.00033	0.02932	0.12872	0.06706	0.02010
3701	0.00572	0.01796	0.00052	0.00006	0.01652	0.15000	0.08312	0.00000	0.00778	

	Sc	Tib	H	Lu	Thy	Li	SPL	K	G	Bic	Gas
1201	0.00001	0.00000	0.00000	0.00000	0.00000	0.00000	0.00000	0.00000	0.00000	0.00000	0.00000
1701	0.00000	0.00000	0.00000	0.00001	0.00000	0.00000	0.00000	0.00000	0.00000	0.00000	0.00000
2701	0.00001	0.00005	0.00000	0.00000	0.00000	0.00212	0.00000	0.00002	0.00000	0.00000	0.00000
2702	0.00001	0.00000	0.00000	0.00000	0.00000	0.00192	0.00000	0.00000	0.00084	0.00000	0.00000
3201	0.00001	0.00001	0.00004	0.00000	0.00000	0.02520	0.00012	0.00000	0.00459	0.00001	0.00014
3701	0.00007	0.00000	0.00000	0.00000	0.00000	0.00001	0.00000	0.00000	0.00000	0.00000	0.00000

Table 4. Raw data of immune response: Spot# in ELISpot plate

ID	Against AAV9					Against AP4M1					Negative Control	Positive Control
1201	0	0	0	0	1	0	0	0	0	0	0	753
1701	0	0	0	0	0	0	0	1	0	0	0	552
2701	0	0	0	0	0	0	0	0	0	0	0	823
2702	0	0	0	0	0	2	1	0	3	3	0	1010
3201	0	0	0	0	0	0	0	0	0	0	0	850
3701	0	0	0	0	0	0	0	2	0	0	0	836
											0	1295
											0	1210

7.2 Test Article Certificate of Analysis

Appendix 12

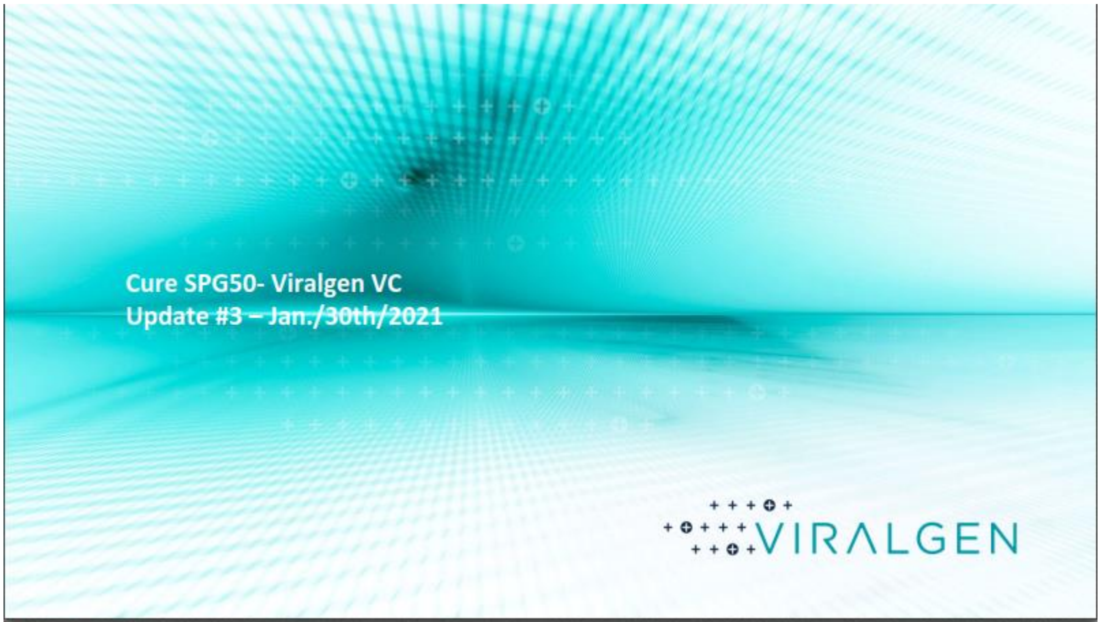
Cure SPG50- Viralgen VC
Update #3 – Jan./30th/2021
VIRALGEN

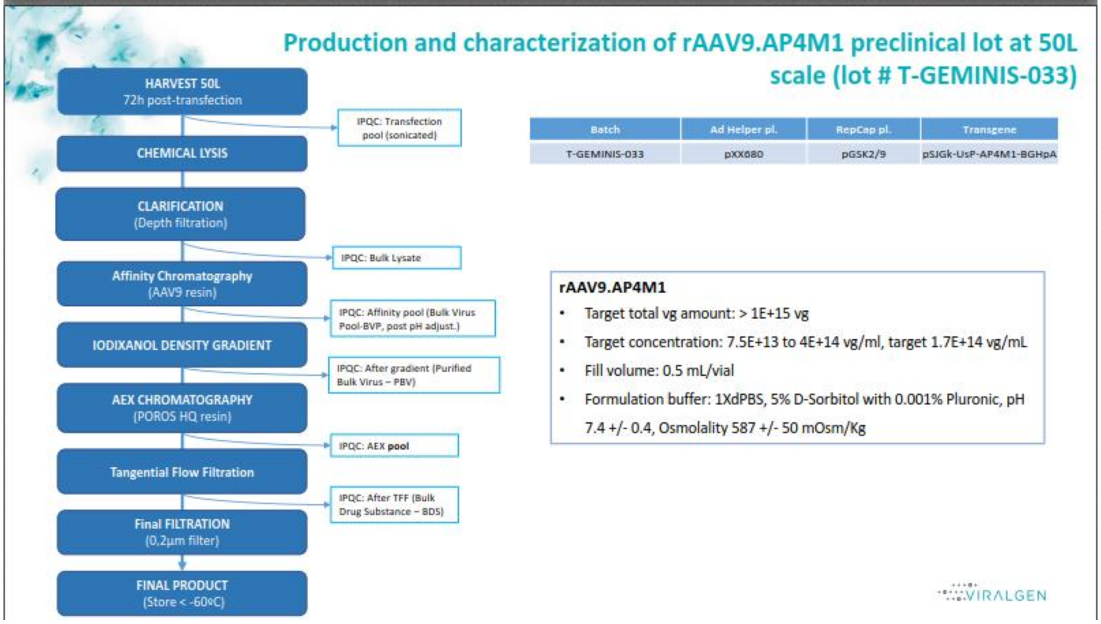
Production and characterization of rAAV9.AP4M1 preclinical lot at 50L scale (lot # T-GEMINIS-033)
HARVEST 50L
72h post-transfection
IPQC: Transfection pool (sonicated)
CHEMICAL LYSIS
CLARIFICATION
(Depth filtration)
IPQC: Bulk Lysate
Affinity Chromatography
(AAV9 resin)
IPQC: Affinity pool (Bulk Virus Pool-BVP, post pH adjust.)
IODIXANOL DENSITY GRADIENT
IPQC: After gradient (Purified Bulk Virus – PBV)
AEX CHROMATOGRAPHY
(POROS HQ resin)
IPQC: AEX pool
Tangential Flow Filtration
IPQC: After TFF (Bulk Drug Substance – BDS)
Final FILTRATION
(0,2µm filter)
FINAL PRODUCT
(Store < -60ºC)
Batch | Ad Helper pl. | RepCap pl. | Transgene
T-GEMINIS-033 | pXX680 | pGSK2/9 | pSJGk-UsP-AP4M1-BGHpA
rAAV9.AP4M1
• Target total vg amount: > 1E+15 vg
• Target concentration: 7.5E+13 to 4E+14 vg/ml, target 1.7E+14 vg/mL
• Fill volume: 0.5 mL/vial
• Formulation buffer: 1XdPBS, 5% D-Sorbitol with 0.001% Pluronic, pH 7.4 +/- 0.4, Osmolality 587 +/- 50 mOsm/Kg
VIRALGEN

Production and characterization of rAAV9.AP4M1 preclinical lot at 50L scale (lot # T-GEMINIS-033)

	T-GEMINIS-033		
	vg/mL	Volume (mL)	Total vg
Transfection Pool (TP)	9,46E+11	50000	4,73E+16
Bulk lysate (BL)	2,51E+11	57789	1,45E+16
**Clarification yield from TP**		**31%**	
Affinity load ratio (vg/mL resin)		2,90E+13	
NRF looses		<LOQ	
Bulk Virus Pool (BVP)	2,67E+13	468,6	1,25E+16
**Affinity Chromatography yield**		**86%**	
Final Purified Bulk Virus (PBV)	4,97E+13	333	1,66E+16
**Ultracentrifugation yield (final PBV)**		**132%**	
AEX load ratio (vg/mL resin)		1,08E+14	
NRF looses		5%	
AEX pool	2,21E+14	59,8	1,32E+16
**AEX Chromatography yield**		**80%**	
Post-TFF	1,71E+14	42,9	7,34E+15
**TFF yield**		**56%**	
BDS (after sampling)	1,71E+14	35	5,99E+15
Final Product	2,17E+14	34,5	7,49E+15

VIRALGEN

Production and characterization of rAAV9.AP4M1 preclinical lot at 50L scale (lot # T-GEMINIS-033)

SDS-PAGE Silver Stain (ITR qPCR titer)

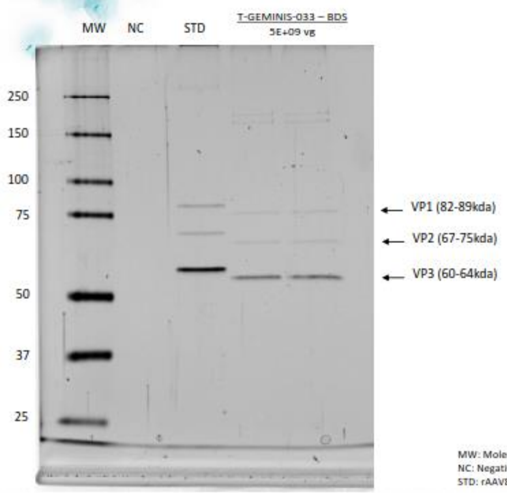

SDS-PAGE Western blot (ITR qPCR titer)

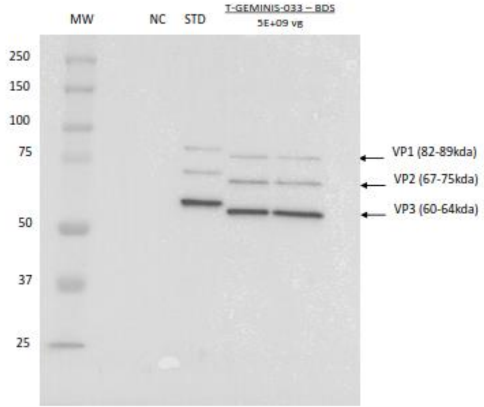

MW: Molecular weight marker
NC: Negative control
STD: rAAV8 (VR-1816) 5E+9vg

VIRALGEN

Appendix 12

Production and characterization of rAAV9.AP4M1 preclinical lot at 50L scale (lot # T-GEMINIS-033) – Drug substance

Test	Method	Specification	Result
**STRENGTH ASSAYS**			
Vector genome titer (vg/mL)	EP 2.6.21; USP<1127> ITR2 qPCR	> TBD vg/mL	1,71E+14
Vector genome titer (vg/mL)	EP 2.6.21; USP<1127> ITR2 ddPCR	> TBD vg/mL	5,17E+13
**PURITY ASSAYS**			
General purity	EP2.2.31; USP<1056> SDS-PAGE / Silver staining	Report result	Detection of VP1,VP2 and VP3. Additional extra bands at 150-200 Kda
Residual HCP	ELISA HEK293	Report result	< 100 ng/mL
Residual Host Cell DNA (pg / mL)	EP 2.6.21; USP<1127> qPCR 18S (2 amplicon sizes)	Report result	123bp Dnase + :6,86E+06 Dnase - : 7,38E+06 254bp Dnase +: 5,02E+06 Dnase - : 5,21E+06
Residual Host Cell DNA (pg/mL)	EP 2.6.21; USP<1127> qPCR E1A	Report result	On-going
Residual Plasmid DNA (copies/mL)	EP 2.6.21; USP<1127> qPCR Antibiotic-R	Report result	Dnase + : 9,61E+11 Dnase - : 1,26E+12
Full/Empty particles ratio	NS/Cryo-TEM	>50% full	On-going

VIRALGEN

Production and characterization of rAAV9.AP4M1 preclinical lot at 50L scale (lot # T-GEMINIS-033) – Drug substance

Test	Method	Specification	Result
**PURITY ASSAYS**			
Aggregation	nsTEM	Report result	On-going
Residual cells lysis reagent	HPLC	Report result	<LOD (1 ppm)
Residual clarifying reagent	HPLC	Report result	>0.21 ppm and <1.03ppm
Residual transfection reagent	HPLC	Report result	>12.57 ppm and <25.13 ppm
Residual Iodixanol	HPLC	Report result	>1.04 ppm and <3.12 ppm
Residual antifoam	ICP-OES	Report result	<LOD (5 ppm)
Residual Immunoaffinity ligand	ELISA	Report result	497.58 ng/mL
rcAAV	Infection of permissive cell line/qPCR	Report result	On-going
**IDENTITY ASSAYS**			
Protein Identity	SDS-PAGE/Western Blot	Detection of VP1, VP2 and VP3	Detection of VP1,VP2 and VP3
Genome identity	Sequencing	100% conform to sequence of reference	On-going

VIRALGEN

Appendix 12

Production and characterization of rAAV9.AP4M1 preclinical lot at 50L scale (lot # T-GEMINIS-033) – Drug product

Test	Method	Specification	Result
**SAFETY ASSAYS**			
Sterility	EP 2.6.1; USP<71>	No growth	No growth
Endotoxin (LAL kinetic chromogenic assay)	EP 2.6.14; USP<85>	< 0.2 EU / mL	0,123 EU/mL
**STRENGTH ASSAYS**			
Vector genome titer (vg/mL)	EP 2.6.21; USP<1127> ITR2 qPCR	7,5E13 to 4E14 vg/mL, targeting 1.7E14 vg/mL	2,17E+14
Vector genome titer (vg/mL)	EP 2.6.21; USP<1127> ITR2 ddPCR	.Report result	5,43E+13
Infectious particles titer (TCID50/mL)	TCID50	Report result	1,02E+10
VG/IP ratio	-	-	2,1E+04
TEA/BAA	CURE SPG50	To be reported by CURE SPG50	TBD
**QUALITY ASSAYS**			
Osmolality (mOsm/Kg)	EP 2.2.35; USP<785>	587 +/-50 mOsm/Kg	570,33
pH	EP 2.2.3; USP<791>	7.4±0.4	7,21
Appearance	Visual inspection	Colorless, clear to slightly opalescent, free of visible particles	Not done – DP vialled in opaque PP vials
Particle size distribution	DLS	Report result	On-going

VIRALGEN

Production and characterization of rAAV9.AP4M1 preclinical lot at 50L scale (lot # T-GEMINIS-033)

Final filling T-GEMINIS-033 -> December 22nd, 2020

- **Fill volume: nominal volume 0.5 mL/vial -> actual 0.65 mL/vial**
- **Filled vials:**
  - **69 x 0.5 mL**
  - **1 x 0.04 mL**
- **Shipment to UTSW on January 19th, 2021**

FINAL PRODUCT - Sampling plan	Actual vials
DP characterization	2 x 0.5 mL
Sterility	2 x 0.5 mL
Retention	2 x 0.5 mL
Reference for GMP lot	2x 0.5 mL
Stability study	27 x 0.5 mL
**TOTAL QC**	**35 x 0.5 mL**
**Final amount of vials available for CURE SPG50**	**34 x 0.5 mL**

VIRALGEN

FINAL REPORT

Study Phase: Pathology

Test Facility Study No. 5550014

Sponsor Reference No. UTSW-Gray-SPG50-004

GLP

TEST FACILITY:
Charles River Laboratories Montreal ULC
Senneville Site (CR-SEN)

TABLE OF CONTENTS

LIST OF APPENDICES 3
1. SUMMARY 4
2. INTRODUCTION 5
3. MATERIALS AND METHODS 5
3.1. Computerized Systems 6
4. RESULTS AND DISCUSSIONS 6
4.1. Mortality 6
4.2. Gross Pathology 6
4.2.1. Terminal Euthanasia Animals (Day 94) 6
4.3. Organ Weights 7
4.3.1. Terminal Euthanasia Animals (Day 94) 7
4.4. Histopathology 7
4.4.1. Terminal Euthanasia Animals (Day 94) 7
5. CONCLUSIONS 13
6. REPORT APPROVAL 14

LIST OF APPENDICES

Appendix 1 Individual Absolute Organ Weights 16
Appendix 2 Individual Organ Weights Relative to Body Weight 26
Appendix 3 Individual Organ Weights Relative to Brain Weight 36
Appendix 4 Individual Macroscopic and Microscopic Pathology 46
Appendix 5 Historical Data 58

1. SUMMARY

The objectives of this study were to determine the potential toxicity, biodistribution, and gene expression of AAV9/AP4M1, when given by a single intrathecal injection to monkeys, to evaluate the potential reversibility and/or progression of any findings, and to determine the potential impact of immunosuppressant (IMS) on the toxicity profile of this AAV9 vector.

One monkey per sex/group was assigned to the control group (reference item: PBS) or the high dose group (AAV9/AP4M1 at $1.68x10^{14}$ vg), and two female animals were assigned to the low dose group (AAV9/AP4M1 at $8.4x10^{13}$ vg). On Day 94, a complete gross pathological examination was performed and organ weights were recorded. A microscopic evaluation, was performed on selected tissues only (brain, spinal cord [cervical, thoracic, lumbar, including the injection site], dorsal root ganglia [cervical, thoracic, lumbar, with dorsal and ventral nerve roots], trigeminal ganglion, draining lymph nodes [deep cervical and iliac], liver, spleen, kidney, heart, skeletal muscle [gastrocnemius and biceps femoris], and nerves [optic, sciatic, sural, and tibial]).

The single intrathecal injection (slow bolus) of AAV9/AP4M1 to male and female monkeys at doses of $8.4x10^{13}$ or $1.68x10^{14}$ vg followed by a 93-day observation period resulted in pathological microscopic findings in the spinal cord (all segments, including the injection site), lumbar dorsal root ganglion (DRG), lumbar and thoracic dorsal nerve roots, brain, trigeminal ganglion, and peripheral nerves (sciatic, sural, and tibial).

At the injection site, axonal degeneration of the cauda equina was seen at $1.68x10^{14}$ vg in both sexes, while axonal degeneration of the dorsal funiculus and perivascular vacuolation of macrophages were seen in the male only. Perivascular infiltrates of mononuclear cells were noted in females at $\geq 8.4x10^{13}$ vg.

In the spinal cord, axonal degeneration of the dorsal funiculus was seen in all three segments and considered AAV9/AP4M1-related at $1.68x10^{14}$ vg in both sexes.

In the lumbar DRG, mononuclear cell infiltrates occurred at $\geq 8.4x10^{13}$ vg in both sexes, and neuronal degeneration was seen at $1.68x10^{14}$ vg in both sexes. In the dorsal lumbar nerve root, there was axonal degeneration at $1.68x10^{14}$ vg in both sexes, and mononuclear cell infiltrates in females at $\geq 8.4x10^{13}$ vg. Mononuclear cell infiltrates also occurred in the dorsal thoracic nerve root in the male at $1.68x10^{14}$ vg.

In the brain, axonal degeneration of the medulla oblongata was noted in the male at $1.68x10^{14}$ vg, and there was an increase in the severity of meningeal mononuclear cell infiltrates in both females at $8.4x10^{13}$ vg.

In the trigeminal ganglion, there was axonal degeneration and mononuclear cell infiltrates in the male at $1.68x10^{14}$ vg.

In the peripheral nerves (sciatic, sural, and tibial), there was an increase in severity of axonal degeneration at $1.68x10^{14}$ vg in both sexes, compared to controls, most likely exacerbated by the

edema/inflammation associated with experimental procedures (repetitive intramuscular injections).

Overall, there was an increase in the severity of axonal degeneration in the central and peripheral nervous system in animals at 1.68×10^{14} vg (severity up to moderate or marked), when compared to animals at 8.4×10^{13} vg (severity minimal or mild).

The results from the pathological evaluation of Fluoro-Jade B, IBA-1 and GFAP stained lumbar spinal cord (including injection site) and lumbar DRG sections generally paralleled/confirmed the results previously mentioned with H&E stained sections.

There were no preterminal deaths, organ weight changes or macroscopic findings related to the administration of AAV9/AP4M1.

2. INTRODUCTION

This report presents the pathology findings in monkeys assigned to Study No. 5550014. The objectives of this study were to determine the potential toxicity, biodistribution, and gene expression of AAV9/AP4M1, for the treatment of Spastic Paraplegia Type 50 (SPG50) caused by the AP4M1 gene mutation, when given by a single intrathecal injection to monkeys, to evaluate the potential reversibility and/or progression of any findings, and to determine the potential impact of immunosuppressant (IMS) on the toxicity profile of this AAV9 vector.

3. MATERIALS AND METHODS

Experimental procedures applicable to pathology investigations are summarized in Text Table 1.

Text Table 1
Experimental Design

Group No.	Test Material	Dose Level (vg)	Dose Volume (mL)	Dose Concentration (vg/mL)	No. of Animals Main Study Males	No. of Animals Main Study Females
1	Reference Item	0	1	0	1	1
2	AAV9/AP4M1	8.4×10^{13}	1.55	5.43×10^{13}	-	2
3	AAV9/AP4M1	1.68×10^{14}	3.10	5.43×10^{13}	1	1

In addition to the administration of AAV9/AP4M1, each main study animal received immunosuppressant material consisting of a single intravenous infusion of methylprednisolone succinate on Day 1, a daily intramuscular injection of methylprednisolone acetate from Day 1 to 93, and a twice daily intramuscular injection of rapamycin from Day -12 to 93 (see Main Report for additional details). Each intramuscular injection was performed in the lateral compartment of the thigh.

A complete gross pathological examination was performed on Day 94 on all main study animals and organ weights were recorded, as specified in the Study Plan. A microscopic evaluation was performed on hematoxylin and eosin (H&E) slides of selected tissues which consisted of: brain, spinal cord (cervical, thoracic, lumbar, including the injection site), dorsal root ganglia (DRG: cervical, thoracic, lumbar, with dorsal and ventral nerve roots), trigeminal ganglion, draining

lymph nodes (deep cervical and iliac), liver, spleen, kidney, heart, skeletal muscle (gastrocnemius and biceps femoris), and nerves (optic, sciatic, sural, and tibial). IBA-1, GFAP (glial fibrillary acidic protein) and Fluoro-Jade B staining were performed on the lumbar spinal cord (including the injection site) and the lumbar DRGs (including nerve roots) for all animals. Tissues that were supposed to be microscopically evaluated per study plan but were not available on the slide (and therefore not evaluated) are listed in the Individual Animal Data of the pathology report as not present. There was one missing tissue that did not affect the outcome or interpretation of the pathology portion of the study based on its single occurrence. Additional details along with deviations from these procedures may be found in the main study report.

3.1. Computerized Systems

Critical computerized systems used in this study phase are listed in Text Table 2.

Text Table 2
Computerized Systems

System Name	Version No.	Description of Data Collected and/or Analyzed
Provantis®	10	Terminal body weight, organ weight data, gross pathology and histopathology.<br>Analyses of numerical terminal data.
M-Files®	21	Reporting and collection of 21 CFR Part 11 compliant signature
Einfotree	7.6.0	Excel Module for collection of 21 CFR Part 11 compliance requirements, security, audit trail and electronic signatures

4. RESULTS AND DISCUSSIONS

4.1. Mortality

There were no unscheduled deaths during the course of this study.

4.2. Gross Pathology

4.2.1. Terminal Euthanasia Animals (Day 94)

(Appendix 4)

No AAV9/AP4M1-related gross findings were noted. The gross findings observed were considered incidental, related to experimental procedures/immunosuppressant administration, of the nature commonly observed in this strain and age of monkeys, and/or were of similar incidence in control and treated animals and, therefore, were considered unrelated to the administration of AAV9/AP4M1.

These included the small thymuses that were observed with a similar incidence across dose groups, including controls, and considered secondary to the administration of immunosuppressants and unrelated to the administration of AAV9/AP4M1. Furthermore, the macroscopic findings noted in the biceps femoris (swelling, thickness, dark/pale foci, and/or mottled discoloration) were seen with a similar incidence across dose groups, including controls,

and were considered secondary to the repetitive procedure of intramuscular injection in the thigh, and unrelated to the administration of AAV9/AP4M1.

4.3. Organ Weights

4.3.1. Terminal Euthanasia Animals (Day 94)

(Appendix 1, Appendix 2, and Appendix 3)

No AAV9/AP4M1-related organ weight changes were noted. There were isolated organ weight values that were different from their respective controls. There were, however, no patterns, trends, or correlating data to suggest these values were toxicologically relevant. Thus, the organ weight differences observed were considered incidental, related to experimental procedures/immunosuppressant administration, and/or related to difference of sexual maturity and unrelated to the administration of AAV9/AP4M1.

These included decreases in thymus weight (absolute or relative to body and brain weight), when compared to organ weight historical data at the Test Facility (Appendix 5), that were observed with a similar magnitude across dose groups, including control, and were considered secondary to the administration of immunosuppressants, and unrelated to the administration of AAV9/AP4M1.

4.4. Histopathology

4.4.1. Terminal Euthanasia Animals (Day 94)

(Appendix 4)

AAV9/AP4M1-related microscopic findings occurred in the spinal cord (all segments, including the injection site), lumbar DRG, lumbar and thoracic dorsal nerve roots, brain, trigeminal ganglion, and peripheral nerves (sciatic, sural, and tibial), and are summarized in Text Table 3 (H&E sections) and Text Table 4 (Fluoro-Jade B, IBA-1 and GFAP stains).

Appendix 13

Text Table 3
Summary of Microscopic Findings (H&E) – Scheduled Euthanasia (Day 94)

	Males		Females		
**Group**	**1**	**3**	**1**	**2**	**3**
**Dose (vg)**	**0**	**$1.68x10^{14}$**	**0**	**$8.4x10^{13}$**	**$1.68x10^{14}$**
**No. Animals per Group**	**1**	**1**	**1**	**2**	**1**
**Injection site (No. Examined)**	1	1	1	2	1
Degeneration; axonal, cauda equina	(0)[a]	(1)	(0)	(0)	(1)
Moderate	-	0	-	-	1
Marked	-	1	-	-	0
Degeneration; axonal, dorsal funiculus	(0)	(1)	(0)	(0)	(0)
Minimal	-	1	-	-	-
Vacuolation; perivascular, macrophage	(0)	(1)	(0)	(0)	(0)
Mild	-	1	-	-	-
Infiltration, mononuclear cell; perivascular	(0)	(0)	(0)	(1)	(1)
Minimal	-	-	-	1	1
**Spinal cord, lumbar (No. Examined)**	1	1	1	2	1
Degeneration; axonal, dorsal funiculus	(0)	(1)	(1)	(1)	(1)
Minimal	-	0	1	1	0
Mild	-	0	0	0	1
Moderate	-	1	0	0	0
**Spinal cord, thoracic (No. Examined)**	1	1	1	2	1
Degeneration; axonal, dorsal funiculus	(0)	(1)	(0)	(1)	(1)
Minimal	-	0	-	1	0
Mild	-	0	-	0	1
Moderate	-	1	-	0	0
**Spinal cord, cervical (No. Examined)**	1	1	1	2	1
Degeneration; axonal, dorsal funiculus	(0)	(1)	(0)	(1)	(1)
Minimal	-	0	-	1	0
Mild	-	1	-	0	1
**DRG, lumbar (No. Examined)**	1	1	1	2	1
Infiltration, mononuclear cell	(0)	(1)	(0)	(2)	(1)
Minimal	-	1	-	2	1
Degeneration; neuronal	(0)	(1)	(0)	(0)	(1)
Minimal	-	1	-	-	1
**Nerve root, dorsal, lumbar (No. Examined)**	1	1	1	2	1
Degeneration; axonal	(0)	(1)	(0)	(0)	(1)
Minimal	-	1	-	-	1
Infiltration, mononuclear cell	(0)	(0)	(0)	(1)	(1)
Minimal	-	-	-	1	1
**Nerve root, dorsal, thoracic (No. Examined)**	1	1	1	2	1
Infiltration, mononuclear cell	(0)	(1)	(0)	(0)	(0)
Minimal	-	1	-	-	-
**Brain (No. Examined)**	1	1	1	2	1
Degeneration; axonal, medulla oblongata	(0)	(1)	(0)	(0)	(0)
Minimal	-	1	-	-	-
Infiltration, mononuclear cell; meninges	(1)	(1)	(0)	(2)	(1)
Minimal	1	1	-	0	1
Mild	0	0	-	2	0

	Males		Females		
**Group**	**1**	**3**	**1**	**2**	**3**
**Dose (vg)**	**0**	**$1.68x10^{14}$**	**0**	**$8.4x10^{13}$**	**$1.68x10^{14}$**
**No. Animals per Group**	**1**	**1**	**1**	**2**	**1**
**Ganglion, trigeminal (No. Examined)**	1	1	1	2	1
Degeneration; axonal	(0)	(1)	(0)	(0)	(0)
Minimal	-	1	-	-	-
Infiltration, mononuclear cell	(0)	(1)	(0)	(0)	(0)
Minimal	-	1	-	-	-
**Nerve, sciatic (No. Examined)**	1	1	1	2	1
Degeneration; axonal	(1)	(1)	(1)	(2)	(1)
Minimal	1	0	0	0	0
Mild	0	1	1	2	0
Moderate	0	0	0	0	1
**Nerve, sural (No. Examined)**	1	1	1	2	1
Degeneration; axonal	(0)	(1)	(0)	(2)	(1)
Minimal	-	0	-	2	0
Mild	-	1	-	0	1
**Nerve, tibial (No. Examined)**	1	1	1	2	1
Degeneration; axonal	(1)	(1)	(1)	(2)	(1)
Minimal	1	0	1	1	0
Mild	0	1	0	1	1

[a] Numbers in parentheses represent the number of animals with the finding.

At the injection site, which was located at the level of the caudal lumbar spinal cord (target L5-L6), there was moderate to marked axonal degeneration of the nerve roots of the cauda equina in both animals given AAV9/AP4M1 at $1.68x10^{14}$ vg. The axonal degeneration consisted of vacuolation of the axonal sheath, swelling of the axon, and/or internal amorphous eosinophilic debris with or without macrophages (digestion chambers). In the male at $1.68x10^{14}$ vg, there was additional minimal axonal degeneration of the dorsal funiculus, as well as mild perivascular vacuolation of macrophages. Minimal perivascular infiltrates of mononuclear cells were also noted in 2 females at $\geq 8.4x10^{13}$ vg. These infiltrates were mainly composed of lymphocytes, with fewer macrophages. In general, the incidence and severity of axonal degeneration and/or mononuclear cell infiltrates at the injection site paralleled the incidence and severity of positive reaction to Fluoro-Jade B fluorescent stain (indicating degenerated axons), and increased intensity of IBA-1 (microglial cells) and/or GFAP (astrocytes) stains in the affected animals (Text Table 4).

In the lumbar spinal cord, there was an increase in the severity of axonal degeneration in the dorsal funiculus in both male and female at $1.68x10^{14}$ vg (graded mild to moderate), when compared to control animals. Positivity to Fluoro-Jade B stain and increased intensity of IBA-1 and/or GFAP stains reflected well this change in both animals at $1.68x10^{14}$ vg. Additionally, based on Fluoro-Jade B stain evaluation, minimal axonal degeneration was observed in the lateral and ventral funiculi of the female at $1.68x10^{14}$ vg.

In the thoracic and cervical spinal cord, there was mild or moderate axonal degeneration in the dorsal funiculus at $1.68x10^{14}$ vg in both sexes. Minimal severity of axonal degeneration seen in one female at $8.4x10^{13}$ vg was not considered related to AAV9/AP4M1 given the similar

incidence and severity of axonal degeneration noted in the spinal cord (lumbar) of the control female.

In the lumbar dorsal root ganglion, minimal mononuclear cell infiltrates occurred in all treated animals at $\geq 8.4x10^{13}$ vg, and correlated with minimal increased intensity of IBA-1 in 2 females at $\geq 8.4x10^{13}$ vg. Minimal neuronal degeneration was also noticed in the lumbar DRG in both male and female at $1.68x10^{14}$ vg. This latter change was characterized by the effacement/loss of rare neuronal cell bodies with presence of glial and/or mononuclear cells. Lumbar DRG findings were coupled with minimal axonal degeneration of the dorsal lumbar nerve root in both animals at $1.68x10^{14}$ vg, as well as minimal mononuclear cell infiltrates in females at $\geq 8.4x10^{13}$ vg, both changes correlating with Fluoro-Jade B stain positivity and/or increased intensity of IBA-1 and/or GFAP in these animals. Minimal mononuclear cell infiltrates were also seen in the dorsal thoracic nerve root in male at $1.68x10^{14}$ vg.

In the brain, there was minimal axonal degeneration at the periphery of the medulla oblongata in the male at $1.68x10^{14}$ vg, and there was an increase in the severity of meningeal mononuclear cell infiltrates in both females at $8.4x10^{13}$ vg (graded mild), when compared to control animals.

In the trigeminal ganglion, there was minimal axonal degeneration and mononuclear cell infiltrates in the male at $1.68x10^{14}$ vg.

In the peripheral nerves (sciatic, sural, and tibial), there was an increase in severity of axonal degeneration at $1.68x10^{14}$ vg in both sexes, compared to controls. This change was generally coupled with the presence of edema with or without mixed cell infiltrates dissecting through the nerve fibers and considered likely secondary to the inflammatory changes seen in the nearby biceps femoris (procedural-related changes, see below). Thus, the increase in severity of axonal degeneration seen in peripheral nerves was considered probably related to AAV9/AP4M1, but likely exacerbated by the edema/inflammation associated with experimental procedures (repetitive intramuscular injections).

Text Table 4
Summary of Microscopic Findings (IHC and special stain) – Scheduled Euthanasia (Day 94)

	Males		Females		
**Group**	**1**	**3**	**1**	**2**	**3**
**Dose (vg)**	**0**	**$1.68x10^{14}$**	**0**	**$8.4x10^{13}$**	**$1.68x10^{14}$**
**No. Animals per Group**	**1**	**1**	**1**	**2**	**1**
**Injection site, Fluoro-Jade B (No. Examined)**	1	1	1	2	1
Degeneration; axonal, cauda equina	(0)[a]	(1)	(0)	(0)	(1)
Moderate	-	1	-	-	1
Degeneration; axonal, dorsal funiculus	(0)	(1)	(0)	(1)	(0)
Minimal	-	1	-	1	-

Appendix 13

	Males		Females		
**Group**	**1**	**3**	**1**	**2**	**3**
**Dose (vg)**	**0**	**$1.68x10^{14}$**	**0**	**$8.4x10^{13}$**	**$1.68x10^{14}$**
**No. Animals per Group**	**1**	**1**	**1**	**2**	**1**
**Injection site, IBA-1 (No. Examined)**	1	1	1	2	1
Intensity, increased; cauda equina	(0)	(1)	(0)	(2)	(1)
Minimal	-	0	-	2	0
Moderate	-	1	-	0	1
Intensity, increased; dorsal funiculus	(0)	(1)	(0)	(0)	(1)
Minimal	-	1	-	-	1
**Injection site, GFAP (No. Examined)**	1	1	1	2	1
Intensity, increased; cauda equina	(0)	(1)	(0)	(2)	(1)
Minimal	-	0	-	2	0
Moderate	-	1	-	0	1
Intensity, increased; dorsal funiculus	(0)	(1)	(0)	(0)	(0)
Minimal	-	1	-	-	-
**Spinal cord, lumbar, Fluoro-Jade B (No. Examined)**	1	1	1	2	1
Degeneration; axonal, dorsal funiculus	(0)	(1)	(1)	(2)	(1)
Minimal	-	0	1	2	0
Mild	-	0	0	0	1
Moderate	-	1	0	0	0
Degeneration; axonal, lateral funiculus	(0)	(0)	(0)	(0)	(1)
Minimal	-	-	-	-	1
Degeneration; axonal, ventral funiculus	(0)	(0)	(0)	(0)	(1)
Minimal	-	-	-	-	1
**Spinal cord, lumbar, IBA-1 (No. Examined)**	1	1	1	2	1
Intensity, increased; dorsal funiculus	(0)	(1)	(0)	(1)	(1)
Minimal	-	0	-	1	0
Mild	-	0	-	0	1
Moderate	-	1	-	0	0
**Spinal cord, lumbar, GFAP (No. Examined)**	1	1	1	2	1
Intensity, increased; dorsal funiculus	(0)	(1)	(0)	(0)	(0)
Mild	-	1	-	-	-
**DRG, lumbar, IBA-1 (No. Examined)**	1	1	1	2	1
Intensity, increased	(0)	(0)	(0)	(1)	(1)
Minimal	-	-	-	1	1
**Nerve root, dorsal, lumbar, Fluoro-Jade B (No. Examined)**	1	1	1	2	1
Degeneration; axonal	(0)	(1)	(0)	(0)	(1)
Minimal	-	1	-	-	1
**Nerve root, dorsal, lumbar, IBA-1 (No. Examined)**	1	1	1	2	1
Intensity, increased	(0)	(0)	(0)	(2)	(1)
Minimal	-	-	-	2	1
**Nerve root, dorsal, lumbar, GFAP (No. Examined)**	1	1	1	2	1
Intensity, increased	(0)	(1)	(0)	(2)	(1)
Minimal	-	1	-	2	1

[a] Numbers in parentheses represent the number of animals with the finding.

Other microscopic findings observed were considered incidental, related to experimental procedures/immunosuppressant administration, of the nature commonly observed in this strain and age of monkeys, and/or were of similar incidence and severity in control and treated animals and, therefore, were considered unrelated to the administration of AAV9/AP4M1.

These included minimal to mild decreased lymphoid cellularity in the cervical and iliac lymph nodes and/or spleen that were seen with a similar incidence and severity in all groups, including controls, and considered secondary to the administration of immunosuppressants and unrelated to the administration of AAV9/AP4M1.

There was minimal axonal degeneration in the spinal cord and minimal mononuclear cell infiltrates in the brain (perivascular) and meninges, that were seen in several animals, including controls, and that were considered secondary to the procedure of intrathecal injection and unrelated to the administration of AAV9/AP4M1.

In the biceps femoris, microscopic findings of edema, necrosis/inflammation, myofiber degeneration/atrophy, and fibrosis (with gross correlates of swelling, thickness, dark/pale foci, and/or mottled discoloration) occurred with a similar incidence and severity across dose groups, including control animals. These findings were all considered secondary to the repetitive procedure of intramuscular injection in the thigh, and unrelated to the administration of AAV9/AP4M1.

In the iliac lymph nodes, minimal or mild sinus histiocytosis was also noted with a similar incidence and severity across dose groups, including controls, and was considered to be a physiological response to the inflammatory changes in the biceps femoris.

5. CONCLUSIONS

The single intrathecal injection (slow bolus) of AAV9/AP4M1 to male and female monkeys at doses of 8.4×10^{13} or 1.68×10^{14} vg followed by a 93-day observation period resulted in pathological microscopic findings in the spinal cord (all segments, including the injection site), lumbar DRG, lumbar and thoracic dorsal nerve roots, brain, trigeminal ganglion, and peripheral nerves (sciatic, sural, and tibial).

At the injection site, axonal degeneration of the cauda equina was seen at 1.68×10^{14} vg in both sexes, while axonal degeneration of the dorsal funiculus and perivascular vacuolation of macrophages were seen in the male only. Perivascular infiltrates of mononuclear cells were noted in females at $\geq 8.4 \times 10^{13}$ vg.

In the spinal cord, axonal degeneration of the dorsal funiculus was seen in all three segments and considered AAV9/AP4M1-related at 1.68×10^{14} vg in both sexes.

In the lumbar DRG, mononuclear cell infiltrates occurred at $\geq 8.4 \times 10^{13}$ vg in both sexes, and neuronal degeneration was seen at 1.68×10^{14} vg in both sexes. In the dorsal lumbar nerve root, there was axonal degeneration at 1.68×10^{14} vg in both sexes, and mononuclear cell infiltrates in females at $\geq 8.4 \times 10^{13}$ vg. Mononuclear cell infiltrates also occurred in the dorsal thoracic nerve root in the male at 1.68×10^{14} vg.

In the brain, axonal degeneration of the medulla oblongata was noted in the male at 1.68×10^{14} vg, and there was an increase in the severity of meningeal mononuclear cell infiltrates in both females at 8.4×10^{13} vg.

In the trigeminal ganglion, there was axonal degeneration and mononuclear cell infiltrates in the male at 1.68×10^{14} vg.

In the peripheral nerves (sciatic, sural, and tibial), there was an increase in severity of axonal degeneration at 1.68×10^{14} vg in both sexes, compared to controls, most likely exacerbated by the edema/inflammation associated with experimental procedures (repetitive intramuscular injections).

Overall, there was an increase in the severity of axonal degeneration in the central and peripheral nervous system in animals at 1.68×10^{14} vg (severity up to moderate or marked), when compared to animals at 8.4×10^{13} vg (severity minimal or mild).

The results from the pathological evaluation of Fluoro-Jade B, IBA-1 and GFAP stained lumbar spinal cord (including injection site) and lumbar DRG sections generally paralleled/confirmed the results previously mentioned with H&E stained sections.

6. REPORT APPROVAL

All electronic signatures appear at the end of the document upon finalization.

Individual Pathology Explanation Page

Abbreviation	Description
(G)	Gross Pathology
(H)	Histopathology
(TGL)	Trackable Gross Lesion
< or >	Value outside the validation rule range in Provantis
Cass	Cassette
GALT	Gut associated lymphoid tissue
ID	Identification
LN	Lymph Node
LT	Left
M	Mass
RT	Right
SS	Special Stain
Wt	Weight
FC	Flag comment
I	Macroscopic pathology – Included in mean
RC	Result comment
BW	Body Weight
LIBW	Lung infused before weighing
UPTD	Unable to performed due to technical difficulty
%bw	% Relative to Body Weight
%br	% Relative to Brain Weight

Note: This is a comprehensive list of abbreviations. All of the abbreviations listed may not be applicable to this report.

Dosing Information

Dosing information is abbreviated on various data outputs; the following represents the dosing information for this study.

Group No.	Test Material	Dose Level (vg)
1	Reference Item	0
2	AAV9/AP4M1	$8.4x10^{13}$
3	AAV9/AP4M1	$1.68x10^{14}$

Appendix 13

Appendix 1

Individual Absolute Organ Weights

5550014

Sex: Male Day(s) Relative to Start Date

0<br>vg<br>Group 1	Terminal Body Wt (Kg)	Organ Weight (Absolute)<br>Brain (g)	Epididymis (g)	Gland Adrenal (g)	Gland Pituitary (g)	Gland Prostate (g)	Thyroid/ Parathyroid (g)
	-	-	-	-	-	-	-
1201	3.1	76.895	5.673	0.373	0.051	0.189 I	0.651 I

I = Include

Appendix 13

Appendix 1

Individual Absolute Organ Weights

5550014

Sex: Male Day(s) Relative to Start Date

0	Organ Weight (Absolute)					
vg<br><br>Group 1	Heart<br><br>(g)	Kidney<br><br>(g)	Liver/<br>Gallbladder<br>(g)	Spleen<br><br>(g)	Testis<br><br>(g)	Thymus<br><br>(g)
	-	-	-	-	-	-
1201	14.685	14.510	68.400	3.102	4.307	0.323 I

I = Include

Appendix 13

Appendix 1

Individual Absolute Organ Weights

5550014

Sex: Male Day(s) Relative to Start Date

1.68 X10E14 vg Group 3		Organ Weight (Absolute)					
	Terminal Body Wt (Kg)	Brain (g)	Epididymis (g)	Gland Adrenal (g)	Gland Pituitary (g)	Gland Prostate (g)	Thyroid/ Parathyroid (g)
	-	-	-	-	-	-	-
3201	2.9	73.555	1.095 I	0.235	0.055	0.566	0.268

I = Include

Appendix 13

Appendix 1

Individual Absolute Organ Weights

5550014

Sex: Male Day(s) Relative to Start Date

1.68 X10E14 vg Group 3	Organ Weight (Absolute)					
	Heart (g)	Kidney (g)	Liver/ Gallbladder (g)	Spleen (g)	Testis (g)	Thymus (g)
	-	-	-	-	-	-
3201	10.743	11.535	60.246	2.696	2.624 I	0.233 I

I = Include

Appendix 13

Appendix 1

Individual Absolute Organ Weights

5550014

Sex: Female Day(s) Relative to Start Date

0	Organ Weight (Absolute)						
vg<br>Group 1	Terminal Body Wt (Kg)	Brain (g)	Gland Adrenal (g)	Gland Pituitary (g)	Thyroid/ Parathyroid (g)	Heart (g)	Kidney (g)
	-	-	-	-	-	-	-
1701	2.6	65.096	0.354	0.048	0.125	10.481	11.646

Appendix 13

Appendix 1

Individual Absolute Organ Weights

5550014

Sex: Female Day(s) Relative to Start Date

0	Organ Weight (Absolute)				
vg<br><br>Group 1	Liver/<br>Gallbladder<br>(g)	Ovary<br><br>(g)	Spleen<br><br>(g)	Thymus<br><br>(g)	Uterus/<br>Cervix<br>(g)
	-	-	-	-	-
1701	61.350	0.208	2.020	OA [a]	3.770

[a] [RC:Sample for bioanalysis already frozen.]

Appendix 13

Appendix 1

Individual Absolute Organ Weights

5550014

Sex: Female Day(s) Relative to Start Date

8.4 X10E13 vg Group 2	Organ Weight (Absolute)						
	Terminal Body Wt (Kg)	Brain (g)	Gland Adrenal (g)	Gland Pituitary (g)	Thyroid/ Parathyroid (g)	Heart (g)	Kidney (g)
	-	-	-	-	-	-	-
2701	2.4	61.512	0.423	0.067	0.355	10.152	14.326
2702	3.3	66.853	0.466	0.065	0.354 I	14.494	14.079

I = Include

Appendix 13

Appendix 1

Individual Absolute Organ Weights

5550014

Sex: Female Day(s) Relative to Start Date

8.4 X10E13 vg Group 2	Organ Weight (Absolute)				
	Liver/ Gallbladder (g)	Ovary (g)	Spleen (g)	Thymus (g)	Uterus/ Cervix (g)
	-	-	-	-	-
2701	62.937	0.370	3.456	0.355 I	7.875
2702	94.766	1.349 I	3.681	0.157 I	7.510

I = Include

Appendix 13

Appendix 1

Individual Absolute Organ Weights

5550014

Sex: Female Day(s) Relative to Start Date

1.68 X10E14 vg Group 3		Organ Weight (Absolute)					
	Terminal Body Wt (Kg)	Brain (g)	Gland Adrenal (g)	Gland Pituitary (g)	Thyroid/ Parathyroid (g)	Heart (g)	Kidney (g)
	-	-	-	-	-	-	-
3701	2.8	58.631	0.318	0.043	0.329	11.606	16.178

Appendix 13

Appendix 1

Individual Absolute Organ Weights

5550014

Sex: Female Day(s) Relative to Start Date

1.68 X10E14 vg Group 3	Organ Weight (Absolute)				
	Liver/ Gallbladder (g)	Ovary (g)	Spleen (g)	Thymus (g)	Uterus/ Cervix (g)
	-	-	-	-	-
3701	69.788	0.428	1.914	0.443 I	5.061

I = Include

Appendix 13

Appendix 2

Individual Organ Weights Relative to Body Weight

5550014

Sex: Male Day(s) Relative to Start Date

0	Organ Weight (BW Kg)						
vg<br><br>Group 1	Brain<br><br>(%bw)	Epididymis<br><br>(%bw)	Gland<br>Adrenal<br>(%bw)	Gland<br>Pituitary<br>(%bw)	Gland<br>Prostate<br>(%bw)	Thyroid/<br>Parathyroid<br>(%bw)	Heart<br><br>(%bw)
	-	-	-	-	-	-	-
1201	2.4805	0.1830	0.0120	0.0016	0.0061	0.0210	0.4737

Appendix 13

Appendix 2

Individual Organ Weights Relative to Body Weight

5550014

Sex: Male Day(s) Relative to Start Date

0	Organ Weight (BW Kg)				
vg<br><br>Group 1	Kidney<br><br>(%bw)	Liver/<br>Gallbladder<br>(%bw)	Spleen<br><br>(%bw)	Testis<br><br>(%bw)	Thymus<br><br>(%bw)
	-	-	-	-	-
1201	0.4681	2.2065	0.1001	0.1389	0.0104

Appendix 13

Appendix 2

Individual Organ Weights Relative to Body Weight

5550014

Sex: Male Day(s) Relative to Start Date

1.68 X10E14 vg Group 3	Organ Weight (BW Kg)						
	Brain (%bw)	Epididymis (%bw)	Gland Adrenal (%bw)	Gland Pituitary (%bw)	Gland Prostate (%bw)	Thyroid/ Parathyroid (%bw)	Heart (%bw)
	-	-	-	-	-	-	-
3201	2.5364	0.0378	0.0081	0.0019	0.0195	0.0092	0.3704

Appendix 13

Appendix 2

Individual Organ Weights Relative to Body Weight

5550014

Sex: Male Day(s) Relative to Start Date

1.68 X10E14 vg Group 3	Organ Weight (BW Kg)				
	Kidney (%bw)	Liver/ Gallbladder (%bw)	Spleen (%bw)	Testis (%bw)	Thymus (%bw)
	-	-	-	-	-
3201	0.3978	2.0774	0.0930	0.0905	0.0080

Appendix 13

Appendix 2

Individual Organ Weights Relative to Body Weight

5550014

Sex: Female Day(s) Relative to Start Date

0	Organ Weight (BW Kg)						
vg<br><br>Group 1	Brain<br><br>(%bw)	Gland<br>Adrenal<br>(%bw)	Gland<br>Pituitary<br>(%bw)	Thyroid/<br>Parathyroid<br>(%bw)	Heart<br><br>(%bw)	Kidney<br><br>(%bw)	Liver/<br>Gallbladder<br>(%bw)
	-	-	-	-	-	-	-
1701	2.5037	0.0136	0.0018	0.0048	0.4031	0.4479	2.3596

Appendix 13

Appendix 2

Individual Organ Weights Relative to Body Weight

5550014

Sex: Female Day(s) Relative to Start Date

0	Organ Weight (BW Kg)		
vg<br><br>Group 1	Ovary<br><br>(%bw)	Spleen<br><br>(%bw)	Uterus/<br>Cervix<br>(%bw)
	-	-	-
1701	0.0080	0.0777	0.1450

Appendix 13

Appendix 2

Individual Organ Weights Relative to Body Weight

5550014

Sex: Female Day(s) Relative to Start Date

8.4 X10E13 vg Group 2	Organ Weight (BW Kg)						
	Brain (%bw)	Gland Adrenal (%bw)	Gland Pituitary (%bw)	Thyroid/ Parathyroid (%bw)	Heart (%bw)	Kidney (%bw)	Liver/ Gallbladder (%bw)
	-	-	-	-	-	-	-
2701	2.5630	0.0176	0.0028	0.0148	0.4230	0.5969	2.6224
2702	2.0258	0.0141	0.0020	0.0107	0.4392	0.4266	2.8717

Appendix 13

Appendix 2

Individual Organ Weights Relative to Body Weight

5550014

Sex: Female Day(s) Relative to Start Date

8.4 X10E13 vg Group 2	Organ Weight (BW Kg)			
	Ovary (%bw)	Spleen (%bw)	Thymus (%bw)	Uterus/ Cervix (%bw)
	-	-	-	-
2701	0.0154	0.1440	0.0148	0.3281
2702	0.0409	0.1115	0.0048	0.2276

Appendix 13

Appendix 2

Individual Organ Weights Relative to Body Weight

5550014

Sex: Female Day(s) Relative to Start Date

1.68 X10E14 vg Group 3	Organ Weight (BW Kg)						
	Brain (%bw)	Gland Adrenal (%bw)	Gland Pituitary (%bw)	Thyroid/ Parathyroid (%bw)	Heart (%bw)	Kidney (%bw)	Liver/ Gallbladder (%bw)
	-	-	-	-	-	-	-
3701	2.0940	0.0114	0.0015	0.0118	0.4145	0.5778	2.4924

Appendix 13

Appendix 2

Individual Organ Weights Relative to Body Weight

5550014

Sex: Female Day(s) Relative to Start Date

1.68 X10E14 vg Group 3	Organ Weight (BW Kg)			
	Ovary (%bw)	Spleen (%bw)	Thymus (%bw)	Uterus/ Cervix (%bw)
	-	-	-	-
3701	0.0153	0.0684	0.0158	0.1808

Appendix 13

Appendix 3

Individual Organ Weights Relative to Brain Weight

5550014

Sex: Male Day(s) Relative to Start Date

0	Organ Weight/Brain Ratio						
vg<br><br>Group 1	Epididymis<br><br>(%br)	Gland Adrenal<br>(%br)	Gland Pituitary<br>(%br)	Gland Prostate<br>(%br)	Thyroid/ Parathyroid<br>(%br)	Heart<br><br>(%br)	Kidney<br><br>(%br)
	-	-	-	-	-	-	-
1201	7.3776	0.4851	0.0663	0.2458	0.8466	19.0975	18.8699

Appendix 13

Appendix 3

Individual Organ Weights Relative to Brain Weight

5550014

Sex: Male Day(s) Relative to Start Date

0	Organ Weight/Brain Ratio			
vg<br><br>Group 1	Liver/<br>Gallbladder<br>(%br)	Spleen<br><br>(%br)	Testis<br><br>(%br)	Thymus<br><br>(%br)
	-	-	-	-
1201	88.9525	4.0341	5.6011	0.4201

Appendix 13

Appendix 3

Individual Organ Weights Relative to Brain Weight

5550014

Sex: Male Day(s) Relative to Start Date

1.68 X10E14 vg Group 3	Organ Weight/Brain Ratio						
	Epididymis (%br)	Gland Adrenal (%br)	Gland Pituitary (%br)	Gland Prostate (%br)	Thyroid/ Parathyroid (%br)	Heart (%br)	Kidney (%br)
	-	-	-	-	-	-	-
3201	1.4887	0.3195	0.0748	0.7695	0.3644	14.6054	15.6821

Appendix 13

Appendix 3

Individual Organ Weights Relative to Brain Weight

5550014

Sex: Male Day(s) Relative to Start Date

1.68 X10E14 vg Group 3	Organ Weight/Brain Ratio			
	Liver/ Gallbladder (%br)	Spleen (%br)	Testis (%br)	Thymus (%br)
	-	-	-	-
3201	81.9061	3.6653	3.5674	0.3168

Appendix 13

Appendix 3

Individual Organ Weights Relative to Brain Weight

5550014

Sex: Female Day(s) Relative to Start Date

0	Organ Weight/Brain Ratio						
vg<br>Group 1	Gland Adrenal (%br)	Gland Pituitary (%br)	Thyroid/ Parathyroid (%br)	Heart (%br)	Kidney (%br)	Liver/ Gallbladder (%br)	Ovary (%br)
	-	-	-	-	-	-	-
1701	0.5438	0.0737	0.1920	16.1008	17.8905	94.2454	0.3195

Appendix 13

Appendix 3

Individual Organ Weights Relative to Brain Weight

5550014

Sex: Female Day(s) Relative to Start Date

0	Organ Weight/Brain Ratio	
vg<br><br>Group 1	Spleen<br><br>(%br)	Uterus/<br>Cervix<br>(%br)
	-	-
1701	3.1031	5.7914

Appendix 13

Appendix 3

Individual Organ Weights Relative to Brain Weight

5550014

Sex: Female Day(s) Relative to Start Date

8.4 X10E13 vg Group 2	Organ Weight/Brain Ratio						
	Gland Adrenal (%br)	Gland Pituitary (%br)	Thyroid/ Parathyroid (%br)	Heart (%br)	Kidney (%br)	Liver/ Gallbladder (%br)	Ovary (%br)
	-	-	-	-	-	-	-
2701	0.6877	0.1089	0.5771	16.5041	23.2898	102.3166	0.6015
2702	0.6971	0.0972	0.5295	21.6804	21.0596	141.7528	2.0179

Appendix 13

Appendix 3

Individual Organ Weights Relative to Brain Weight

5550014

Sex: Female Day(s) Relative to Start Date

8.4 X10E13 vg Group 2	Organ Weight/Brain Ratio		
	Spleen (%br)	Thymus (%br)	Uterus/ Cervix (%br)
	-	-	-
2701	5.6184	0.5771	12.8024
2702	5.5061	0.2348	11.2336

Appendix 13

Appendix 3

Individual Organ Weights Relative to Brain Weight

5550014

Sex: Female Day(s) Relative to Start Date

1.68 X10E14 vg Group 3	Organ Weight/Brain Ratio						
	Gland Adrenal (%br)	Gland Pituitary (%br)	Thyroid/ Parathyroid (%br)	Heart (%br)	Kidney (%br)	Liver/ Gallbladder (%br)	Ovary (%br)
	-	-	-	-	-	-	-
3701	0.5424	0.0733	0.5611	19.7950	27.5929	119.0292	0.7300

Appendix 13

Appendix 3

Individual Organ Weights Relative to Brain Weight

5550014

Sex: Female Day(s) Relative to Start Date

1.68 X10E14 vg Group 3	Organ Weight/Brain Ratio		
	Spleen (%br)	Thymus (%br)	Uterus/ Cervix (%br)
	-	-	-
3701	3.2645	0.7556	8.6320

Individual Macroscopic and Microscopic Pathology

5550014

Animal:	1201	Group:	1	Sex:	Male
Species:	Monkey	Strain:	Cyno Vietnam		
		Dose:	0 vg		
		Removal Reason:	Terminal Euthanasia		
		Study Day (Week) of Death:	94 (14)		

Gross Pathology Animal Details:

No animal details found

Gross Pathology Observations [Correlation]:

GLAND, PROSTATE : Small
GLAND, SEMINAL VESICLE : Small : (Comment) bilateral
GLAND, THYROID : Enlargement : (Comment) bilateral
MUSCLE, BICEPS FEMORIS : Focus, dark : (Comment) 2, left (TGL)
MUSCLE, BICEPS FEMORIS : Thick : (Comment) bilateral (TGL)
MUSCLE, BICEPS FEMORIS : Discoloration, mottled : (Comment) right (TGL)
THYMUS : Small

Any remaining study plan required tissues, which have been examined, have no visible lesions

Gross Pathology - The following Tissues were Not Examined:

None

Histopathology Animal Details:

No animal details found

Histopathology Observations [Correlation]:

BRAIN : Infiltration, mononuclear cell; perivascular, minimal
BRAIN : Infiltration, mononuclear cell; minimal, meninges
LYMPH NODE, CERVICAL : Cellularity, decreased; lymphoid, minimal
LYMPH NODE, ILIAC : Erythrocytosis; minimal : (Comment) with pigment
LYMPH NODE, ILIAC : Cellularity, decreased; lymphoid, minimal
LYMPH NODE, ILIAC : Sinus histiocytosis; mild
LYMPH NODE, ILIAC : Granuloma; minimal
MUSCLE, BICEPS FEMORIS : Edema; moderate [MUSCLE, BICEPS FEMORIS : Discoloration, mottled : (Comment) right (G) | MUSCLE, BICEPS FEMORIS : Thick : (Comment) bilateral (G)]
MUSCLE, BICEPS FEMORIS : Fibrosis; mild : (Comment) with myofiber atrophy
MUSCLE, BICEPS FEMORIS : Degeneration/atrophy; moderate, myofiber : (Comment) with regeneration
MUSCLE, BICEPS FEMORIS : Necrosis/inflammation; moderate : (Comment) with hemorrhage [MUSCLE, BICEPS FEMORIS : Discoloration, mottled : (Comment) right (G) | MUSCLE, BICEPS FEMORIS : Focus, dark : (Comment) 2, left (G)]
NERVE, SCIATIC : Degeneration; axonal, minimal
NERVE, SCIATIC : Infiltration, mononuclear cell; minimal
NERVE, SCIATIC : Edema; minimal : (Comment) with vacuolated macrophages
NERVE, TIBIAL : Degeneration; axonal, minimal
NERVE, TIBIAL : Infiltration, mononuclear cell; minimal
SITE, INJECTION : Infiltration, mononuclear cell; minimal, meninges
SPLEEN : Cellularity, decreased; lymphoid, minimal

Histopathology - The following Tissues were Within Normal Limits:

Individual Macroscopic and Microscopic Pathology

5550014

Animal: 1201 (Continued)	Group: 1	Sex: Male

GANGLION, DORSAL ROOT, CERVICAL; GANGLION, DORSAL ROOT, LUMBAR; GANGLION, DORSAL ROOT, LUMBAR, FLUOROJADE B SS; GANGLION, DORSAL ROOT, LUMBAR, GFAP SS; GANGLION, DORSAL ROOT, LUMBAR, IBA-1 SS; GANGLION, DORSAL ROOT, THORACIC; GANGLION, TRIGEMINAL; HEART; KIDNEY; LIVER; MUSCLE, GASTROCNEMIUS; NERVE, OPTIC; NERVE, SURAL; NERVE ROOT, DORSAL, CERVICAL; NERVE ROOT, DORSAL, LUMBAR; NERVE ROOT, DORSAL, LUMBAR, FLUOROJADE B SS; NERVE ROOT, DORSAL, LUMBAR, GFAP SS; NERVE ROOT, DORSAL, LUMBAR, IBA-1 SS; NERVE ROOT, DORSAL, THORACIC; NERVE ROOT, VENTRAL, CERVICAL; NERVE ROOT, VENTRAL, LUMBAR; NERVE ROOT, VENTRAL, LUMBAR FLUOROJADE B SS; NERVE ROOT, VENTRAL, LUMBAR, GFAP SS; NERVE ROOT, VENTRAL, LUMBAR, IBA-1 SS; NERVE ROOT, VENTRAL, THORACIC; SITE, INJECTION, FLUOROJADE B SS; SITE, INJECTION, GFAP - IHC SS; SITE, INJECTION, IBA-1 - IHC SS; SPINAL CORD, CERVICAL; SPINAL CORD, LUMBAR; SPINAL CORD, LUMBAR, FLUOROJADE B SS; SPINAL CORD, LUMBAR, GFAP SS; SPINAL CORD, LUMBAR, IBA-1 SS; SPINAL CORD, THORACIC

Histopathology - The following Tissues were Not Examined:

None

Individual Macroscopic and Microscopic Pathology

5550014

Animal:	1701	Group:	1	Sex:	Female
Species:	Monkey	Strain:	Cyno Vietnam		
		Dose:	0 vg		
		Removal Reason:	Terminal Euthanasia		
		Study Day (Week) of Death:	94 (14)		

Gross Pathology Animal Details:

No animal details found

Gross Pathology Observations [Correlation]:

GLAND, SALIVARY, MANDIBULAR : Swelling : (Comment) bilateral
MUSCLE, BICEPS FEMORIS : Focus, dark : (Comment) 2 to 3, bilateral (TGL)
MUSCLE, BICEPS FEMORIS : Swelling : (Comment) extending into sciatic nerve, right (TGL)
THYMUS : Small

Any remaining study plan required tissues, which have been examined, have no visible lesions

Gross Pathology - The following Tissues were Not Examined:

None

Histopathology Animal Details:

No animal details found

Histopathology Observations [Correlation]:

BRAIN : Infiltration, mononuclear cell; perivascular, minimal
GANGLION, DORSAL ROOT, THORACIC : Infiltration, mononuclear cell; minimal
LYMPH NODE, ILIAC : Cellularity, decreased; lymphoid, minimal
LYMPH NODE, ILIAC : Sinus histiocytosis; mild
MUSCLE, BICEPS FEMORIS : Edema; moderate [MUSCLE, BICEPS FEMORIS : Swelling : (Comment) extending into sciatic nerve, right (G)]
MUSCLE, BICEPS FEMORIS : Degeneration/atrophy; mild, myofiber : (Comment) with regeneration
MUSCLE, BICEPS FEMORIS : Necrosis/inflammation; moderate : (Comment) with hemorrhage [MUSCLE, BICEPS FEMORIS : Focus, dark : (Comment) 2 to 3, bilateral (G)]
NERVE, SCIATIC : Degeneration; axonal, mild
NERVE, SCIATIC : Hemorrhage; minimal
NERVE, SCIATIC : Edema; minimal : (Comment) with mixed cell infiltrates
NERVE, TIBIAL : Degeneration; axonal, minimal
SPINAL CORD, LUMBAR : Degeneration; axonal, minimal, dorsal funiculus
SPINAL CORD, LUMBAR, FLUOROJADE B SS : Degeneration; axonal, minimal, dorsal funiculus
SPLEEN : Cellularity, decreased; lymphoid, minimal

Histopathology - The following Tissues were Within Normal Limits:

Appendix 13

Appendix 4

Individual Macroscopic and Microscopic Pathology

5550014

Animal: 1701 (Continued)	Group: 1	Sex: Female

GANGLION, DORSAL ROOT, CERVICAL; GANGLION, DORSAL ROOT, LUMBAR; GANGLION, DORSAL ROOT, LUMBAR, FLUOROJADE B SS; GANGLION, DORSAL ROOT, LUMBAR, GFAP SS; GANGLION, DORSAL ROOT, LUMBAR, IBA-1 SS; GANGLION, TRIGEMINAL; HEART; KIDNEY; LIVER; LYMPH NODE, CERVICAL; MUSCLE, GASTROCNEMIUS; NERVE, OPTIC; NERVE, SURAL; NERVE ROOT, DORSAL, CERVICAL; NERVE ROOT, DORSAL, LUMBAR; NERVE ROOT, DORSAL, LUMBAR, FLUOROJADE B SS; NERVE ROOT, DORSAL, LUMBAR, GFAP SS; NERVE ROOT, DORSAL, LUMBAR, IBA-1 SS; NERVE ROOT, DORSAL, THORACIC; NERVE ROOT, VENTRAL, CERVICAL; NERVE ROOT, VENTRAL, LUMBAR; NERVE ROOT, VENTRAL, LUMBAR FLUOROJADE B SS; NERVE ROOT, VENTRAL, LUMBAR, GFAP SS; NERVE ROOT, VENTRAL, LUMBAR, IBA-1 SS; NERVE ROOT, VENTRAL, THORACIC; SITE, INJECTION; SITE, INJECTION, FLUOROJADE B SS; SITE, INJECTION, GFAP - IHC SS; SITE, INJECTION, IBA-1 - IHC SS; SPINAL CORD, CERVICAL; SPINAL CORD, LUMBAR, GFAP SS; SPINAL CORD, LUMBAR, IBA-1 SS; SPINAL CORD, THORACIC

Histopathology - The following Tissues were Not Examined:

None

Appendix 13

Appendix 4

Individual Macroscopic and Microscopic Pathology

5550014

Animal: 2701	Group: 2		Sex: Female
Species: Monkey	Strain: Cyno Vietnam		
	Dose: 8.4 X10E13 vg		
	Removal Reason: Terminal Euthanasia		
	Study Day (Week) of Death: 94 (14)		

Gross Pathology Animal Details:

No animal details found

Gross Pathology Observations [Correlation]:

MUSCLE, BICEPS FEMORIS : Thick : (Comment) right (TGL)
MUSCLE, BICEPS FEMORIS : Focus, pale : (Comment) 5, right (TGL)
THYMUS : Small

Any remaining study plan required tissues, which have been examined, have no visible lesions

Gross Pathology - The following Tissues were Not Examined:

None

Histopathology Animal Details:

No animal details found

Histopathology Observations [Correlation]:

BRAIN : Infiltration, mononuclear cell; perivascular, minimal
BRAIN : Infiltration, mononuclear cell; mild, meninges
GANGLION, DORSAL ROOT, LUMBAR : Infiltration, mononuclear cell; minimal
KIDNEY : Infiltration, mononuclear cell; interstitial, minimal
LIVER : Infiltration, mononuclear cell; minimal
LYMPH NODE, ILIAC : Erythrocytosis; mild : (Comment) with pigment
LYMPH NODE, ILIAC : Cellularity, decreased; lymphoid, minimal
LYMPH NODE, ILIAC : Sinus histiocytosis; minimal
MUSCLE, BICEPS FEMORIS : Edema; mild [MUSCLE, BICEPS FEMORIS : Thick : (Comment) right (G)]
MUSCLE, BICEPS FEMORIS : Fibrosis; mild : (Comment) with myofiber atrophy [MUSCLE, BICEPS FEMORIS : Focus, pale : (Comment) 5, right (G)]
MUSCLE, BICEPS FEMORIS : Degeneration/atrophy; mild, myofiber : (Comment) with regeneration
MUSCLE, BICEPS FEMORIS : Necrosis/inflammation; mild [MUSCLE, BICEPS FEMORIS : Focus, pale : (Comment) 5, right (G)]
MUSCLE, GASTROCNEMIUS : Infiltration, mononuclear cell; minimal
NERVE, SCIATIC : Degeneration; axonal, mild
NERVE, SURAL : Degeneration; axonal, minimal
NERVE, SURAL : Infiltration, mononuclear cell; minimal
NERVE, TIBIAL : Degeneration; axonal, minimal
NERVE ROOT, DORSAL, LUMBAR : Infiltration, mononuclear cell; minimal
NERVE ROOT, DORSAL, LUMBAR, GFAP SS : Intensity, increased; minimal
NERVE ROOT, DORSAL, LUMBAR, IBA-1 SS : Intensity, increased; minimal
SITE, INJECTION : Infiltration, mononuclear cell; perivascular, minimal
SITE, INJECTION, GFAP - IHC SS : Intensity, increased; minimal, cauda equina
SITE, INJECTION, IBA-1 - IHC SS : Intensity, increased; minimal, cauda equina

Appendix 13

Appendix 4

Individual Macroscopic and Microscopic Pathology

5550014

Animal: 2701 (Continued) Group: 2 Sex: Female

Histopathology Observations [Correlation] (Continued):

SPINAL CORD, CERVICAL : Degeneration; axonal, minimal, dorsal funiculus
SPINAL CORD, LUMBAR : Degeneration; axonal, minimal, dorsal funiculus
SPINAL CORD, LUMBAR, FLUOROJADE B SS : Degeneration; axonal, minimal, dorsal funiculus
SPINAL CORD, LUMBAR, IBA-1 SS : Intensity, increased; minimal, dorsal funiculus
SPINAL CORD, THORACIC : Degeneration; axonal, minimal, dorsal funiculus

Histopathology - The following Tissues were Within Normal Limits:

GANGLION, DORSAL ROOT, CERVICAL; GANGLION, DORSAL ROOT, LUMBAR, FLUOROJADE B SS; GANGLION, DORSAL ROOT, LUMBAR, GFAP SS; GANGLION, DORSAL ROOT, LUMBAR, IBA-1 SS; GANGLION, DORSAL ROOT, THORACIC; GANGLION, TRIGEMINAL; HEART; LYMPH NODE, CERVICAL; NERVE, OPTIC; NERVE ROOT, DORSAL, CERVICAL; NERVE ROOT, DORSAL, LUMBAR, FLUOROJADE B SS; NERVE ROOT, DORSAL, THORACIC; NERVE ROOT, VENTRAL, CERVICAL; NERVE ROOT, VENTRAL, LUMBAR; NERVE ROOT, VENTRAL, LUMBAR FLUOROJADE B SS; NERVE ROOT, VENTRAL, LUMBAR, GFAP SS; NERVE ROOT, VENTRAL, LUMBAR, IBA-1 SS; NERVE ROOT, VENTRAL, THORACIC; SITE, INJECTION, FLUOROJADE B SS; SPINAL CORD, LUMBAR, GFAP SS

Histopathology - The following Tissues were Not Examined:

SPLEEN - Not Present In Wet Tissues.

Appendix 13

Appendix 4

Individual Macroscopic and Microscopic Pathology

5550014

Animal:	2702	Group:	2	Sex:	Female
Species:	Monkey	Strain:	Cyno Vietnam		
		Dose:	8.4 X10E13 vg		
		Removal Reason: Terminal Euthanasia			
		Study Day (Week) of Death: 94 (14)			

Gross Pathology Animal Details:

No animal details found

Gross Pathology Observations [Correlation]:

GLAND, THYROID : Cyst, pale : (Comment) 2, right
LIVER : Focus, pale : (Comment) 1, medial right (TGL)
MUSCLE, BICEPS FEMORIS : Swelling : (Comment) bilateral, extending into sciatic nerve (TGL)
OVARY : Cyst, pale : (Comment) 1, left
SUBCUTIS : Focus, dark : (Comment) >10, lumbar
THYMUS : Small

Any remaining study plan required tissues, which have been examined, have no visible lesions

Gross Pathology - The following Tissues were Not Examined:

None

Histopathology Animal Details:

No animal details found

Histopathology Observations [Correlation]:

BRAIN : Infiltration, mononuclear cell; mild, meninges
GANGLION, DORSAL ROOT, LUMBAR : Infiltration, mononuclear cell; minimal
GANGLION, DORSAL ROOT, LUMBAR, IBA-1 SS : Intensity, increased; minimal
LYMPH NODE, CERVICAL : (Note) One Of A Pair Available For Evaluation.
LYMPH NODE, CERVICAL : Cellularity, decreased; lymphoid, minimal
LYMPH NODE, ILIAC : Erythrocytosis; minimal : (Comment) with pigment
LYMPH NODE, ILIAC : Sinus histiocytosis; minimal
MUSCLE, BICEPS FEMORIS : Edema; mild [MUSCLE, BICEPS FEMORIS : Swelling : (Comment) bilateral, extending into sciatic nerve (G)]
MUSCLE, BICEPS FEMORIS : Fibrosis; minimal
MUSCLE, BICEPS FEMORIS : Degeneration/atrophy; mild, myofiber
MUSCLE, BICEPS FEMORIS : Necrosis/inflammation; mild
NERVE, SCIATIC : Degeneration; axonal, mild
NERVE, SCIATIC : Edema; mild : (Comment) with vacuolated macrophages and mixed cell infiltrates
NERVE, SURAL : Degeneration; axonal, minimal
NERVE, SURAL : Edema; mild : (Comment) with vacuolated macrophages
NERVE, TIBIAL : Degeneration; axonal, mild
NERVE, TIBIAL : Edema; mild : (Comment) with vacuolated macrophages and mixed cell infiltrates
NERVE ROOT, DORSAL, LUMBAR, GFAP SS : Intensity, increased; minimal
NERVE ROOT, DORSAL, LUMBAR, IBA-1 SS : Intensity, increased; minimal
SITE, INJECTION, FLUOROJADE B SS : Degeneration; axonal, minimal, dorsal funiculus
SITE, INJECTION, GFAP - IHC SS : Intensity, increased; minimal, cauda equina
SITE, INJECTION, IBA-1 - IHC SS : Intensity, increased; minimal, cauda equina

Appendix 13

Appendix 4

Individual Macroscopic and Microscopic Pathology

5550014

Animal: 2702 (Continued) Group: 2 Sex: Female

Histopathology Observations [Correlation] (Continued):

SPINAL CORD, LUMBAR, FLUOROJADE B SS : Degeneration; axonal, minimal, dorsal funiculus
NO CORRELATE : No correlating lesion [LIVER : Focus, pale : (Comment) 1, medial right (G)]

Histopathology - The following Tissues were Within Normal Limits:

GANGLION, DORSAL ROOT, CERVICAL; GANGLION, DORSAL ROOT, LUMBAR, FLUOROJADE B SS; GANGLION, DORSAL ROOT, LUMBAR, GFAP SS; GANGLION, DORSAL ROOT, THORACIC; GANGLION, TRIGEMINAL; HEART; KIDNEY; LIVER; MUSCLE, GASTROCNEMIUS; NERVE, OPTIC; NERVE ROOT, DORSAL, CERVICAL; NERVE ROOT, DORSAL, LUMBAR; NERVE ROOT, DORSAL, LUMBAR, FLUOROJADE B SS; NERVE ROOT, DORSAL, THORACIC; NERVE ROOT, VENTRAL, CERVICAL; NERVE ROOT, VENTRAL, LUMBAR; NERVE ROOT, VENTRAL, LUMBAR FLUOROJADE B SS; NERVE ROOT, VENTRAL, LUMBAR, GFAP SS; NERVE ROOT, VENTRAL, LUMBAR, IBA-1 SS; NERVE ROOT, VENTRAL, THORACIC; SITE, INJECTION; SPINAL CORD, CERVICAL; SPINAL CORD, LUMBAR; SPINAL CORD, LUMBAR, GFAP SS; SPINAL CORD, LUMBAR, IBA-1 SS; SPINAL CORD, THORACIC; SPLEEN

Histopathology - The following Tissues were Not Examined:

None

Appendix 13

Appendix 4

Individual Macroscopic and Microscopic Pathology

5550014

Animal:	3201	Group:	3	Sex:	Male
Species:	Monkey	Strain:	Cyno Vietnam		
		Dose:	1.68 X10E14 vg		
		Removal Reason: Terminal Euthanasia			
		Study Day (Week) of Death: 94 (14)			

Gross Pathology Animal Details:

No animal details found

Gross Pathology Observations [Correlation]:

EPIDIDYMIS : Small : (Comment) bilateral
LUNG : Focus, pale : (Comment) 3 to >10
MUSCLE, BICEPS FEMORIS : Focus, dark : (Comment) 1, left (TGL)
MUSCLE, BICEPS FEMORIS : Thick : (Comment) bilateral (TGL)
MUSCLE, BICEPS FEMORIS : Focus, pale : (Comment) 3, right (TGL)
TESTIS : Small : (Comment) bilateral
THYMUS : Small

Any remaining study plan required tissues, which have been examined, have no visible lesions

Gross Pathology - The following Tissues were Not Examined:

None

Histopathology Animal Details:

No animal details found

Histopathology Observations [Correlation]:

BRAIN : Degeneration; axonal, minimal, medulla oblongata
BRAIN : Infiltration, mononuclear cell; perivascular, minimal
BRAIN : Infiltration, mononuclear cell; minimal, meninges
GANGLION, DORSAL ROOT, LUMBAR : Infiltration, mononuclear cell; minimal
GANGLION, DORSAL ROOT, LUMBAR : Degeneration; neuronal, minimal
GANGLION, TRIGEMINAL : Infiltration, mononuclear cell; minimal
GANGLION, TRIGEMINAL : Degeneration; axonal, minimal
LYMPH NODE, CERVICAL : Cellularity, decreased; lymphoid, minimal
LYMPH NODE, CERVICAL : Sinus histiocytosis; minimal
LYMPH NODE, ILIAC : Erythrocytosis; minimal
LYMPH NODE, ILIAC : Cellularity, decreased; lymphoid, mild
LYMPH NODE, ILIAC : Sinus histiocytosis; mild
MUSCLE, BICEPS FEMORIS : Edema; moderate [MUSCLE, BICEPS FEMORIS : Thick : (Comment) bilateral (G)]
MUSCLE, BICEPS FEMORIS : Fibrosis; minimal : (Comment) with myofiber atrophy [MUSCLE, BICEPS FEMORIS : Focus, pale : (Comment) 3, right (G)]
MUSCLE, BICEPS FEMORIS : Degeneration/atrophy; moderate, myofiber : (Comment) with regeneration
MUSCLE, BICEPS FEMORIS : Necrosis/inflammation; mild : (Comment) with hemorrhage [MUSCLE, BICEPS FEMORIS : Focus, dark : (Comment) 1, left (G) | MUSCLE, BICEPS FEMORIS : Focus, pale : (Comment) 3, right (G)]
NERVE, SCIATIC : Degeneration; axonal, mild
NERVE, SCIATIC : Edema; mild : (Comment) with vacuolated macrophages and mixed cell infiltrates

Appendix 13

Appendix 4

Individual Macroscopic and Microscopic Pathology

5550014

Animal: 3201 (Continued)	Group: 3	Sex: Male

Histopathology Observations [Correlation] (Continued):

NERVE, SURAL : Degeneration; axonal, mild
NERVE, SURAL : Edema; mild : (Comment) with vacuolated macrophages and mixed cell infiltrates
NERVE, TIBIAL : Degeneration; axonal, mild
NERVE, TIBIAL : Edema; mild : (Comment) with vacuolated macrophages and mixed cell infiltrates
NERVE ROOT, DORSAL, LUMBAR : Degeneration; axonal, minimal
NERVE ROOT, DORSAL, LUMBAR, FLUOROJADE B SS : Degeneration; axonal, minimal
NERVE ROOT, DORSAL, LUMBAR, GFAP SS : Intensity, increased; minimal
NERVE ROOT, DORSAL, THORACIC : (Note) One Of A Pair Available For Evaluation.
NERVE ROOT, DORSAL, THORACIC : Infiltration, mononuclear cell; minimal
SITE, INJECTION : Degeneration; axonal, marked, cauda equina
SITE, INJECTION : Degeneration; axonal, minimal, dorsal funiculus
SITE, INJECTION : Vacuolation; perivascular, mild, macrophage
SITE, INJECTION, FLUOROJADE B SS : Degeneration; axonal, moderate, cauda equina
SITE, INJECTION, FLUOROJADE B SS : Degeneration; axonal, minimal, dorsal funiculus
SITE, INJECTION, GFAP - IHC SS : Intensity, increased; moderate, cauda equina
SITE, INJECTION, GFAP - IHC SS : Intensity, increased; minimal, dorsal funiculus
SITE, INJECTION, IBA-1 - IHC SS : Intensity, increased; moderate, cauda equina
SITE, INJECTION, IBA-1 - IHC SS : Intensity, increased; minimal, dorsal funiculus
SPINAL CORD, CERVICAL : Degeneration; axonal, mild, dorsal funiculus
SPINAL CORD, LUMBAR : Degeneration; axonal, moderate, dorsal funiculus
SPINAL CORD, LUMBAR, FLUOROJADE B SS : Degeneration; axonal, moderate, dorsal funiculus
SPINAL CORD, LUMBAR, GFAP SS : Intensity, increased; mild, dorsal funiculus
SPINAL CORD, LUMBAR, IBA-1 SS : Intensity, increased; moderate, dorsal funiculus
SPINAL CORD, THORACIC : Degeneration; axonal, moderate, dorsal funiculus
SPLEEN : Cellularity, decreased; lymphoid, minimal

Histopathology - The following Tissues were Within Normal Limits:

GANGLION, DORSAL ROOT, CERVICAL; GANGLION, DORSAL ROOT, LUMBAR, FLUOROJADE B SS; GANGLION, DORSAL ROOT, LUMBAR, GFAP SS; GANGLION, DORSAL ROOT, LUMBAR, IBA-1 SS; GANGLION, DORSAL ROOT, THORACIC; HEART; KIDNEY; LIVER; MUSCLE, GASTROCNEMIUS; NERVE, OPTIC; NERVE ROOT, DORSAL, CERVICAL; NERVE ROOT, DORSAL, LUMBAR, IBA-1 SS; NERVE ROOT, VENTRAL, CERVICAL; NERVE ROOT, VENTRAL, LUMBAR; NERVE ROOT, VENTRAL, LUMBAR FLUOROJADE B SS; NERVE ROOT, VENTRAL, LUMBAR, GFAP SS; NERVE ROOT, VENTRAL, LUMBAR, IBA-1 SS; NERVE ROOT, VENTRAL, THORACIC

Histopathology - The following Tissues were Not Examined:

None

Appendix 13

Appendix 4

Individual Macroscopic and Microscopic Pathology

5550014

Animal:	3701	Group:	3	Sex:	Female
Species:	Monkey	Strain:	Cyno Vietnam		
		Dose:	1.68 X10E14 vg		
		Removal Reason: Terminal Euthanasia			
		Study Day (Week) of Death: 94 (14)			

Gross Pathology Animal Details:

No animal details found

Gross Pathology Observations [Correlation]:

LYMPH NODE, ILIAC : Discoloration, mottled : (Comment) right (TGL)
MUSCLE, BICEPS FEMORIS : Focus, dark : (Comment) 3, right (TGL)
MUSCLE, BICEPS FEMORIS : Swelling : (Comment) extending into sciatic nerve, left (TGL)
MUSCLE, BICEPS FEMORIS : Thick : (Comment) right (TGL)
MUSCLE, BICEPS FEMORIS : Focus, pale : (Comment) >10, right (TGL)
THYMUS : Small

Any remaining study plan required tissues, which have been examined, have no visible lesions

Gross Pathology - The following Tissues were Not Examined:

None

Histopathology Animal Details:

No animal details found

Histopathology Observations [Correlation]:

BRAIN : Infiltration, mononuclear cell; perivascular, minimal
BRAIN : Infiltration, mononuclear cell; minimal, meninges
GANGLION, DORSAL ROOT, LUMBAR : Infiltration, mononuclear cell; minimal
GANGLION, DORSAL ROOT, LUMBAR : Degeneration; neuronal, minimal
GANGLION, DORSAL ROOT, LUMBAR, IBA-1 SS : Intensity, increased; minimal
HEART : Infiltration, mononuclear cell; minimal : (Comment) with rare degenerated myocytes
LYMPH NODE, CERVICAL : Cellularity, decreased; lymphoid, minimal
LYMPH NODE, ILIAC : Erythrocytosis; mild [LYMPH NODE, ILIAC : Discoloration, mottled : (Comment) right (G)]
LYMPH NODE, ILIAC : Cellularity, decreased; lymphoid, minimal
LYMPH NODE, ILIAC : Sinus histiocytosis; mild
MUSCLE, BICEPS FEMORIS : Edema; marked [MUSCLE, BICEPS FEMORIS : Swelling : (Comment) extending into sciatic nerve, left (G) | MUSCLE, BICEPS FEMORIS : Thick : (Comment) right (G)]
MUSCLE, BICEPS FEMORIS : Fibrosis; minimal : (Comment) with myofiber atrophy [MUSCLE, BICEPS FEMORIS : Focus, pale : (Comment) >10, right (G)]
MUSCLE, BICEPS FEMORIS : Degeneration/atrophy; moderate, myofiber : (Comment) with regeneration
MUSCLE, BICEPS FEMORIS : Necrosis/inflammation; marked : (Comment) with hemorrhage [MUSCLE, BICEPS FEMORIS : Focus, dark : (Comment) 3, right (G) | MUSCLE, BICEPS FEMORIS : Focus, pale : (Comment) >10, right (G)]
NERVE, SCIATIC : Degeneration; axonal, moderate
NERVE, SCIATIC : Edema; minimal : (Comment) with vacuolated macrophages and mixed cell infiltrates
NERVE, SURAL : Degeneration; axonal, mild
NERVE, SURAL : Edema; minimal : (Comment) with vacuolated macrophages

Individual Macroscopic and Microscopic Pathology

5550014

Animal: 3701 (Continued) Group: 3 Sex: Female

Histopathology Observations [Correlation] (Continued):

NERVE, TIBIAL : Degeneration; axonal, mild
NERVE, TIBIAL : Edema; minimal : (Comment) with vacuolated macrophages and mixed cell infiltrates
NERVE ROOT, DORSAL, LUMBAR : Degeneration; axonal, minimal
NERVE ROOT, DORSAL, LUMBAR : Infiltration, mononuclear cell; minimal
NERVE ROOT, DORSAL, LUMBAR, FLUOROJADE B SS : Degeneration; axonal, minimal
NERVE ROOT, DORSAL, LUMBAR, GFAP SS : Intensity, increased; minimal
NERVE ROOT, DORSAL, LUMBAR, IBA-1 SS : Intensity, increased; minimal
NERVE ROOT, VENTRAL, LUMBAR, GFAP SS : (Note) One Of A Pair Available For Evaluation.
SITE, INJECTION : Infiltration, mononuclear cell; perivascular, minimal
SITE, INJECTION : Degeneration; axonal, moderate, cauda equina
SITE, INJECTION, FLUOROJADE B SS : Degeneration; axonal, moderate, cauda equina
SITE, INJECTION, GFAP - IHC SS : Intensity, increased; moderate, cauda equina
SITE, INJECTION, IBA-1 - IHC SS : Intensity, increased; moderate, cauda equina
SITE, INJECTION, IBA-1 - IHC SS : Intensity, increased; minimal, dorsal funiculus
SPINAL CORD, CERVICAL : Degeneration; axonal, mild, dorsal funiculus
SPINAL CORD, LUMBAR : Degeneration; axonal, mild, dorsal funiculus
SPINAL CORD, LUMBAR, FLUOROJADE B SS : Degeneration; axonal, mild, dorsal funiculus
SPINAL CORD, LUMBAR, FLUOROJADE B SS : Degeneration; axonal, minimal, lateral funiculus
SPINAL CORD, LUMBAR, FLUOROJADE B SS : Degeneration; axonal, minimal, ventral funiculus
SPINAL CORD, LUMBAR, IBA-1 SS : Intensity, increased; mild, dorsal funiculus
SPINAL CORD, THORACIC : Degeneration; axonal, mild, dorsal funiculus
SPLEEN : Cellularity, decreased; lymphoid, mild

Histopathology - The following Tissues were Within Normal Limits:

GANGLION, DORSAL ROOT, CERVICAL; GANGLION, DORSAL ROOT, LUMBAR, FLUOROJADE B SS; GANGLION, DORSAL ROOT, LUMBAR, GFAP SS; GANGLION, DORSAL ROOT, THORACIC; GANGLION, TRIGEMINAL; KIDNEY; LIVER; MUSCLE, GASTROCNEMIUS; NERVE, OPTIC; NERVE ROOT, DORSAL, CERVICAL; NERVE ROOT, DORSAL, THORACIC; NERVE ROOT, VENTRAL, CERVICAL; NERVE ROOT, VENTRAL, LUMBAR; NERVE ROOT, VENTRAL, LUMBAR FLUOROJADE B SS; NERVE ROOT, VENTRAL, LUMBAR, GFAP SS; NERVE ROOT, VENTRAL, LUMBAR, IBA-1 SS; NERVE ROOT, VENTRAL, THORACIC; SPINAL CORD, LUMBAR, GFAP SS

Histopathology - The following Tissues were Not Examined:

None

Appendix 13

Appendix 5

PATHOLOGY
ORGAN WEIGHT HISTORICAL DATA

Species / Strain: Monkey / Cyno Cambodian, Cyno China, Cyno Mauritius Island
Age at Necropsy: Males: From 1.5 to 6.1 years - Females: From 1.5 to 6.1 years
Treatment Duration: Males: 4 weeks - Females: 4 weeks
Route of Admin: All routes

		Males				Females			
**Organ**	**Unit**	**N**	**Mean**	**S.D.**	**Range***	**N**	**Mean**	**S.D.**	**Range***
Bodyweight	kg	78	2.6	0.6	1.8 - 4.6	81	2.5	0.3	2.1 - 3.1
Brain Weight	g	80	68.883	5.708	58.442 - 82.574	81	63.623	4.924	53.962 - 72.361
Epididymis Weight	g	67	0.515	0.233	0.232 - 1.115				
Gland Prostate/Seminal Vesicle	g	3	1.999	1.699	0.750 - 3.933				
Gland, Adrenal Weight	g	80	0.460	0.158	0.272 - 0.930	80	0.451	0.103	0.269 - 0.716
Gland, Pituitary Weight	g	71	0.035	0.010	0.017 - 0.056	72	0.043	0.011	0.022 - 0.070
Gland, Prostate Weight	g	76	0.225	0.340	0.051 - 1.760				
Gland, Salivary Weight	g	3	1.493	0.321	1.160 - 1.800	3	1.536	0.165	1.410 - 1.723
Gland, Seminal Vesicle Weight	g	3	0.479	0.173	0.321 - 0.664				
Gland, Thyroid Weight	g	68	0.327	0.137	0.148 - 0.798	69	0.316	0.088	0.144 - 0.540
Gland, Thyroid/Parathyroid Weigh	g	3	0.365	0.100	0.250 - 0.429	3	0.335	0.021	0.313 - 0.355
Heart Weight	g	78	9.948	2.040	7.518 - 17.920	81	9.474	1.623	7.260 - 13.560
Kidney Weight	g	79	11.363	2.476	8.485 - 20.790	81	10.913	1.752	8.515 - 14.508
Liver Weight	g	76	51.557	11.377	37.258 - 97.770	78	50.231	7.041	36.137 - 75.144
Liver/Gallbladder Weight	g	3	44.352	2.287	41.717 - 45.826	3	42.211	6.284	37.713 - 49.391
Lung Weight	g	33	12.158	1.858	6.559 - 14.955	32	12.161	1.347	9.402 - 14.708
Ovary Weight	g					80	0.238	0.102	0.083 - 0.453
Pancreas Weight	g	3	4.172	0.882	3.203 - 4.928	3	4.180	1.158	3.077 - 5.386
Spleen Weight	g	71	3.497	1.649	1.445 - 8.379	72	3.340	1.365	1.629 - 7.751
Testis Weight	g	78	1.806	4.570	0.455 - 19.847				
Thymus Weight	g	77	2.745	1.189	0.313 - 5.558	78	2.165	1.095	0.257 - 4.806
Uterus Weight	g					63	3.510	2.005	0.445 - 7.394
Uterus/Cervix	g					3	2.924	0.731	2.087 - 3.435

* N < 20: Lowest to highest; N > or equal 20: 2.5-97.5th percentile.

Appendix 13

Appendix 5

PATHOLOGY
ORGAN WEIGHT HISTORICAL DATA
Relative to Bodyweight as percentage

Species / Strain: Monkey / Cyno Cambodian, Cyno China, Cyno Mauritius Island
Age at Necropsy: Males: From 1.5 to 6.1 years - Females: From 1.5 to 6.1 years
Treatment Duration: Males: 4 weeks - Females: 4 weeks
Route of Admin: All routes

		Males				Females			
**Organ**	**Unit**	**N**	**Mean**	**S.D.**	**Range***	**N**	**Mean**	**S.D.**	**Range***
Brain Weight	g	80	2.67713	0.58666	0.98716 - 3.92589	81	2.53588	0.29948	1.98630 - 3.23360
Epididymis Weight	g	68	0.02070	0.00880	0.01060 - 0.04839				
Gland Prostate/Seminal Vesicle	g	3	0.07224	0.06870	0.02679 - 0.15127				
Gland, Adrenal Weight	g	80	0.01723	0.00493	0.01030 - 0.03017	81	0.01801	0.00379	0.01188 - 0.02508
Gland, Pituitary Weight	g	71	0.00138	0.00036	0.00071 - 0.00224	72	0.00173	0.00044	0.00110 - 0.00292
Gland, Prostate Weight	g	75	0.00666	0.00456	0.00212 - 0.02047				
Gland, Salivary Weight	g	3	0.06447	0.01678	0.04833 - 0.08182	3	0.06145	0.00660	0.05640 - 0.06892
Gland, Seminal Vesicle Weight	g	3	0.02038	0.00667	0.01459 - 0.02767				
Gland, Thyroid Weight	g	68	0.01279	0.00492	0.00548 - 0.02645	69	0.01268	0.00366	0.00519 - 0.02160
Gland, Thyroid/Parathyroid Weigh	g	3	0.01484	0.00424	0.01000 - 0.01788	3	0.01501	0.00091	0.01423 - 0.01600
Heart Weight	g	80	0.38243	0.03990	0.31540 - 0.46551	81	0.37315	0.03957	0.30796 - 0.46735
Kidney Weight	g	80	0.43124	0.05830	0.32692 - 0.55501	81	0.43030	0.04459	0.34974 - 0.51068
Liver Weight	g	77	1.94408	0.22426	1.29477 - 2.35589	78	1.97421	0.17383	1.66318 - 2.42400
Liver/Gallbladder Weight	g	3	1.79954	0.12173	1.66868 - 1.90942	3	1.88486	0.17188	1.71423 - 2.05796
Lung Weight	g	33	0.47227	0.04360	0.40120 - 0.57161	32	0.49469	0.04676	0.39500 - 0.62452
Ovary Weight	g					80	0.00933	0.00379	0.00332 - 0.01865
Pancreas Weight	g	3	0.18006	0.04533	0.13346 - 0.22400	3	0.16720	0.04632	0.12308 - 0.21544
Spleen Weight	g	71	0.13552	0.05261	0.06569 - 0.28893	72	0.13207	0.04537	0.07028 - 0.26439
Testis Weight	g	77	0.04797	0.04721	0.01788 - 0.10671				
Thymus Weight	g	77	0.10816	0.04605	0.00398 - 0.20585	78	0.08671	0.04443	0.00857 - 0.20896
Uterus Weight	g					63	0.13795	0.07541	0.02225 - 0.29448
Uterus/Cervix	g					3	0.13093	0.03178	0.09486 - 0.15481

* N < 20: Lowest to highest; N > or equal 20: 2.5-97.5th percentile.

Appendix 13

Appendix 5

PATHOLOGY
ORGAN WEIGHT HISTORICAL DATA
Relative to Brain Weight as percentage

Species / Strain: Monkey / Cyno Cambodian, Cyno China, Cyno Mauritius Island
Age at Necropsy: Males: From 1.5 to 6.1 years - Females: From 1.5 to 6.1 years
Treatment Duration: Males: 4 weeks - Females: 4 weeks
Route of Admin: All routes

		Males				Females			
**Organ**	**Unit**	**N**	**Mean**	**S.D.**	**Range***	**N**	**Mean**	**S.D.**	**Range***
Epididymis Weight	g	67	0.75817	0.36290	0.30217 - 1.53638				
Gland Prostate/Seminal Vesicle	g	3	3.00374	2.61488	1.15390 - 5.99534				
Gland, Adrenal Weight	g	80	0.67071	0.22771	0.39625 - 1.28630	81	0.72000	0.18052	0.43420 - 1.15595
Gland, Pituitary Weight	g	71	0.05147	0.01523	0.02288 - 0.08438	72	0.06802	0.01852	0.03733 - 0.11678
Gland, Prostate Weight	g	76	0.32302	0.46826	0.06867 - 2.55595				
Gland, Salivary Weight	g	3	2.02790	0.34014	1.63671 - 2.25383	3	2.46700	0.23973	2.26443 - 2.73167
Gland, Seminal Vesicle Weight	g	3	0.67205	0.29874	0.39112 - 0.98587				
Gland, Thyroid Weight	g	68	0.47429	0.19022	0.21910 - 1.07874	69	0.49908	0.14236	0.20683 - 0.85626
Gland, Thyroid/Parathyroid Weigh	g	3	0.53017	0.15292	0.36524 - 0.66724	3	0.51493	0.04359	0.47754 - 0.56280
Heart Weight	g	78	14.48705	2.85160	10.00073 - 24.22440	81	14.92849	2.54550	11.77709 - 20.55440
Kidney Weight	g	79	16.55495	3.55860	12.38969 - 29.31125	81	17.22306	2.86043	12.45684 - 24.27687
Liver Weight	g	76	75.05616	16.03733	54.12575 - 132.16627	77	78.67667	10.02926	56.22947 - 102.67506
Liver/Gallbladder Weight	g	3	64.57552	7.83566	55.95917 - 71.27459	3	64.64508	6.46708	57.53845 - 70.18459
Lung Weight	g	33	17.74072	2.66328	9.54383 - 22.65321	32	18.95269	2.58800	14.00056 - 24.90230
Ovary Weight	g					80	0.37800	0.16930	0.13019 - 0.76701
Pancreas Weight	g	3	5.64902	0.77909	4.75561 - 6.18704	3	6.68067	1.68626	5.24817 - 8.53904
Spleen Weight	g	71	5.09425	2.39420	2.10770 - 12.58939	72	5.26568	2.20328	2.55322 - 12.21919
Testis Weight	g	78	2.54940	6.01964	0.62809 - 26.82933				
Thymus Weight	g	77	4.02400	1.80615	0.45455 - 8.62976	78	3.38226	1.68433	0.42585 - 7.92520
Uterus Weight	g					63	5.53916	3.23654	0.76918 - 12.31253
Uterus/Cervix	g					3	4.50354	1.17697	3.18412 - 5.44538

* N < 20: Lowest to highest; N > or equal 20: 2.5-97.5th percentile.

SIGNATURE(S) FOR DOCUMENT: 5550014 - 5550014 Pathology Final Report

**Individual Scientist:**	I approve this document.
Name:	**Debien, Elaine**
	Debien, Elaine
Electronically Signed in M-Files	03-Mar-2022 15:30:36 (UTC+00:00)
	Timestamp

PATHOLOGY PEER REVIEW CERTIFICATE

5550014

A Single-Dose Study of AAV9/AP4M1 by Intrathecal Injection in Immunosuppressed Monkeys

1. PURPOSE:

The purpose of this pathology peer review was to assess the overall quality and consistency of the microscopic data and determine the validity of the study pathologist's conclusions.

A pathology peer review for this study was performed as follows for this study:

2. MATERIALS AND METHODS:

Group No.	Test Material	Dose Level (vg)	Dose Volume (mL)	Dose Concentration (vg/mL)	No. of Animals Main Study	
					Males	Females
1	Reference Item	0	1	0	1	1
2	AAV9/AP4M1	8.4×10^{13}	1.55	5.43×10^{13}	-	2
3	AAV9/AP4M1	1.68×10^{14}	3.10	5.43×10^{13}	1	1

1. Review of study plan and amendments, pathology report, histology records, clinical observations, organ weight data, clinical pathology data, etc.
2. Review of all tissues from the Males and Females in Groups 1 and 3 including animal numbers: 1201, 1701, 3201 and 3701, and Test-Item-target tissues (listed below) from the Females in Group 2 including animal numbers 2701 and 2702. Review included sections stained with hematoxylin and eosin (H&E), Fluoro-Jade B, IBA-1 and GFAP.
3. Review of all target organs from all animals in all groups to verify the effect levels (spinal cord (all segments, including the injection site), lumbar DRG, lumbar and thoracic dorsal nerve roots, brain, trigeminal ganglion, and peripheral nerves (sciatic, sural, and tibial)).

3. RESULTS

Following review of the microscopic findings reported by the study pathologist, any differences of opinion were resolved and agreement on terminology and diagnoses was achieved. The pathology report and histopathology tables accurately reflect the observations and conclusions agreed upon by the peer review pathologist.

All electronic signatures appear at the end of the document upon finalization.

SIGNATURE(S) FOR DOCUMENT: 5550014 - 5550014 Pathology Final Report Peer Review

**Individual Scientist:**	I approve this document.
Name:	**Lambert, Andre-Jean**
Lambert, Andre-Jean	03-Mar-2022 14:58:35 (UTC+00:00)
Electronically Signed in M-Files	Timestamp

SIGNATURE(S) FOR DOCUMENT: 5550014 General Toxicology Final Report

**QA Approval:**	I approve the Quality Assurance Statement for this report.
Name:	**Huang, Bo**
Huang, Bo	07-Mar-2022 16:45:19 (UTC+00:00)
Electronically Signed in M-Files	Timestamp

**Study Director Approval:**	I approve this Report.
Name:	**Cinquino, Stefania**
Cinquino, Stefania	07-Mar-2022 18:12:40 (UTC+00:00)
Electronically Signed in M-Files	Timestamp
