# Supplementary material for: Intrathecal AAV9/AP4M1 gene therapy for hereditary spastic paraplegia 50 shows safety and efficacy in preclinical studies
Source: J Clin Invest. 2023 May 15;133(10):e164575. doi: 10.1172/JCI164575 (PMC10178841; doi:10.1172/JCI164575)
Supplement: Supplemental data [file jci-133-164575-s057.pdf]

A.

	WT/LoF [WT/p.E232GfsX21]			LoF/LoF [p.R306X/p.E232GfsX21]		
**AAV2/*AP4M1***	-	1E4	1E5	-	1E4	1E5
**Mean ATG9A Ratio**	1.30 ± 0.02	1.25 ± 0.02	1.25 ± 0.04	1.71 ± 0.03	1.25 ± 0.02	1.23 ± 0.02
**Mean ATG9A fluorescence inside TGN (A.U.) (SD)**	1085 ± 45.8	1086 ± 26.2	1098 ± 97.9	1625 ± 41.7	1077 ± 22.4	1048 ± 55.1
**Mean ATG9A fluorescence outside TGN (A.U.) (SD)**	835 ± 33.3	869 ± 25.2	880 ± 110.7	953 ± 24.9	859 ± 16.8	852 ± 50.7
**Mean cell count (per well) (SD)**	482 ± 147	454 ± 80	227 ± 34	713 ± 193	415 ± 98	131 ± 22

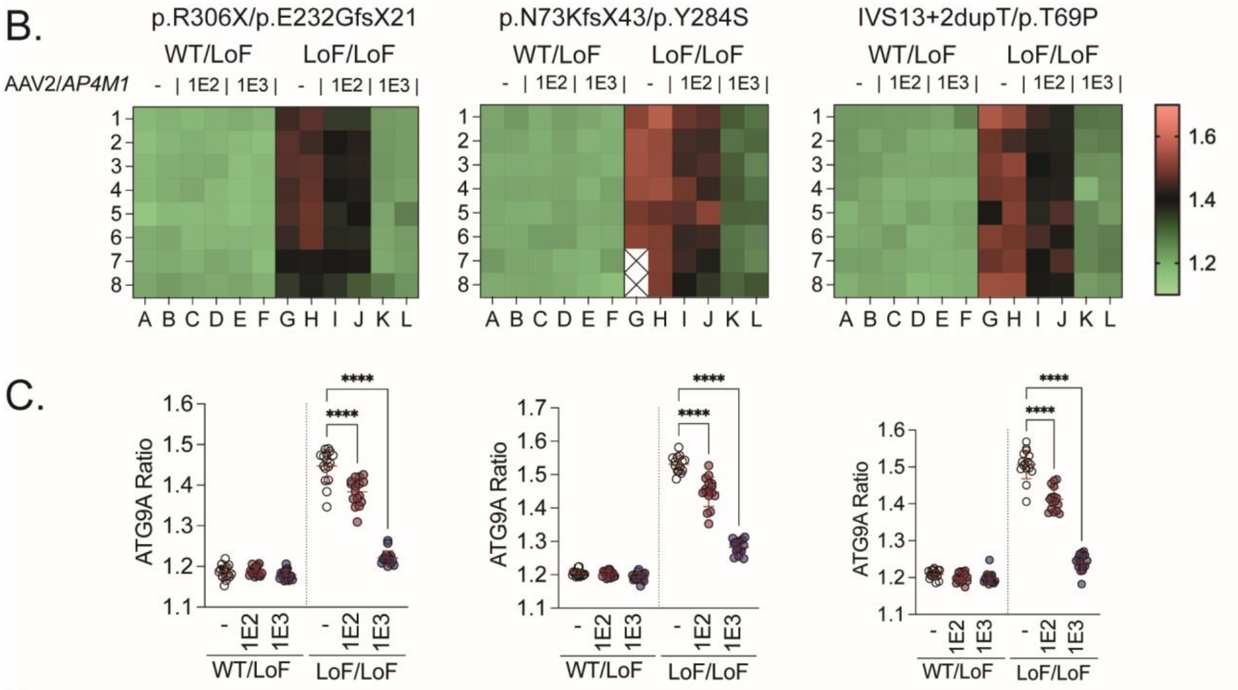

D.

	WT/LoF [WT/p.E232GfsX21]			LoF/LoF [p.R306X/p.E232GfsX21]			WT/LoF [WT/?]		
**AAV2/*AP4M1***	-	1E2	1E3	-	1E2	1E3	-	1E2	1E3
**Mean ATG9A Ratio**	1.19 ± 0.02	1.19 ± 0.01	1.18 ± 0.01	1.45 ± 0.04	1.38 ± 0.03	1.22 ± 0.02	1.20 ± 0.01	1.20 ± 0.01	1.19 ± 0.01
**Mean ATG9A fluorescence inside TGN (A.U.) (SD)**	1534 ± 67.7	1495 ± 56.6	1482 ± 55.1	1982 ± 54.7	1829 ± 58.8	1521 ± 64.7	1513 ± 59.1	1461 ± 33.9	1452 ± 44.2
**Mean ATG9A fluorescence outside TGN (A.U.) (SD)**	1293 ± 62.2	1257 ± 40.9	1257 ± 37.6	1371 ± 70.8	1322 ± 53.9	1245 ± 46.2	1257 ± 50.3	1215 ± 31.5	1217 ± 46.3
**Mean cell count (per well) (SD)**	633 ± 57	594 ± 34	529 ± 55	650 ± 214	577 ± 216	470 ± 158	877 ± 174	927 ± 132	778 ± 122

	LoF/LoF [p.N73KfsX43/p.Y284S]			WT/LoF [WT/IVS13+2dupT]			LoF/LoF [IVS13+2dupT/p.T69P]		
**AAV2/*AP4M1***	-	1E2	1E3	-	1E2	1E3	-	1E2	1E3
**Mean ATG9A Ratio**	1.53 ± 0.03	1.45 ± 0.04	1.28 ± 0.02	1.21 ± 0.01	1.20 ± 0.01	1.20 ± 0.02	1.51 ± 0.04	1.41 ± 0.03	1.24 ± 0.02
**Mean ATG9A fluorescence inside TGN (A.U.) (SD)**	1953 ± 40.3	1780 ± 56.6	1544 ± 39.9	1415 ± 43.2	1432 ± 54.0	1445 ± 79.6	1947 ± 108.5	1751 ± 73.5	1450 ± 63.9
**Mean ATG9A fluorescence outside TGN (A.U.) (SD)**	1275 ± 18.0	1243 ± 21.9	1204 ± 19.4	1171 ± 38.7	1194 ± 43.5	1206 ± 55.5	1293 ± 86.7	1240 ± 55.0	1168 ± 37.2
**Mean cell count (per well) (SD)**	804 ± 226	755 ± 212	528 ± 143	820 ± 122	833 ± 128	647 ± 90	559 ± 170	549 ± 143	375 ± 77

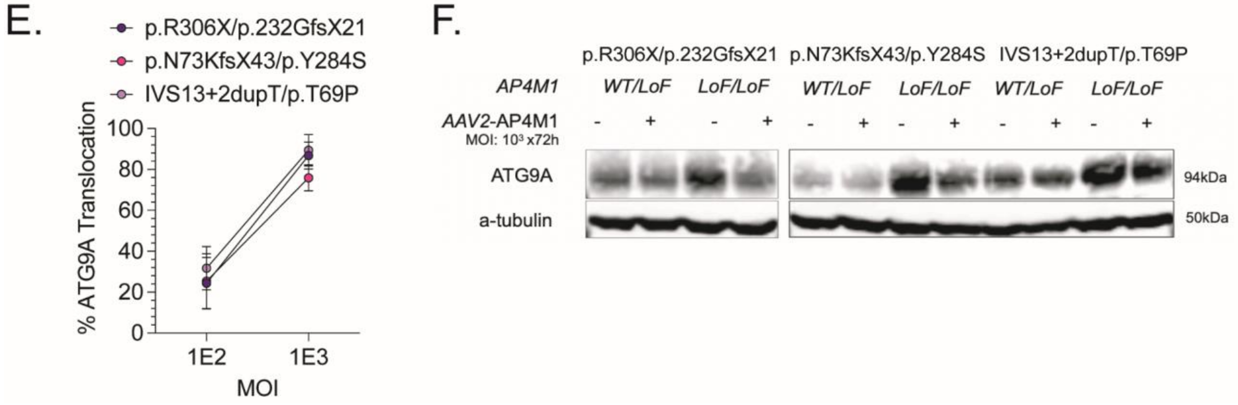

Supplemental Figure 1. AAV2/*AP4M1* vector restored ATG9A trafficking in primary fibroblasts from three patients with SPG50. (**A**) Summary of results of fibroblasts from a clinically-unaffected heterozygous carrier (same-sex parent, *WT/p.E232GfsX21*) and patient with biallelic LoF variants in *AP4M1* (p.R306X/p.E232GfsX21), treated with AAV2/*AP4M1* vector at an MOI of 1E4 or 1E5 for 72 h. Treatment at 1E5 led to a reduction in the average cell count in both control and patient fibroblasts. (**B**) Heatmaps of 96-well microplates indicating the ATG9A ratio in fibroblasts from three SPG50 patients (carrying three different sets of compound heterozygous *AP4M1* variants: p.R306X/p.E232GfsX21, p.N73KfsX43/p.Y284S, or IVS13+2dupT/p.T69P) and controls, treated with AAV2/*AP4M1* vector at an MOI of 1E2 or 1E3 for 72 h. (**C**) The mean ATG9A ratio for all conditions is shown. Scatter plots show the mean and standard deviation for each well (n=16 wells/group). Data sets were compared using one-way ANOVA with α set at 0.05 and Dunnett's correction. ****p<0.0001compared to patient fibroblasts untreated. (**D**) Summary of results of fibroblasts from a clinically-unaffected heterozygous carriers and patients with biallelic LoF variants in *AP4M1*. (**E**) The percent translocation of ATG9A. Translocation is defined as the delta between the mean of the experimental well and the negative control (LoF/LoF without virus), divided by the delta between the negative and positive controls (WT/LoF without virus). A dose dependent effect becomes evident for all patient fibroblast lines tested. (**F**) Western blot from whole cell lysates of fibroblasts from clinically-unaffected heterozygous carriers (parents) and SPG50 patients treated with AAV2/*AP4M1* at an MOI of 1E3 for 72 h. Levels of ATG9A decrease in patient-derived fibroblasts following treatment with AAV2/*AP4M1*1. AAV2, adeno-associated virus 2; AP4M1, adaptor protein complex, subunit μ4; ATG9A, autophagy related 9A; A.U. (arbitrary unit), LoF, loss of function; MOI, multiplicity of infection, SD (standard deviation), TGN, trans-Golgi network; WT, wild-type.

Supplemental Table 1. Fibroblast cell lines from well-characterized SPG50 patients used in this study

ID #	AP4M1 Variants	CADD PHRED	Sex	Age	Race	GMF CS	SPRS	DD/ID	Spasticity	Microcephaly	Epilepsy	Thin Corpus Callosum	Ventriculo-megaly	Periventricular White Matter Changes
#1	c.916C>T<br>p.Arg306X<br>(Nonsense)<br><br>c.694dupG<br>p.Glu232GlyfsX21<br>(Frameshift)	39/33	M	1.5y	White	5	29	+	Diplegia	+	+	+	+	+
#2	c.218dupA<br>p.Asn73LysfsX43<br>(Frameshift)<br><br>c.851A>C<br>p.Tyr284Ser<br>(Missense)	25/30	M	9y	White	5	41	+	Tetraplegia	+	+	+	+	+
#3	c.1025+2dupT<br>p.?<br>(Splice site)<br><br>c.205A>C<br>p.Thr69Pro<br>(Missense)	34/22	M	5y	Black	2	4	+	Diplegia	-	+	+	+	-

Supplemental Table 2. Treatment with AAV2/*AP4M1* vector restores ATG9A trafficking in primary fibroblasts from SPG50 patients

Patient	#1			#1			#1			#1		
Dose	1E2			1E3			1E4			1E5		
Cells	imaged	rescued	% rescue	imaged	rescued	% rescue	imaged	rescued	% rescue	imaged	rescued	% rescue
Exp. 1	91	8	8.8	95	21	22.1	125	36	28.8	128	55	43.0
Exp. 2	106	7	6.6	111	32	28.8	181	58	32.0	116	64	55.2
Mean			7.7±1.1			25.5±3.3			30.4±1.6			49.1±6.1

Patient	#2			#2			#2			#2		
Dose	1E2			1E3			1E4			1E5		
Cells	imaged	rescued	% rescue	imaged	rescued	% rescue	imaged	rescued	% rescue	imaged	rescued	% rescue
Exp. 1	112	22	19.6	97	49	50.5	101	54	53.5	98	78	79.6
Exp. 2	117	32	27.4	107	63	58.9	103	65	63.1	112	83	74.1
Mean			23.5±3.9			54.7±4.2			58.3±4.8			76.8±2.7

Supplemental Table 3. Pathological liver abnormalities in mice from the AP4M1 efficacy study

Mouse ID	Life span (Month)	Sex	Treatment	Pathological liver abnormalities
M1.52.14	16.7	M	Control	Liver tumor
M1.11.85	20.5*	M	Control	Liver tumor
M1.60.83	20.5*	M	Control	Liver tumor
M1.67.57	20.5*	M	control	Liver tumor
M1.33.58	16.1	M	Vehicle	Hepatic lipidosis
M1.36.90	20.5*	M	Vehicle	Liver tumor
M1.54.26	20.5*	M	Vehicle	Liver tumor
M1.24.85	20.5*	M	Low dose	Liver tumor
M1.60.96	20.5*	F	Low dose	Liver tumor
M1.19.52	20.5*	F	Low dose	Liver lipidosis
M1.59.789	8.4	M	High dose	Hepatocellular necrosis
M1.66.51	11.7	M	High dose	Liver tumor
M1.38.09	20.5*	M	High dose	Liver tumor

* Planned endpoint

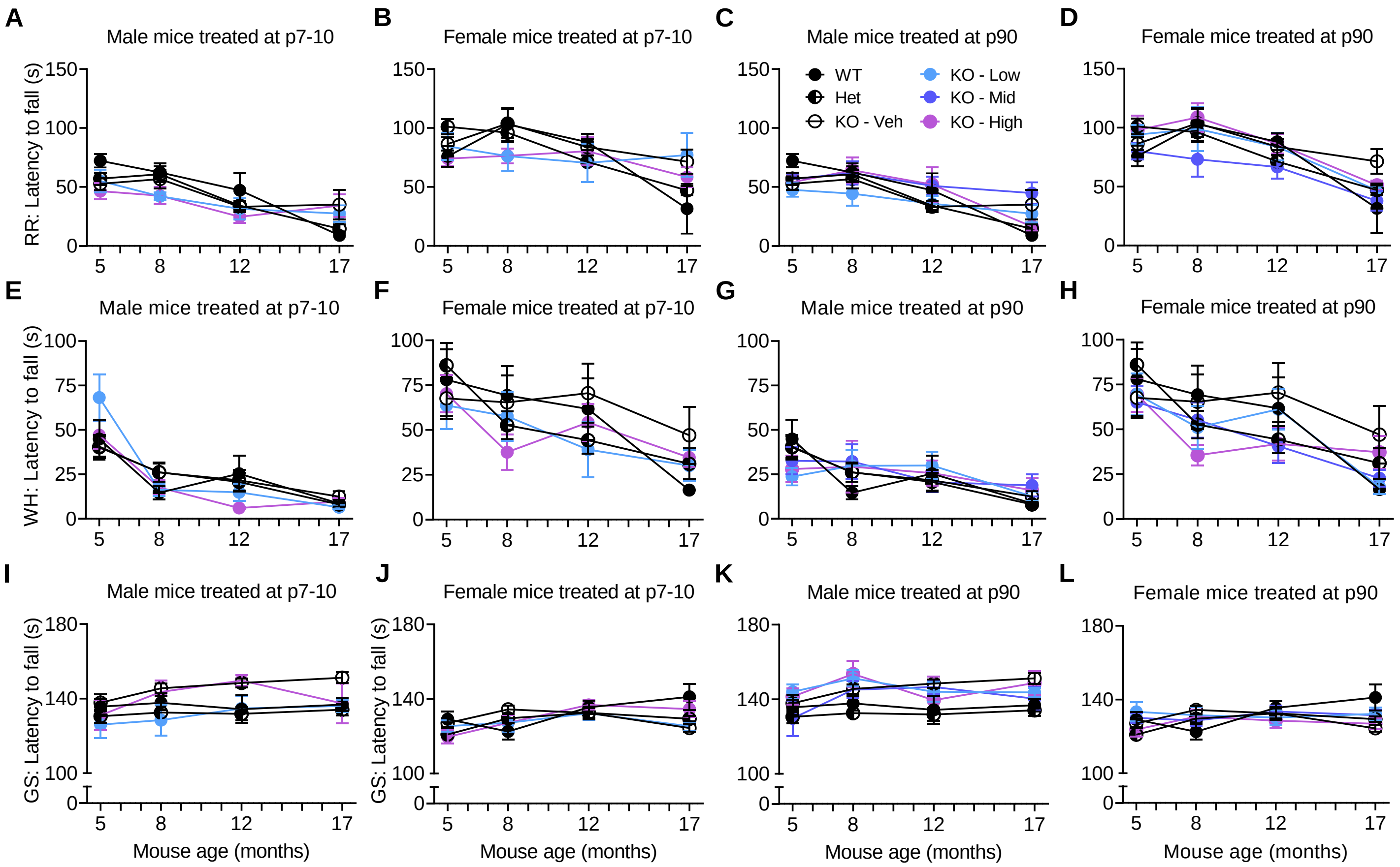

Supplemental Figure 2. No abnormal behavioral phenotypes in *Ap4m1* KO mice in rotarod, wire hang, and grip strength tests. vehicle, low (1.25E11 vg/mouse), mid (2.5E11 vg/mouse), or high (5E11 vg/mouse) doses of AAV9/*AP4M1* vector were administered intrathecally to balanced numbers of male and female mice at postnatal day p7-10 or p90, with WT and Het mice as normal control. The mice were allowed to survey a rotarod (**A-D**), wire hang (**E-H**) and grip strength (**I-L**) tests at 5, 8, 12, and 17 months old. All data are presented as mean ± SEM (male (n=7-26) and female (n=5-24)). Two-way ANOVA with repeated measures was used for statistical analysis. AAV9, adeno-associated virus 9; AP4M1, adaptor protein complex, subunit μ4; GS, grip strength; Het, Heterozygotes; IT, intrathecal; RR, rotarod; vg, vector genome; WH, wire hang; WT, wild-type.

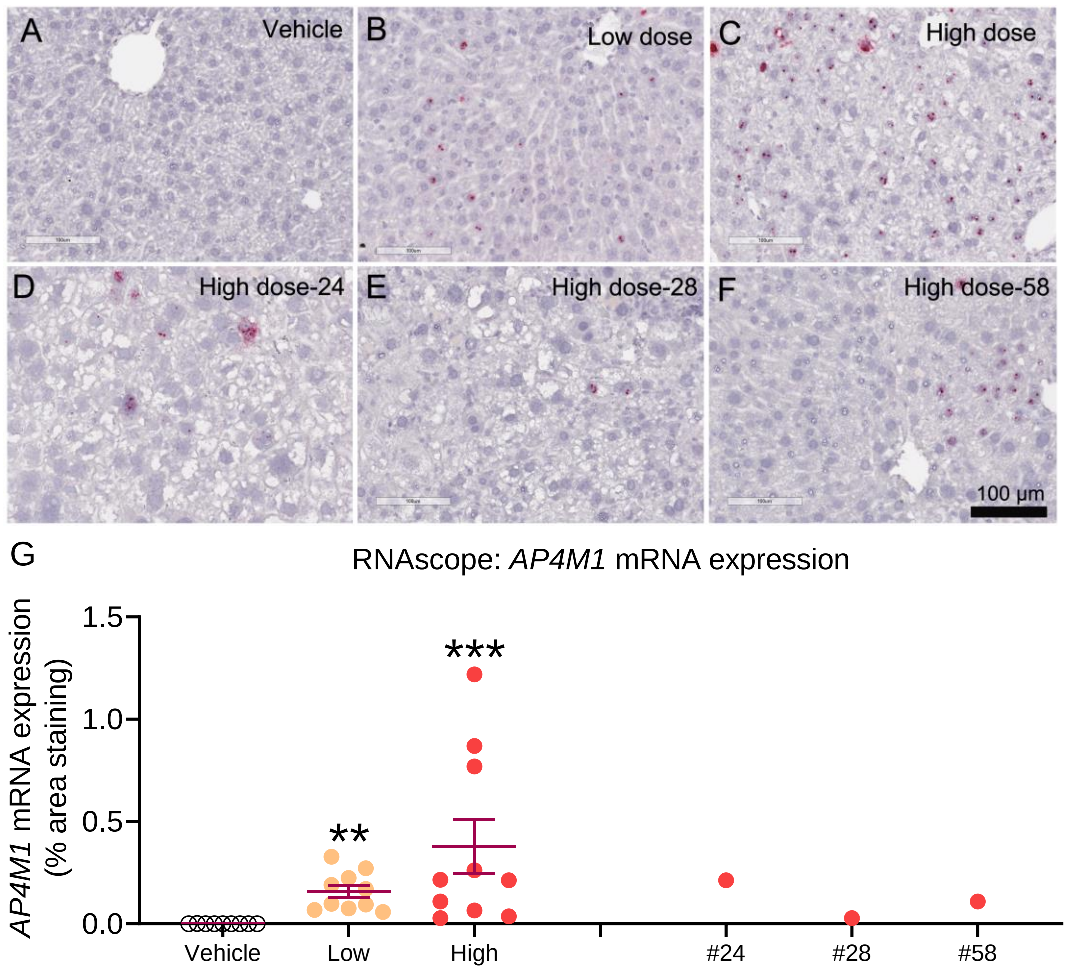

Supplemental Figure 3. Representative RNAscope staining (A-F) and quantification (G) of WT mice livers. Vehicle, low (1.25E11 vg/mouse), or high (5E11 vg/mouse) doses of AAV9/*AP4M1* vector were administered intrathecally to male and female mice at postnatal day p42-56 (n=10/group/sex). Granular appearance was observed during necropsy in the liver of three mice (#24, #28, and #58) which were treated with high dose of AAV9/*AP4M1* for 12 months. Liver histology images at 12 months post-injection (A-F) with one section per animal were digitized with a ScanScope slide scanner and analyzed using custom analysis settings in HALO™ Image Analysis Platform (**G**). Results are presented as % area staining positive for hAP4M1opt mRNA by tissue region. All data in **G** are presented as the mean ± SEM. Data sets that passed tests for normality or homogeneity of variance were analyzed using one-way ANOVA with α set at 0.05 and Dunnett's correction for relevant pairwise comparisons. Data sets that did not pass tests for normality or homogeneity of variance were analyzed using Kruskal-Wallis test with α set at 0.05 and Dunn's correction for relevant pairwise comparisons. **$p<0.01$ and ***$p<0.001$ compared to KO-Vehicle. Scale bars in A-F, 100 μm.

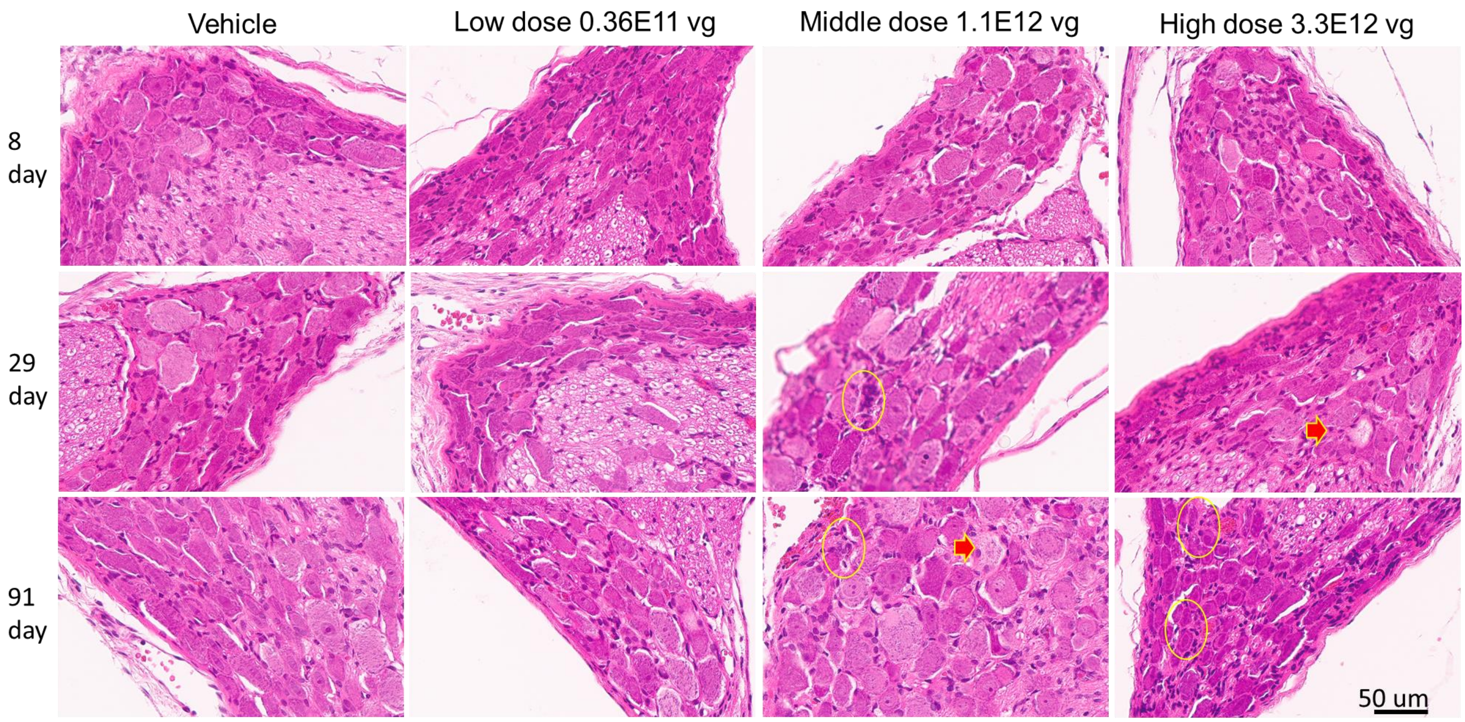

Supplemental Figure 4. Representative images of dorsal root ganglion (DRG) from WT rats IT treated with AAV9/*AP4M1*. WT rats were treated intrathecally with vehicle, low (1.25E11 vg), middle 2.5E11, or 5E11 vg/rat of AAV9/*AP4M1* vector. DRGs from the rats were harvested at 8-, 29-, or 91-days post treatment and stained with hematoxylin and eosin. Histology images with one section per animal were digitized with a ScanScope slide scanner. Red arrows indicate neuron degeneration and yellow circles indicates immune cell infiltrates. Scale bar, 50 μm.

A

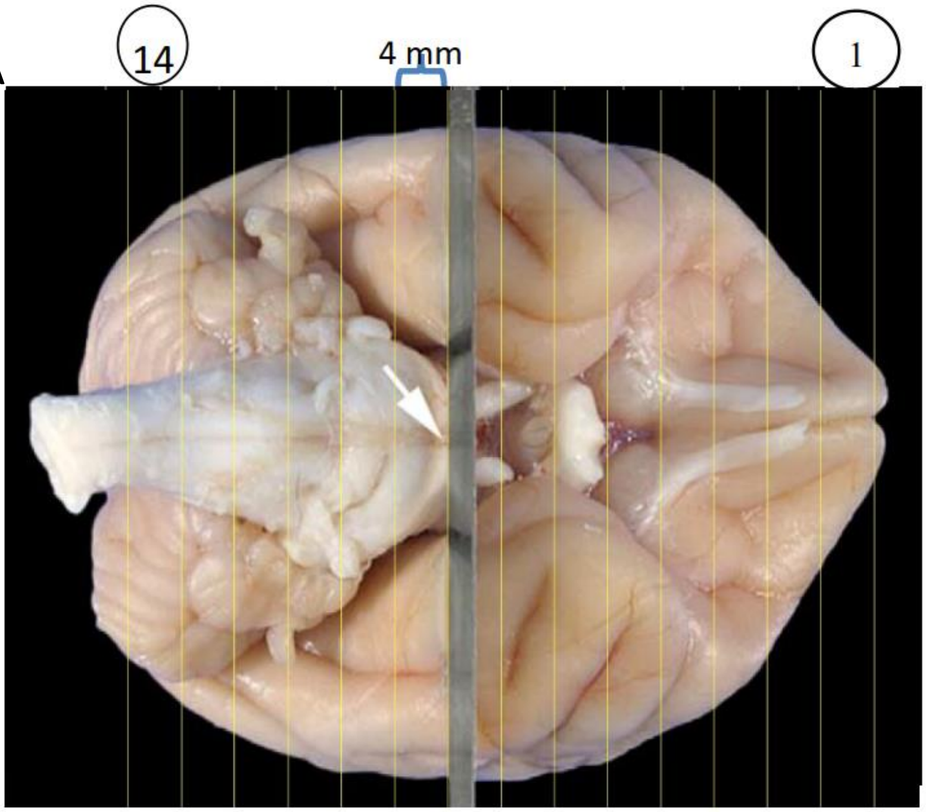

B

Short name	Slice#	Punch #	Brain regions	Hemisphere
Br-1	3	1	Frontal Corex	Right
Br-2	3	2	Frontal Corex	Left
Br-3	7	3	Striatum	Right
Br-4	7	4	Striatum	Left
Br-5	9	5	Parietal Cortex	Right
Br-6	9	6	Parietal Cortex	Left
Br-7	9	7	Temporal Cortex	Right
Br-8	9	8	Temporal Cortex	Left
Br-9	9	9	Hippocampus	Right
Br-10	9	10	Hippocampus	Left
Br-11	9	11	Thalamus	Right
Br-12	9	12	Thalamus	Left
Br-13	11	13	Pons	Right
Br-14	11	14	Pons	Left
Br-15	11	15	Midbrain	Right
Br-16	11	16	Midbrain	Left
Br-17	13	17	Medulla	Right
Br-18	13	18	Medulla	Left
Br-19	15	19	Cerebellum	Right
Br-20	15	20	Cerebellum	Left
Br-21	15	21	Occipital Cortex	Right
Br-22	15	22	Occipital Cortex	Left

Supplemental Figure 5. Brain tissues collected for vector biodistribution and transgene expression in NHP toxicity study. NHP brain was sliced into 15 slices with the initial razor cut being consistent from rostral to pons and all subsequent slices 4mm apart (**A**). Brain tissue from different regions of both hemispheres was punched out and stored at -80C until further analyses for vector biodistribution and transgene expression (**B**).

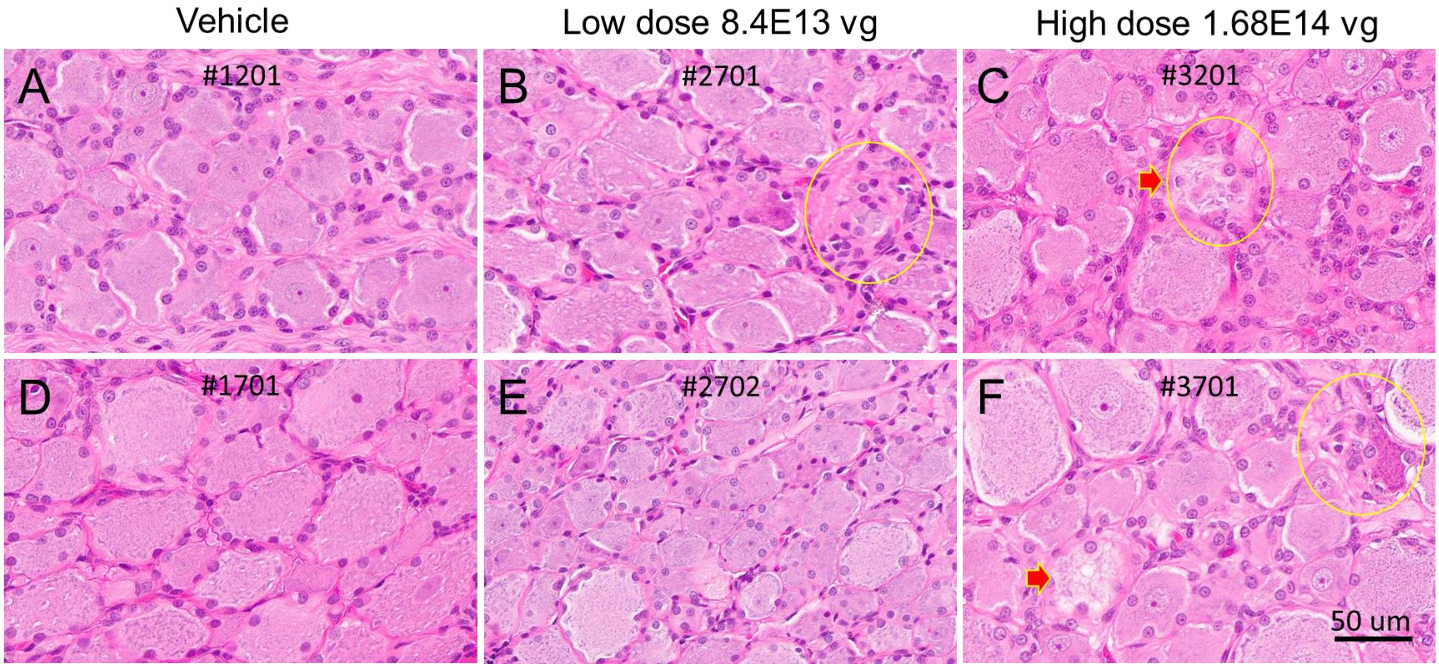

Supplemental Figure 6. Representative images of dorsal root ganglion (DRG) from WT NHPs IT treated with AAV9/*AP4M1*. WT NHPs were treated intrathecally with vehicle, low (8.4E13 vg), or 1.68E14 vg/NHP of AAV9/*AP4M1* vector. DRGs from the NHPs were harvested at 94-days post treatment and stained with hematoxylin and eosin. Histology images with one section per animal were digitized with a ScanScope slide scanner. Red arrows indicate neuron degeneration and yellow circles indicates immune cell infiltrates. Scale bar, 50 μm.

Quality Control Summary

Lot #	8829	Name	AP4M1

Test by qPCR

Test #	Titer, vg/mL	Analyst	Date	File
1	2.81E+12	PZ	10/03/2019	20191003-0945-bh-pz

PAGE analysis

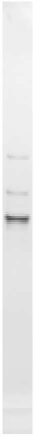

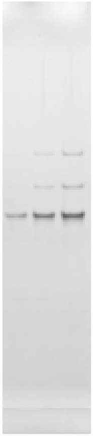

Loaded 5.00E+09 vg 4980E std 2e9vg 5e9vg 1e10vg
Calculated 5.70E+09 vg

Analyst	Ping Zhang
Date	10/07/2019
Reference #	20191007-silver

UNIVERSITY OF NORTH CAROLINA
VECTOR CORE

Quality Control Summary

Lot #	LAV112-conc	Name	AP4M1

Test by qPCR

Test #	Titer, vg/mL	Analyst	Date	File
1	1.03E+14	PZ	11/21/2019	20191121-1007-ghbh-pz

PAGE analysis

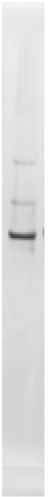

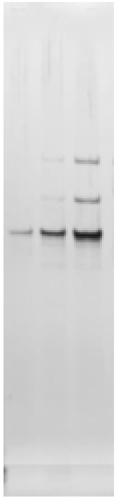

Loaded 5.00E+09 vg   4980E std   2e9vg   5e9vg   1e10vg
Calculated 5.20E+09 vg

Analyst	Ping Zhang
Date	11/19/2019
Reference #	20191119-silver

UNIVERSITY OF NORTH CAROLINA
VECTOR CORE

SEM

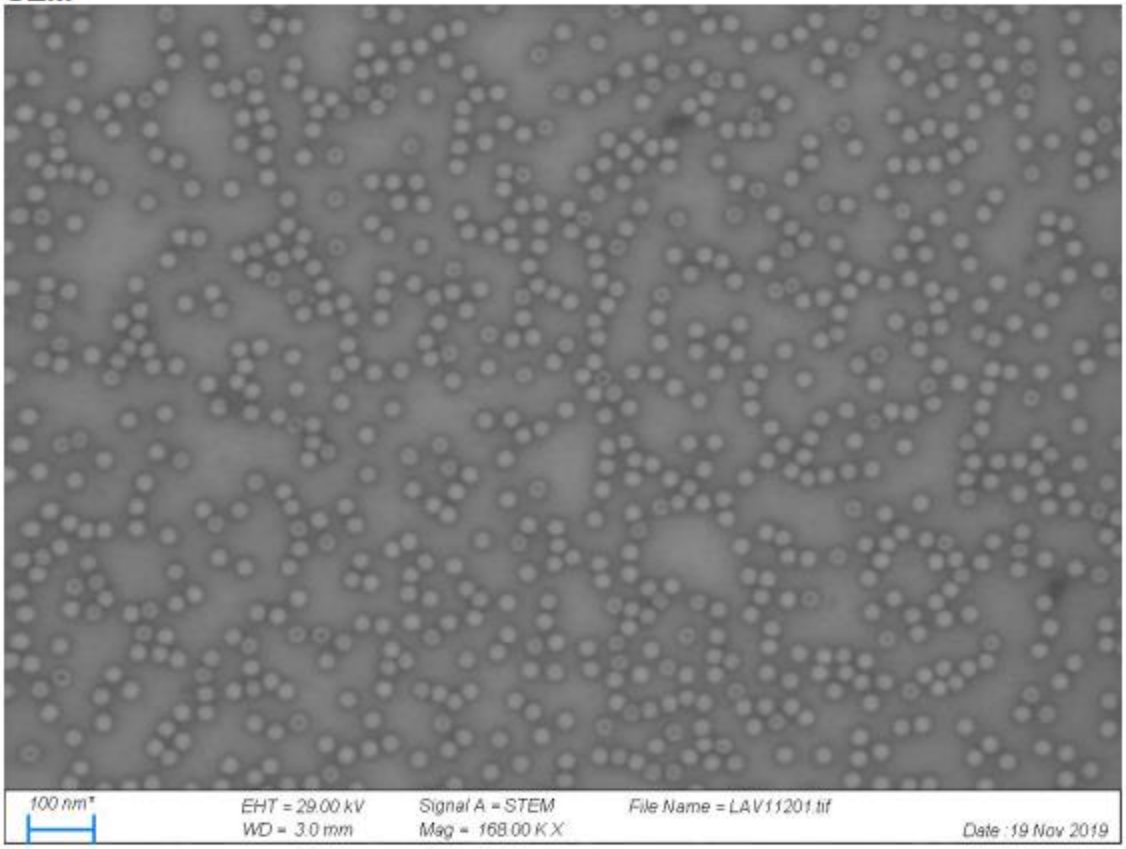

88% full

Analyst	Ping Zhang
Date	11/19/2019
Reference #	20191119-LAV112-02

Certificate Of Analysis

Research grade - Not for human use.

For Research Use Only

Client identification: CURE SPG50

Product Name and type of product: rAAV9, sterile suspension of rAAV9 vector carrying the AP4M1 therapeutic gene.

Batch Number: T-GEMINIS-033

Quality grade: Research grade

Production process: 50 Liters batch

Manufacturing date: 22 December 2020

Formulation buffer (FMR-T-0043): MilliQ Water, 1X dPBS, 5% D-Sorbitol, 0.001% Pluronic

Vials Shipped: 34 x 0.5 ml vialed in polypropylene Cryotubes

Shipment Date: 19 January 2021

Transport conditions: Dry ice

Storage conditions: ≤-60ºC

Performed by:	Paz López (QC Coordinator)	Date: 29-APRIL-2021
Reviewed by	Sandy Douthe (QC Manager)	Date: 29 APRIL 2021
Reviewed by	Begoña Ortega (QA Technician)	Date: 29-APRIL 2021

Parque Tecnológico de Guipuzcoa - Paseo Mikeletegui, 83, 2ª Planta
20009 San Sebastián, Spain - Tel: +34 943 477 733 - viralgenvc.com

rAAV Purified Bulk / Bulk Drug Substance assays:

ASSAY	METHOD	TEST SITE	SPECIFICATION	RESULT
**SAFETY ASSAYS**				
Replication competent AAV	PT/133 & PT/025 Infection of permissive cell line/Rep2 qPCR (based on ITRqPCR titration)	Genosafe	Report result	Not Detected <10 rcAAV in $1x10^{11}$ vg
**STRENGHT ASSAYS**				
Vector genome titer (vg/mL)	PNT-CC-005 ITR qPCR	Viralgen	report results	$1.71x10^{14}$
Vector genome titer (vg/ml)	PNT-CC-049 ITRddPCR	Viralgen	report results	$5.17x10^{13}$
**PURITY ASSAYS**				
General Purity	PNT-CC-012 SDS-Page/ silver stain	Viralgen	Report result	Detection VP1, VP2 and VP3. Presence of extra bands between 150-250 KDa
Residual Host Cell Protein (ng/mL)	PNT-CC-023 HEK293 ELISA Assay	Viralgen	Report result	<100
Residual Host Cell DNA (pg/mL with and without DNase)	PNT-CC-033 18S qPCR (123 and 254 bp amplicons)	Viralgen	Report result	123 bp: $6.86x10^{6}$ (+DNase I) $7.38x10^{6}$ (-DNase I) 254 bp: $5.02x10^{6}$ (+DNAseI) $5.21x10^{6}$ (-DNase I)
Residual Host Cell DNA (pg/mL, without DNase)	SP-M.8303 E1A qPCR	SGS-Vitrology	Report result	$2.6x10^{6}$
Residual Plasmid DNA (copies/mL with and without DNase)	PNT-CC-014 KanaR qPCR	Viralgen	Report result	$9.61x10^{11}$ (+Dnase I) $1.26x10^{12}$ (-Dnase I)
Full/Empty particles ratio	V6725 CryoTEM	Vironova	>50% full	Filled: 84% Empty: 9% Uncertain: 7%

For Research Use only

Parque Tecnológico de Guipuzcoa - Paseo Mikeletegui, 83, 2ª Planta
20009 San Sebastián, Spain - Tel: +34 943 477 733 - viralgenvc.com

ASSAY	METHOD	TEST SITE	SPECIFICATION	RESULT
**PURITY ASSAYS**				
Aggregation	V0149 & V0692 nsTEM	Vironova	Report result	92% of the individual particles at <40nm
Residual Chemical (Transfection reagent)	Refer to DMF	Docuchem	Report result	<25.13 ppm >12.57 ppm
Residual Chemical (Lysis reagent)	Refer to DMF		Report result	<LOD (1 ppm)
Residual Chemical (Clarifying reagent)	Refer to DMF		Report result	<1.03 ppm >0.21 ppm
Residual Chemical (Iodixanol)	HPLC		Report result	<3.12 ppm >1.04 ppm
Residual Chemical (Antifoam)	Refer to DMF		Report result	<LOD (5 ppm)
Residual Affinity Ligand (ng/mL)	PNT-CC-037 ELISA	Viralgen	Report result	511.3
**IDENTITY ASSAYS**				
Protein Identity	PNT-CC-003 SDS-PAGE/ Western Blot	Viralgen	Detection of VP1, VP2 & VP3	Detection of VP1, VP2 and VP3
Genome identity	PNT-143 Sequencing (Sanger)	Secugen	100% conform to sequence of reference	Conform

For Research Use Only

Parque Tecnológico de Guipuzcoa - Paseo Mikeletegui, 83, 2ª Planta
20009 San Sebastián, Spain - Tel: +34 943 477 733 - viralgenvc.com

rAAV Final Product / Drug Product assays:

ASSAY	METHOD	TEST SITE	SPECIFICATION	RESULT
**SAFETY ASSAYS**				
Sterility	LTMI-009	Biolab S.L	No growth	No growth
Endotoxin (EU/ml)	PNT-CC-015 Kinetic chromogenic	Viralgen	<0.2	<0.051
[1]Mycoplasma	PNT-CC-001 PCR end point	Viralgen	Negative	Negative
**STRENGHT ASSAYS**				
Vector genome titer (vg/mL)	PNT-CC-005 ITR qPCR	Viralgen	$0.75x10^{14}$-$4x10^{14}$ Target $1.70x10^{14}$	$2.17x10^{14}$
Vector genome titer (vg/ml)	PNT-CC-049 ITRddPCR	Viralgen	Report Result	$5.43x10^{13}$
Infectious titer (TCID50/mL)	PNT-CC-004 TCID50/ITRqPCR	Viralgen	Report result	$1.02x10^{10}$
vg/TCID50 (ITRqPCR) ratio	N/A	Viralgen	Report result	21237.16
**QUALITY ASSAYS**				
Osmolality (mOsm/Kg)	PNT-CC-016 Freezing point	Viralgen	587 +/-50	570
pH	PNT-CC-044 Potentiometry	Viralgen	7.4±0.4	7.21
Appearence	PNT-CC-017 Visual inspection	Viralgen	Colorless, clear to slightly opalescent, free of visible particles	Not done because vialed in polypropylene cryovials
Particles size distribution	PNT-CC-053 DLS	Viralgen	Report Results	99-99.5% of particulate volume between 23.98-25.25 nm in mean diameter

[1] Mycoplasma assay performed in the transfection pool.

For Research Use

Parque Tecnológico de Guipuzcoa - Paseo Mikeletegui, 83, 2ª Planta
20009 San Sebastián, Spain - Tel: +34 943 477 733 - viralgenvc.com

1. <u>Test Information</u>: General purity and protein identity by SDS-PAGE/Silver staining and Western Blot analysis

According to the standard operating procedure PNT-CC-012_Pureza del vector por SDS_PAGE_TINCION DE PLATA, and the PNT-CC-003_Identificación de proteínas por Western Blot.

1.1 Gel Properties: 10% Acrylamide gel

1.2. SDS-PAGE Results:

SDS-PAGE/Silver staining

$5x10^9$ vg/well (based on ITRqPCR titer result)

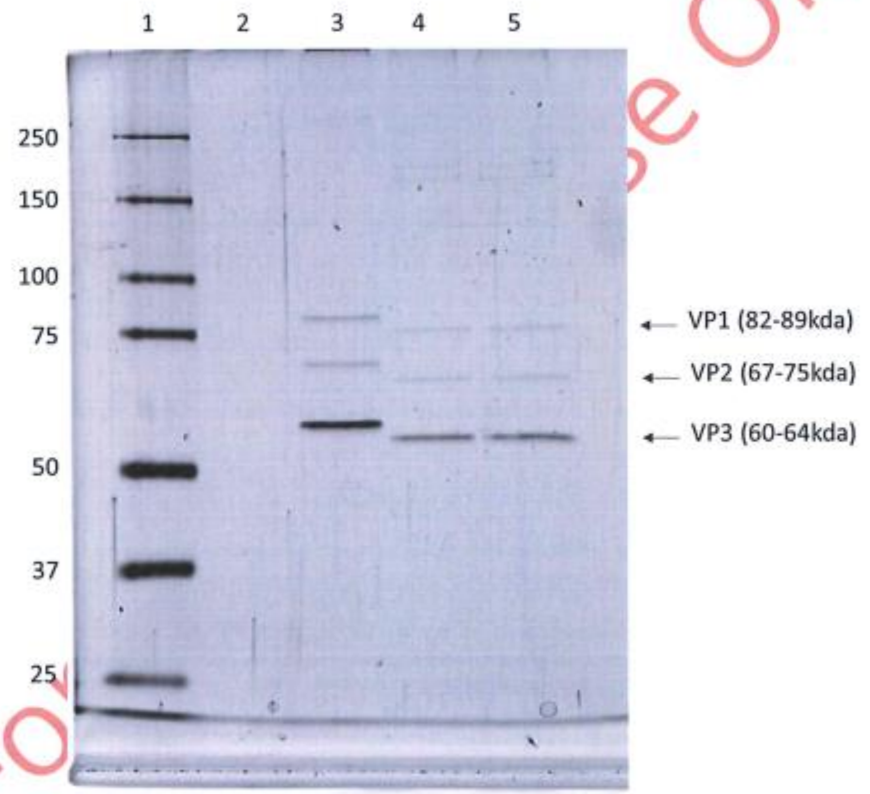

Lane 1: Ladder
Lane 2: Negative Control. Formulation Buffer FMR-T-0043
Lane 3: rAAV8 Standard vector ATCC VR-1816
Lane 4: T-GEMINIS-033 BDS
Lane 5: T-GEMINIS-033 BDS

Presence of extra-bands between 150-250 KDa. An investigational analysis was done and the extra bands were identified as encapsidated (or not accessible to DNase) DNA.

Parque Tecnológico de Guipuzcoa - Paseo Mikeletegui, 83, 2ª Planta
20009 San Sebastián, Spain - Tel: +34 943 477 733 - viralgenvc.com

1.3. SDS-PAGE/Western blot Results:

SDS-Page/Western Blot Results
5x10⁹ vg/well (based on ITRqPCR titer result)

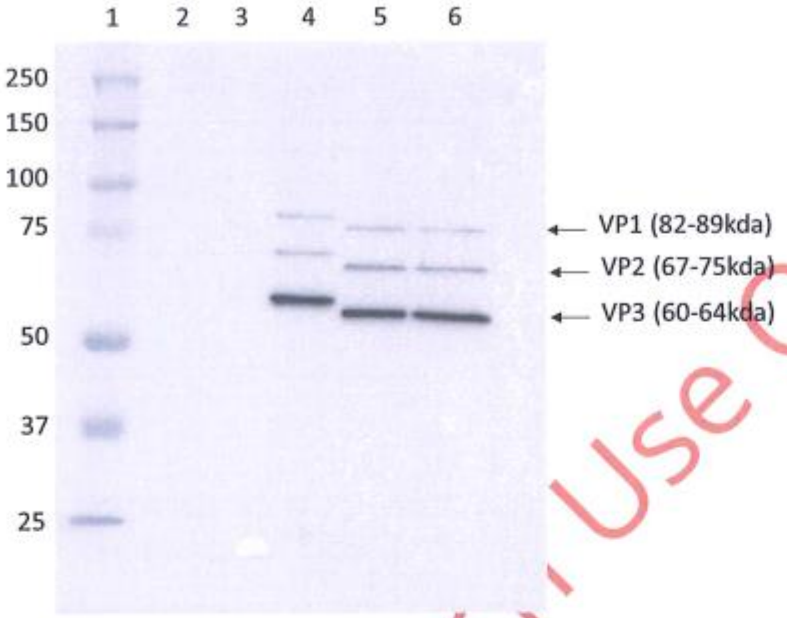

Lane 1: Ladder.
Lane 2: Empty
Lane 3: Negative Control. Formulation buffer FMT-T-0043
Lane 4: rAAV8 Standard vector ATCC VR-1816
Lane 5: T-GEMINIS-033 BDS
Lane 6: T-GEMINIS-033 BDS

For Research Use Only

Parque Tecnológico de Guipuzcoa - Paseo Mikeletegui, 83, 2ª Planta
20009 San Sebastián, Spain - Tel: +34 943 477 733 - viralgenvc.com

Phenotypes associated with this allele

Allele Symbol	$Ap4m1^{tm1b(EUCOMM)Wtsi}$
Allele Name	targeted mutation 1b, Wellcome Trust Sanger Institute
Allele ID	MGI:5636962
Summary	1 genotype

Jump to	Allelic Composition	Genetic Background	Genotype ID
hm1	$Ap4m1^{tm1b(EUCOMM)Wtsi}/Ap4m1^{tm1b(EUCOMM)Wtsi}$	$C57BL/6N\text{-}Ap4m1^{tm1b(EUCOMM)Wtsi}/Wtsi$	MGI:5756714

Genotype			Find Mice		Data Sources
MGI:5756714 hm1	Allelic Composition	$Ap4m1^{tm1b(EUCOMM)Wtsi}/Ap4m1^{tm1b(EUCOMM)Wtsi}$		Using the International Mouse Strain Resource (IMSR) Mouse lines carrying: $Ap4m1^{tm1b(EUCOMM)Wtsi}$ mutation (1 available); any Ap4m1 mutation (24 available)	IMPC Data for Ap4m1
	Genetic Background	$C57BL/6N\text{-}Ap4m1^{tm1b(EUCOMM)Wtsi}/Wtsi$			
	Cell Lines	EPD0808_1_D06			

♀	phenotype observed in females
♂	phenotype observed in males
N	normal phenotype

hematopoietic system

- increased hematocrit (J:211773)
  ♂ IMPC - WTSI
- increased leukocyte cell number (J:211773)
  ♂ IMPC - WTSI

homeostasis/metabolism

- increased circulating cholesterol level (J:211773)
  IMPC - WTSI
- increased circulating iron level (J:211773)
  ♀ IMPC - WTSI
- increased circulating alanine transaminase level (J:211773)
  ♀ IMPC - WTSI
- increased circulating alkaline phosphatase level (J:211773)
  ♀ IMPC - WTSI
- increased circulating aspartate transaminase level (J:211773)
  ♀ IMPC - WTSI
- decreased circulating serum albumin level (J:211773)
  ♀ IMPC - WTSI

immune system

- increased leukocyte cell number (J:211773)
  ♂ IMPC - WTSI

Supplemental Materials and Methods

Plasmid design and development

We designed and developed the UsP-*hAP4M1opt*-BGHpA plasmid (Figure 1A) containing the transgene of a human *AP4M1* codon-optimized construct (*hAP4M1opt*). The transgene consists of a human *AP4M1* codon-optimized DNA coding sequence of 1362 bp (ATUM, Menlo Park, CA) between a 328 bp UsP promoter and a 254 bp BGHpA polyadenylation signal. The UsP promoter is a combination of the minimal synthetic JeT promoter (20, 29, 30) and a synthetic intron sequence (31), which is utilized in this study for its small size allowing for packaging into an scAAV vector as well as to mediate a moderate level of ubiquitous transgene expression (31).

scAAV2/AP4M1 and scAAV9/AP4M1 vector preparation

The UsP-*hAP4M1opt*-BGHpA plasmid was packaged into scAAV2 and scAAV9 vectors (56) which are 10-100 times more efficient at transduction compared to traditional single-stranded (ss)AAV vectors (57, 58). The scAAV2/*AP4M1* vector (Lot# 8829, 2.81E12 vg/mL) was produced at the UNC Vector Core (UNC-VC) for all in vitro cell culture studies. The scAAV9/*AP4M1* vector (Lot# LAV-112-conc, 1.03E14 vg/mL with 88% full capsid) was produced at UNC-VC for all in vivo toxicity and efficacy studies in mice. The AAV2 and the AAV9 vectors made at UNC-VC were titered by qPCR and confirmed by silver staining compared to an internal reference standard at UNC-VC (59). Another scAAV9/*AP4M1* vector (Lot# T-GEMINIS-033, 5.43E13 vg/mL, 84% full capsids) was produced at Viralgen for all in vivo toxicity studies in WT rats and monkeys. The T-GEMINIS-033 lot was titered by Viralgen's ITR-based ddPCR titering assay; using this ddPCR method, the LAV-112-conc lot showed a titer of 9.9E13 vg/mL. The vectors produced at Viralgen underwent additional quality control release testing. The quality control summaries of the scAAV9/*AP4M1* vectors are included in the Supplemental information.

In vitro *SPG50 patient fibroblast culture and treatment*

Dr. Ebrahimi-Fakhari's group tested the AAV2/*AP4M1* vector in fibroblast lines from three different SPG50 patients and heterozygous controls (same sex parent) (Supplemental Table 1). Briefly, to derive fibroblasts, skin punch biopsies were incubated in 0.5% dispase solution (STEMCELL Technologies #07923) to remove the epidermis. Samples were then placed in gelatin-coated wells under growth conditions (37°C, 5% CO_2). Fibroblasts were grown in Dulbecco's Modified Eagle Medium (DMEM) high glucose (Gibco #11960044) supplemented with 20% fetal bovine serum (FBS) (Gibco #10082147), penicillin 100 U/ml and streptomycin 100 mg/ml (Gibco #15140122). Cells were kept in culture for up to eight passages with media changes every three days. For high-content imaging, cells were seeded onto 96 well plates (Greiner Bio-One #655090) at a density of 2E3 per well using the Multidrop Combi Reagent Dispenser (Thermo Fisher Scientific, #11388-558). Each plate carried loss-of-function (LoF/LoF) and matched WT (WT/LoF) cells. After 24 h, the media was replaced with AAV2/*AP4M1*-containing media at the respective MOI. Cells were incubated with AAV2/*AP4M1* for 72 h.

To generate whole cell lysates, cells were seeded in 6 well plates at a density of 5E5 per well. After 24 h, the media was changed to AAV2/*AP4M1*-containing media at the corresponding MOI. After 72 h incubation with AAV2/*AP4M1*, cells were washed twice with PBS and harvested in RIPA-buffer (G-Biosciences #786-489) containing protease inhibitors (Sigma-Aldrich #04693124001) and phosphatase inhibitors (Sigma #04906845001). For western blot analysis, total protein concentrations were determined using the PierceTM BCA Protein Assay Kit (Thermo Fisher Scientific #23225). Equal amounts of protein were solubilized in LDS buffer (Thermo Fisher Scientific # NP0008), heated at 70°C for 10 min and separated by gel electrophoresis using 4-12% Bis-Tris gels (Thermo Fisher Scientific #NP0322BOX) and MOPS buffer (Thermo Fisher Scientific #NP0001) under reducing conditions (Thermo Fisher Scientific #NP0009; Thermo Fisher Scientific #NP0005). Proteins were transferred to a PVDF membrane (EMD Millipore #IPFL00010) in transfer buffer (Thermo Fisher Scientific #NP0006-1). Following blocking with blocking buffer (LI-Cor Biosciences #927-70001) membranes were incubated overnight with the respective primary antibody (ATG9A 1:1000 abcam #ab108338, alpha-tubulin Sigma #T9026-.2ML 1:5000). Near-infrared fluorescent-labeled secondary antibodies (IR800CW #926-32213, IE680LT #926-68022, LI-COR Biosciences) were used and quantification was done using the Odyssey infrared imaging system and Image Studio Software (LI-COR Biosciences).

For immunocytochemistry analysis, fibroblasts were fixed with 4% paraformaldehyde (Electron Microscopy Sciences, #157-8-100) for 20 min. After washing three times with PBS (EMD Millipore #524650-1EA), cells were permeabilized in 0.1% saponin (Sigma #47036-50G-F) for 10 min and consequently incubated in a 1% BSA (Rockland #BSA-50) solution supplemented with 0.01% saponin for blocking. Afterwards, primary antibodies (ATG9A rabbit, abcam #ab108338, 1:400; TGN46 sheep, Bio-Rad #AHP500G, 1:800) diluted in blocking buffer were added for 1 h, followed by three washing steps with blocking buffer and incubation with secondary antibodies (goat anti-rabbit 488, Thermo Fisher Scientific #A11008, 1:2000; donkey anti-sheep, Thermo Fisher Scientific #A11016, 1:2000) as well as with Hoechst (Thermo Fisher Scientific #H3569, 1:2000) and Phalloidin (647 conjugate, Thermo Fisher Scientific #A22287, 1:2000) in blocking buffer for 30 min. Finally, cells were washed three times with PBS.

For high content imaging and automated image analysis, high-throughput confocal imaging was performed using the ImageXpress Micro Confocal Screening System (Molecular Devices) using an experimental pipeline described in Behne et al. (60) and Ebrahimi-Fakhari et al. (32). Images were acquired using a 20× S Plan Fluor objective (NA 0.45µM, WD 8.2-6.9mm). Each well was imaged at nine different sites in a 3 × 3 format. Image analysis was performed using a customized image analysis pipeline in MetaXpress (Molecular Devices). The ATG9A ratio was determined using an automated image analysis pipeline as described previously (32): First cells were identified and outlined based on the presence of a DAPI-positive nucleus inside a phalloidin (actin marker)-positive area. Next, different masks were generated: 1) An actin mask to outline the cell, 2) a TGN46 mask to outline the area of the trans-Golgi network, and 3) an ATG9A mask based on intracellular ATG9A fluorescence. ATG9A fluorescence intensity was then measured inside the

TGN mask as well as inside the actin-staining positive cytoplasm outside the trans-Golgi network (a mask generated by subtracting the TGN46 mask from the actin mask). The ATG9A ratio was calculated for each cell by dividing the ATG9A fluorescence in both compartments. Prior work, using fibroblasts from 18 well-characterized AP-4-associated hereditary spastic paraplegia patients, established that the ATG9A ratio meets standard quality control metrics for screening assays (Z-prime-factor robust >0.3, strictly standardized mean difference >3), and serves as an assay for AP-4 function with robust diagnostic power (ROC analysis: area under the curve: 0.85, 95% confidence interval: 0.849–0.852) (32).

Dr. Bonifacino's group tested the vector in fibroblast lines from two sibling patients with a donor splice site pathogenic mutation in intron 14 of the *AP4M1* gene (c.1137+1G→T) (Patient 1: 87RD38 and Patient 2: 87RD39) (3, 33) comparing them to skin fibroblasts from a control individual (85E0344). The two patients also carried heterozygous and homozygous mutations, respectively, in the ATS gene (2), but these mutations did not account for the SPG50 phenotype (2, 18). Fibroblasts were cultured in DMEM (Gibco) containing 10% FBS (Corning), 2 mM L-glutamine (Gibco), 100 units/mL penicillin and 100 µg/ml streptomycin (Gibco) at 37˚C in a humidified 5% CO_2 atmosphere. Passage-10 fibroblasts were transduced with different MOI of AAV2/*AP4M1* vector for 72 h. Fibroblasts were then fixed for 15 min in 4% w/v paraformaldehyde in PBS followed by 100% methanol for 5 min at -20°C. Cells were then washed twice in PBS and incubated in 0.1% w/v saponin, 1% w/v BSA (Gold Bio) in PBS for 15 min at room temperature. Primary and secondary antibodies were diluted in 0.1% w/v saponin, 1% w/v BSA in PBS and sequentially incubated for 30 min at 37°C. Coverslips were mounted with DAPI Fluoromount-G (Electron Microscopy Sciences). Confocal microscopy images were collected using a Zeiss LSM 780 confocal microscope with a Plan Apochromat 63x objective (N.A. 1.40), and final composite images were created using ImageJ/Fiji (https://fiji.sc/). In these experiments, we used the following primary antibodies: rabbit anti-ATG9A (Abcam; ab108338; IF 1:200), mouse anti-AP4E1 (BD Biosciences; 612028; IF 1:75) and sheep anti-TGN46 (Bio-Rad; AHP500G; IF 1:500), and secondary antibodies: Alexa Fluor 488-conjugated donkey anti-rabbit IgG (Thermo Fisher Scientific; A21206; 1:1000), Alexa Fluor 555-conjugated donkey anti-mouse IgG (Thermo Fisher Scientific; A31570; 1:1000) and Alexa Fluor 647-conjugated donkey anti-Sheep IgG (Thermo Fisher Scientific; A21448; 1:1000).

Ap4m1 KO mice

All research with mice were approved by the Institutional Animal Care and Use Committee (IACUC) of the UTSW Medical Center. *Ap4m1* KO mouse model was generated through targeted mutation 1b by Wellcome Trust Sanger Institute (http://www.informatics.jax.org/allele/MGI:5636962) and recovered by Jackson Laboratories. Briefly, the critical exons 5 to 12 were flanked by loxP sites and subsequent cre expression excised this critical sequence resulting in an *Ap4m1* KO mouse. The *Ap4m1* KO mice were identified by toe tattooing at p6-8 and then randomized into treatment groups based on the ID numbers assigned to them at genotyping. Genotyping was performed using genomic DNA extracted from clipped tail and four primers: (1)

FGEN-AP4M1 WT (TAGAAGC CAGCCATAGTGGT), (2) RGEN-AP4M1 WT (GAGCAAGTGTGTGGACCTGA), (3) FGEN_AP4M1 mut (CGGTCGCTACCATTACCAGT), and (4) RGEN-AP4M1 mut (TGTTTCAGTGTCCCATCTGC). A PCR protocol with annealing temperature of 65°C for 30 cycles was used for genotyping, which generated WT band of 355 bp and mutant band of 435 bp, respectively.

Efficacy study plan in Ap4m1 KO mice

The experimental design for the in vivo efficacy study is summarized in Figure 3A. In brief, both male and female *Ap4m1* KO mice were randomized into treatment cohorts and injected IT at p7-10 (pre-manifesting cohorts) or p90 (early-manifesting cohorts). For IT, 5 µL of vehicle or 1.25E11, 2.5E11, or 5E11 vg/mouse of AAV9/*AP4M1* vector was administrated via lumber IT injection. WT and Het mice without treatment were used as normal controls. All mice were weighed weekly up to 4 weeks old and monthly thereafter, as well as observed for overt signs of adverse effects at the times of weighing. The survival rate was calculated, and all unexpected deaths were investigated by the UTSW veterinary staff. Starting at 5 months of age, behavioral testing was carried out on all study cohorts and then repeated at 8-, 12-, and 17-months post dosing. A battery of behaviors, focusing mainly on motor function and including hindlimb clasping, elevated plus maze, and open field tests, was assessed blindly through the mouse behavior phenotyping core facility. At 3 weeks post-injection, subset of mice treated at p90 from each group were sacrificed to evaluate human *AP4M1* mRNA expression by RNAscope and early histological signs of treatment efficacy. Splenocytes from mice were used in ELISpot assays to detect any immune responses to either AAV9 capsid or AP4M1 peptides. Mouse blood was drawn via cardiac puncture and mouse sera were sent to UTSW Metabolic Phenotyping Core for the measurement of toxicology markers including AST, TBIL, ALB, CK, and BUN. All remaining mice were maintained to evaluate long-term survival and safety until 20.5 months of age, when all major organs were harvested for archival and further analyses.

Tissue preparation for RNAscope staining and image analysis

At necropsy, animals were deeply anesthetized via an intraperitoneal injection of a 2.5% avertin solution in normal saline. Animals were perfused for 5 min with 1×PBS containing 1 U/mL heparin. The left half of tissues were harvested and fixed in 10% neutral-buffered formalin (NBF) for 24 h and transferred to 70% ethanol. Tissues were then processed, embedded in paraffin, and cut into 5 µm sections.

For RNAscope to evaluate human *AP4M1* mRNA expression, sections were deparaffinated with xylene, which was removed with 200 proof ethanol, incubated with Hydrogen Peroxide (322335, Advanced Cell Diagnostics) for 10 min at RT, and then washed with dH_2O. The slides were boiled in 1×Target Retrieval solution (322000, Advanced Cell Diagnostics) for 10 min, washed with dH_2O, dehydrated with 200 proof ethanol, and allowed to air dry. Following this antigen retrieval, Protease Plus (322331, Advanced Cell Diagnostics) was added to each section, and incubated for 30 min at 40°C. The slides were washed with dH_2O, incubated with Hs-AP4M1-codon-No-XMm RNAscope probe (839541, Advanced Cell Diagnostics) in a HybEZ II Oven (Advanced Cell Diagnostics) for 2 h at 40°C, and washed with 1×Wash Buffer. The slides were incubated with AMP 1-6 for 15 min followed by RED solution for 5 min according to the RNAscope 2.5 HD

Detection Kit (322360, Advanced Cell Diagnostics) protocol and then counter stained with Mayer's hematoxylin (72804, Richard-Allan Scientific).

All stained slides with one section for each animal were digitized with a ScanScope slide scanner (Aperio Technologies). Scanned slides were viewed with the ImageScope software package (Version 10.0, Aperio Technologies) and analyzed using custom analysis settings in HALO™ Image Analysis Platform (Halo2.2, Indica Labs). A region of interest (ROI) was hand drawn on each image to allow for analysis by tissue region. Within the brain, regions were drawn around the cortex, sub-cortex, brain stem, and cerebellum. A threshold for each stain was set using positive and negative control images, and the same analysis settings were applied for every image of the same stain. Percent area staining for each marker of interest was recorded for each tissue/ROI. Analysis was done with the observer blinded to treatment group of each sample.

ELISpot analysis

For splenocyte preparation, approximately half of the spleen from each mouse was placed in a well of a 24-well plate containing 1 mL pre-chilled complete RPMI-1640 (cRPMI-1640) medium. The spleen was transferred onto a 70 µm cell strainer sitting on a 50 mL conical tube and crushed/torn apart using the rubber-plunger of a sterile 1 mL syringe. The splenocytes were collected by running 10 mL of cRPMI-1640 medium through the strainer and centrifuged at 400×g for 10 min at 4°C. The splenocytes were then resuspended in 1 mL of ACK lysis buffer (Gibco A1049201) to lyse red blood cells. After 10 min of lysis the reaction was stopped by the addition of 9 mL of chilled cRPMI-1640 medium and centrifuged again. Splenocytes were then resuspended, counted, placed in freezing medium (90% FBS, 10% DMSO), frozen, and placed in the vapor phase of liquid nitrogen until they were analyzed via ELISpot.

For preparation of peptide library, both AAV9 capsid and AP4M1 peptide libraries from Mimotopes (Victoria, Australia) were comprised of 15-mers with a 5 amino acid offset. The AAV9 capsid library pool contained 146 peptides and the final concentration of each peptide in the pool was 0.63 mg/mL in 1.59 % DMSO final. The AP4M1 peptide library pool contained 89 peptides and the final concentration of each peptide in the pool was 1.02 mg/mL in 0.61% DMSO final. Both pools were stored at -80°C.

ELISpot assays were performed using an ImmunoSpot kit (mIFNg-1M/5, Cellular Technology Limited). Briefly, splenocytes were thawed, washed, and resuspended in cRMPI-1640 medium for counting. 2E5 splenocytes in 100 uL of cRPMI-1640 medium were plated into each well of an ELISpot plate in quintuplicates whenever possible. 100 uL of cRPMI-1640 medium containing AAV9 capsid or AP4M1 peptide pool was then added to the wells. The controls included cells with no peptide, cells stimulated with a mixture of Phorbol 12-myristate 13-acetate (PMA) and Ionomycin, (Invitrogen, 00-4970-93), medium with INFγ which was supplied with the kit, or medium only. The splenocytes were incubated for 48 h in a humidified 37°C CO_2 incubator. All other steps were performed according to the manufacturer's recommendation.

Behavioral tests

Animals were assessed in a battery of behavioral tests including hindlimb clasping, elevated plus maze, and open field tests. All behavioral tests were repeated at 5, 8, and 12 months old by personnel blind to the genotype and treatment of the mice.

For hindlimb clasping testing, mice were suspended by the tail for 10 seconds at 20 cm height from the procedure table and the posture of hindlimbs was visually examined and recorded. The response was considered clasping if one (score 1) or both (score 2) hindlimbs retracted and touched the abdomen for more than 5 seconds during the suspension. The mouse was designated as non-clasping (score 0) if the hindlimbs remained splayed outward during the entire time of suspension.

For elevated plus maze testing, mice were placed in the center zone of an elevated maze raised 100 cm from the ground. The maze contained two open arms and two closed arms of equal width and length. All mice were placed in the same central point of the maze and allowed to freely explore for 5 min. Animal activity was monitored using video tracking software (Ethovison 13.1, Noldus). Duration in the open arms, as well as total distance moved, were tracked and recorded. The maze was wiped thoroughly with disinfectant between animals.

For open field testing, mice were placed in the periphery of a novel open field environment (44 cm × 44 cm, walls 30 cm high) in a dimly lit room (approximately 60 lux) and allowed to explore for 10 min. The animals were monitored from above by a video camera connected to a computer running video tracking software (Ethovision XT, version13.0, Noldus, Leesburg, Virginia) to determine the time, distance moved and number of entries into three areas: the periphery (5 cm from the walls), the center (14 cm × 14cm) and the non-periphery (the whole arena excluding the periphery zone). The open field arenas were wiped and allowed to dry between mice.

Non-GLP safety study in WT BL/6J mice

The non-GLP study presented in Figure 6A was designed to identify any long-term safety issues associated with the experimental therapy. The mice were randomized to different groups (n=10/group/sex) and injected IT with 5 µL of vehicle or different doses (1.25E11, or 5E11 vg/mouse) of AAV9/*AP4M1* vector. Mice were monitored following the treatment and appropriate supportive or therapeutic interventions were offered. A detailed necropsy was performed to investigate the reason for the ailment. Mouse sera were collected for a serum toxicity panel. Tissue samples including brain, heart, liver, lung, gonad, spleen, kidney, eyeball, sciatic nerve, cervical spinal cord, and lumbar spinal cord at 12 months following the treatment were collected for RNAscope staining and histopathological assessment. The histopathological evaluation on collected tissue samples was performed and reported by Dr. Mary Wight-Carter, DVM, DACVP, Veterinary Pathologist at the Animal Research Center, UTSW Medical Center (Supplemental information). Dr. Wight-Carter was blind to the treatment of the mice.

GLP safety study in WT SD rats

This animal study was performed at Charles River Laboratories (CRL), Inc. (Quebec, Canada). Male and female SD rats were randomized into cohorts (n=5/group/sex) and dosed as shown in Figure 7A. At the initiation of dosing, the animals assigned to study were 49 to 56 days old. The AAV9/*AP4M1* vector was injected IT once in each animal by a qualified laboratory technician, in a volume of 20 or 60 µL, and a final dose of 0.36E12, 1.1E12, or 3.6E12 vg/rat. All animals were

monitored up to 91 days following the injection. Rats were sacrificed on day 8, 29, or 91 post injection, splenocytes were prepared for ELISpot analysis, and tissues were collected for vector biodistribution, *AP4M1* expression, and toxicity evaluation. For biodistribution and expression, total DNA and RNA were purified from tissue samples collected at necropsy day 29, using a Qiagen Qiacube HT. cDNA synthesis was performed with RT2 HT First Strand Kit (Qiagen, 330411). Quantitative PCR (qPCR) was used to determine *AP4M1* DNA or cDNA copies present in a purified genomic DNA sample or a synthesized cDNA sample, respectively. The total amount of sample DNA or cDNA was determined by SYBR green qPCR analysis with primers specific to rat *GADPH*. The copies of *hAP4M1opt* DNA or cDNA within each sample was determined by SYBR green qPCR analysis with primers specific for *hAP4M1opt*. Details of this study are provided in CRL's final report (Supplemental Information).

GLP safety study in WT NHPs

This animal study was also performed at CRL, Inc. (Quebec, Canada). Cynomolgus Monkeys were randomized into cohorts (n=2/group) and dosed as shown in Figure 8A. At the initiation of dosing, the animals assigned to study were 2-4 years old. The AAV9/*AP4M1* vector was injected IT once in each animal by a qualified laboratory technician in a final dose of 8.4E13, or 1.68E14 vg/monkey. One mL of CSF was withdrawn from each animal immediately prior to vector injection, which verified needle placement. All animals were monitored up to 91 days following the injection. NCV testing was performed at baseline, day 45, and day 77 post-injection. Monkeys were sacrificed on day 91 post-injection, splenocytes were prepared for ELISpot analysis, and tissues were collected for vector biodistribution, *AP4M1* expression, and toxicity evaluation. For biodistribution and expression, total DNA and RNA were purified from tissue samples collected at necropsy day 91, using a Qiagen Qiacube HT. cDNA synthesis was performed with RT2 HT First Strand Kit (Qiagen, 330411). qPCR was used to determine *AP4M1* DNA or cDNA copies present in a purified genomic DNA sample or a synthesized cDNA sample, respectively. The total amount of sample DNA or cDNA was determined by SYBR green qPCR analysis with primers specific to monkey *GADPH*. The copies of *hAP4M1opt* DNA or cDNA within each sample was determined by SYBR green qPCR analysis with primers specific for *hAP4M1opt*. Details of this study are provided in CRL's final report (supplemental information).

Statistical Analysis

All quantitative data in this paper were presented as mean ± SEM, analyzed, and graphed using GraphPad Prism Software (v. 9.2.0, GraphPad Software). Data were tested for normal distribution (Shapiro-Wilk normality test) and homogeneity of variance (Brown-Forsythe test). Data sets that passed these two tests were analyzed using the Student's unpaired t-test for two groups comparison or one-way ANOVA for equal or more than three groups comparison with Dunnett's correction for relevant pairwise comparisons. Data sets that did not pass tests for normality or homogeneity of variance were analyzed using the Mann-Whitley test for two groups comparison or Kruskal-Wallis test with Dunn's correction for relevant pairwise comparisons. Two-way ANOVA with repeated measures was used for the analyses of time course data including body weight, behavior results, mRNA expression, and serum toxicity panel. For survival analysis, data shown in the Kaplan-Meier survival curve were compared with the Log-rank (Mantel-Cox) test. A $p < 0.05$ was considered as significant for all statistical analyses.
